# Global *In-Silico* Scenario of tRNA Genes and Their Organization in Virus Genomes

**DOI:** 10.3390/v11020180

**Published:** 2019-02-21

**Authors:** Sergio Morgado, Ana Carolina Vicente

**Affiliations:** Fundação Oswaldo Cruz, Instituto Oswaldo Cruz, Laboratório de Genética Molecular de Microrganismos, Rio de Janeiro, 22745-271, Brazil; anapaulo@ioc.fiocruz.br

**Keywords:** tRNA gene, tRNA gene cluster, virus, bacteriophages, ssRNA, translation apparatus, host range, codons, ssDNA

## Abstract

Viruses are known to be highly dependent on the host translation machinery for their protein synthesis. However, tRNA genes are occasionally identified in such organisms, and in addition, few of them harbor tRNA gene clusters comprising dozens of genes. Recently, tRNA gene clusters have been shown to occur among the three domains of life. In such a scenario, the viruses could play a role in the dispersion of such structures among these organisms. Thus, in order to reveal the prevalence of tRNA genes as well as tRNA gene clusters in viruses, we performed an unbiased large-scale genome survey. Interestingly, tRNA genes were predicted in ssDNA (single-stranded DNA) and ssRNA (single-stranded RNA) viruses as well in many other dsDNA viruses of families from *Caudovirales* order. In the latter group, tRNA gene clusters composed of 15 to 37 tRNA genes were characterized, mainly in bacteriophages, enlarging the occurrence of such structures within viruses. These bacteriophages were from hosts that encompass five phyla and 34 genera. This *in-silico* study presents the current global scenario of tRNA genes and their organization in virus genomes, contributing and opening questions to be explored in further studies concerning the role of the translation apparatus in these organisms.

## 1. Introduction

Viruses are highly dependent on the host translation machinery for their protein synthesis, presenting compact genomes with a high density of coding regions. However, genes related to replication, transcription and/or translation, mainly tRNA (transfer RNA) genes, are occasionally identified in viruses [1,2,3,4]. Contrasting with this scenario, some giant viruses carry several genes from the translation apparatus, particularly the recently characterized Tupanviruses, lacking only the ribosomal genes [5,6]. To date, tRNA genes have been only observed in some double-stranded DNA virus families, such as *Myoviridae*, *Siphoviridae*, *Podoviridae*, *Mimiviridae*, *Phycodnaviridae*, *Baculoviridae* and *Herpesviridae* [7,8,9,10,11]. Besides, some tRNA genes occurring in these viruses are organized in clusters comprising up to dozens of tRNA genes [2,4,11,12,13,14,15,16]. Interestingly, such tRNA gene organization is particularly common in mitochondrial genomes [17,18,19], but large clusters have been observed in the three domains of life (*Archaea*, *Bacteria* and *Eukarya*) [16,20,21,22,23,24,25]. Studies indicate that the presence of tRNA genes in virus genomes would be to compensate for differences in codon and/or amino acid usage between virus and hosts, favoring an efficient protein synthesis and/or expanding the host range [2,4]. Besides the canonical role of the tRNA genes, some viruses use tRNA genes in regulation of translation, packaging and priming reverse transcription [11]. Since tRNA gene clusters have been shown to be prevalent among the three domains of life and occasionally occurs in viruses, we hypothesized that viruses could play a role in the dispersion of such structures among these organisms. In order to test this hypothesis, and due to the availability of thousands of virus genomes, we performed a large-scale genome survey aiming to identify tRNA genes and tRNA gene clusters in these viruses. This *in-silico* analysis revealed an unsuspected scenario on the prevalence and organization of tRNA genes within viruses, revealing also the presence of tRNA genes in ssDNA and ssRNA genomes.

## 2. Materials and Methods

### 2.1. Genomes Analyzed

The 13,200 viral sequences were retrieved from NCBI FTP (File Transfer Protocol) site (ftp://ftp.ncbi.nlm.nih.gov/genomes/genbank/viral/) in December 2017.

### 2.2. Trna Gene Prediction, Identification, and Classification of tRNA Gene Clusters

The tRNA gene prediction of the data set was mainly performed by ARAGORN v1.2.38 [26] using the standard genetic code. The false-positive rate of this software is correlated with the genome GC (guanine-cytosine) content, being 0.6-3.5 false positives per Gb considering a GC content of 0.2-0.5 and 14 false positives per Gb with a GC content of 0.6 [26]. Since the median GC content of the genomes analyzed here is 0.44, with only one viral family having GC content of 0.62 (*Herpesviridae*), and in general, the viruses have small genomes, the expected rate of false positives is low. In some cases, the isotype and anticodon of the tRNA gene predicted by ARAGORN were not accurately discriminated (i.e., the software indicated two isotypes for a single tRNA gene; e.g., Glu or Gly), requiring a reanalysis using other tRNA gene predictor, tRNAscan-SE 2.0 [27]. The tRNA genes were considered clustered if presenting a tRNA gene density ≥ 2 tRNA/kb [22]. Here we surveyed tRNA gene clusters with a minimum of 15 tRNA genes using an *in-house* script described in a previous study [16]. The identified tRNA gene clusters were classified in groups according to their tRNA gene isotype arrangement (using the single-letter amino acid code abbreviation).

### 2.3. Taxonomic Designation, Sequence Annotation, and Gene Content Analysis

Taxonomic information of the virus sequences was obtained using Kraken v0.10.5 [28], and their annotation performed by Prokka v1.12 using “Viruses” parameter [29]. The gene content and orthologous genes within and flanking (2 kb) the tRNA gene cluster regions were analyzed and compared using GET_HOMOLOGUES v3.0.5 [30] and AcCNET [31] considering 60% of coverage and 40% of identity. The generated networks were visualized using Cytoscape v3.6.0 [32].

### 2.4. Phylogenetic Analysis

The maximum-likelihood tree based on the major capsid protein (MCP) from the *Caudovirales* viruses harboring tRNA gene clusters was reconstructed using PhyML v3.1 [33] with GTR+G+I (general time-reversible + gamma + invariant) substitution model and 100 bootstrap replicates. These amino acid sequences were previously aligned and the low-quality alignment columns were removed by GUIDANCE2 v2.02 [34]. The genetic relationship of the tRNA genes from the tRNA gene clusters was assayed concatenating their nucleotide sequence and submitting to Maximum-likelihood analysis with the GTR substitution model and 100 bootstrap replicates using PhyML v3.1. The substitution models were chosen based on ModelGenerator v85 software [35] and the generated tree figures were edited using iTOL [36].

### 2.5. Codon Bias and Comparative Analyzes of the tRNA Genes

In order to verify whether the codons associated with the tRNA genes from the tRNA gene clusters match with the most used codons in the genome and MCP gene (RSCU >1), we performed a relative synonymous codon usage (RSCU) analysis. A RSCU value of 1 indicates no bias, while values >1 and <1 indicate that the codon occurs more and less frequently than expected, respectively. The RSCU values were calculated using the software CodonW v1.4.2 (https://sourceforge.net/projects/codonw/). The MCP was chosen to be analyzed because is a fundamental component of the virus structure, so it is expected to be highly translated. A high proportion of matching codons would mean that the tRNA gene clusters strongly support the virus fitness.

To explore the possible source of the tRNA gene clusters, all the unique tRNA gene sequences from the tRNA gene clusters were compared to tRNA sequences deposited in the tRNA gene database curated manually by experts (tRNADB-CE) v11.0 [37] considering a global sequence identity of ≥ 90% with CD-HIT [38].

### 2.6. Statistical Analysis

Statistical analyzes were performed with R language R-3.5.2 [39] and RStudio software v1.1.463 [40]. Comparisons between groups were performed using non-parametric tests. A value of *p* < 0.05 was considered statistically significant.

## 3. Results

### 3.1. Data Set Classification and tRNA Gene Distribution

**Figure 1:**
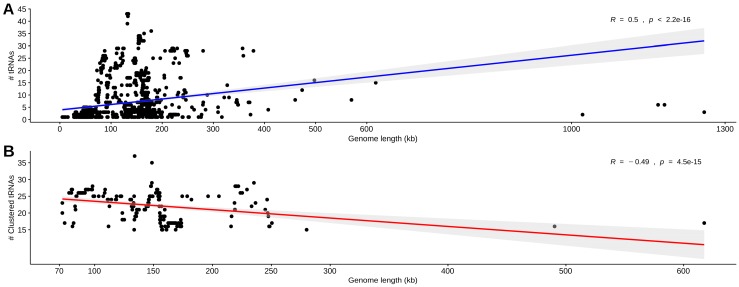
Correlations between tRNA gene number and genome length. (**A**) Correlation between the total number of tRNA genes in each genome and their length (Spearman’s correlation coefficients: *R* = 0.5, *p* = 10^−16^). (**B**) Correlation between the number of clustered tRNA genes and the genome length of viruses carrying tRNA gene clusters (Spearman’s correlation coefficients: *R* = −0.49, *p* = 10^−15^).

In order to define the order/family of the viruses from our data set, we performed a taxonomic designation analysis using Kraken. From 13,200 sequences, 10,249 were designed in 103 families, six of them being the most abundant (~70% of all genomes) (Table S1). To explore the occurrence of tRNA genes in these sequences we applied the ARAGORN software. From the initially 13,200 sequences, approximately 14% (*n =* 1824) presented at least one tRNA gene. The classified organisms and their tRNA gene sequences are provided in Table S2 and Supplementary File. A high proportion of the classified genomes carrying at least one tRNA gene belonged to the *Caudovirales* order (~95%), and the others were from *Herpesvirales*, *Ligamenvirales*, “Megavirales” and *Picornavirales* orders. They were assigned in 22 families, mostly dsDNA, with few ssRNA (+) and ssDNA (Table 1). The ssRNA/DNA viruses are from *Dicistroviridae*, *Inoviridae*, *Luteoviridae*, *Retroviridae* and *Virgaviridae* families [41,42,43,44]. Considering the relative abundance of these genomes, few families presented a high proportion of tRNA genes, such as *Myoviridae* (71%), *Mimiviridae* (83%) and *Phycodnaviridae* (87%). The length of the genomes ranged from ~5 kb to 1.2 Mb, harboring from 1 to 43 tRNA genes (Table 1). The median length of the genomes harboring tRNA genes was significantly higher than those without, 97 kb (IQR, 52–170 kb) and 12 kb (IQR, 5–29 kb) (*p* = 10^−16^), respectively, and the median GC content of the genomes harboring tRNA genes was slightly higher, 43% (IQR, 39–58%) vs. 42% (IQR, 35–48%) (*p* = 10^−16^). A positive correlation was observed between the number of tRNA genes and the genome length (Figure 1A).

### 3.2. Identification, Characterization, and Organization of tRNA Gene Clusters in Phage and Virus Genomes

We identified the presence of tRNA gene clusters in 228/1824 virus genomes harboring tRNA genes (~2% of the total data set and ~12% of the genomes harboring tRNA genes; Table S3). Considering the genomes carrying tRNA genes, those with tRNA gene clusters presented a median length higher than those without tRNA gene clusters, 148 kb (IQR, 106–160 kb) and 77 kb (IQR, 51–171 kb) (*p* = 10^−14^), respectively. 124/228 genomes with tRNA gene clusters have all their tRNA genes clustered, while the others presented a fraction from 55 to 97% (mean of 80%) of their tRNA genes clustered. The majority of genomes from this latter group (*n* = 33) presented 7 tRNA genes outside the clusters, while 22 genomes present only 1 tRNA gene outside the cluster. On the other hand, *Streptomyces* phages carry a total of ~42 tRNA genes, and from these, 16–17 tRNA genes are outside the cluster. The genomes harboring tRNA gene clusters ranged from 72 to 617 kb and the clusters were composed of 15 to 37 tRNA genes, with eight of them harboring the universal 20 tRNA isotypes (most of the clusters harbor 16/20 tRNA isotypes). A negative correlation was observed between the number of the clustered tRNA genes and the genome length (Figure 1B). Interestingly, 44/55 mycobacteriophages present an unusual tRNA isotype, pyrrolysine. The tRNA gene density of these tRNA gene clusters ranged from ~2–10 tRNA/kb. Most of these genomes with tRNA gene clusters are from bacteriophages, while only two are from *Archaea* and *Eukarya* virus. Nevertheless, almost all genomes belong to the *Caudovirales* order, organized in the *Podoviridae* (~4% of the genomes with tRNA gene clusters), *Myoviridae* (~72%) and *Siphoviridae* (~24%) families; and one genome belongs to the “Megavirales” proposed order. The phages were from hosts that encompass five phyla and 34 genera. The phylum *Proteobacteria* represents the majority of the genomes (125/228) and genera (24/34) (Table 2).

Based on the tRNA gene isotype synteny we could define 23 tRNA gene cluster groups and 25 singletons (Figure S1). *Mycobacterium* phages presented three groups, *Bacillus* phages/two groups, *Aeromonas* phages/three groups, *Cronobacter* phages/two groups, *Salmonella* phages/three groups, *Escherichia* phages/three groups, *Vibrio* phages/two groups, *Klebsiella* phages/two groups. The G1, G3, G8, G14, G17, G19, G23 groups are exclusively composed of phages infecting enterobacteria, including *Citrobacter*, *Cronobacter*, *Enterobacter*, *Escherichia*, *Erwinia*, *Klebsiella*, *Salmonella*, *Serratia*, *Shigella* and *Yersinia* genera (*Proteobacteria* phylum). Some groups are genus associated, like G6, G9, and G11 (*Mycobacterium* exclusive), while others present several phage genus hosts, as G1 with several phage genus hosts from *Proteobacteria.* The G1 group is also present in a *Staphylococcus* phage, isolated from *Firmicutes* phylum, however, it mainly differs from the others in G1 group by the deletion of the first four tRNA genes, which correspond to the isotypes [PEMN] (Figure S1). The same groups, defined by the tRNA isotype synteny, were also observed when the tRNA gene sequences were considered (Figure S2). Besides that, some singletons presented relation with tRNA gene cluster groups from same/different genus, e.g., *Streptomyces* phage BRocK and *Gordonia* phage GMA2 (both infecting *Actinobacteria*) with G4 group, composed by *Streptomyces* phages (*Actinobacteria* host); and *Roseobacter* phage DSS3P8 and *Agrobacterium* phage Atu ph07 (both infecting *Proteobacteria*) with G2 group, composed by *Caulobacter* phages (*Proteobacteria* host).

Considering the tRNA gene clusters carried by the *Caudovirales* viruses, their grouping is consistent with the MCP phylogeny, except for the sequences from the G12 group, composed by *Cellulophaga* phages, clustered into two groups (Figure S3). In fact, these phages present a conserved central block of tRNA isotypes, however, the two groups differ by the presence of exclusive block isotype in the right and left sides of the central block (Figure S1). Besides that, these two groups presented differences in genome length (~145 kb vs. ~72 kb) and GC content (0.32 vs. 0.38) (*p* = 0.03) (Table S3). In addition, some genomes presenting unique tRNA isotype arrangement (i.e., not assigned to any tRNA gene cluster group) were grouped considering MCP phylogenetic clusters (e.g., *Synechococcus* phage S-PM2/S-CRM01, *Stenotrophomonas* phage vB SmaS-DLP6/IME-SM1 and *Ralstonia* phage RSP15), suggesting a common origin.

In order to identify whether there was a bias concerning the presence of tRNA gene clusters in virulent or temperate bacteriophages, we search for the presence of integrase genes, which would characterize a temperate one, in the genomes. Among the 226 bacteriophages, only 23 presented an integrase gene, therefore most of the bacteriophages carrying tRNA gene clusters are virulent (*p* = 10^−16^). The temperate bacteriophages were restricted to G2 (*Caulobacter* phages), G6 (*Mycobacterium* phages) and G18 (*Bacillus* phages), besides two singletons (*Roseobacter* and *Sphingobium* phages). The integrase from G6 *Mycobacterium* phages was a serine integrase, while the others harbored tyrosine integrase (Table S3).

### 3.3. Codon Patterns in the tRNA Gene Clusters

**Figure 2:**
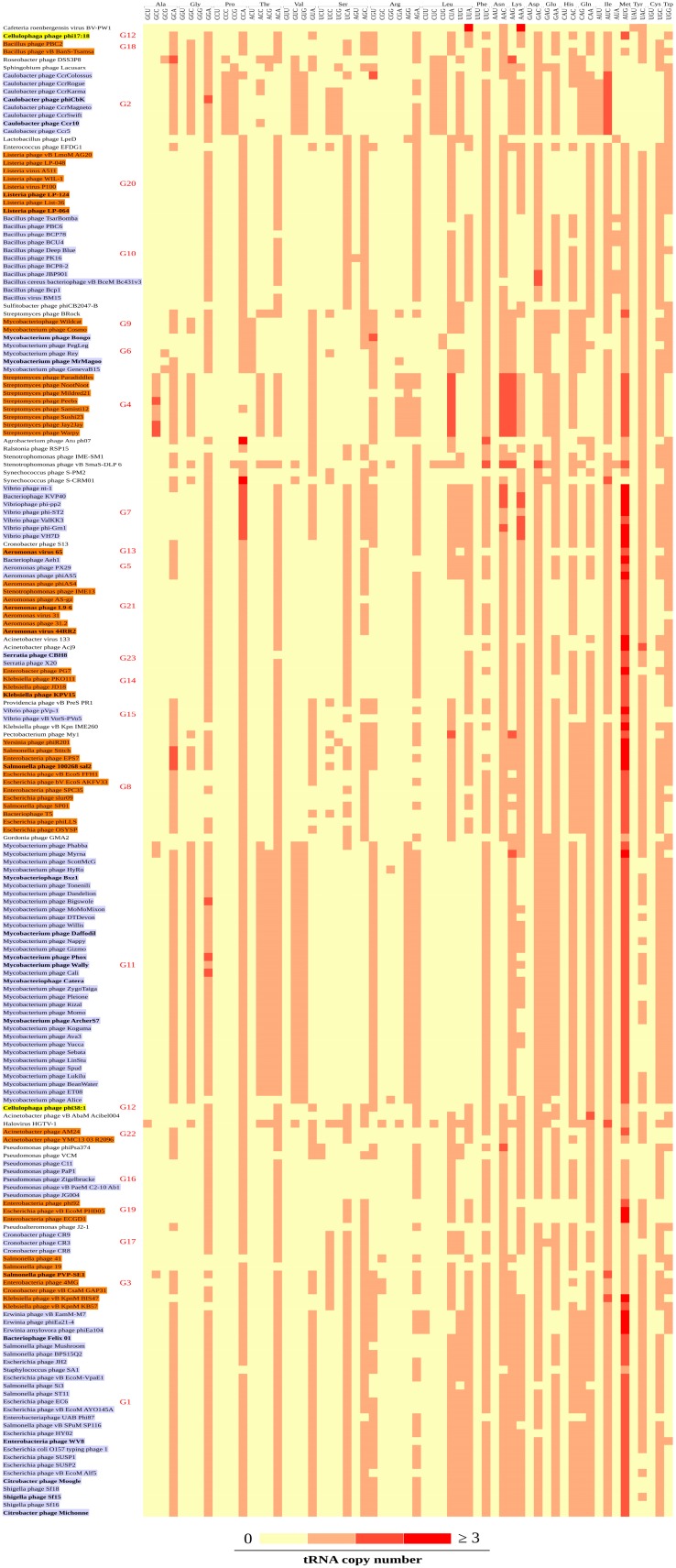
Codon patterns of the tRNA gene clusters. The heatmap shows the tRNA gene copy number (codons and isotypes) of each tRNA gene cluster. The background color of the labels is associated with each tRNA gene cluster group (indicated by the red labels or shown in Figure S1). The yellow background labels represent the *Cellulophaga* phages with the same tRNA gene cluster group. Genomes having identical codon pattern were collapsed, represented by the bold label. A larger version of this figure is provided in Figure S4.

Based on the codons provided by the tRNA genes from the tRNA gene clusters it was possible to discriminate codon patterns among the tRNA gene cluster groups, besides slight intragroup differences. The AUG^Met^ codon was the one presenting, in general, the higher copy number for most tRNA gene clusters (Figure 2). The number of codons per tRNA gene cluster ranged from 5 to 34, however, almost all clusters provided at least 13 codons (Table S4). Only the tRNA gene cluster from Cafeteria roenbergensis virus BV-PW1 presented a low number of codons (*n* = 5) even though the higher number of tRNA genes (*n* = 15). This codon redundancy suggests the occurrence of duplication events in this tRNA gene cluster.

In order to verify a possible contribution of the codons provided by the tRNA genes from the tRNA gene clusters to the host translational machinery, we compared these codons with those most used by the whole genome and MCP gene, an expected highly expressed gene. Therefore, we performed RSCU analyzes based on the whole genomes and MCP genes, comparing them with the codons from the clusters. Among the 228 tRNA gene clusters, 134 provided codons that matched with at least 50% of the MCP codons with RSCU > 1, while that considering the whole genomes, only 39 tRNA gene clusters provided codons that matched with ≥ 50% of the codons most used by the genomes (Table S4). The median percentage of the MCP matching codons was higher (0.50; IQR, 0.39-0.56) than that of the whole genomes (0.42; IQR, 0.31-0.48) (*p* = 10^−15^). These results suggest that the tRNA gene clusters, in general, could participate in the expression of different virus genes, but would provide higher support to highly expressed genes as the MCP gene.

### 3.4. CDS and tRNA Gene Cluster Groups

To find out whether the tRNA gene clusters were associated with particular CDS (coding DNA sequence), we investigated the genes within and flanking the clusters. Most of these genes encoded hypothetical proteins, and a large portion of them was only identified in the carrier virus. Each tRNA gene cluster group presented core genes (i.e., a set of genes present in all clusters from a group), except the G1 group. Among the groups with putative genes: the G4 group presented 13 core genes, one of them being an exonuclease; the G5 group presented 14 core genes, one of them being a dNMP kinase; the G6 group presented four core genes, one of them being an HNH endonuclease; the G9 group presented 14 core genes, being three of them an HNH endonuclease, phosphoribosyl transferase, and tyrosine phosphatase; and the G11 group presented one core gene, a DNA helicase.

In a bipartite network analysis of these CDS, we observed that only a few CDS associated with the tRNA gene cluster groups were shared among them (Figure S5). Besides that, the groups sharing CDS are mostly related to a same bacterial host phylum (Table S5). Considering the 25 singleton clusters, 11 share CDS with other tRNA gene cluster or singletons. Contrasting with this, a bipartite network analysis considering the whole genome gene content revealed a large network including all genomes but *Cafeteria roenbergensis* virus BV-PW1, *Halovirus* HGTV-1 and *Sulfitobacter* phage phiCB2047-B (data not shown). This indicates that the phages harboring tRNA gene cluster, even with different groups, are involved in lateral gene transfer events and may share the same niches.

### 3.5. Source of the Phage tRNA Gene Clusters

To infer the possible source of the bacteriophage tRNA gene clusters we performed a BLAST analysis using as query the tRNA gene cluster regions against bacteria and archaea genomes, and as result none highly similar regions were observed between these two groups. Next, we determined any similarity between the tRNA gene sequences from the clusters (2156 unique sequences) with tRNA gene sequences from bacteria and archaea. 118/2156 tRNA genes from the clusters, comprehending 62 phages, presented high similarity with bacterial tRNA sequences from the same phylum (bacteria/bacteriophage), e.g., *Mycobacterium* phage with similar sequence from *Actinobacteria*. However, in many cases, it was not observed the relation between bacteria phylum and bacteriophage host, e.g., *Mycobacterium* phage Bxz1 with similar sequences to *Cyanobacteria*, *Bacteroidetes* and *Parcubacteria* phyla; and *Streptomyces* phage BRock with similar sequences to *Firmicutes* and *Proteobacteria* phyla (Table S6).

## 4. Discussion

Viruses are dependent on the protein synthesis machinery of their hosts, and therefore, they usually do not harbor translation-related genes. However, eventually, tRNA genes have been identified in virus genomes from lower organisms. The current availability of thousands of virus genomes leads us to perform an *in-silico* survey aiming to identify tRNA genes in viruses. To date, tRNA genes had only been observed in dsDNA viruses [11,45], however, here is revealed a diverse scenario, since tRNA genes were also identified in ssRNA (+) and ssDNA viruses, belonging to *Retroviridae*, *Virgaviridae*, *Luteoviridae*, *Dicistroviridae* and *Inoviridae* families.

Bailly-Bechet et al. [2] analyzed a small set of phages and concluded that the main difference between the phages with and without tRNA genes was at the length of the genome since phages containing tRNAs were significantly longer than those without these genes (average length of 74 kb vs. 32 kb). In the present study with a huge virus genome data set, this same bias was observed, since tRNA genes were observed in longer genomes (average length of 97 kb vs. 12 kb). Stressing again the of correlation between the number of tRNA genes and genome length.

Since the presence of tRNA genes in virus genomes is supposed to be intriguing [2], the presence of large repertoires of these genes is much more intriguing. In this study, considering the large data set analyzed, tRNA gene clusters were only observed in ~2% of the genomes. Interestingly, we observed a correlation between the number of tRNA genes and their organization in clusters. Considering the genomes with 15 or more tRNA genes, 228 (~98%) tended to have their tRNA genes organized in clusters. Besides, although there is a positive correlation between the total number of tRNA genes and the genome length, the inverse occurs considering the clustered tRNA genes. In fact, the organization of tRNA genes in clusters would favor the compaction of the genome, which is a common characteristic of viruses [2,4], especially considering those of small size. Therefore, large viruses would not have a trend to carry highly dense tRNA gene clusters, instead, the tRNA genes are dispersed along the genome. Interestingly, the two recently characterized Tupanviruses that have the highest number of tRNA genes so far identified in viruses (up to 70) [5], presented most of them not arranged in large clusters, as identified in the present study in viruses carrying a large number of tRNA genes. Each one carries 10-11 tRNA genes in small clusters (data not shown). In the present study that considered genomes from 5 kb to 2.5 Mb, tRNA gene clusters were identified in genomes ranging from 72 to 617 kb, being concentrated in those from 100 to 200 kb length, even within viral families with longer genomes. Even though tRNA genes had been identified in several viral families, their arrangement in clusters seems to be restricted to dsDNA viral families: *Myoviridae*, *Podoviridae*, and *Siphoviridae* from *Caudovirales* order. The identification of hundreds of virus genomes harboring tRNA gene clusters contrasts with the previous scenario in which tRNA gene clusters were identified only in few bacteriophages, mainly mycobacteriophages [4,15,16], enlarging significantly the presence and distribution of these structures within viruses.

The *Streptomyces* phages were those presenting the higher number of tRNA genes within and outside the clusters. Curiously, their hosts (*Streptomyces* spp.) are supposed to not carry tRNA arrays [24]. In contrast to this scenario, mycobacteriophages also had a high number of tRNA genes inside and outside the clusters, as well as their hosts (*Mycobacterium* spp.), and in addition, they would act as vectors in the dissemination of tRNA gene clusters in the host [16].

The presence of virus-encoded tRNA genes was associated with selective acquisitions since in several viruses these genes correspond to the codons/amino acids that are enriched in their most expressed genes/proteins, while the remaining tRNA genes would be supplied by the host [2,3,4,11,46]. In fact, in this study, it was shown that different tRNA genes from tRNA gene clusters appear to have been acquired from different bacterial sources. Therefore, it would be expected that the presence of a large repertoire of tRNA genes provided by the virus would ensure greater independence of the host tRNA genes. Indeed, concerning a highly expressed gene, MCP, some tRNA gene clusters presented a high percentage of matching codons that could participate in the translational process. Although the tRNA gene clusters may support the expression of the virus genes, mainly the highly expressed ones, they do not seem to have a fundamental role, and/or they are still under evolutionary process, i.e., a recent acquisition.

Among the bacteriophages harboring the tRNA gene clusters, there was a higher proportion of virulent than temperate ones, and this lifestyle trend was also observed considering tRNA genes [2,4]. Virulent and temperate bacteriophages interact differently with their hosts. Virulent bacteriophages exploit host resources in order to optimize their replicative cycles. The presence of extra tRNA genes would minimize host dependence and extend the host spectrum, improving their fitness [47,48]. In fact, some of the bacteriophages harboring the tRNA gene clusters have been reported presenting a wide range of hosts [49,50,51,52,53,54,55].

Like plasmids, bacteriophages could have a role as vectors of the tRNA arrays/tRNA gene clusters dissemination [16,24]. Indeed, in a study focusing in the *Mycobacterium* genus, there was evidence of the role of mycobacteriophages in the horizontal transfer of tRNA arrays in some *Mycobacterium* species [16]. However, here, we did not find clear evidence supporting this hypothesis considering viruses infecting genera other than *Mycobacterium*. In fact, some mycobacteriophages are temperate phages, whereas most of the viruses carrying tRNA gene clusters, revealed here, are virulent. The temperate lifestyle, which involves a direct genome integration step, raise the chance of traits acquisition by the host, being much more common than virulent ones.

Most of the CDS associated with the tRNA gene clusters are hypothetical, however, in some mycobacteriophages, there was an HNH endonuclease, and it is implicated in the generation of tRNA repertoire diversity [15]. HNH endonuclease belongs to the family of the homing endonuclease that acts as a mobile element, inducing the transfer of its own gene and the flanking regions. It was shown in T4-related phages that the homing endonuclease SegB acts spreading its own gene and the surrounding tRNA genes among related phages [56]. Therefore, the HNH endonuclease in the mycobacteriophages could play the role of dissemination of tRNA gene clusters among related organisms.

## Figures and Tables

**Table 1:** Number and features of viral families harboring tRNA genes.

# Genomes w tRNA/Total Genomes	Family	Order	DNA/RNA	Hosts	Length (Kb)	# tRNAs	Avg GC%
1/24	*Dicistroviridae*	*Picornavirales*	ssRNA(+)	Invertebrates	9	1	45.31
1/9	*Lipothrixviridae*	*Ligamenvirales*	dsDNA	Archaea	32	1	35.66
1/47	*Luteoviridae*	Unassigned	ssRNA(+)	Plants	6	1	50.70
1/7	*Marseilleviridae*	Unassigned	dsDNA	Amoeba	372	2	44.19
1/6	*Nudiviridae*	Unassigned	dsDNA	Insects and marine crustaceans	145	1	25.53
1/97	*Polyomaviridae*	Unassigned	dsDNA	Mammals and birds	5	1	52.35
1/69	*Virgaviridae*	Unassigned	ssRNA(+)	Plants	11	1	48.53
2/48	*Poxviridae*	“Megavirales”	dsDNA	Humans, vertebrates and arthropods	~140	1	51.65
2/8	*Polydnaviridae*	Unassigned	dsDNA	Parasitoid wasps	185–564	7–8	33.72
3/6	*Ascoviridae*	“Megavirales”	dsDNA	Insects	173–198	1–3	42.67
3/47	*Inoviridae*	Unassigned	ssDNA	Bacteria	~5	1	44.30
3/67	*Retroviridae*	Unassigned	ssRNA(+)	Vertebrates	6–8	1	48.80
4/21	*Iridoviridae*	Unassigned	dsDNA	Amphibia, fish and invertebrates	123–190	1	39.92
5/11	*Fuselloviridae*	Unassigned	dsDNA	Thermophilic archaea	~16	1	38.37
5/6	*Mimiviridae*	“Megavirales”	dsDNA	Amoeba	600–1200	2–15	26.12
6/78	*Herpesviridae*	*Herpesvirales*	dsDNA	Vertebrates	119–203	1–18	62.38
7/97	*Adenoviridae*	Unassigned	dsDNA	Vertebrates	33–46	1	52.50
9/84	*Baculoviridae*	Unassigned	dsDNA	Arthropods and crustacean	81–178	1	44.01
21/24	*Phycodnaviridae*	Unassigned	dsDNA	Alga	170–469	2–14	39.77
115/584	*Podoviridae*	*Caudovirales*	dsDNA	Archaea and Bacteria	36–145	1–23	44.78
620/1981	*Siphoviridae*	*Caudovirales*	dsDNA	Archaea and Bacteria	14–280	1–43	55.51
776/1079	*Myoviridae*	*Caudovirales*	dsDNA	Archaea and Bacteria	32–497	1–36	41.59

**Table 2:** Taxonomic information of the hosts of viruses harboring tRNA gene clusters.

# Genomes	Genus	Family	Phylum	Domain
55	*Mycobacterium*	*Mycobacteriaceae*	*Actinobacteria*	*Bacteria*
9	*Streptomyces*	*Streptomycetaceae*	*Actinobacteria*	*Bacteria*
1	*Gordonia*	*Gordoniaceae*	*Actinobacteria*	*Bacteria*
7	*Cellulophaga*	*Flavobacteriaceae*	*Bacteroidetes*	*Bacteria*
2	*Synechococcus*	*Synechococcaceae*	*Cyanobacteria*	*Bacteria*
1	*Halogranum*	*Haloferacaceae*	*Euryarchaeota*	*Archaea*
13	*Bacillus*	*Bacillaceae*	*Firmicutes*	*Bacteria*
1	*Enterococcus*	*Enterobacteriaceae*	*Firmicutes*	*Bacteria*
1	*Staphylococcus*	*Staphylococcaceae*	*Firmicutes*	*Bacteria*
1	*Lactobacillus*	*Lactobacillaceae*	*Firmicutes*	*Bacteria*
10	*Listeria*	*Listeriaceae*	*Firmicutes*	*Bacteria*
5	*Acinetobacter*	*Moraxellaceae*	*Proteobacteria*	*Bacteria*
14	*Aeromonas*	*Aeromonadaceae*	*Proteobacteria*	*Bacteria*
12	*Caulobacter*	*Caulobacteraceae*	*Proteobacteria*	*Bacteria*
4	*Citrobacter*	*Enterobacteriaceae*	*Proteobacteria*	*Bacteria*
5	*Cronobacter*	*Enterobacteriaceae*	*Proteobacteria*	*Bacteria*
3	*Erwinia*	*Enterococcaceae*	*Proteobacteria*	*Bacteria*
23	*Escherichia*	*Enterobacteriaceae*	*Proteobacteria*	*Bacteria*
7	*Klebsiella*	*Enterobacteriaceae*	*Proteobacteria*	*Bacteria*
1	*Pectobacterium*	*Enterobacteriaceae*	*Proteobacteria*	*Bacteria*
1	*Providencia*	*Enterobacteriaceae*	*Proteobacteria*	*Bacteria*
7	*Pseudomonas*	*Pseudomonadaceae*	*Proteobacteria*	*Bacteria*
1	*Roseobacter*	*Rhodobacteraceae*	*Proteobacteria*	*Bacteria*
17	*Salmonella*	*Enterobacteriaceae*	*Proteobacteria*	*Bacteria*
4	*Shigella*	*Enterobacteriaceae*	*Proteobacteria*	*Bacteria*
1	*Sphingobium*	*Sphingomonadaceae*	*Proteobacteria*	*Bacteria*
3	*Stenotrophomonas*	*Xanthomonadaceae*	*Proteobacteria*	*Bacteria*
9	*Vibrio*	*Vibrionaceae*	*Proteobacteria*	*Bacteria*
1	*Yersinia*	*Enterobacteriaceae*	*Proteobacteria*	*Bacteria*
1	*Agrobacterium*	*Rhizobiaceae*	*Proteobacteria*	*Bacteria*
1	*Enterobacter*	*Enterobacteriaceae*	*Proteobacteria*	*Bacteria*
1	*Pseudoalteromonas*	*Pseudoalteromonadaceae*	*Proteobacteria*	*Bacteria*
1	*Ralstonia*	*Burkholderiaceae*	*Proteobacteria*	*Bacteria*
3	*Serratia*	*Enterobacteriaceae*	*Proteobacteria*	*Bacteria*
1	*Sulfitobacter*	*Rhodobacteraceae*	*Proteobacteria*	*Bacteria*
1	*Cafeteria*	*Cafeteriaceae*	*Stramenopiles*	*Eukarya*

## Supplementary Materials

The following are available online at https://www.mdpi.com/1999-4915/11/2/180/s1, Figure S1: tRNA isotype organization, Figure S2: Maximum likelihood tree based on concatenated tRNA gene nucleotide sequences from tRNA gene clusters, Figure S3: Maximum likelihood tree based on Major Capsid Protein (MCP) amino acid sequences, Figure S4: Codon patterns of the tRNA gene clusters, Figure S5: Bipartite network of gene content associated to the tRNA gene clusters, Table S1: Number of genomes and corresponding viral families identified in the data set, Table S2: List of the predicted tRNA genes in the classified virus genomes, Table S3: Features of viral genomes harboring tRNA gene clusters, Table S4: Number of matching codons, Table S5: CDS associated with tRNA gene clusters shared among the phages, Table S6: List of tRNA gene sequences presenting ≥ 90% identity.
