# Supplementary material for: Global In-Silico Scenario of tRNA Genes and Their Organization in Virus Genomes
Source: Viruses. 2019 Feb 21;11(2):180. doi: 10.3390/v11020180 (PMC6409571; doi:10.3390/v11020180)
Supplement: Supplementary file 1 [file viruses-11-00180-s001.zip › viruses-406888-supplementary/TableS5.pdf]

Table S5 CDS associated with tRNA gene clusters shared among the phages

Genome/group (1)	Host phylum (1)	Genome/group (2)	Host phylum (2)	# shared genes	Gene product
Roseobacter phage DSS3P8	*Proteobacteria*	Stenotrophomonas phage vB SmaS-DLP 6	*Proteobacteria*	1	Ribonuclease H-like
Pectobacterium phage My1	*Proteobacteria*	Klebsiella phage IME260	*Proteobacteria*	3	hypothetical (3)
		G8	*Proteobacteria*	4	hypothetical (3)<br>Cell wall hydrolase
		G14	*Proteobacteria*	1	hypothetical
		G15	*Proteobacteria*	1	DUF4326 superfamily
G8	*Proteobacteria*	Klebsiella phage IME260	*Proteobacteria*	3	hypothetical (3)
		G5	*Proteobacteria*	1	hypothetical
		G17	*Proteobacteria*	1	hypothetical
Streptomyces phage Brock	*Actinobacteria*	G4	*Actinobacteria*	1	Permuted papain-like amidase enzyme (NLPC_P60)
Cronobacter phage S13	*Proteobacteria*	G1	*Proteobacteria/Firmicutes*	1	hypothetical
G5	*Proteobacteria*	G7	*Proteobacteria*	1	hypothetical
G6	*Actinobacteria*	G9	*Actinobacteria*	1	HNH endonuclease
G13	*Proteobacteria*	G5	*Proteobacteria*	1	hypothetical
		G21	*Proteobacteria*	1	hypothetical
G14	*Proteobacteria*	G21	*Proteobacteria*	1	hypothetical
		G23	*Proteobacteria*	4	hypothetical (4)
		Acinetobacter phage Acj9	*Proteobacteria*	1	hypothetical
		Acinetobacter phage 133	*Proteobacteria*	1	hypothetical
		G1	*Proteobacteria/Firmicutes*	1	hypothetical
G16	*Proteobacteria*	Pseudomonas phage VCM	*Proteobacteria*	1	hypothetical
		Pseudomonas phage phiPsa374	*Proteobacteria*	1	hypothetical
G20	*Firmicutes*	Enterococcus phage EFDG1	*Firmicutes*	1	PHA02414 superfamily
G21	*Proteobacteria*	G7	*Proteobacteria*	1	hypothetical
G23	*Proteobacteria*	G21	*Proteobacteria*	1	hypothetical
		Acinetobacter phage Acj9	*Proteobacteria*	1	hypothetical
		Acinetobacter phage 133	*Proteobacteria*	1	hypothetical
