# Supplementary material for: Global In-Silico Scenario of tRNA Genes and Their Organization in Virus Genomes
Source: Viruses. 2019 Feb 21;11(2):180. doi: 10.3390/v11020180 (PMC6409571; doi:10.3390/v11020180)
Supplement: Supplementary file 1 [file viruses-11-00180-s001.zip › viruses-406888-supplementary/FigS1.pdf]

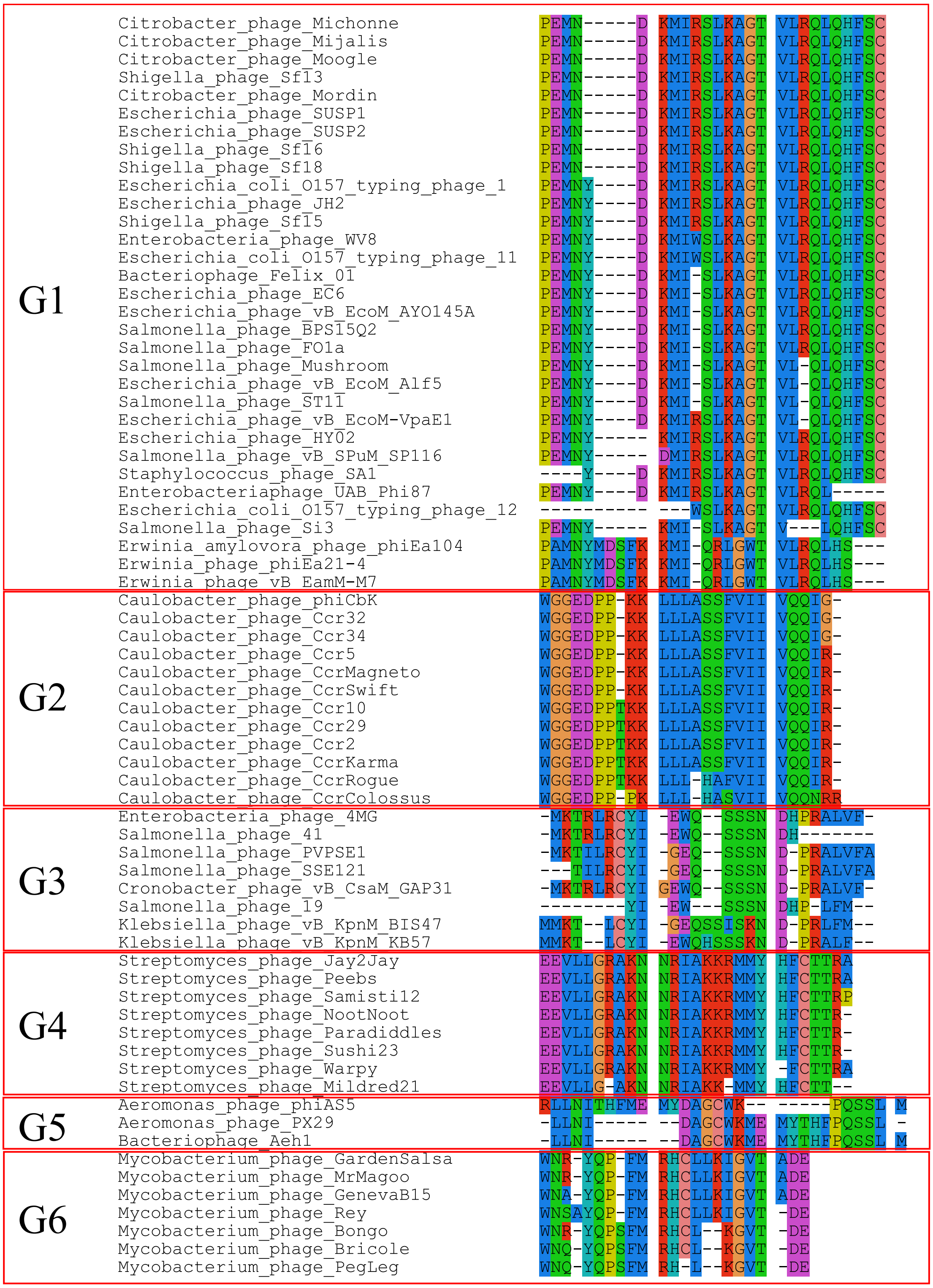

G1
Citrobacter_phage_Michonne
Citrobacter_phage_Mijalis
Citrobacter_phage_Moogle
Shigella_phage_Sf13
Citrobacter_phage_Mordin
Escherichia_phage_SUSP1
Escherichia_phage_SUSP2
Shigella_phage_Sf16
Shigella_phage_Sf18
Escherichia_coli_O157_typing_phage_1
Escherichia_phage_JH2
Shigella_phage_Sf15
Enterobacteria_phage_WV8
Escherichia_coli_O157_typing_phage_11
Bacteriophage_Felix_01
Escherichia_phage_EC6
Escherichia_phage_vB_EcoM_AYO145A
Salmonella_phage_BPS15Q2
Salmonella_phage_FO1a
Salmonella_phage_Mushroom
Escherichia_phage_vB_EcoM_Alf5
Salmonella_phage_ST11
Escherichia_phage_vB_EcoM-VpaE1
Escherichia_phage_HY02
Salmonella_phage_vB_SPuM_SP116
Staphylococcus_phage_SA1
Enterobacteriaphage_UAB_Phi87
Escherichia_coli_O157_typing_phage_12
Salmonella_phage_Si3
Erwinia_amylovora_phage_phiEa104
Erwinia_phage_phiEa21-4
Erwinia_phage_vB_EamM-M7
G2
Caulobacter_phage_phiCbK
Caulobacter_phage_Ccr32
Caulobacter_phage_Ccr34
Caulobacter_phage_Ccr5
Caulobacter_phage_CcrMagneto
Caulobacter_phage_CcrSwift
Caulobacter_phage_Ccr10
Caulobacter_phage_Ccr29
Caulobacter_phage_Ccr2
Caulobacter_phage_CcrKarma
Caulobacter_phage_CcrRogue
Caulobacter_phage_CcrColossus
G3
Enterobacteria_phage_4MG
Salmonella_phage_41
Salmonella_phage_PVPSE1
Salmonella_phage_SSE121
Cronobacter_phage_vB_CsaM_GAP31
Salmonella_phage_19
Klebsiella_phage_vB_KpnM_BIS47
Klebsiella_phage_vB_KpnM_KB57
G4
Streptomyces_phage_Jay2Jay
Streptomyces_phage_Peebs
Streptomyces_phage_Samisti12
Streptomyces_phage_NootNoot
Streptomyces_phage_Paradiddles
Streptomyces_phage_Sushi23
Streptomyces_phage_Warpy
Streptomyces_phage_Mildred21
G5
Aeromonas_phage_phiAS5
Aeromonas_phage_PX29
Bacteriophage_Aeh1
G6
Mycobacterium_phage_GardenSalsa
Mycobacterium_phage_MrMagoo
Mycobacterium_phage_GenevaB15
Mycobacterium_phage_Rey
Mycobacterium_phage_Bongo
Mycobacterium_phage_Bricole
Mycobacterium_phage_PegLeg

Group	Sequence	Alignment
G7	Bacteriophage_KVP40	MPPRLFKKEY MSIWNNDQT- RMGVHCLLS
	Vibrio_phage_phiGrn1	MPPRLFKKEY MSIWNNDQT- RMGVHCLLS
	Vibriophage_phipp2	MPPRLFKKEY MSIWNNDQT- RMGVHCLLS
	Vibrio_phage_VH7D	MPPRLFKKEY MSIW-NDQT- RMGVHCLLS
	Vibrio_phage_phiST2	MPPRLF-KEY MSIW-NDQT- RMGVHCLLS
	Vibrio_phage_ValKK3	MPPRLFKKEY -SIW-NDQT- RMGVHCLLS
	Vibrio_phage_nt1	MPPRLF-KEY MSIWNNDQTA RMGVHCLLS
G8	Bacteriophage_T5	----SYE-FC ND--PMKVAL S-SH-QQ-TI M
	Enterobacteria_phage_SPC35	----SYE-FC NDK-PMKALS ---H-QQGTI M
	Salmonella_phage_SP01	--RSLYE-FC NDK-PMKALS ---H-QQ-TI M
	Escherichia_phage_bV_EcoS_AKFV33	--RSLYEWFC NDK-PMKVAL ---H-QQ-TI M
	Escherichia_phage_vB_EcoS_FFH1	---SLYEWFC ND--PMKVAL ---H-QQ-TI M
	Escherichia_phage_slur09	---SLYEWFC ND--PMK-AL ---H-QQ-TI M
	Escherichia_phage_OSYSP	---SLYE-FC ND--PMKVAL S--H-QQGTI M
	Escherichia_phage_phiLLS	---SLYE-FC ND--PMKAL- ---H-QQGTI M
	Enterobacteria_phage_EPS7	-RSMLYEWFC NDKGPMKVAA L-SHRQQGTI M
	Salmonella_phage_100268_sal2	--SMLYE-WC NDKGPMKVAA LSSHRQQGTI M
	Salmonella_phage_118970_sal2	--SMLYE-WC NDKGPMKVAA LSSHRQQGTI M
	Salmonella_phage_Stitch	--SMLYE-WC NDKGPMKVAA LSSHRQQGTI M
	Yersinia_phage_phiR201	SRSMLYE-WC NDK-PMKVAL ---HRQQGTI M
G9	Mycobacteriophage_Wildcat	WPFMYAVLLG GQDTKCIRSH EEN
	Mycobacterium_phage_Cosmo	WPFMYAVLLG GQDTKCIRSH EEN
G10	Bacillus_cereus_bacteriophage_vB_BceM_Bc431v3	SCCGT-DEDI RSQHPFYLIL -M
	Bacillus_phage_BCP82	SCNDT-G--E RSQHPFYLIL -M
	Bacillus_phage_PK16	SCNDT-G-ES RSQHPFYLIL -M
	Bacillus_virus_BM15	SCNGT-D--E RSQHPFYLIL -M
	Bacillus_phage_PBC6	SCNGT-D--E RSQHPFYILM --
	Bacillus_phage_JBP901	SCNGD-ETDI RSQHPFYLIL -M
	Bacillus_phage_Bcp1	SCNGD-E--I RSQHPFYLIL -M
	Bacillus_phage_BCP78	-CNSG-D-EI RSQHPFYILL -M
	Bacillus_phage_TsarBomba	-CNSGTD-EI RSQHPFYILL -M
	Bacillus_phage_BCU4	-CNSG-DTEI RSQ-PFYILL -M
	Bacillus_phage_Deep_Blue	-CNSG-D-TE RSQHPFYLIL WM
G11	Mycobacteriophage_Bxz1	PWYOMCEH-A FVKE-GTTTG DMIRV----- RQR--
	Mycobacterium_phage_ArcherS7	PWYOMCEH-A FVKE-GTTTG DMIRV----- RQR--
	Mycobacterium_phage_Audrick	PWYOMCEH-A FVKE-GTTTG DMIRV----- RQR--
	Mycobacterium_phage_BeanWater	PWYOMCEH-A FVKE-GTTTG DMIRV----- RQR--
	Mycobacterium_phage_Breeniome	PWYOMCEH-A FVKE-GTTTG DMIRV----- RQR--
	Mycobacterium_phage_Dandelion	PWYOMCEH-A FVKE-GTTTG DMIRV----- RQR--
	Mycobacterium_phage_Drazdys	PWYOMCEH-A FVKE-GTTTG DMIRV----- RQR--
	Mycobacterium_phage_DTDevon	PWYOMCEH-A FVKE-GTTTG DMIRV----- RQR--
	Mycobacterium_phage_Erdmann	PWYOMCEH-A FVKE-GTTTG DMIRV----- RQR--
	Mycobacterium_phage_ET08	PWYOMCEH-A FVKE-GTTTG DMIRV----- RQR--
	Mycobacterium_phage_Gabriel	PWYOMCEH-A FVKE-GTTTG DMIRV----- RQR--
	Mycobacterium_phage_Ghost	PWYOMCEH-A FVKE-GTTTG DMIRV----- RQR--
	Mycobacterium_phage_LRRHood	PWYOMCEH-A FVKE-GTTTG DMIRV----- RQR--
	Mycobacterium_phage_Lukilu	PWYOMCEH-A FVKE-GTTTG DMIRV----- RQR--
	Mycobacterium_phage_Momo	PWYOMCEH-A FVKE-GTTTG DMIRV----- RQR--
	Mycobacterium_phage_Pio	PWYOMCEH-A FVKE-GTTTG DMIRV----- RQR--
	Mycobacterium_phage_Shrimp	PWYOMCEH-A FVKE-GTTTG DMIRV----- RQR--
	Mycobacterium_phage_Tonenili	PWYOMCEH-A FVKE-GTTTG DMIRV----- RQR--
	Mycobacteriophage_Catera	PWYOMCEH-A FVKE-GTTTG DMIRV----- RQRK-
	Mycobacterium_phage_Littleton	PWYOMCEH-A FVKE-GTTTG DMIRV----- RQRK-
	Mycobacterium_phage_Nappy	PWYOMCEH-A FVKE-GTTTG DMIRV----- RQRK-
	Mycobacterium_phage_Rizal	PWYOMCEH-A FVKE-GTTTG DMIRV----- RQRK-
	Mycobacterium_phage_Wally	PWYOMCEH-A FVKE-GTTTG DMIRV----- RQRK-
	Mycobacterium_phage_Bigswole	PWYOMCEH-A FVKEGGTTTG DMIRV----- RQR--
	Mycobacterium_phage_Cali	PWYOMCEH-A FVKEGGTTTG DMIRV----- RQR--
	Mycobacterium_phage_Phox	PWYOMCEH-A FVKEGGTTTG DMIRV----- RQR--
	Mycobacterium_phage_Astraea	PW-OMCEH-A FVKE-GTTTG DMIRV----- RQR--
	Mycobacterium_phage_ErnieJ	PW-OMCEH-A FVKE-GTTTG DMIRV----- RQR--
	Mycobacterium_phage_Koguma	PW-OMCEH-A FVKE-GTTTG DMIRV----- RQR--
	Mycobacterium_phage_LinStu	PW-OMCEH-A FVKE-GTTTG DMIRV----- RQR--
	Mycobacterium_phage_ScottMcG	PW-OMCEH-A FVKE-GTTTG DMIRV----- RQR--
	Mycobacterium_phage_Sebata	PW-OMCEH-A FVKE-GTTTG DMIRV----- RQR--
	Mycobacterium_phage_Spud	PW-OMCEH-A FVKE-GTTTG DMIRV----- RQR--
	Mycobacterium_phage_Ava3	PW-OMCEH-A FVKE-GTTTG DMIRV----- RQRK-
	Mycobacterium_phage_Daffodil	PW-OMCEH-A FVKE-GTTTG DMIRV----- RQRK-
	Mycobacterium_phage_Gizmo	PW-OMCEH-A FVKE-GTTTG DMIRV----- RQRK-
	Mycobacterium_phage_MoMoMixon	PW-OMCEH-A FVKE-GTTTG DMIRV----- RQRK-
	Mycobacterium_phage_Pleione	PW-OMCEH-A FVKE-GTTTG DMIRV----- RQRK-
	Mycobacterium_phage_Willis	PW-OMCEH-A FVKE-GTTTG DMIRV----- RQRK-
	Mycobacterium_phage_Yucca	PW-OMCEH-A FVKE-GTTTG DMIRV----- RQRK-
	Mycobacterium_phage_Zeenon	PW-OMCEH-A FVKE-GTTTG DMIRV----- RQRK-
	Mycobacterium_phage_ZygoTaiga	PW-OMCEH-A FVKE-GTTTG DMIRV----- RQRK-
	Mycobacterium_phage_HyRo	PW-OMCEH-A FVKE-GTTTG DMIRV----- RRQR-
	Mycobacterium_phage_Alice	PWYOMCEH-A FV-------G DMIRV----- RRQRK
	Mycobacterium_phage_Myrna	PY-MACEHGA FVKK-----G DMIRMTVTET RQRK-
	Mycobacterium_phage_Phabba	PW-YMCEHGA FVK------G DMISRTV-ET RQK--

Group	Sequence	Block 1	Block 2	Block 3
G12	Cellulophaga_phage_phi17:2_18	PEQHLLRAKI	GMDTVRMYSN	W-----F
	Cellulophaga_phage_phi17:2	PEQHLLRAKI	GMDTVRMYSN	W-----F
	Cellulophaga_phage_phi4:1_13	PEQHLLRAKI	GMDTVRMYSN	W-----F
	Cellulophaga_phage_phi4:1_18	PEQHLLRAKI	GMDTVRMYSN	W-----F
	Cellulophaga_phage_phi4:1	PEQHLLRAKI	GMDTVRMYSN	W-----F
	Cellulophaga_phage_phi38.1	--------KI	GMDTVRS-SN	FPEQHLR
	Cellulophaga_phage_phi40.1	--------KI	GMDTVRS-SN	FPEQHLR
G13	Aeromonas_phage_65.2	LRLGMMNSKY	WAPTISD	
	Aeromonas_virus_65	LRLGMMNSKY	WAPTISD	
G14	Enterobacter_phage_PG7	IKENDMQHSW	MGPFMRCLT	
	Klebsiella_phage_JD18	Y-KNDMQHSI	WGPMRL--T	
	Klebsiella_phage_KPV15	Y-KNDMQHSI	WGPMRL--T	
	Klebsiella_phage_PKO111	Y-KNDMQHSI	WGPMRL--T	
	Klebsiella_phage_vB_KpnM_KpV477	Y-KNDMQHSI	WGPMLR--T	
G15	Vibrio_phage_pVp1	MKEAMYNDPL	-GTQVIMFRS	HRL
	Vibrio_phage_vB_VorSPVo5	MKEAMYNDPL	SGTQV-----	---
G16	Pseudomonas_phage_C11	QRKLIDMCNP	GFEHT	
	Pseudomonas_phage_JG004	QRKLIDMCNP	GFEHT	
	Pseudomonas_phage_PaP1	QRKLIDMCNP	GFEHT	
	Pseudomonas_phage_vB_PaeM_C210_Ab1	QRKLIDMCNP	GFEHT	
	Pseudomonas_phage_Zigelbrucke	QRKLIDMCNP	GFEHT	
G17	Cronobacter_phage_CR3	TCL-SKNEED	IPMLGQHSSF	
	Cronobacter_phage_CR8	TCL-SKN-ED	IPMLGQHSSF	
	Cronobacter_phage_CR9	TCLYSKN-ED	IPMLGQHSSF	
G18	Bacillus_phage_vB_BanSTsamsa	NDWGQHMFCE	IYL-PATSSG	RR
	Bacillus_phage_PBC2	ND-GQ--LCE	YLIKPATSSG	RR
G19	Enterobacteria_phage_ECGD1	MLSSKYNTGQ	PFLMMI	
	Escherichia_phage_vB_EcoM_PHB05	MLSSKYNTGQ	PFLMMI	
	Enterobacteria_phage_phi92	MLSS-YNTGQ	PFLMI-	
G20	Listeria_phage_List36	CSIDLYTQWK	FSNGRPM	
	Listeria_phage_LP048	CSIDLYTQWK	FSNGRPM	
	Listeria_phage_LP064	CSIDLYTQWK	FSNGRPM	
	Listeria_phage_LP0832	CSIDLYTQWK	FSNGRPM	
	Listeria_phage_LP124	CSIDLYTQWK	FSNGRPM	
	Listeria_phage_LP125	CSIDLYTQWK	FSNGRPM	
	Listeria_phage_vB_LmoM_AG20	CSIDLYTQWK	FSNGRPM	
	Listeria_phage_WIL1	CSIDLYTQWK	FSNGRPM	
	Listeria_virus_A511	CSIDLYTQWK	FSNGRPM	
	Listeria_virus_P100	CSIDLYTQWK	FSNGRPM	
G21	Aeromonas_phage_31.2	LSFNYKHGPT	WISDMMR	
	Aeromonas_phage_44RR2.8t.2	LSFNYKHGPT	WISDMMR	
	Aeromonas_phage_L96	LSFNYKHGPT	WISDMMR	
	Aeromonas_phage_Riv10	LSFNYKHGPT	WISDMMR	
	Aeromonas_phage_SW699	LSFNYKHGPT	WISDMMR	
	Aeromonas_virus_44RR2	LSFNYKHGPT	WISDMMR	
	Aeromonas_phage_phiAS4	LS-NYKHGPT	WISDMMR	
	Stenotrophomonas_phage_IME13	LS-NYKHGPT	WISDMMR	
	Aeromonas_phage_ASgz	LS-NYKHGPT	WI-DMMR	
	Aeromonas_virus_31	LSFNYKHGPT	-ISDMMR	
G22	Acinetobacter_phage_AM24	YINDQAEKFV	SGPLLTMM	
	Acinetobacter_phage_YMC13_03_R2096	YINDQAEKHV	SGPLLTM-	
G23	Serratia_phage_CBH8	YDEKNMQHSP	IWGM-RL	
	Serratia_phage_CHI14	YDEKNMQHSP	IWGM-RL	
	Serratia_phage_X20	YDEKNMQHSP	IWGMLRT	

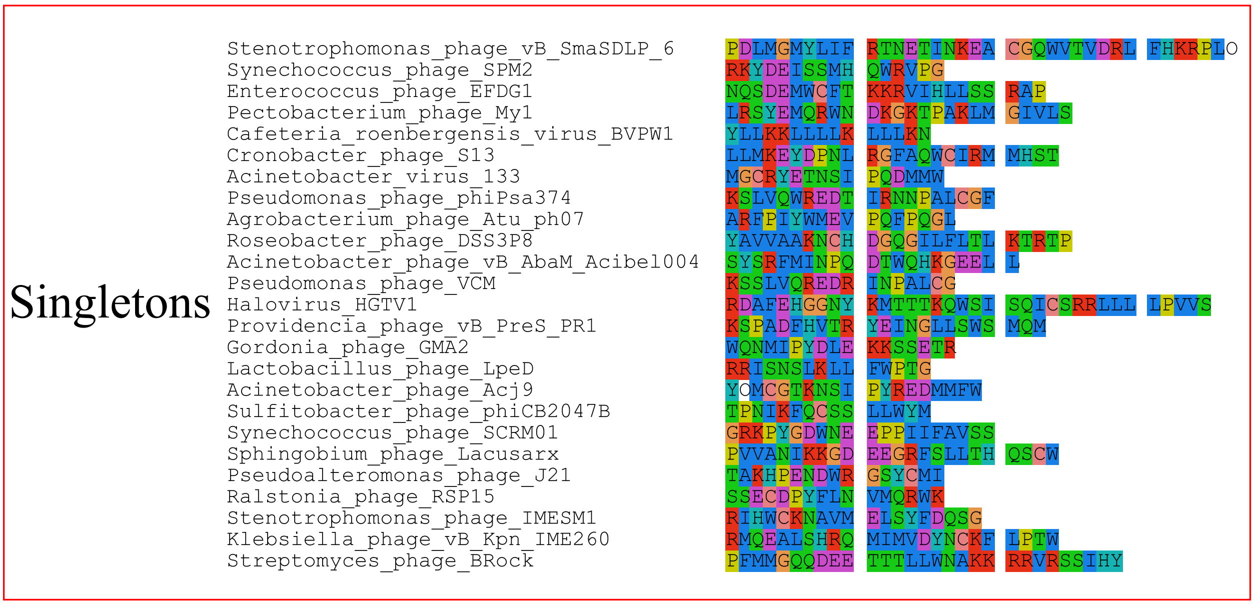
Singletons
Stenotrophomonas_phage_vB_SmaSDLP_6 PDLMGMYLIF RTNETINKEA CGQWVTVDRL FHKRPLO
Synechococcus_phage_SPM2 RKYDEISSMH QWRVPG
Enterococcus_phage_EFDG1 NQSDEMWCFT KKRVIHLLSS RAP
Pectobacterium_phage_My1 LRSYEMQRWN DKGKTPAKLM GIVLS
Cafeteria_roenbergensis_virus_BVPW1 YLLKKLLLLK LLLKN
Cronobacter_phage_S13 LLMKEYDPNL RGFAQWCIRM MHST
Acinetobacter_virus_133 MGCRYETNSI PQDMMW
Pseudomonas_phage_phiPsa374 KSLVQWREDT IRNNPALCGF
Agrobacterium_phage_Atu_ph07 ARFPIYWMEV PQFPQGL
Roseobacter_phage_DSS3P8 YAVVAAKNCH DGQGILFLTL KTRTP
Acinetobacter_phage_vB_AbaM_Acibel004 SYSRFMINPQ DTWQHKGEEL L
Pseudomonas_phage_VCM KSSLVQREDR INPALCG
Halovirus_HGTV1 RDAFEHGGNY KMTTTKQWSI SQICSRRLLL LPVVS
Providencia_phage_vB_PreS_PR1 KSPADFHVTR YEINGLLSWS MQM
Gordonia_phage_GMA2 WQNMIPYDLE KKSSETR
Lactobacillus_phage_LpeD RRISNSLKLL FWPTG
Acinetobacter_phage_Acj9 YOMCGTKNSI PYREDMMFW
Sulfitobacter_phage_phiCB2047B TPNIKFQCSS LLWYM
Synechococcus_phage_SCRM01 GRKPYGDWNE EPPIIFAVSS
Sphingobium_phage_Lacusarx PVVANIKKGD EEGRFSLLTH QSCW
Pseudoalteromonas_phage_J21 TAKHPENDWR GSYCMI
Ralstonia_phage_RSP15 SSECDPYFLN VMQRWK
Stenotrophomonas_phage_IMESM1 RIHWCKNAVM ELSYFDQSG
Klebsiella_phage_vB_Kpn_IME260 RMQEALSHRQ MIMVDYNCKF LPTW
Streptomyces_phage_BRock PFMMGQQDEE TTTLLWNAKK RRVRSSIHY
