# Supplementary material for: Global In-Silico Scenario of tRNA Genes and Their Organization in Virus Genomes
Source: Viruses. 2019 Feb 21;11(2):180. doi: 10.3390/v11020180 (PMC6409571; doi:10.3390/v11020180)
Supplement: Supplementary file 1 [file viruses-11-00180-s001.zip › viruses-406888-supplementary/TableS4.pdf]

Table S4 Number of matching codons

Genome	#Codons provided by the clusters	#MCP codons RSCU >1	#MCP matching codons	% MCP Matching codons / Most used codons	#Genome codons RSCU > 1	#Genome matching codons	%Genome Matching codons / Most used codons
Stenotrophomonas phage vB SmaS-DLP 6	31	23	17	0,74	25	18	0,72
Halovirus HGTV-1	34	24	17	0,71	30	20	0,67
Sphingobium phage Lacusarx	24	23	18	0,78	26	17	0,65
Vibrio phage vB VorS-PVo5	14	24	11	0,46	25	15	0,60
Caulobacter phage Ccr10	24	22	15	0,68	26	15	0,58
Caulobacter phage Ccr2	24	22	15	0,68	26	15	0,58
Caulobacter phage Ccr29	24	22	15	0,68	26	15	0,58
Caulobacter phage CcrKarma	24	22	15	0,68	26	15	0,58
Roseobacter phage DSS3P8	23	24	17	0,71	26	15	0,58
Vibriophage phi-pp2	23	22	14	0,64	26	15	0,58
Erwinia amylovora phage phiEa104	24	23	13	0,57	25	14	0,56
Erwinia phage phiEa21-4	24	24	13	0,54	25	14	0,56
Erwinia phage vB EamM-M7	24	23	12	0,52	24	13	0,54
Caulobacter phage Ccr32	22	22	14	0,64	26	14	0,54
Caulobacter phage Ccr34	22	22	14	0,64	26	14	0,54
Caulobacter phage Ccr5	23	22	14	0,64	26	14	0,54
Caulobacter phage CcrColossus	22	24	15	0,63	26	14	0,54
Caulobacter phage CcrMagneto	23	22	14	0,64	26	14	0,54
Caulobacter phage CcrRogue	23	20	14	0,70	26	14	0,54
Caulobacter phage CcrSwift	23	22	14	0,64	26	14	0,54
Caulobacter phage phiCbK	22	22	14	0,64	26	14	0,54
Mycobacterium phage Myrna	25	24	14	0,58	26	14	0,54
Vibrio phage nt-1	24	23	16	0,70	30	16	0,53
Mycobacterium phage BeanWater	24	24	13	0,54	25	13	0,52
Mycobacterium phage ET08	24	24	13	0,54	25	13	0,52
Streptomyces phage BRock	28	28	17	0,61	27	14	0,52
Bacteriophage KVP40	23	22	14	0,64	29	15	0,52
Vibrio phage pVp-1	20	25	15	0,60	29	15	0,52
Bacillus phage PK16	19	24	13	0,54	28	14	0,50
Escherichia phage SUSP2	24	23	13	0,57	28	14	0,50
Mycobacteriophage Catera	25	25	14	0,56	24	12	0,50
Mycobacteriophage Wildcat	23	27	15	0,56	26	13	0,50
Mycobacterium phage Bigswole	24	24	13	0,54	24	12	0,50
Mycobacterium phage Cosmo	23	26	14	0,54	30	15	0,50
Mycobacterium phage LRRHood	24	24	13	0,54	26	13	0,50
Mycobacterium phage Nappy	25	24	13	0,54	24	12	0,50
Mycobacterium phage Phabba	24	22	12	0,55	26	13	0,50
Shigella phage Sf13	24	23	14	0,61	26	13	0,50
Shigella phage Sf18	24	23	14	0,61	26	13	0,50
Vibrio phage phi-Grn1	23	23	14	0,61	31	15	0,48
Vibrio phage phi-ST2	23	23	14	0,61	31	15	0,48
Bacillus phage Deep Blue	19	25	14	0,56	29	14	0,48
Bacillus virus BM15	18	24	12	0,50	29	14	0,48
Citrobacter phage Mijalis	24	24	14	0,58	25	12	0,48
Citrobacter phage Moogle	24	24	14	0,58	25	12	0,48
Mycobacteriophage Bxz1	24	25	14	0,56	25	12	0,48
Mycobacterium phage ArcherS7	24	24	13	0,54	25	12	0,48
Mycobacterium phage Audrick	24	24	13	0,54	25	12	0,48
Mycobacterium phage Breeniome	24	24	13	0,54	25	12	0,48
Mycobacterium phage Cali	24	24	13	0,54	25	12	0,48
Mycobacterium phage Dandelion	24	24	13	0,54	25	12	0,48
Mycobacterium phage Drazdys	24	24	13	0,54	25	12	0,48
Mycobacterium phage DTDevon	24	24	13	0,54	25	12	0,48
Mycobacterium phage Erdmann	24	24	13	0,54	25	12	0,48
Mycobacterium phage Gabriel	24	24	13	0,54	25	12	0,48
Mycobacterium phage Ghost	24	24	13	0,54	25	12	0,48
Mycobacterium phage HyRo	24	24	12	0,50	25	12	0,48
Mycobacterium phage Littleton	25	25	14	0,56	25	12	0,48
Mycobacterium phage Lukilu	24	24	13	0,54	25	12	0,48
Mycobacterium phage Momo	24	24	13	0,54	25	12	0,48
Mycobacterium phage Phox	24	24	13	0,54	25	12	0,48
Mycobacterium phage Pio	24	25	14	0,56	25	12	0,48
Mycobacterium phage Rizal	25	24	13	0,54	25	12	0,48
Mycobacterium phage Shrimp	24	24	13	0,54	25	12	0,48
Mycobacterium phage Tonenili	24	23	13	0,57	25	12	0,48
Mycobacterium phage Wally	25	24	13	0,54	25	12	0,48
Bacillus phage BCP8-2	18	24	13	0,54	28	13	0,46
Mycobacterium phage Rey	21	23	12	0,52	28	13	0,46
Pseudomonas phage phiPsa374	19	23	12	0,52	28	13	0,46
Staphylococcus phage SA1	22	23	11	0,48	28	13	0,46
Citrobacter phage Mordin	24	23	14	0,61	26	12	0,46
Mycobacterium phage Bongo	18	24	11	0,46	26	12	0,46
Mycobacterium phage Koguma	23	24	12	0,50	26	12	0,46
Shigella phage Sf15	25	23	15	0,65	26	12	0,46
Enterobacteria phage SPC35	20	24	9	0,38	24	11	0,46
Mycobacterium phage Gizmo	24	24	12	0,50	24	11	0,46
Mycobacterium phage Yucca	24	24	12	0,50	24	11	0,46
Bacillus cereus bacteriophage vB BceM Bc431v3	18	25	12	0,48	29	13	0,45
Bacillus phage Bcp1	18	25	11	0,44	29	13	0,45
Bacillus phage BCU4	18	26	12	0,46	29	13	0,45
Bacillus phage JBP901	19	25	13	0,52	29	13	0,45
Bacillus phage TsarBomba	19	26	12	0,46	29	13	0,45
Cronobacter phage S13	22	24	11	0,46	29	13	0,45
Enterobacter phage PG7	17	21	8	0,38	27	12	0,44
Enterococcus phage EFDG1	23	24	13	0,54	27	12	0,44
Escherichia phage SUSP1	24	23	13	0,57	27	12	0,44
Mycobacterium phage Bricole	19	24	11	0,46	27	12	0,44
Citrobacter phage Michonne	24	23	14	0,61	25	11	0,44
Cronobacter phage vB CsaM GAP31	24	21	12	0,57	25	11	0,44
Klebsiella phage vB Kpn IME260	22	21	10	0,48	25	11	0,44
Mycobacterium phage Alice	20	24	10	0,42	25	11	0,44
Mycobacterium phage Astraea	23	24	12	0,50	25	11	0,44
Mycobacterium phage Ava3	24	24	12	0,50	25	11	0,44
Mycobacterium phage Daffodil	24	24	12	0,50	25	11	0,44
Mycobacterium phage ErnieJ	23	24	12	0,50	25	11	0,44
Mycobacterium phage LinStu	23	24	12	0,50	25	11	0,44
Mycobacterium phage MoMoMixon	24	24	12	0,50	25	11	0,44
Mycobacterium phage Pleione	24	24	12	0,50	25	11	0,44
Mycobacterium phage ScottMcG	23	24	12	0,50	25	11	0,44
Mycobacterium phage Sebata	23	24	12	0,50	25	11	0,44
Mycobacterium phage Spud	23	24	12	0,50	25	11	0,44
Mycobacterium phage Willis	24	24	12	0,50	25	11	0,44
Mycobacterium phage Zeenon	24	25	13	0,52	25	11	0,44
Mycobacterium phage ZygoTaiga	24	24	12	0,50	25	11	0,44
Streptomyces phage Samisti12	23	28	15	0,54	30	13	0,43
Mycobacterium phage GenevaB15	21	23	11	0,48	28	12	0,43
Pseudomonas phage VCM	17	22	11	0,50	28	12	0,43
Bacillus phage PBC2	19	23	15	0,65	26	11	0,42
Escherichia coli O157 typing phage 12	17	23	9	0,39	26	11	0,42
Escherichia phage HY02	24	22	12	0,55	26	11	0,42
Escherichia phage JH2	25	24	14	0,58	26	11	0,42
Mycobacterium phage PegLeg	18	24	11	0,46	26	11	0,42
Providencia phage vB PreS PR1	22	25	14	0,56	26	11	0,42
Shigella phage Sf16	24	22	13	0,59	26	11	0,42
Streptomyces phage NootNoot	22	25	14	0,56	31	13	0,42
Bacillus phage BCP78	18	26	11	0,42	29	12	0,41
Bacillus phage PBC6	17	26	13	0,50	29	12	0,41
Pectobacterium phage My1	24	22	11	0,50	27	11	0,41
Salmonella phage vB SPuM SP116	24	24	14	0,58	27	11	0,41
Aeromonas phage phiAS5	22	23	11	0,48	30	12	0,40
Stenotrophomonas phage IME-SM1	19	23	11	0,48	25	10	0,40
Streptomyces phage Jay2Jay	22	26	14	0,54	30	12	0,40
Streptomyces phage Paradiddles	22	26	14	0,54	30	12	0,40
Streptomyces phage Peebs	22	27	14	0,52	30	12	0,40
Streptomyces phage Sushi23	22	27	14	0,52	30	12	0,40
Cellulophaga phage phi38:1	18	24	15	0,63	28	11	0,39
Cellulophaga phage phi4:1	21	24	8	0,33	28	11	0,39
Mycobacterium phage GardenSalsa	21	22	11	0,50	28	11	0,39
Mycobacterium phage MrMagoo	21	22	11	0,50	28	11	0,39
Streptomyces phage Warpy	21	27	13	0,48	31	12	0,39
Bacteriophage Felix 01	24	22	13	0,59	26	10	0,38
Cronobacter phage CR3	19	22	7	0,32	26	10	0,38
Enterobacteria phage ECGD1	14	22	7	0,32	26	10	0,38
Enterobacteria phage WV8	25	23	14	0,61	26	10	0,38
Escherichia coli O157 typing phage 1	25	23	14	0,61	26	10	0,38
Escherichia phage EC6	24	24	14	0,58	26	10	0,38
Escherichia phage vB EcoM AYO145A	24	22	13	0,59	26	10	0,38
Escherichia phage vB EcoM-VpaE1	24	23	13	0,57	26	10	0,38
Salmonella phage BPS15Q2	24	23	14	0,61	26	10	0,38
Salmonella phage FO1a	24	22	13	0,59	26	10	0,38
Yersinia phage phiR201	24	21	11	0,52	26	10	0,38
Bacteriophage Aeh1	21	22	10	0,45	29	11	0,38
Cronobacter phage CR9	19	23	9	0,39	27	10	0,37
Aeromonas phage PX29	21	23	12	0,52	30	11	0,37
Escherichia phage bV EcoS AKFV33	22	23	9	0,39	25	9	0,36
Salmonella phage 118970 sal2	25	22	12	0,55	25	9	0,36
Cellulophaga phage phi17:2_18	21	20	8	0,40	28	10	0,36
Cellulophaga phage phi17:2	21	20	8	0,40	28	10	0,36
Cellulophaga phage phi4:1_13	21	20	8	0,40	28	10	0,36
Cellulophaga phage phi4:1_18	21	20	8	0,40	28	10	0,36
Cellulophaga phage phi40:1	18	20	15	0,75	28	10	0,36
Vibrio phage ValKK3	23	24	14	0,58	31	11	0,35
Bacillus phage vB BanS-Tsamsa	21	20	12	0,60	26	9	0,35
Escherichia phage vB EcoM Alf5	23	25	14	0,56	26	9	0,35
Salmonella phage Mushroom	23	24	13	0,54	26	9	0,35
Salmonella phage PVP-SE1	23	21	10	0,48	26	9	0,35
Salmonella phage ST11	23	23	13	0,57	26	9	0,35
Salmonella phage Stitch	25	21	11	0,52	26	9	0,35
Acinetobacter phage AM24	17	24	13	0,54	27	9	0,33
Acinetobacter phage YMC13 03 R2096	17	23	12	0,52	27	9	0,33
Cronobacter phage CR8	18	22	7	0,32	24	8	0,33

Pseudomonas phage Zigelbrucke	15	25	9	0,36	27	9	0,33
Salmonella phage SSE-121	21	21	10	0,48	27	9	0,33
Streptomyces phage Mildred21	19	25	11	0,44	30	10	0,33
Sulfitobacter phage phiCB2047-B	15	23	9	0,39	27	9	0,33
Escherichia phage OSYSP	21	22	9	0,41	25	8	0,32
Escherichia phage vB EcoS FFH1	20	21	8	0,38	25	8	0,32
Salmonella phage 100268 sal2	25	22	12	0,55	25	8	0,32
Salmonella phage SP01	21	19	6	0,32	25	8	0,32
Acinetobacter phage vB AbaM Acibel004	20	23	9	0,39	29	9	0,31
Bacteriophage T5	20	20	9	0,45	26	8	0,31
Escherichia phage slur09	19	22	7	0,32	26	8	0,31
Klebsiella phage vB KpnM KB57	22	23	12	0,52	26	8	0,31
Pseudoalteromonas phage J2-1	16	24	10	0,42	26	8	0,31
Pseudomonas phage JG004	15	26	9	0,35	26	8	0,31
Salmonella phage Si3	20	25	12	0,48	26	8	0,31
Salmonella phage 41	18	23	9	0,39	27	8	0,30
Serratia phage X20	16	20	6	0,30	24	7	0,29
Vibrio phage VH7D	23	23	14	0,61	31	9	0,29
Gordonia phage GMA2	17	23	9	0,39	28	8	0,29
Pseudomonas phage PaP1	15	25	9	0,36	28	8	0,29
Synechococcus phage S-PM2	16	25	8	0,32	28	8	0,29
Escherichia phage phiLLS	19	21	7	0,33	25	7	0,28
Klebsiella phage PKO111	15	22	4	0,18	25	7	0,28
Klebsiella phage vB KpnM BIS47	20	23	10	0,43	25	7	0,28
Klebsiella phage vB KpnM KpV477	15	23	5	0,22	25	7	0,28
Pseudomonas phage vB PaeM C2-10 Ab1	15	25	9	0,36	25	7	0,28
Salmonella phage 19	14	23	9	0,39	25	7	0,28
Enterobacteriaphage UAB Phi87	20	22	10	0,45	26	7	0,27
Escherichia coli O157 typing phage 11	25	23	14	0,61	26	7	0,27
Listeria phage List-36	17	28	11	0,39	26	7	0,27
Listeria phage LP-048	17	27	10	0,37	26	7	0,27
Listeria phage LP-064	17	28	11	0,39	26	7	0,27
Listeria phage LP-083-2	17	28	11	0,39	26	7	0,27
Listeria phage LP-124	17	28	11	0,39	26	7	0,27
Listeria phage LP-125	17	28	11	0,39	26	7	0,27
Listeria phage vB LmoM AG20	17	28	12	0,43	26	7	0,27
Listeria phage WIL-1	17	27	10	0,37	26	7	0,27
Listeria virus A511	17	28	11	0,39	26	7	0,27
Listeria virus P100	17	28	12	0,43	26	7	0,27
Klebsiella phage JD18	15	23	4	0,17	24	6	0,25
Klebsiella phage KPV15	15	23	5	0,22	24	6	0,25
Pseudomonas phage C11	15	25	9	0,36	28	7	0,25
Serratia phage CBH8	15	22	6	0,27	24	6	0,25
Serratia phage CHI14	15	22	6	0,27	24	6	0,25
Aeromonas phage 31.2	16	23	5	0,22	30	7	0,23
Aeromonas phage 44RR2.8t.2	16	23	5	0,22	30	7	0,23
Aeromonas phage L9-6	16	23	5	0,22	30	7	0,23
Aeromonas phage Riv-10	16	23	5	0,22	30	7	0,23
Aeromonas phage SW69-9	16	23	5	0,22	30	7	0,23
Aeromonas virus 31	15	23	5	0,22	30	7	0,23
Aeromonas virus 44RR2	16	23	5	0,22	30	7	0,23
Agrobacterium phage Atu ph07	14	25	8	0,32	26	6	0,23
Enterobacteria phage 4MG	24	24	13	0,54	26	6	0,23
Enterobacteria phage phi92	13	24	6	0,25	26	6	0,23
Lactobacillus phage LpeD	14	25	7	0,28	26	6	0,23
Aeromonas phage 65.2	16	22	3	0,14	27	6	0,22
Aeromonas virus 65	16	22	3	0,14	27	6	0,22
Escherichia phage vB EcoM PHB05	14	25	8	0,32	27	6	0,22
Synechococcus phage S-CRM01	17	25	10	0,40	27	6	0,22
Acinetobacter phage Acj9	15	25	10	0,40	29	6	0,21
Enterobacteria phage EPS7	26	23	11	0,48	26	5	0,19
Acinetobacter virus 133	14	24	7	0,29	27	5	0,19
Aeromonas phage AS-gz	14	23	5	0,22	27	5	0,19
Ralstonia phage RSP15	16	23	6	0,26	27	5	0,19
Aeromonas phage phiAS4	15	23	6	0,26	28	5	0,18
Stenotrophomonas phage IME13	15	23	6	0,26	28	5	0,18
Cafeteria roenbergensis virus BV-PW1	4	23	4	0,17	27	2	0,07
