# Supplementary material for: Global In-Silico Scenario of tRNA Genes and Their Organization in Virus Genomes
Source: Viruses. 2019 Feb 21;11(2):180. doi: 10.3390/v11020180 (PMC6409571; doi:10.3390/v11020180)
Supplement: Supplementary file 1 [file viruses-11-00180-s001.zip › viruses-406888-supplementary/TableS6.pdf]

Table S6 List of tRNA gene sequences presenting ≥ 90% identity

#ID	tRNA length	Sequence name
Cluster 24672		
0	87nt	tRNA-Bacillus_phage_BCP78-Tyr(gta)
1	87nt	tRNA-Bacillus_phage_BCU4-Tyr(gta)
2	107nt	W141789742|JOTM01000011|Firmicutes|Bacillus
3	107nt	W1610924658|LTAQ01000004|Firmicutes|Bacillus
Cluster 26162		
0	87nt	tRNA-Serratia_phage_X20-Leu(taa)
1	107nt	W1610135943|FACG01000134|Firmicutes|Peptoclostridium
Cluster 31479		
0	86nt	tRNA-Salmonella_phage_PVP-SE1-Leu(taa)
1	106nt	W1610856935|LQOE01000134|Firmicutes|Lactococcus
Cluster 31975		
0	81nt	tRNA-Salmonella_phage_100268_sal2-Leu(tag)
1	105nt	C011616|CP000413|Firmicutes|Lactobacillus
2	105nt	C011639|CP000413|Firmicutes|Lactobacillus
3	105nt	C011868|AE017198|Firmicutes|Lactobacillus
Cluster 32432		
0	86nt	tRNA-Klebsiella_phage_vB_KpnM_BIS47-Leu(tag)
1	105nt	C09107584|CP001674|Betaproteobacteria|Methylovorus
2	105nt	C11114217|CP002252|Betaproteobacteria|Methylovorus
3	105nt	C151104070|LN794158|Betaproteobacteria|Candidatus
Cluster 36399		
0	85nt	tRNA-Pseudomonas_phage_C11-Leu(tag)
1	105nt	W141754977|JNWH01000027|Betaproteobacteria|Delftia
2	105nt	W141755051|JNWI01000023|Betaproteobacteria|Delftia
Cluster 38155		
0	84nt	tRNA-Aeromonas_phage_phiAS5-Leu(tag)
1	105nt	W1610723227|LMDF01000007|Alphaproteobacteria|Caulobacter
Cluster 41419		
0	76nt	tRNA-Stenotrophomonas_phage_vB_SmaS-DLP_6-Phe(gaa)
1	96nt	W09128534|ACLU01000020|Firmicutes|Bacillus
2	96nt	W09128547|ACLU01000025|Firmicutes|Bacillus
3	96nt	W09128611|ACLV01000016|Firmicutes|Bacillus
Cluster 41790		
0	79nt	tRNA-Pseudomonas_phage_C11-Asp(gtc)
1	79nt	tRNA-Pseudomonas_phage_JG004-Asp(gtc)
2	99nt	W141754978|JNWH01000027|Betaproteobacteria|Delftia
3	99nt	W141755052|JNWI01000023|Betaproteobacteria|Delftia
Cluster 43070		
0	76nt	tRNA-Mycobacterium_phage_Myrna-Asp(gtc)
1	98nt	C10110207|CP001744|Planctomycetes|Planctomyces

2	98nt	W09109628|ABUK01000001|Planctomycetes|Planctomyces
Cluster 43905		
0	75nt	tRNA-Mycobacterium_phage_Alice-Pro(tgg)
1	98nt	W09117867|ACCL02000010|Firmicutes|Bryantella
Cluster 44948		
0	77nt	tRNA-Agrobacterium_phage_Atu_ph07-Pro(tgg)
1	98nt	W141220922|AZYH01000006|Bacteroidetes|Cytophaga
2	98nt	W141228737|BAMD01000049|Bacteroidetes|Cytophaga
Cluster 45185		
0	78nt	tRNA-Pseudomonas_phage_C11-Pro(tgg)
1	78nt	tRNA-Pseudomonas_phage_PaP1-Pro(tgg)
2	98nt	W141754981|JNWH01000027|Betaproteobacteria|Delftia
3	98nt	W141755055|JNWI01000023|Betaproteobacteria|Delftia
Cluster 47275		
0	76nt	tRNA-Mycobacterium_phage_Tonenili-Asp(gtc)
1	98nt	W1610735634|LMMR01000005|Actinobacteria|Leifsonia
Cluster 47740		
0	74nt	tRNA-Synechococcus_phage_S-PM2-Pro(tgg)
1	98nt	W1610948307|LVBB01000080|Planctomycetes|Phycisphaerales
Cluster 49461		
0	76nt	tRNA-Acinetobacter_virus_133-Met(cat)
1	97nt	C010423|CP000155|Gammaproteobacteria|Hahella
Cluster 50326		
0	77nt	tRNA-Vibrio_phage_pVp-1-Arg(tct)
1	97nt	C017634|CR354531|Gammaproteobacteria|Photobacterium
2	97nt	W141261176|CCAR010000004|Gammaproteobacteria|Photobacterium
3	97nt	W1610772577|LNTE01000004|Gammaproteobacteria|Photobacterium
Cluster 50605		
0	77nt	tRNA-Stenotrophomonas_phage_vB_SmaS-DLP_6-Arg(acg)
1	97nt	C020930|CP000302|Gammaproteobacteria|Shewanella
2	97nt	C021957|CP000447|Gammaproteobacteria|Shewanella
3	97nt	C08010136|CP000851|Gammaproteobacteria|Shewanella
Cluster 51063		
0	78nt	tRNA-Stenotrophomonas_phage_vB_SmaS-DLP_6-Pro(cgg)
1	97nt	C026950|AE008923|Gammaproteobacteria|Xanthomonas
2	97nt	C027028|CP000050|Gammaproteobacteria|Xanthomonas
3	97nt	C027058|AE008922|Gammaproteobacteria|Xanthomonas
Cluster 52567		
0	76nt	tRNA-Klebsiella_phage_vB_Kpn_IME260-Arg(tct)
1	97nt	C09110565|CP001616|Gammaproteobacteria|Tolumonas
2	97nt	W141215849|AZUK01000002|Gammaproteobacteria|Tolumonas
3	97nt	W1511481617|JWJV01000040|Gammaproteobacteria|Leclercia

Cluster 55830		
0	77nt	tRNA-Cronobacter_phage_CR3-Pro(tgg)
1	77nt	tRNA-Cronobacter_phage_CR8-Pro(tgg)
2	97nt	C121006667|CP003147|Alphaproteobacteria|Pseudovibrio
3	97nt	C121006668|CP003147|Alphaproteobacteria|Pseudovibrio
4	97nt	W09112032|ABXL01000044|Alphaproteobacteria|Pseudovibrio
Cluster 59901		
0	75nt	tRNA-Stenotrophomonas_phage_vB_SmaS-DLP_6-Gly(gcc)
1	97nt	C153001053|CP011211|CandidatusSaccharibacteria|Candidatus
Cluster 59918		
0	77nt	tRNA-Klebsiella_phage_PKO111-Arg(tct)
1	97nt	C161000074|AP013035|Aquificae|Thermosulfidibacter
Cluster 61235		
0	77nt	tRNA-Citrobacter_phage_Michonne-Asn(gtt)
1	77nt	tRNA-Citrobacter_phage_Mordin-Asn(gtt)
2	77nt	tRNA-Escherichia_phage_SUSP1-Asn(gtt)
3	97nt	C161074178|CP014608|Gammaproteobacteria|Obesumbacterium
4	97nt	W121008412|AGCI01000083|Gammaproteobacteria|Hafnia
5	97nt	W1610995009|LXEX01000134|Gammaproteobacteria|Obesumbacterium
Cluster 61456		
0	77nt	tRNA-Aeromonas_phage_65.2-Met(cat)
1	97nt	C161087196|CP015403|Betaproteobacteria|Burkholderiales
2	97nt	W10110999|ADCQ01000047|Betaproteobacteria|Burkholderiales
3	97nt	W11113663|ADCQ01000047|Betaproteobacteria|Burkholderiales
Cluster 63050		
0	76nt	tRNA-Bacillus_phage_BCP8-2-Glu(ttc)
1	76nt	tRNA-Bacillus_phage_Deep_Blue-Glu(ttc)
2	77nt	tRNA-Bacillus_virus_BM15-Glu(ttc)
3	77nt	tRNA-Bacillus_cereus_bacteriophage_vB_BceM_Bc431v3-Glu(ttc)
4	77nt	tRNA-Bacillus_phage_JBP901-Glu(ttc)
5	97nt	W09131060|ACMZ01000145|Firmicutes|Bacillus
6	97nt	W131186879|ARXZ01004211|Firmicutes|Bacillus
7	97nt	W1610794030|LOMO01000001|Firmicutes|Bacillus
Cluster 63051		
0	75nt	tRNA-Bacillus_phage_PBC6-Glu(ttc)
1	97nt	W09131066|ACMZ01000145|Firmicutes|Bacillus
2	97nt	W131186885|ARXZ01004212|Firmicutes|Bacillus
3	97nt	W1610794036|LOMO01000001|Firmicutes|Bacillus
Cluster 63959		
0	75nt	tRNA-Mycobacteriophage_Bxz1-Pro(tgg)
1	73nt	tRNA-Mycobacterium_phage_Bigswole-Pro(tgg)
2	97nt	W11143186|AEPQ01000255|Cyanobacteria|Lyngbya
Cluster 65045		
0	75nt	tRNA-Mycobacteriophage_Catera-Glu(ctc)
1	75nt	tRNA-Mycobacterium_phage_ArcherS7-Glu(ctc)

2	97nt	W121018352|AGSZ01000707|Actinobacteria|Mycobacterium
3	97nt	W1511629795|LDCO01000003|Actinobacteria|Mycobacterium
4	97nt	W1511640156|LDPU01000003|Actinobacteria|Mycobacterium
Cluster 65862		
0	73nt	tRNA-Gordonia_phage_GMA2-Lys(ctt)
1	96nt	C161037750|CP011883|Actinobacteria|Mycobacterium
2	97nt	W121123663|BAED01000061|Actinobacteria|Gordonia
3	96nt	W131060187|ANHL01000007|Actinobacteria|Mycobacterium
Cluster 66172		
0	77nt	tRNA-Bacillus_phage_vB_BanS-Tsamsa-Gln(ttg)
1	97nt	W131004729|AHDZ01000070|Firmicutes|Bacillus
Cluster 66176		
0	75nt	tRNA-Bacillus_phage_BCP8-2-Arg(tct)
1	97nt	W131004736|AHDZ01000070|Firmicutes|Bacillus
Cluster 70777		
0	74nt	tRNA-Streptomyces_phage_BRock-Arg(tct)
1	97nt	W131230103|AUHI01000015|Firmicutes|Halalkalibacillus
Cluster 76100		
0	76nt	tRNA-Pseudomonas_phage_C11-Lys(ttt)
1	77nt	tRNA-Pseudomonas_phage_JG004-Lys(ttt)
2	76nt	tRNA-Pseudomonas_phage_Zigelbrucke-Lys(ttt)
3	97nt	W141754976|JNWH01000027|Betaproteobacteria|Delftia
4	97nt	W141755050|JNWI01000023|Betaproteobacteria|Delftia
Cluster 76101		
0	77nt	tRNA-Pseudomonas_phage_JG004-Phe(gaa)
1	97nt	W141754983|JNWH01000027|Betaproteobacteria|Delftia
2	97nt	W141755057|JNWI01000023|Betaproteobacteria|Delftia
Cluster 80730		
0	76nt	tRNA-Stenotrophomonas_phage_IME-SM1-Ala(tgc)
1	97nt	W1511607578|LCCW01000032|Parcubacteria|Parcubacteria
Cluster 81631		
0	74nt	tRNA-Mycobacterium_phage_Myrna-Lys(ctt)
1	97nt	W1511685519|LFOD01000039|Actinobacteria|Mycobacterium
Cluster 83568		
0	76nt	tRNA-Stenotrophomonas_phage_vB_SmaS-DLP_6-Ala(tgc)
1	96nt	C012545|CP000705|Firmicutes|Lactobacillus
2	96nt	C012566|CP000705|Firmicutes|Lactobacillus
3	96nt	C012578|CP000705|Firmicutes|Lactobacillus
Cluster 83806		
0	78nt	tRNA-Serratia_phage_CBH8-Pro(tgg)
1	97nt	W1610135945|FACG01000134|Firmicutes|Peptoclostridium
Cluster 86236		

0	77nt	tRNA-Ralstonia_phage_RSP15-Lys(ttt)
1	97nt	W1610662946|LJUE01000017|Planctomycetes|Planctomycetes
Cluster 87966		
0	75nt	tRNA-Mycobacterium_phage_Myrna-Met(cat)
1	97nt	W1610816846|LPBG01000047|Betaproteobacteria|Burkholderia
2	97nt	W1610817075|LPBJ01000047|Betaproteobacteria|Burkholderia
3	97nt	W1610825155|LPGQ01000069|Betaproteobacteria|Burkholderia
Cluster 91356		
0	75nt	tRNA-Agrobacterium_phage_Atu_ph07-Val(tac)
1	96nt	C002024|CP000524|Alphaproteobacteria|Bartonella
2	96nt	C003014|BX897699|Alphaproteobacteria|Bartonella
3	96nt	C08002161|AM260525|Alphaproteobacteria|Bartonella
Cluster 92259		
0	74nt	tRNA-Mycobacterium_phage_ArcherS7-Gly(gcc)
1	96nt	C009864|CU207366|Bacteroidetes|Gramella
2	96nt	C10114648|CP001650|Bacteroidetes|Zunongwangia
3	96nt	C161027990|CP010535|Bacteroidetes|Sediminicola
Cluster 92788		
0	76nt	tRNA-Bacillus_phage_PBC6-Thr(tgt)
1	76nt	tRNA-Bacillus_phage_TsarBomba-Thr(tgt)
2	96nt	C013490|L43967|Tenericutes|Mycoplasma
3	96nt	C013970|U00089|Tenericutes|Mycoplasma
4	96nt	C10109513|CP002077|Tenericutes|Mycoplasma
Cluster 93682		
0	76nt	tRNA-Synechococcus_phage_S-PM2-Arg(tct)
1	96nt	C021098|AP008231|Cyanobacteria|Synechococcus
2	96nt	C021141|CP000100|Cyanobacteria|Synechococcus
3	96nt	C151009174|CP006471|Cyanobacteria|Synechococcus
Cluster 94404		
0	76nt	tRNA-Cronobacter_phage_CR9-Thr(tgt)
1	96nt	C08001614|CP001016|Alphaproteobacteria|Beijerinckia
Cluster 96376		
0	74nt	tRNA-Mycobacteriophage_Bxz1-Gly(gcc)
1	74nt	tRNA-Mycobacterium_phage_ScottMcG-Gly(gcc)
2	96nt	C10102650|CP002046|Bacteroidetes|Croceibacter
3	96nt	C151098504|FP476056|Bacteroidetes|Zobellia
4	96nt	W121063876|AJLT01000019|Bacteroidetes|Gillisia
Cluster 98191		
0	77nt	tRNA-Vibrio_phage_vB_VorS-PVo5-Lys(ttt)
1	96nt	C11107054|CP002432|Chrysiogenetes|Desulfurispirillum
2	96nt	W10114085|ADGU01000009|Unknown|bacterium
3	96nt	W11117421|ADGU01000009|Unknown|bacterium
Cluster 98467		
0	73nt	tRNA-Streptomyces_phage_BRock-Asn(gtt)

1	96nt	C11111436|CP002343|Actinobacteria|Intrasporangium
2	96nt	W141142510|AWQS01000365|Actinobacteria|Intrasporangium
3	96nt	W141143049|AWSA01000002|Actinobacteria|Intrasporangium
Cluster 98933		
0	76nt	tRNA-Aeromonas_phage_phiAS5-Lys(ttt)
1	96nt	C11118132|CP002446|Gammaproteobacteria|Pseudoxanthomonas
2	96nt	C151077912|CP011144|Gammaproteobacteria|Pseudoxanthomonas
3	96nt	W131230617|AUHT01000010|Gammaproteobacteria|Lysobacter
Cluster 99194		
0	76nt	tRNA-Agrobacterium_phage_Atu_ph07-Trp(cca)
1	96nt	C11122369|CP001841|Spirochaetes|Treponema
Cluster 104234		
0	76nt	tRNA-Klebsiella_phage_KPV15-His(gtg)
1	96nt	C151101531|HG917868|Firmicutes|Clostridium
Cluster 108267		
0	74nt	tRNA-Bacillus_phage_BCU4-Asp(gtc)
1	74nt	tRNA-Bacillus_phage_Deep_Blue-Asp(gtc)
2	96nt	W09131059|ACMZ01000145|Firmicutes|Bacillus
3	96nt	W131186878|ARXZ01004211|Firmicutes|Bacillus
4	96nt	W1610794029|LOMO01000001|Firmicutes|Bacillus
Cluster 110204		
0	76nt	tRNA-Cronobacter_phage_CR3-Thr(tgt)
1	96nt	W11187313|CAEE01000349|Betaproteobacteria|Chromobacterium
2	96nt	W121126948|CAEE01000349|Betaproteobacteria|Chromobacterium
Cluster 111939		
0	73nt	tRNA-Bacillus_phage_Bcp1-Pro(tgg)
1	96nt	W131004066|AHCU01000068|Firmicutes|Bacillus
2	96nt	W131004258|AHCW01000066|Firmicutes|Bacillus
Cluster 111940		
0	75nt	tRNA-Bacillus_phage_PBC2-Asn(gtt)
1	96nt	W131004727|AHDZ01000070|Firmicutes|Bacillus
Cluster 112773		
0	73nt	tRNA-Enterococcus_phage_EFDG1-Ala(tgc)
1	96nt	W131109850|APIS01000061|Firmicutes|Salinibacillus
Cluster 114642		
0	76nt	tRNA-Streptomyces_phage_BRock-Thr(ggt)
1	96nt	W131179546|ARMM01000005|Actinobacteria|Zimmermannella
Cluster 119675		
0	77nt	tRNA-Stenotrophomonas_phage_vB_SmaS-DLP_6-Pro(tgg)
1	96nt	W141167917|AXUN02000006|Firmicutes|Youngiibacter
Cluster 123412		
0	76nt	tRNA-Pseudomonas_phage_C11-Cys(gca)

1	76nt	tRNA-Pseudomonas_phage_PaP1-Cys(gca)
2	96nt	W141754979|JNWH01000027|Betaproteobacteria|Delftia
3	96nt	W141755053|JNWI01000023|Betaproteobacteria|Delftia
Cluster 123413		
0	78nt	tRNA-Pseudomonas_phage_C11-Asn(gtt)
1	76nt	tRNA-Pseudomonas_phage_JG004-Asn(gtt)
2	96nt	W141754980|JNWH01000027|Betaproteobacteria|Delftia
3	96nt	W141755054|JNWI01000023|Betaproteobacteria|Delftia
Cluster 123414		
0	76nt	tRNA-Pseudomonas_phage_C11-Gly(tcc)
1	96nt	W141754982|JNWH01000027|Betaproteobacteria|Delftia
2	96nt	W141755056|JNWI01000023|Betaproteobacteria|Delftia
Cluster 123415		
0	76nt	tRNA-Pseudomonas_phage_C11-Glu(ttc)
1	76nt	tRNA-Pseudomonas_phage_PaP1-Glu(ttc)
2	76nt	tRNA-Pseudomonas_phage_vB_PaeM_C2-10_Ab1-Glu(ttc)
3	96nt	W141754984|JNWH01000027|Betaproteobacteria|Delftia
4	96nt	W141755058|JNWI01000023|Betaproteobacteria|Delftia
Cluster 124683		
0	76nt	tRNA-Pseudomonas_phage_C11-Ile(gat)
1	76nt	tRNA-Pseudomonas_phage_JG004-Ile(gat)
2	76nt	tRNA-Pseudomonas_phage_PaP1-Ile(gat)
3	96nt	W143017841|JNWH01000027|Betaproteobacteria|Delftia
4	96nt	W143017845|JNWI01000023|Betaproteobacteria|Delftia
Cluster 125389		
0	74nt	tRNA-Mycobacterium_phage_Rey-Asn(gtt)
1	96nt	W1510545397|CSTU01000066|Actinobacteria|Mycobacterium
2	96nt	W1510547779|CSVM01000001|Actinobacteria|Mycobacterium
3	96nt	W1510548503|CSVY01000067|Actinobacteria|Mycobacterium
Cluster 127479		
0	76nt	tRNA-Klebsiella_phage_JD18-Thr(tgt)
1	76nt	tRNA-Klebsiella_phage_vB_KpnM_KpV477-Thr(tgt)
2	96nt	W1511568117|LAIR01000002|Actinobacteria|Luteipulveratus
Cluster 129516		
0	73nt	tRNA-Mycobacteriophage_Bxz1-Lys(ctt)
1	74nt	tRNA-Mycobacterium_phage_BeanWater-Lys(ctt)
2	96nt	W1511616702|LCOM01000012|Parcubacteria|Parcubacteria
Cluster 132072		
0	76nt	tRNA-Enterobacter_phage_PG7-Thr(tgt)
1	96nt	W1610074267|BCWN01000019|Betaproteobacteria|Castellaniella
Cluster 133046		
0	76nt	tRNA-Pseudomonas_phage_phiPsa374-Gly(tcc)
1	96nt	W1610521213|JJOE01000066|Gammaproteobacteria|Pseudomonas

Cluster 136783		
0	72nt	tRNA-Mycobacterium_phage_Myrna-?(Lys|Stop)(tt)
1	96nt	W1610752717|LNDT01000028|Deltaproteobacteria|Bdellovibrionales
Cluster 140608		
0	73nt	tRNA-Listeria_phage_List-36-Gln(ttg)
1	95nt	C002667|AP006627|Firmicutes|Bacillus
2	95nt	C011683|AL592022|Firmicutes|Listeria
3	95nt	C012185|AE017262|Firmicutes|Listeria
Cluster 141221		
0	73nt	tRNA-Bacillus_phage_BCP78-Gln(ttg)
1	95nt	C013494|L43967|Tenericutes|Mycoplasma
2	95nt	C013974|U00089|Tenericutes|Mycoplasma
3	95nt	C10109517|CP002077|Tenericutes|Mycoplasma
Cluster 141691		
0	75nt	tRNA-Serratia_phage_X20-Glu(ttc)
1	95nt	C022624|CP000377|Alphaproteobacteria|Silicibacter
2	95nt	C022625|CP000377|Alphaproteobacteria|Silicibacter
3	95nt	C161085274|CP015230|Alphaproteobacteria|Ruegeria
Cluster 142108		
0	72nt	tRNA-Synechococcus_phage_S-PM2-Val(tac)
1	95nt	C08003964|CP000806|Cyanobacteria|Cyanothece
2	95nt	W11181455|AGJC01000005|Cyanobacteria|Cyanothece
3	95nt	w023474|AAXW01000075|Cyanobacteria|Cyanothece
Cluster 142868		
0	74nt	tRNA-Streptomyces_phage_BRock-Thr(tgt)
1	95nt	C10100660|AP011163|Alphaproteobacteria|Acetobacter
2	95nt	C10100714|AP011121|Alphaproteobacteria|Acetobacter
3	95nt	C10100771|AP011128|Alphaproteobacteria|Acetobacter
Cluster 144983		
0	72nt	tRNA-Synechococcus_phage_S-PM2-Gly(tcc)
1	95nt	C131004519|CP003597|Cyanobacteria|Chroococcidiopsis
2	95nt	W1511401774|JTJC01000084|Cyanobacteria|Scytonema
Cluster 150333		
0	74nt	tRNA-Bacillus_cereus_bacteriophage_vB_BceM_Bc431v3-Asp(gtc)
1	95nt	W131004063|AHCU01000068|Firmicutes|Bacillus
2	95nt	W131004255|AHCW01000066|Firmicutes|Bacillus
Cluster 156045		
0	76nt	tRNA-Pseudomonas_phage_C11-Arg(tct)
1	75nt	tRNA-Pseudomonas_phage_JG004-Arg(tct)
2	75nt	tRNA-Pseudomonas_phage_PaP1-Arg(tct)
3	75nt	tRNA-Pseudomonas_phage_Zigelbrucke-Arg(tct)
4	95nt	W141754975|JNWH01000027|Betaproteobacteria|Delftia
5	95nt	W141755049|JNWI01000023|Betaproteobacteria|Delftia
Cluster 161518		

0	74nt	tRNA-Lactobacillus_phage_LpeD-Asn(gtt)
1	95nt	W1610032431|AYZQ01000002|Firmicutes|Lactobacillus
Cluster 162601		
0	75nt	tRNA-Pseudomonas_phage_phiPsa374-Cys(gca)
1	95nt	W1610521212|JJOE01000066|Gammaproteobacteria|Pseudomonas
Cluster 164387		
0	75nt	tRNA-Synechococcus_phage_S-PM2-Glu(ttc)
1	95nt	W1610748196|LMYZ01000114|Chlorobi|Chlorobi
Cluster 164817		
0	75nt	tRNA-Cronobacter_phage_vB_CsaM_GAP31-Arg(tct)
1	75nt	tRNA-Enterobacteria_phage_4MG-Arg(tct)
2	75nt	tRNA-Salmonella_phage_PVP-SE1-Arg(tct)
3	95nt	W1610856936|LQOE01000134|Firmicutes|Lactococcus
Cluster 165337		
0	72nt	tRNA-Synechococcus_phage_S-CRM01-Pro(tgg)
1	95nt	W1610947072|LUZR01000002|Betaproteobacteria|Burkholderiales
Cluster 170651		
0	71nt	tRNA-Bacillus_phage_Deep_Blue-His(gtg)
1	73nt	tRNA-Bacillus_phage_vB_BanS-Tsamsa-His(gtg)
2	94nt	W131004730|AHDZ01000070|Firmicutes|Bacillus
Cluster 173292		
0	74nt	tRNA-Pseudomonas_phage_C11-Gln(ttg)
1	74nt	tRNA-Pseudomonas_phage_JG004-Gln(ttg)
2	94nt	W141754974|JNWH01000027|Betaproteobacteria|Delftia
3	94nt	W141755048|JNWI01000023|Betaproteobacteria|Delftia
Cluster 177637		
0	74nt	tRNA-Salmonella_phage_PVP-SE1-Cys(gca)
1	94nt	W1610856937|LQOE01000134|Firmicutes|Lactococcus
