# Supplementary material for: Global In-Silico Scenario of tRNA Genes and Their Organization in Virus Genomes
Source: Viruses. 2019 Feb 21;11(2):180. doi: 10.3390/v11020180 (PMC6409571; doi:10.3390/v11020180)
Supplement: Supplementary file 1 [file viruses-11-00180-s001.zip › viruses-406888-supplementary/TableS1.pdf]

Table S1 Number of genomes and corresponding viral families identified in the data set

1	*Alvernaviridae*
1	*Asfarviridae*
1	*Barnaviridae*
1	*Carmotetraviridae*
1	*Corticoviridae*
1	*Gammaflexiviridae*
1	*Guttaviridae*
1	*Malacoherpesviridae*
1	*Marnaviridae*
1	*Mymonaviridae*
1	*Nimaviridae*
1	*Permutotetraviridae*
1	*Plasmaviridae*
1	*Quadriviridae*
1	*Roniviridae*
1	*Sunviridae*
2	*Bidnaviridae*
2	*Globuloviridae*
2	*Hytrosaviridae*
2	*Lavidaviridae*
2	*Picobirnaviridae*
2	*Turriviridae*
3	*Ampullaviridae*
3	*Benyviridae*
3	*Megabirnaviridae*
3	*Ophioviridae*
4	*Alphatetraviridae*
4	*Amalgaviridae*
4	*Hepeviridae*
4	*Nyamiviridae*
5	*Cystoviridae*
6	*Ascoviridae*
6	*Bicaudaviridae*
6	*Mesoniviridae*
6	*Mimiviridae*
6	*Nudiviridae*
6	*Sphaerolipoviridae*
7	*Alloherpesviridae*
7	*Marseilleviridae*
7	*Pneumoviridae*
8	*Birnaviridae*
8	*Chrysoviridae*
8	*Filoviridae*
8	*Nanoviridae*
8	*Polydnaviridae*
9	*Lipothrixviridae*
10	*Rudiviridae*
11	*Fuselloviridae*
11	*Tectiviridae*
14	*Arteriviridae*
14	*Hypoviridae*
14	*Orthomyxoviridae*
15	*Bornaviridae*
15	*Nodaviridae*
18	*Hepadnaviridae*
21	*Iridoviridae*
22	*Leviviridae*
24	*Dicistroviridae*
24	*Phycodnaviridae*
25	*Togaviridae*
29	*Endornaviridae*
31	*Iflaviridae*
34	*Caliciviridae*
36	*Bromoviridae*
38	*Arenaviridae*
38	*Narnaviridae*
39	*Closteroviridae*
41	*Tymoviridae*
47	*Inoviridae*
47	*Luteoviridae*
48	*Astroviridae*
48	*Poxviridae*
50	*Secoviridae*
52	*Partitiviridae*
55	*Alphaflexiviridae*
55	*Coronaviridae*
55	*Genomoviridae*
57	*Paramyxoviridae*
65	*Totiviridae*
67	*Retroviridae*
69	*Tombusviridae*
69	*Virgaviridae*
70	*Caulimoviridae*
77	*Anelloviridae*
78	*Herpesviridae*
84	*Baculoviridae*
89	*Betaflexiviridae*
97	*Adenoviridae*
97	*Polyomaviridae*
100	*Parvoviridae*
121	*Bunyaviridae*
121	*Flaviviridae*

123	*Rhabdoviridae*
125	*Picornaviridae*
145	*Potyviridae*
148	*Papillomaviridae*
180	*Circoviridae*
437	*Geminiviridae*
563	*Microviridae*
584	*Podoviridae*
1079	*Myoviridae*
1981	*Siphoviridae*
2475	*Reoviridae*
