# Supplementary material for: Global In-Silico Scenario of tRNA Genes and Their Organization in Virus Genomes
Source: Viruses. 2019 Feb 21;11(2):180. doi: 10.3390/v11020180 (PMC6409571; doi:10.3390/v11020180)
Supplement: Supplementary file 1 [file viruses-11-00180-s001.zip › viruses-406888-supplementary/TableS3.pdf]

Table S3 Features of viral genomes harboring tRNA gene clusters

Genome	Genome length (bp)	Total tRNAs	tRNAs in cluster	Cluster Length (bp)	BEGIN (bp)	END (bp)	Density (tRNA/kb)	tRNA cluster group	Genome GC%	tRNA cluster GC%	Integrase	Isolation country
Bacteriophage Felix 01	86155	25	25	6482	23699	30180	3.86	G1	0.3901	0.4047	-	-
Citrobacter phage Michonne	90000	25	25	5261	58564	63824	4.75	G1	0.3885	0.4176	-	-
Citrobacter phage Mijalis	87998	25	25	4686	60325	65010	5.55	G1	0.3898	0.421	-	USA
Citrobacter phage Moogle	87999	25	25	4687	60325	65011	5.55	G1	0.3898	0.421	-	-
Citrobacter phage Mordin	89596	25	25	4746	28230	32975	5.48	G1	0.3881	0.4206	-	-
Enterobacteriaphage UAB Phi87	87603	28	21	4070	45	4114	5.16	G1	0.3889	0.4069	-	Spain
Enterobacteria phage WV8	88487	26	26	5571	25986	31556	4.67	G1	0.3889	0.4065	-	Canada
Erwinia amylovora phage phiEa104	84565	27	27	3508	21812	25319	7.7	G1	0.4383	0.4624	-	-
Erwinia phage phiEa21-4	84576	27	27	3506	21823	25328	7.7	G1	0.4381	0.4609	-	-
Erwinia phage vB EamM-M7	84694	27	27	3508	4797	8304	7.7	G1	0.4339	0.4615	-	-
Escherichia coli O157 typing phage 11	88771	26	26	5571	24016	29586	4.67	G1	0.3889	0.4064	-	-
Escherichia coli O157 typing phage 1	88531	26	26	5143	10198	15340	5.06	G1	0.3885	0.4107	-	-
Escherichia phage EC6	86231	25	25	5257	11934	17190	4.76	G1	0.389	0.4134	-	South Korea
Escherichia phage HY02	86252	25	25	4825	25341	30165	5.18	G1	0.3892	0.4114	-	-
Escherichia phage JH2	87712	26	26	5087	69613	74699	5.11	G1	0.3882	0.411	-	Vietnam
Escherichia phage SUSP1	90743	25	25	5730	47552	53281	4.36	G1	0.3976	0.4147	-	USA
Escherichia phage SUSP2	88698	25	25	5468	45779	51246	4.57	G1	0.4016	0.4225	-	USA
Escherichia phage vB EcoM Alf5	87662	24	24	5252	24208	29459	4.57	G1	0.3902	0.4109	-	Lithuania
Escherichia phage vB EcoM AYO145A	87372	25	25	4946	26382	31327	5.05	G1	0.39	0.4143	-	Canada
Escherichia phage vB EcoM-VpaE1	88403	25	25	4838	25483	30320	5.17	G1	0.3894	0.4128	-	Lithuania
Salmonella phage BPS15Q2	89817	25	25	5491	10996	16486	4.55	G1	0.3886	0.4096	-	China
Salmonella phage FO1a	83331	25	25	6482	23621	30102	3.86	G1	0.3893	0.4047	-	-
Salmonella phage Mushroom	87709	24	24	4986	60589	65574	4.81	G1	0.3903	0.4144	-	-
Salmonella phage Si3	84419	22	22	4245	2154	6398	5.18	G1	0.3901	0.4181	-	-
Salmonella phage ST11	82101	24	24	5342	22896	28237	4.49	G1	0.3902	0.4126	-	-
Salmonella phage vB SPuM SP116	87510	25	25	4891	26880	31770	5.11	G1	0.3884	0.4105	-	China
Shigella phage Sf13	87570	25	25	4692	11656	16347	5.33	G1	0.3891	0.4205	-	-
Shigella phage Sf15	88474	26	26	4667	67419	72085	5.57	G1	0.39	0.4225	-	-
Shigella phage Sf16	88580	25	25	5075	16042	21116	4.93	G1	0.3902	0.4114	-	-
Shigella phage Sf18	90270	25	25	4929	66345	71273	5.07	G1	0.39	0.4163	-	-
Staphylococcus phage SA1	147303	25	22	4850	58449	63298	4.54	G1	0.4583	0.4064	-	South Korea
Escherichia coli O157 typing phage 12	88632	26	17	3759	84667	88425	4.52	G1	0.38907	0.40117	-	-
Bacillus cereus bacteriophage vB BceM Bc431v3	158621	20	20	2437	155868	158304	8.21	G10	0.3998	0.461	-	-
Bacillus phage JBP901	159492	20	20	2867	156041	158907	6.98	G10	0.3966	0.4423	-	-
Bacillus phage Bcp1	152778	18	18	2202	30794	32995	8.17	G10	0.39757	0.46367	-	USA
Bacillus phage BCP78	156176	18	18	2744	37	2780	6.55	G10	0.39859	0.43950	-	-
Bacillus phage BCP8-2	159071	18	18	2945	33452	36396	6.11	G10	0.39452	0.43667	-	South Korea
Bacillus phage BCU4	154371	19	19	3699	148023	151721	5.13	G10	0.39857	0.42687	-	-
Bacillus phage Deep Blue	157501	19	19	2461	140342	142802	7.72	G10	0.39947	0.45469	-	-
Bacillus phage PBC6	157147	17	17	2638	37	2674	6.44	G10	0.39910	0.44390	-	-
Bacillus phage PK16	158127	19	19	3005	154511	157515	6.32	G10	0.39858	0.44226	-	South Korea
Bacillus phage TsarBomba	162486	19	19	2594	26558	29151	7.32	G10	0.40074	0.46877	-	Russia
Bacillus virus BM15	165213	18	18	2047	29057	31103	8.79	G10	0.39605	0.46458	-	-

Mycobacteriophage Bxz1	156102	33	26	6668	94073	100740	4.05	G11	0.6477	0.6164	-	-
Mycobacteriophage Catera	153766	34	27	6794	90077	96870	4.12	G11	0.6474	0.6138	-	-
Mycobacterium phage Alice	153401	29	22	6070	90640	96709	3.79	G11	0.6468	0.6115	-	USA
Mycobacterium phage ArcherS7	156558	33	26	7025	91290	98314	3.84	G11	0.6469	0.6115	-	USA
Mycobacterium phage Astraea	154872	32	25	7219	90158	97376	3.6	G11	0.6468	0.6113	-	USA
Mycobacterium phage Audrick	155205	33	26	6544	91423	97966	4.13	G11	0.6466	0.614	-	USA
Mycobacterium phage Ava3	154466	33	26	6394	91216	97609	4.22	G11	0.6476	0.6146	-	USA
Mycobacterium phage BeanWater	154061	33	26	6259	91070	97328	4.31	G11	0.6472	0.6177	-	USA
Mycobacterium phage Bigswole	156514	34	27	6475	93826	100300	4.32	G11	0.6478	0.6161	-	USA
Mycobacterium phage Breeniome	154434	33	26	6605	90723	97327	4.09	G11	0.6478	0.6162	-	USA
Mycobacterium phage Cali	155372	34	27	6865	92217	99081	4.08	G11	0.6472	0.6105	-	-
Mycobacterium phage Daffodil	155034	33	26	6394	91784	98177	4.22	G11	0.6474	0.6146	-	USA
Mycobacterium phage Dandelion	157568	33	26	7371	94760	102130	3.66	G11	0.6473	0.6127	-	USA
Mycobacterium phage Drazdys	156281	33	26	7226	91126	98351	3.74	G11	0.6468	0.6125	-	USA
Mycobacterium phage DTDevon	156754	33	26	6604	93205	99808	4.09	G11	0.6464	0.6154	-	USA
Mycobacterium phage Erdmann	155565	33	26	6262	92444	98705	4.31	G11	0.6475	0.6177	-	USA
Mycobacterium phage ErnieJ	153243	31	25	6539	90472	97010	3.98	G11	0.6473	0.6146	-	USA
Mycobacterium phage ET08	155445	33	26	7108	90457	97564	3.8	G11	0.646	0.6076	-	USA
Mycobacterium phage Gabriel	154474	33	26	6604	90698	97301	4.09	G11	0.6479	0.6155	-	USA
Mycobacterium phage Ghost	155167	33	26	6544	91444	97987	4.13	G11	0.6465	0.614	-	USA
Mycobacterium phage Gizmo	157482	33	26	6827	93378	100204	3.95	G11	0.6464	0.6104	-	USA
Mycobacterium phage HyRo	153714	33	26	6638	90954	97591	4.07	G11	0.6469	0.6125	-	South Africa
Mycobacterium phage Koguma	155759	32	25	6683	89638	96320	3.89	G11	0.6471	0.6104	-	USA
Mycobacterium phage LinStu	153882	32	25	6703	91636	98338	3.88	G11	0.6484	0.6158	-	USA
Mycobacterium phage Littleton	155800	34	27	6807	91780	98586	4.11	G11	0.6472	0.6154	-	USA
Mycobacterium phage LRRHood	154349	33	26	6259	91484	97742	4.31	G11	0.6473	0.6175	-	USA
Mycobacterium phage Lukilu	157034	33	26	6259	92494	98752	4.31	G11	0.6466	0.6177	-	USA
Mycobacterium phage Momo	154553	33	26	6544	90741	97284	4.13	G11	0.6474	0.614	-	USA
Mycobacterium phage MoMoMixon	154573	33	26	6451	91238	97688	4.19	G11	0.6477	0.6151	-	USA
Mycobacterium phage Myrna	164602	36	28	4661	91832	96492	6.22	G11	0.6544	0.5936	-	South Africa
Mycobacterium phage Nappy	156646	35	27	6638	92634	99271	4.22	G11	0.6465	0.6109	-	-
Mycobacterium phage Phabba	164254	33	25	4666	90881	95546	5.57	G11	0.6521	0.5866	-	USA
Mycobacterium phage Phox	154874	35	27	6431	92518	98948	4.35	G11	0.6473	0.6173	-	USA
Mycobacterium phage Pio	156758	34	26	6667	94729	101395	4.05	G11	0.6476	0.6162	-	USA
Mycobacterium phage Pleione	155586	34	26	6742	91912	98653	4	G11	0.6473	0.6133	-	USA
Mycobacterium phage Rizal	153894	35	27	6797	89818	96614	4.12	G11	0.6474	0.6129	-	-
Mycobacterium phage ScottMcG	154017	32	25	6246	90789	97034	4.16	G11	0.6483	0.6177	-	-
Mycobacterium phage Sebata	155286	33	25	6619	92897	99515	3.93	G11	0.6478	0.6166	-	South Africa
Mycobacterium phage Shrimp	155714	34	26	6605	92444	99048	4.09	G11	0.6472	0.6157	-	USA
Mycobacterium phage Spud	154906	32	25	6246	91905	98150	4.16	G11	0.6478	0.6175	-	-
Mycobacterium phage Tonenili	160985	33	26	6711	95715	102425	4.02	G11	0.6409	0.6173	-	USA
Mycobacterium phage Wally	155299	34	27	7330	90313	97642	3.82	G11	0.6467	0.6095	-	USA
Mycobacterium phage Willis	155476	34	26	6394	91680	98073	4.22	G11	0.6472	0.6148	-	USA
Mycobacterium phage Yucca	155582	34	26	7313	91508	98820	3.69	G11	0.6471	0.6095	-	USA
Mycobacterium phage Zeenon	155292	34	26	6879	91594	98472	3.92	G11	0.6474	0.6156	-	USA
Mycobacterium phage ZygoTaiga	157204	34	26	6394	92700	99093	4.22	G11	0.6469	0.615	-	USA

Cellulophaga phage phi17:2_18	145343	23	22	3390	87649	91038	6.49	G12	0.3265	0.4109	-	-
Cellulophaga phage phi17:2	145343	23	22	3390	87649	91038	6.49	G12	0.3265	0.4109	-	Denmark
Cellulophaga phage phi4:1_13	145865	23	22	3603	87196	90798	6.38	G12	0.3267	0.4105	-	-
Cellulophaga phage phi4:1_18	145865	23	22	3603	87196	90798	6.38	G12	0.3267	0.4105	-	-
Cellulophaga phage phi4:1	145865	23	22	3603	87196	90798	6.38	G12	0.3267	0.4105	-	Denmark
Cellulophaga phage phi38:1	72534	20	18	3272	35522	38793	5.50	G12	0.38055	0.41779	-	Denmark
Cellulophaga phage phi40:1	72529	20	18	3270	35520	38789	5.50	G12	0.38055	0.41804	-	Denmark
Aeromonas phage 65.2	236567	18	18	7372	107939	115310	2.44	G13	0.37154	0.38158	-	-
Aeromonas virus 65	235229	18	18	7375	107957	115331	2.44	G13	0.37196	0.38197	-	-
Enterobacter phage PG7	146647	19	19	5345	72104	77448	3.55	G14	0.37473	0.41230	-	China
Klebsiella phage JD18	166313	16	16	4678	57172	61849	3.42	G14	0.39599	0.42219	-	-
Klebsiella phage KPV15	167034	16	16	4712	77431	82142	3.39	G14	0.39522	0.41914	-	-
Klebsiella phage PKO111	168758	16	16	4942	85	5026	3.23	G14	0.39394	0.41845	-	-
Klebsiella phage vB KpnM KpV477	168272	16	16	5401	67989	73389	2.96	G14	0.39321	0.40326	-	Russia
Vibrio phage pVp-1	111506	22	22	7610	37974	45583	2.89	G15	0.3971	0.4336	-	South Korea
Vibrio phage vB VorS-PVo5	80578	15	15	2033	244	2276	7.37	G15	0.40708	0.46850	-	Chile
Pseudomonas phage C11	94109	15	15	2846	22320	25165	5.27	G16	0.49391	0.49438	-	China
Pseudomonas phage JG004	93017	15	15	2626	22657	25282	5.71	G16	0.49261	0.50190	-	Germany
Pseudomonas phage PaP1	91715	15	15	2519	20964	23482	5.95	G16	0.49362	0.49980	-	China
Pseudomonas phage vB PaeM C2-10 Ab1	92777	15	15	2568	21995	24562	5.84	G16	0.49278	0.49494	-	Cote d'Ivoire
Pseudomonas phage Zigelbrucke	92338	15	15	2670	21733	24402	5.61	G16	0.49318	0.50112	-	Switzerland
Cronobacter phage CR3	149273	19	19	4005	144372	148376	4.74	G17	0.50948	0.50637	-	-
Cronobacter phage CR8	149162	18	18	4013	144252	148264	4.48	G17	0.50832	0.50511	-	-
Cronobacter phage CR9	151924	19	19	2892	148134	151025	6.56	G17	0.50556	0.51176	-	-
Bacillus phage vB BanS-Tsamsa	168876	21	21	2039	148615	150653	10.3	G18	0.3432	0.4458	Tyr	-
Bacillus phage PBC2	168689	19	19	2937	148845	151781	6.46	G18	0.34419	0.41539	Tyr	-
Enterobacteria phage ECGD1	148612	16	16	2879	61400	64278	5.55	G19	0.37434	0.40685	-	-
Enterobacteria phage phi92	173276	15	15	2920	51625	54544	5.13	G19	0.39822	0.41721	-	-
Escherichia phage vB EcoM PHB05	147659	16	16	2882	51277	54158	5.55	G19	0.37536	0.41534	-	China
Caulobacter phage Ccr10	219348	29	25	4229	47810	52038	5.91	G2	0.6612	0.6113	Tyr	-
Caulobacter phage Ccr29	229319	29	25	4229	52818	57046	5.91	G2	0.6616	0.6113	Tyr	-
Caulobacter phage Ccr2	220299	29	25	4229	48285	52513	5.91	G2	0.6613	0.6113	Tyr	-
Caulobacter phage Ccr32	215799	29	24	4801	47830	52630	5	G2	0.6616	0.6055	Tyr	-
Caulobacter phage Ccr34	216240	29	24	4801	48270	53070	5	G2	0.6615	0.6055	Tyr	-
Caulobacter phage Ccr5	218729	28	24	4230	47682	51911	5.67	G2	0.6623	0.6113	Tyr	-
Caulobacter phage CcrColossus	279967	28	24	10098	59240	69337	2.38	G2	0.6217	0.6011	Tyr	USA
Caulobacter phage CcrKarma	221828	29	25	4826	47887	52712	5.18	G2	0.6621	0.6055	Tyr	USA
Caulobacter phage CcrMagneto	218929	28	24	4228	47077	51304	5.68	G2	0.6615	0.6119	Tyr	USA
Caulobacter phage CcrRogue	223720	28	24	4452	48466	52917	5.39	G2	0.661	0.6123	Tyr	USA
Caulobacter phage CcrSwift	219216	28	24	4824	47039	51862	4.98	G2	0.6607	0.6066	Tyr	-
Caulobacter phage phiCbK	205504	29	24	4801	155227	160027	5	G2	0.661	0.6055	Tyr	-
Listeria phage List-36	131952	17	17	5659	97557	103215	3	G20	0.36013	0.37692	-	-
Listeria phage LP-048	133048	17	17	5632	27810	33441	3.01	G20	0.35966	0.37820	-	USA
Listeria phage LP-064	135279	17	17	5670	35285	40954	2.99	G20	0.35926	0.37725	-	-
Listeria phage LP-083-2	135831	17	17	5670	31951	37620	2.99	G20	0.35868	0.37725	-	USA
Listeria phage LP-124	135764	17	17	5670	129836	135505	2.99	G20	0.35874	0.37725	-	USA

Listeria phage LP-125	135281	17	17	5670	35284	40953	2.99	G20	0.35930	0.37725	-	-
Listeria phage vB LmoM AG20	133057	17	17	5017	25833	30849	3.38	G20	0.35933	0.38011	-	Canada
Listeria phage WIL-1	134369	17	17	5670	550	6219	2.99	G20	0.35991	0.37954	-	Spain
Listeria virus A511	137619	17	17	5662	25835	31496	3	G20	0.35929	0.37884	-	-
Listeria virus P100	131384	17	17	5661	123712	129372	3	G20	0.36044	0.37979	-	-
Aeromonas phage 31.2	172957	17	17	5321	65027	70347	3.19	G21	0.43916	0.44785	-	-
Aeromonas phage 44RR2.8t.2	173590	17	17	5211	65379	70589	3.26	G21	0.43880	0.43965	-	-
Aeromonas phage AS-gz	162422	15	15	4560	140918	145477	3.28	G21	0.41129	0.44462	-	-
Aeromonas phage L9-6	173578	17	17	4290	66651	70940	3.96	G21	0.43864	0.45664	-	-
Aeromonas phage phiAS4	163875	16	16	4607	102155	106761	3.47	G21	0.41297	0.44389	-	South Korea
Aeromonas phage Riv-10	174311	17	17	5211	66489	71699	3.26	G21	0.43831	0.43984	-	-
Aeromonas phage SW69-9	173097	17	17	4332	66154	70485	3.92	G21	0.43893	0.45406	-	-
Aeromonas virus 31	172963	16	16	5323	65028	70350	3	G21	0.43913	0.44787	-	-
Aeromonas virus 44RR2	173591	17	17	5211	65379	70589	3.26	G21	0.43881	0.43965	-	-
Stenotrophomonas phage IME13	162327	16	16	4903	64250	69152	3.26	G21	0.41179	0.43178	-	China
Acinetobacter phage AM24	97139	18	18	4572	23966	28537	3.93	G22	0.37254	0.39676	-	-
Acinetobacter phage YMC13/03/R2096	98170	17	17	3808	16051	19858	4.46	G22	0.37036	0.42017	-	South Korea
Serratia phage CBH8	171175	16	16	3872	70949	74820	4.13	G23	0.38736	0.43182	-	United Kingdom
Serratia phage CHI14	171175	16	16	3872	70949	74820	4.13	G23	0.38735	0.43182	-	United Kingdom
Serratia phage X20	172450	17	17	4491	70617	75107	3.78	G23	0.38655	0.41327	-	United Kingdom
Cronobacter phage vB CsaM GAP31	147940	25	25	6320	84261	90580	3.96	G3	0.463	0.4729	-	Canada
Enterobacteria phage 4MG	148567	25	25	6615	60264	66878	3.78	G3	0.4633	0.4706	-	-
Klebsiella phage vB KpnM BIS47	147443	24	24	7761	42726	50486	3.09	G3	0.4462	0.4597	-	Poland
Klebsiella phage vB KpnM KB57	142987	25	25	6721	60892	67612	3.72	G3	0.4462	0.4638	-	Russia
Salmonella phage PVP-SE1	145964	25	25	7147	62894	70040	3.5	G3	0.4561	0.4598	-	-
Salmonella phage SSE-121	147745	24	22	4414	28	4441	4.98	G3	0.4529	0.4613	-	-
Salmonella phage 19	94766	16	16	3205	91411	94615	4.99	G3	0.45642	0.47582	-	India
Salmonella phage 41	91721	19	19	6671	85026	91696	2.84	G3	0.45594	0.46005	-	India
Streptomyces phage Jay2Jay	133531	43	27	13685	86672	100356	2.05	G4	0.495	0.4889	-	USA
Streptomyces phage Mildred21	131976	40	23	11824	84530	96353	2.03	G4	0.4947	0.4851	-	USA
Streptomyces phage NootNoot	131086	43	26	12239	83499	95737	2.21	G4	0.5025	0.4987	-	USA
Streptomyces phage Paradiddles	133486	43	26	12754	85797	98550	2.12	G4	0.5012	0.4947	-	USA
Streptomyces phage Peebs	133047	43	27	13783	85888	99670	2.03	G4	0.5006	0.4966	-	USA
Streptomyces phage Samisti12	133710	43	27	13815	87270	101084	2.03	G4	0.4992	0.4971	-	USA
Streptomyces phage Sushi23	133917	43	26	12350	86244	98593	2.19	G4	0.4998	0.4942	-	USA
Streptomyces phage Warpy	132996	42	26	13462	86336	99797	2.01	G4	0.4956	0.4875	-	USA
Aeromonas phage phiAS5	225268	25	24	10076	189405	199480	2.38	G5	0.43	0.4335	-	South Korea
Aeromonas phage PX29	222006	23	22	7897	93173	101069	2.79	G5	0.4204	0.4392	-	-
Bacteriophage Aeh1	233234	23	23	9203	105608	114810	2.5	G5	0.4278	0.4359	-	-
Mycobacterium phage GardenSalsa	80309	21	21	5033	57182	62214	4.17	G6	0.6092	0.5724	Ser	USA
Mycobacterium phage GenevaB15	80123	21	21	5204	56831	62034	4.23	G6	0.6079	0.5694	Ser	USA
Mycobacterium phage MrMagoo	84303	21	21	5034	57182	62215	4.17	G6	0.608	0.5723	Ser	USA
Mycobacterium phage Rey	83724	21	21	5168	57088	62255	4.26	G6	0.6087	0.5739	Ser	USA
Mycobacterium phage Bongo	80228	20	19	5492	54782	60273	3.45	G6	0.61621	0.58430	Ser	USA
Mycobacterium phage Bricole	81128	20	19	5876	54546	60421	3.23	G6	0.61627	0.58696	Ser	USA
Mycobacterium phage PegLeg	80955	19	18	6908	54522	61429	2.60	G6	0.61549	0.58512	Ser	USA

Bacteriophage KVP40	244834	28	28	7449	173137	180585	3.76	G7	0.426	0.4299	-	-
Vibrio phage nt-1	247511	28	28	11081	89870	100950	2.53	G7	0.4133	0.4027	-	-
Vibrio phage phi-Grn1	248605	28	28	7862	37085	44946	3.56	G7	0.4126	0.4144	-	Greece
Vibriophage phi-pp2	246421	28	28	7920	174369	182288	3.54	G7	0.4255	0.4245	-	-
Vibrio phage phi-ST2	250485	26	26	6888	98264	105151	3.77	G7	0.4123	0.421	-	Greece
Vibrio phage ValKK3	248088	26	26	7821	196346	204166	3.32	G7	0.4123	0.4167	-	Malaysia
Vibrio phage VH7D	246964	27	27	6750	110760	117509	4	G7	0.4131	0.4212	-	China
Salmonella phage SP01	117842	22	22	9428	80907	90334	2.33	G8	0.3898	0.4035	-	China
Bacteriophage T5	121750	23	21	9221	28038	37258	2.28	G8	0.3927	0.3885	-	-
Enterobacteria phage EPS7	111382	29	29	12998	29711	42708	2.23	G8	0.399	0.3978	-	-
Enterobacteria phage SPC35	118351	22	21	7625	27768	35392	2.75	G8	0.3939	0.4035	-	-
Escherichia phage bV EcoS AKFV33	108853	23	23	10034	27840	37873	2.29	G8	0.3895	0.4015	-	-
Escherichia phage OSYSP	110901	27	22	9148	899	10046	2.4	G8	0.3916	0.3956	-	-
Escherichia phage phiLLS	107263	21	20	8212	47745	55956	2.44	G8	0.3899	0.3933	-	Mexico
Escherichia phage slur09	111751	21	20	9218	96753	105970	2.17	G8	0.3902	0.3944	-	-
Escherichia phage vB EcoS FFH1	108483	22	21	7719	27984	35702	2.72	G8	0.3924	0.4021	-	-
Salmonella phage 100268 sal2	125114	29	28	9511	29621	39131	2.94	G8	0.4022	0.4008	-	Italy
Salmonella phage 118970 sal2	114180	29	28	9513	28730	38242	2.94	G8	0.4028	0.4006	-	Italy
Salmonella phage Stitch	123475	29	28	9040	28496	37535	3.1	G8	0.4031	0.4067	-	-
Yersinia phage phiR201	122696	26	25	12019	28563	40581	2.08	G8	0.4051	0.4084	-	-
Mycobacteriophage Wildcat	78296	23	23	4440	58812	63251	5.41	G9	0.5685	0.5545	-	-
Mycobacterium phage Cosmo	78229	23	23	4905	58394	63298	4.89	G9	0.5684	0.549	-	South Africa
Acinetobacter phage vB AbaM Acibel004	99730	21	21	3686	59973	63658	5.7	Singleton	0.3727	0.4045	-	-
Cronobacter phage S13	182145	25	24	9685	118148	127832	2.48	Singleton	0.402	0.41	-	-
Enterococcus phage EFDG1	147589	23	23	9118	44887	54004	2.63	Singleton	0.372	0.4001	-	Israel
Halovirus HGTV-1	143855	36	35	11155	42266	53420	3.14	Singleton	0.5036	0.4887	-	-
Klebsiella phage vB Kpn IME260	123490	24	24	9183	30293	39475	2.61	Singleton	0.4537	0.4571	-	China
Pectobacterium phage My1	122024	27	25	12308	28674	40981	2.03	Singleton	0.4061	0.4014	-	-
Providencia phage vB PreS PR1	118537	25	23	9979	28385	38363	2.3	Singleton	0.3952	0.3952	-	Portugal
Pseudomonas phage phiPsa374	97906	20	20	3719	24977	28695	5.38	Singleton	0.4773	0.4784	-	New Zealand
Stenotrophomonas phage IME-SM1	159514	22	19	3696	1537	5232	5.14	Singleton	0.5414	0.534	-	China
Stenotrophomonas phage vB SmaS-DLP 6	168489	38	37	10524	103314	113837	3.51	Singleton	0.5575	0.5348	-	Canada
Streptomyces phage BRock	112523	29	29	11763	95476	107238	2.55	Singleton	0.5232	0.5258	-	USA
Synechococcus phage S-CRM01	178563	36	20	9214	127547	136760	2.17	Singleton	0.3975	0.4371	-	USA
Acinetobacter phage Acj9	169947	19	19	4108	78801	82908	4.62	Singleton	0.40028	0.41334	-	-
Acinetobacter virus 133	159801	16	16	3952	65864	69815	4.04	Singleton	0.39672	0.41827	-	-
Agrobacterium phage Atu ph07	490380	30	17	6615	332329	338943	2.56	Singleton	0.37060	0.39365	-	USA
Cafeteria roenbergensis virus BV-PW1	617453	16	16	2795	509015	511809	5.72	Singleton	0.23343	0.35707	-	USA
Gordonia phage GMA2	103424	17	17	2463	57692	60154	6.90	Singleton	0.53372	0.52659	-	Australia
Lactobacillus phage LpeD	145162	15	15	3368	136332	139699	4.45	Singleton	0.34112	0.38658	-	China
Pseudoalteromonas phage J2-1	142204	16	16	3154	132398	135551	5.07	Singleton	0.37920	0.40679	-	China
Pseudomonas phage VCM	98765	17	17	3871	69564	73434	4.39	Singleton	0.48508	0.48385	-	-
Ralstonia phage RSP15	167619	20	16	7958	87081	95038	2.01	Singleton	0.44702	0.45062	-	-
Sulfitobacter phage phiCB2047-B	74485	16	15	1651	68618	70268	9.08	Singleton	0.42983	0.48577	-	-
Synechococcus phage S-PM2	196280	26	16	4829	118243	123071	3.31	Singleton	0.37820	0.40919	-	United Kingdom
Roseobacter phage DSS3P8	146135	31	25	9638	11149	20786	2.59	Singleton	0.5635	0.5651	Tyr	USA

Sphingobium phage Lacusarx	130138	24	24	4190	66918	71107	5.72	Singleton	0.6017	0.5887	Tyr	Denmark

BEGIN and END columns correspond to the genomic coordenates of the cluster. Tyr: tyrosine recombinase; Ser: serine recombinase.
