# Supplementary material for: Global In-Silico Scenario of tRNA Genes and Their Organization in Virus Genomes
Source: Viruses. 2019 Feb 21;11(2):180. doi: 10.3390/v11020180 (PMC6409571; doi:10.3390/v11020180)
Supplement: Supplementary file 1 [file viruses-11-00180-s001.zip › viruses-406888-supplementary/TableS2.pdf]

Table S2. List of the predicted tRNA genes in the classified virus genomes

Viral families are marked with '*' and genomes with '>'.

*Adenoviridae
>U46933.1 Avian adenovirus CELO, complete genome
1-1tRNA-Ala(tgc)c[39758,39832]
cgacagtgtagctcagccgtaagagcgtcggacttgcactccggaggtcc
ccagatcgaaactgggtactgccga
>AF289262.1 Porcine adenovirus 5, complete genome
1-1tRNA-His(atg)c[20787,20870]
gtggcagaaggccatggatgtggccatgagtcttatggtgcctctgaagg
tagacaccaaggatcttaccttgctgccggacca
>BK001452.1 TPA: Fowl adenovirus 1, complete genome
1-1tRNA-Ala(tgc)c[39758,39832]
cgacagtgtagctcagccgtaagagcgtcggacttgcactccggaggtcc
ccagatcgaaactgggtactgccga
>BK000411.1 TPA: Porcine adenovirus 5, complete genome
1-1tRNA-His(atg)c[20787,20870]
gtggcagaaggccatggatgtggccatgagtcttatggtgcctctgaagg
tagacaccaaggatcttaccttgctgccggacca
>KC493646.1 Fowl adenovirus 5 strain 340, complete genome
1-1tRNA-Leu(taa)c[41720,41794]
cggcggtatagctcagctgtcagagcgtcggacctaaaatccggaggacc
ccagatcaaaactgggtatcgccga
>KF477312.1 Turkey adenovirus 4 isolate TNI1, complete genome
1-1tRNA-Val(aac)c[39044,39118]
cggcggcatagctcaggtggcagagcgtcggactaacagttcggaggtcc
ccagatcgaaactgggtgcagtcga
>KF477313.1 Turkey adenovirus 5 isolate 1277BT, complete genome
1-1tRNA-Phe(aaa)c[39871,39945]
cggcggtgtagctcagccgtaagagcgtcgggtcaaaagtccggaggtcc
ccagatcgaaactgggtatcgccga
*Ascoviridae
>DQ517337.1 Trichoplusia ni ascovirus 2c, complete genome
1-1tRNA-Ile(gat)c[86581,86653]
agtccgttagctcagtggttagagcatcgtgctgataacacgagggtcgt
aggttcaaatcctacatggacta
1-2tRNA-Leu(taa)[126266,126347]
gtcaggttggccgagtggtctaaggcgccagatttaagatctggttccct
cgggagcgtgggttcgaaccccacacctgaca
1-3tRNA-Ile(tat)c[162750,162822]
gcctctttagctcagtggttagagcatcggtcttatacgccggttgtcgt

gagttcgattctcacaagaggta
>KJ755191.1 Heliothis virescens ascovirus 3f isolate LD135790, complete genome
1−1tRNA−Ile(tat)c[43866,43938]
gtctcggtggcgcagtcggtagcgcacggtgcttatatcgccgtggtcgt
gagttcgatcctcacccgagaca
>JX491653.1 Heliothis virescens ascovirus 3g, complete genome
1−1tRNA−Ile(tat)c[46219,46291]
gtctcggtggcgcagttggtagcgcacggtgcttatatcgccgcggtcgt
gagttcgatcctcacccgagaca
*Baculoviridae
>U59461.2 Mamestra configurata NPV−A strain 90/2, complete genome
1−1tRNA−Ile(tat)[17778,17852]
ggtgacgctggtgtaatggttagcatccgtgacttataatcgcgaggagg
gcggttcgattccgccagttgctgc
>AF162221.1 Xestia c−nigrum granulovirus genome, complete sequence
1−1tRNA−Asp(gtc)c[60066,60137]
tcctcggtagtatagtggtgagtatgtccgcctgtcacgcgggagaccag
ggttcgattccctgccgaggag
>L22858.1 Autographa californica nucleopolyhedrovirus clone C6, complete genome
1−1tRNA−Gln(ctg)[107991,108073]
gtctcagtagctcaaggtagagcgtagcgctctggatcgtatagatcttg
ctaaggttgtgagttcaagtctcgcctgagata
>AY349019.1 Neodiprion lecontei NPV, complete genome
1−1tRNA−Ile(aat)c[11456,11529]
ggcccattagctcagttggt.agagcgtcgtgctaataacgcgaaggtcg
aaggttcgatcctttcatgggcca
>DQ486030.3 Antheraea pernyi nucleopolyhedrovirus, complete genome
1−1tRNA−Lys(ttt)[95704,95789]
ttctcagtagctcaacggttagagcgtagcacttttcatcgctttagatg
ttgctaaggttgcaagttcgaatcttgcctgagata
>DQ813662.2 Anticarsia gemmatalis nucleopolyhedrovirus, complete genome
1−1tRNA−Thr(agt)c[28449,28532]
gtctcagtagcttaatggttagagcgtggcgctagttatgtaaacatgat
gacaaggttgcgagttcaagcctcgcctgagata
>EU255577.1 Helicoverpa armigera granulovirus, complete genome
1−1tRNA−Asp(gtc)c[57007,57078]
tcctcggtagtatagtggtgagtatgtccgcctgtcacgcgggagaccag
ggttcgatttcttgccgaggag
>JQ798165.1 Mamestra brassicae MNPV strain K1, complete genome
1−1tRNA−Ile(tat)[17401,17475]
ggtgacgctggtgtaatggttagcatgagtgacttataatcgcgacgatg
gtggttcgattccgccagttgctgc
>KR815466.1 Anticarsia gemmatalis multicapsid nucleopolyhedrovirus isolate AgMNPV−37, complete genome

1-1tRNA-Thr(agt)c[27782,27865]
gtctcagtagcttaatggttagagcgtggcgctagttatgtaaacatgat
gacaaggttgcgagttcaagcctcgcctgagata
*Dicistroviridae
>DQ288865.1 Homalodisca coagulata virus-1, complete genome
1-1tRNA-Ala(cgc)[2402,2479]
cggacagctggttagtgtgtttgatgatttcgcacaacatcaagattttg
ctcaggttccaaaccctgagctatttga
*Fuselloviridae
>AY370762.1 Fusellovirus SSV2, complete genome
1-1tRNA-Gly(ccc)[2557,2630]
ggggtcttagggggtgtcccctaatgccggcctcccaagccggtgatcc
cgggttcaaatcccggcggccgca
>EU030938.1 Sulfolobus spindle-shaped virus 4, complete genome
1-1tRNA-Glu(ttc)[7486,7563]
gtgggggttttagggggtgtccccccctaatgcgggcctttcgagcccgtg
acccgggttcaaatcccggccgcggcac
>EU030939.1 Sulfolobus spindle-shaped virus 5, complete genome
1-1tRNA-Gln(ctg)c[7518,7593]
aacgggggttgtgggggcgttacccccacagggctctggcccctgggacc
agggttcgaatccctgcccggctacc
>FJ870915.1 Sulfolobus spindle-shaped virus 6, complete genome
1-1tRNA-Gln(ctg)c[3758,3833]
aacgggggttgtgggggcgttccccccacagggctctggcccctgggacc
agggttcgaatccctgcccggctacc
>KY579375.1 Sulfolobus spindle-shaped virus 3 strain REY 15/4, complete genome
1-1tRNA-Glu(ttc)[3397,3474]
gtgggggttttagggggtgtccccccctaatgcgggcctttcgagcccgtg
acccgggttcaaatcccggccgcggcac
*Herpesviridae
>U97553.2 Murine herpesvirus 68 strain WUMS, complete genome
1-2tRNA-Gln(ctg)[487,562]
ggccagggtagctcaatcggtagagcagcggttcctggagtccgctggtt
ctcggttcaagcccgagccctggttg
1-3tRNA-Cys(aca)[894,967]
gtcggggtagctcaaatggtagagtggcaggccaacatagccagcagatc
tcggttcaaacccgagccctgacc
1-4tRNA-Met(cat)[1182,1255]
gtcggggtagctcaattggtagagcggcaggctcatcccctgcaggttct
cggttcaatcccgggtcccgacgc
1-5tRNA-Thr(agt)[1588,1659]
gccagggtagctcaattggtagagcatcaggctagtatcctgtcggttcc
ggttcaagtccgggccctggtt
1-6tRNA-Trp(cca)[3675,3749]

tgccagcgtagctcaattgttagagcagcggccaccaagcctgcaggttc
tcggttcaagtccgggcgctggcat
1–7tRNA–Arg(cct)[4946,5031]
ggccagggtagctcaattggtagagcggcagacaccacctacgtggtcta
gtctgtggatctcggttcaagtccgagtcctggcca
1–8tRNA–Gly(gcc)[5384,5460]
caccagagtggctcacctggtagagcaccaggctgcccatcctgttggtt
ctcggttcaaatccgagctctggtgac
>AJ004801.1 Bovine herpesvirus type 1.1 complete genome
1–1tRNA–Pro(cgg)c[103131,103223]
cggcgcccgcgcgggctcggcggcccccgggctcgggcccctgggcgccg
ggcgtcggggcgcgaggcccgggctcgggcccccgggcgccgg
1–2tRNA–Pro(cgg)[134995,135087]
cggcgcccgcgcgggctcggcggcccccgggctcgggcccctgggcgccg
ggcgtcggggcgcgaggcccgggctcgggcccccgggcgccgg
>DQ149153.1 Cercopithecine herpesvirus 16 strain X313, complete genome
1–1tRNA–Val(gac)c[78214,78308]
tcgggtctggccgagctggagcgcttcgaggccctcgacgcggccctgcg
gcgcgagctcgagagcgaggcggcgttcggacccgcgggcgcggc
1–2tRNA–Pro(cgg)[84794,84874]
gtgtccccgggggccggcgcggtccggacgttctcgggggacgtcgcggc
ggacccccgggcctccttcgggggaggcgtg
>KJ668231.1 Falconid herpesvirus 1 strain S–18, complete genome
1–1tRNA–Arg(cct)[4508,4586]
tccctcgttagttcagtggtagaatacccgcctcctctccgggcgggaga
ccgaggttcgattcctcgacgaggaaacc
1–2tRNA–Arg(gcc)[6901,6992]
tcctcgttagtatagcggctagtacgcccgcggccgacccggcatacaga
taccggccgcgggagaccggggttcgagtccccgacgagggt
1–3tRNA–Ala(cgc)[8015,8094]
tcctcgttagtttagaggtagaacgcccatcgtcttcgcgaggagaatgg
gagaccggggttcgagtccccgacggggat
1–4tRNA–Asp(gtc)[8658,8730]
tcctcgttagtatagtggttagtattcccgtctgtcgcacgggagaccgg
ggttcgattccccgacgaggagc
1–5tRNA–Leu(gag)[9452,9525]
gtccccgtttgtacattggtagtactcccgctcgaggtgctgggattgcg
gggttcgagtccccggcggggaca
1–6tRNA–Arg(tcg)[9685,9761]
tcctcgttagtatagtggtagtacaaccggttcgcgcgggcggccgggag
accgaggttcgattcctcggcgaggac
1–7tRNA–SeC(tca)[10069,10145]
tccccgttagtttagtggtataacacccatctcaccttggtagacgggag
accggggttcgattccccgacggggac

1–9tRNA–Tyr(ata)[11448,11527]
tcctcggtagtacaacggttactacgcccgtatatatatagagtaaacgg
gagatcggggttcgactccccgccgaggaa
1–10tRNA–Asp(gtc)[11730,11800]
tcctcgttagtatagtggtagtatgcccgtctgtcgcacgggagaccggg
gttcgattccccgacgaggag
1–11tRNA–Asp(gtc)c[139353,139424]
tcctcgttagtatagtggttaatatccccgtttgtcgcacgggagaccgg
ggttcgaatccccgacgaggag
1–12tRNA–Gly(gcc)c[140378,140453]
ccctcgctagtatagtggttattacgctcggcggtgcctaggccgagaga
ccggggttcgatcccccggcgaggga
1–13tRNA–Ser(gct)c[140864,140946]
atcctcgttagtacagcggttagtacgccaacccaatagctatctgggtt
ggaaaccggggttcaactccccgacgaggatac
1–14tRNA–Ser(gga)c[141574,141647]
tccccgttagtacagtggcagtacgccgtccgtggagcggatggacaacc
ggggttcgattccccgacggggca
1–15tRNA–Thr(ggt)c[142283,142365]
tgcctcgctagtacaggggttagtatgcccgtatggtatatgtgtcacgc
gggagaccggggttcgactccccggcgaggcgt
1–16tRNA–Asn(gtt)c[142502,142584]
gtcctcgttagtacagcggttagtatacgcacttgactgtttgtcaggta
gcgagagcggggttcgactccccggcggggatt
1–17tRNA–Ser(gct)c[142745,142824]
ccctcattagtacagcggttagtacacccggacgctccacggacacacgg
gagagcggggttcgactccccgatgagggc
1–18tRNA–Arg(ccg)c[143382,143473]
tcctcgttagtatagcggctagtacgcccgcggccgacccggcatacaga
taccggccgcgggagaccggggttcgagtccccgacgagggt
1–19tRNA–Arg(cct)c[145788,145866]
tccctcgttagttcagtggtagaatacccgcctcctctccgggcgggaga
ccgaggttcgattcctcgacgaggaaacc
>KT594769.1 Macropodid herpesvirus 1 isolate MaHV1.3076/08, complete genome
1–1tRNA–His(gtg)c[71838,71918]
ccggggggtagacgatggtggtctccgcatctgtgataagtggggggatt
cgaatttcaaggatcttatcctgctctcgaa
>KX589235.1 Columbid alphaherpesvirus 1 strain HLJ, complete genome
1–1tRNA–Arg(cct)[4508,4586]
tccctcgttagttcagtggtagaatacccgcctcctctccgggcgggaga
ccgaggttcgattcctcgacgaggaaacc
1–2tRNA–Arg(ccg)[6901,6992]
tcctcgttagtatagcggctagtacgcccgcggccgacccggcatacaga
taccggccgcgggagaccggggttcgagtccccgacgagggt

1–3tRNA–Ala(cgc)[8016,8095]
tcctcgttagtttagaggtagaacgcccatcgtcttcgcgaggagaatgg
gagaccggggttcgagtccccgacggggat
1–4tRNA–Asp(gtc)[8659,8731]
tcctcgttagtatagtggttagtattcccgtctgtcgcacgggagaccgg
ggttcgattccccgacgaggagc
1–5tRNA–Leu(gag)[9452,9525]
gtccccgtttgtacattggtagtactcccgctcgaggtgctgggattgcg
gggttcgagtccccggcggggaca
1–6tRNA–Arg(tcg)[9687,9763]
tcctcgttagtatagtggtagtacaaccggttcgcgcgggcggccgggag
accgaggttcgattcctcggcgaggac
1–7tRNA–SeC(tca)[10071,10147]
tccccgttagtttagtggtataacacccatctcaccttggtagacgggag
accggggttcgattccccgacggggac
1–9tRNA–Tyr(ata)[11453,11534]
tcctcggtagtacaacggttactacgcccgtatatatatatagagtaaac
gggagatcggggttcgactccccgccgaggaa
1–10tRNA–Asp(gtc)[11736,11806]
tcctcgttagtatagtggtagtatgcccgtctgtcgcacgggagaccggg
gttcgattccccgacgaggag
1–11tRNA–Asp(gtc)c[139527,139598]
tcctcgttagtatagtggttaatatccccgtttgtcgcacgggagaccgg
ggttcgaatccccgacgaggag
1–12tRNA–Gly(gcc)c[140552,140627]
ccctcgctagtatagtggttattacgctcggcggtgcctaggccgagaga
ccggggttcgatcccccggcgaggga
1–13tRNA–Ser(gct)c[141038,141120]
atcctcgttagtacagcggttagtacgccaacccaatagctatctgggtt
ggaaaccggggttcaactccccgacgaggatac
1–14tRNA–Ser(gga)c[141749,141822]
tccccgttagtacagtggcagtacgccgtccgtggagcggatggacaacc
ggggctcgattccccgacggggca
1–15tRNA–Thr(ggt)c[142457,142539]
tgcctcgctagtacaggggttagtatgcccgtatggtatatgtgtcacgc
gggagaccggggttcgactccccggcgaggcgt
1–17tRNA–Ser(gct)c[142918,142997]
ccctcattagtacagcggttagtacacccggacgctccacggacacacgg
gagagcggggttcgactccccgatgagggc
1–18tRNA–Arg(ccg)c[143555,143646]
tcctcgttagtatagcggctagtacgcccgcggccgacccggcatacaga
taccggccgcgggagaccggggttcgagtccccgacgagggt
1–19tRNA–Arg(cct)c[145961,146039]
tccctcgttagttcagtggtagaatacccgcctcctctccgggcgggaga

ccgaggttcgattcctcgacgaggaaacc

*Inoviridae

>JN402401.1 Uncultured phage WW-nAnB, complete genome

1-1tRNA-Ser(act)[1578,1646]

gcggggtggtgcaacgtagcacgccagtctactgaaactggaactgctgg

ttcgaatccagatcccgca

>KJ003982.1 Uncultured phage WW-nAnB strain 3, complete genome

1-1tRNA-Gln(ctg)[1837,1905]

gcggggtggtgcaacggtagcacgtcgcgctctgaacgcgaaactgtagg

ttcgaatcctaaccccgca

>KJ003981.1 Uncultured phage WW-nAnB strain 2, complete genome

1-1tRNA-Gln(ctg)[1837,1910]

ggcggggtagtgcaatggtcgcatgtcactctctgaaggtgaagctgctg

gttcgaatccagaccccgcaacca

*Iridoviridae

>AY380826.1 Lymphocystis disease virus - isolate China, complete genome

1-1tRNA-Leu(taa)c[169350,169425]

ataatttctagttgagacatgaataatttatttttaaaaaataaaacaat

tagaagttcaattttttttaattctc

>AY521625.1 Singapore grouper iridovirus, complete genome

1-1tRNA-Ile(tat)c[115610,115682]

gctccagtagcgcaatcggcagcgcgtggtacttataatgccaaggttgt

gagttcgatcctcacctggagca

>DQ643392.1 Aedes taeniorhynchus iridescent virus, complete genome

1-1tRNA-Tyr(ata)c[3354,3443]

ctctcctataggggaaggagcccttggagcctatatcgccccatagatca

atttcctggaattgtggtccaagaggccatatgagagggg

>KR139659.1 Scale drop disease virus isolate C4575, partial genome

1-1tRNA-Ile(tat)[60628,60703]

tgctctagtggcgcagttaggtagcgcgcagtgcttataacactgaggtc

gtgagttcgatcctcacctagagcat

*Lipothrixviridae

>AJ854042.1 Acidianus filamentous virus 2, partial genome

1-1tRNA-Lys(ctt)c[17151,17237]

gggctcgtagcttagccaggtagagcaacgggctcttaacctagggagat

acccgtaggtcccgggttcgaatcccggcgagcccgc

*Luteoviridae

>KU315178.1 Pepo aphid-borne yellows virus isolate RSA BB Marrow, complete genome

1-1tRNA-Arg(gcg)c[4996,5082]

actctccttgctcgaaccaggatcgagcacttcttgcgaaggagtgttaa

ggtcttgtctttgaccggtttcagccagtgaggagtt

*Marseilleviridae

>AP017398.1 Tokyovirus A1 DNA, nearly complete genome

1-1tRNA-His(gtg)[5265,5337]

gtggatataacttaataggtaaagtgacaatttgtgaagttgtaaatatg
ggttcgattcccattattcaccc
1-2tRNA-Leu(taa)[5341,5424]
gtatcgtttggcggaattggtagacgcgatgggcttaaaacctatttcca
tgaggagtattggttcgattccaataacggttta

*Mimiviridae

>HQ336222.2 Acanthamoeba polyphaga mimivirus, complete genome
1-1tRNA-Leu(taa)c[57234,57316]
gcaaaggtggcggagtggtctaacgcggtagacttaagatctactatctt
ttgatgtcgtgggttcgaatcccacccctttgca
1-2tRNA-His(gtg)[352157,352227]
gatccgttagtttagtggtagaactactgtttgtgggacggtcgacacag
gttcgattcctgtacgggtca
1-3tRNA-Cys(gca)[352275,352346]
ggatcgttagctcaatggtagagcgctcgcctgcagagcgataggttatc
agttcaattctgatacgatcct
1-4tRNA-Trp(cca)[1129299,1129369]
gacctgttagtttaatggtaaaacggtagcctccagagtgattgatactg
gttcgattccggtataggtcc
1-5tRNA-Leu(caa)[1139240,1139322]
gcaaaggtggcggagtggtctaacgcggtagactcaagatctactatctt
ttgatgtcgtgggttcgaatcccacccctttgca
1-6tRNA-Leu(taa)[1167848,1167930]
gcaaaggtggcggagtggtttaacgcgctggacttaagatctagtatcat
ttgatatcgtgggttcgaatcccacccctttgca

>GU244497.1 Cafeteria roenbergensis virus BV-PW1, complete genome
1-1tRNA-Tyr(gta)[509015,509086]
ctcgccttagctcagtggtagagcggcggactgtagttccgtaggtcact
cgttcgaatcgggtaggtgaga
1-2tRNA-Leu(taa)[509181,509265]
gcatatgtgtccgagtggtttaaggagacggttttaagcaccgttggcta
cggcctcgcaggttcgaatcctgtcatatgcacca
1-3tRNA-Leu(taa)[509421,509504]
gtatatgtgtccgagtggtttaaggagacggttttaagcaccgttggcta
cggcctcgcaggttcgaatcctgtcatatacacc
1-4tRNA-Lys(ttt)[509506,509583]
gccttattagctcagttggattagagcagcaggcttttaacctgatggtc
gtgggttcgagtcccacataaggctcca
1-6tRNA-Leu(taa)[509911,509993]
gcatatgtgtccgagtggtttaaggagacggttttaagcaccgttggcta
cggcctcgcaggttcgaatcctgtcatatgcac
1-7tRNA-Tyr(ata)[510066,510135]
gtcactttagcttaagggtaaagcaacaacattatacttgtgaatgtagg
ttcgattcctacaagtgaca

1–8tRNA–Leu(taa)[510177,510261]
gcatatgtgtccgagtggtttaaggagacggttttaagcaccgttggcta
cggcctcgcaggttcgaatcctgtcatatgcacca
1–9tRNA–Leu(taa)[510417,510501]
gtatatgtgtccgagtggtttaaggagacggttttaagcaccgttggcta
cggcctcgcaggttcgaatcctgtcatatacacca
1–10tRNA–Leu(taa)[510656,510739]
gtatatgtgtccgagtggtttaaggagacggttttaagcaccgttggcta
cggcctcgcaggttcgaatcctgtcatatacacc
1–11tRNA–Lys(ttt)[510741,510818]
gccttattagctcagttggattagagcagcaggcttttaacctgatggtc
gtgggttcgaatcccacataaggctcca
1–12tRNA–Leu(taa)[511091,511175]
gcatatgtgtccgagtggtttaaggagacggttttaagcaccgttggcta
cggcctcgcaggttcgaatcctgtcatatgcacca
1–13tRNA–Leu(taa)[511330,511414]
gtatatgtgtccgagtggtttaaggagacggttttaagcaccgttggcta
cggcctcgcaggttcgaatcctgtcatatacacca
1–14tRNA–Leu(taa)[511570,511653]
gtatatgtgtccgagtggtttaaggagacggttttaagcaccgttggcta
cggcctcgcaggttcgaatcctgtcatatacacc
1–15tRNA–Lys(ttt)[511655,511732]
gccttattagctcagttggattagagcagcaggcttttaacctgatggtc
gtgggttcgaatcccacataaggctcca
1–16tRNA–Asn(gtt)[511736,511809]
ggtaccgtagaacaattggttagttcaacagactgttaatctgaaggttg
ttggttcgagtccagccggtaccg
>JN258408.1 Megavirus chiliensis, complete genome
1–1tRNA–Leu(taa)c[15827,15909]
gctaaggtggcggagtggtctaacgcgatagacttaagatctattatcat
ttgatgtcgtgggttcaaatcccacccttagca
1–2tRNA–Leu(caa)c[16963,17045]
gctaaggtggcggagtggtctaacgcggtagactcaagatctactatctt
atgatgtcgtgagttcgaatctcacccttagca
1–3tRNA–Trp(cca)c[62763,62833]
gacctgttagtttaatggtaaaatggtcgcctccagagtgattgatatcg
gttcgattccggtacaggtcc
>JX962719.1 Acanthamoeba polyphaga moumouvirus, complete genome
1–1tRNA–His(gtg)[96801,96872]
gatctgttagtttagttggtagaatacctgtctgtggaacaggagacatc
ggttcgaatccgatacaggtca
1–2tRNA–Cys(gca)[96877,96951]
aggaccattagctcaattggtagagcaatcgcctgcagagcgagaggtta
ctggttcaagtccagtatggtccta

>KF527228.1 Mimivirus terra2 genome

1-1tRNA-His(gtg)[116247,116317]
gatccgttagtttagtggtagaactactgtttgtgggacggtcgacacag
gttcgattcctgtacgggtca

1-2tRNA-Cys(gca)[116365,116436]
ggatcgttagctcaatggtagagcgctcgcctgcagagcgataggttatc
agttcaattctgatacgatcct

1-3tRNA-Leu(taa)c[768570,768652]
gcaaaggtggcggagtggtctaacgcggtagacttaagatctactatctt
ttgatgtcgtgggttcgaatcccacccctttgca

1-4tRNA-Leu(taa)c[960128,960210]
gcaaaggtggcggagtggtttaacgcgctggacttaagatctagtatcat
ttgatatcgtgggttcgaatcccacccctttgca

1-5tRNA-Leu(caa)c[993610,993692]
gcaaaggtggcggagtggtctaacgcggtagactcaagatctactatctt
ttgatgtcgtgggttcgaatcccacccctttgca

1-6tRNA-Trp(cca)c[1003497,1003567]
gacctgttagtttaatggtaaaacggtagcctccagagtgattgatactg
gttcgattccggtataggtcc

*Myoviridae

>AF158101.6 Enterobacteria phage T4, complete genome

1-1tRNA-Arg(tct)c[71173,71247]
gtcccgctggtgtaatggatagcatacgatccttctaagtttgcggtcct
ggttcgatcccagggcgggatacca

1-2tRNA-Met(cat)c[71960,72033]
ggccctgtagctcaatggttagcagcagtcccctcataagggaaaggtta
ccagttcaaatctggtctgggtca

1-3tRNA-Thr(tgt)c[72035,72110]
gctgatttagctcagtaggtagagcacctcacttgtaatgaggatgtcgg
cggttcgattccgtcaatcagcacca

1-4tRNA-Ser(tga)c[72117,72205]
tggaggcgtggcagagtggtttaatgcaccggtcttgaaaaccggcagtc
gctccggcgactcataggttcaaatcctatcgcctccgt

1-5tRNA-Pro(tgg)c[72206,72280]
ctccgtgtagctcagtttggtagagcgcctgatttgggatcaggaggtcc
aaggttcaaatccttgtatggagac

1-6tRNA-Gly(tcc)c[72291,72364]
gcggatatcgtataatggtattacctcagacttccaatctgatgatgtga
gttcgattctcattatccgctcca

1-7tRNA-Leu(taa)c[72370,72456]
gcgagaatggtcaaattggtaaaggcacagcacttaaaatgctgcggaat
gatttccttgtgggttcgagtcccacttctcgcacca

1-8tRNA-Gln(ttg)c[72457,72530]
tgggaattagccaagttggtaaggcatagcactttgactgctagatgcaa

aggttcgagtcctttattcccagc

>U24159.1 Bacteriophage HP1 strain HP1c1, complete genome

1-1tRNA-Lys(ttt)c[356,431]

gggtcgttagctcagtcggtagagcagcggacttttaatccgttggtcga

aggttcgaatccttcacgacccacca

>AF320576.1 Bacteriophage Felix 01, complete genome

1-1tRNA-Pro(tgg)[23699,23775]

ctctgtttagctcagcttggtagagcgttccgtttggggcggtaaggccg

gaggttcaagtcctccaacagagacca

1-2tRNA-Glu(ttc)[23783,23860]

gttccagtagacaaaatggtaaagtcaccactctttcaaagtggatatct

gagggttcaaatcccttctggaacgcca

1-3tRNA-Met(cat)[23952,24028]

tgcgggtatagagaaagggcgtctcacatgtctcattagcatggtatcgg

caggttcgactcctgcacccgcctcca

1-4tRNA-Asn(gtt)[24112,24188]

ggttaggaagcacataaggtatgtgcggtcgcctgttaagcgaatggcac

agggttcgaatccctgactaaccgcca

1-5tRNA-Tyr(gta)[24258,24345]

gtgtcgttatcccgtagatggtagcggtggggactgtaaatcccttgtca

ttgagactcggtaggttcgactcctacacggcacacca

1-6tRNA-Asp(gtc)[24351,24427]

ggctatgtagtttaactggagaaaatactcccctgtcacgggagatgatg

tgagttcaagtctcatcgtagccgcca

1-7tRNA-Lys(ttt)[24856,24931]

ggaagtgtagcagaatggtgatgcggcagacttttaatctgacaggcgat

gggttcgaatccctccacttctacca

1-8tRNA-Met(cat)[24936,25012]

ggttcagtcgcagataaggtaatgcaagggtctcataagccctatgaatg

tgggttcgattcccatctgaacctcca

1-9tRNA-Ile(gat)[25509,25584]

gctgtgaaagcacatatggatgtgcattcggctgataaccgaaaggcaga

aggttcgaatccttctcacagtacca

1-10tRNA-Ser(tga)[26629,26718]

ggtaggtagcggctaatggtagccaaactgtcttgaaaacagttgccact

gtagagatacggtgagggttcgactcctttacttaccgcc

1-11tRNA-Leu(tag)[26975,27052]

gggagattgatggtaattggtaaacctatctcgcttagaacgagatgttt

gagggttcgaatcccttgtctcctacca

1-12tRNA-Lys(ctt)[27060,27135]

gcaggtgtagcaaaatggttatgcggctgactcttaatcagtaagacgat

gggttcaattccctccacctgtacca

1-13tRNA-Ala(tgc)[27142,27217]

tgggtcatagtttatatggttaaaattcgagttttgcaaacttgggaact

gagttcaattctcagtgactccacca
1–14tRNA–Gly(tcc)[27224,27298]
gcatccatagtttaaacgggaaaattacagtcttccaaactgaggttgag
ggttcgattccctctggatgctcca
1–15tRNA–Thr(tgt)[27728,27804]
gctgctttcgtataattggctattacacatcccttgtaaggatggaaatg
caggttcaagtcctgtgagcagcacca
1–16tRNA–Val(tac)[27900,27974]
actcgcttagtttatatggtaaaacatcacccttacaagatgaagaaaaa
ggttcaagtcctttagtgagtacca
1–17tRNA–Leu(caa)[27976,28053]
gttccagtatcccaattggcagaggatgcaagctcaaaccttgtattagt
gacggttcgaatccgtcttggaacacca
1–18tRNA–Arg(acg)[28168,28243]
gcaggattagttcaaatggatagagcaacagtctacgaagctgttaatag
gggttcgaatcccttatcctgcgcca
1–19tRNA–Gln(ttg)[28828,28903]
aggggattagtttacaaggttaaaacctcggtctttgaaatcgaagaagt
tggttcaattccaacatcccccgcca
1–20tRNA–Leu(taa)[28906,28984]
gctccattactccaattggcagagaggccagacttaaaatctgtgttatg
tatcggttcgaatccgatatggagtacca
1–21tRNA–Gln(ctg)[28990,29065]
agcggtatagcataactggcaatgcagcagtctctgaagctgtcctatta
aggttcaaatccttatgccgctgcca
1–22tRNA–His(gtg)[29097,29172]
gtggccttatcataaatggtaatgacccatgctgtgaacatggtctatac
gggttcaaatcccgtaggtcacccca
1–23tRNA–Phe(gaa)[29179,29254]
agtccaagtagcttatatggttaaagcgcgtgtctgaaaaacatgagaag
agggttcaaatcccactggactacca
1–24tRNA–Ser(gct)[30008,30100]
ggaagattaaccctaaaaggtaagggagcagtttgctaaactgccagtag
ctgagaaatcggtgtaccagttcaagtctggtatcttcctcca
1–25tRNA–Cys(gca)[30105,30180]
gaatccgtgacagaaatggatatgtgcctgtctgcaaaacaggtttataa
gggttcaagtcccttcggattctcca
>AY129337.1 Mycobacteriophage Bxz1, complete genome
1–1tRNA–Ser(gct)[32863,32946]
ggagggtgagcatctggtgatgcaggggtcctgctaaggccctacggatt
cacacccgtgagtttcgattactcctccctccgc
1–2tRNA–Leu(cag)[33043,33119]
gccctgctgagcaaactggcaaagctgccgcattcagagtgcgggtcatt
tccgggttcgactcccgggcagggtac

1–3tRNA–Leu(gag)[33239,33313]
gtctctgtaggcaaatcggaaaagccgccatcttgagggggtggtgcgtg
cgggttcgactcccgccagagacac
1–4tRNA–Leu(caa)[33314,33387]
gccgtggtaggccatctggcgagccgccgagttcaagtttcggtgtttgc
gggttcgaatcccgcccacggtac
1–5tRNA–SeC(tca)[69709,69805]
attctggcactggtgggcgagcccaccggcggcagcttcaagctgtcgct
ggccggagaaccgaccggaaacatcccgttcaacgcgacccagggcc
1–6tRNA–Pro(tgg)[94073,94147]
cggggtgtagttcagtttggaagagcgcttggtttgggaccaagatgtcg
caggttcgaatcctgtcaccccgac
1–7tRNA–Trp(cca)[94164,94239]
tggggtgaagccgatctggaaggcagcggtctccaaagccgtctcatagc
gggttcgaatcccgtcacccctgcca
1–8tRNA–Tyr(gta)[94241,94327]
gccgcacatgcccaactggtgtttgggagcaggctgtaaccctgtggcct
tcgggacggtgaggttcgattcctcagtgcggtacca
1–9tRNA–Pyl(cta)[95436,95508]
gcaccatttgctcaatggcagagcggcggcttctaaccccgtgagtgccg
gttcgattccggcatggtgcacc
1–10tRNA–Met(cat)[95658,95732]
agcggtgtagagcagctaggtagctcgccgggctcataacccggaggacg
cgtgttcgaatcacgccaccgccac
1–11tRNA–Cys(gca)[95859,95930]
gcgcctttggcggaatggctacgtgctcggctgcaacccgagttatcccg
gttcgactccgggagggcgctc
1–12tRNA–Glu(ctc)[95998,96069]
gctcccatggggtagtggtaaccctcctggttctcagccaggcgtcccga
gttcgatcctcggtgggagtgc
1–13tRNA–His(gtg)[96071,96144]
gtggccgtagttcagccggtagaacgctgggttgtgatcccagtcgtcga
gggttcgagtccctccggtcaccc
1–14tRNA–Ala(tgc)[96306,96380]
gggcctgtagctccaattggtagagcagcatccttgcaagatgacggctg
tcggttcgaatccgacctggtccac
1–15tRNA–Phe(gaa)[96570,96642]
gccgtcatagctcagttggtagagcactggcctgaaaacccagtggccga
ggttcgagtcctcgtgtcggcac
1–16tRNA–Val(cac)[96648,96721]
gtccgtttagctcagctggaagagcgctcggtccacacccgagaggccgc
aggttcgatccctgcaatggacac
1–17tRNA–Lys(ctt)[96840,96912]
gccttcgtagctcagtggtagagctgtcgcctcttaagcgataggtcgtt

ggttcgaatccagccgggggcac
1-18tRNA-Glu(ttc)[96917,96993]
ggtcgggtcggtctgctggtatggccagtcggattttcactccggacatt
cgcgggttcaattcccgtcccgatcgc
1-19tRNA-Gly(tcc)[97073,97145]
gcgggtgtggccgaatggctcaggcaccagatttccactctggctacgca
ggttcgattcctgtcatccgctc
1-20tRNA-Thr(cgt)[97205,97279]
gctgctgtagctcacctggcagagcgtcggcgtcgtatcccgaaggcatc
cggttcgagtccggacagcagcccc
1-21tRNA-Thr(tgt)[97280,97352]
gcctctgtggtccagcggcacggacatccgccttgtaagcggaggacccc
cgttcgatccggggtagaggctc
1-22tRNA-Thr(ggt)[97760,97832]
gctgggttagctcagtggtagagcgttcctctggtatgggaaaggcccgg
ggttcaatcccccgattcagctc
1-23tRNA-Gly(gcc)[99155,99228]
gcgaaggtagctcagctggcagagcgccaccttgccaaggtggaggtcgc
gggatcgtaacccgttcttcgctc
1-24tRNA-Asp(gtc)[99232,99304]
ggccctgtagctcagaggaagagcgccggtctgtcgaatcggaggtcgcg
gtatcgtaatccgtcagggtcgc
1-25tRNA-Met(cat)[99364,99436]
gcctcactagctcattggtagagccgctcgctcataacgtgcaggtacct
ggttcgattccagggtgaggtac
1-26tRNA-Ile(gat)[99442,99516]
gcctgttagcggactggtcgtccgatccaagctgataacttggcgtaagc
ggtgttcgattcaccgagcaggtac
1-27tRNA-Arg(acg)[99610,99682]
gcctctatggtccaacggatatgacgccggtctacggaaccggagatgcg
tgttcgattcgcgctaggggcac
1-28tRNA-Val(gac)[99725,99797]
gtccgtgtagctcaggggtagagcgcctgctcgacacgcaggaggaccga
ggttcgaaacctcgcatggacac
1-29tRNA-Arg(cct)[99972,100045]
gcctctgtagctcaacggacagagcaacgcggtcctaacgcggtggctgg
aggttcgaatcctctcggaggcac
1-31tRNA-Gln(ttg)[100586,100661]
tggggtatggtggcaatctggcagtcccgccggactttgactccggaggt
gcaggttcgagtcctgctaccccatc
1-32tRNA-Arg(tct)[100665,100740]
gcccttgtagctcagtggacagagcggcgagcttctacctcgcgggccgg
gagttcgaatctctccaggggcacca
1-33tRNA-Gln(ctg)[127267,127341]

tgctcgttggtgtaactggcaacactacggactctgactccgtcatttta
ggttcgaatcctaagcgagcagcca
1-34tRNA-Asn(gtt)[127348,127423]
tggggtgtccgttaatcaggcaaacgagcggactgttaatccgcccctgc
aggttcgaatcctgccaccccagcca
>AY375531.1 Bacteriophage 44RR2.8t, complete genome
1-1tRNA-Arg(tct)c[65379,65455]
ggtctcttagctcagctggacagagcaacgtcgttctaagtcgttggtca
ttggttcgaatccaatagggaccacca
1-2tRNA-Met(cat)c[66424,66498]
ggccctgtagctagacggttcaagcaggcggctcataaccgctcgtagca
ggttcgattcctgccagggccacca
1-3tRNA-Met(cat)c[66706,66782]
tgcgacgtagaggagaggtcgtcctcgtcgggctcatatcccggaaatcg
gtggttcgaatccatccgtcgcatcca
1-4tRNA-Asp(gtc)c[66804,66877]
ggagccatagtttatacggttaaaataatcccctgtcacgggatagcacc
gagttcgatcctcggtggctccgc
1-5tRNA-Ser(gct)c[66951,67038]
ggagaagtggatgagcggtttaaatcgcttccctgctaaggaagtaaacc
gaaaggtttcgagagttcgaatctctccttctccgcca
1-6tRNA-Ile(gat)c[67046,67121]
agtggattagctcagtaggtagagcactcgaccgataatcgagagcgcac
tggttcgaccccagtatccactacca
1-7tRNA-Trp(cca)c[67124,67199]
atgacattggtgttagcggtagcatgccggtctccaaaaccgtgcggcca
gggttcgaatccttgatgtcatgcca
1-8tRNA-Thr(tgt)c[67545,67620]
gccgatttagctcatccggtagagcagttgttttgtaagcatcaggtggt
ccgttcgagtcggacaatcggcacca
1-9tRNA-Pro(tgg)c[68260,68336]
ccgcgtgtaggctagtcaggtaggtcgtctggtttgggaccagaaagtcg
gaggttcgaatccttccacgcggacca
1-10tRNA-Gly(tcc)c[68842,68917]
gcgggtaaggtgtttacggatacatgctagccttccaagcttgagtagac
cggttcgataccggctacccgctcca
1-11tRNA-His(gtg)c[68930,69004]
gtgacggtagctcagctggtagagccccaggttgtgatcctggtcgtcgc
gagttcaatcctcgtccgtcacccc
1-12tRNA-Lys(ttt)c[69125,69200]
gcatcgttaactcaattggcagagtagcggacctttaatccgttggttct
gagttcgaatctcagacggtgtacca
1-13tRNA-Tyr(gta)c[69804,69886]
ggaacgttcgggtaatggtatcccaagcggctgtaacccgctcgcctctg

gcattcttggttcgagtccaaggcgttccacca
1–14tRNA–Asn(gtt)c[69900,69985]
ggtgatgtggctcagaggttgagcggcggactgttaatccgcgaggaatt
tcctctatactggttcgagtccagtcatcaccgcca
1–15tRNA–Phe(gaa)c[70067,70151]
gttgctgtgagccgagtggctgaaggcggtccctggaaacgggacgggtt
gaaagacctcgaaggttcgaatccttccagcaacc
1–16tRNA–Ser(tga)c[70222,70315]
ggaagcttggcagagtctggcttattgcactagtcttgaaaactagcgat
cgtaggaatacggtccatccgttcaaatcggatagcttccgcca
1–17tRNA–Leu(taa)c[70506,70589]
gggagtgtggtggaacggcatacacaggaggcttaaaacctcccgccttc
gggattgagggttcgagtccctcctctcctacca
>AF399011.1 Pseudomonas phage phiKZ, complete genome
1–1tRNA–Thr(tgt)c[266721,266797]
gccgctatagctcagctaggtagagcaacgcacttgtaatgcgtaggtcc
tccgttcgattcggagtggcggcacca
1–2tRNA–Asn(gtt)c[269558,269644]
tggtaagtacccgagtggcagcagggagcggactgttaatccgttggcga
aagcccaccgtaggttcgaatcctaccttaccagcca
1–3tRNA–Asn(gtt)c[271741,271816]
tccgtgatagctcagtcggtagagcaagtgactgttaatcactgggtccc
tggttcgagtccaggtcacggagcca
1–4tRNA–Asp(gtc)c[271823,271899]
gcgctcatagttcagttggttagaatacccgcctgtcacgcgggtggtca
ggggttcgagtccccttgggcgcgcca
1–5tRNA–Met(cat)c[272872,272948]
gggcctatagctcagttggttagagcaggcgactcataatcgcttggtcg
caggttcaagtcctgctgggcccacca
1–6tRNA–Pro(tgg)c[273271,273346]
cggagtgtagcgcagttggtagcgcgcctgctttgggagcaggatgtcgg
gagttcgagtctccccactccgacca
1–7tRNA–Leu(taa)c[273356,273440]
gcccgaatggtgaaattggtaaacacagaagacttaaaatcttccggcta
cggtcttgtcggttcgaatccgacttcgggcacca
>AY266303.2 Bacteriophage Aeh1, complete genome
1–1tRNA–Met(cat)c[105608,105682]
ggccccgtagctggagggttttagcgtgcggctcataaccgcttgatgtg
ggttcgattcccaccggggccacca
1–2tRNA–Leu(tag)c[106157,106243]
gcggatgtggtgaaattggtagacacacgagttttaggtgctcgcgctag
tgatagcgtgtcggttcgagtccgaccatccgtacca
1–3tRNA–Ser(gct)c[106250,106337]
ggagaaatgacagagaggccgaacgtagcggattgctaattcgtaggccc

gcaagggtccgtgggttcgaatcccactttctcctcca
1−4tRNA−Ser(tga)c[106694,106788]
ggaagcgtggcagagtccggcttattgcacaagtcttgaaaacttgcggc
gaggtatcccctcgtccatccgttcgaatcggatcgcttccgcca
1−5tRNA−Gln(ttg)c[107234,107310]
aggggattcgcctagctggcctaaggcatcggactttgactccgatatcg
ttgattcgaattcaacatcccccgcca
1−6tRNA−Pro(tgg)c[107564,107638]
ctcgggttaggcaagtggtatgtcgcctggtttgggaccaggtcgtcgag
tgttcgattcactcacccgagacca
1−7tRNA−Phe(gaa)c[107775,107850]
gggtccttagctcagtaggtagagcatatgattgaagatcatagtgtccc
cagttcaattctgggagggcccacca
1−8tRNA−His(gtg)c[107856,107931]
gtgacggtagttcagttggtagagctccaggctgtgatcttggtagtcac
gggttcgatccccgtccgtcacccca
1−9tRNA−Thr(tgt)c[108011,108085]
gcctctatagctcagtggtagagcaactgccttgtaagcagtaggtcctc
ggttcaatcccgagtgggggctcca
1−10tRNA−Tyr(gta)c[108092,108183]
ggatgtgctgcaatgacggtggtattgcaccagactgtaaatctggcccc
acagggtaaacattgtaggttcgactcctaccgcatccacca
1−11tRNA−Met(cat)c[108208,108282]
tgcgagttggagaagtccggtatctcgttagcctcataagctaaaggtcg
gtggttcaaatccaccactcgcatc
1−12tRNA−Glu(ttc)c[108303,108378]
tctccgttcgtctagtggactaggacactgccctttcacggcggtaacat
ggattcgaattccatacggagaacca
1−13tRNA−Met(cat)c[108870,108944]
ggtgcattagctcagtggtagagctgcggtttcatacgccgtcggtcggt
agttcgaatctaccatgcaccacca
1−14tRNA−Lys(ttt)c[109164,109240]
gcgctgttagctcaatggtttagagcaatcggcttttaaccgataggttc
cgggttcgagtcccgggcggcgtacca
1−15tRNA−Trp(cca)c[109247,109320]
aggggattagcaaaatggttatgcggcggattccaaatccgttgacgtga
gttcgattcttacatcccctgcca
1−16tRNA−Cys(gca)c[110265,110339]
gtgccggtagccaagcggtaaggcaagtgattgcaaatcacttttacgtc
tgttcaaatcagatccggtactcca
1−17tRNA−Gly(tcc)c[110347,110420]
gcctcattggtgtagtggtcacataccgtccttccaagtcgtagtgtcgg
gttcgattcccgaatgaggctcca
1−18tRNA−Ala(tgc)c[110494,110569]

ggggaaatagctcagatgggagagcactagccttgcaagctgggggtcgt
gggttcgagtcccattttctccacca
1-19tRNA-Asp(gtc)c[110939,111013]
ggtgcattagtataatggctattattcgtgactgtctatcacaagatacg
ggttcgattcccgtatgcaccgcca
1-20tRNA-Ile(gat)c[111020,111094]
agtcccgtagctcagaggtagagcgccgtgctgataacgcggatgtggat
ggttcgataccattcgggactacca
1-21tRNA-Asn(gtt)c[111229,111311]
ggaggtgtggctcaaaggttgagcggcagactgttaatctgcgggtaata
accctatgttggttcgagtccaacctcctccgc
1-22tRNA-Leu(taa)c[114632,114720]
gcgagtgtagccgaatcggtataggcagcagacttaaaatttgccggtgc
taacgcgccatgtgagttcgatcctcaccactcgcacca
1-23tRNA-Leu(caa)c[114726,114810]
gggagtatgctggaactggcatacagggcaagctcaaaccttgttgacaa
tagtcgtgtgggttcgaatcccactactcccacca
>AY283928.2 Bacteriophage KVP40, complete genome
1-1tRNA-Ser(gct)c[173137,173225]
ggagatatggtctaaaggtatgacagcaccctgctaaggtgtcggacgtt
aatagcgttctctgggttcgattcccagtatctccgcca
1-2tRNA-Leu(caa)c[173666,173742]
gcccaactagtccaattggcagaggcgctagtttcaaacactagatgatc
cgagttcgaatctcgggttgggcacca
1-3tRNA-Leu(taa)c[173859,173935]
gcgcacgtggtccaattggcagaggcatgaggcttaaaatctcagggatg
gcggttcgaatccgcccgtgcgtacca
1-4tRNA-Cys(gca)c[173938,174011]
gcccgaatcgtataatggaagtatgagggattgcaaatcccgcggtcaga
gttcgattctctgttcgggctcca
1-5tRNA-His(gtg)c[174115,174191]
gtggcagtgggtgaagtggaaataccccccgggttgtgattccggaagatg
cgggttcgatccccgtctgtcacccca
1-6tRNA-Val(tac)c[174509,174582]
ggtcccttagtataatggcagtacgtctcttttacacagagaaagaagtg
gctcgattccactagggactacca
1-7tRNA-Gly(tcc)c[174588,174661]
gcgggtatgatgtaatggtagcatgacgtccttccaagtcgttcgtctcg
gttcgagtccgtgtacccgctcca
1-8tRNA-Met(cat)c[174849,174924]
ggagatgtagctcaagtggtagagcaatggtctcataagccattagatcc
gatttcgagtatcggtgtctccacca
1-9tRNA-Arg(tct)c[175429,175504]
gtcctcatagctcaattggaagagcaacgaccttctaagtcgtgggttgt

gggttcgaatccctctggggatgcca
1-10tRNA-Thr(tgt)c[175901,175976]
gccctgatagcacaattggcagtgcagctcacttgtaatgagcaggttcg
cggttcaaatccgtgtcagggcacca
1-11tRNA-Gln(ttg)c[176105,176178]
aggggattgatgtaaaggcagcatacgtgactttgactcacgcagtaccg
gttcgaatccgttatcccctgcca
1-12tRNA-Asp(gtc)c[176198,176273]
ggagccgaggtgtaagtggttgcatgtctccctgtcacggagaaggtagc
gggttcgaaacccgtcggttccgcca
1-13tRNA-Asn(gtt)c[176496,176571]
gccttattaactcagtcggtagagtgtctggctgttaaccagagagtcgt
tggttcgagtccaacataaggcgcca
1-14tRNA-Asn(gtt)c[176578,176652]
gggtgtgtagctcagaggcagagcagacggctgttaaccgtcaggtcgag
atttcgaaattctccacgcccgcca
1-15tRNA-Trp(cca)c[176936,177009]
agggacatgatgtaacggcagcatgacggattccaaacccgttcgttaga
gttcgaatctctatgtccctgcca
1-16tRNA-Ile(gat)c[177016,177089]
agctccatagtttaacggtaaaacacgcgaccgataatcgcacgttgaca
gttcgagtctgtctggggctacca
1-17tRNA-Ser(tga)c[177154,177239]
gaaagattctgggtagcggcaacggtcttgaaaaccgtcggtcaccggga
ggtgatgttagggttcgaatccctagtcttccgcca
1-18tRNA-Met(cat)c[177260,177335]
tgcgagttagagttctggtgaactcactagtctcataagctagtttaggt
cagttcgattctggcactcgctacca
1-19tRNA-Tyr(gta)c[177438,177523]
ggagcgtaagccgcaaggtgcggcagcggactgtaaatccgtgtcccgta
atgggaagagaggttcgattcctctacgctccacca
1-20tRNA-Glu(ttc)c[177530,177603]
gctcgattcgactatcggtaggtcacttccctttcaaggaagtaggacgg
gttcgactcccgtatcgagtacca
1-21tRNA-Lys(ttt)c[177667,177743]
gcgtcggtagctcatcatggaagagcagggagcttttaactctcaggtgt
ctggttcgagtccagggcggcgtacca
1-22tRNA-Lys(ttt)c[177755,177829]
gggtcgttcgtataacggtagtacatctggcttttaaccagaacggtgag
agttcgaatctcttgcgacccacca
1-23tRNA-Phe(gaa)c[177836,177910]
gcacccttagcttatcaggaaaagcggcggattgaagtcccgcagtgctc
ggttcgattccgagggggtgcacca
1-24tRNA-Leu(tag)c[177912,177989]

gcgcaagtagcccaatctggcagaggcactggtcttagaaaccagaagtt
aagagttcgaatctcttcttgcgtacca
1−25tRNA−Arg(acg)c[178432,178507]
gccctactagctcaattggaagagcagcgtcctacgaaggcgcaggttag
aagttcgaatcttctgtggggtgcca
1−26tRNA−Pro(tgg)c[178515,178591]
ccgtgactagctcaatctggtagagtactccgtttggggcggagaagtta
agcgttcgaatcgcttgtcacggacca
1−27tRNA−Pro(tgg)c[178601,178674]
cgggacgtggcgtaaaggtagcgttcatgctttgggagcatgtggtaaga
gttcgagtctcttcgtcccgacca
1−28tRNA−Met(cat)c[180509,180585]
agccccttagctgtaacggtagtagcagacgactcataatcgtcaggttc
tcggttcgaatccgagaggggctacca
>AX059140.1 Sequence 1 from Patent WO0075335
1−1tRNA−Arg(tct)[37706,37780]
gcgcctgtagctcaaccggaaagagcaccagccttctaagctggtggttg
tgggttcgagtcccaccgggcgctc
>AF234172.1 Enterobacteria phage P1 mod749::IS5 c1.100 mutant, complete genome
1−1tRNA−Asn(gtt)[69348,69423]
gatggtgtagctcagcggttagagcggttgactgttaatcaacgggtcga
tggttcaaatccatccaccatcgcca
1−2tRNA−Thr(tgt)[69426,69501]
gccggtttagctcagttggtagagcgcctgccttgtaagcaggatgtcag
cggttcgagtccgttaatcggcacca
1−3tRNA−Met(cat)[70933,71008]
ggccctttagctcagtggttagagctggcgactcataatcgcacggtcac
cggttcaagtccggtaggggccacca
>AY368235.2 Burkholderia cepacia phage Bcep43, complete genome
1−1tRNA−Leu(taa)[17806,17892]
gccaaggtggtggaacggtagacacagcggacttaaaatccgtcgcgcca
tcggcgcgtgccagttcgaatctggcccttggcacca
>AF543311.2 Burkholderia cepacia phage Bcep781, complete genome
1−1tRNA−Ser(gct)[17815,17906]
ggagacgtggctgagtggtcgaaagcgctcccctgctaagggaacgcctg
gagtaatctgggtcgagggttcgaatccctccgtctccgcca
>AY682195.1 Lactobacillus plantarum bacteriophage LP65, complete genome
1−1tRNA−Gly(tcc)c[49688,49760]
gctcccctagtgtaattggctaacacgctagatttccaatctagtaatgc
cggttcaaatccggctgggagct
1−2tRNA−Thr(tgt)c[49777,49847]
gccttgttagttcaacggtagaactcctgtcttgtaaacaggtgatttca
gtccaactctggaacgaggca
1−3tRNA−Trp(cca)c[49854,49928]

tatcagagtaagcttaactggcaaactgctggactccaaatccggacttc
taggttcgaatcctagctttgatgt
1-4tRNA-Pro(tgg)c[50751,50824]
caggatatagctcaatttggtagagcgcctagtttggggctaggatatta
taggttcaaatcctattatcctga
1-5tRNA-Phe(gaa)c[51456,51529]
gtgtctgtagctcagctaggtagagcatcgtgttgaatccacgagggtcg
taggttcaaaccctactgggcaaa
1-6tRNA-Leu(caa)c[51683,51757]
ggcctagtattccaattggcagagaagttagactcaaaatctatacagtg
taggttcgattcctacctaggccac
1-7tRNA-Leu(tag)c[51767,51841]
tgctctcgtaatccaacggcagagatagtggtcttagaagccatccagtg
tgagttcgaatctcaccgagagcat
1-8tRNA-Leu(taa)c[51952,52026]
tgctctcttaatccaacggcagagataatagacttaaaatctataaagtg
taggttcgaatcctacagagagcat
1-9tRNA-Pro(tgg)c[52591,52668]
aatatagtatagctcaggagacagagcgcctagtatgggactaggtacta
tcgctggttaaagcccagcactataatt
1-10tRNA-Asn(gtt)c[52802,52875]
ttgctcagtagttcagcggtagaataggacactgttaatgtcaaggtcgc
tggttcgatcccagcctgagcagt
1-11tRNA-Ser(gct)c[52976,53065]
tggaaagttgacagagtggttgattgtggctccctgctaaggagttaatc
gtgataagcgattcaggggttcgaatcccttactttccat
1-12tRNA-Ile(tat)c[53204,53277]
tactcgtatagctcagaggcagagcattcaacttataattgaggggtcgg
tggttcgaacccacctacgagtat
1-13tRNA-Arg(cct)c[53541,53613]
gtgctattagtctagttggataaaacatcattttcctaaattgaagtccc
cagttcgaatctgggatagcaca
1-14tRNA-Arg(tct)c[53992,54067]
gcgccccttagctcaactggatagagcaactgacttctaatcagtaggtt
gtccggatcgtacccgacaggacgca
>KF766114.1 Staphylococcus phage K, complete genome
1-1tRNA-Met(cat)c[14229,14300]
ggactcttagcttaaaggtaaagccaaccgctcataacggtttgactgta
ggttcgaatcctgcagagtcca
1-2tRNA-Trp(cca)c[37448,37519]
acacccttagtataattagtagtacaagggtctccaaaacccttagtctt
tgtgcaaatcaaagagggtgtg
1-3tRNA-Phe(gaa)c[37526,37598]
ggtttcttagctcagatggtagagcactagattgaagctctaggtgtcat

tggttcaaatccaatagaaacca
1–4tRNA–Asp(gtc)c[37604,37679]
tggctcattggtgtaactggttaacacactgccctgtcacggcagagagt
acgagttcgagtctcgtatgggtcgt
>AJ298298.1 Bacteriophage P27 complete genome
1–1tRNA–Met(cat)[17158,17233]
ggcccctttagctaagtggtgagagcgagcgactcataatcgccaggtcgc
tggttcaaatccagcaagggccacca
1–2tRNA–Arg(tct)[17335,17411]
gcgttgttagctcagtcggacagagcaattgccttctaagcaatcggtca
ctggttcgaatccagtacaacgcgcca
>AJ630128.1 Bacteriophage S–PM2 complete genome
1–1tRNA–Ser(tga)[102852,102939]
ggaggggcaatccgattggtgacggaacctgtcttgaaaacagttgaggt
gttaaagcccttgggagttcgactctccctccctccgc
1–2tRNA–Arg(tct)[118243,118318]
tgcctcagtagctcagtggaatagagcaaccgccttctaagcggtcggtc
gttggttcgagtccaacctgaggcgt
1–3tRNA–Lys(ttt)[118652,118726]
tgggtctgtaactcagttggtagagtagcgggcttttaacctgtaagtcg
tcggttcgaacccgaccagacccat
1–4tRNA–Tyr(gta)[118765,118848]
gggaggatttccgagtggttaaaggaatctgactgtaaatcagacggctc
tgccttcgcaggttcgaatcctgctcctcccacc
1–5tRNA–Asp(gtc)[118850,118925]
tggcccattagtgtagaggcttatcacgccaccctgtcacggtggagatc
acgggttcgaatcccgtatgggtcgt
1–6tRNA–Glu(ttc)[118982,119056]
gttcctatcgactagcggttaggtcaccaccctttcaaggtggtagcacg
ggttcgaatcccgttaggaatacca
1–7tRNA–Ile(gat)[119058,119131]
ggaaacatagcttagttggtaaagcattcgactgataatcgaaagaccac
tggttcgagtccagttgtttccac
1–8tRNA–Ser(gct)[119134,119224]
ggaagtgtggcagagaggtctaatgcagtggattgctaatccgccgatgt
ctttataggcatccgttggttcgaatccaaccacttccgcc
1–9tRNA–Ser(gga)[119226,119312]
tggagaattgtccgagaggcttatggtgcaaacttggaaagtttgtgtgg
gtaaaaccaccagaggttcgaatcctctattctccgt
1–10tRNA–Met(cat)[119315,119386]
ggcagtgtagttcagtggtagaacaagagattcataccctctatgtcggt
agttcaattctacccactgcct
1–11tRNA–His(gtg)[119388,119461]
gtgtcgttagcctaatggtaaggcaggagattgtggttctccctatgagg

gttcgattccctcacggcacccca

1–12tRNA–Gln(ttg)[119463,119537]

tctgaggtcgccaagtggtaaggcagcgggttttggtcccgccattcgtg

ggttcgaatcctactctcagaacca

1–13tRNA–Trp(cca)[119538,119611]

gttgggttggtctaatggtaagatgcaggtctccaaaaccttgcgatggg

ggttcaaatccctcacccttcgcc

1–14tRNA–Arg(acg)[119612,119685]

gctctcttagctcagtggaacagagcgtttggctacgaaccaaaaggtca

caggttcaaatcctgtagggggct

1–15tRNA–Val(tac)[119727,119798]

gggcgattaactcagcggtagagttcttgccttacaagcaagcagtcact

ggttcgaatccagtatcgccca

1–16tRNA–Pro(tgg)[120240,120313]

cggggtgtagctcagtttggtagagcactcgctttgggagcgagtggccg

taggttcgaatcctatcaccccga

1–17tRNA–Gly(tcc)[123000,123071]

gcgggtgtagttcagtggtagaacgctatccttccaagttagatgtcgtc

ggttcgagtccgatcacccgct

1–18tRNA–Ile(tat)[163382,163456]

gggactattgcttataggttaaagccctctgcttataacggagtgaaccg

agttcgagtctcggtagtcctacca

1–19tRNA–Met(cat)[163460,163533]

gctggtttagctatctggtgaaagcacccgactcataatcggatacaggc

gagttcgatcctcgcaaccagcac

1–20tRNA–Leu(taa)[163577,163662]

tggggacatggtggaattggtagacacaccagacttaaaatctgttgagc

gtatgctcgtgggggttcaactccccctgtccctat

1–21tRNA–Thr(tgt)[164474,164547]

gcctgagtagctcagttggatagagcaacgcttttgtaaagcgtaggtcg

tcggttcgagtccgaccttgggct

1–23tRNA–Ala(tgc)[164733,164806]

ggggaattagctcagttggtagtagcgtttgctttgcaagcaaaatgtca

ggagttcgagtctcctattctcca

1–24tRNA–Met(cat)[164824,164900]

cgcgggatggaacagtctggtagttcagcggtctcataagccgcaggtcg

tgggttcaaatcccactcccgctccca

1–25tRNA–Asn(gtt)[165144,165218]

ttcccttatagctcaattggcagagtaggtgactgttaatcacttggttc

ctggttcgagtccaggtgggggagt

1–26tRNA–Leu(tag)[165220,165302]

gcccaagtggcggaattggtagacgcgctgggtttaggttccagtgaatt

aattcgtggaggttcaagtcctctcttgggcac

>AY303349.1 Enterobacteria phage RB69, complete genome

1−1tRNA−Arg(tct)c[74783,74861]
cgaggcatagctcaattgtatagagcaacggggacttctaatccgtaggt
tgaaggttagaatccttctgtctcgacca
1−2tRNA−Met(cat)c[74868,74941]
ggccctgtagctcaacggttagcagcagtcccctcataagggaaaggtta
ccagttcgaatctggtctgggtca
>AY954970.1 Staphylococcus phage Twort, complete genome
1−1tRNA−Met(cat)c[107491,107564]
tggactcttagcttaatggtaaagcaaacgcctcataagcgttggagtgt
aggttcaatccctacagagtccat
>AY954969.1 Bacteriophage G1, complete genome
1−1tRNA−Met(cat)c[110652,110723]
ggactcttagcttaaaggtaaagccaaccgctcataacggtttgactgta
ggttcgaatcctgcagagtcca
1−2tRNA−Trp(cca)c[133870,133941]
acacccttagtataattagtagtacaagggtctccaaaacccttagtctt
tgtgcaaatcaaagagggtgtg
1−3tRNA−Phe(gaa)c[133948,134020]
ggtttcttagctcagatggtagagcactagattgaagctctaggtgtcat
tggttcaaatccaatagaaacca
1−4tRNA−Asp(gtc)c[134026,134101]
tggctcattggtgtaactggttaacacactgccctgtcacggcagagagt
acgagttcgagtctcgtatgggtcgt
>AY939844.2 Prochlorococcus phage P−SSM2, complete genome
1−1tRNA−Asn(gtt)[162082,162169]
ttgtgaggtgacgaaattggtaaacgtgtcagtctgtttaactgatgttc
ctggcgggacttgaaggttcgactccttccctcacagt
>AY962392.1 Aeromonas phage 31, complete genome
1−1tRNA−Arg(tct)c[65028,65104]
ggtctcttagctcagctggacagagcaacgtcgttctaagtcgttggtca
ttggttcgaatccaatagggaccacca
1−2tRNA−Met(cat)c[66073,66147]
ggccctgtagctagacggttcaagcaggcggctcataaccgctcgtagca
ggttcgattcctgccagggccacca
1−3tRNA−Met(cat)c[66354,66430]
tgcgacgtagaggagaggtcgtcctcgtcgggctcatatcccggaaatcg
gtggttcgaatccatccgtcgcatcca
1−4tRNA−Asp(gtc)c[66452,66525]
ggagccatagtttatacggttaaaataatcccctgtcacgggatagcacc
gagttcgatcctcggtggctccgc
1−5tRNA−Ser(gct)c[66599,66686]
ggagaagtggatgagcggtttaaatcgcttccctgctaaggaagtaaacc
gaaaggtttcgagagttcgaatctctccttctccgcca
1−6tRNA−Ile(gat)c[66694,66769]

agtggattagctcagtaggtagagcactcgaccgataatcgagagcgcac
tggttcgaccccagtatccactacca
1−7tRNA−Thr(tgt)c[67198,67273]
gccgatttagctcatccggtagagcagttgttttgtaagcatcaggtggt
ccgttcgagtcggacaatcggcacca
1−8tRNA−Pro(tgg)c[67913,67989]
ccgcgtgtaggctagtcaggtaggtcgtctggtttgggaccagaaagtcg
gaggttcgaatccttccacgcggacca
1−9tRNA−Gly(tcc)c[68496,68571]
gcgggtaaggtgtttacggatacatgctagccttccaagcttgagtagac
cggttcgataccggctacccgctcca
1−10tRNA−His(gtg)c[68584,68658]
gtgacggtagctcagctggtagagccccaggttgtgatcctggtcgtcgc
gagttcaatcctcgtccgtcacccc
1−11tRNA−Lys(ttt)c[68950,69025]
gcgccgctagcttagttggtagagcatccggcttttaacgggagggtcga
tggttcgaatccatcgcggcgcacca
1−12tRNA−Tyr(gta)c[69083,69165]
ggaacgttcgggtaatggtatcccaagcggctgtaacccgctcgcctctg
gcattcttggttcgagtccaaggcgttccacca
1−13tRNA−Asn(gtt)c[69661,69746]
ggtgatgtggctcagaggttgagcggcggactgttaatccgcgaggaatt
tcctctatactggttcgagtccagtcatcaccgcca
1−14tRNA−Phe(gaa)c[69828,69912]
gttgctgtgagccgagtggctgaaggcggtctctggaaacgggacgggtt
gaaagacctcgaaggttcgaatccttccagcaacc
1−15tRNA−Ser(tga)c[69983,70076]
ggaagcttggcagagtctggcttattgcactagtcttgaaaactagcgat
cgtaggaatacggtccatccgttcaaatcggatagcttccgcca
1−16tRNA−Leu(taa)c[70267,70350]
gggagtgtggtggaatggtatacactggagacttaaaatctcccgccttc
gggattgagggttcgagtccctcctctcctacca
>AY967407.1 Enterobacteria phage RB43, complete genome
1−1tRNA−Met(cat)[50172,50248]
ggcccttttagctcaattggtagagcgaacccctcataagggtgtggtttc
cggttcgagtcacggaaggggccacca
>AJ697969.1 Pseudomonas phage phiEL complete genome
1−1tRNA−Thr(cgt)[102946,103021]
tccgtgatagctcagtaggtagagcaactgtttcgtaaacagtaggtccc
gggttcgaaccctggtcacggaacca
>DQ320509.1 Lactobacillus phage KC5a, complete genome
1−1tRNA−Gln(ttg)[12499,12573]
taggactatagccaaattggtaaggcatcaggttttgatcctgtgtattg
ttggttcgagcccagctagtcctat

1−2tRNA−Tyr(gta)[13163,13246]
tagcaacttagttcaatgggagaacaaagcgtgtacagttaaacaatgtg
cttgagaaggtggttcgattccatcagttgctgt
>DQ529280.1 Aeromonas salmonicida bacteriophage 25, complete genome
1−1tRNA−Arg(tct)c[61945,62020]
tggtcccttagctcagcaggatagagcagcaaccttctaagtcgctggcc
atcggtccaatcccataggactaccc
1−2tRNA−Met(cat)c[62593,62668]
ggccctctgcacagtctgtgaatgtgtccagctcataactggggtaaggc
gggttagattcccgcgagggccacca
1−3tRNA−Met(cat)c[62876,62952]
tgcgacgtagaggagaggtcgtcctcgtcgggctcatatcccgaaaatca
acggttcgaatccgttcgtcgcatcca
1−4tRNA−Asp(gtc)c[62974,63047]
ggagccatagtttatacggttaaaataatcccctgtcacgggatagcacc
gagttcgatcctcggtggctccgc
1−5tRNA−Ser(gct)c[63125,63212]
ggagaagtggatgagcggctgaaatcgcttccctgctaaggaagtaaacc
gaaaggtttcgagagttcgaatctctccttctccgcca
1−6tRNA−Ile(gat)c[63220,63295]
agtggattagctcagtaggtagagcactcgaccgataatcgagagcgcat
tggttcgatcccaatatccactacca
1−7tRNA−Trp(cca)c[63298,63373]
atgacattggtgttagcggtagcatgccggtctccaaaaccgtgcggcca
gggttcgaatccttgatgtcatgcca
1−8tRNA−Thr(tgt)c[63738,63813]
gccaatttagcacagcggtaggtgcaacgtatttgtaatacgtaggtcgt
cagttcgaatctgacaattggctcca
1−9tRNA−Pro(tgg)c[64074,64152]
ccgtgtgtaggctagtcaggttaggtcgtctggtttggggccagaaagtc
gaaggttcgaatcccttccacacggacca
1−10tRNA−Gly(tcc)c[64397,64472]
gcgaatatagctcagttggtagagcttctgccttccaagcagaatgtcat
cggttcgatcccgattattcgctcca
1−11tRNA−Asn(gtt)c[64600,64682]
ggtgatgtagcataacggccatgcggccggctgttaaccggcgaggaaac
tctacgttggttcgactccaaccatcaccgcca
1−12tRNA−Ser(tga)c[64851,64944]
ggaagattggcccgagttggtttaaggcaccggtcttgaaaaccggcgat
cgtaggaatacggtccatccgttcgaatcggatatcttcctcca
1−13tRNA−Leu(taa)c[65136,65220]
gcaagtatggtggaattggtatacactggaggcttaaaacctcccgcctt
cgggattgtgggttcgagtcccactacttgcacca
>DQ997624.1 Thermus thermophilus phage YS40, complete genome

1–1tRNA–Thr(tgt)[52068,52143]
ggcctcgtagctcaacaggaagagcactcgccttgtaagcggggggttgt
gggttcaaatcccaccggggcctcca
1–2tRNA–Met(cat)c[120023,120099]
ggggcgttagctcaagcggtgagagcggtcggctcataaccgattggttg
taggttcgagtcctacacgccccacca
1–3tRNA–Arg(tct)c[120112,120188]
gaggggttagctcaacaggacagagcaagggatttctaatccctaggttg
caggttcaagtcctgcacccctcgcca
>DQ398053.1 Mycobacteriophage Catera, complete genome
1–1tRNA–Ser(gct)[30008,30091]
ggagggtgagcatctggtgatgcaggggtcctgctaaggccctacggatt
cacacccgtgagtttcgattactcctccctccgc
1–2tRNA–Leu(cag)[30188,30264]
gccctgctgagcaaactggcaaagctgccgcattcagagtgcgggtcatt
tccgggttcgactcccgggcagggtac
1–3tRNA–Leu(gag)[30384,30458]
gtctctgtaggcaaatcggaaaagccgccatcttgagggggtggtgcgtg
cgggttcgactcccgccagagacac
1–4tRNA–Leu(caa)[30459,30532]
gccgtggtaggccatctggcgagccgccgagttcaagtttcggtgtttgc
gggttcgaatcccgcccacggtac
1–5tRNA–SeC(tca)[66782,66878]
attctggcactggtgggcgagcccaccggcggcagcttcaagctgtcgct
ggccggagaaccgaccggaaacatcccgttcaacgcgacccagggcc
1–6tRNA–Pro(tgg)[90077,90151]
cggggtgtagttcagtttggaagagcgcttggtttgggaccaagatgtcg
caggttcgaatcctgtcaccccgac
1–7tRNA–Trp(cca)[90166,90236]
gggtctgtgcacagggtgcccgacggtctccaaagccgaaggcgggggtt
cgattccctccaggcctgcca
1–8tRNA–Tyr(gta)[90238,90324]
cccgtacatgcccaactggtgtttgggagcaggctgtaaatctgtggcct
tcgggacggtgaggttcgattcctcagtgcgggacca
1–9tRNA–Pyl(cta)[91378,91451]
tgcgagatcgtgcacggcgcactaggagcttctaaccctccgactcgcgg
gttcgactcccgcatctcgcaccc
1–10tRNA–Met(cat)[91599,91673]
agcggtgtagagcagctaggtagctcgccgggctcatgacccggaggacg
cgtgttcgattcacgccaccgccac
1–11tRNA–Cys(gca)[91800,91871]
gcgcctttggcggaatggctacgtgctcggctgcaacccgagttatcccg
gttcgactccgggagggcgctc
1–12tRNA–Glu(ctc)[91876,91950]

ggtccgttggagtagatggacatctcgccaccctctcaaggtggagatca
cgggttcaagtcccgtacggactgc
1–13tRNA–His(gtg)[91952,92025]
gtggccgtagttcagccggtagaacgctgggttgtgatcccagtcgtcga
gggttcgagtccctccggtcaccc
1–14tRNA–Ala(tgc)[92187,92261]
gggcctgtagctccaattggtagagcagcatccttgcaagatgacggctg
tcggttcgaatccgacctggtccac
1–15tRNA–Phe(gaa)[92451,92523]
gccgtcatagctcagttggtagagcaccggcctgaaaacccggtggccga
ggttcgagtcctcgtgtcggcac
1–16tRNA–Val(cac)[92529,92602]
gtccgtttagctcagctggaagagcgctcggcccacacccgagaggccgc
aggttcgatccctgcaatggacac
1–17tRNA–Lys(ctt)[92721,92793]
gccttcgtagctcagtggtagagctgtcgcctcttaagcgataggtcgtt
ggttcaaatccagccgggggcac
1–18tRNA–Glu(ttc)[92797,92871]
ggtcgggtggtctgttggcaggccggtcggattttcactccggacattcg
cgggttcaattcccgtcccgatcgc
1–19tRNA–Gly(tcc)[92951,93023]
gcgggtgtggccgaatggctcaggcaccagatttccactctggctacgca
ggttcgattcctgtcatccgctc
1–20tRNA–Thr(cgt)[93083,93156]
gctgctgtagctcacctggcagagcgtcggcgtcgtatcccgaaggcatc
cggttcgagtccggacagcagctc
1–21tRNA–Thr(tgt)[93165,93238]
gcccttgtagctcagtggcagagcaccgaccttgtaagtcgggttgtccc
gggttcgattcctggtaggggctc
1–22tRNA–Thr(ggt)[93747,93821]
gctactgaggcccatatggatgggcgcggccatggtaaggccgaggtaga
tgggttcaagtccctccagtagctc
1–23tRNA–Gly(gcc)[95144,95217]
gcgaaggtagctcagctggcagagcgccaccttgccaaggtggaggtcgc
gggatcgtaacccgttcttcgctc
1–24tRNA–Asp(gtc)[95221,95293]
ggccctgtagctcagaggaagagcgccgatctgtcgaatcggaggtcgcg
gtatcgtaatccgtcagggtcgc
1–25tRNA–Met(cat)[95353,95425]
gcctcactagctcattggtagagccgctcgctcataacgtgcaggtacct
ggttcgattccagggtgaggtac
1–26tRNA–Ile(gat)[95431,95505]
gcctgttagcggactggtcgtccgatccaagctgataacttggcgtaagc
ggtgttcgattcaccgagcaggtac

1–27tRNA–Arg(acg)[95599,95671]
gcctctatggtccaacggatatgacgccggtctacggaaccggagatgcg
tgttcgattcgcgctaggggcac
1–28tRNA–Val(gac)[95714,95786]
gtccgtgtagctcaggggtagagcgcctgctcgacacgcaggaggaccga
ggttcgaaacctcgcatggacac
1–29tRNA–Arg(cct)[95960,96033]
gcctctgtagctcaacggacagagcaacgcggtcctaacgcggtggctgg
aggttcgaatcctctcggaggcac
1–31tRNA–Gln(ttg)[96574,96649]
tggggtatggtggcaatctggcagtcccgccggactttgactccggaggt
gcaggttcgagtcctgctaccccatc
1–32tRNA–Arg(tct)[96653,96726]
gcccttgtagctcagtggacagagcggcgagcttctacctcgcgggccgg
gagttcgaatctctccaggggcac
1–33tRNA–Lys(ttt)[96794,96870]
gggccggtatcttagtctggtcaaagaagtggacttttaatccgcgcgcc
gtgggttcgaatcccacccggcccacc
1–34tRNA–Gln(ctg)[124401,124475]
tgctcgttggtgtaactggcaacactacggactctgactccgtcatttta
ggttcgaatcctaagcgagcagcca
1–35tRNA–Asn(gtt)[124482,124557]
tggggtgtccgttaatcaggcaaacgagcggactgttaatccgcccctgc
aggttcgaatcctgccaccccagcca
>DQ149023.2 Synechococcus cyanophage syn9, complete genome
1–1tRNA–Val(tac)[15973,16044]
gggcgaatagctcagcggtagagctactcgtttacaccgagtcggtcggg
ggttcgatcccctcttcgccca
1–2tRNA–Leu(taa)[19727,19813]
tgggagcgtggcggaatcggtagacgcaccagacttaaaatctgttgaga
attaatctcgtgggggttcaattccccccgctcctat
1–3tRNA–Thr(tgt)[20001,20075]
gccaaactagctcagctggatagagcaacggttttgtaaaccgtaggtca
acggttcaagtccgttgtttggctc
1–4tRNA–Ala(tgc)[20078,20150]
ggggaattagctcagttggtagagcgcctgctttgcaagcaggatgtcag
cggttcgagtccgctattctcca
1–5tRNA–Asn(gtt)[20225,20298]
ttcctctttagctcagcggtagagcgattgactgttaatcaattggtccc
tggttcgatcccaggaaggggagt
1–6tRNA–Arg(tct)[162261,162336]
tgggtcagtagctcagctggatagagcaactgccttctaagcagtcggtc
acaggttcgaatcctgtctgacccgt
>DQ904452.1 Bacteriophage RB32, complete genome

1−1tRNA−Arg(tct)c[67482,67558]
cggggcatagctcaattgtatagagcaacggacttctaatccgtaggttg
aaggttagaatccttctgtctcgacca
1−2tRNA−Met(cat)c[68269,68340]
ggccctgtagctggaaggttcaagcaagcgactcataatcgccagatggt
ggttcaattccacccagggcca
1−3tRNA−Thr(tgt)c[68342,68417]
gctgatttagctcagtaggtagagcaactcacttgtaatgagaaggtcgg
cggttcgattccgtcaatcagcacca
1−4tRNA−Ser(tga)c[68419,68507]
tggaggcgtggcagagtggtttaatgcaccggtcttgaaaaccggcagtc
gctccggcgactcataggttcaaatcctatcgcctccgt
1−5tRNA−Pro(tgg)c[68508,68582]
ctccgtgtagctcagtttggtagagcgcctgatttgggatcaggaggtcc
aaggttcaaatccttgtatggagac
1−6tRNA−Gly(tcc)c[68593,68666]
gcggatatcgtataatggcattacctcagacttccaatctgatgatgtga
gttcgattctcattatccgctcca
1−7tRNA−Leu(taa)c[68672,68758]
gcgagaatggtcaaattggtaaaggcacagcacttaaaatgctgcggaat
gatttccttgtgggttcgagtcccacttctcgcacca
1−8tRNA−Gln(ttg)c[68759,68832]
tgggaattagccaagttggtaaggcatagcactttgactgctagatgcaa
aggttcgagtcctttattcccagc
>AB231700.1 Microcystis phage Ma−LMM01 DNA, complete genome
1−1tRNA−Met(cat)c[34160,34232]
gcaggattggccgataggtggaggcaccgtcctcataagacggcttagag
cggttcgattccgctatcctgca
1−2tRNA−Tyr(gta)c[145179,145261]
gggttagtggccgagtagtcgaaggcgacagactgtaaatctgtagagta
attccacgctggtgcaaatccagcctaacccac
>EF583821.1 Bacillus phage 0305phi8−36, complete genome
1−1tRNA−Val(aac)c[183119,183194]
tgtcatcgtagcttaataggtaaagcgctgacctaacaatgtcagagaat
ggggttcgaaaccttacatgatgacc
>DQ003638.2 Listeria phage A511, complete genome
1−1tRNA−Met(cat)c[25835,25908]
ttgtcccgtagctagaaggtcgagcaaggagctcataactcctcggtttg
ggttcgattcccaacggggcaatc
1−2tRNA−Pro(tgg)c[26802,26876]
cagggtgtagctcagtttggttagagtacccgctttggagacgggaagtc
gtaggttcgaatcctaccaccttga
1−3tRNA−Arg(tct)c[27994,28064]
gtccttatggtgtagcggatgcacaagggatttctactcccttagcgcgg

gttcgaatcctgctgaggact
1−4tRNA−Gly(tcc)c[28311,28381]
gcgggtatagtataagggtagtaccaaaggtttccaaccatgtagtgggg
gttcgaatccccctacccgct
1−5tRNA−Asn(gtt)c[28450,28523]
gtgtccttaactcagaggtcagagtgccgtcctgttaagtcggaagtcgc
tggttcaaatccagcaggatacgc
1−6tRNA−Ser(tga)c[29144,29235]
ggaaggttggtagagcttggtaatacgctagtcttgaaaactagtttgcc
cttggaatacagggtacaagggttcaaatcccttaccttcct
1−7tRNA−Phe(gaa)c[29248,29319]
gtagtcctagctgagatggattagcgcttgcttgaaaagcaagagaggca
ggttcgatacctgcggactcca
1−8tRNA−Lys(ttt)c[29325,29396]
ggagttatggtgaaatggctatcactgcgggtttttaccccgttattcta
ggttcgaatcctagtggctcca
1−9tRNA−Trp(cca)c[29521,29594]
taggggtatagtttatctggtaaaatattggtttccaactccaatgaggt
gggttcaagtcctactatccctgt
1−10tRNA−Gln(ttg)c[29596,29668]
tggtctgtagccaagcggtaaggcaacggattttgattccgtgatacgtt
ggttcgaatccaactagaccagc
1−11tRNA−Thr(tgt)c[29687,29758]
gcttgtatagttcaattggtagaacagtggttttgtaagcctcagacgtg
ggttcaagtcctactacaagca
1−12tRNA−Tyr(gta)c[29841,29922]
gtgccattcgcatagaggcaattgcgggggactgtaactcccctcccttc
ggggttccaaggttcgagtccttgatggcgca
1−13tRNA−Leu(tag)c[30283,30367]
tgccgagatggtggaactggtatacacggtagacttagaatctgctgtcc
caaggatatgtgggttcgaatcccactctcggtat
1−14tRNA−Asp(gtc)c[30833,30905]
gtgcgtatgatataatggctattatactcgactgtctatcgagaaatagg
ggttcgattccccttacgtgcgc
1−15tRNA−Ile(gat)c[31009,31081]
accagcatagcttaggaggcaaagcaaccgaccgataatcggtagtcctt
ggttcaattccaagtgttggtac
1−16tRNA−Ser(gct)c[31258,31344]
ggagagttgtcagagaggcttaatgatacgggttgctaactcgttgtact
agtaatagtaccaagggttcgaatcccttactctcct
1−17tRNA−Cys(gca)c[31426,31496]
gcgggtataaccaactggaaaggtagtagactgcaaatctacgtatatgg
gttcaattcccattacccgct
>EF460875.1 Enterobacteria phage phiEcoM−GJ1, complete genome

1-1tRNA-Leu(cag)[14456,14550]
ggacgagtagctcagaaggtttgcattgtgctcagtcaggtagagcaatc
tgttcgacagatgtgtcgttggttcgaatccaaccttgtccgcca
>AP009390.1 Enterococcus phage phiEF24C DNA, complete genome
1-1tRNA-Met(cat)c[136981,137054]
ggacgtttagctcagttggtcagagcattcggctcataaccgaacggtcg
caggttcgagacctgcaatgtcca
1-2tRNA-Leu(tag)c[137677,137761]
gcagaagtgatggaactggtagacaacggtgtcttagaaacatcggtctg
taatggacgtgtgggttcgactcccgccttctgca
1-4tRNA-Arg(tct)c[138482,138557]
tgtaggtgtagctcaataggatggagcatccgccttctaagcggacggtt
gggggttcgattccctccatctacgt
1-5tRNA-Trp(cca)c[139156,139229]
tagtcggttagtgtaactggtaacacgttggtctccaaaaccaataatag
gggttcaaatcctctaccgattgt
1-6tRNA-Asp(gtc)c[139479,139554]
tggcagtatagggcagaggttgtcccaacacgttgtcagcgtggaacaca
cgggttcgagtcccgttactgtcgtc
>EF469154.1 Enterobacteria phage JS98, complete genome
1-1tRNA-Arg(tct)c[70206,70281]
cgaggcatagctcagaaggaagagcaacggtcttctaaaccgtaggtcgt
aggttcgatccctactgtctcgacca
1-2tRNA-Asn(gtt)c[70286,70360]
ggatgtgtagctcagcggtagagcagttgactgttaatcaattggtccat
ggttcgaatccatgcatgtccgcca
1-3tRNA-Met(cat)c[70365,70438]
ggccctgtagctggacggtcaagcgagcgactcataatcgctggatggtg
gttcgattccacccagggccacca
>EF602154.1 Burkholderia phage BcepNY3, complete genome
1-1tRNA-Tyr(gta)[16343,16426]
ggaggcgtggctgagtggtcgaaagcggcggactgtaaatccgttaccaa
ggtcgcggtggttcgaatccacccgtctccacca
>EU197055.1 Pseudomonas phage 201phi2-1, complete genome
1-1tRNA-Leu(taa)c[300263,300349]
gccggtgtgatcgaattggtatagatagcggacttaaaatccgccgcccg
taagggattgtgggatcgaggcccaccaccggcacca
>DQ832317.1 Escherichia coli bacteriophage rv5, complete sequence
1-1tRNA-Pro(tgg)c[61976,62052]
ctccgcgtagctcagcttggtagagcgcctgatttgggatcaggaggtcg
agtgttcgaatcactccgtggagacca
1-2tRNA-Met(cat)c[62064,62138]
ggccctgtagctggaaggttcaagcaagcgactcataatcgccagacggt
ggttcaattccacccagggccacca

1−3tRNA−Thr(tgt)c[62140,62215]
gctgatgtagcacaatcggtagtgcaattgatttgtaatcaataggttgt
aggttcaagtcctgccatcagcacca
1−5tRNA−Ser(tga)c[62418,62508]
ggagggtagggagcaatggtgctcaagcggtcttgaaaaccgtcccgttg
aggatgactcgatgatggttcgattccattactctccgcca
1−6tRNA−Tyr(gta)c[62623,62709]
ggggagttatcccgtagaggtagcggtgtagactgtaaatctattgtcat
tgcgactcgggtggttcgactccaccactccccacca
1−7tRNA−Arg(tct)c[62714,62788]
gccctgttagcttagtggataaagcagcggccttctaagccgttgacact
ggttcgagtccagtacggggtgcca
>AB366653.2 Ralstonia phage RSL1 DNA, complete genome
1−1tRNA−Gly(tcc)c[186301,186376]
gcgggcgtagctcagttggtagagttcttgccttccaagcaagatgtcgc
gagttcgagtctcgtcgcccgctcca
1−3tRNA−Pro(tgg)c[186529,186606]
ccgagtgtagcgcagtctggttagcgcacctgatttgggatcagggggtc
ggaggttcgaatcctctcactcggacca
>EU826466.1 Mycobacterium phage Myrna, complete genome
1−1tRNA−Ser(gct)[32466,32548]
ggagggtgagtatcaggtgatacagcgagattgctaatcccgtacggctc
atacccgtgaggttcaagtcctcctccctccgc
1−2tRNA−Leu(cag)[32662,32737]
gcccccgtgggcaaattggcaaagtcgctgcactcagactgcagagtttt
ccgggttcgagtcccggcgggggtac
1−3tRNA−Leu(gag)[32799,32872]
ggaccggtagcccaaaggcagaggcagcacgttgaggacgtgtccagtgc
gggttcgactcccgctcggtctac
1−4tRNA−Leu(caa)[32873,32955]
gctccagtggcgtaatggcagccgcgccgcactcaaaatgcggtgtccgt
aggacgtgcgagttcgagtctcgcctggagtac
1−5tRNA−Pyl(cta)[33522,33593]
gcgagattggtctggtgacctagcgagcttctaaacctcgcgatcgaccg
gttcgactccggtatctcgcgc
1−6tRNA−Pro(tgg)[91832,91904]
cgggatatggtgtaagggttagcacgattggtttgggaccattctgacgg
ggttcgagtccctgtttcccgac
1−7tRNA−Tyr(gta)[92391,92475]
gccgtgtatgcccaactggtgtttgggagcaggctgtaaccctgtggcct
tcgggacggcggagttcgattctctgacgcggcac
1−8tRNA−Met(cat)[92614,92688]
agcggggtagagcagctcggtagctcgccaggctcataacctggaggccg
tgggttcaaatcccactcccgccac

1-9tRNA-Ala(tgc)[92828,92901]
ggggatgcatgttccttgggggcgactgatccttgcaagatcggtgaggt
gggttcgattcccatcatctccac
1-10tRNA-Cys(gca)[92905,92977]
gtgctgatggccgagatggttaggcactgggttgcaaccccaggcacgca
ggttcgagtcctgttcggcactc
1-11tRNA-Glu(ctc)[93010,93081]
ggtcctatggggtagtggtagcccatctccctctcacggagacggcccga
gttcaattctcggtaggactgc
1-12tRNA-His(gtg)[93083,93155]
gtggccatagttcaatggcagaacgccgggttgtgatcccggtcgcgagg
gttcgattccctctggtcacccc
1-13tRNA-Gly(tcc)[93156,93227]
gcgctcgtggcatattggttgtgctccagccttccaagctggctaagcag
gttcgattcctgtcgggcgctc
1-14tRNA-Ala(ggc)[93384,93457]
ggggctgtagctcaatcggtagagcgtctcgctggcagtgagaaggcagt
cggttcgatcccgaccagttccac
1-15tRNA-Phe(gaa)[93490,93562]
gccttcatagctcagttggtagagcgtcggactgaaaacccggaggccgt
ggttcgattccacgtgttggcac
1-16tRNA-Val(cac)[93566,93634]
gctcctctagtctgggacagaagtcgggtcacatccgatcgagcggggtt
cgattccctgggggagtac
1-17tRNA-Lys(ctt)[93647,93721]
ggccctagagcacggtctggtagtgctacggactcttaatccgcaggtcg
taggttcaaatcctactggggccac
1-18tRNA-Lys(ctt)[93732,93805]
gcgctcgtagctcagctggtagagctgctgactcttaatcagcatgtcgg
gggttcgagtccctccgggcgtac
1-19tRNA-Gly(gcc)[94110,94183]
gtgcttgaggtgtaaatggtcgcacgacaccttgccaaggtgtaggaagc
cggttcgatcccggtcaggcactc
1-20tRNA-Asp(gtc)[94262,94337]
ggcctatcggtccagtctggagtggacaccgccctgtcacggcggagagc
acgggttcaaatcccgtataggtcgc
1-21tRNA-Met(cat)[94418,94491]
gcctgtgtagctcatctggcagagcgccctgctcataacggggaggtaag
tggttcgagaccactcataggtac
1-22tRNA-Ile(gat)[94589,94663]
gcctgttagcggactggtcgtccgatccaagctgataacttggcgcaagg
agtgttcgattcactcagcaggtac
1-24tRNA-Arg(acg)[94809,94882]
ctcccggtggttcaacggatagagcgccagactacgaatctggagagtgg

gggttcgattccctctcgggagac
1–25tRNA–Met(cat)[94903,94977]
gtcggttatcgtttaacggaaaaacaattcactcataatgaatcactccg
ggttcgagtcccggttccggctcca
1–26tRNA–Thr(ggt)[94998,95069]
gctggattagcatagtggtagtgcccttccttggtatggaagtagccggg
gttcgattccccgattcagctc
1–27tRNA–Val(gac)[95075,95147]
gctcttgtagctcagtggtagagcaccagcccgacatgctggttgtccga
ggttcgattcctcgttggagcac
1–28tRNA–Thr(tgt)[95326,95397]
gcctccatcgttcagtggtaggacgcctgccttgtaagcaggctgcgggc
gttcgattcgtcctggaggctc
1–29tRNA–Glu(ttc)[95403,95477]
ggttcggtggtctgctggctgtgccactcaggttttcaccctgggatacg
cgggttcgattcccgtccgaactac
1–30tRNA–Thr(cgt)[95561,95635]
gctggtgaagcgtgaacggtttacgcacggccctcgtaacgccgagaagt
cggttcgactccgacctccagctcc
1–31tRNA–Arg(cct)[96160,96234]
gccgctgtagctcatgcggacagagcgaccgtttcctaaacggtaggcag
ggggttcgagtcctcccagcggtgc
1–32tRNA–Gln(ttg)[96267,96340]
tgaggtgtggtctgctggcaggccagcgggcctttgacccccgcctccgt
aggttcgattcctgccacctcagc
1–33tRNA–Arg(tct)[96341,96416]
gcctctgtagctcagtggatagagcaccgagtttctacctcgggtgccgg
gagttcgatcctctccaggggcacca
1–34tRNA–Lys(ttt)[96419,96492]
ggggcgttggtgaacgcggctatcactcttggcttttacccttgaatacc
gggatcgagacccggacgccccac
1–35tRNA–Gln(ctg)[122545,122616]
tgcctgttggtgtaatggcaacacgccggtttctgacaccggtattcttg
gttcgagtccagggcgggcagc
1–36tRNA–Asn(gtt)[122636,122710]
tggtcactagctcaattaggcagagcaacgccctgttaaggcgtcaggtg
aaggttcgagtccttcgtggccagc
>EU826468.1 Mycobacterium phage Spud, complete genome
1–1tRNA–Ser(gct)[31867,31950]
ggagggtgagcatctggtgatgcaggggtcctgctaaggccctacggatt
cacacccgtgagtttcgattactcctccctccgc
1–2tRNA–Leu(cag)[32047,32123]
gccctgctgagcaaactggcaaagctgccgcattcagagtgcgggtcatt
tccgggttcgactcccgggcagggtac

1-3tRNA-Leu(gag)[32243,32317]
gtctctgtaggcaaatcggaaaagccgccatcttgagggggtggtgcgtg
cgggttcgactcccgccagagacac
1-4tRNA-Leu(caa)[32318,32391]
gccgtggtaggccatctggcgagccgccgagttcaagtttcggtgtttgc
gggttcgaatcccgcccacggtac
1-5tRNA-Pro(tgg)[91905,91979]
cggggtgtagttcagtttggaagagcgcttggtttgggaccaagttgtcg
caggttcgaatcctgtcaccccgac
1-6tRNA-Trp(cca)[91994,92064]
gggtctgtgcacagggtgcccgacggtctccaaagccgaaggcgggggtt
cgattccctccaggcctgcca
1-7tRNA-Pyl(cta)[93255,93327]
gcaccatttgctcaatggcagagcggcggcttctaaaaccgtgagtgccg
gttcgactccggcatggtgcacc
1-8tRNA-Met(cat)[93477,93551]
agcggtgtagagcagctaggtagctcgccgggctcataacccggaggacg
cgtgttcgaatcacgccaccgccac
1-9tRNA-Cys(gca)[93678,93749]
gcgcctttggcggaatggctacgtgctcggctgcaacccgagttatcccg
gttcgactccgggagggcgctc
1-10tRNA-Glu(ctc)[93754,93828]
ggtccgttggagtagatggatatctcgccaccctctcaaggtggagatca
cgggttcaagtcccgtacggactgc
1-11tRNA-His(gtg)[93830,93903]
gtggccgtagttcagccggtagaacgctgggttgtgatcccagtcgtcga
gggttcgagtccctccggtcaccc
1-12tRNA-Ala(tgc)[94065,94139]
gggcctgtagctccaattggtagagcagcatccttgcaagatgacggctg
tcggttcgaatccgacctggtccac
1-13tRNA-Phe(gaa)[94329,94401]
gccgtcatagctcagttggtagagcactggcctgaaaacccagtggccga
ggttcgattcctcgtgtcggcac
1-14tRNA-Val(cac)[94407,94480]
gtccgtttagctcagctggaagagcgctcggtccacacccgagaggccgc
aggttcgatccctgcaatggacac
1-15tRNA-Lys(ctt)[94599,94671]
gccttcgtagctcagtggtagagctgtcgcctcttaagcgataggtcgtt
ggttcgaatccagccgggggcac
1-16tRNA-Glu(ttc)[94676,94752]
ggtcgggtcggtctgctggtatggccagtcggattttcactccggacatt
cgcgggttcaattcccgtcccgatcgc
1-17tRNA-Gly(tcc)[94832,94904]
gcgggtgtggccgaatggctcaggcaccagatttccactctggctacgca

ggttcgattcctgtcatccgctc
1-18tRNA-Thr(cgt)[94964,95038]
gctgctgtagctcacctggcagagcgtcggcgtcgtatcccgaaggcatc
cggttcgagtccggacagcagcccc
1-19tRNA-Thr(tgt)[95039,95111]
gcctctgtggtccagcggcacggacatccgccttgtaagcggaggacccc
cgttcgatccggggtagaggctc
1-20tRNA-Thr(ggt)[95171,95243]
gctgggttagctcagtggtagagcgttcctctggtatgggaaaggcccgg
ggttcaatcccccgactcagctc
1-21tRNA-Gly(gcc)[96566,96639]
gcgaaggtagctcagctggcagagcgccaccttgccaaggtggaggtcgc
gggatcgcaacccgttcttcgctc
1-22tRNA-Asp(gtc)[96643,96715]
ggccctgtagctcagaggaagagcgccggtctgtcgaatcggaggtcgcg
gtatcgtaatccgtcagggtcgc
1-23tRNA-Met(cat)[96775,96847]
gcctcactagctcattggtagagccgctcgctcataacgtgcaggtacct
ggttcgattccagggtgaggtac
1-24tRNA-Ile(gat)[96853,96927]
gcctgttagcggactggtcgtccgatccaagctgataacttggcgtaagc
ggtgttcgattcaccgagcaggtac
1-25tRNA-Arg(acg)[97021,97093]
gcctctatggtccaacggatatgacgccggtctacggaaccggagatgcg
tgttcgattcgcgctaggggcac
1-26tRNA-Val(gac)[97136,97208]
gtccgtgtagctcaggggtagagcgcctgctcgacacgcaggaggaccga
ggttcgaaacctcgcatggacac
1-27tRNA-Arg(cct)[97382,97455]
gcctctgtagctcaacggacagagcaacgcggtcctaacgcggtggctgg
aggttcgaatcctctcggaggcac
1-29tRNA-Gln(ttg)[97996,98071]
tggggtatggtggcaatctggcagtcccgccggactttgactccggaggt
gcaggttcgagtcctgctaccccatc
1-30tRNA-Arg(tct)[98075,98150]
gcccttgtagctcagtggacagagcggcgagcttctacctcgcgggccgg
gagttcgaatctctccaggggcacca
1-31tRNA-Gln(ctg)[125262,125336]
tgctcgttggtgtaactggcaacactacggactctgactccgtcatttta
ggttcgaatcctaagcgagcagcca
1-32tRNA-Asn(gtt)[125343,125418]
tggggtgtccgttaatcaggcaaacgagcggactgttaatccgcccctgc
aggttcgaatcctgccaccccagcca

>FJ839693.1 Enterobacteria phage RB51, complete genome

1-1tRNA-Arg(tct)c[71249,71325]
cgaggcatagctcaattgtatagagcaacggacttctaatccgtaggttg
aaggttagaatccttctgtctcgacca
1-2tRNA-Asn(gtt)c[71330,71404]
ggatgtgtagctcagtggtagagcagttgactgttaatcaattggtccat
ggttcgaatccatgcatgtccgcca
1-3tRNA-Tyr(gta)c[71409,71495]
ggggagttatcccgtagaggtagcggtgtggactgtaaattcattgtcat
tgcgactcgggtggttcgactccatcactccccacca
1-4tRNA-Met(cat)c[71508,71582]
ggccctgtagctggaaggttcaagcaagcgactcataatcgccagatggt
ggttcaattccacccagggccacca
1-5tRNA-Thr(tgt)c[71584,71659]
gctgatttagctcagtaggtagagcaactcacttgtaatgagaaggtcgg
cggttcgattccgtcaatcagcacca
1-6tRNA-Ser(tga)c[71665,71754]
ggaggtgtggcagagtggttgaatgcaccggtcttgaaaaccggcagtcg
ctccggcgattcataggttcgaatcctatcacctccgcca
1-7tRNA-Gly(tcc)c[72416,72489]
gcggatatcgtataatggtattacctcagacttccaatctgatgatgtga
gttcgattctcattatccgctcca
1-8tRNA-Leu(taa)c[72495,72581]
gcgagaatggtcaaattggtaaaggcacagcacttaaaatgctgcggaat
gatttccttgtgggttcgagtcccacttctcgcacca
1-9tRNA-Gln(ttg)c[72582,72655]
tgggaattagccaagttggtaaggcactggattttgattccaggatgcaa
aggttcgagtcctttattcccagc
>EU826467.1 Mycobacterium phage Rizal, complete genome
1-1tRNA-Ser(gct)[30469,30552]
ggagggtgagcatctggtgatgcaggggtcctgctaaggccctacggatt
cacacccgtgagtttcgattactcctccctccgc
1-2tRNA-Leu(cag)[30649,30725]
gccctgctgagcaaactggcaaagctgccgcattcagagtgcgggtcatt
tccgggttcgactcccgggcagggtac
1-3tRNA-Leu(gag)[30845,30919]
gtctctgtaggcaaatcggaaaaagccgccatcttgagggggtggtgcgtg
cgggttcgactcccgccagagacac
1-4tRNA-Leu(caa)[30920,30993]
gccgtggtaggccatctggcgagccgccgagttcaagtttcggtgtttgc
gggttcgaatcccgcccacggtac
1-5tRNA-SeC(tca)[66513,66609]
attctggcactggtgggcgagcccaccggcggcagcttcaagctgtcgct
ggccggagaaccgaccggaaacatcccgttcaacgcgacccagggcc
1-6tRNA-Pro(tgg)[89818,89892]

cggggtgtagttcagtttggaagagcgcttggtttgggaccaagatgtcg
caggttcgaatcctgtcaccccgac
1-7tRNA-Trp(cca)[89907,89977]
gggtctgtgcacagggtgcccgacggtctccaaagccgaaggcgggggtt
cgattccctccaggcctgcca
1-8tRNA-Tyr(gta)[89979,90065]
cccgtacatgcccaactggtgtttgggagcaggctgtaaatctgtggcct
tcgggacggtgaggttcgattcctcagtgcgggacca
1-9tRNA-Pyl(cta)[91119,91192]
tgcgagatcgtgcacggcgcactaggagcttctaaccctccgactcgcgg
gttcgactcccgcatctcgcaccc
1-10tRNA-Met(cat)[91340,91414]
agcggtgtagagcagctaggtagctcgccgggctcatgacccggaggacg
cgtgttcgattcacgccaccgccac
1-11tRNA-Cys(gca)[91541,91612]
gcgcctttggcggaatggctacgtgctcggctgcaacccgagttatcccg
gttcgactccgggagggcgctc
1-12tRNA-Glu(ctc)[91617,91691]
ggtccgttggagtagatggatatctcgccaccctctcaaggtggagatca
cgggttcaagtcccgtacggactgc
1-13tRNA-His(gtg)[91693,91766]
gtggccgtagttcagccggtagaacgctgggttgtgatcccagtcgtcga
gggttcgagtccctccggtcaccc
1-14tRNA-Ala(tgc)[91928,92002]
gggcctgtagctccaattggtagagcagcatccttgcaagatgacggctg
tcggttcgaatccgacctggtccac
1-15tRNA-Phe(gaa)[92192,92264]
gccgtcatagctcagttggtagagcactggcctgaaaacccagtggccga
ggttcgattcctcgtgtcggcac
1-16tRNA-Val(cac)[92270,92343]
gtccgtttagctcagctggaagagcgctcggtccacacccgagaggccgc
aggttcgatccctgcaatggacac
1-17tRNA-Lys(ctt)[92462,92534]
gccttcgtagctcagtggtagagctgtcgcctcttaagcgataggtcgtt
ggttcgaatccagccgggggcac
1-18tRNA-Glu(ttc)[92539,92615]
ggtcgggtcggtctgctggtatggccagtcggattttcactccggacatt
cgcgggttcaattcccgtcccgatcgc
1-19tRNA-Gly(tcc)[92695,92767]
gcgggtgtggccgaatggctcaggcaccagatttccactctggctacgca
ggttcgattcctgtcatccgctc
1-20tRNA-Thr(cgt)[92827,92900]
gctgctgtagctcacctggcagagcgtcggcgtcgtatcccgaaggcatc
cggttcgagtccggacagcagctc

1−21tRNA−Thr(tgt)[92909,92982]
gcccttgtagctcagtggcagagcaccgaccttgtaagtcgggttgtccc
gggttcgattcctggtaggggctc
1−22tRNA−Thr(ggt)[93491,93565]
gctactgaggcccatatggatgggcgcggccatggtaaggccgaggtaga
tgggttcaagtccctccagtagctc
1−23tRNA−Gly(gcc)[94888,94961]
gcgaaggtagctcagctggcagagcgccaccttgccaaggtggaggtcgc
gggatcgtaacccgttcttcgctc
1−24tRNA−Asp(gtc)[94965,95037]
ggccctgtagctcagaggaagagcgccggtctgtcgaatcggaggtcgcg
gtatcgtaatccgtcagggtcgc
1−25tRNA−Met(cat)[95097,95169]
gcctcactagctcattggtagagccgctcgctcataacgtgcaggtacct
ggttcgattccagggtgaggtac
1−26tRNA−Ile(gat)[95175,95249]
gcctgttagcggactggtcgtccgatccaagctgataacttggcgtaagc
ggtgttcgattcaccgagcaggtac
1−27tRNA−Arg(acg)[95343,95415]
gcctctatggtccaacggatatgacgccggtctacggaaccggagatgcg
tgttcgattcgcgctaggggcac
1−28tRNA−Val(gac)[95458,95530]
gtccgtgtagctcaggggtagagcgcctgctcgacacgcaggaggaccga
ggttcgaaacctcgcatggacac
1−29tRNA−Arg(cct)[95704,95777]
gcctctgtagctcaacggacagagcaacgcggtcctaacgcggtggctgg
aggttcgaatcctctcggaggcac
1−31tRNA−Gln(ttg)[96318,96393]
tggggtatggtggcaatctggcagtcccgccggactttgactccggaggt
gcaggttcgagtcctgctaccccatc
1−32tRNA−Arg(tct)[96397,96470]
gcccttgtagctcagtggacagagcggcgagcttctacctcgcgggccgg
gagttcgaatctctccaggggcac
1−33tRNA−Lys(ttt)[96538,96614]
gggccggtatcttagtctggtcaaagaagtggacttttaatccgcgcgcc
gtgggttcgaatcccacccggcccacc
1−34tRNA−Gln(ctg)[124144,124218]
tgctcgttggtgtaactggcaacactacggactctgactccgtcatttta
ggttcgaatcctaagcgagcagcca
1−35tRNA−Asn(gtt)[124225,124300]
tggggtgtccgttaatcaggcaaacgagcggactgttaatccgcccctgc
aggttcgaatcctgccaccccagcca
>EU826471.1 Mycobacterium phage Cali, complete genome
1−1tRNA−Ser(gct)[31290,31373]

ggagggtgagcatcaggtgatgcagcgagattgctaatcccgtacggtaa
ccaccccgtgaggttcgaatcctcctccctccgc
1-2tRNA-Leu(cag)[31566,31641]
gctcccgtagcccaattggcaggaggcaccagattcaggatctgggcagt
gtgagttcgaatctcaccgggagtac
1-3tRNA-Leu(gag)[31761,31835]
gtctctgtaggcaaatcggaaaagccgccatcttgagggggtggtgcgtg
cgggttcgactcccgccagagacac
1-4tRNA-Leu(caa)[31836,31909]
gccgtggtaggccatctggcgagccgccgagttcaagtttcggtgtttgc
gggttcgaatcccgcccacggtac
1-5tRNA-SeC(tca)[68508,68604]
attctggcactggtgggcgagcccaccggcggcagcttcaagctgtcgct
ggccggagaaccgaccggaaacatcccgttcaacgcgacccagggcc
1-6tRNA-Pro(tgg)[92217,92291]
cggggtgtagttcagtttggaagagcgcttggtttgggaccaagatgtcg
caggttcgaatcctgtcaccccgac
1-7tRNA-Trp(cca)[92306,92376]
gggtctgtgcacagggtgcccgacggtctccaaagccgaaggcgggggtt
cgattccctccaggcctgcca
1-8tRNA-Tyr(gta)[92378,92464]
cccgtacatgcccaactggtgtttgggagcaggctgtaaccctgtggcct
tcgggacggtgaggttcgattcctcagtgcgggacca
1-9tRNA-Pyl(cta)[93578,93650]
gcaccatttgctcaatggcagagcggcggcttctaaaaccgtgagtgccg
gttcgactccggcatggtgcacc
1-10tRNA-Met(cat)[93800,93874]
agcggtgtagagcagctaggtagctcgccgggctcataacccggaggacg
cgtgttcgaatcacgccaccgccac
1-11tRNA-Cys(gca)[94001,94072]
gcgcctttggcggaatggctacgtgctcggctgcaacccgagttatcccg
gttcgactccgggagggcgctc
1-12tRNA-Glu(ctc)[94077,94151]
ggtccgttggagtagatggatatctcgccaccctctcaaggtggagatca
cgggttcaagtcccgtacggactgc
1-13tRNA-His(gtg)[94153,94226]
gtggccgtagttcagccggtagaacgctgggttgtgatcccagtcgtcga
gggttcgagtccctccggtcaccc
1-14tRNA-Ala(tgc)[94388,94462]
gggcctgtagctccaattggtagagcagcatccttgcaagatgacggctg
tcggttcgaatccgacctggtccac
1-15tRNA-Phe(gaa)[94652,94724]
gccgtcatagctcagttggtagagcactggcctgaaaacccagtggccga
ggttcgattcctcgtgtcggcac

1-16tRNA-Val(cac)[94730,94803]
gtccgtttagctcagctggaagagcgctcggtccacacccgagaggccgc
aggttcgatccctgcaatggacac
1-17tRNA-Lys(ctt)[94923,94996]
gccttcgtagctcagttggtagagctctcgcctcttaagcgagatgtcgc
aggttcgacccctgccggaggcac
1-18tRNA-Glu(ttc)[95001,95077]
ggtcgggtcggtctgctggtatggccagtcggattttcactccggacatt
cgcgggttcaattcccgtcccgatcgc
1-19tRNA-Gly(tcc)[95157,95229]
gcgggtgtggccgaatggctcaggcaccagatttccactctggctacgca
ggttcgattcctgtcatccgctc
1-20tRNA-Gly(tcc)[95329,95401]
gcgggtgtggccgaatggctcaggcaccagatttccactctggctacgca
ggttcgattcctgtcatccgctc
1-21tRNA-Thr(cgt)[95461,95535]
gctgctgtagctcacctggcagagcgtcggcgtcgtatcccgaaggcatc
cggttcgagtccggacagcagcccc
1-22tRNA-Thr(tgt)[95536,95608]
gcctctgtggtccagcggcacggacatccgccttgtaagcggaggacccc
cgttcgatccggggtagaggctc
1-23tRNA-Thr(ggt)[95668,95740]
gctgggttagctcagtggtagagcgttcctctggtatgggaaaggcccgg
ggttcaatcccccgactcagctc
1-24tRNA-Gly(gcc)[97485,97560]
gcggttatagctcagcctggccagagcatcacgttgccaacgtgaacgtc
gcgggttcaaatcccgctagccgctc
1-25tRNA-Asp(gtc)[97574,97646]
ggccctgtagctcagaggaagagcgccggtctgtcgaatcggaggtcgcg
gtatcgtaatccgtcagggtcgc
1-26tRNA-Met(cat)[97706,97778]
gcctcactagctcattggtagagccgctcgctcataacgtgcaggtacct
ggttcgattccagggtgaggtac
1-27tRNA-Ile(gat)[97784,97858]
gcctgttagcggactggtcgtccgatccaagctgataacttggcgtaagc
ggtgttcgattcaccgagcaggtac
1-28tRNA-Arg(acg)[97952,98024]
gcctctatggtccaacggatatgacgccggtctacggaaccggagatgcg
tgttcgattcgcgctaggggcac
1-29tRNA-Val(gac)[98067,98139]
gtccgtgtagctcagtggtagagcgcctgctcgacacgcaggaggaccga
ggttcgaaacctcgcatggacac
1-30tRNA-Arg(cct)[98313,98386]
gcctctgtagctcaacggacagagcaacgcggtcctaacgcggtggctgg

aggttcgaatcctctcggaggcac
1−32tRNA−Gln(ttg)[98927,99002]
tggggtatggtggcaatctggcagtcccgccggactttgactccggaggt
gcaggttcgagtcctgctaccccatc
1−33tRNA−Arg(tct)[99006,99081]
gcccttgtagctcagtggacagagcggcgagcttctacctcgcgggccgg
gagttcgaatctctccaggggcacca
1−34tRNA−Gln(ctg)[125659,125733]
tgctcgttggtgtaactggcaacactacggactctgactccgtcattctt
ggttcgaatccagggcgagcaacca
1−35tRNA−Asn(gtt)[125740,125815]
tggggtgtccgttaatcaggcaaacgagcggactgttaatccgtccctgc
aggttcgaatcctgccaccccagcca
>FJ230960.1 Bacillus phage SPO1, complete genome
1−1tRNA−Asn(gtt)[28208,28282]
tcctctatagctcaatggtagagcacatgactgttaatcatggggttgta
ggttcgaatcctactggaggagcca
1−2tRNA−Ser(gct)[28368,28457]
tggaatgttacccaagtggttaaggggtctggttgctaaccagatagcgg
gctttatgtccggcataggttcgaatcctatacattccgt
1−3tRNA−Arg(cct)[32596,32670]
tgcccatgtagctcagtggatagagcacgtccctcctaaggacggtgtcg
ggagttcaaatctctccatgggcgt
>EU710883.1 Erwinia phage phiEa21−4, complete genome
1−2tRNA−Pro(tgg)[22037,22114]
ctccgtttagctcagtatggatagagtaggccgtttggggcggtcgggtc
gaaggttcgaatccttcaacggagacca
1−3tRNA−Ala(tgc)[22235,22307]
gggtcagtagcttaattgggaaagcatctcacttgcaatgagaaggatga
gggttcaagtccctcctgtatcc
1−4tRNA−Met(cat)[22490,22564]
agcgggatggagcagtggtagcttgccagtttcattatctggaggtcgta
ggttcgaatcctactcccgcttcca
1−5tRNA−Asn(gtt)[22573,22648]
gggtttgaagcacatatggatgtgcggctggctgttaaccagatggtagt
gggttcgagtcccacctttcccgcca
1−6tRNA−Tyr(gta)[22655,22737]
gcgtctatggcagagatggtcaatgcagcggtctgtaaaaccgccccgaa
aggttactggttcgagtccagttaggcgcacca
1−7tRNA−Met(cat)[22744,22817]
tgcgaagtagtgaaatggcatcatactggtttcattatccggtgtaggta
gttcgaatctacccttcgcatcca
1−8tRNA−Asp(gtc)[22897,22973]
ggggatgtggcagacttggtaattgtaccgcactgtctatgcggaatatg

agggttcaaatcccttcatcctcgcca
1–9tRNA–Ser(tga)[23201,23291]
ggaagggcaaatcgactggcgacgaaaacggtcttgaaaaccgccgagcg
ttaatagcgccttgagggttcgactccctctccttccgcca
1–10tRNA–Phe(gaa)[23293,23367]
ggtctaattagcttatatggtaaagcaacggtctgaaaagccgtggaaca
gggttcgaatcccgatagaccgcca
1–11tRNA–Lys(ttt)[23375,23450]
cggtgtgtaactcaattggcagagtgggtggcttttaaccacagggttgc
aggttcgatgcctgtcacaccgacca
1–12tRNA–Lys(ctt)[23458,23534]
ggactattaactcaactggtcagagtacccgactcttaatcgggaggttc
agggttcgactccctgatagtctacca
1–13tRNA–Met(cat)[23546,23619]
agtggtatggcagagatggtcatgcacttccttcatacggaagattacat
cggttcaagtccgattaccacttc
1–14tRNA–Ile(gat)[23633,23707]
tgtgggttagcataaatggtaatgcagacggctgataaccgtcagaagag
ggttcgataccctcacctacgacca
1–15tRNA–Gln(ttg)[23714,23789]
cggggtatggagtaattggcaactctacggtttttgaggccgtcagtttt
cggttcgaatccgaatgcccccgcca
1–16tRNA–Arg(tct)[23796,23872]
gcacccttagaacaactggacagttccgctgtcttctaaacagctagtta
caggttcgagtcctgtagggtgtgcca
1–17tRNA–Leu(caa)[24290,24366]
ggcgtaatatcccaattggcagaggaagcaggttcaaaccctgttcagtg
tgggttcgactcccacttacgctacca
1–18tRNA–Gly(tcc)[24496,24571]
gcaagtatgatgtcaactggtaacatggcgtccttccaagtcgctcttac
gggttcgaatcccgttacttgctcca
1–19tRNA–Trp(cca)[24577,24652]
taggggattagtttaactggcaaaatgtcggcctccaaaaccgcacgttg
agggttcgaatccctcatcccctgtc
1–20tRNA–Thr(tgt)[24663,24737]
gcctccatcatataagggctattatgcctgttttgtaatcaggtcatcgc
ggttcgaatccgtgtgggggcacca
1–21tRNA–Val(tac)[24745,24820]
actcgtatagctcagatggtagagcggcatctttacacgttgctggtcag
gcgttcgagtcgccttgcgagtacca
1–22tRNA–Leu(aag)[24827,24904]
ggctgtgtagcccaacttggcagaggcactacgctaagaacgtatacagt
gagagttcgaatctctccacagctacca
1–23tRNA–Arg(acg)[24911,24987]

agccctttatcttaaatggatagagactcaagctacgaacttgagaggtt
tgggttcgaatcccaaaagggcttcca
1−24tRNA−Gln(ctg)[24990,25066]
aggatgttcgtatagttggcctattacaccggactctgaatccggttacg
atggttcgaatccatcacgtcctgcca
1−25tRNA−Leu(taa)[25073,25149]
aggtgattaccccaattggcagagggtaggagcttaaacctccgatgttg
agagttcgaatctctcatcacctacca
1−26tRNA−His(gtg)[25153,25229]
gagttggtatcataaatggataatgaccctgactgtgaatcaggtctatg
cgggttcgaatcccgtccttctcccca
1−27tRNA−Ser(gct)[25236,25328]
ggaagattaaccctaaaaggtaagggaagtgtttgctaaacactcagtaa
ccgagaaatcggcgtaccagttcaagtctggtatcttcctcca
>EU863409.1 Enterobacteria phage JS10, complete sequence
1−1tRNA−Arg(tct)c[68440,68515]
cgaggcatagctcagaaggaagagcaacggtcttctaaaccgtaggtcgt
aggttcgatccctactgtctcgacca
1−2tRNA−Asn(gtt)c[68520,68594]
ggatgtgtagctcagtggtagagcagttgactgttaatcaattggtccat
ggttcgaatccatgcatgtccgcca
1−3tRNA−Met(cat)c[68599,68672]
ggccctgtagctggacggtcaagcgagcgactcataatcgctggatggtg
gttcgattccacccagggccacca
>EU826469.1 Mycobacterium phage ScottMcG, complete genome
1−1tRNA−Ser(gct)[30892,30975]
ggagggtgagcatctggtgatgcaggggtcctgctaaggccctacggatt
cacacccgtgagtttcgattactcctccctccgc
1−2tRNA−Leu(cag)[31072,31148]
gccctgctgagcaaactggcaaagctgccgcattcagagtgcgggtcatt
tccgggttcgactcccgggcagggtac
1−3tRNA−Leu(gag)[31268,31342]
gtctctgtaggcaaatcggaaaagccgccatcttgagggggtggtgcgtg
cgggttcgactcccgccagagacac
1−4tRNA−Leu(caa)[31343,31416]
gccgtggtaggccatctggcgagccgccgagttcaagtttcggtgtttgc
gggttcgaatcccgcccacggtac
1−5tRNA−Pro(tgg)[90789,90863]
cggggtgtagttcagtttggaagagcgcttggtttgggaccaagttgtcg
caggttcgaatcctgtcaccccgac
1−6tRNA−Trp(cca)[90878,90948]
gggtctgtgcacagggtgcccgacggtctccaaagccgaaggcgggggtt
cgattccctccaggcctgcca
1−7tRNA−Pyl(cta)[92139,92211]

gcaccatttgctcaatggcagagcggcggcttctaaaaccgtgagtgccg
gttcgactccggcatggtgcacc
1-8tRNA-Met(cat)[92361,92435]
agcggtgtagagcagctaggtagctcgccgggctcataacccggaggacg
cgtgttcgaatcacgccaccgccac
1-9tRNA-Cys(gca)[92562,92633]
gcgcctttggcggaatggctacgtgctcggctgcaacccgagttatcccg
gttcgactccgggagggcgctc
1-10tRNA-Glu(ctc)[92638,92712]
ggtccgttggagtagatggatatctcgccaccctctcaaggtggagatca
cgggttcaagtcccgtacggactgc
1-11tRNA-His(gtg)[92714,92787]
gtggccgtagttcagccggtagaacgctgggttgtgatcccagtcgtcga
gggttcgagtccctccggtcaccc
1-12tRNA-Ala(tgc)[92949,93023]
gggcctgtagctccaattggtagagcagcatccttgcaagatgacggctg
tcggttcgaatccgacctggtccac
1-13tRNA-Phe(gaa)[93213,93285]
gccgtcatagctcagttggtagagcactggcctgaaaacccagtggccga
ggttcgattcctcgtgtcggcac
1-14tRNA-Val(cac)[93291,93364]
gtccgtttagctcagctggaagagcgctcggtccacacccgagaggccgc
aggttcgatccctgcaatggacac
1-15tRNA-Lys(ctt)[93483,93555]
gccttcgtagctcagtggtagagctgtcgcctcttaagcgataggtcgtt
ggttcgaatccagccgggggcac
1-16tRNA-Glu(ttc)[93560,93636]
ggtcgggtcggtctgctggtatggccagtcggattttcactccggacatt
cgcgggttcaattcccgtcccgatcgc
1-17tRNA-Gly(tcc)[93716,93788]
gcgggtgtggccgaatggctcaggcaccagatttccactctggctacgca
ggttcgattcctgtcatccgctc
1-18tRNA-Thr(cgt)[93848,93922]
gctgctgtagctcacctggcagagcgtcggcgtcgtatcccgaaggcatc
cggttcgagtccggacagcagcccc
1-19tRNA-Thr(tgt)[93923,93995]
gcctctgtggtccagcggcacggacatccgccttgtaagcggaggacccc
cgttcgatccggggtagaggctc
1-20tRNA-Thr(ggt)[94055,94127]
gctgggttagctcagtggtagagcgttcctctggtatgggaaaggcccgg
ggttcaatcccccgactcagctc
1-21tRNA-Gly(gcc)[95450,95523]
gcgaaggtagctcagctggcagagcgccaccttgccaaggtggaggtcgc
gggatcgcaacccgttcttcgctc

1−22tRNA−Asp(gtc)[95527,95599]
ggccctgtagctcagaggaagagcgccggtctgtcgaatcggaggtcgcg
gtatcgtaatccgtcagggtcgc
1−23tRNA−Met(cat)[95659,95731]
gcctcactagctcattggtagagccgctcgctcataacgtgcaggtacct
ggttcgattccagggtgaggtac
1−24tRNA−Ile(gat)[95737,95811]
gcctgttagcggactggtcgtccgatccaagctgataacttggcgtaagc
ggtgttcgattcaccgagcaggtac
1−25tRNA−Arg(acg)[95905,95977]
gcctctatggtccaacggatatgacgccggtctacggaaccggagatgcg
tgttcgattcgcgctaggggcac
1−26tRNA−Val(gac)[96020,96092]
gtccgtgtagctcaggggtagagcgcctgctcgacacgcaggaggaccga
ggttcgaaacctcgcatggacac
1−27tRNA−Arg(cct)[96266,96339]
gcctctgtagctcaacggacagagcaacgcggtcctaacgcggtggctgg
aggttcgaatcctctcggaggcac
1−29tRNA−Gln(ttg)[96880,96955]
tggggtatggtggcaatctggcagtcccgccggactttgactccggaggt
gcaggttcgagtcctgctaccccatc
1−30tRNA−Arg(tct)[96959,97034]
gcccttgtagctcagtggacagagcggcgagcttctacctcgcgggccgg
gagttcgaatctctccaggggcacca
1−31tRNA−Gln(ctg)[124908,124982]
tgctcgttggtgtaactggcaacactacggactctgactccgtcatttta
ggttcgaatcctaagcgagcagcca
1−32tRNA−Asn(gtt)[124989,125064]
tggggtgtccgttaatcaggcaaacgagcggactgttaatccgcccctgc
aggttcgaatcctgccaccccagcca
>FJ822135.1 Lactobacillus phage Lb338−1, complete genome
1−2tRNA−Arg(cct)c[23807,23880]
tgtttccatagaacaatggatagtttaccggtttcctaaaccgttaattc
cggttcgagtccggatggaaacat
1−3tRNA−Arg(tct)c[24706,24779]
gttcccatagttcaattggatagtaacatctgccctctaagcagagactc
ccagttcgagtctgggtgggaaca
1−4tRNA−Ile(tat)c[25603,25691]
tagtcctgtagcactatggcagtgcaccgggtttataacccgtactcctg
ataagagggttaagtgggtccgattcccaccgggactat
1−5tRNA−Thr(tgt)c[27758,27832]
tgcttccttagctcatatggtagagcatctgttttgtaatcagaaggtga
ctggttcgatcccagtaggaagcat
>FJ839692.1 Enterobacteria phage RB14, complete genome

1–1tRNA–Arg(tct)c[68093,68168]
cgaggcatagctcagaaggaagagcaaggaccttctaagtcctgggtcgt
aggttcgatccctactgcctcgacca
1–2tRNA–Asn(gtt)c[68173,68247]
ggatgtgtagctcaatggcagagcgatcgcctgttaagcgattggttata
ggttcgaatcctatcacgtccgcca
1–3tRNA–Tyr(gta)c[68252,68338]
ggggagttatcccgtagaggtagcggtgtggactgtaaattcattgtcat
tgcgactcgggtggttcgactccatcactccccacca
1–4tRNA–Met(cat)c[68348,68422]
ggccctgtagctggaaggttcaagcaagcgactcataatcgccagatggt
ggttcaattccacccagggccacca
1–5tRNA–Thr(tgt)c[68424,68499]
gctgatttagctcagtaggtagagcaactcacttgtaatgagaaggtcgg
cggttcgattccgtcaatcagcacca
1–6tRNA–Ser(tga)c[68505,68594]
ggaggcgtggcagagtggtttaatgcaccggtcttgaaaaccggcagtcg
ctccggcgactcataggttcaaatcctatcgcctccgcca
1–7tRNA–Pro(tgg)c[68596,68670]
ctccgtgtagctcagtttggtagagcgcctgatttgggatcaggaggtcc
aaggttcaaatccttgtatggagac
1–8tRNA–Gly(tcc)c[68681,68754]
gcggatatcgtataatggcattacctcagacttccaatctgatgatgtga
gttcgattctcattatccgctcca
1–9tRNA–Leu(taa)c[68760,68846]
gcgagaatggtcaaattggtaaaggcacagcacttaaaatgctgcggaat
gatttccttgtgggttcgagtcccacttctcgcacca
1–10tRNA–Gln(ttg)c[68847,68920]
tgggaattagccaagttggtaaggcactggattttgattccaggatgcaa
aggttcgagtcctttattcccagc
>GQ303260.1 Mycobacterium phage ET08, complete genome
1–1tRNA–Ser(gct)[29156,29239]
ggagggtgagcatcaggtgatgcagcgagattgctaatcccgtacggtaa
ccaccccgtgaggttcgaatcctcctccctccgc
1–2tRNA–Leu(cag)[29432,29507]
gctcccgtagcccaattggcaggaggcaccagattcaggatctgggcagt
gtgagttcgaatctcaccgggagtac
1–3tRNA–Leu(gag)[29627,29701]
gtctctgtaggcaaatcggaaaagccgccatcttgagggggtggtgcgtg
cgggttcgactcccgccagagacac
1–4tRNA–Leu(caa)[29702,29775]
gccgtggtaggccatctggcgagccgccgagttcaagtttcggtgtttgc
gggttcgaatcccgcccacggtac
1–5tRNA–SeC(tca)[67162,67258]

attctggcactggtgggcgagcccaccggcggcagcttcaagctgtcgct
ggccggagagaatcgaccggaaacatcccgttcaacgcgacccagggcc
1-6tRNA-Pro(tgg)[90457,90528]
cggggtgtagtgaaaaggcatcatgctggttttgggtaccagtggttccg
gttcgagtccgggctccccgac
1-7tRNA-Trp(cca)[90967,91042]
aggggtagagccagctgggacggcagcggattccaaacccgccattgacg
gagttcgattctccgtacccctgcca
1-8tRNA-Tyr(gta)[91044,91129]
ccgggtaagccatgttggttatggcaggcagactgtaaatctgtcgtctt
cggacttcgggggttcgattccctctacccggacca
1-9tRNA-Pyl(cta)[92188,92260]
gcaccatttgctcaatggcagagcggcggcttctaaaaccgtgagtgccg
gttcgactccggcatggtgcacc
1-10tRNA-Met(cat)[92410,92484]
agcggtgtagagcagctaggtagctcgccgggctcatgacccggaggacg
cgtgttcgattcacgccaccgccac
1-11tRNA-Cys(gca)[92611,92682]
gcgcctttggcggaatggctacgtgctcggctgcaacccgagttatcccg
gttcgactccgggagggcgctc
1-12tRNA-Glu(ctc)[92687,92761]
ggtccgttggagtagatggatatctcgccaccctctcaaggtggagatca
cgggttcaagtcccgtacggactgc
1-13tRNA-His(gtg)[92763,92836]
gtggccgtagttcagccggtagaacgctgggttgtgatcccagtcgtcga
gggttcgagtccctccggtcaccc
1-14tRNA-Ala(tgc)[92999,93072]
gggcctatagctcatctggtagagcgcctgccttgcaagcaggaggcggc
aggttcaagtcctgttaggtccac
1-15tRNA-Phe(gaa)[93262,93334]
gccgtcatagctcagttggtagagcaccggcctgaaaacccggtggccga
ggttcgagtcctcgtgtcggcac
1-16tRNA-Val(cac)[93340,93413]
gtccgtttagctcagctggaagagcgctcggcccacacccgagaggccgc
aggttcgatccctgcaatggacac
1-17tRNA-Lys(ctt)[93532,93604]
gccttcgtagctcagtggtagagctgtcgcctcttaagcgataggtcgtt
ggttcaaatccagccgggggcac
1-18tRNA-Glu(ttc)[93608,93683]
ggtcgggtggtctgttggcaggccggtcgggttttcaccccggcgtcatc
gcgggttcgattcccgtcccgactgc
1-19tRNA-Gly(tcc)[93813,93885]
gcgggtgtggccgaatggctcaggcaccagacttccactctggctacgca
ggttcgattcctgtcatccgctc

1-20tRNA-Thr(cgt)[93945,94018]
gctgctgtagctcacctggcagagcgtcggcgtcgtatcccgaaggcatc
cggttcgagtccggacagcagctc
1-21tRNA-Thr(tgt)[94027,94100]
gcccttgtagctcagtggcagagcaccgaccttgtaagtcgggttgtccc
gggttcgattcctggtaggggctc
1-22tRNA-Thr(ggt)[94151,94223]
gctgatttagctcagtggtagagcgccgacttggtatgtcggaggccccg
ggttcgatccccggattcagctc
1-23tRNA-Gly(gcc)[95968,96043]
gcggttatagctcagcctggccagagcatcacgttgccaacgtgaacgtc
gcgggttcaaatcccgctagccgctc
1-24tRNA-Asp(gtc)[96057,96129]
ggccctgtagctcagaggaagagcgccggtctgtcgaatcggaggtcgcg
gtatcgtaatccgtcagggtcgc
1-25tRNA-Met(cat)[96189,96261]
gcctcactagctcattggtagagccgctcgctcataacgtgcaggtacct
ggttcgattccagggtgaggtac
1-26tRNA-Ile(gat)[96267,96341]
gcctgttagcggactggtcgtccgatccaagctgataacttggcgtaagc
ggtgttcgattcaccgagcaggtac
1-27tRNA-Arg(acg)[96435,96507]
gcctctatggtccaacggatatgacgccggtctacggaaccggagatgcg
tgttcgattcgcgctaggggcac
1-28tRNA-Val(gac)[96550,96622]
gtccgtgtagctcaggggtagagcgcctgctcgacacgcaggaggaccga
ggttcgaaacctcgcatggacac
1-29tRNA-Arg(cct)[96796,96869]
gcctctgtagctcaacggacagagcaacgcggtcctaacgcggtggctgg
aggttcgaatcctctcggaggcac
1-31tRNA-Gln(ttg)[97410,97485]
tggggtatggtggcaatctggcagtcccgccggactttgactccggaggt
gcaggttcgagtcctgctaccccatc
1-32tRNA-Arg(tct)[97489,97564]
gcccttgtagctcagtggacagagcggcgagcttctacctcgcgggccgg
gagttcgaatctctccaggggcacca
1-33tRNA-Gln(ctg)[125485,125559]
tgctcgttggtgtaactggcaacactacggactctggctccgtcattctt
ggttcgaatccagggcgagcaacca
1-34tRNA-Asn(gtt)[125566,125639]
tggggtgtagttcaactggaagaacgctcgactgttaatcgagtagttga
aggttcgagtccttccactccagc
>EU877232.1 Enterobacteria phage WV8, complete sequence
1-1tRNA-Pro(tgg)[25986,26062]

ctctgtttagctcagcttggtagagcgttccgtttggggcggtaaggccg
gaggttcaagtcctccaacagagacca
1-2tRNA-Glu(ttc)[26070,26147]
gttccagtagacaaaatggtaaagtcaccactctttcaaagtggatatct
gagggttcaaatcccttctggaacgcca
1-3tRNA-Met(cat)[26238,26314]
tgcgggtatagagaaagggcgtctcacatgtctcattagcatggtatcgg
caggttcgactcctgcacccgcctcca
1-4tRNA-Asn(gtt)[26398,26475]
ggtt.aggaagcacataaggtatgtgcggtcgcctgttaagcgaatggca
cagggttcgaatccctgactaaccgcca
1-5tRNA-Tyr(gta)[26485,26572]
gtgtcgttatcccgtagatggtagcggtggggactgtaaatcccttgtca
ttgagactcggtaggttcgactcctacacggcacacca
1-6tRNA-Asp(gtc)[26578,26655]
ggttatgtagtttaatctggttaaaatactcccctgtcacgggagatgat
gagggttcgaatcccttcgtaaccgcca
1-7tRNA-Lys(ttt)[27085,27160]
ggaagtgtagcagaatggtgatgcggcagacttttaatctgacaggcgat
gggttcgaatccctccacttctacca
1-8tRNA-Met(cat)[27165,27241]
ggttcagtcgcagataaggtaatgcaagggtctcataagccctatgaatg
tgggttcgattcccatctgaacctcca
1-9tRNA-Ile(gat)[27243,27318]
gctggtatagttaagaaggttataacactcccctgataagggaacatcgg
tggttcgattccacctaccagtacca
1-10tRNA-Trp(cca)[27798,27868]
aggggtgagctatggcagcaatagagtctccaaaattcttgattagagtt
cgaatctctacacccctgcca
1-11tRNA-Ser(tga)[28301,28390]
ggtaggtagcggctaatggcagccaaacagtcttgaaaactgttgccact
gtagagatacggtgagggttcgactcctttacttaccgcc
1-12tRNA-Leu(tag)[28645,28721]
gcacctatagcccaactggtagaggcagcagacttagaatctgctcagtg
tgagttcgaatctctctaggtgtacca
1-13tRNA-Lys(ctt)[28729,28804]
gcaggtgtagcaaaatggttatgcggctgactcttaatcagtaagacgat
gggttcaattccctccacctgtacca
1-14tRNA-Ala(tgc)[28811,28886]
ggggtcatagtttatatggttaaaattcgagttttgcaaacttgggaact
gagttcaattctcagtgactccacca
1-15tRNA-Gly(tcc)[28893,28967]
gcatccatagtttaaacgggaaaattacagtcttccaaactgaggttgag
ggttcgattccctctggatgctcca

1-16tRNA-Thr(tgt)[28975,29050]
gctcctatcgtataattggttattacggttgccttgtaagcaacttatca
gggttcgagtccttgtgggagcacca
1-17tRNA-Val(tac)[29147,29221]
actcgcttagtttatatggtaaaacatcacccttacaagatgaagaaaaa
ggttcaagtcctttagtgagtacca
1-18tRNA-Leu(caa)[29223,29300]
gttccagtatcccaattggcagaggatgcaagctcaaaccttgtattagt
gacggttcgaatccgtcttggaacacca
1-19tRNA-Arg(acg)[29306,29381]
gcaggattagttcaaatggaaagagcaacagtctacgaagctgttaatag
gggttcgaatcccttatcctgcgcca
1-20tRNA-Gln(ttg)[30204,30279]
aggggattagtttacaaggttaaaacctcggtctttgaaatcgaagaagt
tggttcaattccaacatcccccgcca
1-21tRNA-Leu(taa)[30282,30360]
gctccattactccaattggcagagaggccagacttaaaatctgtgttatg
tatcggttcgaatccgatatggagtacca
1-22tRNA-Gln(ctg)[30366,30441]
agcggtatagcataactggcaatgcagcagtctctgaagctgtcctatta
aggttcaaatccttatgccgctgcca
1-23tRNA-His(gtg)[30473,30548]
gtggccttatcataaatggtaatgacccatgctgtgaacatggtctatac
gggttcaaatcccgtaggtcacccca
1-24tRNA-Phe(gaa)[30555,30630]
agtccaagtagcttatatggttaaagcgcgtgtctgaaaaacatgagaag
agggttcaaatcccactggactacca
1-25tRNA-Ser(gct)[31384,31476]
ggaagattaaccctaaaaggtaagggagcagtttgctaaactgccagtag
ctgagaaatcggtgtaccagttcaagtctggtatcttcctcca
1-26tRNA-Cys(gca)[31481,31556]
gaatccgtgacagaaacggctatgtgcctgtctgcaaaacaggtttataa
gggttcgagtcccttcggattctcca
>FM207411.1 Synechococcus phage S-RSM4 complete genome
1-1tRNA-Gly(tcc)c[29459,29530]
gcggagttagttcagtggtagaacgctatccttccaagttagatgtcgtc
ggttcgaatccgatactccgct
1-2tRNA-Pro(tgg)c[31213,31288]
tcggggtgtagctcagcttggtagagcgccgtctttgggaggcggatgcc
gtaggttcgaatcctatcaccccgat
1-3tRNA-Ser(tga)c[31291,31376]
ggagaggtggtcgagtggttgaaggctctagtcttgaaaactagcgaggt
gaaagcctccgtgggttcgaatcccaccctctccgc
1-4tRNA-Val(tac)c[31852,31923]

gggcgattagcgcagcggtagcgcagttgctttacacgcaattggtcggg
ggttcgaatccctcatcgccca
1–5tRNA–Leu(tag)c[31926,32008]
gtcggtatggcggaattggtagacgcgctgggtttaggttccagtgtcct
tgcgacgtggaggttcaagtcctcttaccgaca
1–6tRNA–Arg(tct)c[32009,32085]
gactcaatagctcagctggatagagcaactgccttctaagcagtcggtcg
taggttcgaatcctacttgagtcgcca
1–7tRNA–Tyr(gta)c[176122,176203]
gggtaggtgtccgagtggttaatggaggcggactgtaaatccgctggctc
tgcctacgggggttcaaatccctccctgccca
1–8tRNA–Asn(gtt)c[176206,176279]
ttggcgtgtagctcaatggcagagcagggagctgttaactctctggttgc
aagttcgagtcttgccacgccagt
1–9tRNA–Ala(tgc)c[176306,176378]
ggggaattagctcagttggtagagcacctgctttgcaagcaggctgtcag
gagttcgagtctcctattctcca
1–10tRNA–Thr(tgt)c[176379,176455]
gccactatagctcagctggatagagcaacggttttgtaaaccgtaggtcg
tcggttcaagtccgacttgtggctcca
1–11tRNA–Asp(gtc)c[176458,176533]
tggggttgtagctcagtcggttagagcgcctgcctgtcacgcaggaagtc
gagggttcaagtcccttcagtcccgt
1–12tRNA–Leu(taa)c[177356,177440]
gggacggtggtggaattggtagacacaccagacttaaaatctgttgattg
taaagatcgtgcgggttcaagtcccgctcgtccta
>GU295964.1 Klebsiella phage KP15, complete genome
1–1tRNA–Met(cat)[49401,49477]
ggccccttagctcaataggtagagctaatcactcataatggttaggttcc
cggttcaagtcacgggaggggccacca
>HM452126.1 Aeromonas phage phiAS5, complete genome
1–1tRNA–Ala(cgc)c[181347,181421]
acgcgattagctcagtgggagagcgcctctgatcgcagagaagtatcggg
accggttcgaatccggtatagtgtg
1–2tRNA–Met(cat)c[189405,189479]
ggccccgtaacataagggtaaatgtgctcgcctcataagcgaagaaaagc
ggttcgagtccgctcggggccacca
1–3tRNA–Leu(tag)c[189963,190046]
gcggatgtggcgaaattggcagacgcactagatttaggttctagcgccat
aggcgtgtcggttcgagtccgaccatccgcacca
1–4tRNA–Ser(gct)c[190053,190140]
ggagaaatgacagagaggccgaacgtagcggattgctaattcgtaggccc
gcaagggtccgtgggttcgaatcccactttctcctcca
1–5tRNA–Ser(tga)c[190949,191042]

ggaaggtcaacccgatagccggcgacgggagcggtcttgaaaaccgtcga
gggttaacagccccttgtgagttcgaccctcacaccttccgcca
1−6tRNA−Gln(ttg)c[191317,191392]
aggggtatcgccaagttggaaaggcaacggattttgattccgtcattcgc
tggttcgagtccagctacccccgcca
1−7tRNA−Pro(tgg)c[191652,191726]
ctcgggttaggcaagtggtatgtcgcctggtttgggaccaggtcgtcgag
tgttcgattcactcacccgagacca
1−8tRNA−Lys(ttt)c[191862,191937]
gcgccgttagcttagttggtagagcatcggacttttaatccgagggtcga
tggttcgagtccatcacggcgcacca
1−9tRNA−Trp(cca)c[191944,192017]
aggggcgtagcaaatcggttatgcggcggattccaaatccgttgaggtgg
gttcgactcctaccgcccctgcca
1−10tRNA−Cys(gca)c[192845,192919]
gcgcaggtagccaagcggtaaggcaagtgattgcaaatcactttatcgtc
tgttcgatccagatcctgtgctcca
1−11tRNA−Gly(tcc)c[192927,193000]
gccttcttagtttaatggtaaaaccactgccttccaagcagacgtcgtgg
gttcgattcccacaggaggctcca
1−12tRNA−Ala(tgc)c[193012,193087]
ggggatatagctcagttgggagagcactagccttgcaagctgggggtcgt
gggttcgagtcccattgtctccacca
1−13tRNA−Asp(gtc)c[193373,193447]
ggtgcattagtataatggctattattcgtgactgtctatcacaggatacg
ggttcgattcccgtatgtaccgcca
1−14tRNA−Tyr(gta)c[193571,193662]
ggagacgctgcaatgacggtggtattgcaccagactgtaaatctggcccc
acagggtgaacattgtaggttcgaatcctaccggctccacca
1−15tRNA−Met(cat)c[193686,193760]
agcgggatggagaagtctggaatctcgttagcctcataagctaaaggtcg
gtggttcaaatccacctcccgcttc
1−16tRNA−Glu(ttc)c[193781,193877]
tctccgttcatctaacggctaggatatcgccctttcacggctaggatatc
gccctttcacggcgagtacacgggttcgaatcccgtacggagaacca
1−17tRNA−Met(cat)c[194416,194490]
ggtgcattagctcagtggtagagctgcggtttcatacgccgtcggtcggt
agttcaaatctaccatgcaccacca
1−18tRNA−Phe(gaa)c[194703,194778]
ggacctgtagctcagatggtagagcaccggattgaaaatccgggtgtcgt
cagttcgatcctgaccgggcccacca
1−19tRNA−His(gtg)c[194784,194859]
gtgacggtagttcagttggtagaactccaggttgtgatcctggtagtcac
gggttcgaaacccgtccgtcacccca

1-20tRNA-Thr(tgt)c[194940,195013]
gctcttatagttcagtggcagaacaagtgatttgtaaccattagacctca
gttcgattctgagttggagcacca
1-21tRNA-Ile(gat)c[195020,195094]
agtcccgtagctcagaggtagagcgccgtgctgataacgcggatgtggat
ggttcgataccattcgggactacca
1-22tRNA-Asn(gtt)c[195770,195852]
ggaggtgtggctcaaaggttgagcggcagactgttaatctgcgggtaata
accctatgttggttcgagtccaacctcctccgc
1-23tRNA-Leu(taa)c[199140,199223]
gtgggtatggcgtaattggtagccgcaggagacttaaaatctcccgcttc
tggcgtgtgggttcgagtcccactacccgcacca
1-24tRNA-Leu(caa)c[199229,199313]
gggagattggcgtaattggcagccgcagtagattcaaaatctaccgacga
aagtcgtaccggttcgagtccggtatctcctacca
1-25tRNA-Arg(tct)c[199406,199480]
cggggcgtagctcagttggatagagcatgtgccttctaagcacacggtcg
ctggttcgagcccagtcgcctcggc
>GU911519.1 Acinetobacter phage Acj61, complete genome
1-1tRNA-Trp(cca)c[79124,79194]
aggtcgctagtgtaatggtaccacgacgggctccaaccccgttaatcttc
gttcgaatcgaaggcgattct
1-2tRNA-Phe(gaa)c[79201,79276]
gcacgagtaactcagttggtagagtggctgcctgaagagcagcaggtcac
atgttcgaatcatgtctcgtgcacca
1-3tRNA-Met(cat)c[79283,79356]
cgcgatatggtgtaaaggttgcacatagggctcataacccaaaggttcca
gttcgagtctggatatcgctacca
1-4tRNA-Gln(ttg)c[79363,79436]
tgtcgcatggtgtaatggtagcaccacagaatttgactctgttagtaaag
gttcgatcccttttgcgacttcca
1-5tRNA-Pro(tgg)c[79527,79603]
ctctctatagggcagtctggtagcctacttcttttggaaagaagatgtcg
ttggttcaaatccaactagggagacca
1-6tRNA-Ile(gat)c[79660,79735]
cgtcgagtagctcagttggtagagcaacggaccgataatccgcaggtcac
atgttcgaatcatgtctcgacgacca
1-7tRNA-Ser(tga)c[79736,79830]
ggtgaagtggcagagtctggcttattgcagtagtcttgaaaactaccgtc
tcatagtgatatggggccctgggttcaaatcccagtttcacctcc
1-8tRNA-Asn(gtt)c[80035,80110]
tcctcagtagctcagttggtagagcagcagactgttaatctgtgggtcac
acgttcgatccgtgtctggggagcca
1-9tRNA-His(gtg)c[80144,80218]

tggaccatggtgtaatggttagcaccgcgatttgtgatgtcgttagtctc
agttcgaatctgtgtggtccaacca
1-10tRNA-Glu(ttc)c[80227,80302]
gctcttatcgtctagtcggttaggacgtcagcctttcacgttgaaaactc
gggttcaagtcccgataagagtacca
1-11tRNA-Tyr(gta)c[80309,80394]
ggggacgtaggaaaattggtaaccccatcggactgtaaatccgacgccaa
aagcatctgtgagttcaagtctcaccggccccacca
1-12tRNA-Thr(tgt)c[80410,80481]
tctggcatcatatagtggtattatgctggttttgtactccagtcaccgag
tttcgattactcgtgccagaac
1-13tRNA-Cys(gca)c[80595,80670]
ggcgttgtggtagaatggttatacggcggattgcaaatccgcgtcgatgc
aggttcgagtcctgttgacgcctcca
1-14tRNA-Arg(tct)c[80679,80755]
gtctcgctggccgagttggattaggcatcgtagttctattgcgacttata
tgagttcgaatctcatgcggggctcca
>FJ373894.1 Shigella phage phiSboM-AG3, complete genome
1-1tRNA-Ser(tga)[137688,137767]
tggaggatgatggaaggggtagacattccctgaggggatggcggcagccg
ttgaaggttcgaatccttctcctccaacca
1-2tRNA-Asn(gtt)[138197,138272]
gacgatgtagttcagtcggtagaacggcggtctgttaaaccgtatgtcgc
aggttcaagtcctgccatcgtcgcca
1-3tRNA-Tyr(gta)[138530,138613]
gtgagtgtggcagagcggtcgaatgcaggagactgtaaatctccccgtaa
cagcgcggtggttcgaatccatccactcacacca
1-4tRNA-Ser(gct)[138924,139012]
ggaaggttgcccgagaggtttaagggactcgactgctaatcgagtggggc
ttttagcccccgaaggttcgaatccttcaccttccgcca
>GQ981382.1 Shigella phage SP18, complete genome
1-1tRNA-Arg(tct)c[69216,69291]
cgaggcatagctcagaaggaagagcaaggaccttctaagtcctaggtcgt
aggttcgatccctactgtctcgacca
1-2tRNA-Asn(gtt)c[69298,69370]
ggatgcatagctcagtggtagagcagttgactgttaatcaattggtccat
ggttcgaatccatgtgtgtccgc
1-3tRNA-Met(cat)c[69375,69449]
ggcccttttagctcaatgggagagctgtcagctcataactgataggtagct
ggatcgaaaccagcaagggccacca
>HM004124.1 Acinetobacter phage Acj9, complete genome
1-1tRNA-Trp(cca)c[78801,78872]
gggtcgatagtgtagttggaaacactgcggattccaaatccgccatcctg
ggttcgagtcctagtcggcgcc

1-2tRNA-Phe(gaa)c[78879,78954]
ggggatttagctgagacagattagcgcctgcctgaagagcaggattagat
tggtgcaattccaatagtccccgcca
1-3tRNA-Met(cat)c[78956,79030]
ggtgcattagctcagtggtagagcagaggattcatattcctcgtgtcaca
tgttcgaatcatgtatgcacctcca
1-4tRNA-Met(cat)c[79037,79114]
tgcgaagtattttcagtctggtagaaagagaggttcataacctcttatgc
gctcgttcgaatcgggccttcgcttcca
1-5tRNA-Asp(gtc)c[79122,79197]
agactattagctcaatcgggagagcactggcctgtcacgccagaggtagc
gagatcgaaactcgtatagtctgcca
1-6tRNA-Glu(ttc)c[79204,79279]
gctctaatcgtctagttggacaggacgctagcctttcacgttagaaaccg
gagttcgaatctccgttagagtacca
1-7tRNA-Arg(tct)c[79288,79363]
gcgtcattggccgagtggattaggcatcggaattctagtccgactcacat
gggttcgattcctatatggcgctcca
1-8tRNA-Tyr(gta)c[79372,79457]
ggtgatggttgggttgttggtcaaacccatcggactgtaactccgacgct
aacgctatctaggttcgaatcctagcgtcaccacca
1-9tRNA-Pro(tgg)c[79465,79543]
ccgtgtgtgactggagcttggttatcagtctcgctttggaggcgagaata
cgtaggttcaaatcctaccgcacggacca
1-10tRNA-Ile(gat)c[79603,79678]
cgtcgaatagctcagttggtagagcaacggaccgataatccgcaggtcgc
tggttcgaatccagcttcgacgacca
1-11tRNA-Ser(tga)c[79684,79779]
ggtagagtggcagagtctggcttattgctccggtcttgaaaaccggcgat
ccattgcaaaatgggtccgtgggttcaaatcccacctctatctcca
1-12tRNA-Asn(gtt)c[79787,79862]
tcctcgatagctcagttggtagagcagcagactgttactctgtttgtccc
acgttcgaaccgtggtcggggagcca
1-13tRNA-Lys(ttt)c[79870,79945]
tggacgttagctcagttggtagagcagaggatttttaatcctaaggtcaa
tggttcgaacccattacgtccaacca
1-14tRNA-Thr(tgt)c[79955,80030]
gccatcatagctcagttggtagagcagcggttttgtaatccgaaggtcgt
aggttcgattcctactgatggcacca
1-15tRNA-Gly(tcc)c[81552,81628]
gcggtattagctcagttggtaagagcgtcgtccttccaagtcgaaagcca
tcggttcgagtccgatatgccgctcca
1-16tRNA-Cys(gca)c[81632,81707]
agtgcgatggcagaatggtcatgcggtggattgcaaatccacgtcaatat

gggttcgagtcccattcgcacttcca
1−17tRNA−Met(cat)c[81715,81790]
gggcctttagctcaattggcagagcagcccactcataatgggttggtttt
cggttcgagtccgggaaggcccacca
1−18tRNA−Pyl(cta)c[82721,82793]
cgctcagtaggttagtggtagacttacagactctaaatctggatgcaacg
gttcgaatccgttctgagtgacc
1−19tRNA−Tyr(gta)c[82816,82908]
cggtgatgagctagttggttaagcttatgatgatgtacacatcatgtaaa
acgggtactcctgttgtcatcagttcgaatctggtattatcgt
>HM071924.1 Enterobacteria phage IME08, complete sequence
1−1tRNA−Arg(tct)c[69402,69477]
cgaggcatagctcagaaggaagagcaaggaccttctaagtcctgggtcgt
aggttcgatccctactgtctcggcca
1−2tRNA−Asn(gtt)c[69482,69556]
ggatgtgtagctcagtggtagagcagttgactgttaatcaattggtccat
ggttcgaatccatgcatgtccgcca
1−3tRNA−Met(cat)c[69561,69634]
ggccctgtagctggacggtcaagcgagcgactcataatcgctggatggtg
gttcgattccacccagggccacca
>HM134276.1 Enterobacteria phage RB16, complete genome
1−1tRNA−Met(cat)[49781,49857]
ggccctttagcttaattggttagagcgggtgactcataatcgcgcggttg
ctggttcgaatccagcaggggccacca
1−2tRNA−Gly(tcc)[49863,49938]
gcaggtgtagttcaattggtagaatatctggtttccatccagattgttga
gggttcgagtcccttcacctgctcca
>GU323318.1 Enterobacteria phage CC31, complete genome
1−1tRNA−Gln(ttg)c[69064,69137]
tggggtatagccaagttggtaaggcagtagattttgattctacgattccc
tggttcgagtccaggtacactagc
1−2tRNA−Met(cat)c[69144,69220]
tgcgggttaacttcagttggtagaatgacgggttcatatcccgttacgcg
atggttcgagtccatcacccgcctcca
1−3tRNA−Asp(gtc)c[69341,69416]
ggacctatagtttcagcggtgaaaatactcgcctgtcacgtgagagtcac
gggttcgaatcccgttaggtccgcca
1−4tRNA−Asn(gtt)c[69659,69743]
gggtcgttggctgagagggtaagcggcggactgttaatccgtgtcagaaa
tgactaggcaggttcgatacctgcacgacccgcca
1−5tRNA−Glu(ttc)c[69749,69823]
tctccgttcgtctatcggttaggacgccggcctttcacgtcggaaagaag
agttcaattctcttacggagaacca
1−6tRNA−Lys(ttt)c[69825,69902]

aggacgttagctcagttggttagagcatccgacttttaatcggaatgtcg
atgggttcaactccctcacgtcctacca
1-7tRNA-Ile(gat)c[69941,70016]
gggaatatagctcagttggtagagcaaacgaccgataatcgttaggtcac
tggttcgagtccagttattcccacca
>HM452125.1 Aeromonas phage phiAS4, complete genome
1-1tRNA-Leu(taa)[102155,102239]
gcaagtatggtggaattggtatacactggaggcttaaaacctcccgcctt
cgggattgtgggttcgagtcccactacttgcacca
1-2tRNA-Ser(tga)[102431,102524]
ggaagattggcccgagttggtttaaggcaccggtcttgaaaaccggcgat
cgtaggaatacggtccatccgttcgaatcggatatcttcctcca
1-3tRNA-Asn(gtt)[102693,102775]
ggtgatgtagcataacggtcatgcggccggctgttaaccggtgaggaaac
tctacgttggttcgactccaaccatcaccgcca
1-4tRNA-Tyr(gta)[102789,102871]
ggaacgttcgggtaatggtatcccaagcggctgtaacccgctcgcctctg
gcattcttggttcgagtccaaggcgttccacca
1-5tRNA-Lys(ttt)[103428,103503]
gcatctctaactcaattggtagagtagcggacctttaatccgtcaagtct
gagttcgagtctcaggggatgcacca
1-6tRNA-His(gtg)[103793,103867]
gtgaccttagtagtaatggtagctatcctggatgtgacccagggagtacg
ggttcgaaccccgtaggtcacccca
1-7tRNA-Gly(tcc)[103880,103955]
gcgggtaaggtgtttatggatacatgctagccttccaagcttgagtagac
cggttcgataccggctacccgctcca
1-8tRNA-Pro(tgg)[104200,104274]
ctcagcgtaggcaagtggtatgtcgttccgtttgggacggaattttcggg
tgttcgattcaccccgctgagacca
1-9tRNA-Thr(tgt)[104910,104985]
gccggtttagctcatctggtagagcacagcctttgtaacgctggggtggt
ccgttcgagtcggacaatcggcacca
1-10tRNA-Trp(cca)[105350,105425]
atgacattggtgttagcggtagcatgccggtctccaaaaccgtgcggcca
gggttcgaatccttgatgtcatgcca
1-11tRNA-Ile(gat)[105428,105503]
agtggattagctcagtaggtagagcactcgaccgataatcgagagcgcat
tggttcgatcccaatatccactacca
1-12tRNA-Ser(gct)[105512,105599]
ggagaagtggatgagcggctgaaatcgcttccctgctaaggaagtaaacc
gaaaggtttcgagagttcaaatctctccttctccgcca
1-13tRNA-Asp(gtc)[105677,105750]
ggagccatagtttatttggttaaaatagtcccctgtcacgggacagcacc

gagttcgatcctcggtggctccgc
1-14tRNA-Met(cat)[105763,105839]
tgcgatgtagaggagaggtcgtcctcgtcgggctcatatcccgaaaatca
gcggttcgaatccgctcatcgcttcca
1-15tRNA-Met(cat)[106042,106116]
ggccctgtagctagacggttcaagcaggcggctcataaccgctcgtagta
ggttcgattcctaccagggtcacca
1-16tRNA-Arg(tct)[106685,106761]
ggtctcttagctcagttggatagagcagcggccttctaagccgcgggtca
ttggttcaaatccaatagggactgcca
>HM032710.1 Acinetobacter phage Ac42, complete genome
1-1tRNA-Thr(tgt)c[79430,79505]
tccgaattagctcagttggtagagcagtggttttgtaatccaaaggtcat
ccgttcgagtcggatattcggaacca
1-2tRNA-Trp(cca)c[79609,79682]
aggtcattggtgtaatggcagcacagcgggctccaaccccgttagtcaga
gttcaaatctttggtggcctgcca
1-3tRNA-Tyr(gta)c[79806,79891]
gtgcatcgtcgggttgttggtcaaacccagcggactgtaactccgccacg
aaagttatctgtgttcgaatcacagggtgcacacca
1-4tRNA-Ser(tga)c[79897,79987]
ggaagagtaaaccaagtcggtctacgggtcgctgtcttgaaaacagttgg
gtgtcacagccgtgtgagttcaagtctcacctcttccgcca
1-5tRNA-Arg(tct)c[80411,80487]
ccgctagtagctcagatggatagagcatccgccttctaagtggatggtca
ctggttcgagtccagtttagcggacca
1-6tRNA-Met(cat)c[80657,80731]
gggcctataactcaatagtagagtaccgatctcataaatcggaggattca
ggagcgtaacctgttgggcccacca
>HM563683.1 Enterobacteria phage vB_EcoM-VR7, complete genome
1-1tRNA-Arg(tct)c[66571,66647]
cggggtgtaactcaattgtatagagtagcggtcctctaaaccgcaggtta
aaggttagaatcctttcaccccgacca
1-2tRNA-Met(cat)c[66652,66726]
ggccctttagctcaatgggagagctgtcagctcataactgataggtagct
ggatcgaaaccagcaagggccacca
>GU071095.1 Synechococcus phage S-SM2, complete genome
1-1tRNA-Leu(taa)[22309,22394]
tgggagcgtggcggaatcggtagacgcaccagacttaaaatctgttgaga
attatctcgtgggggttcaagtccccccgctcctat
1-2tRNA-Thr(tgt)[22699,22772]
gccaacttagctcagttggatagagcagggtttttgtaaagctcaggtca
tcggttcaagtccgatagttggct
1-3tRNA-Ala(tgc)[129310,129382]

ggggttatagctcaactggtagagcgcctgctttgcacgcaggaggtttg
gggttcgagtccccataactcca
1−4tRNA−Arg(tct)[140954,141029]
gactcagtagctcagttggatagagcaactgccttctaagcagtcggtcg
taggttcgagtcctacctgggtcgcc
1−5tRNA−Gly(tcc)[141032,141103]
gcgggtgtggtgtagcggtaacatgcgagccttccaagctcttgtcacgg
gttcgatccccgtcacccgctc
1−6tRNA−Asn(gtt)[141254,141328]
ttcctcagtagctcagcggcagagccgacgactgttaatcgtctggtcgt
aggttcaaatcctacctggggagtc
1−7tRNA−Val(tac)[141330,141403]
tgcttgcttagctcagaggtagagcatctcgtttacaccgaggcggtcgg
cggttcgatcccgtcagcaagcat
1−8tRNA−Ser(tga)[141678,141764]
ggaagagtggtcgagtggtttatggcactggtcttgaaaaccagcgaggg
tgcaagttctccgtgggttcaaatcccacctcttccg
1−9tRNA−Pro(tgg)[142214,142287]
cggggtgtagctcagtttggtagagcactcgctttgggagcgagtggccg
taggttcaaatcctatcaccccga
1−10tRNA−Ile(tat)[142480,142553]
gggactgtcgcctaacggttaaggcccactgcttataacggtgtgacctg
ggttcaactcccagcagtcctacc
1−11tRNA−Met(cat)[142555,142629]
tggaggattagcaatctggtgaatgcagcaaactcataatttgcctaagg
tgagttcgatcctcacatcctctat
>GU071099.1 Prochlorococcus phage P−RSM4, complete genome
1−1tRNA−Leu(taa)[22119,22203]
gggagtgtggcggaatcggtagacgcaccagacttaaaatctgttgggca
tgtgcccgtgagagttcaagtctctctactcctac
1−2tRNA−Met(cat)c[109227,109301]
tgagggtgtagcaatctggtgaatgcaccgaactcataattcggctaagg
cgagttcgatcctcgccacccttat
1−3tRNA−Arg(tct)[165836,165910]
tgggacagtagctcagcggaaagagcaactgccttctaagcagtcggtcg
taggttcaattcctacctgtctcgt
>GU071098.1 Synechococcus phage S−SSM7, complete genome
1−2tRNA−Gly(tcc)[151563,151633]
gcggatgtagtttaatggtaaaatacgaggtttccaacctcttgtcctca
gttcgattctgagtatccgct
1−3tRNA−Ile(tat)[216422,216494]
gggactatcgcatattggttaatgccctctgcttataacggagtgaaccg
agttcaattctcggtagtcctac
1−4tRNA−Thr(tgt)[216497,216572]

gccactttagctcagttggatagagcaacgcatttgtaatgcgtaggtcg
tcggttcaagtccgacaagtggctcc
1–5tRNA–Arg(tct)[216575,216650]
gactcaatagctcagttggatagagcaactgccttctaagcagtcggtcg
taggttcgagtcctacttgagtcgcc
1–6tRNA–Leu(taa)[216652,216737]
tgggagtgtggtggaatcggtagacacaccagacttaaaatctgttgaca
gcaatgtcgtgggggttcaagtccccccactcccat
>FQ482083.1 Erwinia amylovora phage phiEa104 complete genome
1–2tRNA–Pro(tgg)[22026,22103]
ctccgtttagctcagtatggatagagtaggccgtttggggcggtcgggtc
gaaggttcgaatccttcaacggagacca
1–3tRNA–Ala(tgc)[22224,22296]
gggtcagtagcttaattgggaaagcatctcacttgcaatgagaaggatga
gggttcaagtccctcctgtatcc
1–4tRNA–Met(cat)[22479,22553]
agcgggatggagcagtggtagcttgccagtttcattatctggaggtcgta
ggttcgaatcctactcccgcttcca
1–5tRNA–Asn(gtt)[22562,22637]
gggtttgaagcacatatggatgtgcggctggctgttaaccagatggtagt
gggttcgagtcccacctttcccgcca
1–6tRNA–Tyr(gta)[22644,22726]
gcgcctatggcagagatggtcaatgcagcggtctgtaaaaccgccccgaa
aggttactggttcgagtccagttaggcgcacca
1–7tRNA–Met(cat)[22733,22806]
tgcgaagtagtgaaatggcatcatactggtttcattatccggtgtaggca
gttcgaatctgcccttcgcatcca
1–8tRNA–Asp(gtc)[22886,22962]
ggggatgtggcagacttggtaattgtaccgcactgtctatgcggaatatg
agggttcaaatcccttcatcctcgcca
1–9tRNA–Ser(tga)[23191,23281]
ggaagggcaaatcgactggcgacgaaaacggtcttgaaaaccgccgagcg
ttaatagcgccttgagggttcgactccctctccttccgcca
1–10tRNA–Phe(gaa)[23283,23357]
ggtctaattagcttatatggtaaagcaacggtctgaaaagccgtggaaca
gggttcgaatcccgatagaccgcca
1–11tRNA–Lys(ttt)[23365,23440]
cggtgtgtaactcaattggcagagtgggtggcttttaaccacagggttgc
aggttcgatgcctgtcacaccgacca
1–12tRNA–Lys(ctt)[23448,23524]
ggactattaactcaactggtcagagtacccgactcttaatcgggaggttc
agggttcgactccctgatagtctacca
1–13tRNA–Met(cat)[23536,23609]
agtggtatggcagagatggtcatgcacttccttcatacggaagcctacat

cggttcaagtccgattaccacttc
1–14tRNA–Ile(gat)[23623,23697]
tgtgggttagcataaatggtaatgcaaacgcctgataagcgttagaagag
ggttcgataccctcacctacgacca
1–15tRNA–Gln(ttg)[23704,23779]
cggggtatggagtaattggcaactctacggtttttgaggccgtcagtttt
cggttcgaatccgaatgcccccgcca
1–16tRNA–Arg(tct)[23786,23862]
gcacccttagaacaaacggatagttcccctgtcttctaaacagttagtta
caggttcgaatcctgtagggtgtgcca
1–17tRNA–Leu(caa)[24280,24356]
ggcgtaatatcccaattggcagaggaagcaggttcaaaccctgttcagtg
tgggttcgactcccacttacgctacca
1–18tRNA–Gly(tcc)[24487,24562]
gcaagtatgatgtcaactggtaacatggcgtccttccaagtcgctcttac
gggttcgaatcccgttacttgctcca
1–19tRNA–Trp(cca)[24568,24643]
taggggattagtttaactggcaaaatgtcggcctccaaaaccgcacgttg
agggttcgaatccctcatcccctgtc
1–20tRNA–Thr(tgt)[24654,24728]
gcctccatcatataagggctattatgcctgttttgtaatcaggtcatcgc
ggttcgaatccgtgtgggggcacca
1–21tRNA–Val(tac)[24736,24811]
actcgtatagctcagatggtagagcggcatctttacacgttgctggtcag
gcgttcgagtcgccttgcgagtacca
1–22tRNA–Leu(aag)[24818,24895]
ggctgtgtagcccaacttggcagaggcactacgctaagaacgtatacagt
gagagttcgaatctctccacagctacca
1–23tRNA–Arg(acg)[24902,24978]
agccctttatcttaaatggatagagactcaagctacgaacttgagaggtt
tgggttcgaatcccaaaagggcttcca
1–24tRNA–Gln(ctg)[24981,25057]
aggatgttcgtatagttggcctattacaccggactctgaatccggttacg
atggttcgaatccatcacgtcctgcca
1–25tRNA–Leu(taa)[25064,25140]
aggtgattaccccaattggcagagggtaggagcttaaacctccgatgttg
agagttcgaatctctcatcacctacca
1–26tRNA–His(gtg)[25144,25220]
gagttggtatcataaatggataatgaccctgactgtgaatcaggtctatg
cgggttcgaatcccgtccttctcccca
1–27tRNA–Ser(gct)[25227,25319]
ggaagattaaccctaaaaggtaagggaagtgtttgctaaacactcagtaa
ccgagaaatcggcgtaccagttcaagtctggtatcttcctcca
>FQ312032.1 Salmonella phage Vi01 complete sequence

1−1tRNA−Met(cat)[137231,137306]
ggccccgtagctcagtggttagagcagtcgactcataatcgattggtcgc
tggttcaagtccagccagggtcacca
1−2tRNA−Asn(gtt)[137626,137701]
gacgatgtagttcagtcggtagaacggtggactgttaatccatatgtcgc
aggttcaagtcctgccatcgtcgcca
1−3tRNA−Gln(ttg)[138089,138164]
aggagcatagccaagttggttaaggcagtgggttttgatcccatgatctc
aggttcaaatcctgatgttccttcca
1−4tRNA−Tyr(gta)[138314,138395]
gtgagtgtggcagagcggtcgaatgcgcctgactgtaaatcaggtatccc
acgcggtggttcaaatccatccactcacacca
1−5tRNA−Ser(gct)[138472,138560]
ggaaggttgcccgagaggtttaagggactcgactgctaatcgagtggggc
ttttagcccccgaaggttcgaatccttcaccttccgcca
>AP011617.1 Thermus phage TMA DNA, complete genome
1−1tRNA−Thr(tgt)[51806,51881]
ggcctcgtagctcaacaggaagagcacccgccttgtaagcggggggttgt
gggttcaaatcccaccggggcctcca
1−2tRNA−Met(cat)c[119137,119213]
ggggcgttagctcaagcggtgagagcggtcggctcataaccgattggttg
taggttcgagtcctacacgccccacca
1−3tRNA−Arg(tct)c[119226,119302]
gaggggttagctcaacaggatagagcaagggccttctaagccctaggttg
caggttcaagtcctgcacccctcgcca
>HM114315.1 Acinetobacter phage 133, complete genome
1−1tRNA−Trp(cca)c[65864,65935]
gggtcgttagtgtagttggaaacactgcggattccaaatccgccatcgag
ggttcgagtccttcacggcgcc
1−2tRNA−Met(cat)c[66679,66754]
ggtagtttagctcagtaggtagagcagcggaatcataatccgtaggtcgg
tggttcgagtccaccaattaccacca
1−3tRNA−Met(cat)c[66755,66831]
tgcgaggtaacttcagcctggtagaatgagaggctcataacctcttacgc
gcacgttcaaatcgtgtcctcgcttcc
1−4tRNA−Asp(gtc)c[66937,67012]
agattcgtagctcaatcggaagagcacccgcctgtcacgcgggaggtagc
gggatcgaaacccgtcgggtctgcca
1−5tRNA−Gln(ttg)c[67251,67324]
tgtcgcatggtgtaacggtagcaccacagaatttgactctgttagtaaag
gttcgattccttttgcgacttcca
1−6tRNA−Pro(tgg)c[67417,67493]
ctctcattagggcagtctggtagcctacttcttttggaaagaagatgtcg
ttggttcaaatccaacatgggagacca

1−7tRNA−Ile(gat)c[67500,67575]
cgtcgagtagctcagttggtagagcatcggaccgataatccggtggtcgc
tggttcgaatccagcctcgacgacca
1−8tRNA−Ser(tga)c[67825,67916]
ggtaaggtggcagagtctggcttattgcggtggtcttgaaaaccactgac
gctttcgggcgtccctgggttcgaatcccagtcttacctcca
1−9tRNA−Asn(gtt)c[68224,68299]
tcctcagtagctcagttggtagagcgacgcactgttactgcgttggtcgt
acgttcgaaccgtacctggggagcca
1−10tRNA−Thr(tgt)c[68306,68381]
gccgttatagctcagatggtagagcaggcgatttgtaatcgtcaggtcct
gggttcgaatcctagtgacggcacca
1−11tRNA−Glu(ttc)c[68383,68458]
gttcatatcgtccagcggtctaggacgccagcctttcacgttggtaaccg
gggttcaaatccccgtatgaacacca
1−12tRNA−Tyr(gta)c[68460,68544]
gagtgtgtaggaaaattggtaaccccagcggattgtaaatccgccgccgc
aagcactgaccgttcgactcggtccacactcgcca
1−13tRNA−Arg(tct)c[68572,68647]
ccctctctggccgaatggattaggcatcgtagttctattgcgatttatac
aggttcgagtcctgtgagggggtcca
1−14tRNA−Cys(gca)c[69475,69550]
ggtgtcatggtagaatggtcatacggcggattgcaaatccgcgtcgatac
tggttcgagtccagttgacacctcca
1−15tRNA−Gly(tcc)c[69656,69731]
gcggttgtaactcagatggtagagtgctgtccttccaagtcagatgtcgc
gagttcgaatctcgtcaaccgctcca
1−16tRNA−Met(cat)c[69740,69815]
gggcctatagctcaattggcagagcagcggactcataatccgttggtttc
aggttcgagtcctgctgggcccacca
>GU071096.1 Synechococcus phage S−ShM2, complete genome
1−1tRNA−Arg(tct)[164009,164081]
gggtcagtagctcagtggatagagcatcgcacttctaatgcgttggtcgg
gggttcaaatccctcctgacccg
>GU071097.1 Synechococcus phage S−SSM5, complete genome
1−1tRNA−Val(tac)[15313,15384]
gggagaatagctcagcggtagagctactcgtttacaccgagtcggtcggg
ggttcgatcccctcttctccca
1−2tRNA−Leu(taa)[19381,19468]
tgggagtgtggcggaatcggtagacgcaccagacttaaaatctgttgaga
attaatctcgtgggggttcaagtccccccactcctatc
1−3tRNA−Thr(tgt)[19469,19542]
gccactttagctcagctggatagagcaacggttttgtaaaccgtaggtcg
tcggttcaagtccgacatgtggct

1-4tRNA-Arg(tct)[161698,161772]
tgggtcagtagctcagcggatagagcaactgccttctaagcagtcggtcg
taggttcgatccctacctgacccgt
>KC862297.1 Pseudomonas phage PAK_P1, complete genome
1-1tRNA-Gln(ttg)[90515,90588]
tgccgcttcgttcaatggtaggacgccagactttgaatctggagatgatg
gttcgatcccatcagtggctgcca
1-2tRNA-Arg(tct)[90895,90969]
gctcgtatagtgtaatggatgcacaacggtcttctaagccgtaaggtcta
ggttcgagtcctagtacgagcgcca
1-3tRNA-Lys(ttt)[90979,91054]
tggacggaagctaaagtggataggcatctggcttttaaccagactatagt
gagttcgagtctcacccgtccaacca
1-4tRNA-Leu(tag)[91329,91413]
ggccctgtggtggaattggtatacacatcagtcttagaaactgacgccga
gaggattgagggttcaagtccctccggggccacca
1-5tRNA-Ile(gat)[91624,91699]
agccgggtagctcaattggtagagcacccgaccgataatcgggtggttga
aggttcaagtccttctctggctacca
1-6tRNA-Asp(gtc)[91709,91787]
ggcccattagctcagtctggactagagcaagcccctgtctagggcaaggt
cgtcggttcgaatccgacatgggtcgcca
1-7tRNA-Cys(gca)[92198,92273]
cccgcgttggccgagaggtttaggcggcggattgcaaatccgtctcacat
cggttcaaatccgatacgcggctcca
1-8tRNA-Asn(gtt)[92282,92356]
tgggatgtagctcagtggtagagcagggagctgttaactctcaggtcgca
ggttcgagccctgccgtcccagcca
1-9tRNA-Pro(tgg)[92420,92497]
ctcctcgtagctcagtctggttagagtgtctgatttggaatcagaaggtc
gaaggttcaaatccttccggggtgacca
1-10tRNA-Gly(tcc)[92504,92579]
gcgggtatagctcagttggtagagcgtctgccttccaagcagttcgtcgt
cggttcaagtccgtctatccgctcca
1-11tRNA-Phe(gaa)[92586,92661]
gcatttgaagctaactaggtagaagcgctgggttgaaattccagaggact
tggatcgttaccaagcagatgcacca
1-12tRNA-Glu(ttc)[92668,92743]
gcagttatagattaattggttaaatcgccagactttcaatctggtgttcc
gggttcgatccccggtaactgctcca
1-13tRNA-His(gtg)[92804,92878]
gtggagattgtgtagcccggatgctgcctttcggttgtgacccgattgtt
ggggttcgagtccccgcctccaacc
1-14tRNA-Thr(tgt)[93065,93139]

gccctttaagcatttatggtgatgcaccggctttgtaacccggcgaattc
tgttcaagtcaggaatggggcacca
>HM242243.1 Brochothrix phage A9, complete genome
1–1tRNA–Arg(tct)c[25601,25676]
tgccccggtaactcaaacggatagagtatcaaacttctaatttgaaagtt
acaggttcgagtcctgttcggggcat
1–2tRNA–Arg(cct)c[25761,25832]
gcctttgtagtttaacggataaaacagacccctcctaagggttagctaga
ggttcgattcctctcaagggca
1–3tRNA–Lys(ttt)c[26744,26818]
agataaggtagctcaattagtagagcagggagccttttaagccccagata
taggtgcaaaccctatccttgtttt
1–4tRNA–Met(cat)c[26848,26920]
ggacccttagctcagttggtagagccccctgctcataacagggtttgcgt
aggttcaagtcctacagggtcca
1–5tRNA–Met(cat)c[27656,27728]
cgaggagtagagaagtggtcatctcaccagcctcataagctgggaatcga
gggttagagtccttcctcctcaa
>AB472900.2 Pseudomonas phage KPP10 DNA, complete genome
1–1tRNA–Asn(gtt)[83391,83467]
tccgttcggtccctcaaggtgagggagcttgactgttaatcaagacgtgc
ctggttcgattccagggagcggagcca
1–2tRNA–Tyr(gta)[83525,83612]
ggaggggtggcagagcggtttaatgcaccggactgtaaatccggcgtccg
accgggcatcgctggttcaaatccagccccctccacca
1–3tRNA–Gln(ttg)[83814,83890]
aggcgtgtggcgaaaaggtttaacgcactggactttgactccagcatttg
tgggttcgaatcccaccacgtctgcca
>HM035025.1 Shigella phage Shfl2, complete genome
1–1tRNA–Arg(tct)c[67981,68057]
cgaggcatagctcaattgtatagagcaacggacttctaatccgtgggttg
aaggttagaatccttctgtctcgacca
1–2tRNA–Asn(gtt)c[68062,68136]
ggatgtgtagctcaatggcagagcgatcgcctgttaagcgattggttata
ggttcgaatcctatcacgtccgcca
1–3tRNA–Tyr(gta)c[68141,68227]
ggggagttatcccgtagaggtagcggtgtggactgtaaatccattgtcat
tgcgactcgggtggttcgactccatcactccccacca
1–4tRNA–Met(cat)c[68237,68311]
ggccctgtagctggaaggttcaagcaagcgactcataatcgccagatggt
ggttcaattccacccagggccacca
1–5tRNA–Thr(tgt)c[68313,68388]
gctgatttagctcagtaggtagagcacctcacttgtaatgaggacgtcgg
cggttcgattccgtcaatcagcacca

1–6tRNA–Ser(tga)c[68390,68478]

tggaggcgtggcagagtggtttaatgcaccggtcttgaaaaccggcagtc

gctccggcgactcataggttcaaatcctatcgcctccgt

1–7tRNA–Pro(tgg)c[68479,68553]

ctccgtgtagctcagtttggtagagcgtctgctttgggagcagaatgtcg

taggttcaaatcctgccacggagac

1–8tRNA–Gly(tcc)c[68564,68637]

gcggatatcgtataatggcattacctcagacttccaatctgatgatgtga

gttcgattctcattatccgctcca

1–9tRNA–Leu(taa)c[68643,68729]

gcgagaatggccaaattggtaaaggcacagcacttaaaatgctgcggaat

gatttccttgtgggttcgagtcccacttctcgcacca

1–10tRNA–Gln(ttg)c[68730,68803]

tgggaattagccaagttggtaaggcactggattttgattccaggatgcaa

aggttcgagtcctttattcccagc

>HQ615693.1 Synechococcus phage S–CRM01, complete genome

1–2tRNA–Leu(taa)c[88543,88618]

tgccggtgtagcccaatggtaggaggcagctgatttaagcccagcccagt

gcaagttcgagtcttgtcaccggtat

1–3tRNA–Met(cat)c[88642,88716]

tgctcgtttagctatctggtgaaagcagcgaactcataattcgcctcagg

tgggttcgatcccctcaacgagcat

1–4tRNA–Gly(tcc)[127547,127619]

gcgatcgtagttcagcggtagaacgctatccttccaagttagatgtcgct

ggttcaaatccagtcggtcgctc

1–5tRNA–Arg(tct)[127623,127698]

gccctcgtagttcagcccggatagaacaccgcacttctaatgcgtttgtc

gtaggttcaaatcctaccgagggtgc

1–6tRNA–Lys(ttt)[128952,129026]

tgggttcgtagcaatgtggccaactgcacccggcttttaaccggagtatg

tgggttcgattcccaccggacctat

1–7tRNA–Pro(tgg)[129165,129236]

cggggtgtggctcagaggtagagcgcttgttttgggaacaagacgtcgca

ggttcgatccctgccatcccga

1–8tRNA–Tyr(gta)[129535,129620]

gggtgtatggcaactattggtatgttgcggttgtctgtaaaacaactgtc

cctgactattggaggttcgattcctcttgcgcccac

1–9tRNA–Gly(gcc)[130519,130591]

gcgaatgtaattcagtggtagaatgtctgcttgccaagtagattgtcgtc

ggttcaaatccgatcattcgctc

1–10tRNA–Asp(gtc)[130857,130931]

ggtcctgtagcatagtggtctaatgcactaccctgtcacggtaggttacg

tcggttcaaatccgatcaggatcgc

1–11tRNA–Trp(cca)[130984,131056]

tgcctagttcgtctaatggtaagacaagagcttccaaacctcttgacgtg
ggttcgattcctacactgggtgt
1−12tRNA−Asn(gtt)[131716,131787]
tggcatgtagttcagcggtagaacgcttggctgttaaccaagttgtcgca
ggttcgatccctgccgtgccag
1−13tRNA−Glu(ttc)[131866,131942]
gctccagtagacaaattggtaaagtcgcctctctttcaaagaggacattt
tgcgagttcgaggctcgtctggagttc
1−14tRNA−Glu(ctc)[132042,132116]
tacagtcatcgactagcggtctaggtcgctaccctctcaaggtggaaaca
ccagttcaaatctggttggctgtat
1−15tRNA−Pro(tgg)[134674,134748]
ccgggtgtagctcagtttggccagagcattccgtttggggcggaggggtc
agaggttcaaatcctctcactcgga
1−16tRNA−Pro(tgg)[134812,134885]
tctgcattgggcgagtctggaactccgccgggcctggcgcccggagacgt
tggttcaaatccaacatgcaggtc
1−17tRNA−Ile(gat)[134944,135015]
ggagatatagctcaacggcagagcaaccggccgattaccggatgatgaga
gttcgactctttctatctctac
1−18tRNA−Ile(gat)[135054,135129]
taggaacatagcatagtggcctaatgcacacggctgataaccgtgagatc
gtcagttcaaatctgactgttcctat
1−19tRNA−Phe(gaa)[135201,135274]
gggaacttagctcagttgcgtagagcacttggttgaagcccaaggtgtcg
tcggttcgattccgtcagttccca
1−20tRNA−Ala(tgc)[135312,135386]
tggggtcttagcacaactggtagtgcaattgctttgcaagcaataggtta
ggggttcgagtcccctagattccat
1−21tRNA−Val(gac)[135761,135834]
taggtccataactcagcggcagagtgctacctcgacacggtagaagtcgt
cagttcaatcctgactggacctat
1−22tRNA−Ser(gct)[136579,136664]
ggaagtgtggcagagcggctaattgcagttgtttgctaaacaaccgggct
aataacccacacaggttcaaatcctgtcacttccgc
1−23tRNA−Ser(gga)[136667,136760]
tggtgagttgtccgagaggcttatggtgcgatcttggaaagatcgtgtgt
gagattgtatcaagcaccagaggttcaaatcctctactcaccgt
1−24tRNA−Met(cat)[139590,139660]
ggctctatagttcaacggcagaacaggagattcatacccctccatatggaa
gttcaattcttcctagagtca
1−25tRNA−His(gtg)[139662,139738]
gtgtaggtagaataattggttaattcaccagcctgtgaagctggtttatg
cgagttcaagtctcgtcctacacccca

1−26tRNA−Thr(ggt)[139738,139811]
agcccgcatagttcagaggcagaacgccaacttggtaagttggaagtcgt
gagttcaattctcactgtgggctt
1−27tRNA−Thr(tgt)[141268,141341]
gctgatgtagctcaatctggcagagcaggtaccttgtaagtatcaggttg
cgggttcaattcctgtcatcagct
1−28tRNA−Ser(tga)[141517,141587]
tctggagtcgtctaatggcaggacacggcgctttgaacgcttcaatgtgg
gttcgatccctacctccagaa
1−29tRNA−Lys(ctt)[141838,141913]
tgggtgtgtaactcaattaggcagagtagctggctcttacccagtaagtt
gcaggttcgattcctgtcacacccat
1−30tRNA−Cys(gca)[142050,142123]
gtctcagtcgccaagtggcctaaggctctggattgcaaacccagtattcg
tcggttcaaatccgacctgagact
1−31tRNA−Leu(caa)[145010,145084]
gcccgtgtagcgcaattggtaggaggcagcacattcaaaacgtgcacagt
gtgggttcaaatccctccacgggta
1−32tRNA−Ile(tat)[145590,145679]
gggggtatgatgtatggagcatcgtggggtttatattcccaagcgcaccc
gattagtgccgtgttagggttcgattccctataccccctac
1−33tRNA−Arg(acg)[149226,149301]
gcccgattagctcagaggacagagcacttggctacgaaccaaggttatgc
gtaggttcgactcctacatcgggtgc
1−34tRNA−Ser(tga)[149328,149413]
ggaaggccaagccgatgggtgacggcaactgtcttgaaaacagtcgagtg
taaaagccttgggagttcgactctcccgtcttccgc
1−35tRNA−Met(cat)[149448,149521]
tgcgggatagagcagtctggtagctcatcagtctcataagctgaagatcg
gtggttcaaatccacctcccgcct
1−36tRNA−Leu(gag)[149554,149627]
gccgatgtaacccaactggcagaggtagatgtatgagatgcatctcagtg
caggttcaaatcctgtcatcggta
1−37tRNA−Val(tac)[149711,149784]
tgggagcataactcagtggtagagttcctgttttacacgcaggcagtcgg
gggttcaaatccctctgctcctat
>HQ259103.1 Salmonella phage SFP10, complete genome
1−1tRNA−Met(cat)[137450,137525]
ggccccgtagctcagtggttagagcagtcgactcataatcgattggtcgc
tggttcaagtccagccagggtcacca
1−2tRNA−Asn(gtt)[137939,138014]
gacgatgtagttcagtcggtagaacggcggtctgttaaaccgtatgtcgc
aggttcaagtcctgccatcgtcgcca
1−3tRNA−Ser(gct)[138683,138771]

ggaaggttgcccgagaggtttaagggactcgactgctaatcgagtggggc
ttttagcccccgaaggttcgaatccttcaccttccgcca
>GU459069.1 Aeromonas phage 65, complete genome
1–1tRNA–Leu(taa)[107957,108041]
gcttgtgtggtggaactggtatacacattagacttaaaatctaacgcctt
cgggattgggggttcgaatccccccacaagcacca
1–2tRNA–Arg(tct)[112203,112279]
gcgctgttagctcagttggatagagcaacgttcttctaaagcgtgggtcg
ctggttcgaatccagcacggcgcacca
1–3tRNA–Leu(tag)[112286,112369]
ggggatgtggtgaaattggcagacacaccagatttaggttctggcgctta
tagcgtgacggttcgagtccgtccatccccacca
1–4tRNA–Gly(tcc)[112478,112553]
gcgggtgtagctcagttggtagagcttctgatttccaatcagaatgtcgc
gtgttcgagtcacgttacccgctcca
1–6tRNA–Met(cat)[112642,112719]
ggcccccatagctcagttggttagagcgtccgactcataatcgggtggtc
actggttcgagtccagttggggccacca
1–7tRNA–Met(cat)[113178,113253]
tgcgagttggagaagtggtcatctcgacaggctcattacctgtaggtcgg
ttgttcgaatcaaccactcgcaacca
1–8tRNA–Asn(gtt)[113260,113335]
gggcgtgtagttccagcggtagaacgatggactgttaatccatgtgtcgg
gggttcgaatccctccacgcccgcca
1–9tRNA–Ser(tga)[113465,113558]
ggaaggtcaacccgacggtaggcgacgggagcggtcttgaaaaccgccga
ggcgtaacagcgccttgtgagttcgatcctcacatcttccgcca
1–10tRNA–Lys(ttt)[113568,113643]
gcatcgttagctcagcaggtagagcatgggacttttaatctcagggtcgc
tggttcgaatccagcacgatgtacca
1–11tRNA–Tyr(gta)[113650,113733]
ggatgcataggcaaatggttaagccagcggtctgtaaaaccgtagccttc
gggcttcttggttcgaatccaagtgtatccacca
1–12tRNA–Trp(cca)[113740,113814]
aggtctatggtgtagtggtaacatgccggattccaaatccgtgcgtctgg
ggttcaagtcctcatgggcctgcca
1–13tRNA–Ala(tgc)[113820,113896]
gggggattagctcagttggaatgagcgactggtttgcaaccagtaggtca
tcggttcgaccccgatatcctccacca
1–14tRNA–Pro(tgg)[114340,114417]
ccgtgtgtaggctaggcaggtcaagtcgtctgttttgggaccagaagatc
gaaggttcgaatccttccacacggacca
1–15tRNA–Thr(tgt)[114991,115066]
gccgaattagctcatctggtagagcacagcctttgtaacgctggggtggt

ccgttcgagtcggacattcggcacca
1–16tRNA–Ile(gat)[115079,115154]
agtggattagctcagtaggtagagcactcgaccgataatcgagagcgcat
tggttcaaccccaatatccactacca
1–17tRNA–Ser(gct)[115161,115248]
ggagaagtggatgagcggcttaaatcgctcccctgctaagggagtaaacc
ggaaggtttcgagagttcgaatctctccttctccgcca
1–18tRNA–Asp(gtc)[115256,115331]
gaggatgtagtatagtggtcaatatccctgcctgtcacgtaggagatcgc
gggttcgagtcccgtcatcctcgcca
>GU071094.1 Synechococcus phage S–SM1, complete genome
1–1tRNA–Val(tac)[15012,15085]
tgggagattagctcagcggtagagctattcgtttacaccgaatcggtcac
tggttcgattccagtatctcccat
1–2tRNA–Leu(taa)[18953,19039]
tgggagcgtggcggaatcggtagacgcaccagacttaaaatctgttgaga
attaatctcgtgggggttcaagtccccccgctcctat
1–3tRNA–Thr(tgt)[19409,19485]
gccactctagctcagctggatagagcaacggttttgtaaaccgtaggtca
acggttcaagtccgttgagtggctcca
1–4tRNA–Ala(tgc)[19486,19558]
ggggaattagctcaattggtagagcacctgctttgcacgcaggggggttag
cggttcgagtccgctattctcca
1–5tRNA–Asn(gtt)[19607,19680]
ttcctctttagctcagcggtagagcggttgactgttaatcaatttgtccc
tggttcgatcccaggaaggggagt
1–6tRNA–Gly(acc)c[74450,74541]
gggttagttaaactaatggagaggttcgtgccgttaccaatggcaccata
aatttcattaaagttgtcattgatcttatcgccgccagcacg
1–7tRNA–Arg(tct)[160615,160689]
tgggtcagtagctcagcggatagagcaactgccttctaagcagttggtcg
caggttcaaatcctgcctgactcgt
>GU071106.1 Synechococcus phage Syn19, complete genome
1–1tRNA–Val(tac)[17789,17861]
gggcgaatagctcagcggtagagctactcgtttacaccgagtcggtcggg
ggttcgatcccctcttcgcccac
1–2tRNA–Leu(taa)[22314,22400]
tgggagtgtggcggaatcggtagacgcaccagacttaaaatctgttgaga
attaatctcgtgggggttcaagtcccccactcctat
1–3tRNA–Thr(tgt)[22402,22473]
gcccttatagctcagtggtagagcaacgcttttgtaaagcgtaggtcgtt
ggttcaaatccgactgggggct
1–4tRNA–Ala(tgc)[22476,22549]
ggggaattagctcagttggtagagcgcctgctttgcaagcaggatgtcag

gagttcgagtctcctattctccac
1-5tRNA-Asn(gtt)[22554,22625]
tcctctttagctcagcggtagagcggttgactgttaatcaattggtccct
ggttcgatcccaggaaggggag
1-6tRNA-Pyl(cta)[163912,163986]
tgggtcagtagttcagcggatagaacaacgctcttctaaagcgtgtgtcg
ggggttcgattccctcctgacccgt
>GU071103.1 Prochlorococcus phage P-SSM7, complete genome
1-1tRNA-Ile(tat)[117038,117109]
gggactatcgcatattggttaatgcccactgcttataacggtgtgaaacg
ggttcaattcccgttagtccta
1-2tRNA-Arg(tct)[117112,117186]
tgactcagtagctcaaaggatagagcaattgccttctaagcaattggttg
taggttcgagtcctacctgagtcgt
1-3tRNA-Leu(taa)[117189,117261]
gggagtgtagtccaacggcagagacaggagacttaaaatctctacagtgt
gggttcgaatcccaccactccta
1-4tRNA-Asn(gtt)[118123,118209]
ttgtgaggtggcgaaacggtaaacgctctagtctgtttaactagtgtctc
tggcgggacttgtaggttcgactcctaccctcacagt
>FQ482085.1 Erwinia tasmaniensis phage phiEt88 complete genome
1-1tRNA-Phe(gaa)[44731,44806]
ggaactctggcgtaggtggttcgcgcggatgtctgaaaaacctcaggaac
tggttcgattccagcgggttccacca
>GU296433.1 Campylobacter phage NCTC12673, complete genome
1-1tRNA-Tyr(gta)c[85876,85960]
gtagaagtaggataattggtaatccaccagactgtaaatctggcgtcttt
tggcattgatggttcaagtccatccttctacacca
1-2tRNA-Asn(gtt)c[85979,86053]
tgcagattagcacagcggtagtgcaatcgactgttaatcgatgagtcaga
ggttcgaatcctctatctgcagcca
1-3tRNA-Met(cat)c[86147,86221]
gggcgagtagctcaatggtagagcaaccggctcataaccggttggttata
ggttcgattcctgtttcgcccacca
>GU070616.1 Salmonella phage PVP-SE1, complete genome
1-1tRNA-Ala(ggc)c[62894,62975]
tgcgtggtagtgaaacggatatatagatcatgttggcctcataagccgaa
gattgcgggttcgactcccgctcacgcatcca
1-2tRNA-Phe(gaa)c[62982,63057]
ggggttgtaactcagttggtagagtgcctgcctgaagagcagatggtcgg
tggttcgagtccacccggccccacca
1-3tRNA-Val(tac)c[63065,63139]
gcttccttagttcaatggtagaacgttgtctttacacggcaagcgtcggt
ggttcgattccatcaggaagtacca

1–4tRNA–Leu(tag)c[63147,63223]
gggcgattgatggaattggtatacgtgccgtccttagaagtcggattttg
ggggttcgagtcccctgtcgcccacca
1–5tRNA–Ala(tgc)c[63330,63408]
ggggaatgggtctgcttggagtggacacctcgcttgcaccgaggatatca
gaagagttcgaatctcttattctccacca
1–6tRNA–Arg(acg)c[63412,63487]
gcaggtatagttcaactggacagagcatcagcctacgaagctgagaatcg
gggttcgaatccctgtacttgcacca
1–7tRNA–Pro(tgg)c[63675,63750]
cagtccgtagcgcagttggtagcgtgggagccttggatgcttcgggtcgc
aggttcgagtcctgccgggctgacca
1–8tRNA–Asp(gtc)c[63848,63924]
ggggccgtggctgacaaggtaatggtgccgcactgtctatgcggaaaatg
agggttcgatccccttcggcctcgcca
1–9tRNA–Asn(gtt)c[63932,64016]
gacgagttggcctagtggttgggcgacggcctgttaagccgtgagtgaaa
actcaaggaaggttcaaatccttcactcgtcgcca
1–10tRNA–Lys(ttt)c[64081,64163]
gggtctgtagccaagtggtgacgcaagtcgaaggcatccgacttttaatc
ggaggatcgggggttcgaatccctccagacccacca
1–11tRNA–Ser(tga)c[64170,64255]
ggagagtgccgtccaacggtggacaagcaggtttgaaccctgtggtgctg
gaaacggtaggggttcgattcctccactctcctcca
1–12tRNA–Ser(gct)c[64257,64350]
ggaagattaaccctaatcaggtaagggatctctttgctaaagagacagta
gccccgaaaggggtgtgtcagttcaagtctgacatcttcctcca
1–13tRNA–Ser(gga)c[64356,64445]
ggagaagcaaatcgacaggtgacgaaaacaccttggaaaggtgctgactg
gtaaaacggcttgagagttcgattctctccttctccgcca
1–14tRNA–Gln(ttg)c[64559,64635]
agggggttagcatagctggcctaatgcatcgggctttgaactcgacatcg
gaggttcgaatcctccacccccctgcca
1–15tRNA–Glu(ttc)c[64725,64801]
gtcctgttagacaaactggtaaagtcaccaccctttcaaggtggggtttg
cgggttcgatccccgcacaggacgcca
1–16tRNA–Gly(tcc)c[64809,64882]
gcgtcattagtgttagcggtccagcatcccgtccttccaagtcggtggca
tcggttcgaatccgatatgacgct
1–17tRNA–Ile(gat)c[64890,64965]
gggagtatagctcagttagtagagcgctcgaccgataatcgagaggtcgc
aggagcaaagcctgctactcccacca
1–18tRNA–Tyr(gta)c[64973,65056]
ggctcattggcagaacggtgattgcagcggattgtaaatccgtgcccttc

ggggttcctggttcgaatccagggtgggccacca
1–19tRNA–Cys(gca)c[65709,65782]
gcactgttgctggagtggtaacagcttcgtctgcaaaacgaatgtcgtgg
gttcgattccctcacagtgctcca
1–20tRNA–Arg(tct)c[65784,65858]
cggggcatagtctagcggataggcaggagtcttctaaactcctttacgcg
ggttcgaatcctgctgcctcgacca
1–21tRNA–Leu(taa)c[65866,65951]
gcaggtgtggtggaaatggagatacacaggagacttaaaatctcccgctt
aaatgattacgagttcgagtctcgtcacctgcacca
1–22tRNA–Ile(gat)c[66926,67001]
tcctcgttagctcaagttggtagagcagtcccaagattcgggataagcac
aggttcgattcctgtacgaggagcca
1–23tRNA–Thr(tgt)c[67008,67082]
gctcctaaagcataagtggcgatgcagcggccttgtaagccgaagaagtg
ggttcgattcctactgggagcacca
1–24tRNA–Lys(ctt)c[69196,69271]
ggggtattaacacagcggttagtgtagcggcctcttaagccgaaggtcga
aggttcgaatccttcatgccccacca
1–25tRNA–Met(cat)c[69964,70040]
ggttctgtagcttagttggttagagcgcttccctcataaggaagaggtcg
caggttcaattcctgccagaactacca
>JQ691612.1 Cronobacter phage CR3, complete genome
1–1tRNA–Thr(tgt)[144372,144447]
gctggattagctcagtaggtagagcgcctcacttgtaatgaggatgtcgc
gggttcgattcctgcatccagcacca
1–2tRNA–Cys(gca)[144454,144530]
gcaacgttgccggagtctggtaacggctctcgctgcaaacgagatgagca
ctggttcgaatccagtacgttgctcca
1–3tRNA–Leu(caa)[144532,144608]
gcgtctttggtccaattggtagaggcactacattcaaaatgtaggggttc
ccagttcgaatctgggaaggcgcacca
1–4tRNA–Ser(gga)[145443,145537]
agtaggatggctgagtggcttaaagcagcatcttggaaaggtgtcggatc
aggtaacccctggtcccgggagttcaaatctccctcctactgcca
1–5tRNA–Lys(ttt)[145604,145680]
ggatcgttagctcaattggttagagcaagggacttttaatcccgaggttc
ggggttcgagtccctgacggtccacca
1–6tRNA–Asn(gtt)[145846,145921]
tgacggtgagcatatatggacatgcggggacctgttaagtccaggataac
tggttcgaatccagtaccgtcagcca
1–7tRNA–Glu(ttc)[146316,146393]
gtcctggtagacaaactggataaagtcaccaccctttcaaggtggagttt
gagggttcgatcccctctcaggacgcca

1−8tRNA−Glu(ctc)[146400,146479]
tacccggtgcgacaagcggtaagtcgtccaccgctcgaatgaacgtggag
acacaggttcgatccctgtccgggtaacca
1−9tRNA−Asp(gtc)[146575,146650]
ggtcctatggtattagcggtaaacatactcgcctgtcacgcgagagtcac
gggttcgaatcccgttaggaccgcca
1−10tRNA−Ile(gat)[146658,146732]
tggggattagctcaggggtagagcgcacaactgataattgtgaggtcggt
ggttcgaaaccaccatccccaacca
1−11tRNA−Pro(tgg)[146803,146879]
cggtgtatagcgcagcctggtagcgcatctggtttgggaccagagggtcg
ctggttcgagtccagctacaccgacca
1−12tRNA−Met(cat)[146886,146960]
tgcgaggtagttcagcggtagaacaggggtttcatacgcctcatgtcggc
agttcgattctgcccttcgcaacca
1−13tRNA−Leu(tag)[146967,147044]
gctccagtagcccaattggtagaggcacttgatttaggatcatgtcaatg
atgagttcgaatctctcctggagtacca
1−14tRNA−Gly(tcc)[147051,147124]
gcgggcatggtatagtggttgtgccttagccttccaagctaatgagtccg
gttcgatcccggatgtccgctcca
1−15tRNA−Gln(ttg)[147198,147273]
aggggattagtttaattggtaaaacatcgggttttgatcccgacatcgtg
aggttcgaatcctccatcccccgcca
1−16tRNA−His(gtg)[147356,147431]
gtggccttaattcagcaggtagaatccccggttgtgaaccgggcagtcgt
gggttcaagtcccacaggtcacccca
1−17tRNA−Ser(tga)[148112,148204]
ggaagatagggcgtagtggtacgcacccggatttgaaccccggcccgctg
tggcgaccccacggtgatagttcgactctattatcttccgcca
1−18tRNA−Ser(gct)[148209,148295]
agaagagtggcgaaatcggtagccgcaccggactgctaatccggggtccc
tcggggcctgagagttcaagtctctcctcttctgcca
1−19tRNA−Phe(gaa)[148301,148376]
ggaactgtagctcagttggtagagcggttgcctgaagagcagcgcgtcag
cggttcaaatccgttcagttccacca
>JN132397.1 Campylobacter phage CPX, complete genome
1−1tRNA−Tyr(gta)c[38265,38350]
gtaagcatatgggtaattggtaacccaccagactgtaaatctggcgtctc
ttggcactgcaggttcgagtcctgctgcttacacca
1−2tRNA−Arg(tct)c[38361,38437]
ctccatgtagctcagctggatagagcaagaatcttctaagttctaggtcg
ggtgttcaaatcactccatggaggcca
1−3tRNA−Asn(gtt)c[38447,38522]

tgcggattagcacagtggtagtgcaatcgactgttaatcgatggggtcat
aggttcgaatcctatatccgtagcca
1–4tRNA–Met(cat)c[38793,38867]
agcagagtgacgcagtagtagcgtgctgggctcataacccagatgtcgta
ggtgcaaatcctacctctgtaacca
1–5tRNA–Leu(taa)c[38873,38950]
tggacatgtagcgctcaagtggtagagcagccgacttaaaatctgcagat
tgatggttcgatcccatccatgtccaac
1–6tRNA–Met(cat)c[38955,39029]
gggcgagtagctcaatggtagagcaaccggctcataaccggttggttata
ggttcgattcctatttcgcccacca
>JN593240.1 Escherichia virus CBA120, complete genome
1–1tRNA–Met(cat)[136807,136881]
ggtcctgtagctcagtggtagagcagccccctcataagggattggtcgct
ggttcaagtccagccagggtcacca
1–2tRNA–Asn(gtt)[137294,137369]
gacgatgtagttcagtcggtagaacggcggtctgttaaatcgtatgtcgc
aggttcaagtcctgccgtcgtcgcca
1–3tRNA–Ile(gat)[137986,138069]
gcgggagccataggggttggccagtagccgactgatcatcggcgacgatc
actggacaggttcgaatcctgttctcccgctcca
1–4tRNA–Ser(gct)[138074,138162]
ggaaggttgcccgagaggtttaagggactcgactgctaatcgagtggggc
ttttagcccccgaaggttcgaatccttcaccttccgcca
>AJ972879.2 Yersinia phage phiR1–37 complete genome
1–1tRNA–Arg(tct)[67659,67735]
cggggtgtagcataataggataatgcaccgaccttctaagtcggcttacg
tgggttcgactccctccgtcccgacca
1–2tRNA–Leu(tag)[67746,67829]
tgcgaatgtggcgaaattggtagacgccccagatttaggttctggtatcg
aaaggtgtgtgggttcgactccctccattcgcaa
1–3tRNA–Leu(taa)[67838,67925]
gtctgcttgtccaaattggtaaaggaaggagacttaaaatctcccggctg
taacagccttatgggttcgactcccttagcagatacca
1–4tRNA–Asn(gtt)[71536,71613]
gactcgctagctcagttggttagtagcggatgactgttaatcatcaggtc
gcaggttcaagccctgcgcgagtcgcca
>JN022534.1 Xanthomonas phage vB_XveM_DIBBI, complete genome
1–1tRNA–Asn(gtt)[1645,1720]
ggccgattagcaagtgggacacgcggcggactgttaatccgtgaattacc
tggttcgactccaggattggccgcca
1–2tRNA–Ser(gct)[1911,1999]
ggcgggttgtcagagcggcttattgaagttgactgctaatcaaccggggc
gcaagtcccgcagaggttcgaatcctctacccgccgcca

1−3tRNA−Asp(gtc)[2162,2238]
aggcccgtagtgtaggcggttaacatactcccctgtcacgggagagatcg
cgggttcaaaccccgtcgggcctgcca
1−4tRNA−Ala(ggc)c[16132,16220]
gattgtatcggctggcgtgtcgctgttgctggcgtcgatagttccgatgt
gtactgtcacattgttcacggttccacccgctacagttg
>HM144387.1 Bacillus phage W.Ph., complete genome
1−1tRNA−Asn(gtt)[8115,8190]
gtgcttgtagctcagtaggtagagcgtcagattgttaatctgggggtcgc
aggttcaatccctgcctagcacgcca
1−2tRNA−Glu(ttc)[8195,8271]
gtcgcattggtgaaattggctaacacactcggctttctaccgaggattta
ggggttcgagccccctatgcgattcca
1−3tRNA−Asp(gtc)[8275,8348]
tggggatatagtgtagtggtgaacacgcacggctgtctaccgtgaagcac
gagttcgagtctcgttatcctcgt
>JN627160.1 Pseudomonas phage OBP, complete genome
1−1tRNA−Met(cat)c[16040,16116]
ggccccgtagctcagacggttagagcaggtgactcataatcgcttggtcg
cgtgttcaagtcacgccggggccacca
1−2tRNA−Arg(tct)c[16121,16196]
gcgctcttagctcagtggatagagcagtagccttctaagctattggtcac
aggttcgaatcctgtagggcgcgcca
1−3tRNA−Ser(gct)c[16733,16821]
agagagttggatgagttggtttaagtcacctccctgctaaggaggcagtc
cttcggggctcatgggttcgaatcccatactctctgcca
1−4tRNA−Asn(gtt)c[16826,16901]
gctcgtgtagttcagtcggtagaacggcggactgttaatccgtatgtcac
aggttcgagccctgtcacgagcgcca
>JN882285.1 Cronobacter phage vB_CsaM_GAP32, complete genome
1−1tRNA−Ser(gct)[271095,271185]
ggtgagttgggggagtcggtttaacccagcaccctgctaaggtgtcggcc
tcgcaagaggcccgtgggttcaaatcccacactcaccgcca
1−2tRNA−Trp(cca)[275545,275617]
aggagtatagtataacggctattatgtgtggctccaaaccacaagatcag
ggttcgattccttgtgctcctgc
1−3tRNA−Thr(tgt)[278688,278770]
gctgccttcccataattggtattggaccgcacttgtaatgcggcggggaa
acccattctcggttcgagtccgaggggtagcac
1−4tRNA−Leu(tag)[284186,284268]
ggggatgtggtgaaattggcagacacgccagatttaggttctggttcttc
ggagtgcgagttcgagtctcgccatccccacca
1−5tRNA−Arg(tct)[292115,292188]
ctcctgttagttcaaacggaagaacatcgaccttctaagtcgcaggttac

tggttcgagtccagtacgggagac
1–6tRNA–Pyl(cta)[293273,293345]
gcccttgtagttcagcggtagaacgggagtctctaaaactccttgtcgca
ggttcgaatcctgtcaagggcac
1–7tRNA–Leu(taa)[293351,293433]
gcgggtatggtgaaattggtaaacacaggggatttaagttcccccgctga
ggcttgacggttcgagtccgtctacccgcacca
1–8tRNA–Met(cat)[293844,293918]
gggactgtagcttaaagttaaagcagtggcctcataagccaacgagtggg
agttagagtctccccggtcccacca
1–9tRNA–Leu(taa)[296175,296262]
gcgggtgtggcggtattggcagtacgcacaagacttaaaatcttgtaggg
gtttccccttgtgagttcgaatctcatcacccgtacca
1–10tRNA–Leu(caa)[301093,301180]
ggggatatggcgaaattggtagcacgcaggagactcaaaatctcccgcca
gagatggcgtgtcggttcgagtccgactatccccacca
1–11tRNA–Glu(ttc)[306268,306340]
gttcccatcgtctagcggctaggacgcttccctttcaaggaggaaaccgg
agttcgattctccgtgggaacgc
1–12tRNA–Tyr(gta)[306352,306433]
ggggagttggcagagcggtcgaatgcacctgactgtaaatcaggcacacc
acaccctggttcgaatccaggacgccccacca
1–13tRNA–Ser(tga)[306770,306864]
ggaagattggcagagtctggtttattgcaccgcacttgaaatgcggcgaa
ctagtaaccctagttcctacggttcaaatccgtaatcttcctcca
1–14tRNA–Ile(gat)[308436,308510]
gggaatatagcacaattggtcagtgcactcgcccgataagcgagatgtta
cgtgttcgagtcacgttattcccac
1–15tRNA–Asn(gtt)[310988,311061]
ggttacgtagctcagcggttagagcgttcgcctgttaagcgaagggtcgc
tggttcaaatccagccgtgatcgc
1–16tRNA–Gln(ttg)[311137,311210]
aggggattagccaagttggtaaggcacatggttttgatccatgtatgcgg
aggttcgagtcctccatcccctgc
1–17tRNA–Gly(tcc)[311346,311418]
gcatccatcgtatatcggctattataattggcttccacccaatagaacgg
agttcgaccctccgtggatgctc
1–18tRNA–Asp(gtc)[312880,312952]
ggagccatcgttcaacggttaggatacggcactgtcactgctgagatggg
gtttcgattacccctggttccgc
1–19tRNA–Arg(acg)[313536,313612]
gttcgtgtagctcaactggaattagagcgttcgtctacgaagcgaaaggt
tataggttcgatccctatcatgaacac
1–20tRNA–Pro(tgg)[315418,315493]

cggagtgtgggctaattggttagccgcctagtttgggactaggagtttat
gcaggttcgagtcctgtctctccgac
1–21tRNA–Phe(gaa)[317155,317229]
gggtcggtaactcagttggtcagagtgatacactgaaaatgtattggtcg
cgagttcgaatctcgcccgtcccac
1–22tRNA–Lys(ttt)[318210,318283]
acctcgttaactcagttggtagagtaacggtcttttaaaccgtgagtcgc
tggttcgaacccagcacgaggtac
1–23tRNA–Cys(gca)[322498,322575]
gcagggatgcctgagatggcttaaaggagcggattgcaaatccgatgttc
gggggttcgaatccccctccctgctcca
1–24tRNA–Met(cat)[323476,323551]
ggtcagttagttcagttggtagaacgtttcactcataatggagaggcttc
actggttcaagtccagtactgaccac
1–25tRNA–Met(cat)[324419,324498]
tgcggagtagagcagtctggtagcttgcaaggctcataaccttgaggtcg
tgtttggttcgaatcccacctccgcatcca
1–26tRNA–Ala(tgc)[324515,324588]
ggggctatagctcagttgggagagcgtctgccttgcacgtagaaggtcaa
cggttcgaacccgtttagctccac
>HQ698922.4 Acinetobacter phage ZZ1, complete genome
1–1tRNA–Trp(cca)c[73037,73108]
gggtcgttagtgtagttggaaacacggtggattccaaatccgcaatccag
ggttcgagtccttggcgacgcc
1–2tRNA–Pro(tgg)c[73349,73425]
ctccgtatagctcagtctggtagagcactgcgtttgggacgcaggtgtcg
aatgttcaaatcattctacggagacca
1–3tRNA–Ser(tga)c[73639,73733]
ggaaggtcaagccgaaagctggcgacggcagctgacttgaaatcagtcga
gcgtggtgacacgccttgagggttcgaccccctcaccttccgcca
1–4tRNA–Thr(tgt)c[73887,73962]
gccgtcatagctcagttggtagagcgtctgttttgtaatcagagggtccg
gggttcaaatcctcgtgtcggcacca
1–5tRNA–Cys(gca)c[75677,75753]
gacgtcgtggcagaattggttatgcggcggattgcaaatccgcgtcgatg
agggttcgaatccatccggcgtctcca
1–6tRNA–Met(cat)c[75945,76021]
gggcctttagctcaattggttagagcagcggactcataatccgttggttc
ccagttcaagtctggggaggcccacca
1–7tRNA–Arg(tct)c[76027,76102]
ccgtctgtggccgaatggattaggcatcgtagttctattgcgactcacac
aggttcgaatcctgtcaggcggacca
1–8tRNA–Pyl(cta)c[76196,76269]
cgttcagtaggttaacggtagaccaggagtccctaaaactctgtgtgacg

gttcgaatccgtcctgaacgacca
>JN797797.1 Bacillus phage BCP78, complete genome
1–1tRNA–Met(cat)c[37,109]
agggttatagctcagtggtagtagcgtgggtctcataagcccaaggtcgt
aggttcgactcctactagcccta
1–2tRNA–Leu(tag)c[253,327]
tgccgaagtaatccaatggcagagatagcggttttagaaaccgtacagtg
tgggttcgagtcccaccttcggtat
1–3tRNA–Leu(taa)c[332,419]
ggcggagtgttggaattggtagacatatggcacttaaaatgctatgtccg
tatgggcgtggtgggttcgagtccccctccgctacca
1–4tRNA–Ile(gat)c[435,511]
gctagggtagctcagtcaggtagagcagtgtcttgataaggcattggtcg
taggttcaaatcctacccctagtacca
1–5tRNA–Tyr(gta)c[513,599]
gggcgagtatgcaaattggtgaagcaagcggtctgtaaaaccgtgacgta
agatacattgcaggttcgagtcctgcctttcccacca
1–6tRNA–Phe(gaa)c[739,814]
ggacggatagctcagttggtagagcagagggctgaaaatcctcgtgtcgt
aggttcgatccctactccgtccacca
1–7tRNA–Pro(tgg)c[929,1005]
tgtaggtgtagctcagattggctagagcgcttgctttgggagcaagaagt
cgcaggttcaagtcctgccacccgcat
1–8tRNA–His(gtg)c[1047,1118]
cggggtgtggcgtaacggttaacgcaagtgactgtggatcactgaatagg
ggttcgattcccctcatcctga
1–9tRNA–Gln(ttg)c[1201,1273]
tttcggagtagccaagtggtaaggcaatagactttgactctatgatcggt
ggttcgagaccatcctccgaagt
1–10tRNA–Ser(tga)c[1351,1442]
ggaagggtactcaagtggataaaagaggtcggtcttgaaaaccgctaggc
gtgtaaaagcgtgcgtgggttcgaatcccactccttccgcca
1–11tRNA–Arg(tct)c[1447,1523]
tatcccattagccaagttggatcaaggcaacgggcttctatcccgttaat
cgtgggttcgagtcctacatgggatgt
1–12tRNA–Ile(tat)c[1603,1677]
gccccttttagccaagcggttcaaggcagtgggattatgtcctgcgaatcg
gaagttcgaatcttccaaggggagc
1–13tRNA–Glu(ttc)c[1681,1754]
ggggtattggtgaagtggtcaaacacatccgactttctatcggagatacg
agggttcgaatcccttatacccctt
1–14tRNA–Asp(gtc)c[1883,1955]
tggggtattagtttagtggtaaaatactgcactgtctatgcagagtcagg
ggttcaactcccctatacctcgt

1-15tRNA-Gly(tcc)c[1960,2035]
ggggcattagtatatcggttcattattcctggcttccaaccaggggaggt
cggttcgattccgacatgtccctcca
1-16tRNA-Ser(gct)c[2042,2132]
ggagagttgtcagagtggcttatcgtgcctgtttgctaaataggtgtacg
tccttaacgtaccacaggttcaaatcctgtactctccgcca
1-17tRNA-Asn(gtt)c[2619,2693]
gctcctgtagcgtagcggtagcgcagcagcctgttaagctgttggtcggt
ggttcgaatccacccaggagcgcca
1-18tRNA-Cys(gca)c[2707,2780]
gaaggtgtaccgaagtggcttaacgggctagattgcaaccctagttttcg
tgggttcgaatcccaccactttct
>JX569801.1 Campylobacter phage CP30A, complete genome
1-1tRNA-Tyr(gta)c[88608,88693]
gtaagcatatgggtaattggtaacccaccagactgtaaatctggcgtctt
ttggcattgatggttcaaatccatcctcctacacca
1-2tRNA-Arg(tct)c[88704,88780]
ctccatgtagctcagctggatagagcaagaatcttctaagttctaggtcg
ggtgttcaaatcactccatggaggcca
1-3tRNA-Asn(gtt)c[88790,88864]
tgcggattagcacagtggtagtgcaatcgactgttaatcgatgggtcata
ggttcgaatcctatatccgcagcca
1-4tRNA-Met(cat)c[88958,89032]
gggcgagtagctcaatggtagagcaaccggctcataaccggttggttata
ggttcgattcctatttcgcccacca
>JF966203.1 Bacillus phage Bastille, complete genome
1-1tRNA-Asn(gtt)[5856,5930]
tgtgcttgtagctcagtcggtagagcgccagattgttaatctgatggtcg
caggttcgattcctgcttggcacgt
1-2tRNA-Trp(cca)[6692,6765]
gggagtttagtttagtggtaaaacctcggtctccaaaaccgaagtcatat
gttcgattcgtatagctcctgcca
1-3tRNA-Ser(tga)[6767,6854]
ggagggttggcagagtggattattgcagcggtcttgaaaaccgccgaggg
taaaacctccgtgagttcaaatctcacaccctcctcca
1-4tRNA-Asp(gtc)[6856,6929]
tggggatatagtgtagaggtaaacacgtacggctgtctaccgtgaagcac
gagttcgaatctcgttatcctcgt
1-5tRNA-Leu(tag)[7151,7228]
tgccgaagtaatccaatcaggtagagatagcggttttagaaaccgtccag
tgtgggttcgaccccctccttcggtatc
1-6tRNA-Tyr(gta)[7235,7318]
tggcagaatagtcaagcggcaacgacaggtgactgtaaatcacccctcat
tcgagttcgtaggttcgagtcctgcttctgctat

1-7tRNA-Ser(gct)[8231,8318]
ggaggggtactcaagcggtgaagaggttagtttgctaaactaataggtcg
agtaatcggcgcgagggttcgaatccctccccctccac
>JN986846.1 Enterobacteria phage vB_EcoM_ACG-C40, complete genome
1-2tRNA-Arg(tct)c[68043,68118]
cggggcatagctcagaaggaagagcaaggaccttctaagtcctgggtcgt
aggttcgatccctactgcctcgacca
1-3tRNA-His(gtg)c[68123,68198]
gtggccgtagttcagttggtagaactcgagattgtgattctcgtagtcat
gggttcgactcccatcggtcacccca
1-4tRNA-Asn(gtt)c[68313,68387]
ggatgtgtagctcaatggcagagcgatcgcctgttaagcgattggttata
ggttcgaatcctatcacgtccgcca
1-5tRNA-Tyr(gta)c[68392,68478]
ggggagttatcccgtagaggtagcggtgtggactgtaaattcattgtcat
tgcgactcgggtggttcgactccatcactccccacca
1-6tRNA-Met(cat)c[68491,68565]
ggccctgtagctggaaggttcaagcaagcgactcataatcgccagatggt
ggttcaattccacccagggccacca
1-7tRNA-Thr(tgt)c[68567,68642]
gctgatttagctcagtaggtagagcaactcacttgtaatgagaaggtcgg
cggttcgattccgtcaatcagcacca
1-8tRNA-Ser(tga)c[68648,68737]
ggaggtgtggcagagtggtttaatgcaccggtcttgaaaaccggcagtcg
ctccggcgactcataggttcaaatcctatcgcctccgcca
1-9tRNA-Pro(tgg)c[68739,68813]
ctccgtgtagctcagtttggtagagcgcctgatttgggatcaggaggtcc
aaggttcaaatccttgtatggagac
1-10tRNA-Gly(tcc)c[68824,68897]
gcggatatcgtataatggcattacctcagacttccaatctgatgatgtga
gttcgattctcattatccgctcca
1-11tRNA-Leu(taa)c[68903,68989]
gcgagaatggtcaaattggtaaaggcacagcacttaaaatgctgcggaat
gatttccttgtgggttcgagtcccacttctcgcacca
>JN882284.1 Cronobacter phage vB_CsaM_GAP31, complete genome
1-1tRNA-Met(cat)[84261,84337]
ggttctgtagcttagttggttagagcgcttccctcataaggaagaggtcg
caggttcaatccctgccagaactacca
1-2tRNA-Lys(ctt)[85032,85107]
ggggtattaactcagcggttagagtagcggcctcttaagccgaaggtcga
aggttcgaatccttcatgccccacca
1-3tRNA-Thr(tgt)[87106,87180]
gctcctaaagcataagtggcgatgcagcggccttgtaagccgaggaagtg
ggttcgattcctactgggagcacca

1-4tRNA-Arg(gcg)[87187,87260]
tcgttgatagccttagcggtaaaggccccctctgcgcaaggggttcagtg
gttcgaatccacttcagcgagcca
1-5tRNA-Leu(taa)[87563,87640]
gcgctcttggtccaatctggtagaggcatgggtgttaagctcccagggtt
cccggttcgagtccgggagggcgcacca
1-6tRNA-Arg(tct)[87645,87719]
cggggtatagtctagaggataggcaggagtcttctaaactcctttacgtg
ggttcgaatcctactacctcgacca
1-7tRNA-Cys(gca)[87725,87798]
gcgcggttgctggagtggcaacagcttcggctgcaaaccgaatgtcaggt
gttcgattcacctaccgtgctcca
1-8tRNA-Tyr(gta)[88253,88340]
ggggcggttcccggagcggcaaacgggcgggattgtaaatcccttggcat
cgcgtccttcgtaggttcgagtcctactcgccccacca
1-9tRNA-Ile(gat)[88347,88421]
gggagtatagctcagtggtagagcaaacggtcgataaccgttaggtcgct
ggttcaagcccagctactcccacca
1-10tRNA-Gly(tcc)[88428,88503]
gcgttattagtgcagtggtccagcatcccgtccttccaagtcggtgacat
cggttcgaatccgatataacgctcca
1-11tRNA-Glu(ttc)[88511,88586]
gctcctatcggctaaacggtcaggccgccagactttcaatctggaaatcc
gagttcgattctcggtgggagtgcca
1-13tRNA-Trp(cca)[88672,88748]
agggacttagcacaattggctagtgcagcggattccaaatccgcaggttc
tgggttcgaatcccagggtccctgcca
1-14tRNA-Gln(ttg)[88886,88961]
aggaggatggtgtaattggttagcacacggggctttgatctccggagtta
gggttcgagtccttatcttcctgcca
1-15tRNA-Ser(gga)[89077,89169]
ggtggaatggtcgagcggtttaagacaacaccttggaaaggtgtcggccc
cttcaaacggggtccgtaggttcgaatcctacttccaccgcca
1-16tRNA-Ser(gct)[89331,89422]
ggaagattaaccctaacggtaagggatctctttgctaaagagacagtagc
ccccaaaaggggtgtgtcagttcgagtctgacatcttcctcc
1-17tRNA-Ser(tga)[89423,89511]
ggagagcaggctgcatggtgcagactccggtttgaacccggtcccatcgt
agcgatacggtgacagttcgattctgttgctctcctcca
1-18tRNA-Asn(gtt)[89773,89861]
ggttctttgaccgagaggccgatggtagcaggctgttaacctgccgggag
caatccccgacgaaggttcgaatccttcaggaaccgcca
1-19tRNA-Asp(gtc)[89867,89942]
ggagatatagtttaaacggataaaatacacccctgtcacgggtatgttcc

gggttcgatgcccggtatctccgcca
1–20tRNA–Pro(tgg)[90066,90143]
cggagcgtagcgcagtctggtcagcgtgggtgccttggatgcactgggtc
gcaggttcgaatcctgccgctccgacca
1–21tRNA–Arg(acg)[90149,90223]
gcaggtatagttcaacggatagagcatcagtttacgaagctgagaatcgg
ggttcgaatccctgtatctgcacca
1–22tRNA–Ala(tgc)[90228,90303]
ggggctgtagtttatttggataaaatcgcaggcttgcaccttgcagaact
gagttcgattctcagtggctccacca
1–23tRNA–Leu(tag)[90339,90415]
gggcgattgatggaattggtatacgtgtcggatttagattccgaattttg
ggggttcgagtcccctgtcgcccacca
1–24tRNA–Val(tac)[90424,90497]
gcttccttagtttaatggtagaaccctgtctttacacggcagttgcggta
gttcgattctaccaggaagtacca
1–25tRNA–Phe(gaa)[90505,90580]
ggggttgtaactcagttggtagagtgcctgcctgaagagcagatggtcgg
tggttcgagtccacccggccccacca
>HM997020.1 Escherichia phage wV7, complete genome
1–1tRNA–Arg(tct)c[69195,69270]
cggggcatagctcagaaggaagagcaaggaccttctaagtcctaggtcgt
aggttcgatccctactgcctcgacca
1–2tRNA–His(gtg)c[69275,69350]
gtgaccgtagttcagttggtagaactcgagattgtgattctcgtagtcat
gggttcgactcccatcggtcacccca
1–3tRNA–Asn(gtt)c[69465,69539]
ggatgtgtagctcaatggcagagcgatcgcctgttaagcgattggttata
ggttcgaatcctatcacgtccgcca
1–4tRNA–Tyr(gta)c[69544,69630]
ggggagttatcccgtagaggtagcggtgtggactgtaaattcattgtcat
tgcgactcgggtggttcgactccaccactccccacca
1–5tRNA–Met(cat)c[69643,69717]
ggccctgtagctggaaggttcaagcaagcgactcataatcgccagatggt
ggttcaattccacccagggccacca
1–6tRNA–Thr(tgt)c[69719,69794]
gctgatttagctcagtaggtagagcaactcacttgtaatgagaaggtcgg
cggttcgattccgtcaatcagcacca
1–7tRNA–Ser(tga)c[69800,69889]
ggaggtgtggcagagtggttgaatgcaccggtcttgaaaaccggcagtcg
ctccggcgattcataggttcgaatcctatcacctccgcca
1–8tRNA–Pro(tgg)c[69891,69965]
ctccgtgtagctcagtttggtagagcgcctgatttgggatcaggaggtcc
aaggttcaaatccttgtatggagac

1-9tRNA-Gly(tcc)c[69976,70049]
gcggatatcgtataatggtattacctcagacttccaatctgatgatgtga
gttcgattctcattatccgctcca
1-10tRNA-Leu(taa)c[70055,70141]
gcgagaatggtcaaattggtaaaggcacagcacttaaaatgctgcggaat
gatttccttgtgggttcgagtcccacttctcgcacca
1-11tRNA-Gln(ttg)c[70142,70215]
tgggaattagccaagttggtaaggcatagcactttgactgctagatgcaa
aggttcgagtcctttattcccagc
>JN126049.1 Salmonella phage PhiSH19, complete genome
1-1tRNA-Met(cat)[137303,137378]
ggtcctgtagctcagtggttagagcagtcgactcataatcgattggtcat
tggttcaaacccaatcaggatcacca
1-2tRNA-Asn(gtt)[137806,137881]
gacgatgtagttcagtcggtagaacggtggactgttaatccatatgtcgc
aggttcaagtcctgccatcgtcgcca
1-3tRNA-Tyr(gta)[138497,138580]
gtgagtgtggcagagcggtcgaatgcaggagactgtaaatctccccgtaa
cagcgcggtggttcaaatccatccactcacacca
1-4tRNA-Ser(gct)[138657,138745]
ggaaggttgcccgagaggtttaagggactcgactgctaatcgagtggggc
ttttagcccccgaaggttcgaatccttcaccttccgcca
>JX536493.1 Enterobacteria phage HX01, complete genome
1-1tRNA-Met(cat)[31995,32069]
ggccctgtagctcaatgggagagctgtcagctcataactgataggtagct
ggatcgaaaccagccagggtcacca
1-2tRNA-Arg(tct)[32073,32149]
cgaggcatagctcaattgtatagagcaacggacttctaatccgtaggttg
aaggttagaatccttctgtctcgacca
>JN882287.1 Cronobacter phage vB_CsaM_GAP161, complete genome
1-1tRNA-Met(cat)[49480,49556]
ggcccttagcttaattggttagagcgggtgactcataatcgcgcggttg
ctggttcaagtccagcaggggccacca
1-2tRNA-Gly(tcc)[49562,49637]
gcaggtgtagttcaattggtagaatatctggtttccatccagattgttga
gggttcgagtcccttcacctgctcca
>JQ686190.1 Staphylococcus phage GH15, complete genome
1-1tRNA-Met(cat)c[15353,15424]
ggactcttagcttaaaggtaaagccaaccgctcataacggtttgactgta
ggttcgaatcctgcagagtcca
1-2tRNA-Trp(cca)c[37771,37842]
acacccttagtataattagtagtacaagggtctccaaaacccttagtctt
tgtgcaaatcaaagagggtgtg
1-3tRNA-Phe(gaa)c[37849,37921]

ggtttcttagctcagatggtagagcactagattgaagctctaggtgtcat
tggttcaaatccaatagaaacca
1−4tRNA−Asp(gtc)c[37927,38002]
tggctcattggtgtaactggttaacacactgccctgtcacggcagagagt
acgagttcgagtctcgtatgagtcgt
>JN371769.1 Synechococcus phage metaG−MbCM1, complete genome
1−1tRNA−Leu(taa)[17905,17989]
gggagtgtggcggaataggtagacgcaccagacttaaaatctgttgagca
tgtgctcgtgggagttcaagtctccctactcctac
1−2tRNA−Arg(tct)[159596,159670]
tgggtcagtagcacagcggatagtgcaactgccttctaagcagtcggtcg
caggttcaaatcctgcctgacccgt
>JN377894.1 Aeromonas phage Aes508, complete genome
1−1tRNA−Arg(tct)c[60935,61011]
ggtctcttagctcagtcggatagagcagcggccttctaagccgcgggtca
ttggttcgaatccaatagggactgcca
1−2tRNA−Met(cat)c[61580,61655]
ggccctctgcacagtctgtgaatgtgtccagctcataactggggtaagcc
gggttagattcccgggagggccacca
1−3tRNA−Met(cat)c[61821,61897]
tgcgatgtagaggagaggtcgtcctcgtcgggctcatatcccgaaaatca
gcggttcgaatccgttcatcgcctcca
1−4tRNA−Asp(gtc)c[61910,61983]
ggagccatagtttatttggttaaaatagtcccctgtcacgggtcagcacc
gagttcgatcctcggtggctccgc
1−5tRNA−Thr(tgt)c[62384,62459]
gccggtttagctcatctggtagagcacagcctttgtaacgctggggtggt
ccgttcgagtcggacaatcggcacca
1−6tRNA−Pro(tgg)c[63093,63167]
ctcagcgtaggcaagtggtatgtcgttccgtttggggcggaattttcggg
tgttcgattcaccccgctgagacca
1−7tRNA−Gly(tcc)c[63413,63488]
gcgatattagctcagttggtagagcttctgatttccaatcagaatgtcat
cggttcgagaccgatatatcgctcca
1−8tRNA−His(gtg)c[63500,63574]
gtgaccttagtagtaatggtagctatcctggatgtgacccagggagtacg
ggttcgaaccccgtaggtcacccca
1−9tRNA−Tyr(gta)c[64544,64634]
ggatactcggctacgatggcggtgtagcggcagactgtaaatctgttccc
tcagggcaaacaagatggttcgaatccatcagtatccacca
1−10tRNA−Asn(gtt)c[64648,64730]
ggtgatgtagcataacggccatgcggccggctgttaaccggtgaggaaac
tctacgttggttcgactccaaccatcaccgcca
1−11tRNA−Ser(tga)c[64899,64992]

ggaagattggcccgagttggtttaaggcaccggtcttgaaaaccggcgat
cgtaggaatacggtccatccgttcgaatcggatatcttcctcca
1-12tRNA-Leu(taa)c[65184,65269]
gcaagtatggtggaatttggtatacactggagacttaaaatcttccgcct
tcgggattgtgggttcgagtcccactacttgcacca
>GU988610.2 Pseudomonas phage JG004, complete genome
1-1tRNA-Gln(ttg)[22657,22730]
tgccgcttcgttcaatggtaggacgccagactttgaatctggagatgatg
gttcgatcccatcagtggctgcca
1-2tRNA-Arg(tct)[23036,23110]
gctcgtatagtgtaatggatgcacaacggccttctaagccgtaaggtcta
ggttcgagtcctagtatgagcgcca
1-3tRNA-Lys(ttt)[23118,23194]
tggacggaagctaaagtggatcagcatctggcttttaaccagacttatag
tgagttcgagtctcacccgttcaacca
1-4tRNA-Leu(tag)[23469,23553]
ggccctgtggtggaattggtatacacatcagtcttagaaactgacgccga
gaggattgagggttcaagtccctccggggccacca
1-5tRNA-Ile(gat)[23764,23839]
ggccagatagctcaattggtagagcccccgaccgataatcgggtggttga
aggttcaagtccttctctggccacca
1-6tRNA-Asp(gtc)[23849,23927]
ggcccattagctcagtctggactagagcaagcccctgtctagggcaaggt
cgccggttcgaatccggcatgggtcgcca
1-7tRNA-Met(cat)[24028,24102]
agcgttaagactggctggcgctgtcactcggctcataaccgaatacaagt
ggttcgattccacctctcgcttcca
1-8tRNA-Cys(gca)[24337,24412]
cccgcgttggccgagaggtttaggcggcggattgcaaatccgtctcacat
cggttcaaatccgatacgcggctcca
1-9tRNA-Asn(gtt)[24423,24498]
tgggatgtagctcagttggtagagcagggagctgttaactctcaggtcgc
aggttcgagccctgccgtcccagcca
1-10tRNA-Pro(tgg)[24562,24639]
ctcctcgtagctcagtctggttagagtgtcggatttggaatccgaaggtc
gaaggttcaaatccttccggggtgacca
1-11tRNA-Gly(tcc)[24646,24721]
gcgggtatagctcagttggtagagcgtctgccttccaagcagttcgtcgt
cggttcgagtccgtctatccgctcca
1-12tRNA-Phe(gaa)[24728,24804]
gggactgtagctcagaaggttagagcggtggattgaaatcccacaggtcg
ggcgttcgattcgccccggtcccacca
1-13tRNA-Glu(ttc)[24811,24886]
gcagttatagattaattggttaaatcgccagactttcaatctggtgttcc

gggttcgatccccggtaactgctcca
1−14tRNA−His(gtg)[24947,25021]
gtggagattgtgtagcccggatgctgcctttcggttgtgacccgattgtt
ggggttcgagtccccgcctccaacc
1−15tRNA−Thr(tgt)[25208,25282]
gccctttaagcatttatggtgatgcaccggctttgtaacccggcgaattc
tgttcaagtcaggaatggggcacca
>JN371768.1 Synechococcus phage S−MbCM6, complete genome
1−1tRNA−Leu(taa)[17220,17306]
tgggagtgtggcggaatcggtagacgcaccagacttaaaatctgttgaga
attaatctcgtgggagttcaagtctccctactcccat
1−2tRNA−Thr(tgt)[19061,19133]
gcctccgtagctcagtggtagagcagggcttttgtaaagctcaggtcgca
agttcaaatcttgtcagaggctc
1−3tRNA−Asn(gtt)[19138,19210]
tcctctatagctcagttggtagagcaggtgactgttaatcaccctgtccc
tggttcgagtccaggtggaggag
1−4tRNA−Arg(tct)[158217,158291]
tgggtcagtagctcagcggatagagccacgcacttctaatgcgttggtcg
caggttcgattcctgcctgactcgt
>JN673056.1 Escherichia phage PhaxI, complete genome
1−1tRNA−Met(cat)[136135,136211]
gggtctgtagtttaataggttagaacaacggactcataatccgtcaggtc
tcagttcaaatctgggtggacccacca
1−2tRNA−Asn(gtt)[136693,136768]
gacgatgtagttcagtcggtagaacggtggactgttaatccatatgtcgc
aggttcaagtcctgccatcgtcgcca
1−3tRNA−Ser(gct)[137453,137541]
ggaaggttgcccgagaggtttaagggactcgactgctaatcgagtggggc
ttttagcccccgaaggttcgaatccttcaccttccgcca
>JQ362498.1 Sphingomonas phage PAU, complete genome
1−1tRNA−Asn(gtt)[1273,1347]
tatcttgtagctcagctggttagagcaagtgactgttaatcactaggtcg
atggttcgaatccatccaaggtagc
1−2tRNA−Trp(cca)[1352,1425]
agggatatagctcagctggtagagcaatggtctccaaaaccatcggtcgg
tagttcgaatctatctgtccctgc
1−3tRNA−Cys(gca)[1431,1508]
gcacatatggctgagtcaggtcaaaagcaacggtctgcaaaaccgtacaa
ccattggttcgaatccaattatgtgctc
1−4tRNA−Met(cat)[1518,1592]
tgcggggtagagcagatggagtgctcgcgggtctcataagcccgaggtca
tgggttcgagtcccatccctgctac
1−5tRNA−Met(cat)[1598,1672]

gctcctttagctcagttggtcagagcaacagactcataatctgaaggtca
taggttcaagtcctgtagggagcac
1-6tRNA-Met(cat)[1681,1754]
agctcttctagctcaattggtagagtgatagattcattatctattggata
tgggttcgaatcccatgagagcgc
>JN202312.1 Enterobacteria phage ime09, complete genome
1-1tRNA-Arg(tct)c[69044,69119]
cgaggcatagctcagaaggaagagcaaggaccttctaagtcctaggtcgt
aggttcgatccctactgcctcgacca
1-2tRNA-Asn(gtt)c[69124,69198]
ggatgtgtagctcaatggcagagcgatcgcctgttaagcgattggttata
ggttcgaatcctatcacgtccgcca
1-3tRNA-Tyr(gta)c[69203,69289]
ggggagttatcccgtagaggtagcggtgtggactgtaaatccattgtcat
tgcgactcgggtggttcgactccatcactccccacca
1-4tRNA-Met(cat)c[69303,69377]
ggccctgtagctggaaggttcaagcaagcgactcataatcgccagatggt
ggttcaattccacccagggccacca
1-5tRNA-Thr(tgt)c[69379,69454]
gctgatttagctcagtaggtagagcaactcacttgtaatgagaaggtcgg
cggttcgattccgtcaatcagcacca
1-6tRNA-Ser(tga)c[69460,69549]
ggaggcgtggcagagtggtttaatgcaccggtcttgaaaaccggcagtcg
ctccggcgactcataggttcaaatcctatcgcctccgcca
1-7tRNA-Pro(tgg)c[69551,69625]
ctccgtgtagctcagtttggtagagcgcctgatttgggatcaggaggtcc
aaggttcaaatccttgtatggagac
1-8tRNA-Gly(tcc)c[69636,69709]
gcggatatcgtataatggtattacctcagacttccaatctgatgatgtga
gttcgattctcattatccgctcca
1-9tRNA-Leu(taa)c[69715,69801]
gcgagaatggccaaattggtaaaggcacagcacttaaaatgctgcggaat
gatttccttgtgggttcgagtcccacttctcgcacca
1-10tRNA-Gln(ttg)c[69802,69875]
tgggaattagccaagttggtaaggcactggattttgattccaggatgcaa
aggttcgagtcctttattcccagc
>JQ245707.1 Cyanophage S-TIM5, complete genome
1-1tRNA-Pro(tgg)[15402,15476]
cgggaattagcgcagtttggtagcgcgcctgctttgggagcaggatgcca
caggttcaaatcctgtattcccgac
1-2tRNA-Arg(tct)[17047,17122]
tggttcagtagctcagttggatagagcaactgccttctaagcagtcggtc
gctggttcgagtccagcctgaatcgt
1-3tRNA-Ile(tat)[23166,23237]

gggactgtcgcctaaaggtaaaggccctctgcttataacggagtgatctg
ggttcaagtcccagcagtccta
1−4tRNA−Met(cat)[23240,23314]
gctcctttagcaatctggtgaatgcagcgaactcataattcgcctaaggt
gtgttcgatccacacaaggagcacc
1−5tRNA−Leu(taa)[23322,23405]
gcgagtatggcggaatcggtagacgcaccagacttaaaatctgttgggca
ttagcccgtgggagttcaagtctccctactcgca
1−6tRNA−Asn(gtt)[26444,26515]
tcccaagtagctcagtggcagagccgccgactgttaatcggctggtcgct
ggttcaaatccagccttgggag
1−7tRNA−Thr(tgt)[26519,26591]
gcctacctagctcagctggtagagcagggcttttgtaaagctcaggtcgt
cggttcaagtccgtcggtaggct
1−8tRNA−Leu(tag)[26911,26996]
tgcgcaagtggcggaattggtagacgcccaggttttaggtacctgtgccc
tagtggcgtgaaggttcaagtcctttcttgcgcatc
1−9tRNA−Gly(tcc)[27617,27689]
gcggagttagttcagcggtagaacgctatccttccaagttagatgtcgtc
ggttcgattccgatactccgctc
1−10tRNA−Val(tac)[28879,28952]
tgcaagattagctcagcggtagagcatctcgtttacaccgaggcggtcgg
cggttcaatcccgtcatcttgcat
>HE815464.1 Campylobacter phage CP21 complete sequence
1−1tRNA−Thr(tgt)c[166676,166751]
gcctgattagctcaactggtagagcattcgccttgtaagcgaaaggttga
gagttcgagtctctcattaggctcca
1−2tRNA−Pro(tgg)c[167466,167543]
cggggtgtagcgcagtctggttagcgcacttggtttgggaccaaggggcc
gaaggttcgaatcctttcaccccgacca
>HE983845.1 Pseudomonas phage vB_PaeM_C2−10_Ab1 complete genome
1−1tRNA−Gln(ttg)[21995,22068]
tgccgcttcgttcaatggtaggacgccagactttgaatctggagatgatg
gttcgatcccatcagtggctgcca
1−2tRNA−Arg(tct)[22274,22349]
gctcatatagtgtaatggacagcacaacggtcttctaagccgtaaggtct
aggttcgaatcctagtatgagcgcca
1−3tRNA−Lys(ttt)[22357,22433]
tggacggaagctaaagtggatcagcatctggcttttaaccagacttatag
tgagttcgagtctcacccgttcaacca
1−4tRNA−Leu(tag)[22695,22779]
ggccctgtggtggaattggtatacacatcagtcttagaaactgacgccga
gaggattgagggttcaagtccctccggggccacca
1−5tRNA−Ile(gat)[22986,23061]

agccggatagctcaattggtagagcacccgaccgataatcggatggttga
aggttcaagtccttctctggctacca
1-6tRNA-Asp(gtc)[23071,23149]
ggcccattagctcagtctggactagagcaagcccctgtctagggcaaggt
cgtcggttcgaatccgacatgggtcgcca
1-7tRNA-Met(cat)[23250,23324]
agcgttaagactagctggagctgtcactcggctcataaccgaatacaagt
ggttcgattccacctctcgcttcca
1-8tRNA-Cys(gca)[23561,23636]
ccctcgttggccgagaggatcaggcagcagattgcaaatctgccatacat
cggttcaaatccgatacgaggctcca
1-9tRNA-Asn(gtt)[23646,23722]
tccgtacggcccatcaaggcgatggggctcggctgttaaccgaaacgcgc
taggttcgattcctaggtacggagcca
1-10tRNA-Pro(tgg)[23785,23862]
ctcctcgtagctcagtctggttagagtgtcggatttggaatccgaaggtc
gaaggttcaaatccttccggggtgacca
1-11tRNA-Gly(tcc)[23870,23946]
gcgggtttcgtatagtctggttattattcttggcttccacccaagagact
agggttcgaatccctaagcccgcacca
1-12tRNA-Phe(gaa)[24009,24084]
gcgtctgaagctaactaggtagaagcaccgggttgaaattccggaggact
tggatcgttaccaagcgggcgcacca
1-13tRNA-Glu(ttc)[24091,24166]
gcagttatagattaaatggataaatcgccagactttcaatctggtgttcc
gggttcgatccccggtaactgctcca
1-14tRNA-His(gtg)[24227,24301]
gtggagattgtgtagcccggatgctgcctttcggttgtgacccgattgtt
ggggttcgagtccccgcctccaacc
1-15tRNA-Thr(tgt)[24488,24562]
gccctttaagcatttatggtgatgcaccggctttgtaacccggcgaattc
tgttcaagtcaggaatggggcacca
>HE600015.1 Dickeya phage vB_DsoM_LIMEstone1 complete genome
1-1tRNA-Met(cat)[129994,130070]
gatggtgtagttcagttggttagaacgtgcgactcataatcgctttgtca
ctggttcaagtcctgccgccatcgcca
>JQ177062.1 Bdellovibrio phage phi1422, complete genome
1-1tRNA-Pro(tgg)[15181,15256]
cggggattggagaagtttggagttctcgcgtgttttgggaacatgagatc
acaggttcaaatcctgtatccccgac
>JQ031132.1 Enterobacteria phage vB_EcoM-FV3, complete genome
1-1tRNA-Pro(tgg)c[61201,61277]
ctccgcgtagctcagcttggtagagcgcctgatttgggatcaggaggtcg
agtgttcgaatcactccgtggagacca

1–2tRNA–Met(cat)c[61289,61363]
ggccctgtagctggaaggttcaagcaagcgactcataatcgccagacggt
ggttcaattccacccagggccacca
1–3tRNA–Thr(tgt)c[61365,61440]
gctgatgtagcacaattggtagtgcaattgatttgtaatcaataggttgc
aggttcgagtcctgctatcagcacca
1–5tRNA–Ser(tga)c[61643,61733]
tgagggtagggagcaatggtgctcaagcggtcttgaaaaccgtcccgttg
aggatgactcgatgatggttcgattccattaccctcagcca
1–6tRNA–Tyr(gta)c[61848,61934]
ggggagttatcccgtagaggtagcggtgtagactgtaaatctattgtcat
tgcgactcgggtggttcgactccaccactccccacca
1–7tRNA–Arg(tct)c[61939,62013]
gccctgttagcttagtggataaagcagcggccttctaagccgttgacact
ggttcgagtccagtacggggtgcca
>JQ513383.1 Enterobacteria phage vB_KleM–RaK2, complete genome
1–1tRNA–Leu(taa)[176487,176574]
ggccgtatgtccaaattggtaaaggagacagacttaaaatctgttggtga
tattcaccttctcgattcgagttcgagtgcggctacca
1–2tRNA–Gly(tcc)[176941,177016]
gcggttatcgtataaaggctattacctcggttttccaaaccgatgatgag
ggttcgacgtccctctaaccgctcca
1–3tRNA–Arg(tct)[178303,178376]
gtccccttagttcagttggatagaacaactgccttctaagcagtaggtca
ctggttcgagtccagtaggggata
1–4tRNA–Ser(gct)[178529,178617]
tagtgagttggatgagtggttgaaatcaccaccctgctaaggtggcatat
gcgaaagcgtatcgtgggttcgaatcccacactcactgt
1–5tRNA–Ser(tga)[178680,178767]
ggaaggatggccgagtggtttaaggcactggtcttgaaaaccagcaatcc
gaaagggttctagagttcaaatctctatccttccacca
1–6tRNA–Met(cat)[178885,178962]
ggccctttactctcaagtggttagagaatccgactcataatcggtaggct
ctgagttcgaatctcagaagggccacca
1–7tRNA–Asn(gtt)[179699,179772]
gactctgtagcacaatcggtggtgcgattgactgttaatcaattggttgg
ggattcgagttcctccagagtcgc
1–8tRNA–Thr(tgt)[182045,182119]
accgccttagaacaattggtagtgtaacttgatttgtaatcagtaggtta
tatgttcgagtcagataggtggcac
>JX123262.1 Aeromonas phage CC2, complete genome
1–1tRNA–Leu(taa)[197457,197549]
gcgtgagtggcgaagttggttgaaacgcatcggacttaaaatccgatact
gcataatggcacatcgtgggttcgattcccacctcacgcacca

1-2tRNA-Arg(tct)[198613,198689]
gcgctgttagctcagttggatagagcaacgttcttctaaagcgtgggtca
ctggttcgaatccagtacggcgcacca
1-3tRNA-Leu(tag)[198690,198773]
cgggatgtggtgaaattggcagacacactagatttaggttctagcgccat
aggcgtgacggttcgagtccgtccatcccgacca
1-4tRNA-Gly(tcc)[198775,198850]
gcgggtaaggtgtttatggatacatgcaagccttccaagtttgagtagac
cggatcgttaccggctacccgctcca
1-5tRNA-Met(cat)[198935,199011]
ggccccttagctcagttggttagagcagccgactcataatcggttggtca
ctggttcgagtccagtaggggccacca
1-6tRNA-Met(cat)[199377,199454]
agcggggtggagaagtcaggagttctcaccagactcattatctggaaatc
ggtggttcaaatccacctcccgcttcca
1-7tRNA-Asn(gtt)[199456,199532]
tggcgtgtaactcagtaggttagagtggcggactgttaatccgtatgtcg
tgggttcgaatcccacctcgccagcca
1-8tRNA-Tyr(gta)[199538,199628]
ggatgttcggctacgatggcggtgtagcggcggactgtaaatccgttccc
tctgggtaaacaagttggttcgaatccaacagcatccacca
1-9tRNA-Pro(tgg)[199782,199857]
cggtgattagcgcagttggtagcgcgtctggtttgggaccagagggtcac
aggttcgagtcctgtatcaccgacca
1-10tRNA-Thr(tgt)[200134,200209]
gccgggttagctcagttgatagagcgacggtcttgtaatccgtaggtcgt
gggtttgagtcccacactcggctcca
1-11tRNA-Trp(cca)[200764,200838]
agggtgttagtatagtggtattatgccggattccaaatccgtgcgactgg
ggttcgaatcctcaacgccctgcca
>JX878671.1 Staphylococcus phage JD007, complete genome
1-1tRNA-Asp(gtc)[50343,50418]
tggctcattggtgtaactggttaacacactgccctgtcacggcagagagt
acgagttcgagtctcgtatgggtcgt
1-2tRNA-Phe(gaa)[50424,50496]
ggtttcttagctcagatggtagagcactagattgaagctctaggtgtcat
tggttcaaatccaatagaaacca
1-3tRNA-Trp(cca)[50503,50574]
acacccttagtataattagtagtacaagggtctccaaaacccttagtctt
tgtgcaaatcaaagagggtgtg
1-4tRNA-Met(cat)[72975,73046]
ggactcttagcttaaaggtaaagccaaccgctcataacggtttgactgta
ggttcgagtcctgcagagtcca
>HQ832595.1 Pseudomonas phage PaP1, complete genome

1-1tRNA-Gln(ttg)[20964,21037]
tgccgcttcgttcaatggtaggacgccagactttgaatctggagatgatg
gttcgatcccatcagcggctgcca
1-2tRNA-Arg(tct)[21243,21317]
gctcgtatagtgtaacggatgcacaacggtcttctaagccgtaaggtcta
ggttcgaatcctagtatgagcgcca
1-3tRNA-Lys(ttt)[21325,21401]
tggacggaagctaaagtggatcagcatctggcttttaaccagacttatag
tgagttcgagtctcacccgttcaacca
1-4tRNA-Leu(tag)[21665,21749]
ggccctgtggtggaattggtatacacatcagtcttagaaactgacgccga
gaggattgagggttcaagtccctccggggccacca
1-5tRNA-Ile(gat)[21963,22038]
agccggatagctcaattggtagagcacccgaccgataatcggatggttga
aggttcaagtccttctctggctacca
1-6tRNA-Asp(gtc)[22048,22126]
ggcccattagctcagtctggactagagcaagcccctgtctagggcaaggt
cgtcggttcgaatccgacatgggtcgcca
1-7tRNA-Met(cat)[22227,22301]
agcgttaagactggctggcgctgtcactcggctcataaccgaatacaagt
ggttcgattccacctctcgcttcca
1-8tRNA-Cys(gca)[22537,22612]
cccgcgttggccgagaggtttaggcggcggattgcaaatccgtctcatat
cggttcaaatccgatacgcggctcca
1-9tRNA-Asn(gtt)[22623,22698]
tgggatgtagctcagttggtagagcagggagctgttaactctcaggtcgc
aggttcgagccctgccgtcccagcca
1-10tRNA-Pro(tgg)[22762,22839]
ctcctcgtagctcagtctggttagagtgtctgatttggaatcagaaggtc
gaaggttcaaatccttccggggtgacca
1-11tRNA-Gly(tcc)[22846,22921]
gcgggtatagctcagttggtagagcgtctgccttccaagcagttcgtcgt
cggttcgagtccgtctatccgctcca
1-12tRNA-Phe(gaa)[22928,23004]
gggactgtagctcagaaggttagagcggtggattgaaatcccacaggtcg
ggcgttcgattcgccccggtcccacca
1-13tRNA-Glu(ttc)[23011,23086]
gcagttatagattaaatggttaaatcgccagactttcaatctggtgttcc
gggttcgatccccggtaactgctcca
1-14tRNA-His(gtg)[23147,23221]
gtggagattgtgtagcccggatgctgcctttcggttgtgacccgattgtt
ggggttcgagtccccgcctccaacc
1-15tRNA-Thr(tgt)[23408,23482]
gcccttaagcatttatggtgatgcaccggctttgtaacccggcgaattc

tgttcaagtcaggaatggggcacca
>JF974288.1 Synechococcus phage S-RIM8 A.HR1, complete genome
1-1tRNA-Gly(tcc)[13713,13787]
gcggatgtaattcagtggtagaatgtcagccttccaagctgaacgtcagg
ggttcgagtccccttatccgctcca
1-2tRNA-Val(tac)[13788,13859]
gggcgattaactcagcggtagagtgcctcgtttacaccgagtatgtcggg
ggttcgaatccctcatcgccca
1-3tRNA-Leu(taa)[18355,18440]
tgggacggtggcggaatcggtagacgcaccagacttaaaatctgttgggc
aatagcccgtgagagttcaagtctctctcgtcctat
1-4tRNA-Thr(tgt)[19096,19172]
gcctccgtagctcagctggatagagcaacggttttgtaaaccgtaggtcg
tcggttcaagtccgaccgtgggctcca
1-5tRNA-Ala(tgc)[19173,19245]
ggggaattagctcagttggtagagcgcctgctttgcaagcaggatgtcag
cggttcgagtccgctattctcca
1-6tRNA-Asn(gtt)[19270,19344]
ttcctctatagctcagtcggtagagcgtttgactgttaatcaaaatgtcc
ctggttcgagcccaggtggaggagt
1-7tRNA-Arg(tct)[156607,156682]
tgggtcagtagctcagttggatagagcaactgccttctaagcagtcggtc
acaggttcgagtcctgtctgacccgt
1-8tRNA-Pro(tgg)[157690,157764]
cggggtgtagcgcagcttggtagcgcggttgctttgggagcaataggtcg
caggttcgaatcctgtcaccccgac
>HE956709.1 Yersinia phage phiR1-RT complete genome
1-1tRNA-Gly(tcc)c[72343,72418]
gcagagttcgtatagtggcttaatactattggtttccgcccagtaaacat
cggttcgaatccgatactctgctcca
1-2tRNA-Trp(cca)c[72671,72745]
aggtctttagtatagtggcgattatgctaggctccaaacctagtgacggg
tgttcgattcacccaaggcctgcca
1-3tRNA-Arg(tct)c[74027,74103]
gtcctcatagctcaacaggacagagcaacggtcttctaaaccgtaggttg
ctggttcgattccagctggggacacca
1-4tRNA-Met(cat)c[74902,74978]
ggcccctttagctcagatggttagagcagtcgactcataatcgattggtca
ctggttcaaatccagtaagggccacca
>JX181829.1 Salmonella phage SKML-39, complete genome
1-2tRNA-Ser(gct)c[145196,145284]
ggaaggttgcccgagaggtttaagggactcgactgctaatcgagtggggc
ttttagcccccgaaggttcgaatccttcaccttccgcca
1-3tRNA-Asn(gtt)c[145768,145843]

gatggtgtagttcagtcggtagaacggcggtctgttaaatcgtatgtcgc
aggttcaaatcctgacaccatcgcca
1-4tRNA-Arg(tct)c[147533,147608]
tgtttccctagctcaattggatagagcataggatttctaatccttgtgtt
gcaggttcgaatcctgcgggaaacgt
1-5tRNA-Met(cat)c[147620,147696]
ggtcctgtagctcagtcggttagagcagtcgactcataatcgattggtca
ttggttcaaacccaatcaggatcacca
>HQ918180.1 Klebsiella phage KP27, complete genome
1-1tRNA-Met(cat)[49034,49110]
ggccccttagctcaataggtagagctaatcactcataatggttaggttcc
cggttcaagtcacgggagggggccacca
>JX238501.3 Bacillus phage phiAGATE, complete genome
1-1tRNA-Met(cat)c[119285,119360]
tgggtctctagcttagtggtaaagctgccagctcataactggtaagagag
ggggttcgaatcccttgggacccatc
1-2tRNA-Glu(ttc)c[120905,120976]
gtggtcttagtttaatgatagaacagtctgttttcgccagacaggtacgg
gttcaactcccttagaccaacc
1-3tRNA-Phe(gaa)c[122052,122126]
tggactggtagctcagtcggtagagcagggggctgaaaatccctgtgtcg
gaggttcgaacccttctcagtccat
1-4tRNA-Asn(gtt)c[122474,122547]
ttgtcgtataactcaatggtagagtgcttgactgttaatcaagaagttgt
aggttcgagtcctactacgacagt
>JX878496.1 Serratia phage phiMAM1, complete genome
1-1tRNA-Pro(agg)c[19252,19325]
cgggaagtagtttagcggtaaaacgcaagcacagggtgcttgagtcctag
gttcgaatcctagactcccggcca
1-2tRNA-Met(cat)c[19329,19404]
agtcccttagctcagtggttagagcatgcgactcataatcgcttggtcgt
tggttcaaagccaacagggactacca
1-3tRNA-Cys(aca)c[21050,21141]
tggccgggtagctcaattggtagagtggcgcgcgtacagcctgaggcgac
attagcgcgatgttgtggcttcgagaaccaccccggccaccc
>JQ015307.1 Pectobacterium phage phiTE, complete genome
1-1tRNA-Cys(gca)[105433,105507]
gcaacgttgccggagtatggtaacggctctcgctgcaaacgagatgttca
ctggttcaaatccagtacgttgctc
1-2tRNA-Leu(caa)[105511,105590]
gcccctctggcccaacaaaaggtagaggctctgctttcaaacggcaggag
ttcccggttcgaccccggggaggggcacca
1-3tRNA-Tyr(gta)[106329,106410]
ggggacgtggcagagcggtcgaatgcatctgactgtaaatcgggtatccc

acacggtggttcgaatccacccggctccacca
>HQ331142.1 Salmonella phage S16, complete genome
1–1tRNA–Arg(tct)c[61310,61386]
cgaggcatagcttaatagtatagagcaacggtcttctaaaccgtaggttg
aaggttagaatccttctgtctcgacca
1–2tRNA–Gln(ttg)c[62011,62086]
tgggaattagccaagttggtaaggcaccggattttgattccgggatgcac
tggttcgagcccagtattcccagcca
1–3tRNA–Met(cat)c[62093,62167]
ggcccttttagctcaatgggagagctgtcagctcataactgataggtagct
ggatcgaaaccagcaagggccacca
>KC465899.1 Pelagibacter phage HTVC008M, complete genome
1–1tRNA–Gly(acc)[91140,91213]
gcgggtgtggtatagaagtattacgccagtttaccaaactggaaatgcag
gagcgttacctgccatccgctcca
>HQ634157.1 Vibrio phage 11895–B1 genomic sequence
1–1tRNA–Tyr(gta)c[103393,103476]
gggttgttgggtgaattggttaaaccagcagactgtaaatctgccgcctc
tggcatccaagttcgagtcttgggcaacccacca
1–2tRNA–Pro(tgg)c[103479,103555]
ctccgtatagctcagtttggtagagcgcatcatttgggatgatgaggtct
gatgttcaaatcatcatgcggagacca
>HQ316583.1 Cyanophage S–SSM4 genomic sequence
1–1tRNA–Val(tac)[1514,1585]
gggagattagctcagcggtagagctattcgtttacaccgaatcggtcatt
ggttcaagtccaatatctccca
1–2tRNA–Leu(taa)[6709,6790]
gggagcatggcggaatcggtagacgcatcggacttaaaatccgctgaggt
aactcgtgggggttcaagtccccctgctccta
1–3tRNA–Arg(tct)[150850,150924]
tggtccagtagctcagcggaaagagcaactgccttctaagcagttggcca
taggttcaaatcctatctggatcgt
>JQ797329.1 Listeria phage vB_LmoM_AG20, complete genome
1–1tRNA–Met(cat)c[25833,25906]
ttgtcccgtagctagaaggtcgagcaaggagctcataactcctcggtttg
ggttcgattcccaacggggcaatc
1–2tRNA–Pro(tgg)c[26799,26873]
cagggtgtagctcagtttggttagagtacccgctttggagacgggaagtc
gtaggttcgaatcctaccaccttga
1–3tRNA–Arg(tct)c[27991,28061]
gtccttatggtgtagcggatgcacaagggatttctactcccttagcgcgg
gttcgaatcctgctgaggact
1–4tRNA–Gly(tcc)c[28319,28389]
gcgggtatagtataagggtagtacccaaggtttccaaccatgtagtgggg

gttcgaatccccctacccgct
1-5tRNA-Asn(gtt)c[28458,28531]
gtgtccttaactcagaggtcagagtgccgtcctgttaagtcggaagtcgc
tggttcaaatccagcaggatacgc
1-6tRNA-Ser(tga)c[28541,28632]
ggaaggttggtagagcttggtaatacgctagtcttgaaaactagtttacc
cttggaatacagggtacaagggttcgaatcccttaccttcct
1-7tRNA-Phe(gaa)c[28645,28716]
gtagtcctagctgagatggattagcgcttgcttgaaaagcaagcgaggca
ggttcgatacctgcggactcca
1-8tRNA-Lys(ttt)c[28722,28793]
ggagttatggtgaaatggctatcactgcgggtttttaccccgttattcta
ggttcgaatcctagtggctcca
1-9tRNA-Trp(cca)c[28918,28991]
taggggtgtagtttatctggtaaaatattggtttccaactccaatgaggt
gggttcaagtcctactatccctgt
1-10tRNA-Gln(ttg)c[28993,29065]
tggtctgtaaccaagcggtaaggcaacggattttgattccgtgatacgtt
ggttcgagtccaactagaccagc
1-11tRNA-Thr(tgt)c[29084,29155]
gcttgtatagttcaattggtagaacagtggttttgtaagcctcagacgtg
ggttcaagtcctactacaagca
1-12tRNA-Tyr(gta)c[29237,29318]
gtgccattcgcatagtggcaattgcaggggactgtaactcccctcccttc
ggggttccaaggttcgagtccttgatggcaca
1-13tRNA-Leu(tag)c[29639,29723]
tgccgagatggtggaactggtatacacggtagacttagaatctattgtcc
caaggatatgtgggttcgaatcccactctcggtat
1-14tRNA-Asp(gtc)c[30189,30261]
gtgcgtatgatataatggctattatactcgactgtctatcgagaaatagg
ggttcaattccccttacgtgcgc
1-15tRNA-Ile(gat)c[30365,30437]
gccagcatagcttagtaggcaaagcaaccgaccgataatcggtagtcctt
ggttcaattccaagtgttggtac
1-16tRNA-Ser(gct)c[30614,30700]
ggagagttgtcagagaggcttaatgatacgggttgctaactcgttgtact
agtaatagtaccaagggttcgaatcccttactctcct
1-17tRNA-Cys(gca)c[30779,30849]
gcgggtataaccaactggaaaggtagtagactgcaaatctacgtatatgg
gttcaattcccattacccgct
>HQ634193.1 Cyanophage P-RSM6 genomic sequence
1-1tRNA-Leu(taa)c[38808,38883]
tgggagtgtagcccaatcggcagaggcagcagacttaaaatctgcacagt
gtgggttcgaatcccaccactcctat

1−2tRNA−Arg(tct)c[38885,38960]
tgactcagtagctcagtggacaagagcatcgctcttctaaagcgttggtc
gtaggttcgaatcctacctgagtcgt
1−3tRNA−Ile(tat)c[38964,39037]
gggactatcgcctattggttaaggctcactgcttataacggtgcgaactg
ggttcgattcccagtagtcctacc
>JX094431.1 Bacillus cereus bacteriophage vB_BceM_Bc431v3, complete genome
1−1tRNA−Met(cat)c[155868,155942]
taggattatagctcagtggtagagcgtgggtctcataagcccgaggtcga
tggttcgatcccatctattcctatc
1−2tRNA−Leu(tag)c[156094,156178]
tgtcgaagtgttggaactggtatacatgcggcacttaggatgccgtgcct
tcgggattgtgggttcgactcccaccttcgacatc
1−3tRNA−Ile(gat)c[156186,156262]
actagtgtagctcagtcaggtagagcagtgtcttgataaggcattggtca
caggttcgaatcctgtcactagtacca
1−4tRNA−Leu(taa)c[156266,156352]
atcggagtgttggaattggtagacatatggcacttaaaatgctatgtccg
tatggacgtaagggttcgagtcccttctccgatacca
1−5tRNA−Tyr(gta)c[156356,156438]
gggcgtgcaatcattggagagataagctgactgtaaatcagtggtcattg
actgtggaggttcgaatcctcccattcccacca
1−6tRNA−Phe(gaa)c[156445,156519]
ggacagatagctgagacggattagcgattggctgaaaaccaatagaggtt
ggatcgttaccaactctgtccacca
1−7tRNA−Pro(tgg)c[156620,156692]
acgggtgtaggctagaggtcagtcactgcgtttggggcgcagatcacgtt
ggttcgatcccaaccactcgtac
1−8tRNA−His(gtg)c[156701,156774]
cagtgtgtggtgaagtggcttaacacggacgactgtggatcgtctattcg
ctggttcgaatccagtcatgctga
1−9tRNA−Gln(ttg)c[156851,156927]
tttcggagtggacaaactggtaaagtcgcttggctttgacccaagagcgt
gaaggttcgaccccttcctccgaagtc
1−10tRNA−Ser(tga)c[157005,157094]
cggggaatactcaagtggataaaagaggttagtcttgaaaactaataggc
gtgtgaaagcgtgcgggggttcgaatccctcttcctcggc
1−11tRNA−Arg(tct)c[157152,157227]
taccccttttagccaagtggactaaggcaacgggcttctatcccgtggatc
gtgggttcgattcctacagggggtgt
1−12tRNA−Ile(tat)c[157347,157420]
atccctttagccaagcggttaaggcagtaggattatgtcctacgtatcgg
gagttcgaacctcccaaggggcgc
1−13tRNA−Asp(gtc)c[157422,157495]

tggggatatagtgtagtggttaacacgcacggctgtctaccgtgaagcac
gggttcgaatcccgttatcctcgt
1-14tRNA-Glu(ttc)c[157499,157575]
gtcgcattggtgaaattggctaacacactcggctttctaccgagcattca
ggggttcgaatcccctatgcgatacca
1-15tRNA-Asp(gtc)c[157585,157659]
ggcttgttagtatatgggttaacacacttgcctgtcacgcaagagaaacg
ggttcgagtcccgtacaggccgcca
1-16tRNA-Thr(tgt)c[157665,157741]
gccttcttagctcagttggttagagcgactgccttgtaagcagtaggtcg
tgggttcgattcctacagtcggcacca
1-17tRNA-Gly(tcc)c[157746,157821]
ggggcattagtatatcggttaattattcctggcttccaaccaggggaggt
cggttcgattccgacatgtccctcca
1-19tRNA-Cys(gca)c[158134,158206]
gaggacgtaccgaaatggttaacgggctaggttgcaaccctagtattcgc
tggttcgaatccagtcgttctct
1-20tRNA-Ser(gct)c[158216,158304]
ggaagggtactcaagttggtgaagaggtcaacctgctaagttgatagtac
ctattgtaggtagcgagggttcgaatccctttccttcct
>JX561091.1 Escherichia phage phAPEC8, complete genome
1-1tRNA-Met(cat)[59143,59217]
ggccctgtagctcaatgggagagctgtcggctcataaccgataggtagct
ggatcgaaaccagccagggccacca
1-2tRNA-Arg(tct)[59220,59294]
gtctttatggtgtaatggatagcacaggagtcttctaaactcttagtcaa
ggttcgattccttgtaaagacacca
1-3tRNA-Ser(gct)[59624,59709]
ggtagattggtgaaatggtagccacgacagtttgctaaactgtagtcgga
aacggcgtgtaggttcgagtcctacatctaccgcca
1-4tRNA-Tyr(gta)[59837,59920]
gtgagattggcagagtggtcgattgcgggggactgtaaatcccttctgaa
aggcgcggtggttcgaatccatcatctcacacca
1-5tRNA-Asn(gtt)[59927,60012]
gatgagttggctgaatggtttaagcggcggactgttaatccgtgttcgaa
agaacgatataggttcaaatcctatactcatcgcca
1-6tRNA-Thr(tgt)[60103,60177]
gctcctatagtataatggctattacaacggttttgtaatccgtggatctc
tgttcgattcagagtgggagcacca
1-7tRNA-Gly(tcc)[60494,60567]
gcgttcttggtgtagcggtagcattccgtccttccaagtcggcggcacga
gttcgatcctcgtagaacgctcca
1-8tRNA-Gln(ttg)[60666,60741]
tggtttgtagtgtagtccggtagcacacgacactttgactgtcgtaggtt

tggttcaaatccagacagaccagcca
1-9tRNA-Pro(tgg)[60835,60911]
cggaatttagctcagtttggtagagtgtacgctttgggagcgtattgtcg
caggttcgaatcctgcaattccgacca
1-10tRNA-Ile(gat)[60918,60991]
tgtttcgtagttcaatggtagaacccacgactgataatcgtgagatacaa
gttcgattcttgtcgaaacaacca
1-11tRNA-Met(cat)[61070,61148]
agcgggatagaggagtctggtcgtcctcgccagtttcatatgctggagat
cattggttcaaatccaattcccgcctcca
>HQ634177.1 Synechococcus phage S-CAM1 genomic sequence
1-1tRNA-Asn(gtt)c[74005,74080]
ttcctctatagctcaatcaggcagagcggttgactgttaatcaataggtt
cctggttcgattccaggtggaggagt
1-2tRNA-Ala(tgc)c[74130,74203]
ggggaattagctcagctggtagagcgcctgctttgcaagcaggatgtcag
gagttcgagtctcctattctccac
1-3tRNA-Leu(tag)c[74805,74886]
gtcgatgtggcggaattggtagacgcgctgggtttaggttccagtggatt
tatccgtggaggttcaagtcctctcatcgaca
1-4tRNA-Thr(tgt)c[74890,74963]
gcctgtttagctcagctggtagagcaacgcttttgtaaagcgtaggtcgt
cagttcaagtctgtcaacaggctc
1-5tRNA-Leu(taa)c[75783,75866]
ggggcagtggtggaatcggtagacacaccagacttaaaatctgttgacct
taaggtcgtgcgagttcaattctcgcttgcccta
1-6tRNA-Gly(tcc)c[106145,106215]
gcgggattagtttagaggcaaaactaaaggtttccaacctttcgtcacca
gttcgattctggtatcccgct
1-7tRNA-Val(tac)c[108518,108589]
gggcgattagcgcagcggtagcgcacctcctttacacggagagggtcggg
ggttcgattccctcatcgccca
1-8tRNA-Arg(tct)c[108590,108665]
gggcacgtagcataatggataatgcatcactcttctaaagtgccgattgc
tggttcgagtccagccgtgcctgcca
>HQ317292.1 Synechococcus phage S-RIM2 R1_1999, complete genome
1-1tRNA-Val(tac)[13794,13865]
gcccgaatagctcagcggtagagcacctcgtttacaccgagattgtcggg
ggttcgatccccctcttcgggca
1-2tRNA-Leu(taa)[17791,17877]
tgggagtatggcggaatcggtagacgcaccagacttaaaatttgttgagg
gttaacctcgtgagagttcaagtctctctactcctat
1-3tRNA-Thr(tgt)[17934,18006]
gcccccgtagctcagtggtagagcagggcttttgtaaagctcaggtcgca

agttcaaatcttgtcaggggctc
1−4tRNA−Ala(tgc)[18009,18081]
ggggaattagctcagttggtagagcgcctgctttgcaagcaggatgtcag
cggttcgagtccgctattctcca
1−5tRNA−Asn(gtt)[18128,18201]
ttcctcagtagctcagcggcagagccatcgactgttaatcgattggtcgt
aggttcaaatcctacctggggagt
1−6tRNA−Arg(tct)[161238,161312]
tgggtcagtagctcagcggatagagcaaccgccttctaagcggttggtcg
caggttcaaatcctgcctgacccgt
>JX846613.1 Staphylococcus phage vB_SauM_Romulus, complete genome
1−1tRNA−Ser(gct)c[126593,126680]
ggagtattactcaagtggcttaagaggttgggttgctaactcaataggtg
atttatttcactcataggttcaaatcctatatgctccg
>JN377895.1 Aeromonas phage Aes012, complete genome
1−1tRNA−Arg(tct)c[62227,62303]
ggtctcttagctcagtcggatagagtggcggccttctaagccgcgggtca
ttggttcgaatccaatagggactgcca
1−2tRNA−Met(cat)c[62872,62947]
ggccctctgcacagtctgtgaatgtgtccagctcataactggggtaagcc
gggttagattcccgggagggccacca
1−3tRNA−Met(cat)c[63150,63226]
tgcaccgtagaggagaggccgtcctcgccagtctcataagctggagatcg
tgagttcgaatctcaccggagcatcca
1−4tRNA−Asp(gtc)c[63239,63312]
ggagccatagtttatttggttaaaatagtcccctgtcacgggacagcacc
gagttcgatcctcggtggctccgc
1−5tRNA−Trp(cca)c[63386,63460]
aggggtgtagtgtaatggtagcacgccggcctccaaagccgtgtagtctg
gattcgaattctagcatccctgcca
1−6tRNA−Thr(tgt)c[63821,63896]
gccggtttagctcatttggtagagcacttcacttgtaatgatggggtggt
ccgttcgaatcggacaaccggcacca
1−7tRNA−Pro(tgg)c[64154,64228]
ctcagcgtaggcaagtggtatgtcgttccgtttgggacggaattttcggg
tgttcgattcaccccgctgagacca
1−8tRNA−Gly(tcc)c[64943,65018]
gcgggtaaggtgtttatggatacacgctagccttccaagcttgagtagac
cggttcgataccggctacccgctcca
1−9tRNA−His(gtg)c[65030,65105]
gtgacggtagctcaattggtagagccccggattgtgattccggccgttgc
gagatcatccctcgtccgtcacccca
1−10tRNA−Lys(ttt)c[65397,65472]
gcgtcggtagctaagcggtaaagcacctgacttttaatcaggatactcga

tggttcgaatccatcccggcgcacca
1−11tRNA−Asn(gtt)c[65640,65716]
ggttgattagctcagttggttagagcggcggactgttaacccgcgggtct
cccgttcgagtcgggaatcaaccgcca
1−12tRNA−Arg(cct)c[65796,65878]
ggtgctgtgagccaagcggcttaaggcggtcctaatgcaggacatacgaa
agcgtatcgtaggttcgaatcctaccagcaccc
1−13tRNA−Ser(tga)c[65885,65976]
ggagacttgggtgagaggcttaaaccaccagtcttgaaaactggcgatcg
taggaatacggtccatccgttcaaatcggatagtctccgcca
1−14tRNA−Leu(taa)c[66168,66252]
gcaagtatggtggaattggtatacactggaggcttaaaacctcccgcctt
cgggattgtgggttcgagtcccactacttgcacca
>KC748970.1 Mycobacterium phage ArcherS7, complete genome
1−1tRNA−Ser(gct)[30783,30866]
ggagggtgagcatcaggtgatgcagcgagattgctaatcccgtacggtaa
ccaccccgtgaggttcgaatcctcctccctccgc
1−2tRNA−Leu(cag)[31059,31134]
gctcccgtagcccaattggcaggaggcaccagattcaggatctgggcagt
gtgagttcgaatctcaccgggagtac
1−3tRNA−Leu(gag)[31254,31328]
gtctctgtaggcaaatcggaaaagccgccatcttgagggggtggtgcgtg
cgggttcgactcccgccagagacac
1−4tRNA−Leu(caa)[31329,31402]
gccgtggtaggccatctggcgagccgccgagttcaagtttcggtgtttgc
gggttcgaatcccgcccacggtac
1−5tRNA−SeC(tca)[67326,67422]
attctggcactggtgggcgagcccaccggcggcagcttcaagctgtcgct
ggccggagaaccgaccggaaacatcccgttcaacgcgacccagggcc
1−6tRNA−Pro(tgg)[91290,91361]
cggggtgtagtgaaaaggcatcatgctggttttgggtaccagtggttccg
gttcgagtccgggctccccgac
1−7tRNA−Trp(cca)[91800,91875]
tggggtgaagccgatctggaaggcagcggtctccaaagccgtctcatagc
gggttcgaatcccgtcacccctgcca
1−8tRNA−Tyr(gta)[91877,91963]
gccgcacatgcccaactggtgtttgggagcaggctgtaaccctgtggcct
tcgggacggtgaggttcgattcctcagtgcggtacca
1−9tRNA−Pyl(cta)[93072,93144]
gcaccatttgctcaatggcagagcggcggcttctaaaaccgtgagtgccg
gttcgactccggcatggtgcacc
1−10tRNA−Met(cat)[93294,93368]
agcggtgtagagcagctaggtagctcgccgggctcataacccggaggacg
cgtgttcgaatcacgccaccgccac

1-11tRNA-Cys(gca)[93495,93566]
gcgcctttggcggaatggctacgtgctcggctgcaacccgagttatcccg
gttcgactccgggagggcgctc
1-12tRNA-Glu(ctc)[93571,93645]
ggtccgttggagtagatggatatctcgccaccctctcaaggtggagatca
cgggttcaagtcccgtacggactgc
1-13tRNA-His(gtg)[93647,93720]
gtggccgtagttcagccggtagaacgctgggttgtgatcccagtcgtcga
gggttcgagtccctccggtcaccc
1-14tRNA-Ala(tgc)[93882,93955]
gggcctatagctcatctggtagagcgcctgccttgcaagcaggaggcggc
aggttcaagtcctgttaggtccac
1-15tRNA-Phe(gaa)[94145,94217]
gccgtcatagctcagttggtagagcactggcctgaaaacccagtggccga
ggttcgattcctcgtgtcggcac
1-16tRNA-Val(cac)[94223,94296]
gtccgtttagctcagctggaagagcgctcggtccacacccgagaggccgc
aggttcgatccctgcaatggacac
1-17tRNA-Lys(ctt)[94415,94487]
gccttcgtagctcagtggtagagctgtcgcctcttaagcgataggtcgtt
ggttcgaatccagccgggggcac
1-18tRNA-Glu(ttc)[94492,94568]
ggtcgggtcggtctgctggtatggccagtcggattttcactccggacatt
cgcgggttcaattcccgtcccgatcgc
1-19tRNA-Gly(tcc)[94648,94720]
gcgggtgtggccgaatggctcaggcaccagatttccactctggctacgca
ggttcgattcctgtcatccgctc
1-20tRNA-Thr(cgt)[94780,94854]
gctgctgtagctcacctggcagagcgtcggcgtcgtatcccgaaggcatc
cggttcgagtccggacagcagcccc
1-21tRNA-Thr(tgt)[94855,94927]
gcctctgtggtccagcggcacggacatccgccttgtaagcggaggacccc
cgttcgatccggggtagaggctc
1-22tRNA-Thr(ggt)[95335,95407]
gctgggttagctcagtggtagagcgttcctctggtatgggaaaggcccgg
ggttcaatcccccgattcagctc
1-23tRNA-Gly(gcc)[96730,96803]
gcgaaggtagctcagctggtagagcgccaccttgccaaggtggaggtcgc
gggatcgtaacccgttcttcgctc
1-24tRNA-Asp(gtc)[96807,96879]
ggccctgtagctcagaggaagagcgccggtctgtcgaatcggaggtcgcg
gtatcgtaatccgtcagggtcgc
1-25tRNA-Met(cat)[96939,97011]
gcctcactagctcattggtagagccgctcgctcataacgtgcaggtacct

ggttcgattccagggtgaggtac
1-26tRNA-Ile(gat)[97017,97091]
gcctgttagcggactggtcgtccgatccaagctgataacttggcgtaagc
ggtgttcgattcaccgagcaggtac
1-27tRNA-Arg(acg)[97185,97257]
gcctctatggtccaacggatatgacgccggtctacggaaccggagatgcg
tgttcgattcgcgctaggggcac
1-28tRNA-Val(gac)[97300,97372]
gtccgtgtagctcaggggtagagcgcctgctcgacacgcaggaggaccga
ggttcgaaacctcgcatggacac
1-29tRNA-Arg(cct)[97546,97619]
gcctctgtagctcaacggacagagcaacgcggtcctaacgcggtggctgg
aggttcgaatcctctcggaggcac
1-31tRNA-Gln(ttg)[98160,98235]
tggggtatggtggcaatctggcagtcccgccggactttgactccggaggt
gcaggttcgagtcctgctaccccatc
1-32tRNA-Arg(tct)[98239,98314]
gcccttgtagctcagtggacagagcggcgagcttctacctcgcgggccgg
gagttcgaatctctccaggggcacca
1-33tRNA-Gln(ctg)[126933,127007]
tgctcgttggtgtaactggcaacactacggactctgactccgtcatttta
ggttcgaatcctaagcgagcagcca
1-34tRNA-Asn(gtt)[127014,127089]
tggggtgtccgttaatcaggcaaacgagcggactgttaatccgcccctgc
aggttcgaatcctgccaccccagcca
>KC801932.2 Escherichia phage Lw1, complete genome
1-1tRNA-Met(cat)[49530,49607]
ggccccttagctcaattggttagagcgaacccctcataagggtgtggttt
ccggttcaagtcacggaaggggccacca
>HQ634178.1 Synechococcus phage S-CAM8 strain S-CAM8 06008BI06, complete genome
1-1tRNA-Val(tac)[14508,14579]
gcccgaatagctcagcggtagagcagcacctttacacggtgaatgtcggg
ggttcgatcccctctttgggca
1-2tRNA-Leu(taa)[18531,18617]
tgggagtgtggcggaatcggtagacgcaccagacttaaaatctgttgacc
aataaggtcgtgggagttcaagtctccctactcctat
1-3tRNA-Thr(tgt)[18671,18743]
gcctccgtagctcagtggtagagcagggcttttgtaaagctcaggtcgca
agttcaaatcttgtcagaggctc
1-4tRNA-Asn(gtt)[18748,18819]
tcctccttagctcagcggtagagcggttgactgttaatcaatttgtccct
ggttcgatcccaggagggggag
1-5tRNA-Arg(tct)[155048,155123]
tgggtcagtagctcagatggatagagcaactgccttctaagcagtcggcc

acaggttcgagtcctgtctgacccgt
>AP013029.1 Bacillus phage phiNIT1 DNA, complete genome
1-1tRNA-Ser(gct)[33425,33513]
tggataggtacccaagcggtaaaggggtctggttgctaaccagatagtag
gctgaggtctagcggaggttcgaatcctctcctatccgt
1-2tRNA-Asn(gtt)[33749,33822]
ttgtcccatagctcaatggtagagcgcccgactgttaatcggaaggttgc
tggttcgagcccagctgggacagt
1-4tRNA-Asp(gtc)[35511,35584]
tggctccatagtgcagcggttaacacgcccgcctgtcacgtgggtagcac
gggttcgaatcccgttggagccgt
1-5tRNA-Tyr(gta)[35750,35833]
gggtgagcggtaacgttggagagttacggcagactgtaaatctgttccct
tcggggttagagtgttcgaatcactccttgccca
>HQ634175.1 Cyanophage P-RSM1 genomic sequence
1-1tRNA-Leu(taa)c[39357,39440]
gggagtgtggcggaatcggtagacgcaccagacttaaaatctgttgacct
tgagtcgtgggggttcaagtccccccactcctac
1-2tRNA-Arg(tct)c[71616,71690]
tgggtcagtagcacagcggatagtgcaactgccttctaagcagtcggtcg
caggttcaaatcctgcctgacccgt
>KC691257.1 Mycobacterium phage Astraea, complete genome
1-1tRNA-Ser(gct)[30739,30822]
ggagggtgagcatctggtgatgcaggggtcctgctaaggccctacggatt
cacacccgtgagtttcgattactcctccctccgc
1-2tRNA-Leu(cag)[30919,30995]
gccctgctgagcaaactggcaaagctgccgcattcagagtgcgggtcatt
tccgggttcgactcccgggcagggtac
1-3tRNA-Leu(gag)[31115,31189]
gtctctgtaggcaaatcggaaaagccgccatcttgagggggtggtgcgtg
cgggttcgactcccgccagagacac
1-4tRNA-Leu(caa)[31190,31263]
gccgtggtaggccatctggcgagccgccgagttcaagtttcggtgtttgc
gggttcgaatcccgcccacggtac
1-5tRNA-SeC(tca)[66777,66873]
attctggcactggtgggcgagcccaccggcggcagcttcaagctgtcgct
ggccggagaaccgaccggaaacatcccgttcaacgcgacccagggcc
1-6tRNA-Pro(tgg)[90158,90232]
cggggtgtagttcagtttggaagagcgcttggtttgggaccaagttgtcg
caggttcgaatcctgtcaccccgac
1-7tRNA-Trp(cca)[90247,90317]
gggtctgtgcacagggtgcccgacggtctccaaagccgaaggcgggggtt
cgattccctccaggcctgcca
1-8tRNA-Pyl(cta)[91509,91581]

gcaccatttgctcaatggcagagcggcggcttctaaaaccgtgagtgccg
gttcgactccggcatggtgcacc
1–9tRNA–Met(cat)[91731,91805]
agcggtgtagagcagctaggtagctcgccgggctcataacccggaggacg
cgtgttcgaatcacgccaccgccac
1–10tRNA–Cys(gca)[91932,92003]
gcgcctttggcggaatggctacgtgctcggctgcaacccgagttatcccg
gttcgactccgggagggcgctc
1–11tRNA–Glu(ctc)[92071,92142]
gctcccatggggtagtggtaaccctcctggttctcagccaggcgtcccga
gttcgatcctcggtgggagtgc
1–12tRNA–His(gtg)[92197,92270]
gtggtagtagttcagatggaagaacgtcgccttgtgacggcgaaggtcgg
gggttcgaagcccctctatcaccc
1–13tRNA–Ala(tgc)[92941,93015]
gggcctgtagctccaattggtagagcagcatccttgcaagatgacggctg
tcggttcgaatccgacctggtccac
1–14tRNA–Phe(gaa)[93205,93277]
gccgtcatagctcagttggtagagcactggcctgaaaacccagtggccga
ggttcgattcctcgtgtcggcac
1–15tRNA–Val(cac)[93283,93356]
gtccgtttagctcagctggaagagcgctcggtccacacccgagaggccgc
aggttcgatccctgcaatggacac
1–16tRNA–Lys(ctt)[93475,93547]
gccttcgtagctcagtggtagagctgtcgcctcttaagcgataggtcgtt
ggttcgaatccagccgggggcac
1–17tRNA–Glu(ttc)[93552,93628]
ggtcgggtcggtctgctggtatggccagtcggattttcactccggacatt
cgcgggttcaattcccgtcccgatcgc
1–18tRNA–Gly(tcc)[93708,93780]
gcgggtgtggccgaatggctcaggcaccagatttccactctggctacgca
ggttcgattcctgtcatccgctc
1–19tRNA–Thr(cgt)[93841,93915]
gctgctgtagctcacctggcagagcgtcggcgtcgtatcccgaaggcatc
cggttcgagtccggacagcagcccc
1–20tRNA–Thr(tgt)[93916,93988]
gcctctgtggtccagcggcacggacatccgccttgtaagcggaggacccc
cgttcgatccggggtagaggctc
1–21tRNA–Thr(ggt)[94396,94468]
gctgggttagctcagtggtagagcgttcctctggtatgggaaaggcccgg
ggttcaatcccccgactcagctc
1–22tRNA–Gly(gcc)[95791,95864]
gcgaaggtagctcagctggcagagcgccaccttgccaaggtggaggtcgc
gggatcgtaacccgttcttcgctc

1-23tRNA-Asp(gtc)[95868,95940]
ggccctgtagctcagaggaagagcgccggtctgtcgaatcggaggtcgcg
gtatcgtaatccgtcagggtcgc
1-24tRNA-Met(cat)[96000,96072]
gcctcactagctcattggtagagccgctcgctcataacgtgcaggtacct
ggttcgattccagggtgaggtac
1-25tRNA-Ile(gat)[96078,96152]
gcctgttagcggactggtcgtccgatccaagctgataacttggcgtaagc
ggtgttcgattcaccgagcaggtac
1-26tRNA-Arg(acg)[96246,96318]
gcctctatggtccaacggatatgacgccggtctacggaaccggagatgcg
tgttcgattcgcgctaggggcac
1-27tRNA-Val(gac)[96361,96433]
gtccgtgtagctcaggggtagagcgcctgctcgacacgcaggaggaccga
ggttcgaaacctcgcatggacac
1-28tRNA-Arg(cct)[96608,96681]
gcctctgtagctcaacggacagagcaacgcggtcctaacgcggtggctgg
aggttcgaatcctctcggaggcac
1-30tRNA-Gln(ttg)[97222,97297]
tggggtatggtggcaatctggcagtcccgccggactttgactccggaggt
gcaggttcgagtcctgctaccccatc
1-31tRNA-Arg(tct)[97301,97376]
gcccttgtagctcagtggacagagcggcgagcttctacctcgcgggccgg
gagttcgaatctctccaggggcacca
1-32tRNA-Gln(ctg)[124792,124866]
tgctcgttggtgtaactggcaacactacggactctgactccgtcatttta
ggttcgaatcctaagcgagcagcca
1-33tRNA-Asn(gtt)[124873,124948]
tggggtgtccgttaatcaggcaaacgagcggactgttaatccgcccctgc
aggttcgaatcctgccaccccagcca
>HQ317383.1 Synechococcus phage S-IOM18 genomic sequence
1-1tRNA-Val(tac)[4930,5003]
tgggcgaatagctcagcggtagagctactcgtttacaccgagtcggtcgg
gggttcgatcccctcttcgcccat
1-2tRNA-Leu(taa)[9367,9452]
tgggagcgtggcggaatcggtagacgcaccagacttaaaatctgttgggc
aatagcccgtgagagttcaagtctctccgttcctat
1-3tRNA-Thr(tgt)[9697,9770]
gcccccgtagctcagtggtagagcagggcttttgtaaagctcaggtcgca
agttcaaatcttgtcaggggctcc
1-4tRNA-Ala(tgc)[9772,9847]
tggggaattagctcagttggtagagcgcctgctttgcaagcaggatgtca
gcggttcgagtccgctattctccatc
1-5tRNA-Asn(gtt)[9897,9968]

tcccaagtagctcagtggcagagcaggtgactgttaatcactcggtcgca
ggttcaaatcctgccttgggag
1-6tRNA-Arg(tct)[147052,147126]
tgggtcagtagctcagcggatagagcaactgccttctaagcagttggtcg
caggttcaaatcctgcctgactcgt
1-7tRNA-Pro(tgg)[148131,148204]
cggggtgtagcttagcttggtagagcgcccgctttgggagcgggaggtcg
caggttcaaatcctgccaccccga
>JX126918.2 Listeria phage LP-125, complete genome
1-1tRNA-Met(cat)c[35284,35357]
ttgtcccgtagctagaaggtcgagcaaggagctcataactcctcggtttg
ggttcgattcccaacggggcaatc
1-2tRNA-Pro(tgg)c[36250,36324]
cagggtgtagctcagtttggttagagtacccgctttggagacgggaagtc
gtaggttcgagtcctaccaccttga
1-3tRNA-Arg(tct)c[37442,37512]
gtccttatggtgtagtggatgcacaagggatttctactcccttagcgcgg
gttcgaatcctgctgaggact
1-4tRNA-Gly(tcc)c[37770,37840]
gcgggtatagtataagggtagtacccaaggtttccaaccatgtagtgggg
gttcgaatccccctacccgct
1-5tRNA-Asn(gtt)c[37909,37982]
gtgtccttaactcagaggtcagagtgccgtcctgttaagtcggaagtcgc
tggttcaaatccagcaggatacgc
1-6tRNA-Ser(tga)c[38604,38695]
ggaaggttggtagagcttggtaatacgctagtcttgaaaactagtttgcc
cttggaatacagggtacaagggttcaaatcccttaccttcct
1-7tRNA-Phe(gaa)c[38708,38779]
gtagtcctagctgagatggattagcgcttgcttgaaaagcaagagaggca
ggttcgatacctgcggactcca
1-8tRNA-Lys(ttt)c[38785,38856]
ggagttatggtgaaatggctatcactgcgggtttttaccccgttattcta
ggttcgaatcctagtggctcca
1-9tRNA-Trp(cca)c[38981,39054]
taggggtatagtttatctggtaaaatattggtttccaactccaatgaggt
gggttcaagtcctactatccctgt
1-10tRNA-Gln(ttg)c[39056,39128]
tggtctgtagccaagcggtaaggcaacggattttgattccgtgatacgtt
ggttcgaatccaactagaccagc
1-11tRNA-Thr(tgt)c[39147,39218]
gcttgtatagttcaattggtagaacagtggttttgtaagcctcagacgtg
ggttcaagtcctactacaagca
1-12tRNA-Tyr(gta)c[39301,39382]
gtgccattcgcatagaggcaattgcaggggactgtaactcccctcccttc

ggggttccaaggttcgagtccttgatggcgca
1-13tRNA-Leu(tag)c[39743,39827]
tgccgagatggtggaactggtatacacggtagacttagaatctgctgtcc
taaggatatgtgggttcgaatcccactctcggtat
1-14tRNA-Asp(gtc)c[40293,40365]
gtgcgtatgatataatggctattatactcggctgtctatcgagaaatagg
ggttcgattccccttacgtgcgc
1-15tRNA-Ile(gat)c[40469,40541]
gccagcatagcttagtaggcaaagcaaccgaccgataatcggtagtcctt
ggttcaattccaagtgttggtac
1-16tRNA-Ser(gct)c[40718,40804]
ggagagttgtcagagaggcttaatgatacgggttgctaactcgttgtact
agtaatagtaccaagggttcgaatcccttactctcct
1-17tRNA-Cys(gca)c[40883,40953]
gcgggtataaccaactggaaaggtagtagactgcaaatctacgtatatgg
gttcaattcccattacccgct
>KC748968.1 Mycobacterium phage Gizmo, complete genome
1-1tRNA-Ser(gct)[31235,31318]
ggagggtgagcatctggtgatgcaggggtcctgctaaggccctacggatt
cacacccgtgagtttcgattactcctccctccgc
1-2tRNA-Leu(cag)[31415,31491]
gccctgctgagcaaactggcaaagctgccgcattcagagtgcgggtcatt
tccgggttcgactcccgggcagggtac
1-3tRNA-Leu(gag)[31611,31685]
gtctctgtaggcaaatcggaaaagccgccatcttgagggggtggtgcgtg
cgggttcgactcccgccagagacac
1-4tRNA-Leu(caa)[31686,31759]
gccgtggtaggccatctggcgagccgccgagttcaagtttcggtgtttgc
gggttcgaatcccgcccacggtac
1-5tRNA-SeC(tca)[70109,70205]
attctggcactggtgggcgagcccaccggcggcagcttcaagctgtcgct
ggccggagaaccgaccggaaacatcccgttcaacgcgacccagggcc
1-6tRNA-Pro(tgg)[93378,93452]
cggggtgtagttcagtttggaagagcgcttggtttgggaccaagttgtcg
caggttcgaatcctgtcaccccgac
1-7tRNA-Trp(cca)[93467,93537]
gggtctgtgcacagggtgcccgacggtctccaaagccgaaggcgggggtt
cgattccctccaggcctgcca
1-8tRNA-Pyl(cta)[94734,94806]
gcaccatttgctcaatggcagagcggcggcttctaaaaccgtgagtgccg
gttcgactccggcatggtgcacc
1-9tRNA-Met(cat)[94956,95030]
agcggtgtagagcagctaggtagctcgccgggctcataacccggaggacg
cgtgttcgaatcacgccaccgccac

1-10tRNA-Cys(gca)[95157,95228]
gcgcctttggcggaatggctacgtgctcggctgcaacccgagttatcccg
gttcgactccgggagggcgctc
1-11tRNA-Glu(ctc)[95233,95307]
ggtccgttggagtagatggatatctcgccaccctctcaaggtggagatca
cgggttcaagtcccgtacggactgc
1-12tRNA-His(gtg)[95309,95382]
gtggccgtagttcagccggtagaacgctgggttgtgatcccagtcgtcga
gggttcgagtccctccggtcaccc
1-13tRNA-Ala(tgc)[95544,95618]
gggcctgtagctccaattggtagagcagcatccttgcaagatgacggctg
tcggttcgaatccgacctggtccac
1-14tRNA-Phe(gaa)[95808,95880]
gccgtcatagctcagttggtagagcactggcctgaaaacccagtggccga
ggttcgattcctcgtgtcggcac
1-15tRNA-Val(cac)[95886,95959]
gtccgtttagctcagctggaagagcgctcggtccacacccgagaggccgc
aggttcgatccctgcaatggacac
1-16tRNA-Lys(ctt)[96078,96150]
gccttcgtagctcagtggtagagctgtcgcctcttaagcgataggtcgtt
ggttcgaatccagccgggggcac
1-17tRNA-Glu(ttc)[96155,96231]
ggtcgggtcggtctgctggtatggccagtcggattttcactccggacatt
cgcgggttcaattcccgtcccgatcgc
1-18tRNA-Gly(tcc)[96311,96383]
gcgggtgtggccgaatggctcaggcaccagatttccactctggctacgca
ggttcgattcctgtcatccgctc
1-19tRNA-Thr(cgt)[96443,96517]
gctgctgtagctcacctggcagagcgtcggcgtcgtatcccgaaggtatc
cggttcgagtccggacagcagcccc
1-20tRNA-Thr(tgt)[96518,96590]
gcctctgtggtccagcggcacggacatccgccttgtaagcggaggacccc
cgttcgatccggggtagaggctc
1-21tRNA-Thr(ggt)[96650,96722]
gctgggttagctcagtggtagagcgttcctctggtatgggaaaggcccgg
ggttcaatcccccgactcagctc
1-22tRNA-Gly(gcc)[98467,98542]
gcggttatagctcagcctggccagagcatcacgttgccaacgtgaacgtc
gcgggttcaaatcccgctagccgctc
1-23tRNA-Asp(gtc)[98556,98628]
ggccctgtagctcagaggaagagcacccgcctgtcgagcgggaggtcgcg
gtatcgtaatccgtcagggtcgc
1-24tRNA-Met(cat)[98688,98760]
gcctcactagctcattggtagagccgctcgctcataacgtgcaggtacct

ggttcgattccagggtgaggtac
1-25tRNA-Ile(gat)[98766,98840]
gcctgttagcggactggtcgtccgatccaagctgataacttggcgtaagc
ggtgttcgattcaccgagcaggtac
1-26tRNA-Arg(acg)[98933,99005]
gcctctatggtccaacggatatgacgccggtctacggaaccggagatgcg
tgttcgattcgcgctaggggcac
1-27tRNA-Val(gac)[99048,99120]
gtccgtgtagctcaggggtagagcgcctgctcgacacgcaggaggaccga
ggttcgaaacctcgcatggacac
1-28tRNA-Arg(cct)[99294,99367]
gcctctgtagctcaacggacagagcaacgcggtcctaacgcggtggctgg
aggttcgaatcctctcggaggcac
1-30tRNA-Gln(ttg)[99908,99983]
tggggtatggtggcaatctggcagtcccgccggactttgactccggaggt
gcaggttcgagtcctgctaccccatc
1-31tRNA-Arg(tct)[99987,100060]
gcccttgtagctcagtggacagagcggcgagcttctacctcgcgggccgg
gagttcgaatctctccaggggcac
1-32tRNA-Lys(ttt)[100128,100204]
gggccggtatcttagtctggtcaaagaagtggacttttaatccgcgcgcc
gtgggttcgaatcccacccggcccacc
1-33tRNA-Gln(ctg)[128217,128291]
tgctcgttggtgtaactggcaacactacggactctgactccgtcatttta
ggttcgaatcctaagcgagcagcca
1-34tRNA-Asn(gtt)[128298,128373]
tggggtgtccgttaatcaggcaaacgagcggactgttaatccgcccctgc
aggttcgaatcctgccaccccagcca
>HQ317393.2 Vibrio phage nt-1, complete genome
1-1tRNA-Ser(gct)c[89870,89958]
ggagatatggtctaatggtatgacagcaccctgctaaggtgtcggacgtt
aatagcgttctctgggttcgactcccagtatctccgcca
1-2tRNA-Leu(caa)c[90888,90964]
gcccaactagtccaattggcagaggcgctagtttcaaacactagatgatc
cgagttcgaatctcgggttgggcacca
1-3tRNA-Leu(taa)c[90971,91047]
gcgcatgtggtccaattggcagaggcatgaggtttaaattctcagggatg
gcggttcgaatccgcccatgcgtacca
1-4tRNA-Cys(gca)c[91492,91565]
gcccgaatcgtataatggaagtatgagggattgcaaatcccgcggtcaga
gttcgattctctgttcgggctcca
1-5tRNA-His(gtg)c[91966,92040]
gtggctgtggcagagtggtaatgcccctgattgtgattcaggaactcgcg
ggttcgattcccgtcggtcacccca

1–6tRNA–Val(tac)c[92720,92793]
agccccttagtataatgggagtacgcttctcttacacagaagtagagtcg
gctcgaatccgacaggggctacca
1–7tRNA–Gly(tcc)c[92954,93028]
gcgggtatgatgtaattggtagcatgatgtccttccaagtcgttcgtctc
ggttcgagtccgtgtacccgctcca
1–8tRNA–Met(cat)c[93321,93396]
ggagatgtagctcaagtggtagagcggcgctttcatacggcgttagatcc
gatttcgagtatcggtgtctccacca
1–9tRNA–Arg(tct)c[94086,94161]
gtcctcatagctcaattggaagagcacgtcccttctaaggatgtggttct
aggttcgaatcctagtggggatgcca
1–10tRNA–Ala(tgc)c[94236,94311]
ggggatgtagctcaattgggagagcacctgccttgcaagtaggaggttgc
gagttcgagtctcgtcatttccacca
1–11tRNA–Thr(tgt)c[95030,95105]
gccctgatagcacaactggcagtgcagctcacttgtaatgagcaggttcg
cggttcaaatccgtgtcagggcacca
1–12tRNA–Gln(ttg)c[95232,95305]
tgggatatggtgtaatggtagcactacggattttgattccgtcagttaga
gttcgaatctctatatcccagcca
1–13tRNA–Asp(gtc)c[95602,95677]
ggagccgaggtgtaactgggtgcatattcccctgtcacgggaaaggaagc
gggttcgagccccgtcggttccgcca
1–14tRNA–Asn(gtt)c[95897,95972]
gcttcattaactcagtcggtagagtgacggactgttaatccgtgagtcgt
tggttcgagcccaacatgaagcgcca
1–15tRNA–Asn(gtt)c[95978,96052]
gacggagtagctcagaggcagagcagcgggctgttaacccgcaggtcgag
atttcgaaattctccttcgtcgcca
1–16tRNA–Trp(cca)c[96129,96202]
agggacatgatgtaacggcagcatgacggattccaaacccgttcgttaga
gttcgaatctctatgtccctgcca
1–17tRNA–Ile(gat)c[96209,96282]
agctccatagtttaacggtaaaacacgcgaccgataatcgcacgttgaca
gttcgagtctgtctggggctacca
1–18tRNA–Ser(tga)c[96345,96430]
gaaagattcggggtagcggcaacggtcttgaaaaccgtcggggcatggga
atgcctgttagggttcgaatccctagtcttccgcca
1–19tRNA–Met(cat)c[96455,96526]
tgcggagtagagcagtggtcagctcaccagtctcataagctggaggtcgt
tggttcgaatccaacctcacgc
1–20tRNA–Tyr(gta)c[96629,96715]
ggttcgttagactaatggtaagtcaggggattgtaaatccctcgccctta

atcgggcttctaggttcgagtcctaggcggaccacca
1-21tRNA-Glu(ttc)c[96722,96796]
tcacccgtagactaatggtaggtcacttccctttcaaggaagtatgcgcg
agttcgaatctcgtcgggtgaacca
1-22tRNA-Lys(ttt)c[96861,96934]
gggtctatgatgtaatggtagcatatgggacttttaatccttcagtcaga
gttcgaatctctgtggacccacca
1-23tRNA-Phe(gaa)c[97023,97098]
gcacccttagctcagttggtagagcggcggattgaagtcccgcaggtcac
ggtctcgatagccgtagggtgcacca
1-24tRNA-Leu(tag)c[97151,97228]
gcgcatgtagcccaatctggcagaggcactggtcttagaaaccagaagtt
aagagttcgaatctcttcatgcgtacca
1-25tRNA-Arg(acg)c[97435,97510]
gcccgattagctcaattggaagagcagcgccctacgaaggcgaaggttac
aagttcgaatcttgtattgggtgcca
1-26tRNA-Pro(tgg)c[97518,97594]
ccgtgactagctcaatctggtagagtactccgtttggggcggagaagtta
agcgttcgaatcgcttgtcacggacca
1-27tRNA-Pro(tgg)c[97604,97679]
cggtgtgtggtgtttaatgggagcatggtcgctttgggagcgagcggaaa
gagttcgaatctcttcacaccgacca
1-28tRNA-Met(cat)c[100873,100950]
ggtcctatagctcaactcggttagagcaaccgactcataatcggtaggtt
acaggttcgagtcctgttggggtcacca
>JF974314.1 Rhizobium phage RR1-A genomic sequence
1-1tRNA-Met(cat)[35772,35847]
gtcggagtagggaagaggtcatcccgcgtggctcataaccacgagatcgc
gagttcgaatctcgcctccgcaacca
>JX846612.1 Staphylococcus phage vB_SauM_Remus, complete genome
1-1tRNA-Ser(gct)c[129904,129991]
ggagtattactcaagtggcttaagaggttgggttgctaactcaataggtg
atttatttcactcataggttcaaatcctatatgctccg
>KF669649.1 Bacillus phage CampHawk, complete genome
1-1tRNA-Asn(gtt)[29027,29101]
tcctctatagctcaatggtagagcacatgactgttaatcatggggttgta
ggttcgaatcctactggaggagcca
1-2tRNA-Ser(gct)[29189,29278]
tggaatgttacccaagcggttaaggggtctggttgctaaccagatagcgg
gcttgatgtccggcataggttcgaatcctatacattccgt
1-3tRNA-Arg(cct)[33500,33574]
tgcccatgtagctcagtggatagagcacgtccctcctaaggacggtgtcg
ggagttcaaatctctccatgggcgt
>KF669654.1 Salmonella phage Maynard, complete genome

1-1tRNA-Ser(gct)c[19769,19857]
ggaaggttgcccgagaggtttaagggactcgactgctaatcgagtggggc
ttttagcccccgaaggttcgaatccttcaccttccgcca
1-2tRNA-Ile(gat)c[19862,19945]
gcgggagccataggggttggccagtagccgactgatcatcggcgacgatc
actggacaggttcgaatcctgttctcccgctcca
1-3tRNA-Asn(gtt)c[20307,20381]
ggtgctttagctaaatggtgaagcggcggactgttaatccgtgatagcaa
ggttcgagtccttgaggcaccgcca
1-4tRNA-Trp(cca)c[20390,20465]
gtgcccttgatctagcggctatgaggtcggtctccaaaaccgtacaaccc
aggttcgagtcctggagggcatgcca
1-5tRNA-Met(cat)c[20534,20610]
ggtcctgtagctcagtcggttagagcagtgaactcataattcattggtcg
ttggttcaaacccaaccaggatcacca
>AB853331.1 Staphylococcus phage S25-4 DNA, complete genome
1-1tRNA-Trp(cca)c[40531,40602]
acacccttagtataattagtagtacaagggtctccaaaacccttagtctt
tgtgcaaatcaaagagggtgtg
1-2tRNA-Phe(gaa)c[40609,40681]
ggtttcttagctcagatggtagagcactagattgaagctctaggtgtcat
tggttcaaatccaatagaaacca
1-3tRNA-Asp(gtc)c[40687,40762]
tggctcattggtgtaactggttaacacactgccctgtcacggcagagagt
acgagttcgagtctcgtatgagtcgt
>KF147891.1 Pseudomonas phage PaBG, complete genome
1-1tRNA-Thr(tgt)[120228,120303]
gccggtatagctcagctggtagagcaactgacttgtaatcagtaggtctc
gggttcgatccctgatgccggcacca
1-2tRNA-Ala(tgc)[122295,122388]
gacccagtagctcagttggtagagcgcgtgaacgcctgcgccgatagcgg
gagtaacgtcatagaggtcgagagttcaaatctctcctgggtct
1-3tRNA-Lys(ttt)[122606,122681]
gggtcgttagctcagttggtagagcatggcgcttttaacgccgtggtcgt
tggttcgagtccaacacgacccacca
1-4tRNA-Asp(gtc)[122690,122765]
ggccccatagtcgagcggttaagacgcttgcctgtcacgcaagaaatcgc
gggttcgattcccgctggggtcgcca
1-5tRNA-Lys(ttt)[122767,122843]
aggacgttagctcagttggttagagcatctgacttttaatcagagggtcc
tgggttcgagtcccagacgtcctacca
>AB797215.1 Klebsiella phage 0507-KN2-1 DNA, complete genome
1-1tRNA-Gln(ttg)c[83731,83804]
aggagcatagccaagcggtaaggcaatgggctttgatcccatgatctcag

gttcgaatcctggtgctccttcca
1−2tRNA−Asn(gtt)c[84392,84467]
gatggtgtagttcagtcggtagaacggtggactgttaatccatatgtcgc
aggttcaagtcctgccaccatcgcca
1−3tRNA−Trp(cca)c[84477,84552]
atctccttggtctagcggctatgatgccggtctccaaaaccgtgcgaccc
aggttcgagtcctggaggggatgcca
1−4tRNA−Met(cat)c[85017,85092]
ggtcctgtagctcagtggttagagcagtcgactcataatcgattggtcat
tggttcaaaaccaatcaggatcacca
1−5tRNA−Thr(ggt)c[140280,140370]
cacttcaatggattgggaaatttcgcctttggtggaggcgatatcttcgc
cgcgcaggacgaatttattgttcagaacggcgtggaaatgg
>KF669653.1 Salmonella phage Marshall, complete genome
1−1tRNA−Ser(gct)c[21402,21490]
ggaaggttgcccgagaggtttaagggactcgactgctaatcgagtggggc
ttttagcccccgaaggttcgaatccttcaccttccgcca
1−2tRNA−Ile(gat)c[21495,21578]
gcgggagccataggggttggccagtagccgactgatcatcggcgacgatc
actggacaggttcgaatcctgttctcccgctcca
1−3tRNA−Asn(gtt)c[21940,22014]
ggtgctttagctaaatggtgaagcggcggactgttaatccgtgatagcaa
ggttcgagtccttgaggcaccgcca
1−4tRNA−Trp(cca)c[22023,22098]
gtgcccttgatctagcggctatgaggtcggtctccaaaaccgtacaaccc
aggttcgagtcctggagggcatgcca
1−5tRNA−Met(cat)c[22167,22243]
ggtcctgtagctcagtcggttagagcagtgaactcataattcattggtcg
ttggttcaaacccaaccaggatcacca
>KF669652.1 Bacillus phage Grass, complete genome
1−1tRNA−Ser(gct)[31229,31317]
tggataggtatccaagcggtaaaggggtctggttgctaaccagatagtag
gctgaggtctagcggaggttcgaatcctctcctatccgt
1−2tRNA−Asn(gtt)[31569,31642]
ttgtcccatagctcaatggtagagcgcccgactgttaatcggaaggttgc
tggttcgagcccagctgggacagt
1−3tRNA−Gln(ttg)[32860,32932]
ttgccgagtagccaagtggtaaggcagcgggctttgaactcgctatcgtt
ggttcgatcccagcctcggcagt
1−4tRNA−Asp(gtc)[33035,33108]
tggctccatagtgcagcggttaacacgcccgcctgtcacgtgggtagcat
gggttcgaatcccgttggagccgt
1−5tRNA−Tyr(gta)[33274,33357]
gggtgagcggtaacgttggagagttacggcagactgtaaatctgctccct

tcggggttagagtgttcgaatcactccttaccca
>AB853330.1 Staphylococcus phage S25–3 DNA, complete genome
1–1tRNA–Met(cat)c[13017,13088]
ggactcttagcttaaaggtaaagccaaccgctcataacggtttgactgta
ggttcgagtcctgcagagtcca
1–2tRNA–Trp(cca)c[35664,35735]
acacccttagtataattagtagtacaagggtctccaaaacccttagtctt
tgtgcaaatcaaagagggtgtg
1–3tRNA–Phe(gaa)c[35742,35814]
ggtttcttagctcagatggtagagcactagattgaagctctaggtgtcat
tggttcaaatccaatagaaacca
1–4tRNA–Asp(gtc)c[35820,35895]
tggctcattggtgtaactggttaacacactgccctgtcacggcagagagt
acgagttcgagtctcgtatgagtcgt
>KC862299.1 Pseudomonas phage PAK_P3, complete genome
1–1tRNA–Asn(gtt)[86082,86158]
tccgttcggtccctcaaggtgagggagcttgactgttaatcaagacgtgc
ctggttcgattccagggagcggagcca
1–2tRNA–Tyr(gta)[86216,86303]
ggaggggtggcagagcggtttaatgcaccggactgtaaatccggcgtccg
accgggcatcgctggttcaaatccagccccctccacca
1–3tRNA–Gln(ttg)[86505,86581]
aggcgtgtggcgaaaaggtttaacgcactggactttgactccagcatttg
tgggttcgaatcccaccacgtctgcca
>KC862298.1 Pseudomonas phage PAK_P2, complete genome
1–1tRNA–Gln(ttg)[89404,89477]
tgccgcttcgttcaatggtaggacgccagactttgaatctggagatgatg
gttcgatcccatcagcggctgcca
1–2tRNA–Lys(ttt)[90226,90302]
tggacggaagctaaagtggatcagcatctggcttttaaccagacttatag
tgagttcgagtctcacccgttcaacca
1–3tRNA–Leu(tag)[90566,90650]
ggccctgtggtggaattggtatacacatcagtcttagaaactgacgccga
gaggattgagggttcaagtccctccggggccacca
1–4tRNA–Ile(gat)[90861,90936]
agccgggtagctcaattggtagagcacccgaccgataatcgggtggttga
aggttcaagtccttctctggctacca
1–5tRNA–Asp(gtc)[90946,91024]
ggcccattagctcagtctggactagagcaagcccctgtctagggcaaggt
cgtcggttcgaatccgacatgggtcgcca
1–6tRNA–Met(cat)[91125,91199]
agcgttaagactggctggcgctgtcactcggctcataaccgaatacaagt
ggttcgattccacctctcgcttcca
1–7tRNA–Cys(gca)[91435,91510]

cccgcgttggccgagaggtttaggcggcggattgcaaatccgtctcacat
cggttcaaatccgatacgcggctcca
1-8tRNA-Asn(gtt)[91521,91596]
tgggatgtagctcagttggtagagcagggagctgttaactctcaggtcgc
aggttcgaaccctgccgtcccagcca
1-9tRNA-Pro(tgg)[91659,91736]
ctcctcgtagctcagtctggttagagtgtcggatttggaatccgaaggtc
gaaggttcaaatccttccggggtgacca
1-10tRNA-Gly(tcc)[91744,91820]
gcgggtttcgtatagtctggttattattcttggcttccacccaagagact
agggttcgaatccctaagcccgcacca
1-11tRNA-Phe(gaa)[91883,91958]
gcgcttgaagctaactaggtagaagcaccgggttgaaattccggaggact
tggatcgttaccaagcgggcgcacca
1-12tRNA-Glu(ttc)[91965,92040]
gcagttatagattaaatggataaatcgccagactttcaatctggtgttcc
gggttcgatccccggtaactgctcca
1-13tRNA-His(gtg)[92101,92175]
gtggagattgtgtagcccggatgctgcctttcggttgtgacccgattgtt
ggggttcgagtccccgcctccaacc
1-14tRNA-Thr(tgt)[92362,92436]
gcccttta agcatttatggtgatgcaccggctttgtaacccggcgaattc
tgttcaagtcaggaatggggcacca
>KC690136.1 Escherichia phage 2 JES-2013, complete genome
1-1tRNA-Pro(tgg)c[61326,61402]
ctccgcgtagctcagcttggtagagcgcctgatttgggatcaggaggtcg
agtgttcgaatcactccgtggagacca
1-2tRNA-Met(cat)c[61414,61488]
ggccctgtagctggaaggttcaagcaagcgactcataatcgccagacggt
ggttcaattccacccagggccacca
1-3tRNA-Thr(tgt)c[61490,61565]
gctgatgtagcacaattggtagtgcaattgatttgtaatcaataggttgc
aggttcaagtcctgccatcagcacca
1-4tRNA-Arg(tct)c[61788,61862]
gccctgttagcttagtggataaagcagcggccttctaagccgttgacact
ggttcgagtccagtacggggtgcca
>KC862300.1 Pseudomonas phage PAK_P4, complete genome
1-1tRNA-Gln(ttg)[90172,90245]
tgccgcttcgttcaatggtaggacgccagactttgaatctggagatgatg
gttcgaccccctcagcggctgcca
1-2tRNA-Arg(tct)[90451,90525]
gctcgtatagtgtaacggatgcacaacggtcttctaagccgtaaggtcta
ggttcgaatcctagtatgagcgcca
1-3tRNA-Leu(tag)[91273,91357]

ggccctgtggtggaattggtatacacatcagtcttagaaactgacgccga
gaggattgagggttcaagtccctccggggccacca
1−4tRNA−Ile(gat)[91568,91643]
ggccagatagctcaattggtagagcacccgaccgataatcgggtggttga
aggttcaagtccttctctggccacca
1−5tRNA−Asp(gtc)[91653,91731]
ggcccattagctcagtctggactagagcaagcccctgtctagggcaaggt
cgccggttcgaatccggcatgggtcgcca
1−6tRNA−Met(cat)[91832,91906]
agcgttaagactggctggagctgtcactcggctcataaccgagcacaagt
ggttcgattccacctctcgctacca
1−7tRNA−Cys(gca)[92143,92218]
cccgcgttggccgagaggtttaggcggcggattgcaaatccgtctcacat
cggttcaaatccgatacgcggctcca
1−8tRNA−Asn(gtt)[92229,92304]
tgggatgtagctcagttggtagagcagggagctgttaactctcaggtcgc
aggttcgagccctgccgtcccagcca
1−9tRNA−Pro(tgg)[92368,92445]
ctcctcgtagctcagtctggttagagtgtctgatttggaatcagaaggtc
gaaggttcaaatccttccggggtgacca
1−10tRNA−Gly(tcc)[92452,92527]
gcgggtatagctcagttggtagagcgtctgccttccaagcagttcgtcgt
cggttcaagtccgtctatccgctcca
1−11tRNA−Phe(gaa)[92534,92610]
gggactgtagctcataaggttagagcggtggattgaaatcccacaggtcg
ggcgttcgattcgccccggtcccacca
1−12tRNA−Glu(ttc)[92617,92692]
gcagttatagattaaacggttaaatcgccagactttcaatctggtgttcc
gggttcgatccccggtaactgctcca
1−13tRNA−His(gtg)[92753,92827]
gtggagattgtgtagcccggatgctgcctttcggttgtgacccgattgtt
ggggttcgagtccccgcctccaacc
1−14tRNA−Thr(tgt)[93014,93088]
gcccttttaagcatttatggtgatgcaccggctttgtaacccggcgaattc
tgttcaagtcaggaatggggcacca
>HQ163896.1 Staphylococcus phage Sb−1, complete genome
1−1tRNA−Met(cat)c[7193,7264]
ggactcttagcttaaaggtaaagccaaccgctcataacggtttgactgta
ggttcgaatcctgcagagtcca
1−2tRNA−Trp(cca)c[30349,30420]
acacccttagtataattagtagtacaagggtctccaaaacccttagtctt
tgtgcaaatcaaagagggtgtg
1−3tRNA−Phe(gaa)c[30427,30499]
ggtttcttagctcagatggtagagcactagattgaagctctaggtgtcat

tggttcaaatccaatagaaacca
1–4tRNA–Asp(gtc)c[30505,30580]
tggctcattggtgtaactggttaacacactgccctgtcacggcagagagt
acgagttcgagtctcgtatgggtcgt
>KC131129.1 Vibrio phage VH7D, complete genome
1–1tRNA–Ser(gct)c[110760,110848]
ggaagcatggtctaaaggtatgacagcaccctgctaaggtgtcggacgtt
aatagcgttctctgggttcgattcccagtgcttccgcca
1–2tRNA–Leu(caa)c[111017,111093]
gcccaactagtccaattggcagaggcactagtttcaaacactaggagttc
cgagttcgaatctcgggttgggcacca
1–3tRNA–Leu(taa)c[111210,111286]
gcgcacgtggtccaattggcagaggcatgaggcttaaaatctcagggatg
gcggttcgaatccgcccgtgcgtacca
1–4tRNA–Cys(gca)c[111289,111362]
gctcgaatcgtataatggaagtatgacggattgcaaatccgcaggtcagg
gttcgattccctgttcgagctcca
1–5tRNA–His(gtg)c[111375,111451]
gtggcagtgggtgaagtggaaatacccccgggttgtgattccggaagatg
cgggttcgatccccgtctgtcacccca
1–6tRNA–Val(tac)c[111853,111926]
ggtcccttagtataatggaagtacgtctcttttacacagagaaagaagtg
gctcgattccactagggactacca
1–7tRNA–Gly(tcc)c[111933,112006]
gcgggtatgatgtaatggtagcatgacgtccttccaagtcgttcgtctcg
gttcgagtccgtgtacccgctcca
1–8tRNA–Met(cat)c[112196,112271]
ggtgatgtagctcaagtggcagagcaacgctttcatacggcgtaagatcc
gatttcgagtatcggtgtcacctcca
1–9tRNA–Arg(tct)c[112797,112872]
gcgctgttagctcaattggaagagcacgtcccttctaaggatggggttat
gagttcgaatctcatacggcgtgcca
1–10tRNA–Thr(tgt)c[113263,113338]
gccctgatagcacaactggcagtgcagctcacttgtaatgagcaggttcg
cggttcgaatccgtgtcagggcacca
1–11tRNA–Gln(ttg)c[113466,113539]
tggggattggtgtaaaggcagcataacgtactttgaatgcgttggtatca
gttcgaatctgttatccccagcca
1–12tRNA–Asp(gtc)c[113559,113634]
ggagccgaggtgtaagtggttgcatgtctccctgtcacggagaaggtagc
gggttcgaaacccgtcggttccgcca
1–13tRNA–Asn(gtt)c[113705,113780]
ggatcgctaactcaatggttagagtgctcgcctgttaagcgaggagttcc
gagttcgagtctcgggcggtccgcca

1–14tRNA–Trp(cca)c[113856,113929]
aggggcatgatgtaaaggcagcatgacggattccaaacccgttcgttaga
gttcgaatctctatgcccctgcca
1–15tRNA–Ile(gat)c[113936,114009]
agtcccttagctcaacggtagagcgtgcgaccgataatcgcttggtaaga
gttcgattctcttagggactacca
1–16tRNA–Ser(tga)c[114073,114158]
gaaagattctgggtagcggcaacggtcttgaaaaccgtcggtcaccggga
ggtgatgttagggttcgaatccctagtcttccgcca
1–17tRNA–Met(cat)c[114183,114256]
ttgcggggtagagaagtggtcatctcgtcggtctcataagccggagaacg
ctggttcgaatccagccccacgca
1–18tRNA–Tyr(gta)c[114358,114443]
ggagcgtacgtctcaaggtgagacaggggactgtaaatccctgacccttta
acgggtagagaggttcgattcctctacgctccacca
1–19tRNA–Glu(ttc)c[114450,114524]
tctcccgtggactaacggtaggtcatcaccctttcaaggtgaagtgcgcg
agttcgaatctcgtcgggagaacca
1–20tRNA–Lys(ttt)c[114588,114664]
gcgtcggtagctcatcatggaagagcagggagcttttaactctcaggtgt
ctggttcgagtccagagcggcgtacca
1–21tRNA–Lys(ttt)c[114676,114749]
gggtctatgatgtaatggtagcatatgggacttttaatccttcagtcaga
gttcgaatctctgtggacccacca
1–22tRNA–Phe(gaa)c[114838,114912]
gcacccttagcttatcaggaaaagcggcggattgaagtcccgcagtgctc
ggttcgattccgagggggtgcacca
1–23tRNA–Leu(tag)c[114914,114990]
gcgcaagtagcccaattggcagaggcactggtcttagaaaccagaagtta
agagttcgaatctcttcttgcgtacca
1–24tRNA–Arg(acg)c[115345,115420]
gcccgattagctcaattggaagagcagcgccctacgaaggcgaaggttac
aagttcgaatcttgtattgggtgcca
1–25tRNA–Pro(tgg)c[115428,115504]
ccgtgactagctcaatctggtagagtactccgtttggggcggagaagtta
agcgttcgaatcgcttgtcacggacca
1–26tRNA–Pro(tgg)c[115514,115587]
cgggacgtggcgtaaaggtagcgttcatgctttgggagcatgtggtaaga
gttcgagtctcttcgtcccgacca
1–27tRNA–Met(cat)c[117432,117509]
ggccctatagctcaacacggttagagcaaccgactcataatcggtaggtt
acaggttcgagtcctgttggggtcacca
>KF806589.1 Erwinia phage Ea35–70, complete genome
1–1tRNA–Asn(gtt)[165191,165266]

tggtctatagttcagtcggtagaacagcggactgttaatccgtaggtcgc
tggttcgaatccagctagaccagcca

>FR775895.2 Enterobacteria phage phi92, complete genome

1-1tRNA-Met(cat)[51625,51700]
ggttccatagctcactgacgagagcgcacacctcataagtgtcgagggca
gggattgtaaccctggggaaccacca
1-2tRNA-Leu(taa)[51732,51817]
gcaagtgtggtggaattggtgatacacattgtgcttaaaacgcaacgctt
aaatgcttgagggttcgaatccctccacttgcacca
1-3tRNA-Ser(tga)[52019,52108]
ggaaggtagggcgcagcggtgcgcaaccaggtttgaaccctggcccattg
tagcgatacggtgatggttcgactccattatcttcctcca
1-4tRNA-Ser(gct)[52288,52373]
ggtagattggtgaaatggtagccacaacagtttgctaaactgtcgtcgga
aacggcgtgtaggttcaagtcctacatctaccgcca
1-5tRNA-Lys(ttt)[52508,52583]
acatcgttagcttagttggtagagcaagggtcttttaactccaaggtcga
aggttcgaatccttcacgatgtacca
1-6tRNA-Tyr(gta)[52589,52675]
gtaggtatcgcatagcggcaattgctggagactgtaaatctcttgccctt
cggggcttcgttggttcgagtccaactacctgcacca
1-7tRNA-Asn(gtt)[52683,52767]
gatggattggcctagtggttgggcgacggactgttaatccgtgaacgaaa
gttctagcaaggttcaaatccttgatccatcgcca
1-8tRNA-Thr(tgt)[52847,52921]
gctcctatcgtataacggctattacggttgccttgtaagcaacttatcag
ggttcgagtccttgtgggagcacca
1-9tRNA-Gly(tcc)[53323,53397]
gcgggtatagtgttaatggtagcatatgaggtttccaccctccaggtgca
ggttcaagtcctgttactcgctcca
1-10tRNA-Gln(ttg)[53829,53903]
tggagattggcctagtgggaaggcaacggcctttgaagtcgtgatgacta
ggttcgattcctagatctccagcca
1-11tRNA-Pro(tgg)[53911,53986]
cagtgcgtagcgcagttggtagcgtgggagccttggacgcttcaggtcgc
aggttcgagtcctgccgcactgacca
1-12tRNA-Phe(gaa)[53992,54066]
gcagagatagctgagacggattagtgctaccctgaaaaggtagaaaggat
ggatcgttaccatctctctgcacca
1-13tRNA-Leu(tag)[54144,54228]
ggctaagtggtggaatggtatacacgatacgcttagaacgtattgcccca
agggattgtgagttcgagtctcaccttagctacca
1-14tRNA-Met(cat)[54244,54322]
cgcgggatagaggagtctggtcgtcctcgccagtttcatatgctggagat

cgtaggttcaaatcctactcccgttacca

1–15tRNA–Ile(gat)[54469,54544]

tgtggtatcgtacagatggttagtacactcgaccgataatcgagaaacaa

cggttcgactccgtttaccacgacca

>KF550303.1 Enterobacteria phage 4MG, complete genome

1–1tRNA–Phe(gaa)c[60264,60339]

gggattgtaactcagttggtagagtgcctgcctgaagagcagatggtcgg

tggttcgagtccacccggtcccacca

1–2tRNA–Val(tac)c[60347,60420]

gcttccttagtttaatggtagaaccctgtctttacacggcagttgcggta

gttcgattctaccaggaagtacca

1–3tRNA–Leu(tag)c[60428,60504]

gggcgattgatggaattggtatacgtgtcggatttagattccgaattttg

ggggttcgagtcccctgtcgcccacca

1–4tRNA–Ala(tgc)c[60614,60689]

ggggctgtagtttatttggataaaatcacaggcttgcaccttgtagaact

gagttcgattctcagtggctccacca

1–5tRNA–Arg(acg)c[60694,60769]

gcaggtatagttcaactggacagagcatcagtttacgaagctgagaatcg

gggttcgaatccctgtacttgcacca

1–6tRNA–Pro(tgg)c[60776,60853]

cggagcgtagcgcagtctggtcagcgtgggtgccttggatgcactgggtc

gcaggttcgaatcctgccgctccgacca

1–7tRNA–His(gtg)c[61038,61111]

gggaatttagttaaggggcataatggccgcttgtgctgcggttgtctcct

gttcgagtcaggaagttcccgcca

1–8tRNA–Asp(gtc)c[61116,61191]

ggagatatagtttaaacggataaaatacacccctgtcacgggtatgttcc

gggttcgatgcccggtatctccgcca

1–9tRNA–Asn(gtt)c[61197,61285]

ggttctttgaccgagaggccgatggtagcaggctgttaacctgcctggag

caatccacaacgaaggttcgaatccttcaggaaccgcca

1–10tRNA–Ser(tga)c[61548,61636]

ggagagcaggctgcatggtgcagactccggtttgaacccggtcccatcgt

agcgatacggtgacagttcgattctgttgctctcctcca

1–11tRNA–Ser(gct)c[61637,61729]

ggaagattaaccctaatcaggtaagggatctctttgctaaagagacagta

gccccgaaacgggtgtgtcagttcgagtctgacatcttcctcc

1–12tRNA–Ser(gga)c[61890,61982]

ggtggaatggtcgagcggtttaagacagcaccttggaaaggtgtcggccc

cttcaaacggggtccgtaggttcgaatcctacttccaccgcca

1–13tRNA–Gln(ttg)c[62097,62172]

aggaggatggtgtaattggttagcacacggggctttgatctccggagtta

gggttcgagtccttatcttcctgcca

1-14tRNA-Trp(cca)c[62178,62254]
agggacttagcacaattggctagtgcagcggattccaaatccgcaggttc
tgggttcgaatcccagggtccctgcca
1-16tRNA-Glu(ttc)c[62340,62417]
gctgtcatcgtctaattggactaggacatcagactttcaatctgagaaat
acgggttcgaatcccgttgacagtacca
1-17tRNA-Ile(gat)c[62505,62581]
gggagtatagctcagatggatagagcgctcgcccgataagcgagaggtcg
ctggttcaatcccagctactcccacca
1-18tRNA-Tyr(gta)c[62588,62675]
ggggcggttcccggagcggcaaacgggcgggattgtaaatcccttggcat
cgcgtccttcgtaggttcgagtcctactcgccccacca
1-19tRNA-Cys(gca)c[63130,63206]
gcgcggttgccggaattggttaacggtcctgtctgcaaaataggtatttg
tcagttcgagtctgacaccgtgctcca
1-20tRNA-Arg(tct)c[63208,63282]
cggggcatagtctagaggataggcaggagtcttctaaactcctttacgtg
ggttcgaatcctactgcctcgacca
1-21tRNA-Leu(taa)c[63289,63366]
gcgctcttggtccaatctggtagaggcatgaggcttaagacttcagggtt
cccagttcgagtctgggagggcgcacca
1-22tRNA-Arg(gcg)c[63670,63743]
tcgttgatagccttagcggtaaaggccccctctgcgcaaggggttcagtg
gttcgaatccacttcagcgagcca
1-23tRNA-Thr(tgt)c[63750,63824]
gctcctaaagcataagtggcgatgcagcggccttgtaagccgaagaagtg
ggttcgattcctactgggagcacca
1-24tRNA-Lys(ctt)c[66032,66107]
ggggtattaactcagcggttagagtagcggcctcttaagccgaaggtcga
aggttcgaatccttcatgccccacca
1-25tRNA-Met(cat)c[66802,66878]
ggttctgtagcttagttggttagagcgcttccctcataaggaagaggtcg
caggttcaatccctgccagaactacca
>KC862301.1 Pseudomonas phage PAK_P5, complete genome
1-1tRNA-Asn(gtt)[86121,86197]
tccgttcggtccctcaaggtgagggagcttgactgttaatcaagacgtgc
ctggttcgattccagggagcggagcca
1-2tRNA-Tyr(gta)[86255,86342]
ggaggggtggcagagcggtttaatgcaccggactgtaaatccggcgtccg
accgggcatcgctggttcaaatccagccccctccacca
1-3tRNA-Gln(ttg)[86544,86620]
aggcgtgtggcgaaaaggtttaacgcactggactttgactccagcatttg
tgggttcgaatcccaccacgtctgcca
>AB775548.1 Pseudomonas phage PPpW-3 DNA, complete sequence

1−1tRNA−Arg(tct)c[21063,21139]
ggccccgtagctcagctggatagagcattcgccttctaagcgaacggtca
caggttcgaatcctgtcggggtcgcca

>KC862295.1 Pseudomonas phage CHA_P1, complete genome

1−1tRNA−Asn(gtt)[86241,86317]
tccgttcggtccctcaaggtgagggagcttgactgttaatcaagacgtgc
ctggttcgattccagggagcggagcca
1−2tRNA−Tyr(gta)[86375,86462]
ggaggggtggcagagcggtttaatgcaccggactgtaaatccggcgtccg
accgggcatcgctggttcaaatccagccccctccacca
1−3tRNA−Gln(ttg)[86664,86740]
aggcgtgtggcgaaaaggtttaacgcactggactttgactccagcatttg
tgggttcgaatcccaccacgtctgcca

>KJ409772.1 Pseudomonas phage phiPsa374, complete genome

1−1tRNA−Lys(ttt)[24977,25053]
tggacgttggccgaccggcgcgaggcaacaggcttttaacctgtattaac
tgagttcgattctcaggcgttcaacca
1−2tRNA−Ser(aga)[25261,25350]
ggcgggtagtgatcatggcgatcaagctgtttagaaagcagtaccattgg
ctagtacccggtgagagttcgattcccttacccgcctcca
1−3tRNA−Leu(caa)[25357,25433]
cggcagttgaagcaactggtaatcttcgtcagctcaaacctgatgtattc
tgagttcgaatctcagactgccgacca
1−4tRNA−Val(tac)[25440,25513]
gcttctttagtttaatggtagaacgccatccttacacggtggctgcggtg
gttcgattccatcaagaagcacca
1−5tRNA−Gln(ttg)[25660,25736]
tggggattagaatagatggcctaattcatcggtctttgacaccgacagca
ctggttcgaatccagtatcccctgcca
1−6tRNA−Trp(cca)[26408,26484]
aggggtgaagtgtaatggctatgcacgcgggattccaaatccttgcagac
tgggttcgattcctagcacgcctgcca
1−7tRNA−Arg(tct)[26578,26654]
ggccttatagtgtaagggaattagcacatcatccttctaagttgatagat
aaggttcgaatccttatagggctacca
1−8tRNA−Glu(ttc)[26874,26950]
gctcttgtcgtctaaaggtataggatggctgactttcaatcagttgatgt
tgggttcgagtcccaccaagagtacca
1−9tRNA−Asp(gtc)[26955,27030]
ggcttatgtttcctctgggaaggaaggcacactgtctatgtgtcagttga
gggttcgaatcccttataggtcgcca
1−10tRNA−Thr(tgt)[27091,27165]
gcccttgtcatataaaggctattatgcctgttttgtaatcaggttattgc
ggttcgaatccgtactggggcacca

1–11tRNA–Ile(gat)[27172,27246]
tgccggatagtatagtggtctgtacactcgcctgataagcgataggtgaa
agttcgattctttctctggcaacca
1–12tRNA–Arg(acg)[27253,27330]
atagctgtggcccgaactggactaggcaccatcctacgaagttggtaaat
gcaggttcgaatcctgtcagctatgcca
1–13tRNA–Asn(gtt)[27486,27562]
tggtaatagtctcccacggcgggggtctgcggctgttaaccgcaagttgc
ttggttcgattccaagattaccagcca
1–14tRNA–Asn(gtt)[27569,27655]
ttccgtgtgtaggggaactggtagacccacctgactgttaatcaggcgct
aacgcaactgtaggttcgaagcctaccaccggagcca
1–15tRNA–Pro(tgg)[27774,27847]
ctctcggaagcgttacggtagcgtactttgtttggaacgaagtggcggtg
gttcgactccaccccgagtgacca
1–16tRNA–Ala(tgc)[27991,28064]
gggctgtaagaattatgatagttcgctacgcttgcaccgtagaagccggg
gtttgactccccgacggtccacca
1–17tRNA–Leu(tag)[28324,28400]
gctcctgtaggccaactggtagagtcagtgcgcttagaacgcataaagtc
tcggttcgaatccgagtgggagtacca
1–18tRNA–Cys(gca)[28458,28532]
gtgtctttgtccgagcggctgaggtagcggattgcaaacccgtttaggtt
ggttcaactccaacaagatactcca
1–19tRNA–Gly(tcc)[28539,28614]
gcgtgattcgtatagaggtctaatactctcggcttccacccgaggaacca
cggttcgactccgtgatcccgcacca
1–20tRNA–Phe(gaa)[28620,28695]
gcactctaagctgactcggtagtagcgacggcctgaaaagtcgtaggact
tggttcgactccaagagattgcacca
>KJ101592.1 Enterobacter phage PG7, complete genome
1–1tRNA–Thr(tgt)c[72104,72179]
gccggtttagctcagttggtagagcagctcatttgtaatgagagggtcag
cggttcgaatccgttaaccggcacca
1–2tRNA–Leu(taa)c[72181,72265]
gcatcaatggtggaactggtatacacaggagacttaaaatctcccgccga
aaggattgagggttcgaatccctcttgatgcacca
1–3tRNA–Cys(gca)c[72272,72356]
gctagggttgctgagtggccaaaggcagcggattgcaaatccgccgggta
tcccttcgtgagttcgaatctcactcctagctcca
1–4tRNA–Arg(tct)c[72361,72438]
cggggtatagctcagctggatagagcagtggacttctaatccacaggtcg
acgggttcgaatccctctacctcgacca
1–5tRNA–Met(cat)c[74609,74685]

ggccctatagctcaattggttagagcacgcgactcataatcgctaggttg
caggttcgagtcctgctggggtcacca
1-6tRNA-Phe(gaa)c[75607,75681]
gggaagttagctgagatggattagcgcgtgcctgaagagcatgagaggtt
ggttcgattccaacacttcccacca
1-7tRNA-Pro(tgg)c[75683,75759]
ctccgtgtagctcagtttggtagagcatccggtttgggaccggagggtcc
gaggttcaaatcctcgcatggagacca
1-8tRNA-Gly(tcc)c[75766,75840]
gcatccatcgtatagcggctattatgactggcttccacccagtagatgag
agttcgattctctctggatgctcca
1-9tRNA-Met(cat)c[75846,75940]
acggaacgttcggatatggtagaggcatggccttcataagataaattgtg
acgtcaatcgcaatttaacctggttcgaatccaggcgttccgtac
1-10tRNA-Trp(cca)c[75991,76064]
aggtctctcgtatagtggtattatcctgagctccaacctcagtgacgtgg
gttcgattcctacggggcctgcca
1-11tRNA-Ser(tga)c[76069,76160]
ggaagcgtggtagagcggcttattacaccggtcttgaaaaccggaggccg
tagtgatacggtccgtgggttcgaatcccaccgcttcctcca
1-12tRNA-His(gtg)c[76165,76240]
gtggccgtagttcagttggtagaactcgagattgtgattctcgtagccat
gggttcaactcccatcggtcacccca
1-13tRNA-Gln(ttg)c[76455,76530]
tggggtatagccaagttggtaaggcagtagattttgattctacgattccc
tggttcgagtccaggtaccccagcca
1-14tRNA-Met(cat)c[76537,76613]
tgcgggttaacttcagttggtagaatgacgggttcatatcccgttacgcg
atggttcgagtccatcacccgcctcca
1-15tRNA-Asp(gtc)c[76771,76846]
ggacctatagtttcagcggtgaaaatactcgcctgtcacgcgagagtcac
gggttcgaatcccgttaggtccgcca
1-16tRNA-Asn(gtt)c[77091,77175]
gggtcgttggctgagagggtaagctgcggactgttaatccgtgtcagaaa
tgactaggcaggttcgatacctgcacgacccgcca
1-17tRNA-Glu(ttc)c[77181,77255]
tctccgttcgtctatcggttaggacgccggcctttcacgtcggaaagaag
agttcaattctcttacggagaacca
1-18tRNA-Lys(ttt)c[77257,77334]
aggacgttagctcagttggttagagcatccgacttttaatcggaatgtcg
atgggttcaactccctcacgtcctacca
1-19tRNA-Ile(gat)c[77373,77448]
gggaatatagctcagttggtagagcaaacgaccgataatcgttaggccac
tggttcgagtccagttattcccacca

>AB903967.1 Staphylococcus phage phiSA012 DNA, complete genome

1-1tRNA-Met(cat)c[8109,8180]

ggactcttagcttaaaggtaaagccaaccgctcataacggtttgactgta

ggttcgagtcctgcagagtcca

1-2tRNA-Phe(gaa)c[30344,30416]

ggtttcttagctcagatggtagagcactagattgaagctctaggtgtcat

tggttcaaatccaatagaaacca

1-3tRNA-Asp(gtc)c[30422,30497]

tggctcattggtgtaactggttaacacactgccctgtcacggcagagagt

acgagttcgagtctcgtatgggtcgt

>KF156340.1 Synechococcus phage S-MbCM100, complete genome

1-1tRNA-Val(tac)[13860,13931]

gggcgaataactcagcggtagagtgcctcctttacacggagattgtcggg

ggttcgatcccctcttcgccca

1-2tRNA-Leu(taa)[18850,18936]

tgggagtgtggcggaatcggtagacgcaccagacttaaaatctgttgaga

attaatctcgtgggggttcaagtccccсttctcccat

1-3tRNA-Thr(tgt)[19560,19632]

gcctccgtagctcagtggtagagcagggcttttgtaaagctcaggtcgca

agttcaaatcttgtcagaggctc

1-4tRNA-Asn(gtt)[19637,19709]

tcctctatagctcagttggtagagcaggtgactgttaatcaccctgtccc

tggttcgagtccaggtggaggag

1-5tRNA-Arg(tct)[154513,154588]

tgggtcagtagctcagatggatagagcaactgccttctaagcagtcggcc

acaggttcgagtcctgtctgacccgt

>JN624850.1 Mycobacterium phage Pleione, complete genome

1-1tRNA-Ser(gct)[31551,31634]

ggagggtgagcatctggtgatgcaggggtcctgctaaggccctacggatt

cacacccgtgagtttcgattactcctccctccgc

1-2tRNA-Leu(cag)[31731,31807]

gccctgctgagcaaactggcaaagctgccgcattcagagtgcgggtcatt

tccgggttcgactcccgggcagggtac

1-3tRNA-Leu(gag)[31927,32001]

gtctctgtaggcaaatcggaaaagccgccatcttgagggggtggtgcgtg

cgggttcgactcccgccagagacac

1-4tRNA-Leu(caa)[32002,32075]

gccgtggtaggccatctggcgagccgccgagttcaagtttcggtgtttgc

gggttcgaatcccgcccacggtac

1-5tRNA-SeC(tca)[68643,68739]

attctggcactggtgggcgagcccaccggcggcagcttcaagctgtcgct

ggccggagaaccgaccggaaacatcccgttcaacgcgacccagggcc

1-6tRNA-Pro(tgg)[91912,91986]

cggggtgtagttcagtttggaagagcgcttggtttgggaccaagttgtcg

caggttcgaatcctgtcaccccgac
1−7tRNA−Trp(cca)[92001,92071]
gggtctgtgcacagggtgcccgacggtctccaaagccgaaggcgggggtt
cgattccctccaggcctgcca
1−8tRNA−Pyl(cta)[93268,93340]
gcaccatttgctcaatggcagagcggcggcttctaaaaccgtgagtgccg
gttcgactccggcatggtgcacc
1−9tRNA−Met(cat)[93490,93564]
agcggtgtagagcagctaggtagctcgccgggctcataacccggaggacg
cgtgttcgaatcacgccaccgccac
1−10tRNA−Cys(gca)[93691,93762]
gcgcctttggcggaatggctacgtgctcggctgcaacccgagttatcccg
gttcgactccgggagggcgctc
1−11tRNA−Glu(ctc)[93767,93841]
ggtccgttggagtagatggatatctcgccaccctctcaaggtggagatca
cgggttcaagtcccgtacggactgc
1−12tRNA−His(gtg)[93843,93916]
gtggccgtagttcagccggtagaacgctgggttgtgatcccagtcgtcga
gggttcgagtccctccggtcaccc
1−13tRNA−Ala(tgc)[94078,94152]
gggcctgtagctccaattggtagagcagcatccttgcaagatgacggctg
tcggttcgaatccgacctggtccac
1−14tRNA−Phe(gaa)[94342,94414]
gccgtcatagctcagttggtagagcactggcctgaaaacccagtggccga
ggttcgattcctcgtgtcggcac
1−15tRNA−Val(cac)[94420,94493]
gtccgtttagctcagctggaagagcgctcggtccacacccgagaggccgc
aggttcgatccctgcaatggacac
1−16tRNA−Lys(ctt)[94612,94684]
gccttcgtagctcagtggtagagctgtcgcctcttaagcgataggtcgtt
ggttcgaatccagccgggggcac
1−17tRNA−Glu(ttc)[94689,94765]
ggtcgggtcggtctgctggtatggccagtcggattttcactccggacatt
cgcgggttcaattcccgtcccgatcgc
1−18tRNA−Gly(tcc)[94845,94917]
gcgggtgtggccgaatggctcaggcaccagatttccactctggctacgca
ggttcgattcctgtcatccgctc
1−19tRNA−Thr(cgt)[94977,95051]
gctgctgtagctcacctggcagagcgtcggcgtcgtatcccgaaggcatc
cggttcgagtccggacagcagcccc
1−20tRNA−Thr(tgt)[95052,95124]
gcctctgtggtccagcggcacggacatccgccttgtaagcggaggacccc
cgttcgatccggggtagaggctc
1−21tRNA−Thr(ggt)[95532,95604]

gctgggttagctcagtggtagagcgttcctctggtatgggaaaggcccgg
ggttcaatcccccgattcagctc
1-22tRNA-Gly(gcc)[96927,97000]
gcgaaggtagctcagctggcagagcgccaccttgccaaggtggaggtcgc
gggatcgtaacccgttcttcgctc
1-23tRNA-Asp(gtc)[97004,97076]
ggccctgtagctcagaggaagagcacccgcctgtcgagcgggaggtcgcg
gtatcgtaatccgtcagggtcgc
1-24tRNA-Met(cat)[97136,97208]
gcctcactagctcattggtagagccgctcgctcataacgtgcaggtacct
ggttcgattccagggtgaggtac
1-25tRNA-Ile(gat)[97214,97288]
gcctgttagcggactggtcgtccgatccaagctgataacttggcgtaagc
ggtgttcgattcaccgagcaggtac
1-26tRNA-Arg(acg)[97381,97453]
gcctctatggtccaacggagatgacgccggtctacggaaccggagatgcg
tgttcgattcgcgctaggggcac
1-27tRNA-Val(gac)[97496,97568]
gtccgtgtagctcaggggtagagcgcctgctcgacacgcaggaggaccga
ggttcgaaacctcgcatggacac
1-28tRNA-Arg(cct)[97743,97816]
gcctctgtagctcaacggacagagcaacgcggtcctaacgcggtggctgg
aggttcgaatcctctcggaggcac
1-30tRNA-Gln(ttg)[98357,98432]
tggggtatggtggcaatctggcagtcccgccggactttgactccggaggt
gcaggttcgagtcctgctaccccatc
1-31tRNA-Arg(tct)[98436,98509]
gcccttgtagctcagtggacagagcggcgagcttctacctcgcgggccgg
gagttcgaatctctccaggggcac
1-32tRNA-Lys(ttt)[98577,98653]
gggccggtatcttagtctggtcaaagaagtggacttttaatccgcgcgcc
gtgggttcgaatcccacccggcccacc
1-33tRNA-Gln(ctg)[126654,126728]
tgctcgttggtgtaactggcaacactacggactctgactccgtcatttta
ggttcgaatcctaagcgagcagcca
1-34tRNA-Asn(gtt)[126735,126810]
tggggtgtccgttaatcaggcaaacgagcggactgttaatccgcccctgc
aggttcgaatcctgccaccccagcca
>JN638751.1 Bacillus phage G, complete genome
1-1tRNA-Cys(gca)[58584,58654]
gcgtttatagccaagtggtaaggcctcggtctgcaaaaccgatatcgcga
gttcaaatctcgctaaacgct
1-2tRNA-Asp(gtc)[58662,58737]
ggctccgtggtgtagcggttaacacgcctgcctgtcacgtaggagatcgc

cggttcgaatccggtcggggccgcca
1−3tRNA−Phe(gaa)[59296,59371]
ggaggagtagctcagttggtagagcggcagattgaagatctgcgcgtcgg
gggttcaagtcccttttcctccacca
1−4tRNA−Tyr(gta)[59377,59461]
ggggagatagaccgtaagtggaagcgggcaggactgtaaatcctgtgaga
aatccttgggtgttcgattcgccctctccccacca
1−5tRNA−Glu(ttc)[59475,59550]
ggcgcgttagccaagtggtaaggcatccggctttctaccggagattacac
gagttcgaacctcgtacgcgctacca
1−6tRNA−Thr(tgt)[59556,59630]
gccggtttagctcagcggtagagcgctccccttgtaagggagatgtcgtg
ggttcaaatcctataaccggctcca
1−7tRNA−Ser(tga)[60123,60212]
ggagggttactctaattggtaagagaacagtcttgaaaactgtcgtaggt
ataaaagccgatgggggttcgagtccctcaccctccgcca
1−8tRNA−Arg(tct)[403940,404015]
tgcgcctgtagctcagctggatagagcacgagccttctaagcccgcggtc
gtaggttcgaatcctaccaggcgcgt
1−9tRNA−Ser(gct)[410128,410215]
ggggtcgtgggtgagaggctgaaaccacctccctgctaaggaggcagtcc
gcaagggctcgagggttcgaatccctccgactccgcca
1−10tRNA−Asn(gtt)[410921,410996]
tcaccagtagctcagcggtagtagcacctgactgttaatcagggggtcgt
aggttcgaatcctacctggtgagcca
1−11tRNA−Gln(ttg)[411003,411077]
tgccggatagccaagtggtaaggcaatggactttgactccatgatacaga
ggttcgaatccttttccggcagcca
1−12tRNA−His(gtg)[411235,411309]
gtggttgtagctcaattggttagagcgctagattgtggatctagaggttg
tgggttcgatccccatcatccaccc
1−13tRNA−Glu(ttc)[411320,411394]
ggggtgttggcgaagtgataacgcatccgactttctatcggacattcgcg
ggtttgaatcccgtacacccttcca
1−14tRNA−Met(cat)[411855,411926]
gctggcgggaggtaggcatctcggggagtttcatgaactctcattaattc
ggttcgaatccggagcccagca
1−17tRNA−Gly(tcc)[412560,412632]
gcgggtgtggtataacggctattatttctgccttccaagcagacgatacg
ggttcgattcccgttactcgctc
1−18tRNA−Met(cat)[414001,414080]
ggaccgttgcgccgagatggtcgaaggcacccagctcataactgggaaaa
tcaccgggggttcgactccctcacggtcca
>JN412588.1 Mycobacterium phage Dandelion, complete genome

1–1tRNA–Ser(gct)[34223,34306]
ggagggtgagcatctggtgatgcaggggtcctgctaaggccctacggatt
cacacccgtgagtttcgattactcctccctccgc
1–2tRNA–Leu(cag)[34403,34479]
gccctgctgagcaaactggcaaagctgccgcattcagagtgcgggtcatt
tccgggttcgactcccgggcagggtac
1–3tRNA–Leu(gag)[34599,34673]
gtctctgtaggcaaatcggaaaagccgccatcttgagggggtggtgcgtg
cgggttcgactcccgccagagacac
1–4tRNA–Leu(caa)[34674,34747]
gccgtggtaggccatctggcgagccgccgagttcaagtttcggtgtttgc
gggttcgaatcccgcccacggtac
1–5tRNA–SeC(tca)[70671,70767]
attctggcactggtgggcgagcccaccggcggcagcttcaagctgtcgct
ggccggagaaccgaccggaaacatcccgttcaacgcgacccagggcc
1–6tRNA–Pro(tgg)[94760,94831]
cggggtgtagtgaaaaggcatcatgctggttttgggtaccagtggttccg
gttcgagtccgggctccccgac
1–7tRNA–Trp(cca)[95270,95345]
aggggtagagccagctgggacggcagcggattccaaacccgccattgacg
gagttcgattctccgtaccccctgcca
1–8tRNA–Tyr(gta)[95347,95432]
ccgggtaagccatgttggttatggcaggcagactgtaaatctgtcgtctt
cggacttcgggggttcgattccctctacccggacca
1–9tRNA–Pyl(cta)[96492,96565]
tgcgagatcgtgcacggcgcactaggagcttctaaccctccgactcgcgg
gttcgactcccgcatctcgcaccc
1–10tRNA–Met(cat)[96713,96787]
agcggtgtagagcagctaggtagctcgccgggctcataacccggaggacg
cgtgttcgaatcacgccaccgccac
1–11tRNA–Cys(gca)[96915,96986]
gcgcctttggcggaatggctacgtgctcggctgcaacccgagttatcccg
gttcgactccgggagggcgctc
1–12tRNA–Glu(ctc)[97054,97125]
gctcccatggggtagtggtaaccctcctggttctcagccaggcgtcccga
gttcgatcctcggtgggagtgc
1–13tRNA–His(gtg)[97127,97200]
gtggccgtagttcagccggtagaacgctgggttgtgatcccagtcgtcga
gggttcgagtccctccggtcaccc
1–14tRNA–Ala(tgc)[97364,97437]
gggcctatagctcatctggtagagcgcctgccttgcaagcaggaggcggc
aggttcaagtcctgttaggtccac
1–15tRNA–Phe(gaa)[97626,97698]
gccgtcatagctcagttggtagagcaccggcctgaaaacccggtggccga

ggttcgagtcctcgtgtcggcac
1-16tRNA-Val(cac)[97704,97777]
gtccgtttagctcagctggaagagcgctcggtccacacccgagaggccgc
aggttcgatccctgcaatggacac
1-17tRNA-Lys(ctt)[97896,97968]
gccttcgtagctcagtggtagagctgtcgcctcttaagcgataggtcgtt
ggttcaaatccagccgggggcac
1-18tRNA-Glu(ttc)[97972,98046]
ggtcgggtggtctgttggcaggccggtcgggttttcaccccggacattcg
cgggttcaattcccgtcccgatcgc
1-19tRNA-Gly(tcc)[98126,98198]
gcgggtgtggccgaatggctcaggcaccagatttccactctggctacgca
ggttcgattcctgtcatccgctc
1-20tRNA-Thr(cgt)[98280,98353]
gctgctgtagctcacctggtagagcgtcggcgtcgtatcccgaaggcatc
cggttcgagtccggacagcagctc
1-21tRNA-Thr(tgt)[98427,98500]
gcccttgtagctcagtggcagagcaccgatcttgtaaatcgggttgtcga
gcgttcgattcgttcctggggctc
1-22tRNA-Thr(ggt)[99002,99076]
gctactgaggcccatatggatgggcgcggccatggtaaggccgaggtaga
tgggttcaagtccctccagtagctc
1-23tRNA-Gly(gcc)[100546,100619]
gcgaaggtagctcagctggcagagcgccaccttgccaaggtggaggtcgc
gggatcgtaacccgttcttcgctc
1-24tRNA-Asp(gtc)[100623,100695]
ggccctgtagctcagaggaagagcgccggtctgtcgaatcggaggtcgcg
gtatcgtaatccgtcagggtcgc
1-25tRNA-Met(cat)[100755,100827]
gcctcactagctcattggtagagccgctcgctcataacgtgcaggtacct
ggttcgattccagggtgaggtac
1-26tRNA-Ile(gat)[100833,100907]
gcctgttagcggactggtcgtccgatccaagctgataacttggcgtaagc
ggtgttcgattcaccgagcaggtac
1-27tRNA-Arg(acg)[101001,101073]
gcctctatggtccaacggatatgacgccggtctacggaaccggagatgcg
tgttcgattcgcgctaggggcac
1-28tRNA-Val(gac)[101116,101188]
gtccgtgtagctcaggggtagagcgcctgctcgacacgcaggaggaccga
ggttcgaaacctcgcatggacac
1-29tRNA-Arg(cct)[101362,101435]
gcctctgtagctcaacggacagagcaacgcggtcctaacgcggtggctgg
aggttcgaatcctctcggaggcac
1-31tRNA-Gln(ttg)[101976,102051]

tggggtatggtggcaatctggcagtcccgccggactttgactccggaggt
gcaggttcgagtcctgctaccccatc
1-32tRNA-Arg(tct)[102055,102130]
gcccttgtagctcagtggacagagcggcgagcttctacctcgcgggccgg
gagttcgaatctctccaggggcacca
1-33tRNA-Gln(ctg)[128819,128893]
tgctcgttggtgtaactggcaacactacggactctgactccgtcatttta
ggttcgaatcctaagcgagcagcca
1-34tRNA-Asn(gtt)[128900,128975]
tggggtgtccgttaatcaggcaaacgagcggactgttaatccgcccctgc
aggttcgaatcctgccaccccagcca

>JN699627.1 Mycobacterium phage Nappy, complete genome

1-1tRNA-Ser(gct)[31854,31937]
ggagggtgagcatctggtgatgcaggggtcctgctaaggccctacggatt
cacacccgtgagtttcgattactcctccctccgc
1-2tRNA-Leu(cag)[32034,32110]
gccctgctgagcaaactggcaaagctgccgcattcagagtgcgggtcatt
tccgggttcgactcccgggcagggtac
1-3tRNA-Leu(gag)[32230,32304]
gtctctgtaggcaaatcggaaaagccgccatcttgagggggtggtgcgtg
cgggttcgactcccgccagagacac
1-4tRNA-Leu(caa)[32305,32378]
gccgtggtaggccatctggcgagccgccgagttcaagtttcggtgtttgc
gggttcgaatcccgcccacggtac
1-5tRNA-SeC(tca)[69403,69499]
attctggcactggtgggcgggcccaccggcggcagcttcaagctgtcgct
ggccggagaaccgaccggaaacatcccgttcaacgcgacccagggcc
1-6tRNA-Pro(tgg)[92634,92705]
cggggtgtagtgaaaaggcatcatgctggttttgggtaccagtggttccg
gttcgagtccgggctccccgac
1-7tRNA-Trp(cca)[93144,93219]
aggggtagagccagctgggacggcagcggattccaaacccgccattgacg
gagttcgattctccgtacccctgcca
1-8tRNA-Tyr(gta)[93221,93304]
ccgggtaagccatgttggttatggcaggcagactgtaaatctgtcgtctt
cggacttcgggggttcgattccctctacccggac
1-9tRNA-Pyl(cta)[94096,94168]
gcaccatttgctcaatggcagagcggcggcttctaaaaccgtgagtgccg
gttcgactccggcatggtgcacc
1-10tRNA-Met(cat)[94318,94392]
agcggtgtagagcagctaggtagctcgccgggctcataacccggaggacg
cgtgttcgaatcacgccaccgccac
1-11tRNA-Cys(gca)[94519,94590]
gcgcctttggcggaatggctacgtgctcggctgcaacccgagttatcccg

gttcgactccgggagggcgctc
1-12tRNA-Glu(ctc)[94658,94729]
gctcccatggggtagtggtaaccctcctggttctcagccaggcgtcccga
gttcgatcctcggtgggagtgc
1-13tRNA-His(gtg)[94731,94804]
gtggccgtagttcagccggtagaacgctgggttgtgatcccagtcgtcga
gggttcgagtccctccggtcaccc
1-14tRNA-Ala(tgc)[94966,95040]
gggcctgtagctccaattggtagagcagcatccttgcaagatgacggctg
tcggttcgaatccgacctggtccac
1-15tRNA-Phe(gaa)[95230,95302]
gccgtcatagctcagttggtagagcactggcctgaaaacccagtggccga
ggttcgattcctcgtgtcggcac
1-16tRNA-Val(cac)[95308,95381]
gtccgtttagctcagctggaagagcgctcggtccacacccgagaggccgc
aggttcgatccctgcaatggacac
1-17tRNA-Lys(ctt)[95501,95574]
gccttcgtagctcagttggtagagctctcgcctcttaagcgagatgtcgc
aggttcgacccctgccggaggcac
1-18tRNA-Glu(ttc)[95579,95655]
ggtcgggtcggtctgctggtatggccagtcggattttcactccggacatt
cgcgggttcaattcccgtcccgatcgc
1-19tRNA-Gly(tcc)[95735,95807]
gcgggtgtggccgaatggctcaggcaccagatttccactctggctacgca
ggttcgattcctgtcatccgctc
1-20tRNA-Thr(cgt)[95867,95940]
gctgctgtagctcacctggcagagcgtcggcgtcgtatcccgaaggcatc
cggttcgagtccggacagcagccc
1-21tRNA-Thr(tgt)[96014,96087]
gcccttgtagctcagtggcagagcaccgatcttgtaaatcgggttgtcga
gcgttcgattcgttcctggggctc
1-22tRNA-Thr(ggt)[96131,96203]
gctgggttagctcagtggtagagcgttccctcggtacgggaaaggcccgg
ggttcaatcccccgactcagctc
1-23tRNA-Gly(gcc)[97526,97599]
gcgaaggtagctcagctggcagagcgccaccttgccaaggtggaggtcgc
gggatcgtaacccgttcttcgctc
1-24tRNA-Asp(gtc)[97603,97675]
ggccctgtagctcagaggaagagcgccggtctgtcgaatcggaggtcgcg
gtatcgtaatccgtcagggtcgc
1-25tRNA-Met(cat)[97735,97807]
gcctcactagctcattggtagagccgctcgctcataacgtgcaggtacct
ggttcgattccagggtgaggtac
1-26tRNA-Ile(gat)[97813,97887]

gcctgttagcggactggtcgtccgatccaagctgataacttggcgtaagc
ggtgttcgattcaccgagcaggtac
1-27tRNA-Arg(acg)[97981,98053]
gcctctatggtccaacggatatgacgccggtctacggaaccggagatgcg
tgttcgattcgcgctaggggcac
1-28tRNA-Val(gac)[98096,98168]
gtccgtgtagctcaggggtagagcgcctgctcgacacgcaggaggaccga
ggttcgaaacctcgcatggacac
1-29tRNA-Arg(cct)[98342,98415]
gcctctgtagctcaacggacagagcaacgcggtcctaacgcggtggctgg
aggttcgaatcctctcagaggcac
1-31tRNA-Gln(ttg)[98975,99050]
tggggtatggtggcaatctggcagtcccgccggactttgacttcggaggt
gcaggttcgagtcctgctaccccatc
1-32tRNA-Arg(tct)[99054,99127]
gcccttgtagctcagtggacagagcggcgagcttctacctcgcgggccgg
gagttcgaatctctccaggggcac
1-33tRNA-Lys(ttt)[99195,99271]
gggccggtatcttagtctggtcaaagaagcggacttttaatccgcgcgcc
gtgggttcgaatcccacccggcccacc
1-34tRNA-Gln(ctg)[127392,127466]
tgctcgttggtgtaactggcaacactacggactctgactccgtcattctt
ggttcgaatccagggcgagcaacca
1-35tRNA-Asn(gtt)[127473,127549]
tggggtgtagttcaatctggcagaacgctcgactgttaatcgagtagttg
aaggttcgagtccttccatcccagcca
>KJ174317.1 Salmonella phage vB_SalM_SJ2, complete genome
1-1tRNA-Ser(gct)c[96090,96178]
ggaaggttgcccgagaggtttaagggactcgactgctaatcgagtggggc
ttttagcccccgaaggttcgaatccttcaccttccgcca
1-2tRNA-Ile(gat)c[96183,96266]
gcgggagccataggggttggccagtagccgactgatcatcggcgacgatc
actggacaggttcgaatcctgttctcccgctcca
1-3tRNA-Asn(gtt)c[96741,96816]
gacgatgtagttcagtcggtagaacggcggtctgttaaatcgtatgtcgc
aggttcaagtcctgccatcgtcgcca
1-4tRNA-Met(cat)c[97683,97759]
tggtcctgtagctcagtggtaagtagcagtgaactcataattcattggtc
gttggttcaaatccaaccaggatcatc
>KJ668714.2 Escherichia phage e11/2, complete genome
1-1tRNA-Lys(ttt)c[69809,69902]
acggaacgttcggatatggtcgagatatggccttttaaaatattgagtag
cgtcaactgcttgataaccgggttcgaatcccggcgtttcgtac
1-2tRNA-Arg(tct)c[70075,70151]

cggggcatagctcaattgtatagagcaacggacttctaatccgtaggttg
aaggttagaatccttctgcctcgacca
1-3tRNA-Met(cat)c[70863,70934]
ggccctgtagctggaaggttcaagcaagcgactcataatcgccagatggt
ggttcaattccacccagggcca
1-4tRNA-Thr(tgt)c[70941,71013]
gctgatttagctcagtaggtagagcacctcacttgtaatgaggacgtcgg
cggttcgattccgccaatcagca
1-5tRNA-Ser(tga)c[71020,71108]
tggaggcgtggcagagtggtttaatgcaccggtcttgaaaaccggcagtc
gctccggcgactcataggttcgaatcctatcgcctccgt
1-6tRNA-Pro(tgg)c[71109,71183]
cttcgtgtagctcagtttggtagagcgtctgctttgggagcagaatgtcg
caggttcaaatcctgccgcggagac
1-7tRNA-Gly(tcc)c[71196,71267]
gcggatatcgtataatggcattacctcagacttccaatctgatgatgtga
gttcgattctcattatccgctc
1-8tRNA-Leu(taa)c[71274,71360]
gcgagaatggtcaaattggtaaaggcacagcacttaaaatgctgcggaat
gatttccttgtgggttcgagtcccacttctcgtacca
1-9tRNA-Gln(ttg)c[71361,71434]
tgggaattagccaagttggtaaggcatagcactttgactgctagatgcaa
aggttcgagtcctttattcccagc
>KJ451625.1 Bacillus phage Bcp1, complete genome
1-1tRNA-Ser(gct)[30794,30882]
ggaagggtactcaagttggtgaagaggtcagtttgctaaactgatagtac
ctattacaggtagcaagggttcgaacccctttccttcca
1-2tRNA-Cys(gca)[30893,30966]
gagggtgtaccgaagcggctcaacggcacagattgcaaccctgttgttcg
tgggttcaaatcccaccatcctct
1-3tRNA-Asn(gtt)[31012,31087]
gtgcttgtagctcagtcggtagagctggtggctgttaaccactgtgtcgt
aggttcgattcctacctagcacgcca
1-4tRNA-Gly(tcc)[31091,31165]
ggggcattggtatattggctattactcttggcttccaaccaagcaaggtc
ggttcgattccgacatgtccctcca
1-5tRNA-Asp(gtc)[31170,31243]
ggctcgttagtttaacggtaaaatacatgactgtctatcatgggtcacgg
gttcaactcccgtacgggtcgcca
1-6tRNA-Glu(ttc)[31252,31327]
tggcgcattggtgaagcggtttaacacactcggctttctaccgaggatgc
gtgggttcgatccccacatgcgctat
1-7tRNA-Ile(tat)[31330,31403]
atccctttagccaagtggtcaaggcagtaggattatgtcctgcgtagcgg

aggttcgactcctccaaggggcgc
1-8tRNA-Arg(tct)[31523,31598]
taccccttagccaagtggactaaggcaacgggcttctatcccgaagatc
gtgggttcaaatcctacagggggtgt
1-9tRNA-Ser(tga)[31689,31774]
cagggaatactcaaacggttaagagggtagtcttgaaaactactaggcgt
gaaagcgtgcgggggttcgaatccctcttcctcggc
1-10tRNA-Gln(ttg)[31854,31929]
tttcggtgtggacaaactggtaaagtcgtcaggctttgaccctgaagttt
ggaggttcgaccccttccaccgaagt
1-11tRNA-His(gtg)[32007,32080]
cagtgtgtggtgaagtggcttaacacggacgactgtggatcgttcattcg
caggttcgaatcctgtcatgctga
1-12tRNA-Pro(tgg)[32177,32249]
gtggatataggctagttggtcagtcactccgtttggggcggaggtcacgc
aggttcgactcctgctatccgca
1-13tRNA-Phe(gaa)[32351,32425]
ggatagatagctgagatggattagcgagtggttgaaacccactagaggtt
ggatcgttaccaactctgtccacca
1-14tRNA-Tyr(gta)[32432,32514]
gggcgtgcaatcattggagagataagctgactgtaaatcagtggtcattg
actgtgaaggttcgaatccttccattcccacca
1-15tRNA-Leu(taa)[32516,32602]
atcggaatgttggaattggtagacataacggacttaaaatccgttgctcc
ttggggcgtgagggttcgagtccctcttccgatacca
1-16tRNA-Ile(gat)[32604,32678]
actagtgtagctcagaggtagagcagtgtcttgataaggcattggtcgtt
ggttcaatcccaaccactagtacca
1-17tRNA-Leu(tag)[32686,32770]
tgtcggagtgttggaactggtatacatgcggcacttagaatgccgtgcct
tcgggattgagggttcgactccctccttcgacatc
1-18tRNA-Met(cat)[32921,32995]
tacggttatagctcagtggtagagtgtgggtctcataagcccaaggtcga
tggttcaatcccatctaaccgtatc
>KF156338.1 Synechococcus phage S-MbCM7, complete genome
1-1tRNA-Leu(taa)[17996,18079]
gcgagtatggcggaatcggtagacgcatcagacttaaaatctgctgacca
ctggtcgtgggagttcaagtctccctattcgcac
1-2tRNA-Thr(tgt)[19399,19472]
gccactttagctcagttggatagagcaacggttttgtaaaccgtaggtcg
tcggttcaagtccgacatgtggct
1-3tRNA-Asn(gtt)[19498,19572]
ttcctctatagctcagatggtagagcgagtgactgttaatcactatgtcc
ctggttcgatcccaggtggaggagt

1-4tRNA-Arg(tct)[152502,152577]
gtctcagtagctcagatggatagagcaattcacttctaatgaattggtcg
ggggttcgagtccctcctgagacgcc
1-5tRNA-Leu(tag)[152580,152662]
gtcggtatggcggaattggtagacgcgctggttttaggtaccagtgtctt
atgacgtggaggttcaagtcctcttaccgacac
1-6tRNA-Val(tac)[152665,152736]
gctcgaatagctcagaggtagagcacctcctttacacggagattgtcggg
ggttcgatcccctcttcgagca
1-7tRNA-Gly(tcc)[155268,155340]
gcgagtgtagttcagtggtagaacgttagccttccaagctaaatgtcgtc
ggttcaagtccgatctctcgctc
>JN699626.1 Mycobacterium phage MoMoMixon, complete genome
1-1tRNA-Ser(gct)[31665,31748]
ggagggtgagcatctggtgatgcaggggtcctgctaaggccctacggatt
cacacccgtgagtttcgattactcctccctccgc
1-2tRNA-Leu(cag)[31845,31921]
gccctgctgagcaaactggcaaagctgccgcattcagagtgcgggtcatt
tccgggttcgactcccgggcagggtac
1-3tRNA-Leu(gag)[32041,32115]
gtctctgtaggcaaatcggaaaagccgccatcttgagggggtggtgcgtg
cgggttcgactcccgccagagacac
1-4tRNA-Leu(caa)[32116,32189]
gccgtggtaggccatctggcgagccgccgagttcaagtttcggtgtttgc
gggttcgaatcccgcccacggtac
1-5tRNA-SeC(tca)[67969,68065]
attctggcactggtgggcgagcccaccggcggcagcttcaagctgtcgct
ggccggagaaccgaccggaaacatcccgttcaacgcgacccagggcc
1-6tRNA-Pro(tgg)[91238,91312]
cggggtgtagttcagtttggaagagcgcttggtttgggaccaagttgtcg
caggttcgaatcctgtcaccccgac
1-7tRNA-Trp(cca)[91327,91397]
gggtctgtgcacagggtgcccgacggtctccaaagccgaaggcgggggtt
cgattccctccaggcctgcca
1-8tRNA-Pyl(cta)[92594,92666]
gcaccatttgctcaatggcagagcggcggcttctaaaaccgtgagtgccg
gttcgactccggcatggtgcacc
1-9tRNA-Met(cat)[92816,92890]
agcggtgtagagcagctaggtagctcgccgggctcataacccggaggacg
cgtgttcgaatcacgccaccgccac
1-10tRNA-Cys(gca)[93017,93088]
gcgcctttggcggaatggctacgtgctcggctgcaacccgagttatcccg
gttcgactccgggagggcgctc
1-11tRNA-Glu(ctc)[93093,93167]

ggtccgttggagtagatggatatctcgccaccctctcaaggtggagatca
cgggttcaagtcccgtacggactgc
1-12tRNA-His(gtg)[93169,93242]
gtggccgtagttcagccggtagaacgctgggttgtgatcccagtcgtcga
gggttcgagtccctccggtcaccc
1-13tRNA-Ala(tgc)[93404,93478]
gggcctgtagctccaattggtagagcagcatccttgcaagatgacggctg
tcggttcgaatccgacctggtccac
1-14tRNA-Phe(gaa)[93668,93740]
gccgtcatagctcagttggtagagcactggcctgaaaacccagtggccga
ggttcgattcctcgtgtcggcac
1-15tRNA-Val(cac)[93746,93819]
gtccgtttagctcagctggaagagcgctcggtccacacccgagaggccgc
aggttcgatccctgcaatggacac
1-16tRNA-Lys(ctt)[93938,94010]
gccttcgtagctcagtggtagagctgtcgcctcttaagcgataggtcgtt
ggttcgaatccagccgggggcac
1-17tRNA-Glu(ttc)[94015,94091]
ggtcgggtcggtctgctggtatggccagtcggattttcactccggacatt
cgcgggttcaattcccgtcccgatcgc
1-18tRNA-Gly(tcc)[94171,94243]
gcgggtgtggccgaatggctcaggcaccagatttccactctggctacgca
ggttcgattcctgtcatccgctc
1-19tRNA-Thr(cgt)[94303,94376]
gctgctgtagctcacctggcagagcgtcggcgtcgtatcccgaaggcatc
cggttcgagtccggacagcagctc
1-20tRNA-Thr(tgt)[94450,94523]
gcccttgtagctcagtggcagagcaccgatcttgtaaatcgggttgtcga
gcgttcgattcgttcctggggctc
1-21tRNA-Thr(ggt)[94567,94639]
gctgggttagctcagtggtagagcgttcccttggtatgggaaaggcccgg
ggttcaatcccccgactcagctc
1-22tRNA-Gly(gcc)[95962,96035]
gcgaaggtagctcagctggcagagcgccaccttgccaaggtggaggtcgc
gggatcgtaacccgttcttcgctc
1-23tRNA-Asp(gtc)[96039,96111]
ggccctgtagctcagaggaagagcgccggtctgtcgaatcggaggtcgcg
gtatcgtaatccgtcagggtcgc
1-24tRNA-Met(cat)[96171,96243]
gcctcactagctcattggtagagccgctcgctcataacgtgcaggtacct
ggttcgattccagggtgaggtac
1-25tRNA-Ile(gat)[96249,96323]
gcctgttagcggactggtcgtccgatccaagctgataacttggcgtaagc
ggtgttcgattcaccgagcaggtac

1−26tRNA−Arg(acg)[96417,96489]
gcctctatggtccaacggatatgacgccggtctacggaaccggagatgcg
tgttcgattcgcgctaggggcac
1−27tRNA−Val(gac)[96532,96604]
gtccgtgtagctcaggggtagagcgcctgctcgacacgcaggaggaccga
ggttcgaaacctcgcatggacac
1−28tRNA−Arg(cct)[96778,96851]
gcctctgtagctcaacggacagagcaacgcggtcctaacgcggtggctgg
aggttcgaatcctctcggaggcac
1−30tRNA−Gln(ttg)[97392,97467]
tggggtatggtggcaatctggcagtcccgccggactttgactccggaggt
gcaggttcgagtcctgctaccccatc
1−31tRNA−Arg(tct)[97471,97544]
gcccttgtagctcagtggacagagcggcgagcttctacctcgcgggccgg
gagttcgaatctctccaggggcac
1−32tRNA−Lys(ttt)[97612,97688]
gggccggtatcttagtctggtcaaagaagtggacttttaatccgcgcgcc
gtgggttcgaatcccacccggcccacc
1−33tRNA−Gln(ctg)[126103,126177]
tgctcgttggtgtaactggcaacactacggactctgactccgtcatttta
ggttcgaatcctaagcgagcagcca
1−34tRNA−Asn(gtt)[126184,126259]
tggggtgtccgttaatcaggcaaacgagcggactgttaatccgcccctgc
aggttcgaatcctgccaccccagcca
>KF582788.2 Escherichia phage vB_EcoM_JS09, complete genome
1−1tRNA−Met(cat)[95924,95998]
ggccctgtagctcaatgggagagctgtcagctcataactgataggtagct
ggatcgaaaccagccagggtcacca
1−2tRNA−Arg(tct)[96002,96078]
cgaggcatagctcaattgtatagagcaacggacttctaatccgtaggttg
aaggttagaatccttctgtctcgacca
>KJ190158.1 Escherichia phage vB_EcoM_FFH2, complete genome
1−1tRNA−Pro(tgg)c[61919,61995]
ctccgcgtagctcagcttggtagagcgcctgatttgggatcaggaggtcg
agtgttcgaatcactccgtggagacca
1−2tRNA−Met(cat)c[62007,62081]
ggccctgtagctggaaggttcaagcaagcgactcataatcgccagacggt
ggttcaattccacccagggccacca
1−3tRNA−Thr(tgt)c[62083,62158]
gctgatgtagcacaatcggtagtgcaattgatttgtaatcaataggttgt
aggttcaagtcctgccatcagcacca
1−5tRNA−Ser(tga)c[62361,62451]
ggagggtagggagcaatggtgctcaagcggtcttgaaaaccgtcccgttg
aggatgactcgatgatggttcgattccattactctccgcca

1−6tRNA−Tyr(gta)c[62566,62652]
ggggagttatcccgtagaggtagcggtgtagactgtaaatctattgtcat
tgcgactcgggtggttcgactccaccactccccacca
1−7tRNA−Arg(tct)c[62657,62731]
gccctgttagcttagtggataaagcagcggccttctaagccgttgacact
ggttcgagtccagtacggggtgcca
>JN412592.1 Mycobacterium phage LinStu, complete genome
1−1tRNA−Ser(gct)[31919,32002]
ggagggtgagcatctggtgatgcaggggtcctgctaaggccctacggatt
cacacccgtgagtttcgattactcctccctccgc
1−2tRNA−Leu(cag)[32099,32175]
gccctgctgagcaaactggcaaagctgccgcattcagagtgcgggtcatt
tccgggttcgactcccgggcagggtac
1−3tRNA−Leu(gag)[32295,32369]
gtctctgtaggcaaatcggaaaagccgccatcttgagggggtggtgcgtg
cgggttcgactcccgccagagacac
1−4tRNA−Leu(caa)[32370,32443]
gccgtggtaggccatctggcgagccgccgagttcaagtttcggtgtttgc
gggttcgaatcccgcccacggtac
1−5tRNA−SeC(tca)[68341,68437]
attctggcactggtgggcgagcccaccggcggcagcttcaagctgtcgct
ggccggagaaccgaccggaaacatcccgttcaacgcgacccagggcc
1−6tRNA−Pro(tgg)[91636,91710]
cggggtgtagttcagtttggaagagcgcttggtttgggaccaagatgtcg
caggttcgaatcctgtcaccccgac
1−7tRNA−Trp(cca)[91725,91795]
gggtctgtgcacagggtgcccgacggtctccaaagccgaaggcgggggtt
cgattccctccaggcctgcca
1−8tRNA−Pyl(cta)[92932,93005]
tgcgagatcgtgcacggcgcactaggagcttctaaccctccgactcgcgg
gttcgactcccgcatctcgcaccc
1−9tRNA−Met(cat)[93154,93228]
agcggtgtagagcagctaggtagctcgccgggctcatgacccggaggacg
cgtgttcgattcacgccaccgccac
1−10tRNA−Cys(gca)[93356,93427]
gcgcctttggcggaatggctgcgtgctcggctgcaacccgagttatcccg
gttcgactccgggagggcgctc
1−11tRNA−Glu(ctc)[93495,93566]
gctcccatggggtagtggtaaccctcctggttctcagccaggcgtcccga
gttcgatcctcggtgggagtgc
1−12tRNA−His(gtg)[93621,93694]
gtggtagtagttcagatggaagaacgtcgccttgtgacggcgaaggtcgg
gggttcgaagcccctctatcaccc
1−13tRNA−Ala(tgc)[93858,93931]

gggcctatagctcatctggtagagcgcctgccttgcaagcaggaggcggc
aggttcaagtcctgttaggtccac
1-14tRNA-Phe(gaa)[94121,94193]
gccgtcatagctcagttggtagagcactggcctgaaaacccagtggccga
ggttcgagtcctcgtgtcggcac
1-15tRNA-Val(cac)[94199,94272]
gtccgtttagctcagctggaagagcgctcggcccacacccgagaggccgc
aggttcgatccctgcaatggacac
1-16tRNA-Lys(ctt)[94391,94463]
gccttcgtagctcagtggtagagctgtcgcctcttaagcgataggtcgtt
ggttcaaatccagccgggggcac
1-17tRNA-Glu(ttc)[94467,94542]
ggtcgggtggtctgttggcaggccggtcgggttttcaccccggcgtcatc
gcgggttcgattcccgtcccgactgc
1-18tRNA-Gly(tcc)[94672,94744]
gcgggtgtggccgaatggctcaggcaccagacttccactctggctaagca
ggttcgattcctgtcatccgctc
1-19tRNA-Thr(cgt)[94804,94878]
gctgctgtagctcacctggcagagcgtcggcgtcgtatcccgaaggcatc
cggttcgagtccggacagcagcccc
1-20tRNA-Thr(tgt)[94879,94951]
gcctctgtggtccagcggcacggacatccgccttgtaagcggaggacccc
cgttcgatccggggtagaggctc
1-21tRNA-Thr(ggt)[95359,95431]
gctgggttagctcagtggtagagcgttcctctggtatgggaaaggcccgg
ggttcaatcccccgattcagctc
1-22tRNA-Gly(gcc)[96754,96827]
gcgaaggtagctcagctggcagagcgccaccttgccaaggtggaggtcgc
gggatcgtaacccgttcttcgctc
1-23tRNA-Asp(gtc)[96831,96903]
ggccctgtagctcagaggaagagcgccggtctgtcgaatcggaggtcgcg
gtatcgtaatccgtcagggtcgc
1-24tRNA-Met(cat)[96963,97035]
gcctcactagctcattggtagagccgctcgctcataacgtgcaggtacct
ggttcgattccagggtgaggtac
1-25tRNA-Ile(gat)[97041,97115]
gcctgttagcggactggtcgtccgatccaagctgataacttggcgtaagc
ggtgttcgattcaccgagcaggtac
1-26tRNA-Arg(acg)[97209,97281]
gcctctatggtccaacggatatgacgccggtctacggaaccggagatgcg
tgttcgattcgcgctaggggcac
1-27tRNA-Val(gac)[97324,97396]
gtccgtgtagctcaggggtagagcgcctgctcgacacgcaggaggaccga
ggttcgaaacctcgcatggacac

1-28tRNA-Arg(cct)[97570,97643]
gcctctgtagctcaacggacagagcaacgcggtcctaacgcggtggctgg
aggttcgaatcctctcggaggcac
1-30tRNA-Gln(ttg)[98184,98259]
tggggtatggtggcaatctggcagtcccgccggactttgactccggaggt
gcaggttcgagtcctgctaccccatc
1-31tRNA-Arg(tct)[98263,98338]
gcccttgtagctcagtggacagagcggcgagcttctacctcgcgggccgg
gagttcgaatctctccaggggcacca
1-32tRNA-Gln(ctg)[124919,124993]
tgctcgttggtgtaactggcaacactacggactctgactccgtcattctt
ggttcgaatccagggcgagcaacca
1-33tRNA-Asn(gtt)[125000,125075]
tggggtgtccgttaatcaggcaaacgagcggactgttaatccgtccctgc
aggttcgaatcctgccaccccagcca
>GU396103.1 Aeromonas phage PX29, complete genome
1-1tRNA-Ala(cgc)c[84087,84161]
tcgcgattagttcagtgggagaacacctttgatcgcaaagaagtatcgag
tacggttcgaatccgttatagtgtg
1-2tRNA-Leu(tag)c[93173,93257]
gcggatgtggtgaaattggcagtcacactagatttaggatctagcgcctc
cgggcatgtcggttcgagtccgaccatccgcacca
1-3tRNA-Ser(gct)c[93264,93351]
ggagaaatgacagagaggccgaacgtagcggattgctaattcgtaggccc
gcaagagtccgtgggttcgaatcccactttctcctcca
1-4tRNA-Ser(tga)c[93708,93799]
ggaagcgtggcagagtccggcttattgcaacactcttgaaaagtgtcggc
ggttaaccccgtccatccgttcgaatcggatcgcttccgcca
1-5tRNA-Gln(ttg)c[93940,94013]
aggggtatagccaagttggtaaggcaacggactttgattccgtcatgcac
tggttcgagcccagttacccccgc
1-6tRNA-Pro(tgg)c[94126,94200]
ccgtcattagcgcagtccggtagcgcatctggtttgggatcagagggtca
taggttcaaatcctatatgacggac
1-7tRNA-Phe(gaa)c[94347,94423]
gcatcggtaactcagtatggtagagtggcggattgaaaatccgctggtca
ctggttcaagtccagtccgatgcacca
1-8tRNA-His(gtg)c[94431,94507]
gtggcggtagttcagtttggtagaacttcaggttgtgatcctgatagtca
cgggttcgaagcccgtccgccacccca
1-9tRNA-Thr(tgt)c[94589,94673]
gctcggttggcagagtggttgaatgcactggttttgtaatccagaccgaa
aggcatcgcaagttcgaatcttgtgccgagcacca
1-10tRNA-Tyr(gta)c[94683,94774]

ggatgtgctgcaatgacggtggtattgcaccagactgtaaatctggcccc
acagggtaaacgttgtaggttcgaatcctaccgcatccacca
1–11tRNA–Met(cat)c[94799,94873]
tgcgagttggaggagaggccgtcctcgctgggctcatatcccagagatca
cctgttcgaatcaggtactcgcttc
1–12tRNA–Glu(ttc)c[94894,94969]
tctccgttcgtctagtggactaggacactgccctttcacggcggtaacat
ggattcgaattccatacggagaacca
1–13tRNA–Met(cat)c[95503,95577]
ggtgcattagctcagtggtagagctgcggtttcatacgccgtcggtcagt
agttcaaatctactatgcaccacca
1–14tRNA–Lys(ttt)c[95770,95845]
ccgccgttagctcaactggtagagcaatcggcttttaaccgataggttgg
tggatcatacccaccacggcggacca
1–15tRNA–Trp(cca)c[95853,95925]
taggggcgtagcaaatcggttatgcggcggattccaaatccgttgaggtg
ggttcgactcctaccgcccctga
1–16tRNA–Cys(gca)c[96741,96815]
gcgcaggtagccaagaggtaaggcaagtgattgcaaatcactttatcgtc
tgttcgatccagatcctgtgctcca
1–17tRNA–Gly(tcc)c[96825,96898]
gcctcattggtgtagtggtcacataccgtccttccaagtcgtagtgtcgg
gttcgattcccgaatgaggctcca
1–18tRNA–Ala(tgc)c[96967,97041]
gggggtttagctcagttggaacgagcgactggtttgcaaccagtaggtca
agggttcgagccccttaacctccac
1–19tRNA–Asp(gtc)c[97317,97393]
agtgacgtagtttagttggttaaaattccggcctgtcacgtcggagagcg
cgggttcgagccccgtcgtcactgcca
1–20tRNA–Ile(gat)c[97400,97474]
agccatgtagctcagtggtagagcacacccctgataagggtggggtcggc
agttcaatcctgcccatggctacca
1–21tRNA–Asn(gtt)c[97611,97692]
ggagacgtggctcaaaggttgagcgacggactgttaatccgtgaggttaa
ctctatgctggttcgagtccagccgtctccgc
1–22tRNA–Leu(taa)c[100895,100979]
gcggatatggcgtaatcggtagccgcaggggatttaaaatcccaagtctt
cgggcgtgtgggttcgagtcccactatccgcacca
1–23tRNA–Leu(caa)c[100987,101069]
gggagattggtgtaattggtagccgcagtggattcaaaatctaccgtctt
ctgacgtaccggttcgagtccggtatctcccac
>JQ691611.1 Cronobacter phage CR9, complete genome
1–1tRNA–Thr(tgt)[148134,148209]
gctggattagctcagttggtagagcaactgatttgtaatcagaaggtcgc

gggttcgaaccctgcatccagcacca
1–2tRNA–Cys(gca)[148216,148292]
gcaacgttgccggagtctggtaacggctctcgctgcaaacgagatgagca
ctggttcgaatccagtacgttgctcca
1–3tRNA–Leu(caa)[148294,148373]
gccccctctggcccaacaaaaggtagaggcgctgctttcaaacggcaggag
ttcccggttcgaccccggggaggggcacca
1–4tRNA–Tyr(gta)[149115,149201]
gctgtgatagaccgtaggggtagcggggcagactgtaaatctgctgttca
tagaactcgggtggttcgactccatctcgcagcacca
1–5tRNA–Ser(gga)[149208,149302]
agtaggatggctgagtggcttaaagcagcatcttggaaaggtgtcggatc
aggtaacccctggtcccgggagttcaaatctccctcctactgcca
1–6tRNA–Lys(ttt)[149369,149445]
ggatcgttagctcaattggttagagcaagggacttttaatcccgaggttc
ggggttcgagtccctgacggtccacca
1–7tRNA–Asn(gtt)[149613,149688]
tgacggtgagcatatatggacatgcgggggcctgttaagcccaggataac
tggttcgaatccagtaccgtcagcca
1–8tRNA–Glu(ttc)[149740,149817]
gcacctatcgtctaattggaataggacaccgacctttcacgtcggcaaat
acgggttcgagtcccgttgggtgtacca
1–9tRNA–Asp(gtc)[149912,149987]
ggtcctatggtattagcggaaaacatactcccctgtcacgggagagtcac
gggttcgaatcccgttaggaccgcca
1–10tRNA–Ile(gat)[149994,150069]
tggggattagctcagttggtagagcgctcgaccgataatcgagaggtcac
tggttcaagtccagtatccccaacca
1–11tRNA–Pro(tgg)[150074,150150]
cggtgtgtagctcagtttggtagagcgattgctttgggagcattaggtcc
gaggttcgaatcctcgtacaccgacca
1–12tRNA–Met(cat)[150157,150231]
tgcgaggtagttcagcggtagaacaggggtctcatacgcctcatgtcggc
agttcgattctgcccttcgcaacca
1–13tRNA–Leu(tag)[150238,150315]
gctccagtagcccaattggtagaggcacttgatttaggatcatgtcagtg
atgagttcgaatctctcctggagtacca
1–14tRNA–Gly(tcc)[150324,150400]
gcatccatagtgtcagcggcctaacactccgtccttccaagtcggaaccc
tcggttcgaatccgagtggatgctcca
1–15tRNA–Gln(ttg)[150441,150516]
aggggattagtttaattggcaaaacagcgggttttgatcccgctaacgcg
aggttcgaatcctccatcccccgcca
1–16tRNA–His(gtg)[150589,150664]

gtggccttaattcagtaggtagaatccccggttgtgaaccgggcagtcgt
gggttcaagtcccacaggtcacccca
1-17tRNA-Ser(tga)[150669,150761]
ggaaggtagggcgtagtggtacgcacccggatttgaaccccggcccgctg
tggcgaccccacggtgatagttcgactctattatcttccgcca
1-18tRNA-Ser(gct)[150766,150852]
agaagagtggcgaaatcggtagccgcaccggactgctaatccggggtccc
taggggcctgagagttcaagtctctcctcttctgcca
1-19tRNA-Phe(gaa)[150950,151025]
ggaactgtagctcagttggtagagcggttgcctgaagagcatcgcgtcgg
cggttcaaatccgttcagttccacca
>KF534715.1 Pectobacterium phage PM1, complete genome
1-1tRNA-Cys(gca)[15487,15560]
gggtgcatgtcagaatggttatgtggcgtattgcaaatccgcttatactg
gttcgattccagttgtaccctcca
>KJ025957.1 Serratia phage PS2, complete genome
1-1tRNA-Gly(tcc)c[66451,66526]
gcggttgtagctcagttggtagagcttctgatttccaatcagactgtcgt
cggttcgaacccgatcagccgctcca
1-2tRNA-Arg(tct)c[68385,68460]
gcccttgtagtgtagtggatagcacacgatcgttctaaggtcggtagcct
gggttcgaatcctagcaggggtacca
1-3tRNA-Met(cat)c[68919,68992]
ggccctgtagctggacggtcaagcgagcgactcataatcgctggatggtg
gttcgattccacccagggccacca
1-4tRNA-Trp(cca)c[69518,69591]
aggggattagcctagtggctaggcagtggattccaaatccatctagagag
gttcgattcctctatcccctgcca
>KJ489400.1 Bacillus phage Hoody T, complete genome
1-1tRNA-Asn(gtt)[32368,32442]
tgtgcttgtagctcagtcggtagagctggtggctgttaaccactgtgtcg
taggttcgagccctacctagcacgt
1-2tRNA-Gln(ttg)[32448,32520]
tgcctcttagccaagcggtaaggcacgggactttgactctcggacgcgct
agttcgaatctagcagaggcatc
1-3tRNA-Trp(cca)[32689,32762]
gggagtttagtttagtggtaaaacctcggtctccaaaaccgaagtcatat
gttcgattcgtatagctcctgcca
1-4tRNA-Ser(tga)[32764,32851]
ggagggttggcagagtggattattgcagcggtcttgaaaaccgccgaggg
taaaacctccgtgagttcaaatctcacaccctcctcca
1-5tRNA-Asp(gtc)[32853,32926]
tggggatatagtgtagaggtaaacacgcacggctgtctaccgtgaagcac
gggttcgaatcccgttatcctcgt

1-6tRNA-Leu(tag)[33071,33148]
tgccgaagtaatccaatcaggtagagatagcggttttagaaaccgtccag
tgtgggttcgaccccctccttcggtatc
1-7tRNA-Tyr(gta)[33155,33238]
tggcagaatagtcaagcggcaacgacaggtgactgtaaatcacccctcat
tcgagttctcaggttcgagtcctgattctgctat
1-8tRNA-Ser(gct)[34151,34237]
ggaggggtactcaagcggcgaagagggggacttgctaagtctctagtacc
ttaacaggtagcgagggttcgaatcccttcccctcct
>KJ489397.1 Bacillus phage CAM003, complete genome
1-1tRNA-Asn(gtt)[32547,32621]
tgtgcttgtagctcagtcggtagagctggtggctgttaaccactgtgtcg
taggttcgagccctacctagcacgt
1-2tRNA-Gln(ttg)[32627,32699]
tgcctcttagccaagcggtaaggcacgggactttgactctcggacgcgct
agttcgaatctagcagaggcatc
1-3tRNA-Trp(cca)[32868,32941]
aggagtttagtttagtggtaaaacctcggtctccaaaaccgaagtcatat
gttcgattcgtatagctcctgcca
1-4tRNA-Ser(tga)[32943,33030]
ggagggttggcagagtggattattgcagcggtcttgaaaaccgccgaggg
taaaacctccgtgagttcaaatctcacaccctcctcca
1-5tRNA-Asp(gtc)[33032,33105]
tggggatatagtgtagaggtaaacacgcacggctgtctaccgtgaagcac
gggttcgaatcccgttatcctcgt
1-6tRNA-Leu(tag)[33250,33327]
tgccgaagtaatccaatcaggtagagatagcggttttagaaaccgtccag
tgtgggttcgaccccctccttcggtatc
1-7tRNA-Tyr(gta)[33334,33417]
tggcagaatagtcaagcggcaacgacaggtgactgtaaatcacccctcat
tcgagttctcaggttcgagtcctgattctgctat
1-8tRNA-Ser(gct)[34330,34416]
ggaggggtactcaagcggcgaagagggggacttgctaagtctctagtacc
ttaacaggtagcgagggttcgaatcccttcccctcct
>KJ094033.1 Listeria phage LP-048, complete genome
1-1tRNA-Met(cat)c[27810,27883]
ttgtcccgtagctagaaggtcgagcaaggagctcataactcctcggtttg
ggttcgattcccaacggggcaatc
1-2tRNA-Pro(tgg)c[28776,28850]
cagggtgtagctcagtttggttagagtacccgctttggagacgggaagtc
gtaggttcgaatcctaccaccttga
1-3tRNA-Arg(tct)c[29970,30040]
gtccttatggtgtagtggatgcacaagggatttctactcccttagcgcgg
gttcgaatcctgctgaggact

1-4tRNA-Gly(tcc)c[30298,30368]
gcgggtatagtataagggtagtacccaaggtttccaaccatgtagtgggg
gttcgaatccccctacccgct
1-5tRNA-Asn(gtt)c[30437,30510]
gtgtccttaactcagaggtcagagtgccgtcctgttaagtcggaagtcgc
tggttcaaatccagcaggatacgc
1-6tRNA-Ser(tga)c[31132,31223]
ggaaggttggtagagcttggtaatacgctagtcttgaaaactagtttgcc
ctcgtaatacagggtacaagggttcgaatcccttaccttcct
1-7tRNA-Phe(gaa)c[31236,31307]
gtagtcctagctgagatggattagcgcttgcttgaaaagcaagagaggca
ggttcgatacctgcggactcca
1-8tRNA-Lys(ttt)c[31313,31384]
ggagttatggtgaaatggctatcactgcgggtttttaccccgttattcta
ggttcgaatcctagtggctcca
1-9tRNA-Trp(cca)c[31509,31582]
taggggtatagtttatctggtaaaatattggtttccaactccaatgaggt
gggttcaagtcctactatccctgt
1-10tRNA-Gln(ttg)c[31584,31656]
tggtctgtagccaagcggtaaggcaacggattttgattccgtgatacgtt
ggttcgaatccaactagaccagc
1-11tRNA-Thr(tgt)c[31675,31746]
gcttgtatagttcaattggtagaacagtggttttgtaagcctcagacgtg
ggttcaagtcctactacaagca
1-12tRNA-Tyr(gta)c[31829,31910]
gtgtcattcgcatagaggcaattgcaggggattgtaactcccctcccttc
ggggttccaaggttcgagtccttgatggcaca
1-13tRNA-Leu(tag)c[32229,32313]
tgccgagatggtggaactggtatacacggtagacttagaatctgctgtcc
caaggatatgtgggttcgaatcccactctcggtat
1-14tRNA-Asp(gtc)c[32779,32851]
gtgcgtatgatataatggctattatactcgactgtctatcgagaaatagg
ggttcgattccccttacgtgcgc
1-15tRNA-Ile(gat)c[32955,33027]
accagcatagcttaggaggcaaagcaaccgaccgataatcggtagtcctt
ggttcaattccaagtgttggtac
1-16tRNA-Ser(gct)c[33204,33290]
ggagagttgtcagataggcttaatgatacgggttgctaactcgttgtact
agtaatagtaccaagggttcgaatcccttactctcct
1-17tRNA-Cys(gca)c[33371,33441]
gcgggtataaccaactggaaaggtagtagactgcaaatctacgtatatgg
gttcaattcccattacccgct
>KM051843.1 Bacillus phage Bobb, complete genome
1-1tRNA-Met(cat)c[128786,128860]

tgggtctctagcttagtggtaaagctgccagctcataactggtaagagag
gggttcgaatcccttgggacccatc
1−2tRNA−Trp(cca)c[129919,129993]
tgagggtctagctcaactggtagagcgtcggtctccaaaaccgaaggtta
caggttcgagtcctgtggctcttgt
1−3tRNA−His(gtg)c[130822,130892]
gagaatgtagcttaacggtaaagcgctgggacgtgactccagctatccaa
gttcgattcttggtattctca
1−4tRNA−Gly(tcc)c[130903,130976]
gtggtcttagtttaatggtagaacagtcggtttccacccgacagatatgg
gttcaactcccttagaccatacca
1−5tRNA−Met(cat)c[132152,132225]
tacgatgctgaggggtcggtacccgacgaggtctcataagccttgtttag
taagttcaactcttgccttagcaa
1−6tRNA−Phe(gaa)c[132299,132373]
tggactggtagctcagttggtagagcagggggctgaaaatccctgtgtcg
gaggttcgaatccttctcagtccat
1−7tRNA−Asn(gtt)c[132750,132823]
ttgtcgtataactcaatggtagagtgcttgactgttaatcaagaagttgt
aggttcgagtcctactacgacagt
>KJ174318.1 Salmonella phage vB_SalM_SJ3, complete genome
1−1tRNA−Ser(gct)c[81717,81805]
ggaaggttgcccgagaggtttaagggactcgactgctaatcgagtggggc
ttttagcccccgaaggttcgaatccttcaccttccgcca
1−2tRNA−Tyr(gta)c[81881,81962]
gtgagtgtggcagagcggtcgaatgcgcctgactgtaaatcaggtatccc
acgcggtggttcaaatccatccactcacacca
1−3tRNA−Asn(gtt)c[82719,82794]
gacgatgtagttcagtcggtagaacggcggtctgttaaaccgtatgtcgc
aggttcaagtcctgccatcgtcgcca
1−4tRNA−Met(cat)c[83207,83282]
ggccccgtagctcagtggttagagcagtcgactcataatcgattggtcgc
tggttcaagtccagccagggtcacca
>KJ489398.1 Bacillus phage Evoli, complete genome
1−1tRNA−Asn(gtt)[32278,32352]
tgtgcttgtagctcagtcggtagagctggtggctgttaaccactgtgtcg
taggttcgagccctacctagcacgt
1−2tRNA−Gln(ttg)[32358,32430]
tgcctcttagccaagcggtaaggcacgggactttgactctcggacgcgct
agttcgaatctagcagaggcatc
1−3tRNA−Trp(cca)[32599,32672]
gggagtttagtttagtggtaaaacctcggtctccaaaaccgaagtcatat
gttcgattcgtatagctcctgcca
1−4tRNA−Ser(tga)[32674,32761]

ggagggttggcagagtggattattgcagcggtcttgaaaaccgccgaggg
taaaacctccgtgagttcaaatctcacaccctcctcca
1-5tRNA-Asp(gtc)[32763,32836]
tggggatatagtgtagaggtaaacacgcacggctgtctaccgtgaagcac
gggttcgaatcccgttatcctcgt
1-6tRNA-Leu(tag)[32981,33058]
tgccgaagtaatccaatcaggtagagatagcggttttagaaaccgtccag
tgtgggttcgaccccctccttcggtatc
1-7tRNA-Tyr(gta)[33065,33148]
tggcagaatagtcaagcggcaacgacaggtgactgtaaatcacccctcat
tcgagttctcaggttcgagtcctgattctgctat
1-8tRNA-Ser(gct)[34061,34147]
ggaggggtactcaagcggcgaagagggggacttgctaagtctctagtacc
ttaacaggtagcgagggttcgaatcccttcccctcct
>KJ535721.1 Listeria phage List-36, complete genome
1-1tRNA-Cys(gca)[97557,97627]
gcgggtataaccaactggaaaggtagtagactgcaaatctacgtatatgg
gttcaattcccattacccgct
1-2tRNA-Ser(gct)[97706,97792]
ggagagttgtcagagaggcttaatgatacgggttgctaactcgctgtact
agtaatagtaccaagggttcgaatcccttactctcct
1-3tRNA-Ile(gat)[97969,98041]
gccagcatagcttagtaggcaaagcaaccgaccgataatcggtagtcctt
ggttcaattccaagtgttggtac
1-4tRNA-Asp(gtc)[98145,98217]
gtgcgtatgatataatggctattatactcgactgtctatcgagaaatagg
ggttcgattccccttacgtgcgc
1-5tRNA-Leu(tag)[98683,98767]
tgccgagatggtggaactggtatacacggtagacttagaatctgctgtcc
taaggatatgtgggttcgaatcccactctcggtat
1-6tRNA-Tyr(gta)[99128,99209]
gtgccattcgcatagaggcaattgcaggggactgtaactcccctcccttc
ggggttccaaggttcgagtccttgatggcgca
1-7tRNA-Thr(tgt)[99292,99363]
gcttgtatagttcaattggtagaacagtggttttgtaagcctcagacgtg
ggttcaagtcctactacaagca
1-8tRNA-Gln(ttg)[99382,99454]
tggtctgtagccaagcggtaaggcaacggattttgattccgtgatacgtt
ggttcgaatccaactagaccagc
1-9tRNA-Trp(cca)[99456,99529]
taggggtatagtttatctggtaaaatattggtttccaactccaatgaggt
gggttcaagtcctactatccctgt
1-10tRNA-Lys(ttt)[99654,99725]
ggagttatggtgaaatggctatcactgcgggtttttaccccgttattcta

ggttcgaatcctagtggctcca
1-11tRNA-Phe(gaa)[99731,99802]
gtagtcctagctgagatggattagcacttgcttgaaaagcaagagaggca
ggttcgatacctgcggactcca
1-12tRNA-Ser(tga)[99815,99906]
ggaaggttggtagagcttggtaatacgctagtcttgaaaactagtttgcc
cttggaatacagggtacaagggttcaaatcccttaccttcct
1-13tRNA-Asn(gtt)[100528,100601]
gtgtccttaactcagaggtcagagtgccgtcctgttaagtcggaagtcgc
tggttcaaatccagcaggatacgc
1-14tRNA-Gly(tcc)[100670,100740]
gcgggtatagtataagggtagtacccaaggtttccaaccatgtagtgggg
gttcgaatccccctacccgct
1-15tRNA-Arg(tct)[100987,101057]
gtccttatggtgtagtggatgcacaagggatttctactcccttagcgcgg
gttcgaatcctgctgaggact
1-16tRNA-Pro(tgg)[102175,102249]
cagggtgtagctcagtttggttagagtacccgctttggagacgggaagtc
gtaggttcgagtcctaccaccttga
1-17tRNA-Met(cat)[103142,103215]
ttgtcccgtagctagaaggtcgagcaaggagctcataactcctcggtttg
ggttcgattcccaacggggcaatc
>KJ535722.1 Listeria phage LMSP-25, complete genome
1-1tRNA-Met(cat)c[41701,41774]
ttgtcccgtagctagaaggtcgagcaaggagctcataactcctcggtttg
ggttcgattcccaacggggcaatc
1-2tRNA-Pro(tgg)c[42667,42741]
cagggtgtagctcagtttggttagagtacccgctttggagacgggaagtc
gtaggttcgaatcctaccaccttga
1-3tRNA-Arg(tct)c[43859,43929]
gtccttatggtgtagtggatgcacaagggatttctactcccttagcgcgg
gttcgaatcctgctgaggact
1-4tRNA-Gly(tcc)c[44176,44246]
gcgggtatagtataagggtagtacccaaggtttccaaccatgtagtgggg
gttcgaatccccctacccgct
1-5tRNA-Asn(gtt)c[44315,44388]
gtgtccttaactcagaggtcagagtgccgtcctgttaagtcggaagtcgc
tggttcaaatccagcaggatacgc
1-6tRNA-Ser(tga)c[44626,44717]
ggaaggttggtagagcttggtaatacgctagtcttgaaaactagcttacc
cttggaatacagggtacaagggttcgaatcccttaccttcct
1-7tRNA-Phe(gaa)c[44730,44801]
gtagtcctagctgagatggattagcgcttgcttgaaaagcaagagaggca
ggttcgatacctgcggactcca

1-8tRNA-Lys(ttt)c[44807,44878]
ggagttatggtgaaatggctatcactgcgggtttttaccccgttattcta
ggttcgaatcctagtggctcca
1-9tRNA-Tyr(gta)c[45202,45283]
gtgtcattcgcatagtggcaattgcaggggactgtaactcccctcccttc
ggggttccaaggttcgagtccttgatggcgca
1-10tRNA-Leu(tag)c[45644,45728]
tgccgagatggtggaactggtatacacggtagacttagaatctgctgtcc
caaggatatgtgggttcgaatcccactctcggtat
1-11tRNA-Asp(gtc)c[46194,46266]
gtgcgtatgatataatggctattatactcgactgtctatcgagaaatagg
ggttcgattccccttacgtgcgc
1-12tRNA-Ile(gat)c[46370,46442]
gccagcatagcttagtaggcaaagcaaccgaccgataatcggtagtcctt
ggttcaattccaagtgttggtac
1-13tRNA-Ser(gct)c[46619,46705]
ggagagttgtcagagaggcttaatgatacgggttgctaactcgttgtact
agtaatagtaccaagggttcgaatcccttactctcct
1-14tRNA-Cys(gca)c[46785,46855]
gcgggtataaccaactggaaaggtagtagactgcaaatctacgtatatgg
gttcaattcccattacccgct
>KC954774.1 Cronobacter phage CR8, complete genome
1-1tRNA-Thr(tgt)[144252,144327]
gctggattagctcagtaggtagagcgcctcacttgtaatgaggatgtcgc
gggttcgattcctgcatccagcacca
1-2tRNA-Cys(gca)[144334,144410]
gcaacgttgccggagtctggtaacggctctcgctgcaaacgagatgagca
ctggttcgaatccagtacgttgctcca
1-3tRNA-Leu(caa)[144412,144488]
gcgtctttggtccaattggtagaggcactacattcaaaatgtaggggttc
ccagttcgaatctgggaaggcgcacca
1-4tRNA-Ser(gga)[145194,145288]
agtaggatggctgagtggcttaaagcagcaccttggaaaggtgtcggatc
gggcaacccctggtcccgggagttcaaatctccctcctactgcca
1-5tRNA-Lys(ttt)[145293,145369]
ggatcgttagctcaattggttagagcaagggacttttaatcccgaggttc
ggggttcgagtccctgacggtccacca
1-6tRNA-Asn(gtt)[145535,145610]
tggtctttcgtataatcggttattacggcggactgttaatccgtttatcc
gggttcgagtcccggaggaccagcca
1-7tRNA-Glu(ttc)[145649,145727]
gtcctggtagacaaacttggttaaagtcaccaccctttcaaggtggagtt
tgagggttcgatcccctctcaggacgcca
1-8tRNA-Asp(gtc)[145822,145897]

ggtcctatggtatcagcggtaaacatactcgcctgtcacgcgagagtcac
gggttcgaatcccgttaggaccgcca
1−9tRNA−Ile(gat)[146579,146654]
tggggattagctcagttggtagagcgctcgaccgataatcgagaggtcac
tggttcaagtccggtatccccaacca
1−10tRNA−Pro(tgg)[146725,146801]
cggtgtatagcgcagcctggtagcgcatctggtttgggaccagagggtcg
ctggttcgattccagctacaccgacca
1−11tRNA−Met(cat)[146808,146882]
tgcgaggtagttcagcggtagaacaggggtttcatacgcctcatgtcggc
agttcgattctgcccttcgcaacca
1−12tRNA−Leu(tag)[146888,146965]
gctccagtagcccaattggtagaggcacttgatttaggatcatgtcagtg
atgagttcgaatctctcctggagtacca
1−13tRNA−Gly(tcc)[146972,147045]
gcgggcatggtatagtggttgtgccttagccttccaagctaatgagtccg
gttcgatcccggatgcccgctcca
1−14tRNA−Gln(ttg)[147086,147161]
aggggattagtttaattggtaaaacatcgggttttgatcccgacatcgtg
aggttcgaatcctccatcccccgcca
1−15tRNA−His(gtg)[147244,147319]
gtggccttaattcagaaggtagaatccccggttgtgaaccgggcagtcgt
gggttcaagtcccacaggtcacccca
1−16tRNA−Ser(tga)[148000,148092]
ggaagatagggcgtagtggtacgcacccggatttgaaccccggcccgctg
tggcgaccccacggtgatagttcgactctattatcttccgcca
1−17tRNA−Ser(gct)[148097,148183]
agaagagtggcgaaatcggtagccgcaccggactgctaatccggggtccc
tcggggcctgagagttcaagtctctcctcttctgcca
1−18tRNA−Phe(gaa)[148189,148264]
ggaactgtagctcagttggtagagcggttgcctgaagagcagcgcgtcag
cggttcaaatccgttcagttccacca
>KJ094030.1 Listeria phage LP−083−2, complete genome
1−1tRNA−Met(cat)c[31951,32024]
ttgtcccgtagctagaaggtcgagcaaggagctcataactcctcggtttg
ggttcgattcccaacggggcaatc
1−2tRNA−Pro(tgg)c[32917,32991]
cagggtgtagctcagtttggttagagtacccgctttggagacgggaagtc
gtaggttcgagtcctaccaccttga
1−3tRNA−Arg(tct)c[34109,34179]
gtccttatggtgtagtggatgcacaagggatttctactcccttagcgcgg
gttcgaatcctgctgaggact
1−4tRNA−Gly(tcc)c[34437,34507]
gcgggtatagtataagggtagtacccaaggtttccaaccatgtagtgggg

gttcgaatccccctacccgct
1-5tRNA-Asn(gtt)c[34576,34649]
gtgtccttaactcagaggtcagagtgccgtcctgttaagtcggaagtcgc
tggttcaaatccagcaggatacgc
1-6tRNA-Ser(tga)c[35271,35362]
ggaaggttggtagagcttggtaatacgctagtcttgaaaactagtttgcc
cttggaatacagggtacaagggttcaaatcccttaccttcct
1-7tRNA-Phe(gaa)c[35375,35446]
gtagtcctagctgagatggattagcgcttgcttgaaaagcaagagaggca
ggttcgatacctgcggactcca
1-8tRNA-Lys(ttt)c[35452,35523]
ggagttatggtgaaatggctatcactgcgggtttttaccccgttattcta
ggttcgaatcctagtggctcca
1-9tRNA-Trp(cca)c[35648,35721]
taggggtatagtttatctggtaaaatattggtttccaactccaatgaggt
gggttcaagtcctactatccctgt
1-10tRNA-Gln(ttg)c[35723,35795]
tggtctgtagccaagcggtaaggcaacggattttgattccgtgatacgtt
ggttcgaatccaactagaccagc
1-11tRNA-Thr(tgt)c[35814,35885]
gcttgtatagttcaattggtagaacagtggttttgtaagcctcagacgtg
ggttcaagtcctactacaagca
1-12tRNA-Tyr(gta)c[35968,36049]
gtgccattcgcatagaggcaattgcaggggactgtaactcccctcccttc
ggggttccaaggttcgagtccttgatggcgca
1-13tRNA-Leu(tag)c[36410,36494]
tgccgagatggtggaactggtatacacggtagacttagaatctgctgtcc
taaggatatgtgggttcgaatcccactctcggtat
1-14tRNA-Asp(gtc)c[36960,37032]
gtgcgtatgatataatggctattatactcggctgtctatcgagaaatagg
ggttcgattccccttacgtgcgc
1-15tRNA-Ile(gat)c[37136,37208]
gccagcatagcttagtaggcaaagcaaccgaccgataatcggtagtcctt
ggttcaattccaagtgttggtac
1-16tRNA-Ser(gct)c[37385,37471]
ggagagttgtcagagaggcttaatgatacgggttgctaactcgttgtact
agtaatagtaccaagggttcgaatcccttactctcct
1-17tRNA-Cys(gca)c[37550,37620]
gcgggtataaccaactggaaaggtagtagactgcaaatctacgtatatgg
gttcaattcccattacccgct
>KJ591604.1 Listeria phage LMTA-148, complete genome
1-1tRNA-Met(cat)c[34489,34562]
ttgtcccgtagctagaaggtcgagcaaggagctcataactcctcggtttg
ggttcgattcccaacggggcaatc

1−2tRNA−Pro(tgg)c[35455,35529]
cagggtgtagctcagtttggttagagtacccgctttggagacgggaagtc
gtaggttcgaatcctaccaccttga
1−3tRNA−Arg(tct)c[36649,36719]
gtccttatggtgtagtggatgcacaagggatttctactcccttagcgcgg
gttcgaatcctgctgaggact
1−4tRNA−Gly(tcc)c[36966,37036]
gcgggtatagtataagggtagtacccaaggtttccaaccatgtagtgggg
gttcgaatccccctacccgct
1−5tRNA−Asn(gtt)c[37105,37178]
gtgtccttaactcagaggtcagagtgccgtcctgttaagtcggaagtcgc
tggttcaaatccagcaggatacgc
1−6tRNA−Leu(tag)c[37717,37801]
tgccgagatggtggaactggtatacacggtagacttagaatctgctgtcc
caaggatatgtgggttcgaatcccactctcggtat
1−7tRNA−Asp(gtc)c[38267,38339]
gtgcgtatgatataatggctattatactcgactgtctatcgagaaatagg
ggttcgattccccttacgtgcgc
1−8tRNA−Ile(gat)c[38443,38515]
accagcatagcttaggaggcaaagcaaccgaccgataatcggtagtcctt
ggttcaattccaagtgttggtac
1−9tRNA−Ser(gct)c[38692,38778]
ggagagttgtcagagaggcttaatgatacgggttgctaactcgttgtact
agtaatagtaccaagggttcgaatcccttactctcct
1−10tRNA−Cys(gca)c[38857,38927]
gcgggtataaccaactggaaaggtagtagactgcaaatctacgtatatgg
gttcaattcccattacccgct
>AB981169.1 Ralstonia phage RSY1 DNA, complete genome
1−1tRNA−Pro(cgg)c[18954,19043]
gcccacggtcggcgggttccaggtgcgcgtccggcccgcgcggcgctcgg
cccacgggcgccaggtggtggtgaggccaccggtgcgcac
>KJ888149.1 Staphylococcus phage MCE−2014, complete genome
1−1tRNA−Met(cat)c[16397,16468]
ggactcttagcttaaaggtaaagccaaccgctcataacggtttgactgta
ggttcgaatcctacagagtcca
1−2tRNA−Trp(cca)c[38715,38786]
acacccttagtataattagtagtacaagggtctccaaaacccttagtctt
tgtgcaaatcaaagagggtgtg
1−3tRNA−Phe(gaa)c[38793,38865]
ggtttcttagctcagatggtagagcactagattgaagctctaggtgtcat
tggttcaaatccaatagaaacca
1−4tRNA−Asp(gtc)c[38871,38946]
tggctcattggtgtaactggttaacacactgccctgtcacggcagagagt
acgagttcgagtctcgtatgggtcgt

>KC012913.1 Staphylococcus phage Team1, complete genome
1−1tRNA−Met(cat)c[14737,14808]
ggactcttagcttaaaggtaaagccaaccgctcataacggtttgactgta
ggttcgaatcctgcagagtcca
1−2tRNA−Trp(cca)c[37954,38025]
acacccttagtataattagtagtacaagggtctccaaaacccttagtctt
tgtgcaaatcaaagagggtgtg
1−3tRNA−Phe(gaa)c[38032,38104]
ggtttcttagctcagatggtagagcactagattgaagctctaggtgtcat
tggttcaaatccaatagaaacca
1−4tRNA−Asp(gtc)c[38110,38185]
tggctcattggtgtaactggttaacacactgccctgtcacggcagagagt
acgagttcgagtctcgtatgggtcgt
>KJ716335.1 Dickeya phage RC−2014, complete genome
1−1tRNA−Met(cat)[108684,108760]
gatggtgtagttcagttggttagaacgtgcgactcataatcgctttgtca
ctggttcaagtcctgccgccatcgcca
>JX128257.1 Escherichia phage ECML−4, complete genome
1−1tRNA−Ile(gat)c[75600,75675]
tgcttcatagctcagtaggtagagcgttcccctgataagggagaggtcac
tggttcgaatccagttgaagcaacca
1−2tRNA−Ser(gct)c[75679,75767]
ggaaggttgcccgagaggtttaagggactcgactgctaatcgagtggggc
ttttagcccccgaaggttcgaatccttcaccttccgcca
1−3tRNA−Tyr(gta)c[76053,76136]
gtgagtgtggcagagcggtcgaatgcaggagactgtaaatctccccgtaa
cagcgcggtggttcgaatccatccactcacacca
1−4tRNA−Asn(gtt)c[76393,76468]
gacgatgtagttcagtcggtagaacggtggactgttaatccatatgtcgc
aggttcaagtcctgccatcgtcgcca
1−5tRNA−Met(cat)c[76788,76863]
ggccccgtagctcagtggttagagcagtcgactcataatcgattggtcgc
tggttcaagtccagccagggtcacca
>KJ473422.1 Acinetobacter phage vB_AbaM_Acibel004, complete genome
1−1tRNA−Ser(tga)[59973,60061]
ggtagatagcgtcaaggtgacaaagtggatttgaaatccatgctattggt
tagtagccgataagggttcgattcctttatctacctcca
1−2tRNA−Tyr(gta)[60064,60151]
gggagttagacgactgttggttggtcgtagcagactgtaaatctgccctc
acaaagcatcattggttcgattccaatagctcccacca
1−3tRNA−Ser(gct)[60159,60247]
agagaataaatagcttaggtagctagactccctgctaaggagatcgactg
attaaatcggtttcgtttcgagtacgatgttctctgcca
1−4tRNA−Arg(tct)[60663,60738]

acgtcattggtcgagcggattaggcatcacacttctaatgtgacttacag
gggttcgaatcctctatggcgtacca
1−5tRNA−Phe(gaa)[60826,60900]
gggttggtaactcagtggtagagtgactgtctgaagaacagtaggtcgta
agttcgattcttacccttcccacca
1−6tRNA−Met(cat)[61018,61092]
agcggtatagtgtaataggtagcacgaaaggctcataaccttttagttcg
ggttcgattcccgataccgcaacca
1−7tRNA−Ile(gat)[61099,61174]
ggtcgattagctcagttggtagagcagtagcccgataagctattggtcgc
tggttcaaatccagcatcgactacca
1−8tRNA−Asn(gtt)[61181,61266]
tcttgtgtagacaaactggtaaagtcagtggactgttaatccaccgctta
attgcactgtaggttcgaagcctaccacaagagcca
1−9tRNA−Pro(tgg)[61273,61349]
cagggtatagggcagtctggtagcctatttcttttggaaagaaaatgtcg
atggttcaaatccatctaccttgacca
1−10tRNA−Gln(ttg)[61562,61637]
tcatgtggaagtatttacaggagatgcactatcggttggcggtaggttaa
gttggttcgaatccaaccccatgacc
1−11tRNA−Asp(gtc)[61689,61764]
ggctctgtagcatagcggattatgcgcctccctgtctaggagaaaaacga
gggttcgagtcccttcagagtcgcca
1−12tRNA−Thr(tgt)[61994,62067]
gctggtatcgtataatggtattacgcttgttttgtaatcaagtcatggca
gttcgattctgtctaccagcacca
1−13tRNA−Trp(cca)[62400,62470]
aggtcgttagtgtaaaggttgcacagtggattccaaatcctcttgaatgg
gttcgattcctatacgattct
1−14tRNA−Gln(ttg)[62534,62607]
tgtcgtatggtgtaatggtagcacctcagaatttgactctgatagcgaag
gttcgatcccttctacgacttcca
1−15tRNA−His(gtg)[62610,62684]
gtagtcttaggttatatggtaaacccacaggttgtgaccctgaaaaacat
ggttcgagtccatgagattgcacca
1−16tRNA−Lys(ttt)[62687,62763]
tgatcggaagctaaagtggaggaagcactggacttttaatccagataaag
agggttcaactccctcccgatcaacca
1−17tRNA−Gly(tcc)[63016,63090]
gcgtgtgtcgtatagtggcaattataggtggcttccaaccatcggacgag
ggttcgattccctccacccgcacca
1−18tRNA−Glu(ctc)[63097,63172]
gttctcatcgtctaactggtcaggatgccacactctcaatgtggtgattt
gggttcgatccccaatgagaatacca

1-19tRNA-Glu(ttc)[63179,63253]
gctctattcgtctaatggctaggatttctccctttcaaggagaagaagag
ggatcgaaaccctcatagagtacca
1-20tRNA-Leu(tag)[63499,63575]
gctcaggtaagagaattggcatatcgcacagtcttagaaactgtgatttg
tgggttcgagtcccaccctgagtacca
1-21tRNA-Leu(taa)[63582,63658]
gatcgggtaggctaactggcaaagctgacagacttaaaatctgttgtttg
tgggttcgactcccaccccgattacca
>KJ018209.1 Shewanella sp. phage 1/4, complete genome
1-1tRNA-Gly(tcc)[42049,42124]
gcgtaattagctcagatggtagagcgctatccttccaagttagtggtcat
cggttcgaatccgatattacgctcca
1-2tRNA-Arg(tct)[42192,42268]
atctcgttagctcagttggatagagcaacagccttctaagctgtgggtcg
aaagttcgaatctttcacgggatacca
>JX128259.1 Escherichia phage ECML-134, complete genome
1-1tRNA-Arg(tct)c[68419,68494]
cgaggcatagctcagaaggaagagcaaggaccttctaagtcctgggtcgt
aggttcgatccctactgcctcgacca
1-2tRNA-Asn(gtt)c[68499,68573]
ggatgtgtagctcaatggcagagcgatcgcctgttaagcgattggttata
ggttcgaatcctatcacgtccgcca
1-3tRNA-Tyr(gta)c[68578,68664]
ggggagttatcccgtagaggtagcggtgtggactgtaaatccattgtcat
tgcgactcgggtggttcgactccaccactccccacca
1-4tRNA-Met(cat)c[68674,68748]
ggccctgtagctggaaggttcaagcaagcgactcataatcgccagatggt
ggttcaattccacccagggccacca
1-5tRNA-Thr(tgt)c[68750,68825]
gctgatttagctcagtaggtagagcaactcacttgtaatgagaaggtcgg
cggttcgattccgtcaatcagcacca
1-6tRNA-Ser(tga)c[68831,68920]
ggaggcgtggcagagtggtttaatgcaccggtcttgaaaaccggaagtcg
ctccggcgactcataggttcaaatcctatcgcctccgcca
1-7tRNA-Gly(tcc)c[69582,69655]
gcggatatcgtataatggtattacctcagacttccaatctgatgatgtga
gttcgattctcattatccgctcca
1-8tRNA-Leu(taa)c[69661,69747]
gcgagaatggtcaaattggtaaaggcacagcacttaaaatgctgcggaat
gatttccttgtgggttcgagtcccacttctcgcacca
1-9tRNA-Gln(ttg)c[69748,69821]
tgggaattagccaagttggtaaggcactggattttgattccaggatgcaa
aggttcgagtcctttattcccagc

>KM407600.1 Shigella phage Shf125875, complete genome
1-1tRNA-Arg(tct)c[74227,74303]
cgaggcatagctcaattgtatagagcaacggacttctaatccgtaggttg
aaggttagaatccttctgtctcgacca
1-2tRNA-Met(cat)c[74307,74381]
ggccctgtagctcaatgggagagctgtcagctcataactgataggtagct
ggatcgaaaccagccagggtcacca
>KF562341.1 Escherichia phage vB_EcoM_PhAPEC2, complete genome
1-1tRNA-Arg(tct)c[74040,74115]
cgaggcatagctcagaaggaagagcaaggaccttctaagtcctaggtcgt
aggttcgatccctactgtctcgacca
1-2tRNA-Asn(gtt)c[74120,74194]
ggatgtgtagctcagtggtagagcagttgactgttaatcaattggcccat
ggttcgaatccatgcacgtccgcca
1-3tRNA-Met(cat)c[74199,74273]
ggccctgtagctcaatgggagagctgtcagctcataactgataggtagct
ggatcgaaaccagccagggccacca
>KF554508.2 Bacillus phage CP-51, complete genome
1-1tRNA-Met(cat)[98,171]
ggttccttagctcaattggttagagctaccggctcataaccggtgggttg
taggttcgagtcctacaggaatca
1-3tRNA-Trp(cca)[1433,1506]
tgggggtatagtttaacaggtaaaacagtggtctccaaaaccaccgtcct
aggttcgaatcctagtaccctcgt
>KM216423.1 Staphylococcus phage P108, complete genome
1-1tRNA-Met(cat)c[94979,95050]
ggactcttagcttaaaggtaaagccaaccgctcataacggtttgactgta
ggttcgaatcctacagagtcca
1-2tRNA-Phe(gaa)c[116672,116744]
ggtttcttagctcagatggtagagcactagattgaagctctaggtgtcat
tggttcaaatccaatagaaacca
1-3tRNA-Asp(gtc)c[116750,116825]
tggctcattggtgtaactggttaacacactgccctgtcacggcagagagt
acgagttcgagtctcgtatgagtcgt
>KM373208.1 Listeria phage WIL-1, complete genome
1-1tRNA-Cys(gca)[550,620]
gcgggtataaccaactggaaaggtagtagactgcaaatctacgtatatgg
gttcaattcccattacccgct
1-2tRNA-Ser(gct)[699,785]
ggagagttgtcagagaggcttaatgatacgggttgctaactcgttgtact
agtaatagtaccaagggttcgaatcccttactctcct
1-3tRNA-Ile(gat)[962,1034]
gccagcatagcttagtaggcaaagcaaccgaccgataatcggtagtcctt
ggttcaattccaagtgttggtac

1–4tRNA–Asp(gtc)[1138,1210]
gtgcgtatgatataatggctattatactcggctgtctatcgagaaatagg
ggttcgattccccttacgtgcgc
1–5tRNA–Leu(tag)[1676,1760]
tgccgagatggtggaactggtatacacggtagacttagaatctgctgtcc
taaggatatgtgggttcgaatcccactctcggtat
1–6tRNA–Tyr(gta)[2121,2202]
gtgccattcgcatagaggcaattgcaggggactgtaactcccctcccttc
ggggttccaaggttcgagtccttgatggcgca
1–7tRNA–Thr(tgt)[2285,2356]
gcttgtatagttcaattggtagaacagtggttttgtaagcctcagacgtg
ggttcaagtcctactacaagca
1–8tRNA–Gln(ttg)[2375,2447]
tggtctgtagccaagcggtaaggcaacggattttgattccgtgatacgtt
ggttcgaatccaactagaccagc
1–9tRNA–Trp(cca)[2449,2522]
taggggtatagtttatctggtaaaatattggtttccaactccaatgaggt
gggttcaagtcctactatccctgt
1–10tRNA–Lys(ttt)[2647,2718]
ggagttatggtgaaatggctatcactgcgggtttttaccccgttattcta
ggttcgaatcctagtggctcca
1–11tRNA–Phe(gaa)[2724,2795]
gtagtcctagctgagatggattagcgcttgcttgaaaagcaagagaggca
ggttcgatacctgcggactcca
1–12tRNA–Ser(tga)[2808,2899]
ggaaggttggtagagcttggtaatacgctagtcttgaaaactagtttgcc
cttggaatacagggtacaagggttcaaatcccttaccttcct
1–13tRNA–Asn(gtt)[3521,3594]
gtgtccttaactcagaggtcagagtgccgtcctgttaagtcggaagtcgc
tggttcaaatccagcaggatacgc
1–14tRNA–Gly(tcc)[3663,3733]
gcgggtatagtataagggtagtacccaaggtttccaaccatgtagtgggg
gttcgaatccccctacccgct
1–15tRNA–Arg(tct)[3991,4061]
gtccttatggtgtagtggatgcacaagggatttctactcccttagcgcgg
gttcgaatcctgctgaggact
1–16tRNA–Pro(tgg)[5179,5253]
cagggtgtagctcagtttggttagagtacccgctttggagacgggaagtc
gtaggttcgaatcctaccaccttga
1–17tRNA–Met(cat)[6146,6219]
ttgtcccgtagctagaaggtcgagcaaggagctcataactcctcggtttg
ggttcgattcccaacggggcaatc
>KM507819.1 Escherichia phage 121Q, complete genome
1–1tRNA–Gly(tcc)[72682,72754]

gcaggtaaagtgttgatggcggcatacagtacttccgattctgggggtgg
ggttcaaatccccatacctgctc
1-2tRNA-Ser(tga)[73491,73580]
ggtgaagtggcagagtggtttaatgcaacggtcttgaaaaccgtcgaggg
tgaaagtcctccgtgagttcaaatctcaccttcaccgcca
1-3tRNA-Ser(act)[75464,75555]
ggaagggtggcagagcggttgattgcactcgcctactaagcgagcaagtg
ttagaaataacgcttcgtgggttcaaatcccacctctttcgc
1-4tRNA-Met(cat)[75563,75637]
gggcctgtagctcagtggtagagcggtcggctcataaccgattggtcgca
ggatcgttccctgccaggcccacca
1-5tRNA-Asn(gtt)[75728,75802]
ggttcgttggcctattggttgggcagcaaactgttaatttgccttagact
agttcaattctagtacgaaccgcca
1-6tRNA-Thr(tgt)[77896,77969]
gccgacttagctcagtaggtagagcgttcctcttgtaagggaaatgtcac
cagttcaattccggtagtcggcac
>KF301602.1 Caulobacter phage Cr30, complete genome
1-1tRNA-Arg(tct)[81958,82034]
ggtcgcatagctcaactggagagagcacaagccttctaagcttggggttg
taggttcgagtcctactgcgatcgcca
1-2tRNA-Asn(gtt)c[143895,143970]
tgaatagtagctcagttggtagagcgcctgactgttactcagaatgtcac
aggttcgagtcctgtctattcagcca
1-3tRNA-Gly(tcc)c[143974,144050]
gcatctatggtgatattggctagcacatctggtttccacccagaaggatt
cggttcgagctccgaatagatgctcca
1-4tRNA-Met(cat)c[151271,151345]
ggaccagtagctcattggtaagaactggctgctcataacagtctggtgat
tgttcgattcaatcctggtctacca
1-5tRNA-Pro(tgg)c[153143,153219]
ctctcagtagctcaattggttagagtacgggctttgggagctcggggttc
ctcgttcgagtcgaggctgggagacca
>KJ018211.1 Shewanella sp. phage 1/40, complete genome
1-1tRNA-Gly(tcc)[42797,42872]
gcgtaattagctcagatggtagagcgctatccttccaagttagtggtcat
cggttcgaatccgatattacgctcca
1-2tRNA-Arg(tct)[42940,43016]
atctcgttagctcagttggatagagcaacagccttctaagctgtgggtcg
aaagttcgaatctttcacgggatacca
>KM190144.1 Escherichia phage Av-05, complete genome
1-1tRNA-Phe(gaa)c[4068,4143]
gtgacggtaactcagttggtagagtgccttgctgaagactaggtggtcac
aggttcgaatcctgtccgtcacacca

1-2tRNA-Leu(tag)c[4149,4225]
gggcgattgatggaattggtatacatgtcaggcttagaacctgaattttg
ggggttcgagtccctcatcgcctacca
1-3tRNA-Pro(tgg)c[4474,4551]
ctgtccgtagcgcagcttgggtagcgcgggtcacttgggatgatcaggtc
gttggttcaaatccaaccggacagacca
1-4tRNA-Asn(gtt)c[4636,4726]
ggttctttgaccgagtggtcgatggtgacggcctgttaagccgttgggag
gaatctccccaccgaaggttcgaatccttcaggaaccgcca
1-5tRNA-Lys(ttt)c[4736,4812]
ggatctgtagccaaatggtttaaggcacttgacttttaatcgagggattg
ttagttcgaatctaaccagatccacca
1-6tRNA-Ser(tga)c[5035,5123]
ggaggctggggtgcatggtgcacaagctggtttgaacccagtcccgctgt
agagatatggtgacagttcgattctgtcagcctcctcca
1-7tRNA-Ser(gct)c[5202,5295]
ggaagattaaccctaatttggtaaggggtctccctgctaaggagatagta
acccagaaatgggtgtgccagttcaagtctggcatcttcctcca
1-8tRNA-Ser(gga)c[5317,5406]
ggagaagcaaatcgattggcgacgaaaccacattggaaatgtgctgactg
gtaaaacggcttgagagttcgattctctccttctccacca
1-9tRNA-Gln(ttg)c[5416,5491]
aggggattagcatagtggcctaatgcaacgggctttgaccccgtgatcgg
ttgttcgaatcaaccatcccctgcca
1-10tRNA-Gly(tcc)c[6493,6567]
gcatccatagtttaattgggaaaactacagccttccaagctgaagtcgag
ggttcgataccctctggatgctcca
1-11tRNA-Tyr(gta)c[6717,6806]
gtgtcgttagaccgtaggggtagcgggggagactgtaaatctcctgtctc
gaaagggactcgggtagttcgactctatcacggcacacca
1-12tRNA-Cys(gca)c[7241,7317]
gcacggttgccggaatttggttacggatctgtctgcaaaacggatatatg
tcggttcaagtccgacaccgtgctcca
1-13tRNA-Thr(tgt)c[7779,7853]
gctcgtatagctcaacggtagagcacctgccttgtaagcagggggttgca
ggttcgaaacctgctgcgagcacca
1-14tRNA-Met(cat)c[9584,9659]
ggccccttagctcagtggttagagcaggtgactcataatcgcttggtcgc
tggttcaagtccagcaggggccacca
>KJ858521.1 Aeromonas phage pAh6-C, complete genome
1-1tRNA-Pro(cgg)[30212,30283]
ctggggatgggctaagtaagccatgcgcacggggtgcgtaggatctaggt
acaaatcctaggccccagacca
1-2tRNA-Thr(tgt)[30285,30360]

gccagtttagcttagttggaagagcaaccgatttgtaatcggtaggtcgt
aggttcgagccctacaactggcacca
>KM501444.1 Shigella phage pSs-1, complete genome
1-1tRNA-Arg(tct)c[73554,73629]
cgaggcatagctcagaaggaagagcaaggaccttctaagtcctaggtcgt
aggttcgatccctactgcctcgacca
1-2tRNA-Asn(gtt)c[73634,73708]
ggatgtgtagctcaatggcagagcgatcgcctgttaagcgattggttata
ggttcgaatcctatcacgtccgcca
1-3tRNA-Tyr(gta)c[73713,73799]
ggggagttatcccgtagaggtagcggtgtggactgtaaatccattgtcat
tgcgactcgggtggttcgactccatcactccccacca
1-4tRNA-Met(cat)c[73809,73883]
ggccctgtagctggaaggttcaagcaagcgactcataatcgccagatggt
ggttcaattccacccagggccacca
1-5tRNA-Thr(tgt)c[73885,73960]
gctgatttagctcagtaggtagagcaactcacttgtaatgagaaggtcgg
cggttcgattccgtcaatcagcacca
1-6tRNA-Ser(tga)c[73966,74055]
ggaggcgtggcagagtggtttaatgcaccggtcttgaaaaccggcagtcg
ctccggcgactcataggttcaaatcctatcgcctccgcca
1-7tRNA-Pro(tgg)c[74057,74131]
ctccgtgtagctcagtttggtagagcgcctgatttgggatcaggaggtcc
aaggttcaaatccttgtatggagac
1-8tRNA-Gly(tcc)c[74142,74215]
gcggatatcgtataatggcattacctcagacttccaatctgatgatgtga
gttcgattctcattatccgctcca
1-9tRNA-Leu(taa)c[74221,74307]
gcgagaatggtcaaattggtaaaggcacagcacttaaaatgctgcggaat
gatttccttgtgggttcgagtcccacttctcgcacca
1-10tRNA-Gln(ttg)c[74308,74381]
tgggaattagccaagttggtaaggcactggattttgattccaggatgcaa
aggttcgagtcctttattcccagc
>AP014629.1 Edwardsiella phage GF-2 DNA, complete sequence
1-1tRNA-Asn(gtt)[39837,39912]
ggtggggaagcacatacggatgtgcggcaggctgttaacctgatggtata
gggttcgagtccctaacccaccgcca
1-2tRNA-Thr(tgt)[39914,39988]
gccggaatagttcagtggcagaacggatgccttgtaagcatcgcgtcaga
ggttcgattccttttctcggcacca
>KJ000058.2 Salmonella phage STP4-a, complete genome
1-1tRNA-Arg(tct)c[61904,61980]
cgaggcatagctcaatagtatagagcaacggtcttctaaaccgtaggttg
aaggttagaatccttctgtctcgacca

1−2tRNA−Asn(gtt)c[61987,62058]
tggtgcatcgtataaaggttattacggggagctgttaactcctttatctt
ggttcgagtccaagtgcgccag
1−3tRNA−Gln(ttg)c[62367,62442]
tgggaattagccaagttggtaaggcaccggattttgattccgggatgcac
tggttcgagcccagtattcccagcca
1−4tRNA−Met(cat)c[62449,62524]
ggccccttagctcagtggttagagcaggcgactcataatcgcttggtcgc
tggttcaagtccagcaggggccacca
>LN610573.1 Pseudomonas phage vB_PaeM_PAO1_Ab03, complete genome
1−1tRNA−Asn(gtt)[25015,25091]
tccgttcggtccctcaaggcgagggagcttgactgttaatcaagacgtgc
ctggatcgattccagggagcggagcca
1−2tRNA−Tyr(gta)[25149,25236]
ggaggggtggcagagcggtttaatgcaccggcctgtaaatccggcgtccg
accgagcatcgctggttcaaatccagccccctccacca
1−3tRNA−Gln(ttg)[25438,25513]
aggcgtgtggcgaaaaggttaacgcactggactttgactccagcattcga
gggttcgaatccctccacgtctgcca
>KJ019069.1 Synechococcus phage ACG−2014j isolate Syn7803US103, complete genome
1−1tRNA−Met(cat)[17049,17124]
tgcttccttagcaatctggtgaatgcagcaaactcataatttgcctaagg
agagttcgatcctctcaggaagcatc
1−2tRNA−Leu(taa)[17125,17211]
tgcgagtatggcggaatcggtagacgcaccagacttaaaatctgttgagc
attatgctcgtgggagttcaagtctccctactcgcat
1−3tRNA−Thr(tgt)[18029,18101]
gccaaactagctcagtggtagagcagggcttttgtaaagctcaggtcgca
ggttcaaatcctgtgtttggctc
1−4tRNA−Asn(gtt)[18106,18177]
tcctctttagctcagcggtagagcggttgactgttaatcaatttgtccct
ggttcgatcccaggaaggggag
1−5tRNA−Arg(tct)[158077,158153]
gactcagtagctcagttggatagagcaactgccttctaagcagtcggtcg
ttggttcgagtccaacctgagtcgcca
1−6tRNA−Leu(tag)[158154,158236]
gtcggtatggcggaattggtagacgcgccaggtttaggttctggtgtctt
atgacgtggaggttcaagtcctcttaccgacac
1−7tRNA−Val(tac)[158239,158310]
gctcgaatagctcagaggtagagcacctcctttacacggagattgtcggg
ggttcgatccccctcttcgagca
1−8tRNA−Gly(tcc)[161447,161518]
gcgggtgtagctcagtggtagagcgtcagttttccaaactgaatgtcgtc
ggttcaagtccgatctcccgct

>KJ019156.1 Synechococcus phage ACG-2014e isolate Syn7803C2, complete genome

1-1tRNA-Met(cat)[17776,17848]
gcttccttagcaatctggtgaatgcagcaaactcataatttgcctaagga
gagttcgatcctctcaggaagca
1-2tRNA-Leu(taa)[17851,17937]
tgcgagtgtggcggaatcggtagacgcaccagacttaaaatctgttgagc
attatgctcgtgggagttcaagtctccctactcgcat
1-3tRNA-Asp(gtc)[18754,18829]
tggggttgtagctcagttggttagagcgcctgcctgtcacgcaggaagtc
gtgggttcaagtcccatcaatcccgt
1-4tRNA-Thr(tgt)[18832,18904]
gccaaactagctcagtggtagagcagggcttttgtaaagctcaggtcgca
ggttcaaatcctgtgtttggctc
1-5tRNA-Asn(gtt)[18909,18980]
tcctccttagctcagcggtagagcggttgactgttaatcaatttgtccct
ggttcgatcccaggagggggag
1-6tRNA-Arg(tct)[154258,154334]
gactcagtagctcagttggatagagcaactgccttctaagcagtcggtcg
ttggttcgagtccaacctgagtcgcca
1-7tRNA-Leu(tag)[154335,154417]
gtcggtatggcggaattggtagacgcgccaggtttaggttctggtgtctt
atgacgtggaggttcaagtcctcttaccgacac
1-8tRNA-Val(tac)[154420,154491]
gctcgaatagctcagaggtagagcacctcctttacacggagattgtcggg
ggttcgatcccctcttcgagca
1-9tRNA-Gly(tcc)[156950,157021]
gcgggtgtagctcagtggtagagcgtcagttttccaaactgaatgtcgtc
ggttcaagtccgatctcccgct

>KJ019071.1 Synechococcus phage ACG-2014g isolate Syn7803US105, complete genome

1-1tRNA-Val(tac)[13160,13233]
tgggcgaataactcagcggtagagtgtctcctttacacggaggttgtcgg
gggttcgatcccctcttcgcccat
1-2tRNA-Leu(taa)[18087,18173]
tgggagtgtggcggaatcggtagacgcaccagacttaaaatctgttgagc
aatatgctcgtgagagttcaagtctctctactcctat
1-3tRNA-Thr(tgt)[18387,18459]
gcctccgtagctcagtggtagagcagggcttttgtaaagctcaggtcgca
agttcaaatcttgtcagaggctc
1-4tRNA-Asn(gtt)[18463,18536]
ttcctccttagctcagcggtagagcggttgactgttaatcaatttgtccc
tggttcgatcccaggagggggagt
1-5tRNA-Arg(tct)[157432,157507]
tgggtcagtagctcagatggatagagcaactgccttctaagcagtcggtc
gcaggttcgagtcctgcctgacccgt

>KJ019136.1 Synechococcus phage ACG-2014d isolate Syn7803C102, complete genome
1-1tRNA-Val(tac)[13624,13697]
tgggtgattaactcagtggtagagtgactgctttacacgcagtaggtcac
tggttcaaatccagtattacccat
1-2tRNA-Arg(tct)[153716,153790]
tgggcaagtagctcagtggatagagcatcgcacttctaatgcgttggtcg
ggggttcaaatccctccttgcccgt
1-3tRNA-Asn(gtt)[153811,153884]
ttcccaagtagctcagtggcagagccgccgactgttaatcggctggtcgc
tggttcaaatccagccttgggagt
>KJ019059.1 Synechococcus phage ACG-2014f isolate Syn7803C90, complete genome
1-1tRNA-Arg(tct)[132746,132819]
gggtcagtagctcagatggatagagcaattcacttctaatgaattggtcg
ggggttcgagtccctcctgacccg
1-2tRNA-Asn(gtt)[133033,133104]
tccacaatagctcagcggtagagtcggcgactgttaatcgccttgtccct
ggttcgaatccaggttgtggag
>KM236237.1 Citrobacter phage Miller, complete genome
1-1tRNA-Met(cat)[49270,49346]
ggcccttagctcaattggtagagcgaacccctcataagggtgtggtttc
cggttcgagtcacggaaggggccacca
>KP797973.1 Salmonella phage Det7, complete genome
1-1tRNA-Ser(gct)c[132125,132213]
ggaaggttgcccgagaggtttaagggactcgactgctaatcgagtggggc
ttttagcccccgaaggttcgaatccttcaccttccgcca
1-2tRNA-Tyr(gta)c[132486,132569]
gtgagtgtggcagagcggtcgaatgcaggagactgtaaatctccccgtaa
cagcgcggtggttcgaatccatccactcacacca
1-3tRNA-Asn(gtt)c[132827,132902]
gacgatgtagttcagtcggtagaacggcggtctgttaaaccgtatgtcgc
aggttcaagtcctgccatcgtcgcca
1-4tRNA-Met(cat)c[133074,133150]
ggtcctgtagctcagtcggttagagcagtgaactcataattcattggtca
ttggttcaaacccaatcaggatcacca
>KJ019134.1 Synechococcus phage ACG-2014b isolate Syn7803C100, complete genome
1-1tRNA-Thr(tgt)[13153,13225]
gcctccgtagctcagtggtagagcagggcttttgtaaagctcaggtcgca
agttcaaatcttgtcagaggctc
1-2tRNA-Asn(gtt)[13230,13302]
tcctctatagctcagttggtagagcaggtgactgttaatcaccctgtccc
tggttcgagtccaggtggaggag
1-3tRNA-Val(tac)[13305,13376]
gggcgaataactcagcggtagagtgcctcctttacacggagattgtcggg
ggttcgatcccctcttcgccca

1-4tRNA-Leu(taa)[18740,18826]
tgggagtgtggcggaatcggtagacgcaccagacttaaaatctgttgaga
attaatctcgtgggggttcaagtccccccactcctat
1-5tRNA-Arg(tct)[155502,155576]
tgggtcagtagctcagcggatagagcatcgcacttctaatgcgttggtcg
caggttcgatccctgcctgacccgt
>KJ019082.1 Synechococcus phage ACG-2014i isolate Syn7803US120, complete genome
1-1tRNA-Met(cat)[17929,18001]
gcttccttagcaatctggtgaatgcagcaaactcataatttgcctaaggt
gagttcgatcctcacaggaagca
1-2tRNA-Leu(taa)[18005,18088]
gcgagtatggcggaatcggtagacgcaccagacttaaaatctgttgagag
caatctcgtgggagttcaagtctccctactcgca
1-3tRNA-Asp(gtc)[18907,18982]
tggggttgtagctcagttggttagagcgcctgcctgtcacgcaggaagtc
gtgggttcaagtcccatcaatcccgt
1-4tRNA-Thr(tgt)[18985,19057]
gcctccgtagctcagtggtagagcagggcttttgtaaagctcaggtcgca
agttcaaatcttgtcagaggctc
1-5tRNA-Asn(gtt)[19062,19133]
tcctccttagctcagcggtagagcggttgactgttaatcaatttgtccct
ggttcgatcccaggagggggag
1-6tRNA-Arg(tct)[153173,153249]
gggtcagtagctcagatggatagagcaattcacttctaatgaattggtcg
ggggttcgagtccctcctgacccgcca
1-7tRNA-Leu(tag)[153250,153333]
gtcggtatggcggaattggtagacgcgccaggtttaggttctggtgtctt
tatgacgtggaggttcaagtcctcttaccgacac
1-8tRNA-Val(tac)[153336,153407]
gctcgaatagctcagaggtagagcagcacctttacacggtgaatgtcggg
ggttcgatccccctcttcgagca
1-9tRNA-Gly(tcc)[155856,155927]
gcgggtgtagctcagtggtagagcgtcagttttccaaactgaatgtcgtc
ggttcaagtccgatctcccgct
>KF381361.1 Sinorhizobium phage phiM12, complete genome
1-1tRNA-Thr(tgt)c[81122,81205]
gccggtatagccaagcggtcgaaggcacctgatttgtaatcaggtattca
aacgtcggcagttcgaatctgtctaccggctcca
1-2tRNA-Gln(ttg)c[81206,81279]
ttgcccttagttcagttggtagaacgtcgaattttggtttcgaaggtccg
tggttcgaatccacgagggtaagc
1-3tRNA-His(gtg)[86152,86228]
tcatggatggtgttagtctggtagcacgcgcggatgtggaccgcgaagca
taggttcaaatcctattccatggacca

1–4tRNA–Leu(caa)[94993,95080]
tgggagataaaggaaacttaccagttcaattctggtttctccaagagtaa
tcgagtggttgtaggttcgagtcctacctccccagcca
1–5tRNA–Met(cat)[95631,95707]
tgcggggtagtagcagtggttagctcgtctgcctcataagcagaaggtca
tcagttcgaatctgatccccgcaacca
1–6tRNA–Cys(gca)[96394,96468]
gacgagaaggcagaagggttatgcgcggcgttgcaaccgccgtatgtgtc
ggttcaagtccggctctcgtctcca
1–7tRNA–Lys(ttt)[101026,101102]
gcgcgcttaactcagttggttagagtggcgaccttttaagtcgtttgtcc
ccggttcgagtccgggagcgcgcacca
>KJ801817.1 Enterococcus phage ECP3, complete genome
1–1tRNA–Asp(gtc)[100086,100161]
tggcagtatagggcagaggttgtcccaacacgttgtcagcgtggaacaca
cgggttcgagtcccgttactgtcgtc
1–2tRNA–Trp(cca)[100412,100485]
tagtcggttagtgtaactggtaacacgttggtctccaaaaccaataatag
gggttcaaatcctctaccgattgt
1–3tRNA–Arg(tct)[100969,101044]
tgtaggtgtagctcaatcggatagagcatccgccttctaagcggacggtt
gggggttcgattccctccatctacgt
1–5tRNA–Leu(tag)[101766,101850]
gcagaagtgatggaactggtagacaacggtgtcttagaaacatcggtctg
taatggacgtgtgggttcgactcccgccttctgca
1–6tRNA–Met(cat)[102473,102546]
ggacgtttagctcagttggtcagagcattcggctcataaccgaacggtcg
caggttcgagacctgcaatgtcca
>KC295538.1 Escherichia phage PBECO 4, complete genome
1–1tRNA–Thr(tgt)c[207837,207910]
gccgacttagctcagtaggtagagcaatccccttgtaagggaaaggtcac
cagttcgattccggtagtcggcac
1–2tRNA–Asn(gtt)c[208949,209021]
gactctgtagttcagcggtagaacgtcaaactgttaatttggttgtcggg
ggttcgaatccctccagagtcgc
1–3tRNA–Met(cat)c[209026,209104]
gggccattagctcacgcgtggttagagcaaacgactcataatcgttcggt
acggtgttcgactcaccgatggcccacca
1–4tRNA–Ser(gct)c[209109,209198]
agtgagttggatgagtggttgaaatcactaccctgctaaggtagcatatg
cgaaagcgtatcgtgggttcgaatcccacactcactgcca
1–5tRNA–Ser(tga)c[210921,211010]
ggtgaagtggcagagcggtttaatgcaacggtcttgaaaaccgtcgaggg
cgaaagtcctccgtgagttcaaatctcaccttcaccgcca

1−6tRNA−Gly(tcc)c[212040,212112]
gcaggtaaagtgttgatggcggcatacagtacttccgattctgggggtgg
ggttcaaatccccatacctgctc
>KJ676859.1 Bacillus phage JBP901, complete genome
1−1tRNA−Met(cat)c[156041,156115]
taggattatagctcagtggtagagcgtgggtctcataagcccaaggtcga
tggttcgatcccatctattcctatc
1−2tRNA−Leu(tag)c[156207,156291]
gtcgaagtgttggaactggtatacatgcggcacttagaatgccgtgcctt
cgggattgtgggttcgactcccaccttcgacacca
1−3tRNA−Ile(gat)c[156300,156376]
actagtgtagctcagtcaggtagagcagtgtcttgataaggcattggtcg
taggttcaaatcctaccactagtacca
1−4tRNA−Leu(taa)c[156383,156469]
atcggaatgttggaattggtagacataacggacttaaaatccgttgctcg
tagagcgtggcgggttcgagtccccccctccgatacca
1−5tRNA−Tyr(gta)c[156473,156555]
gggcgtgcaatcattggagagataagctgactgtaaatcagtggtcattg
actgtggaggttcgaatccttccattcccacca
1−6tRNA−Phe(gaa)c[156562,156636]
ggatagatagctgagatggattagcgaaggattgaaactccttagaggtt
ggatcgttaccaactctgtccacca
1−7tRNA−Pro(tgg)c[156731,156802]
gtagatgtaggctagaggtcagtcactccgtttggggcggaggtcacgca
ggttcgatccctgccatctgca
1−8tRNA−His(gtg)c[156909,156982]
cagtgtgtggtgaagtggcttaacacggacgactgtggatcgttcattag
caggttcgaatcctgtcatgctga
1−9tRNA−Gln(ttg)c[157060,157135]
tttcggtgtggacaaactggtaaagtcactaggctttgaccctaggtttt
ggaggttcgacccccttccaccgaagt
1−10tRNA−Ser(tga)c[157217,157306]
cggggaatactcaagtggataaaagagggtagtcttgaaaactactaggc
gtataaaagcgtgcgggggttcgaatccctcttcctcggc
1−11tRNA−Arg(tct)c[157312,157387]
taccccttagccaagtggactaaggcagcgggcttctatcccgaagatc
gtgggttcaaatcctacagggggtgt
1−12tRNA−Ile(tat)c[157507,157580]
atccctttagccaagcggttaaggcagtaggattatgtcctgcgtatcgg
gagttcgatcctcccaaggggcgc
1−13tRNA−Asp(gtc)c[157582,157655]
tggggatatagtgtagaggttaacacgcacggctgtctaccgtgaagcac
gggttcgaatcccgttatcctcgt
1−14tRNA−Thr(tgt)c[157743,157819]

gccttcttagctcagttggttagagcgactgccttgtaagcagtaggtcg
tgggttcgaatcctacagtcggcacca
1-15tRNA-Glu(ttc)c[158378,158454]
tgtcgcattggtgaaattggctaacacactcggctttctaccgagcattc
gcaggttcgaatcctgtatgcgatatc
1-16tRNA-Asp(gtc)c[158462,158535]
ggctcgttagtttaacggtaaaatacatgactgtctatcatgggtcacgg
gttcaactcccgtacgggtcgcca
1-17tRNA-Gly(tcc)c[158540,158614]
ggggcattggtatattggctattattcttggcttccaaccaagcgaggtc
ggttcgattccgacatgtccctcca
1-18tRNA-Asn(gtt)c[158618,158693]
gtgcttgtagctcagtcggtagagctggtggctgttaaccactgtgtcgt
aggttcgattcctacctagcacgcca
1-19tRNA-Cys(gca)c[158739,158812]
gagggtgtaccgaagcggctcaacggcacagattgcaaccctgttgttcg
tgggttcaaatcccaccatcctct
1-20tRNA-Ser(gct)c[158819,158907]
ggaagggtactcaagttggtgaagaggtcaacctgctaagttgatagtac
ctgttaaaggtagcgagggttcgaatccctttccttcct
>JN225449.1 Enterobacteriaphage UAB_Phi87, complete genome
1-1tRNA-Leu(taa)c[45,123]
gctccattactccaattggcagagaggccagacttaaaatctgtgttatg
tatcggttcgaatccgatatggagtacca
1-2tRNA-Gln(ttg)c[126,201]
aggggattagtttacaaggttaaaacctcggtctttgaaatcgaagaagt
tggttcaattccaacatcccccgcca
1-3tRNA-Arg(acg)c[1025,1100]
gcaggattagttcaaatggatagagcaacagtctacgaagctgttaatag
gggttcgaatcccttatcctgcgcca
1-4tRNA-Leu(caa)c[1106,1183]
gttccagtatcccaattggcagaggatgcaagctcaaaccttgtactagt
gacggttcgaatccgtcttggaacacca
1-5tRNA-Val(tac)c[1185,1259]
actcgcttagtttatatggtaaaacatcacccttacaagatgaagaaaaa
ggttcaagtcctttagtgagtacca
1-6tRNA-Thr(tgt)c[1355,1430]
gctcctatcgtataattggctattacggttgccttgtaagcaacttatca
gggttcgaatccttgtgggagcacca
1-7tRNA-Gly(tcc)c[1438,1512]
gcatccatagtttaaacgggaaaattacagtcttccaaactgaggttgag
ggttcgattccctctggatgctcca
1-8tRNA-Ala(tgc)c[1519,1594]
ggggtcatagtttatatggttaaaattcgagttttgcaaacttgggaact

gagttcaattctcagtgactccacca
1–9tRNA–Lys(ctt)c[1601,1676]
gcaggtgtagcaaaatggttatgcggctgactcttaatcagtaagacgat
gggttcaattccctccacctgtacca
1–10tRNA–Leu(tag)c[1684,1761]
gggagattgacggtaattggtaaacctaccatccttagaagttggtgttt
gagggttcgaatcccttgtctcctacca
1–11tRNA–Ser(tga)c[2018,2107]
ggtaggtagcggctaatggtagccaaactgtcttgaaaacagttgccact
gtagagatacggtgagggttcgactcctttacttaccgcc
1–12tRNA–Arg(tct)c[2346,2422]
gcacccttagttcaattggatagagcaacggtcttctaaatcgttagtta
caggttcgaatcctgtagggtgtgcca
1–13tRNA–Ile(gat)c[2759,2834]
gctggtatagttaagaaggttataacactcccctgataagggaacatcgg
tggttcgattccacctaccagtacca
1–14tRNA–Met(cat)c[2836,2912]
ggttcagtcgcagataaggtaatgcaagggtctcataagccctatgaatg
tgggttcgattcccatctgaacctcca
1–15tRNA–Lys(ttt)c[2917,2992]
ggaagtgtagcagaatggtgatgcggcagacttttaatctgacaggcgat
gggttcgaatccctccacttctacca
1–16tRNA–Asp(gtc)c[3451,3527]
ggttatgtagtttaactggttaaaatactcccctgtcacgggagatgatg
agggttcaaatcccttcgtaaccgcca
1–17tRNA–Tyr(gta)c[3534,3621]
gtgtcgttatcccgtagatggtagcggtggggactgtaaatcccttgtca
ttgagactcggtaggttcgactcctacacggcacacca
1–18tRNA–Asn(gtt)c[3631,3707]
ggttaggaagcacataaggtatgtgcggtcgcctgttaagcgaatggcac
agggttcgaatccctgactaaccgcca
1–19tRNA–Met(cat)c[3785,3861]
tgcgggtatagagaaagggcgtctcacatgtctcattagcatggtatcgg
caggttcgactcctgcacccgcctcca
1–20tRNA–Glu(ttc)c[3953,4030]
gttccagtagacaaaacggtaaagtcaccactctttcaaagtggatattt
gagggttcaaatcccttctggaacgcca
1–21tRNA–Pro(tgg)c[4038,4114]
ctctgtttagctcagcttggtagagcgttccgtttggggcggtaaggccg
gaggttcaagtcctccaacagagacca
1–22tRNA–Cys(gca)c[85911,85986]
gaatccgtgacagaaatggctatgtgcctgtctgcaaaacaggtttataa
gggttcgagtcccttcggattctcca
1–23tRNA–Ser(gct)c[85991,86083]

ggaagattaaccctaaaaggtaagggagcagtttgctaaactgccagtag
ctgagaaatcggtgtaccagttcaagtctggtatcttcctcca
1–24tRNA–Phe(gaa)c[86836,86911]
agtccaagtagcttatatggttaaagcgcgtgtctgaaaaacatgagaag
agggttcaaatcccactggactacca
1–25tRNA–His(gtg)c[86918,86993]
gtggccttatcataaatggtaatgacccatgctgtgaacatggtctatac
gggttcaaatcccgtaggtcacccca
1–26tRNA–Gln(ctg)c[87025,87100]
agcggtatagcataactggcaatgcagcagtctctgaagctgtcctatta
aggttcaaatccttatgccgctgcca
1–27tRNA–Leu(taa)c[87106,87184]
gctccattactccaattggcagagaggccagacttaaaatctgtgttatg
tatcggttcgaatccgatatggagtacca
1–28tRNA–Gln(ttg)c[87187,87262]
aggggattagtttacaaggttaaaacctcggtctttgaaatcgaagaagt
tggttcaattccaacatcccccgcca
>KP010413.1 Salmonella phage vB_SPuM_SP116, complete genome
1–1tRNA–Pro(tgg)[26880,26956]
ctctgtttagctcagcttggtagagcgttccgtttggggcggtaaggccg
gaggttcaagtcctccaacagagacca
1–2tRNA–Glu(ttc)[26964,27041]
gttccagtagacaaaatggtatagtcaccactctttcaaagtggatattt
gagggttcaaatcccttctggaacgcca
1–3tRNA–Met(cat)[27133,27209]
tgcgggtatagagaaagggcgtctcacatgtctcattagcatggtatcgg
caggttcgactcctgcacccgcctcca
1–4tRNA–Asn(gtt)[27288,27364]
ggttaggaagcacataaggtatgtgcggtcgcctgttaagcgaatggcac
agggttcgaatccctgactaaccgcca
1–5tRNA–Tyr(gta)[27434,27521]
gtgtcgttatcccgtagatggtagcggtggggactgtaaatcccttgtca
ttgagactcggtaggttcgactcctacacggcacacca
1–6tRNA–Asp(gtc)[27527,27604]
ggctatgtagtttaatagggttaaaatactcccctgtcacgggagatgat
gtgagttcaagtctcatcgtagccgcca
1–7tRNA–Met(cat)[27841,27917]
ggttcagtcgcagataaggtaatgcaagggtttcataagccctatgaatg
tgggttcgaatcccatctgaacctcca
1–8tRNA–Ile(gat)[27920,27994]
tgtgggttagcataaatggtaatgcaaacggctgataaccgttagaagag
ggttcgataccctcacctacaacca
1–9tRNA–Arg(tct)[28331,28407]
gcacccttagttcaactggacagaacaaatgacttctaatcattaggtta

caggttcgaatcctgtagggtgtgcca
1-10tRNA-Ser(tga)[28646,28735]
ggtaggtagcggctaatggtagccaaacagtcttgaaaactgttgccact
gtagagatacggtgagggttcgactcctttacttaccgcc
1-11tRNA-Leu(tag)[28988,29064]
gcacctatagcccaactggtagaggcagcagacttagaatctgctcagtg
tgagttcgaatctctctaggtgtacca
1-12tRNA-Lys(ctt)[29072,29147]
gcaggtgtagcaaaatggttatgcggctgactcttaatcagtaagacgat
gggttcaattccctccacctgtacca
1-13tRNA-Ala(tgc)[29154,29229]
ggggatgtagtttacatggttaaaacataagttttgcaaacttaagtaca
gggttcaattccctgcttctccacca
1-14tRNA-Gly(tcc)[29236,29310]
gcatccatagtttaaacgggaaaattacagtcttccaaactgaggttgag
ggttcgattccctctggatgctcca
1-15tRNA-Thr(tgt)[29318,29394]
gctgctttcgtataattggctattacacatccattgtaaggatggaaatg
caggttcgagtcctgtgagcagcacca
1-16tRNA-Val(tac)[29490,29564]
actcgcttagtttatatggtaaaacatcacccttacaagatgaagaaaaa
ggttcaagtcctttagtgagtacca
1-17tRNA-Leu(caa)[29566,29643]
gttccagtatcccaattggcagaggatgcaagctcaaaccttgtattagt
gacggttcgaatccgtcttggaacacca
1-18tRNA-Arg(acg)[29758,29833]
gcaggattagttcaaatggatagagcagcagtctacgaagctgttaatag
gggttcgaatcccttatcctgcgcca
1-19tRNA-Gln(ttg)[30419,30494]
aggggattagtttacaaggttaaaacctcggtctttgaaatcgaagaagt
tggttcaattccaacatcccccgcca
1-20tRNA-Leu(taa)[30497,30575]
gctccattactccaattggcagagaggccagacttaaaatctgtgttatg
tatcggttcgaatccgatatggagtacca
1-21tRNA-Gln(ctg)[30581,30656]
agcggtatagcataactggcaatgcagcagtctctgaagctgtcttatta
aggttcaaatccttatgccgctgcca
1-22tRNA-His(gtg)[30688,30763]
gtggccttatcataaatggtaatgacccatgctgtgaacatggtctatac
gggttcaaatcccgtaggtcacccca
1-23tRNA-Phe(gaa)[30770,30845]
agtccaagtagcttatatggttaaagcgcgtgtctgaaaaacatgagaag
agggttcaaatcccactggactacca
1-24tRNA-Ser(gct)[31598,31690]

ggaagattaaccctaaaaggtaagggagcagtttgctaaactgccagtag
ctgagaaatcggtgtaccagttcaagtctggtatcttcctcca
1-25tRNA-Cys(gca)[31695,31770]
gaatccgtgacagaaatggctatgtgcctgtctgcaaaacaggtttataa
gggttcgagtcccttcggattctcca
>KP037007.1 Erwinia phage phiEa2809, complete genome
1-1tRNA-Lys(ttt)[147462,147537]
ttcgtgatagctcagttggtagagcagcctccttttaagaggcaggtcac
tggttcaagtccagttcacggcacca
>JX560968.1 Escherichia phage EC6, complete genome
1-1tRNA-Pro(tgg)[11934,12010]
ctctgtttagctcagattggtagagcgttccgtttggggcggtaaggccg
gaggttcaagtcctccaacagagacca
1-2tRNA-Glu(ttc)[12018,12095]
gttccagtagacaaaatggtatagtcaccactctttcaaagtggatatct
gagggttcaaatcccttctggaacgcca
1-3tRNA-Met(cat)[12181,12257]
cgcgggtatagagaaagggcgtctcacatgtctcattagcatggtaacgg
ttggttcgactccaacacccgcctcca
1-4tRNA-Asn(gtt)[12343,12419]
ggttaggaagcacataaggtatgtgcggtcgcctgttaagcgaatggcac
agggttcgaatccctgactaaccgcca
1-5tRNA-Tyr(gta)[12428,12515]
gtgtcgttatcccgtagatggtagcggtggggactgtaaatcccttgtca
ttgagactcggtaggttcgactcctacacggcacacca
1-6tRNA-Asp(gtc)[12521,12597]
ggctatgtagtttaactggagaaaatactcccctgtcacgggagatgatg
tgagttcaagtctcatcgtagccgcca
1-7tRNA-Lys(ttt)[13024,13099]
ggaagtgtagcagaatggtgatgcggcagacttttaatctgacaggcgat
gggttcgaatccctccacttctacca
1-8tRNA-Met(cat)[13104,13180]
ggttcagtcgcagataaggtaatgcaagggtctcataagccctatgaatg
tgggttcgaatcccatctgaacctcca
1-9tRNA-Ile(gat)[13183,13257]
tgtgggttagcataaatggtaatgcaaacggctgataaccgttagaagag
ggttcgataccctcacctacaacca
1-10tRNA-Ser(tga)[13827,13916]
ggtaggtagcggctaatggtagccaaactgtcttgaaaacagttgccact
gtagagatacggtgagggttcgactcctttacttaccgcc
1-11tRNA-Leu(tag)[14170,14247]
gggagattgacggtaattggtaaacctatctcgcttagaacgagatgttt
gagggttcgaatcccttgtctcctacca
1-12tRNA-Lys(ctt)[14255,14330]

gcaggtgtagcaaaatggttatgcggctgactcttaatcagtaagacgat
gggttcaattccctccacctgtacca
1-13tRNA-Ala(tgc)[14336,14411]
ggggtcatagtttatatggttaaaattcgagttttgcaaacttgggaact
gagttcaattctcagtgactccacca
1-14tRNA-Gly(tcc)[14418,14492]
gcatccatagtttaaacgggaaaattacagtcttccaaactgaggttgag
ggttcgattccctctggatgctcca
1-15tRNA-Thr(tgt)[14500,14576]
gctgctttcgtataattggctattacacatcccttgtaaggatggaaatg
caggttcgagtcctgtgagcagcacca
1-16tRNA-Val(tac)[14672,14746]
actcgcttagtttatatggtaaagccccatccttacaagttggtgaaaaa
ggttcaagtcctttagtgagtacca
1-17tRNA-Leu(caa)[14748,14825]
gttccagtatcccaattggcagaggatgcaagctcaaaccttgtactagt
gacggttcgaatccgtcttggaacacca
1-18tRNA-Arg(acg)[14940,15015]
gcaggattagttcaaatggatagagcaacagtctacgaagctgttaatag
gggttcgaatcccttatcctgcgcca
1-19tRNA-Gln(ttg)[15839,15914]
aggggattagtttacaaggttaaaacctcggtctttgaaatcgaagaagt
tggttcaattccaacatcccccgcca
1-20tRNA-Leu(taa)[15917,15995]
gctccattactccaattggcagagaggccagacttaaaatctgtgttatg
tatcggttcgaatccgatatggagtacca
1-21tRNA-Gln(ctg)[16001,16076]
agcggtatagcataactggcaatgcagcagtctctgaagctgtcttatta
aggttcaaatccttatgccgctgcca
1-22tRNA-His(gtg)[16108,16183]
gtggccttatcataaatggtaatgacccatgctgtgaacatggtctatac
gggttcaaatcccgtaggtcacccca
1-23tRNA-Phe(gaa)[16190,16265]
agtccaagtagcttatatggttaaagcgcgtgtctgaaaaacatgagaag
agggttcaaatcccactggactacca
1-24tRNA-Ser(gct)[17018,17110]
ggaagattaaccctaaaaggtaagggagcagtttgctaaactgccagtag
ctgagaaatcggtgtaccagttcaagtctggtatcttcctcca
1-25tRNA-Cys(gca)[17115,17190]
gaatccgtgacagaaatggctatgtacctgtctgcaaaacaggtttataa
gggttcgagtcccttcggattctcca
>KM236240.1 Citrobacter phage Moon, complete genome
1-1tRNA-Leu(taa)c[70996,71080]
gcatcgatggtggaactggtatacacatggcacttaaaatgccacgccgt

aaggattgagggttcaaatccctctcgatgcacca
1-2tRNA-Met(cat)c[71082,71158]
ggccctatagctcaattggttagagcaaccggctcataactggtaggttt
ctggttcaagtccagatggggtcacca
1-3tRNA-Thr(tgt)c[71197,71272]
gctgatttagcttagtaggtagagcaactcacttgtaatgagaaggtcgg
cggttcgattccgtcaatcagcacca
1-4tRNA-Pro(tgg)c[71277,71353]
ctccgcatagctcagcttggtagagcgcctggtttgggaccaggaggtcg
aatgttcgagtcattctgtggagacca
1-5tRNA-Gly(tcc)c[71665,71739]
gcatccatcgtatagcggatattatgactggcttccacccagtagatgag
agttcgattctctctggatgctcca
1-6tRNA-Met(cat)c[71892,71964]
tgcgggttaacttcagtggtagaatgacgggttcatatcccgttacgcga
tggttcgagtccatcacccgcca
1-7tRNA-His(gtg)c[72279,72354]
gtggccgtagttcagttggtagaactcgagattgtggttctcatagtcat
ggattagaattccacaggtcacccca
1-8tRNA-Gln(ttg)c[72462,72536]
tgaatcgtagccaagctggtaaggcaataggttttgatcctatgatccct
ggttcgagtccaggcggttcagcca
1-9tRNA-Asn(gtt)c[72543,72618]
ggatgtatagctcagctggtagagcggtcgcccgttaagtgattggtccg
cggttcgaatccgtgtacatccgcca
>KF925357.1 Escherichia phage HY01, complete genome
1-1tRNA-Arg(tct)c[68788,68863]
cgaggcatagctcagaaggaagagcaaggaccttctaagtcctaggtcgt
aggttcgatccctactgcctcgacca
1-2tRNA-Asn(gtt)c[68868,68942]
ggatgtgtagctcaatggcagagcgatcgcctgttaagcgattggttata
ggttcgaatcctatcacgtccgcca
1-3tRNA-Tyr(gta)c[68947,69033]
ggggagttattccgtagaggtagcggtgtggactgtaaatccattgtcat
tgcgactcgggtggttcgactccatcactccccacca
1-4tRNA-Met(cat)c[69043,69117]
ggccctgtagctggaaggttcaagcaagcgactcataatcgccagatggt
ggttcaattccacccagggccacca
1-5tRNA-Thr(tgt)c[69119,69194]
gctgatttagctcagtaggtagagcaactcacttgtaatgagaaggtcgg
cggttcgattccgtcaatcagcacca
1-6tRNA-Ser(tga)c[69200,69289]
ggaggcgtggcagagtggtttaatgcaccggtcttgaaaaccggcagtcg
ctccggcgactcataggttcgaatcctatcgcctccgcca

1-7tRNA-Gly(tcc)c[69951,70024]
gcggatatcgtataatggcattacctcagacttccaatctgatgatgtga
gttcgattctcattatccgctcca
1-8tRNA-Leu(taa)c[70030,70116]
gcgagaatggtcaaattggtaaaggcacagcacttaaaatgctgcggaat
gatttccttgtgggttcgagtcccacttctcgcacca
1-9tRNA-Gln(ttg)c[70117,70190]
tgggaattagccaagttggtaaggcactggattttgattccaggatgcaa
aggttcaagtcctttattcccagc
>KJ081346.1 Bacillus phage BCP8-2, complete genome
1-1tRNA-Met(cat)c[33452,33526]
tacggttatagctcagtggtagagtgtgggtctcataagcccaaggtcga
tggttcaatcccatctaaccgtatc
1-2tRNA-Leu(tag)c[33706,33791]
gtcagagtgttggaattggtagacttggcagatttagaatctgctgtcct
agtggcgtgagggttcaagtccctcctctgacacca
1-3tRNA-Ile(gat)c[33800,33876]
actagtgtagctcagtcaggtagagcagtgtcttgataaggcattggtca
caggttcaaatcctgtcactagtacca
1-4tRNA-Leu(taa)c[33947,34033]
atcggaatgttggaattggtagacataacgcacttaaaatgcgttgctcc
ttggggcgtgagggttcgacttcctcttccgatacca
1-5tRNA-Tyr(gta)c[34037,34119]
gggcgtgcaatcattggagagataagctgactgtaaatcagtggtcgttg
actgtggaggttcgaatccttccattcccacca
1-6tRNA-Phe(gaa)c[34126,34200]
ggatagatagctgagatggattagcgaaggattgaaactccttagaggtt
ggatcgttaccaactctgtccacca
1-7tRNA-Pro(tgg)c[34298,34369]
gtagatgtaggctagaggtcagtcactccgtttggggcggaggtcacgca
ggttcgatccctgccatctgca
1-8tRNA-His(gtg)c[34477,34550]
cagtgtgtggtgaagtggcttaacacgaacgactgtggctcgtttattcg
caggttcgaatcctgtcatgctga
1-9tRNA-Gln(ttg)c[34627,34703]
tttcgggatggacaaactggtaaagtcactaggctttgaccctaggtttt
ggaggttcgaccccttctcccgaagtc
1-10tRNA-Ser(tga)c[34778,34865]
ggagggctactctaactggtaagagacctgccttgaaagcagacgtaggg
taaaaccgataggagttcgagtctcctgccctcctcca
1-11tRNA-Arg(tct)c[35009,35083]
acctctatcgccaagcggatcaaggcaacgggcttctatcccgttaatcg
ggagttcgaatctctctaggggtgc
1-12tRNA-Glu(ttc)c[35086,35161]

tgtcgcattggtgaagcggtctaacacactcggctttctaccgagcatac
gcaggttcgaatcctgtatgcgatat
1-13tRNA-Gly(tcc)c[35666,35741]
ggggcattcgtatagtggcttattatacctggcttccaaccaggggaggt
ggtttcgattaccacatgtccctcca
1-14tRNA-Thr(tgt)c[35747,35823]
gccttcttagctcagttggttagagcgactgccttgtaagcagtaggtcg
tgcgttcgaatcgtacagtcggcacca
1-15tRNA-Asp(gtc)c[35836,35909]
ggctcgttagtttaacggtaaaatacatgactgtctatcatgggtcacgg
gttcaactcccgtacgggtcgcca
1-16tRNA-Asn(gtt)c[36104,36179]
gtgcctgtagctcagttggtagagcagaaccctgttaaggttaaggtcgt
aggttcgaaccctaccaggtacgcca
1-17tRNA-Cys(gca)c[36225,36298]
gagggtgtaccgaagcggctcaacggcacagattgcaaccctgttgttca
ggagttcgaatctcctcatcctct
1-18tRNA-Ser(gct)c[36309,36396]
ggaaaggtactcaagtcggtgaagagatcagtttgctaaactgatagtac
ctgtaaaggtagcaggggttcgagtcccttcctttcca
>JX181824.1 Salmonella phage SSE-121, complete genome
1-1tRNA-Thr(tgt)[28,102]
gctcctaaagcataagtggcgatgcagcggccttgtaagccgaagaagtg
ggttcgattcctactgggagcacca
1-2tRNA-Ile(gat)[109,184]
tcctcgttagctcaagttggtagagcagtcccaagattcgggataagcac
aggttcgattcctgtacgaggagcca
1-3tRNA-Leu(taa)[973,1058]
gcaggtgtggtggaaatggagatacacaggagacttaaaatctcccgctt
aaatgattacgagttcgagtctcgtcacctgcacca
1-4tRNA-Arg(tct)[1066,1140]
cggggtgtagtctaagggataggcagcggtcttctaaaccgctcaatgct
ggttcgaatccagccacctcgacca
1-5tRNA-Cys(gca)[1259,1332]
gcacggttgctggagtggcaacagcttcggctgcaaaccgaatgtcagga
gttcgattcttctaccgtgctcca
1-6tRNA-Tyr(gta)[1985,2068]
ggctcattggcagaacggtgattgcagcggattgtaaatccgtgcccttc
ggggttcctggttcgaatccagggtgggccacca
1-7tRNA-Ile(gat)[2076,2151]
gggagtatagctcagttagtagagcgctcgaccgataatcgagaggtcgc
aggagcaaagcctgctactcccacca
1-8tRNA-Gly(tcc)[2159,2232]
gcgtcattagtgttagcggtccagcatcccgtccttccaagtcggtggca

tcggttcgaatccgatatgacgct
1−9tRNA−Glu(ttc)[2240,2316]
gtcctgttagacaaactggtaaagtcaccaccctttcaaggtggggtttg
cgggttcgatccccgcacaggacgcca
1−10tRNA−Gln(ttg)[2406,2482]
agggggttagcatagctggcctaatgcatcgggctttgaactcgacatcg
gaggttcgaatcctccaccccctgcca
1−11tRNA−Ser(gga)[2596,2687]
ggtggaatggtcgagcggtttaagacagcatcttggaaaggtgtcggccc
cttaacaggggtccgtaggttcaaatcctacttccaccgcca
1−12tRNA−Ser(gct)[2982,3074]
ggaagattaaccctaatcaggtaagggatctctttgctaaagagacagta
gccccgaaaggggtgtgtcagttcaagtctgacatcttcctcc
1−13tRNA−Ser(tga)[3075,3163]
ggagagcaggacgcatggtgcgtaatccggtttgaacccggacccatcgt
agcgatacggtgacagttcgattctgttgctctcctcca
1−14tRNA−Asn(gtt)[3316,3400]
gacgagttggcctagtggttgggcgacggcctgttaagccgtgagtgaaa
actcaaggaaggttcaaatccttcactcgtcgcca
1−15tRNA−Asp(gtc)[3408,3484]
ggggccgtggctgacaaggtaatggtgccgcactgtctatgcggaaaatg
agggttcgatccccttcggcctcgcca
1−16tRNA−Pro(tgg)[3582,3659]
ctgttcgtagcgcagtctggtcagcgtgggtcccttggaaggatcaggtc
ggtggttcaaatccacccggacagacca
1−17tRNA−Arg(acg)[3847,3923]
gcgagcatagcttaattgaatagagcttcgtcatacgaaggcggaagatc
ggggtttgaatccctgtacttgcacca
1−18tRNA−Ala(tgc)[3927,4005]
ggggaatgggtctgcttggagtggacacctcgcttgcaccgaggatatca
gaagagttcgaatctcttattctccacca
1−19tRNA−Leu(tag)[4112,4188]
gggcgattgatggaattggtatacgtgccgtccttagaagtcggattttg
ggggttcgagtcccctgtcgcccacca
1−20tRNA−Val(tac)[4196,4270]
gcttccttagttcaatggtagaacgttgtctttacacggcaagcgtcggt
ggttcgattccatcaggaagtacca
1−21tRNA−Phe(gaa)[4278,4353]
ggggttgtaactcagttggtagagtgcctgcctgaagagcagatggtcgg
tggttcgagtccacccggccccacca
1−22tRNA−Ala(ggc)[4360,4441]
tgcgtggtagtgaaacggatatatagatcatgttggcctcataagccgaa
gattgcgggttcgactcccgctcacgcatcca
1−23tRNA−Met(cat)[144815,144891]

ggttctgtagcttagttggttagagcgcttccctcataaggaagaggtcg
caggttcaattcctgccagaactacca
1-24tRNA-Lys(ctt)[145584,145659]
ggggtattaacacagcggttagtgtagcggcctcttaagccgaaggtcga
aggttcgaatccttcatgccccacca
>AB897757.1 Klebsiella phage K64-1 DNA, complete genome
1-1tRNA-Leu(taa)[177141,177228]
ggccgtatgtccaaattggtaaaggagacagacttaaaatctgttggtga
tattcaccttctcgattcgagttcgagtgcggctacca
1-2tRNA-Gly(tcc)[177595,177670]
gcggttatcgtataaaggctattacctcggttttccaaaccgatgatgag
ggttcgacgtccctctaaccgctcca
1-3tRNA-Arg(tct)[178961,179034]
gtccccttagttcagttggatagaacaactgccttctaagcagtaggtca
ctggttcgagtccagtaggggata
1-4tRNA-Ser(gct)[179186,179274]
tagtgagttggatgagtggttgaaatcaccaccctgctaaggtggcatat
gcgaaagcgtatcgtgggttcgaatcccacactcactgt
1-5tRNA-Ser(tga)[179335,179422]
ggaaggatggccgagtggtttaaggcactggtcttgaaaaccagcaatcc
gaaagggttctagagttcaaatctctatccttccacca
1-6tRNA-Met(cat)[179541,179618]
ggccctttactctcaagtggttagagaatccgactcataatcggtaggct
ctgagttcgaatctcagaagggccacca
1-7tRNA-Asn(gtt)[180355,180428]
gactctgtagcacaatcggtggtgcgattgactgttaatcaattggttgg
ggattcgagttcctccagagtcgc
>JX181825.1 Salmonella phage STML-198, complete genome
1-1tRNA-Arg(tct)c[102970,103046]
cgaggcatagcttaatagtatagagcaacggtcttctaaaccgtaggttg
aaggttagaatccttctgtctcgacca
1-2tRNA-Gln(ttg)c[103686,103761]
tgggaattagccaagttggtaaggcaccggattttgattccgggatgcac
tggttcgagcccagtattcccagcca
1-3tRNA-Met(cat)c[103768,103843]
ggccccttagctcagtggttagagcaggcgactcataatcgcttggtcgc
tggttcaagtccagcaggggccacca
>KM657822.1 Escherichia phage vB_EcoM-VpaE1, complete genome
1-1tRNA-Pro(tgg)[25483,25559]
ctctgtttagctcagcttggtagagcgttccgtttggggcggtaaggccg
gaggttcaagtcctccaacagagacca
1-2tRNA-Glu(ttc)[25567,25644]
gttccagtagacaaaatggtatagtcaccactctttcaaagtggatattt
gagggttcaaatcccttctggaacgcca

1-3tRNA-Met(cat)[25736,25812]
tgcgggtatagagaaagggcgtctcacatgtctcattagcatggtatcgg
caggttcgactcctgcacccgcctcca
1-4tRNA-Asn(gtt)[25898,25974]
ggttaggaagcacataaggtatgtgcggtcgcctgttaagcgaatggcac
agggttcgaatccctgactaaccgcca
1-5tRNA-Tyr(gta)[25984,26071]
gtgtcgttatcccgtagatggtagcggtggggactgtaaatcccttgtca
ttgagactcggtaggttcgactcctacacggcacacca
1-6tRNA-Asp(gtc)[26077,26154]
ggttatgtagtttaatctggttaaaatactcccctgtcacgggagatgat
gagggttcgaatcccttcgtaaccgcca
1-7tRNA-Lys(ttt)[26584,26659]
ggaagtgtagcagaatggtgatgcggcagacttttaatctgacaggcgat
gggttcgaatccctccacttctacca
1-8tRNA-Met(cat)[26664,26740]
ggttcagtcgcagataaggtaatgcaggggtctcataagccctatgaatg
tgggttcgattcccatctgaacctcca
1-9tRNA-Ile(gat)[26743,26817]
tgtgggttagcataaatggtaatgcaaacggctgataaccgttagaagag
ggttcgataccctcacctacaacca
1-10tRNA-Arg(tct)[27067,27143]
gcacccttagttcaactggacagagcaaatgacttctaatcattgagtta
caggttcgaatcctgtagggtgtgcca
1-11tRNA-Ser(tga)[27382,27471]
ggtaggtagcggctaatggtagccaaactgtcttgaaaacagttgccact
gtagagatacggtgagggttcgactcctttacttaccgcc
1-12tRNA-Leu(tag)[27728,27805]
gggagattgacggtaattggtaaacctatctcgcttagaacgagatgttt
gagggttcgaatcccttgtctcctacca
1-13tRNA-Lys(ctt)[27813,27888]
gcaggtgtagcaaaatggttatgcggctgactcttaatcagtaagacgat
gggttcaattccctccacctgtacca
1-14tRNA-Ala(tgc)[27895,27970]
ggggtcatagtttatatggttaaaattcgagttttgcaaacttgggaact
gagttcaattctcagtgactccacca
1-15tRNA-Gly(tcc)[27977,28051]
gcatccatagtttaaacgggaaaattacagtcttccaaactgaggttgag
ggttcgattccctctggatgctcca
1-16tRNA-Thr(tgt)[28058,28134]
gctgctttcgtataattggctattacacatcccttgtaaggatggaaatg
caggttcgagtcctgtgagcagcacca
1-17tRNA-Val(tac)[28230,28304]
actcgcttagtttatatggtaaaacatcacccttacaagatgaagaaaaa

ggttcaagtcctttagtgagtacca
1−18tRNA−Leu(caa)[28306,28383]
gttccagtatcccaattggcagaggatgcaagctcaaaccttgtactagt
gacggttcgaatccgtcttggaacacca
1−19tRNA−Gln(ttg)[28969,29044]
aggggattagtttacaaggttaaaacctcggtctttgaaatcgaagaagt
tggttcaattccaacatcccccgcca
1−20tRNA−Leu(taa)[29047,29125]
gctccattactccaattggcagagaggccagacttaaaatctgtgttatg
tatcggttcgaatccgatatggagtacca
1−21tRNA−Gln(ctg)[29131,29206]
agcggtatagcataactggcaatgcaacagtctctgaagctgtcctatta
aggttcaaatccttatgccgctgcca
1−22tRNA−His(gtg)[29238,29313]
gtggccttatcataaatggtaatgacccatgctgtgaacatggtctatac
gggttcaaatcccgtaggtcacccca
1−23tRNA−Phe(gaa)[29320,29395]
agtccaagtagcttatatggttaaagcgcgtgtctgaaaaacatgagaag
agggttcaaatcccactggactacca
1−24tRNA−Ser(gct)[30148,30240]
ggaagattaaccctaaaaggtaagggagcagtttgctaaactgctagtag
ctgagaaatcggtgtaccagttcaagtctggtatcttcctcca
1−25tRNA−Cys(gca)[30245,30320]
gaatccgtgacagaaatggatatgtgcctgtctgcaaaacaggtttataa
gggttcaagtcccttcggattctcca
>HE956711.1 Yersinia phage phiD1 complete genome
1−1tRNA−Arg(tct)c[68808,68883]
cgaggcatagctcagaaggaagagcaaggaccttctaagtcctgggtcgt
aggttcgatccctactgcctcgacca
1−2tRNA−Asn(gtt)c[68888,68962]
ggatgtgtagctcaatggcagagcgatcgcctgttaagcgattggttata
ggttcgaatcctatcacgtccgcca
1−3tRNA−Tyr(gta)c[68967,69053]
ggggagttatcccgtagaggtagcggtgtggactgtaaattcattgtcat
tgcgactcgggtggttcgactccatcactccccacca
1−4tRNA−Met(cat)c[69066,69140]
ggccctgtagctggaaggttcaagcaagcgactcataatcgccagatggt
ggttcaattccacccagggccacca
1−5tRNA−Thr(tgt)c[69142,69217]
gctgatttagctcagtaggtagagcacctcacttgtaatgaggacgtcgg
cggttcgattccgtcaatcagcacca
1−6tRNA−Ser(tga)c[69224,69313]
ggaggcgtggcagagtggtttaatgcaccggtcttgaaaaccggcagtcg
ctccggcgactcataggttcaaatcctatcgcctccgcca

1-7tRNA-Pro(tgg)c[69315,69389]
ctccgtgtagctcagtttggtagagcgcctgatttgggatcaggaggtcc
aaggttcaaatccttgtatggagac
1-8tRNA-Gly(tcc)c[69400,69473]
gcggatatcgtataatggtattacctcagacttccaatctgatgatgtga
gttcgattctcattatccgctcca
1-9tRNA-Leu(taa)c[69479,69565]
gcgagaatggtcaaattggtaaaggcacagcacttaaaatgctgcggaat
gatttccttgtgggttcgagtcccacttctcgcacca
>KF208315.1 Yersinia phage PST, complete genome
1-1tRNA-Arg(tct)c[67617,67691]
gtcccgctggtgtaatggatagcatacgatccttctaagtttgcggtcct
ggttcgatcccagggcgggatacca
1-2tRNA-Tyr(gta)c[68409,68495]
ggggagttatcccgtagaggtagcggtgtggactgtaaattcattgtcat
tgcgactcaggtggttcgactccatcactccccacca
1-3tRNA-Met(cat)c[68505,68581]
ggccctgtagctcaatggttagcagcagtcccctcataagggaaaggtta
ccagttcaaatctggtctgggtcacca
1-4tRNA-Thr(tgt)c[68583,68658]
gctgatttagctcagtaggtagagcacctcacttgtaatgaggatgtcgg
cggttcgattccgtcaatcagcacca
1-5tRNA-Ser(tga)c[68660,68748]
tggaggcgtggcagagtggtttaatgcaccggtcttgaaaaccggcagtc
gctccggcgactcataggttcaaatcctatcgcctccgt
1-6tRNA-Pro(tgg)c[68749,68823]
ctccgtgtagctcagtttggtagagcgtctgctttgggagcagaatgtcg
caggttcaaatcctgccgcggagac
1-7tRNA-Gly(tcc)c[68834,68907]
gcggatatcgtataatggtattacctcagacttccaatctgatgatgtga
gttcgattctcattatccgctcca
1-8tRNA-Leu(taa)c[68913,68999]
gcgagaatggtcaaattggtaaaggcacagcacttaaaatgctgcggaat
gatttccttgtgggttcgagtcccacttctcgcacca
1-9tRNA-Gln(ttg)c[69000,69073]
tgggaattagccaagttggtaaggcatagcactttgactgctagatgcaa
aggttcgagtcctttattcccagc
>KM672662.1 Acinetobacter phage YMC13/03/R2096, complete genome
1-1tRNA-Tyr(gta)[16051,16136]
gggtgatgttggggtgttggtcaaccccatcagactgtaaatctgacgtg
aaagctgtctaggttcgaatcctagatcacccacca
1-2tRNA-Ile(gat)[16140,16214]
agtaccttagttcaggggtagaacgcagaactgataattctgaggtcgat
ggttcgaaaccatcaggtactacca

1–3tRNA–Asn(gtt)[16244,16317]
tctagtgtagcacaacggtagtgcagcagactgttaatctgttggttgtt
ggttcgaatccagcctctagagcc
1–4tRNA–Asp(gtc)[17058,17133]
ggctctgtagcatagtggattatgcgcctccctgtctaggagaaaaacgc
cagttcgagtctggtcagagtcgcca
1–5tRNA–Gln(ttg)[17138,17212]
tgtcgtatagtgtaatggttagcacagaagactttgactcttctagttta
ggttcgagtcctaattcgactacca
1–6tRNA–Ala(tgc)[17216,17287]
gggtcactagcacaatggtagtgcagttgctttgcaagcatcaggttaag
ggttcgagtcccttgtgtatcc
1–7tRNA–Glu(ttc)[17294,17368]
attccattcgactagcggttaggtcgcttccctttcaaggaagtagcacg
agttcgaatctcgtatggaatacca
1–8tRNA–Lys(ttt)[17371,17447]
aggacgttagctcagttggttagagcaagagccttttaagttcgaggtca
taggttcaaatcctatacgtcctacca
1–9tRNA–His(gtg)[17454,17527]
gtgactttggtgtaatggtttgcatcactccctgtgaaggagaaggtcag
ggttcaaatcccgaagtcacacca
1–10tRNA–Val(tac)[18226,18301]
agtcccttagctcaatcgggagagcaacactcttacaaggtgaaggtagt
aggttcgaaacctacagggactacca
1–11tRNA–Ser(tga)[18303,18391]
ggtgggtagcgtcaaggtgacaaagcgggcttgaaccccgtgccaattgt
gatgagcagttgagagttcgattcttttacccatcgcca
1–12tRNA–Gly(tcc)[18393,18466]
gcgtttatagtgtagtggtaacaccctgtacttccaattcagtatcttcg
gttcgaatccgaatagacgctcca
1–13tRNA–Pro(tgg)[18620,18696]
ctctgtgtagctcagtttggtagagcactcgctttgggagtgaggggtcg
taagttcaattcttactacagagacca
1–14tRNA–Leu(tag)[18784,18860]
gggtagttgaagggaatggcatacctttcacgcttagaacgtgaagtttc
tgagttcgagtctcagactacctacca
1–15tRNA–Leu(taa)[18867,18942]
ggggatatagcccaacggcagaggcaagaggtttaaaccctctcaagttt
cagttcgaatctgaatatccctacca
1–16tRNA–Thr(tgt)[19336,19411]
gccgaattagctcagttggtagagcagcgcacttgtaatgcgaaggtcgt
agactcggattctacattcggcacca
1–17tRNA–Met(cat)[19784,19858]
agcgaggtagtgtaacggtttgcacaggggactcataatcctctggtttg

ggttcgaatcccaacttcgcttcca
>JX509734.1 Enterobacteria phage SfI, complete genome
1−1tRNA−Asn(gtt)[35738,35812]
gggtcagtcgtataaaggtcattacggaaggctgttaaccttcttatcgt
ggttcgagtccacgctgtcccgcca
1−2tRNA−Thr(tgt)[35818,35893]
gctggtttagctccaatggtagagcggtcgccttgtaagcgaatgggtag
cggttcaagtccgttaaccagcacca
>JN641803.1 Salmonella phage SPN3US, complete genome
1−1tRNA−Trp(cca)[1771,1846]
gggggtatggcgcaattggtagcgcaccggtctccaaaaccgaaggttcg
aggttcgaaacctcgtgccccccgcca
>FN667789.1 Campylobacter phage CPt10, complete genome
1−1tRNA−Arg(tct)[112367,112443]
cgggatgtagctcagctggatagagcgacagccttctaagccgtaggtca
aaggttcgagtcctttcatcccgacca
1−2tRNA−Tyr(gta)[112455,112540]
gtaagcattcgggtaattggtaacccaccagactgtaaatctggcgtctc
ttggcactgcaggttcgagtcctgctgcttacacca
>GU169904.1 Staphylococcus phage SA1, complete genome
1−1tRNA−Tyr(gta)[58449,58536]
gtgtcgttatcccgtagatggtagcggtggggactgtaaatcccttgtca
ttgagactcggtaggttcgactcctacacggcacacca
1−2tRNA−Asp(gtc)[58542,58618]
ggttatgtagtttaatttggttaaaatacccccctgtcacgggagatgatg
agggttcaaatcccttcgtaaccgcca
1−3tRNA−Lys(ttt)[59047,59122]
ggaagtgtagcagaatggtgatgcggcagacttttaatctgacaggcgat
gggttcgaatccctccacttctacca
1−4tRNA−Met(cat)[59127,59203]
ggttcagtcgcagataaggtaatgcaagggtctcataagccctatgaatg
tgggttcgattcccatctgaacctcca
1−5tRNA−Ile(gat)[59206,59280]
tgtgggttagcataaatggtaatgcaaacggctgataaccgttagaagag
ggttcgataccctcacctacaacca
1−6tRNA−Arg(tct)[59617,59693]
gcacccttagttcaattggatagagcaacggtcttctaaatcgttagtta
caggttcgaatcctgtagggtgtacca
1−7tRNA−Ser(tga)[59932,60021]
ggtaggtagcggctaatggtagccaaactgtcttgaaaacagttgccact
gtagagatacggtgagggttcgactcctttacttaccgcc
1−8tRNA−Leu(tag)[60278,60355]
gggagattgacggtaattggtaaacctaccatccttagaagttggtgttt
gagggttcgaatcccttgtctcctacca

1-9tRNA-Lys(ctt)[60363,60438]
gcaggtgtagcaaaatggttatgcggctgactcttcatcagtaagacgat
gggttcaattccctccacctgtacca
1-10tRNA-Ala(tgc)[60445,60520]
ggggtcatagtttatatggttaaaattcgagttttgcaaacttgggaact
gagttcaattctcagtgactccacca
1-11tRNA-Gly(tcc)[60527,60601]
gcatccatagtttaaacgggaaaattacagtcttccaaactgaggttgag
ggttcgattccctctggatgctcca
1-12tRNA-Thr(tgt)[60609,60684]
gctcctatcgtataattggctattacggttgccttgtaagcaacttatca
gggttcgaatccttgtgggagcacca
1-13tRNA-Val(tac)[60780,60854]
actcgcttagtttatatggtaaaacatcacccttacaagatgaagaaaaa
ggttcaagtcctttagtgagtacca
1-14tRNA-Leu(caa)[60856,60933]
gttccagtatcccaattggcagaggatgcaagctcaaaccttgtattagt
gacggttcgaatccgtcttggaacacca
1-15tRNA-Arg(acg)[61048,61123]
gcaggattagttcaaatggatagagcaacagtctacgaagctgttaatag
gggttcgaatcccttatcctgcgcca
1-16tRNA-Gln(ttg)[61947,62022]
aggggattagtttacaaggttaaaacctcggtctttgaaatcgaagaagt
tggttcaattccaacatccccctcca
1-17tRNA-Leu(taa)[62025,62103]
actccattactccaattggcagagaggccagacttaaaatctgtgttatg
tatcggttcgaatccgatatggagtacca
1-18tRNA-Gln(ctg)[62109,62184]
agcggtatagcataactggcaatgcagcagtctctgaagctgtctcatta
aggttcaaatccttatgccgctgcca
1-19tRNA-His(gtg)[62216,62291]
gtggccttatcataaatggtaatgacccatgctgtgaacatggtctatac
gggttcaaatcccgtaggtcacccca
1-20tRNA-Phe(gaa)[62298,62373]
agtccaagtagcttatatggttaaagcgcgtgtctgaaaaacatgagaag
agggttcaaatcccactggactacca
1-21tRNA-Ser(gct)[63126,63218]
ggaagattaaccctaaaaggtaagggagcagtttgctaaactgccagtag
ctgagaaatcggtgtaccagttcaagtctggtatcttcctcca
1-22tRNA-Cys(gca)[63223,63298]
gaatccgtgacagaaatggctatgtgcctgtctgcaaaacaggtttataa
gggttcaagtcccttcggattctcca
1-23tRNA-Pro(tgg)[146853,146929]
ctctgtttagctcagcttggtagagcgttccgtttggggcggtaaggccg

gaggttcaagtcctccaacagagacca
1-24tRNA-Glu(ttc)[146937,147014]
gttccagtagacaaaatggtaaagtcaccactctttcaaagtggatattt
gagggttcaaatcccttctggaacgcca
1-25tRNA-Met(cat)[147105,147181]
tgcgggtatagagaaagggcgtctcacatgtctcattagcatggtatcgg
caggttcgactcctgcacccgcctcca
>FN667788.1 Campylobacter phage CP220, complete genome
1-1tRNA-Arg(tct)[111946,112022]
cgggatgtagctcagctggatagagcgacagccttctaagccgtaggtca
aaggttcgagtcctttcatcccgacca
1-2tRNA-Tyr(gta)[112034,112119]
gtaagcattcgggtaattgttaacccaccagactgtaaatctggcgtctc
ttggcactgcaggttcgagtcctgctgcttacacca
>AP011113.1 Enterobacteria phage AR1 DNA, complete genome
1-1tRNA-Arg(tct)c[68329,68404]
cggggcatagctcagaaggaagagcaaggaccttctaagtcctaggtcgt
aggttcgatccctactgcctcgacca
1-2tRNA-His(gtg)c[68409,68484]
gtggccgtagttcagttggtagaactcgagattgtgattctcgtagtcat
gggttcgactcccatcggtcacccca
1-3tRNA-Asn(gtt)c[68598,68672]
ggatgtgtagctcaatggcagagcgatcgcctgttaagcgattggttata
ggttcgaatcctatcacgtccgcca
1-4tRNA-Tyr(gta)c[68677,68763]
ggggagttatcccgtagaggtagcggtgtggactgtaaatccattgtcat
tgcgactcgggtggttcgactccatcactccccacca
1-5tRNA-Met(cat)c[68777,68851]
ggccctgtagctggaaggttcaagcaagcgactcataatcgccagatggt
ggttcaattccacccagggccacca
1-6tRNA-Thr(tgt)c[68853,68928]
gctgatttagctcagtaggtagagcacctcacttgtaatgaggatgtcgg
cggttcgattccgtcaatcagcacca
1-7tRNA-Ser(tga)c[68934,69023]
ggaggcgtggcagagtggtttaatgcaccggtcttgaaaaccggcagtcg
ctccggcgactcataggttcaaatcctatcgcctccgcca
1-8tRNA-Gly(tcc)c[69685,69758]
gcggatatcgtataatggtattacctcagacttccaatctgatgatgtga
gttcgattctcattatccgctcca
1-9tRNA-Leu(taa)c[69764,69850]
gcgagaatggtcaaattggtaaaggcacagcacttaaaatgctgcggaat
gatttccttgtgggttcgagtcccacttctcgcacca
1-10tRNA-Gln(ttg)c[69851,69924]
tgggaattagccaagttggtaaggcactggattttgattccaggatgcaa

aggttcgagtcctttattcccagc
>LN881727.1 Escherichia phage slur16, complete genome
1-1tRNA-Pro(tgg)c[95063,95139]
ctccgcgtagctcagcttggtagagcgcctgatttgggatcaggaggtcg
agtgttcgaatcactccgtggagacca
1-2tRNA-Met(cat)c[95151,95225]
ggccctgtagctggaaggttcaagcaagcgactcataatcgccagacggt
ggttcaattccacccagggccacca
1-3tRNA-Thr(tgt)c[95227,95302]
gctgatgtagcacaattggtagtgcaattgatttgtaatcaataggttgc
aggttcaagtcctgccatcagcacca
1-5tRNA-Ser(tga)c[95506,95596]
tgagggtagggagcaatggtgctcaagcggtcttgaaaaccgtcccgttg
aggatgactcgatgatggttcgattccattaccctcagcca
1-6tRNA-Tyr(gta)c[95711,95797]
ggggagttatcccgtagaggtagcggtgtagactgtaaatctattgtcat
tacgactcgggtggttcgactccaccactccccacca
1-7tRNA-Arg(tct)c[95802,95876]
gccctgttagcttagtggataaagcagcggccttctaagccgttgacact
ggttcgagtccagtacggggtgcca
>LN881736.1 Escherichia phage slur14, complete genome
1-1tRNA-Arg(tct)c[12042,12117]
cggggcatagctcagaaggaagagcaaggaccttctaagtcctaggtcgt
aggttcgatccctactgcctcgacca
1-2tRNA-His(gtg)c[12122,12197]
gtggccgtagttcagttggtagaactcgagattgtgattctcgtagtcat
gggttcgactcccatcggtcacccca
1-3tRNA-Asn(gtt)c[12312,12386]
ggatgtgtagctcaatggcagagcgatcgcctgttaagcgattggttata
ggttcgaatcctatcacgtccgcca
1-4tRNA-Tyr(gta)c[12391,12477]
ggggagttatcccgtagaggtagcggtgtggactgtaaattcattgtcat
tgcgactcgggtggttcgactccaccactccccacca
1-5tRNA-Met(cat)c[12487,12561]
ggccctgtagctggaaggttcaagcaagcgactcataatcgccagatggt
ggttcaattccacccagggccacca
1-6tRNA-Thr(tgt)c[12563,12638]
gctgatttagctcagtaggtagagcacctcacttgtaatgaggacgtcgg
cggttcgattccgtcaatcagcacca
1-7tRNA-Ser(tga)c[12645,12734]
ggaggcgtggcagagtggtttaatgcaccggtcttgaaaaccggcagtcg
ctccggcgactcataggttcaaatcctatcgcctccgcca
1-8tRNA-Pro(tgg)c[12736,12810]
ctccgtgtagctcagtttggtagagcgcctgatttgggatcaggaggtcc

aaggttcaaatccttgtatggagac
1-9tRNA-Gly(tcc)c[12821,12894]
gcggatatcgtataatggtattacctcagacttccaatctgatgatgtga
gttcgattctcattatccgctcca
1-10tRNA-Leu(taa)c[12900,12986]
gcgagaatggtcaaattggtaaaggcacagcacttaaaatgctgcggaat
gatttccttgtgggttcgagtcccacttctcgcacca
>KT804923.1 Pseudomonas phage C11, complete genome
1-1tRNA-Gln(ttg)[22320,22393]
tgccgcttcgttcaatggtaggacgccagactttgaatctggagatgatg
gttcgatcccatcagcggctgcca
1-2tRNA-Arg(tct)[22705,22780]
gctcatatagtgtaatggacagcacaacggtcttctaagccgtaaggtct
aggttcgaatcctagtatgagcgcca
1-3tRNA-Lys(ttt)[22790,22865]
tggacggaagctaaagtggatcagcatctggcttttaaccagactatagt
gagttcgagtctcacccgtccaacca
1-4tRNA-Leu(tag)[23140,23224]
ggccctgtggtggaattggtatacacatcagtcttagaaactgacgccga
gaggattgagggttcaagtccctccggggccacca
1-5tRNA-Ile(gat)[23435,23510]
agccgggtagctcaattggcagagcacccgaccgataatcgggtggttga
aggttcaagtccttctctggctacca
1-6tRNA-Asp(gtc)[23520,23598]
ggcccattagctcagtctggactagagcaagcccctgtctagggcaaggt
cgtcggttcgaatccgacatgggtcgcca
1-7tRNA-Met(cat)[23699,23773]
agcgttaagactggctggagctgtcactcggctcataaccgagcacaagt
ggttcgattccacctctcgctacca
1-8tRNA-Cys(gca)[24055,24130]
cccgcgttggccgagaggtttaggcggcggattgcaaatccgtctcacat
cggttcaaatccgatacgcggctcca
1-9tRNA-Asn(gtt)[24141,24218]
tgggatgtagctcagttggtagagcagggagctgttaactctcaggacgg
cagggttcgaaccctgccgtcctagcca
1-10tRNA-Pro(tgg)[24281,24358]
ctcctcgtagctcagtctggttagagtgtcggatttggaatccgaaggtc
gaaggttcaaatccttccggggtgacca
1-11tRNA-Gly(tcc)[24530,24605]
gcgggtatagctcagttggtagagcgtctgccttccaagcagttcgtcgt
cggttcgagtccgtctatccgctcca
1-12tRNA-Phe(gaa)[24612,24687]
gcatttgaagctaactaggtagaagcgctgggttgaaattccagaggact
tggatcgttaccaagcagatgcacca

1–13tRNA–Glu(ttc)[24694,24769]
gcagttatagattaattggttaaatcgccagactttcaatctggtgttcc
gggttcgatccccggtaactgctcca
1–14tRNA–His(gtg)[24830,24904]
gtggagattgtgtagcccggatgctgcctttcggttgtgacccgattgtt
ggggttcgagtccccgcctccaacc
1–15tRNA–Thr(tgt)[25091,25165]
gccctttaagcatttatggtgatgcaccggctttgtaacccggcgaattc
tgttcaagtcaggaatggggcacca
>AP014714.1 Edwardsiella phage PEi20 DNA, complete sequence
1–1tRNA–Asp(gtc)c[75815,75890]
ggacctatagtttcagcggttaaaatactcccctgtcacgggagcgtcac
gagttcgaatctcgttaggtccgcca
1–2tRNA–His(gtg)c[76114,76189]
gtggccttaattcagttggtagaattcaagattgtgattcttgtagtcat
gggttcaagtcccataggtcacccca
1–3tRNA–Asn(gtt)c[76223,76298]
ggatgtgtagctcagttggtagagcggtcgcccgttaagtgattggtccg
cggttcgaatccgtgcacatccgcca
1–4tRNA–Lys(ttt)c[76844,76921]
aggacgttagctcagctggttagagcactcgacttttaatcgagatgtcg
atgggttcaaatccctcacgtcctacca
1–5tRNA–Met(cat)c[77987,78063]
ggccctatagctcaattggttagagcgcccggctcataactggtaggttt
ccggttcaagtccggatggggccacca
1–6tRNA–Leu(taa)c[78529,78617]
gcgggtgtggcggtattggcagtacgcatggcacttaaaatgccataggg
cttaagctcttacgagttcgaatctcgtcacccgtacca
>KT353109.1 Cronobacter phage PBES 02, complete genome
1–1tRNA–Thr(tgt)[50654,50729]
gctggattagctcagtaggtagagcgcctcacttgtaatgaggatgtcgc
gggttcgattcctgcatccagcacca
1–2tRNA–Cys(gca)[50736,50812]
gcaacgttgccggagtctggtaacggctctcgctgcaaacgagatgagca
ctggttcgaatccagtacgttgctcca
1–3tRNA–Leu(caa)[50814,50890]
gcgtctttggtccaattggtagaggcactacattcaaaatgtaggggttc
ccagttcgaatctgggaaggcgcacca
1–4tRNA–Tyr(gta)[51485,51571]
gctgtgatagaccgtaggggtagcggggcagactgtaaatctgctgttca
tagaactcgggtggttcgactccatctcgcagcacca
1–5tRNA–Ser(gga)[51579,51673]
ggtaggatggctgagtggcttaaagcagcatcttggaaaggtgtcggtcg
tggtaaccccacggcccgagagttcgaatctctctcctacctcca

1−6tRNA−Asn(gtt)[51844,51919]
tgacggtgagcatatatggacatgcggggacctgttaagtccaggataac
tggttcgaatccagtaccgtcagcca
1−7tRNA−Glu(ttc)[51958,52035]
gtcctggtagacaaacttggtaaagtcaccaccctttcacggtggagttt
gagggttcgatcccctctcaggacgcca
1−8tRNA−Asp(gtc)[52130,52205]
ggtcctatggtatcagcggtaaacatactcgcctgtcacgcgagagtcac
gggttcgaatcccgttaggaccgcca
1−9tRNA−Ile(gat)[52210,52285]
tggggattagctcagttggtagagcaggctcttgataagggtcaggtcac
tggttcaagtccagtatccccaacca
1−10tRNA−Pro(tgg)[52356,52432]
cggtgtatagcgcagtctggtagcgcgaatgctttgggagcattaggtcg
ttggttcgagtccaactacaccgacca
1−11tRNA−Gly(tcc)[52470,52543]
gcgggcatggtatagtggttgtgccttagccttccaagctaatgagtccg
gttcgatcccggatgtccgctcca
1−12tRNA−Gln(ttg)[52583,52658]
tgggggctagtttaattggtagaacagcgggttttgatcccgttagcgcg
aggttcgaaccctccgcccccggcca
1−13tRNA−Ser(gct)[53125,53211]
agaagagtggcgaaatcggtagccgcaccggactgctaatccggggtcct
tcggggcctgagagttcaagtctctcctcttctgcca
1−14tRNA−Phe(gaa)[53299,53374]
ggaactgtagctcagttggtagagcggttgcctgaagagcagcgcgtcag
cggttcaaatccgttcagttccacca
>KT239446.1 Klebsiella phage JD18, complete genome
1−1tRNA−Thr(tgt)c[57172,57247]
gccgatttagctcagctggtagagcgcttcacttgtaatgaagatgtcgc
gggttcgactcctgcaatcggcacca
1−2tRNA−Leu(taa)c[57348,57432]
gcatcgatggtggaactggtatacacaggagacttaaaatctcccgccgc
aaggattgagggttcgaatccctctcggtgcacca
1−3tRNA−Arg(tct)c[57437,57513]
cggggcatagctcagttggatagagcagcggacttctaatccgcaggtcg
aaggttcgaatccttctgcctcgacca
1−4tRNA−Met(cat)c[58860,58936]
ggccctgtagctcaattggtagagcgttcccctcataagggattggttgc
atgttcgagtctttgccagggtcacca
1−5tRNA−Pro(tgg)c[60204,60280]
ctccgtatagctcagtctggtagagcgctccatttgggatggagaggtcg
aatgttcgagtcattctatggagacca
1−6tRNA−Gly(tcc)c[60286,60360]

gcatccatcgtatagcggatattatgtctggcttccacccagaagatggg
agttcgattctccctggatgctcca
1−7tRNA−Trp(cca)c[60366,60440]
aggttcttagtataacggctattatgctgggctccaaacccagtgatgag
ggttcgattccttcagggcctgcca
1−8tRNA−Ile(gat)c[60447,60522]
gggagtatagctcatttggtagagctctcgaccgataatcgagcggtgac
tggttcgagtccagttactcccacca
1−9tRNA−Ser(tga)c[60589,60673]
ggagagtagcgctagtggtagcaaaccggacttgaaatccgggccatcgg
aaacggtgagggttcaactcctttactctccgcca
1−10tRNA−His(gtg)c[60770,60845]
gtggccgtagttcagttggtagaactcgagattgtgattctcgtagtcgc
ggattcaaattccgccggtcacccca
1−11tRNA−Gln(ttg)c[60942,61016]
tggactatagacaagcggttaagtcaccggcctttgactccggtatctct
ggttcgaatccagatagtccagcca
1−12tRNA−Met(cat)c[61023,61099]
tgcggggtaacttcagttggtagaatgttgggctcatatcccgacacgcg
caggttcgagtcctgcctccgcctcca
1−13tRNA−Asp(gtc)c[61261,61336]
ggacctatagtttcagcggttaaaatactcgcctgtcacgcgagagtcac
ggattcgaattccgttaggtccgcca
1−14tRNA−Asn(gtt)c[61590,61674]
gggtcgttggctgagagggtaagcgacggactgttaatccgtgtcagaaa
tgactaggcaggttcgatacctgcacggcccgcca
1−15tRNA−Lys(ttt)c[61680,61756]
gggatactagctcagttggttagagcaccggacttttaatccgggtgtcc
gaagttcgagtcttcggtgtcccacca
1−16tRNA−Tyr(gta)c[61763,61849]
ggggagttagaccgtaggggtagcgggacagactgtaaatctgttgctca
aaaggctcgagtggttcgactccattactccccacca
>KT934943.1 Klebsiella phage vB_KpnM_KB57, complete genome
1−1tRNA−Phe(gaa)c[60892,60966]
gcaactttgacatagacggatgtgtgctggcctgaaaagccagatagtca
ggatcgttacctgaaggttgcacca
1−2tRNA−Leu(tag)c[60973,61049]
gggcgattgatggaattggtatacgtgtcggatttagattccgaattttg
ggggttcgagtcccctgtcgcccacca
1−3tRNA−Ala(tgc)c[61085,61160]
ggggttatagtttatttggataaaattacaggcttgcaccttgtagaact
gagttcgattctcagtgactccacca
1−4tRNA−Arg(acg)c[61164,61236]
gtgggtgtcctgacggacaggcaacggtctacgaagccgtttgatgtagg

ttcgagtccttccatccacacca
1−5tRNA−Pro(tgg)c[61243,61319]
ctccgtgtagctcaatagtgtagagcgcctggtttgggaccaggaggttg
agtgttcgagtcactctatggagacca
1−6tRNA−Asp(gtc)c[61464,61539]
ggggatatggtattagcggcaaacatactggcctgtcacgccggagtcac
gggttcgaatcccgttatcctcgcca
1−7tRNA−Asn(gtt)c[61547,61631]
gacgagctggttcattggtagaacggcggactgttaatccgcgagcatta
gctctatgtaggttcaattcctacgctcgtcgcca
1−8tRNA−Lys(ttt)c[61692,61767]
gggtaggtagccgagcggccaggcattcgccttttaagcgaagagacgat
gggttcaaatccctcccttcccacca
1−9tRNA−Ser(tga)c[61848,61940]
ggaagatagggcgtagtggtacgcaaccagatttgaaccctggcccatcg
gggcgatcccccggtgatggttcgactccattatcttcctcca
1−10tRNA−Ser(gct)c[62023,62116]
ggaagattaaccctaatcaggtaagggatctctttgctaaagagacagta
gctccgaaaggggtgtgcgagttcaagtcttgcatcttccgcca
1−11tRNA−Ser(gga)c[62278,62368]
ggtggaatggtcgagaggtttaagacagcaccttggaaaggtgtaggccc
ccaaaaggggtccgtaggttcgaatcctacttccaccgcca
1−12tRNA−His(gtg)c[62454,62529]
gtggccttaattcagttggtagaattcccggttgtgatccgggcagtcat
gggttcaagtcccataggtcacccca
1−13tRNA−Gln(ttg)c[62590,62664]
aggagattagccaagcggttacggcactggtctttgatatcagcatcggt
ggttcgaatccaccatctcctgcca
1−14tRNA−Trp(cca)c[62779,62855]
agggacttagcacaactggctagtgcagcggattccaaatccgcaggttc
tgggttcgaatcccagggtccctgcca
1−16tRNA−Glu(ttc)c[63069,63144]
gctcctatcggctaaatggacaggccgtcagactttcaatctggaaatcc
gagttcgatcctcggtgggagtgcca
1−17tRNA−Ile(gat)c[63297,63371]
gggggtatagttcagcggtagaacgctcgatcgataatcgagaggtctct
ggttcgatcccagatactcccacca
1−18tRNA−Tyr(gta)c[63378,63464]
gcgtcgttagaccgtaggggtagcgggacagactgtaaatctgttgctca
aaaggctcgggtagttcgactctatcacggcgcacca
1−19tRNA−Cys(gca)c[63903,63976]
gcacggttgctggagtggcaacagcttcggctgcaaaccgaatgtcaggt
gttcgattcacctaccgtgctcca
1−20tRNA−Leu(taa)c[64182,64259]

tgcgtccttggtccaatctggcagaggcatgggtgttaagcccccagggt
tcccggttcgagtccgggagggcgcatc
1−21tRNA−Ser(cga)c[64536,64610]
ttctcgatagcttactttggttagagcggtcccacgatagggacggtaca
ggttcgattcctgttcgagaagcca
1−22tRNA−Thr(tgt)c[64617,64692]
gctcgtatagctcagttggtagagcaactcccttgtaaggagaaggtcgt
gggttcaagtcctactgcgagcacca
1−23tRNA−Lys(ctt)c[66919,66994]
ggggtattaacacagcggttagtgtagcggccccttaagccgaaggtcga
aggttcgaatccttcatgccccacca
1−24tRNA−Met(cat)c[67460,67537]
agccctcaagctcattaggtatgagcaatcgactcataatcgagaggtag
gcaggttcgaatcctccgggggccacca
1−25tRNA−Met(cat)c[67540,67612]
cgttcagtagctcagttggtagagcaggttcttcatacggacaaggtcag
tggttcaattccactctgaacga
>KP211958.1 Cyanophage P−TIM40, complete genome
1−1tRNA−Arg(tct)[171831,171905]
tgattcagtagctcagtggatagagcaactgtcttctaaacagtcggtcg
ttggttcgaatccaacctgagtcgt
>KM434186.1 Pseudomonas phage vB_PaeM_PS24, complete genome
1−1tRNA−Asn(gtt)[24047,24123]
tccgttcggtccctcaaggtgagggagcttgactgttaatcaagacgtgc
ctggttcgattccagggagcggagcca
1−2tRNA−Tyr(gta)[24181,24268]
ggagggggtggcagagcggtttaatgcaccggactgtaaatccggcgtccg
accgggcatcgctggttcaaatccagccccctccacca
1−3tRNA−Gln(ttg)[24470,24546]
aggcgtgtggcgaaaaggtttaacgcactggactttgactccagcatttg
tgggttcgaatcccaccacgtctgcca
>KP890823.1 Proteus phage vB_PmiM_Pm5461, complete genome
1−1tRNA−Glu(ttc)c[63149,63223]
acacccgtcgtctagtggtaaggacccctgcctttcgagcaggtaaccgg
agttcgaatctccgtgggtgtgcca
1−2tRNA−Ser(tga)c[63229,63320]
ggagagttgtcagagaggtttaatgagacggtcttgaaaaccgttgggta
ctggaaggtgctccatgggttcgaatcccatactctcttcca
1−3tRNA−Asp(gtc)c[63665,63740]
ggtcttatggtgtacctggttaacatagttccctgtcacggaacagcacc
gagttcgattctcggtaagaccgcca
1−4tRNA−Gly(tcc)c[63940,64014]
gcggccttggtatagcgggtattatttctggcttccacccagaagatagg
agttcaattctcctaggtcgctcca

1-5tRNA-Pro(tgg)c[64408,64484]
ctgtacgtagcgcagtctggtagcgtaggtgctttggatgcatcgggtcg
aaggttcgaatccttccgtacagacca
1-6tRNA-Met(cat)c[64687,64762]
ggccctctagcttactggttgaaagcgaacgcctcataagcgttggagca
tggttcgattccatggtgggtcacca
1-7tRNA-Tyr(gta)c[64768,64852]
ggtatcgtagtctagcggcaatgacaggagactgtaaatctccaggcttc
ggccttcggtagttcgagtctacccggtaccacca
1-8tRNA-Arg(tct)c[64860,64936]
gcacacttagctcagttggatagagcaaggaccttctaagccctaggtca
caggttcgaatcctgtagtgtgtacca
>KT454805.1 Escherichia phage SUSP1, complete genome
1-1tRNA-Pro(tgg)[47552,47628]
ctccgtttagctcagtttggtagagtgcgccgtttggggcggtgaggtcg
aaggttcaatcccttcaacggagacca
1-2tRNA-Glu(ttc)[47636,47713]
gttccagtagacaaattggtaaagtcaccacactttcaatgtggagtttt
gagggttcaagtcccttctggaacgcca
1-3tRNA-Met(cat)[47797,47873]
cgcgggtatagagaaagggcgtctcacatgtctcattagcatggtatcgg
caggttcgactcctgcacccgcctcca
1-4tRNA-Asn(gtt)[47956,48032]
ggtgaggaagcacataaggcatgtgcggtcggctgttaaccgaatggtat
agggttcgaatccctaactcaccgcca
1-5tRNA-Asp(gtc)[48197,48274]
ggttatgtagtttaatctggttaaaatacctcactgtcactgaggaagat
gagggttcaaatcccttcgtaaccgcca
1-6tRNA-Lys(ttt)[48702,48777]
gggggtgtagcagaaaggtgatgcggcagacttttaatctgacaggcgac
gggttcgaatccctccactcccacca
1-7tRNA-Met(cat)[48782,48858]
ggttcagtcgcagataaggtaatgcaagggtttcataagccctatgaatg
tgggttcgaatcccatctgaacctcca
1-8tRNA-Ile(gat)[48860,48935]
gctggtatagttaagaaggttataacactcccctgataagggaacatcgg
tggttcgattccacctaccagtacca
1-9tRNA-Arg(tct)[49213,49289]
gcacccttagttcaattggatagagcaagtgccttctaagcactgagtta
caggttcgagtcctgtagggtgtgcca
1-10tRNA-Ser(tga)[50091,50180]
ggtagatagcggctaatggaagccaaattgtcttgaaaacaattgccatt
gtagtgatacggtgagggttcgactcctttatctaccgcc
1-11tRNA-Leu(tag)[50422,50499]

gggagattgacggtaattggtaaacctaccatccttagaagttggtgttt
gagggttcgaatcccttgtctcctacca
1-12tRNA-Lys(ctt)[50507,50582]
gcaggtgtagcaaaatggttatgcggctgactcttaatcagtaagacgat
gggttcaattccctccacctgtacca
1-13tRNA-Ala(tgc)[50589,50664]
ggggttgtagtttaaatggttaaaacataagttttgcaaacttaagtaca
gggttcgattccctgcttctccacca
1-14tRNA-Gly(tcc)[50671,50745]
gcatccatagtttaaacgggaaaattacagtcttccaaactgaggttgag
ggttcgattccctctggatgctcca
1-15tRNA-Thr(tgt)[50753,50828]
gctcctatcgtataattggctattacggttgccttgtaagcaacttatca
gggttcgaatccttgtgggagcacca
1-16tRNA-Val(tac)[50925,50999]
actcgcttagtttatacggtaaagccccatccttacaagttggtgaagaa
ggttcaagtccttcagcgagtacca
1-17tRNA-Leu(caa)[51002,51079]
gttccagtatcccaattggcagaggatgcaagctcaaaccttgtattagt
gacggttcgaatccgtcttggaacacca
1-18tRNA-Arg(acg)[51258,51334]
gcagaattagttcaaatggatagagcagcagcctacgaagttgcttgtta
ggggttcgaatcccttattctgcacca
1-19tRNA-Gln(ttg)[51920,51995]
aggggattagtttacaaggttaaaacctcggtctttgaaatcgaagaagt
tggttcaattccaacatcccccgcca
1-20tRNA-Leu(taa)[51998,52076]
gctccattactccaattggcagagaggccagacttaaaatctgtgttatg
tatcggttcgaatccgatatggagtacca
1-21tRNA-Gln(ctg)[52084,52159]
agcagtgtagcataactggcaatgctccagcctctgaagctgaaagatta
aggttcaaatccttacgctgctgcca
1-22tRNA-His(gtg)[52191,52267]
gtgtccttatcataaatggataatgacccatgctgtgaacatggtctata
cgggttcgattcccgtaggtcacccca
1-23tRNA-Phe(gaa)[52276,52351]
gggttaagtagcttatatggttaaagcgcgtgtctgaaaaacatgagaag
agggttcaaatcccactaacccgcca
1-24tRNA-Ser(gct)[53106,53198]
ggaagattaaccctaaaaggtaagggaacagtttgctaaactgtcagtaa
ccgagaaatcggcgtaccagttcaagtctggtatcttcctcca
1-25tRNA-Cys(gca)[53206,53281]
gcgtcgatgacagaaatggctatgtgcctgtctgcaaaacaggattatga
gggttcgactccctttcgacgctcca

>KT176190.1 Enterobacteria phage QL01, complete genome

1–1tRNA–Arg(tct)c[68858,68932]
gtcccgctggtgtaatggatagcatacgatccttctaagtttgcggtcct
ggttcgatcccagggcgggatacca

1–2tRNA–Met(cat)c[68938,69014]
ggccctgtagctcaatggttagcagcagtcccctcataagggaaaggtta
ccagttcaaatctggtctgggtcacca

>KR052482.1 Sinorhizobium phage phiN3, complete genome

1–1tRNA–Thr(tgt)c[82832,82915]
gccggtatagccaagcggtcgaaggcacctgatttgtaatcaggtattca
aacgtcggcagttcgaatctgtctaccggctcca

1–2tRNA–Gln(ttg)c[82916,82989]
ttgcccttagttcagttggtagaacgtcgaattttggtttcgaaggtccg
tggttcgaatccacgagggtaagc

1–3tRNA–His(gtg)[87863,87939]
tcatggatggtgttagtcaggtagcacgcggcactgtggatgccgaagca
taggttcaaatcctattccatggacca

1–4tRNA–Asn(gtt)[96665,96738]
tggggtgtagctcagcggtagtagcgacggtctgttaaaccgtaggtcgt
aggttcgatccctacctccccagc

1–5tRNA–Met(cat)[96755,96831]
tgcggggtagtagcagtggttagctcgtctgcctcataagcagaaggtca
tcagttcgaatctgatccccgcaacca

>LN881726.1 Escherichia phage slur02, complete genome

1–1tRNA–Arg(tct)c[139969,140044]
cggggcatagctcagaaggaagagcaaggaccttctaagtcctaggtcgt
aggttcgatccctactgcctcgacca

1–2tRNA–His(gtg)c[140049,140124]
gtggccgtagttcagttggtagaactcgagattgtgattctcgtagtcat
gggttcgactcccatcggtcacccca

1–3tRNA–Asn(gtt)c[140239,140313]
ggatgtgtagctcaatggtagagcgatcgcctgttaagcgattggttata
ggttcgaatcctatcacgtccgcca

1–4tRNA–Tyr(gta)c[140318,140404]
ggggagttatcccgtagaggtagcggtgtggactgtaaattcattgtcat
tgcgactcgggtggttcgactccaccactccccacca

1–5tRNA–Met(cat)c[140417,140491]
ggccctgtagctggaaggttcaagcaagcgactcataatcgccagatggt
ggttcaattccacccagggccacca

1–6tRNA–Thr(tgt)c[140493,140568]
gctgatttagctcagtaggtagagcaactcacttgtaatgaggacgtcgg
cggttcgattccgtcaatcagcacca

1–7tRNA–Ser(tga)c[140575,140664]
ggaggcgtggcagagtggtttaatgcaccggtcttgaaaaccggcagtcg

ctccggcgactcataggttcaaatcctatcgcctccgcca
1−8tRNA−Pro(tgg)c[140666,140740]
ctccgtgtagctcagtttggtagagcgcctgatttgggatcaggaggtcc
aaggttcaaatccttgtatggagac
1−9tRNA−Gly(tcc)c[140751,140824]
gcggatatcgtataatggtattacctcagacttccaatctgatgatgtga
gttcgattctcattatccgctcca
1−10tRNA−Leu(taa)c[140830,140916]
gcgagaatggtcaaattggtaaaggcacagcacttaaaatgctgcggaat
gatttccttgtgggttcgagtcccacttctcgcacca
1−11tRNA−Gln(ttg)c[140917,140990]
tgggaattagccaagttggtaaggcactggattttgattccaggatgcaa
aggttcgagtcctttattcccagc
>KP671755.1 Vibrio phage ValKK3, complete genome
1−1tRNA−Met(cat)[196346,196423]
ggccctatagctcaacacggttagagcaaccgactcataatcggtaggtt
acaggttcgagtcctgttggggtcacca
1−2tRNA−Pro(tgg)[198599,198672]
cgggacgtggcgtaaaggtagcgttcatgctttgggagcatgtggtaaga
gttcgagtctcttcgtcccgacca
1−3tRNA−Pro(tgg)[198682,198758]
ccgtgactagctcaatctggtagagtactccgtttggggcggagaagtta
agcgttcgaatcgcttgtcacggacca
1−4tRNA−Arg(acg)[198767,198842]
gcccgattagctcaattggaagagcagcgtcctacgaaggcgaaggttac
aagttcgaatcttgtattgggtgcca
1−5tRNA−Leu(tag)[199390,199467]
gcgcaagtagcccaatctggcagaggcactggtcttagaaaccagaagtt
aagagttcgaatctcttcttgcgtacca
1−6tRNA−Phe(gaa)[199469,199543]
gcacccttagcttatcaggaaaagcggcggattgaagtcccgcagtgctc
ggttcgattccgagggggtgcacca
1−7tRNA−Lys(ttt)[199632,199706]
gggtcgttagtataacggtagtacatctggcttttaaccagaagggtgag
agttcgaatctcttgcgacccacca
1−8tRNA−Lys(ttt)[199718,199794]
gcgtcggtagctcatcatggaagagcagggagcttttaactctcaggtgt
ctggttcgagtccagagcggcgtacca
1−9tRNA−Glu(ttc)[199858,199932]
tctcccgtggactaacggtaggtcatcaccctttcaaggtgaagtgcgcg
agttcgaatctcgtcgggagaacca
1−10tRNA−Tyr(gta)[199939,200024]
ggagcgtacgtctcaaggtgagacaggggactgtaaatccctgacccttа
acgggtagagaggttcgattcctctacgctccacca

1-11tRNA-Ser(tga)[200224,200309]
gaaagattctgggtagcggcaacggtcttgaaaaccgtcggtcaccggga
ggtgatgttagggttcgaatccctagtcttccgcca
1-12tRNA-Ile(gat)[200373,200446]
agtcccttagctcaacggtagagcgtgcgaccgataatcgcttggtaaga
gttcgattctcttagggactacca
1-13tRNA-Trp(cca)[200453,200526]
aggggcatgatgtaaaggcagcatgacggattccaaacccgttcgttaga
gttcgaatctctatgcccctgcca
1-14tRNA-Asn(gtt)[200601,200676]
ggatcgctaactcaatggttagagtgctcgcctgttaagcgaggagttcc
gagttcgagtctcgggcggtccgcca
1-15tRNA-Asp(gtc)[200746,200821]
ggagccgaggtgtaaaaggttgcatgtctccctgtcacggagaaggtagc
gggttcgaaacccgtcggttccgcca
1-16tRNA-Gln(ttg)[200841,200914]
tggggattggtgtaaaggcagcataacgtactttgaatgcgttggtatca
gttcgaatctgttatccccagcca
1-17tRNA-Thr(tgt)[201044,201118]
gctcctgaagcattgatggcgatgcagcggtcttgtaaaccgcagaattc
ggttcgattccgaacgggagcacca
1-18tRNA-Arg(tct)[201840,201915]
gcgctgttagctcaattggaagagcacgtcccttctaaggatggggttat
gagttcgaatctcatacggcgtgcca
1-19tRNA-Met(cat)[202438,202513]
ggtgacgtagctcaagtggcagagccacgctttcatacggcgtaagatcc
gatttcgagtatcggtgtcacctcca
1-20tRNA-Gly(tcc)[202701,202774]
gcgggtatgatgtaatggtagcatgacgtccttccaagtcgttcgtctcg
gttcgagtccgtgtacccgctcca
1-21tRNA-Val(tac)[202781,202854]
ggtcccttagtataatggcagtacgtctcttttacacagagaaagaagtg
gctcgattccactagggactacca
1-22tRNA-His(gtg)[203270,203346]
gtggcagtgggtgaagtggaaatacccccgggttgtgattccggaagatg
cgggttcgatccccgtctgtcacccca
1-23tRNA-Cys(gca)[203359,203432]
gctcgaatcgtataatggcagtatgacggattgcaaatccgcaggtcagg
gttcgattccctgttcgagctcca
1-24tRNA-Leu(taa)[203641,203717]
gcgcacgtggtccaattggcagaggcatgaggcttaaaatctcagggatg
gcggttcgaatccgcccgtgcgtacca
1-25tRNA-Leu(caa)[203833,203909]
gcccaactagtccaattggcagaggcgctagtttcaaacactagatgatc

cgagttcgaatctcgggttgggcacca
1-26tRNA-Ser(gct)[204078,204166]
ggaagcatggtctaaaggtatgacagcaccctgctaaggtgtcggacgtt
aatagcgttctctgggttcgactcccagtgcttccgcca
>KR014248.1 Escherichia phage vB_EcoM_AYO145A, complete genome
1-1tRNA-Pro(tgg)[26382,26458]
ctctgtttagctcagcttggtagagcgttccgtttggggcggtaaggccg
gaggttcaagtcctccaacagagacca
1-2tRNA-Glu(ttc)[26466,26543]
gttccagtagacaaaatggtaaagtcaccactctttcaaagtggatattt
gagggttcaaatcccttctggaacgcca
1-3tRNA-Met(cat)[26635,26711]
tgcgggtatagagaaagggcgtctcacatgtctcattagcatggtatcgg
caggttcgactcctgcacccgcctcca
1-4tRNA-Asn(gtt)[26789,26865]
ggttaggaagcacataaggtatgtgcggtcgcctgttaagcgaatggcac
agggttcgaatccctgactaaccgcca
1-5tRNA-Tyr(gta)[26875,26962]
gtgtcgttatcccgtagatggtagcggtggggactgtaaatcccttgtca
ttgagactcggtaggttcgactcctacacggcacacca
1-6tRNA-Asp(gtc)[26969,27046]
ggttatgtagtttaatctggttaaaatactcccctgtcacgggagatgat
gagggttcaaatcccttcgtaaccgcca
1-7tRNA-Lys(ttt)[27506,27581]
ggaagtgtagcagaatggtgatgcggcagacttttaatctgacaggcgat
gggttcgaatccctccacttctacca
1-8tRNA-Met(cat)[27586,27662]
ggttcagtcgcagataaggtaatgcaggggtctcataagccctatgaatg
tgggttcgattcccatctgaacctcca
1-9tRNA-Ile(gat)[27664,27739]
gctggtatagttaagaaggttataacactcccctgataagggaacatcgg
tggttcgattccacctaccagtacca
1-10tRNA-Ser(tga)[28308,28397]
ggtaggtagcggctaatggtagccaaactgtcttgaaaacagttgccact
gtagagatacggtgagggttcgactcctttacttaccgcc
1-11tRNA-Leu(tag)[28653,28730]
gggagattgacggtaattggtaaacctatctcgcttagaacgagatgttt
gagggttcgaatcccttgtctcctacca
1-12tRNA-Lys(ctt)[28738,28813]
gcaggtgtagcaaaatggttatgcggctgactcttaatcagtaagacgat
gggttcaattccctccacctgtacca
1-13tRNA-Ala(tgc)[28820,28895]
ggggatgtagtttacatggttaaaacataagttttgcaaacttaagtaca
gggttcaattccctgcttctccacca

1−14tRNA−Gly(tcc)[28902,28976]
gcatccatagtttaaacgggaaaattacagtcttccaaactgaggttgag
ggttcgattccctctggatgctcca
1−15tRNA−Thr(tgt)[28983,29059]
gctgctttcgtataattggctattacacatcccttgtaaggatggaaatg
caggttcgagtcctgtgagcagcacca
1−16tRNA−Val(tac)[29156,29230]
actcgcttagtttatatggtaaaacatcacccttacaagatgaagaaaaa
ggttcaagccctttagtgagtacca
1−17tRNA−Leu(caa)[29232,29309]
gttccagtatcccaattggcagaggatgcaagctcaaaccttgtattagt
gacggttcgaatccgtcttggaacacca
1−18tRNA−Arg(acg)[29315,29390]
gcaggattagttcaaatggaaagagcaacagtctacgaagctgttaatag
gggttcgaatcccttatcctgcgcca
1−19tRNA−Gln(ttg)[29976,30051]
aggggattagtttacaaggttaaaacctcggtctttgaaatcgaagaagt
tggttcaattccaacatcccccgcca
1−20tRNA−Leu(taa)[30054,30132]
gctccattactccaattggcagagaggccagacttaaaatctgtgttatg
tatcggttcgaatccgatatggagtacca
1−21tRNA−Gln(ctg)[30138,30213]
agcggtatagcataactggcaatgcaacagtctctgaagctgtcctatta
aggttcaaatccttatgccgctgcca
1−22tRNA−His(gtg)[30245,30320]
gtggccttatcataaaaggtaatgacccatgctgtgaacatggtctatac
gggttcaaatcccgtaggtcacccca
1−23tRNA−Phe(gaa)[30327,30402]
agtccaagtagcttatatggttaaagcgcgtgtctgaaaaacatgagaag
agggttcaaatcccactggactacca
1−24tRNA−Ser(gct)[31155,31247]
ggaagattaaccctaaaaggtaagggagcagtttgctaaactgccagtag
ctgagaaatcggtgtaccagttcaagtctggtatcttcctcca
1−25tRNA−Cys(gca)[31252,31327]
gaatccgtgacagaaatggctatgtgcctgtctgcaaaacaggtttataa
gggttcgagtcccttcggattctcca
>HQ630627.1 Pseudomonas phage PhiPA3, complete genome
1−1tRNA−Ser(tga)c[294661,294749]
ggaagcgtggccgagtggtttaaggcacctgacttgaaatcagacgggtc
caaagggcccctagagttcgaatctctgcgcttccgcca
1−2tRNA−Thr(tgt)c[294861,294937]
gccgttatagctcagctggtagagcaactgacttgtaatcagtaggtcga
cgggttcgaatccctctgacggcacca
1−3tRNA−Tyr(gta)c[299238,299317]

ttgatgtatagacagatggctactgtcaggggtctgtaacaccccccgcct
aggctagaggttcaattccttccttcaacc
1-4tRNA-Asn(gtt)c[299475,299550]
tccggaatagctcagctggtagagcaacaagctgttaacttgtgggtcac
atgttcgaatcatgtttccggagcca
1-5tRNA-Leu(taa)c[303149,303233]
gcccgaatggcgtaaaggcagccgctgctgacttaaaatcagttgtcctt
gtgacgtgtgggttcgagtcccacttcgggtacca
>LN881732.1 Escherichia phage slur07, complete genome
1-1tRNA-Gln(ttg)[92843,92916]
tgggaattagccaagttggtaaggcactggattttgattccaggatgcaa
aggttcgagtcctttattcccagc
1-2tRNA-Leu(taa)[92917,93003]
gcgagaatggtcaaattggtaaaggcacagcacttaaaatgctgcggaat
gatttccttgtgggttcgagtcccacttctcgcacca
1-3tRNA-Gly(tcc)[93009,93082]
gcggatatcgtataatggtattacctcagacttccaatctgatgatgtga
gttcgattctcattatccgctcca
1-4tRNA-Ser(tga)[93744,93833]
ggaggcgtggcagagtggtttaatgcaccggtcttgaaaaccggcagtcg
ctccggcgactcataggttcaaatcctatcgcctccgcca
1-5tRNA-Thr(tgt)[93840,93915]
gctgatttagctcagtaggtagagcacctcacttgtaatgaggacgtcgg
cggttcgattccgtcaatcagcacca
1-6tRNA-Met(cat)[93917,93991]
ggccctgtagctggaaggttcaagcaagcgactcataatcgccagatggt
ggttcaattccacccagggccacca
1-7tRNA-Tyr(gta)[94004,94090]
ggggagttatcccgtagaggtagcggtgtggactgtaaattcattgtcat
tgcgactcgggtggttcgactccaccactccccacca
1-8tRNA-Asn(gtt)[94095,94169]
ggatgtgtagctcaatggcagagcgatcgcctgttaagcgattggttata
ggttcgaatcctatcacgtccgcca
1-9tRNA-Arg(tct)[94174,94249]
cgaggcatagctcagaaggaagagcaaggaccttctaagtcctgggtcgt
aggttcgatccctactgcctcgacca
>KT454806.1 Escherichia phage SUSP2, complete genome
1-1tRNA-Pro(tgg)[45779,45855]
ctccgtttagctcagtttggtagagtgcgccgtttggggcggtgaggtcg
aaggttcaatcccttcaacggagacca
1-2tRNA-Glu(ttc)[45863,45940]
gttccagtagacaaattggtaaagtcaccacactttcaatgtggagtttt
gagggttcaagtcccttctggaacgcca
1-3tRNA-Met(cat)[46024,46100]

cgcgggtatagagaaagggcgtctcacatgtctcattagcatggtatcgg
caggttcgactcctgcacccgcctcca
1-4tRNA-Asn(gtt)[46183,46259]
ggtgaggaagcacataaggcatgtgcggtcggctgttaaccgaatggtat
agggttcgaatccctaactcaccgcca
1-5tRNA-Asp(gtc)[46424,46501]
ggttatgtagtttaatctggttaaaatactcccctgtcacgggagatgat
gagggttcgaatcccttcgtaaccgcca
1-6tRNA-Lys(ttt)[46930,47005]
gggagtgtagcagaatggtgatgcggcagacttttaatctgacaggcgac
gggttcgaatccctccactcctacca
1-7tRNA-Met(cat)[47012,47088]
ggttcagtcgcagataaggtaatgcaagggtttcataagccctatgaatg
tgggttcgaatcccatctgaacctcca
1-8tRNA-Ile(gat)[47094,47170]
gttgggaaagctcatatggatgtagcactcggctgataaccgagaggcag
agggttcgaatccctctcctaacacca
1-9tRNA-Arg(tct)[47447,47523]
gcacccttagttcaattggatagagcaagtgccttctaagcactgagtta
caggttcgagtcctgtagggtgtgcca
1-10tRNA-Ser(tga)[48056,48144]
ggaaggtggcgagcaacggcgctcaactagtcttgaaaactagggctgct
cagagatgggtaagggttcgaatccttcactttccgcca
1-11tRNA-Leu(tag)[48384,48461]
gggagattgatggtaattggtaaacctatctcgcttagaacgagatgttt
gagggttcgaatcccttgtctcctacca
1-12tRNA-Lys(ctt)[48469,48544]
gcaggtgtagcaaaatggttatgcggctgactcttaatcagtaagacgat
gggttcaattccctccacctgtacca
1-13tRNA-Ala(tgc)[48551,48626]
ggggatgtagtttacatggttaaaacataagttttgcaaacttaagtaca
gggttcaattccctgcttctccacca
1-14tRNA-Gly(tcc)[48633,48707]
gcatccatagtttaaacgggaaaattacagtcttccaaactgaggttgag
ggttcgattccctctggatgctcca
1-15tRNA-Thr(tgt)[48714,48790]
gctgctttcgtataattggctattacacatcccttgtaaggatggaaatg
caggttcgagtcctgtgagcagcacca
1-16tRNA-Val(tac)[48890,48964]
actcgcttagtttatatggtaaagccccatccttacaagttggtgaagaa
ggttcaagtccttcagcgagtacca
1-17tRNA-Leu(caa)[48967,49044]
gttccagtatcccaattggcagaggatgcaagctcaaaccttgtattagt
gacggttcgaatccgtcttggaacacca

1–18tRNA–Arg(acg)[49223,49299]
gcagaattagttcaaatggatagagcagcagcctacgaagttgcttgtta
ggggttcgaatcccttattctgcacca
1–19tRNA–Gln(ttg)[49885,49960]
aggggattagtttacaaggttaaaacctcggtctttgaaatcgaagaagt
tggttcaattccaacatcccccgcca
1–20tRNA–Leu(taa)[49963,50041]
gctccattactccaattggcagagaggccagacttaaaatctgtgttatg
tatcggttcgaatccgatatggagtacca
1–21tRNA–Gln(ctg)[50049,50124]
agcagtgtagcataactggcaatgctccagcctctgaagctgaaagatta
aggttcaaatccttacgctgctgcca
1–22tRNA–His(gtg)[50156,50232]
gtggccttatcataaatggataatgacccatgctgtgaacatggtctata
cgggttcgattcccgtaggtcacccca
1–23tRNA–Phe(gaa)[50241,50316]
gggttaagtagcttatatggttaaagcgcgtgtctgaaaaacatgagaag
agggttcaaatcccactaacccgcca
1–24tRNA–Ser(gct)[51071,51163]
ggaagattaaccctaaaaggtaagggaacagtttgctaaactgtcagtaa
ccgagaaatcggcgtaccagttcaagtctggtatcttcctcca
1–25tRNA–Cys(gca)[51171,51246]
gcgtcgatgacagaaacggctatgtgcctgtctgcaaaacaggattatga
gggttcaactccctttcgacgctcca
>KR869820.1 Citrobacter phage IME–CF2, complete genome
1–1tRNA–Gly(tcc)c[69604,69679]
gcaggtatagttcaattggtagaatatctggcttccatccagaatgttga
gggttcgagtccctttacctgctcca
1–2tRNA–Met(cat)c[69685,69761]
ggccctttagctcaattggtagagcgaacccctcataagggtgtggtttc
cggttcgaatcgcgggaggggccacca
>JX306041.1 Stenotrophomonas phage IME13, complete genome
1–1tRNA–Arg(tct)c[64250,64326]
ggtctcttagctcagttggatagagcagcggccttctaagccgcgggtca
ttggttcaaatccaatagggactgcca
1–2tRNA–Met(cat)c[65607,65682]
ggccctctgcacagtctgtgaatgtgtccagctcataactggggtaaggc
gggttagattcccgcgagggccacca
1–3tRNA–Met(cat)c[65885,65961]
tgcgatgtagaggagaggtcgtcctcgtcgggctcatatcccgaaaatcg
gcagttcgaatctgtccgtcgcttcca
1–4tRNA–Asp(gtc)c[65974,66047]
ggagccatagtttatttggttaaaatagtcccctgtcacgggtcagcacc
gagttcgatcctcggtggctccgc

1-5tRNA-Ser(gct)c[66125,66212]
ggagaagtggatgagcggctgaaatcgcttccctgctaaggaagtaaacc
gaaaggtttcgagagttcgaatctctccttctccgcca
1-6tRNA-Ile(gat)c[66220,66295]
agtggattagctcagtaggtagagcactcgaccgataatcgagagcgcat
tggttcgatcccaatatccactacca
1-7tRNA-Trp(cca)c[66298,66373]
atgacattggtgttagcggtagcatgccggtctccaaaaccgtgcggcca
gggttcgaatccttgatgtcatgcca
1-8tRNA-Thr(tgt)c[66738,66813]
gccggtttagctcatttggtagagcacttcacttgtaatgatggggtggt
ccgttcgaatcggacaaccggcacca
1-9tRNA-Pro(tgg)c[67071,67148]
ccgtgtgtaggctagtcaggttaggtcgtctggtttggggtcagaaagtc
gaaggttcgaatccttccacacggacca
1-10tRNA-Gly(tcc)c[67393,67468]
gcgatattagctcagttggtagagcttctgatttccaatcagaatgtcat
cggttcgagaccgatatatcgctcca
1-11tRNA-His(gtg)c[67481,67555]
gtgaccttagtagtaatggttagctatcctggatgtgacccagggagtac
gggttcaaatcccgtaggtcacccc
1-12tRNA-Lys(ttt)c[67754,67828]
gcgccggtagcttagtggtaaagcaagggacttttaatcccgagagcgaa
ggttcgagtccttctcggcgtacca
1-13tRNA-Tyr(gta)c[67858,67940]
ggaacgttcgggtaatggtatcccaagcggctgtaacccgctcgcctctg
gcattcttggttcgagtccaaggcgttccacca
1-14tRNA-Asn(gtt)c[68537,68613]
ggttgattagctcagttggttagagcggcggactgttaacccgcgggtct
cccgttcgagtcgggaatcaaccgcca
1-15tRNA-Ser(tga)c[68782,68875]
ggaagattggcccgagttggtttaaggcaccggtcttgaaaaccggcgat
cgtaggaatacggtccatccgttcgaatcggatatcttcctcca
1-16tRNA-Leu(taa)c[69068,69152]
gcaagtatggtggaattggtatacactggaggcttaaaacctcccgcctt
cgggattgtgggttcgagtcccactacttgcacca
>KT281790.1 Mycobacterium phage HyRo, complete genome
1-1tRNA-Ser(gct)[30120,30203]
ggagggtgagcatctggtgatgcaggggtcctgctaaggccctacggatt
cacacccgtgagtttcgattactcctccctccgc
1-2tRNA-Leu(cag)[30300,30376]
gccctgctgagcaaactggcaaagctgccgcattcagagtgcgggtcatt
tccgggttcgactcccgggcagggtac
1-3tRNA-Leu(gag)[30496,30570]

gtctctgtaggcaaatcggaaaagccgccatcttgagggggtggtgcgtg
cgggttcgactcccgccagagacac
1-4tRNA-Leu(caa)[30571,30644]
gccgtggtaggccatctggcgagccgccgagttcaagtttcggtgtttgc
gggttcgaatcccgcccacggtac
1-5tRNA-SeC(tca)[67440,67536]
attctggcactggtgggcgagcccaccggcggcagcttcaagctgtcgct
ggccggagaatcgaccggaaacatcccgttcaacgcgacccagggcc
1-6tRNA-Pro(tgg)[90954,91028]
cggggtgtagttcagtttggaagagcgcttggtttgggaccaagttgtcg
caggttcgaatcctgtcaccccgac
1-7tRNA-Trp(cca)[91043,91113]
gggtctgtgcacagggtgcccgacggtctccaaagccgaaggcgggggtt
cgattccctccaggcctgcca
1-8tRNA-Pyl(cta)[92250,92323]
tgcgagatcgtgcacggcgcactaggagcttctaaccctccgactcgcgg
gttcgactcccgcatctcgcaccc
1-9tRNA-Met(cat)[92472,92546]
agcggtgtagagcagctaggtagctcgccgggctcatgacccggaggacg
cgtgttcgattcacgccaccgccac
1-10tRNA-Cys(gca)[92674,92745]
gcgcctttggcggaatggctacgtgctcggctgcaacccgagttatcccg
gttcgactccgggagggcgctc
1-11tRNA-Glu(ctc)[92813,92884]
gctcccatggggtagtggtaaccctcctggttctcagccaggcgtcccga
gttcgatcctcggtgggagtgc
1-12tRNA-His(gtg)[92939,93012]
gtggtagtagttcagatggaagaacgtcgccttgtgacggcgaaggtcgg
gggttcgaagcccctctatcaccc
1-13tRNA-Ala(tgc)[93176,93249]
gggcctatagctcatctggtagagcgcctgccttgcaagcaggaggcggc
aggttcaagtcctgttaggtccac
1-14tRNA-Phe(gaa)[93439,93511]
gccgtcatagctcagttggtagagcactggcctgaaaacccagtggccga
ggttcgattcctcgtgtcggcac
1-15tRNA-Val(cac)[93517,93590]
gtccgtttagctcagctggaagagcgctcggtccacacccgagaggccgc
aggttcgatccctgcaatggacac
1-16tRNA-Lys(ctt)[93712,93785]
gccttcgtagctcagttggtagagctctcgcctcttaagcgagatgtcgc
aggttcgacccctgccggaggcac
1-17tRNA-Glu(ttc)[93790,93866]
ggtcgggtcggtctgctggtatggccagtcggattttcactccggacatt
cgcgggttcaattcccgtcccgatcgc

1-18tRNA-Gly(tcc)[93946,94018]
gcgggtgtggccgaatggctcaggcaccagacttccactctggctaagca
ggttcgattcctgtcatccgctc
1-19tRNA-Thr(cgt)[94079,94153]
gctgctgtagctcacctggcagagcgtcggcgtcgtatcccgaaggcatc
cggttcgagtccggacagcagcccc
1-20tRNA-Thr(tgt)[94154,94226]
gcctctgtggtccagcggcacggacatccgccttgtaagcggaggacccc
cgttcgatccggggtagaggctc
1-21tRNA-Thr(ggt)[94634,94706]
gctgggttagctcagtggtagagcgttcctctggtatgggaaaggcccgg
ggttcaatcccccgactcagctc
1-22tRNA-Gly(gcc)[96029,96102]
gcgaaggtagctcagctggcagagcgccaccttgccaaggtggaggtcgc
gggatcgtaacccgttcttcgctc
1-23tRNA-Asp(gtc)[96106,96178]
ggccctgtagctcagaggaagagcacccgcctgtcgagcgggaggtcgcg
gtatcgtaatccgtcagggtcgc
1-24tRNA-Met(cat)[96238,96310]
gcctcactagctcattggtagagccgctcgctcataacgtgcaggtacct
ggttcgattccagggtgaggtac
1-25tRNA-Ile(gat)[96316,96390]
gcctgttagcggactggtcgtccgatccaagctgataacttggcgtaagc
ggtgttcgattcaccgagcaggtac
1-26tRNA-Arg(acg)[96484,96556]
gcctctatggtccaacggagatgacgccggtctacggaaccggagatgcg
tgttcgattcgcgctaggggcac
1-27tRNA-Val(gac)[96599,96671]
gtccgtgtagctcaggggtagagcgcctgctcgacacgcaggaggaccga
ggttcgaaacctcgcatggacac
1-28tRNA-Arg(ccg)[96766,96835]
gtcctcgctgacggactctgccctggccctccggaggccgggtgggtggt
tcgactccacccggggacac
1-29tRNA-Arg(cct)[96843,96916]
gcctctgtagctcaacggacagagcaacgcggtcctaacgcggtggctgg
aggttcgaatcctctcggaggcac
1-31tRNA-Gln(ttg)[97437,97512]
tggggtatggtggcaatctggcagtcccgccggactttgactccggaggt
gcaggttcgagtcctgctaccccatc
1-32tRNA-Arg(tct)[97516,97591]
gcccttgtagctcagtggacagagcggcgagcttctacctcgcgggccgg
gagttcgaatctctccaggggcacca
1-33tRNA-Gln(ctg)[125143,125217]
tgctcgttggtgtaactggcaacactacggactctggctccgtcattctt

ggttcgaatccagggcgagcaacca
1-34tRNA-Asn(gtt)[125224,125299]
tggggtgtccgttaatcaggcaaacgagcggactgttaatccgtccctgc
aggttcgaatcctgccaccccagcca
>KF835987.1 Pectobacterium bacteriophage PM2, complete genome
1-1tRNA-Gly(tcc)c[71819,71894]
gcatccatcgtataatggcttattatgactggcttccacccagtagatga
ggtttcgattacctctggatgctcca
1-2tRNA-Trp(cca)c[73340,73413]
aggttcttagtataatggtattaccgtgagctccaacctcattgatgtgg
gttcgattcctacagggcctgcca
1-3tRNA-Ser(tga)c[73735,73826]
ggaagattggtagagaggctaattacacctgacttgaaatcaggaggccg
aagaaattcggtccgagggttcgaatccttcatcttccgcca
1-4tRNA-His(gtg)c[73831,73906]
gtggccgtagttcagttggtagaacctccgattgtgattcggaatgtcac
ggattcaaattccgtcggtcacccca
1-5tRNA-Gln(ttg)c[74004,74079]
tggggattatccaagttggtaaggcctaggactttgaatcctatatgcag
aggttcgagacctctatccccagcca
1-6tRNA-Met(cat)c[74090,74166]
tgcgggttaacttcagttggtagaatgttaggttcatatcctaatatgcg
tcggttcgagtccggcacccgcctcca
1-7tRNA-Asn(gtt)c[74762,74846]
gggtcgttggctgagagggtaagcgacggactgttaatccgtgtcagaaa
tgactaggcaggttcgatacctgcacggcccgcca
1-8tRNA-Ile(gat)c[74851,74925]
tgcttcatagctcagtggtagagcagtcgcccgataagcgattggtcgct
ggttcgaatccggctgaagcaacca
1-9tRNA-Pro(tgg)c[74942,75019]
ccgtgtgtagcgcagcttggtcagcgtaggagctttggatgcttcgggtc
gttggttcgaatccaaccacacggacca
1-10tRNA-Tyr(gta)c[75027,75107]
gtgggattagccaagtggtcgacggcgtctgactgtaaatcagatattta
cacggtggttcgaatccaccatcccacacca
1-11tRNA-Arg(tct)c[75397,75471]
gccctcatagttaaatggatataacagggaccttctaagtcctagttcca
agttcgattcttggtgagggtacca
1-12tRNA-Met(cat)c[75475,75549]
ggccctttagctcagaggtagagcaggcaactcataattgctcggtcgct
ggttcgaatccggcaagggtcacca
1-13tRNA-Leu(taa)c[75560,75647]
ccggatgtggtggtattggcagtacacacgggatttaaaatcccacagag
gtttcctcttacgagttcgaatctcgtcatccggacca

>KC954775.1 UNVERIFIED: Cronobacter phage S13, complete genome

1−1tRNA−Leu(taa)[118148,118224]
gcgacggtagcccaattggcagaggcacatcacttaaaatgatgaaagtt
cgggttcgaatcccgagcgtcgtacca
1−2tRNA−Leu(caa)[118229,118305]
gctcgcgtgacgcaattggcagacgtgtccctctcaaaaggggaatgttg
agagttcgagtctctccgcgagcacca
1−3tRNA−Met(cat)[118726,118801]
ggtcccttagcacaaaggctagtgcgcccaactcataattggaacgatgt
gggttcgaatcccacggggaccacca
1−4tRNA−Lys(ttt)[120360,120436]
cggtcattagctcaacggttgagagcaatcgacttttaatcgataggttt
agggttcgaatccctaatgaccgacca
1−5tRNA−Glu(ttc)[120551,120626]
atcccattcgtactaatggtcaggtcgtcaccctttcaaggtgaaggtac
gagttcgaatctcgtatgggatacca
1−6tRNA−Tyr(gta)[120635,120719]
gcgtcgttggcagaatggctattgcagcggactgtaaattcgccctcttc
ggagattcctagttcgagtctaggacggcgcacca
1−7tRNA−Asp(gtc)[120840,120916]
ggtcgtgtagtttagatggtgaaaatacccgcctgtcacgtgggagatcg
cgagttcgatcctcgtcacgaccgcca
1−8tRNA−Pro(tgg)[121000,121077]
ccgtgcgtagctcagtctggtcagagtgcaccgtttggggcggtgatgtc
gagagttcaaatccctccgcacggacca
1−9tRNA−Asn(gtt)[121315,121389]
ggaccattagctccaacggttagagcggggacctgttaagtccacggctg
ggagttcaaatctctcatggtccgc
1−10tRNA−Leu(tag)[121651,121728]
gggtatgtggcgcaatctggcagacgcgtcggtcttagaaaccgaatgtt
gagagttcgagtctctccatacccacca
1−11tRNA−Arg(acg)[122018,122093]
gcagatatagcttaatggataaagccccagcttacgaatctggtgaattt
gagttcgagtctcaatatctgctcca
1−12tRNA−Gly(tcc)[122095,122170]
gcggtagtaactcagttggtagagtgccgtccttccaagtcggatgtcgc
gagttcgaatctcgtctgccgctcca
1−13tRNA−Phe(gaa)[122642,122714]
gggtccttagcttaaatgggaaagcgggtgattgaagatctccagttcac
ggttcgattccgtggggacccac
1−14tRNA−Ala(tgc)[122727,122802]
gggcgtgtagctcaattgggagagcaactgctttgcaagcagaaggttga
gggttcgagtcccttcttgtccacca
1−15tRNA−Gln(ttg)[122809,122883]

tgggatatcgtataatggttagtacatcggcctttgactccgatagcgga
agttcgactcttcctatcccagcca
1−16tRNA−Trp(cca)[122991,123066]
aggagcatagtttaacaggcgaaaatttcggtctccaaaaccgacgttct
gagttcgaatctcagtgctcctgcca
1−17tRNA−Cys(gca)[123077,123152]
gcctgggtacccgagaggttaagggcgcggattgcaaatccgttgtgcgt
cagttcgaatctgactccaggctcca
1−18tRNA−Ile(gat)[123154,123230]
agtcccgtagctcagttggccagagcaaacgaccgataatcgttgtgtca
cgcgttcgaatcgcgtcgggactacca
1−19tRNA−Arg(tct)[123238,123314]
gcttgcgtagctcaaccggatagagcatcggtcttctaaaccgagggtta
tgtgttcgagtcacatcgtgagtacca
1−20tRNA−Met(cat)[127251,127324]
tgcgcggtagtgaaatggtatcatttggggttcataaccctaagttggaa
gttcgaatcttccctgcgcatcca
1−21tRNA−Met(cat)[127327,127402]
ggtgaattagctcaactggcagagcacgtccttcatacggatgaggttat
gagttcgaatctcatattcacctcca
1−22tRNA−His(gtg)[127435,127511]
gtggccaaagcctttatctggatgaggttcgacctgtgacgtcgaggaag
agggttcgattccctctggtcacccca
1−23tRNA−Ser(tga)[127516,127609]
ggagagttggcagagtttggtttattgcacctgacttgaaatcagacgat
cccctaaaaagggtccgtgggttcgaatcccacactctcctcca
1−24tRNA−Thr(tgt)[127757,127832]
gctcctatcgtataactggctattacggctgacttgtaatcagcttatca
gggttcgagtccttgtgggagcacca
1−25tRNA−Ser(gct)[135021,135109]
ggtgagttgtccgaatggtaaggaagcggattgctaatccgtagatcgtc
tctgacggtctgagagttcgagtctctcactcaccgcca

>KP007360.1 Enterobacteria phage vB_EcoM_VR20, complete genome

1−1tRNA−Arg(tct)c[69911,69987]
cggggtgtagctcaattgtatagagcaacggtcttctaaaccgtaggttg
tgggttagaatcccaccaccccgacca
1−2tRNA−Met(cat)c[69989,70063]
ggccctgtagctcaattggttagagcatccggctcataactggcaggttt
ccggttcaagtccggatggggccac

>KT736033.1 Pseudomonas phage K8, complete genome

1−1tRNA−Gln(ttg)[22297,22370]
tgccgcttcgttcaatggtaggacgccagactttgaatctggagatgatg
gttcgatcccatcagtggctgcca
1−2tRNA−Arg(tct)[22576,22650]

gctcgtatagtgtaacggatgcacaacggtcttctaagccgtaaggtcta
ggttcgaatcctagtatgagcgcca
1−3tRNA−Lys(ttt)[22658,22734]
tggacggaagctaaagtggataggcatctggcttttaaccagatttatag
tgagttcgagtctcacccgttcaacca
1−4tRNA−Leu(tag)[23009,23093]
ggccctgtggtggaattggtatacacatcagtcttagaaactgacgccga
gaggattgagggttcaagtccctccggggccacca
1−5tRNA−Ile(gat)[23304,23379]
ggccagatagctcaattggtagagcacccgaccgataatcgggtggttga
aggttcaagtccttctctggccacca
1−6tRNA−Asp(gtc)[23389,23467]
ggcccattagctcagtctggactagagcaagcccctgtctagggcaaggt
cgccggttcgaatccggcatgggtcgcca
1−7tRNA−Cys(gca)[23724,23799]
ccctcgttggccgagaggatcaggcagcagattgcaaatctgccatacat
cggttcaaatccgatacgaggctcca
1−8tRNA−Asn(gtt)[23809,23885]
tccgtacggcccatcaaggcgatggggctcggctgttaaccgaaacgcgc
taggttcgattcctaggtacggagcca
1−9tRNA−Pro(tgg)[23948,24025]
ctcctcgtagctcagtctggttagagtgtcggatttggaatccgaaggtc
gaaggttcaaatccttccggggtgacca
1−10tRNA−Gly(tcc)[24033,24109]
gcgggtttcgtatagtctggttattattcttggcttccacccaagagact
agggttcgaatccctaagcccgcacca
1−11tRNA−Phe(gaa)[24172,24247]
gcgtctgaagctaactaggtagaagcgctgggttgaaattccagaggact
tggatcgttaccaagcagatgcacca
1−12tRNA−Glu(ttc)[24254,24329]
gcagttatagattaattggttaaatcgccagactttcaatctggtgttcc
gggttcgatccccggtaactgctcca
1−13tRNA−His(gtg)[24390,24464]
gtggagattgtgtagcccggatgctgcctttcggttgtgacccgattgtt
ggggttcgagtccccgcctccaacc
1−14tRNA−Thr(tgt)[24651,24725]
gccctttaagcatttatggtgatgcaccggctttgtaacccggcgaattc
tgttcaagtcaggaatggggcacca
>KT224359.1 Bacillus phage TsarBomba, complete genome
1−1tRNA−Cys(gca)[26558,26631]
gaaggtgtaccgaagtggcttaacgggctaggttgcaaccctagtgctcg
tgggttcgaatcccaccatcttct
1−2tRNA−Asn(gtt)[26645,26719]
gctcctgtagcgtagcggtagcgcagcagcctgttaagctgttggtcggt

ggttcgaatccacccaggagcgcca
1–3tRNA–Ser(gct)[26724,26814]
ggagagttgtcagagtggcttatcgtgcctgtttgctaaataggtgtacg
tccttaacgtaccacaggttcaaatcctgtactctccgcca
1–4tRNA–Gly(tcc)[27005,27080]
ggggcattagtatatcggttcattattcctggcttccaaccaggggaggt
cggttcgattccgacatgtccctcca
1–5tRNA–Thr(tgt)[27085,27160]
gccttcttagctcagttggtagagcgattgacttgtaatcaataggtcgt
gggttcgaatcctacagtcggcacca
1–6tRNA–Asp(gtc)[27166,27238]
tggggtattagtttagtggtaaaatactgcactgtctatgcagagtcagg
ggttcaactcccctatacctcgt
1–7tRNA–Glu(ttc)[27367,27440]
ggggtattggtgaagtggtcaaacacatccgactttctatcggagatacg
agggttcgaatcccttataccctt
1–8tRNA–Ile(tat)[27444,27518]
gccccttagccaagcggttcaaggcagtgggattatgtcctgcgaatcg
gaagttcgaatcttccaaggggagc
1–9tRNA–Arg(tct)[27618,27693]
tatccctttagccaagtggattaaggcatcgggcttctatcccgtagatc
gtgggttcgattcctacagggggtgt
1–10tRNA–Ser(tga)[27782,27873]
ggaagggtactcaagtggataaaagagggcggtcttgaaaaccgctaggc
gtgtaaaagcgtgcgtgggttcgaatcccactccttccgcca
1–11tRNA–Gln(ttg)[27951,28023]
tttcggagtagccaagtggtaaggcaatagactttgactctatgatcggt
ggttcgagaccatcctccgaagt
1–12tRNA–His(gtg)[28106,28178]
cggggtgtggcgtaacggttaacgcaagtgactgtggatcactgaatagg
ggttcgattcccctcatcctgac
1–13tRNA–Pro(tgg)[28186,28261]
gtgggtgtagctcagattggctagagcgcttgctttgggagcaagaagtc
gcaggttcaagtcctgtcacccgccc
1–14tRNA–Phe(gaa)[28369,28444]
ggacggatagctcagttggtagagcagagggctgaaaatcctcgtgtcgt
aggttcgatccctactccgtccacca
1–15tRNA–Tyr(gta)[28586,28672]
gggcgagtatgcaaattggtgaagcaagcggtctgtaaaaccgtgacgta
agatacattgcaggttcgaatcctgcctttcccacca
1–16tRNA–Ile(gat)[28674,28750]
gctagggtagctcagtcaggtagagcagtgtcttgataaggcattggtcg
taggttcgaatcctacccctagtacca
1–17tRNA–Leu(taa)[28761,28848]

ggcggagtgttggaattggtagacatatggcacttaaaatgctatgtccg
tacgggcgtggtgggttcgagtcccccctccgctacca
1-18tRNA-Leu(tag)[28854,28929]
tgccgaagtaatccaatggcagagatagcggttttagaaaccgtacagtg
tgggttcgagtcccaccttcggtatc
1-19tRNA-Met(cat)[29079,29151]
agggttatagctcagtggtagtagcgtgggtctcataagcccaaggtcgt
aggttcaactcctactagcccta
>KP339049.1 Enterococcus phage EFDG1, complete genome
1-1tRNA-Pro(tgg)c[44887,44959]
cgggaaatagcgcagttggtagcgcacacgttttgggagcgtggggtcgt
aggttcgagtcctgctttcccga
1-2tRNA-Ala(tgc)c[45279,45351]
ggggcattagctcagttgggagagcgctacccttgcaaggtagatgtcag
cggttcaagtccgctatgctcca
1-3tRNA-Arg(tct)c[45359,45433]
tatctccgtaggctaattggataaaccacaggatttctaatcccgcattc
tgagttcgaatctcagcggaggtgt
1-4tRNA-Ser(tga)c[45835,45923]
tcggggagtatcataactggtattcagcgtgatttgaaatcatgtagacg
tgtaaaagcgtttgggggttcgagtccctcctcctcggt
1-5tRNA-Ser(gga)c[46019,46105]
ggaaggttactcaaacggtaaagaggcttggttggaaaccaagtagatgt
gtaacagcatgtgtgggttcgaatcccatgccttcca
1-7tRNA-Leu(tag)c[47280,47355]
tgcgagagtatcccaactggcagaggaagtagacttagaatctattcagt
gagggttcgaatccctcctcttgtat
1-8tRNA-Leu(taa)c[47997,48082]
tgcgggtatggcggaattggtatacgctgtggacttaaaatccattgtcc
tttgtgacatgcaggttcgactcctgttacctgtat
1-9tRNA-His(gtg)c[48259,48329]
gtgactgtagcctaatggtaaggcagtgttctgtggaaacatgaatctca
gttcgattctgagcagtcaca
1-10tRNA-Ile(gat)c[49038,49111]
accagtgtagctcagttggttagagcatcgtcttgataaggcgggtgtca
gaggttcaaatcctctcactggta
1-11tRNA-Val(tac)c[49265,49336]
accgtcttagttcagtgggagaacgccacccttacaaggtggatgtcaca
ggttcaaatcctgtagacggta
1-12tRNA-Arg(acg)c[49569,49641]
cgacagttcgttcaattggataggatgctagactacgaatctagtgatag
aggttcgattcctttactgtcgg
1-13tRNA-Lys(ttt)c[49742,49818]
tgtcttcatagctcaatcggctagagcacccgacttttaatcggggggtt

cggggttcaaatccctgtggggacatc
1-14tRNA-Lys(ctt)c[49951,50026]
tatctccttagctcagttggtggtagcacctgactcttaatcagggggtc
acaggttcgaatcctgtaggggatat
1-15tRNA-Thr(tgt)c[50218,50289]
gctgatttagtataacggctattactccggctttgtaacccgggaatggg
agttcgattctctcaatcagca
1-16tRNA-Phe(gaa)c[51446,51519]
ggagaggtagctcagtcggttagagcactagcctgaaaaactaggggtcg
gtggttcaattccacccctctcca
1-17tRNA-Cys(gca)c[51641,51713]
gcgggagtagccaagtgggttaaggctagggattgcaaacccctgaacgt
gagttcgaatctcaccttctgct
1-18tRNA-Trp(cca)c[51953,52026]
tgcgctgttggtgtagtggctatcatttccggctccaacccggaagacgg
gggttcgaatccttcacggcgtgt
1-19tRNA-Met(cat)c[52237,52309]
ggactattagctcagtaggtcagagccgtcagctcataactgacaggttt
gggttcgattcccaagtagtcca
1-20tRNA-Glu(ttc)c[52516,52589]
tggcgcattggtcaagcggtcaagacaccggtctttcgaaccggaatcga
gagttcgactctctcatgcgctat
1-21tRNA-Asp(gtc)c[52593,52669]
tgggctattggagcagtcaggagtgctcgtctccctgtcacggagaaggt
catgggttcaagtcccatatagctcgt
1-22tRNA-Ser(gct)c[53569,53660]
tggaaggttactcaaatggttaagagggctgattgctaatcagttaggtc
ggagaaatccggtgtaagggttcgaccccccttaccttccatc
1-23tRNA-Gln(ttg)c[53768,53841]
ttgtcaggtcgtctaacaggtaggacacgaccctttgaaggttgcgatac
aggttcgaaccctgtcctgacagt
1-24tRNA-Asn(gtt)c[53932,54004]
ttcacctatgatgaaacggtatcatgactggctgttaaccagttcttgta
ggttcaagtcctgctaggtgagt
>KM092515.1 Escherichia phage HY02, complete genome
1-1tRNA-Pro(tgg)[25341,25417]
ctctgtttagctcagcttggtagagcgttccgtttggggcggtaaggccg
gaggttcaagtcctccaacagagacca
1-2tRNA-Glu(ttc)[25425,25502]
gttccagtagacaaaatggtatagtcaccactctttcaaagtggatattt
gagggttcaaatccattctggaacgcca
1-3tRNA-Met(cat)[25594,25670]
tgcgggtatagagaaagggcgtctcacatgtctcattagcatggtatcgg
caggttcgactcctgcacccgcctcca

1-4tRNA-Asn(gtt)[25748,25822]
ggttaggaagcacatgaggtatgtgcggtcgcctgttaagcgaatggcac
agggttcgaatccctgactaaccgc
1-5tRNA-Tyr(gta)[25833,25920]
gtgtcgttatcccgtagatggtagcggtggggactgtaaatcccttgtca
ttgagactcggtaggttcgactcctacacggcacacca
1-6tRNA-Lys(ttt)[26348,26423]
ggaagtgtagcagaatggtgatgcggcagacttttaatctgacaggcgat
gggttcgaatccctccacttctacca
1-7tRNA-Met(cat)[26428,26504]
ggttcagtcgcagataaggtaatgcaagggtctcataagccctatgaatg
tgggttcgattcccatctgaacctcca
1-8tRNA-Ile(gat)[26506,26581]
gctggtatagttaagaaggttataacactcccctgataagggaacatcgg
tggttcgattccacctaccagtacca
1-9tRNA-Arg(tct)[26829,26905]
gcacccttagttcaattggatagagcaacggtcttctaaatcgttagtta
caggttcgaatcctgtagggtgtgcca
1-10tRNA-Ser(tga)[27144,27233]
ggtaggtagcggctaatggtagccaaactgtcttgaaaacagttgccact
gtagagatacggtgagggttcgactcctttacttaccgcc
1-11tRNA-Leu(tag)[27490,27567]
gggagattgacggtaattggtaaacctaccatccttagaagttggtgttt
gagggttcgaatcccttgtctcccacca
1-12tRNA-Lys(ctt)[27575,27650]
gcaggtgtagcaaaatggttatgcggctgactcttaatcagtaagacgat
gggttcaattccctccacctgtacca
1-13tRNA-Ala(tgc)[27657,27732]
ggggtcatagtttatatggttaaaattcgagttttgcaaacttgggaact
gagttcaattctcagtgactccacca
1-14tRNA-Gly(tcc)[27739,27813]
gcatccatagtttaaacgggaaaattacagtcttccaaactgaggttgag
ggttcgattccctctggatgctcca
1-15tRNA-Thr(tgt)[27821,27896]
gctcctatcgtataattggctattacggttgccttgtaagcaacttatca
gggttcgaatccttgtgggagcacca
1-16tRNA-Val(tac)[27994,28068]
actcgcttagtttatatggtaaaacatcacccttacaagatgaagaaaaa
ggttcaagtcctttagtgagtacca
1-17tRNA-Leu(caa)[28070,28147]
gttccagtatcccaattggcagaggatgcaagctcaaaccttgtactagt
gacggttcgaatccgtcttggaacacca
1-18tRNA-Arg(acg)[28153,28228]
gcaggattagttcaaatggatagagcaacagtctacgaagctgttaatag

gggttcgaatcccttatcctgcgcca
1–19tRNA–Gln(ttg)[28814,28889]
aggggattagtttacaaggttaaaacctcggtctttgaaatcgaagaagt
tggttcaattccaacatcccccgcca
1–20tRNA–Leu(taa)[28892,28970]
gctccattactccaattggcagagaggccagacttaaaatctgtgttatg
tatcggttcgaatccgatatggagtacca
1–21tRNA–Gln(ctg)[28976,29051]
agcggtatagcataactggcaatgcagcagtctctgaagctgtcttatta
aggttcaaatccttatgccgctgcca
1–22tRNA–His(gtg)[29083,29158]
gtggccttatcataaatggtaatgacccatgctgtgaacatggtctatac
gggttcaaatcccgtaggtcacccca
1–23tRNA–Phe(gaa)[29165,29240]
agtccaagtagcttatatggttaaagcgcgtgtctgaaaaacatgagaag
agggttcaaatcccactggactacca
1–24tRNA–Ser(gct)[29993,30085]
ggaagattaaccctaaaaggtaagggagcagtttgctaaactgccagtag
ctgagaaatcggtgtaccagttcaagtctggtatcttcctcca
1–25tRNA–Cys(gca)[30090,30165]
gaatccgtgacagaaatggctatgtgcctgtctgcaaaacaggtttataa
gggttcgagtcccttcggattctcca

>KF055347.1 Escherichia phage JH2, complete genome

1–1tRNA–Cys(gca)c[69613,69688]
gaatccgtgacagaaatggatatgtgcctgtctgcaaaacaggtttataa
gggttcgagtcccttcggattctcca
1–2tRNA–Ser(gct)c[69693,69785]
ggaagattaaccctaaaaggtaagggagcagtttgctaaactgccagtag
ctgagaaatcggtgtaccagttcaagtctggtatcttcctcca
1–3tRNA–Phe(gaa)c[70538,70613]
agtccaagtagcttatatggttaaagcgcgtgtctgaaaaacatgagaag
agggttcaaatcccactggactacca
1–4tRNA–His(gtg)c[70620,70695]
gtggccttatcataaaaggtaatgacccatgctgtgaacatggtctatac
gggttcaaatcccgtaggtcacccca
1–5tRNA–Gln(ctg)c[70727,70802]
agcggtatagcataactggcaatgcagcagtctctgaagctgtcttatta
aggttcaaatccttatgccgctgcca
1–6tRNA–Leu(taa)c[70808,70886]
gctccattactccaattggcagagaggccagacttaaaatctgtgttatg
tatcggttcgaatccgatatggagtacca
1–7tRNA–Gln(ttg)c[70889,70964]
aggggattagtttacaaggttaaaacctcggtctttgaaatcgaagaagt
tggttcaattccaacatcccccgcca

1-8tRNA-Arg(acg)c[71550,71625]
gcaggattagttcaaatggatagagcaacagtctacgaagctgttaatag
gggttcgaatcccttatcctgcacca
1-9tRNA-Leu(caa)c[71631,71708]
gttccagtatcccaattggcagaggatgcaagctcaaaccttgtattagt
gacggttcgaatccgtcttggaacacca
1-10tRNA-Val(tac)c[71710,71784]
actcgcttagtttatatggtaaaacatcacccttacaagatgaagaaaaa
ggttcaagtcctttagtgagtacca
1-11tRNA-Thr(tgt)c[71880,71955]
gctcctatcgtataattggctattacggttgccttgtaagcaacttatca
gggttcgaatccttgtgggagcacca
1-12tRNA-Gly(tcc)c[71963,72037]
gcatccatagtttaaacgggaaaattacagtcttccaaactgaggttgag
ggttcgattccctctggatgctcca
1-13tRNA-Ala(tgc)c[72044,72119]
ggggtcatagtttatatggttaaaattcgagttttgcaaactcgggaact
gagttcaattctcagtgactccacca
1-14tRNA-Lys(ctt)c[72126,72201]
gcaggtgtagcaaaatggttatgcggctgactcttaatcagtaagacgat
gggttcaattccctccacctgcacca
1-15tRNA-Leu(tag)c[72209,72286]
gggagattgacggtaattggtaaacctaccatccttagaagttggtgttt
gagggttcgaatcccttgtctcctacca
1-16tRNA-Ser(tga)c[72542,72631]
ggtaggtagcggctaatggcagccaaacagtcttgaaaactgttgccact
gtagagatacggtgagggttcgactcctttacttaccgcc
1-17tRNA-Arg(tct)c[72870,72946]
gcacccttagttcaactggacagagcaaatgacttctaatcattgagtta
caggttcgaatcctgtagggtgtgcca
1-18tRNA-Ile(gat)c[73283,73357]
tgtgggttagcataaatggtaatgcaaacggctgataaccgttagaagag
ggttcgataccctcacctacaacca
1-19tRNA-Met(cat)c[73360,73436]
ggttcagtcgcagataaggtaatgcaagggtttcataagccctatgaatg
tgggttcgattcccatctgaacctcca
1-20tRNA-Lys(ttt)c[73441,73516]
ggaagtgtagcagaatggtgatgcggcagacttttaatctgacaggcgat
gggttcgaatccctccacttctacca
1-21tRNA-Asp(gtc)c[73974,74051]
ggttatgtagtttaatctggttaaaatactcccctgtcacgggagatgat
gagggttcaaatcccttcgtaaccgcca
1-22tRNA-Tyr(gta)c[74058,74145]
gtgtcgttatcccgtagatggtagcggtggggactgtaaatcccttgtca

ttgagactcggtaggttcgactcctacacggcacacca
1-23tRNA-Asn(gtt)c[74216,74292]
ggttaggaagcacataaggtatgtgcggtcgcctgttaagcgaatggcac
agggttcgaatccctgactaaccgcca
1-24tRNA-Met(cat)c[74370,74446]
tgcgggtatagagaaagggcgtctcacatgtctcattagcatggtatcgg
caggttcgactcctgcacccgcctcca
1-25tRNA-Glu(ttc)c[74538,74615]
gttccagtagacaaaatggtaaagtcaccactctttcaaagtggatattt
gagggttcaaatcccttctggaacgcca
1-26tRNA-Pro(tgg)c[74623,74699]
ctctgtttagctcagcttggtagagcgttccgtttggggcggtaaggccg
gaggttcaagtcctccaacagagacca
>KC595512.2 Bacillus phage JL, complete genome
1-1tRNA-Met(cat)[32882,32956]
ggttccttagctcaattggttagagctaccggctcataaccggtgggttg
taggttcgattcctacaggaatcac
1-3tRNA-Trp(cca)[33994,34069]
tgggggtatagttttaactggataaaaccgtggtctccaacaccactatt
ctaggttcgaatcctagtgccctcgt
>KP007361.1 Enterobacteria phage vB_EcoM_VR25, complete genome
1-1tRNA-Arg(tct)c[68958,69033]
cgaggcatagctcagaaggaagagcaaggaccttctaagtcctaggtcgt
aggttcgatccctactgtctcgacca
1-2tRNA-Met(cat)c[69168,69241]
ggccctgtagctggacggtcaagcgagcgactcataatcgctggatggtg
gttcgattccacccagggccacca
>KP027446.1 Staphylococcus phage phiIPLA-RODI, complete genome
1-1tRNA-Met(cat)c[8503,8574]
ggactcttagcttaaaggtaaagccaaccgctcataacggtttgactgta
ggttcgaatcctgcagagtcca
1-2tRNA-Trp(cca)c[30818,30889]
acacccttagtataattagtagtacaagggtctccaaaacccttagtctt
tgtgcaaatcaaagagggtgtg
1-3tRNA-Phe(gaa)c[30896,30968]
ggtttcttagctcagatggtagagcactagattgaagctctaggtgtcat
tggttcaaatccaatagaaacca
1-4tRNA-Asp(gtc)c[30974,31049]
tggctcattggtgtaactggttaacacactgccctgtcacggcagagagt
acgagttcgagtctcgtatgagtcgt
>KP202158.1 Yersinia phage vB_YenM_TG1, complete genome
1-1tRNA-Gly(tcc)c[64617,64692]
gcagagttcgtatagtggcttaatactattggtttccgcccagtaaacat
cggttcgaatccgatactctgctcca

1–2tRNA–Trp(cca)c[64945,65019]
aggtctttagtatagtggcgattatgctaggctccaaacctagtgacgag
tgttcgattcactcaaggcctgcca
1–3tRNA–Arg(tct)c[66301,66377]
gtcctcatagctcaacaggacagagcaacggtcttctaaaccgtaggttg
ctggttcgattccagctggggacacca
1–4tRNA–Met(cat)c[67178,67254]
ggcccttagctcagatggatagagcagtcgactcataatcgattggtca
ctggttcaaatccagtaagggccacca
>KF356199.1 Microcystis phage MaMV–DC, complete genome
1–1tRNA–Met(cat)c[38445,38517]
gcaggattggccgataggtggaggcaccgtcctcataagacggcttagag
cggttcgattccgctatcctgca
1–2tRNA–Tyr(gta)c[152269,152351]
gggttagtggccgagtagtcgaaggcaacagactgtaaatctgtagagta
attccacgctggtgcaaatccagcctaacccac
>KP007359.1 Enterobacteria phage vB_EcoM_VR5, complete genome
1–1tRNA–Arg(tct)c[67942,68017]
cgaggcatagctcagaaggaagagcaaggaccttctaagtcctaggtcgt
aggttcgatccctactgtctcgacca
1–2tRNA–Met(cat)c[68152,68225]
ggccctgtagctggacggtcaagcgagcgactcataatcgctggatggtg
gttcgattccacccagggccacca
>KC595513.2 Bacillus phage Shanette, complete genome
1–1tRNA–Met(cat)[33565,33641]
tggttccttagctcaattggttagagcacacggctcataaccgtgcggtt
gtaggttcgattcctacaggaatcatc
1–3tRNA–Trp(cca)[34766,34841]
tgggggtatagttttaactggctaaaaccgtggtctccaacaccactatt
ctaggttcgaatcctagtgccctcgt
>JF704092.1 Mycobacterium phage Alice, complete sequence
1–1tRNA–Ser(gct)[29314,29397]
ggagggtgagcatctggtgatgcaggggtcctgctaaggccctacggatt
catacccgtgagtttcgattactcctccctccgc
1–2tRNA–Leu(cag)[29494,29568]
gctccgctatgcaaactggcaaagcagccgtgttcagagcgcggtgtttc
tcggttcgaccccgaggcggagtgc
1–3tRNA–Leu(gag)[29572,29646]
gtctctgtaggcaaactggcaaagccgccatcttgagggggtggtgcgtg
cgggttcgactcccgccagagacac
1–4tRNA–Leu(caa)[29647,29720]
gccgtggtaggccatctggcgagccgccgagttcaagtttcggtgtttgc
gggttcgaatcccgcccacggtac
1–5tRNA–SeC(tca)[67477,67573]

attctggcactggtgggcgagcccaccggcggcagcttcaagctgtcgct
ggccggagaatcgaccggaaacatcccgttcaacgcgacccagggcc
1-6tRNA-Pro(tgg)[90640,90714]
cggggtgtagttcagtttggaagagcgcttggtttgggaccaagttgtcg
caggttcgaatcctgtcaccccgac
1-7tRNA-Trp(cca)[90729,90799]
gggtctgtgcacagggtgcccgacggtctccaaagccgaaggcgggggtt
cgattccctccaggcctgcca
1-8tRNA-Tyr(gta)[90801,90885]
cccgtacatgcccaactggtgtttgggagcaggctgtaaccctgtggcct
tcgggacggtgaggttcgattcctcagtgcgggac
1-9tRNA-Pyl(cta)[92340,92412]
gcaccatttgctcaatggcagagcggcggcttctaaaaccgtgagtgtcg
gttcgactccggcatggtgcacc
1-10tRNA-Met(cat)[92562,92636]
agcggtgtagagcagctaggtagctcgccgggctcatgacccggaggacg
cgtgttcgattcacgccaccgccac
1-11tRNA-Cys(gca)[92763,92834]
gcgcctttggcggaatggctacgtgctcggctgcaacccgagttatcccg
gttcgactccgggagggcgctc
1-12tRNA-Glu(ctc)[92902,92973]
gctcccatggggtagtggtaaccctcctggttctcagccaggcgtcccga
gttcgatcctcggtgggagtgc
1-13tRNA-His(gtg)[92975,93048]
gtggccgtagttcagccggtagaacgctgggttgtgatcccagtcgtcga
gggttcgagtccctccggtcaccc
1-14tRNA-Ala(tgc)[93212,93285]
gggcctatagctcatctggtagagcgcctgccttgcaagcaggaggcggc
aggttcaagtcctgttaggtccac
1-15tRNA-Phe(gaa)[93475,93547]
gccgtcatagctcagttggtagagcactggcctgaaaacccggtggccga
ggttcgagtcctcgtgtcggcac
1-16tRNA-Val(cac)[93553,93626]
gtccgtttagctcagctggaagagcgctcggtccacacccgagaggccgc
aggttcgatccctgcaatggacac
1-17tRNA-Gly(gcc)[95021,95096]
gcgaaggtagctcagcctggccagagcatcacgttgccaaggtggaggtc
gcgggatcgtaacccgttcttcgctc
1-18tRNA-Asp(gtc)[95100,95172]
ggccctgtagctcagaggaagagcacccgcctgtcgagcgggaggtcgcg
gtatcgtaatccgtcagggtcgc
1-19tRNA-Met(cat)[95232,95304]
gcctcactagctcattggtagagccgctcgctcataacgtgcaggtacct
ggttcgattccagggtgaggtac

1-20tRNA-Ile(gat)[95310,95384]
gcctgttagcggactggtcgtccgatccaagctgataacttggcgtaagc
ggtgttcgattcaccgagcaggtac
1-21tRNA-Arg(acg)[95478,95550]
gcctctatggtccaacggagatgacgccggtctacggaaccggagatgcg
tgttcgattcgcgctaggggcac
1-22tRNA-Val(gac)[95593,95665]
gtccgtgtagctcaggggtagagcgcctgctcgacacgcaggaggaccga
ggttcgaaacctcgcatggacac
1-23tRNA-Arg(ccg)[95760,95829]
gtcctcgctgacggactctgccctggccctccggaggccgggtgggtggt
tcgattccacccggggacac
1-24tRNA-Arg(cct)[95837,95910]
gcctctgtagctcaacggacagagcaacgcggtcctaacgcggtggctgg
aggttcgaatcctctcggaggcac
1-26tRNA-Gln(ttg)[96432,96507]
tggggtatggtggcaatctggcagtcccgccggactttgactccggaggt
gcaggttcgagccctgctaccccatc
1-27tRNA-Arg(tct)[96511,96586]
gcccttgtagctcagtggacagagcggcgagcttctatctcgcgggccgg
gagttcgaatctctccaggggcacca
1-28tRNA-Lys(ttt)[96636,96709]
ggggcgttggtgaacgtggttatcactcttggcttttacccttgaatacc
gggatcgagacccggacgccccac
1-29tRNA-Gln(ctg)[124380,124454]
tgctcgttggtgtaactggcaacactacggactctggctccgtcattctt
ggttcgaatccagggcgagcaacca
1-30tRNA-Asn(gtt)[124461,124534]
tggggtgtagttcaactggaagaacgctcgactgttaatcgagtagttga
aggttcgagtccttccactccagc
>KP007362.1 Enterobacteria phage vB_EcoM_VR26, complete genome
1-1tRNA-Arg(tct)c[67359,67435]
cggggtgtaactcaattgtatagagtagcggtcctctaaaccgcaggtta
aaggttagaatcctttcaccccgacca
1-2tRNA-Met(cat)c[67440,67514]
ggccctttagctcaatgggagagctgtcagctcataactgataggtagct
ggatcgaaaccagcaagggccacca
>KF767351.1 Lactobacillus phage phi jlb1, complete genome
1-1tRNA-Glu(ttc)[12231,12304]
tggctcgttggtcaaatggctaagactccacactttcacggtggagttac
aagttcaattcttgtacgagctat
>KR296692.1 Salmonella phage 38, complete genome
1-1tRNA-Ser(gct)c[17952,18040]
ggaaggttgcccgagaggtttaagggactcgactgctaatcgagtggggc

ttttagcccccgaaggttcgaatccttcaccttccgcca
1-2tRNA-Tyr(gta)c[18117,18200]
gtgagtgtggcagagcggtcgaatgcaggagactgtaaatctccccgtaa
cagcgcggtggttcaaatccatccactcacacca
1-3tRNA-Asn(gtt)c[18955,19030]
gacgatgtagttcagtcggtagaacggcggtctgttaaaccgtatgtcgc
aggttcaagtcctgccatcgtcgcca
1-4tRNA-Met(cat)c[19443,19517]
ggcccgtagctcagtggttagagcagtcgactcataatcgattggtcgct
ggttcaagtccagccagggtcacca
>KF006817.1 Mycobacterium phage Breeniome, complete genome
1-1tRNA-Ser(gct)[30286,30369]
ggagggtgagcatcaggtgatgcagcgagattgctaatcccgtacggtaa
ccaccccgtgaggttcgaatcctcctccctccgc
1-2tRNA-Leu(cag)[30562,30637]
gctcccgtagcccaattggcaggaggcaccagattcaggatctgggcagt
gtgagttcgaatctcaccgggagtac
1-3tRNA-Leu(gag)[30757,30831]
gtctctgtaggcaaatcggaaaagccgccatcttgagggggtggtgcgtg
cgggttcgactcccgccagagacac
1-4tRNA-Leu(caa)[30832,30905]
gccgtggtaggccatctggcgagccgccgagttcaagtttcggtgtttgc
gggttcgaatcccgcccacggtac
1-5tRNA-SeC(tca)[66355,66451]
attctggcactggtgggcgagcccaccggcggcagcttcaagctgtcgct
ggccggagaaccgaccggaaacatcccgttcaacgcgacccagggcc
1-6tRNA-Pro(tgg)[90723,90795]
cggggtgtagttcagtggaagagcgcttggtttgggaccaagatgtcgca
ggttcgaatcctgtcaccccgac
1-7tRNA-Trp(cca)[90812,90887]
tggggtgaagccgatctggaaggcagcggtctccaaagccgtctcatagc
gggttcgaatcccgtcacccctgcca
1-8tRNA-Tyr(gta)[90889,90975]
gccgcacatgcccaactggtgtttgggagcaggctgtaaccctgtggcct
tcgggacggtgaggttcgattcctcagtgcggtacca
1-9tRNA-Pyl(cta)[92084,92156]
gcaccatttgctcaatggcagagcggcggcttctaaaaccgtgagtgccg
gttcgactccggcatggtgcacc
1-10tRNA-Met(cat)[92306,92380]
agcggtgtagagcagctaggtagctcgccgggctcataacccggaggacg
cgtgttcgaatcacgccaccgccac
1-11tRNA-Cys(gca)[92507,92578]
gcgcctttggcggaatggctacgtgctcggctgcaacccgagttatcccg
gttcgactccgggagggcgctc

1-12tRNA-Glu(ctc)[92583,92657]
ggtccgttggagtagatggatatctcgccaccctctcaaggtggagatca
cgggttcaagtcccgtacggactgc
1-13tRNA-His(gtg)[92659,92732]
gtggccgtagttcagccggtagaacgctgggttgtgatcccagtcgtcga
gggttcgagtccctccggtcaccc
1-14tRNA-Ala(tgc)[92894,92968]
gggcctgtagctccaattggtagagcagcatccttgcaagatgacggctg
tcggttcgaatccgacctggtccac
1-15tRNA-Phe(gaa)[93158,93230]
gccgtcatagctcagttggtagagcactggcctgaaaacccagtggccga
ggttcgattcctcgtgtcggcac
1-16tRNA-Val(cac)[93236,93309]
gtccgtttagctcagctggaagagcgctcggtccacacccgagaggccgc
aggttcgatccctgcaatggacac
1-17tRNA-Lys(ctt)[93428,93500]
gccttcgtagctcagtggtagagctgtcgcctcttaagcgataggtcgtt
ggttcgaatccagccgggggcac
1-18tRNA-Glu(ttc)[93505,93581]
ggtcgggtcggtctgctggtatggccagtcggattttcactccggacatt
cgcgggttcaattcccgtcccgatcgc
1-19tRNA-Gly(tcc)[93661,93733]
gcgggtgtggccgaatggctcaggcaccagatttccactctggctacgca
ggttcgattcctgtcatccgctc
1-20tRNA-Thr(cgt)[93793,93867]
gctgctgtagctcacctggcagagcgtcggcgtcgtatcccgaaggcatc
cggttcgagtccggacagcagcccc
1-21tRNA-Thr(tgt)[93868,93940]
gcctctgtggtccagcggcacggacatccgccttgtaagcggaggacccc
cgttcgatccggggtagaggctc
1-22tRNA-Thr(ggt)[94348,94420]
gctgggttagctcagtggtagagcgttccctcggtacgggaaaggcccgg
ggttcaatcccccgactcagctc
1-23tRNA-Gly(gcc)[95743,95816]
gcgaaggtagctcagctggtagagcgccaccttgccaaggtggaggtcgc
gggatcgtaacccgttcttcgctc
1-24tRNA-Asp(gtc)[95820,95892]
ggccctgtagctcagaggaagagcgccggtctgtcgaatcggaggtcgcg
gtatcgtaatccgtcagggtcgc
1-25tRNA-Met(cat)[95952,96024]
gcctcactagctcattggtagagccgctcgctcataacgtgcaggtacct
ggttcgattccagggtgaggtac
1-26tRNA-Ile(gat)[96030,96104]
gcctgttagcggactggtcgtccgatccaagctgataacttggcgtaagc

ggtgttcgattcaccgagcaggtac
1-27tRNA-Arg(acg)[96198,96270]
gcctctatggtccaacggatatgacgccggtctacggaaccggagatgcg
tgttcgattcgcgctaggggcac
1-28tRNA-Val(gac)[96313,96385]
gtccgtgtagctcaggggtagagcgcctgctcgacacgcaggaggaccga
ggttcgaaacctcgcatggacac
1-29tRNA-Arg(cct)[96559,96632]
gcctctgtagctcaacggacagagcaacgcggtcctaacgcggtggctgg
aggttcgaatcctctcggaggcac
1-31tRNA-Gln(ttg)[97173,97248]
tggggtatggtggcaatctggcagtcccgccggactttgactccggaggt
gcaggttcgagtcctgctaccccatc
1-32tRNA-Arg(tct)[97252,97327]
gcccttgtagctcagtggacagagcggcgagcttctacctcgcgggccgg
gagttcgaatctctccaggggcacca
1-33tRNA-Gln(ctg)[124745,124819]
tgctcgttggtgtaactggcaacactacggactctgactccgtcatttta
ggttcgaatcctaagcgagcagcca
1-34tRNA-Asn(gtt)[124826,124901]
tggggtgtccgttaatcaggcaaacgagcggactgttaatccgcccctgc
aggttcgaatcctgccaccccagcca
>KR296685.1 Salmonella phage 21, complete genome
1-1tRNA-Leu(tag)c[21556,21628]
tggcgattgatggaattggtatcctgccgtccttagaagtcggataaggg
ggttcaagccccctgtcgccacc
1-2tRNA-Gln(ttg)c[22981,23054]
tggaagatggtgtaggattggtagcacacgtgtctttgaaaaccgtagtt
caggttcaagtcctgatcttcccc
>KR296684.1 Salmonella phage 19, complete genome
1-1tRNA-Tyr(gta)[91411,91500]
gtgtcgttagaccgtaggggtagcggggcagactgtaaatctgctggctc
gaaagggcttcgggtagttcgactctatcacggcacacca
1-2tRNA-Ile(gat)[91508,91583]
gggagtatagctcagttagtagagcgctcgaccgataatcgagaggtcgc
aggagcaaagcctgctactcccacca
1-3tRNA-Glu(ttc)[92351,92426]
gctcctatcggctaagtggataggccgccagactttcaatctggaaatcc
gagttcgattctcggtaggagtgcca
1-5tRNA-Trp(cca)[92512,92588]
agggacttagcacaattggctagtgcagcggattccaaatccgcaggttc
tgggttcgaatcccaaggtccctgcca
1-6tRNA-Ser(tga)[92594,92668]
aggaggatggtgtaattggttagcacacgggtcttgaaatccgtagttca

ggttcaagtcctgatcttcctgcca
1-7tRNA-Ser(gga)[92901,92992]
ggtggaatggtcgagcggttaaagacagcaccttggaaaggtgtcggccc
cctcacaggggtccgtaggttcgaatcctacttccaccgcca
1-8tRNA-Ser(gct)[92998,93091]
ggaagattaaccctaatcaggtaagggatctctttgctaaagagacagta
gccccggaaaggggtgtgtcagttcaaatctgacatcttcctcc
1-9tRNA-Ser(tga)[93092,93180]
ggagagcagggcgcgtggtgcgtaatccggtttgaacccggacccatcgt
agcgatacggtgacagttcgattctgttgctctcctcca
1-10tRNA-Asn(gtt)[93328,93412]
gacgagttggcctagtggttgggcggcggcctgttaagccgtgagtgaaa
actcaaggaaggttcaaatccttcactcgtcgcca
1-11tRNA-Asp(gtc)[93420,93495]
ggggatatggtattagcggtaaacatactggcctgtcacgccggagtcac
gggttcgaatcccgttatcctcgcca
1-12tRNA-His(gtg)[93499,93570]
tgggaaattagttaagtggtataatggcctctggtgctgaggcgtctcct
gttcgattcaggaattcccgcc
1-13tRNA-Pro(tgg)[93691,93766]
cagtccgtagcgcagttggtagcgtgggagccttggatgcttcgggtcgc
aggttcgagtcctgccgggctgacca
1-14tRNA-Leu(tag)[94013,94088]
tgggcgattgatggaattggaatacgtgccgtccttagaagtcggatttt
gggggttcgagtccctgtcgcccacc
1-15tRNA-Phe(gaa)[94209,94284]
ggggttgtagctcagttggtagagcacctgattgaagatcagggtgtcgg
tggttcgagtccacccggccccacca
1-16tRNA-Met(cat)[94540,94615]
tgcgagctagaattctggtgaattcacgagtctcataagcttgttcagag
gggttcgattccgctgctcgcaacca
>KP054477.1 Lactobacillus phage LfeInf, complete genome
1-1tRNA-Ser(gct)c[30472,30562]
tggagaattacccaagttggttgaaggggcgcccctgctaagggtgtagg
ccgggaaaccggtgcgagggttcaaatccctcattctccgt
1-2tRNA-Thr(tgt)c[30637,30709]
gctaatttagctcaatcggcagagcgcctgccttgtaagcaggaggttac
aagttcgattcttgtaattagca
>KJ206559.1 Staphylococcus phage 812, complete genome
1-1tRNA-Met(cat)c[14398,14469]
ggactcttagcttaaaggtaaagccaaccgctcataacggtttgactgta
ggttcgaatcctgcagagtcca
1-2tRNA-Trp(cca)c[37617,37688]
acacccttagtataattagtagtacaagggtctccaaaacccttagtctt

tgtgcaaatcaaagagggtgtg

1−3tRNA−Phe(gaa)c[37695,37767]

ggtttcttagctcagatggtagagcactagattgaagctctaggtgtcat

tggttcaaatccaatagaaacca

1−4tRNA−Asp(gtc)c[37773,37848]

tggctcattggtgtaactggttaacacactgccctgtcacggcagagagt

acgagttcgagtctcgtatgggtcgt

>LN887844.1 Pseudomonas phage VCM genome assembly VCM, chromosome : 1

1−1tRNA−Gly(tcc)c[69564,69636]

gtgtgtcgtataacggctattatatctggcttccacccagaggactaggg

ttcgattccctacacccgcacca

1−2tRNA−Cys(gca)c[69644,69718]

tccttgttggctgaacggctaggcggctgattgcaaaccagcattaagtg

ggttcaactcccacacaaggctcca

1−3tRNA−Leu(tag)c[69783,69867]

gggttcgtggtggaactggtatacacactagtcttagaaactagcgccga

aaggattgagggatcgtgaccctccgagcctacca

1−4tRNA−Ala(tgc)c[70116,70191]

gggcacatgttccaagggatggcgagtttgccttgcacgcagactgtgaa

gagttcgattctctttgtctccacca

1−5tRNA−Pro(tgg)c[70385,70458]

ctctcggaagcgttacggtagcgtactttgtttggaacgaagtggcggtg

gttcgactccaccccgagtgacca

1−6tRNA−Asn(gtt)c[70593,70669]

aggcaatgatctgactaggtgttggtctgtgactgttaatcacaagatgg

gaggttcgaatcctctattgcctgcca

1−7tRNA−Ile(gat)c[70821,70894]

tgctcgttagcatagtggtaatgcaatcggctgataaccgatagagaggt

gttcaattcacttacgagcaacca

1−8tRNA−Arg(acg)c[70901,70975]

agtcgcttggccgattggattaggcaaaggtctacgaagccttttagagt

ggttcgatcccactagtgactacca

1−9tRNA−Asp(gtc)c[71118,71193]

ggctcattagtgttagcggttagcacgcttgactgtctatcaggaaggag

ggattcgaattccctatgagccgcca

1−10tRNA−Glu(ttc)c[71198,71274]

gctcccatcgtctatctggtctaggattcctgactttcaatcaggggaac

agggttcgaatccctgtgggagtacca

1−11tRNA−Arg(tct)c[71504,71580]

ggccttatagtgtaagggaattagcacatcatccttctaagttgatagac

ttggttcgaatccaagtagggctacca

1−12tRNA−Gln(ttg)c[72332,72406]

ggccggttggcgaagtgggaacgcagcgagctttgacctcgttatgagta

ggttcgatccctacaccggacgcca

1-13tRNA-Val(tac)c[72754,72827]
gcctcattagttcaatgggagaacgccatccttacaaggtggctacggtg
gttcgattccatcatgaggcacca
1-14tRNA-Leu(caa)c[72836,72922]
ggggatatggcggaactggtattcgcagcaggctcaaaccctgtgggccg
tgaggccatgtaggttcgactcctattatccccacca
1-15tRNA-Ser(aga)c[72927,73016]
ggcgagttggtagagtggacgattgctctagtttagaaaactagcgagtc
agaaatggctccgtgggttcgaatcccacactcgcctcca
1-16tRNA-Ser(gct)c[73265,73354]
ggcgtattggtcgagaggctgatgacagcgggttgctaacccgtactctc
cgaaaggggagcgaaggttcgaatccttcattcgccacca
1-17tRNA-Lys(ttt)c[73360,73434]
tggacggaagctaagcggtcaggcatcggacttttaatccgaacatggtg
ggttcgattcccactcgtccaacca
>KR422352.1 Escherichia phage APCEc01, complete genome
1-1tRNA-Met(cat)[110541,110614]
ggccctgtagctcaatggttagcagcagtcccctcataagggaaaggtta
ccagttcgaatctggtctgggtca
1-2tRNA-Arg(tct)[110621,110697]
cgaggcatagctcaattgtatagagcaacggacttctaatccgtaggttg
aaggttagaatccttctgtctcgacca
>KJ010547.1 Bacillus phage Bp8p-C, complete genome
1-1tRNA-Met(cat)c[106842,106913]
gggtctttagcttaaaggtaaagcaatcagctcataactgataggatagg
agttcgagtctcttaggaccca
1-2tRNA-Tyr(gta)c[109352,109435]
gggatagcgctaacgttggagagttaggcgagactgtaaatctcgtgtcg
gatcactgagtgggttcgaatcccacctgtccca
1-3tRNA-His(gtg)c[109549,109619]
gagaatgtagcttaacggtaaagcgctgggacgtgactccagttatccaa
gttcgagtcttggtattctca
1-4tRNA-Gly(tcc)c[109630,109703]
gtggtcttagtttaacggtagaattgctggcttccaaccagcgggtaagg
gttcgactcccttagaccatacca
1-5tRNA-Met(cat)c[110832,110905]
tacgatgctgaggggtcggtacccgacgaggtctcataagccttgtttag
caagttcaactcttgccttagcaa
1-6tRNA-Phe(gaa)c[110979,111053]
tggactggtagctcagtcggtagagcagggggctgaaaatccctgtgtcg
gaggttcgaatccttctcagtccat
1-7tRNA-Asn(gtt)c[111433,111506]
ttgtcgtataactcaatggtagagtgcttgactgttaatcaagaagttgt
aggttcgagtcctactacgacagt

>KU297675.1 Pseudomonas phage PaoP5, complete genome

1-1tRNA-Gln(ttg)[21140,21213]
tgccgcttcgttcaatggtaggacgccagactttgaatctggagatgatg
gttcgatcccatcagtggctgcca
1-2tRNA-Arg(tct)[21419,21494]
gctcgtatagtgtaatggacagcacaacggtcttctaagccgtaaggtct
aggttcgaatcctagtatgagcgcca
1-3tRNA-Leu(tag)[22310,22394]
gaccctgtggtggaattggtatacacatcagtcttagaaactgacgccga
gaggattgagggttcaagtccctccggggccacca
1-4tRNA-Ile(gat)[22605,22680]
agccggatagctcaattggtagagcacccgaccgataatcggatggttga
aggttcaagtccttctctggctacca
1-5tRNA-Asp(gtc)[22690,22768]
ggcccattagctcagtctggactagagcaagcccctgtctagggcaaggt
cgtcggttcgaatccgacatgggtcgcca
1-6tRNA-Met(cat)[22869,22943]
agcgttaagactggctggagctgtcactcggctcataaccgagcacaagt
ggttcgattccacctctcgcttcca
1-7tRNA-Cys(gca)[23179,23254]
cccgcgttggccgagaggtttaggcggcggattgcaaatccgtctcacat
cggttcaaatccgatacgcggctcca
1-8tRNA-Asn(gtt)[23265,23340]
tgggatgtagctcagttggtagagcagggagctgttaactctcaggtcgc
aggttcgagccctgccgtcccagcca
1-9tRNA-Pro(tgg)[23404,23481]
ctcctcgtagctcagtctggttagagtgtcggatttggaatccgaaggtc
gaaggttcaaatccttccggggtgacca
1-10tRNA-Gly(tcc)[23653,23728]
gcgggtatagctcagttggtagagcgtctgccttccaagcagttcgtcgt
cggttcgagtccgtctatccgctcca
1-11tRNA-Phe(gaa)[23735,23810]
gcatttgaagctaactaggtagaagcgctgggttgaaattccagaggact
tggatcgttaccaagcagatgcacca
1-12tRNA-Glu(ttc)[23817,23892]
gcagttatagattaattggttaaatcgccagactttcaatctggtgttcc
gggttcgatccccggtaactgctcca
1-13tRNA-His(gtg)[23953,24027]
gtggagattgtgtagcccggatgctgcctttcggttgtgacccgattgtt
ggggttcgagtccccgcctccaacc
1-14tRNA-Thr(tgt)[24214,24288]
gcccttta agcatttgtggtgatgcaccggctttgtaacccggcgaattc
tgttcaaatcaggaatggggcacca

>KU130126.1 Pseudomonas phage vB_PsyM_KIL1, complete genome

1−1tRNA−Ser(tga)[16011,16105]
ggcttggtaagccgagatggcctagcggcagcggtcttgaaaaccgaggg
ttcacggaagtggacgtgtgagttcgagtctcacccaatccgcca
1−2tRNA−Met(cat)[16112,16188]
tggcctttagcataattggttaatgcaatcaactcataattgataagata
cgagttcaagtctcgtggggccaacca
1−3tRNA−Arg(tct)[16759,16835]
atctccatagctcaactggacagagcaagggttttctacacccaaggttg
agggttcgaatccttctggggatacca
1−4tRNA−Thr(tgt)[17460,17535]
gccggaatagcacaattggtagtgcagtcgccttgtaagcgaaaggttga
gggttcaagtcctttttcggcacca
1−5tRNA−Arg(acg)[17725,17799]
agtcgcttggccgattggattaggcaaaggtccacgaagcctgctagagt
ggttcgactccactagttactacca
>KT887559.1 Pseudomonas phage phi3, complete genome
1−1tRNA−Met(cat)[2854,2930]
gggcctatagctcagtcggttagagcagaggactcataatcctttggtcc
acggttcgagtccgtgtgggcccacca
>KU497559.1 Pseudomonas phage K5, complete genome
1−1tRNA−Gln(ttg)[22291,22364]
tgccgcttcgttcaatggtaggacgccagactttgaatctggagatgatg
gttcgatcccatcagtggctgcca
1−2tRNA−Arg(tct)[22570,22644]
gctcgtatagtgtaacggatgcacaacggtcttctaagccgtaaggtcta
ggttcgaatcctagtatgagcgcca
1−3tRNA−Lys(ttt)[22652,22728]
tggacggaagctaaagtggataggcatctggcttttaaccagatttatag
tgagttcgagtctcacccgttcaacca
1−4tRNA−Leu(tag)[23003,23087]
ggccctgtggtggaattggtatacacatcagtcttagaaactgacgccga
gaggattgagggttcaagtccctccggggccacca
1−5tRNA−Ile(gat)[23298,23373]
ggccagatagctcaattggtagagcacccgaccgataatcgggtggttga
aggttcaagtccttctctggccacca
1−6tRNA−Asp(gtc)[23383,23461]
ggcccattagctcagtctggactagagcaagcccctgtctagggcaaggt
cgccggttcgaatccggcatgggtcgcca
1−7tRNA−Cys(gca)[23718,23793]
ccctcgttggccgagaggatcaggcagcagattgcaaatctgccatacat
cggttcaaatccgatacgaggctcca
1−8tRNA−Asn(gtt)[23803,23879]
tccgtacggcccatcaaggcgatggggctcggctgttaaccgaaacgcgc
taggttcgattcctaggtacggagcca

1-9tRNA-Pro(tgg)[23942,24019]
ctcctcgtagctcagtctggttagagtgtcggatttggaatccgaaggtc
gaaggttcaaatccttccggggtgacca
1-10tRNA-Gly(tcc)[24027,24103]
gcgggtttcgtatagtctggttattattcttggcttccacccaagagact
agggttcgaatccctaagcccgcacca
1-11tRNA-Phe(gaa)[24166,24241]
gcgtctgaagctaactaggtagaagcgctgggttgaaattccagaggact
tggatcgttaccaagcagatgcacca
1-12tRNA-Glu(ttc)[24248,24323]
gcagttatagattaattggttaaatcgccagactttcaatctggtgttcc
gggttcgatccccggtaactgctcca
1-13tRNA-His(gtg)[24384,24458]
gtggagattgtgtagcccggatgctgcctttcggttgtgacccgattgtt
ggggttcgagtccccgcctccaacc
1-14tRNA-Thr(tgt)[24645,24719]
gcccttaagcatttatggtgatgcaccggctttgtaacccggcgaattc
tgttcaagtcaggaatggggcacca
>LC121084.1 Ralstonia phage RSP15 DNA, complete genome
1-1tRNA-Leu(caa)c[85007,85091]
ggggatgtgggggaattggtagacccacctgactcaaaatcaggcgctga
aaggcgtgtgggttcgagtcccaccatccctacca
1-2tRNA-Gly(tcc)c[85106,85183]
gcgggtataactcagtccggtcagagtgtcagccttccaagctgttcgtc
atcggttcaaatccgattacccgctcca
1-3tRNA-Ile(gat)c[85392,85468]
ggtggattagctcagttggttagagcatcgtcttgataaggcgggggcca
taggttcaagtcctatatccaccacca
1-4tRNA-Thr(tgt)c[85589,85664]
gccacgttagcacaattggtagtgcagcggatttgtaatccgaaggttag
ggattcgatttctctacgtggcacca
1-5tRNA-Lys(ttt)c[87081,87157]
gagggtgtagctcaattggttagagcaacggacttttaatccgtaggttg
agggttcgattccctccgccctcacca
1-6tRNA-Trp(cca)c[87407,87481]
gggcggatagtataatggctattatgtcggtttccaagtccgaagattga
ggttcgattccttgtctgcccgcca
1-7tRNA-Arg(tct)c[87489,87564]
gcggcattagctcaatggatagagcaagggatttctactccctaggttgt
gggttcaagtcctgcatgccgcgcca
1-8tRNA-Gln(ttg)c[90562,90637]
cggggtgtagttcagttggtagaacagcgggttttgatcccgtaggtcgt
aggttcgagtcctaccgcccctgcca
1-9tRNA-Met(cat)c[90734,90809]

ggtaatatagttcagttggtagaacagcagattcataatctgtaccgcgc
tggttcaaatccagctattaccacca
1-10tRNA-Val(tac)c[91136,91210]
gggaaattagctcagaggcagagcagcgcccttacaaggcgaaggtcggg
gtttcgatatcctcatttcctacca
1-11tRNA-Asn(gtt)c[91439,91515]
tccgtagtagctcagtcggtagtagcgttcgcctgttaagcgaaaggtcg
gtggatcgtacccaccctacggagcca
1-12tRNA-Leu(tag)c[92265,92350]
gcgcgagtgatggaattaggtagtcttctgtgccttagaagcatgggcct
tcgggcgtgtcggttcgagtccgaccttgcgcacca
1-13tRNA-Phe(gaa)c[92665,92740]
gcgcgggtagctcagttggtagagcgccagcctgaagagttgggcgtcgg
aggttcgatcccttccccgcgcacca
1-14tRNA-Tyr(gta)c[92941,93022]
gggttggatgctctaatggtagggcagcgggctgtaaccccgtggcttcg
gcaagtaggttcgatccctactcaacccacca
1-15tRNA-Pro(tgg)c[93034,93108]
ccgtggttagcctagaggtcaggcacttgatttgggatcaagacgacgaa
ggttcgatcccttcatcacggacca
1-16tRNA-Asp(gtc)c[93341,93416]
ggtccaaaagtgtttatggacgcacacatgcctgtcacgcatgtagagtg
gggatcgttaccccccttggaccgcca
1-17tRNA-Cys(gca)c[93434,93507]
gtgagtttggccgagtgactaggcgataggctgcaaccctatctaggaaa
gttaaaatctttcaattcactcca
1-18tRNA-Glu(ttc)c[93924,94002]
gtccgcatagtttacggtaggtttagaacacatgcctttcacgcatgtaa
atcgggttcgaccccccgatgtggacgcca
1-19tRNA-Ser(gct)c[94108,94198]
ggaagattggcagagtggccgattgcagcgggttgctaacccgtagggtc
tgaataaggctccataggttcaaatcctatatcttccgcca
1-20tRNA-Ser(tga)c[94946,95038]
ggaagctttagccgagatggcctagcggcagcggtcttgaaaaccgaagg
ctccgaaaggggtgtgtgagttcgagtctcacagcttccgcca
>KU253712.1 Bacillus phage Eldridge, complete genome
1-1tRNA-Asn(gtt)[31289,31363]
tcctccttagccaagtggtaaggcatcacactgttaatgtgactatcgca
agttcgaatcttgcaggaggagcca
1-2tRNA-Trp(cca)[31366,31439]
tatcggtctagtgtaatggtagcacagaggtctccaaaacctttagtctg
ggttcaaatcctagggctggtgtc
1-3tRNA-Met(cat)[31833,31904]
atctttgtagctcagtggtagagcgcccgactcatattcggaaggtcgtt

ggttcaaacccaaccagagata
>AB930182.1 Bacillus phage SPG24 DNA, contig00001 sequence
1-1tRNA-Ser(gct)[108723,108811]
tggataggtacccaagcggtaaaggggtctggttgctaaccagatagtag
gctgaggtctagcggaggttcgaatcctctcctatccgt
1-2tRNA-Asn(gtt)[109047,109120]
ttgtcccatagctcaatggtagagcgcccgactgttaatcggaaggttgc
tggttcgagcccagctgggacagt
1-4tRNA-Asp(gtc)[110903,110976]
tggctccatagtgcagcggttaacacgcccgcctgtcacgtgggtagcac
gggttcgaatcccgttggagccgt
1-5tRNA-Tyr(gta)[111142,111225]
gggtgagcggtaacgttggagagttacggcagactgtaaatctgttccct
tcggggttagagtgttcgaatcactccttacccc
>KX130861.1 Shigella phage SHFML-11, complete genome
1-1tRNA-Arg(tct)c[16071,16147]
cgaggcatagctcaattgtatagagcaacggacttctaatccgtaggttg
aaggttagaatccttctgcctcgacca
1-2tRNA-Met(cat)c[16858,16929]
ggccctgtagctggaaggttcaagcaagcgactcataatcgccagatggt
ggttcaattccacccagggcca
1-3tRNA-Thr(tgt)c[16936,17008]
gctgatttagctcagtaggtagagcaactcacttgtaatgagaaggtcgg
cggttcgattccgtcaatcagca
1-4tRNA-Ser(tga)c[17015,17103]
tggaggcgtggcagagtggtttaatgcaccggtcttgaaaaccggcagtc
gctccggcgactcataggttcaaatcctatcgcctccgt
1-5tRNA-Pro(tgg)c[17104,17178]
cttcgtgtagctcagtttggtagagcgtctgctttgggagcagaatgtcg
caggttcaaatcctgccgcggagac
1-6tRNA-Gly(tcc)c[17191,17262]
gcggatatcgtataatggtattacctcagacttccaatctgatgatgtga
gttcgattctcattatccgctc
1-7tRNA-Leu(taa)c[17269,17355]
gcgagaatggtcaaattggtaaagacacagcacttaaaatgctgcggaat
gatttccttgtgggttcgagtcccacttctcgtacca
1-8tRNA-Gln(ttg)c[17356,17429]
tgggaattagccaagttggtaaggcatagcactttgactgctagatgcaa
aggttcgagtcctttattcccagc
>KX397366.1 Erwinia phage vB_EamM_ChrisDB, complete genome
1-1tRNA-Trp(cca)[977,1052]
agggaagtagctcagcaggcagagcgctggtctccaaaaccagatgtcgc
aggttcgattcctgtcttccctgcca
1-2tRNA-Cys(gca)[1369,1444]

ggggcaatggctcgagcggtcaggcgctagattgcaaatctggattacgg
gggttcaattccctcttgcccctcca
1−3tRNA−Tyr(gta)[3038,3123]
ggcgcggtactcaagcggtcaacgagagcagactgtaaatctgttgcctt
cgggcttcgtaggttcaaatccttccctcgccacca
1−4tRNA−Gln(ttg)[3130,3204]
aggggtatagccaagcggtaaggcccagggctttgatccctgtatgctct
ggttcaaatccagatgccccttcca
1−5tRNA−Ile(gat)[3211,3287]
tgtctcgtagctcagttggtcagagcgctcgcctgataagcgggaggtca
gtggttcaagtccactcgagacaacca
1−6tRNA−Met(cat)[3292,3367]
ggttctgtagctcagtcggtagagcacgccactcataatggcgcggtcgt
tggttcgataccagccggaaccacca
1−7tRNA−Glu(ttc)[3573,3648]
tctcccatcatctagcggtctaggatcgccgcctttcgagcggttaaccg
gggttcaaatccccgtgggagaacca
1−8tRNA−His(gtg)[3918,3994]
gtgtacgtagttcagttggttagaattctggcttgtgacgtcggaggtcg
agggttcaagtccctccgtacacccca
1−9tRNA−Asp(gtc)[4000,4075]
agtgccgtagtcgagtggctaagacgctcccctgtcacgggagagatcgt
gggttcaaaccccatcggcactgcca
1−10tRNA−Ser(gct)[4081,4168]
ggagaattgaccgagaggccgaaagtgctcccctgctaagggagtggtcc
ttaggggcccgaaggttcgaatccttcattctccgcca
1−11tRNA−Asn(gtt)[4446,4522]
ggttccatagctcagttggttagagcggctgcctgttaagcagtaggtcc
ctggttcgagtccaggtggtaccgcca
>KR269720.1 Klebsiella phage PKO111, complete genome
1−1tRNA−Thr(tgt)c[85,160]
gccgatttagctcagctggtagagcgcttcacttgtaatgaagatgtcgc
gggttcgactcctgcaatcggcacca
1−2tRNA−Leu(taa)c[261,345]
gcatcgatggtggaactggtatacacatagcacttaaaatgctacgccgc
aaggattgagggttcgaatccctctcggtgcacca
1−3tRNA−Arg(tct)c[350,426]
cggggcatagctcagctggatagagcagcggacttctaatccgcaggtcg
aaggttcgaatccttctgcctcgacca
1−4tRNA−Met(cat)c[2039,2113]
ggccctgtagctcaatggtagagcacgcaactcataattgctaggtttct
ggttcgagtccaggctgggtcacca
1−5tRNA−Pro(tgg)c[3381,3457]
ctccgtatagctcagtctggtagagcgctccatttgggatggagaggtcg

aatgttcgagtcattctatggagacca
1-6tRNA-Gly(tcc)c[3463,3537]
gcatccatcgtatagcggatattatgtctggcttccacccagaagatagg
agttcgattctccttggatgctcca
1-7tRNA-Trp(cca)c[3543,3617]
aggttcttagtataacggctattatgctgggctccaaacccagtgatgag
ggttcgattccttcagggcctgcca
1-8tRNA-Ile(gat)c[3624,3699]
gggagtatagctcatttggtagagctctcgaccgataatagagcggtgac
tggttcgagtccagttactcccacca
1-9tRNA-Ser(tga)c[3766,3850]
ggagagtagcgctagtggtagcaaaccggacttgaaatccgggccatcgg
aaacggtgagggttcaactcctttactctccgcca
1-10tRNA-His(gtg)c[3948,4023]
gtggtcgtagttcagttggtagaacccgaggttgtgatcctcgatgtcac
ggattcgaattccgtcggccacccca
1-11tRNA-Gln(ttg)c[4119,4193]
tgaatcatagccaagttggtaaggcagtaggttttgatcctacgatccct
ggttcgagtccaggtggttcagcca
1-12tRNA-Met(cat)c[4200,4276]
tgcggagtaacttcagttggtagaatgttgggctcatatcccgacacgcg
caggttcgattcctgcctccgcctcca
1-13tRNA-Asp(gtc)c[4438,4513]
ggacctatagtttcagcggttaaaatactcgcctgtcacgcgagagtcac
ggattcgaattccgttaggtccgcca
1-14tRNA-Asn(gtt)c[4767,4851]
gggtcgttggctgagagggtaagcgacggactgttaatccgtgtcagaaa
tgactaggcaggttcgatacctgcacggcccgcca
1-15tRNA-Lys(ttt)c[4857,4933]
gggatactagctcagttggttagagcaccggacttttaatccgggtgtcc
gaagttcgagtcttcggtgtcccacca
1-16tRNA-Tyr(gta)c[4940,5026]
ggggagttagaccgtaggggtagcgggacagactgtaaatctgttgctca
aaaggctcgagtggttcgactccattactccccacca
>KU878088.1 Bacillus phage AR9, complete genome
1-1tRNA-Asn(gtt)[208904,208977]
ttcccctttagctcaatggtagagcatctcactgttactgagatggttgc
aagttcgagtcttgcagggggagt
>KX147229.1 Bacillus phage Belinda, complete genome
1-1tRNA-Asn(gtt)[33227,33300]
gtgcttgtagctcagtcggtagagctggtggctgttaaccactgtgtcgt
aggttcgattcctacctagcacgc
1-2tRNA-Glu(ttc)[33307,33383]
gtcgcattggtgaaattggctaacacactcggctttctaccgaggattta

ggggttcgagccccctatgcgattcca
1-3tRNA-Asp(gtc)[33388,33461]
tggggatatagtgtagaggtaaacacgcacggctgtctaccgtgaagcac
gagttcgaatctcgttatcctcgt
>KX009778.3 Escherichia phage UFV-AREG1, complete genome
1-1tRNA-Asn(gtt)c[68817,68891]
ggatgtgtagctcagtggtagagcagttgactgttaatcaattggtccat
ggttcgaatccatgcatgtccgcca
1-2tRNA-Tyr(gta)c[68896,68982]
ggggagttatcccgtagaggtagcggtgtggactgtaaatccattgtcat
tgcgactcgggtggttcgactccatcactccccacca
1-3tRNA-Met(cat)c[68992,69068]
ggccctgtagctcaatggttagcagcagtcccctcataagggaaaggtta
ccagttcaaatctggtctgggtcacca
1-4tRNA-Thr(tgt)c[69070,69145]
gctgatttagctcagtaggtagagcacctcacttgtaatgaggatgtcgg
cggttcgattccgtcaatcagcacca
1-5tRNA-Ser(tga)c[69147,69235]
tggaggcgtggcagagtggtttaatgcaccggtcttgaaaaccggcagtc
gctccggcgactcataggttcaaatcctatcgcctccgt
1-6tRNA-Pro(tgg)c[69236,69310]
ctccgtgtagctcagtttggtagagcgcctgatttgggatcaggaggtcc
aaggttcaaatccttgtatggagac
1-7tRNA-Gly(tcc)c[69902,69975]
gcggatatcgtataatggcattacctcagacttccaatctgatgatgtga
gttcgattctcattatccgctcca
1-8tRNA-Leu(taa)c[69982,70068]
gcgagaatggtcaaattggtaaaggcacagcacttaaaatgctgcggaat
gatttccttgtgggttcgagtcccacttctcgtacca
1-9tRNA-Gln(ttg)c[70069,70142]
tgggaattagccaagttggtaaggcatagcactttgactgctagatgcaa
aggttcgagtcctttattcccagc
1-10tRNA-Arg(tct)[169204,169280]
cggggtgtaactcaattgtatagagtagcggtcctctaaaccgcaggtta
aaggttagaatcctttcaccctggcca
>KU761955.1 Pseudomonas phage phiMK, complete genome
1-1tRNA-Thr(tgt)c[67352,67426]
gcccctttaagcatttatggtgatgcaccggctttgtaacccggcgaattc
tgttcaagtcaggaatggggcacca
1-2tRNA-His(gtg)c[67613,67687]
gtggagattgtgtagcccggatgctgcctttcggttgtgacccgattgtt
ggggttcgagtccccgcctccaacc
1-3tRNA-Glu(ttc)c[67748,67823]
gcagttatagattaattggttaaatcgccagactttcaatctggtgttcc

gggttcgatccccggtaactgctcca
1−4tRNA−Phe(gaa)c[67830,67905]
gcatttgaagctaactaggtagaagcgctgggttgaaattccagaggact
tggatcgttaccaagcagatgcacca
1−5tRNA−Gly(tcc)c[67912,67987]
gcgggtatagctcagttggtagagcgtctgccttccaagcagttcgtcgt
cggttcgagtccgtctatccgctcca
1−6tRNA−Pro(tgg)c[68159,68236]
ctcctcgtagctcagtctggttagagtgtcggatttggaatccgaaggtc
gaaggttcaaatccttccggggtgacca
1−7tRNA−Asn(gtt)c[68299,68374]
tgggatgtagctcagttggtagagcagggagctgttaactctcaggtcgc
aggttcgaaccctgccgtcctagcca
1−8tRNA−Cys(gca)c[68385,68460]
cccgcgttggccgagaggtttaggcggcggattgcaaatccgtctcacat
cggttcaaatccgatacgcggctcca
1−9tRNA−Asp(gtc)c[68916,68994]
ggcccattagctcagtctggactagagcaagcccctgtctagggcaaggt
cgccggttcgaatccggcatgggtcgcca
1−10tRNA−Ile(gat)c[69004,69079]
ggccagatagctcaattggtagagcacccgaccgataatcgggtggttga
aggttcaagtccttctctggccacca
1−11tRNA−Leu(tag)c[69290,69374]
ggccctgtggtggaattggtatacacatcagtcttagaaactgacgccga
gaggattgagggttcaagtccctccggggccacca
1−12tRNA−Arg(tct)c[69732,69806]
gctcgtatagtgtaacggatgcacaacggtcttctaagccgtaaggtcta
ggttcgaatcctagtatgagcgcca
1−13tRNA−Gln(ttg)c[70012,70085]
tgccgcttcgttcaatggtaggacgccagactttgaatctggagatgatg
gttcgatcccatcagtggctgcca
>KU760857.1 Salmonella phage SJ46, complete genome
1−1tRNA−Met(cat)c[98316,98391]
ggccctttagctcagtggttagagctggcgactcataatcgcacggtcac
cggttcaagtccggtaggggccacca
1−2tRNA−Thr(tgt)c[99544,99619]
gccggtttagctcagttggtagagcgcctgccttgtaagcaggatgtcag
cggttcgagtccgttaatcggcacca
1−3tRNA−Asn(gtt)c[99622,99697]
gatggtgtagctcagcggttagagcggttgactgttaatcaacgggtcga
tggttcaaatccatccaccatcgcca
>KU867876.1 Escherichia phage vB_EcoM−UFV13, complete genome
1−1tRNA−Arg(tct)c[69811,69886]
cgaggcatagctcagaaggaagagcaaggaccttctaagtcctaggtcgt

aggttcgatccctactgcctcgacca
1-2tRNA-Asn(gtt)c[69891,69965]
ggatgtgtagctcaatggcagagcgatcgcctgttaagcgattggttata
ggttcgaatcctatcacgtccgcca
1-3tRNA-Tyr(gta)c[69970,70056]
ggggagttatcccgtagaggtagcggtgtggactgtaaatccattgtcat
tgcgactcgggtggttcgactccatcactccccacca
1-4tRNA-Met(cat)c[70069,70143]
ggccctgtagctggaaggttcaagcaagcgactcataatcgccagatggt
ggttcaattccacccagggccacca
1-5tRNA-Thr(tgt)c[70145,70220]
gctgatttagctcagtaggtagagcaactcacttgtaatgagaaggtcgg
cggttcgattccgtcaatcagcacca
1-6tRNA-Ser(tga)c[70226,70315]
ggaggcgtggcagagtggtttaatgcaccggtcttgaaaaccggcagtcg
ctccggcgactcataggttcaaatcctatcgcctccgcca
1-7tRNA-Pro(tgg)c[70317,70391]
ctccgtgtagctcagtttggtagagcgcctgatttgggatcaggaggtcc
aaggttcaaatccttgtatggagac
1-8tRNA-Gly(tcc)c[70402,70475]
gcggatatcgtataatggtattacctcagacttccaatctgatgatgtga
gttcgattctcattatccgctcca
1-9tRNA-Leu(taa)c[70481,70567]
gcgagaatggtcaaattggtaaaggcacagcacttaaaatgctgcggaat
gatttccttgtgggttcgagtcccacttctcgcacca
1-10tRNA-Gln(ttg)c[70568,70641]
tgggaattagccaagttggtaaggcactggattttgattccaggatgcaa
aggttcgagtcctttattcccagc
>KX397369.1 Erwinia phage vB_EamM_Kwan, complete genome
1-1tRNA-Gly(tcc)[1976,2051]
gcaggcatagctcagttggtagagcatctgatttccaatcagagggtcgt
cagttcgaacctgactgtctgctcca
1-2tRNA-Tyr(gta)[2256,2340]
ggcgggttactcaagcggcaacgagagcagactgtaaatctgttgacttc
ggtcttcggtggttcgagtccaccacacgccacca
1-3tRNA-Glu(ttc)[2346,2420]
acacccatcgtctagcggttaggaccgctgcctttcgagcagttaaccgg
ggttcaaatccccgtgggtgtacca
1-4tRNA-Glu(ttc)[2424,2499]
attcccgtcatctagttggtaaggatacgacactttcactgtcggtacac
gagttcgagtctcgtcgggaatacca
1-5tRNA-Trp(cca)[2623,2698]
agggaagtagttcaattggtagagcgtcggtctccaaaaccggttgttgg
aggttcgagtcctctcttccctgcca

1−6tRNA−Asp(gtc)[2705,2781]
aggactgtagctcagacggttagagtgccgccctgtcacggcggaggtcg
cgggttcgaaacccgtcagtcctgcca
1−7tRNA−Ser(gct)[2787,2876]
ggagaattggatgagtggcttaagtcgctcccctgctaagggagtgtatc
cgaaagggtaccgttggttcgaatccaacattctccgcca
1−8tRNA−Asn(gtt)[3253,3328]
tggtgattagttcagtcggtagaacagtggactgttaatccatatgtcgc
aggttcgattcctgcatcaccagcca
>KX397364.1 Erwinia phage vB_EamM_Asesino, complete genome
1−1tRNA−Trp(cca)[613,688]
gggggagtagttcaactggtagagcgtcggtctccaaaaccgaatgttgc
aggttcgagtcctgtctcccctgcca
1−2tRNA−Cys(gca)[1007,1082]
ggggcaatggctcgagtggttaggcgacgaattgcaaattcggatcaggt
cggttcaagtccggcttgcccctcca
1−3tRNA−Gly(ccc)[3308,3383]
gcgagtatagctcagctggtagagcatctggttcccaaccagagggtcgt
gagttcgaatctcattactcgctcca
1−4tRNA−Tyr(gta)[3492,3577]
ggcgcggtactcaagcggtcaacgagagcagactgtaaatctgttgcctt
cgggcttcgtaggttcaaatccttccctcgccacca
1−5tRNA−Glu(ttc)[3584,3659]
gttcctgtcgtctagtggtccaggacacctggttttcaaccaggcaaccg
gagttcaattctccgcaggaacgcca
1−6tRNA−Asp(gtc)[3815,3891]
agagccgtagtcgagttggttaagacgcctccctgtcacggaggagatcg
tgggttcgagccccatcggttctgcca
1−7tRNA−Ile(gat)[4027,4103]
tgtctcgtagctcagttggttagagcgctcgcctgataagcgggaggtcg
gtagttcaagtctactcgagacaacca
1−8tRNA−His(gtg)[4199,4275]
gtgtatgtagttcagctggttagaatactggcttgtgacgtcagtggtcg
agagttcgagtctctccatacacccca
1−9tRNA−Ser(gct)[4281,4370]
ggagaattgtccgagtggctgaaagagctcccctgctaagggagtggtgg
cgcaagctgcccgaaggttcgaatccttcattctccgcca
1−10tRNA−Met(cat)[4506,4579]
aacccgttagcttgaggaaaaagcacccgacccataatcggatttttcac
tggttcgaatccagtacggtgttc
1−11tRNA−Asn(gtt)[4589,4664]
gactctgtagttcagttggtagaacggatgactgttaattatctcgtcgc
aggttcgagtcctgccggagtcgcca
>KU737349.1 Bacillus phage DIGNKC, complete genome

1-1tRNA-Asn(gtt)[33193,33266]
gtgcttgtagctcagtcggtagagctggtggctgttaaccactgtgtcgt
aggttcgattcctacctagcacgc
1-2tRNA-Glu(ttc)[33273,33349]
gtcgcattggtgaaattggttaacacactcggctttctaccgaggattta
ggggttcgagccccctatgcgattcca
1-3tRNA-Asp(gtc)[33354,33427]
tggggatatagtgtagaggtaaacacgcacggctgtctaccgtgaagcac
gagttcgaatctcgttatcctcgt
>KX245890.1 Citrobacter phage vB_CfrM_CfP1, complete genome
1-1tRNA-Met(cat)[51490,51567]
ggccccttagctcaattggttagagcgaaccccctcataagggtgtggttt
ccggttcgagtcacggaaggggccacca
>KX078569.1 Morganella phage vB_MmoM_MP1, complete genome
1-1tRNA-Met(cat)c[62923,62996]
tgcgagatagtgaagaggtctcacacgaggttcatgccctcgaaacccta
gttcgaatctaggtctcgcttcca
1-2tRNA-Glu(ttc)c[63866,63940]
gcacctatcgtctagtggttaggacatcaggttttcaacctggcaaccgg
agttcaattctccgtaggtgtacca
1-3tRNA-His(gtg)c[63946,64021]
gtggccgtaattcagttggtagaatccctgattgtgattcaggcagtcac
cggttcgaagccggtcggtcacccca
1-4tRNA-Ser(tga)c[64083,64172]
agcgggttggccgagaggcttatggcgctggtcttgaaaaccagaggacg
ggaaaccgttccgtgagttcgaatctcacacccgctgcca
1-5tRNA-Pro(tgg)c[64380,64456]
ctgtacgtagcgcagtctggtagcgtaggtgctttggatgcatcgggtcg
gaggttcgaatcctcccgtacagacca
1-6tRNA-Asn(gtt)c[64462,64536]
tgacctatcgtataaaggctattacggtggaccgttaatccacttatcct
ggttcgagtccaggtgggtcagcca
1-7tRNA-Lys(ttt)c[65250,65323]
gcgtcgttagctcagctggttagagcatccgacttttaatcggaatgtcg
gtagttcgaatctaccacgacgca
1-8tRNA-Trp(cca)c[65619,65692]
aggtcggtagttcaatggtagaatagcggattccaaatccgaagacgaga
gttcgactctttctcggcctgcca
1-9tRNA-Arg(tct)c[66403,66479]
gcactcttagctcagctggatagagcaacggccttctaagccgtaggtca
caggttcgaatcctgtagggtgtgcca
1-10tRNA-Leu(taa)c[66487,66571]
gtctgagtggtggaactggtatacacaggagatttaaaatctcccgcctt
atggattgtgggttcaaatcccaccttggacacca

1−11tRNA−Met(cat)c[66763,66835]
ggccctttagcttactggttgaaagctgacgactcataatcgttagagca
tggttcgattccatgaagggcca
>KX130863.1 Shigella phage SHSML−45, complete genome
1−1tRNA−Met(cat)c[55871,55946]
agttagttggcagagtggttatgcacctccttcatacggagcgactacag
tggttcaaatccactactaactacca
1−2tRNA−Ile(gat)c[56043,56119]
gcttcggtagcttagcgatctaaagcactcggctgataaccgagagatcg
ggggtttaaatccctcccggagtacca
1−3tRNA−Asp(gtc)c[57205,57281]
gcgaccggggctggcttggtaatggtactcccctgtcacgggagggaatg
tgggttcaaatcccatcggtcgcgcca
1−4tRNA−Asn(gtt)c[57579,57661]
gggtcgttagccaagcggtttggcggcggattgttaatccgtgtcgaaag
acaacgtaggttcgaatcctacacggcccgcca
1−5tRNA−Cys(gca)c[57814,57889]
cgaccgttggctgaatggcttaggcgaaggattgcaaatcctttttatgt
gagttcaaatctcatgcggtcgtcca
1−6tRNA−Tyr(gta)c[58407,58490]
gcatcgttggcagaatgtctattgcagcggtctgtaaatccgtgcccttc
ggggttggtggttcaaatccatcacgatgcacca
1−7tRNA−Met(cat)c[59367,59441]
gggtcggtagcttaaagttaaagcggtggcctcataagctaacgagtagg
agttagattctcctccgacccacca
1−8tRNA−Ser(gct)c[59448,59536]
ggaagaatagcataacggtattgcagcagattgctaatctgtcggtttga
aatatagccttgtgggttcgattccccttcttccgcca
1−9tRNA−Arg(tct)c[61548,61622]
cggggtgtagtctaagggagaggcaggagtcttctaaattcctttatgca
ggttcgaatcctgtcacctcggcca
1−10tRNA−Ser(act)c[62123,62196]
tgccgatctagtataaacggtattatcaagccgtaactggtggaagatgt
aggttcgaatcctacggtcggtaa
>KX438380.2 Salmonella phage vB_SalM_PM10, complete genome
1−1tRNA−Met(cat)[101837,101913]
ggtcctgtagctcagtggtaagtagcagtgaactcataattcattggtcg
ttggttcaaatccaaccaggatcacca
1−2tRNA−Asn(gtt)[102153,102228]
gacgatgtagttcagtcggtagaacggcggtctgttaaaccgtatgtcgc
aggttcaagtcctgccatcgtcgcca
1−3tRNA−Tyr(gta)[103283,103366]
gtgagtgtggcagagcggtcgaatgcaggagactgtaaatctccccgtaa
cagcgcggtggttcaaatccatccactcacacca

1-4tRNA-Ser(gct)[103443,103531]
ggaaggttgcccgagaggtttaagggactcgactgctaatcgagtggggc
ttttagcccccgaaggttcgaatccttcaccttccgcca
>KX231828.1 Enterobacter phage Arya, complete genome
1-1tRNA-Arg(tct)c[25273,25349]
ggccccatagctcaaccggatagagcgacagccttctaagctgtaggttc
caggttcgattcctggtggggtcgcca
>KX377933.1 Escherichia phage vB_EcoM_Alf5, complete genome
1-1tRNA-Pro(tgg)[24208,24284]
ctctgtttagctcagcttggtagagcgttccgtttggggcggtaaggccg
gaggttcaagtcctccaacagagacca
1-2tRNA-Glu(ttc)[24292,24369]
gttccagtagacaaaatggtaaagtcaccactctttcaaagtggatattt
gagggttcaaatcccttctggaacgcca
1-3tRNA-Met(cat)[24460,24536]
tgcgggtatagagaaagggcgtctcacatgtctcattagcatggtatcgg
caggttcgactcctgcacccgcctcca
1-4tRNA-Asn(gtt)[24620,24696]
ggttaggaagcacataaggtatgtgcggtcgcctgttaagcgaatggcac
agggttcgaatccctgactaaccgcca
1-5tRNA-Tyr(gta)[24766,24853]
gtgtcgttatcccgtagatggtagcggtggggactgtaaatcccttgtca
ttgagactcggtaggttcgactcctacacggcacacca
1-6tRNA-Asp(gtc)[24859,24936]
ggttatgtagtttaatttggttaaaatactcccctgtcacgggagatgat
gagggttcaaatcccttcgtagccgcca
1-7tRNA-Lys(ttt)[25365,25440]
ggaagtgtagcagaatggtgatgcggcagacttttaatctgacaggcgat
gggttcgaatccctccacttctacca
1-8tRNA-Met(cat)[25445,25521]
ggttcagtcgcagataaggtaatgcaagggtctcataagccctatgaatg
tgggttcgattcccatctgaacctcca
1-9tRNA-Ile(gat)[25523,25598]
gctggtatagttaagaaggttataacactcccctgataagggaacatcgg
tggttcgattccacctaccagtacca
1-10tRNA-Ser(tga)[26524,26613]
ggtaggtagcggctaatggtagccaaactgtcttgaaaacagttgccact
gtagagatacggtgagggttcgactcctttacttaccgcc
1-11tRNA-Leu(tag)[26869,26946]
gggagattgacggtaattggtaaacctaccatccttagaagttggtgttt
gagggttcgaatcccttgtctcctacca
1-12tRNA-Lys(ctt)[26954,27029]
gcaggtgtagcaaaatggttatgcggctgactcttaatcagtaagacgac
gggttcaattccctccacctgtacca

1-13tRNA-Ala(tgc)[27035,27110]
ggggtcatagtttatatggttaaaattcgagttttgcaaacttgggaact
gagttcaattctcagtgactccacca
1-14tRNA-Gly(tcc)[27117,27191]
gcatccatagtttaaacgggaaaattacagtcttccaaactgaggttgag
ggttcgattccctctggatgctcca
1-15tRNA-Thr(tgt)[27199,27274]
gctcctatcgtataatcggctattacggttgccttgtaagcaacttatca
gggttcgaatccttgtgggagcacca
1-16tRNA-Val(tac)[27370,27444]
actcgcttagtttatatggtaaaacatcacccttacaagatgaagaaaaa
ggttcaagtcctttagtgagtacca
1-17tRNA-Leu(caa)[27446,27523]
gttccagtatcccaattggcagaggatgcaagctcaaaccttgtattagt
gacggttcgaatccgtcttggaacacca
1-18tRNA-Gln(ttg)[28108,28183]
aggggattagtttacaaggttaaaacctcggtctttgaaatcgaagaagt
tggttcaattccaacatcccccgcca
1-19tRNA-Leu(taa)[28186,28264]
gctccattactccaattggcagagaggccagacttaaaatctgtgttatg
tatcggttcgaatccgatatggagtacca
1-20tRNA-Gln(ctg)[28270,28345]
agcggtatagcataactggcaatgcagcagtctctgaagctgtcctatta
aggttcaaatccttatgccgctgcca
1-21tRNA-His(gtg)[28377,28452]
gtggccttatcataaatggtaatgacccatgctgtgaacatggtctatac
gggttcgactcccgtaggtcacccca
1-22tRNA-Phe(gaa)[28459,28534]
agtccaagtagcttatatggttaaagcgcgtgtctgaaaaacatgagaag
agggttcaaatcccactggactacca
1-23tRNA-Ser(gct)[29287,29379]
ggaagattaaccctaaaaggtaagggagcagtttgctaaactgccagtag
ctgagaaatcggtgtaccagttcaagtctggtatcttcctcca
1-24tRNA-Cys(gca)[29384,29459]
gaatccgtgacagaaatggatatgtgcctgtctgcaaaacaggtttataa
gggttcaagtcccttcggattctcca
>KU737348.1 Bacillus phage Zuko, complete genome
1-1tRNA-Asn(gtt)[33777,33850]
gtgcttgtagctcagtcggtagagctggtggctgttaaccactgtgtcgt
aggttcgattcctacctagcacgc
1-2tRNA-Glu(ttc)[33857,33933]
gtcgcattggtgaaattggctaacacactcggctttctaccgaggattta
ggggttcgagccccctatgcgattcca
1-3tRNA-Asp(gtc)[33938,34011]

tggggatatagtgtagaggtaaacacgcacggctgtctaccgtgaagcac
gagttcgaatctcgttatcctcgt
>KR233164.1 Salmonella phage NR01, complete genome
1-1tRNA-Ser(gga)c[108,201]
ggaaaagcaaatagattggtgactaaacccgattggaaatcggttgagtg
gtagcaataccgccttatgggttcaactcccatcttttccgcca
1-2tRNA-Leu(tag)c[208,292]
gggttagtggtgtaatgggtagccacgagggtcttagaagcccttgctta
agtgcgtgagagttcgagtctctcctaacccacca
1-3tRNA-Ala(tgc)c[299,373]
gggcgaatagtgtcagcgggagcacaccagacttgcaatctggtagggag
ggttcgagtccctctttgtccacca
1-4tRNA-Pro(tgg)c[1124,1201]
cagggtaagggtgataaggtacgccacctcgtttggggcgaggaaatctt
tagagttcgaatctctataccctgacca
1-5tRNA-Asp(gtc)c[1583,1657]
ggtactatagtctaacggttaggatactcccctgtcacgggagagatagg
gtttcgattacccttagtaccccca
1-6tRNA-Asn(gtt)c[1956,2038]
gggtcgttagccaagcggtttggcggcggattgttaatccgtgtcgaaag
acaacgtaggttcgaatcctacacggcccgcca
1-7tRNA-Met(cat)c[2633,2707]
gggtcggtagcttaaagttaaagcggtggcctcataagctaatgagtagg
agttagattctcctccgacccacca
1-8tRNA-Ser(gct)c[2714,2807]
ggaagattaaccctaaaggtaagggagctgtttgctaaacagccagtaac
tgtggaaacacggtgtgccagttcgagtctggcatcttcctcca
1-9tRNA-Arg(tct)c[6350,6426]
gctccgttggccaaattggataaggcaagatcgttctaagttcttgatta
ggggttcgaatcccttacggagtacca
>KX258185.1 Klebsiella phage vB_KpnM_KpV477, complete genome
1-1tRNA-Thr(tgt)c[67989,68064]
gccgatttagctcagttggtagagcgcttcacttgtaatgaagatgccgc
gggttcgactcctgcaatcggcacca
1-2tRNA-Arg(tct)c[68608,68683]
gcccttatagtgtaatggatagcacacgatcgttctaaggtcggtagtcc
gggttcgagtcctggtgggggtacca
1-3tRNA-Leu(taa)c[68686,68772]
gcgtcgatgttggaattggtagacaaaggagacttaaaatctcccgggat
taaacccgtacgagttcgagtctcgttcgacgcacca
1-4tRNA-Met(cat)c[69948,70024]
ggccctgtagctcaattggtagagcgttcccctcataagggattggttgc
atgttcgagtctttgccagggtcacca
1-5tRNA-Pro(tgg)c[71292,71368]

ctccgtatagctcagtctggtagagcgctccatttgggatggagaggtcg
aatgttcgagtcattctatggagacca
1-6tRNA-Gly(tcc)c[71374,71448]
gcatccatcgtataacggctattataggtgacttccactcacttgataag
ggttcgattccctttggatgctcca
1-7tRNA-Trp(cca)c[71454,71528]
aggttcttagtataacggctattatgctgggctccaaacccagtgatgag
ggttcgattccttcagggcctgcca
1-8tRNA-Ile(gat)c[71535,71610]
gggagtatagctcatttggtagagctctcgaccgataatcgagcggtgac
tggttcgagtccagttactcccacca
1-9tRNA-Ser(tga)c[71677,71761]
ggagagtagcgctagtggtagcaaaccggacttgaaatccgggccaccgg
aaacggtgagggttcaactcctttactctccgcca
1-10tRNA-His(gtg)c[71859,71934]
gtggccgtagttcagttggtagaactcgagattgtgattctcgtagtcat
gggttcaactcccatcggtcacccca
1-11tRNA-Gln(ttg)c[72031,72105]
tggactatagacaagcggttaagtcaccggcctttgactccggtatctct
ggttcgaatccagatagtccagcca
1-12tRNA-Met(cat)c[72111,72187]
tgcggggtaacttcagttggtagaatgttgggctcatatcccgacacgcg
caggttcgagtcctgcctccgcctcca
1-13tRNA-Asp(gtc)c[72349,72425]
ggacctatagtttagttggataaaatactcccctgtcacgggagagagcg
cgagttcgatcctcgctaggtccgcca
1-14tRNA-Asn(gtt)c[72656,72740]
gggtcgttggctgagagggtaagcgacggactgttaatccgtgtcagaaa
tgactaggcaggttcgatacctgcacggcccgcca
1-15tRNA-Lys(ttt)c[72747,72824]
agaacgttagctcatctggttagagcactcgacttttaatcgagatgtcg
atgggttcaaatccctcacgttctacca
1-16tRNA-Tyr(gta)c[73303,73389]
ggggcgttagaccgtaggggtagcgggacagactgtaaatctgttgctca
aaaggctcgggtggttcgactccatcactccccacca
>KX130864.1 Shigella phage SHBML-50-1, complete genome
1-1tRNA-Arg(tct)c[70296,70371]
cggggcatagctcagaaggaagagcaaggaccttctaagtcctaggtcgt
aggttcgatccctactgcctcgacca
1-2tRNA-His(gtg)c[70376,70451]
gtggccgtagttcagttggtagaactcgagattgtgattctcgtagtcat
gggttcgactcccatcggtcacccca
1-3tRNA-Asn(gtt)c[70566,70640]
ggatgtgtagctcagtggtagagcagttgactgttaatcaattggtccat

ggttcgaatccatgcatgtccgcca
1–4tRNA–Tyr(gta)c[70645,70731]
ggggagttatcccgtagaggtagcggtgtggactgtaaatccattgtcat
tgcgactcgggtggttcgactccatcactccccacca
1–5tRNA–Met(cat)c[70744,70818]
ggccctgtagctggaaggttcaagcaagcgactcataatcgccagatggt
ggttcaattccacccagggccacca
1–6tRNA–Thr(tgt)c[70820,70895]
gctgatttagctcagtaggtagagcaactcacttgtaatgagaaggtcgg
cggttcgattccgtcaatcagcacca
1–7tRNA–Ser(tga)c[70902,70991]
ggaggcgtggcagagtggtttaatgcaccggtcttgaaaaccggcagtcg
ctccggcgactcataggttcaaatcctatcgcctccgcca
1–8tRNA–Pro(tgg)c[70993,71067]
ctccgtgtagctcagtttggtagagcgcctgatttgggatcaggaggtcc
aaggttcaaatccttgtatggagac
1–9tRNA–Gly(tcc)c[71078,71151]
gcggatatcgtataatggcattacctcagacttccaatctgatgatgtga
gttcgattctcattatccgctcca
1–10tRNA–Leu(taa)c[71157,71243]
gcgagaatggtcaaattggtaaaggcacagcacttaaaatgctgcggaat
gatttccttgtgggttcgagtcccacttctcgcacca
1–11tRNA–Gln(ttg)c[71244,71317]
tgggaattagccaagttggtaaggcactggattttgattccaggatgcaa
aggttcgagtcctttattcccagc

>KU577463.1 Bacillus phage Deep Blue, complete genome

1–1tRNA–Met(cat)c[140342,140416]
taggattatagctcagtggtagagcgtgggtctcataagcccgaggtcga
tggttcgaccccatctattcctatc
1–2tRNA–Trp(cca)c[140420,140493]
gggggtttagtttagtggaaaaatgacaggctccaaacctgtagtccgag
gttcgattccttgcgcccttgcca
1–3tRNA–Leu(tag)c[140500,140575]
gccgaggtaatccaacggaagagatagtggtcttagaagccatacagtga
gggttcgaatccctctctcggtacca
1–4tRNA–Ile(gat)c[140584,140660]
actagtgtagctcagttaggtagagcagtgtcttgataaggcattggtca
caggttcaaatcctgtcactagtacca
1–5tRNA–Leu(taa)c[140662,140748]
atcggagtgttggaattggtagacatacgggacttaaaatcccgtgccat
tgagtgcgtgagggttcgagtccctcctccgatacca
1–6tRNA–Tyr(gta)c[140756,140837]
gggcgtgctatccttggagagataagctggctgtaaaccagtggcgtaag
ctgagaaggttcgaatccttccattcccacca

1-7tRNA-Phe(gaa)c[140844,140918]
ggacaggtagctgagacggattagcgactggttgaaacccattagagagt
ggatcgttaccactcctgtccacca
1-8tRNA-Pro(tgg)c[141014,141088]
tgtggatgtaggctagttggtcagtcacctcgtttggggcgagggtcacg
cacgttcgaatcgtgtcatccgcat
1-9tRNA-His(gtg)c[141191,141261]
cagtgtgtggcgtagtggtaacgcagtggactgtgaatccatgaacgaga
gttcgattctctccatactga
1-10tRNA-Gln(ttg)c[141339,141415]
tttcggagtggacaaactggtaaagtcgtcaggctttgaccctgaagtgt
ggtggttcgatcccatcctccgaagtc
1-11tRNA-Ser(tga)c[141494,141581]
cggggaatactcaagtgggtaaaagaggatggtcttgaaaaccattaggc
gtgaaagcgtgcgggggttcgaatccctcttcctcggc
1-12tRNA-Arg(tct)c[141672,141747]
taccccttagccaagtggactaaggcaacgggcttctatcccgtggatc
gtgcgttcgaatcgtacagggggtgt
1-13tRNA-Glu(ttc)c[141841,141916]
tgtcgcattggtgaaactggctaacacactcggctttctaccgagcattc
acgagttcgaatctcgtatgcgatat
1-14tRNA-Thr(tgt)c[142030,142104]
gccttcttagctcaggtggctagagcgactgccttgtaagcagtaggtcg
tgggttcgaatcctacagtcggcac
1-15tRNA-Asp(gtc)c[142110,142183]
tggggatatagtgtagcggtgaacacacttgactgtctatcaagtagcac
gggttcaagtcccgttatcctcgt
1-16tRNA-Gly(tcc)c[142272,142346]
ggggcattggtatattggctattattcttggcttccaaccaagcgaggtc
ggttcgattccgacatgtccctcca
1-17tRNA-Ser(gct)c[142532,142622]
ggaaggttgtcagagcggtttattgttcctgtttgctaaacaggtgtacg
tccttaacgtaccacaggttcaaatcctgtaccttccacca
1-18tRNA-Asn(gtt)c[142627,142701]
gtacttgtagctcagaggtagagcagcagattgttaatctgaaggtcggt
ggttcgatcccaccctggtacgcca
1-19tRNA-Cys(gca)c[142729,142802]
gaaggtgtaccgaagtggctcaacggcacagattgcaaccctgttgttcg
ggggttcgaatccctccactttct
>KX397368.1 Erwinia phage vB_EamM_Huxley, complete genome
1-1tRNA-Trp(cca)[883,959]
gggggagtagttcaattggcgagagcgtcggattccaaatccggttgttg
caggttcgagtcctgtctcccctgcca
1-2tRNA-Cys(gca)[1281,1354]

ggggcgctggccgagtggttaggcgctggattgcaaatcctgttaccgcg
gttcgattccgcggcgtccctcca
1-3tRNA-Tyr(gta)[4117,4202]
ggcgggttactcaagcggtcaacgagagcagactgtaaatctgttgcctt
cgggcttcgtaggttcaaatccttcacacgccacca
1-4tRNA-Asp(gtc)[4504,4579]
ggtgctatcgtctagtggccgaggatactaccctgtcacggtagagaccg
gagttcgattctccgtagcaccgcca
1-5tRNA-Ile(gat)[4588,4663]
tgcgtcttagctcagttggtagagcgcacccctgataagggtgaggccac
tggttcaagtccagtagatgcaacca
1-6tRNA-Glu(ttc)[4671,4747]
attcccgtcatctagttggtccaggatacgacactttcactgtcggtaca
cgagttcaaatctcgtcgggaatacca
1-7tRNA-Ser(gct)[4753,4843]
agagaattgactgagttggtttaaggtgctcccctgctaagggagtgtat
gcgaaagtgtaccattggttcgaatccaatattctctgcca
1-8tRNA-Ile(aat)[5308,5401]
tggctccgtagctaaatcggtagaggcagactttaattagttctccaacg
attttataatcgcgtggtgtaggttcaagtcctaccggagcaag
1-9tRNA-Asn(gtt)[5485,5560]
gactctgtagttcagttggtagaacggtggactgttaatccatatgtcgc
aggttcgagtcctgccagagtcgcca
>KR269718.1 Escherichia phage HY03, complete genome
1-1tRNA-Arg(tct)c[7,83]
cggggcatagctcaattgtatagagcaacggacttctaatccgtaggttg
aaggttagaatccttctgcctcgacca
1-2tRNA-Met(cat)c[794,865]
ggccctgtagctggaaggttcaagcaagcgactcataatcgccagatggt
ggttcaattccacccagggcca
1-3tRNA-Thr(tgt)c[872,944]
gctgatttagctcagtaggtagagcaactcacttgtaatgaggacgtcgg
cggttcgattccgtcaatcagca
1-4tRNA-Ser(tga)c[951,1039]
tggaggcgtggcagagtggtttaatgcaccggtcttgaaaaccggcagtc
gctccggcgactcataggttcaaatcctatcgcctccgt
1-5tRNA-Pro(tgg)c[1040,1114]
ctccgtgtagctcagtttggtagagcgtctgctttgggagcagaatgtcg
caggttcaaatcctgccgcggagac
1-6tRNA-Gly(tcc)c[1127,1198]
gcggatatcgtataatggtattacctcagacttccaatctgatgatgtga
gttcgattctcattatccgctc
1-7tRNA-Leu(taa)c[1205,1291]
gcgagaatggtcaaattggtaaaggcacagcacttaaaatgctgcggaat

gatttccttgtgggttcgagtcccacttctcgtacca
>KU867307.1 Salmonella phage vB_SnwM_CGG4-1, complete genome
1-1tRNA-Arg(tct)c[57710,57785]
cgaggcatagctcagaaggaagagcaaggaccttctaagtcctaggtcgt
aggttcgatccctactgtctcgacca
1-2tRNA-Gln(ttg)c[58425,58500]
tgggaattagccaagttggtaaggcaccggattttgattccgggatgcac
tggttcgagtccagtattcccagcca
1-3tRNA-Met(cat)c[58507,58581]
ggcccctttagctcaatgggagagctgtcagctcataactgataggtagct
ggatcgaaaccagcaagggccacca
>KX130862.1 Shigella phage SHFML-26, complete genome
1-1tRNA-Arg(tct)c[133433,133508]
cggggcatagctcagaaggaagagcaaggaccttctaagtcctgggtcgt
aggttcgatccctactgcctcgacca
1-2tRNA-Met(cat)c[134219,134290]
ggccctgtagctggaaggttcaagcaagcgactcataatcgccagatggt
ggttcaattccacccagggcca
1-3tRNA-Thr(tgt)c[134297,134369]
gctgatttagctcagtaggtagagcaactcacttgtaatgagaaggtcgg
cggttcgattccgtcaatcagca
1-4tRNA-Ser(tga)c[134376,134464]
tggaggcgtggcagagtggtttaatgcaccggtcttgaaaaccggcagtc
gctccggcgactcataggttcaaatcctatcgcctccgt
1-5tRNA-Pro(tgg)c[134465,134539]
cttcgtgtagctcagtttggtagagcgtctgctttgggagcagaatgtcg
caggttcaaatcctgccgcggagac
1-6tRNA-Gly(tcc)c[134552,134623]
gcggatatcgtataatggtattacctcagacttccaatctgatgatgtga
gttcgattctcattatccgctc
1-7tRNA-Leu(taa)c[134630,134716]
gcgagaatggtcaaattggtaaaggcacagcacttaaaatgctgcggaat
gatttccttgtgggttcgagtcccacttctcgtacca
1-8tRNA-Gln(ttg)c[134717,134790]
tgggaattagccaagttggtaaggcactggattttgattccaggatgcaa
aggttcgagtcctttattcccagc
>KX752698.1 Mycobacterium phage Tonenili, complete genome
1-1tRNA-Ser(gct)[35875,35958]
ggagggtgagcatcaggtgatgcagcgagattgctaatcccgtacggtaa
ccaccccgtgaggttcgaatcctcctccctccgc
1-2tRNA-Leu(cag)[36151,36226]
gctcccgtagcccaattggcaggaggcaccagattcaggatctgggcagt
gtgagttcgaatctcaccgggagtac
1-3tRNA-Leu(gag)[36346,36420]

gtctctgtaggcaaaccggaaaagccgccatcttgagggggtggtgcgtg
cgggttcgactcccgccagagacac
1-4tRNA-Leu(caa)[36421,36494]
gccgtggtaggccatctggcgagccgccgaattcaagtttcggtgtttgc
gggttcgaatcccgcccacggtac
1-5tRNA-Pro(tgg)[95715,95789]
cggggtgtagttcagtttggaagagcgcttggtttgggaccaagatgtcg
caggttcgaatcctgtcaccccgac
1-6tRNA-Trp(cca)[95806,95881]
tggggtgaagccgatccggaaggcagcggtctccaaagccgtctcatagc
gggttcgaatcccgtcacccctgcca
1-7tRNA-Tyr(gta)[95883,95968]
gccgcacatgcccaactggtgttgggagcagactgtaaatctgtggccct
agggacggtgaggttcgattcctcagtgcggtacca
1-8tRNA-Pyl(cta)[97314,97385]
gcaccatttgctcaatggcagagcggcggcttctaaccccgtgagtgccg
gttcgattccggcatggtgcac
1-9tRNA-Met(cat)[97536,97610]
agcggtgtagagcagctaggtagctcgccgggctcataacccggaggacg
cgtgttcgaatcacgccaccgccac
1-10tRNA-Cys(gca)[97824,97895]
gcgcctttggcggaatggctacgtgctcggctgcaacccgagttatcccg
gttcgactccgggagggcgctc
1-11tRNA-Glu(ctc)[97900,97974]
ggtccgttggagtagatggatatctcgccaccctctcaaggtggagatca
cgggttcaagtcccgtacggactgc
1-12tRNA-His(gtg)[97976,98048]
gtggccgtagttcagcggtagaacaccgggttgtgatcccggacgccgtc
ggttcgaatccgaccggtcaccc
1-13tRNA-Ala(tgc)[98208,98281]
gggcctatagctcatctggtagagcgcctgccttgcaagcaggaggcggc
aggttcaagtcctgttaggtccac
1-14tRNA-Phe(gaa)[98471,98543]
gccgtcatagctcagttggtagagcactggcctgaaaacccagtggccga
ggttcgattcctcgtgtcggcac
1-15tRNA-Val(cac)[98626,98699]
gtccgtttagctcagctggaagagcgctcggtccacacccgagaggccgc
aggttcgatccctgcaatggacac
1-16tRNA-Lys(ctt)[98821,98893]
gccttcgtagctcagtggtagagctgccgcctcttaagcggtaggtcgtt
ggttcgaatccagccgggggcac
1-17tRNA-Glu(ttc)[98897,98972]
ggtcgggtggtctgttggcaggccggtcgggttttcaccccggcgtcatc
gcgggttcgattcccgtcccgactgc

1-18tRNA-Gly(tcc)[99102,99174]
gcgggtgtggccgaatggctcaggcaccagacttccactctggctaagca
ggttcgattcctgtcatccgctc
1-19tRNA-Thr(cgt)[99234,99307]
gctgctgtagctcacctggcagagcgtcggcgtcgtatcccgaaggcatc
cggttcgagtccggacagcagctc
1-20tRNA-Thr(tgt)[99316,99389]
gcccttgtagctcagtggtagagcaccgaccttgtaagtcgggttgtccc
gggttcgattcctggttggggccc
1-21tRNA-Thr(ggt)[99449,99521]
gctgatttagctcagtggtagagcgccgacttggtatgtcggaggccccg
ggttcgatccccggattcagctc
1-22tRNA-Gly(gcc)[100844,100917]
gcgaaggtagctcagctggtagagcgccaccttgccaaggtggaggtcgc
gggatcgtaacccgttcttcgctc
1-23tRNA-Asp(gtc)[100921,100996]
ggccctgtggagcagctaggagtgctcgccaccctgtcacggtggaggtc
gacggttcaaatccgttcagggtcgc
1-24tRNA-Met(cat)[101083,101155]
gcctcactagctcattggtagagccgctcgctcataacgtgcaggtacct
ggttcgattccagggtgaggtac
1-25tRNA-Ile(gat)[101160,101236]
gcctgttagcggactggtcgtccgatccaagctgataacttggcgtaagc
ggtgttcgattcaccgagcaggtacca
1-26tRNA-Arg(acg)[101298,101370]
gcctctatggtccaacggagatgacgccggtctacggaaccggagatgcg
tgttcgactcgcgctaggggcac
1-27tRNA-Val(gac)[101413,101485]
gtccgtgtagctcaggggtagagcgcctgctcgacacgcaggaggaccga
ggttcgaaacctcgcatggacac
1-28tRNA-Arg(cct)[101657,101730]
gcctctgtagctcaacggacagagcaacgcggtcctaacgcggtggctgg
aggttcgaatcctctcggaggcac
1-30tRNA-Gln(ttg)[102271,102346]
tggggtatggtggcaatctggcagtcccgccggactttgactccggaggt
gcaggttcgagtcctgctaccccatc
1-31tRNA-Arg(tct)[102350,102425]
gcccttgtagctcagtggacagagcggcgagcttctacctcgcgggccgg
gagttcgaatctctccaggggcacca
1-32tRNA-Gln(ctg)[131096,131170]
tgctcgttggtgtaactggcaacactacggactctgactccgtcattctt
ggttcgaatccaggacgagcaacca
1-33tRNA-Asn(gtt)[131177,131253]
tggggtgtagttcaatctggcagaacgctcgactgttaatcgagtagttg

aaggttcgagtccttccatcccagcca
>KX130865.1 Shigella phage SHSML-52-1, complete genome
1-1tRNA-Met(cat)[130607,130681]
ggccctgtagctcaatgggagagctgtcagctcataactgataggtagct
ggatcgaaaccagccagggtcacca
1-2tRNA-Arg(tct)[130685,130761]
cgaggcatagctcaattgtatagagcaacggacttctaatccgtaggttg
aaggttagaatccttctgtctcgacca
>KU594606.1 Cyanophage S-RIM32 isolate RW_108_0702, complete genome
1-1tRNA-Leu(taa)[17872,17956]
tgggaccatgatggaattggtagacattgcggacttaaaatccgttggca
ttacgccgtgcgagttcaagtctcgctggtcctat
1-2tRNA-Asp(gtc)[19287,19362]
ggggtagtagctcagttggttagagtacctgcctgtcacgcagggtgccg
tgggttcaagtcccatctatcccgcc
1-3tRNA-Thr(tgt)[19365,19438]
gcctccgtagctcagctggatagagcaacggttttgtaaaccgtaggtcg
tcggttcaagtccgaccgtgggct
1-4tRNA-Leu(caa)[19491,19563]
catctggtagtctattggtaaggacgggtggacaacacacatggaaactg
ggttcgattcctagacagatgac
1-5tRNA-Ala(tgc)[19649,19721]
ggggaattagctcagttggtagagcacctgctttgcaagcaggctgtcag
gagttcgagtctcctattctcca
1-6tRNA-Asn(gtt)[19749,19822]
tcctctatagctcagtcggtagagcgagtgactgttaatcactatgtccc
tggttcgagcccaggtggaggagc
1-7tRNA-Leu(tag)[19826,19907]
gtcgttgtggcggaattggtagacgcgctgggtttaggttccagtgaagt
aattcgtggaggttcaagtcctctcagcgaca
1-8tRNA-Ser(tga)[146887,146975]
tggaggggcaatccgattggtgacggaacctgtcttgaaaacagttgagg
tgttaaagcccttgggagttcgactctccctccctccgt
1-9tRNA-Arg(tct)[165780,165855]
gggtcagtagctcagttggatagagcaactgccttctaagcagtcggtca
caggttcgagtcctgtctgacccgcc
1-10tRNA-Val(tac)[165857,165930]
tgcccgcttaactcagcggtagagtgtctcctttacacggagggtgtcgg
cggttcgaatccgtcagcgggcat
1-11tRNA-Pro(tgg)[167482,167557]
tcggggcgtagctcagtttggtagagcactcgctttgggagcgagtggcc
gtaggttcaaatcctatcgccccgat
1-12tRNA-Gly(tcc)[169948,170019]
gcgggtgtagttcagtggtagaacgctatccttccaagttagatgtcgtc

ggttcgaatccgatctcccgct
>KU594605.1 Cyanophage S-RIM50 isolate RW_29_0704, complete genome
1-1tRNA-Gly(tcc)[13531,13605]
gcggatgtagttcagtcaggtagaacgctatccttccaagttagatgtcg
tcggttcgattccgatcatccgctc
1-2tRNA-Val(tac)[13607,13680]
tgggcgattaactcagcggtagagtgcctcctttacacggagtaggtcgg
gggttcgaatccctcatcgcccat
1-3tRNA-Leu(taa)[17898,17985]
tgggacggtggcggaatcggtagacgcatcggacttaaaatccgctgagg
attaaccctcgtgagagttcaagtctctctcgtcctat
1-4tRNA-Thr(tgt)[18058,18131]
gccactatagctcagctggatagagcaacggttttgtaaaccgtaggtcg
tcggttcaagtccgacttgtggct
1-5tRNA-Ala(tgc)[18249,18321]
gggggattagctcagttggtagagcacctgctttgcaagcaggctgtcag
gagttcgagtctcctattctcca
1-6tRNA-Asn(gtt)[18348,18422]
ttcctctatagctcagtcggtagagcgtctgactgttaatcagaatgtcc
ctggttcgagcccaggtggaggagt
1-7tRNA-Arg(tct)[159822,159897]
tgggtcagtagctcagttggatagagcaactgccttctaagcagtcggtc
acaggttcgagtcctgtctgacccgt
1-8tRNA-Pro(tgg)[160906,160978]
cggggtgtagcgcagaggtagcgcggctgttttgggaacagcaggtcgca
ggttcgatccctgtcaccccgac
>KJ628499.1 Acinetobacter phage vB_AbaM_phiAbaA1, complete genome
1-1tRNA-Arg(tct)[7851,7927]
acgccattagctcagatggatagagcatctgccttctaagtagatggtca
cgggttcgaatcctgtatggcgtacca
1-2tRNA-Tyr(gta)[8226,8311]
gtatgtgtgacccgaattggtaagggagcggactgtaactccgttgtcaa
aagcaattgtaggttcaagtcctaccacatacacca
1-3tRNA-Ile(gat)[8318,8392]
ggtcgattagctcagcggtagagcagtagcccgataagctattggtcgct
ggttcaaatccagcatcgactacca
1-4tRNA-Pro(tgg)[8980,9056]
cggtgtgtttcgagaaagggtaatcgactgcctttggagcgcagaatatg
taggttcgagtcctaccgcaccgacca
1-5tRNA-Asp(gtc)[9262,9337]
gactcattagctcaatcgggagagcgccacactgtcactgtggaggtaag
gagatcgaaactcctatgggtcgcca
1-6tRNA-Thr(tgt)[9608,9681]
gctggtatcgtatagtggtattatgctggtcttgtaatccagtcacggtg

gttcgattcctcctaccagcacca
1-7tRNA-Trp(cca)[10009,10079]
gggttgaccgctgggcggtgacgaggctccaaaccttgtcttgctacgtt
cgattcgtagtcgatctgcca
1-8tRNA-Gln(ttg)[10201,10276]
tgggacgtagtgtagttggttagcacatgagactttgactctcatagatt
cagttcgaacctgaacgtcccttcca
1-9tRNA-His(gtg)[10373,10448]
gtgatcatggtgtaattggctagcaccgcgatttgtgatgtcgttagtct
gagttcgagcctcagtgatcacacca
1-10tRNA-Gly(tcc)[10451,10524]
gcgtgattggtataatggtattatgtctggcttccacccagaagacgaga
gttcgattctctcatccagcgcca
1-11tRNA-Glu(ttc)[10531,10605]
gttccattcgactagcggttaggtcgcttccctttcaaggaagtagcacg
agttcaaatctcgtatggaatacca
1-12tRNA-Cys(gca)[10933,11007]
ataccataatctaaagggacgaagtgctggcttgcaaacccagaatagtg
agttcgagtctcacatggtattcca
1-13tRNA-Leu(tag)[11022,11099]
gtacaggtgacgggaactggcatacctatcacgcttagaacgtggtgctt
gtgggttcgaagcccaccctgtatacca
>KX405003.1 Salmonella phage BPS15Q2, complete genome
1-1tRNA-Pro(tgg)[10996,11072]
ctctgtttagctcagcttggtagagcgttccgtttggggcggtaaggccg
gaggttcaagtcctccaacagagacca
1-2tRNA-Glu(ttc)[11080,11157]
gttccagtagacaaaatggtatagtcaccactctttcaaagtggatattt
gagggttcaaatcccttctggaacgcca
1-3tRNA-Met(cat)[11248,11324]
tgcgggtatagagaaagggcgtctcacatgtctcattagcatggtatcgg
caggttcgactcctgcacccgcctcca
1-4tRNA-Asn(gtt)[11403,11479]
ggttaggaagcacataaggtatgtgcggtcgcctgttaagcgaatggcac
agggttcgaatccctgactaaccgcca
1-5tRNA-Tyr(gta)[11551,11638]
gtgtcgttatcccgtagatggtagcggtggggactgtaaatcccttgtca
ttgagactcggtaggttcgactcctacacggcacacca
1-6tRNA-Asp(gtc)[11645,11721]
ggttatgtagtttaactggataaaatactcccctgtcacgggagatgatg
tgagttcaagtctcatcataaccgcca
1-7tRNA-Lys(ttt)[12154,12229]
ggaagtgtagcagaatggtgatgcggcagacttttaatctgacaggcgat
gggttcgaatccctccacttctacca

1-8tRNA-Met(cat)[12234,12310]
ggttcagtcgcagataaggtaatgcaggggtctcataagccctatgaatg
tgggttcgattcccatctgaacctcca
1-9tRNA-Ile(gat)[12313,12387]
tgtgggttagcataaatggtaatgcaaacggctgataaccgttagaagag
ggttcgataccctcacctacaacca
1-10tRNA-Ser(tga)[13046,13135]
ggtaggtagcggctaatggtagccaaactgtcttgaaaacagttgccact
gtagagatacggtgagggttcgactcctttacttaccgcc
1-11tRNA-Leu(tag)[13391,13467]
gcacctatagcccaactggtagaggcagcagacttagaatctgctcagtg
tgagttcgaatctctctaggtgtacca
1-12tRNA-Lys(ctt)[13475,13550]
gcaggtgtagcaaaatggttatgcggctgactcttaatcagtaagacgat
gggttcaattccctccacctgtacca
1-13tRNA-Ala(tgc)[13557,13632]
ggggatgtagtttacatggttaaaacataagttttgcaaacttaagtaca
gggttcaattccctgcttctccacca
1-14tRNA-Gly(tcc)[13639,13713]
gcatccatagtttaaacgggaaaattacagtcttccaaactgaggttgag
ggttcgattccctctggatgctcca
1-15tRNA-Thr(tgt)[14142,14218]
tgctgctttcgtataattggctattacacatcccttgtaaggatggaaat
gcaggttcgaatccttgtgggagcacc
1-16tRNA-Val(tac)[14315,14389]
actcgcttagtttatatggtaaaacatcacccttacaagatgaagaagaa
ggttcaagtcctttagtgagtacca
1-17tRNA-Leu(caa)[14391,14468]
gttccagtatcccaattggcagaggatgcaagctcaaaccttgtactagt
gacggttcgaatccgtcttggaacacca
1-18tRNA-Arg(acg)[14474,14549]
gcaggattagttcaaatggatagagcaacagtctacgaagctgttaatag
gggttcgaatcccttatcctgcgcca
1-19tRNA-Gln(ttg)[15135,15210]
aggggattagtttacaaggttaaaacctcggtctttgaaatcgaagaagt
tggttcaattccaacatcccccgcca
1-20tRNA-Leu(taa)[15213,15291]
gctccattactccaattggcagagaggccagacttaaaatctgtgttatg
tatcggttcgaatccgatatggagtacca
1-21tRNA-Gln(ctg)[15297,15372]
agcggtatagcataactggcaatgcagcagtctctgaagctgtcctatta
aggttcaaatccttatgccgctgcca
1-22tRNA-His(gtg)[15404,15479]
gtggccttatcataaatggtaatgacccatgctgtgaacatggtctacac

gggttcaaatcccgtaggtcacccca
1-23tRNA-Phe(gaa)[15486,15561]
agtccaagtagcttatatggttaaagcgcgtgtctgaaaaacatgagaag
agggttcaaatcccactggactacca
1-24tRNA-Ser(gct)[16314,16406]
ggaagattaaccctaaaaggtaagggagcagtttgctaaactgccagtag
ctgataaatcggtgtaccagttcaagtctggtatcttcctcca
1-25tRNA-Cys(gca)[16411,16486]
gaatccgtgacagaaatggatatgtgcctgtctgcaaaacaggtttataa
gggttcgagtcccttcggattctcca
>KU686211.1 Synechococcus phage S-WAM2 isolate 0810PA29, complete genome
1-1tRNA-Leu(taa)[16149,16232]
gggacggtggtggaattggtagacaccccagacttaaaatctgttgagcg
tatgctcgtgcgggttcaagtcccgctcgtccta
1-2tRNA-Asp(gtc)[17704,17779]
tggggttgtagctcagtcggttagagcgcctgcctgtcacgcaggaagtc
gagggttcaagtcccttcagtcccgt
1-3tRNA-Thr(tgt)[17782,17858]
gccactttagctcagctggatagagcagggtttttgtaaagctcaggtca
cccgttcaagtcgggtaagtggctcca
1-4tRNA-Ala(tgc)[17859,17931]
ggggaattagctcagttggtagagcgcctgctttgcaagcaggatgtcag
cggttcgagtccgctattctcca
1-5tRNA-Asn(gtt)[18292,18366]
ttggggtgtagctcagcggtagtagcgggaagctgttaacttctaggtcg
caggttcgatccctgccgccccagt
1-6tRNA-Tyr(gta)[18369,18450]
gggtaggtgtccgagtggttaatggaggcggactgtaaatccgctggctc
tgcctacgggggttcaaatccctccctgccca
1-7tRNA-Arg(tct)[157420,157496]
gtctcagtagctcagctggatagagcaactgccttctaagcagtcggtcg
ctggttcgaatccagcctgagacgcca
1-8tRNA-Leu(tag)[157497,157579]
gtcggtatggcggaattggtagacgcgctgggtttaggttccagtgtcct
tgcgacgtggaggttcaagtcctcttaccgaca
1-9tRNA-Val(tac)[157581,157654]
tgcccgaatagcgcagcggtagcgcacctcctttacacggagatggtcgg
gggttcgaatccctcttcgggcat
1-10tRNA-Ser(tga)[158117,158203]
tggaaaggtggtcgagtggttgatggctctggtcttgaaaaccagcgaag
tgagagcttccgtgggttcgaatcccacccctttccgt
1-11tRNA-Pro(tgg)[158206,158280]
cggggtgtagctcagtttggtagagcgccgtctttgggaggcggatgccg
taggttcgaatcctatcaccccgac

1-12tRNA-Gly(tcc)[160808,160879]
gcggagttagttcagtggtagaacgctatccttccaagttagatgtcgtc
ggttcgaatccgatactccgct
>KU878968.1 Escherichia phage WG01, complete genome
1-1tRNA-Arg(tct)c[68899,68973]
gtcccgctggtgtaatggatagcatacgatccttctaagtttgcggtcct
ggttcgatcccagggcgggatacca
1-2tRNA-Met(cat)c[68979,69055]
ggccctgtagctcaatggttagcagcagtcccctcataagggaaaggtta
ccagttcaaatctggtctgggtcacca
>KX236333.1 Campylobacter phage PC14, complete genome
1-1tRNA-Tyr(gta)c[120868,120952]
gtagaagtaggataattggtaatccaccagactgtaaatctggcgtcttt
tggcattgatggttcaagtccatccttctacacca
1-2tRNA-Asn(gtt)c[120971,121045]
tgcagattagcacagcggtagtgcaatcgactgttaatcgatgagtcaga
ggttcgaatcctctatctgcagcca
1-3tRNA-Met(cat)c[121139,121213]
gggcgagtagctcaatggtagagcaaccggctcataaccggttggttata
ggttcgattcctgtttcgcccacca
>KU878969.1 Escherichia phage MX01, complete genome
1-1tRNA-Arg(tct)c[69836,69910]
gtcccgctggtgtaatggatagcatacgatccttctaagtttgcggtcct
ggttcgatcccagggcgggatacca
1-2tRNA-Met(cat)c[69916,69992]
ggccccgtagctcaatggttagcagcagtcccctcataagggaaaggtta
ccagttcaaatctggtccgggtcacca
>KU686210.1 Synechococcus phage S-WAM1 isolate 0810PA09, complete genome
1-1tRNA-Thr(tgt)[18964,19038]
gcctgattagctcagctggatagagcaacggttttgtaaaccgtaggtcg
tcggttcaagtccgacatcaggctc
1-2tRNA-Asn(gtt)[19044,19117]
ttcctccttagttcagcggtagaacgaacgactgttaatcgttaagtccc
tggttcgatcccaggagggggagt
1-3tRNA-Arg(tct)[165410,165485]
tggtccagtagctcagtggaaaagagcaactgccttctaagcagtcggtc
gtaggttcgagtcctacctggatcgt
1-4tRNA-Val(tac)[165488,165560]
gggagattagctcagcggtagagcacctcgtttacaccgagattgtcggg
ggttcgatcccctcatctcccac
>KX245012.1 Salmonella phage GG32, complete genome
1-1tRNA-Met(cat)[19041,19116]
ggccccgtagctcagtggttagagcagtcgactcataatcgattggtcgc
tggttcaagtccagccagggtcacca

1-2tRNA-Asn(gtt)[19529,19604]
gacgatgtagttcagtcggtagaacggcggtctgttaaatcgtatgtcgc
aggttcaagtcctgccatcgtcgcca
1-3tRNA-Ser(act)[20272,20360]
ggaaggttgcccgagaggtttaagggactcgactactaatcgagtggggc
ttttagcccccgaaggttcgaatccttcaccttccacca
>KT001918.1 Klebsiella phage Matisse, complete genome
1-1tRNA-Met(cat)[49953,50029]
ggccccttagctcaataggtagagctaatcactcataatggttaggttcc
cggttcaagtcacgggaggggccacca
>KM607000.1 Enterobacteria phage RB27, complete genome
1-1tRNA-Arg(tct)c[67633,67708]
cgaggcatagctcagaaggaagagcaaggaccttctaagtcctgggtcgt
aggttcgatccctactgcctcgacca
1-2tRNA-Asn(gtt)c[67713,67787]
ggatgtgtagctcaatggcagagcgatcgcctgttaagcgattggttata
ggttcgaatcctatcacgtccgcca
1-3tRNA-Tyr(gta)c[67792,67878]
ggggagttatcccgtagaggtagcggtgtggactgtaaatccattgtcat
tgcgactcgggtggttcgactccatcactccccacca
1-4tRNA-Met(cat)c[67888,67962]
ggccctgtagctggaaggttcaagcaagcgactcataatcgccagatggt
ggttcaattccacccagggccacca
1-5tRNA-Thr(tgt)c[67964,68039]
gctgatttagctcagtaggtagagcaactcacttgtaatgagaaggtcgg
cggttcgattccgtcaatcagcacca
1-6tRNA-Ser(tga)c[68045,68134]
ggaggcgtggcagagtggtttaatgcaccggtcttgaaaaccggcagtcg
ctccggcgactcataggttcaaatcctatcgcctccgcca
1-7tRNA-Pro(tgg)c[68136,68210]
ctccgtgtagctcagtttggtagagcgcctgatttgggatcaggaggtcc
aaggttcaaatccttgtatggagac
1-8tRNA-Gly(tcc)c[68221,68294]
gcggatatcgtataatggcattacctcagacttccaatctgatgatgtga
gttcgattctcattatccgctcca
1-9tRNA-Leu(taa)c[68300,68386]
gcgagaatggtcaaattggtaaaggcacagcacttaaaatgctgcggaat
gatttccttgtgggttcgagtcccacttctcgcacca
1-10tRNA-Gln(ttg)c[68387,68460]
tgggaattagccaagttggtaaggcatagcactttgactgctagatgcaa
aggttcgagtcctttattcccagc
>KT001915.1 Citrobacter phage Merlin, complete genome
1-1tRNA-Met(cat)c[72013,72089]
ggccccttagctcagttggttagagcagtcgactcataatcgattggtcg

ctggttcgagtccagcaggggccacca
1-2tRNA-Arg(tct)c[72094,72169]
gttcttgtagctcagtggatagagcaacggtcttctaaaccgtgggtcgt
tggttcaaatccaaccaggaacacca
1-3tRNA-Pro(tgg)c[72175,72251]
cagtacgtagcgcagtctggtagcgtaggagctttggatgcttcgggtcg
taggttcgaatcctaccgtactgacca
1-4tRNA-Gly(tcc)c[72259,72333]
gcatccatcgtatagcggctattatgactggcttccacccagtagatgag
agttcgattctctctggatgctcca
1-5tRNA-Ser(tga)c[72704,72796]
ggaagcgtggtagagttggtttattacaccggtcttgaaaaccggaggcc
gtagtgatacggtccgtgggttcgaatcccaccgcttcctcca
1-6tRNA-His(gtg)c[72801,72876]
gtggccgtagttcagttggtagaactcgagattgtgattctcgtagtcat
gggttcaactcccatcggtcacccca
1-7tRNA-Gln(ttg)c[72982,73057]
tggggtatagccaagttggtacggcagtagattttgattctacgattccc
tggttcgagtccaggtaccccagcca
1-8tRNA-Met(cat)c[73065,73141]
tgcgggttaacttcagttggtagaatgacgggttcatatcccgttacgcg
atggttcgagtccatcacccgcctcca
1-9tRNA-Asp(gtc)c[73299,73374]
ggacctatagtttcagcggttaaaatactcgcctgtcacgtgagagtcac
gggttcgaatcccgttaggtccgcca
1-10tRNA-Asn(gtt)c[73891,73975]
ggtcagttggctgagagggtaagcggcggactgttaatccgcgtcggtaa
cgacaaggcaggttcgatacctgcactgaccgcca
1-11tRNA-Lys(ttt)c[73981,74057]
gggttgctagctcagttggttagagcaccggacttttaatccgggtgtcc
gaagttcgaatcttcggcaacccacca
>KT381880.1 Citrobacter phage Margaery, complete genome
1-1tRNA-Gly(tcc)[49264,49339]
gcaggtgtagttcaattggtagaatatctggtttccatccagattgttga
gggttcgagtcccttcacctgctcca
>KM236245.1 Bacillus phage Mater, complete genome
1-1tRNA-Thr(tgt)c[132627,132702]
gctcttgtagctcagttggcagagcagctgatttgtaatcagcaggtcgt
aggttcgagacctaccgagagcacca
1-2tRNA-Asp(gtc)c[133079,133151]
tggcccattagtgtagcggtaacacgactgactgtctatcagttatcctc
ggttcaaatccgggatgggtcgt
1-3tRNA-Gly(tcc)c[133873,133946]
ctcccattggtctagcggtttatgactcttgccttccaagcaagcagcct

cggttcgattccgagatgggggtc
1−4tRNA−Glu(ttc)c[133948,134023]
agcgctgtggtgaagcggcattaacacactcgactttctatcgagcattc
gcgggttcgagtcccgtcagcgctac
1−5tRNA−Met(cat)c[134034,134105]
tgctgaggggtcggtacccagtgaggtctcataagccttacttagtaagt
tcaactcttacctcagcaacca
1−6tRNA−Asn(gtt)c[134107,134182]
tgactagtagctcagttggtagagcaatcggctgttaaccgatcggtcgc
aggttcgagtcctgcctagtcagcca
>KM607004.1 Enterobacteria phage RB68, complete genome
1−1tRNA−Arg(tct)c[71249,71325]
cgaggcatagctcaattgtatagagcaacggacttctaatccgtaggttg
aaggttagaatccttctgtctcgacca
1−2tRNA−Asn(gtt)c[71330,71404]
ggatgtgtagctcagtggtagagcagttgactgttaatcaattggtccat
ggttcgaatccatgcatgtccgcca
1−3tRNA−Tyr(gta)c[71409,71495]
ggggagttatcccgtagaggtagcggtgtggactgtaaattcattgtcat
tgcgactcgggtggttcgactccatcactccccacca
1−4tRNA−Met(cat)c[71508,71582]
ggccctgtagctggaaggttcaagcaagcgactcataatcgccagatggt
ggttcaattccacccagggccacca
1−5tRNA−Thr(tgt)c[71584,71659]
gctgatttagctcagtaggtagagcaactcacttgtaatgagaaggtcgg
cggttcgattccgtcaatcagcacca
1−6tRNA−Ser(tga)c[71665,71754]
ggaggtgtggcagagtggttgaatgcaccggtcttgaaaaccggcagtcg
ctccggcgattcataggttcgaatcctatcacctccgcca
1−7tRNA−Gly(tcc)c[72416,72489]
gcggatatcgtataatggtattacctcagacttccaatctgatgatgtga
gttcgattctcattatccgctcca
1−8tRNA−Leu(taa)c[72495,72581]
gcgagaatggtcaaattggtaaaggcacagcacttaaaatgctgcggaat
gatttccttgtgggttcgagtcccacttctcgcacca
1−9tRNA−Gln(ttg)c[72582,72655]
tgggaattagccaagttggtaaggcactggattttgattccaggatgcaa
aggttcgagtcctttattcccagc
>KT001916.1 Citrobacter phage Michonne, complete genome
1−1tRNA−Cys(gca)c[58564,58639]
gtatcggtgacagaaatggatatgtccctgtctgcaaaacaggtttatga
gggttcaagtcccttccgatactcca
1−2tRNA−Ser(gct)c[58646,58738]
ggaagattaaccctaaaaggtaagggagcagtttgctaaactgccagtaa

ccgagaaatcggcgtaccagttcaagtctggtatcttcctcca
1−3tRNA−Phe(gaa)c[59491,59566]
agctcaattagcttatatggttaaagcgcgtgtctgaaaaacatgagaac
agggttcaaatcccgatgggctacca
1−4tRNA−His(gtg)c[59574,59650]
gtggccttatcataaatggataatgacccatgctgtgaacatggtctata
cgggttcgattcccgtaggtcacccca
1−5tRNA−Gln(ctg)c[59682,59757]
agcagtatagcataactggcaatgctccagcctctgaagctgaaagatta
aggttcaaatccttatactgctgcca
1−6tRNA−Leu(taa)c[59764,59842]
gctccattactccaattggcagagaggccagacttaaaatctgtgttatg
tatcggttcgaatccgatatggagtacca
1−7tRNA−Gln(ttg)c[59845,59920]
aggggattagtttacaaggttaaaacctcggtctttgaaatcgaagaaga
tggttcaattccatcatcccccgcca
1−8tRNA−Arg(acg)c[59930,60006]
gcagaattagttcaaatggacagagcagcaacctacgaagttgttagtta
ggggttcgaatcctctattctgcacca
1−9tRNA−Leu(caa)c[60185,60262]
gttccagtatcccaattggcagaggatgcaagctcaaaccttgtattagt
gacggttcgaatccgtcttggaacacca
1−10tRNA−Val(tac)c[60264,60338]
gctcgcttagtttatatggtaaagccccatccttacaagttggtgaagaa
ggttcaagtccttcagcgagtacca
1−11tRNA−Thr(tgt)c[60434,60510]
gctgctttcgtataattggctattgcacatcccttgtaaggatggagatg
caggttcgagtcctgtgagcagcacca
1−12tRNA−Gly(tcc)c[60517,60591]
gcatccatagtttaaatgggaaaactacagtcttccaaactgaagttgag
ggttcgattccctctggatgctcca
1−13tRNA−Ala(tgc)c[60598,60673]
ggggtcatagtttatatggttaaaatccgagttttgcaaacttgggaact
gagttcaattctcagtgactccacca
1−14tRNA−Lys(ctt)c[60680,60755]
gcaggtgtagcaaaatggttatgcggctgactcttaatcagtaagacgat
gggttcaattccctccacctgtacca
1−15tRNA−Leu(tag)c[60763,60839]
gcacctatagcccaattggaagaggcagcagacttagaatctgctcagtg
tgagttcgaatctctctaggtgtacca
1−16tRNA−Ser(tga)c[61560,61649]
ggtaggtagcggctaatggaagccaaactgtcttgaaaacagttgccatt
gtagagatacggtgagggttcgactcctttatctaccgcc
1−17tRNA−Arg(tct)c[62040,62115]

gctccgttagtctaaaggataggcaactgtcttctaaacagttcatatat
aggttcgagtcctatacggagtgcca
1–18tRNA–Ile(gat)c[62364,62439]
gctggtatagttaagaaggttataacactcccctgataagggaatatcgg
tggttcgattccacctaccagtacca
1–19tRNA–Met(cat)c[62442,62518]
ggttcagtcgcagataaggtaatgcaagggtttcataagccctatgaatg
tgggttcgattcccacctgaacctcca
1–20tRNA–Lys(ttt)c[62600,62675]
ggaagtgtagcagaatggtgatgcggcagacttttaatctgacaggcgac
gggttcgaatccctccacttctacca
1–21tRNA–Asp(gtc)c[63099,63175]
ggttatgtagtttaactggataaaatacctccctgtcacggaggaagatg
agggttcgattcccttcgtaaccgcca
1–22tRNA–Asn(gtt)c[63339,63415]
ggttaggaagcacataaggcatgtgcggtcggctgttaaccgaatggtac
agggttcgaatccctgactaaccgcca
1–23tRNA–Met(cat)c[63500,63576]
tgcgggtatagagaaagggcgtctcacatgtctcattagcatggtatcgg
taggttcgactcctacacccgcctcca
1–24tRNA–Glu(ttc)c[63664,63741]
attcccgtagacaaattaggtaaagtcaacaccctttcaaggtgtggttt
gagggttcaagtcccttcgggaatgcca
1–25tRNA–Pro(tgg)c[63748,63824]
ctccatttagctcagcttggtagagcgttccgtttggggcggtaaggccg
aaggttcaagtccttcaatggagacca
>KM236239.1 Citrobacter phage Moogle, complete genome
1–1tRNA–Cys(gca)c[60325,60400]
gtatcggtgacagaaatggatatgtgcctgtctgcaaaacaggtttatga
gggttcaagtcccttccgatactcca
1–2tRNA–Ser(gct)c[60408,60500]
ggaagattaaccataaaaggtaagggagcagtttgctaaactgccagtaa
ccgagaaatcggcgtaccagttcaagtctggtatcttcctcca
1–3tRNA–Phe(gaa)c[61252,61327]
agctcaattagcttatatggttaaagcgcgtgtctgaaaaacatgagaac
agggttcaaatcccgatgggctacca
1–4tRNA–His(gtg)c[61335,61411]
gtggccttatcataaatggataatgacccatgctgtgaacatggtctata
cgggttcgattcccgtaggtcacccca
1–5tRNA–Gln(ctg)c[61443,61518]
agcagtgtagcataactggcaatgcaccagcctctgaagctgaatgatta
aggttcaaatccttacgctgctgcca
1–6tRNA–Leu(taa)c[61525,61603]
gctccattactccaattggcagagagaccagacttaaaatctgcgttatg

tatcggttcgaatccgatatggagtacca
1-7tRNA-Gln(ttg)c[61606,61681]
aggggattagtttacacggttaaaacctcggtctttgaaatcgaagaaga
tggttcaattccatcatcccccgcca
1-8tRNA-Arg(acg)c[61689,61765]
gcagaattagttcaaatggacagagcagcagcctacgaagttgttagtta
gaggttcgaatcctctattctgcacca
1-9tRNA-Leu(caa)c[61944,62021]
gttccagtatcccaattggcagaggatgcaagctcaaaccttgtattagt
gacggttcgaatccgtcttggaacacca
1-10tRNA-Val(tac)c[62023,62097]
gctcgcttagtttatatggtaaagccccatccttacaagttggtgaagaa
ggttcaagtccttcagcgagtacca
1-11tRNA-Thr(tgt)c[62192,62267]
gctcctatcgtataactggctattacggttgccttgtaagcaacttatca
gggttcgaatccttgtgggagcacca
1-12tRNA-Gly(tcc)c[62274,62348]
gcatccatagtttaaacgggaaaattacagtcttccaaactgaggttgag
ggttcgattccctctggatgctcca
1-13tRNA-Ala(tgc)c[62355,62430]
ggggatgtagtttacatggttaaaacataagttttgcaaacttaagtaca
gggttcaattccctgcttctccacca
1-14tRNA-Lys(ctt)c[62437,62512]
gcaggtgtagcaaaatggttatgcggctgactcttaatcagtaagacgat
gggttcaattccctccacctgtacca
1-15tRNA-Leu(tag)c[62520,62596]
gcacctatagcccaattggaagaggcagcagacttagaatctgctcagtg
tgagttcgagtctctctaggtgtacca
1-16tRNA-Ser(tga)c[62848,62937]
ggtaggtagcggctaatggaagccaaactgtcttgaaaacagttgccatt
gtagagatacggtgagggttcgactcctttatctaccgcc
1-17tRNA-Arg(tct)c[63328,63404]
gcacccttagttcaaacggatagagcaacggtcttctaaaccgtcagtta
caggttcgaatcctgtagggtgtgcca
1-19tRNA-Ile(gat)c[63682,63757]
gctggtatagttaagacggttataacactcccctgataagggaacatcgg
tggttcgattccacctaccagtacca
1-20tRNA-Met(cat)c[63760,63836]
ggttcagtcgcagataaggtaatgcaagggtttcataagccctatgaatg
tgggttcgattcccacctgaacctcca
1-21tRNA-Lys(ttt)c[63843,63918]
ggaagtgtagcagaatggtgatgcggcagacttttaatctgacaggcgat
gggttcgaatccctccacttctacca
1-22tRNA-Asp(gtc)c[64346,64422]

ggttatgtagtttaactggataaaatacctccctgtcacggaggatgatg
agggttcgattcccttcgtaaccgcca
1−23tRNA−Asn(gtt)c[64528,64603]
ggtaagttagctaacttggtaaagcgcctgactgttaatcagcgatatca
gggttcaaatccctgacttaccgcca
1−24tRNA−Met(cat)c[64688,64764]
tgcgggtatagagaaagggcgtctcacatgtctcattagcatggtatcgg
taggttcgactcctacacccgcctcca
1−25tRNA−Glu(ttc)c[64851,64928]
attcccgtagacaaattaggtaaagtcaacaccctttcaaggtgtggttt
gagggttcaagtcccttcgggaatgcca
1−26tRNA−Pro(tgg)c[64935,65011]
ctccatttagctcagcttggtagagcgttccgtttggggcggtaaggccg
aaggttcaagtccttcaatggagacca
>KM236246.1 Bacillus phage Moonbeam, complete genome
1−1tRNA−Ser(gct)[28945,29034]
tggaggggtactctagcggtcgatgaggttcggttgctaaccggatagac
gggtaaaaccgtgcgagagttcgaatctctctccctccgt
1−2tRNA−Asn(gtt)[29314,29388]
tgactagtagctcagtggtagagcaatcggctgttaaccgatcggtcgta
ggttcgagtcctacctagtcagcca
1−3tRNA−Trp(cca)[29391,29464]
tatcgagctagtgtaatggtagcacaggagtctccaaaacttctagtctg
ggttcaaatcctaggttcggtgtc
>KM606994.1 Enterobacteria phage RB3, complete genome
1−1tRNA−Arg(tct)c[67424,67499]
cgaggcatagctcagaaggaagagcaaggaccttctaagtcctgggtcgt
aggttcgatccctactgcctcgacca
1−2tRNA−Asn(gtt)c[67504,67578]
ggatgtgtagctcaatggcagagcgatcgcctgttaagcgattggttata
ggttcgaatcctatcacgtccgcca
1−3tRNA−Tyr(gta)c[67583,67669]
ggggagttatcccgtagaggtagcggtgtggactgtaaatccattgtcat
tgcgactcgggtggttcgactccatcactccccacca
1−4tRNA−Met(cat)c[67679,67753]
ggccctgtagctggaaggttcaagcaagcgactcataatcgccagatggt
ggttcaattccacccagggccacca
1−5tRNA−Thr(tgt)c[67755,67830]
gctgatttagctcagtaggtagagcacctcacttgtaatgaggatgtcgg
cggttcgattccgtcaatcagcgcca
1−6tRNA−Ser(tga)c[67836,67925]
ggaggcgtggcagagtggtttaatgcaccggtcttgaaaaccggcagtcg
ctccggcgactcataggttcaaatcctatcgcctccgcca
1−7tRNA−Pro(tgg)c[67927,68001]

ctccgtgtagctcagtttggtagagcgcctgatttgggatcaggaggtcc
aaggttcaaatccttgtatggagac
1-8tRNA-Gly(tcc)c[68012,68085]
gcggatatcgtataatggcattacctcagacttccaatctgatgatgtga
gttcgattctcattatccgctcca
1-9tRNA-Leu(taa)c[68091,68177]
gcgagaatggtcaaattggtaaaggcacagcacttaaaatgctgcggaat
gatttccttgtgggttcgagtcccacttctcgcacca
1-10tRNA-Gln(ttg)c[68178,68251]
tgggaattagccaagttggtaaggcactggattttgattccaggatgcaa
aggttcgagtcctttattcccagc
>LN681537.2 Clostridium phage phiCD211, complete genome
1-1tRNA-Ser(gct)[6133,6223]
ggagaggtactcaagtggtaaagaggatagtttgctaaactattaggtca
attaatttgatacgagggttcaaatcccttcctctcttcca
>KU686201.1 Synechococcus phage S-CAM4 isolate 0809SB33, complete genome
1-1tRNA-Met(cat)[17049,17124]
tgcttccttagcaatctggtgaatgcagcaaactcataatttgcctaagg
agagttcgatcctctcaggaagcatc
1-2tRNA-Leu(taa)[17125,17211]
tgcgagtatggcggaatcggtagacgcaccagacttaaaatctgttgagc
attatgctcgtgggagttcaagtctccctactcgcat
1-3tRNA-Thr(tgt)[18029,18101]
gccaaactagctcagtggtagagcagggcttttgtaaagctcaggtcgca
ggttcaaatcctgtgtttggctc
1-4tRNA-Asn(gtt)[18106,18177]
tcctctttagctcagcggtagagcggttgactgttaatcaatttgtccct
ggttcgatcccaggaaggggag
1-5tRNA-Arg(tct)[158001,158077]
gactcagtagctcagttggatagagcaactgccttctaagcagtcggtcg
ttggttcgagtccaacctgagtcgcca
1-6tRNA-Leu(tag)[158078,158160]
gtcggtatggcggaattggtagacgcgccaggtttaggttctggtgtctt
atgacgtggaggttcaagtcctcttaccgacac
1-7tRNA-Val(tac)[158163,158234]
gctcgaatagctcagaggtagagcacctcctttacacggagattgtcggg
ggttcgatcccctcttcgagca
1-8tRNA-Gly(tcc)[161371,161442]
gcgggtgtagctcagtggtagagcgtcagttttccaaactgaatgtcgtc
ggttcaagtccgatctcccgct
>HQ317290.1 Synechococcus phage S-RIM2 R21_2007, complete genome
1-1tRNA-Val(tac)[13794,13865]
gcccgaatagctcagcggtagagcacctcctttacacggagattgtcggg
ggttcgatcccctcttcgggca

1−2tRNA−Leu(taa)[17791,17877]
tgggagtatggcggaatcggtagacgcaccagacttaaaatttgttgagg
gttaacctcgtgagagttcaagtctctctactcctat
1−3tRNA−Thr(tgt)[17934,18006]
gcccccgtagctcagtggtagagcagggcttttgtaaagctcaggtcgca
agttcaaatcttgtcaggggctc
1−4tRNA−Ala(tgc)[18009,18081]
ggggaattagctcagttggtagagcgcctgctttgcaagcaggatgtcag
cggttcgagtccgctattctcca
1−5tRNA−Asn(gtt)[18128,18201]
ttcctcagtagctcagcggcagagccatcgactgttaatcgattggtcgt
aggttcaaatcctacctggggagt
1−6tRNA−Arg(tct)[161238,161312]
tgggtcagtagctcagcggatagagcaaccgccttctaagcggttggtcg
caggttcaaatcctgcctgacccgt
>KJ019029.1 Synechococcus phage ACG−2014d isolate Syn7803C46, complete genome
1−1tRNA−Val(tac)[13618,13691]
tgggtgattaactcagtggtagagtgactgctttacacgcagtaggtcac
tggttcaaatccagtattacccat
1−2tRNA−Arg(tct)[153719,153793]
tgggcaagtagctcagtggatagagcatcgcacttctaatgcgttggtcg
ggggttcaaatccctccttgcccgt
1−3tRNA−Asn(gtt)[153814,153887]
ttcccaagtagctcagtggcagagccgccgactgttaatcggctggtcgc
tggttcaaatccagccttgggagt
>KJ019028.1 Synechococcus phage ACG−2014d isolate Syn7803C45, complete genome
1−1tRNA−Val(tac)[13624,13697]
tgggtgattaactcagtggtagagtgactgctttacacgcagtaggtcac
tggttcaaatccagtattacccat
1−2tRNA−Arg(tct)[153727,153801]
tgggcaagtagctcagtggatagagcatcgcacttctaatgcgttggtcg
ggggttcaaatccctccttgcccgt
1−3tRNA−Asn(gtt)[153822,153895]
ttcccaagtagctcagtggcagagccgccgactgttaatcggctggtcgc
tggttcaaatccagccttgggagt
>KX349226.1 Synechococcus phage S−RIM2 isolate Fa_02_0709, complete genome
1−1tRNA−Val(tac)[13788,13859]
gcccgaatagctcagcggtagagcacctcgtttacaccgagattgtcggc
ggttcgatcccgtcttcgggca
1−2tRNA−Leu(taa)[17785,17871]
tgggagtatggcggaatcggtagacgcaccagacttaaaatttgttgagg
gttaacctcgtgagagttcaagtctctctactcctat
1−3tRNA−Thr(tgt)[17928,18000]
gcccccgtagctcagtggtagagcagggcttttgtaaagctcaggtcgca

agttcaaatcttgtcaggggctc
1−4tRNA−Ala(tgc)[18003,18075]
ggggaattagctcagttggtagagcgcctgctttgcaagcaggatgtcag
cggttcgagtccgctattctcca
1−5tRNA−Asn(gtt)[18122,18195]
ttcctcagtagctcagcggcagagccatcgactgttaatcgattggtcgt
aggttcaaatcctacctggggagt
1−6tRNA−Arg(tct)[161217,161291]
tgggtcagtagctcagcggatagagcaaccgccttctaagcggttggtcg
caggttcaaatcctgcctgacccgt
>KX349227.1 Synechococcus phage S−RIM2 isolate Fa_10_0709, complete genome
1−1tRNA−Val(tac)[13794,13865]
gcccgaatagctcagcggtagagcacctcgtttacaccgagattgtcggc
ggttcgatcccgtcttcgggca
1−2tRNA−Leu(taa)[17791,17877]
tgggagtatggcggaatcggtagacgcaccagacttaaaatttgttgagg
gttaacctcgtgagagttcaagtctctctactcctat
1−3tRNA−Thr(tgt)[17934,18006]
gcccccgtagctcagtggtagagcagggcttttgtaaagctcaggtcgca
agttcaaatcttgtcaggggctc
1−4tRNA−Ala(tgc)[18009,18081]
ggggaattagctcagttggtagagcgcctgctttgcaagcaggatgtcag
cggttcgagtccgctattctcca
1−5tRNA−Asn(gtt)[18128,18201]
ttcctcagtagctcagcggcagagccatcgactgttaatcgattggtcgt
aggttcaaatcctacctggggagt
1−6tRNA−Arg(tct)[161239,161313]
tgggtcagtagctcagcggatagagcaaccgccttctaagcggttggtcg
caggttcaaatcctgcctgacccgt
>KX349228.1 Synechococcus phage S−RIM2 isolate Fa_24_0709, complete genome
1−1tRNA−Val(tac)[13794,13865]
gcccgaatagctcagcggtagagcacctcgtttacaccgagattgtcggg
ggttcgatccccctcttcgggca
1−2tRNA−Leu(taa)[17791,17877]
tgggagtatggcggaatcggtagacgcaccagacttaaaatttgttgagg
gttaacctcgtgagagttcaagtctctctactcctat
1−3tRNA−Thr(tgt)[17934,18006]
gcccccgtagctcagtggtagagcagggcttttgtaaagctcaggtcgca
agttcaaatcttgtcaggggctc
1−4tRNA−Ala(tgc)[18009,18081]
ggggaattagctcagttggtagagcgcctgctttgcaagcaggatgtcag
cggttcgagtccgctattctcca
1−5tRNA−Asn(gtt)[18128,18201]
ttcctcagtagctcagcggcagagccatcgactgttaatcgattggtcgt

aggttcaaatcctacctggggagt
1−6tRNA−Arg(tct)[161237,161311]
tgggtcagtagctcagcggatagagcaaccgccttctaagcggttggtcg
caggttcaaatcctgcctgacccgt
>KX349229.1 Synechococcus phage S−RIM2 isolate LIS_01_1010, complete genome
1−1tRNA−Val(tac)[13895,13966]
gcccgaatagctcagcggtagagcacctcgtttacaccgagattgtcggc
ggttcgatcccgtcttcgggca
1−2tRNA−Leu(taa)[17892,17978]
tgggagtatggcggaatcggtagacgcaccagacttaaaatttgttgagg
gttaacctcgtgagagttcaagtctctctactcctat
1−3tRNA−Thr(tgt)[18035,18110]
gccttcttagctcagctggatagagcaacggttttgtaaaccgtaggtcg
tcggttcaagtccgacagaaggctcc
1−4tRNA−Ala(tgc)[18112,18187]
tggggaattagctcagttggtagtagcgtttgctttgcaagcaaaatgtc
agcggttcgagtccgctattctccat
1−5tRNA−Asn(gtt)[18233,18306]
ttcctcagtagctcagcggcagagctatcgactgttaatcgattggtcgt
aggttcaaatcctacctggggagt
1−6tRNA−Arg(tct)[160889,160963]
tgggtcagtagctcagcggatagagcaaccgccttctaagcggttggtcg
caggttcaaatcctgcctgacccgt
>KX349230.1 Synechococcus phage S−RIM2 isolate LIS_02_1013, complete genome
1−1tRNA−Val(tac)[13794,13865]
gcccgaatagctcagcggtagagcacctcgtttacaccgagattgtcggg
ggttcgatccccctcttcgggca
1−2tRNA−Leu(taa)[17791,17877]
tgggagtatggcggaatcggtagacgcaccagacttaaaatttgttgagg
gttaacctcgtgagagttcaagtctctctactcctat
1−3tRNA−Thr(tgt)[17934,18006]
gcccccgtagctcagtggtagagcagggcttttgtaaagctcaggtcgca
agttcaaatcttgtcaggggctc
1−4tRNA−Ala(tgc)[18352,18425]
ggggaattagctcagtctggtagagcgcctgctttgcaagcaggatgtca
gcggttcgagtccgctattctcca
1−5tRNA−Asn(gtt)[18472,18545]
ttcctcagtagctcagcggcagagccatcgactgttaatcgattggtcgt
aggttcaaatcctacctggggagt
1−6tRNA−Arg(tct)[161584,161658]
tgggtcagtagctcagcggatagagcaaccgccttctaagcggttggtcg
caggttcaaatcctgcctgacccgt
>KX349231.1 Synechococcus phage S−RIM2 isolate LIS_06_1010, complete genome
1−1tRNA−Val(tac)[13904,13975]

gcccgaatagctcagcggtagagcacctcgtttacaccgagattgtcggc
ggttcgatcccgtcttcgggca
1–2tRNA–Leu(taa)[17901,17987]
tgggagtatggcggaatcggtagacgcaccagacttaaaatttgttgagg
gttaacctcgtgagagttcaagtctctctactcctat
1–3tRNA–Thr(tgt)[18044,18116]
gcccccgtagctcagtggtagagcagggcttttgtaaagctcaggtcgca
agttcaaatcttgtcaggggctc
1–4tRNA–Ala(tgc)[18462,18535]
ggggaattagctcagtctggtagagcgcctgctttgcaagcaggatgtca
gcggttcgagtccgctattctcca
1–5tRNA–Asn(gtt)[18582,18655]
ttcctcagtagctcagcggcagagctatcgactgttaatcgattggtcgt
aggttcaaatcctacctggggagt
1–6tRNA–Arg(tct)[161246,161320]
tgggtcagtagctcagcggatagagcaaccgccttctaagcggttggtcg
caggttcaaatcctgcctgacccgt
>KX349232.1 Synechococcus phage S–RIM2 isolate LIS_09_1010, complete genome
1–1tRNA–Val(tac)[13785,13856]
gcccgaatagctcagcggtagagcacctcgtttacaccgagattgtcggc
ggttcgatcccgtcttcgggca
1–2tRNA–Leu(taa)[17783,17867]
gggagtatggcggaatcggtagacgcaccagacttaaaatttgttgaggg
ttaacctcgtgagagttcaagtctctctactccta
1–3tRNA–Thr(tgt)[17925,17997]
gcccccgtagctcagtggtagagcagggcttttgtaaagctcaggtcgca
agttcaaatcttgtcaggggctc
1–4tRNA–Ala(tgc)[18000,18072]
ggggaattagctcagttggtagagcgcctgctttgcaagcaggatgtcag
cggttcgagtccgctattctcca
1–5tRNA–Asn(gtt)[18119,18192]
ttcctcagtagctcagcggcagagccatcgactgttaatcgattggtcgt
aggttcaaatcctacctggggagt
1–6tRNA–Arg(tct)[161238,161312]
tgggtcagtagctcagcggatagagcaaccgccttctaagcggttggtcg
caggttcaaatcctgcctgacccgt
>KX349233.1 Synechococcus phage S–RIM2 isolate LIS_11_1010, complete genome
1–1tRNA–Val(tac)[13806,13877]
gcccgaatagctcagcggtagagcacctcgtttacaccgagattgtcggg
ggttcgatccccctcttcgggca
1–2tRNA–Leu(taa)[17804,17888]
gggagtatggcggaatcggtagacgcaccagacttaaaatttgttgaggg
ttaacctcgtgagagttcaagtctctctactccta
1–3tRNA–Thr(tgt)[17946,18018]

gcccccgtagctcagtggtagagcagggcttttgtaaagctcaggtcgca
agttcaaatcttgtcaggggctc
1-4tRNA-Ala(tgc)[18021,18093]
ggggaattagctcagttggtagagcgcctgctttgcaagcaggatgtcag
cggttcgagtccgctattctcca
1-5tRNA-Asn(gtt)[18140,18213]
ttcctcagtagctcagcggcagagccatcgactgttaatcgattggtcgt
aggttcaaatcctacctggggagt
1-6tRNA-Arg(tct)[161254,161328]
tgggtcagtagctcagcggatagagcaaccgccttctaagcggttggtcg
caggttcaaatcctgcctgacccgt
>KX349234.1 Synechococcus phage S-RIM2 isolate LIS_12_1010, complete genome
1-1tRNA-Val(tac)[13892,13963]
gcccgaatagctcagcggtagagcacctcgtttacaccgagattgtcggc
ggttcgatcccgtcttcgggca
1-2tRNA-Leu(taa)[17889,17975]
tgggagtatggcggaatcggtagacgcaccagacttaaaatttgttgagg
gttaacctcgtgagagttcaagtctctctactcctat
1-3tRNA-Thr(tgt)[18032,18105]
gcccccgtagctcagtggtagagcagggcttttgtaaagctcaggtcgca
agttcaaatcttgtcaggggctcc
1-4tRNA-Ala(tgc)[18107,18182]
tggggaattagctcagtctggtagagcgcctgctttgcaagcaggatgtc
agcggttcgagtccgctattctccat
1-5tRNA-Asn(gtt)[18228,18301]
ttcctcagtagctcagcggcagagctatcgactgttaatcgattggtcgt
aggttcaaatcctacctggggagt
1-6tRNA-Arg(tct)[160890,160964]
tgggtcagtagctcagcggatagagcaaccgccttctaagcggttggtcg
caggttcaaatcctgcctgacccgt
>KX349235.1 Synechococcus phage S-RIM2 isolate LIS_14_1013, complete genome
1-1tRNA-Val(tac)[13797,13868]
gcccgaatagctcagcggtagagcacctcgtttacaccgagattgtcggg
ggttcgatccccctcttcgggca
1-2tRNA-Leu(taa)[17794,17880]
tgggagtatggcggaatcggtagacgcaccagacttaaaatttgttgagg
gttaacctcgtgagagttcaagtctctctactcctat
1-3tRNA-Thr(tgt)[17937,18009]
gcccccgtagctcagtggtagagcagggcttttgtaaagctcaggtcgca
agttcaaatcttgtcaggggctc
1-4tRNA-Ala(tgc)[18012,18084]
ggggaattagctcagttggtagagcgcctgctttgcaagcaggatgtcag
cggttcgagtccgctattctcca
1-5tRNA-Asn(gtt)[18131,18204]

ttcctcagtagctcagcggcagagccatcgactgttaatcgattggtcgt
aggttcaaatcctacctggggagt
1-6tRNA-Arg(tct)[161246,161320]
tgggtcagtagctcagcggatagagcaaccgccttctaagcggttggtcg
caggttcaaatcctgcctgacccgt
>KX349236.1 Synechococcus phage S-RIM2 isolate NJ_05_1013, complete genome
1-1tRNA-Val(tac)[13791,13862]
gcccgaatagctcagcggtagagcacctcgtttacaccgagattgtcggc
ggttcgatcccgtcttcgggca
1-2tRNA-Leu(taa)[17788,17874]
tgggagtatggcggaatcggtagacgcaccagacttaaaatttgttgagg
gttaacctcgtgagagttcaagtctctctactcctat
1-3tRNA-Thr(tgt)[17931,18003]
gcccccgtagctcagtggtagagcagggcttttgtaaagctcaggtcgca
agttcaaatcttgtcaggggctc
1-4tRNA-Ala(tgc)[18006,18078]
ggggaattagctcagttggtagagcgcctgctttgcaagcaggatgtcag
cggttcgagtccgctattctcca
1-5tRNA-Asn(gtt)[18125,18198]
ttcctcagtagctcagcggcagagccatcgactgttaatcgattggtcgt
aggttcaaatcctacctggggagt
1-6tRNA-Arg(tct)[160088,160162]
tgggtcagtagctcagcggatagagcaaccgccttctaagcggttggtcg
caggttcaaatcctgcctgacccgt
>KX349237.1 Synechococcus phage S-RIM2 isolate Np_01_0709, complete genome
1-1tRNA-Val(tac)[13794,13865]
gcccgaatagctcagcggtagagcacctcgtttacaccgagattgtcggg
ggttcgatcccctcttcgggca
1-2tRNA-Leu(taa)[17792,17876]
gggagtatggcggaatcggtagacgcaccagacttaaaatttgttgaggg
ttaacctcgtgagagttcaagtctctctactccta
1-3tRNA-Thr(tgt)[17934,18006]
gcccccgtagctcagtggtagagcagggcttttgtaaagctcaggtcgca
agttcaaatcttgtcaggggctc
1-4tRNA-Ala(tgc)[18009,18081]
ggggaattagctcagttggtagagcgcctgctttgcaagcaggatgtcag
cggttcgagtccgctattctcca
1-5tRNA-Asn(gtt)[18128,18201]
ttcctcagtggcgcagcggtagcgcagttgactgttaatcaatgggtcgc
aagttcgaatcttgcctggggagt
1-6tRNA-Arg(tct)[161147,161221]
tgggtcagtagctcagcggatagagcaaccgccttctaagcggttggtcg
caggttcaaatcctgcctgacccgt
>KX349238.1 Synechococcus phage S-RIM2 isolate Np_01_1112, complete genome

1-1tRNA-Val(tac)[13794,13865]
gcccgaatagctcagcggtagagcacctcgtttacaccgagattgtcggc
ggttcgatcccgtcttcgggca
1-2tRNA-Leu(taa)[17791,17877]
tgggagtatggcggaatcggtagacgcaccagacttaaaatttgttgagg
gttaacctcgtgagagttcaagtctctctactcctat
1-3tRNA-Thr(tgt)[17934,18006]
gcccccgtagctcagtggtagagcagggcttttgtaaagctcaggtcgca
agttcaaatcttgtcaggggctc
1-4tRNA-Ala(tgc)[18009,18081]
ggggaattagctcagttggtagagcgcctgctttgcaagcaggatgtcag
cggttcgagtccgctattctcca
1-5tRNA-Asn(gtt)[18128,18201]
ttcctcagtagctcagcggcagagccatcgactgttaatcgattggtcgt
aggttcaaatcctacctggggagt
1-6tRNA-Arg(tct)[161239,161313]
tgggtcagtagctcagcggatagagcaaccgccttctaagcggttggtcg
caggttcaaatcctgcctgacccgt
>KX349239.1 Synechococcus phage S-RIM2 isolate Np_03_0709, complete genome
1-1tRNA-Val(tac)[13794,13865]
gcccgaatagctcagcggtagagcacctcgtttacaccgagattgtcggc
ggttcgatcccgtcttcgggca
1-2tRNA-Leu(taa)[17791,17877]
tgggagtatggcggaatcggtagacgcaccagacttaaaatttgttgagg
gttaacctcgtgagagttcaagtctctctactcctat
1-3tRNA-Thr(tgt)[17934,18006]
gcccccgtagctcagtggtagagcagggcttttgtaaagctcaggtcgca
agttcaaatcttgtcaggggctc
1-4tRNA-Ala(tgc)[18009,18081]
ggggaattagctcagttggtagagcgcctgctttgcaagcaggatgtcag
cggttcgagtccgctattctcca
1-5tRNA-Asn(gtt)[18128,18201]
ttcctcagtagctcagcggcagagccatcgactgttaatcgattggtcgt
aggttcaaatcctacctggggagt
1-6tRNA-Arg(tct)[161241,161315]
tgggtcagtagctcagcggatagagcaaccgccttctaagcggttggtcg
caggttcaaatcctgcctgacccgt
>KX349240.1 Synechococcus phage S-RIM2 isolate Np_03_1112, complete genome
1-1tRNA-Val(tac)[13785,13856]
gcccgaatagctcagcggtagagcacctcgtttacaccgagattgtcggc
ggttcgatcccgtcttcgggca
1-2tRNA-Leu(taa)[17783,17867]
gggagtatggcggaatcggtagacgcaccagacttaaaatttgttgaggg
ttaacctcgtgagagttcaagtctctctactccta

1-3tRNA-Thr(tgt)[17925,17997]
gcccccgtagctcagtggtagagcagggcttttgtaaagctcaggtcgca
agttcaaatcttgtcaggggctc
1-4tRNA-Ala(tgc)[18000,18072]
ggggaattagctcagttggtagagcgcctgctttgcaagcaggatgtcag
cggttcgagtccgctattctcca
1-5tRNA-Asn(gtt)[18119,18192]
ttcctcagtagctcagcggcagagccatcgactgttaatcgattggtcgt
aggttcaaatcctacctggggagt
1-6tRNA-Arg(tct)[160071,160145]
tgggtcagtagctcagcggatagagcaaccgccttctaagcggttggtcg
caggttcaaatcctgcctgacccgt
>KX349241.1 Synechococcus phage S-RIM2 isolate Np_04_1112, complete genome
1-1tRNA-Val(tac)[13797,13868]
gcccgaatagctcagcggtagagcacctcgtttacaccgagattgtcggg
ggttcgatcccctcttcgggca
1-2tRNA-Leu(taa)[17795,17879]
gggagtatggcggaatcggtagacgcaccagacttaaaatttgttgaggg
ttaacctcgtgagagttcaagtctctctactccta
1-3tRNA-Thr(tgt)[17937,18009]
gcccccgtagctcagtggtagagcagggcttttgtaaagctcaggtcgca
agttcaaatcttgtcaggggctc
1-4tRNA-Ala(tgc)[18012,18084]
ggggaattagctcagttggtagagcgcctgctttgcaagcaggatgtcag
cggttcgagtccgctattctcca
1-5tRNA-Asn(gtt)[18131,18204]
ttcctcagtagctcagcggcagagccatcgactgttaatcgattggtcgt
aggttcaaatcctacctggggagt
1-6tRNA-Arg(tct)[161244,161318]
tgggtcagtagctcagcggatagagcaaccgccttctaagcggttggtcg
caggttcaaatcctgcctgacccgt
>KX349242.1 Synechococcus phage S-RIM2 isolate Np_06_0912, complete genome
1-1tRNA-Val(tac)[13799,13870]
gcccgaatagctcagcggtagagcacctcgtttacaccgagattgtcggg
ggttcgatcccctcttcgggca
1-2tRNA-Leu(taa)[17797,17881]
gggagtatggcggaatcggtagacgcaccagacttaaaatttgttgaggg
ttaacctcgtgagagttcaagtctctctactccta
1-3tRNA-Thr(tgt)[17939,18011]
gcccccgtagctcagtggtagagcagggcttttgtaaagctcaggtcgca
agttcaaatcttgtcaggggctc
1-4tRNA-Ala(tgc)[18014,18086]
ggggaattagctcagttggtagagcgcctgctttgcaagcaggatgtcag
cggttcgagtccgctattctcca

1-5tRNA-Asn(gtt)[18133,18206]
ttcctcagtggcgcagcggtagcgcagttgactgttaatcaatgggtcgc
aagttcgaatcttgcctggggagt
1-6tRNA-Arg(tct)[161247,161321]
tgggtcagtagctcagcggatagagcaaccgccttctaagcggttggtcg
caggttcaaatcctgcctgacccgt
>KX349243.1 Synechococcus phage S-RIM2 isolate Np_11_1112, complete genome
1-1tRNA-Val(tac)[13800,13871]
gcccgaatagctcagcggtagagcacctcgtttacaccgagattgtcggc
ggttcgatcccgtcttcgggca
1-2tRNA-Leu(taa)[17797,17883]
tgggagtatggcggaatcggtagacgcaccagacttaaaatttgttgagg
gttaacctcgtgagagttcaagtctctctactcctat
1-3tRNA-Thr(tgt)[17940,18012]
gcccccgtagctcagtggtagagcagggcttttgtaaagctcaggtcgca
agttcaaatcttgtcaggggctc
1-4tRNA-Ala(tgc)[18015,18087]
ggggaattagctcagttggtagagcgcctgctttgcaagcaggatgtcag
cggttcgagtccgctattctcca
1-5tRNA-Asn(gtt)[18134,18207]
ttcctcagtagctcagcggcagagccatcgactgttaatcgattggtcgt
aggttcaaatcctacctggggagt
1-6tRNA-Arg(tct)[161249,161323]
tgggtcagtagctcagcggatagagcaaccgccttctaagcggttggtcg
caggttcaaatcctgcctgacccgt
>KX349244.1 Synechococcus phage S-RIM2 isolate Np_12_0912, complete genome
1-1tRNA-Val(tac)[13797,13868]
gcccgaatagctcagcggtagagcacctcgtttacaccgagattgtcggc
ggttcgatcccgtcttcgggca
1-2tRNA-Leu(taa)[17794,17880]
tgggagtatggcggaatcggtagacgcaccagacttaaaatttgttgagg
gttaacctcgtgagagttcaagtctctctactcctat
1-3tRNA-Thr(tgt)[17937,18009]
gcccccgtagctcagtggtagagcagggcttttgtaaagctcaggtcgca
agttcaaatcttgtcaggggctc
1-4tRNA-Ala(tgc)[18012,18084]
ggggaattagctcagttggtagagcgcctgctttgcaagcaggatgtcag
cggttcgagtccgctattctcca
1-5tRNA-Asn(gtt)[18131,18204]
ttcctcagtggcgcagcggtagcgcagttgactgttaatcaatgggtcgc
aagttcgaatcttgcctggggagt
1-6tRNA-Arg(tct)[161247,161321]
tgggtcagtagctcagcggatagagcaaccgccttctaagcggttggtcg
caggttcaaatcctgcctgacccgt

>KX349245.1 Synechococcus phage S-RIM2 isolate Np_14_0912, complete genome

1-1tRNA-Val(tac)[13782,13853]
gcccgaatagctcagcggtagagcacctcgtttacaccgagattgtcggc
ggttcgatcccgtcttcgggca
1-2tRNA-Leu(taa)[17779,17865]
tgggagtatggcggaatcggtagacgcaccagacttaaaatttgttgagg
gttaacctcgtgagagttcaagtctctctactcctat
1-3tRNA-Thr(tgt)[17922,17994]
gcccccgtagctcagtggtagagcagggcttttgtaaagctcaggtcgca
agttcaaatcttgtcaggggctc
1-4tRNA-Ala(tgc)[17997,18069]
ggggaattagctcagttggtagagcgcctgctttgcaagcaggatgtcag
cggttcgagtccgctattctcca
1-5tRNA-Asn(gtt)[18116,18189]
ttcctcagtagctcagcggcagagccatcgactgttaatcgattggtcgt
aggttcaaatcctacctggggagt
1-6tRNA-Arg(tct)[161229,161303]
tgggtcagtagctcagcggatagagcaaccgccttctaagcggttggtcg
caggttcaaatcctgcctgacccgt

>KX349246.1 Synechococcus phage S-RIM2 isolate Np_15_0709, complete genome

1-1tRNA-Val(tac)[13809,13880]
gcccgaatagctcagcggtagagcacctcgtttacaccgagattgtcggc
ggttcgatcccgtcttcgggca
1-2tRNA-Leu(taa)[17806,17892]
tgggagtatggcggaatcggtagacgcaccagacttaaaatttgttgagg
gttaacctcgtgagagttcaagtctctctactcctat
1-3tRNA-Thr(tgt)[17949,18021]
gcccccgtagctcagtggtagagcagggcttttgtaaagctcaggtcgca
agttcaaatcttgtcaggggctc
1-4tRNA-Ala(tgc)[18024,18096]
ggggaattagctcagttggtagagcgcctgctttgcaagcaggatgtcag
cggttcgagtccgctattctcca
1-5tRNA-Asn(gtt)[18143,18216]
ttcctcagtagctcagcggcagagccatcgactgttaatcgattggtcgt
aggttcaaatcctacctggggagt
1-6tRNA-Arg(tct)[161262,161336]
tgggtcagtagctcagcggatagagcaaccgccttctaagcggttggtcg
caggttcaaatcctgcctgacccgt

>KX349247.1 Synechococcus phage S-RIM2 isolate Np_15_1112, complete genome

1-1tRNA-Val(tac)[13794,13865]
gcccgaatagctcagcggtagagcacctcgtttacaccgagattgtcggc
ggttcgatcccgtcttcgggca
1-2tRNA-Leu(taa)[17791,17877]
tgggagtatggcggaatcggtagacgcaccagacttaaaatttgttgagg

gttaacctcgtgagagttcaagtctctctactcctat
1-3tRNA-Thr(tgt)[17934,18006]
gcccccgtagctcagtggtagagcagggcttttgtaaagctcaggtcgca
agttcaaatcttgtcaggggctc
1-4tRNA-Ala(tgc)[18009,18081]
ggggaattagctcagttggtagagcgcctgctttgcaagcaggatgtcag
cggttcgagtccgctattctcca
1-5tRNA-Asn(gtt)[18128,18201]
ttcctcagtagctcagcggcagagccatcgactgttaatcgattggtcgt
aggttcaaatcctacctggggagt
1-6tRNA-Arg(tct)[161244,161318]
tgggtcagtagctcagcggatagagcaaccgccttctaagcggttggtcg
caggttcaaatcctgcctgacccgt
>KX349248.1 Synechococcus phage S-RIM2 isolate Np_19_1112, complete genome
1-1tRNA-Val(tac)[13788,13859]
gcccgaatagctcagcggtagagcacctcgtttacaccgagattgtcggg
ggttcgatcccctcttcgggca
1-2tRNA-Leu(taa)[17785,17871]
tgggagtatggcggaatcggtagacgcaccagacttaaaatttgttgagg
gttaacctcgtgagagttcaagtctctctactcctat
1-3tRNA-Thr(tgt)[17928,18000]
gcccccgtagctcagtggtagagcagggcttttgtaaagctcaggtcgca
agttcaaatcttgtcaggggctc
1-4tRNA-Ala(tgc)[18003,18075]
ggggaattagctcagttggtagagcgcctgctttgcaagcaggatgtcag
cggttcgagtccgctattctcca
1-5tRNA-Asn(gtt)[18122,18195]
ttcctcagtagctcagcggcagagccatcgactgttaatcgattggtcgt
aggttcaaatcctacctggggagt
1-6tRNA-Arg(tct)[160075,160149]
tgggtcagtagctcagcggatagagcaaccgccttctaagcggttggtcg
caggttcaaatcctgcctgacccgt
>KX349250.1 Synechococcus phage S-RIM2 isolate Np_23_1112, complete genome
1-1tRNA-Val(tac)[13797,13868]
gcccgaatagctcagcggtagagcacctcgtttacaccgagattgtcggc
ggttcgatcccgtcttcgggca
1-2tRNA-Leu(taa)[17795,17879]
gggagtatggcggaatcggtagacgcaccagacttaaaatttgttgaggg
ttaacctcgtgagagttcaagtctctctactccta
1-3tRNA-Thr(tgt)[17937,18009]
gcccccgtagctcagtggtagagcagggcttttgtaaagctcaggtcgca
agttcaaatcttgtcaggggctc
1-4tRNA-Ala(tgc)[18012,18084]
ggggaattagctcagttggtagagcgcctgctttgcaagcaggatgtcag

cggttcgagtccgctattctcca
1-5tRNA-Asn(gtt)[18131,18204]
ttcctcagtagctcagcggcagagccatcgactgttaatcgattggtcgt
aggttcaaatcctacctggggagt
1-6tRNA-Arg(tct)[161244,161318]
tgggtcagtagctcagcggatagagcaaccgccttctaagcggttggtcg
caggttcaaatcctgcctgacccgt
>KX349251.1 Synechococcus phage S-RIM2 isolate Np_24_1112, complete genome
1-1tRNA-Val(tac)[13794,13865]
gcccgaatagctcagcggtagagcacctcgtttacaccgagattgtcggg
ggttcgatcccctcttcgggca
1-2tRNA-Leu(taa)[17791,17877]
tgggagtatggcggaatcggtagacgcaccagacttaaaatttgttgagg
gttaacctcgtgagagttcaagtctctctactcctat
1-3tRNA-Thr(tgt)[17934,18006]
gcccccgtagctcagtggtagagcagggcttttgtaaagctcaggtcgca
agttcaaatcttgtcaggggctc
1-4tRNA-Ala(tgc)[18009,18081]
ggggaattagctcagttggtagagcgcctgctttgcaagcaggatgtcag
cggttcgagtccgctattctcca
1-5tRNA-Asn(gtt)[18128,18201]
ttcctcagtagctcagcggcagagccatcgactgttaatcgattggtcgt
aggttcaaatcctacctggggagt
1-6tRNA-Arg(tct)[161240,161314]
tgggtcagtagctcagcggatagagcaaccgccttctaagcggttggtcg
caggttcaaatcctgcctgacccgt
>KX349252.1 Synechococcus phage S-RIM2 isolate Np_31_1112, complete genome
1-1tRNA-Val(tac)[13782,13853]
gcccgaatagctcagcggtagagcacctcgtttacaccgagattgtcggg
ggttcgatcccctcttcgggca
1-2tRNA-Leu(taa)[17780,17864]
gggagtatggcggaatcggtagacgcaccagacttaaaatttgttgaggg
ttaacctcgtgagagttcaagtctctctactccta
1-3tRNA-Thr(tgt)[17922,17994]
gcccccgtagctcagtggtagagcagggcttttgtaaagctcaggtcgca
agttcaaatcttgtcaggggctc
1-4tRNA-Ala(tgc)[17997,18069]
ggggaattagctcagttggtagagcgcctgctttgcaagcaggatgtcag
cggttcgagtccgctattctcca
1-5tRNA-Asn(gtt)[18116,18189]
ttcctcagtagctcagcggcagagccatcgactgttaatcgattggtcgt
aggttcaaatcctacctggggagt
1-6tRNA-Arg(tct)[161231,161305]
tgggtcagtagctcagcggatagagcaaccgccttctaagcggttggtcg

caggttcaaatcctgcctgacccgt
>KX349253.1 Synechococcus phage S-RIM2 isolate Np_33_0912, complete genome
1-1tRNA-Val(tac)[13791,13862]
gcccgaatagctcagcggtagagcacctcgtttacaccgagattgtcggc
ggttcgatcccgtcttcgggca
1-2tRNA-Leu(taa)[17788,17874]
tgggagtatggcggaatcggtagacgcaccagacttaaaatttgttgagg
gttaacctcgtgagagttcaagtctctctactcctat
1-3tRNA-Thr(tgt)[17931,18003]
gcccccgtagctcagtggtagagcagggcttttgtaaagctcaggtcgca
agttcaaatcttgtcaggggctc
1-4tRNA-Ala(tgc)[18006,18078]
ggggaattagctcagttggtagagcgcctgctttgcaagcaggatgtcag
cggttcgagtccgctattctcca
1-5tRNA-Asn(gtt)[18125,18198]
ttcctcagtagctcagcggcagagccatcgactgttaatcgattggtcgt
aggttcaaatcctacctggggagt
1-6tRNA-Arg(tct)[161244,161318]
tgggtcagtagctcagcggatagagcaaccgccttctaagcggttggtcg
caggttcaaatcctgcctgacccgt
>KX349254.1 Synechococcus phage S-RIM2 isolate Np_36_1112, complete genome
1-1tRNA-Val(tac)[13803,13874]
gcccgaatagctcagcggtagagcacctcgtttacaccgagattgtcggc
ggttcgatcccgtcttcgggca
1-2tRNA-Leu(taa)[17800,17886]
tgggagtatggcggaatcggtagacgcaccagacttaaaatttgttgagg
gttaacctcgtgagagttcaagtctctctactcctat
1-3tRNA-Thr(tgt)[17943,18015]
gcccccgtagctcagtggtagagcagggcttttgtaaagctcaggtcgca
agttcaaatcttgtcaggggctc
1-4tRNA-Ala(tgc)[18018,18090]
ggggaattagctcagttggtagagcgcctgctttgcaagcaggatgtcag
cggttcgagtccgctattctcca
1-5tRNA-Asn(gtt)[18137,18210]
ttcctcagtagctcagcggcagagccatcgactgttaatcgattggtcgt
aggttcaaatcctacctggggagt
1-6tRNA-Arg(tct)[161247,161321]
tgggtcagtagctcagcggatagagcaaccgccttctaagcggttggtcg
caggttcaaatcctgcctgacccgt
>KX349255.1 Synechococcus phage S-RIM2 isolate RW_01_0709, complete genome
1-1tRNA-Val(tac)[13791,13862]
gcccgaatagctcagcggtagagcacctcgtttacaccgagattgtcggc
ggttcgatcccgtcttcgggca
1-2tRNA-Leu(taa)[17789,17873]

gggagtatggcggaatcggtagacgcaccagacttaaaatttgttgaggg
ttaacctcgtgagagttcaagtctctctactccta
1−3tRNA−Thr(tgt)[17931,18003]
gcccccgtagctcagtggtagagcagggcttttgtaaagctcaggtcgca
agttcaaatcttgtcaggggctc
1−4tRNA−Ala(tgc)[18006,18078]
ggggaattagctcagttggtagagcgcctgctttgcaagcaggatgtcag
cggttcgagtccgctattctcca
1−5tRNA−Asn(gtt)[18125,18198]
ttcctcagtagctcagcggcagagccatcgactgttaatcgattggtcgt
aggttcaaatcctacctggggagt
1−6tRNA−Arg(tct)[161188,161262]
tgggtcagtagctcagcggatagagcaaccgccttctaagcggttggtcg
caggttcaaatcctgcctgacccgt
>KX349256.1 Synechococcus phage S−RIM2 isolate RW_02_0113, complete genome
1−1tRNA−Val(tac)[13794,13865]
gcccgaatagctcagcggtagagcacctcgtttacaccgagattgtcggc
ggttcgatcccgtcttcgggca
1−2tRNA−Leu(taa)[17791,17877]
tgggagtatggcggaatcggtagacgcaccagacttaaaatttgttgagg
gttaacctcgtgagagttcaagtctctctactcctat
1−3tRNA−Thr(tgt)[17934,18006]
gcccccgtagctcagtggtagagcagggcttttgtaaagctcaggtcgca
agttcaaatcttgtcaggggctc
1−4tRNA−Ala(tgc)[18009,18081]
ggggaattagctcagttggtagagcgcctgctttgcaagcaggatgtcag
cggttcgagtccgctattctcca
1−5tRNA−Asn(gtt)[18128,18201]
ttcctcagtagctcagcggcagagccatcgactgttaatcgattggtcgt
aggttcaaatcctacctggggagt
1−6tRNA−Arg(tct)[161241,161315]
tgggtcagtagctcagcggatagagcaaccgccttctaagcggttggtcg
caggttcaaatcctgcctgacccgt
>KX349257.1 Synechococcus phage S−RIM2 isolate RW_02_0709, complete genome
1−1tRNA−Val(tac)[13794,13865]
gcccgaatagctcagcggtagagcacctcgtttacaccgagattgtcggc
ggttcgatcccgtcttcgggca
1−2tRNA−Leu(taa)[17791,17877]
tgggagtatggcggaatcggtagacgcaccagacttaaaatttgttgagg
gttaacctcgtgagagttcaagtctctctactcctat
1−3tRNA−Thr(tgt)[17934,18006]
gcccccgtagctcagtggtagagcagggcttttgtaaagctcaggtcgca
agttcaaatcttgtcaggggctc
1−4tRNA−Ala(tgc)[18009,18081]

ggggaattagctcagttggtagagcgcctgctttgcaagcaggatgtcag
cggttcgagtccgctattctcca
1-5tRNA-Asn(gtt)[18128,18201]
ttcctcagtagctcagcggcagagccatcgactgttaatcgattggtcgt
aggttcaaatcctacctggggagt
1-6tRNA-Arg(tct)[161238,161312]
tgggtcagtagctcagcggatagagcaaccgccttctaagcggttggtcg
caggttcaaatcctgcctgacccgt
>KX349258.1 Synechococcus phage S-RIM2 isolate RW_03_0709, complete genome
1-1tRNA-Val(tac)[13794,13865]
gcccgaatagctcagcggtagagcagcacctttacacggtgaatgtcggg
ggttcgatcccctcttcgggca
1-2tRNA-Leu(taa)[17791,17877]
tgggagtatggcggaatcggtagacgcaccagacttaaaatttgttgagg
gttaacctcgtgagagttcaagtctctctactcctat
1-3tRNA-Thr(tgt)[17934,18006]
gcccccgtagctcagtggtagagcagggcttttgtaaagctcaggtcgca
agttcaaatcttgtcaggggctc
1-4tRNA-Ala(tgc)[18009,18081]
ggggaattagctcagttggtagagcgcctgctttgcaagcaggatgtcag
cggttcgagtccgctattctcca
1-5tRNA-Asn(gtt)[18128,18201]
ttcctcagtagctcagcggcagagccatcgactgttaatcgattggtcgt
aggttcaaatcctacctggggagt
1-6tRNA-Arg(tct)[161235,161309]
tgggtcagtagctcagcggatagagcaaccgccttctaagcggttggtcg
caggttcaaatcctgcctgacccgt
>KX349259.1 Synechococcus phage S-RIM2 isolate RW_08_1112, complete genome
1-1tRNA-Val(tac)[13797,13868]
gcccgaatagctcagcggtagagcacctcgtttacaccgagattgtcggc
ggttcgatcccgtcttcgggca
1-2tRNA-Leu(taa)[17794,17880]
tgggagtatggcggaatcggtagacgcaccagacttaaaatttgttgagg
gttaacctcgtgagagttcaagtctctctactcctat
1-3tRNA-Thr(tgt)[17937,18009]
gcccccgtagctcagtggtagagcagggcttttgtaaagctcaggtcgca
agttcaaatcttgtcaggggctc
1-4tRNA-Ala(tgc)[18012,18084]
ggggaattagctcagttggtagagcgcctgctttgcaagcaggatgtcag
cggttcgagtccgctattctcca
1-5tRNA-Asn(gtt)[18131,18204]
ttcctcagtagctcagcggcagagccatcgactgttaatcgattggtcgt
aggttcaaatcctacctggggagt
1-6tRNA-Arg(tct)[160080,160154]

tgggtcagtagctcagcggatagagcaaccgccttctaagcggttggtcg
caggttcaaatcctgcctgacccgt
>KX349260.1 Synechococcus phage S-RIM2 isolate RW_11_0905, complete genome
1-1tRNA-Val(tac)[13794,13865]
gcccgaatagctcagcggtagagcacctcgtttacaccgagattgtcggg
ggttcgatcccctcttcgggca
1-2tRNA-Leu(taa)[17791,17877]
tgggagtatggcggaatcggtagacgcaccagacttaaaatttgttgagg
gttaacctcgtgagagttcaagtctctctactcctat
1-3tRNA-Thr(tgt)[17934,18006]
gcccccgtagctcagtggtagagcagggcttttgtaaagctcaggtcgca
agttcaaatcttgtcaggggctc
1-4tRNA-Ala(tgc)[18009,18081]
ggggaattagctcagttggtagagcgcctgctttgcaagcaggatgtcag
cggttcgagtccgctattctcca
1-5tRNA-Asn(gtt)[18128,18201]
ttcctcagtagctcagcggcagagccatcgactgttaatcgattggtcgt
aggttcaaatcctacctggggagt
1-6tRNA-Arg(tct)[161242,161316]
tgggtcagtagctcagcggatagagcaaccgccttctaagcggttggtcg
caggttcaaatcctgcctgacccgt
>KX349261.1 Synechococcus phage S-RIM2 isolate RW_12_0113, complete genome
1-1tRNA-Val(tac)[13797,13868]
gcccgaatagctcagcggtagagcacctcgtttacaccgagattgtcggc
ggttcgatcccgtcttcgggca
1-2tRNA-Leu(taa)[17794,17880]
tgggagtatggcggaatcggtagacgcaccagacttaaaatttgttgagg
gttaacctcgtgagagttcaagtctctctactcctat
1-3tRNA-Thr(tgt)[17937,18009]
gcccccgtagctcagtggtagagcagggcttttgtaaagctcaggtcgca
agttcaaatcttgtcaggggctc
1-4tRNA-Ala(tgc)[18012,18084]
ggggaattagctcagttggtagagcgcctgctttgcaagcaggatgtcag
cggttcgagtccgctattctcca
1-5tRNA-Asn(gtt)[18131,18204]
ttcctcagtagctcagcggcagagccatcgactgttaatcgattggtcgt
aggttcaaatcctacctggggagt
1-6tRNA-Arg(tct)[160702,160776]
tgggtcagtagctcagcggatagagcaaccgccttctaagcggttggtcg
caggttcaaatcctgcctgacccgt
>KX349262.1 Synechococcus phage S-RIM2 isolate RW_12_0709, complete genome
1-1tRNA-Val(tac)[13794,13865]
gcccgaatagctcagcggtagagcacctcgtttacaccgagattgtcggg
ggttcgatcccctcttcgggca

1-2tRNA-Leu(taa)[17791,17877]
tgggagtatggcggaatcggtagacgcaccagacttaaaatttgttgagg
gttaacctcgtgagagttcaagtctctctactcctat
1-3tRNA-Thr(tgt)[17934,18006]
gcccccgtagctcagtggtagagcagggcttttgtaaagctcaggtcgca
agttcaaatcttgtcaggggctc
1-4tRNA-Ala(tgc)[18009,18081]
ggggaattagctcagttggtagagcgcctgctttgcaagcaggatgtcag
cggttcgagtccgctattctcca
1-5tRNA-Asn(gtt)[18128,18201]
ttcctcagtagctcagcggcagagccatcgactgttaatcgattggtcgt
aggttcaaatcctacctggggagt
1-6tRNA-Arg(tct)[160952,161026]
tgggtcagtagctcagcggatagagcaaccgccttctaagcggttggtcg
caggttcaaatcctgcctgacccgt
>KX349263.1 Synechococcus phage S-RIM2 isolate RW_14_1112, complete genome
1-1tRNA-Val(tac)[13803,13874]
gcccgaatagctcagcggtagagcacctcgtttacaccgagattgtcggg
ggttcgatccccctcttcgggca
1-2tRNA-Leu(taa)[17800,17886]
tgggagtatggcggaatcggtagacgcaccagacttaaaatttgttgagg
gttaacctcgtgagagttcaagtctctctactcctat
1-3tRNA-Thr(tgt)[17943,18015]
gcccccgtagctcagtggtagagcagggcttttgtaaagctcaggtcgca
agttcaaatcttgtcaggggctc
1-4tRNA-Ala(tgc)[18018,18090]
ggggaattagctcagttggtagagcgcctgctttgcaagcaggatgtcag
cggttcgagtccgctattctcca
1-5tRNA-Asn(gtt)[18137,18210]
ttcctcagtagctcagcggcagagccatcgactgttaatcgattggtcgt
aggttcaaatcctacctggggagt
1-6tRNA-Arg(tct)[161250,161324]
tgggtcagtagctcagcggatagagcaaccgccttctaagcggttggtcg
caggttcaaatcctgcctgacccgt
>KX349264.1 Synechococcus phage S-RIM2 isolate RW_16_0905, complete genome
1-1tRNA-Val(tac)[13794,13865]
gcccgaatagctcagcggtagagcacctcgtttacaccgagattgtcggc
ggttcgatcccgtcttcgggca
1-2tRNA-Leu(taa)[17792,17876]
gggagtatggcggaatcggtagacgcaccagacttaaaatttgttgaggg
ttaacctcgtgagagttcaagtctctctactccta
1-3tRNA-Thr(tgt)[17934,18006]
gcccccgtagctcagtggtagagcagggcttttgtaaagctcaggtcgca
agttcaaatcttgtcaggggctc

1-4tRNA-Ala(tgc)[18009,18081]
ggggaattagctcagttggtagagcgcctgctttgcaagcaggatgtcag
cggttcgagtccgctattctcca
1-5tRNA-Asn(gtt)[18128,18201]
ttcctcagtagctcagcggcagagccatcgactgttaatcgattggtcgt
aggttcaaatcctacctggggagt
1-6tRNA-Arg(tct)[161240,161314]
tgggtcagtagctcagcggatagagcaaccgccttctaagcggttggtcg
caggttcaaatcctgcctgacccgt
>KX349265.1 Synechococcus phage S-RIM2 isolate RW_17_0113, complete genome
1-1tRNA-Val(tac)[13785,13856]
gcccgaatagctcagcggtagagcacctcgtttacaccgagattgtcggc
ggttcgatcccgtcttcgggca
1-2tRNA-Leu(taa)[17782,17868]
tgggagtatggcggaatcggtagacgcaccagacttaaaatttgttgagg
gttaacctcgtgagagttcaagtctctctactcctat
1-3tRNA-Thr(tgt)[17925,17997]
gcccccgtagctcagtggtagagcagggcttttgtaaagctcaggtcgca
agttcaaatcttgtcaggggctc
1-4tRNA-Ala(tgc)[18000,18072]
ggggaattagctcagttggtagagcgcctgctttgcaagcaggatgtcag
cggttcgagtccgctattctcca
1-5tRNA-Asn(gtt)[18119,18192]
ttcctcagtagctcagcggcagagccatcgactgttaatcgattggtcgt
aggttcaaatcctacctggggagt
1-6tRNA-Arg(tct)[161231,161305]
tgggtcagtagctcagcggatagagcaaccgccttctaagcggttggtcg
caggttcaaatcctgcctgacccgt
>KX349266.1 Synechococcus phage S-RIM2 isolate RW_26_0905, complete genome
1-1tRNA-Val(tac)[13794,13865]
gcccgaatagctcagcggtagagcacctcgtttacaccgagattgtcggg
ggttcgatcccctcttcgggca
1-2tRNA-Leu(taa)[17792,17876]
gggagtatggcggaatcggtagacgcaccagacttaaaatttgttgaggg
ttaacctcgtgagagttcaagtctctctactccta
1-3tRNA-Thr(tgt)[17934,18006]
gcccccgtagctcagtggtagagcagggcttttgtaaagctcaggtcgca
agttcaaatcttgtcaggggctc
1-4tRNA-Ala(tgc)[18009,18081]
ggggaattagctcagttggtagagcgcctgctttgcaagcaggatgtcag
cggttcgagtccgctattctcca
1-5tRNA-Asn(gtt)[18128,18201]
ttcctcagtagctcagcggcagagccatcgactgttaatcgattggtcgt
aggttcaaatcctacctggggagt

1-6tRNA-Arg(tct)[160078,160152]
tgggtcagtagctcagcggatagagcaaccgccttctaagcggttggtcg
caggttcaaatcctgcctgacccgt
>KX349267.1 Synechococcus phage S-RIM2 isolate RW_29_1112, complete genome
1-1tRNA-Val(tac)[13797,13868]
gcccgaatagctcagcggtagagcacctcgtttacaccgagattgtcggg
ggttcgatcccctcttcgggca
1-2tRNA-Leu(taa)[17795,17879]
gggagtatggcggaatcggtagacgcaccagacttaaaatttgttgaggg
ttaacctcgtgagagttcaagtctctctactccta
1-3tRNA-Thr(tgt)[17937,18009]
gcccccgtagctcagtggtagagcagggcttttgtaaagctcaggtcgca
agttcaaatcttgtcaggggctc
1-4tRNA-Ala(tgc)[18012,18084]
ggggaattagctcagttggtagagcgcctgctttgcaagcaggatgtcag
cggttcgagtccgctattctcca
1-5tRNA-Asn(gtt)[18131,18204]
ttcctcagtagctcagcggcagagccatcgactgttaatcgattggtcgt
aggttcaaatcctacctggggagt
1-6tRNA-Arg(tct)[161241,161315]
tgggtcagtagctcagcggatagagcaaccgccttctaagcggttggtcg
caggttcaaatcctgcctgacccgt
>KX349268.1 Synechococcus phage S-RIM2 isolate RW_30_0905, complete genome
1-1tRNA-Val(tac)[13797,13868]
gcccgaatagctcagcggtagagcacctcgtttacaccgagattgtcggc
ggttcgatcccgtcttcgggca
1-2tRNA-Leu(taa)[17794,17880]
tgggagtatggcggaatcggtagacgcaccagacttaaaatttgttgagg
gttaacctcgtgagagttcaagtctctctactcctat
1-3tRNA-Thr(tgt)[17937,18009]
gcccccgtagctcagtggtagagcagggcttttgtaaagctcaggtcgca
agttcaaatcttgtcaggggctc
1-4tRNA-Ala(tgc)[18012,18084]
ggggaattagctcagttggtagagcgcctgctttgcaagcaggatgtcag
cggttcgagtccgctattctcca
1-5tRNA-Asn(gtt)[18131,18204]
ttcctcagtagctcagcggcagagccatcgactgttaatcgattggtcgt
aggttcaaatcctacctggggagt
1-6tRNA-Arg(tct)[161243,161317]
tgggtcagtagctcagcggatagagcaaccgccttctaagcggttggtcg
caggttcaaatcctgcctgacccgt
>KX349269.1 Synechococcus phage S-RIM2 isolate RW_34_0905, complete genome
1-1tRNA-Val(tac)[13796,13867]
gcccgaatagctcagcggtagagcacctcgtttacaccgagattgtcggc

ggttcgatcccgtcttcgggca
1-2tRNA-Leu(taa)[17793,17879]
tgggagtatggcggaatcggtagacgcaccagacttaaaatttgttgagg
gttaacctcgtgagagttcaagtctctctactcctat
1-3tRNA-Thr(tgt)[17936,18008]
gcccccgtagctcagtggtagagcagggcttttgtaaagctcaggtcgca
agttcaaatcttgtcaggggctc
1-4tRNA-Ala(tgc)[18011,18083]
ggggaattagctcagttggtagagcgcctgctttgcaagcaggatgtcag
cggttcgagtccgctattctcca
1-5tRNA-Asn(gtt)[18130,18203]
ttcctcagtagctcagcggcagagccatcgactgttaatcgattggtcgt
aggttcaaatcctacctggggagt
1-6tRNA-Arg(tct)[161241,161315]
tgggtcagtagctcagcggatagagcaaccgccttctaagcggttggtcg
caggttcaaatcctgcctgacccgt
>KX349270.1 Synechococcus phage S-RIM2 isolate RW_40_1112, complete genome
1-1tRNA-Val(tac)[13797,13868]
gcccgaatagctcagcggtagagcacctcgtttacaccgagattgtcggc
ggttcgatcccgtcttcgggca
1-2tRNA-Leu(taa)[17794,17880]
tgggagtatggcggaatcggtagacgcaccagacttaaaatttgttgagg
gttaacctcgtgagagttcaagtctctctactcctat
1-3tRNA-Thr(tgt)[17937,18009]
gcccccgtagctcagtggtagagcagggcttttgtaaagctcaggtcgca
agttcaaatcttgtcaggggctc
1-4tRNA-Ala(tgc)[18012,18084]
ggggaattagctcagttggtagagcgcctgctttgcaagcaggatgtcag
cggttcgagtccgctattctcca
1-5tRNA-Asn(gtt)[18131,18204]
ttcctcagtagctcagcggcagagccatcgactgttaatcgattggtcgt
aggttcaaatcctacctggggagt
1-6tRNA-Arg(tct)[161243,161317]
tgggtcagtagctcagcggatagagcaaccgccttctaagcggttggtcg
caggttcaaatcctgcctgacccgt
>KX349271.1 Synechococcus phage S-RIM2 isolate Sn_25_0709, complete genome
1-1tRNA-Val(tac)[13800,13871]
gcccgaatagctcagcggtagagcacctcgtttacaccgagattgtcggc
ggttcgatcccgtcttcgggca
1-2tRNA-Leu(taa)[17797,17883]
tgggagtatggcggaatcggtagacgcaccagacttaaaatttgttgagg
gttaacctcgtgagagttcaagtctctctactcctat
1-3tRNA-Thr(tgt)[17940,18012]
gcccccgtagctcagtggtagagcagggcttttgtaaagctcaggtcgca

agttcaaatcttgtcaggggctc
1–4tRNA–Ala(tgc)[18015,18087]
ggggaattagctcagttggtagagcgcctgctttgcaagcaggatgtcag
cggttcgagtccgctattctcca
1–5tRNA–Asn(gtt)[18134,18207]
ttcctcagtagctcagcggcagagccatcgactgttaatcgattggtcgt
aggttcaaatcctacctggggagt
1–6tRNA–Arg(tct)[161246,161320]
tgggtcagtagctcagcggatagagcaaccgccttctaagcggttggtcg
caggttcaaatcctgcctgacccgt

>KX349272.1 Synechococcus phage S–RIM2 isolate W1_01_0709, complete genome

1–1tRNA–Val(tac)[13794,13865]
gcccgaatagctcagcggtagagcacctcgtttacaccgagattgtcggc
ggttcgatcccgtcttcgggca
1–2tRNA–Leu(taa)[17791,17877]
tgggagtatggcggaatcggtagacgcaccagacttaaaatttgttgagg
gttaacctcgtgagagttcaagtctctctactcctat
1–3tRNA–Thr(tgt)[17934,18006]
gcccccgtagctcagtggtagagcagggcttttgtaaagctcaggtcgca
agttcaaatcttgtcaggggctc
1–4tRNA–Ala(tgc)[18009,18081]
ggggaattatctcagttggtagagcgcctgctttgcaagcaggatgtcag
cggttcgagtccgctattctcca
1–5tRNA–Asn(gtt)[18128,18201]
ttcctcagtagctcagcggcagagccatcgactgttaatcgattggtcgt
aggttcaaatcctacctggggagt
1–6tRNA–Arg(tct)[161238,161312]
tgggtcagtagctcagcggatagagcaaccgccttctaagcggttggtcg
caggttcaaatcctgcctgacccgt

>KX349273.1 Synechococcus phage S–RIM2 isolate W1_01_0910, complete genome

1–1tRNA–Val(tac)[13895,13966]
gcccgaatagctcagcggtagagcacctcgtttacaccgagattgtcggc
ggttcgatcccgtcttcgggca
1–2tRNA–Leu(taa)[17892,17978]
tgggagtatggcggaatcggtagacgcaccagacttaaaatttgttgagg
gttaacctcgtgagagttcaagtctctctactcctat
1–3tRNA–Thr(tgt)[18035,18108]
gcccccgtagctcagtggtagagcagggcttttgtaaagctcaggtcgca
agttcaaatcttgtcaggggctcc
1–4tRNA–Ala(tgc)[18110,18185]
tggggaattagctcagtctggtagagcgcctgctttgcaagcaggatgtc
agcggttcgagtccgctattctccat
1–5tRNA–Asn(gtt)[18231,18304]
ttcctcagtagctcagcggcagagctatcgactgttaatcgattggtcgt

aggttcaaatcctacctggggagt
1-6tRNA-Arg(tct)[161117,161191]
tgggtcagtagctcagcggatagagcaaccgccttctaagcggttggtcg
caggttcaaatcctgcctgacccgt
>KX349274.1 Synechococcus phage S-RIM2 isolate W1_03_0709, complete genome
1-1tRNA-Val(tac)[13895,13966]
gcccgaatagctcagcggtagagcacctcgtttacaccgagattgtcggc
ggttcgatcccgtcttcgggca
1-2tRNA-Leu(taa)[17892,17978]
tgggagtatggcggaatcggtagacgcaccagacttaaaatttgttgagg
gttaacctcgtgagagttcaagtctctctactcctat
1-3tRNA-Thr(tgt)[18035,18107]
gcccccgtagctcagtggtagagcagggcttttgtaaagctcaggtcgca
agttcaaatcttgtcaggggctc
1-4tRNA-Ala(tgc)[18453,18526]
ggggaattagctcagtctggtagagcgcctgctttgcaagcaggatgtca
gcggttcgagtccgctattctcca
1-5tRNA-Asn(gtt)[18573,18646]
ttcctcagtagctcagcggcagagctatcgactgttaatcgattggtcgt
aggttcaaatcctacctggggagt
1-6tRNA-Arg(tct)[161455,161529]
tgggtcagtagctcagcggatagagcaaccgccttctaagcggttggtcg
caggttcaaatcctgcctgacccgt
>KX349275.1 Synechococcus phage S-RIM2 isolate W1_08_0709, complete genome
1-1tRNA-Val(tac)[13895,13966]
gcccgaatagctcagcggtagagcacctcgtttacaccgagattgtcggc
ggttcgatcccgtcttcgggca
1-2tRNA-Leu(taa)[17892,17978]
tgggagtatggcggaatcggtagacgcaccagacttaaaatttgttgagg
gttaacctcgtgagagttcaagtctctctactcctat
1-3tRNA-Thr(tgt)[18035,18108]
gcccccgtagctcagtggtagagcagggcttttgtaaagctcaggtcgca
agttcaaatcttgtcaggggctcc
1-4tRNA-Ala(tgc)[18110,18184]
tggggaattagctcagttggtagagcgcctgctttgcaagcaggatgtca
gcggttcgagtccgctattctccat
1-5tRNA-Asn(gtt)[18230,18303]
ttcctcagtagctcagcggcagagctatcgactgttaatcgattggtcgt
aggttcaaatcctacctggggagt
1-6tRNA-Arg(tct)[161068,161142]
tgggtcagtagctcagcggatagagcaaccgccttctaagcggttggtcg
caggttcaaatcctgcctgacccgt
>KX349276.1 Synechococcus phage S-RIM2 isolate W1_09_0709, complete genome
1-1tRNA-Val(tac)[13794,13865]

gcccgaatagctcagcggtagagcacctcgtttacaccgagattgtcggc
ggttcgatcccgtcttcgggca
1−2tRNA−Leu(taa)[17791,17877]
tgggagtatggcggaatcggtagacgcaccagacttaaaatttgttgagg
gttaacctcgtgagagttcaagtctctctactcctat
1−3tRNA−Thr(tgt)[17934,18006]
gcccccgtagctcagtggtagagcagggcttttgtaaagctcaggtcgca
agttcaaatcttgtcaggggctc
1−4tRNA−Ala(tgc)[18009,18081]
ggggaattagctcagttggtagagcgcctgctttgcaagcaggatgtcag
cggttcgagtccgctattctcca
1−5tRNA−Asn(gtt)[18128,18201]
ttcctcagtagctcagcggcagagccatcgactgttaatcgattggtcgt
aggttcaaatcctacctggggagt
1−6tRNA−Arg(tct)[161239,161313]
tgggtcagtagctcagcggatagagcaaccgccttctaagcggttggtcg
caggttcaaatcctgcctgacccgt
>KX349277.1 Synechococcus phage S−RIM2 isolate W1_12_0909, complete genome
1−1tRNA−Val(tac)[13895,13966]
gcccgaatagctcagcggtagagcacctcgtttacaccgagattgtcggc
ggttcgatcccgtcttcgggca
1−2tRNA−Leu(taa)[17892,17978]
tgggagtatggcggaatcggtagacgcaccagacttaaaatttgttgagg
gttaacctcgtgagagttcaagtctctctactcctat
1−3tRNA−Thr(tgt)[18035,18108]
gcccccgtagctcagtggtagagcagggcttttgtaaagctcaggtcgca
agttcaaatcttgtcaggggctcc
1−4tRNA−Ala(tgc)[18110,18184]
tggggaattagctcagttggtagagcgcctgctttgcaagcaggatgtca
gcggttcgagtccgctattctccat
1−5tRNA−Asn(gtt)[18230,18303]
ttcctcagtagctcagcggcagagctatcgactgttaatcgattggtcgt
aggttcaaatcctacctggggagt
1−6tRNA−Arg(tct)[161116,161190]
tgggtcagtagctcagcggatagagcaaccgccttctaagcggttggtcg
caggttcaaatcctgcctgacccgt
>KX349278.1 Synechococcus phage S−RIM2 isolate W1_13_0709, complete genome
1−1tRNA−Val(tac)[13895,13966]
gcccgaatagctcagcggtagagcacctcgtttacaccgagattgtcggc
ggttcgatcccgtcttcgggca
1−2tRNA−Leu(taa)[17892,17978]
tgggagtatggcggaatcggtagacgcaccagacttaaaatttgttgagg
gttaacctcgtgagagttcaagtctctctactcctat
1−3tRNA−Thr(tgt)[18035,18108]

gcccccgtagctcagtggtagagcagggcttttgtaaagctcaggtcgca
agttcaaatcttgtcaggggctcc
1–4tRNA–Ala(tgc)[18110,18184]
tggggaattagctcagttggtagagcgcctgctttgcaagcaggatgtca
gcggttcgagtccgctattctccat
1–5tRNA–Asn(gtt)[18230,18303]
ttcctcagtagctcagcggcagagctatcgactgttaatcgattggtcgt
aggttcaaatcctacctggggagt
1–6tRNA–Arg(tct)[161119,161193]
tgggtcagtagctcagcggatagagcaaccgccttctaagcggttggtcg
caggttcaaatcctgcctgacccgt
>KX349279.1 Synechococcus phage S–RIM2 isolate W1_16_0709, complete genome
1–1tRNA–Val(tac)[13797,13868]
gcccgaatagctcagcggtagagcacctcgtttacaccgagattgtcggc
ggttcgatcccgtcttcgggca
1–2tRNA–Leu(taa)[17794,17880]
tgggagtatggcggaatcggtagacgcaccagacttaaaatttgttgagg
gttaacctcgtgagagttcaagtctctctactcctat
1–3tRNA–Thr(tgt)[17937,18009]
gcccccgtagctcagtggtagagcagggcttttgtaaagctcaggtcgca
agttcaaatcttgtcaggggctc
1–4tRNA–Ala(tgc)[18012,18084]
ggggaattagctcagttggtagagcgcctgctttgcaagcaggatgtcag
cggttcgagtccgctattctcca
1–5tRNA–Asn(gtt)[18131,18204]
ttcctcagtagctcagcggcagagccatcgactgttaatcgattggtcgt
aggttcaaatcctacctggggagt
1–6tRNA–Arg(tct)[161243,161317]
tgggtcagtagctcagcggatagagcaaccgccttctaagcggttggtcg
caggttcaaatcctgcctgacccgt
>KX349280.1 Synechococcus phage S–RIM2 isolate W2_02_0709, complete genome
1–1tRNA–Val(tac)[13895,13966]
gcccgaatagctcagcggtagagcacctcgtttacaccgagattgtcggc
ggttcgatcccgtcttcgggca
1–2tRNA–Leu(taa)[17892,17978]
tgggagtatggcggaatcggtagacgcaccagacttaaaatttgttgagg
gttaacctcgtgagagttcaagtctctctactcctat
1–3tRNA–Thr(tgt)[18035,18108]
gcccccgtagctcagtggtagagcagggcttttgtaaagctcaggtcgca
agttcaaatcttgtcaggggctcc
1–4tRNA–Ala(tgc)[18110,18184]
tggggaattagctcagttggtagagcgcctgctttgcaagcaggatgtca
gcggttcgagtccgctattctccat
1–5tRNA–Asn(gtt)[18230,18303]

ttcctcagtagctcagcggcagagctatcgactgttaatcgattggtcgt
aggttcaaatcctacctggggagt
1–6tRNA–Arg(tct)[161114,161188]
tgggtcagtagctcagcggatagagcaaccgccttctaagcggttggtcg
caggttcaaatcctgcctgacccgt
>KX349281.1 Synechococcus phage S–RIM2 isolate W2_13_0910, complete genome
1–1tRNA–Val(tac)[13794,13865]
gcccgaatagctcagcggtagagcacctcgtttacaccgagattgtcggc
ggttcgatcccgtcttcgggca
1–2tRNA–Leu(taa)[17791,17877]
tgggagtatggcggaatcggtagacgcaccagacttaaaatttgttgagg
gttaacctcgtgagagttcaagtctctctactcctat
1–3tRNA–Thr(tgt)[17934,18006]
gcccccgtagctcagtggtagagcagggcttttgtaaagctcaggtcgca
agttcaaatcttgtcaggggctc
1–4tRNA–Ala(tgc)[18009,18081]
ggggaattagctcagttggtagagcgcctgctttgcaagcaggatgtcag
cggttcgagtccgctattctcca
1–5tRNA–Asn(gtt)[18128,18201]
ttcctcagtagctcagcggcagagccatcgactgttaatcgattggtcgt
aggttcaaatcctacctggggagt
1–6tRNA–Arg(tct)[161246,161320]
tgggtcagtagctcagcggatagagcaaccgccttctaagcggttggtcg
caggttcaaatcctgcctgacccgt
>KX349282.1 Synechococcus phage S–RIM2 isolate W2_14_0910, complete genome
1–1tRNA–Val(tac)[13803,13874]
gcccgaatagctcagcggtagagcacctcgtttacaccgagattgtcggc
ggttcgatcccgtcttcgggca
1–2tRNA–Leu(taa)[17801,17885]
gggagtatggcggaatcggtagacgcaccagacttaaaatttgttgaggg
ttaacctcgtgagagttcaagtctctctactccta
1–3tRNA–Thr(tgt)[17943,18015]
gcccccgtagctcagtggtagagcagggcttttgtaaagctcaggtcgca
agttcaaatcttgtcaggggctc
1–4tRNA–Ala(tgc)[18018,18090]
ggggaattagctcagttggtagagcgcctgctttgcaagcaggatgtcag
cggttcgagtccgctattctcca
1–5tRNA–Asn(gtt)[18137,18210]
ttcctcagtagctcagcggcagagccatcgactgttaatcgattggtcgt
aggttcaaatcctacctggggagt
1–6tRNA–Arg(tct)[161255,161329]
tgggtcagtagctcagcggatagagcaaccgccttctaagcggttggtcg
caggttcaaatcctgcctgacccgt
>KX349283.1 Synechococcus phage S–RIM2 isolate W2_32_0910, complete genome

1−1tRNA−Val(tac)[13883,13954]
gcccgaatagctcagcggtagagcacctcgtttacaccgagattgtcggc
ggttcgatcccgtcttcgggca
1−2tRNA−Leu(taa)[17880,17966]
tgggagtatggcggaatcggtagacgcaccagacttaaaatttgttgagg
gttaacctcgtgagagttcaagtctctctactcctat
1−3tRNA−Thr(tgt)[18023,18096]
gcccccgtagctcagtggtagagcagggcttttgtaaagctcaggtcgca
agttcaaatcttgtcaggggctcc
1−4tRNA−Ala(tgc)[18098,18172]
tggggaattagctcagttggtagagcgcctgctttgcaagcaggatgtca
gcggttcgagtccgctattctccat
1−5tRNA−Asn(gtt)[18218,18291]
ttcctcagtagctcagcggcagagctatcgactgttaatcgattggtcgt
aggttcaaatcctacctggggagt
1−6tRNA−Arg(tct)[161054,161128]
tgggtcagtagctcagcggatagagcaaccgccttctaagcggttggtcg
caggttcaaatcctgcctgacccgt
>KX349284.1 Synechococcus phage S−RIM2 isolate W2_39_0910, complete genome
1−1tRNA−Val(tac)[13904,13975]
gcccgaatagctcagcggtagagcacctcgtttacaccgagattgtcggc
ggttcgatcccgtcttcgggca
1−2tRNA−Leu(taa)[17901,17987]
tgggagtatggcggaatcggtagacgcaccagacttaaaatttgttgagg
gttaacctcgtgagagttcaagtctctctactcctat
1−3tRNA−Thr(tgt)[18044,18117]
gcccccgtagctcagtggtagagcagggcttttgtaaagctcaggtcgca
agttcaaatcttgtcaggggctcc
1−4tRNA−Ala(tgc)[18119,18194]
tggggaattagctcagtctggtagagcgcctgctttgcaagcaggatgtc
agcggttcgagtccgctattctccat
1−5tRNA−Asn(gtt)[18240,18313]
ttcctcagtagctcagcggcagagctatcgactgttaatcgattggtcgt
aggttcaaatcctacctggggagt
1−6tRNA−Arg(tct)[161141,161215]
tgggtcagtagctcagcggatagagcaaccgccttctaagcggttggtcg
caggttcaaatcctgcctgacccgt
>KJ019031.1 Synechococcus phage ACG−2014d isolate Syn7803C48, complete genome
1−1tRNA−Val(tac)[13618,13691]
tgggtgattaactcagtggtagagtgactgctttacacgcagtaggtcac
tggttcaaatccagtattacccat
1−2tRNA−Arg(tct)[153722,153796]
tgggcaagtagctcagtggatagagcatcgcacttctaatgcgttggtcg
ggggttcaaatccctccttgcccgt

1-3tRNA-Asn(gtt)[153817,153890]
ttcccaagtagctcagtggcagagccgccgactgttaatcggctggtcgc
tggttcaaatccagccttgggagt
>KJ019035.1 Synechococcus phage ACG-2014d isolate Syn7803C55, complete genome
1-1tRNA-Val(tac)[11908,11981]
tgggtgattaactcagtggtagagtgactgctttacacgcagtaggtcac
tggttcaaatccagtattacccat
1-2tRNA-Arg(tct)[152060,152134]
tgggcaagtagctcagtggatagagcatcgcacttctaatgcgttggtcg
ggggttcaaatccctccttgcccgt
1-3tRNA-Asn(gtt)[152155,152228]
ttcccaagtagctcagtggcagagccgccgactgttaatcggctggtcgc
tggttcaaatccagccttgggagt
>KJ019036.1 Synechococcus phage ACG-2014d isolate Syn7803C57, complete genome
1-1tRNA-Val(tac)[13612,13685]
tgggtgattaactcagtggtagagtgactgctttacacgcagtaggtcac
tggttcaaatccagtattacccat
1-2tRNA-Arg(tct)[153716,153790]
tgggcaagtagctcagtggatagagcatcgcacttctaatgcgttggtcg
ggggttcaaatccctccttgcccgt
1-3tRNA-Asn(gtt)[153811,153884]
ttcccaagtagctcagtggcagagccgccgactgttaatcggctggtcgc
tggttcaaatccagccttgggagt
>KJ019047.1 Synechococcus phage ACG-2014d isolate Syn7803C73, complete genome
1-1tRNA-Val(tac)[13618,13691]
tgggtgattaactcagtggtagagtgactgctttacacgcagtaggtcac
tggttcaaatccagtattacccat
1-2tRNA-Arg(tct)[153722,153796]
tgggcaagtagctcagtggatagagcatcgcacttctaatgcgttggtcg
ggggttcaaatccctccttgcccgt
1-3tRNA-Asn(gtt)[153817,153890]
ttcccaagtagctcagtggcagagccgccgactgttaatcggctggtcgc
tggttcaaatccagccttgggagt
>KJ019048.1 Synechococcus phage ACG-2014d isolate Syn7803C75, complete genome
1-1tRNA-Val(tac)[13618,13691]
tgggtgattaactcagtggtagagtgactgctttacacgcagtaggtcac
tggttcaaatccagtattacccat
1-2tRNA-Arg(tct)[153484,153558]
tgggcaagtagctcagtggatagagcatcgcacttctaatgcgttggtcg
ggggttcaaatccctccttgcccgt
1-3tRNA-Asn(gtt)[153579,153652]
ttcccaagtagctcagtggcagagccgccgactgttaatcggctggtcgc
tggttcaaatccagccttgggagt
>KJ019062.1 Synechococcus phage ACG-2014d isolate Syn7803C93, complete genome

1−1tRNA−Val(tac)[13621,13694]
tgggtgattaactcagtggtagagtgactgctttacacgcagtaggtcac
tggttcaaatccagtattacccat
1−2tRNA−Arg(tct)[153722,153796]
tgggcaagtagctcagtggatagagcatcgcacttctaatgcgttggtcg
ggggttcaaatccctccttgcccgt
1−3tRNA−Asn(gtt)[153817,153890]
ttcccaagtagctcagtggcagagccgccgactgttaatcggctggtcgc
tggttcaaatccagccttgggagt
>KJ019072.1 Synechococcus phage ACG−2014d isolate Syn7803US108, complete genome
1−1tRNA−Val(tac)[13618,13691]
tgggtgattaactcagtggtagagtgactgctttacacgcagtaggtcac
tggttcaaatccagtattacccat
1−2tRNA−Arg(tct)[153723,153797]
tgggcaagtagctcagtggatagagcatcgcacttctaatgcgttggtcg
ggggttcaaatccctccttgcccgt
1−3tRNA−Asn(gtt)[153818,153891]
ttcccaagtagctcagtggcagagccgccgactgttaatcggctggtcgc
tggttcaaatccagccttgggagt
>KJ019073.1 Synechococcus phage ACG−2014d isolate Syn7803US109, complete genome
1−1tRNA−Val(tac)[13621,13694]
tgggtgattaactcagtggtagagtgactgctttacacgcagtaggtcac
tggttcaaatccagtattacccat
1−2tRNA−Arg(tct)[153731,153805]
tgggcaagtagctcagtggatagagcatcgcacttctaatgcgttggtcg
ggggttcaaatccctccttgcccgt
1−3tRNA−Asn(gtt)[153826,153899]
ttcccaagtagctcagtggcagagccgccgactgttaatcggctggtcgc
tggttcaaatccagccttgggagt
>KJ019075.1 Synechococcus phage ACG−2014d isolate Syn7803US111, complete genome
1−1tRNA−Val(tac)[13618,13691]
tgggtgattaactcagtggtagagtgactgctttacacgcagtaggtcac
tggttcaaatccagtattacccat
1−2tRNA−Arg(tct)[153715,153789]
tgggcaagtagctcagtggatagagcatcgcacttctaatgcgttggtcg
ggggttcaaatccctccttgcccgt
1−3tRNA−Asn(gtt)[153810,153883]
ttcccaagtagctcagtggcagagccgccgactgttaatcggctggtcgc
tggttcaaatccagccttgggagt
>KJ019078.1 Synechococcus phage ACG−2014d isolate Syn7803US114, complete genome
1−1tRNA−Val(tac)[13618,13691]
tgggtgattaactcagtggtagagtgactgctttacacgcagtaggtcac
tggttcaaatccagtattacccat
1−2tRNA−Arg(tct)[153718,153792]

tgggcaagtagctcagtggatagagcatcgcacttctaatgcgttggtcg
ggggttcaaatccctccttgcccgt
1-3tRNA-Asn(gtt)[153813,153886]
ttcccaagtagctcagtggcagagccgccgactgttaatcggctggtcgc
tggttcaaatccagccttgggagt
>KJ019079.1 Synechococcus phage ACG-2014d isolate Syn7803US115, complete genome
1-1tRNA-Val(tac)[13618,13691]
tgggtgattaactcagtggtagagtgactgctttacacgcagtaggtcac
tggttcaaatccagtattacccat
1-2tRNA-Arg(tct)[153722,153796]
tgggcaagtagctcagtggatagagcatcgcacttctaatgcgttggtcg
ggggttcaaatccctccttgcccgt
1-3tRNA-Asn(gtt)[153817,153890]
ttcccaagtagctcagtggcagagccgccgactgttaatcggctggtcgc
tggttcaaatccagccttgggagt
>KJ019112.1 Synechococcus phage ACG-2014d isolate Syn7803US59, complete genome
1-1tRNA-Val(tac)[13618,13691]
tgggtgattaactcagtggtagagtgactgctttacacgcagtaggtcac
tggttcaaatccagtattacccat
1-2tRNA-Arg(tct)[153722,153796]
tgggcaagtagctcagtggatagagcatcgcacttctaatgcgttggtcg
ggggttcaaatccctccttgcccgt
1-3tRNA-Asn(gtt)[153817,153890]
ttcccaagtagctcagtggcagagccgccgactgttaatcggctggtcgc
tggttcaaatccagccttgggagt
>KJ019113.1 Synechococcus phage ACG-2014d isolate Syn7803US5, complete genome
1-1tRNA-Val(tac)[13624,13697]
tgggtgattaactcagtggtagagtgactgctttacacgcagtaggtcac
tggttcaaatccagtattacccat
1-2tRNA-Arg(tct)[153868,153942]
tgggcaagtagctcagtggatagagcatcgcacttctaatgcgttggtcg
ggggttcaaatccctccttgcccgt
1-3tRNA-Asn(gtt)[153963,154036]
ttcccaagtagctcagtggcagagccgccgactgttaatcggctggtcgc
tggttcaaatccagccttgggagt
>KJ019115.1 Synechococcus phage ACG-2014d isolate Syn7803US61, complete genome
1-1tRNA-Val(tac)[13627,13700]
tgggtgattaactcagtggtagagtgactgctttacacgcagtaggtcac
tggttcaaatccagtattacccat
1-2tRNA-Arg(tct)[153652,153726]
tgggcaagtagctcagtggatagagcatcgcacttctaatgcgttggtcg
ggggttcaaatccctccttgcccgt
1-3tRNA-Asn(gtt)[153747,153820]
ttcccaagtagctcagtggcagagccgccgactgttaatcggctggtcgc

tggttcaaatccagccttgggagt
>KJ019117.1 Synechococcus phage ACG–2014d isolate Syn7803US63, complete genome
1–1tRNA–Val(tac)[13603,13676]
tgggtgattaactcagtggtagagtgactgctttacacgcagtaggtcac
tggttcaaatccagtattacccat
1–2tRNA–Arg(tct)[153769,153843]
tgggcaagtagctcagtggatagagcatcgcacttctaatgcgttggtcg
ggggttcaaatccctccttgcccgt
1–3tRNA–Asn(gtt)[153864,153937]
ttcccaagtagctcagtggcagagccgccgactgttaatcggctggtcgc
tggttcaaatccagccttgggagt
>KJ019119.1 Synechococcus phage ACG–2014d isolate Syn7803US65, complete genome
1–1tRNA–Val(tac)[13624,13697]
tgggtgattaactcagtggtagagtgactgctttacacgcagtaggtcac
tggttcaaatccagtattacccat
1–2tRNA–Arg(tct)[153777,153851]
tgggcaagtagctcagtggatagagcatcgcacttctaatgcgttggtcg
ggggttcaaatccctccttgcccgt
1–3tRNA–Asn(gtt)[153872,153945]
ttcccaagtagctcagtggcagagccgccgactgttaatcggctggtcgc
tggttcaaatccagccttgggagt
>KJ019120.1 Synechococcus phage ACG–2014d isolate Syn7803US71, complete genome
1–1tRNA–Val(tac)[13621,13694]
tgggtgattaactcagtggtagagtgactgctttacacgcagtaggtcac
tggttcaaatccagtattacccat
1–2tRNA–Arg(tct)[153744,153818]
tgggcaagtagctcagtggatagagcatcgcacttctaatgcgttggtcg
ggggttcaaatccctccttgcccgt
1–3tRNA–Asn(gtt)[153839,153912]
ttcccaagtagctcagtggcagagccgccgactgttaatcggctggtcgc
tggttcaaatccagccttgggagt
>KJ019125.1 Synechococcus phage ACG–2014d isolate Syn7803US82, complete genome
1–1tRNA–Val(tac)[13621,13694]
tgggtgattaactcagtggtagagtgactgctttacacgcagtaggtcac
tggttcaaatccagtattacccat
1–2tRNA–Arg(tct)[153820,153894]
tgggcaagtagctcagtggatagagcatcgcacttctaatgcgttggtcg
ggggttcaaatccctccttgcccgt
1–3tRNA–Asn(gtt)[153915,153988]
ttcccaagtagctcagtggcagagccgccgactgttaatcggctggtcgc
tggttcaaatccagccttgggagt
>KJ019127.1 Synechococcus phage ACG–2014d isolate Syn7803US85, complete genome
1–1tRNA–Val(tac)[13624,13697]
tgggtgattaactcagtggtagagtgactgctttacacgcagtaggtcac

tggttcaaatccagtattacccat
1-2tRNA-Arg(tct)[153708,153782]
tgggcaagtagctcagtggatagagcatcgcacttctaatgcgttggtcg
ggggttcaaatccctccttgcccgt
1-3tRNA-Asn(gtt)[153803,153876]
ttcccaagtagctcagtggcagagccgccgactgttaatcggctggtcgc
tggttcaaatccagccttgggagt
>KJ019129.1 Synechococcus phage ACG-2014d isolate Syn7803US89, complete genome
1-1tRNA-Val(tac)[13621,13694]
tgggtgattaactcagtggtagagtgactgctttacacgcagtaggtcac
tggttcaaatccagtattacccat
1-2tRNA-Arg(tct)[153753,153827]
tgggcaagtagctcagtggatagagcatcgcacttctaatgcgttggtcg
ggggttcaaatccctccttgcccgt
1-3tRNA-Asn(gtt)[153848,153921]
ttcccaagtagctcagtggcagagccgccgactgttaatcggctggtcgc
tggttcaaatccagccttgggagt
>KJ019130.1 Synechococcus phage ACG-2014d isolate Syn7803US94, complete genome
1-1tRNA-Val(tac)[13622,13695]
tgggtgattaactcagtggtagagtgactgctttacacgcagtaggtcac
tggttcaaatccagtattacccat
1-2tRNA-Arg(tct)[153821,153895]
tgggcaagtagctcagtggatagagcatcgcacttctaatgcgttggtcg
ggggttcaaatccctccttgcccgt
1-3tRNA-Asn(gtt)[153916,153989]
ttcccaagtagctcagtggcagagccgccgactgttaatcggctggtcgc
tggttcaaatccagccttgggagt
>KJ019139.1 Synechococcus phage ACG-2014d isolate Syn7803C108, complete genome
1-1tRNA-Val(tac)[13618,13691]
tgggtgattaactcagtggtagagtgactgctttacacgcagtaggtcac
tggttcaaatccagtattacccat
1-2tRNA-Arg(tct)[153817,153891]
tgggcaagtagctcagtggatagagcatcgcacttctaatgcgttggtcg
ggggttcaaatccctccttgcccgt
1-3tRNA-Asn(gtt)[153912,153985]
ttcccaagtagctcagtggcagagccgccgactgttaatcggctggtcgc
tggttcaaatccagccttgggagt
>KJ019160.1 Synechococcus phage ACG-2014d isolate Syn7803C35, complete genome
1-1tRNA-Val(tac)[13621,13694]
tgggtgattaactcagtggtagagtgactgctttacacgcagtaggtcac
tggttcaaatccagtattacccat
1-2tRNA-Arg(tct)[153821,153895]
tgggcaagtagctcagtggatagagcatcgcacttctaatgcgttggtcg
ggggttcaaatccctccttgcccgt

1−3tRNA−Asn(gtt)[153916,153989]
ttcccaagtagctcagtggcagagccgccgactgttaatcggctggtcgc
tggttcaaatccagccttgggagt
>KJ019165.1 Synechococcus phage ACG−2014d isolate Syn7803C40, complete genome
1−1tRNA−Val(tac)[13620,13693]
tgggtgattaactcagtggtagagtgactgctttacacgcagtaggtcac
tggttcaaatccagtattacccat
1−2tRNA−Arg(tct)[153819,153893]
tgggcaagtagctcagtggatagagcatcgcacttctaatgcgttggtcg
ggggttcaaatccctccttgcccgt
1−3tRNA−Asn(gtt)[153914,153987]
ttcccaagtagctcagtggcagagccgccgactgttaatcggctggtcgc
tggttcaaatccagccttgggagt
>KJ019032.1 Synechococcus phage ACG−2014d isolate Syn7803C49, complete genome
1−1tRNA−Val(tac)[13624,13697]
tgggtgattaactcagtggtagagtgactgctttacacgcagtaggtcac
tggttcaaatccagtattacccat
1−2tRNA−Arg(tct)[153844,153918]
tgggcaagtagctcagtggatagagcatcgcacttctaatgcgttggtcg
ggggttcaaatccctccttgcccgt
1−3tRNA−Asn(gtt)[153939,154012]
ttcccaagtagctcagtggcagagccgccgactgttaatcggctggtcgc
tggttcaaatccagccttgggagt
>KJ019070.1 Synechococcus phage ACG−2014d isolate Syn7803US104, complete genome
1−1tRNA−Val(tac)[13621,13694]
tgggtgattaactcagtggtagagtgactgctttacacgcagtaggtcac
tggttcaaatccagtattacccat
1−2tRNA−Arg(tct)[153868,153942]
tgggcaagtagctcagtggatagagcatcgcacttctaatgcgttggtcg
ggggttcaaatccctccttgcccgt
1−3tRNA−Asn(gtt)[153963,154036]
ttcccaagtagctcagtggcagagccgccgactgttaatcggctggtcgc
tggttcaaatccagccttgggagt
>KJ019080.1 Synechococcus phage ACG−2014d isolate Syn7803US116, complete genome
1−1tRNA−Val(tac)[13618,13691]
tgggtgattaactcagtggtagagtgactgctttacacgcagtaggtcac
tggttcaaatccagtattacccat
1−2tRNA−Arg(tct)[153508,153582]
tgggcaagtagctcagtggatagagcatcgcacttctaatgcgttggtcg
ggggttcaaatccctccttgcccgt
1−3tRNA−Asn(gtt)[153603,153676]
ttcccaagtagctcagtggcagagccgccgactgttaatcggctggtcgc
tggttcaaatccagccttgggagt
>KJ019121.1 Synechococcus phage ACG−2014d isolate Syn7803US78, complete genome

1-1tRNA-Val(tac)[13615,13688]
tgggtgattaactcagtggtagagtgactgctttacacgcagtaggtcac
tggttcaaatccagtattacccat
1-2tRNA-Arg(tct)[153719,153793]
tgggcaagtagctcagtggatagagcatcgcacttctaatgcgttggtcg
ggggttcaaatccctccttgcccgt
1-3tRNA-Asn(gtt)[153814,153887]
ttcccaagtagctcagtggcagagccgccgactgttaatcggctggtcgc
tggttcaaatccagccttgggagt
>KJ019124.1 Synechococcus phage ACG-2014d isolate Syn7803US80, complete genome
1-1tRNA-Val(tac)[13624,13697]
tgggtgattaactcagtggtagagtgactgctttacacgcagtaggtcac
tggttcaaatccagtattacccat
1-2tRNA-Arg(tct)[153871,153945]
tgggcaagtagctcagtggatagagcatcgcacttctaatgcgttggtcg
ggggttcaaatccctccttgcccgt
1-3tRNA-Asn(gtt)[153966,154039]
ttcccaagtagctcagtggcagagccgccgactgttaatcggctggtcgc
tggttcaaatccagccttgggagt
>KJ019118.1 Synechococcus phage ACG-2014d isolate Syn7803US64, complete genome
1-1tRNA-Val(tac)[13618,13691]
tgggtgattaactcagtggtagagtgactgctttacacgcagtaggtcac
tggttcaaatccagtattacccat
1-2tRNA-Arg(tct)[153817,153891]
tgggcaagtagctcagtggatagagcatcgcacttctaatgcgttggtcg
ggggttcaaatccctccttgcccgt
1-3tRNA-Asn(gtt)[153912,153985]
ttcccaagtagctcagtggcagagccgccgactgttaatcggctggtcgc
tggttcaaatccagccttgggagt
>KJ019126.1 Synechococcus phage ACG-2014d isolate Syn7803US83, complete genome
1-1tRNA-Val(tac)[13618,13691]
tgggtgattaactcagtggtagagtgactgctttacacgcagtaggtcac
tggttcaaatccagtattacccat
1-2tRNA-Arg(tct)[153814,153888]
tgggcaagtagctcagtggatagagcatcgcacttctaatgcgttggtcg
ggggttcaaatccctccttgcccgt
1-3tRNA-Asn(gtt)[153909,153982]
ttcccaagtagctcagtggcagagccgccgactgttaatcggctggtcgc
tggttcaaatccagccttgggagt
>KJ019131.1 Synechococcus phage ACG-2014d isolate Syn7803US95, complete genome
1-1tRNA-Val(tac)[13618,13691]
tgggtgattaactcagtggtagagtgactgctttacacgcagtaggtcac
tggttcaaatccagtattacccat
1-2tRNA-Arg(tct)[153723,153797]

tgggcaagtagctcagtggatagagcatcgcacttctaatgcgttggtcg
ggggttcaaatccctccttgcccgt
1−3tRNA−Asn(gtt)[153818,153891]
ttcccaagtagctcagtggcagagccgccgactgttaatcggctggtcgc
tggttcaaatccagccttgggagt
>KJ019140.1 Synechococcus phage ACG−2014d isolate Syn7803C109, complete genome
1−1tRNA−Val(tac)[13618,13691]
tgggtgattaactcagtggtagagtgactgctttacacgcagtaggtcac
tggttcaaatccagtattacccat
1−2tRNA−Arg(tct)[153726,153800]
tgggcaagtagctcagtggatagagcatcgcacttctaatgcgttggtcg
ggggttcaaatccctccttgcccgt
1−3tRNA−Asn(gtt)[153821,153894]
ttcccaagtagctcagtggcagagccgccgactgttaatcggctggtcgc
tggttcaaatccagccttgggagt
>KJ019162.1 Synechococcus phage ACG−2014d isolate Syn7803C37, complete genome
1−1tRNA−Val(tac)[13618,13691]
tgggtgattaactcagtggtagagtgactgctttacacgcagtaggtcac
tggttcaaatccagtattacccat
1−2tRNA−Arg(tct)[153865,153939]
tgggcaagtagctcagtggatagagcatcgcacttctaatgcgttggtcg
ggggttcaaatccctccttgcccgt
1−3tRNA−Asn(gtt)[153960,154033]
ttcccaagtagctcagtggcagagccgccgactgttaatcggctggtcgc
tggttcaaatccagccttgggagt
>KJ019164.1 Synechococcus phage ACG−2014d isolate Syn7803C39, complete genome
1−1tRNA−Val(tac)[13684,13757]
tgggtgattaactcagtggtagagtgactgctttacacgcagtaggtcac
tggttcaaatccagtattacccat
1−2tRNA−Arg(tct)[153788,153862]
tgggcaagtagctcagtggatagagcatcgcacttctaatgcgttggtcg
ggggttcaaatccctccttgcccgt
1−3tRNA−Asn(gtt)[153883,153956]
ttcccaagtagctcagtggcagagccgccgactgttaatcggctggtcgc
tggttcaaatccagccttgggagt
>KJ019034.1 Synechococcus phage ACG−2014d isolate Syn7803C54, complete genome
1−1tRNA−Val(tac)[13618,13691]
tgggtgattaactcagtggtagagtgactgctttacacgcagtaggtcac
tggttcaaatccagtattacccat
1−2tRNA−Arg(tct)[153817,153891]
tgggcaagtagctcagtggatagagcatcgcacttctaatgcgttggtcg
ggggttcaaatccctccttgcccgt
1−3tRNA−Asn(gtt)[153912,153985]
ttcccaagtagctcagtggcagagccgccgactgttaatcggctggtcgc

tggttcaaatccagccttgggagt
>KJ019046.1 Synechococcus phage ACG−2014d isolate Syn7803C72, complete genome
1−1tRNA−Val(tac)[13618,13691]
tgggtgattaactcagtggtagagtgactgctttacacgcagtaggtcac
tggttcaaatccagtattacccat
1−2tRNA−Arg(tct)[153819,153893]
tgggcaagtagctcagtggatagagcatcgcacttctaatgcgttggtcg
ggggttcaaatccctccttgcccgt
1−3tRNA−Asn(gtt)[153914,153987]
ttcccaagtagctcagtggcagagccgccgactgttaatcggctggtcgc
tggttcaaatccagccttgggagt
>KJ019050.1 Synechococcus phage ACG−2014d isolate Syn7803C77, complete genome
1−1tRNA−Val(tac)[13618,13691]
tgggtgattaactcagtggtagagtgactgctttacacgcagtaggtcac
tggttcaaatccagtattacccat
1−2tRNA−Arg(tct)[153818,153892]
tgggcaagtagctcagtggatagagcatcgcacttctaatgcgttggtcg
ggggttcaaatccctccttgcccgt
1−3tRNA−Asn(gtt)[153913,153986]
ttcccaagtagctcagtggcagagccgccgactgttaatcggctggtcgc
tggttcaaatccagccttgggagt
>KJ019057.1 Synechococcus phage ACG−2014d isolate Syn7803C89, complete genome
1−1tRNA−Val(tac)[13618,13691]
tgggtgattaactcagtggtagagtgactgctttacacgcagtaggtcac
tggttcaaatccagtattacccat
1−2tRNA−Arg(tct)[153722,153796]
tgggcaagtagctcagtggatagagcatcgcacttctaatgcgttggtcg
ggggttcaaatccctccttgcccgt
1−3tRNA−Asn(gtt)[153817,153890]
ttcccaagtagctcagtggcagagccgccgactgttaatcggctggtcgc
tggttcaaatccagccttgggagt
>KJ019074.1 Synechococcus phage ACG−2014d isolate Syn7803US110, complete genome
1−1tRNA−Val(tac)[13618,13691]
tgggtgattaactcagtggtagagtgactgctttacacgcagtaggtcac
tggttcaaatccagtattacccat
1−2tRNA−Arg(tct)[153722,153796]
tgggcaagtagctcagtggatagagcatcgcacttctaatgcgttggtcg
ggggttcaaatccctccttgcccgt
1−3tRNA−Asn(gtt)[153817,153890]
ttcccaagtagctcagtggcagagccgccgactgttaatcggctggtcgc
tggttcaaatccagccttgggagt
>KJ019077.1 Synechococcus phage ACG−2014d isolate Syn7803US113, complete genome
1−1tRNA−Val(tac)[13621,13694]
tgggtgattaactcagtggtagagtgactgctttacacgcagtaggtcac

tggttcaaatccagtattacccat
1−2tRNA−Arg(tct)[153820,153894]
tgggcaagtagctcagtggatagagcatcgcacttctaatgcgttggtcg
ggggttcaaatccctccttgcccgt
1−3tRNA−Asn(gtt)[153915,153988]
ttcccaagtagctcagtggcagagccgccgactgttaatcggctggtcgc
tggttcaaatccagccttgggagt
>KJ019083.1 Synechococcus phage ACG−2014d isolate Syn7803US122, complete genome
1−1tRNA−Val(tac)[13618,13691]
tgggtgattaactcagtggtagagtgactgctttacacgcagtaggtcac
tggttcaaatccagtattacccat
1−2tRNA−Arg(tct)[153722,153796]
tgggcaagtagctcagtggatagagcatcgcacttctaatgcgttggtcg
ggggttcaaatccctccttgcccgt
1−3tRNA−Asn(gtt)[153817,153890]
ttcccaagtagctcagtggcagagccgccgactgttaatcggctggtcgc
tggttcaaatccagccttgggagt
>AP013540.1 Uncultured Mediterranean phage uvMED DNA, complete genome, group G8, isolate: uvMED−CGR−C62A−MedDCM−OCT−S28−C10
1−1tRNA−Leu(taa)c[26490,26573]
gctcgggtgatggaattggtagacatacgggacttaaaatcccgggaccg
ttaggtcgtgagggttcgagtccctccctgagca
>KJ019037.1 Synechococcus phage ACG−2014f isolate Syn7803C58, complete genome
1−1tRNA−Arg(tct)[129914,129987]
gggtcagtagctcagatggatagagcaattcacttctaatgaattggtcg
ggggttcgagtccctcctgacccg
1−2tRNA−Asn(gtt)[130201,130272]
tccacaatagctcagcggtagagtcggcgactgttaatcgccttgtccct
ggttcgaatccaggttgtggag
>KJ019045.1 Synechococcus phage ACG−2014f isolate Syn7803C6, complete genome
1−1tRNA−Arg(tct)[130564,130637]
gggttaatagctcagctggacagagcaacgctcttctaaagcgtcggtcg
ttggttcgaatccaacttaacccg
1−2tRNA−Asn(gtt)[130851,130922]
tccacaatagctcagcggtagagtcggcgactgttaatcgccttgtccct
ggttcgaatccaggttgtggag
>KJ019052.1 Synechococcus phage ACG−2014f isolate Syn7803C7, complete genome
1−1tRNA−Arg(tct)[127858,127931]
gggttaatagctcagctggacagagcaacgctcttctaaagcgtcggtcg
ttggttcgaatccaacttaacccg
1−2tRNA−Asn(gtt)[128145,128216]
tccacaatagctcagcggtagagtcggcgactgttaatcgccttgtccct
ggttcgaatccaggttgtggag
>KJ019053.1 Synechococcus phage ACG−2014f isolate Syn7803C80, complete genome

1−1tRNA−Arg(tct)[130447,130520]
gggtcagtagctcagatggatagagcaattcacttctaatgaattggtcg
ggggttcgagtccctcctgacccg
1−2tRNA−Asn(gtt)[130734,130805]
tccacaatagctcagcggtagagtcggcgactgttaatcgccttgtccct
ggttcgaatccaggttgtggag
>KJ019058.1 Synechococcus phage ACG−2014f isolate Syn7803C8, complete genome
1−1tRNA−Arg(tct)[128055,128128]
gggttaatagctcagctggacagagcaacgctcttctaaagcgtcggtcg
ttggttcgaatccaacttaacccg
1−2tRNA−Asn(gtt)[128342,128413]
tccacaatagctcagcggtagagtcggcgactgttaatcgccttgtccct
ggttcgaatccaggttgtggag
>KJ019066.1 Synechococcus phage ACG−2014f isolate Syn7803C9, complete genome
1−1tRNA−Arg(tct)[130889,130962]
gggtcagtagctcagatggatagagcaattcacttctaatgaattggtcg
ggggttcgagtccctcctgacccg
1−2tRNA−Asn(gtt)[131176,131247]
tccacaatagctcagcggtagagtcggcgactgttaatcgccttgtccct
ggttcgaatccaggttgtggag
>KJ019085.1 Synechococcus phage ACG−2014f isolate Syn7803US13, complete genome
1−1tRNA−Arg(tct)[126042,126115]
gggttaatagctcagctggacagagcaacgctcttctaaagcgtcggtcg
ttggttcgaatccaacttaacccg
1−2tRNA−Asn(gtt)[126329,126400]
tccacaatagctcagcggtagagtcggcgactgttaatcgccttgtccct
ggttcgaatccaggttgtggag
>KJ019086.1 Synechococcus phage ACG−2014f isolate Syn7803US17, complete genome
1−1tRNA−Arg(tct)[128795,128868]
gggttaatagctcagctggacagagcaacgctcttctaaagcgtcggtcg
ttggttcgaatccaacttaacccg
1−2tRNA−Asn(gtt)[129082,129153]
tccacaatagctcagcggtagagtcggcgactgttaatcgccttgtccct
ggttcgaatccaggttgtggag
>KJ019090.1 Synechococcus phage ACG−2014f isolate Syn7803US24, complete genome
1−1tRNA−Arg(tct)[128060,128133]
gggttaatagctcagctggacagagcaatactcttctaaagtatcggtcg
ttggttcgaatccaacttaacccg
1−2tRNA−Asn(gtt)[128347,128418]
tccacaatagctcagcggtagagtcggcgactgttaatcgccttgtccct
ggttcgaatccaggttgtggag
>KJ019091.1 Synechococcus phage ACG−2014f isolate Syn7803US26, complete genome
1−1tRNA−Arg(tct)[126211,126284]
gggttaatagctcagctggacagagcaacgctcttctaaagcgtcggtcg

ttggttcgaatccaacttaacccg
1−2tRNA−Asn(gtt)[126498,126569]
tccacaatagctcagcggtagagtcggcgactgttaatcgccttgtccct
ggttcgaatccaggttgtggag
>KJ019092.1 Synechococcus phage ACG−2014f isolate Syn7803US2, complete genome
1−1tRNA−Arg(tct)[129445,129518]
gggttaatagctcagctggacagagcaacgctcttctaaagcgtcggtcg
ttggttcgaatccaacttaacccg
1−2tRNA−Asn(gtt)[129732,129803]
tccacaatagctcagcggtagagtcggcgactgttaatcgccttgtccct
ggttcgaatccaggttgtggag
>KJ019093.1 Synechococcus phage ACG−2014f isolate Syn7803US30, complete genome
1−1tRNA−Arg(tct)[126955,127028]
gggttaatagctcagctggacagagcaacgctcttctaaagcgtcggtcg
ttggttcgaatccaacttaacccg
1−2tRNA−Asn(gtt)[127242,127313]
tccacaatagctcagcggtagagtcggcgactgttaatcgccttgtccct
ggttcgaatccaggttgtggag
>KJ019095.1 Synechococcus phage ACG−2014f isolate Syn7803US34, complete genome
1−1tRNA−Arg(tct)[130385,130458]
gggttaatagctcagctggacagagcaacgctcttctaaagcgtcggtcg
ttggttcgaatccaacttaacccg
1−2tRNA−Asn(gtt)[130672,130743]
tccacaatagctcagcggtagagtcggcgactgttaatcgccttgtccct
ggttcgaatccaggttgtggag
>KJ019096.1 Synechococcus phage ACG−2014f isolate Syn7803US36, complete genome
1−1tRNA−Arg(tct)[128792,128865]
gggttaatagctcagctggacagagcaacgctcttctaaagcgtcggtcg
ttggttcgaatccaacttaacccg
1−2tRNA−Asn(gtt)[129079,129150]
tccacaatagctcagcggtagagtcggcgactgttaatcgccttgtccct
ggttcgaatccaggttgtggag
>KJ019097.1 Synechococcus phage ACG−2014f isolate Syn7803US37, complete genome
1−1tRNA−Arg(tct)[131822,131895]
gggtcagtagctcagatggatagagcaattcacttctaatgaattggtcg
ggggttcgagtccctcctgacccg
1−2tRNA−Asn(gtt)[132109,132180]
tccacaatagctcagcggtagagtcggcgactgttaatcgccttgtccct
ggttcgaatccaggttgtggag
>KJ019098.1 Synechococcus phage ACG−2014f isolate Syn7803US39, complete genome
1−1tRNA−Arg(tct)[131659,131732]
gggtcagtagctcagatggatagagcaattcacttctaatgaattggtcg
ggggttcgagtccctcctgacccg
1−2tRNA−Asn(gtt)[131946,132017]

tccacaatagctcagcggtagagtcggcgactgttaatcgccttgtccct
ggttcgaatccaggttgtggag
>KJ019099.1 Synechococcus phage ACG−2014f isolate Syn7803US3, complete genome
1−1tRNA−Arg(tct)[130232,130305]
gggtcagtagctcagatggatagagcaattcacttctaatgaattggtcg
ggggttcgagtccctcctgacccg
1−2tRNA−Asn(gtt)[130519,130590]
tccacaatagctcagcggtagagtcggcgactgttaatcgccttgtccct
ggttcgaatccaggttgtggag
>KJ019100.1 Synechococcus phage ACG−2014f isolate Syn7803US40, complete genome
1−1tRNA−Arg(tct)[128559,128632]
gggttaatagctcagctggacagagcaacgctcttctaaagcgtcggtcg
ttggttcgaatccaacttaacccg
1−2tRNA−Asn(gtt)[128846,128917]
tccacaatagctcagcggtagagtcggcgactgttaatcgccttgtccct
ggttcgaatccaggttgtggag
>KJ019101.1 Synechococcus phage ACG−2014f isolate Syn7803US42, complete genome
1−1tRNA−Arg(tct)[128398,128471]
gggtcagtagctcagatggatagagcaattcacttctaatgaattggtcg
ggggttcgagtccctcctgacccg
1−2tRNA−Asn(gtt)[128685,128756]
tccacaatagctcagcggtagagtcggcgactgttaatcgccttgtccct
ggttcgaatccaggttgtggag
>KJ019102.1 Synechococcus phage ACG−2014f isolate Syn7803US43, complete genome
1−1tRNA−Arg(tct)[129626,129699]
gggtcagtagctcagatggatagagcaattcacttctaatgaattggtcg
ggggttcgagtccctcctgacccg
1−2tRNA−Asn(gtt)[129913,129984]
tccacaatagctcagcggtagagtcggcgactgttaatcgccttgtccct
ggttcgaatccaggttgtggag
>KJ019103.1 Synechococcus phage ACG−2014f isolate Syn7803US44, complete genome
1−1tRNA−Arg(tct)[128491,128564]
gggttaatagctcagctggacagagcaatactcttctaaagtatcggtcg
ttggttcgaatccaacttaacccg
1−2tRNA−Asn(gtt)[128778,128849]
tccacaatagctcagcggtagagtcggcgactgttaatcgccttgtccct
ggttcgaatccaggttgtggag
>KJ019105.1 Synechococcus phage ACG−2014f isolate Syn7803US4, complete genome
1−1tRNA−Arg(tct)[126959,127032]
gggttaatagctcagctggacagagcaacgctcttctaaagcgtcggtcg
ttggttcgaatccaacttaacccg
1−2tRNA−Asn(gtt)[127246,127317]
tccacaatagctcagcggtagagtcggcgactgttaatcgccttgtccct
ggttcgaatccaggttgtggag

>KJ019106.1 Synechococcus phage ACG-2014f isolate Syn7803US50, complete genome
1-1tRNA-Arg(tct)[128265,128338]
gggtcagtagctcagatggatagagcaattcacttctaatgaattggtcg
ggggttcgagtccctcctgacccg
1-2tRNA-Asn(gtt)[128552,128623]
tccacaatagctcagcggtagagtcggcgactgttaatcgccttgtccct
ggttcgaatccaggttgtggag
>KJ019107.1 Synechococcus phage ACG-2014f isolate Syn7803US52, complete genome
1-1tRNA-Arg(tct)[126562,126635]
gggttaatagctcagctggacagagcaacgctcttctaaagcgtcggtcg
ttggttcgaatccaacttaacccg
1-2tRNA-Asn(gtt)[126849,126920]
tccacaatagctcagcggtagagtcggcgactgttaatcgccttgtccct
ggttcgaatccaggttgtggag
>KJ019111.1 Synechococcus phage ACG-2014f isolate Syn7803US57, complete genome
1-1tRNA-Arg(tct)[126245,126318]
gggttaatagctcagctggacagagcaacgctcttctaaagcgtcggtcg
ttggttcgaatccaacttaacccg
1-2tRNA-Asn(gtt)[126532,126603]
tccacaatagctcagcggtagagtcggcgactgttaatcgccttgtccct
ggttcgaatccaggttgtggag
>KJ019123.1 Synechococcus phage ACG-2014f isolate Syn7803US7, complete genome
1-1tRNA-Arg(tct)[125134,125207]
gggttaatagctcagctggacagagcaacgctcttctaaagcgtcggtcg
ttggttcgaatccaacttaacccg
1-2tRNA-Asn(gtt)[125421,125492]
tccacaatagctcagcggtagagtcggcgactgttaatcgccttgtccct
ggttcgaatccaggttgtggag
>KJ019141.1 Synechococcus phage ACG-2014f isolate Syn7803C10, complete genome
1-1tRNA-Arg(tct)[126776,126849]
gggttaatagctcagctggacagagcaacgctcttctaaagcgtcggtcg
ttggttcgaatccaacttaacccg
1-2tRNA-Asn(gtt)[127063,127134]
tccacaatagctcagcggtagagtcggcgactgttaatcgccttgtccct
ggttcgaatccaggttgtggag
>KJ019142.1 Synechococcus phage ACG-2014f isolate Syn7803C11, complete genome
1-1tRNA-Arg(tct)[126895,126968]
gggttaatagctcagctggacagagcaacgctcttctaaagcgtcggtcg
ttggttcgaatccaacttaacccg
1-2tRNA-Asn(gtt)[127182,127253]
tccacaatagctcagcggtagagtcggcgactgttaatcgccttgtccct
ggttcgaatccaggttgtggag
>KJ019143.1 Synechococcus phage ACG-2014f isolate Syn7803C12, complete genome
1-1tRNA-Arg(tct)[128169,128242]

gggttaatagctcagctggacagagcaacgctcttctaaagcgtcggtcg
ttggttcgaatccaacttaacccg
1−2tRNA−Asn(gtt)[128456,128527]
tccacaatagctcagcggtagagtcggcgactgttaatcgccttgtccct
ggttcgaatccaggttgtggag
>KJ019144.1 Synechococcus phage ACG−2014f isolate Syn7803C14, complete genome
1−1tRNA−Arg(tct)[126240,126313]
gggttaatagctcagctggacagagcaacgctcttctaaagcgtcggtcg
ttggttcgaatccaacttaacccg
1−2tRNA−Asn(gtt)[126527,126598]
tccacaatagctcagcggtagagtcggcgactgttaatcgccttgtccct
ggttcgaatccaggttgtggag
>KJ019145.1 Synechococcus phage ACG−2014f isolate Syn7803C15, complete genome
1−1tRNA−Arg(tct)[129910,129983]
gggtcagtagctcagatggatagagcaattcacttctaatgaattggtcg
ggggttcgagtccctcctgacccg
1−2tRNA−Asn(gtt)[130197,130268]
tccacaatagctcagcggtagagtcggcgactgttaatcgccttgtccct
ggttcgaatccaggttgtggag
>KJ019146.1 Synechococcus phage ACG−2014f isolate Syn7803C16, complete genome
1−1tRNA−Arg(tct)[129741,129814]
gggttaatagctcagctggacagagcaacgctcttctaaagcgtcggtcg
ttggttcgaatccaacttaacccg
1−2tRNA−Asn(gtt)[130028,130099]
tccacaatagctcagcggtagagtcggcgactgttaatcgccttgtccct
ggttcgaatccaggttgtggag
>KJ019147.1 Synechococcus phage ACG−2014f isolate Syn7803C17, complete genome
1−1tRNA−Arg(tct)[127192,127265]
gggtcagtagctcagatggatagagcaattcacttctaatgaattggtcg
ggggttcgagtccctcctgacccg
1−2tRNA−Asn(gtt)[127479,127550]
tccacaatagctcagcggtagagtcggcgactgttaatcgccttgtccct
ggttcgaatccaggttgtggag
>KJ019148.1 Synechococcus phage ACG−2014f isolate Syn7803C19, complete genome
1−1tRNA−Arg(tct)[125246,125319]
gggtcagtagctcagatggatagagcaattcacttctaatgaattggtcg
ggggttcgagtccctcctgacccg
1−2tRNA−Asn(gtt)[125533,125604]
tccacaatagctcagcggtagagtcggcgactgttaatcgccttgtccct
ggttcgaatccaggttgtggag
>KJ019149.1 Synechococcus phage ACG−2014f isolate Syn7803C21, complete genome
1−1tRNA−Arg(tct)[127725,127798]
gggtcagtagctcagatggatagagcaattcacttctaatgaattggtcg
ggggttcgagtccctcctgacccg

1−2tRNA−Asn(gtt)[128012,128083]
tccacaatagctcagcggtagagtcggcgactgttaatcgccttgtccct
ggttcgaatccaggttgtggag
>KJ019150.1 Synechococcus phage ACG−2014f isolate Syn7803C22, complete genome
1−1tRNA−Arg(tct)[131303,131376]
gggttaatagctcagctggacagagcaacgctcttctaaagcgtcggtcg
ttggttcgaatccaacttaacccg
1−2tRNA−Asn(gtt)[131590,131661]
tccacaatagctcagcggtagagtcggcgactgttaatcgccttgtccct
ggttcgaatccaggttgtggag
>KJ019151.1 Synechococcus phage ACG−2014f isolate Syn7803C24, complete genome
1−1tRNA−Arg(tct)[128601,128674]
gggttaatagctcagctggacagagcaacgctcttctaaagcgtcggtcg
ttggttcgaatccaacttaacccg
1−2tRNA−Asn(gtt)[128888,128959]
tccacaatagctcagcggtagagtcggcgactgttaatcgccttgtccct
ggttcgaatccaggttgtggag
>KJ019152.1 Synechococcus phage ACG−2014f isolate Syn7803C25, complete genome
1−1tRNA−Arg(tct)[129664,129737]
gggtcagtagctcagatggatagagcaattcacttctaatgaattggtcg
ggggttcgagtccctcctgacccg
1−2tRNA−Asn(gtt)[129951,130022]
tccacaatagctcagcggtagagtcggcgactgttaatcgccttgtccct
ggttcgaatccaggttgtggag
>KJ019155.1 Synechococcus phage ACG−2014f isolate Syn7803C29, complete genome
1−1tRNA−Arg(tct)[126281,126354]
gggttaatagctcagctggacagagcaacgctcttctaaagcgtcggtcg
ttggttcgaatccaacttaacccg
1−2tRNA−Asn(gtt)[126568,126639]
tccacaatagctcagcggtagagtcggcgactgttaatcgccttgtccct
ggttcgaatccaggttgtggag
>KJ019159.1 Synechococcus phage ACG−2014f isolate Syn7803C34, complete genome
1−1tRNA−Arg(tct)[128066,128139]
gggttaatagctcagctggacagagcaatactcttctaaagtatcggtcg
ttggttcgaatccaacttaacccg
1−2tRNA−Asn(gtt)[128353,128424]
tccacaatagctcagcggtagagtcggcgactgttaatcgccttgtccct
ggttcgaatccaggttgtggag
>KJ019026.1 Synechococcus phage ACG−2014a isolate Syn7803C42, complete genome
1−1tRNA−Val(tac)[13070,13141]
gggcgaataactcagcggtagagtgcctcctttacacggagattgtcggg
ggttcgatcccctcttcgccca
1−2tRNA−Leu(taa)[18060,18146]
tgggagtgtggcggaatcggtagacgcaccagacttaaaatctgttgaga

attaatctcgtgggggttcaagtccccccttctcccat
1−3tRNA−Thr(tgt)[18770,18842]
gcctccgtagctcagtggtagagcagggcttttgtaaagctcaggtcgca
agttcaaatcttgtcagaggctc
1−4tRNA−Asn(gtt)[18847,18919]
tcctctatagctcagttggtagagcaggtgactgttaatcaccctgtccc
tggttcgagtccaggtggaggag
1−5tRNA−Arg(tct)[154484,154559]
tgggtcagtagctcagatggatagagcaactgccttctaagcagtcggcc
acaggttcgagtcctgtctgacccgt
>KJ019030.1 Synechococcus phage ACG−2014a isolate Syn7803C47, complete genome
1−1tRNA−Val(tac)[13064,13135]
gggcgaataactcagcggtagagtgcctcctttacacggagattgtcggg
ggttcgatccccctcttcgccca
1−2tRNA−Leu(taa)[18054,18140]
tgggagtgtggcggaatcggtagacgcaccagacttaaaatctgttgaga
attaatctcgtgggggttcaagtccccccttctcccat
1−3tRNA−Thr(tgt)[18764,18836]
gcctccgtagctcagtggtagagcagggcttttgtaaagctcaggtcgca
agttcaaatcttgtcagaggctc
1−4tRNA−Asn(gtt)[18841,18913]
tcctctatagctcagttggtagagcaggtgactgttaatcaccctgtccc
tggttcgagtccaggtggaggag
1−5tRNA−Arg(tct)[154576,154651]
tgggtcagtagctcagatggatagagcaactgccttctaagcagtcggcc
acaggttcgagtcctgtctgacccgt
>KJ019033.1 Synechococcus phage ACG−2014a isolate Syn7803C53, complete genome
1−1tRNA−Val(tac)[13064,13135]
gggcgaataactcagcggtagagtgcctcctttacacggagattgtcggg
ggttcgatccccctcttcgccca
1−2tRNA−Leu(taa)[18054,18140]
tgggagtgtggcggaatcggtagacgcaccagacttaaaatctgttgaga
attaatctcgtgggggttcaagtccccccttctcccat
1−3tRNA−Thr(tgt)[18764,18836]
gcctccgtagctcagtggtagagcagggcttttgtaaagctcaggtcgca
agttcaaatcttgtcagaggctc
1−4tRNA−Asn(gtt)[18841,18913]
tcctctatagctcagttggtagagcaggtgactgttaatcaccctgtccc
tggttcgagtccaggtggaggag
1−5tRNA−Arg(tct)[154326,154401]
tgggtcagtagctcagatggatagagcaactgccttctaagcagtcggcc
acaggttcgagtcctgtctgacccgt
>KJ019038.1 Synechococcus phage ACG−2014a isolate Syn7803C59, complete genome
1−1tRNA−Val(tac)[13064,13135]

gggcgaataactcagcggtagagtgcctcctttacacggagattgtcggg
ggttcgatcccctcttcgccca
1-2tRNA-Leu(taa)[18054,18140]
tgggagtgtggcggaatcggtagacgcaccagacttaaaatctgttgaga
attaatctcgtgggggttcaagtccccccttctcccat
1-3tRNA-Thr(tgt)[18764,18836]
gcctccgtagctcagtggtagagcagggcttttgtaaagctcaggtcgca
agttcaaatcttgtcagaggctc
1-4tRNA-Asn(gtt)[18841,18913]
tcctctatagctcagttggtagagcaggtgactgttaatcaccctgtccc
tggttcgagtccaggtggaggag
1-5tRNA-Arg(tct)[154355,154430]
tgggtcagtagctcagatggatagagcaactgccttctaagcagtcggcc
acaggttcgagtcctgtctgacccgt
>KJ019039.1 Synechococcus phage ACG-2014a isolate Syn7803C60, complete genome
1-1tRNA-Val(tac)[13064,13135]
gggcgaataactcagcggtagagtgcctcctttacacggagattgtcggg
ggttcgatcccctcttcgccca
1-2tRNA-Leu(taa)[18054,18140]
tgggagtgtggcggaatcggtagacgcaccagacttaaaatctgttgaga
attaatctcgtgggggttcaagtccccccttctcccat
1-3tRNA-Thr(tgt)[18764,18836]
gcctccgtagctcagtggtagagcagggcttttgtaaagctcaggtcgca
agttcaaatcttgtcagaggctc
1-4tRNA-Asn(gtt)[18841,18913]
tcctctatagctcagttggtagagcaggtgactgttaatcaccctgtccc
tggttcgagtccaggtggaggag
1-5tRNA-Arg(tct)[154581,154656]
tgggtcagtagctcagatggatagagcaactgccttctaagcagtcggcc
acaggttcgagtcctgtctgacccgt
>KJ019055.1 Synechococcus phage ACG-2014a isolate Syn7803C86, complete genome
1-1tRNA-Val(tac)[13064,13135]
gggcgaataactcagcggtagagtgcctcctttacacggagattgtcggg
ggttcgatcccctcttcgccca
1-2tRNA-Leu(taa)[18054,18140]
tgggagtgtggcggaatcggtagacgcaccagacttaaaatctgttgaga
attaatctcgtgggggttcaagtccccccttctcccat
1-3tRNA-Thr(tgt)[18764,18836]
gcctccgtagctcagtggtagagcagggcttttgtaaagctcaggtcgca
agttcaaatcttgtcagaggctc
1-4tRNA-Asn(gtt)[18841,18913]
tcctctatagctcagttggtagagcaggtgactgttaatcaccctgtccc
tggttcgagtccaggtggaggag
1-5tRNA-Arg(tct)[154576,154651]

tgggtcagtagctcagatggatagagcaactgccttctaagcagtcggcc
acaggttcgagtcctgtctgacccgt
>KJ019065.1 Synechococcus phage ACG−2014a isolate Syn7803C99, complete genome
1−1tRNA−Val(tac)[13064,13135]
gggcgaataactcagcggtagagtgcctcctttacacggagattgtcggg
ggttcgatcccctcttcgccca
1−2tRNA−Leu(taa)[18054,18140]
tgggagtgtggcggaatcggtagacgcaccagacttaaaatctgttgaga
attaatctcgtgggggttcaagtccccccttctcccat
1−3tRNA−Thr(tgt)[18764,18836]
gcctccgtagctcagtggtagagcagggcttttgtaaagctcaggtcgca
agttcaaatcttgtcagaggctc
1−4tRNA−Asn(gtt)[18841,18913]
tcctctatagctcagttggtagagcaggtgactgttaatcaccctgtccc
tggttcgagtccaggtggaggag
1−5tRNA−Arg(tct)[154470,154545]
tgggtcagtagctcagatggatagagcaactgccttctaagcagtcggcc
acaggttcgagtcctgtctgacccgt
>KJ019067.1 Synechococcus phage ACG−2014a isolate Syn7803US101, complete genome
1−1tRNA−Val(tac)[13064,13135]
gggcgaataactcagcggtagagtgcctcctttacacggagattgtcggg
ggttcgatcccctcttcgccca
1−2tRNA−Leu(taa)[18054,18140]
tgggagtgtggcggaatcggtagacgcaccagacttaaaatctgttgaga
attaatctcgtgggggttcaagtccccccttctcccat
1−3tRNA−Thr(tgt)[18765,18837]
gcctccgtagctcagtggtagagcagggcttttgtaaagctcaggtcgca
agttcaaatcttgtcagaggctc
1−4tRNA−Asn(gtt)[18842,18914]
tcctctatagctcagttggtagagcaggtgactgttaatcaccctgtccc
tggttcgagtccaggtggaggag
1−5tRNA−Arg(tct)[154483,154558]
tgggtcagtagctcagatggatagagcaactgccttctaagcagtcggcc
acaggttcgagtcctgtctgacccgt
>KJ019068.1 Synechococcus phage ACG−2014a isolate Syn7803US102, complete genome
1−1tRNA−Val(tac)[13064,13135]
gggcgaataactcagcggtagagtgcctcctttacacggagattgtcggg
ggttcgatcccctcttcgccca
1−2tRNA−Leu(taa)[18054,18140]
tgggagtgtggcggaatcggtagacgcaccagacttaaaatctgttgaga
attaatctcgtgggggttcaagtccccccttctcccat
1−3tRNA−Thr(tgt)[18764,18836]
gcctccgtagctcagtggtagagcagggcttttgtaaagctcaggtcgca
agttcaaatcttgtcagaggctc

1−4tRNA−Asn(gtt)[18841,18913]
tcctctatagctcagttggtagagcaggtgactgttaatcaccctgtccc
tggttcgagtccaggtggaggag
1−5tRNA−Arg(tct)[154479,154554]
tgggtcagtagctcagatggatagagcaactgccttctaagcagtcggcc
acaggttcgagtcctgtctgacccgt
>KJ019076.1 Synechococcus phage ACG−2014a isolate Syn7803US112, complete genome
1−1tRNA−Val(tac)[13064,13135]
gggcgaataactcagcggtagagtgcctcctttacacggagattgtcggg
ggttcgatcccctcttcgccca
1−2tRNA−Leu(taa)[18054,18140]
tgggagtgtggcggaatcggtagacgcaccagacttaaaatctgttgaga
attaatctcgtgggggttcaagtccccccttctcccat
1−3tRNA−Thr(tgt)[18764,18836]
gcctccgtagctcagtggtagagcagggcttttgtaaagctcaggtcgca
agttcaaatcttgtcagaggctc
1−4tRNA−Asn(gtt)[18841,18913]
tcctctatagctcagttggtagagcaggtgactgttaatcaccctgtccc
tggttcgagtccaggtggaggag
1−5tRNA−Arg(tct)[155669,155744]
tgggtcagtagctcagatggatagagcaactgccttctaagcagtcggcc
acaggttcgagtcctgtctgacccgt
>KJ019081.1 Synechococcus phage ACG−2014a isolate Syn7803US117, complete genome
1−1tRNA−Val(tac)[13064,13135]
gggcgaataactcagcggtagagtgcctcctttacacggagattgtcggg
ggttcgatcccctcttcgccca
1−2tRNA−Leu(taa)[18054,18140]
tgggagtgtggcggaatcggtagacgcaccagacttaaaatctgttgaga
attaatctcgtgggggttcaagtccccccttctcccat
1−3tRNA−Thr(tgt)[18765,18837]
gcctccgtagctcagtggtagagcagggcttttgtaaagctcaggtcgca
agttcaaatcttgtcagaggctc
1−4tRNA−Asn(gtt)[18842,18914]
tcctctatagctcagttggtagagcaggtgactgttaatcaccctgtccc
tggttcgagtccaggtggaggag
1−5tRNA−Arg(tct)[154483,154558]
tgggtcagtagctcagatggatagagcaactgccttctaagcagtcggcc
acaggttcgagtcctgtctgacccgt
>KJ019084.1 Synechococcus phage ACG−2014a isolate Syn7803US123, complete genome
1−1tRNA−Val(tac)[13064,13135]
gggcgaataactcagcggtagagtgcctcctttacacggagattgtcggg
ggttcgatcccctcttcgccca
1−2tRNA−Leu(taa)[18054,18140]
tgggagtgtggcggaatcggtagacgcaccagacttaaaatctgttgaga

attaatctcgtgggggttcaagtccccccttctcccat
1−3tRNA−Thr(tgt)[18764,18836]
gcctccgtagctcagtggtagagcagggcttttgtaaagctcaggtcgca
agttcaaatcttgtcagaggctc
1−4tRNA−Asn(gtt)[18841,18913]
tcctctatagctcagttggtagagcaggtgactgttaatcaccctgtccc
tggttcgagtccaggtggaggag
1−5tRNA−Arg(tct)[154480,154555]
tgggtcagtagctcagatggatagagcaactgccttctaagcagtcggcc
acaggttcgagtcctgtctgacccgt
>KJ019087.1 Synechococcus phage ACG−2014a isolate Syn7803US19, complete genome
1−1tRNA−Val(tac)[13064,13135]
gggcgaataactcagcggtagagtgcctcctttacacggagattgtcggg
ggttcgatccccctcttcgccca
1−2tRNA−Leu(taa)[18054,18140]
tgggagtgtggcggaatcggtagacgcaccagacttaaaatctgttgaga
attaatctcgtgggggttcaagtccccccttctcccat
1−3tRNA−Thr(tgt)[18764,18836]
gcctccgtagctcagtggtagagcagggcttttgtaaagctcaggtcgca
agttcaaatcttgtcagaggctc
1−4tRNA−Asn(gtt)[18841,18913]
tcctctatagctcagttggtagagcaggtgactgttaatcaccctgtccc
tggttcgagtccaggtggaggag
1−5tRNA−Arg(tct)[154520,154595]
tgggtcagtagctcagatggatagagcaactgccttctaagcagtcggcc
acaggttcgagtcctgtctgacccgt
>KJ019088.1 Synechococcus phage ACG−2014a isolate Syn7803US1, complete genome
1−1tRNA−Val(tac)[13058,13129]
gggcgaataactcagcggtagagtgcctcctttacacggagattgtcggg
ggttcgatccccctcttcgccca
1−2tRNA−Leu(taa)[18048,18134]
tgggagtgtggcggaatcggtagacgcaccagacttaaaatctgttgaga
attaatctcgtgggggttcaagtccccccttctcccat
1−3tRNA−Thr(tgt)[18758,18830]
gcctccgtagctcagtggtagagcagggcttttgtaaagctcaggtcgca
agttcaaatcttgtcagaggctc
1−4tRNA−Asn(gtt)[18835,18907]
tcctctatagctcagttggtagagcaggtgactgttaatcaccctgtccc
tggttcgagtccaggtggaggag
1−5tRNA−Arg(tct)[154558,154633]
tgggtcagtagctcagatggatagagcaactgccttctaagcagtcggcc
acaggttcgagtcctgtctgacccgt
>KJ019114.1 Synechococcus phage ACG−2014a isolate Syn7803US60, complete genome
1−1tRNA−Val(tac)[13064,13135]

gggcgaataactcagcggtagagtgcctcctttacacggagattgtcggg
ggttcgatcccctcttcgccca
1−2tRNA−Leu(taa)[18054,18140]
tgggagtgtggcggaatcggtagacgcaccagacttaaaatctgttgaga
attaatctcgtgggggttcaagtccccccttctcccat
1−3tRNA−Thr(tgt)[18765,18837]
gcctccgtagctcagtggtagagcagggcttttgtaaagctcaggtcgca
agttcaaatcttgtcagaggctc
1−4tRNA−Asn(gtt)[18842,18914]
tcctctatagctcagttggtagagcaggtgactgttaatcaccctgtccc
tggttcgagtccaggtggaggag
1−5tRNA−Arg(tct)[154483,154558]
tgggtcagtagctcagatggatagagcaactgccttctaagcagtcggcc
acaggttcgagtcctgtctgacccgt
>KJ019116.1 Synechococcus phage ACG−2014a isolate Syn7803US62, complete genome
1−1tRNA−Val(tac)[13064,13135]
gggcgaataactcagcggtagagtgcctcctttacacggagattgtcggg
ggttcgatcccctcttcgccca
1−2tRNA−Leu(taa)[17866,17952]
tgggagtgtggcggaatcggtagacgcaccagacttaaaatctgttgaga
attaatctcgtgggggttcaagtccccccttctcccat
1−3tRNA−Thr(tgt)[18576,18648]
gcctccgtagctcagtggtagagcagggcttttgtaaagctcaggtcgca
agttcaaatcttgtcagaggctc
1−4tRNA−Asn(gtt)[18653,18725]
tcctctatagctcagttggtagagcaggtgactgttaatcaccctgtccc
tggttcgagtccaggtggaggag
1−5tRNA−Arg(tct)[153978,154053]
tgggtcagtagctcagatggatagagcaactgccttctaagcagtcggcc
acaggttcgagtcctgtctgacccgt
>KJ019122.1 Synechococcus phage ACG−2014a isolate Syn7803US79, complete genome
1−1tRNA−Val(tac)[13064,13135]
gggcgaataactcagcggtagagtgcctcctttacacggagattgtcggg
ggttcgatcccctcttcgccca
1−2tRNA−Leu(taa)[18054,18140]
tgggagtgtggcggaatcggtagacgcaccagacttaaaatctgttgaga
attaatctcgtgggggttcaagtccccccttctcccat
1−3tRNA−Thr(tgt)[18764,18836]
gcctccgtagctcagtggtagagcagggcttttgtaaagctcaggtcgca
agttcaaatcttgtcagaggctc
1−4tRNA−Asn(gtt)[18841,18913]
tcctctatagctcagttggtagagcaggtgactgttaatcaccctgtccc
tggttcgagtccaggtggaggag
1−5tRNA−Arg(tct)[154472,154547]

tgggtcagtagctcagatggatagagcaactgccttctaagcagtcggcc
acaggttcgagtcctgtctgacccgt

>KJ019135.1 Synechococcus phage ACG-2014a isolate Syn7803C101, complete genome

1-1tRNA-Val(tac)[13064,13135]
gggcgaataactcagcggtagagtgcctcctttacacggagattgtcggg
ggttcgatcccctcttcgccca
1-2tRNA-Leu(taa)[18054,18140]
tgggagtgtggcggaatcggtagacgcaccagacttaaaatctgttgaga
attaatctcgtgggggttcaagtccccccttctcccat
1-3tRNA-Thr(tgt)[18764,18836]
gcctccgtagctcagtggtagagcagggcttttgtaaagctcaggtcgca
agttcaaatcttgtcagaggctc
1-4tRNA-Asn(gtt)[18841,18913]
tcctctatagctcagttggtagagcaggtgactgttaatcaccctgtccc
tggttcgagtccaggtggaggag
1-5tRNA-Arg(tct)[154571,154646]
tgggtcagtagctcagatggatagagcaactgccttctaagcagtcggcc
acaggttcgagtcctgtctgacccgt

>KJ019137.1 Synechococcus phage ACG-2014a isolate Syn7803C104, complete genome

1-1tRNA-Val(tac)[13064,13135]
gggcgaataactcagcggtagagtgcctcctttacacggagattgtcggg
ggttcgatcccctcttcgccca
1-2tRNA-Leu(taa)[18149,18235]
tgggagtgtggcggaatcggtagacgcaccagacttaaaatctgttgaga
attaatctcgtgggggttcaagtccccccttctcccat
1-3tRNA-Thr(tgt)[18859,18931]
gcctccgtagctcagtggtagagcagggcttttgtaaagctcaggtcgca
agttcaaatcttgtcagaggctc
1-4tRNA-Asn(gtt)[18936,19008]
tcctctatagctcagttggtagagcaggtgactgttaatcaccctgtccc
tggttcgagtccaggtggaggag
1-5tRNA-Arg(tct)[154571,154646]
tgggtcagtagctcagatggatagagcaactgccttctaagcagtcggcc
acaggttcgagtcctgtctgacccgt

>KJ019138.1 Synechococcus phage ACG-2014a isolate Syn7803C107, complete genome

1-1tRNA-Val(tac)[13076,13147]
gggcgaataactcagcggtagagtgcctcctttacacggagattgtcggg
ggttcgatcccctcttcgccca
1-2tRNA-Leu(taa)[18066,18152]
tgggagtgtggcggaatcggtagacgcaccagacttaaaatctgttgaga
attaatctcgtgggggttcaagtccccccttctcccat
1-3tRNA-Thr(tgt)[18776,18848]
gcctccgtagctcagtggtagagcagggcttttgtaaagctcaggtcgca
agttcaaatcttgtcagaggctc

1-4tRNA-Asn(gtt)[18853,18925]
tcctctatagctcagttggtagagcaggtgactgttaatcaccctgtccc
tggttcgagtccaggtggaggag
1-5tRNA-Arg(tct)[154577,154652]
tgggtcagtagctcagatggatagagcaactgccttctaagcagtcggcc
acaggttcgagtcctgtctgacccgt
>KJ019153.1 Synechococcus phage ACG-2014a isolate Syn7803C26, complete genome
1-1tRNA-Val(tac)[13064,13135]
gggcgaataactcagcggtagagtgcctcctttacacggagattgtcggg
ggttcgatcccctcttcgccca
1-2tRNA-Leu(taa)[18054,18140]
tgggagtgtggcggaatcggtagacgcaccagacttaaaatctgttgaga
attaatctcgtgggggttcaagtccccccttctcccat
1-3tRNA-Thr(tgt)[18764,18836]
gcctccgtagctcagtggtagagcagggcttttgtaaagctcaggtcgca
agttcaaatcttgtcagaggctc
1-4tRNA-Asn(gtt)[18841,18913]
tcctctatagctcagttggtagagcaggtgactgttaatcaccctgtccc
tggttcgagtccaggtggaggag
1-5tRNA-Arg(tct)[154448,154523]
tgggtcagtagctcagatggatagagcaactgccttctaagcagtcggcc
acaggttcgagtcctgtctgacccgt
>KJ019157.1 Synechococcus phage ACG-2014a isolate Syn7803C31, complete genome
1-1tRNA-Val(tac)[13159,13230]
gggcgaataactcagcggtagagtgcctcctttacacggagattgtcggg
ggttcgatcccctcttcgccca
1-2tRNA-Leu(taa)[18149,18235]
tgggagtgtggcggaatcggtagacgcaccagacttaaaatctgttgaga
attaatctcgtgggggttcaagtccccccttctcccat
1-3tRNA-Thr(tgt)[18859,18931]
gcctccgtagctcagtggtagagcagggcttttgtaaagctcaggtcgca
agttcaaatcttgtcagaggctc
1-4tRNA-Asn(gtt)[18936,19008]
tcctctatagctcagttggtagagcaggtgactgttaatcaccctgtccc
tggttcgagtccaggtggaggag
1-5tRNA-Arg(tct)[154574,154649]
tgggtcagtagctcagatggatagagcaactgccttctaagcagtcggcc
acaggttcgagtcctgtctgacccgt
>KJ019163.1 Synechococcus phage ACG-2014a isolate Syn7803C38, complete genome
1-1tRNA-Val(tac)[13064,13135]
gggcgaataactcagcggtagagtgcctcctttacacggagattgtcggg
ggttcgatcccctcttcgccca
1-2tRNA-Leu(taa)[18054,18140]
tgggagtgtggcggaatcggtagacgcaccagacttaaaatctgttgaga

attaatctcgtgggggttcaagtccccccttctcccat
1–3tRNA–Thr(tgt)[18764,18836]
gcctccgtagctcagtggtagagcagggcttttgtaaagctcaggtcgca
agttcaaatcttgtcagaggctc
1–4tRNA–Asn(gtt)[18841,18913]
tcctctatagctcagttggtagagcaggtgactgttaatcaccctgtccc
tggttcgagtccaggtggaggag
1–5tRNA–Arg(tct)[154470,154545]
tgggtcagtagctcagatggatagagcaactgccttctaagcagtcggcc
acaggttcgagtcctgtctgacccgt
>KJ019040.1 Synechococcus phage ACG–2014b isolate Syn7803C61, complete genome
1–1tRNA–Thr(tgt)[13058,13130]
gcctccgtagctcagtggtagagcagggcttttgtaaagctcaggtcgca
agttcaaatcttgtcagaggctc
1–2tRNA–Asn(gtt)[13135,13207]
tcctctatagctcagttggtagagcaggtgactgttaatcaccctgtccc
tggttcgagtccaggtggaggag
1–3tRNA–Val(tac)[13210,13281]
gggcgaataactcagcggtagagtgcctcctttacacggagattgtcggg
ggttcgatcccctcttcgccca
1–4tRNA–Leu(taa)[18645,18731]
tgggagtgtggcggaatcggtagacgcaccagacttaaaatctgttgaga
attaatctcgtgggggttcaagtccccccactcctat
1–5tRNA–Arg(tct)[137804,137878]
tgggtcagtagctcagcggatagagcatcgcacttctaatgcgttggtcg
caggttcgatccctgcctgacccgt
>KJ019041.1 Synechococcus phage ACG–2014b isolate Syn7803C66, complete genome
1–1tRNA–Thr(tgt)[13046,13118]
gcctccgtagctcagtggtagagcagggcttttgtaaagctcaggtcgca
agttcaaatcttgtcagaggctc
1–2tRNA–Asn(gtt)[13123,13195]
tcctctatagctcagttggtagagcaggtgactgttaatcaccctgtccc
tggttcgagtccaggtggaggag
1–3tRNA–Val(tac)[13198,13269]
gggcgaataactcagcggtagagtgcctcctttacacggagattgtcggg
ggttcgatcccctcttcgccca
1–4tRNA–Leu(taa)[18633,18719]
tgggagtgtggcggaatcggtagacgcaccagacttaaaatctgttgaga
attaatctcgtgggggttcaagtccccccactcctat
1–5tRNA–Arg(tct)[155616,155690]
tgggtcagtagctcagcggatagagcatcgcacttctaatgcgttggtcg
caggttcgatccctgcctgacccgt
>KJ019042.1 Synechococcus phage ACG–2014b isolate Syn7803C67, complete genome
1–1tRNA–Thr(tgt)[13058,13130]

gcctccgtagctcagtggtagagcagggcttttgtaaagctcaggtcgca
agttcaaatcttgtcagaggctc
1−2tRNA−Asn(gtt)[13135,13207]
tcctctatagctcagttggtagagcaggtgactgttaatcaccctgtccc
tggttcgagtccaggtggaggag
1−3tRNA−Val(tac)[13210,13281]
gggcgaataactcagcggtagagtgcctcctttacacggagattgtcggg
ggttcgatcccctcttcgccca
1−4tRNA−Leu(taa)[18645,18731]
tgggagtgtggcggaatcggtagacgcaccagacttaaaatctgttgaga
attaatctcgtgggggttcaagtccccccactcctat
1−5tRNA−Arg(tct)[155500,155574]
tgggtcagtagctcagcggatagagcatcgcacttctaatgcgttggtcg
caggttcgatccctgcctgacccgt
>KJ019043.1 Synechococcus phage ACG−2014b isolate Syn7803C68, complete genome
1−1tRNA−Thr(tgt)[13058,13130]
gcctccgtagctcagtggtagagcagggcttttgtaaagctcaggtcgca
agttcaaatcttgtcagaggctc
1−2tRNA−Asn(gtt)[13135,13207]
tcctctatagctcagttggtagagcaggtgactgttaatcaccctgtccc
tggttcgagtccaggtggaggag
1−3tRNA−Val(tac)[13210,13281]
gggcgaataactcagcggtagagtgcctcctttacacggagattgtcggg
ggttcgatcccctcttcgccca
1−4tRNA−Leu(taa)[18645,18731]
tgggagtgtggcggaatcggtagacgcaccagacttaaaatctgttgaga
attaatctcgtgggggttcaagtccccccactcctat
1−5tRNA−Arg(tct)[155502,155576]
tgggtcagtagctcagcggatagagcatcgcacttctaatgcgttggtcg
caggttcgatccctgcctgacccgt
>KJ019044.1 Synechococcus phage ACG−2014b isolate Syn7803C69, complete genome
1−1tRNA−Thr(tgt)[13058,13130]
gcctccgtagctcagtggtagagcagggcttttgtaaagctcaggtcgca
agttcaaatcttgtcagaggctc
1−2tRNA−Asn(gtt)[13135,13207]
tcctctatagctcagttggtagagcaggtgactgttaatcaccctgtccc
tggttcgagtccaggtggaggag
1−3tRNA−Val(tac)[13210,13281]
gggcgaataactcagcggtagagtgcctcctttacacggagattgtcggg
ggttcgatcccctcttcgccca
1−4tRNA−Leu(taa)[18733,18819]
tgggagtgtggcggaatcggtagacgcaccagacttaaaatctgttgaga
attaatctcgtgggggttcaagtccccccactcctat
1−5tRNA−Arg(tct)[155495,155569]

tgggtcagtagctcagcggatagagcatcgcacttctaatgcgttggtcg
caggttcgatccctgcctgacccgt
>KJ019049.1 Synechococcus phage ACG−2014b isolate Syn7803C76, complete genome
1−1tRNA−Thr(tgt)[13058,13130]
gcctccgtagctcagtggtagagcagggcttttgtaaagctcaggtcgca
agttcaaatcttgtcagaggctc
1−2tRNA−Asn(gtt)[13135,13207]
tcctctatagctcagttggtagagcaggtgactgttaatcaccctgtccc
tggttcgagtccaggtggaggag
1−3tRNA−Val(tac)[13210,13281]
gggcgaataactcagcggtagagtgcctcctttacacggagattgtcggg
ggttcgatcccctcttcgccca
1−4tRNA−Leu(taa)[18645,18731]
tgggagtgtggcggaatcggtagacgcaccagacttaaaatctgttgaga
attaatctcgtgggggttcaagtccccccactcctat
1−5tRNA−Arg(tct)[155502,155576]
tgggtcagtagctcagcggatagagcatcgcacttctaatgcgttggtcg
caggttcgatccctgcctgacccgt
>KJ019051.1 Synechococcus phage ACG−2014b isolate Syn7803C78, complete genome
1−1tRNA−Thr(tgt)[13058,13130]
gcctccgtagctcagtggtagagcagggcttttgtaaagctcaggtcgca
agttcaaatcttgtcagaggctc
1−2tRNA−Asn(gtt)[13135,13207]
tcctctatagctcagttggtagagcaggtgactgttaatcaccctgtccc
tggttcgagtccaggtggaggag
1−3tRNA−Val(tac)[13210,13281]
gggcgaataactcagcggtagagtgcctcctttacacggagattgtcggg
ggttcgatcccctcttcgccca
1−4tRNA−Leu(taa)[18645,18731]
tgggagtgtggcggaatcggtagacgcaccagacttaaaatctgttgaga
attaatctcgtgggggttcaagtccccccactcctat
1−5tRNA−Arg(tct)[155502,155576]
tgggtcagtagctcagcggatagagcatcgcacttctaatgcgttggtcg
caggttcgatccctgcctgacccgt
>KJ019060.1 Synechococcus phage ACG−2014b isolate Syn7803C91, complete genome
1−1tRNA−Thr(tgt)[13058,13130]
gcctccgtagctcagtggtagagcagggcttttgtaaagctcaggtcgca
agttcaaatcttgtcagaggctc
1−2tRNA−Asn(gtt)[13135,13207]
tcctctatagctcagttggtagagcaggtgactgttaatcaccctgtccc
tggttcgagtccaggtggaggag
1−3tRNA−Val(tac)[13210,13281]
gggcgaataactcagcggtagagtgcctcctttacacggagattgtcggg
ggttcgatcccctcttcgccca

1−4tRNA−Leu(taa)[18645,18731]
tgggagtgtggcggaatcggtagacgcaccagacttaaaatctgttgaga
attaatctcgtgggggttcaagtccccccactcctat
1−5tRNA−Arg(tct)[155502,155576]
tgggtcagtagctcagcggatagagcatcgcacttctaatgcgttggtcg
caggttcgatccctgcctgacccgt
>KJ019061.1 Synechococcus phage ACG−2014b isolate Syn7803C92, complete genome
1−1tRNA−Thr(tgt)[13058,13130]
gcctccgtagctcagtggtagagcagggcttttgtaaagctcaggtcgca
agttcaaatcttgtcagaggctc
1−2tRNA−Asn(gtt)[13135,13207]
tcctctatagctcagttggtagagcaggtgactgttaatcaccctgtccc
tggttcgagtccaggtggaggag
1−3tRNA−Val(tac)[13210,13281]
gggcgaataactcagcggtagagtgcctcctttacacggagattgtcggg
ggttcgatcccctcttcgccca
1−4tRNA−Leu(taa)[18645,18731]
tgggagtgtggcggaatcggtagacgcaccagacttaaaatctgttgaga
attaatctcgtgggggttcaagtccccccactcctat
1−5tRNA−Arg(tct)[155505,155579]
tgggtcagtagctcagcggatagagcatcgcacttctaatgcgttggtcg
caggttcgatccctgcctgacccgt
>KJ019104.1 Synechococcus phage ACG−2014b isolate Syn7803US49, complete genome
1−1tRNA−Thr(tgt)[13058,13130]
gcctccgtagctcagtggtagagcagggcttttgtaaagctcaggtcgca
agttcaaatcttgtcagaggctc
1−2tRNA−Asn(gtt)[13135,13207]
tcctctatagctcagttggtagagcaggtgactgttaatcaccctgtccc
tggttcgagtccaggtggaggag
1−3tRNA−Val(tac)[13210,13281]
gggcgaataactcagcggtagagtgcctcctttacacggagattgtcggg
ggttcgatcccctcttcgccca
1−4tRNA−Leu(taa)[18645,18731]
tgggagtgtggcggaatcggtagacgcaccagacttaaaatctgttgaga
attaatctcgtgggggttcaagtccccccactcctat
1−5tRNA−Arg(tct)[155502,155576]
tgggtcagtagctcagcggatagagcatcgcacttctaatgcgttggtcg
caggttcgatccctgcctgacccgt
>KJ019108.1 Synechococcus phage ACG−2014b isolate Syn7803US53, complete genome
1−1tRNA−Thr(tgt)[13058,13130]
gcctccgtagctcagtggtagagcagggcttttgtaaagctcaggtcgca
agttcaaatcttgtcagaggctc
1−2tRNA−Asn(gtt)[13135,13207]
tcctctatagctcagttggtagagcaggtgactgttaatcaccctgtccc

tggttcgagtccaggtggaggag
1−3tRNA−Val(tac)[13210,13281]
gggcgaataactcagcggtagagtgcctcctttacacggagattgtcggg
ggttcgatcccctcttcgccca
1−4tRNA−Leu(taa)[18645,18731]
tgggagtgtggcggaatcggtagacgcaccagacttaaaatctgttgaga
attaatctcgtgggggttcaagtccccccactcctat
1−5tRNA−Arg(tct)[155407,155481]
tgggtcagtagctcagcggatagagcatcgcacttctaatgcgttggtcg
caggttcgatccctgcctgacccgt
>KJ019109.1 Synechococcus phage ACG−2014b isolate Syn7803US54, complete genome
1−1tRNA−Thr(tgt)[13058,13130]
gcctccgtagctcagtggtagagcagggcttttgtaaagctcaggtcgca
agttcaaatcttgtcagaggctc
1−2tRNA−Asn(gtt)[13135,13207]
tcctctatagctcagttggtagagcaggtgactgttaatcaccctgtccc
tggttcgagtccaggtggaggag
1−3tRNA−Val(tac)[13210,13281]
gggcgaataactcagcggtagagtgcctcctttacacggagattgtcggg
ggttcgatcccctcttcgccca
1−4tRNA−Leu(taa)[18645,18731]
tgggagtgtggcggaatcggtagacgcaccagacttaaaatctgttgaga
attaatctcgtgggggttcaagtccccccactcctat
1−5tRNA−Arg(tct)[155502,155576]
tgggtcagtagctcagcggatagagcatcgcacttctaatgcgttggtcg
caggttcgatccctgcctgacccgt
>KJ019110.1 Synechococcus phage ACG−2014b isolate Syn7803US56, complete genome
1−1tRNA−Thr(tgt)[13153,13225]
gcctccgtagctcagtggtagagcagggcttttgtaaagctcaggtcgca
agttcaaatcttgtcagaggctc
1−2tRNA−Asn(gtt)[13230,13302]
tcctctatagctcagttggtagagcaggtgactgttaatcaccctgtccc
tggttcgagtccaggtggaggag
1−3tRNA−Val(tac)[13305,13376]
gggcgaataactcagcggtagagtgcctcctttacacggagattgtcggg
ggttcgatcccctcttcgccca
1−4tRNA−Leu(taa)[18740,18826]
tgggagtgtggcggaatcggtagacgcaccagacttaaaatctgttgaga
attaatctcgtgggggttcaagtccccccactcctat
1−5tRNA−Arg(tct)[155502,155576]
tgggtcagtagctcagcggatagagcatcgcacttctaatgcgttggtcg
caggttcgatccctgcctgacccgt
>KJ019132.1 Synechococcus phage ACG−2014b isolate Syn9311C1, complete genome
1−1tRNA−Thr(tgt)[13058,13130]

gcctccgtagctcagtggtagagcagggcttttgtaaagctcaggtcgca
agttcaaatcttgtcagaggctc
1-2tRNA-Asn(gtt)[13135,13207]
tcctctatagctcagttggtagagcaggtgactgttaatcaccctgtccc
tggttcgagtccaggtggaggag
1-3tRNA-Val(tac)[13210,13281]
gggcgaataactcagcggtagagtgcctcctttacacggagattgtcggg
ggttcgatcccctcttcgccca
1-4tRNA-Leu(taa)[18645,18731]
tgggagtgtggcggaatcggtagacgcaccagacttaaaatctgttgaga
attaatctcgtgggggttcaagtccccccactcctat
1-5tRNA-Arg(tct)[155075,155149]
tgggtcagtagctcagcggatagagcatcgcacttctaatgcgttggtcg
caggttcgatccctgcctgacccgt
>KJ019133.1 Synechococcus phage ACG-2014b isolate Syn9311C4, complete genome
1-1tRNA-Thr(tgt)[13058,13130]
gcctccgtagctcagtggtagagcagggcttttgtaaagctcaggtcgca
agttcaaatcttgtcagaggctc
1-2tRNA-Asn(gtt)[13135,13207]
tcctctatagctcagttggtagagcaggtgactgttaatcaccctgtccc
tggttcgagtccaggtggaggag
1-3tRNA-Val(tac)[13210,13281]
gggcgaataactcagcggtagagtgcctcctttacacggagattgtcggg
ggttcgatcccctcttcgccca
1-4tRNA-Leu(taa)[18645,18731]
tgggagtgtggcggaatcggtagacgcaccagacttaaaatctgttgaga
attaatctcgtgggggttcaagtccccccactcctat
1-5tRNA-Arg(tct)[155688,155762]
tgggtcagtagctcagcggatagagcatcgcacttctaatgcgttggtcg
caggttcgatccctgcctgacccgt
>KJ019154.1 Synechococcus phage ACG-2014b isolate Syn7803C28, complete genome
1-1tRNA-Thr(tgt)[13058,13130]
gcctccgtagctcagtggtagagcagggcttttgtaaagctcaggtcgca
agttcaaatcttgtcagaggctc
1-2tRNA-Asn(gtt)[13135,13207]
tcctctatagctcagttggtagagcaggtgactgttaatcaccctgtccc
tggttcgagtccaggtggaggag
1-3tRNA-Val(tac)[13210,13281]
gggcgaataactcagcggtagagtgcctcctttacacggagattgtcggg
ggttcgatcccctcttcgccca
1-4tRNA-Leu(taa)[18645,18731]
tgggagtgtggcggaatcggtagacgcaccagacttaaaatctgttgaga
attaatctcgtgggggttcaagtccccccactcctat
1-5tRNA-Arg(tct)[155495,155569]

tgggtcagtagctcagcggatagagcatcgcacttctaatgcgttggtcg
caggttcgatccctgcctgacccgt
>KJ019161.1 Synechococcus phage ACG-2014b isolate Syn7803C36, complete genome
1-1tRNA-Thr(tgt)[13058,13130]
gcctccgtagctcagtggtagagcagggcttttgtaaagctcaggtcgca
agttcaaatcttgtcagaggctc
1-2tRNA-Asn(gtt)[13135,13207]
tcctctatagctcagttggtagagcaggtgactgttaatcaccctgtccc
tggttcgagtccaggtggaggag
1-3tRNA-Val(tac)[13210,13281]
gggcgaataactcagcggtagagtgcctcctttacacggagattgtcggg
ggttcgatcccctcttcgccca
1-4tRNA-Leu(taa)[18645,18731]
tgggagtgtggcggaatcggtagacgcaccagacttaaaatctgttgaga
attaatctcgtgggggttcaagtccccccactcctat
1-5tRNA-Arg(tct)[155498,155572]
tgggtcagtagctcagcggatagagcatcgcacttctaatgcgttggtcg
caggttcgatccctgcctgacccgt
>FR852584.1 Staphylococcus phage ISP complete genome
1-1tRNA-Met(cat)c[110656,110727]
ggactcttagcttaaaggtaaagccaaccgctcataacggtttgactgta
ggttcgaatcctgcagagtcca
1-2tRNA-Trp(cca)c[133874,133945]
acacccttagtataattagtagtacaagggtctccaaaacccttagtctt
tgtgcaaatcaaagagggtgtg
1-3tRNA-Phe(gaa)c[133952,134024]
ggtttcttagctcagatggtagagcactagattgaagctctaggtgtcat
tggttcaaatccaatagaaacca
1-4tRNA-Asp(gtc)c[134030,134105]
tggctcattggtgtaactggttaacacactgccctgtcacggcagagagt
acgagttcgagtctcgtatgggtcgt
>JX875065.1 Staphylococcus phage SA5, complete genome
1-1tRNA-Asp(gtc)[33538,33613]
tggctcattggtgtaactggttaacacactgccctgtcacggcagagagt
acgagttcgagtctcgtatgggtcgt
1-2tRNA-Phe(gaa)[33619,33691]
ggtttcttagctcagatggtagagcactagattgaagctctaggtgtcat
tggttcaaatccaatagaaacca
1-3tRNA-Trp(cca)[33698,33769]
acacccttagtataattagtagtacaagggtctccaaaacccttagtctt
tgtgcaaatcaaagagggtgtg
1-4tRNA-Met(cat)[56903,56974]
ggactcttagcttaaaggtaaagccaaccgctcataacggtttgactgta
ggttcgaatcctgcagagtcca

>JX080300.2 Staphylococcus phage Staph1N, complete genome
1-1tRNA-Met(cat)c[14198,14269]
ggactcttagcttaaaggtaaagccaaccgctcataacggtttgactgta
ggttcgaatcctgcagagtcca
1-2tRNA-Trp(cca)c[37417,37488]
acacccttagtataattagtagtacaagggtctccaaaacccttagtctt
tgtgcaaatcaaagagggtgtg
1-3tRNA-Phe(gaa)c[37495,37567]
ggtttcttagctcagatggtagagcactagattgaagctctaggtgtcat
tggttcaaatccaatagaaacca
1-4tRNA-Asp(gtc)c[37573,37648]
tggctcattggtgtaactggttaacacactgccctgtcacggcagagagt
acgagttcgagtctcgtatgggtcgt
>JX080301.2 Staphylococcus phage A3R, complete genome
1-1tRNA-Met(cat)c[14073,14144]
ggactcttagcttaaaggtaaagccaaccgctcataacggtttgactgta
ggttcgaatcctgcagagtcca
1-2tRNA-Trp(cca)c[37292,37363]
acacccttagtataattagtagtacaagggtctccaaaacccttagtctt
tgtgcaaatcaaagagggtgtg
1-3tRNA-Phe(gaa)c[37370,37442]
ggtttcttagctcagatggtagagcactagattgaagctctaggtgtcat
tggttcaaatccaatagaaacca
1-4tRNA-Asp(gtc)c[37448,37523]
tggctcattggtgtaactggttaacacactgccctgtcacggcagagagt
acgagttcgagtctcgtatgggtcgt
>JX080302.2 Staphylococcus phage 676Z, complete genome
1-1tRNA-Met(cat)c[15120,15191]
ggactcttagcttaaaggtaaagccaaccgctcataacggtttgactgta
ggttcgaatcctgcagagtcca
1-2tRNA-Trp(cca)c[38339,38410]
acacccttagtataattagtagtacaagggtctccaaaacccttagtctt
tgtgcaaatcaaagagggtgtg
1-3tRNA-Phe(gaa)c[38417,38489]
ggtttcttagctcagatggtagagcactagattgaagctctaggtgtcat
tggttcaaatccaatagaaacca
1-4tRNA-Asp(gtc)c[38495,38570]
tggctcattggtgtaactggttaacacactgccctgtcacggcagagagt
acgagttcgagtctcgtatgggtcgt
>JX080303.2 Staphylococcus phage Fi200W, complete genome
1-1tRNA-Met(cat)c[15079,15150]
ggactcttagcttaaaggtaaagccaaccgctcataacggtttgactgta
ggttcgaatcctgcagagtcca
1-2tRNA-Trp(cca)c[38295,38366]

acacccttagtataattagtagtacaagggtctccaaaacccttagtctt
tgtgcaaatcaaagagggtgtg
1−3tRNA−Phe(gaa)c[38373,38445]
ggtttcttagctcagatggtagagcactagattgaagctctaggtgtcat
tggttcaaatccaatagaaacca
1−4tRNA−Asp(gtc)c[38451,38526]
tggctcattggtgtaactggttaacacactgccctgtcacggcagagagt
acgagttcgagtctcgtatgggtcgt
>JX080304.2 Staphylococcus phage MSA6, complete genome
1−1tRNA−Met(cat)c[14722,14793]
ggactcttagcttaaaggtaaagccaaccgctcataacggtttgactgta
ggttcgaatcctgcagagtcca
1−2tRNA−Trp(cca)c[37937,38008]
acacccttagtataattagtagtacaagggtctccaaaacccttagtctt
tgtgcaaatcaaagagggtgtg
1−3tRNA−Phe(gaa)c[38015,38087]
ggtttcttagctcagatggtagagcactagattgaagctctaggtgtcat
tggttcaaatccaatagaaacca
1−4tRNA−Asp(gtc)c[38093,38168]
tggctcattggtgtaactggttaacacactgccctgtcacggcagagagt
acgagttcgagtctcgtatgggtcgt
>JX080305.2 Staphylococcus phage P4W, complete genome
1−1tRNA−Met(cat)c[14181,14252]
ggactcttagcttaaaggtaaagccaaccgctcataacggtttgactgta
ggttcgaatcctgcagagtcca
1−2tRNA−Trp(cca)c[37397,37468]
acacccttagtataattagtagtacaagggtctccaaaacccttagtctt
tgtgcaaatcaaagagggtgtg
1−3tRNA−Phe(gaa)c[37475,37547]
ggtttcttagctcagatggtagagcactagattgaagctctaggtgtcat
tggttcaaatccaatagaaacca
1−4tRNA−Asp(gtc)c[37553,37628]
tggctcattggtgtaactggttaacacactgccctgtcacggcagagagt
acgagttcgagtctcgtatgggtcgt
>EU418428.2 Staphylococcus phage A5W, complete genome
1−1tRNA−Met(cat)c[14198,14269]
ggactcttagcttaaaggtaaagccaaccgctcataacggtttgactgta
ggttcgaatcctgcagagtcca
1−2tRNA−Trp(cca)c[37420,37491]
acacccttagtataattagtagtacaagggtctccaaaacccttagtctt
tgtgcaaatcaaagagggtgtg
1−3tRNA−Phe(gaa)c[37498,37570]
ggtttcttagctcagatggtagagcactagattgaagctctaggtgtcat
tggttcaaatccaatagaaacca

1-4tRNA-Asp(gtc)c[37576,37651]
tggctcattggtgtaactggttaacacactgccctgtcacggcagagagt
acgagttcgagtctcgtatgggtcgt
>KP687431.1 Staphylococcus phage IME-SA1, complete genome
1-1tRNA-Trp(cca)c[10772,10843]
acacccttagtataattagtagtacaagggtctccaaaacccttagtctt
tgtgcaaatcaaagagggtgtg
1-2tRNA-Phe(gaa)c[10850,10922]
ggtttcttagctcagatggtagagcactagattgaagctctaggtgtcat
tggttcaaatccaatagaaacca
1-3tRNA-Asp(gtc)c[10928,11003]
tggctcattggtgtaactggttaacacactgccctgtcacggcagagagt
acgagttcgagtctcgtatgggtcgt
1-4tRNA-Met(cat)c[127774,127845]
ggactcttagcttaaaggtaaagccaaccgctcataacggtttgactgta
ggttcgaatcctgcagagtcca
>KP687432.1 Staphylococcus phage IME-SA2, complete genome
1-1tRNA-Met(cat)c[5906,5977]
ggactcttagcttaaaggtaaagccaaccgctcataacggtttgactgta
ggttcgaatcctgcagagtcca
1-2tRNA-Trp(cca)c[29115,29186]
acacccttagtataattagtagtacaagggtctccaaaacccttagtctt
tgtgcaaatcaaagagggtgtg
1-3tRNA-Phe(gaa)c[29193,29265]
ggtttcttagctcagatggtagagcactagattgaagctctaggtgtcat
tggttcaaatccaatagaaacca
1-4tRNA-Asp(gtc)c[29271,29346]
tggctcattggtgtaactggttaacacactgccctgtcacggcagagagt
acgagttcgagtctcgtatgggtcgt
>KR902361.1 Staphylococcus phage IME-SA118, complete genome
1-1tRNA-Met(cat)c[20652,20723]
ggactcttagcttaaaggtaaagccaaccgctcataacggtttgactgta
ggttcgaatcctgcagagtcca
1-2tRNA-Trp(cca)c[43866,43937]
acacccttagtataattagtagtacaagggtctccaaaacccttagtctt
tgtgcaaatcaaagagggtgtg
1-3tRNA-Phe(gaa)c[43944,44016]
ggtttcttagctcagatggtagagcactagattgaagctctaggtgtcat
tggttcaaatccaatagaaacca
1-4tRNA-Asp(gtc)c[44022,44097]
tggctcattggtgtaactggttaacacactgccctgtcacggcagagagt
acgagttcgagtctcgtatgggtcgt
>KR908644.1 Staphylococcus phage IME-SA119, complete genome
1-1tRNA-Asp(gtc)[7628,7703]

tggctcattggtgtaactggttaacacactgccctgtcacggcagagagt
acgagttcgagtctcgtatgggtcgt
1-2tRNA-Phe(gaa)[7709,7781]
ggtttcttagctcagatggtagagcactagattgaagctctaggtgtcat
tggttcaaatccaatagaaacca
1-3tRNA-Trp(cca)[7788,7859]
acacccttagtataattagtagtacaagggtctccaaaacccttagtctt
tgtgcaaatcaaagagggtgtg
1-4tRNA-Met(cat)[31005,31076]
ggactcttagcttaaaggtaaagccaaccgctcataacggtttgactgta
ggttcgaatcctgcagagtcca
>KX349285.1 Synechococcus phage S-RIM8 isolate RW_01_0212_WH8101, complete genome
1-1tRNA-Gly(tcc)[13712,13786]
gcggatgtaattcagtggtagaatgtcagccttccaagctgaacgtcagg
ggttcgagtccccttatccgctcca
1-2tRNA-Val(tac)[13787,13858]
gggcgattaactcagcggtagagtgcctcgtttacaccgagtatgtcggg
ggttcgaatccctcatcgccca
1-3tRNA-Leu(taa)[18353,18438]
tgggacggtggcggaatcggtagacgcaccagacttaaaatctgttgggc
aatagcccgtgagagttcaagtctctctcgtcctat
1-4tRNA-Thr(tgt)[19094,19170]
gcctccgtagctcagctggatagagcaacggttttgtaaaccgtaggtcg
tcggttcaagtccgaccgtgggctcca
1-5tRNA-Ala(tgc)[19171,19243]
ggggaattagctcagttggtagagcgcctgctttgcaagcaggatgtcag
cggttcgagtccgctattctcca
1-6tRNA-Asn(gtt)[19268,19342]
ttcctctatagctcagtcggtagagcgtttgactgttaatcaaaatgtcc
ctggttcgagcccaggtggaggagt
1-7tRNA-Arg(tct)[157166,157241]
tgggtcagtagctcagttggatagagcaactgccttctaagcagtcggtc
acaggttcgagtcctgtctgacccgt
1-8tRNA-Pro(tgg)[158249,158323]
cggggtgtagcgcagcttggtagcgcggttgctttgggagcaataggtcg
caggttcgaatcctgtcaccccgac
>KX349286.1 Synechococcus phage S-RIM8 isolate RW_03_0807_WH8101, complete genome
1-1tRNA-Gly(tcc)[13707,13781]
gcggatgtaattcagtggtagaatgtcagccttccaagctgaacgtcagg
ggttcgagtccccttatccgctcca
1-2tRNA-Val(tac)[13782,13853]
gggcgattaactcagtggtagagtgcctcgtttacaccgagtatgtcggg
ggttcgaatccctcatcgccca
1-3tRNA-Leu(taa)[18348,18433]

tgggacggtggcggaatcggtagacgcaccagacttaaaatctgttgggc
aatagcccgtgagagttcaagtctctctcgtcctat
1-4tRNA-Thr(tgt)[19089,19162]
gcctccgtagctcagctggatagagcaacggttttgtaaaccgtaggtcg
tcggttcaagtccgaccgtgggct
1-6tRNA-Ala(tgc)[19490,19564]
tggggaattagctcagttggtagagcgcctgctttgcaagcaggatgtca
gcggttcgagtccgctattctccat
1-7tRNA-Asn(gtt)[19588,19662]
ttcctctatagctcagtcggtagagcgtttgactgttaatcaaaatgtcc
ctggttcgagcccaggtggaggagt
1-8tRNA-Arg(tct)[155937,156012]
tgggtcagtagctcagttggatagagcaactgccttctaagcagtcggtc
acaggttcgagtcctgtctgacccgt
1-9tRNA-Pro(tgg)[157020,157094]
cggggtgtagcgcagcttggtagcgcggttgctttgggagcaataggtcg
caggttcgaatcctgtcaccccgac

>KX349287.1 Synechococcus phage S-RIM8 isolate RW_06_0613, complete genome
1-1tRNA-Gly(tcc)[13701,13775]
gcggatgtaattcagtggtagaatgtcagccttccaagctgaacgtcagg
ggttcgagtccccttatccgctcca
1-2tRNA-Val(tac)[13776,13847]
gggcgattaactcagcggtagagtgcctcgtttacaccgagtatgtcggg
ggttcgaatccctcatcgccca
1-3tRNA-Leu(taa)[18342,18427]
tgggacggtggcggaatcggtagacgcaccagacttaaaatctgttgggc
aatagcccgtgagagttcaagtctctctcgtcctat
1-4tRNA-Thr(tgt)[19083,19159]
gcctccgtagctcagctggatagagcaacggttttgtaaaccgtaggtcg
tcggttcaagtccgaccgtgggctcca
1-5tRNA-Ala(tgc)[19160,19232]
ggggaattagctcagttggtagagcgcctgctttgcaagcaggatgtcag
cggttcgagtccgctattctcca
1-6tRNA-Asn(gtt)[19257,19331]
ttcctctatagctcagtcggtagagcgtttgactgttaatcaaaatgtcc
ctggttcgagcccaggtggaggagt
1-7tRNA-Arg(tct)[156851,156926]
tgggtcagtagctcagttggatagagcaactgccttctaagcagtcggtc
acaggttcgagtcctgtctgacccgt
1-8tRNA-Pro(tgg)[157934,158008]
cggggtgtagcgcagcttggtagcgcggttgctttgggagcaataggtcg
caggttcgaatcctgtcaccccgac

>KX349288.1 Synechococcus phage S-RIM8 isolate RW_08_0711, complete genome
1-1tRNA-Gly(tcc)[13701,13775]

gcggatgtaattcagtggtagaatgtcagccttccaagctgaacgtcagg
ggttcgagtccccttatccgctcca
1-2tRNA-Val(tac)[13776,13847]
gggcgattaactcagcggtagagtgcctcgtttacaccgagtatgtcggg
ggttcgaatccctcatcgccca
1-3tRNA-Leu(taa)[18342,18427]
tgggacggtggcggaatcggtagacgcaccagacttaaaatctgttgggc
aatagcccgtgagagttcaagtctctctcgtcctat
1-4tRNA-Thr(tgt)[19083,19159]
gcctccgtagctcagctggatagagcaacggttttgtaaaccgtaggtcg
tcggttcaagtccgaccgtgggctcca
1-5tRNA-Ala(tgc)[19160,19232]
ggggaattagctcagttggtagagcgcctgctttgcaagcaggatgtcag
cggttcgagtccgctattctcca
1-6tRNA-Asn(gtt)[19257,19331]
ttcctctatagctcagtcggtagagcgtttgactgttaatcaaaatgtcc
ctggttcgagcccaggtggaggagt
1-7tRNA-Arg(tct)[156257,156332]
tgggtcagtagctcagttggatagagcaactgccttctaagcagtcggtc
acaggttcgagtcctgtctgacccgt
1-8tRNA-Pro(tgg)[157340,157414]
cggggtgtagcgcagcttggtagcgcggttgctttgggagcaataggtcg
caggttcgaatcctgtcaccccgac
>KX349289.1 Synechococcus phage S-RIM8 isolate RW_22_0300, complete genome
1-1tRNA-Gly(tcc)[13713,13787]
gcggatgtaattcagtggtagaatgtcagccttccaagctgaacgtcagg
ggttcgagtccccttatccgctcca
1-2tRNA-Val(tac)[13788,13859]
gggcgattaactcagcggtagagtgcctcgtttacaccgagtatgtcggg
ggttcgaatccctcatcgccca
1-3tRNA-Leu(taa)[18356,18441]
tgggacggtggcggaatcggtagacgcaccagacttaaaatctgttgggc
aatagcccgtgagagttcaagtctctctcgtcctat
1-4tRNA-Thr(tgt)[19097,19173]
gcctccgtagctcagctggatagagcaacggttttgtaaaccgtaggtcg
tcggttcaagtccgaccgtgggctcca
1-5tRNA-Ala(tgc)[19174,19246]
ggggaattagctcagttggtagagcgcctgctttgcaagcaggatgtcag
cggttcgagtccgctattctcca
1-6tRNA-Asn(gtt)[19271,19345]
ttcctctatagctcagtcggtagagcgtttgactgttaatcaaaatgtcc
ctggttcgagcccaggtggaggagt
1-7tRNA-Arg(tct)[156612,156687]
tgggtcagtagctcagttggatagagcaactgccttctaagcagtcggtc

acaggttcgagtcctgtctgacccgt
1−8tRNA−Pro(tgg)[157695,157769]
cggggtgtagcgcagcttggtagcgcggttgctttgggagcaataggtcg
caggttcgaatcctgtcaccccgac
>KX349290.1 Synechococcus phage S−RIM8 isolate RW_25_1112, complete genome
1−1tRNA−Gly(tcc)[13701,13775]
gcggatgtaattcagtggtagaatgtcagccttccaagctgaacgtcagg
ggttcgagtccccttatccgctcca
1−2tRNA−Val(tac)[13776,13847]
gggcgattaactcagcggtagagtgcctcgtttacaccgagtatgtcggg
ggttcgaatccctcatcgccca
1−3tRNA−Leu(taa)[18342,18427]
tgggacggtggcggaatcggtagacgcaccagacttaaaatctgttgggc
aatagcccgtgagagttcaagtctctctcgtcctat
1−4tRNA−Thr(tgt)[19083,19159]
gcctccgtagctcagctggatagagcaacggttttgtaaaccgtaggtcg
tcggttcaagtccgaccgtgggctcca
1−5tRNA−Ala(tgc)[19160,19232]
ggggaattagctcagttggtagagcgcctgctttgcaagcaggatgtcag
cggttcgagtccgctattctcca
1−6tRNA−Asn(gtt)[19257,19331]
ttcctctatagctcagtcggtagagcgtttgactgttaatcaaaatgtcc
ctggttcgagcccaggtggaggagt
1−7tRNA−Arg(tct)[156851,156926]
tgggtcagtagctcagttggatagagcaactgccttctaagcagtcggtc
acaggttcgagtcctgtctgacccgt
1−8tRNA−Pro(tgg)[157934,158008]
cggggtgtagcgcagcttggtagcgcggttgctttgggagcaataggtcg
caggttcgaatcctgtcaccccgac
>HQ317385.1 Synechococcus phage S−RIM8 A.HR5, complete genome
1−1tRNA−Gly(tcc)[13713,13787]
gcggatgtaattcagtggtagaatgtcagccttccaagctgaacgtcagg
ggttcgagtccccttatccgctcca
1−2tRNA−Val(tac)[13788,13859]
gggcgattaactcagcggtagagtgcctcgtttacaccgagtatgtcggg
ggttcgaatccctcatcgccca
1−3tRNA−Leu(taa)[18355,18440]
tgggacggtggcggaatcggtagacgcaccagacttaaaatctgttgggc
aatagcccgtgagagttcaagtctctctcgtcctat
1−4tRNA−Thr(tgt)[19096,19172]
gcctccgtagctcagctggatagagcaacggttttgtaaaccgtaggtcg
tcggttcaagtccgaccgtgggctcca
1−5tRNA−Ala(tgc)[19173,19245]
ggggaattagctcagttggtagagcgcctgctttgcaagcaggatgtcag

cggttcgagtccgctattctcca
1–6tRNA–Asn(gtt)[19270,19344]
ttcctctatagctcagtcggtagagcgtttgactgttaatcaaaatgtcc
ctggttcgagcccaggtggaggagt
1–7tRNA–Arg(tct)[153724,153799]
tgggtcagtagctcagttggatagagcaactgccttctaagcagtcggtc
acaggttcgagtcctgtctgacccgt
1–8tRNA–Pro(tgg)[154807,154881]
cggggtgtagcgcagcttggtagcgcggttgctttgggagcaataggtcg
caggttcgaatcctgtcaccccgac
>JF974289.1 Synechococcus phage S–RIM8 A.HR3, complete genome
1–1tRNA–Gly(tcc)[13713,13787]
gcggatgtaattcagtggtagaatgtcagccttccaagctgaacgtcagg
ggttcgagtccccttatccgctcca
1–2tRNA–Val(tac)[13788,13859]
gggcgattaactcagcggtagagtgcctcgtttacaccgagtatgtcggg
ggttcgaatccctcatcgccca
1–3tRNA–Leu(taa)[18355,18440]
tgggacggtggcggaatcggtagacgcaccagacttaaaatctgttgggc
aatagcccgtgagagttcaagtctctctcgtcctat
1–4tRNA–Thr(tgt)[19096,19172]
gcctccgtagctcagctggatagagcaacggttttgtaaaccgtaggtcg
tcggttcaagtccgaccgtgggctcca
1–5tRNA–Ala(tgc)[19173,19245]
ggggaattagctcagttggtagagcgcctgctttgcaagcaggatgtcag
cggttcgagtccgctattctcca
1–6tRNA–Asn(gtt)[19270,19344]
ttcctctatagctcagtcggtagagcgtttgactgttaatcaaaatgtcc
ctggttcgagcccaggtggaggagt
1–7tRNA–Arg(tct)[156607,156682]
tgggtcagtagctcagttggatagagcaactgccttctaagcagtcggtc
acaggttcgagtcctgtctgacccgt
1–8tRNA–Pro(tgg)[157690,157764]
cggggtgtagcgcagcttggtagcgcggttgctttgggagcaataggtcg
caggttcgaatcctgtcaccccgac
>JF974293.1 Cyanophage KBS–M–1A genomic sequence
1–1tRNA–Asn(gtt)c[26777,26851]
ttcctctatagctcagtcggtagagcgtttgactgttaatcaaaatgtcc
ctggttcgagcccaggtggaggagt
1–2tRNA–Ala(tgc)c[26876,26948]
ggggaattagctcagttggtagagcgcctgctttgcaagcaggatgtcag
cggttcgagtccgctattctcca
1–3tRNA–Thr(tgt)c[26949,27025]
gcctccgtagctcagctggatagagcaacggttttgtaaaccgtaggtcg

tcggttcaagtccgaccgtgggctcca
1-4tRNA-Leu(taa)c[27681,27766]
tgggacggtggcggaatcggtagacgcaccagacttaaaatctgttgggc
aatagcccgtgagagttcaagtctctctcgtcctat
1-5tRNA-Val(tac)c[32262,32333]
gggcgattaactcagcggtagagtgcctcgtttacaccgagtatgtcggg
ggttcgaatccctcatcgccca
1-6tRNA-Gly(tcc)c[32334,32408]
gcggatgtaattcagtggtagaatgtcagccttccaagctgaacgtcagg
ggttcgagtccccttatccgctcca
1-7tRNA-Pro(tgg)c[59568,59642]
cggggtgtagcgcagcttggtagcgcggttgctttgggagcaataggtcg
caggttcgaatcctgtcaccccgac
1-8tRNA-Arg(tct)c[60650,60725]
tgggtcagtagctcagttggatagagcaactgccttctaagcagtcggtc
acaggttcgagtcctgtctgacccgt

>JF704096.1 Mycobacterium phage Ghost, complete sequence

1-1tRNA-Ser(gct)[30462,30545]
ggagggtgagcatcaggtgatgcagcgagattgctaatcccgtacggtaa
ccaccccgtgaggttcgaatcctcctccctccgc
1-2tRNA-Leu(cag)[30738,30813]
gctcccgtagcccaattggcaggaggcaccagattcaggatctgggcagt
gtgagttcgaatctcaccgggagtac
1-3tRNA-Leu(gag)[30933,31007]
gtctctgtaggcaaatcggaaaagccgccatcttgagggggtggtgcgtg
cgggttcgactcccgccagagacac
1-4tRNA-Leu(caa)[31008,31081]
gccgtggtaggccatctggcgagccgccgagttcaagtttcggtgtttgc
gggttcgaatcccgcccacggtac
1-5tRNA-SeC(tca)[67487,67583]
attctggcactggtgggcgagcccaccggcggcagcttcaagctgtcgct
ggccggagaaccgaccggaaacatcccgttcaacgcgacccagggcc
1-6tRNA-Pro(tgg)[91444,91518]
cggggtgtagttcagtttggaagagcgcttggtttgggaccaagatgtcg
caggttcgaatcctgtcaccccgac
1-7tRNA-Trp(cca)[91533,91603]
gggtctgtgcacagggtgcccgacggtctccaaagccgaaggcgggggtt
cgattccctccaggcctgcca
1-8tRNA-Tyr(gta)[91605,91691]
cccgtacatgcccaactggtgtttgggagcaggctgtaaatctgtggcct
tcgggacggtgaggttcgattcctcagtgcgggacca
1-9tRNA-Pyl(cta)[92745,92818]
tgcgagatcgtgcacggcgcactaggagcttctaaccctccgactcgcgg
gttcgactcccgcatctcgcaccc

1-10tRNA-Met(cat)[92966,93040]
agcggtgtagagcagctaggtagctcgccgggctcataacccggaggacg
cgtgttcgattcacgccaccgccac
1-11tRNA-Cys(gca)[93167,93238]
gcgcctttggcggaatggctacgtgctcggctgcaacccgagttatcccg
gttcgactccgggagggcgctc
1-12tRNA-Glu(ctc)[93243,93317]
ggtccgttggagtagatggacatctcgccaccctctcaaggtggagatca
cgggttcaagtcccgtacggactgc
1-13tRNA-His(gtg)[93319,93392]
gtggccgtagttcagccggtagaacgctgggttgtgatcccagtcgtcga
gggttcgagtccctccggtcaccc
1-14tRNA-Ala(tgc)[93554,93628]
gggcctgtagctccaattggtagagcagcatccttgcaagatgacggctg
tcggttcgaatccgacctggtccac
1-15tRNA-Phe(gaa)[93818,93890]
gccgtcatagctcagttggtagagcactggcctgaaaacccagtggccga
ggttcgattcctcgtgtcggcac
1-16tRNA-Val(cac)[93896,93969]
gtccgtttagctcagctggaagagcgctcggtccacacccgagaggccgc
aggttcgatccctgcaatggacac
1-17tRNA-Lys(ctt)[94088,94160]
gccttcgtagctcagtggtagagctgtcgcctcttaagcgataggtcgtt
ggttcgaatccagccgggggcac
1-18tRNA-Glu(ttc)[94165,94241]
ggtcgggtcggtctgctggtatggccagtcggattttcactccggacatt
cgcgggttcaattcccgtcccgatcgc
1-19tRNA-Gly(tcc)[94321,94393]
gcgggtgtggccgaatggctcaggcaccagatttccactctggctacgca
ggttcgattcctgtcatccgctc
1-20tRNA-Thr(cgt)[94453,94527]
gctgctgtagctcacctggcagagcgtcggcgtcgtatcccgaaggcatc
cggttcgagtccggacagcagcccc
1-21tRNA-Thr(tgt)[94528,94600]
gcctctgtggtccagcggcacggacatccgccttgtaagcggaggacccc
cgttcgatccggggtagaggctc
1-22tRNA-Thr(ggt)[95008,95080]
gctgggttagctcagtggtagagcgttcctctggtatgggaaaggcccgg
ggttcaatcccccgactcagctc
1-23tRNA-Gly(gcc)[96403,96476]
gcgaaggtagctcagctggcagagcgccaccttgccaaggtggaggtcgc
gggatcgtaacccgttcttcgctc
1-24tRNA-Asp(gtc)[96480,96552]
ggccctgtagctcagaggaagagcgccggtctgtcgaatcggaggtcgcg

gtatcgtaatccgtcagggtcgc
1-25tRNA-Met(cat)[96612,96684]
gcctcactagctcattggtagagccgctcgctcataacgtgcaggtacct
ggttcgattccagggtgaggtac
1-26tRNA-Ile(gat)[96690,96764]
gcctgttagcggactggtcgtccgatccaagctgataacttggcgtaagc
ggtgttcgattcaccgagcaggtac
1-27tRNA-Arg(acg)[96858,96930]
gcctctatggtccaacggatatgacgccggtctacggaaccggagatgcg
tgttcgattcgcgctaggggcac
1-28tRNA-Val(gac)[96973,97045]
gtccgtgtagctcaggggtagagcgcctgctcgacacgcaggaggaccga
ggttcgaaacctcgcatggacac
1-29tRNA-Arg(cct)[97219,97292]
gcctctgtagctcaacggacagagcaacgcggtcctaacgcggtggctgg
aggttcgaatcctctcggaggcac
1-31tRNA-Gln(ttg)[97833,97908]
tggggtatggtggcaatctggcagtcccgccggactttgactccggaggt
gcaggttcgagtcctgctaccccatc
1-32tRNA-Arg(tct)[97912,97987]
gcccttgtagctcagtggacagagcggcgagcttctacctcgcgggccgg
gagttcgaatctctccaggggcacca
1-33tRNA-Gln(ctg)[125802,125876]
tgctcgttggtgtaactggcaacactacggactctgactccgtcatttta
ggttcgaatcctaagcgagcagcca
1-34tRNA-Asn(gtt)[125883,125958]
tggggtgtccgttaatcaggcaaacgagcggactgttaatccgcccctgc
aggttcgaatcctgccaccccagcca
>JF704116.1 Mycobacterium phage Drazdys, complete sequence
1-1tRNA-Ser(gct)[31257,31340]
ggagggtgagcatcaggtgatgcagcgagattgctaatcccgtacggtaa
ccaccccgtgaggttcgaatcctcctccctccgc
1-2tRNA-Leu(cag)[31533,31608]
gctcccgtagcccaattggcaggaggcaccagattcaggatctgggcagt
gtgagttcgaatctcaccgggagtac
1-3tRNA-Leu(gag)[31728,31802]
gtctctgtaggcaaatcggaaaagccgccatcttgagggggtggtgcgtg
cgggttcgactcccgccagagacac
1-4tRNA-Leu(caa)[31803,31876]
gccgtggtaggccatctggcgagccgccgagttcaagtttcggtgtttgc
gggttcgaatcccgcccacggtac
1-5tRNA-SeC(tca)[67162,67258]
attctggcactggtgggcgagcccaccggcggcagcttcaagctgtcgct
ggccggagaaccgaccggaaacatcccgttcaacgcgacccagggcc

1-6tRNA-Pro(tgg)[91126,91198]
cggggtgtagttcagtggaagagcgcttggtttgggaccaagatgtcgca
ggttcgaatcctgtcaccccgac
1-7tRNA-Trp(cca)[91215,91290]
tggggtgaagccgatctggaaggcagcggtctccaaagccgtctcatagc
gggttcgaatcccgtcacccctgcca
1-8tRNA-Tyr(gta)[91292,91378]
gccgcacatgcccaactggtgtttgggagcaggctgtaaccctgtggcct
tcgggacggtgaggttcgattcctcagtgcggtacca
1-9tRNA-Pyl(cta)[92487,92559]
gcaccatttgctcaatggcagagcggcggcttctaaaaccgtgagtgccg
gttcgactccggcatggtgcacc
1-10tRNA-Met(cat)[92709,92783]
agcggtgtagagcagctaggtagctcgccgggctcataacccggaggacg
cgtgttcgaatcacgccaccgccac
1-11tRNA-Cys(gca)[92910,92981]
gcgcctttggcggaatggctacgtgctcggctgcaacccgagttatcccg
gttcgactccgggagggcgctc
1-12tRNA-Glu(ctc)[93049,93120]
gctcccatggggtagtggtaaccctcctggttctcagccaggcgtcccga
gttcgatcctcggtgggagtgc
1-13tRNA-His(gtg)[93175,93248]
gtggtagtagttcagatggaagaacgtcgccttgtgacggcgaaggtcgg
gggttcgaagcccctctatcaccc
1-14tRNA-Ala(tgc)[93919,93992]
gggcctatagctcatctggtagagcgcctgccttgcaagcaggaggcggc
aggttcaagtcctgttaggtccac
1-15tRNA-Phe(gaa)[94182,94254]
gccgtcatagctcagttggtagagcactggcctgaaaacccagtggccga
ggttcgattcctcgtgtcggcac
1-16tRNA-Val(cac)[94260,94333]
gtccgtttagctcagctggaagagcgctcggtccacacccgagaggccgc
aggttcgatccctgcaatggacac
1-17tRNA-Lys(ctt)[94452,94524]
gccttcgtagctcagtggtagagctgtcgcctcttaagcgataggtcgtt
ggttcgaatccagccgggggcac
1-18tRNA-Glu(ttc)[94529,94605]
ggtcgggtcggtctgctggtatggccagtcggattttcactccggacatt
cgcgggttcaattcccgtcccgatcgc
1-19tRNA-Gly(tcc)[94685,94757]
gcgggtgtggccgaatggctcaggcaccagatttccactctggctacgca
ggttcgattcctgtcatccgctc
1-20tRNA-Thr(cgt)[94817,94891]
gctgctgtagctcacctggcagagcgtcggcgtcgtatcccgaaggcatc

cggttcgagtccggacagcagcccc
1-21tRNA-Thr(tgt)[94892,94964]
gcctctgtggtccagcggcacggacatccgccttgtaagcggaggacccc
cgttcgatccggggtagaggctc
1-22tRNA-Thr(ggt)[95372,95444]
gctgggttagctcagtggtagagcgttcctctggtatgggaaaggcccgg
ggttcaatcccccgattcagctc
1-23tRNA-Gly(gcc)[96767,96840]
gcgaaggtagctcagctggtagagcgccaccttgccaaggtggaggtcgc
gggatcgtaacccgttcttcgctc
1-24tRNA-Asp(gtc)[96844,96916]
ggccctgtagctcagaggaagagcgccggtctgtcgaatcggaggtcgcg
gtatcgtaatccgtcagggtcgc
1-25tRNA-Met(cat)[96976,97048]
gcctcactagctcattggtagagccgctcgctcataacgtgcaggcacct
ggttcgattccagggtgaggtac
1-26tRNA-Ile(gat)[97054,97128]
gcctgttagcggactggtcgtccgatccaagctgataacttggcgtaagc
ggtgttcgattcaccgagcaggtac
1-27tRNA-Arg(acg)[97222,97294]
gcctctatggtccaacggatatgacgccggtctacggaaccggagatgcg
tgttcgattcgcgctaggggcac
1-28tRNA-Val(gac)[97337,97409]
gtccgtgtagctcaggggtagagcgcctgctcgacacgcaggaggaccga
ggttcgaaacctcgcatggacac
1-29tRNA-Arg(cct)[97583,97656]
gcctctgtagctcaacggacagagcaacgcggtcctaacgcggtggctgg
aggttcgaatcctctcggaggcac
1-31tRNA-Gln(ttg)[98197,98272]
tggggtatggtggcaatctggcagtcccgccggactttgactccggaggt
gcaggttcgagtcctgctaccccatc
1-32tRNA-Arg(tct)[98276,98351]
gcccttgtagctcagtggacagagcggcgagcttctacctcgcgggccgg
gagttcgaatctctccaggggcacca
1-33tRNA-Gln(ctg)[126574,126648]
tgctcgttggtgtaactggcaacactacggactctgactccgtcatttta
ggttcgaatcctaagcgagcagcca
1-34tRNA-Asn(gtt)[126655,126730]
tggggtgtccgttaatcaggcaaacgagcggactgttaatccgcccctgc
aggttcgaatcctgccaccccagcca
>JN699625.1 Mycobacterium phage Wally, complete genome
1-1tRNA-Ser(gct)[30107,30190]
ggagggtgagcatctggtgatgcaggggtcctgctaaggccctacggatt
cacacccgtgagtttcgattactcctccctccgc

1-2tRNA-Leu(cag)[30287,30363]
gccctgctgagcaaactggcaaagctgccgcattcagagtgcgggtcatt
tccgggttcgactcccgggcagggtac
1-3tRNA-Leu(gag)[30483,30557]
gtctctgtaggcaaatcggaaaagccgccatcttgagggggtggtgcgtg
cgggttcgactcccgccagagacac
1-4tRNA-Leu(caa)[30558,30631]
gccgtggtaggccatctggcgagccgccgagttcaagtttcggtgtttgc
gggttcgaatcccgcccacggtac
1-5tRNA-Pro(tgg)[90313,90387]
cggggtgtagttcagtttggaagagcgcttggtttgggaccaagatgtcg
caggttcgaatcctgtcaccccgac
1-6tRNA-Trp(cca)[90402,90472]
gggtctgtgcacagggtgcccgacggtctccaaagccgaaggcgggggtt
cgattccctccaggcctgcca
1-7tRNA-Tyr(gta)[90474,90560]
cccgtacatgcccaactggtgtttgggagcaggctgtaaatctgtggcct
tcgggacggtgaggttcgattcctcagtgcgggacca
1-8tRNA-Pyl(cta)[91614,91687]
tgcgagatcgtgcacggcgcactaggagcttctaaccctccgactcgcgg
gttcgactcccgcatctcgcaccc
1-9tRNA-Met(cat)[91835,91909]
agcggtgtagagcagctaggtagctcgccgggctcatgacccggaggacg
cgtgttcgattcacgccaccgccac
1-10tRNA-Cys(gca)[92036,92107]
gcgcctttggcggaatggctacgtgctcggctgcaacccgagttatcccg
gttcgactccgggagggcgctc
1-11tRNA-Glu(ctc)[92175,92246]
gctcccatggggtagtggtaaccctcctggttctcagccaggcgtcccga
gttcgatcctcggtgggagtgc
1-12tRNA-His(gtg)[92301,92374]
gtggtagtagttcagatggaagaacgtcgccttgtgacggcgaaggtcgg
gggttcgaagcccctctatcaccc
1-13tRNA-Ala(tgc)[93045,93119]
gggcctgtagctccaattggtagagcagcatccttgcaagatgacggctg
tcggttcgaatccgacctggtccac
1-14tRNA-Phe(gaa)[93309,93381]
gccgtcatagctcagttggtagagcactggcctgaaaacccagtggccga
ggttcgattcctcgtgtcggcac
1-15tRNA-Val(cac)[93387,93460]
gtccgtttagctcagctggaagagcgctcggtccacacccgagaggccgc
aggttcgatccctgcaatggacac
1-16tRNA-Lys(ctt)[93579,93651]
gccttcgtagctcagtggtagagctgtcgcctcttaagcgataggtcgtt

ggttcgaatccagccgggggcac
1-17tRNA-Glu(ttc)[93656,93732]
ggtcgggtcggtctgctggtatggccagtcggattttcactccggacatt
cgcgggttcaattcccgtcccgatcgc
1-18tRNA-Gly(tcc)[93812,93884]
gcgggtgtggccgaatggctcaggcaccagatttccactctggctacgca
ggttcgattcctgtcatccgctc
1-19tRNA-Thr(cgt)[93966,94040]
gctgctgtagctcacctggtagagcgtcggcgtcgtatcccgaaggcatc
cggttcgagtccggacagcagcccc
1-20tRNA-Thr(tgt)[94041,94113]
gcctctgtggtccagcggcacggacatccgccttgtaagcggaggacccc
cgttcgatccggggtagaggctc
1-21tRNA-Thr(ggt)[94521,94593]
gctgggttagctcagtggtagagcgttcctctggtatgggaaaggcccgg
ggttcaatcccccgactcagctc
1-22tRNA-Gly(gcc)[95916,95989]
gcgaaggtagctcagctggcagagcgccaccttgccaaggtggaggtcgc
gggatcgtaacccgttcttcgctc
1-23tRNA-Asp(gtc)[95993,96065]
ggccctgtagctcagaggaagagcgccgatctgtcgaatcggaggtcgcg
gtatcgtaatccgtcagggtcgc
1-24tRNA-Met(cat)[96125,96197]
gcctcactagctcattggtagagccgctcgctcataacgtgcaggtacct
ggttcgattccagggtgaggtac
1-25tRNA-Ile(gat)[96203,96277]
gcctgttagcggactggtcgtccgatccaagctgataacttggcgtaagc
ggtgttcgattcaccgagcaggtac
1-26tRNA-Arg(acg)[96371,96443]
gcctctatggtccaacggatatgacgccggtctacggaaccggagatgcg
tgttcgattcgcgctaggggcac
1-27tRNA-Val(gac)[96486,96558]
gtccgtgtagctcaggggtagagcgcctgctcgacacgcaggaggaccga
ggttcgaaacctcgcatggacac
1-28tRNA-Arg(cct)[96732,96805]
gcctctgtagctcaacggacagagcaacgcggtcctaacgcggtggctgg
aggttcgaatcctctcggaggcac
1-30tRNA-Gln(ttg)[97346,97421]
tggggtatggtggcaatctggcagtcccgccggactttgactccggaggt
gcaggttcgagtcctgctaccccatc
1-31tRNA-Arg(tct)[97425,97498]
gcccttgtagctcagtggacagagcggcgagcttctacctcgcgggccgg
gagttcgaatctctccaggggcac
1-32tRNA-Lys(ttt)[97566,97642]

gggccggtatcttagtctggtcaaagaagtggacttttaatccgcgcgcc
gtgggttcgaatcccacccggcccacc
1−33tRNA−Gln(ctg)[125172,125246]
tgctcgttggtgtaactggcaacactacggactctgactccgtcatttta
ggttcgaatcctaagcgagcagcca
1−34tRNA−Asn(gtt)[125253,125328]
tggggtgtccgttaatcaggcaaacgagcggactgttaatccgcccctgc
aggttcgaatcctgccaccccagcca
>JN699013.1 Mycobacterium phage Pio, complete genome
1−1tRNA−Ser(gct)[32481,32564]
ggagggtgagcatctggtgatgcaggggtcctgctaaggccctacggatt
cacacccgtgagtttcgattactcctccctccgc
1−2tRNA−Leu(cag)[32661,32737]
gccctgctgagcaaactggcaaagctgccgcattcagagtgcgggtcatt
tccgggttcgactcccgggcagggtac
1−3tRNA−Leu(gag)[32857,32931]
gtctctgtaggcaaatcggaaaagccgccatcttgagggggtggtgcgtg
cgggttcgactcccgccagagacac
1−4tRNA−Leu(caa)[32932,33005]
gccgtggtaggccatctggcgagccgccgagttcaagtttcggtgtttgc
gggttcgaatcccgcccacggtac
1−5tRNA−SeC(tca)[70365,70461]
attctggcactggtgggcgagcccaccggcggcagcttcaagctgtcgct
ggccggagaaccgaccggaaacatcccgttcaacgcgacccagggcc
1−6tRNA−Pro(tgg)[94729,94803]
cggggtgtagttcagtttggaagagcgcttggtttgggaccaagatgtcg
caggttcgaatcctgtcaccccgac
1−7tRNA−Trp(cca)[94820,94895]
tggggtgaagccgatctggaaggcagcggtctccaaagccgtctcatagc
gggttcgaatcccgtcacccctgcca
1−8tRNA−Tyr(gta)[94897,94983]
gccgcacatgcccaactggtgtttgggagcaggctgtaaccctgtggcct
tcgggacggtgaggttcgattcctcagtgcggtacca
1−9tRNA−Pyl(cta)[96092,96164]
gcaccatttgctcaatggcagagcggcggcttctaaccccgtgagtgccg
gttcgattccggcatggtgcacc
1−10tRNA−Met(cat)[96314,96388]
agcggtgtagagcagctaggtagctcgccgggctcataacccggaggacg
cgtgttcgaatcacgccaccgccac
1−11tRNA−Cys(gca)[96515,96586]
gcgcctttggcggaatggctacgtgctcggctgcaacccgagttatcccg
gttcgactccgggagggcgctc
1−12tRNA−Glu(ctc)[96654,96725]
gctcccatggggtagtggtaaccctcctggttctcagccaggcgtcccga

gttcgatcctcggtgggagtgc
1-13tRNA-His(gtg)[96727,96800]
gtggccgtagttcagccggtagaacgctgggttgtgatcccagtcgtcga
gggttcgagtccctccggtcaccc
1-14tRNA-Ala(tgc)[96962,97036]
gggcctgtagctccaattggtagagcagcatccttgcaagatgacggctg
tcggttcgaatccgacctggtccac
1-15tRNA-Phe(gaa)[97226,97298]
gccgtcatagctcagttggtagagcactggcctgaaaacccagtggccga
ggttcgagtcctcgtgtcggcac
1-16tRNA-Val(cac)[97304,97377]
gtccgtttagctcagctggaagagcgctcggtccacacccgagaggccgc
aggttcgatccctgcaatggacac
1-17tRNA-Lys(ctt)[97496,97568]
gccttcgtagctcagtggtagagctgtcgcctcttaagcgataggtcgtt
ggttcgaatccagccgggggcac
1-18tRNA-Glu(ttc)[97573,97649]
ggtcgggtcggtctgctggtatggccagtcggattttcactccggacatt
cgcgggttcaattcccgtcccgatcgc
1-19tRNA-Gly(tcc)[97729,97801]
gcgggtgtggccgaatggctcaggcaccagatttccactctggctacgca
ggttcgattcctgtcatccgctc
1-20tRNA-Thr(cgt)[97861,97935]
gctgctgtagctcacctggcagagcgtcggcgtcgtatcccgaaggcatc
cggttcgagtccggacagcagcccc
1-21tRNA-Thr(tgt)[97936,98008]
gcctctgtggtccagcggcacggacatccgccttgtaagcggaggacccc
cgttcgatccggggtagaggctc
1-22tRNA-Thr(ggt)[98416,98488]
gctgggttagctcagtggtagagcgttcctctggtatgggaaaggcccgg
ggttcaatcccccgattcagctc
1-23tRNA-Gly(gcc)[99811,99884]
gcgaaggtagctcagctggcagagcgccaccttgccaaggtggaggtcgc
gggatcgtaacccgttcttcgctc
1-24tRNA-Asp(gtc)[99888,99960]
ggccctgtagctcagaggaagagcgccggtctgtcgaatcggaggtcgcg
gtatcgtaatccgtcagggtcgc
1-25tRNA-Met(cat)[100020,100092]
gcctcactagctcattggtagagccgctcgctcataacgtgcaggtacct
ggttcgattccagggtgaggtac
1-26tRNA-Ile(gat)[100098,100172]
gcctgttagcggactggtcgtccgatccaagctgataacttggcgtaagc
ggtgttcgattcaccgagcaggtac
1-27tRNA-Arg(acg)[100266,100338]

gcctctatggtccaacggatatgacgccggtctacggaaccggagatgcg
tgttcgattcgcgctaggggcac
1-28tRNA-Val(gac)[100381,100453]
gtccgtgtagctcaggggtagagcgcctgctcgacacgcaggaggaccga
ggttcgaaacctcgcatggacac
1-29tRNA-Arg(cct)[100627,100700]
gcctctgtagctcaacggacagagcaacgcggtcctaacgcggtggctgg
aggttcgaatcctctcggaggcac
1-31tRNA-Gln(ttg)[101241,101316]
tggggtatggtggcaatctggcagtcccgccggactttgactccggaggt
gcaggttcgagtcctgctaccccatc
1-32tRNA-Arg(tct)[101320,101395]
gcccttgtagctcagtggacagagcggcgagcttctacctcgcgggccgg
gagttcgaatctctccaggggcacca
1-33tRNA-Gln(ctg)[127922,127996]
tgctcgttggtgtaactggcaacactacggactctgactccgtcatttta
ggttcgaatcctaagcgagcagcca
1-34tRNA-Asn(gtt)[128003,128078]
tggggtgtccgttaatcaggcaaacgagcggactgttaatccgcccctgc
aggttcgaatcctgccaccccagcca
>JQ911768.1 Mycobacterium phage Ava3, complete genome
1-1tRNA-Ser(gct)[31611,31694]
ggagggtgagcatctggtgatgcaggggtcctgctaaggccctacggatt
cacacccgtgagtttcgattactcctccctccgc
1-2tRNA-Leu(cag)[31791,31867]
gccctgctgagcaaactggcaaagctgccgcattcagagtgcgggtcatt
tccgggttcgactcccgggcagggtac
1-3tRNA-Leu(gag)[31987,32061]
gtctctgtaggcaaatcggaaaagccgccatcttgagggggtggtgcgtg
cgggttcgactcccgccagagacac
1-4tRNA-Leu(caa)[32062,32135]
gccgtggtaggccatctggcgagccgccgagttcaagtttcggtgtttgc
gggttcgaatcccgcccacggtac
1-5tRNA-SeC(tca)[67921,68017]
attctggcactggtgggcgagcccaccggcggcagcttcaagctgtcgct
ggccggagaaccgaccggaaacatcccgttcaacgcgacccagggcc
1-6tRNA-Pro(tgg)[91216,91290]
cggggtgtagttcagtttggaagagcgcttggtttgggaccaagttgtcg
caggttcgaatcctgtcaccccgac
1-7tRNA-Trp(cca)[91305,91375]
gggtctgtgcacagggtgcccgacggtctccaaagccgaaggcgggggtt
cgattccctccaggcctgcca
1-8tRNA-Pyl(cta)[92572,92644]
gcaccatttgctcaatggcagagcggcggcttctaaaaccgtgagtgccg

gttcgactccggcatggtgcacc
1-9tRNA-Met(cat)[92794,92868]
agcggtgtagagcagctaggtagctcgccgggctcataacccggaggacg
cgtgttcgaatcacgccaccgccac
1-10tRNA-Cys(gca)[92995,93066]
gcgcctttggcggaatggctacgtgctcggctgcaacccgagttatcccg
gttcgactccgggagggcgctc
1-11tRNA-Glu(ctc)[93071,93145]
ggtccgttggagtagatggatatctcgccaccctctcaaggtggagatca
cgggttcaagtcccgtacggactgc
1-12tRNA-His(gtg)[93147,93220]
gtggccgtagttcagccggtagaacgctgggttgtgatcccagtcgtcga
gggttcgagtccctccggtcaccc
1-13tRNA-Ala(tgc)[93382,93456]
gggcctgtagctccaattggtagagcagcatccttgcaagatgacggctg
tcggttcgaatccgacctggtccac
1-14tRNA-Phe(gaa)[93646,93718]
gccgtcatagctcagttggtagagcactggcctgaaaacccagtggccga
ggttcgattcctcgtgtcggcac
1-15tRNA-Val(cac)[93724,93797]
gtccgtttagctcagctggaagagcgctcggtccacacccgagaggccgc
aggttcgatccctgcaatggacac
1-16tRNA-Lys(ctt)[93916,93988]
gccttcgtagctcagtggtagagctgtcgcctcttaagcgataggtcgtt
ggttcgaatccagccgggggcac
1-17tRNA-Glu(ttc)[93993,94069]
ggtcgggtcggtctgctggtatggccagtcggattttcactccggacatt
cgcgggttcaattcccgtcccgatcgc
1-18tRNA-Gly(tcc)[94149,94221]
gcgggtgtggccgaatggctcaggcaccagatttccactctggctacgca
ggttcgattcctgtcatccgctc
1-19tRNA-Thr(cgt)[94281,94355]
gctgctgtagctcacctggcagagcgtcggcgtcgtatcccgaaggcatc
cggttcgagtccggacagcagcccc
1-20tRNA-Thr(tgt)[94356,94428]
gcctctgtggtccagcggcacggacatccgccttgtaagcggaggacccc
cgttcgatccggggtagaggctc
1-21tRNA-Thr(ggt)[94488,94560]
gctgggttagctcagtggtagagcgttcccttggtatgggaaaggcccgg
ggttcaatcccccgactcagctc
1-22tRNA-Gly(gcc)[95883,95956]
gcgaaggtagctcagctggcagagcgccaccttgccaaggtggaggtcgc
gggatcgtaacccgttcttcgctc
1-23tRNA-Asp(gtc)[95960,96032]

ggccctgtagctcagaggaagagcgccggtctgtcgaatcggaggtcgcg
gtatcgtaatccgtcagggtcgc
1-24tRNA-Met(cat)[96092,96164]
gcctcactagctcattggtagagccgctcgctcataacgtgcaggtacct
ggttcgattccagggtgaggtac
1-25tRNA-Ile(gat)[96170,96244]
gcctgttagcggactggtcgtccgatccaagctgataacttggcgtaagc
ggtgttcgattcaccgagcaggtac
1-26tRNA-Arg(acg)[96338,96410]
gcctctatggtccaacggatatgacgccggtctacggaaccggagatgcg
tgttcgattcgcgctaggggcac
1-27tRNA-Val(gac)[96453,96525]
gtccgtgtagctcaggggtagagcgcctgctcgacacgcaggaggaccga
ggttcgaaacctcgcatggacac
1-28tRNA-Arg(cct)[96699,96772]
gcctctgtagctcaacggacagagcaacgcggtcctaacgcggtggctgg
aggttcgaatcctctcggaggcac
1-30tRNA-Gln(ttg)[97313,97388]
tggggtatggtggcaatctggcagtcccgccggactttgactccggaggt
gcaggttcgagtcctgctaccccatc
1-31tRNA-Arg(tct)[97392,97465]
gcccttgtagctcagtggacagagcggcgagcttctacctcgcgggccgg
gagttcgaatctctccaggggcac
1-32tRNA-Lys(ttt)[97533,97609]
gggccggtatcttagtctggtcaaagaagtggacttttaatccgcgcgcc
gtgggttcgaatcccacccggcccacc
1-33tRNA-Gln(ctg)[125647,125721]
tgctcgttggtgtaactggcaacactacggactctgactccgtcatttta
ggttcgaatcctaagcgagcagcca
1-34tRNA-Asn(gtt)[125728,125803]
tggggtgtccgttaatcaggcaaacgagcggactgttaatccgcccctgc
aggttcgaatcctgccaccccagcca
>KF024734.1 Mycobacterium phage Shrimp, complete genome
1-1tRNA-Ser(gct)[31556,31639]
ggagggtgagcatctggtgatgcaggggtcctgctaaggccctacggatt
cacacccgtgagtttcgattactcctccctccgc
1-2tRNA-Leu(cag)[31736,31812]
gccctgctgagcaaactggcaaagctgccgcattcagagtgcgggtcatt
tccgggttcgactcccgggcagggtac
1-3tRNA-Leu(gag)[31932,32006]
gtctctgtaggcaaatcggaaaagccgccatcttgagggggtggtgcgtg
cgggttcgactcccgccagagacac
1-4tRNA-Leu(caa)[32007,32080]
gccgtggtaggccatctggcgagccgccgagttcaagtttcggtgtttgc

gggttcgaatcccgcccacggtac
1−5tRNA−SeC(tca)[68484,68580]
attctggcactggtgggcgagcccaccggcggcagcttcaagctgtcgct
ggccggagaaccgaccggaaacatcccgttcaacgcgacccagggcc
1−6tRNA−Pro(tgg)[92444,92516]
cggggtgtagttcagtggaagagcgcttggtttgggaccaagatgtcgca
ggttcgaatcctgtcaccccgac
1−7tRNA−Trp(cca)[92533,92608]
tggggtgaagccgatctggaaggcagcggtctccaaagccgtctcatagc
gggttcgaatcccgtcacccctgcca
1−8tRNA−Tyr(gta)[92610,92695]
gccgcacatgcccaactggtgtttgggagcaggctgtaaccctgtggcct
cgggacggtgaggttcgattcctcagtgcggtacca
1−9tRNA−Pyl(cta)[93804,93876]
gcaccatttgctcaatggcagagcggcggcttctaaccccgtgagtgccg
gttcgattccggcatggtgcacc
1−10tRNA−Met(cat)[94026,94100]
agcggtgtagagcagctaggtagctcgccgggctcataacccggaggacg
cgtgttcgaatcacgccaccgccac
1−11tRNA−Cys(gca)[94227,94298]
gcgcctttggcggaatggctacgtgctcggctgcaacccgagttatcccg
gttcgactccgggagggcgctc
1−12tRNA−Glu(ctc)[94303,94377]
ggtccgttggagtagatggatatctcgccaccctctcaaggtggagatca
cgggttcaagtcccgtacggactgc
1−13tRNA−His(gtg)[94379,94452]
gtggccgtagttcagccggtagaacgctgggttgtgatcccagtcgtcga
gggttcgagtccctccggtcaccc
1−14tRNA−Ala(tgc)[94614,94688]
gggcctgtagctccaattggtagagcagcatccttgcaagatgacggctg
tcggttcgaatccgacctggtccac
1−15tRNA−Phe(gaa)[94878,94950]
gccgtcatagctcagttggtagagcactggcctgaaaacccagtggccga
ggttcgattcctcgtgtcggcac
1−16tRNA−Val(cac)[94956,95029]
gtccgtttagctcagctggaagagcgctcggtccacacccgagaggccgc
aggttcgatccctgcaatggacac
1−17tRNA−Lys(ctt)[95148,95220]
cgccttcgtagctcagtggtagagctgtcgcctcttaagcgataggtcgt
tggttcgaatccagccggggcac
1−18tRNA−Glu(ttc)[95225,95301]
ggtcgggtcggtctgctggtatggccagtcggattttcactccggacatt
cgcgggttcaattcccgtcccgatcgc
1−19tRNA−Gly(tcc)[95382,95454]

gcgggtgtggccgaatggctcaggcaccagatttccactctggctacgca
ggttcgattcctgtcatccgctc
1-20tRNA-Thr(cgt)[95514,95588]
gctgctgtagctcacctggcagagcgtcggcgtcgtatcccgaaggcatc
cggttcgagtccggacagcagcccc
1-21tRNA-Thr(tgt)[95589,95661]
gcctctgtggtccagcggcacggacatccgccttgtaagcggaggacccc
cgttcgatccggggtagaggctc
1-22tRNA-Thr(ggt)[96069,96141]
gctgggttagctcagtggtagagcgttcccttggtatgggaaaggcccgg
ggttcaatcccccgactcagctc
1-23tRNA-Gly(gcc)[97464,97537]
gcgaaggtagctcagctggcagagcgccaccttgccaaggtggaggtcgc
gggatcgtaacccgttcttcgctc
1-24tRNA-Asp(gtc)[97541,97613]
ggccctgtagctcagaggaagagcgccggtctgtcgaatcggaggtcgcg
gtatcgtaatccgtcagggtcgc
1-25tRNA-Met(cat)[97673,97745]
gcctcactagctcattggtagagccgctcgctcataacgtgcaggtacct
ggttcgattccagggtgaggtac
1-26tRNA-Ile(gat)[97751,97825]
gcctgttagcggactggtcgtccgatccaagctgataacttggcgtaagc
ggtgttcgattcaccgagcaggtac
1-27tRNA-Arg(acg)[97919,97991]
gcctctatggtccaacggatatgacgccggtctacggaaccggagatgcg
tgttcgattcgcgctaggggcac
1-28tRNA-Val(gac)[98034,98106]
gtccgtgtagctcaggggtagagcgcctgctcgacacgcaggaggaccga
ggttcgaaacctcgcatggacac
1-29tRNA-Arg(cct)[98280,98353]
gcctctgtagctcaacggacagagcaacgcggtcctaacgcggtggctgg
aggttcgaatcctctcggaggcac
1-31tRNA-Gln(ttg)[98894,98969]
tggggtatggtggcaatctggcagtcccgccggactttgactccggaggt
gcaggttcgagtcctgctaccccatc
1-32tRNA-Arg(tct)[98973,99048]
gcccttgtagctcagtggacagagcggcgagcttctacctcgcgggccgg
gagttcgaatctctccaggggcacca
1-33tRNA-Gln(ctg)[126955,127029]
tgctcgttggtgtaactggcaacactacggactctgactccgtcatttta
ggttcgaatcctaagcgagcagcca
1-34tRNA-Asn(gtt)[127036,127111]
tggggtgtccgttaatcaggcaaacgagcggactgttaatccgcccctgc
aggttcgaatcctgccaccccagcca

>KJ595575.1 Mycobacterium phage Willis, complete genome

1-1tRNA-Ser(gct)[31447,31530]

ggagggtgagcatctggtgatgcaggggtcctgctaaggccctacggatt

cacacccgtgagtttcgattactcctccctccgc

1-2tRNA-Leu(cag)[31627,31703]

gccctgctgagcaaactggcaaagctgccgcattcagagtgcgggtcatt

tccgggttcgactcccgggcagggtac

1-3tRNA-Leu(gag)[31823,31897]

gtctctgtaggcaaatcggaaaagccgccatcttgaggggggtggtgcgtg

cgggttcgactcccgccagagacac

1-4tRNA-Leu(caa)[31898,31971]

gccgtggtaggccatctggcgagccgccgagttcaagtttcggtgtttgc

gggttcgaatcccgcccacggtac

1-5tRNA-SeC(tca)[68375,68471]

attctggcactggtgggcgagcccaccggcggcagcttcaagctgtcgct

ggccggagaaccgaccggaaacatcccgttcaacgcgacccagggcc

1-6tRNA-Pro(tgg)[91680,91754]

cggggtgtagttcagtttggaagagcgcttggtttgggaccaagttgtcg

caggttcgaatcctgtcaccccgac

1-7tRNA-Trp(cca)[91769,91839]

gggtctgtgcacagggtgcccgacggtctccaaagccgaaggcgggggtt

cgattccctccaggcctgcca

1-8tRNA-Pyl(cta)[93036,93108]

gcaccatttgctcaatggcagagcggcggcttctaaaaccgtgagtgccg

gttcgactccggcatggtgcacc

1-9tRNA-Met(cat)[93258,93332]

agcggtgtagagcagctaggtagctcgccgggctcataacccggaggacg

cgtgttcgaatcacgccaccgccac

1-10tRNA-Cys(gca)[93459,93530]

gcgcctttggcggaatggctacgtgctcggctgcaacccgagttatcccg

gttcgactccgggagggcgctc

1-11tRNA-Glu(ctc)[93535,93609]

ggtccgttggagtagatggatatctcgccaccctctcaaggtggagatca

cgggttcaagtcccgtacggactgc

1-12tRNA-His(gtg)[93611,93684]

gtggccgtagttcagccggtagaacgctgggttgtgatcccagtcgtcga

gggttcgagtccctccggtcaccc

1-13tRNA-Ala(tgc)[93846,93920]

gggcctgtagctccaattggtagagcagcatccttgcaagatgacggctg

tcggttcgaatccgacctggtccac

1-14tRNA-Phe(gaa)[94110,94182]

gccgtcatagctcagttggtagagcactggcctgaaaacccagtggccga

ggttcgattcctcgtgtcggcac

1-15tRNA-Val(cac)[94188,94261]

gtccgtttagctcagctggaagagcgctcggtccacacccgagaggccgc
aggttcgatccctgcaatggacac
1-16tRNA-Lys(ctt)[94380,94452]
gccttcgtagctcagtggtagagctgtcgcctcttaagcgataggtcgtt
ggttcgaatccagccgggggcac
1-17tRNA-Glu(ttc)[94457,94533]
ggtcgggtcggtctgctggtatggccagtcggattttcactccggacatt
cgcgggttcaattcccgtcccgatcgc
1-18tRNA-Gly(tcc)[94613,94685]
gcgggtgtggccgaatggctcaggcaccagatttccactctggctacgca
ggttcgattcctgtcatccgctc
1-19tRNA-Thr(cgt)[94745,94819]
gctgctgtagctcacctggcagagcgtcggcgtcgtatcccgaaggcatc
cggttcgagtccggacagcagcccc
1-20tRNA-Thr(tgt)[94820,94892]
gcctctgtggtccagcggcacggacatccgccttgtaagcggaggacccc
cgttcgatccggggtagaggctc
1-21tRNA-Thr(ggt)[94952,95024]
gctgggttagctcagtggtagagcgttcccttggtatgggaaaggcccgg
ggttcaatcccccgactcagctc
1-22tRNA-Gly(gcc)[96347,96420]
gcgaaggtagctcagctggcagagcgccaccttgccaaggtgggggtcgc
gggatcgtaacccgttcttcgctc
1-23tRNA-Asp(gtc)[96424,96496]
ggccctgtagctcagaggaagagcgccggtctgtcgaatcggaggtcgcg
gtatcgtaatccgtcagggtcgc
1-24tRNA-Met(cat)[96556,96628]
gcctcactagctcattggtagagccgctcgctcataacgtgcaggtacct
ggttcgattccagggtgaggtac
1-25tRNA-Ile(gat)[96634,96708]
gcctgttagcggactggtcgtccgatccaagctgataacttggcgtaagc
ggtgttcgattcaccgagcaggtac
1-26tRNA-Arg(acg)[96802,96874]
gcctctatggtccaacggatatgacgccggtctacggaaccggagatgcg
tgttcgattcgcgctaggggcac
1-27tRNA-Val(gac)[96917,96989]
gtccgtgtagctcaggggtagagcgcctgctcgacacgcaggaggaccga
ggttcgaaacctcgcatggacac
1-28tRNA-Arg(cct)[97163,97236]
gcctctgtagctcaacggacagagcaacgcggtcctaacgcggtggctgg
aggttcgaatcctctcggaggcac
1-30tRNA-Gln(ttg)[97777,97852]
tggggtatggtggcaatctggcagtcccgccggactttgactccggaggt
gcaggttcgagtcctgctaccccatc

1-31tRNA-Arg(tct)[97856,97929]
gcccttgtagctcagtggacagagcggcgagcttctacctcgcgggccgg
gagttcgaatctctccaggggcac
1-32tRNA-Lys(ttt)[97997,98073]
gggccggtatcttagtctggtcaaagaagtggacttttaatccgcgcgcc
gtgggttcgaatcccacccggcccacc
1-33tRNA-Gln(ctg)[126111,126185]
tgctcgttggtgtaactggcaacactacggactctgactccgtcatttta
ggttcgaatcctaagcgagcagcca
1-34tRNA-Asn(gtt)[126192,126267]
tggggtgtccgttaatcaggcaaacgagcggactgttaatccgcccctgc
aggttcgaatcctgccaccccagcca
>KM881426.1 Mycobacterium phage ZygoTaiga
1-1tRNA-Ser(gct)[31553,31636]
ggagggtgagcatctggtgatgcaggggtcctgctaaggccctacggatt
cacacccgtgagtttcgattactcctccctccgc
1-2tRNA-Leu(cag)[31733,31809]
gccctgctgagcaaactggcaaagctgccgcattcagagtgcgggtcatt
tccgggttcgactcccgggcagggtac
1-3tRNA-Leu(gag)[31929,32003]
gtctctgtaggcaaatcggaaaagccgccatcttgagggggtggtgcgtg
cgggttcgactcccgccagagacac
1-4tRNA-Leu(caa)[32004,32077]
gccgtggtaggccatctggcgagccgccgagttcaagtttcggtgtttgc
gggttcgaatcccgcccacggtac
1-5tRNA-SeC(tca)[69405,69501]
attctggcactggtgggcgagcccaccggcggcagcttcaagctgtcgct
ggccggagaaccgaccggaaacatcccgttcaacgcgacccagggcc
1-6tRNA-Pro(tgg)[92700,92774]
cggggtgtagttcagtttggaagagcgcttggtttgggaccaagatgtcg
caggttcgaatcctgtcaccccgac
1-7tRNA-Trp(cca)[92789,92859]
gggtctgtgcacagggtgcccgacggtctccaaagccgaaggcgggggtt
cgattccctccaggcctgcca
1-8tRNA-Pyl(cta)[94056,94128]
gcaccatttgctcaatggcagagcggcggcttctaaaaccgtgagtgccg
gttcgactccggcatggtgcacc
1-9tRNA-Met(cat)[94278,94352]
agcggtgtagagcagctaggtagctcgccgggctcataacccggaggacg
cgtgttcgaatcacgccaccgccac
1-10tRNA-Cys(gca)[94479,94550]
gcgcctttggcggaatggctacgtgctcggctgcaacccgagttatcccg
gttcgactccgggagggcgctc
1-11tRNA-Glu(ctc)[94555,94629]

ggtccgttggagtagatggatatctcgccaccctctcaaggtggagatca
cgggttcaagtcccgtacggactgc
1-12tRNA-His(gtg)[94631,94704]
gtggccgtagttcagccggtagaacgctgggttgtgatcccagtcgtcga
gggttcgagtccctccggtcaccc
1-13tRNA-Ala(tgc)[94866,94940]
gggcctgtagctccaattggtagagcagcatccttgcaagatgacggctg
tcggttcgaatccgacctggtccac
1-14tRNA-Phe(gaa)[95130,95202]
gccgtcatagctcagttggtagagcactggcctgaaaacccagtggccga
ggttcgattcctcgtgtcggcac
1-15tRNA-Val(cac)[95208,95281]
gtccgtttagctcagctggaagagcgctcggtccacacccgagaggccgc
aggttcgatccctgcaatggacac
1-16tRNA-Lys(ctt)[95400,95472]
gccttcgtagctcagtggtagagctgtcgcctcttaagcgataggtcgtt
ggttcgaatccagccgggggcac
1-17tRNA-Glu(ttc)[95477,95553]
ggtcgggtcggtctgctggtatggccagtcggattttcactccggacatt
cgcgggttcaattcccgtcccgatcgc
1-18tRNA-Gly(tcc)[95633,95705]
gcgggtgtggccgaatggctcaggcaccagatttccactctggctacgca
ggttcgattcctgtcatccgctc
1-19tRNA-Thr(cgt)[95765,95839]
gctgctgtagctcacctggcagagcgtcggcgtcgtatcccgaaggcatc
cggttcgagtccggacagcagcccc
1-20tRNA-Thr(tgt)[95840,95912]
gcctctgtggtccagcggcacggacatccgccttgtaagcggaggacccc
cgttcgatccggggtagaggctc
1-21tRNA-Thr(ggt)[95972,96044]
gctgggttagctcagtggtagagcgttcccttggtatgggaaaggcccgg
ggttcaatcccccgactcagctc
1-22tRNA-Gly(gcc)[97367,97440]
gcgaaggtagctcagctggcagagcgccaccttgccaaggtggaggtcgc
gggatcgtaacccgttcttcgctc
1-23tRNA-Asp(gtc)[97444,97516]
ggccctgtagctcagaggaagagcgccggtctgtcgaatcggaggtcgcg
gtatcgtaatccgtcagggtcgc
1-24tRNA-Met(cat)[97576,97648]
gcctcactagctcattggtagagccgctcgctcataacgtgcaggtacct
ggttcgattccagggtgaggtac
1-25tRNA-Ile(gat)[97654,97728]
gcctgttagcggactggtcgtccgatccaagctgataacttggcgtaagc
ggtgttcgattcaccgagcaggtac

1–26tRNA–Arg(acg)[97822,97894]
gcctctatggtccaacggatatgacgccggtctacggaaccggagatgcg
tgttcgattcgcgctaggggcac
1–27tRNA–Val(gac)[97937,98009]
gtccgtgtagctcaggggtagagcgcctgctcgacacgcaggaggaccga
ggttcgaaacctcgcatggacac
1–28tRNA–Arg(cct)[98183,98256]
gcctctgtagctcaacggacagagcaacgcggtcctaacgcggtggctgg
aggttcgaatcctctcggaggcac
1–30tRNA–Gln(ttg)[98797,98872]
tggggtatggtggcaatctggcagtcccgccggactttgactccggaggt
gcaggttcgagtcctgctaccccatc
1–31tRNA–Arg(tct)[98876,98949]
gcccttgtagctcagtggacagagcggcgagcttctacctcgcgggccgg
gagttcgaatctctccaggggcac
1–32tRNA–Lys(ttt)[99017,99093]
gggccggtatcttagtctggtcaaagaagtggacttttaatccgcgcgcc
gtgggttcgaatcccacccggcccacc
1–33tRNA–Gln(ctg)[127131,127205]
tgctcgttggtgtaactggcaacactacggactctgactccgtcatttta
ggttcgaatcctaagcgagcagcca
1–34tRNA–Asn(gtt)[127212,127287]
tggggtgtccgttaatcaggcaaacgagcggactgttaatccgcccctgc
aggttcgaatcctgccaccccagcca
>KR080196.1 Mycobacterium phage Momo, complete genome
1–1tRNA–Ser(gct)[30462,30545]
ggagggtgagcatcaggtgatgcagcgagattgctaatcccgtacggtaa
ccaccccgtgaggttcgaatcctcctccctccgc
1–2tRNA–Leu(cag)[30738,30813]
gctcccgtagcccaattggcaggaggcaccagattcaggatctgggcagt
gtgagttcgaatctcaccgggagtac
1–3tRNA–Leu(gag)[30933,31007]
gtctctgtaggcaaatcggaaaagccgccatcttgagggggtggtgcgtg
cgggttcgactcccgccagagacac
1–4tRNA–Leu(caa)[31008,31081]
gccgtggtaggccatctggcgagccgccgagttcaagtttcggtgtttgc
gggttcgaatcccgcccacggtac
1–5tRNA–SeC(tca)[67487,67583]
attctggcactggtgggcgagcccaccggcggcagcttcaagctgtcgct
ggccggagaaccgaccggaaacatcccgttcaacgcgacccagggcc
1–6tRNA–Pro(tgg)[90741,90815]
cggggtgtagttcagtttggaagagcgcttggtttgggaccaagatgtcg
caggttcgaatcctgtcaccccgac
1–7tRNA–Trp(cca)[90830,90900]

gggtctgtgcacagggtgcccgacggtctccaaagccgaaggcgggggtt
cgattccctccaggcctgcca
1-8tRNA-Tyr(gta)[90902,90988]
cccgtacatgcccaactggtgtttgggagcaggctgtaaatctgtggcct
tcgggacggtgaggttcgattcctcagtgcgggacca
1-9tRNA-Pyl(cta)[92042,92115]
tgcgagatcgtgcacggcgcactaggagcttctaaccctccgactcgcgg
gttcgactcccgcatctcgcaccc
1-10tRNA-Met(cat)[92263,92337]
agcggtgtagagcagctaggtagctcgccgggctcataacccggaggacg
cgtgttcgattcacgccaccgccac
1-11tRNA-Cys(gca)[92464,92535]
gcgcctttggcggaatggctacgtgctcggctgcaacccgagttatcccg
gttcgactccgggagggcgctc
1-12tRNA-Glu(ctc)[92540,92614]
ggtccgttggagtagatggacatctcgccaccctctcaaggtggagatca
cgggttcaagtcccgtacggactgc
1-13tRNA-His(gtg)[92616,92689]
gtggccgtagttcagccggtagaacgctgggttgtgatcccagtcgtcga
gggttcgagtccctccggtcaccc
1-14tRNA-Ala(tgc)[92851,92925]
gggcctgtagctccaattggtagagcagcatccttgcaagatgacggctg
tcggttcgaatccgacctggtccac
1-15tRNA-Phe(gaa)[93115,93187]
gccgtcatagctcagttggtagagcactggcctgaaaacccagtggccga
ggttcgattcctcgtgtcggcac
1-16tRNA-Val(cac)[93193,93266]
gtccgtttagctcagctggaagagcgctcggtccacacccgagaggccgc
aggttcgatccctgcaatggacac
1-17tRNA-Lys(ctt)[93385,93457]
gccttcgtagctcagtggtagagctgtcgcctcttaagcgataggtcgtt
ggttcgaatccagccgggggcac
1-18tRNA-Glu(ttc)[93462,93538]
ggtcgggtcggtctgctggtatggccagtcggattttcactccggacatt
cgcgggttcaattcccgtcccgatcgc
1-19tRNA-Gly(tcc)[93618,93690]
gcgggtgtggccgaatggctcaggcaccagatttccactctggctacgca
ggttcgattcctgtcatccgctc
1-20tRNA-Thr(cgt)[93750,93824]
gctgctgtagctcacctggcagagcgtcggcgtcgtatcccgaaggcatc
cggttcgagtccggacagcagcccc
1-21tRNA-Thr(tgt)[93825,93897]
gcctctgtggtccagcggcacggacatccgccttgtaagcggaggacccc
cgttcgatccggggtagaggctc

1–22tRNA–Thr(ggt)[94305,94377]
gctgggttagctcagtggtagagcgttcctctggtatgggaaaggcccgg
ggttcaatcccccgactcagctc
1–23tRNA–Gly(gcc)[95700,95773]
gcgaaggtagctcagctggcagagcgccaccttgccaaggtggaggtcgc
gggatcgtaacccgttcttcgctc
1–24tRNA–Asp(gtc)[95777,95849]
ggccctgtagctcagaggaagagcgccggtctgtcgaatcggaggtcgcg
gtatcgtaatccgtcagggtcgc
1–25tRNA–Met(cat)[95909,95981]
gcctcactagctcattggtagagccgctcgctcataacgtgcaggtacct
ggttcgattccagggtgaggtac
1–26tRNA–Ile(gat)[95987,96061]
gcctgttagcggactggtcgtccgatccaagctgataacttggcgtaagc
ggtgttcgattcaccgagcaggtac
1–27tRNA–Arg(acg)[96155,96227]
gcctctatggtccaacggatatgacgccggtctacggaaccggagatgcg
tgttcgattcgcgctaggggcac
1–28tRNA–Val(gac)[96270,96342]
gtccgtgtagctcaggggtagagcgcctgctcgacacgcaggaggaccga
ggttcgaaacctcgcatggacac
1–29tRNA–Arg(cct)[96516,96589]
gcctctgtagctcaacggacagagcaacgcggtcctaacgcggtggctgg
aggttcgaatcctctcggaggcac
1–31tRNA–Gln(ttg)[97130,97205]
tggggtatggtggcaatctggcagtcccgccggactttgactccggaggt
gcaggttcgagtcctgctaccccatc
1–32tRNA–Arg(tct)[97209,97284]
gcccttgtagctcagtggacagagcggcgagcttctacctcgcgggccgg
gagttcgaatctctccaggggcacca
1–33tRNA–Gln(ctg)[125188,125262]
tgctcgttggtgtaactggcaacactacggactctgactccgtcatttta
ggttcgaatcctaagcgagcagcca
1–34tRNA–Asn(gtt)[125269,125344]
tggggtgtccgttaatcaggcaaacgagcggactgttaatccgcccctgc
aggttcgaatcctgccaccccagcca
>KX349298.1 Cyanophage S–RIM14 isolate LIS_02_1110, complete genome
1–1tRNA–Arg(tct)[164005,164077]
gggtcagtagctcagtggatagagcatcgcacttctaatgcgttggtcgg
gggttcaaatccctcctgacccg
>KX349299.1 Cyanophage S–RIM14 isolate LIS_22_0610, complete genome
1–1tRNA–Arg(tct)[164005,164077]
gggtcagtagctcagtggatagagcatcgcacttctaatgcgttggtcgg
gggttcaaatccctcctgacccg

>KX349300.1 Cyanophage S-RIM14 isolate Np_11_1211, complete genome
1-1tRNA-Arg(tct)[164012,164084]
gggtcagtagctcagtggatagagcatcgcacttctaatgcgttggtcgg
gggttcaaatccctcctgacccg
>KX349301.1 Cyanophage S-RIM14 isolate Np_45_0711, complete genome
1-1tRNA-Arg(tct)[164005,164077]
gggtcagtagctcagtggatagagcatcgcacttctaatgcgttggtcgg
gggttcaaatccctcctgacccg
>KX349302.1 Cyanophage S-RIM14 isolate RW_03_0110, complete genome
1-1tRNA-Arg(tct)[164005,164077]
gggtcagtagctcagtggatagagcatcgcacttctaatgcgttggtcgg
gggttcaaatccctcctgacccg
>KX349303.1 Cyanophage S-RIM14 isolate Sn_11_0110, complete genome
1-1tRNA-Arg(tct)[165497,165569]
gggtcagtagctcagtggatagagcatcgcacttctaatgcgttggtcgg
gggttcaaatccctcctgacccg
>KX349304.1 Cyanophage S-RIM14 isolate Sn_18_0910, complete genome
1-1tRNA-Arg(tct)[164004,164076]
gggtcagtagctcagtggatagagcatcgcacttctaatgcgttggtcgg
gggttcaaatccctcctgacccg
>KX349305.1 Cyanophage S-RIM14 isolate Sn_23_0910, complete genome
1-1tRNA-Arg(tct)[164006,164078]
gggtcagtagctcagtggatagagcatcgcacttctaatgcgttggtcgg
gggttcaaatccctcctgacccg
>KX349306.1 Cyanophage S-RIM14 isolate W1_23_0910, complete genome
1-1tRNA-Arg(tct)[164558,164630]
gggtcagtagctcagtggatagagcatcgcacttctaatgcgttggtcgg
gggttcaaatccctcctgacccg
>HM137666.1 Enterobacteria phage T4T, complete genome
1-1tRNA-Arg(tct)c[71151,71225]
gtcccgctggtgtaatggatagcatacgatccttctaagtttgcggtcct
ggttcgatcccagggcgggatacca
1-2tRNA-Met(cat)c[71938,72011]
ggccctgtagctcaatggttagcagcagtcccctcataagggaaaggtta
ccagttcaaatctggtctgggtca
1-3tRNA-Thr(tgt)c[72013,72088]
gctgatttagctcagtaggtagagcacctcacttgtaatgaggatgtcgg
cggttcgattccgtcaatcagcacca
1-4tRNA-Ser(tga)c[72095,72183]
tggaggcgtggcagagtggtttaatgcaccggtcttgaaaaccggcagtc
gctccggcgactcataggttcaaatcctatcgcctccgt
1-5tRNA-Pro(tgg)c[72184,72258]
ctccgtgtagctcagtttggtagagcgcctgatttgggatcaggaggtcc
aaggttcaaatccttgtatggagac

1-6tRNA-Gly(tcc)c[72269,72342]
gcggatatcgtataatggtattacctcagacttccaatctgatgatgtga
gttcgattctcattatccgctcca
1-7tRNA-Leu(taa)c[72348,72434]
gcgagaatggtcaaattggtaaaggcacagcacttaaaatgctgcggaat
gatttccttgtgggttcgagtcccacttctcgcacca
1-8tRNA-Gln(ttg)c[72435,72508]
tgggaattagccaagttggtaaggcatagcactttgactgctagatgcaa
aggttcgagtcctttattcccagc
>KJ477684.1 Enterobacteria phage T4 strain wild, complete genome
1-1tRNA-Arg(tct)c[71153,71227]
gtcccgctggtgtaatggatagcatacgatccttctaagtttgcggtcct
ggttcgatcccagggcgggatacca
1-2tRNA-Met(cat)c[71940,72013]
ggccctgtagctcaatggttagcagcagtcccctcataagggaaaggtta
ccagttcaaatctggtctgggtca
1-3tRNA-Thr(tgt)c[72015,72090]
gctgatttagctcagtaggtagagcacctcacttgtaatgaggatgtcgg
cggttcgattccgtcaatcagcacca
1-4tRNA-Ser(tga)c[72097,72185]
tggaggcgtggcagagtggtttaatgcaccggtcttgaaaaccggcagtc
gctccggcgactcataggttcaaatcctatcgcctccgt
1-5tRNA-Pro(tgg)c[72186,72260]
ctccgtgtagctcagtttggtagagcgcctgatttgggatcaggaggtcc
aaggttcaaatccttgtatggagac
1-6tRNA-Gly(tcc)c[72271,72344]
gcggatatcgtataatggtattacctcagacttccaatctgatgatgtga
gttcgattctcattatccgctcca
1-7tRNA-Leu(taa)c[72350,72436]
gcgagaatggtcaaattggtaaaggcacagcacttaaaatgctgcggaat
gatttccttgtgggttcgagtcccacttctcgcacca
1-8tRNA-Gln(ttg)c[72437,72510]
tgggaattagccaagttggtaaggcatagcactttgactgctagatgcaa
aggttcgagtcctttattcccagc
>KJ477685.1 Enterobacteria phage T4 strain 147, complete genome
1-1tRNA-Arg(tct)c[69046,69120]
gtcccgctggtgtaatggatagcatacgatccttctaagtttgcggtcct
ggttcgatcccagggcgggatacca
1-2tRNA-Met(cat)c[69833,69906]
ggccctgtagctcaatggttagcagcagtcccctcataagggaaaggtta
ccagttcaaatctggtctgggtca
1-3tRNA-Thr(tgt)c[69908,69983]
gctgatttagctcagtaggtagagcacctcacttgtaatgaggatgtcgg
cggttcgattccgtcaatcagcacca

1-4tRNA-Ser(tga)c[69990,70078]
tggaggcgtggcagagtggtttaatgcaccggtcttgaaaaccggcagtc
gctccggcgactcataggttcaaatcctatcgcctccgt
1-5tRNA-Pro(tgg)c[70079,70153]
ctccgtgtagctcagtttggtagagcgcctgatttgggatcaggaggtcc
aaggttcaaatccttgtatggagac
1-6tRNA-Gly(tcc)c[70164,70237]
gcggatatcgtataatggtattacctcagacttccaatctgatgatgtga
gttcgattctcattatccgctcca
1-7tRNA-Leu(taa)c[70243,70329]
gcgagaatggtcaaattggtaaaggcacagcacttaaaatgctgcggaat
gatttccttgtgggttcgagtcccacttctcgcacca
1-8tRNA-Gln(ttg)c[70330,70403]
tgggaattagccaagttggtaaggcatagcactttgactgctagatgcaa
aggttcgagtcctttattcccagc
>KJ477686.1 Enterobacteria phage T4 strain GT7, complete genome
1-1tRNA-Arg(tct)c[71151,71225]
gtcccgctggtgtaatggatagcatacgatccttctaagtttgcggtcct
ggttcgatcccagggcgggatacca
1-2tRNA-Met(cat)c[71938,72011]
ggccctgtagctcaatggttagcagcagtcccctcataagggaaaggtta
ccagttcaaatctggtctgggtca
1-3tRNA-Thr(tgt)c[72013,72088]
gctgatttagctcagtaggtagagcacctcacttgtaatgaggatgtcgg
cggttcgattccgtcaatcagcacca
1-4tRNA-Ser(tga)c[72095,72183]
tggaggcgtggcagagtggtttaatgcaccggtcttgaaaaccggcagtc
gctccggcgactcataggttcaaatcctatcgcctccgt
1-5tRNA-Pro(tgg)c[72184,72258]
ctccgtgtagctcagtttggtagagcgcctgatttgggatcaggaggtcc
aaggttcaaatccttgtatggagac
1-6tRNA-Gly(tcc)c[72269,72342]
gcggatatcgtataatggtattacctcagacttccaatctgatgatgtga
gttcgattctcattatccgctcca
1-7tRNA-Leu(taa)c[72348,72434]
gcgagaatggtcaaattggtaaaggcacagcacttaaaatgctgcggaat
gatttccttgtgggttcgagtcccacttctcgcacca
1-8tRNA-Gln(ttg)c[72435,72508]
tgggaattagccaagttggtaaggcatagcactttgactgctagatgcaa
aggttcgagtcctttattcccagc
>KM607002.1 Enterobacteria phage RB55, complete genome
1-1tRNA-Arg(tct)c[71116,71190]
gtcccgctggtgtaatggatagcatacgatccttctaagtttgcggtcct
ggttcgatcccagggcgggatacca

1-2tRNA-Met(cat)c[71903,71976]
ggccctgtagctcaatggttagcagcagtcccctcataagggaaaggtta
ccagttcaaatctggtctgggtca
1-3tRNA-Thr(tgt)c[71978,72053]
gctgatttagctcagtaggtagagcacctcacttgtaatgaggatgtcgg
cggttcgattccgtcaatcagcacca
1-4tRNA-Ser(tga)c[72060,72148]
tggaggcgtggcagagtggtttaatgcaccggtcttgaaaaccggcagtc
gctccggcgactcataggttcaaatcctatcgcctccgt
1-5tRNA-Pro(tgg)c[72149,72223]
ctccgtgtagctcagtttggtagagcgcctgatttgggatcaggaggtcc
aaggttcaaatccttgtatggagac
1-6tRNA-Gly(tcc)c[72234,72307]
gcggatatcgtataatggtattacctcagacttccaatctgatgatgtga
gttcgattctcattatccgctcca
1-7tRNA-Leu(taa)c[72313,72399]
gcgagaatggtcaaattggtaaaggcacagcacttaaaatgctgcggaat
gatttccttgtgggttcgagtcccacttctcgcacca
1-8tRNA-Gln(ttg)c[72400,72473]
tgggaattagccaagttggtaaggcatagcactttgactgctagatgcaa
aggttcgagtcctttattcccagc
>KM607003.1 Enterobacteria phage RB59, complete genome
1-1tRNA-Arg(tct)c[71129,71203]
gtcccgctggtgtaatggatagcatacgatccttctaagtttgcggtcct
ggttcgatcccagggcgggatacca
1-2tRNA-Met(cat)c[71916,71989]
ggccctgtagctcaatggttagcagcagtcccctcataagggaaaggtta
ccagttcaaatctggtctgggtca
1-3tRNA-Thr(tgt)c[71991,72066]
gctgatttagctcagtaggtagagcacctcacttgtaatgaggatgtcgg
cggttcgattccgtcaatcagcacca
1-4tRNA-Ser(tga)c[72073,72161]
tggaggcgtggcagagtggtttaatgcaccggtcttgaaaaccggcagtc
gctccggcgactcataggttcaaatcctatcgcctccgt
1-5tRNA-Pro(tgg)c[72162,72236]
ctccgtgtagctcagtttggtagagcgcctgatttgggatcaggaggtcc
aaggttcaaatccttgtatggagac
1-6tRNA-Gly(tcc)c[72247,72320]
gcggatatcgtataatggtattacctcagacttccaatctgatgatgtga
gttcgattctcattatccgctcca
1-7tRNA-Leu(taa)c[72326,72412]
gcgagaatggtcaaattggtaaaggcacagcacttaaaatgctgcggaat
gatttccttgtgggttcgagtcccacttctcgcacca
1-8tRNA-Gln(ttg)c[72413,72486]

tgggaattagccaagttggtaaggcatagcactttgactgctagatgcaa
aggttcgagtcctttattcccagc
>MF360957.1 Bacillus virus PBS1, complete genome
1-1tRNA-Asn(gtt)[119223,119298]
ttgggatatagccaagtggtaaggcaacatgctgttaacatgtctatttc
gtaggttcgaatcctactatcccagt
>KT321476.1 Mycobacterium phage Zeenon, complete genome
1-1tRNA-Ser(gct)[31539,31622]
ggagggtgagcatctggtgatgcaggggtcctgctaaggccctacggatt
cacacccgtgagtttcgattactcctccctccgc
1-2tRNA-Leu(cag)[31719,31795]
gccctgctgagcaaactggcaaagctgccgcattcagagtgcgggtcatt
tccgggttcgactcccgggcagggtac
1-3tRNA-Leu(gag)[31915,31989]
gtctctgtaggcaaatcggaaaagccgccatcttgagggggtggtgcgtg
cgggttcgactcccgccagagacac
1-4tRNA-Leu(caa)[31990,32063]
gccgtggtaggccatctggcgagccgccgagttcaagtttcggtgtttgc
gggttcgaatcccgcccacggtac
1-5tRNA-SeC(tca)[68245,68341]
attctggcactggtgggcgagcccaccggcggcagcttcaagctgtcgct
ggccggagaaccgaccggaaacatcccgttcaacgcgacccagggcc
1-6tRNA-Pro(tgg)[91594,91668]
cggggtgtagttcagtttggaagagcgcttggtttgggaccaagttgtcg
caggttcgaatcctgtcaccccgac
1-7tRNA-Trp(cca)[91683,91753]
gggtctgtgcacagggtgcccgacggtctccaaagccgaaggcgggggtt
cgattccctccaggcctgcca
1-8tRNA-Pyl(cta)[92890,92963]
tgcgagatcgtgcacggcgcactaggagcttctaaccctccgactcgcgg
gttcgactcccgcatctcgcaccc
1-9tRNA-Met(cat)[93111,93185]
agcggtgtagagcagctaggtagctcgccgggctcatgacccggaggacg
cgtgttcgattcacgccaccgccac
1-10tRNA-Cys(gca)[93400,93471]
gcgcctttggcggaatggctacgtgctcggctgcaacccgagttatcccg
gttcgactccgggagggcgctc
1-11tRNA-Glu(ctc)[93539,93610]
gctcccatggggtagtggtaaccctcctggttctcagccaggcgtcccga
gttcgatcctcggtgggagtgc
1-12tRNA-His(gtg)[93612,93685]
gtggccgtagttcagccggtagaacgctgggttgtgatcccagtcgtcga
gggttcgagtccctccggtcaccc
1-13tRNA-Ala(tgc)[93850,93923]

gggcctatagctcatctggtagagcgcctgccttgcaagcaggaggcggc
aggttcaagtcctgttaggtccac
1-14tRNA-Phe(gaa)[94113,94185]
gccgtcatagctcagttggtagagcactggcctgaaaacccagtggccga
ggttcgagtcctcgtgtcggcac
1-15tRNA-Val(cac)[94191,94264]
gtccgtttagctcagctggaagagcgctcggtccacacccgagaggccgc
aggttcgatccctgcaatggacac
1-16tRNA-Lys(ctt)[94383,94455]
gccttcgtagctcagtggtagagctgtcgcctcttaagcgataggtcgtt
ggttcaaatccagccgggggcac
1-17tRNA-Glu(ttc)[94459,94534]
ggtcgggtggtctgttggcaggccggtcgggttttcaccccggcgtcatc
gcgggttcgattcccgtcccgactgc
1-18tRNA-Gly(tcc)[94664,94736]
gcgggtgtggccgaatggctcaggcaccagacttccattctggctacgca
ggttcgattcctgtcatccgctc
1-19tRNA-Thr(cgt)[94796,94870]
gctgctgtagctcacctggcagagcgtcggcgtcgtatcccgaaggcatc
cggttcgagtccggacagcagcccc
1-20tRNA-Thr(tgt)[94871,94943]
gcctctgtggtccagcggcacggacatccgccttgtaagcggaggacccc
cgttcgatccggggtagaggctc
1-21tRNA-Thr(ggt)[95351,95423]
gctgggttagctcagtggtagagcgttcctctggtatgggaaaggcccgg
ggttcaatcccccgactcagctc
1-22tRNA-Gly(gcc)[96746,96819]
gcgaaggtagctcagctggcagagcgccaccttgccaaggtggaggtcgc
gggatcgtaacccgttcttcgctc
1-23tRNA-Asp(gtc)[96823,96895]
ggccctgtagctcagaggaagagcgccggtctgtcgaatcggaggtcgcg
gtatcgtaatccgtcagggtcgc
1-24tRNA-Met(cat)[96955,97027]
gcctcactagctcattggtagagccgctcgctcataacgtgcaggtacct
ggttcgattccagggtgaggtac
1-25tRNA-Ile(gat)[97033,97107]
gcctgttagcggactggtcgtccgatccaagctgataacttggcgtaagc
ggtgttcgattcaccgagcaggtac
1-26tRNA-Arg(acg)[97201,97273]
gcctctatggtccaacggatatgacgccggtctacggaaccggagatgcg
tgttcgattcgcgctaggggcac
1-27tRNA-Val(gac)[97316,97388]
gtccgtgtagctcaggggtagagcgcctgctcgacacgcaggaggaccga
ggttcgaaacctcgcatggacac

1−28tRNA−Arg(cct)[97562,97635]
gcctctgtagctcaacggacagagcaacgcggtcctaacgcggtggctgg
aggttcgaatcctctcggaggcac
1−30tRNA−Gln(ttg)[98176,98251]
tggggtatggtggcaatctggcagtcccgccggactttgactccggaggt
gcaggttcgagtcctgctaccccatc
1−31tRNA−Arg(tct)[98255,98328]
gcccttgtagctcagtggacagagcggcgagcttctacctcgcgggccgg
gagttcgaatctctccaggggcac
1−32tRNA−Lys(ttt)[98396,98472]
gggccggtatcttagtctggtcaaagaagtggacttttaatccgcgcgcc
gtgggttcgaatcccacccggcccacc
1−33tRNA−Gln(ctg)[126474,126548]
tgctcgttggtgtaactggcaacactacggactctgactccgtcattta
ggttcgaatcctaagcgagcagcca
1−34tRNA−Asn(gtt)[126555,126630]
tggggtgtccgttaatcaggcaaacgagcggactgttaatccgcccctgc
aggttcgaatcctgccaccccagcca
>KU726251.1 Enterobacteria phage SEGD1, complete genome
1−1tRNA−Trp(cca)[1573,1648]
gggggtatggcgcaattggtagcgcaccggtctccaaaaccgaaggttcg
aggttcgaaacctcgtgcccccgcca
>KM209228.1 Dickeya phage phiD3, complete genome
1−1tRNA−Met(cat)[133174,133250]
gatggtgtagttcagttggttagaacgtgcgactcataatcgctttgtca
ctggttcaagtcctgccgccatcgcca
>JN797798.1 Bacillus phage BCU4, complete genome
1−1tRNA−Met(cat)c[148023,148095]
agggttatagctcagtggtagtagcgtgggtctcataagcccaaggtcgt
aggttcaactcctactagcccta
1−2tRNA−Leu(tag)c[148239,148313]
tgccgaagtaatccaatggcagagatagcggttttagaaaccgtacagtg
tgggttcgagtcccaccttcggtat
1−3tRNA−Leu(taa)c[148318,148405]
ggcggagtgttggaattggtagacatatggcacttaaaatgctatgtccg
tacgggcgtggtgggttcgagtccccccctccgctacca
1−4tRNA−Ile(gat)c[148421,148497]
gctagggtagctcagtcaggtagagcagagtcttgataaggctttggtcg
taggttcgaatcctacccctagtacca
1−5tRNA−Tyr(gta)c[148499,148585]
gggcgagtatgcaaattggtgaagcaagcggtctgtaaaaccgtgacgta
agatacattgcaggttcgaatcctgcctttcccacca
1−6tRNA−Phe(gaa)c[148724,148799]
ggacggatagctcagttggtagagcagagggctgaaaatcctcgtgtcgt

aggttcgattcctactccgtccacca
1−7tRNA−Pro(tgg)c[148880,148955]
tgtggatatagctcagattggtagagcgcttgctttgggagcaagaagtc
gtaggttcaagtcctgctatccgcat
1−8tRNA−Gln(ttg)c[149269,149341]
tttcggagtagccaagtggtaaggcaatagactttgactctatgatcggt
ggttcgagaccatcctccgaagt
1−9tRNA−Ser(tga)c[149419,149510]
ggaaggatactcaagtggataaaagagggcggtcttgaaaaccgctaggc
gtgtaaaagcgtgcgtgggttcgaatcccactccttccgcca
1−10tRNA−Arg(tct)c[149515,149591]
tatcccattagccaagttggatcaaggcaacgggcttctatcccgttaat
cgtgggttcgagtcctacatgggatgt
1−11tRNA−Ile(tat)c[149671,149745]
gcccctttagccaagcggttcaaggcagtgggattatgtcctgcgaatcg
gaagttcgaatcttccaaggggagc
1−12tRNA−Glu(ttc)c[149749,149822]
ggggtattggtgaagtggtcaaacacatccgactttctatcggagatacg
agggttcgaatcccttataccctt
1−13tRNA−Thr(tgt)c[149952,150025]
gccttcttagctcagttgatagagcgattgacttgtaatcaataggtcga
gggttcgaatccttcagtcggcac
1−14tRNA−Asp(gtc)c[150145,150218]
tggggatatagtgtagcggtgaacacacttgactgtctatcaagtagcac
gggttcgagtcccgttatcctcgt
1−16tRNA−Gly(tcc)c[150870,150942]
ggggcattagtatatcggttcattattcctggcttccaaccaggggaggt
cggttcgactccgacatgcccct
1−17tRNA−Ser(gct)c[150948,151038]
ggaaggttgtcagagcggtttattgtgcctgtttgctaaataggtgtacg
tccttaacgtaccacaggttcgaatcctgtaccttccgcca
1−18tRNA−Asn(gtt)c[151553,151628]
gtgcctgtagctcagtcggtagagcaaaaccctgttaaggttgaggtcgt
aggttcgagccctaccaggtacgcca
1−19tRNA−Cys(gca)c[151648,151721]
gaaggtgtaccgaagcggcttaacggtctaggttgcaaccctagttttcg
tgggttcgaatcccaccactttct
>KT365398.1 Mycobacterium phage DTDevon, complete genome
1−1tRNA−Ser(gct)[31626,31709]
ggagggtgagcatcaggtgatgcagcgagattgctaatcccgtacggtaa
ccaccccgtgaggttcgaatcctcctccctccgc
1−2tRNA−Leu(cag)[31902,31977]
gctcccgtagcccaattggcaggaggcaccagattcaggatctgggcagt
gtgagttcgaatctcaccgggagtac

1–3tRNA–Leu(gag)[32097,32171]
gtctctgtaggcaaatcggaaaagccgccatcttgagggggtggtgcgtg
cgggttcgactcccgccagagacac
1–4tRNA–Leu(caa)[32172,32245]
gccgtggtaggccatctggcgagccgccgagttcaagtttcggtgtttgc
gggttcgaatcccgcccacggtac
1–5tRNA–SeC(tca)[68548,68644]
attctggcactggtgggcgagcccaccggcggcagcttcaagctgtcgct
ggccggagaatcgaccggaaacatcccgttcaacgcgacccagggcc
1–6tRNA–Pro(tgg)[93205,93277]
cggggtgtagttcagtggaagagcgcttggtttgggaccaagatgtcgca
ggttcgaatcctgtcaccccgac
1–7tRNA–Trp(cca)[93294,93369]
tggggtgaagccgatctggaaggcagcggtctccaaagccgtctcatagc
gggttcgaatcccgtcacccctgcca
1–8tRNA–Tyr(gta)[93371,93457]
gccgcacatgcccaactggtgtttgggagcaggctgtaaccctgtggcct
tcgggacggtgaggttcgattcctcagtgcggtacca
1–9tRNA–Pyl(cta)[94566,94638]
gcaccatttgctcaatggcagagcggcggcttctaaaaccgtgagtgccg
gttcgactccggcatggtgcacc
1–10tRNA–Met(cat)[94788,94862]
agcggtgtagagcagctaggtagctcgccgggctcataacccggaggacg
cgtgttcgaatcacgccaccgccac
1–11tRNA–Cys(gca)[94989,95060]
gcgcctttggcggaatggctacgtgctcggctgcaacccgagttatcccg
gttcgactccgggagggcgctc
1–12tRNA–Glu(ctc)[95065,95139]
ggtccgttggagtagatggatatctcgccaccctctcaaggtggagatca
cgggttcaagtcccgtacggactgc
1–13tRNA–His(gtg)[95141,95214]
gtggccgtagttcagccggtagaacgctgggttgtgatcccagtcgtcga
gggttcgagtccctccggtcaccc
1–14tRNA–Ala(tgc)[95376,95449]
gggcctatagctcatctggtagagcgcctgccttgcaagcaggaggcggc
aggttcaagtcctgttaggtccac
1–15tRNA–Phe(gaa)[95639,95711]
gccgtcatagctcagttggtagagcactggcctgaaaacccagtggccga
ggttcgattcctcgtgtcggcac
1–16tRNA–Val(cac)[95717,95790]
gtccgtttagctcagctggaagagcgctcggtccacacccgagaggccgc
aggttcgatccctgcaatggacac
1–17tRNA–Lys(ctt)[95909,95981]
gccttcgtagctcagtggtagagctgtcgcctcttaagcgataggtcgtt

ggttcgaatccagccgggggcac
1-18tRNA-Glu(ttc)[95986,96062]
ggtcgggtcggtctgctggtatggccagtcggattttcactccggacatt
cgcgggttcaattcccgtcccgatcgc
1-19tRNA-Gly(tcc)[96142,96214]
gcgggtgtggccgaatggctcaggcaccagatttccactctggctacgca
ggttcgattcctgtcatccgctc
1-20tRNA-Thr(cgt)[96274,96348]
gctgctgtagctcacctggcagagcgtcggcgtcgtatcccgaaggcatc
cggttcgagtccggacagcagcccc
1-21tRNA-Thr(tgt)[96349,96421]
gcctctgtggtccagcggcacggacatccgccttgtaagcggaggacccc
cgttcgatccggggtagaggctc
1-22tRNA-Thr(ggt)[96829,96901]
gctgggttagctcagtggtagagcgttcctctggtatgggaaaggcccgg
ggttcaatcccccgattcagctc
1-23tRNA-Gly(gcc)[98224,98297]
gcgaaggtagctcagctggtagagcgccaccttgccaaggtggaggtcgc
gggatcgtaacccgttcttcgctc
1-24tRNA-Asp(gtc)[98301,98373]
ggccctgtagctcagaggaagagcgccggtctgtcgaatcggaggtcgcg
gtatcgtaatccgtcagggtcgc
1-25tRNA-Met(cat)[98433,98505]
gcctcactagctcattggtagagccgctcgctcataacgtgcaggtacct
ggttcgattccagggtgaggtac
1-26tRNA-Ile(gat)[98511,98585]
gcctgttagcggactggtcgtccgatccaagctgataacttggcgtaagc
ggtgttcgattcaccgagcaggtac
1-27tRNA-Arg(acg)[98679,98751]
gcctctatggtccaacggatatgacgccggtctacggaaccggagatgcg
tgttcgattcgcgctaggggcac
1-28tRNA-Val(gac)[98794,98866]
gtccgtgtagctcaggggtagagcgcctgctcgacacgcaggaggaccga
ggttcgaaacctcgcatggacac
1-29tRNA-Arg(cct)[99040,99113]
gcctctgtagctcaacggacagagcaacgcggtcctaacgcggtggctgg
aggttcgaatcctctcggaggcac
1-31tRNA-Gln(ttg)[99654,99729]
tggggtatggtggcaatctggcagtcccgccggactttgactccggaggt
gcaggttcgagtcctgctaccccatc
1-32tRNA-Arg(tct)[99733,99808]
gcccttgtagctcagtggacagagcggcgagcttctacctcgcgggccgg
gagttcgaatctctccaggggcacca
1-33tRNA-Gln(ctg)[127929,128003]

tgctcgttggtgtaactggcaacactacggactctgactccgtcatttta
ggttcgaatcctaagcgagcagcca
1–34tRNA–Asn(gtt)[128010,128085]
tggggtgtccgttaatcaggcaaacgagcggactgttaatccgcccctgc
aggttcgaatcctgccaccccagcca
>KJ019158.1 Synechococcus phage ACG–2014a isolate Syn7803C33, complete genome
1–1tRNA–Val(tac)[13064,13135]
gggcgaataactcagcggtagagtgcctcctttacacggagattgtcggg
ggttcgatcccctcttcgccca
1–2tRNA–Leu(taa)[18054,18140]
tgggagtgtggcggaatcggtagacgcaccagacttaaaatctgttgaga
attaatctcgtgggggttcaagtccccccttctcccat
1–3tRNA–Thr(tgt)[18764,18836]
gcctccgtagctcagtggtagagcagggcttttgtaaagctcaggtcgca
agttcaaatcttgtcagaggctc
1–4tRNA–Asn(gtt)[18841,18913]
tcctctatagctcagttggtagagcaggtgactgttaatcaccctgtccc
tggttcgagtccaggtggaggag
1–5tRNA–Arg(tct)[154574,154649]
tgggtcagtagctcagatggatagagcaactgccttctaagcagtcggcc
acaggttcgagtcctgtctgacccgt
>KF302034.1 UNVERIFIED: Pseudoalteromonas phage HM1, complete genome
1–1tRNA–Lys(ttt)c[71990,72059]
gccttcgtagctaactggtaagcgacttacttttaatgaggatatgttgg
ttcaattccaaccgaaggca
1–2tRNA–Arg(tct)c[72061,72137]
gcaagagtagaacaattggatagttcatctcccttctaaggagacggttg
taagttcgaatcttacctcttgtgcca
>KR233165.1 Escherichia phage PEC04, complete genome
1–1tRNA–Ser(tga)[6,95]
ggaggcgtggcagagtggtttaatgcaccggtcttgaaaaccggcagtcg
ctccggcgactcataggttcgaatcctatcgcctccgcca
1–2tRNA–Thr(tgt)[101,176]
gctgatttagctcagtaggtagagcaactcacttgtaatgagaaggtcgg
cggttcgattccgtcaatcagcacca
1–3tRNA–Met(cat)[178,252]
ggccctgtagctggaaggttcaagcaagcgactcataatcgccagatggt
ggttcaattccacccagggccacca
1–4tRNA–Tyr(gta)[262,348]
ggggagttattccgtagaggtagcggtgtggactgtaaatccattgtcat
tgcgactcgggtggttcgactccatcactccccacca
1–5tRNA–Asn(gtt)[353,427]
ggatgtgtagctcaatggcagagcgatcgcctgttaagcgattggttata
ggttcgaatcctatcacgtccgcca

1−6tRNA−Arg(tct)[432,507]
cgaggcatagctcagaaggaagagcaaggaccttctaagtcctaggtcgt
aggttcgatccctactgcctcgacca
1−7tRNA−Gln(ttg)[166657,166730]
tgggaattagccaagttggtaaggcactggattttgattccaggatgcaa
aggttcaagtcctttattcccagc
1−8tRNA−Leu(taa)[166731,166817]
gcgagaatggtcaaattggtaaaggcacagcacttaaaatgctgcggaat
gatttccttgtgggttcgagtcccacttctcgcacca
1−9tRNA−Gly(tcc)[166823,166896]
gcggatatcgtataatggcattacctcagacttccaatctgatgatgtga
gttcgattctcattatccgctcca
>KJ010548.1 Bacillus phage Bp8p−T, complete genome
1−1tRNA−Met(cat)c[106844,106915]
gggtctttagcttaaaggtaaagcaatcagctcataactgataggatagg
agttcgagtctcttaggaccca
1−2tRNA−Tyr(gta)c[109354,109437]
gggatagcgctaacgttggagagttaggcgagactgtaaatctcgtgtcg
gatcactgagtgggttcgaatcccacctgtccca
1−3tRNA−His(gtg)c[109551,109621]
gagaatgtagcttaacggtaaagcgctgggacgtgactccagttatccaa
gttcgagtcttggtattctca
1−4tRNA−Gly(tcc)c[109632,109705]
gtggtcttagtttaacggtagaattgctggcttccaaccagcgggtaagg
gttcgactcccttagaccatacca
1−5tRNA−Met(cat)c[110834,110907]
tacgatgctgaggggtcggtacccgacgaggtctcataagccttgtttag
caagttcaactcttgccttagcaa
1−6tRNA−Phe(gaa)c[110981,111055]
tggactggtagctcagtcggtagagcagggggctgaaaatccctgtgtcg
gaggttcgaatccttctcagtccat
1−7tRNA−Asn(gtt)c[111435,111508]
ttgtcgtataactcaatggtagagtgcttgactgttaatcaagaagttgt
aggttcgagtcctactacgacagt
>KJ094029.1 Listeria phage LP−064, complete genome
1−1tRNA−Met(cat)c[35285,35358]
ttgtcccgtagctagaaggtcgagcaaggagctcataactcctcggtttg
ggttcgattcccaacggggcaatc
1−2tRNA−Pro(tgg)c[36251,36325]
cagggtgtagctcagtttggttagagtacccgctttggagacgggaagtc
gtaggttcgagtcctaccaccttga
1−3tRNA−Arg(tct)c[37443,37513]
gtccttatggtgtagtggatgcacaagggatttctactcccttagcgcgg
gttcgaatcctgctgaggact

1−4tRNA−Gly(tcc)c[37771,37841]
gcgggtatagtataagggtagtacccaaggtttccaaccatgtagtgggg
gttcgaatccccctacccgct
1−5tRNA−Asn(gtt)c[37910,37983]
gtgtccttaactcagaggtcagagtgccgtcctgttaagtcggaagtcgc
tggttcaaatccagcaggatacgc
1−6tRNA−Ser(tga)c[38605,38696]
ggaaggttggtagagcttggtaatacgctagtcttgaaaactagtttgcc
cttggaatacagggtacaagggttcaaatcccttaccttcct
1−7tRNA−Phe(gaa)c[38709,38780]
gtagtcctagctgagatggattagcgcttgcttgaaaagcaagagaggca
ggttcgatacctgcggactcca
1−8tRNA−Lys(ttt)c[38786,38857]
ggagttatggtgaaatggctatcactgcgggtttttaccccgttattcta
ggttcgaatcctagtggctcca
1−9tRNA−Trp(cca)c[38982,39055]
taggggtatagtttatctggtaaaatattggtttccaactccaatgaggt
gggttcaagtcctactatccctgt
1−10tRNA−Gln(ttg)c[39057,39129]
tggtctgtagccaagcggtaaggcaacggattttgattccgtgatacgtt
ggttcgaatccaactagaccagc
1−11tRNA−Thr(tgt)c[39148,39219]
gcttgtatagttcaattggtagaacagtggttttgtaagcctcagacgtg
ggttcaagtcctactacaagca
1−12tRNA−Tyr(gta)c[39302,39383]
gtgccattcgcatagaggcaattgcaggggactgtaactcccctcccttc
ggggttccaaggttcgagtccttgatggcgca
1−13tRNA−Leu(tag)c[39744,39828]
tgccgagatggtggaactggtatacacggtagacttagaatctgctgtcc
taaggatatgtgggttcgaatcccactctcggtat
1−14tRNA−Asp(gtc)c[40294,40366]
gtgcgtatgatataatggctattatactcggctgtctatcgagaaatagg
ggttcgattccccttacgtgcgc
1−15tRNA−Ile(gat)c[40470,40542]
gccagcatagcttagtaggcaaagcaaccgaccgataatcggtagtcctt
ggttcaattccaagtgttggtac
1−16tRNA−Ser(gct)c[40719,40805]
ggagagttgtcagagaggcttaatgatacgggttgctaactcgttgtact
agtaatagtaccaagggttcgaatcccttactctcct
1−17tRNA−Cys(gca)c[40884,40954]
gcgggtataaccaactggaaaggtagtagactgcaaatctacgtatatgg
gttcaattcccattacccgct
>KJ094031.2 Listeria phage LP−124, complete genome
1−1tRNA−Met(cat)c[129836,129909]

ttgtcccgtagctagaaggtcgagcaaggagctcataactcctcggtttg
ggttcgattcccaacggggcaatc
1-2tRNA-Pro(tgg)c[130802,130876]
cagggtgtagctcagtttggttagagtacccgctttggagacgggaagtc
gtaggttcgagtcctaccaccttga
1-3tRNA-Arg(tct)c[131994,132064]
gtccttatggtgtagtggatgcacaagggatttctactcccttagcgcgg
gttcgaatcctgctgaggact
1-4tRNA-Gly(tcc)c[132322,132392]
gcgggtatagtataagggtagtacccaaggtttccaaccatgtagtgggg
gttcgaatccccctacccgct
1-5tRNA-Asn(gtt)c[132461,132534]
gtgtccttaactcagaggtcagagtgccgtcctgttaagtcggaagtcgc
tggttcaaatccagcaggatacgc
1-6tRNA-Ser(tga)c[133156,133247]
ggaaggttggtagagcttggtaatacgctagtcttgaaaactagtttgcc
cttggaatacagggtacaagggttcaaatcccttaccttcct
1-7tRNA-Phe(gaa)c[133260,133331]
gtagtcctagctgagatggattagcgcttgcttgaaaagcaagagaggca
ggttcgatacctgcggactcca
1-8tRNA-Lys(ttt)c[133337,133408]
ggagttatggtgaaatggctatcactgcgggtttttaccccgttattcta
ggttcgaatcctagtggctcca
1-9tRNA-Trp(cca)c[133533,133606]
taggggtatagtttatctggtaaaatattggtttccaactccaatgaggt
gggttcaagtcctactatccctgt
1-10tRNA-Gln(ttg)c[133608,133680]
tggtctgtagccaagcggtaaggcaacggattttgattccgtgatacgtt
ggttcgaatccaactagaccagc
1-11tRNA-Thr(tgt)c[133699,133770]
gcttgtatagttcaattggtagaacagtggttttgtaagcctcagacgtg
ggttcaagtcctactacaagca
1-12tRNA-Tyr(gta)c[133853,133934]
gtgccattcgcatagaggcaattgcaggggactgtaactcccctcccttc
ggggttccaaggttcgagtccttgatggcgca
1-13tRNA-Leu(tag)c[134295,134379]
tgccgagatggtggaactggtatacacggtagacttagaatctgctgtcc
taaggatatgtgggttcgaatcccactctcggtat
1-14tRNA-Asp(gtc)c[134845,134917]
gtgcgtatgatataatggctattatactcggctgtctatcgagaaatagg
ggttcgattccccttacgtgcgc
1-15tRNA-Ile(gat)c[135021,135093]
gccagcatagcttagtaggcaaagcaaccgaccgataatcggtagtcctt
ggttcaattccaagtgttggtac

1–16tRNA–Ser(gct)c[135270,135356]
ggagagttgtcagagaggcttaatgatacgggttgctaactcgttgtact
agtaatagtaccaagggttcgaatcccttactctcct
1–17tRNA–Cys(gca)c[135435,135505]
gcgggtataaccaactggaaaggtagtagactgcaaatctacgtatatgg
gttcaattcccattacccgct
>KU737346.1 Bacillus phage Vinny, complete genome
1–1tRNA–Asn(gtt)[32383,32457]
tgtgcttgtagctcagtcggtagagctggtggctgttaaccactgtgtcg
taggttcgagccctacctagcacgt
1–2tRNA–Gln(ttg)[32463,32535]
tgcctcttagccaagcggtaaggcacgggactttgactctcggacgcgct
agttcgaatctagcagaggcatc
1–3tRNA–Trp(cca)[32704,32777]
gggagtttagtttagtggtaaaacctcggtctccaaaaccgaagtcatat
gttcgattcgtatagctcctgcca
1–4tRNA–Ser(tga)[32779,32866]
ggagggttggcagagtggattattgcagcggtcttgaaaaccgccgaggg
taaaacctccgtgagttcaaatctcacaccctcctcca
1–5tRNA–Leu(tag)[33586,33663]
tgccgaagtaatccaatcaggtagagatagcggttttagaaaccgtccag
tgtgggttcgacccccctccttcggtatc
1–6tRNA–Tyr(gta)[33670,33753]
tggcagaatagtcaagcggcaacgacaggtgactgtaaatcacccctcat
tcgagttcgtaggttcgagtcctgcttctgctat
1–7tRNA–Ser(gct)[34666,34752]
ggaggggtactcaagcggcgaagagggggacttgctaagtctctagtacc
ttaacaggtagcgagggttcgaatcccttcccctcct
>KJ019089.1 Synechococcus phage ACG–2014j isolate Syn7803US23, complete genome
1–1tRNA–Met(cat)[17105,17179]
tgcttccttagcaatctggtgaatgcagcaaactcataatttgcctaagg
agagttcgatcctctcaggaagcat
1–2tRNA–Leu(taa)[17181,17267]
tgcgagtatggcggaatcggtagacgcaccagacttaaaatctgttgagc
attatgctcgtgggagttcaagtctccctactcgcat
1–3tRNA–Thr(tgt)[18085,18158]
gccaaactagctcagctggtagagcagggcttttgtaaagctcaggtcgc
aggttcaagtcctgtgtttggctc
1–4tRNA–Asn(gtt)[18162,18235]
ttcctctttagctcagcggtagagcggttgactgttaatcaatttgtccc
tggttcgatcccaggaaggggagt
1–5tRNA–Arg(tct)[158075,158150]
gtctcagtagctcagatggatagagcaattcacttctaatgaattggtcg
gggttcgagtccctcctgagacgcc

1−6tRNA−Leu(tag)[158153,158235]
gtcggtatggcggaattggtagacgcgccaggtttaggttctggtgtctt
atgacgtggaggttcaagtcctcttaccgacac
1−7tRNA−Val(tac)[158238,158309]
gctcgaatagctcagaggtagagcacctcctttacacggagattgtcggg
ggttcgatcccctcttcgagca
1−8tRNA−Gly(tcc)[160795,160867]
gcgggtgtagctcagatggtagagcgtcagttttccaaactgaatgtcgt
cggttcaagtccgatctcccgct
>KP869109.1 Escherichia coli O157 typing phage 11, complete genome
1−1tRNA−Pro(tgg)[24016,24092]
ctctgtttagctcagcttggtagagcgttccgtttggggcggtaaggccg
gaggttcaagtcctccaacagagacca
1−2tRNA−Glu(ttc)[24100,24177]
gttccagtagacaaaatggtaaagtcaccactctttcaaagtggatatct
gagggttcaaatcccttctggaacgcca
1−3tRNA−Met(cat)[24268,24344]
tgcgggtatagagaaagggcgtctcacatgtctcattagcatggtatcgg
caggttcgactcctgcacccgcctcca
1−4tRNA−Asn(gtt)[24429,24505]
ggttaggaagcacataaggtatgtgcggtcgcctgttaagcgaatggcac
agggttcgaatccctgactaaccgcca
1−5tRNA−Tyr(gta)[24515,24602]
gtgtcgttatcccgtagatggtagcggtggggactgtaaatcccttgtca
ttgagactcggtaggttcgactcctacacggcacacca
1−6tRNA−Asp(gtc)[24608,24685]
ggttatgtagtttaatctggttaaaatactcccctgtcacgggagatgat
gagggttcgaatcccttcgtaaccgcca
1−7tRNA−Lys(ttt)[25115,25190]
ggaagtgtagcagaatggtgatgcggcagacttttaatctgacaggcgat
gggttcgaatccctccacttctacca
1−8tRNA−Met(cat)[25195,25271]
ggttcagtcgcagataaggtaatgcaagggtctcataagccctatgaatg
tgggttcgattcccatctgaacctcca
1−9tRNA−Ile(gat)[25273,25348]
gctggtatagttaagaaggttataacactcccctgataagggaacatcgg
tggttcgattccacctaccagtacca
1−10tRNA−Trp(cca)[25828,25898]
aggggtgagctatggcagcaatagagtctccaaaattcttgattagagtt
cgaatctctacacccctgcca
1−11tRNA−Ser(tga)[26331,26420]
ggtaggtagcggctaatggcagccaaacagtcttgaaaactgttgccact
gtagagatacggtgagggttcgactcctttacttaccgcc
1−12tRNA−Leu(tag)[26675,26751]

gcacctatagcccaactggtagaggcagcagacttagaatctgctcagtg
tgagttcgaatctctctaggtgtacca
1-13tRNA-Lys(ctt)[26759,26834]
gcaggtgtagcaaaatggttatgcggctgactcttaatcagtaagacgat
gggttcaattccctccacctgtacca
1-14tRNA-Ala(tgc)[26841,26916]
ggggtcatagtttatatggttaaaattcgagttttgcaaacttgggaact
gagttcaattctcagtgactccacca
1-15tRNA-Gly(tcc)[26923,26997]
gcatccatagtttaaacgggaaaattacagtcttccaaactgaggttgag
ggttcgattccctctggatgctcca
1-16tRNA-Thr(tgt)[27005,27080]
gctcctatcgtataattggttattacggttgccttgtaagcaacttatca
gggttcgagtccttgtgggagcacca
1-17tRNA-Val(tac)[27177,27251]
actcgcttagtttatatggtaaaacatcacccttacaagatgaagaaaaa
ggttcaagtcctttagtgagtacca
1-18tRNA-Leu(caa)[27253,27330]
gttccagtatcccaattggcagaggatgcaagctcaaaccttgtattagt
gacggttcgaatccgtcttggaacacca
1-19tRNA-Arg(acg)[27336,27411]
gcaggattagttcaaatggaaagagcaacagtctacgaagctgttaatag
gggttcgaatcccttatcctgcgcca
1-20tRNA-Gln(ttg)[28234,28309]
aggggattagtttacaaggttaaaacctcggtctttgaaatcgaagaagt
tggttcaattccaacatcccccgcca
1-21tRNA-Leu(taa)[28312,28390]
gctccattactccaattggcagagaggccagacttaaaatctgtgttatg
tatcggttcgaatccgatatggagtacca
1-22tRNA-Gln(ctg)[28396,28471]
agcggtatagcataactggcaatgcagcagtctctgaagctgtcctatta
aggttcaaatccttatgccgctgcca
1-23tRNA-His(gtg)[28503,28578]
gtggccttatcataaatggtaatgacccatgctgtgaacatggtctatac
gggttcaaatcccgtaggtcacccca
1-24tRNA-Phe(gaa)[28585,28660]
agtccaagtagcttatatggttaaagcgcgtgtctgaaaaacatgagaag
agggttcaaatcccactggactacca
1-25tRNA-Ser(gct)[29414,29506]
ggaagattaaccctaaaaggtaagggagcagtttgctaaactgccagtag
ctgagaaatcggtgtaccagttcaagtctggtatcttcctcca
1-26tRNA-Cys(gca)[29511,29586]
gaatccgtgacagaaacggctatgtgcctgtctgcaaaacaggtttataa
gggttcgagtcccttcggattctcca

>KP869110.1 Escherichia coli O157 typing phage 12, complete genome

1-1tRNA-Ile(gat)c[419,494]
gctggtatagttaagaaggttataacactcccctgataagggaacatcgg
tggttcgattccacctaccagtacca
1-2tRNA-Met(cat)c[496,572]
ggttcagtcgcagataaggtaatgcaagggtctcataagccctatgaatg
tgggttcgattcccatctgaacctcca
1-3tRNA-Lys(ttt)c[577,652]
ggaagtgtagcagaatggtgatgcggcagacttttaatctgacaggcgat
gggttcgaatccctccacttctacca
1-4tRNA-Asp(gtc)c[1082,1159]
ggttatgtagtttaatctggttaaaatactcccctgtcacgggagatgat
gagggttcgaatcccttcgtaaccgcca
1-5tRNA-Tyr(gta)c[1165,1252]
gtgtcgttatcccgtagatggtagcggtggggactgtaaatcccttgtca
ttgagactcggtaggttcgactcctacacggcacacca
1-6tRNA-Asn(gtt)c[1262,1338]
ggttaggaagcacataaggtatgtgcggtcgcctgttaagcgaatggcac
agggttcgaatccctgactaaccgcca
1-7tRNA-Met(cat)c[1423,1499]
tgcgggtatagagaaagggcgtctcacatgtctcattagcatggtatcgg
caggttcgactcctgcacccgcctcca
1-8tRNA-Glu(ttc)c[1590,1667]
gttccagtagacaaaatggtaaagtcaccactctttcaaagtggatatct
gagggttcaaatcccttctggaacgcca
1-9tRNA-Pro(tgg)c[1675,1751]
ctctgtttagctcagcttggtagagcgttccgtttggggcggtaaggccg
gaggttcaagtcctccaacagagacca
1-10tRNA-Cys(gca)c[84667,84742]
gaatccgtgacagaaacggctatgtgcctgtctgcaaaacaggtttataa
gggttcgagtcccttcggattctcca
1-11tRNA-Ser(gct)c[84747,84839]
ggaagattaaccctaaaaggtaagggagcagtttgctaaactgccagtag
ctgagaaatcggtgtaccagttcaagtctggtatcttcctcca
1-12tRNA-Phe(gaa)c[85593,85668]
agtccaagtagcttatatggttaaagcgcgtgtctgaaaaacatgagaag
agggttcaaatcccactggactacca
1-13tRNA-His(gtg)c[85675,85750]
gtggccttatcataaatggtaatgacccatgctgtgaacatggtctatac
gggttcaaatcccgtaggtcacccca
1-14tRNA-Gln(ctg)c[85782,85857]
agcggtatagcataactggcaatgcagcagtctctgaagctgtcctatta
aggttcaaatccttatgccgctgcca
1-15tRNA-Leu(taa)c[85863,85941]

gctccattactccaattggcagagaggccagacttaaaatctgtgttatg
tatcggttcgaatccgatatggagtacca
1-16tRNA-Gln(ttg)c[85944,86019]
aggggattagtttacaaggttaaaacctcggtctttgaaatcgaagaagt
tggttcaattccaacatcccccgcca
1-17tRNA-Arg(acg)c[86842,86917]
gcaggattagttcaaatggaaagagcaacagtctacgaagctgttaatag
gggttcgaatcccttatcctgcgcca
1-18tRNA-Leu(caa)c[86923,87000]
gttccagtatcccaattggcagaggatgcaagctcaaaccttgtattagt
gacggttcgaatccgtcttggaacacca
1-19tRNA-Val(tac)c[87002,87076]
actcgcttagtttatatggtaaaacatcacccttacaagatgaagaaaaa
ggttcaagtcctttagtgagtacca
1-20tRNA-Thr(tgt)c[87173,87248]
gctcctatcgtataattggttattacggttgccttgtaagcaacttatca
gggttcgagtccttgtgggagcacca
1-21tRNA-Gly(tcc)c[87256,87330]
gcatccatagtttaaacgggaaaattacagtcttccaaactgaggttgag
ggttcgattccctctggatgctcca
1-22tRNA-Ala(tgc)c[87337,87412]
ggggtcatagtttatatggttaaaattcgagttttgcaaacttgggaact
gagttcaattctcagtgactccacca
1-23tRNA-Lys(ctt)c[87419,87494]
gcaggtgtagcaaaatggttatgcggctgactcttaatcagtaagacgat
gggttcaattccctccacctgtacca
1-24tRNA-Leu(tag)c[87502,87578]
gcacctatagcccaactggtagaggcagcagacttagaatctgctcagtg
tgagttcgaatctctctaggtgtacca
1-25tRNA-Ser(tga)c[87833,87922]
ggtaggtagcggctaatggcagccaaacagtcttgaaaactgttgccact
gtagagatacggtgagggttcgactcctttacttaccgcc
1-26tRNA-Trp(cca)c[88355,88425]
aggggtgagctatggcagcaatagagtctccaaaattcttgattagagtt
cgaatctctacacccctgcca
>KP869101.1 Escherichia coli O157 typing phage 3, complete genome
1-1tRNA-Met(cat)[76953,77029]
ggccctgtagctcaatggttagcagcagtcccctcataagggaaaggtta
ccagttcgaatctggtctgggtcacca
1-2tRNA-Arg(tct)[77033,77109]
cgaggcatagctcaattgtatagagcaacggacttctaatccgtaggttg
aaggttagaatccttctgtctcgacca
>KP869103.1 Escherichia coli O157 typing phage 5, complete genome
1-1tRNA-Pro(tgg)c[86825,86901]

ctccgcgtagctcagcttggtagagcgcctgatttgggatcaggaggtcg
agtgttcgaatcactccgtggagacca
1-2tRNA-Met(cat)c[86913,86987]
ggccctgtagctggaaggttcaagcaagcgactcataatcgccagacggt
ggttcaattccacccagggccacca
1-3tRNA-Thr(tgt)c[86989,87064]
gctgatgtagcacaatcggtagtgcaattgatttgtaatcaataggttgt
aggttcaagtcctgccatcagcacca
1-5tRNA-Ser(tga)c[87267,87357]
ggagggtagggagcaatggtgctcaagcggtcttgaaaaccgtcccgttg
aggatgactcgatgatggttcgattccattactctccgcca
1-6tRNA-Tyr(gta)c[87472,87558]
ggggagttatcccgtagaggtagcggtgtagactgtaaatctattgtcat
tgcgactcgggtggttcgactccaccactccccacca
1-7tRNA-Arg(tct)c[87563,87637]
gccctgttagcttagtggataaagcagcggccttctaagccgttgacact
ggttcgagtccagtacggggtgcca
>KX229736.1 Campylobacter phage PC5, complete genome
1-1tRNA-Met(cat)[60294,60368]
gggcgagtagctcaatggtagagcaaccggctcataaccggttggttata
ggttcgattcctatttcgcccacca
1-2tRNA-Asn(gtt)[60567,60641]
tgcagattagcacagtggtagtgcaatcgactgttaatcgatgagtcaga
ggttcgaatcctctatctgcagcca
1-3tRNA-Tyr(gta)[60660,60744]
gtagaagtaggataattggtaatccaccagactgtaaatctggcgtcttt
tggcattgatggttcaagtccatccttctacacca
>CP011970.1 Peptoclostridium phage phiCDIF1296T strain DSM 1296, complete sequence
1-1tRNA-Ser(gct)[128597,128687]
ggagaggtactcaagtggtaaagaggatagtttgctaaactattaggtca
attaatttgatacgagggttcaaatcccttcctctcttcca
>KR052481.1 Sinorhizobium phage phiM19, complete genome
1-1tRNA-Thr(tgt)c[78771,78854]
gccggtatagccaagcggtcgaaggcacctgatttgtaatcaggtattca
aacgtcggcagttcgaatctgtctaccggctcca
1-2tRNA-Gln(ttg)c[78855,78928]
ttgcccttagttcagttggtagaacgtcgaattttggtttcgaaggtccg
tggttcgaatccacgagggtaagc
1-3tRNA-His(gtg)[83801,83877]
tcatggatggtgttagtctggtagcacgcgcggatgtggaccgcgaagca
taggttcaaatcctattccatggacca
1-4tRNA-Leu(caa)[92642,92729]
tgggagataaaggaaacttaccagttcaattctggtttctccaagagtaa
tcgagtggttgtaggttcgagtcctacctccccagcca

1-5tRNA-Met(cat)[93280,93356]
tgcggggtagtagcagtggttagctcgtctgcctcataagcagaaggtca
tcagttcgaatctgatccccgcaacca
1-6tRNA-Cys(gca)[94043,94117]
gacgagaaggcagaagggttatgcgcggcgttgcaaccgccgtatgtgtc
ggttcaagtccggctctcgtctcca
1-7tRNA-Lys(ttt)[98675,98751]
gcgcgcttaactcagttggttagagtggcgaccttttaagtcgtttgtcc
ccggttcgagtccgggagcgcgcacca
>KR296695.1 Salmonella phage 41, complete genome
1-1tRNA-Met(cat)[85026,85102]
ggttctgtagcttagatggttagagcgcttccctcataaggaaggggtcg
caggttcaaatcctgccagaaccacca
1-2tRNA-Lys(ctt)[85796,85871]
ggggtattaactcagcggttagagtagcggcctcttaagccgaaggtcga
aggttcgaatccttcatgccccacca
1-3tRNA-Thr(tgt)[88074,88149]
gctcgtatagctcagttggtagagcaactcccttgtaaggagaaggtcgt
gggttcgaagcctactgcgagcacca
1-4tRNA-Arg(gcg)[88156,88229]
tcgttgatagccttagcggtaaaggcccactctgcgcaagtggttcagtg
gttcgaatccacttcaacgagcca
1-5tRNA-Leu(taa)[88531,88606]
tgcgtccttggtccaatttggtagaggcatgaggcttaagacttcaggtt
cccagttcgaatctggagggcgcacc
1-6tRNA-Arg(tct)[88609,88682]
cggggtgtagtctaagggatagcagcggtcttctaaaccgctatatgctg
gttcgaatccagtcaccccgacca
1-7tRNA-Cys(gca)[88799,88872]
gcacggttgctggagtggcaacagcttcggatgcaaaccgaatgtcagga
gttcgattcttctaccgtgctcca
1-8tRNA-Tyr(gta)[89528,89617]
gtgtcgttagaccgtaggggtagcggggcagactgtaaatctgctggctc
gaaagggcttcgggtagttcgactctatcacggcacacca
1-9tRNA-Ile(gat)[89625,89700]
gggagtatagctcagttagtagagcgctcgaccgataatcgagaggtcgc
aggagcaaagcctgctactcccacca
1-10tRNA-Glu(ttc)[90467,90542]
gctcctatcggctaagtggataggccgccagactttcaatctggaaatcc
gagttcgattctcggtaggagtgcca
1-12tRNA-Trp(cca)[90628,90704]
agggacttagcacaattggctagtgcagcggattccaaatccgcaggttc
tgggttcgaatcccaaggtccctgcca
1-13tRNA-Gln(ttg)[90710,90784]

aggaggatggtgtaattggttagcacacgggtctttgaaatccgtagttc
aggttcagtcctgatcttcctgcca
1−14tRNA−Ser(gga)[91017,91108]
ggtggaatggtcgagcggttaaagacagcaccttggaaaggtgtcggccc
cctcacaggggtccgtaggttcgaatcctacttccaccgcca
1−15tRNA−Ser(gct)[91115,91208]
ggaagattaaccctaatcaggtaagggatctctttgctaaagagacagta
gccccggaaaggggtgtgtcagttcaaatctgacatcttcctcc
1−16tRNA−Ser(tga)[91209,91297]
ggagagcagggcgcgtggtgcgtaatccggtttgaacccggacccatcgt
agcgatacggtgacagttcgattctgttgctctcctcca
1−17tRNA−Asn(gtt)[91451,91535]
gacgagttggcctagtggttgggcggcggcccgttaagccgtgagtgaaa
actcaaggaaggttcaaatccttcactcgtcgcca
1−18tRNA−Asp(gtc)[91543,91618]
ggggatatggtattagcggtaaacatactggcctgtcacgccggagtcac
gggttcgaatcccgttatcctcgcca
1−19tRNA−His(gtg)[91623,91696]
gggaaattagttaagtggtataatggcctctggtgctgagggcgtctcct
gttcgattcaggaatttcccgcca
>KR698074.1 Escherichia phage APCEc02, complete genome
1−1tRNA−Arg(tct)[77260,77334]
gccctgttagcttagtggataaagcagcggccttctaagccgttgacact
ggttcgagtccagtacggggtgcca
1−2tRNA−Tyr(gta)[77339,77425]
ggggagttatcccgtagaggtagcggtgtagactgtaaatctattgtcat
tgcgactcgggtggttcgactccaccactccccacca
1−3tRNA−Ser(tga)[77540,77630]
tgagggtagggagcaatggtgctcaagcggtcttgaaaaccgtcccgttg
aggatgactcgatgatggttcgattccattaccctcagcca
1−5tRNA−Thr(tgt)[77833,77908]
gctgatgtagcacaattggtagtgcaattgatttgtaatcaataggttgc
aggttcgagtcctgctatcagcacca
1−6tRNA−Met(cat)[77910,77984]
ggccctgtagctggaaggttcaagcaagcgactcataatcgccagacggt
ggttcaattccacccagggccacca
1−7tRNA−Pro(tgg)[77996,78072]
ctccgcgtagctcagcttggtagagcgcctgatttgggatcaggaggtcg
agtgttcgaatcactccgtggagacca
>KT187252.1 Bacillus phage PBC6, complete genome
1−1tRNA−Met(cat)c[37,109]
agggttatagctcagtggtagtagcgtgggtctcataagcccaaggtcgt
aggttcgactcctactagcccta
1−2tRNA−Leu(taa)c[253,339]

tggcggagtgttggaattggtagacataacggacttaaaatccgttgctc
gttagggcgtggtgggttcgagtcccaccttcggtat
1-3tRNA-Ile(gat)c[349,425]
gctagggtagctcagtcaggtagagcagtgtcttgataaggcattggtcg
aaggttcaaatcctacccctagtacca
1-4tRNA-Tyr(gta)c[427,513]
gggcgagtatgcaaattggtgaagcaagcggtctgtaaaaccgtgacgta
agatacattgcaggttcgagtcctgcctttcccacca
1-5tRNA-Phe(gaa)c[650,725]
ggacggatagctcagttggtagagcagagggctgaaaatcctcgtgtcgt
aggttcgatccctactccgtccacca
1-6tRNA-Pro(tgg)c[806,881]
tgtggatatagctcagattggtagagcgcttgctttgggagcaagaagtc
gtaggttcgattcctgctatccgcat
1-7tRNA-His(gtg)c[888,960]
cggggtgtggcgtaacggttaacgcaagtgactgtggatcactgaatagg
ggttcgattcccctcatcctgac
1-8tRNA-Gln(ttg)c[1043,1115]
tttcggagtagccaagtggtaaggcaatagactttgactctatgatcggt
ggttcgagaccatcctccgaagt
1-9tRNA-Ser(tga)c[1197,1284]
cgaggaatactcaagtggataaaagaggatggtcttgaaaaccattaggc
gtgaaagcgtgcgggggttcgaatccctcttcctcggc
1-10tRNA-Arg(tct)c[1286,1362]
acctctatcgccaagcggatcaaggcaacgggcttctatcccgttaatcg
ggcgttcgaatcgctctaggggtgcca
1-11tRNA-Glu(ttc)c[1403,1477]
gtcccattggtgaaattggctaacacactcgactttctatcgagcattca
ggggttcgaatcccctatggggtac
1-12tRNA-Asp(gtc)c[1984,2057]
ggctcgttagtttaacggtaaaatacatgactgtctatcatgggtcacgg
gttcgactcccgtacgggtcgcca
1-13tRNA-Thr(tgt)c[2063,2138]
gccttcttagctcagttggtagagcgattgacttgtaatcaataggtcgt
gggttcgattcctacagtcggcacca
1-14tRNA-Gly(tcc)c[2143,2218]
ggggcattagtatatcggttcattattcctggcttccaaccaggggaggt
cggttcgattccgacatgtccctcca
1-15tRNA-Asn(gtt)c[2409,2484]
gtgcctgtagctcagttggtagagcaaaaccctgttaaggttgaggtcgt
aggttcgaaccctaccaggtacgcca
1-16tRNA-Cys(gca)c[2499,2571]
gaggatgtaccgaagcggctaacggtctaggttgcaaccctagtgttcat
gggttcgagtcccatcattctct

1-17tRNA-Ser(gct)c[2581,2674]
tggaggggtactcaagcggtgaagagggcagtttgctaaactgttagtac
ctactaataataggtagcaagggttcgaatcccttcccctccgt
>KT001917.1 Escherichia phage Murica, complete genome
1-1tRNA-Pro(tgg)c[60714,60790]
ctccgcgtagctcagcttggtagagcgcctgatttgggatcaggaggtcg
agtgttcgaatcactccgtggagacca
1-2tRNA-Met(cat)c[60802,60876]
ggccctgtagctggaaggttcaagcaagcgactcataatcgccagacggt
ggttcaattccacccagggccacca
1-3tRNA-Thr(tgt)c[60878,60953]
gctgatgtagcacaatcggtagtgcaattgatttgtaatcaataggttgt
aggttcaagtcctgccatcagcacca
1-5tRNA-Ser(tga)c[61156,61246]
ggagggtagggagcaatggtgctcaagcggtcttgaaaaccgtcccgttg
aggatgactcgatgatggttcgattccattactctccgcca
1-6tRNA-Tyr(gta)c[61361,61447]
ggggagttatcccgtagaggtagcggtgtagactgtaaatctattgtcat
tgcgactcgggtggttcgactccaccactccccacca
1-7tRNA-Arg(tct)c[61452,61526]
gccctgttagcttagtggataaagcagcggccttctaagccgttgacact
ggttcgagtccagtacggggtgcca
>KU521356.1 Pseudomonas phage KTN4, complete genome
1-1tRNA-Thr(tgt)c[267087,267163]
gccgctatagctcagctaggtagagcaacgcacttgtaatgcgtaggtcc
tccgttcgattcggagtggcggcacca
1-2tRNA-Asn(gtt)c[270882,270957]
tccgtgatagctcagtcggtagagcaagtgactgttaatcactgggtccc
tggttcgagtccaggtcacggagcca
1-3tRNA-Asp(gtc)c[270964,271040]
gcgctcatagttcagttggttagaatacccgcctgtcacgcgggtggtca
ggggttcgagtccccttgggcgcgcca
1-4tRNA-Met(cat)c[272013,272089]
gggcctatagctcagttggttagagcaggcgactcataatcgcttggtcg
caggttcaagtcctgctgggcccacca
1-5tRNA-Pro(tgg)c[272412,272487]
cggagtgtagcgcagttggtagcgcgcctgctttgggagcaggatgtcgg
gagttcgagtctccccactccgacca
1-6tRNA-Leu(taa)c[272497,272581]
gcccgaatggtgaaattggtaaacacagaagacttaaaatcttccggcta
cggtcttgtcggttcgaatccgacttcgggcacca
>KU160494.1 Vibrio phage vB_VmeM-32, complete genome
1-1tRNA-Met(cat)c[27879,27954]
ggccccttagctcagtggttagagcggtcggctcataaccgattggtcat

gtgttcaagtcacataggggccacca
1-2tRNA-Arg(tct)c[27971,28047]
gcttcgttaactcaattggatagagtacctgccttctaagcaggttgtta
caagttcaaatcttgtacgaagcgcca
1-3tRNA-Asn(gtt)c[28049,28124]
ggtggattaactcagttggtagagtgattgactgttaatcaattggccac
aggttcgattcctgtatccaccgcca
>KU255730.1 Escherichia phage phiE142, complete genome
1-2tRNA-Arg(tct)c[87920,87996]
cgaggcatagctcaattgtatagagcaacggacttctaatccgtaggttg
aaggttagaatccttctgtctcggcca
1-3tRNA-Met(cat)c[88003,88076]
ggccctgtagctcaacggttagcagcagtcccctcataagggaaaggtta
ccagttcgaatctggtctgggtca
>KU522583.1 Enterobacteria phage ECGD1, complete genome
1-1tRNA-Met(cat)[61400,61475]
ggttccatagctcactgacgagagcgcacacctcataagtgtcgagggca
gggattgtaaccctggggaaccacca
1-2tRNA-Leu(taa)[61507,61592]
gcaagtgtggtggaattggtgatacacattgtgcttaaaacgcaacgctt
aaatgattgagggttcgaatccctccacttgcacca
1-3tRNA-Ser(tga)[61920,62009]
ggaaggtagggcgcagcggtgcgcaaccaggtttgaaccctggcccattg
tagcgatacggtgatggttcgactccattatcttcctcca
1-4tRNA-Ser(gct)[62189,62274]
ggtagattggtgaaatggtagccacaacagtttgctaaactgtcgtcgga
aacggcgtgtaggttcaagtcctacatctaccgcca
1-5tRNA-Lys(ttt)[62401,62476]
acatcgttagcttagttggtagagcaaggggcttttaactccaaggtcga
aggttcgaatccttcacgatgtacca
1-6tRNA-Tyr(gta)[62482,62567]
gtaggtatcgcatagcggcaattgctggagactgtaaatctcttgccctt
cgggcttcgttggttcgagtccaactacctgcacca
1-7tRNA-Asn(gtt)[62575,62659]
gatggattggcctagtggttgggcgacggactgttaatccgtgaacgaaa
gttctagcaaggttcaaatccttgatccatcgcca
1-8tRNA-Thr(tgt)[62750,62823]
gctcctatcgtataacggctattacggttgccttgtaagcaacttatcag
gttcgagtccttgtgggagcacca
1-9tRNA-Gly(tcc)[63226,63300]
gcgggtatagtgttaatggtagcatatgaggtttccaccctccaggtgca
ggttcaagtcctgttactcgctcca
1-10tRNA-Gln(ttg)[63400,63473]
tggagattagcctagtgggaagcaacggcctttgaagtcgtgatgactag

gttcgattcctagatctccagcca
1-11tRNA-Pro(tgg)[63481,63556]
cagtgcgtagcgcagttggtagcgtgggagctttggatgcttcgggtcgc
aggttcgagtcctgccgcactgacca
1-12tRNA-Phe(gaa)[63562,63636]
gcagagatagctgagacggattagtgctaccctgaaaaggtagaaaggat
ggatcgttaccatctctctgcacca
1-13tRNA-Leu(tag)[63714,63798]
ggctaagtggtggaatggtatacacgatacgcttagaacgtattgcccca
agggattgtgagttcgagtctcaccttagctacca
1-14tRNA-Met(cat)[63887,63962]
tgatagtgtgaagctggcgtttcgagtgctttcataaggcattgcgagaa
gagttcgattctcttactatcaacca
1-15tRNA-Met(cat)[63978,64056]
cgcgggatagaggagtctggtcgtcctcgccagtttcatatgctggagat
catcggttcaaatccgattcccgcctcca
1-16tRNA-Ile(gat)[64203,64278]
tgtggtatcgtacagatggttagtacactcgaccgataatcgagaaacaa
cggttcgactccgtttaccacgacca
>KU574722.1 Pectobacterium phage CBB, complete genome
1-1tRNA-Ser(gct)[268791,268880]
ggtgagttgggggagtggtttaacccagcaccctgctaaggtgtcggcct
cgcaagaggcccgtgggttcaaatcccacactcaccgcca
1-2tRNA-Trp(cca)[272675,272749]
gggagtatcgtatatcggctattatgttcggttccaacccgaaagagcag
ggttcaactccttgtgctcccgcca
1-3tRNA-Thr(tgt)[275819,275892]
gcttctatcgtataatggcttattactttggctttgtaacccaaaaatct
gcgttcgattcgcagtggaagcac
1-4tRNA-Leu(tag)[280357,280442]
ggggatgtggcgaaattggtagcacgcatgagttttaggtactcacgcct
tcgggcgtgtcggttcgagtccgaccatccccacca
1-5tRNA-Arg(tct)[288345,288419]
gctcctatagttaagtggaaataacaggaaccttctaagttctagtccct
ggttcgattccaggtgggagtgcca
1-6tRNA-Pyl(cta)[289995,290067]
gcctttgtagctcagcggtagagcggggatctctaaaatcctttgtcgtg
ggttcgaatccctccatcggcac
1-7tRNA-Leu(taa)[290283,290369]
gcgggtgtggcggtattggtagtacgcaagggatttaaaatccctcagac
ttcggtcttgtgagttcgaatctcatcacccgtacca
1-8tRNA-Met(cat)[290748,290824]
ggccctttgtactgtcgcgtgaaagtactctgctcataacagatgtaagg
tgggttagactcccacaagggccacca

1–9tRNA–Leu(taa)[292672,292756]
gcgtttgtggtgaaattggtaaacacaggagacttaaaatctcccgcctt
taggtttgtcggttcgagtccgaccagacgcacca
1–10tRNA–Leu(caa)[299089,299174]
gggaatatggcgaaattggtagtacgcaccacactcaaaatgtggcgagg
caactcgtgtcggttcgagtccgactattcccacca
1–11tRNA–Glu(ttc)[302061,302132]
gttcccatcgtctagtggtaggacttttgactttctatcaaaaaacctcg
gttcgaatccgggtgggaacgc
1–12tRNA–Tyr(gta)[302143,302231]
gggggaatagtccgtagttggtagcggggcagactgtaaatctgctacac
gaaagtgttcgggtggttcgactccatcttccctcacca
1–13tRNA–Ser(tga)[302564,302654]
ggaagattggcagagcggtttaatgcacatgtcttgaaaacatgcggggg
ttaatagccctccctgggttcgaatcccaggtcttccacca
1–14tRNA–Ile(gat)[304227,304300]
agcgggttagtatagaggcattacactcgactgataatcgagcgaaggcg
gatcgttaccgccacccgctacca
1–15tRNA–Asn(gtt)[307123,307195]
gactgtatagctcagaggcagagcggctccctgttaaggagaaggtcggg
atttcgaaattccctacggtcgc
1–16tRNA–Gln(ttg)[307716,307789]
aggcgtatagtgtaattggttagcacaccaggttttgatcctggtaatcg
aggttcaagtccttgtatgcctgc
1–17tRNA–Gly(tcc)[308195,308267]
gcatccatcgtatatcggctattataattggcttccacccaatagaacgg
agttcgaccctccgtggatgctc
1–18tRNA–Asp(gtc)[309725,309797]
ggagccatcgttcaacggttaggatacggcactgtcactgctgagatggg
gtttcgattacccctggttccgc
1–19tRNA–Arg(acg)[310642,310718]
gtttgtgtagctcagttggaattagagcatttgtctacgaaacaaagggt
cgcaggttcgagtcctgccatgaacac
1–20tRNA–Pro(tgg)[311781,311853]
ctccggttgggtcaatggttgatcgcttgttttgggaacaagaatatgga
agttcgattctttcactggagac
1–21tRNA–Pro(tgg)[311858,311933]
cgggatgtagctcagttgatagagtgcgtcgtttggggcgatgaagccgc
tggtttgagtccagccatcccgacca
1–22tRNA–His(gtg)[312274,312350]
gtgactatgagcgtaactggctaacgttggagattgtgaaactcctgatt
cgggttcaagccccgatagtcacacca
1–23tRNA–Phe(gaa)[312636,312712]
gggtcggtaactcagttggtcagagtggtacactgaaaatgtattggtcg

cgagttcgaatctcgcccgtcccacca
1-24tRNA-Lys(ttt)[313822,313895]
acctcgttaactcagttggtagagtaacggtcttttaaaccgtgagtggt
gtgttcgattcacacacgaggtac
1-25tRNA-Cys(gca)[318801,318878]
gcggggatgcctgagttggcttaaaggagcggattgcaaatccgatgttc
gtgggttcgaatcccactccctgctcca
1-26tRNA-Met(cat)[319753,319828]
ggtcagttagttcagttggtagaacgctgcactcataatgcagaagtcac
tggatcgtagccagtactgaccacca
1-27tRNA-Met(cat)[320334,320411]
tgcggagtagagcagtctggttagcttgcaaggctcataaccttgaggtc
ggtggttcaaatccatcctccgcatcca
1-28tRNA-Ala(tgc)[320478,320551]
ggggctatagctcagttgggagagcgtctgccttgcacgtagaaggtcaa
cggttcgaacccgtttagctccac
>KU847400.1 UNVERIFIED: Bacillus phage Crookii, complete genome
1-1tRNA-Asn(gtt)[10253,10328]
gtgcttgtagctcagtaggtagagcgagtggctgttaaccactgtgtcgt
tggttcgaatccatcctagcacgcca
1-2tRNA-Asp(gtc)[10333,10406]
tggggatatagtgtagtggtaaacacgcacggctgtctaccgtgaagcac
gagttcgagtctcgttatcctcgt
>KU925172.1 Escherichia phage PE37, complete genome
1-1tRNA-Gln(ttg)[93727,93800]
tgggaattagccaagttggtaaggcactggattttgattccaggatgcaa
aggttcgagtcctttattcccagc
1-2tRNA-Leu(taa)[93801,93887]
gcgagaatggtcaaattggtaaaggcacagcacttaaaatgctgcggaat
gatttccttgtgggttcgagtcccacttctcgcacca
1-3tRNA-Gly(tcc)[93893,93966]
gcggatatcgtataatggtattacctcagacttccaatctgatgatgtga
gttcgattctcattatccgctcca
1-4tRNA-Pro(tgg)[93977,94051]
ctccgtgtagctcagtttggtagagcgcctgatttgggatcaggaggtcc
aaggttcaaatccttgtatggagac
1-5tRNA-Ser(tga)[94053,94142]
ggaggcgtggcagagtggtttaatgcaccggtcttgaaaaccggcagtcg
ctccggcgactcataggttcaaatcctatcgcctccgcca
1-6tRNA-Thr(tgt)[94148,94223]
gctgatttagctcagtaggtagagcaactcacttgtaatgagaaggtcgg
cggttcgattccgtcaatcagcacca
1-7tRNA-Met(cat)[94225,94299]
ggccctgtagctggaaggttcaagcaagcgactcataatcgccagatggt

ggttcaattccacccagggccacca
1-8tRNA-Tyr(gta)[94309,94395]
ggggagttatcccgtagaggtagcggtgtggactgtaaatccattgtcat
tgcgactcgggtggttcgactccatcactccccacca
1-9tRNA-Asn(gtt)[94400,94474]
ggatgtgtagctcaatggcagagcgatcgcctgttaagcgattggttata
ggttcgaatcctatcacgtccgcca
1-10tRNA-Arg(tct)[94479,94554]
cgaggcatagctcagaaggaagagcaaggaccttctaagtcctaggtcgt
aggttcgatccctactgcctcgacca
>KX171211.1 Salmonella phage vB_SenM-2, complete genome
1-1tRNA-Ser(gct)c[132306,132396]
ggaaggttgcccgagaggtttaagggactcgactgctaatcgagagtggg
gcttttagcccccgaaggttcgaatccttcaccttccgcca
1-2tRNA-Tyr(gta)c[132824,132905]
gtgagtgtggcagagcggtcgaatgcgcctgactgtaaatcaggtatccc
acgcggtggttcgaatccatccactcacacca
1-3tRNA-Asn(gtt)c[133301,133376]
gacgatgtagttcagtcggtagaacggtggactgttaatccatatgtcgc
aggttcaagtcctgccatcgtcgcca
1-4tRNA-Met(cat)c[133562,133637]
ggccccgtagctcagtggttagagcagtgaactcataattcattggtcgc
tggttcaagtccagccagggtcacca
>KX495186.1 Bacillus phage PK16, complete genome
1-1tRNA-Met(cat)c[154511,154585]
tacggttatagctcagtggtagagtgtgggtctcataagcccaaggtcga
tggttcaatcccatctaaccgtatc
1-2tRNA-Leu(tag)c[154678,154762]
gtcagagtgttggaactggtatacatgcggcacttagaatgccgtgcctt
cgggattgtgggttcgactcccacctctgacacca
1-3tRNA-Ile(gat)c[154771,154847]
actagtgtagctcagtcaggtagagcagtgtcttgataaggcattggtca
caggttcgaatcctgtcactagtacca
1-4tRNA-Leu(taa)c[154854,154940]
atcggaatgttggaattggtagacataacgcacttaaaatgcgttgctcc
ttggggcgtgagggttcgagtccctcttccgatacca
1-5tRNA-Tyr(gta)c[154944,155027]
gggcgtgctatcattggagagataagctgactgtaaatcagtggtcgtaa
gactgtggaggttcgaatccttccattcccacca
1-6tRNA-Phe(gaa)c[155034,155108]
ggatagatagctgagacggattagcgagtggttgaaacccactagaggtt
ggatcgttcccaactctatccacca
1-7tRNA-Pro(tgg)c[155202,155274]
acgggtgtaggctagaggtcagtcactgcgtttggggcgcagatcacgtt

ggttcgatcccaaccactcgtac
1-8tRNA-His(gtg)c[155368,155441]
cagtgtgtggtgaagtggcttaacacggacgactgtggatcgttcattcg
caggttcgaatcctgtcatgctga
1-9tRNA-Gln(ttg)c[155519,155594]
tttcggtgtggacaaactggtaaagtcactaggctttgaccctaggtttt
ggaggttcgacccccttccaccgaagt
1-10tRNA-Ser(tga)c[155670,155757]
ggagggctactctaactggtaagagacctgccttgaaagcagacgtaggg
taaaaccgataggagttcgagtctcctgccctcctcca
1-11tRNA-Arg(tct)c[155848,155923]
taccccttagccaagtggattaaggcaacgggcttctatcccgaagatc
gtgggttcgattcctacagggggtgt
1-12tRNA-Ser(act)c[156043,156116]
atccctttagccaagcggttaaggcagtgggacactctcccacgaatcgg
gagttcgatcctcccaaggggcgc
1-13tRNA-Glu(ttc)c[156119,156194]
tggcgcattggtgaagcggtttaacacactcggctttctaccgaggatgc
gtgggttcgaaacccacatgcgctat
1-14tRNA-Gly(tcc)c[156792,156867]
ggggcattcgtatagtggcttattatacctggcttccaaccaggggaggt
ggtttcgattaccacatgtccctcca
1-15tRNA-Thr(tgt)c[156873,156949]
gccttcttagctcagttggttagagcgactgccttgtaagcagtaggtcg
tgcgttcgaatcgtacagtcggcacca
1-16tRNA-Asp(gtc)c[156962,157035]
ggctcgttagtttaacggtaaaatacatgactgtctatcatgggtcacgg
gttcaactcccgtacgggtcgcca
1-17tRNA-Asn(gtt)c[157227,157302]
gtgcctgtagctcagtcggtagagcagaaccctgttaaggttaaggtcgt
aggttcgaaccctaccaggtacgcca
1-18tRNA-Cys(gca)c[157348,157420]
gaggacgtaccgaaacggctaacggtctaggttgcaaccctagtatgtgt
gagttcgaatctcaccgttctct
1-19tRNA-Ser(gct)c[157427,157515]
ggaagggtactcaagttggtgaagaggtcaacctgctaagttgatagtac
ctgttaaaggtagcgagggttcgaatccctttccttcct
>KX443552.1 Cronobacter phage vB_CsaM_leE, complete genome
1-1tRNA-Gly(tcc)[50310,50385]
gcaggtgtagttcaattggtagaatatctggtttccatccagattgttga
gggttcgagtcccttcacctgctcca
>KX552041.2 Escherichia phage ESCO13, complete genome
1-1tRNA-Met(cat)[57537,57611]
ggccctgtagctcaatgggagagctgtcggctcataaccgataggtagct

ggatcgaaaccagccagggccacca
1–2tRNA–Arg(tct)[57614,57688]
gtctttatggtgtaatggatagcacaggagtcttctaaactcttagtcaa
ggttcgattccttgtaaagacacca
1–3tRNA–Ser(gct)[58019,58104]
ggtagattggtgaaatggtagccacgacagtttgctaaactgtagtcgga
aacggcgtgtaggttcgagtcctacatctaccgcca
1–4tRNA–Asn(gtt)[58329,58414]
gatgagttggctgaatggtttaagcggcggactgttaatccgtgttcgaa
agaacaatataggttcgaatcctatactcatcgcca
1–5tRNA–Thr(tgt)[58506,58580]
gctcctatagtataatggctattacaacggttttgtaatccgtggatctc
tgttcgattcagtgtgggagcacca
1–6tRNA–Gly(tcc)[58895,58968]
gcgttcttggtgtagcggtagcattccgtccttccaagtcggcggcacga
gttcgatcctcgtagaacgctcca
1–7tRNA–Gln(ttg)[59067,59142]
tggtttgtagtgtagtccggtagcacacgacactttgactgtcgtaggtt
tggttcaaatccagacagaccagcca
1–8tRNA–Pro(tgg)[59236,59312]
cgggacgtagcacagcttggtagtgcgctcgctttgggagcgagaggtcg
agtgttcgaatcactccgtcccgacca
1–9tRNA–Ile(gat)[59319,59392]
tgtttcgtagttcaatggtagaacccacgactgataatcgtgagacacaa
gttcgattcttgtcgaaacaacca
1–10tRNA–Met(cat)[59471,59549]
agcgggatagaggagtctggtcgtcctcgccagtttcatatgctggagat
catcggttcaaatccgattcccgcctcca
>KX431559.1 Cronobacter phage vB_CsaM_leB, complete genome
1–1tRNA–Gly(tcc)[49614,49689]
gcaggtgtagttcaattggtagaatatctggtttccatccagattgttga
gggttcgagtcccttcacctgctcca
>KX431560.1 Cronobacter phage vB_CsaM_leN, complete genome
1–1tRNA–Gly(tcc)[49078,49153]
gcaggtgtagttcaattggtagaatatctggtttccatccagattgttga
gggttcgagtcccttcacctgctcca
>KX721256.1 Mycobacterium phage Erdmann, complete genome
1–1tRNA–Ser(gct)[31552,31635]
ggagggtgagcatctggtgatgcaggggtcctgctaaggccctacggatt
cacacccgtgagtttcgattactcctccctccgc
1–2tRNA–Leu(cag)[31732,31808]
gccctgctgagcaaactggcaaagctgccgcattcagagtgcgggtcatt
tccgggttcgactcccgggcagggtac
1–3tRNA–Leu(gag)[31928,32002]

gtctctgtaggcaaatcggaaaagccgccatcttgagggggtggtgcgtg
cgggttcgactcccgccagagacac
1-4tRNA-Leu(caa)[32003,32076]
gccgtggtaggccatctggcgagccgccgagttcaagtttcggtgtttgc
gggttcgaatcccgcccacggtac
1-5tRNA-SeC(tca)[68480,68576]
attctggcactggtgggcgagcccaccggcggcagcttcaagctgtcgct
ggccggagaaccgaccggaaacatcccgttcaacgcgacccagggcc
1-6tRNA-Pro(tgg)[92444,92518]
cggggtgtagttcagtttggaagagcgcttggtttgggaccaagatgtcg
caggttcgaatcctgtcaccccgac
1-7tRNA-Trp(cca)[92535,92610]
tggggtgaagccgatctggaaggcagcggtctccaaagccgtctcatagc
gggttcgaatcccgtcacccctgcca
1-8tRNA-Tyr(gta)[92612,92698]
gccgcacatgcccaactggtgtttgggagcaggctgtaaccctgtggcct
ccgggacggtgaggttcgattcctcagtgcggtacca
1-9tRNA-Pyl(cta)[93808,93880]
gcaccatttgctcaatggcagagcggcggcttctaactccgtgagtgccg
gttcgattccggcatggtgcacc
1-10tRNA-Met(cat)[94030,94104]
agcggtgtagagcagctaggtagctcgccgggctcataacccggaggacg
cgtgttcgaatcacgccaccgccac
1-11tRNA-Cys(gca)[94231,94302]
gcgcctttggcggaatggctacgtgctcggctgcaacccgagttatcccg
gttcgactccgggagggcgctc
1-12tRNA-Glu(ctc)[94307,94381]
ggtccgttggagtagatggatatctcgccaccctctcaaggtggagatca
cgggttcaagtcccgtacggactgc
1-13tRNA-His(gtg)[94383,94456]
gtggccgtagttcagccggtagaacgctgggttgtgatcccagtcgtcga
gggttcgagtccctccggtcaccc
1-14tRNA-Ala(tgc)[94618,94692]
gggcctgtagctccaattggtagagcagcatccttgcaagatgacggctg
tcggttcgaatccgacctggtccac
1-15tRNA-Phe(gaa)[94882,94954]
gccgtcatagctcagttggtagagcactggcctgaaaacccagtggccga
ggttcgattcctcgtgtcggcac
1-16tRNA-Val(cac)[94960,95033]
gtccgtttagctcagctggaagagcgctcggtccacacccgagaggccgc
aggttcgatccctgcaatggacac
1-17tRNA-Lys(ctt)[95152,95224]
gccttcgtagctcagtggtagagctgtcgcctcttaagcgataggtcgtt
ggttcgaatccagccgggggcac

1-18tRNA-Glu(ttc)[95229,95305]
ggtcgggtcggtctgctggtatggccagtcggattttcactccggacatt
cgcgggttcaattcccgtcccgatcgc
1-19tRNA-Gly(tcc)[95385,95457]
gcgggtgtggccgaatggctcaggcaccagatttccactctggctacgca
ggttcgattcctgtcatccgctc
1-20tRNA-Thr(cgt)[95518,95592]
gctgctgtagctcacctggtagagcgtcggcgtcgtatcccgaaggcatc
cggttcgagtccggacagcagcccc
1-21tRNA-Thr(tgt)[95593,95665]
gcctctgtggtccagcggcacggacatccgccttgtaagcggaggacccc
cgttcgatccggggtagaggctc
1-22tRNA-Thr(ggt)[95725,95797]
gctgggttagctcagtggtagagcgttcctctggtatgggaaaggcccgg
ggttcaatcccccgactcagctc
1-23tRNA-Gly(gcc)[97120,97193]
gcgaaggtagctcagctggcagagcgccaccttgccaaggtggaggtcgc
gggatcgtaacccgttcttcgctc
1-24tRNA-Asp(gtc)[97197,97269]
ggccctgtagctcagaggaagagcgccggtctgtcgaatcggaggtcgcg
gtatcgtaatccgtcagggtcgc
1-25tRNA-Met(cat)[97329,97401]
gcctcactagctcattggtagagccgctcgctcataacgtgcaggtacct
ggttcgattccagggtgaggtac
1-26tRNA-Ile(gat)[97407,97481]
gcctgttagcggactggtcgtccgatccaagctgataacttggcgtaagc
ggtgttcgattcaccgagcaggtac
1-27tRNA-Arg(acg)[97575,97647]
gcctctatggtccaacggatatgacgccggtctacggaaccggagatgcg
tgttcgattcgcgctaggggcac
1-28tRNA-Val(gac)[97691,97763]
gtccgtgtagctcaggggtagagcgcctgctcgacacgcaggaggaccga
ggttcgaaacctcgcatggacac
1-29tRNA-Arg(cct)[97937,98010]
gcctctgtagctcaacggacagagcaacgcggtcctaacgcggtggctgg
aggttcgaatcctctcggaggcac
1-31tRNA-Gln(ttg)[98551,98626]
tggggtatggtggcaatctggcagtcccgccggactttgactccggaggt
gcaggttcgagtcctgctaccccatc
1-32tRNA-Arg(tct)[98630,98705]
gcccttgtagctcagtggacagagcggcgagcttctacctcgcgggccgg
gagttcgaatctctccaggggcacca
1-33tRNA-Gln(ctg)[126301,126375]
tgctcgttggtgtaactggcaacactacggactctgactccgtcatttta

ggttcgaatcctaagcgagcagcca
1-34tRNA-Asn(gtt)[126382,126457]
tggggtgtccgttaatcaggcaaacgagcggactgttaatccgcccctgc
aggttcgaatcctgccaccccagcca
>KX721255.1 Mycobacterium phage Yucca, complete genome
1-1tRNA-Ser(gct)[31474,31557]
ggagggtgagcatctggtgatgcaggggtcctgctaaggccctacggatt
cacacccgtgagtttcgattactcctccctccgc
1-2tRNA-Leu(cag)[31654,31730]
gccctgctgagcaaactggcaaagctgccgcattcagagtgcgggtcatt
tccgggttcgactcccgggcagggtac
1-3tRNA-Leu(gag)[31850,31924]
gtctctgtaggcaaatcggaaaagccgccatcttgagggggtggtgcgtg
cgggttcgactcccgccagagacac
1-4tRNA-Leu(caa)[31925,31998]
gccgtggtaggccatctggcgagccgccgagttcaagtttcggtgtttgc
gggttcgaatcccgcccacggtac
1-5tRNA-SeC(tca)[68159,68255]
attctggcactggtgggcgagcccaccggcggcagcttcaagctgtcgct
ggccggagaaccgaccggaaacatcccgttcaacgcgacccagggcc
1-6tRNA-Pro(tgg)[91508,91582]
cggggtgtagttcagtttggaagagcgcttggtttgggaccaagttgtcg
caggttcgaatcctgtcaccccgac
1-7tRNA-Trp(cca)[91597,91667]
gggtctgtgcacagggtgcccgacggtctccaaagccgaaggcgggggtt
cgattccctccaggcctgcca
1-8tRNA-Pyl(cta)[92804,92877]
tgcgagatcgtgcacggcgcactaggagcttctaaccctccgactcgcgg
gttcgactcccgcatctcgcaccc
1-9tRNA-Met(cat)[93025,93099]
agcggtgtagagcagctaggtagctcgccgggctcatgacccggaggacg
cgtgttcgattcacgccaccgccac
1-10tRNA-Cys(gca)[93314,93385]
gcgcctttggcggaatggctacgtgctcggctgcaacccgagttatcccg
gttcgactccgggagggcgctc
1-11tRNA-Glu(ctc)[93453,93524]
gctcccatggggtagtggtaaccctcctggttctcagccaggcgtcccga
gttcgatcctcggtgggagtgc
1-12tRNA-His(gtg)[93526,93599]
gtggccgtagttcagccggtagaacgctgggttgtgatcccagtcgtcga
gggttcgagtccctccggtcaccc
1-13tRNA-Ala(tgc)[93764,93837]
gggcctatagctcatctggtagagcgcctgccttgcaagcaggaggcggc
aggttcaagtcctgttaggtccac

1-14tRNA-Phe(gaa)[94027,94099]
gccgtcatagctcagttggtagagcactggcctgaaaacccagtggccga
ggttcgagtcctcgtgtcggcac
1-15tRNA-Val(cac)[94105,94178]
gtccgtttagctcagctggaagagcgctcggtccacacccgagaggccgc
aggttcgatccctgcaatggacac
1-16tRNA-Lys(ctt)[94297,94369]
gccttcgtagctcagtggtagagctgtcgcctcttaagcgataggtcgtt
ggttcaaatccagccgggggcac
1-17tRNA-Glu(ttc)[94373,94448]
ggtcgggtggtctgttggcaggccggtcgggttttcaccccggcgtcatc
gcgggttcgattcccgtcccgactgc
1-18tRNA-Gly(tcc)[94578,94650]
gcgggtgtggccgaatggctcaggcaccagacttccattctggctacgca
ggttcgattcctgtcatccgctc
1-19tRNA-Thr(cgt)[94710,94784]
gctgctgtagctcacctggcagagcgtcggcgtcgtatcccgaaggcatc
cggttcgagtccggacagcagcccc
1-20tRNA-Thr(tgt)[94785,94857]
gcctctgtggtccagcggcacggacatccgccttgtaagcggaggacccc
cgttcgatccggggtagaggctc
1-21tRNA-Thr(ggt)[95265,95337]
gctgggttagctcagtggtagagcgttcctctggtatgggaaaggcccgg
ggttcaatcccccgactcagctc
1-22tRNA-Gly(gcc)[97082,97157]
gcggttatagctcagcctggccagagcatcacgttgccaacgtgaacgtc
gcgggttcaaatcccgctagccgctc
1-23tRNA-Asp(gtc)[97171,97243]
ggccctgtagctcagaggaagagcacccgcctgtcgagcgggaggtcgcg
gtatcgtaatccgtcagggtcgc
1-24tRNA-Met(cat)[97303,97375]
gcctcactagctcattggtagagccgctcgctcataacgtgcaggtacct
ggttcgattccagggtgaggtac
1-25tRNA-Ile(gat)[97381,97455]
gcctgttagcggactggtcgtccgatccaagctgataacttggcgtaagc
ggtgttcgattcaccgagcaggtac
1-26tRNA-Arg(acg)[97549,97621]
gcctctatggtccaacggatatgacgccggtctacggaaccggagatgcg
tgttcgattcgcgctaggggcac
1-27tRNA-Val(gac)[97664,97736]
gtccgtgtagctcaggggtagagcgcctgctcgacacgcaggaggaccga
ggttcgaaacctcgcatggacac
1-28tRNA-Arg(cct)[97910,97983]
gcctctgtagctcaacggacagagcaacgcggtcctaacgcggtggctgg

aggttcgaatcctctcggaggcac
1-30tRNA-Gln(ttg)[98524,98599]
tggggtatggtggcaatctggcagtcccgccggactttgactccggaggt
gcaggttcgagtcctgctaccccatc
1-31tRNA-Arg(tct)[98603,98676]
gcccttgtagctcagtggacagagcggcgagcttctacctcgcgggccgg
gagttcgaatctctccaggggcac
1-32tRNA-Lys(ttt)[98744,98820]
gggccggtatcttagtctggtcaaagaagtggacttttaatccgcgcgcc
gtgggttcgaatcccacccggcccacc
1-33tRNA-Gln(ctg)[126834,126908]
tgctcgttggtgtaactggcaacactacggactctgactccgtcatttta
ggttcgaatcctaagcgagcagcca
1-34tRNA-Asn(gtt)[126915,126990]
tggggtgtccgttaatcaggcaaacgagcggactgttaatccgcccctgc
aggttcgaatcctgccaccccagcca
>KX781992.1 Mycobacterium phage Gabriel, complete genome
1-1tRNA-Ser(gct)[30695,30778]
ggagggtgagcatcaggtgatgcagcgagattgctaatcccgtacggtaa
ccaccccgtgaggttcgaatcctccctccgc
1-2tRNA-Leu(cag)[30971,31046]
gctcccgtagcccaattggcaggaggcaccagattcaggatctgggcagt
gtgagttcgaatctcaccgggagtac
1-3tRNA-Leu(gag)[31166,31240]
gtctctgtaggcaaatcggaaaagccgccatcttgagggggtggtgcgtg
cgggttcgactcccgccagagacac
1-4tRNA-Leu(caa)[31241,31314]
gccgtggtaggccatctggcgagccgccgagttcaagtttcggtgtttgc
gggttcgaatcccgcccacggtac
1-5tRNA-SeC(tca)[66734,66830]
attctggcactggtgggcgagcccaccggcggcagcttcaagctgtcgct
ggccggagaaccgaccggaaacatcccgttcaacgcgacccagggcc
1-6tRNA-Pro(tgg)[90698,90770]
cggggtgtagttcagtggaagagcgcttggtttgggaccaagatgtcgca
ggttcgaatcctgtcaccccgac
1-7tRNA-Trp(cca)[90787,90862]
tggggtgaagccgatctggaaggcagcggtctccaaagccgtctcatagc
gggttcgaatcccgtcacccctgcca
1-8tRNA-Tyr(gta)[90864,90950]
gccgcacatgcccaactggtgtttgggagcaggctgtaaccctgtggcct
tcgggacggtgaggttcgattcctcagtgcggtacca
1-9tRNA-Pyl(cta)[92059,92131]
gcaccatttgctcaatggcagagcggcggcttctaaaaccgtgagtgccg
gttcgactccggcatggtgcacc

1-10tRNA-Met(cat)[92281,92355]
agcggtgtagagcagctaggtagctcgccgggctcataacccggaggacg
cgtgttcgaatcacgccaccgccac
1-11tRNA-Cys(gca)[92482,92553]
gcgcctttggcggaatggctacgtgctcggctgcaacccgagttatcccg
gttcgactccgggagggcgctc
1-12tRNA-Glu(ctc)[92558,92632]
ggtccgttggagtagatggatatctcgccaccctctcaaggtggagatca
cgggttcaagtcccgtacggactgc
1-13tRNA-His(gtg)[92634,92707]
gtggccgtagttcagccggtagaacgctgggttgtgatcccagtcgtcga
gggttcgagtccctccggtcaccc
1-14tRNA-Ala(tgc)[92869,92942]
gggcctatagctcatctggtagagcgcctgccttgcaagcaggaggcggc
aggttcaagtcctgttaggtccac
1-15tRNA-Phe(gaa)[93132,93204]
gccgtcatagctcagttggtagagcactggcctgaaaacccagtggccga
ggttcgattcctcgtgtcggcac
1-16tRNA-Val(cac)[93210,93283]
gtccgtttagctcagctggaagagcgctcggtccacacccgagaggccgc
aggttcgatccctgcaatggacac
1-17tRNA-Lys(ctt)[93402,93474]
gccttcgtagctcagtggtagagctgtcgcctcttaagcgataggtcgtt
ggttcgaatccagccgggggcac
1-18tRNA-Glu(ttc)[93479,93555]
ggtcgggtcggtctgctggtatggccagtcggattttcactccggacatt
cgcgggttcaattcccgtcccgatcgc
1-19tRNA-Gly(tcc)[93635,93707]
gcgggtgtggccgaatggctcaggcaccagatttccactctggctacgca
ggttcgattcctgtcatccgctc
1-20tRNA-Thr(cgt)[93767,93841]
gctgctgtagctcacctggcagagcgtcggcgtcgtatcccgaaggcatc
cggttcgagtccggacagcagcccc
1-21tRNA-Thr(tgt)[93842,93914]
gcctctgtggtccagcggcacggacatccgccttgtaagcggaggacccc
cgttcgatccggggtagaggctc
1-22tRNA-Thr(ggt)[94322,94394]
gctgggttagctcagtggtagagcgttcctctggtatgggaaaggcccgg
ggttcaatcccccgattcagctc
1-23tRNA-Gly(gcc)[95717,95790]
gcgaaggtagctcagctggcagagcgccaccttgccaaggtggaggtcgc
gggatcgtaacccgttcttcgctc
1-24tRNA-Asp(gtc)[95794,95866]
ggccctgtagctcagaggaagagcgccggtctgtcgaatcggaggtcgcg

gtatcgtaatccgtcagggtcgc
1–25tRNA–Met(cat)[95926,95998]
gcctcactagctcattggtagagccgctcgctcataacgtgcaggtacct
ggttcgattccagggtgaggtac
1–26tRNA–Ile(gat)[96004,96078]
gcctgttagcggactggtcgtccgatccaagctgataacttggcgtaagc
ggtgttcgattcaccgagcaggtac
1–27tRNA–Arg(acg)[96172,96244]
gcctctatggtccaacggatatgacgccggtctacggaaccggagatgcg
tgttcgattcgcgctaggggcac
1–28tRNA–Val(gac)[96287,96359]
gtccgtgtagctcaggggtagagcgcctgctcgacacgcaggaggaccga
ggttcgaaacctcgcatggacac
1–29tRNA–Arg(cct)[96533,96606]
gcctctgtagctcaacggacagagcaacgcggtcctaacgcggtggctgg
aggttcgaatcctctcggaggcac
1–31tRNA–Gln(ttg)[97147,97222]
tggggtatggtggcaatctggcagtcccgccggactttgactccggaggt
gcaggttcgagtcctgctaccccatc
1–32tRNA–Arg(tct)[97226,97301]
gcccttgtagctcagtggacagagcggcgagcttctacctcgcgggccgg
gagttcgaatctctccaggggcacca
1–33tRNA–Gln(ctg)[124821,124895]
tgctcgttggtgtaactggcaacactacggactctgactccgtcatttta
ggttcgaatcctaagcgagcagcca
1–34tRNA–Asn(gtt)[124902,124977]
tggggtgtccgttaatcaggcaaacgagcggactgttaatccgcccctgc
aggttcgaatcctgccaccccagcca
>KX664695.2 Escherichia phage ESCO5, complete genome
1–1tRNA–Met(cat)[57159,57233]
ggccctgtagctcaatgggagagctgtcggctcataaccgataggtagct
ggatcgaaaccagccagggccacca
1–2tRNA–Arg(tct)[57236,57310]
gtctttatggtgtaatggatagcacaggagtcttctaaactcttagtcaa
ggttcgattccttgtaaagacacca
1–3tRNA–Ser(gct)[57641,57726]
ggtagattggtgaaatggtagccacgacagtttgctaaactgtagtcgga
aacggcgtgtaggttcgagtcctacatctaccgcca
1–4tRNA–Asn(gtt)[57951,58036]
gatgagttggctgaatggtttaagcggcggactgttaatccgtgttcgaa
agaacaatataggttcgaatcctatactcatcgcca
1–5tRNA–Thr(tgt)[58127,58201]
gctcctatagtataatggctattacaacggttttgtaatccgtggatctc
tgttcgattcagtgtgggagcacca

1-6tRNA-Gly(tcc)[58516,58589]
gcgttcttggtgtagcggtagcattccgtccttccaagtcggcggcacga
gttcgatcctcgtagaacgctcca
1-7tRNA-Gln(ttg)[58688,58763]
tggtttgtagtgtagtccggtagcacacgacactttgactgtcgtaggtt
tggttcaaatccagacagaccagcca
1-8tRNA-Pro(tgg)[58857,58933]
cgggacgtagcacagcttggtagtgcgctcgctttgggagcgagaggtcg
agtgttcgaatcactccgtcccgacca
1-9tRNA-Ile(gat)[58940,59013]
tgtttcgtagttcaatggtagaacccacgactgataatcgtgagacacaa
gttcgattcttgtcgaaacaacca
1-10tRNA-Met(cat)[59092,59170]
agcgggatagaggagtctggtcgtcctcgccagtttcatatgctggagat
cattggttcaaatccaattcccgcctcca

>KX879627.1 Campylobacter phage vB_CjeM_Los1, complete genome
1-1tRNA-Tyr(gta)c[38402,38487]
gtaagcatatgggtaattggtaacccaccagactgtaaatctggcgtctt
ttggcattgatggttcaaatccatcctcctacacca
1-2tRNA-Arg(tct)c[38498,38574]
ctccatgtagctcagctggatagagcaagaatcttctaagttctaggtcg
ggtgttcaaatcactccatggaggcca
1-3tRNA-Asn(gtt)c[38584,38658]
tgcggattagcacagtggtagtgcaatcgactgttaatcgatgggtcata
ggttcgaatcctatatccgcagcca
1-4tRNA-Met(cat)c[38752,38826]
gggcgagtagctcaatggtagagcaaccggctcataaccggttggttata
ggttcgattcctatttcgcccacca

>KY652726.1 Klebsiella phage vB_KpnM_BIS47, complete genome
1-1tRNA-Met(cat)c[42726,42801]
tgcgggttagaactcagggagttcatcggtctcataagccgaacgaagag
cagttcgagtctgctacccgcatcca
1-2tRNA-Phe(gaa)c[43296,43370]
gcagagatagctgagacggattagcgcttgcctgaagagcttgagaggaa
ggttcgataccttctctctgcacca
1-3tRNA-Leu(tag)c[43377,43462]
gggcaagtggcggaattggtagacgcactggatttaggttccagcgccct
tgcggtgtgagagttcgagtctctccttgcccacca
1-4tRNA-Arg(acg)c[43710,43782]
gtgggtgtcctgacggacaggcaacggtctacgaagccgtttgatgtagg
ttcgagtccttccatccacacca
1-5tRNA-Pro(tgg)c[43842,43918]
ctctctttagctcagtttggtagagcatctgctttgggagcagagggtcg
catgttcaagtcgtgcaagggagacca

1-6tRNA-Asp(gtc)c[44060,44135]
ggggatatggtattagcggcaaacatactggcctgtcacgccggagtcac
gggttcgaatcccgttatcctcgcca
1-7tRNA-Asn(gtt)c[44143,44217]
ggattggtagctcagaggcagagcgctcgcctgttaagcgaggggtcggg
atttcgaaactcccccttccgcca
1-8tRNA-Lys(ttt)c[44278,44353]
gggtaggtagccgagcggtcaggcattcgccttttaagcgaagagacgat
gggttcaaatccctcccttcccacca
1-9tRNA-Ser(tga)c[44362,44450]
ggagagcaggctgcaaggtgcagacaccggtttgaacccggtcccaccgt
agcgatacggtgacagtccgattctgttgctctcttcca
1-10tRNA-Ile(gat)c[44805,44880]
gctctgatagttcagttggttagaacaggcgaccgataatcgccaaacac
tggttcgagtccagttcggagtacca
1-11tRNA-Ser(gct)c[44887,44972]
ggaagattggtgaaatggtagccacagcggtttgctaaaccgccatccga
aagggtgtgcaagttcaacccttgcatcttccgcca
1-12tRNA-Ser(gga)c[45134,45224]
ggtggaatggtcgagaggtttaagacagcaccttggaaaggtgtaggccc
ccaaaaggggtccgtaggttcgaatcctacttccaccgcca
1-13tRNA-Gln(ttg)c[45419,45494]
agggagatggtgtaattggttagcacacgggtctttgaaatccgtagttc
aggttcaagtcctgatctccctgcca
1-14tRNA-Glu(ttc)c[45847,45922]
gctcctatcggctaaatggacaggccgtcagactttcaatctggaaatcc
gagttcgatcctcggtgggagtacca
1-15tRNA-Gly(tcc)c[46005,46081]
gcgttattagtgttagcggtccagcactccgtccttccaagtcggaagca
tcggttcgaatccgatataacgctcca
1-16tRNA-Ile(gat)c[46172,46247]
gggagtatagctcagtcagtagagcgctcgaccgataatcgagaggtcgc
aggagcaaagcctgctactcccacca
1-17tRNA-Tyr(gta)c[46254,46337]
ggctcgttggcagaatggttattgcagcggattgtaaatccgtgcccttc
ggggttcctggttcgagtccagggtgggccacca
1-18tRNA-Cys(gca)c[46776,46849]
gcacggttgctggagtggcaacagcttcggctgcaaaccgaatgtcaggt
gttcgattcacctaccgtgctcca
1-19tRNA-Leu(taa)c[47055,47132]
tgcgtccttggtccaatctggcagaggcatgggtgttaagctcccagggt
tcccggttcgagtccgggagggcgcatc
1-21tRNA-Thr(tgt)c[47490,47565]
gctcgtatagctcagttggtagagcaactcccttgtaaggagaaggtcgt

gggttcaagtcctactgcgagcacca
1-22tRNA-Lys(ctt)c[49792,49867]
ggggtattaacacagcggttagtgtagcggccccttaagccgaaggtcga
aggttcgaatccttcatgccccacca
1-23tRNA-Met(cat)c[50333,50410]
ggccctcaagctcattaggtatgagcaatcgactcataatcgagaggtag
gcaggttcgaatcctccgggggccacca
1-24tRNA-Met(cat)c[50414,50486]
cgttcagtagctcagttggtagagcaggttcttcatacggacaaggtcag
tggttcaattccactctgaacga
>KY000003.1 Salmonella phage STP07, complete genome
1-1tRNA-Met(cat)[3339,3413]
ggtcctgtagctcagtggtagagcagccccctcataagggattggtcgct
ggttcaagtccagccagggtcacca
1-2tRNA-Asn(gtt)[3826,3901]
gacgatgtagttcagtcggtagaacggcggtctgttaaatcgtatgtcgc
aggttcaagtcctgccatcgtcgcca
1-3tRNA-Ser(act)[4569,4657]
ggaaggttgcccgagaggtttaagggactcgactactaatcgagtggggc
ttttagcccccgaaggttcgaatccttcaccttccacca
>KX961632.1 Bacillus phage SBP8a, complete genome
1-1tRNA-Asn(gtt)[32688,32761]
gtgcttgtagctcagtcggtagagctggtggctgttaaccactgtgtcgt
aggttcgattcctacctagcacgc
1-2tRNA-Glu(ttc)[32768,32844]
gtcgcattggtgaaattggttaacacactcggctttctaccgaggattta
ggggttcgagccccctatgcgattcca
1-3tRNA-Asp(gtc)[32849,32922]
tggggatatagtgtagaggtaaacacgcacggctgtctaccgtgaagcac
gagttcgaatctcgttatcctcgt
>KX961629.1 Bacillus phage BJ4, complete genome
1-1tRNA-Asn(gtt)[32254,32327]
gtgcttgtagctcagtcggtagagctggtggctgttaaccactgtgtcgt
aggttcgattcctacctagcacgc
1-2tRNA-Glu(ttc)[32334,32410]
gtcgcattggtgaaattggttaacacactcggctttctaccgaggattta
ggggttcgagccccctatgcgattcca
1-3tRNA-Asp(gtc)[32415,32488]
tggggatatagtgtagaggtaaacacgcacggctgtctaccgtgaagcac
gagttcgaatctcgttatcctcgt
>KX961630.1 Bacillus phage QCM8, complete genome
1-1tRNA-Cys(gca)[26999,27071]
gaggatgtaccgaagcggctaacggtctagactgcaaatctagtgttcat
gggttcgagtcccatcattctct

1−2tRNA−Asn(gtt)[27086,27161]
gtgcctgtagctcagtcggtagagcaaaaccctgttaaggttgaggtcgt
aggttcgagccctaccaggtacgcca
1−3tRNA−Gly(tcc)[27354,27429]
ggggcattagtatattggttcattattcctggcttccaaccaggggaggt
cggttcgactccgacatgtccctcca
1−4tRNA−Asp(gtc)[27434,27506]
tggggtattagtttagtggtaaaatactgcactgtctatgcagagtcagg
ggttcgactcccctatacctcgt
1−5tRNA−Glu(ttc)[27623,27698]
tgtcccgttggtgaaattggctaacacactcggctttctaccgagcattc
gcaggttcgaatcctgtacgggatat
1−6tRNA−Gln(ttg)[27822,27894]
tttcggagtagccaagtggtaaggcaatagactttgactctatgatcggt
ggttcgataccatcctccgaagt
1−7tRNA−His(gtg)[28051,28123]
cagggtgtggtgaagtggttaacacgggcgattgtggctcgtccattcga
gggttcgattccctcctccctga
1−8tRNA−Pro(tgg)[28131,28203]
gtgggtgtaggctagaggtcagtcactgcgtttgggacgcaggatacgca
cgttcgaatcgtgccacccgcac
1−9tRNA−Phe(gaa)[28308,28380]
ggacggatagctgagatggattagcgaaggattgaaaccccttagaggtt
ggatcgttaccaactctgtccac
1−10tRNA−Tyr(gta)[28506,28588]
gggtcagtcgcatagtggcaattgcaggagactgtaaatctcccccgaaa
ggttcgttggttcgagtccaacctgacccacca
1−11tRNA−Leu(tag)[28594,28679]
tgccgaagtggtggaattggtatacacgcagcacttaggatgctgtgccc
tagaggattgagggttcgacccccctccttcggtatc
1−12tRNA−Trp(cca)[28717,28789]
tggggggtttagtttagtggtaaaattttggtctccaacaccaaagtccaa
ggttcgaatccttgcgccctcgt
1−13tRNA−Met(cat)[28796,28868]
aggagcatagctcagtggtagtagcgggtgtctcatacgcaccaggtcga
aggttcgaatccttctgttccta
>KY630163.1 Salmonella phage S8, complete genome
1−1tRNA−Ser(gct)c[97599,97687]
ggaaggttgcccgagaggtttaagggactcgactgctaatcgagtggggc
ttttagcccccgaaggttcgaatccttcaccttccgcca
1−2tRNA−Tyr(gta)c[97764,97847]
gtgagtgtggcagagcggtcgaatgcaggagactgtaaatctccccgtaa
cagcgcggtggttcaaatccatccactcacacca
1−3tRNA−Asn(gtt)c[98605,98680]

gacgatgtagttcagtcggtagaacggcggtctgttaaatcgtatgtcgc
aggttcaagtcctgtcatcgtcgcca
1-4tRNA-Met(cat)c[99093,99168]
ggccccgtagctcagtggttagagcagtcgactcataatcgattggtcgc
tggttcaagtccagccagggtcacca
>KX831080.1 Mycobacterium phage Lukilu, complete genome
1-1tRNA-Ser(gct)[30369,30452]
ggagggtgagcatcaggtgatgcagcgagattgctaatcccgtacggtaa
ccaccccgtgaggttcgaatcctcctccctccgc
1-2tRNA-Leu(cag)[30645,30730]
gcctcggtgacgtaatggaagccgtgctgcactcagactgcagtgtccga
aagggcgtgagggttcgactccctcccgaggtacca
1-3tRNA-Leu(gag)[30732,30805]
gcccctgtatcccaacggcagaggaagcggtctgagaggccgttcagtga
gggttcgaatccctctgggggcac
1-4tRNA-Leu(caa)[30806,30880]
gccgcagtatgcaaaccggaaaagcagccgtactcaaaatgcggtgcgtg
cgggttcgatccccgcctgcggtac
1-5tRNA-SeC(tca)[68732,68828]
attctggcactggtgggcgagcccaccggcggcagcttcaagctgtcgct
ggccggagaatcgaccggaaacatcccgttcaacgcgacccagggcc
1-6tRNA-Pro(tgg)[92494,92568]
cggggtgtagttcagtttggaagagcgcttggtttgggaccaagatgtcg
caggttcgaatcctgtcaccccgac
1-7tRNA-Trp(cca)[92583,92653]
gggtctgtgcacagggtgcccgacggtctccaaagccgaaggcgggggtt
cgattccctccaggcctgcca
1-8tRNA-Tyr(gta)[92655,92741]
cccgtacatgcccaactggtgtttgggagcaggctgtaaccctgtggcct
tcgggacggtgaggttcgattcctcagtgcgggacca
1-9tRNA-Pyl(cta)[93855,93927]
gcaccatttgctcaatggcagagcggcggcttctaaaaccgtgagtgccg
gttcgactccggcatggtgcacc
1-10tRNA-Met(cat)[94077,94151]
agcggtgtagagcagctaggtagctcgccgggctcataacccggaggacg
cgtgttcgaatcacgccaccgccac
1-11tRNA-Cys(gca)[94278,94349]
gcgcctttggcggaatggctacgtgctcggctgcaacccgagttatcccg
gttcgactccgggagggcgctc
1-12tRNA-Glu(ctc)[94354,94428]
ggtccgttggagtagatggatatctcgccaccctctcaaggtggagatca
cgggttcaagtcccgtacggactgc
1-13tRNA-His(gtg)[94430,94503]
gtggccgtagttcagccggtagaacgctgggttgtgatcccagtcgtcga

gggttcgagtccctccggtcaccc
1-14tRNA-Ala(tgc)[94665,94739]
gggcctgtagctccaattggtagagcagcatccttgcaagatgacggctg
tcggttcgaatccgacctggtccac
1-15tRNA-Phe(gaa)[94929,95001]
gccgtcatagctcagttggtagagcactggcctgaaaacccagtggccga
ggttcgattcctcgtgtcggcac
1-16tRNA-Val(cac)[95007,95080]
gtccgtttagctcagctggaagagcgctcggtccacacccgagaggccgc
aggttcgatccctgcaatggacac
1-17tRNA-Lys(ctt)[95200,95273]
gccttcgtagctcagttggtagagctctcgcctcttaagcgagatgtcgc
aggttcgacccctgccggaggcac
1-18tRNA-Glu(ttc)[95278,95354]
ggtcgggtcggtctgctggtatggccagtcggattttcactccggacatt
cgcgggttcaattcccgtcccgatcgc
1-19tRNA-Gly(tcc)[95434,95506]
gcgggtgtggccgaatggctcaggcaccagatttccactctggctacgca
ggttcgattcctgtcatccgctc
1-20tRNA-Thr(cgt)[95566,95640]
gctgctgtagctcacctggcagagcgtcggcgtcgtatcccgaaggcatc
cggttcgagtccggacagcagcccc
1-21tRNA-Thr(tgt)[95641,95713]
gcctctgtggtccagcggcacggacatccgccttgtaagcggaggacccc
cgttcgatccggggtagaggctc
1-22tRNA-Thr(ggt)[95773,95845]
gctgggttagctcagtggtagagcgttcctctggtatgggaaaggcccgg
ggttcaatcccccgactcagctc
1-23tRNA-Gly(gcc)[97168,97241]
gcgaaggtagctcagctggcagagcgccaccttgccaaggtggaggtcgc
gggatcgtaacccgttcttcgctc
1-24tRNA-Asp(gtc)[97245,97317]
ggccctgtagctcagaggaagagcgccggtctgtcgaatcggaggtcgcg
gtatcgtaatccgtcagggtcgc
1-25tRNA-Met(cat)[97377,97449]
gcctcactagctcattggtagagccgctcgctcataacgtgcaggtacct
ggttcgattccagggtgaggtac
1-26tRNA-Ile(gat)[97455,97529]
gcctgttagcggactggtcgtccgatccaagctgataacttggcgtaagc
ggtgttcgattcaccgagcaggtac
1-27tRNA-Arg(acg)[97623,97695]
gcctctatggtccaacggatatgacgccggtctacggaaccggagatgcg
tgttcgattcgcgctaggggcac
1-28tRNA-Val(gac)[97738,97810]

gtccgtgtagctcaggggtagagcgcctgctcgacacgcaggaggaccga
ggttcgaaacctcgcatggacac
1-29tRNA-Arg(cct)[97984,98057]
gcctctgtagctcaacggacagagcaacgcggtcctaacgcggtggctgg
aggttcgaatcctctcggaggcac
1-31tRNA-Gln(ttg)[98598,98673]
tggggtatggtggcaatctggcagtcccgccggactttgactccggaggt
gcaggttcgagtcctgctaccccatc
1-32tRNA-Arg(tct)[98677,98752]
gcccttgtagctcagtggacagagcggcgagcttctacctcgcgggccgg
gagttcgaatctctccaggggcacca
1-33tRNA-Gln(ctg)[126638,126712]
tgctcgttggtgtaactggcaacactacggactctgactccgtcatttta
ggttcgaatcctaagcgagcagcca
1-34tRNA-Asn(gtt)[126719,126794]
tggggtgtccgttaatcaggcaaacgagcggactgttaatccgcccctgc
aggttcgaatcctgccaccccagcca
>KX828711.1 Shigella phage SH7, complete genome
1-1tRNA-Arg(tct)c[67336,67411]
cggggcatagctcagaaggaagagcaaggaccttctaagtcctaggtcgt
aggttcgatccctactgcctcgacca
1-2tRNA-His(gtg)c[67416,67491]
gtggccgtagttcagttggtagaactcgagattgtgattctcgtagtcat
gggttcgactcccatcggtcacccca
1-3tRNA-Asn(gtt)c[67606,67680]
ggatgtgtagctcaatggcagagcgatcgcctgttaagcgattggttata
ggttcgaatcctatcacgtccgcca
1-4tRNA-Tyr(gta)c[67685,67771]
ggggagttatcccgtagaggtagcggtgtggactgtaaatccattgtcat
tgcgactcgggtggttcgactccatcactccccacca
1-5tRNA-Met(cat)c[67785,67859]
ggccctgtagctggaaggttcaagcaagcgactcataatcgccagatggt
ggttcaattccacctagggccacca
1-6tRNA-Thr(tgt)c[67861,67936]
gctgatttagctcagtaggtagagcaactcacttgtaatgagaaggtcgg
cggttcgattccgtcaatcagcacca
1-7tRNA-Ser(tga)c[67942,68031]
ggaggcgtggcagagtggtttaatgcaccggtcttgaaaaccggcagtcg
ctccggcgactcataggttcaaatcctattgcctccgcca
1-8tRNA-Pro(tgg)c[68033,68107]
ctccgtgtagctcagtttggtagagcgcctgatttgggatcaggaggtcc
aaggttcaaatccttgtatggagac
1-9tRNA-Gly(tcc)c[68118,68191]
gcggatatcgtataatggcattacctcagacttccaatctgatgatgtga

gttcgattctcattatccgctcca
1-10tRNA-Leu(taa)c[68197,68283]
gcgagaatggtcaaattggtaaaggcacagcacttaaaatgctgcggaat
gatttccttgtgggttcgagtcccacttctcgcacca
1-11tRNA-Gln(ttg)c[68284,68357]
tgggaattagccaagttggtaaggcactggattttgattccaggatgcaa
aggttcgagtcctttattcccagc
>KY000079.1 Acinetobacter phage AM24, complete genome
1-1tRNA-Tyr(gta)[23966,24047]
gtagtagtgcccgagtggtcgaagggggtggactgtaaattcactatatc
acacgttggttcgaatccaacctgctacacca
1-2tRNA-Ile(gat)[24492,24566]
agtaccttagttcagtggtagaacgcagaactgataattctgaggtcgat
ggttcgaaaccatcaggtactacca
1-3tRNA-Asn(gtt)[24708,24781]
tctagtgtagcacaacggtagtgcagcagattgttaatctgttggttgtt
ggttcgaatccagcctctagagcc
1-4tRNA-Asp(gtc)[25572,25647]
ggctctgtagcatagtggattatgcgactccctgtctaggagaaaaacgc
cagttcgagtctggtcagagtcgcca
1-5tRNA-Gln(ttg)[25652,25726]
tgtcgtatggtgtaatggttagcacagaagactttgactcttctagttta
ggttcgagtcctaattcgactgcca
1-6tRNA-Ala(tgc)[25730,25801]
gggtcactagcacaatggtagtgcagttgctttgcaagcatcaggttaag
ggttcgagtcccttgtgtatcc
1-7tRNA-Glu(ttc)[25808,25882]
attcctatcgaacaatggttagttcgctggactttcactccagaaatctc
ggttcaaatccgtgtaggaatacca
1-8tRNA-Lys(ttt)[25885,25961]
aggacgttagctcagttggttagagcaagagccttttaagttcgaggtca
taggttcaaatcctatacgtcctacca
1-9tRNA-Phe(gaa)[26316,26390]
gcacaagtagcttagatggattagcgtaaccctgaaaaggttgagaggtt
ggatcgttaccaacattgtgcacca
1-10tRNA-Val(tac)[26601,26676]
gattccttagctcaattggtagagcatttcccttacaaggaagaggtcgt
aggttcgaaacctacaggaatcacca
1-11tRNA-Ser(tga)[26680,26770]
ggaggaataatccgagttggcctaacggatgctgtcttgaaaacagttag
gtgtaatagccgtgtgagttcgagtctcactttctccgcca
1-12tRNA-Gly(tcc)[26772,26845]
gcgtctatagtgtagtggtaacaccctgtacttccaattcagtatcttcg
gttcgaatccgaatggacgctcca

1-13tRNA-Pro(tgg)[27000,27076]
ctctgtgtagctcagtttggtagagcactcgctttgggagtgaggggtcg
taagttcaattcttactacagagacca
1-14tRNA-Leu(tag)[27083,27159]
gggtagttgaagggaatggcatacctttcacgcttagaacgtgaagtttc
tgagttcgagtctcagactacctacca
1-15tRNA-Leu(taa)[27166,27241]
ggggatatagcccaacggcagaggcaagaggtttaaaccctctcaagttt
cagttcgagtctgaatatccctacca
1-16tRNA-Thr(tgt)[28132,28206]
tccgaattagctcaatggtagagcagcgcttttgtaatgcgaaggttgtg
tgttcgactcacatattcggaacca
1-17tRNA-Met(cat)[28212,28287]
ggtcctttagcacatactggtctgtgcaccatgctcataacgtggaggat
tttggttcgattccaaaaaggaccac
1-18tRNA-Met(cat)[28463,28537]
agcgaggtagtgtaacggtttgcacaggggactcataatcctctggtttg
ggttcgaatcccaacttcgcttcca
>KY000080.1 Klebsiella phage KPV15, complete genome
1-1tRNA-Thr(tgt)c[77431,77506]
gccgatttagctcagctggtagagcgcttcacttgtaatgaagatgtcgc
gggttcgactcctgcaatcggcacca
1-2tRNA-Leu(taa)c[77607,77691]
gcatcgatggtggaactggtatacacaggagacttaaaatctcccgccgc
aaggattgagggttcgaatccctctcggtgcacca
1-3tRNA-Arg(tct)c[77696,77772]
cggggcatagctcagttggatagagcagcggacttctaatccgcaggtcg
aaggttcgaatccttctgcctcgacca
1-4tRNA-Met(cat)c[79153,79229]
ggccctgtagctcaattggtagagcgttcccctcataagggattggttgc
atgttcgagtctttgccagggtcacca
1-5tRNA-Pro(tgg)c[80497,80573]
ctccgtatagctcagtctggtagagcgctccatttgggatggagaggtcg
aatgttcgagtcattctatggagacca
1-6tRNA-Gly(tcc)c[80579,80653]
gcatccatcgtatagcggatattatgtctggcttccacccagaagatagg
agttcgattctccttggatgctcca
1-7tRNA-Trp(cca)c[80659,80733]
aggttcttagtataacggctattatgctgggctccaaacccagtgatgag
ggttcgattccttcagggcctgcca
1-8tRNA-Ile(gat)c[80740,80815]
gggagtatagctcatttggtagagctctcgaccgataatcgagcggtgac
tggttcgagtccagttactcccacca
1-9tRNA-Ser(tga)c[80882,80966]

ggagagtagcgctagtggtagcaaaccggacttgaaatccgggccatcgg
aaacggtgagggttcaactcctttactctccgcca
1–10tRNA–His(gtg)c[81064,81139]
gtggccgtagttcagttggtagaactcgagattgtgattctcgtagtcat
gggttcaactcccatcggtcacccca
1–11tRNA–Gln(ttg)c[81235,81309]
tgaatcatagccaagttggtaaggcagtaggttttgatcctacgatccct
ggttcgagtccaggtggttcagcca
1–12tRNA–Met(cat)c[81316,81392]
tgcggggtaacttcagttggtagaatgttgggctcatatcccgacacgcg
caggttcgagtcctgcctccgcctcca
1–13tRNA–Asp(gtc)c[81554,81629]
ggacctatagtttcagcggttaaaatactcgcctgtcacgcgagagtcac
ggattcgaattccgttaggtccgcca
1–14tRNA–Asn(gtt)c[81883,81967]
gggtcgttggctgagagggtaagcgacggactgttaatccgtgtcagaaa
tgactaggcaggttcgatacctgcacggcccgcca
1–15tRNA–Lys(ttt)c[81973,82049]
gggatactagctcagttggttagagcaccggacttttaatccgggtgtcc
gaagttcgagtcttcggtgtcccacca
1–16tRNA–Tyr(gta)c[82056,82142]
ggggagttagaccgtaggggtagcgggacagactgtaaatctgttgctca
aaaggctcgagtggttcgactccattactccccacca
>KY000083.1 Pseudomonas phage PA10, complete genome
1–1tRNA–Gln(ttg)[67563,67636]
tgccgcttcgttcaatggtaggacgccagactttgaatctggagatgatg
gttcgatcccatcagcggctgcca
1–2tRNA–Arg(tct)[67942,68017]
gctcgtatagtgtaatggatagcacaacggtcttctaagccgtaaggtct
aggttcgaatcctagtatgagcgcca
1–3tRNA–Lys(ttt)[68028,68103]
tggacggaagctaaagtggataggcatcgggcttttaacccgactatagt
gagttcgagtctcacccgttcaacca
1–4tRNA–Leu(tag)[68366,68450]
ggccctgtggtggaattggtatacacatcagtcttagaaactgacgccga
gaggattgagggttcaagtccctccggggccacca
1–5tRNA–Ile(gat)[68661,68736]
ggccagatagctcaattggtagagcacccgaccgataatcgggtggttga
aggttcaagtccttctctggccacca
1–6tRNA–Asp(gtc)[68746,68824]
ggcccattagctcagtctggactagagcaagcccctgtctagggcaaggt
cgtcggttcgaatccgacatgggtcgcca
1–7tRNA–Met(cat)[68925,68999]
agcgttaagactggctggcgctgtcactcggctcataaccgaatacaagt

ggttcgattccacctctcgcttcca
1-8tRNA-Cys(gca)[69234,69309]
cccgcgttggccgagaggtttaggcggcggattgcaaatccgtctcacat
cggttcaaatccgatacgcggctcca
1-9tRNA-Asn(gtt)[69320,69395]
tgggatgtagctcagttggtagagcagggagctgttaactctcaggtcgc
aggttcgagccctgccgtcccagcca
1-10tRNA-Pro(tgg)[69459,69536]
ctcctcgtagctcagtctggttagagtgtcggatttggaatccgaaggtc
gaaggttcaaatccttccggggtgacca
1-11tRNA-Gly(tcc)[69543,69618]
gcgggtatagctcagttggtagagcgtctgccttccaagcagttcgtcgt
cggttcgagtccgtctatccgctcca
1-12tRNA-Phe(gaa)[69625,69701]
gggactgtagctcagaaggttagagcggtggattgaaatcccacaggtcg
ggcgttcgattcgccccggtcccacca
1-13tRNA-Glu(ttc)[69708,69783]
gcagttatagattaattggttaaatcgccagactttcaatctggtgttcc
gggttcgatccccggtaactgctcca
1-14tRNA-Thr(tgt)[70105,70179]
gccctttaagcatttatggtgatgcaccggctttgtaacccggcgaattc
tgttcaagtcaggaatggggcacca
>KY073228.1 Pseudomonas phage Zigelbrucke, complete genome
1-1tRNA-Gln(ttg)[21733,21806]
tgccgcttcgttcaatggtaggacgccagactttgaatctggagatgatg
gttcgatcccatcagcggctgcca
1-2tRNA-Arg(tct)[22112,22186]
gctcgtatagtgtaatggatgcacaacggccttctaagccgtaaggtcta
ggttcgagtcctagtacgagcgcca
1-3tRNA-Lys(ttt)[22196,22271]
tggacggaagctaaagtggataggcatctggcttttaaccagactatagt
gagttcgagtctcacccgtccaacca
1-4tRNA-Leu(tag)[22546,22630]
ggccctgtggtggaattggtatacacatcagtcttagaaactgacgccga
gaggattgagggttcaagtccctccggggccacca
1-5tRNA-Ile(gat)[22839,22914]
agccggatagctcaattggtagagcacccgaccgataatcggatggttga
aggttcaagtccttctctggctacca
1-6tRNA-Asp(gtc)[22924,23002]
ggcccattagctcagtctggactagagcaagcccctgtctagggcaaggt
cgccggttcgaatccggcatgggtcgcca
1-7tRNA-Met(cat)[23103,23177]
agcgttaagactggctggagctgtcactcggctcataaccgagcacaagt
ggttcgattccacctctcgctacca

1−8tRNA−Cys(gca)[23457,23532]
cccgcgttggccgagaggtttaggcggcggattgcaaatccgtctcacat
cggttcaaatccgatacgcggctcca
1−9tRNA−Asn(gtt)[23543,23618]
tgggatgtagctcagttggtagagcagggagctgttaactctcaggtcgc
aggttcgagccctgccgtcccagcca
1−10tRNA−Pro(tgg)[23682,23759]
ctcctcgtagctcagtctggttagagtgtcggatttggaatccgaaggtc
gaaggttcaaatccttccggggtgacca
1−11tRNA−Gly(tcc)[23766,23841]
gcgggtatagctcagttggtagagcgtctgccttccaagcagttcgtcgt
cggttcgagtccgtctatccgctcca
1−12tRNA−Phe(gaa)[23848,23924]
gggactgtagctcagaaggttagagcggtggattgaaatcccacaggtcg
ggcgttcgattcgccccggtcccacca
1−13tRNA−Glu(ttc)[23931,24006]
gcagttatagattaattggttaaatcgccagactttcaatctggtgttcc
gggttcgatccccggtaactgctcca
1−14tRNA−His(gtg)[24067,24141]
gtggagattgtgtagcccggatgctgcctttcggttgtgacccgattgtt
ggggttcgagtccccgcctccaacc
1−15tRNA−Thr(tgt)[24328,24402]
gccctttaagcatttatggtgatgcaccggctttgtaacccggcgaattc
tgttcaagtcaggaatggggcacca
>AP017925.1 Ralstonia phage RP31 DNA, complete genome
1−1tRNA−Asn(gtt)c[128734,128809]
tccgaggtagctcagtcggtagagcagcggcctgttaagccgttggtcgc
tggttcgaacccagcccttggagcca
1−2tRNA−Ser(aga)[267002,267096]
ggtaaaaaagcgctgtggttaccagcgcctcagatagagtctgagctgtt
cagcaaggaacttatccttgatctggttcagcccatagttgacct
>KY549443.1 Enterococcus phage EFP01, complete genome
1−1tRNA−Arg(tcg)[105868,105941]
ttgttaggtcgtctaacaggtaagacacaattcttcggaaattgtgatac
aggttcgaaccctgtcctgacagt
1−2tRNA−Trp(cca)[107475,107548]
tgcgctgttggtgtagtggctatcatttccggctccaacccggaagacgg
gggttcgaatccttcacggcgtgt
1−3tRNA−Thr(tgt)[107690,107763]
gctgatattccgtaattggtaacgggacacacttgtaatgtgtatacttc
taggttcgagtcctagtatcagca
1−4tRNA−Lys(ctt)[107955,108030]
tatctccttagctcagttggtggtagcacctgattcttaatcagggggtc
acaggttcgaatcctgtaggggatat

1−5tRNA−Lys(ttt)[108163,108239]
tgtctccatagctcaatcggctagagcactcgacttttaatcgaggggtt
cggggttcaaatccctgtggggacatc
1−6tRNA−Arg(acg)[108338,108410]
cggcagttcgtccaattggctaggatgctagactacgaatctagtgatag
aggttcgattcctctgctgtcgg
1−7tRNA−Ser(gga)[112142,112228]
ggaaggttactcaaacggtaaagaggcttggttggaaaccaagtagatgt
gtaacagcatgtgtgggttcgaatcccatgccttcca
>KY363465.1 Providencia phage vB_PreS_PR1, complete genome
1−1tRNA−Met(cat)c[28385,28460]
ggttctatagctcagtcggtagagcgtcgctttcatacggcgtgtgtcgc
tggttcgattccagctagaacctcca
1−2tRNA−Gln(ttg)c[28588,28663]
tggggaatggtgtaggtggttagcacatcagcctttgactctgacagcgt
gggttcgaatcccgcttccccttcca
1−3tRNA−Met(cat)c[28670,28752]
agcgagttagagttctgggaactcaggaggctcattccctccggcaatag
ccaagatcggttcgattccggtactcgcaacca
1−4tRNA−Ser(tga)c[29978,30066]
ggtaggagtgcccaatggtgggcagcctgacttgaaatcaggtacgttgt
ggggacacggcgggggttcgattcctcctcctaccgcca
1−5tRNA−Trp(cca)c[30927,31000]
atgggcttcgtatagtggtattaccttggcttccaaacccaacgacgggg
gttcgattcctccagcccgtgcca
1−6tRNA−Ser(gga)c[31761,31849]
agaacaaaccgatcggcgacgggaactgcttggaaagcagctgagtatag
cgatatgccttgtgggttcgactcccacttcttctgcca
1−7tRNA−Leu(tag)c[31859,31935]
gggactatgacggaactggcatacgtgctgtgcttagaacgcagatttta
ggagttcgactctccttagtcccacca
1−8tRNA−Leu(taa)c[32072,32148]
gtcgaggtgacgtaattggcaaacgtacagcacttaaaatgctggttctg
cgggttcgatccccgccctcgatacca
1−9tRNA−Gly(tcc)c[33985,34060]
gcgtgcgtaagatagcggctattcggtcgggcttccaacccgtacagcag
gggttcgagtcccctcgcccgcgcca
1−10tRNA−Asn(gtt)c[35038,35122]
gggtggttagctgagtggtttagcgacggactgttaatccgtgttcgaaa
ggacaacgtaggttcaaatcctacaccgcccgcca
1−11tRNA−Ile(gat)c[35129,35205]
actgcgatagctcagttggtagagcgcttgactgataatcgagaggttcc
ctggttcaaacccaggtcgcagtacca
1−12tRNA−Glu(ttc)c[35508,35585]

actgtagtggacaaactggtaaagtcaccacccctttcaaggtggagcatt
gagggttcgactcccttctacagtgcca
1−13tRNA−Tyr(gta)c[35666,35750]
gcaggattagtctagcggcaatgacaggagactgtaaatcttcacccctc
ggggttccgtggttcgagtccacgatcctgcacca
1−14tRNA−Arg(acg)c[35840,35915]
gcatgcttaactcagtggacagagttcttcgctacgaacgaagtggtcac
aggttcgaatcctgtagtgtgctcca
1−15tRNA−Thr(tgt)c[36098,36173]
gttgacgtagctcagttggtagagcagttgccttgtaagcatcaggtcgc
aggttcgatgcctgtcgtcagcacca
1−16tRNA−Val(tac)c[36179,36252]
actcgcttagtataacggtagtgctagtcctttacacggactaagcggtg
gttcgattcctccagcgagtacca
1−17tRNA−His(gtg)c[36478,36555]
gtggcggtagcagaattggtttaatgcccccgattgtgattcgggaatgt
gcgggttcaagtcccgtccgtcacccca
1−18tRNA−Phe(gaa)c[36696,36770]
gcaccgttagctgagatggattagcgcaggtttgaagagcctgatagagt
ggttcgataccactatggtgcacca
1−19tRNA−Asp(gtc)c[36776,36852]
aggtatgaggtcgaaatggacaagacaccgcactgtcactgcggagatag
cgggttcgatccccgtcatacctgcca
1−20tRNA−Ala(tgc)c[36859,36934]
ggggatgtagctcaattggtagagcacctgctttgcaagcagacggttag
gggttcaaatccccttttctccacca
1−21tRNA−Pro(tgg)c[36942,37018]
ctcggattagctcagtctggtagagtacgccgtttggggcggtggggtca
aaggttcaaatcctttattcgagacca
1−22tRNA−Ser(gct)c[37477,37570]
ggaagagtaaccctaatggtaagggaactgtttgctaaacagttagtagc
tgtcgaaagacggtgtgcgggttcgagccccgcttcttcctcca
1−23tRNA−Lys(ttt)c[38288,38363]
gcgtcgttagctcaattggcagagcaagggatttttaatcccgaggttgt
aggttcgactcctatacggcgtacca
1−24tRNA−Met(cat)c[40456,40531]
gcaccagtagcttaatggtagagcgtgcccctcataagggcttggttgtg
ggttcgagtccctcctaggtgcacca
1−25tRNA−Arg(tct)c[41390,41464]
gtccccttagtctagaggataggcatcggtcttctaaaccgatatacata
ggttcgaatcctatagggggacgcca
>KT995480.1 Bacillus phage BM15, complete genome
1−1tRNA−Ser(gct)[29057,29145]
ggaagggtactcaagttggtgaagaggtcagtttgctaaactgatagtac

ctattacaggtagcaagggttcgaaccccctttccttcca
1–2tRNA–Cys(gca)[29156,29229]
gagggtgtaccgaagcggctcaacggcacagattgcaaccctgttgttcg
tgggttcaaatcccaccatcctct
1–3tRNA–Asn(gtt)[29275,29350]
gtgcttgtagctcagtcggtagagctggtggctgttaaccactgtgtcgt
tggttcgattccaacctagcacgcca
1–4tRNA–Gly(tcc)[29354,29428]
ggggcattggtatattggctattactcttggcttccaaccaagcaaggtc
ggttcgactccgacatgtccctcca
1–5tRNA–Thr(tgt)[29435,29511]
gccttcttagctcagttggttagagcgactgccttgtaagcagtaggtcg
tgggttcgaatcctacagtcggcacca
1–6tRNA–Asp(gtc)[29517,29590]
ggctcgttagtttaacggtaaaatactgcactgtctatgcagggtcacgg
gttcaactcccgtacgggtcgcca
1–7tRNA–Glu(ttc)[29599,29675]
tgtcgcattggtgaaattggctaacacactcggctttctaccgagcattc
aggggttcgaatcccctatgcgatatc
1–8tRNA–Arg(tct)[29769,29844]
taccccctttagccaagtggactaaggcatcgggcttctatcccgtagatc
gtgggttcgattcctacagggggtgt
1–9tRNA–Ser(tga)[29850,29935]
cggggaatactcaaatggttaagagggtagtcttgaaaactactaggcgt
gaaagcgtgcgggggttcgaatccctcttcctcggc
1–10tRNA–Gln(ttg)[30015,30090]
tttcggtgtggacaaactggtaaagtcgtcaggctttgaccctgaagttt
ggaggttcgacccccttccaccgaagt
1–11tRNA–His(gtg)[30168,30241]
cagtgtgtggtgaagtggcttaacacggacgactgtggatcgttcattcg
caggttcgaatcctgtcatgctga
1–12tRNA–Pro(tgg)[30339,30410]
gtagatgtaggctagaggtcagtcactccgtttggggcggaggtcacgca
ggttcgatccctgccatctgca
1–13tRNA–Phe(gaa)[30513,30587]
ggacagatagctgagacggattagcgattggctgaaaaccaatagaggtt
ggatcgttaccaactctgtccacca
1–14tRNA–Tyr(gta)[30594,30676]
gggcgtgcaatcattggagagataagctgactgtaaatcagtggtcattg
actgtgaaggttcgaatccttccattcccacca
1–15tRNA–Leu(taa)[30678,30764]
atcggaatgttggaattggtagacataacggacttaaaatccgttgctcc
ttggggcgtgagggttcgagtccctcttccgatacca
1–16tRNA–Ile(gat)[30766,30840]

actagtgtagctcagaggtagagcagtgtcttgataaggcattggtcgtt
ggttcgatcccaaccactagtacca
1–17tRNA–Leu(tag)[30850,30934]
gtcagagtgttggaactggtatacatgcggcacttagaatgccgtgcctt
cgggattgtgggttcgactcccacctctgacacca
1–18tRNA–Met(cat)[31029,31103]
tacggttatagctcagtggtagagtgtgggtctcataagcccaaggtcga
tggttcaatcccatctaaccgtatc
>KY290975.2 Escherichia phage YUEEL01, complete genome
1–1tRNA–Arg(tct)c[68828,68903]
cggggcatagctcagaaggaagagcaaggaccttctaagtcctaggtcgt
aggttcgatccctactgcctcgacca
1–2tRNA–His(gtg)c[68908,68983]
gtggccgtagttcagttggtagaactcgagattgtgattctcgtagtcat
gggttcgactcccatcggtcacccca
1–3tRNA–Asn(gtt)c[69098,69172]
ggatgtgtagctcaatggcagagcgatcgcctgttaagcgattggttata
ggttcgaatcctatcacgtccgcca
1–4tRNA–Tyr(gta)c[69177,69263]
ggggagttatcccgtagaggtagcggtgtggactgtaaatccattgtcat
tgcgactcgggtggttcgactccaccactccccacca
1–5tRNA–Met(cat)c[69273,69347]
ggccctgtagctggaaggttcaagcaagcgactcataatcgccagatggt
ggttcaattccacccagggccacca
1–6tRNA–Thr(tgt)c[69349,69424]
gctgatttagctcagtaggtagagcaactcacttgtaatgagaaggtcgg
cggttcgattccgtcaatcagcacca
1–7tRNA–Ser(tga)c[69430,69519]
ggaggcgtggcagagtggtttaatgcaccggtcttgaaaaccggcagtcg
ctccggcgactcataggttcaaatcctatcgcctccgcca
1–8tRNA–Pro(tgg)c[69521,69595]
ctccgtgtagctcagtttggtagagcgtctgctttgggagcagaatgtcg
taggttcaaatcctgccacggagac
1–9tRNA–Gly(tcc)c[69606,69679]
gcggatatcgtataatggtattacctcagacttccaatctgatgatgtga
gttcgattctcattatccgctcca
1–10tRNA–Leu(taa)c[69685,69771]
gcgagaatggccaaattggtaaaggcacagcacttaaaatgctgcggaat
gatttccttgtgggttcgagtcccacttctcgcacca
1–11tRNA–Gln(ttg)c[69772,69845]
tgggaattagccaagttggtaaggcactggattttgattccaggatgcaa
aggttcgagtcctttattcccagc
>KY290955.1 Aeromonas phage 65.2, complete genome
1–1tRNA–Leu(taa)[107939,108023]

gcttgtgtggtggaactggtatacacattagacttaaaatctaacgcctt
cgggattgggggttcgaatccccccacaagcacca
1−2tRNA−Arg(tct)[112185,112261]
gcgctgttagctcagttggatagagcaacgttcttctaaagcgtgggtcg
ctggttcgaatccagcacggcgcacca
1−3tRNA−Leu(tag)[112268,112351]
ggggatgtggtgaaattggcagacacaccagatttaggttctggcgctta
tagcgtgacggttcgagtccgtccatccccacca
1−4tRNA−Gly(tcc)[112459,112534]
gcgggtgtagctcagttggtagagcttctgatttccaatcagaatgtcgc
gtgttcgagtcacgttacccgctcca
1−6tRNA−Met(cat)[112623,112699]
ggccccatagctcagttggttagagcgtccgactcataatcgggtggtca
ctggttcgagtccagttggggccacca
1−7tRNA−Met(cat)[113158,113233]
tgcgagttggagaagtggtcatctcgacaggctcattacctgtaggtcgg
ttgttcgaatcaaccactcgcaacca
1−8tRNA−Asn(gtt)[113240,113315]
gggcgtgtagttccagcggtagaacgatggactgttaatccatgtgtcgg
gggttcgaatccctccacgcccgcca
1−9tRNA−Ser(tga)[113445,113538]
ggaaggtcaacccgacggtaggcgacgggagcggtcttgaaaaccgccga
gcgttaacagcgccttgtgagttcgatcctcacatcttccgcca
1−10tRNA−Lys(ttt)[113548,113623]
gcatcgttagctcagcaggtagagcatgggacttttaatctcagggtcgc
tggttcgaatccagcacgatgtacca
1−11tRNA−Tyr(gta)[113630,113713]
ggatgcataggcaaatggttaagccagcggtctgtaaaaccgtagccttc
gggcttcttggttcgaatccaagtgtatccacca
1−12tRNA−Trp(cca)[113720,113794]
aggtctatggtgtagtggtaacatgccggattccaaatccgtgcgtctgg
ggttcaagtcctcatgggcctgcca
1−13tRNA−Ala(tgc)[113800,113876]
gggggattagctcagttggaatgagcgactggtttgcaaccagtaggtca
tcggttcgaccccgatatcctccacca
1−14tRNA−Pro(tgg)[114320,114397]
ccgtgtgtaggctaggcaggtcaagtcgtctggtttgggaccagaagatc
gaaggttcgaatccttccacacggacca
1−15tRNA−Thr(tgt)[114970,115045]
gccgaattagctcatctggtagagcacagcctttgtaacgctggggtggt
ccgttcgagtcggacattcggcacca
1−16tRNA−Ile(gat)[115058,115133]
agtggattagctcagtaggtagagcactcgaccgataatcgagagcgcat
tggttcaaccccaatatccactacca

1–17tRNA–Ser(gct)[115140,115227]
ggagaagtggatgagcggcttaaatcgctcccctgctaagggagtaaacc
ggaaggtttcgagagttcgaatctctccttctccgcca
1–18tRNA–Asp(gtc)[115235,115310]
gaggatgtagtatagtggtcaatatccctgcctgtcacgtaggagatcgc
gggttcgagtcccgtcatcctcgcca
>KY290951.1 Aeromonas phage 31.2, complete genome
1–1tRNA–Arg(tct)c[65027,65103]
ggtctcttagctcagctggacagagcaacgtcgttctaagtcgttggtca
ttggttcgaatccaatagggaccacca
1–2tRNA–Met(cat)c[66072,66146]
ggccctgtagctagacggttcaagcaggcggctcataaccgctcgtagca
ggttcgattcctgccagggccacca
1–3tRNA–Met(cat)c[66353,66429]
tgcgacgtagaggagaggtcgtcctcgtcgggctcatatcccggaaatcg
gtggttcgaatccatccgtcgcatcca
1–4tRNA–Asp(gtc)c[66451,66524]
ggagccatagtttatacggttaaaataatcccctgtcacgggatagcacc
gagttcgatcctcggtggctccgc
1–5tRNA–Ser(gct)c[66598,66685]
ggagaagtggatgagcggtttaaatcgcttccctgctaaggaagtaaacc
gaaaggtttcgagagttcgaatctctccttctccgcca
1–6tRNA–Ile(gat)c[66693,66768]
agtggattagctcagtaggtagagcactcgaccgataatcgagagcgcac
tggttcgaccccagtatccactacca
1–7tRNA–Trp(cca)c[66771,66846]
atgacattggtgttagcggtagcatgccggtctccaaaaccgtgcggcca
gggttcgaatccttgatgtcatgcca
1–8tRNA–Thr(tgt)c[67195,67270]
gccgatttagctcatccggtagagcagttgttttgtaagcatcaggtggt
ccgttcgagtcggacaatcggcacca
1–9tRNA–Pro(tgg)c[67910,67986]
ccgcgtgtaggctagtcaggtaggtcgtctggtttgggaccagaaagtcg
gaggttcgaatccttccacgcggacca
1–10tRNA–Gly(tcc)c[68493,68568]
gcgggtaaggtgtttacggatacatgctagccttccaagcttgagtagac
cggttcgataccggctacccgctcca
1–11tRNA–His(gtg)c[68581,68655]
gtgacggtagctcagctggtagagccccaggttgtgatcctggtcgtcgc
gagttcaatcctcgtccgtcacccc
1–12tRNA–Lys(ttt)c[68947,69022]
gcgccgctagcttagttggtagagcatccggcttttaaccggagggtcga
tggttcgaatccatcgcggcgcacca
1–13tRNA–Tyr(gta)c[69080,69162]

ggaacgttcgggtaatggtatcccaagcggctgtaacccgctcgcctctg
gcattcttggttcgagtccaaggcgttccacca
1-14tRNA-Asn(gtt)c[69658,69743]
ggtgatgtggctcagaggttgagcggcggactgttaatccgcgaggaatt
tcctctatactggttcgagtccagtcatcaccgcca
1-15tRNA-Phe(gaa)c[69825,69909]
gttgctgtgagccgagtggctgaaggcggtctctggaaacgggacgggtt
gaaagacctcgaaggttcgaatccttccagcaacc
1-16tRNA-Ser(tga)c[69980,70073]
ggaagcttggcagagtctggcttattgcactagtcttgaaaactagcgat
cgtaggaatacggtccatccgttcaaatcggatagcttccgcca
1-17tRNA-Leu(taa)c[70264,70347]
gggagtgtggtggaatggtatacactggagacttaaaatctcccgccttc
gggattgagggttcgagtccctcctctcctacca
>KY290948.1 Aeromonas phage 44RR2.8t.2, complete genome
1-1tRNA-Arg(tct)c[65379,65455]
ggtctcttagctcagctggacagagcaacgtcgttctaagtcgttggtca
ttggttcgaatccaatagggaccacca
1-2tRNA-Met(cat)c[66424,66498]
ggccctgtagctagacggttcaagcaggcggctcataaccgctcgtagca
ggttcgattcctgccagggccacca
1-3tRNA-Met(cat)c[66706,66782]
tgcgacgtagaggagaggtcgtcctcgtcgggctcatatcccggaaatcg
gtggttcgaatccatccgtcgcatcca
1-4tRNA-Asp(gtc)c[66804,66877]
ggagccatagtttatacggttaaaataatcccctgtcacgggatagcacc
gagttcgatcctcggtggctccgc
1-5tRNA-Ser(gct)c[66951,67038]
ggagaagtggatgagcggtttaaatcgcttccctgctaaggaagtaaacc
gaaaggtttcgagagttcgaatctctccttctccgcca
1-6tRNA-Ile(gat)c[67046,67121]
agtggattagctcagtaggtagagcactcgaccgataatcgagagcgcac
tggttcgaccccagtatccactacca
1-7tRNA-Trp(cca)c[67124,67199]
atgacattggtgttagcggtagcatgccggtctccaaaaccgtgcggcca
gggttcgaatccttgatgtcatgcca
1-8tRNA-Thr(tgt)c[67545,67620]
gccgatttagctcatccggtagagcagttgttttgtaagcatcaggtggt
ccgttcgagtcggacaatcggcacca
1-9tRNA-Pro(tgg)c[68260,68336]
ccgcgtgtaggctagtcaggtaggtcgtctggtttgggaccagaaagtcg
gaggttcgaatccttccacgcggacca
1-10tRNA-Gly(tcc)c[68842,68917]
gcgggtaaggtgtttacggatacatgctagccttccaagcttgagtagac

cggttcgataccggctacccgctcca
1–11tRNA–His(gtg)c[68930,69004]
gtgacggtagctcagctggtagagccccaggttgtgatcctggtcgtcgc
gagttcaatcctcgtccgtcacccc
1–12tRNA–Lys(ttt)c[69125,69200]
gcatcgttaactcaattggcagagtagcggacctttaatccgttggttct
gagttcgaatctcagacggtgtacca
1–13tRNA–Tyr(gta)c[69804,69886]
ggaacgttcgggtaatggtatcccaagcggctgtaacccgctcgcctctg
gcattcttggttcgagtccaaggcgttccacca
1–14tRNA–Asn(gtt)c[69900,69985]
ggtgatgtggctcagaggttgagcggcggactgttaatccgcgaggaatt
tcctctatactggttcgagtccagtcatcaccgcca
1–15tRNA–Phe(gaa)c[70067,70151]
gttgctgtgagccgagtggctgaaggcggtccctggaaacgggacgggtt
gaaagacctcgaaggttcgaatccttccagcaacc
1–16tRNA–Ser(tga)c[70222,70315]
ggaagcttggcagagtctggcttattgcactagtcttgaaaactagcgat
cgtaggaatacggtccatccgttcaaatcggatagcttccgcca
1–17tRNA–Leu(taa)c[70506,70589]
gggagtgtggtggaacggcatacacaggaggcttaaaacctcccgccttc
gggattgagggttcgagtccctcctctcctacca
>KY290956.1 Aeromonas phage L9–6, complete genome
1–1tRNA–Arg(tct)c[66651,66727]
ggtctcttagctcagctggacagagcaacgtcgttctaagtcgttggtca
ttggttcgaatccaatagggaccacca
1–2tRNA–Met(cat)c[67696,67770]
ggccctgtagctagacggttcaagcaggcggctcataaccgctcgtagca
ggttcgattcctgccagggccacca
1–3tRNA–Met(cat)c[67978,68054]
tgcgacgtagaggagaggtcgtcctcgtcgggctcatatcccggaaatcg
gtggttcgaatccatccgtcgcatcca
1–4tRNA–Asp(gtc)c[68076,68149]
ggagccatagtttatacggttaaaataatcccctgtcacgggatagcacc
gagttcgatcctcggtggctccgc
1–5tRNA–Ser(gct)c[68223,68310]
ggagaagtggatgagcggtttaaatcgcttccctgctaaggaagtaaacc
gaaaggtttcgagagttcgaatctctccttctccgcca
1–6tRNA–Ile(gat)c[68318,68393]
agtggattagctcagtaggtagagcactcgaccgataatcgagagcgcac
tggttcgaccccagtatccactacca
1–7tRNA–Trp(cca)c[68396,68471]
atgacattggtgttagcggtagcatgccggtctccaaaaccgtgcggcca
gggttcgaatccttgatgtcatgcca

1−8tRNA−Thr(tgt)c[68820,68895]
gctggattagctcatctggaagagcagttgttttgtaagcatcaggtggt
ccgttcgagtcggacatccagcacca
1−9tRNA−Pro(tgg)c[69162,69238]
ccgcgtgtaggctagtcaggtaggtcgtctggtttgggaccagaaagtcg
gaggttcgaatccttccacgcggacca
1−10tRNA−Gly(tcc)c[69745,69820]
gcgggtaaggtgtttacggatacatgctagccttccaagcttgagtagac
cggttcgataccggctacccgctcca
1−11tRNA−His(gtg)c[69833,69907]
gtgacggtagctcagctggtagagccccaggttgtgatcctggtcgtcgc
gagttcaatcctcgtccgtcacccc
1−12tRNA−Lys(ttt)c[70028,70103]
gcgccgctagcttagttggtagagcatccggcttttaaccggagggtcga
tggttcgaatccatcgcggcgcacca
1−13tRNA−Tyr(gta)c[70161,70243]
ggaacgttcgggtaatggtatcccaagcggctgtaacccgctcgcctctg
gcattcttggttcgagtccaaggcgttccacca
1−14tRNA−Asn(gtt)c[70259,70334]
ggttcattagctcagtggttagagcgttcgcctgttaagcgaggggtcac
cggttcaaatccggtatggaccgcca
1−15tRNA−Phe(gaa)c[70418,70502]
gttgctgtgagccgagtggctgaaggcggtctctggaaacgggacgggtt
gaaagacctcgaaggttcgaatccttccagcaacc
1−16tRNA−Ser(tga)c[70573,70666]
ggaagcttggcagagtctggcttattgcactagtcttgaaaactagcgat
cgtaggaatacggtccatccgttcaaatcggatagcttccgcca
1−17tRNA−Leu(taa)c[70857,70940]
gggagtgtggtggaacggcatacacaggaggcttaaaacctcccgccttc
gggattgagggttcgagtccctcctctcctacca
>KY290957.1 Aeromonas phage Riv−10, complete genome
1−1tRNA−Arg(tct)c[66489,66565]
ggtctcttagctcagctggacagagcaacgtcgttctaagtcgttggtca
ttggttcgaatccaatagggaccacca
1−2tRNA−Met(cat)c[67534,67608]
ggccctgtagctagacggttcaagcaggcggctcataaccgctcgtagca
ggttcgattcctgccagggccacca
1−3tRNA−Met(cat)c[67816,67892]
tgcgacgtagaggagaggtcgtcctcgtcgggctcatatcccggaaatcg
gtggttcgaatccatccgtcgcatcca
1−4tRNA−Asp(gtc)c[67914,67987]
ggagccatagtttatacggttaaaataatcccctgtcacgggatagcacc
gagttcgatcctcggtggctccgc
1−5tRNA−Ser(gct)c[68061,68148]

ggagaagtggatgagcggtttaaatcgcttccctgctaaggaagtaaacc
gaaaggtttcgagagttcgaatctctccttctccgcca
1-6tRNA-Ile(gat)c[68156,68231]
agtggattagctcagtaggtagagcactcgaccgataatcgagagcgcac
tggttcgaccccagtatccactacca
1-7tRNA-Trp(cca)c[68234,68309]
atgacattggtgttagcggtagcatgccggtctccaaaaccgtgcggcca
gggttcgaatccttgatgtcatgcca
1-8tRNA-Thr(tgt)c[68655,68730]
gccgatttagctcatccggtagagcagttgttttgtaagcatcaggtggt
ccgttcgagtcggacaatcggcacca
1-9tRNA-Pro(tgg)c[69370,69446]
ccgcgtgtaggctagtcaggtaggtcgtctggtttgggaccagaaagtcg
gaggttcgaatccttccacgcggacca
1-10tRNA-Gly(tcc)c[69952,70027]
gcgggtaaggtgtttacggatacatgctagccttccaagcttgagtagac
cggttcgataccggctacccgctcca
1-11tRNA-His(gtg)c[70040,70114]
gtgacggtagctcagctggtagagccccaggttgtgatcctggtcgtcgc
gagttcaatcctcgtccgtcacccc
1-12tRNA-Lys(ttt)c[70235,70310]
gcatcgttaactcaattggcagagtagcggacctttaatccgttggttct
gagttcgaatctcagacggtgtacca
1-13tRNA-Tyr(gta)c[70914,70996]
ggaacgttcgggtaatggtatcccaagcggctgtaacccgctcgcctctg
gcattcttggttcgagtccaaggcgttccacca
1-14tRNA-Asn(gtt)c[71010,71095]
ggtgatgtggctcagaggttgagcggcggactgttaatccgcgaggaatt
tcctctatactggttcgagtccagtcatcaccgcca
1-15tRNA-Phe(gaa)c[71177,71261]
gttgctgtgagccgagtggctgaaggcggtccctggaaacgggacgggtt
gaaagacctcgaaggttcgaatccttccagcaacc
1-16tRNA-Ser(tga)c[71332,71425]
ggaagcttggcagagtctggcttattgcactagtcttgaaaactagcgat
cgtaggaatacggtccatccgttcaaatcggatagcttccgcca
1-17tRNA-Leu(taa)c[71616,71699]
gggagtgtggtggaacggcatacacaggaggcttaaaacctcccgccttc
gggattgagggttcgagtccctcctctcctacca
>KY290958.1 Aeromonas phage SW69-9, complete genome
1-1tRNA-Arg(tct)c[66154,66230]
ggtctcttagctcagctggacagagcaacgtcgttctaagtcgttggtca
ttggttcgaatccaatagggaccacca
1-2tRNA-Met(cat)c[67199,67273]
ggccctgtagctagacggttcaagcaggcggctcataaccgctcgtagca

ggttcgattcctgccagggccacca
1–3tRNA–Met(cat)c[67481,67557]
tgcgacgtagaggagaggtcgtcctcgtcgggctcatatcccggaaatcg
gtggttcgaatccatccgtcgcatcca
1–4tRNA–Asp(gtc)c[67579,67652]
ggagccatagtttatacggttaaaataatcccctgtcacgggatagcacc
gagttcgatcctcggtggctccgc
1–5tRNA–Ser(gct)c[67726,67813]
ggagaagtggatgagcggtttaaatcgcttccctgctaaggaagtaaacc
gaaaggtttcgagagttcgaatctctccttctccgcca
1–6tRNA–Ile(gat)c[67821,67896]
agtggattagctcagtaggtagagcactcgaccgataatcgagagcgcac
tggttcgaccccagtatccactacca
1–7tRNA–Trp(cca)c[67899,67974]
atgacattggtgttagcggtagcatgccggtctccaaaaccgtgcggcca
gggttcgaatccttgatgtcatgcca
1–8tRNA–Thr(tgt)c[68320,68395]
gccgatttagctcatccggtagagcagttgttttgtaagcatcaggtggt
ccgttcgagtcggacaatcggcacca
1–9tRNA–Pro(tgg)c[69037,69111]
ctcggtgtaggcaagtggtatgtcgttccgtttgggacggaattttcgag
tgttcgattcactccaccgagacca
1–10tRNA–Gly(tcc)c[69300,69375]
gcgggtaaggtgtttatggatacatgctagccttccaagcttgagtagac
cggttcgataccggctacccgctcca
1–11tRNA–His(gtg)c[69388,69462]
gtgacggtagctcagctggtagagccccaggctgtgatcctggtcgtcgc
gagttcaatcctcgtccgtcacccc
1–12tRNA–Lys(ttt)c[69583,69658]
ccgttgctagcttagctggtagagcatccggcttttaaccggagggtcga
tggttcgaatccatcgcggcgcacca
1–13tRNA–Tyr(gta)c[69706,69788]
ggaacgttcgggtaatggtatcccaagcggctgtaacccgctcgcctctg
gcattcttggttcgagtccaaggcgttccacca
1–14tRNA–Asn(gtt)c[69804,69879]
ggttcattagctcagtggttagagcgttcgcctgttaagcgaggggtcac
cggttcaaatccggtatggaccgcca
1–15tRNA–Phe(gaa)c[69963,70047]
gttgctgtgagccgagtggctgaaggcggtctctggaaacgggacgggtt
gaaagacctcgaaggttcgaatccttccagcaacc
1–16tRNA–Ser(tga)c[70118,70211]
ggaagcttggcagagtctggcttattgcactagtcttgaaaactagcgat
cgtaggaatacggtccatccgttcaaatcggatagcttccgcca
1–17tRNA–Leu(taa)c[70402,70485]

gggagtgtggtggaatggtatacacaggaggcttaaaacctcccgccttc
gggattgagggttcgagtccctcctctcctacca
>KY368639.1 Bacillus phage vB_BsuM-Goe2, complete genome
1-1tRNA-Asn(gtt)[28541,28615]
tcctctatagctcaatggtagagcacatgactgttaatcatggggttgta
ggttcgaatcctactggaggagcca
1-2tRNA-Ser(gct)[28703,28792]
tggaatgttacccaagcggttaaggggtctggttgctaaccagatagcgg
gcttgatgtccggcataggttcgaatcctatacattccgt
1-3tRNA-Arg(cct)[32867,32941]
tgcccatgtagctcaggggatagagcacgtccctcctaaggacggtgtcg
ggagttcaattctctccatgggcgt
>KY368640.1 Bacillus phage vB_BsuM-Goe3, complete genome
1-1tRNA-Asn(gtt)[30657,30730]
ttgtcccatagctcaatggtagagcgtccggctgttaaccgggaggttgc
tggttcgaacccagctgggacagt
1-2tRNA-Met(cat)[32239,32308]
tgcagggagaattggtattccatccggtctcataagccggactccgaggg
ttcgactccctcctctgcaa
1-3tRNA-Arg(tct)[32712,32786]
tgcctctgtggtgaaataggatatcacagcagacttctattctgctattc
taggttcgactcctagcaggggcgt
1-4tRNA-Asp(gtc)[32787,32860]
tggctctatagtgtagcggtcaacacgccagcctgtcacgctggtatccc
gggttcgaatcccggtagagtcgt
1-5tRNA-Tyr(gta)[33266,33349]
gggtgagcggtaacgtaggagagttacgatggactgtaaatccattcctt
tcgaggttagagtgttcgaatcactccttaccca
>KY581279.1 Staphylococcus phage pSa-3, complete genome
1-1tRNA-Met(cat)c[11602,11673]
ggactcttagcttaaaggtaaagccaaccgctcataacggtttgactgta
ggttcgaatcctgcagagtcca
1-2tRNA-Trp(cca)c[34820,34891]
acacccttagtataattagtagtacaagggtctccaaaacccttagtctt
tgtgcaaatcaaagagggtgtg
1-3tRNA-Phe(gaa)c[34898,34970]
ggtttcttagctcagatggtagagcactagattgaagctctaggtgtcat
tggttcaaatccaatagaaacca
1-4tRNA-Asp(gtc)c[34976,35051]
tggctcattggtgtaactggttaacacactgccctgtcacggcagagagt
acgagttcgagtctcgtatgggtcgt
>KY630187.1 Serratia phage BF, complete genome
1-1tRNA-Ser(gct)[269274,269366]
ggtagattgggtgagtggtttaaaccagcttcctgctaagaagtcggtcg

ctcaaaaggcggcccgtgggttcaaatcccacatctacctcca
1-2tRNA-Trp(cca)[273739,273810]
aggagattggccgattggtcaggcagcggattccaaatccgtctaggtgg
gttcaattcctacatctcctgc
1-3tRNA-Thr(tgt)[276483,276555]
ggttggttcatataaaggctattatggcggttttgtaatccgttcatcag
ggttcgagtccctgactaaccac
1-4tRNA-Leu(tag)[281227,281308]
ggggatatggtggaattggtagacaccgtggtcttagaagccaccgcctt
gtgcgtgggagttcgagtctccctatccccac
1-5tRNA-Arg(tct)[288101,288173]
ctcctgttagctcaatggatagagcagtagccttctaagctatagatata
ggttcgattcctatacgggagac
1-6tRNA-Pyl(cta)[289666,289741]
gcctttgtagctcagttggtagagcgggaatctctaaaattctttgtcgt
cggttcgagtccgaccatcggcacca
1-7tRNA-Met(cat)[290082,290156]
ggcccttttagctcagtggtagagcaggcgactcataatcgcttggtcgtt
ggatcgttcccaacaagggccacca
1-8tRNA-Leu(taa)[291529,291617]
gcgggtgtggcggtattggcagtacgcacaggacttaaaatcctgtaggg
gtttcccccttgtgagttcgaatctcatcacccgtacca
1-9tRNA-Leu(caa)[298104,298189]
ggggatatggcgaaattggtaagacgcaccacactcaaaatgtggcggtt
taatccgtgtcggttcgactccgactatccccacca
1-10tRNA-Glu(ttc)[301155,301229]
gttctgttagacaaattggtaaagtcaccaccctttcaaggtggggttta
cgggttcgaccccccgtacagaacgc
1-11tRNA-Ser(tga)[301575,301667]
ggtaggttggcagagtttggcttattgcacttgtcttgaaaacaagcggg
ggttaacagccctccgagggttcaaatccctcacctacctcca
1-12tRNA-Ser(gga)[302319,302411]
ggtaaagtggcagagcggctgaatgcaaccgcttggaaagcggtcaggtt
aggtaatccctagcctcgtaggttcaaatcctatctttaccgc
1-13tRNA-Ile(gat)[304238,304311]
gggaatataattcagttggtagaatgctcgaccgataatcgagtcgccgc
tggttcgagtccagctattcccac
1-14tRNA-Asn(gtt)[306231,306303]
ggttatgtcgtataaaggttattacggagagctgttaactcttttatctt
ggttcgattccaagcatgaccgc
1-15tRNA-Gln(ttg)[306480,306553]
aggagtatggtgtaattggttagcacacgtgactttgactcacgtagttg
aggttcgagtccttatattcctgc
1-16tRNA-Gly(tcc)[306704,306786]

gcgggattagctcagtggtagagcacttgccttccaagcaagaggcgaat
aggttccatcggttcgaatccgatattccgctc
1–17tRNA–Asp(gtc)[308007,308079]
ggagctatcgtatatcggttagtatactcccctgtcacgggagagaggcg
ggttcgattcccgctagttccgc
1–18tRNA–Arg(acg)[308761,308835]
gttctcgtagctcattcggatagagcgtcacgctacgaacgtgaaggtag
taggttcgatcccttccgagaacgc
1–19tRNA–Pro(tgg)[310225,310301]
cggggtgtagctcagttgattagagtgcatattttggggatatgaagccg
ctggtttgagtccagccatcccgacca
1–20tRNA–Pro(tgg)[310311,310383]
ctccgattgggtcattggttgatcgcatagtttgggactatgattaaggg
tgttcgattcactcattggagac
1–21tRNA–His(gtg)[312101,312177]
gtggctataagcatagttggttaatgttcctgcctgtgacgcagatgacg
tgggttcgactcccactagccacacca
1–22tRNA–Phe(gaa)[312444,312521]
gggtctgtagctcagtaggtttagagcgttggtttgaaacaccaaaggtc
gagggttcgattccctctggacccacca
1–23tRNA–Lys(ttt)[313219,313305]
gggtcgttcactcagttggttagagtaccggacttttaatccggcgggta
tatcccatcgaaggttcgaatccttcacggcctacca
1–24tRNA–Tyr(gta)[315603,315687]
ggcatcgtcgcatagcggcaattgcaggagactgtaaatctctcgccttc
gggcttcgttggttcgagtccaaccggtgccacca
1–25tRNA–Cys(gca)[317506,317583]
gcaacggtgcctgagatggcttaaaggagcggattgcaaatccgatgttc
gggggttcgaatccctcccgttgctcca
1–26tRNA–Lys(ctt)[317955,318031]
gggctgttaactcagtaggttagagtagccgactcttaatcggtttgtcc
agggttcgaatccctgacagcccacca
1–27tRNA–Met(cat)[318316,318389]
ggtcagttagttcagttggtagaacgcttcactcataatgaagaagtcac
tggatcgtagccagtactgaccac
1–28tRNA–Met(cat)[319253,319328]
tgcggagtagagcagtggtagcttgcaaggctcataaccttgaggtcgtg
tttggttcgaatcccacctccgcatc
1–29tRNA–Ala(tgc)[319346,319419]
ggggatatagctcagttgggagagcatctgccttgcacgtagaaggtcat
cggttcgaccccgattatctccac
>KY593455.1 Yersinia phage fHe–Yen9–01, complete genome
1–1tRNA–Gly(tcc)c[75566,75641]
gcagagttcgtatagtggcttaatactattgacttccacccagtaaacat

cggttcgaatccgatactctgctcca
1–2tRNA–Trp(cca)c[75892,75966]
aggtctttagtatagtggcgattatgctgggctccaaacctagtgacggg
tgttcgattcacccaaggcctgcca
1–3tRNA–Arg(tct)c[77249,77325]
gtcctcatagctcaacaggacagagcaacggtcttctaaaccgtaggttg
ctggttcgattccagctggggacacca
1–4tRNA–Met(cat)c[77562,77638]
ggcccttagctcagaagagtagagcgagcgactcataatcgttaggtca
ctggttcgaatccagtaagggtcacca
>KY608967.1 UNVERIFIED: Escherichia phage CF2, complete genome
1–1tRNA–Gln(ttg)[87968,88041]
tgggaattagccaagttggtaaggcactggattttgattccaggatgcaa
aggttcgagtcctttattcccagc
1–2tRNA–Leu(taa)[88042,88128]
gcgagaatggccaaattggtaaaggcacagcacttaaaatgctgcggaat
gatttccttgtgggttcgagtcccacttctcgcacca
1–3tRNA–Gly(tcc)[88134,88207]
gcggatatcgtataatggtattacctcagacttccaatctgatgatgtga
gttcgattctcattatccgctcca
1–4tRNA–Pro(tgg)[88218,88292]
ctccgtgtagctcagtttggtagagcgtctgctttgggagcagaatgtcg
taggttcaaatcctgccacggagac
1–5tRNA–Ser(tga)[88294,88383]
ggaggcgtggcagagtggtttaatgcaccggtcttgaaaaccggcagtcg
ctccggcgactcataggttcaaatcctatcgcctccgcca
1–6tRNA–Thr(tgt)[88389,88464]
gctgatttagctcagtaggtagagcaactcacttgtaatgagaaggtcgg
cggttcgattccgtcaatcagcacca
1–7tRNA–Met(cat)[88466,88540]
ggccctgtagctggaaggttcaagcaagcgactcataatcgccagatggt
ggttcaattccacccagggccacca
1–8tRNA–Tyr(gta)[88550,88636]
ggggagttatcccgtagaggtagcggtgtggactgtaaattcattgtcat
tgcgactcgggtggttcgactccaccactccccacca
1–9tRNA–Asn(gtt)[88641,88715]
ggatgtgtagctcaatggcagagcgatcgcctgttaagcgattggttata
ggttcgaatcctatcacgtccgcca
1–10tRNA–His(gtg)[88830,88905]
gtggccgtagttcagttggtagaactcgagattgtgattctcgtagtcat
gggttcgactcccatcggtcacccca
1–11tRNA–Arg(tct)[88910,88985]
cggggcatagctcagaaggaagagcaaggaccttctaagtcctaggtcgt
aggttcgatccctactgcctcgacca

>KY608965.1 UNVERIFIED: Escherichia phage HP3, complete genome

1-1tRNA-Arg(tct)c[84135,84211]

cgaggcataactcaattgtatagagcaacggacttctaatccgtaggttg
aaggttagaatccttctgtctcgacca

1-2tRNA-Met(cat)c[84218,84291]

ggccctgtagctcaatggttagcagcagtcccctcataagggaaaggtta
ccagttcgaatctggtctgggtca

>KY626162.1 Salmonella phage Si3, complete genome

1-1tRNA-Pro(tgg)[2154,2230]

ctctgtttagctcagcttggtagagcgttccgtttggggcggtaaggccg
gaggttcaagtcctccaacagagacca

1-2tRNA-Glu(ttc)[2238,2315]

gttccagtagacaaaatggtatagtcaccactctttcaaagtggatattt
gagggttcaaatcccttctggaacgcca

1-3tRNA-Met(cat)[2407,2483]

tgcgggtatagagaaagggcgtctcacatgtctcattagcatggtatcgg
caggttcgactcctgcacccgcctcca

1-4tRNA-Asn(gtt)[2567,2643]

ggttaggaagcacataaggtatgtgcggtcgcctgttaagcgaatggcac
agggttcgaatccctgactaaccgcca

1-5tRNA-Tyr(gta)[2713,2800]

gtgtcgttatcccgtagatggtagcggtggggactgtaaatcccttgtca
ttgagactcggtaggttcgactcctacacggcacacca

1-6tRNA-Asp(gtc)[2806,2882]

ggttatgtagtttaatttggttaaaatactccctgtcacgggagatgatg
agggttcaaatcccttcgtaaccgcca

1-7tRNA-Lys(ttt)[3311,3386]

ggaagtgtagcagaatggtgatgcggcagacttttaatctgacaggcgat
gggttcgaatccctccacttctacca

1-8tRNA-Met(cat)[3391,3467]

ggttcagtcgcagataaggtaatgcaagggtctcataagccctatgaatg
tgggttcgattcccatctgaacctcca

1-9tRNA-Ile(gat)[3470,3544]

tgtgggttagcataaatggtaatgcaaacggctgataaccgttagaagag
ggttcgataccctcacctacaacca

1-10tRNA-Ser(tga)[4201,4290]

ggtaggtagcggctaatggtagccaaactgtcttgaaaacagttgccact
gtagagatacggtgagggttcgactcctttacttaccgcc

1-11tRNA-Leu(tag)[4546,4623]

gggagattgacggtaattggtaaacctacctcgcttagaacgaggtgttt
gagggttcgaatcccttgtctcctacca

1-12tRNA-Lys(ctt)[4631,4706]

gcaggtgtagcaaaatggttatgcggctgactcttaatcagtaagacgat
gggttcaattccctccacctgtacca

1-13tRNA-Ala(tgc)[4713,4788]
ggggatgtagtttacatggttaaaacataagttttgcaaacttgggaact
gagttcaattctcagtgactccacca
1-14tRNA-Gly(tcc)[4795,4869]
gcatccatagtttaaacgggaaaattacagtcttccaaactgaggttgag
ggttcgattccctctggatgctcca
1-15tRNA-Thr(tgt)[4876,4952]
gctgctttcgtataattggctattacacatcccttgtaaggatggaaatg
caggttcgagtcctgtgagcagcacca
1-16tRNA-Val(tac)[5048,5122]
actcgcttagtttatatggtaaaacatcacccttacaagatgaagaaaaa
ggttcaagtcctttagtgagtacca
1-17tRNA-Leu(taa)[5124,5202]
gttccagtatcccaattggcagagaggccagacttaaaatctgtgttatg
tatcggttcgaatccgatatggagtacca
1-18tRNA-Gln(ctg)[5208,5283]
agcggtatagcataactggcaatgcagcagtctctgaagctgtcctatta
aggttcaaatccttatgccgctgcca
1-19tRNA-His(gtg)[5315,5390]
gtggccttatcataaacggtaatgacccatgctgtgaacatggtctatac
gggttcaaatcccgtaggtcacccca
1-20tRNA-Phe(gaa)[5397,5472]
agtccaagtagcttatatggttaaagcgcgtgtctgaaaaacatgagaag
agggttcaaatcccactggactacca
1-21tRNA-Ser(gct)[6226,6318]
ggaagattaaccctaaaaggtaagggagcagtttgctaaactgccagtag
ctgagaaatcggtgtaccagttcaagtctggtatcttcctcca
1-22tRNA-Cys(gca)[6323,6398]
gaatccgtgacagaaatggatatgtgcctgtctgcaaaacaggtttataa
gggttcaagtcccttcggattctcca

>KP143762.1 Salmonella phage Mushroom, complete genome

1-1tRNA-Cys(gca)c[60589,60664]
gaatccgtgacagaaatggatatgtgcctgtctgcaaaacaggtttataa
gggttcaagtcccttcggattctcca
1-2tRNA-Ser(gct)c[60669,60761]
ggaagattaaccctaaaaggtaagggagcagtttgctaaactgccagtag
ctgagaaatcggtgtaccagttcaagtctggtatcttcctcca
1-3tRNA-Phe(gaa)c[61514,61589]
agtccaagtagcttatatggttaaagcgcgtgtctgaaaaacatgagaag
agggttcaaatcccactggactacca
1-4tRNA-His(gtg)c[61596,61671]
gtggccttatcataaacggtaatgacccatgctgtgaacatggtctatac
gggttcaaatcccgtaggtcacccca
1-5tRNA-Gln(ctg)c[61703,61778]

agcggtatagcataactggcaatgcagcagtctctgaagctgtcctatta
aggttcaaatccttatgccgctgcca
1-6tRNA-Leu(taa)c[61784,61862]
gctccattactccaattggcagagaggccagacttaaaatctgtgttatg
tatcggttcgaatccgatatggagtacca
1-7tRNA-Gln(ttg)c[61865,61940]
aggggattagtttacaaggttaaaacctcggtctttgaaatcgaagaagt
tggttcaattccaacatcccccgcca
1-8tRNA-Leu(caa)c[62525,62602]
gttccagtatcccaattggcagaggatgcaagctcaaaccttgtattagt
gacggttcgaatccgtcttggaacacca
1-9tRNA-Val(tac)c[62604,62678]
actcgcttagtttatatggtaaaacatcacccttacaagatgaagaaaaa
ggttcaagtcctttagtgagtacca
1-10tRNA-Thr(tgt)c[62774,62850]
gctgctttcgtataattggctattacacatcccttgtaaggatggaaatg
caggttcgagtcctgtgagcagcacca
1-11tRNA-Gly(tcc)c[62857,62931]
gcatccatagtttaaacgggaaaattacagtcttccaaactgaggttgag
ggttcgattccctctggatgctcca
1-12tRNA-Ala(tgc)c[62938,63013]
tgggtcatagtttatatggttaaaattcgagttttgcaaacttgggaact
gagttcaattctcagtgactccacca
1-13tRNA-Lys(ctt)c[63020,63095]
gcaggtgtagcaaaatggttatgcggctgactcttaatcagtaagacgat
gggttcaattccctccacctgtacca
1-14tRNA-Leu(tag)c[63103,63180]
gggagattgacggtaattggtaaacctaccatccttagaagttggtgttt
gagggttcgaatcccttgtctcctacca
1-15tRNA-Ser(tga)c[63436,63525]
ggtaggtagcggctaatggtagccaaactgtcttgaaaacagttgccact
gtagagatacggtgagggttcgactcctttacttaccgcc
1-16tRNA-Ile(gat)c[64183,64257]
tgtgggttagcataaatggtaatgcaaacggctgataaccgttagaagag
ggttcgataccctcacctacaacca
1-17tRNA-Met(cat)c[64260,64336]
ggttcagtcgcagataaggtaatgcaagggtctcataagccctatgaatg
tgggttcgattcccatctgaacctcca
1-18tRNA-Lys(ttt)c[64341,64416]
ggaagtgtagcagaatggtgatgcggcagacttttaatctgacaggcgat
gggttcgaatccctccacttctacca
1-19tRNA-Asp(gtc)c[64845,64922]
ggttatgtagtttaatttggttaaaatactcccctgtcacgggagatgat
gagggttcaaatcccttcgtaaccgcca

1-20tRNA-Tyr(gta)c[64928,65015]
gtgtcgttatcccgtagatggtagcggtggggactgtaaatcccttgtca
ttgagactcggtaggttcgactcctacacggcacacca
1-21tRNA-Asn(gtt)c[65085,65161]
ggttaggaagcacataaggtatgtgcggtcgcctgttaagcgaatggcac
agggttcgaatccctgactaaccgcca
1-22tRNA-Met(cat)c[65245,65321]
tgcgggtatagagaaagggcgtctcacatgtctcattagcatggtatcgg
caggttcgactcctgcacccgcctcca
1-23tRNA-Glu(ttc)c[65413,65490]
gttccagtagacaaaatggtatagtcaccactctttcaaagtggatattt
gagggttcaaatcccttctggaacgcca
1-24tRNA-Pro(tgg)c[65498,65574]
ctctgtttagctcagcttggtagagcgttccgtttggggcggtaaggccg
gaggttcaagtcctccaacagagacca
>KY654690.2 Citrobacter phage Mijalis, complete genome
1-1tRNA-Cys(gca)c[60325,60400]
gtatcggtgacagaaatggatatgtgcctgtctgcaaaacaggtttatga
gggttcaagtcccttccgatactcca
1-2tRNA-Ser(gct)c[60408,60500]
ggaagattaaccataaaaggtaagggagcagtttgctaaactgccagtaa
ccgagaaatcggcgtaccagttcaagtctggtatcttcctcca
1-3tRNA-Phe(gaa)c[61252,61327]
agctcaattagcttatatggttaaagcgcgtgtctgaaaaacatgagaac
agggttcaaatcccgatgggctacca
1-4tRNA-His(gtg)c[61335,61411]
gtggccttatcataaatggataatgacccatgctgtgaacatggtctata
cgggttcgattcccgtaggtcacccca
1-5tRNA-Gln(ctg)c[61443,61518]
agcagtgtagcataactggcaatgcaccagcctctgaagctgaatgatta
aggttcaaatccttacgctgctgcca
1-6tRNA-Leu(taa)c[61525,61603]
gctccattactccaattggcagagagaccagacttaaaatctgcgttatg
tatcggttcgaatccgatatggagtacca
1-7tRNA-Gln(ttg)c[61606,61681]
aggggattagtttacacggttaaaacctcggtctttgaaatcgaagaaga
tggttcaattccatcatcccccgcca
1-8tRNA-Arg(acg)c[61689,61765]
gcagaattagttcaaatggacagagcagcagcctacgaagttgttagtta
gaggttcgaatcctctattctgcacca
1-9tRNA-Leu(caa)c[61944,62021]
gttccagtatcccaattggcagaggatgcaagctcaaaccttgtattagt
gacggttcgaatccgtcttggaacacca
1-10tRNA-Val(tac)c[62023,62097]

gctcgcttagtttatatggtaaagccccatccttacaagttggtgaagaa
ggttcaagtccttcagcgagtacca
1−11tRNA−Thr(tgt)c[62192,62267]
gctcctatcgtataactggctattacggttgccttgtaagcaacttatca
gggttcgaatccttgtgggagcacca
1−12tRNA−Gly(tcc)c[62274,62348]
gcatccatagtttaaacgggaaaattacagtcttccaaactgaggttgag
ggttcgattccctctggatgctcca
1−13tRNA−Ala(tgc)c[62355,62430]
ggggatgtagtttacatggttaaaacataagttttgcaaacttaagtaca
gggttcaattccctgcttctccacca
1−14tRNA−Lys(ctt)c[62437,62512]
gcaggtgtagcaaaatggttatgcggctgactcttaatcagtaagacgat
gggttcaattccctccacctgtacca
1−15tRNA−Leu(tag)c[62520,62596]
gcacctatagcccaattggaagaggcagcagacttagaatctgctcagtg
tgagttcgagtctctctaggtgtacca
1−16tRNA−Ser(tga)c[62848,62937]
ggtaggtagcggctaatggaagccaaactgtcttgaaaacagttgccatt
gtagagatacggtgagggttcgactcctttatctaccgcc
1−17tRNA−Arg(tct)c[63328,63404]
gcacccttagttcaaacggatagagcaacggtcttctaaaccgtcagtta
caggttcgaatcctgtagggtgtgcca
1−19tRNA−Ile(gat)c[63682,63757]
gctggtatagttaagacggttataacactcccctgataagggaacatcgg
tggttcgattccacctaccagtacca
1−20tRNA−Met(cat)c[63760,63836]
ggttcagtcgcagataaggtaatgcaagggtttcataagccctatgaatg
tgggttcgattcccacctgaacctcca
1−21tRNA−Lys(ttt)c[63843,63918]
ggaagtgtagcagaatggtgatgcggcagacttttaatctgacaggcgat
gggttcgaatccctccacttctacca
1−22tRNA−Asp(gtc)c[64345,64421]
ggttatgtagtttaactggataaaatacctccctgtcacggaggatgatg
agggttcgattcccttcgtaaccgcca
1−23tRNA−Asn(gtt)c[64527,64602]
ggtaagttagctaacttggtaaagcgcctgactgttaatcagcgatatca
gggttcaaatccctgacttaccgcca
1−24tRNA−Met(cat)c[64687,64763]
tgcgggtatagagaaagggcgtctcacatgtctcattagcatggtatcgg
taggttcgactcctacacccgcctcca
1−25tRNA−Glu(ttc)c[64850,64927]
attcccgtagacaaattaggtaaagtcaacaccctttcaaggtgtggttt
gagggttcaagtcccttcgggaatgcca

1−26tRNA−Pro(tgg)c[64934,65010]
ctccatttagctcagcttggtagagcgttccgtttggggcggtaaggccg
aaggttcaagtccttcaatggagacca
>KY779849.1 Staphylococcus phage qdsa002, complete genome
1−1tRNA−Met(cat)c[19488,19559]
ggactcttagcttaaaggtaaagccaaccgctcataacggtttgactgta
ggttcgaatcctgcagagtcca
1−2tRNA−Trp(cca)c[41139,41210]
acacccttagtataattagtagtacaagggtctccaaaacccttagtctt
tgtgcaaatcaaagagggtgtg
1−3tRNA−Phe(gaa)c[41217,41289]
ggtttcttagctcagatggtagagcactagattgaagctctaggtgtcat
tggttcaaatccaatagaaacca
1−4tRNA−Asp(gtc)c[41295,41370]
tggctcattggtgtaactggttaacacactgccctgtcacggcagagagt
acgagttcgagtctcgtatgagtcgt
>KY703222.1 Escherichia phage phiC120, complete genome
1−1tRNA−Arg(tct)c[164441,164517]
cgaggcatagctcaattgtatagagcaacggacttctaatccgtaggttg
aaggttagaatccttctgtctcggcca
1−2tRNA−Met(cat)c[164524,164597]
ggccctgtagctcaacggttagcagcagtcccctcataagggaaaggtta
ccagttcgaatctggtctgggtca
>KR052480.1 Sinorhizobium phage phiM7, complete genome
1−1tRNA−Thr(tgt)c[78511,78594]
gccggtatagccaagcggtcgaaggcacctgatttgtaatcaggtattca
aacgtcggcagttcgaatctgtctaccggctcca
1−2tRNA−Gln(ttg)c[78595,78668]
ttgcccttagttcagttggtagaacgtcgaattttggtttcgaaggtccg
tggttcgaatccacgagggtaagc
1−3tRNA−His(gtg)[83541,83617]
tcatggatggtgttagtctggtagcacgcgcggatgtggaccgcgaagca
taggttcaaatcctattccatggacca
1−4tRNA−Leu(caa)[92539,92626]
tgggagataaaggaaacttaccagttcaattctggtttctccaagagtaa
tcgagtggttgtaggttcgagtcctacctccccagcca
1−5tRNA−Met(cat)[93177,93253]
tgcggggtagtagcagtggttagctcgtctgcctcataagcagaaggtca
tcagttcgaatctgatccccgcaacca
1−6tRNA−Cys(gca)[93940,94014]
gacgagaaggcagaagggttatgcgcggcgttgcaaccgccgtatgtgtc
ggttcaagtccggctctcgtctcca
1−7tRNA−Lys(ttt)[98572,98648]
gcgcgcttaactcagttggttagagtggcgaccttttaagtcgtttgtcc

ccggttcgagtccgggagcgcgcacca
>KY683736.1 Escherichia phage V18, complete genome
1-1tRNA-Met(cat)c[56620,56694]
tgccctgtagctggaaggttcaagcaagcgactcataatcgccagacggt
ggttcaattccacccagggccacca
1-2tRNA-Thr(tgt)c[56695,56770]
gctgatgtagcacaattggtagtgcaattgatttgtaatcaataggttgc
aggttcgagtcctgctatcagcacca
1-3tRNA-Arg(tct)c[56868,56942]
gccctgttagcttagtggataaagcagcggccttctaagccgttgacact
ggttcgagtccagtacggggtgcca
>KY794641.1 Staphylococcus phage vB_Sau_CG, complete genome
1-1tRNA-Met(cat)c[117543,117614]
ggactcttagcttaaaggtaaagccaaccgctcataacggtttgactgta
ggttcgaatcctgcagagtcca
1-2tRNA-Trp(cca)c[138456,138527]
acacccttagtataattagtagtacaagggtctccaaaacccttagtctt
tgtgcaaatcaaagagggtgtg
1-3tRNA-Phe(gaa)c[138534,138606]
ggtttcttagctcagatggtagagcactagattgaagctctaggtgtcat
tggttcaaatccaatagaaatca
1-4tRNA-Asp(gtc)c[138612,138687]
tggctcattggtgtaactggttaacacactgccctgtcacggcagagagt
acgagttcgagtctcgtatgagtcgt
>KY794642.1 Staphylococcus phage vB_Sau_Clo6, complete genome
1-1tRNA-Asp(gtc)c[139472,139547]
tggctcattggtgtaactggttaacacactgccctgtcacggcagagagt
acgagttcgagtctcgtatgagtcgt
>KY794643.1 Staphylococcus phage vB_Sau_S24, complete genome
1-1tRNA-Asp(gtc)c[135731,135806]
tggctcattggtgtaactggttaacacactgccctgtcacggcagagagt
acgagttcgagtctcgtatgagtcgt
>KY994101.1 Pseudomonas phage vB_PaeM_G1, complete genome
1-1tRNA-Asn(gtt)[23850,23926]
tccgttcggtccctcaaggtgagggagcttgactgttaatcaagacgtgc
ctggttcgattccagggagcggagcca
1-2tRNA-Tyr(gta)[23984,24071]
ggaggggtggcagagcggtttaatgcaccggactgtaaatccggcgtccg
actgggcatcgctggttcaaatccagccccctccacca
1-3tRNA-Gln(ttg)[24273,24349]
aggcgtgtggcgaaaaggtttaacgcactggactttgactccagcatttg
tgggttcgaatcccaccacgtctgcca
>KY945241.1 Synechococcus phage S-H35, complete genome
1-1tRNA-Asn(gtt)c[37966,38040]

ttcctctatagctcagtcggtagagcgagtgactgttaatcactatgtcc
ctggttcgagcccaggtggaggagt
1−2tRNA−Ala(tgc)c[38065,38137]
ggggaattagctcagttggtagagcacctgctttgcacgcagggggtcag
cggttcgagtccgctattctcca
1−3tRNA−Thr(tgt)c[38140,38215]
gccactatagctcagctggatagagcaacggttttgtaaaccgtaggtcg
tcggttcaagtccgacttgtggctcc
1−4tRNA−Leu(taa)c[39538,39620]
gggacggtggcggaatggtagacgcaccagacttaaaatctgttgactgt
atagtcgtgagggttcaagtccctctcgtccta
1−5tRNA−Pro(tgg)c[40174,40248]
cggggtgtagcgcagtttggtagcgcatctgctttgggagcagagggtcg
caggttcgaatcctgtcaccccgac
1−6tRNA−Val(tac)c[43880,43951]
gggcgattaactcagcggtagagtggcctccttacaagtggtaagtcact
ggttcgaatccagtatcgccca
1−7tRNA−Leu(tag)c[70872,70953]
gcacatgtggcggaattggtagacgcgctgggtttaggttccagtagagc
aatctgtggaggttcaagtcctctcatgtgta
1−8tRNA−Arg(tct)c[72650,72725]
tggtccagtagctcagttggatagagcaactgccttctaagcagtcggtc
gctggttcgagtccagcctggatcgt
>KY942056.1 Dickeya phage JA15, complete genome
1−1tRNA−Met(cat)[131324,131400]
gatggtgtagttcagttggttagaacgtgcgactcataatcgctttgtca
ctggttcaagtcctgccgccatcgcca
>KY942057.1 Dickeya phage XF4, complete genome
1−1tRNA−Met(cat)[129075,129151]
gatggtgtagttcagttggttagaacgtgcgactcataatcgctttgtca
ctggttcaagtcctgccgccatcgcca
>KU886224.1 Erwinia phage vB_EamM_RAY, complete genome
1−1tRNA−Asn(gtt)[165666,165741]
tggtctatagttcagtcggtagaacagcggactgttaatccgtaggtcgc
tggttcgaatccagctagaccagcca
>KU886223.1 Erwinia phage vB_EamM_Simmy50, complete genome
1−1tRNA−Asn(gtt)[165487,165562]
tggtctatagttcagtcggtagaacagcggactgttaatccgtaggtcgc
tggttcgaatccagctagaccagcca
>KT363872.2 Citrobacter phage Mordin, complete genome
1−1tRNA−Pro(tgg)[28230,28306]
ctccatttagctcagcttggtagagcgttccgtttggggcggtaaggccg
aaggttcaagtccttcaatggagacca
1−2tRNA−Glu(ttc)[28313,28390]

attcccgtagacaaattaggtaaagtcaacaccctttcaaggtgtggttt
gagggttcaagtcccttcgggaatgcca
1-3tRNA-Met(cat)[28478,28554]
tgcgggtatagagaaagggcgtctcacatgtctcattagcatggtatcgg
taggttcgactcctacacccgcctcca
1-4tRNA-Asn(gtt)[28639,28715]
ggttaggaagcacataaggcatgtgcggtcggctgttaaccgaatggcac
agggttcgaatccctgactaaccgcca
1-5tRNA-Asp(gtc)[28879,28955]
ggttatgtagtttaactggtcaaaatacctccctgtcacggaggatgatg
agggttcgaatcccttcgtaaccgcca
1-7tRNA-Lys(ttt)[29406,29481]
ggaagtgtagcagaatggtgatgcggcagacttttaatctgacaggcgac
gggttcgaatccctccacttctacca
1-8tRNA-Met(cat)[29488,29564]
ggttcagtcgcagataaggtaatgcaagggtttcataagccctatgaatg
tgggttcgattcccacctgaacctcca
1-9tRNA-Ile(gat)[29567,29642]
gctggtatagttaagaaggttataacactcccctgataagggaatatcgg
tggttcgattccacctaccagtacca
1-10tRNA-Arg(tct)[29891,29967]
gcacccttagttcaaacggatagagcaacggtcttctaaaccgtcagtta
caggttcgaatcctgtagggtgtgcca
1-11tRNA-Ser(tga)[30358,30447]
ggtaggtagcggctaatggaagccaaatagtcttgaaaactattgccatt
gtagagatacggtgagggttcgactcctttatctaccgcc
1-12tRNA-Leu(tag)[30700,30777]
gggagattgacggtaattggtaaacctaccatccttagaagttggtgttt
gagggttcgaatcccttgtctcctacca
1-13tRNA-Lys(ctt)[30785,30860]
gcaggtgtagcaaaatggttatgcggctgactcttaatcagtaagacgat
gggttcaattccctccacctgtacca
1-14tRNA-Ala(tgc)[30867,30942]
ggggtcatagtttatatggttaaaatccgagttttgcaaacttgggaact
gagttcaattctcagtgactccacca
1-15tRNA-Gly(tcc)[30949,31023]
gcatccatagtttaaatgggaaaactacagtcttccaaactgaagttgag
ggttcgattccctctggatgctcca
1-16tRNA-Thr(tgt)[31030,31105]
gctcttatcgtataattggttattacggttgccttgtaagcaacttatca
gggttcgaatccttgtgggagcacca
1-17tRNA-Val(tac)[31201,31275]
gctcgcttagtttatatggtaaagccccatccttacaagttggtgaagaa
ggttcaagtccttcagcgagtacca

1-18tRNA-Leu(caa)[31277,31354]
gttccagtatcccaattggcagaggatgcaagctcaaaccttgtattagt
gacggttcgaatccgtcttggaacacca
1-19tRNA-Arg(acg)[31533,31609]
gcagaattagttcaaatggacagagcagcaacctacgaagttgttagtta
ggggttcgaatcctctattctgcacca
1-20tRNA-Gln(ttg)[31619,31694]
aggggattagtttacacggttaaaacctcggtctttgaaatcgaagaaga
tggttcaattccatcatcccccgcca
1-21tRNA-Leu(taa)[31697,31775]
gctccattactccaattggcagagaggccagatttaaaatctgtgttatg
tatcggttcgaatccgatatggagtacca
1-22tRNA-Gln(ctg)[31782,31857]
agcagtatagcataactggcaatgctccagcctctgaagctgaaagatta
aggttcaaatccttatactgctgcca
1-23tRNA-His(gtg)[31889,31965]
gtggccttatcataaatggataatgacccatgctgtgaacatggtctata
cgggttcgattcccgtaggtcacccca
1-24tRNA-Phe(gaa)[31973,32048]
agctcaattagcttatatggttaaagcgcgtgtctgaaaaacatgagaac
agggttcaaatcccgatgggctacca
1-25tRNA-Ser(gct)[32801,32893]
ggaagattaaccctaaaaggtaagggagcagtttgctaaactgccagtaa
ccgagaaatcggcgtaccagttcaagtctggtatcttcctcca
1-26tRNA-Cys(gca)[32900,32975]
gtatcggtgacagaaatggatatgtccctgtctgcaaaacaggtttatga
gggttcaagtcccttccgatactcca
>HQ728263.1 Erwinia phage vB_EamM-M7, complete genome
1-2tRNA-Pro(tgg)[5012,5089]
ctccgtttagctcagtatggatagagtaggccgtttggggcggtcgggtc
gaaggttcaaatccttcaacggagacca
1-3tRNA-Ala(tgc)[5210,5282]
gggtcagtagcttaattgggaaagcatctcacttgcaatgagaaggatga
gggttcaagtccctcctgtatcc
1-4tRNA-Met(cat)[5465,5539]
agcgggatggagcagtggtagcttgccagtttcattatctggaggtcgta
ggttcgaatcctactcccgcttcca
1-5tRNA-Asn(gtt)[5548,5623]
gggtttgaagcacatatggatgtgcggctggctgttaaccagatggtagt
gggttcgagtcccacctttcccgcca
1-6tRNA-Tyr(gta)[5630,5712]
gcgtctatggcagagatggtcaatgcagcggtctgtaaaaccgccccgaa
aggttactggttcgagtccagttaggcgcacca
1-7tRNA-Met(cat)[5719,5792]

tgcgaagtagtgaaatggcatcatactggtttcattatccggtgtaggta
gttcgaatctacccttcgcatcca
1-8tRNA-Asp(gtc)[5872,5948]
ggggatgtggcagacttggtaattgtaccgcactgtctatgcggaatatg
agggttcaaatcccttcatcctcgcca
1-9tRNA-Ser(tga)[6175,6265]
ggaagggcaaatcgactggcgacgaaaacggtcttgaaaaccgccgagcg
ttaatagcgccttgagggttcgactccctctccttccgcca
1-10tRNA-Phe(gaa)[6267,6341]
ggtctaattagcttatatggtaaagcaacggtctgaaaagccgtggaaca
gggttcgaatcccgatagaccgcca
1-11tRNA-Lys(ttt)[6349,6424]
cggtgtgtaactcaattggcagagtgggtggcttttaaccacagggttgc
aggttcgatgcctgtcacaccgacca
1-12tRNA-Lys(ctt)[6433,6509]
ggactattaactcaactggtcagagtacccgactcttaatcgggaggttc
agggttcgactccctgatagtctacca
1-13tRNA-Met(cat)[6521,6594]
agtggtatggcagagatggtcatgcacttccttcatacggaagcctacat
cggttcaagtccgattaccacttc
1-14tRNA-Ile(gat)[6608,6682]
tgtgggttagcataaatggtaatgcaaacgcctgataagcgttagaagag
ggttcgataccctcacctacgacca
1-15tRNA-Gln(ttg)[6689,6764]
cggggtatggagtaattggcaactctacggtttttgaggccgtcagtttt
cggttcgaatccgaatgcccccgcca
1-16tRNA-Arg(tct)[6771,6847]
gcacccttagaacaaacggatagttccactgtcttctaaacagttagtta
caggttcgaatcctgtagggtgtgcca
1-17tRNA-Leu(caa)[7266,7342]
ggcgtaatatcccaattggcagaggaagcaggttcaaaccctgttcagtg
tgggttcgactcccacttacgctacca
1-18tRNA-Gly(tcc)[7473,7548]
gcaagtatgatgtcaataggtaacatggcgtccttccaagtcgctcttgc
gggttcgagtcccgctacttgctcca
1-19tRNA-Trp(cca)[7555,7630]
agggggttagtttaactggcaaaatatcggcttccaaacccgtaagttga
aggttcgaatccttcacccccttgcca
1-20tRNA-Thr(tgt)[7640,7714]
gcctccatcatataagggctattatgcctgttttgtaatcaggtcatcgc
ggttcgaatccgtgtgggggcacca
1-21tRNA-Val(tac)[7721,7796]
actcgtatagctcagatggtagagcggcatctttacacgttgctggtcag
gcgttcgagtcgccttgcgagtacca

1–22tRNA–Leu(aag)[7803,7880]
ggctgtgtagcccaacttggcagaggcactacgctaagaacgtattcagt
gtgggttcgaatccctccacagctacca
1–23tRNA–Arg(acg)[7887,7963]
agacctttatcttaaatggacagagactcaagctacgaacttgagaggtt
cgggttcgaatcccgaaaggtcttcca
1–24tRNA–Gln(ctg)[7966,8042]
aggatgttcgtatagttggcctattacaccggactctgaatccggttacg
atggttcgagtccatcacgtcctgcca
1–25tRNA–Leu(taa)[8049,8125]
aggtgattaccccaattggcagagggtaggagcttaaacctccgatgttg
agagttcgaatctctcatcacctacca
1–26tRNA–His(gtg)[8129,8205]
gagttggtatcataaatggataatgaccctgactgtgaatcaggtctatg
cgggttcgaatcccgtccttctcccca
1–27tRNA–Ser(gct)[8212,8304]
ggaagattaaccctaaatggtaagggaagtgtttgctaaacactcagtaa
ccgagaaatcggcgtaccagttcaagtctggtatcttcctcca
>KP869100.1 Escherichia coli O157 typing phage 1, complete genome
1–1tRNA–Cys(gca)c[10198,10273]
gaatccgtgacagaaacggctatgtgcctgtctgcaaaacaggtttataa
gggttcgagtcccttcggattctcca
1–2tRNA–Ser(gct)c[10278,10370]
ggaagattaaccctaaaaggtaagggagcagtttgctaaactgccagtag
ctgagaaatcggtgtaccagttcaagtctggtatcttcctcca
1–3tRNA–Phe(gaa)c[11124,11199]
agtccaagtagcttatatggttaaagcgcgtgtctgaaaaacatgagaag
agggttcaaatcccactggactacca
1–4tRNA–His(gtg)c[11206,11281]
gtggccttatcataaatggtaatgacccatgctgtgaacatggtctatac
gggttcaaatcccgtaggtcacccca
1–5tRNA–Gln(ctg)c[11313,11388]
agcggtatagcataactggcaatgcagcagtctctgaagctgtcctatta
aggttcaaatccttatgccgctgcca
1–6tRNA–Leu(taa)c[11394,11472]
gctccattactccaattggcagagaggccagacttaaaatctgtgttatg
tatcggttcgaatccgatatggagtacca
1–7tRNA–Gln(ttg)c[11475,11550]
aggggattagtttacaaggttaaaacctcggtctttgaaatcgaagaagt
tggttcaattccaacatcccccgcca
1–8tRNA–Arg(acg)c[12373,12448]
gcaggattagttcaaatggaaagagcaacagtctacgaagctgttaatag
gggttcgaatcccttatcctgcgcca
1–9tRNA–Leu(caa)c[12454,12531]

gttccagtatcccaattggcagaggatgcaagctcaaaccttgtattagt
gacggttcgaatccgtcttggaacacca
1−10tRNA−Val(tac)c[12533,12607]
actcgcttagtttatatggtaaaacatcacccttacaagatgaagaaaaa
ggttcaagtcctttagtgagtacca
1−11tRNA−Thr(tgt)c[12704,12779]
gctcctatcgtataattggttattacggttgccttgtaagcaacttatca
gggttcgagtccttgtgggagcacca
1−12tRNA−Gly(tcc)c[12787,12861]
gcatccatagtttaaacgggaaaattacagtcttccaaactgaggttgag
ggttcgattccctctggatgctcca
1−13tRNA−Ala(tgc)c[12868,12943]
ggggtcatagtttatatggttaaaattcgagttttgcaaacttgggaact
gagttcaattctcagtgactccacca
1−14tRNA−Lys(ctt)c[12950,13025]
gcaggtgtagcaaaatggttatgcggctgactcttaatcagtaagacgat
gggttcaattccctccacctgtacca
1−15tRNA−Leu(tag)c[13033,13109]
gcacctatagcccaactggtagaggcagcagacttagaatctgctcagtg
tgagttcgaatctctctaggtgtacca
1−16tRNA−Ser(tga)c[13364,13453]
ggtaggtagcggctaatggcagccaaacagtcttgaaaactgttgccact
gtagagatacggtgagggttcgactcctttacttaccgcc
1−17tRNA−Arg(tct)c[13692,13768]
gcacccttagttcaattggatagagcaacggtcttctaaatcgttagtta
caggttcgaatcctgtagggtgtacca
1−18tRNA−Ile(gat)c[14015,14090]
gctggtatagttaagaaggttataacactcccctgataagggaacatcgg
tggttcgattccacctaccagtacca
1−19tRNA−Met(cat)c[14092,14168]
ggttcagtcgcagataaggtaatgcaagggtctcataagccctatgaatg
tgggttcgattcccatctgaacctcca
1−20tRNA−Lys(ttt)c[14173,14248]
ggaagtgtagcagaatggtgatgcggcagacttttaatctgacaggcgat
gggttcgaatccctccacttctacca
1−21tRNA−Asp(gtc)c[14678,14754]
ggctatgtagtttaactggagaaaatactcccctgtcacgggagatgatg
tgagttcaagtctcatcgtagccgcca
1−22tRNA−Tyr(gta)c[14760,14847]
gtgtcgttatcccgtagatggtagcggtggggactgtaaatcccttgtca
ttgagactctgtaggttcgactcctacacggcacacca
1−23tRNA−Asn(gtt)c[14857,14933]
ggttaggaagcacataaggtatgtgcggtcgcctgttaagcgaatggcac
agggttcgaatccctgactaaccgcca

1–24tRNA–Met(cat)c[15011,15087]
tgcgggtatagagaaagggcgtctcacatgtctcattagcatggtatcgg
caggttcgactcctgcacccgcctcca
1–25tRNA–Glu(ttc)c[15179,15256]
gttccagtagacaaaatggtaaagtcaccactctttcaaagtggatattt
gagggttcaaatcccttctggaacgcca
1–26tRNA–Pro(tgg)c[15264,15340]
ctctgtttagctcagcttggtagagcgttccgtttggggcggtaaggccg
gaggttcaagtcctccaacagagacca
>KT321315.1 Enterobacter phage phiEap–3, complete genome
1–1tRNA–Met(cat)[48303,48379]
ggccccttagctcaataggtagagctaatcactcataatggttaggttcc
cggttcaaatcacgggagggggccacca
>KT001919.1 Klebsiella phage Miro, complete genome
1–1tRNA–Met(cat)[49904,49980]
ggccccttagctcaataggtagagctaatcactcataatggttaggttcc
cggttcaagtcacgggagggggccacca
>MF044457.1 Escherichia phage ST0, complete genome
1–1tRNA–Gln(ttg)[155681,155754]
tgggaattagccaagttggtaaggcactggattttgattccaggatgcaa
aggttcgagtcctttattcccagc
1–2tRNA–Leu(taa)[155755,155841]
gcgagaatggccaaattggtaaaggcacagcacttaaaatgctgcggaat
gatttccttgtgggttcgagtcccacttctcgcacca
1–3tRNA–Gly(tcc)[155847,155920]
gcggatatcgtataatggcattacctcagacttccaatctgatgatgtga
gttcgattctcattatccgctcca
1–4tRNA–Pro(tgg)[155931,156005]
ctccgtgtagctcagtttggtagagcgcctgatttgggatcaggaggtcc
aaggttcaaatccttgtatggagac
1–5tRNA–Ser(tga)[156007,156096]
ggaggcgtggcagagtggtttaatgcaccggtcttgaaaaccggcagtcg
ctccggcgactcataggttcaaatcctatcgcctccgcca
1–6tRNA–Thr(tgt)[156102,156177]
gctgatttagctcagtaggtagagcaactcacttgtaatgagaaggtcgg
cggttcgattccgtcaatcagcacca
1–7tRNA–Met(cat)[156179,156253]
ggccctgtagctggaaggttcaagcaagcgactcataatcgccagatggt
ggttcaattccacccagggccacca
1–8tRNA–Tyr(gta)[156266,156352]
ggggagttatcccgtagaggtagcggtgtggactgtaaatccattgtcat
tgcgactcgggtggttcgactccatcactccccacca
1–9tRNA–Asn(gtt)[156357,156431]
ggatgtgtagctcaatggcagagcgatcgcctgttaagcgattggttata

ggttcgaatcctatcacgtccgcca
1-10tRNA-Arg(tct)[156436,156511]
cgaggcatagctcagaaggaagagcaaggaccttctaagtcctaggtcgt
aggttcgatccctactgtctcgacca
>MF044458.1 Escherichia phage ST32, complete genome
1-1tRNA-Leu(cag)c[37452,37546]
ggacgagtagctcagaaggtttgcattgtgctcagtcaggtagagcaatc
tgttcgacagatgtgtcgttggttcgaatccaacctcgtccgcca
>JN204348.1 Mycobacterium phage Sebata, complete genome
1-1tRNA-Ser(gct)[32038,32121]
ggagggtgagcatctggtgatgcaggggtcctgctaaggccctacggatt
cacacccgtgagtttcgattactcctccctccgc
1-2tRNA-Leu(cag)[32218,32294]
gccctgctgagcaaactggcaaagctgccgcattcagagtgcgggtcatt
tccgggttcgactcccgggcagggtac
1-3tRNA-Leu(gag)[32414,32488]
gtctctgtaggcaaatcggaaaagccgccatcttgagggggtggtgcgtg
cgggttcgactcccgccagagacac
1-4tRNA-Leu(caa)[32489,32562]
gccgtggtaggccatctggcgagccgccgagttcaagtttcggtgtttgc
gggttcgaatcccgcccacggtac
1-5tRNA-SeC(tca)[68899,68995]
attctggcactggtgggcgagcccaccggcggcagcttcaagctgtcgct
ggccggagaaccgaccggaaacatcccgttcaacgcgacccagggcc
1-6tRNA-Pro(tgg)[92897,92971]
cggggtgtagttcagtttggaagagcgcttggtttgggaccaagatgtcg
caggttcgaatcctgtcaccccgac
1-7tRNA-Trp(cca)[92986,93056]
gggtctgtgcacagggtgcccgacggtctccaaagccgaaggcgggggtt
cgattccctccaggcctgcca
1-8tRNA-Pyl(cta)[94193,94265]
gcaccatttgctcaatggcagagcggcggcttctaaaaccgtgagtgtcg
gttcgactccggcatggtgcacc
1-9tRNA-Met(cat)[94415,94489]
agcggtgtagagcagctaggtagctcgccgggctcatgacccggaggacg
cgtgttcgattcacgccaccgccac
1-10tRNA-Cys(gca)[94616,94687]
gcgcctttggcggaatggctacgtgctcggctgcaacccgagttatcccg
gttcgactccgggagggcgctc
1-11tRNA-Glu(ctc)[94755,94826]
gctcccatggggtagtggtaaccctcctggttctcagccaggcgtcccga
gttcgatcctcggtgggagtgc
1-12tRNA-His(gtg)[94828,94901]
gtggccgtagttcagccggtagaacgctgggttgtgatcccagtcgtcga

gggttcgagtccctccggtcaccc
1–13tRNA–Ala(tgc)[95065,95138]
gggcctatagctcatctggtagagcgcctgccttgcaagcaggaggcggc
aggttcaagtcctgttaggtccac
1–14tRNA–Phe(gaa)[95327,95399]
gccgtcatagctcagttggtagagcaccggcctgaaaacccggtggccga
ggttcgagtcctcgtgtcggcac
1–15tRNA–Val(cac)[95405,95478]
gtccgtttagctcagctggaagagcgctcggtccacacccgagaggccgc
aggttcgatccctgcaatggacac
1–16tRNA–Lys(ctt)[95597,95669]
gccttcgtagctcagtggtagagctgtcgcctcttaagcgataggtcgtt
ggttcaaatccagccgggggcac
1–17tRNA–Glu(ttc)[95673,95747]
ggtcgggtggtctgttggcaggccggtcgggttttcaccccggacattcg
cgggttcaattcccgtcccgatcgc
1–18tRNA–Gly(tcc)[95827,95899]
gcgggtgtggccgaatggctcaggcaccagatttccactctggctacgca
ggttcgattcctgtcatccgctc
1–19tRNA–Thr(cgt)[95981,96055]
gctgctgtagctcacctggtagagcgtcggcgtcgtatcccgaaggcatc
cggttcgagtccggacagcagcccc
1–20tRNA–Thr(tgt)[96056,96128]
gcctctgtggtccagcggcacggacatccgccttgtaagcggaggacccc
cgttcgatccggggtagaggctc
1–21tRNA–Thr(ggt)[96536,96608]
gctgggttagctcagtggtagagcgttcctctggtatgggaaaggcccgg
ggttcaatcccccgactcagctc
1–22tRNA–Gly(gcc)[97931,98004]
gcgaaggtagctcagctggcagagcgccaccttgccaaggtggaggtcgc
gggatcgtaacccgttcttcgctc
1–23tRNA–Asp(gtc)[98008,98080]
ggccctgtagctcagaggaagagcgccggtctgtcgaatcggaggtcgcg
gtatcgtaatccgtcagggtcgc
1–24tRNA–Met(cat)[98140,98212]
gcctcactagctcattggtagagccgctcgctcataacgtgcaggtacct
ggttcgattccagggtgaggtac
1–25tRNA–Ile(gat)[98218,98292]
gcctgttagcggactggtcgtccgatccaagctgataacttggcgtaagc
ggtgttcgattcaccgagcaggtac
1–26tRNA–Arg(acg)[98386,98458]
gcctctatggtccaacggatatgacgccggtctacggaaccggagatgcg
tgttcgattcgcgctaggggcac
1–27tRNA–Val(gac)[98501,98573]

gtccgtgtagctcaggggtagagcgcctgctcgacacgcaggaggaccga
ggttcgaaacctcgcatggacac
1–28tRNA–Arg(cct)[98747,98820]
gcctctgtagctcaacggacagagcaacgcggtcctaacgcggtggctgg
aggttcgaatcctctcggaggcac
1–30tRNA–Gln(ttg)[99361,99436]
tggggtatggtggcaatctggcagtcccgccggactttgactccggaggt
gcaggttcgagtcctgctaccccatc
1–31tRNA–Arg(tct)[99440,99515]
gcccttgtagctcagtggacagagcggcgagcttctacctcgcgggccgg
gagttcgaatctctccaggggcacca
1–32tRNA–Gln(ctg)[126655,126729]
tgctcgttggtgtaactggcaacactacggactctgactccgtcatttta
ggttcgaatcctaagcgagcagcca
1–33tRNA–Asn(gtt)[126736,126809]
tggggtgtccgttaatcaggcaaacgagcggactgttaatccgcccctgc
aggttcgaatcctgccaccccagc
>MF042360.1 Pseudomonas phage Phabio, complete genome
1–1tRNA–Ser(tga)c[294586,294674]
ggagagatggccgagtggcttaaggcggcggtcttgaaaaccgcaggtcc
tatcgggaccctagagttcgaatctctatctctccgcca
1–2tRNA–Leu(taa)c[294680,294765]
acgggattggtggaatcggtagacacaccagacttaaaatctgttgctct
tagagcgtgcgagttcgagtctcgcatcccgtacca
1–3tRNA–Asn(gtt)c[296411,296486]
ttcgagatagctcagttggtagagcagcggactgttaatccgttggtccc
aggttcgaaccctggtctcggagcca
>MF036691.1 Serratia phage CBH8, complete genome
1–1tRNA–Leu(taa)c[70949,71033]
gcatcgatggtggaactggtatacacaggagacttaaaatctcccgccgc
aaggattgagggttcgaatccctctcggtgcacca
1–2tRNA–Arg(tct)c[71039,71115]
cgaggcatagctcagttggatagagcagtggacttctaatccaccggtcg
aaagttcgaatctttctgtctcgacca
1–3tRNA–Met(cat)c[72200,72275]
ggccctgtagctcagcggtagagcgctcccctcataagggattggtcact
ggttcgaatctcagtcagggtcacca
1–4tRNA–Gly(tcc)c[72662,72736]
gcatccatcgtatagcggctattatgactggcttccacccagtagatgag
agttcgattctctctggatgctcca
1–5tRNA–Trp(cca)c[72738,72811]
aggtctctcgtatagtggtattaccctgagctccaacctcagtgacgtgg
gttcaattcctacggggcctgcca
1–6tRNA–Ile(gat)c[72980,73055]

gcgaatatagctcagttggtagagcaaccgcccgataagcggtaggtccc
tggttcgagcccaggtattcgcacca
1-7tRNA-Pro(tgg)c[73060,73137]
ctccgtgtagctcagtttggtagcagcgcctggtttgggaccaggaggtc
caaggttcaaatccttgcatggagacca
1-8tRNA-Ser(tga)c[73410,73503]
ggaagattggcagagtctggctaattgcaccacacttgaaatgcggaggt
cctggtgacaggtcccgtgagttcgaatctcacatcttcctcca
1-9tRNA-His(gtg)c[73508,73583]
gtggccgtagttcagttggtagaacccctaattgtgattcaggatgtcgt
ggattcgagttccaccggtcacccca
1-10tRNA-Gln(ttg)c[73680,73755]
tagggtatagccaagttggtaaggcaggagattttgattctcccatgctc
tggttcgagtccagataccctagcca
1-11tRNA-Met(cat)c[74196,74272]
tgcgggttaacttcagttggtagaatgacgggttcatatcccgttacgcg
atggttcgagtccatcacccgcctcca
1-12tRNA-Asn(gtt)c[74398,74481]
gggtagttggctgagcggtttaagcggcggactgttaatccgtgtcgaaa
gacaacgtaggttcgaatcctacactacccgcca
1-13tRNA-Lys(ttt)c[74489,74565]
gggatattagctcagttggttagagcaccggacttttaatccgggtgtcc
atggttcgagtccatgatgtcccacca
1-14tRNA-Glu(ttc)c[74571,74645]
gctctgttcgtctatcggttaggacgcctgcctttcacgcaggaaagagg
agttcaattctcctacagagtacca
1-15tRNA-Asp(gtc)c[74650,74725]
ggacctatagtttcagcggttaaaatactcccctgtcacgggagcgtcac
gagttcgaatctcgttaggtccgcca
1-16tRNA-Tyr(gta)c[74732,74820]
gcgttgatgacccgagtggcaaagggtggagactgtaaatcttctgcttg
taatgagcttcgtaggttcgagtccttctcggcgcacca
>MF036690.1 Serratia phage CHI14, complete genome
1-1tRNA-Leu(taa)c[70949,71033]
gcatcgatggtggaactggtatacacaggagacttaaaatctcccgccgc
aaggattgagggttcgaatccctctcggtgcacca
1-2tRNA-Arg(tct)c[71039,71115]
cgaggcatagctcagttggatagagcagtggacttctaatccaccggtcg
aaagttcgaatctttctgtctcgacca
1-3tRNA-Met(cat)c[72200,72275]
ggccctgtagctcagcggtagagcgctcccctcataagggattggtcact
ggttcgaatctcagtcagggtcacca
1-4tRNA-Gly(tcc)c[72662,72736]
gcatccatcgtatagcggctattatgactggcttccacccagtagatgag

agttcgattctctctggatgctcca
1–5tRNA–Trp(cca)c[72738,72811]
aggtctctcgtatagtggtattaccctgagctccaacctcagtgacgtgg
gttcaattcctacggggcctgcca
1–6tRNA–Ile(gat)c[72980,73055]
gcgaatatagctcagttggtagagcaaccgcccgataagcggtaggtccc
tggttcgagcccaggtattcgcacca
1–7tRNA–Pro(tgg)c[73060,73137]
ctccgtgtagctcagtttggtagcagcgcctggtttgggaccaggaggtc
caaggttcaaatccttgcatggagacca
1–8tRNA–Ser(tga)c[73410,73503]
ggaagattggcagagtctggctaattgcaccacacttgaaatgcggaggt
cctggtgacaggtcccgtgagttcgaatctcacatcttcctcca
1–9tRNA–His(gtg)c[73508,73583]
gtggccgtagttcagttggtagaacccctaattgtgattcaggatgtcgt
ggattcgagttccaccggtcacccca
1–10tRNA–Gln(ttg)c[73680,73755]
tagggtatagccaagttggtaaggcaggagattttgattctcccatgctc
tggttcgagtccagataccctagcca
1–11tRNA–Met(cat)c[74196,74272]
tgcgggttaacttcagttggtagaatgacgggttcatatcccgttacgcg
atggttcgagtccatcacccgcctcca
1–12tRNA–Asn(gtt)c[74398,74481]
gggtagttggctgagcggtttaagcggcggactgttaatccgtgtcgaaa
gacaacgtaggttcgaatcctacactacccgcca
1–13tRNA–Lys(ttt)c[74489,74565]
gggatattagctcagttggttagagcaccggacttttaatccgggtgtcc
atggttcgagtccatgatgtcccacca
1–14tRNA–Glu(ttc)c[74571,74645]
gctctgttcgtctatcggttaggacgcctgcctttcacgcaggaaagagg
agttcaattctcctacagagtacca
1–15tRNA–Asp(gtc)c[74650,74725]
ggacctatagtttcagcggttaaaatactcccctgtcacgggagcgtcac
gagttcgaatctcgttaggtccgcca
1–16tRNA–Tyr(gta)c[74732,74820]
gcgttgatgacccgagtggcaaagggtggagactgtaaatcttctgcttg
taatgagcttcgtaggttcgagtccttctcggcgcacca
>MF036692.1 Serratia phage X20, complete genome
1–1tRNA–Thr(tgt)c[70617,70692]
gccgatttagctcagtaggtagagcagctcacttgtaatgagaaggtcgt
gggttcaattcctacaaccagcacca
1–2tRNA–Arg(tct)c[71236,71311]
gctcttatagtgtaatggatagcacacgatcgttctaaggtcggtagtct
gggttcgagtcctagtaggagcacca

1-3tRNA-Leu(taa)c[71313,71399]
gcgagaatggtcaaattggtaaaggcacggcacttaaaatgccgcggagg
gttctccttgtgggttcgagtcccacttctcgcacca
1-4tRNA-Met(cat)c[72484,72559]
ggccctgtagctcagcggtagagcgctcccctcataagggattggtcact
ggttcgaatctcagtcagggtcacca
1-5tRNA-Gly(tcc)c[72946,73019]
gcgtctatagtataatggaattatgactggcttccacccagtagatgaga
gttcaattctccctagacgctcca
1-6tRNA-Trp(cca)c[73027,73100]
aggtctctcgtatagtggtattaccctgagctccaacctcagtgacgtgg
gttcaattcctacggggcctgcca
1-7tRNA-Ile(gat)c[73269,73344]
gcgaatatagctcagttggtagagcaaccgcccgataagcggtaggtccc
tggttcgagcccaggtattcgcacca
1-8tRNA-Pro(tgg)c[73349,73426]
ctccgtgtagctcagtttggtagcagcgcctggtttgggaccaggaggtc
caaggttcaaatccttgcatggagacca
1-9tRNA-Ser(tga)c[73699,73790]
ggaagattggcagagaggctaattgcaccgcacttgaaatgcggaggtcc
tggcgacaggtcccgtgagttcgaatctcacatcttcctcca
1-10tRNA-His(gtg)c[73795,73870]
gtggccgtagttcagttggtagaacccctaattgtgattcaggatgtcgt
ggattcgagttccaccggccaccccca
1-11tRNA-Gln(ttg)c[73967,74042]
tagggtatagccaagttggtaaggcaggagattttgattctcccatgctc
tggttcgagtccagataccctagcca
1-12tRNA-Met(cat)c[74482,74558]
tgcgggttaacttcagttggtagaatgacgggttcatatcccgttacgcg
atggttcgagtccatcacccgcctcca
1-13tRNA-Asn(gtt)c[74684,74767]
gggtagttggctgagcggtttaagcggcggactgttaatccgtgtcgaaa
gacaacgtaggttcgaatcctacactacccgcca
1-14tRNA-Lys(ttt)c[74775,74851]
gggatattagctcagttggttagagcaccggacttttaatccgggtgtcc
atggttcgagtccatgatgtcccacca
1-15tRNA-Glu(ttc)c[74857,74931]
gctctgttcgtctatcggttaggacaccaggttttcaacctgggaagagg
agttcaattctcctacagagtacca
1-16tRNA-Asp(gtc)c[74936,75012]
ggtcctatagtttcaattggttaaaatactcccctgtcacgggaatgtta
cgagttcgagtctcgttaggaccgcca
1-17tRNA-Tyr(gta)c[75019,75107]
gcgtcgatgacccgagtggcaaagggtggagactgtaaatctcctgcttg

taatgagcttcgtaggttcgagtccttcacggcgcacca
>KY979109.1 Salmonella phage SHP1, complete genome
1-1tRNA-Met(cat)[6863,6936]
ggccctgtagctggacggtcaagcgagcgactcataatcgctggatggtg
gttcgattccacccagggccacca
1-2tRNA-Asn(gtt)[6941,7015]
ggatgtgtagctcagtggtagagcagttgactgttaatcaattggtccat
ggttcgaatccatgcatgtccgcca
1-3tRNA-Arg(tct)[7020,7095]
cgaggcatagctcagaaggaagagcaaggaccttctaagtcctgggtcgt
aggttcgatccctactgtctcggcca
>MF370225.1 Salmonella phage ST11, complete genome
1-1tRNA-Pro(tgg)[22896,22972]
ctctgtttagctcagcttggtagagcgttccgtttggggcggtaaggccg
gaggttcaagtcctccaacagagacca
1-2tRNA-Glu(ttc)[22980,23057]
gttccagtagacaaaatggtatagtcaccactctttcaaagtggatattt
gagggttcaaatcccttctggaacgcca
1-3tRNA-Met(cat)[23149,23225]
tgcgggtatagagaaagggcgtctcacatgtctcattagcatggtatcgg
caggttcgactcctgcacccgcctcca
1-4tRNA-Asn(gtt)[23309,23385]
ggttaggaagcacataaggtatgtgcggtcgcctgttaagcgaatggcac
agggttcgaatccctgactaaccgcca
1-5tRNA-Tyr(gta)[23455,23542]
gtgtcgttatcccgtagatggtagcggtggggactgtaaatcccttgtca
ttgagactcggtaggttcgactcctacacggcacacca
1-6tRNA-Asp(gtc)[23548,23625]
ggttatgtagtttaatttggttaaaatactcccctgtcacgggagatgat
gagggttcaaatcccttcgtaaccgcca
1-7tRNA-Lys(ttt)[24052,24127]
ggaagtgtagcagaatggtgatgcggcagacttttaatctgacaggcgat
gggttcgaatccctccacttctacca
1-8tRNA-Met(cat)[24132,24207]
ggttcagtcgcagataaggtaatgcaagggtctcataagcctatgaatgt
gggttcgattcccatctgaacctcca
1-9tRNA-Ile(gat)[24210,24284]
tgtgggttagcataaatggtaatgcaaacggctgataaccgttagaagag
ggttcgataccctcacctacaacca
1-10tRNA-Ser(tga)[24999,25088]
ggtaggtagcggctaatggtagccaaactgtcttgaaaacagttgccact
gtagagatacggtgagggttcgactcctttacttaccgcc
1-11tRNA-Leu(tag)[25344,25421]
gggagattgacggtaattggtaaacctatctcgcttagaacgagatgttt

gagggttcgaatcccttgtctcctacca
1-12tRNA-Lys(ctt)[25429,25504]
gcaggtgtagcaaaatggttatgcggctgactcttaatcagtaagacgat
gggttcaattccctccacctgtacca
1-13tRNA-Ala(tgc)[25511,25586]
tgggtcatagtttatatggttaaaattcgagttttgcaaacttgggaact
gagttcaattctcagtgactccacca
1-14tRNA-Gly(tcc)[25593,25667]
gcatccatagtttaaacgggaaaattacagtcttccaaactgaggttgag
ggttcgattccctctggatgctcca
1-15tRNA-Thr(tgt)[25876,25952]
gctgctttcgtataattggctattacacatcccttgtaaggatggaaatg
caggttcgagtcctgtgagcagcacca
1-16tRNA-Val(tac)[26048,26122]
actcgcttagtttatatggtaaaacatcacccttacaagatgaagaaaaa
ggttcaagtcctttagtgagtacca
1-17tRNA-Leu(caa)[26124,26201]
gttccagtatcccaattggcagaggatgcaagctcaaaccttgtattagt
gacggttcgaatccgtcttggaacacca
1-18tRNA-Gln(ttg)[26886,26961]
aggggattagtttacaaggttaaaacctcggtctttgaaatcgaagaagt
tggttcaattccaacatcccccgcca
1-19tRNA-Leu(taa)[26964,27042]
gctccattactccaattggcagagaggccagacttaaaatctgtgttatg
tatcggttcgaatccgatatggagtacca
1-20tRNA-Gln(ctg)[27048,27123]
agcggtatagcataactggcaatgcagcagtctctgaagctgtcctatta
aggttcaaatccttatgccgctgcca
1-21tRNA-His(gtg)[27155,27230]
gtggccttatcataaacggtaatgacccatgctgtgaacatggtctatac
gggttcaaatcccgtaggtcaccccca
1-22tRNA-Phe(gaa)[27237,27312]
agtccaagtagcttatatggttaaagcgcgtgtctgaaaaacatgagaag
agggttcaaatcccactggactacca
1-23tRNA-Ser(gct)[28065,28157]
ggaagattaaccctaaaaggtaagggagcagtttgctaaactgccagtag
ctgagaaatcggtgtaccagttcaagtctggtatcttcctcca
1-24tRNA-Cys(gca)[28162,28237]
gaatccgtgacagaaatggatatgtgcctgtctgcaaaacaggtttataa
gggctcaagtcccttcggattctcca
>MF351863.1 Synechococcus phage Bellamy, complete genome
1-1tRNA-Leu(taa)[23706,23791]
tgggagcgtggcggaatcggtagacgcaccagacttaaaatctgttgaga
attatctcgtgggggttcaagtccccccgctcctat

1−2tRNA−Thr(tgt)[24100,24173]
gccaacttagctcagctggatagagcagggtttttgtaaagctcaggtca
tcggttcaagtccgatagttggct
1−3tRNA−Ala(tgc)[143368,143440]
ggggttatagctcagttggtagagcgcctgctttgcaagcaggatgtcag
cggttcgagtccgcttaactcca
1−4tRNA−Arg(tct)[155413,155487]
gactcgctagctcagttggatagagcaactgccttctaagcagtcggtcg
aaggttcgagtccttcgcgagtcgc
1−5tRNA−Gly(tcc)[155539,155609]
gcgagtgtggtgtagcggtaacatgcgagccttccaagctcttgtcacgg
gttcgatccccgtcactcgct
1−6tRNA−Asn(gtt)[155736,155808]
tcctctatagctcagttggtagagcaggtgactgttaatcaccctgtccc
tggttcgagtccaggtggaggag
1−7tRNA−Val(tac)[155812,155883]
gggagtttggcgcagcggtagcgcatctgctttacacgcagctggtcact
ggttcgatcccagtaactccca
1−8tRNA−Ser(tga)[156157,156243]
ggaagagtggtcgagtggtttatggcactggtcttgaaaaccagcgaggg
tgcaagtcctccgtgggttcaaatcccacctcttccg
1−9tRNA−Pro(tgg)[156693,156766]
cggggtgtagctcagtttggtagagcactcgctttgggagcgagtggccg
aaggttcaaatcctttcaccccga
1−10tRNA−Met(cat)[156959,157031]
gggggtttagcaatctggtgaatgcagcaaactcataatttgcctaaggt
gagttcgatcctcacaacccta
>MF370964.1 Pseudoalteromonas phage SL20, complete genome
1−1tRNA−Lys(ttt)c[2264,2333]
gccttcgtagctaactagtaagcgacttacttttaatgaggatatgttgg
ttcaattccaaccgaaggca
1−2tRNA−Arg(tct)c[2335,2411]
gcaagagtagaacaattggatagttcatctcccttctaaggagacggttg
taagttcgaatcttacctcttgtgcca
1−3tRNA−Val(cac)c[41928,41998]
atttcctctggtgtaaggaaaccactgtattcacgttccagtaaactttt
gttccaatcctgaatgaaatc
>MF448340.1 Aeromonas phage AS−zj, complete genome
1−1tRNA−Thr(tgt)c[168891,168966]
gccgaattagctcatctggtagagcacagcctttgtaacgctggggtggt
ccgttcgagtcggacattcggcacca
1−2tRNA−Pro(tgg)c[169245,169320]
cggtgattagcgcagttggtagcgcgtctggtttgggaccagagggtcac
aggttcgagtcctgtatcaccgacca

1–3tRNA–Tyr(gta)c[169475,169565]
ggatgttcggctacgatggcggtgtagcggcggactgtaaatccgttccc
tctgggtaaacaagttggttcgaatccaacagcatccacca
1–4tRNA–Asn(gtt)c[169572,169647]
tggcgtgtagttcagtaggtagaacagcggactgttaatccgtatgtcgt
gggttcgaatcccacctcgccagcca
1–5tRNA–Met(cat)c[169649,169726]
agcggggtggagaagtcaggagttctcgccagactcattatctggaaatc
ggtggttcaaatccacctcccgcttcca
1–6tRNA–Met(cat)c[170108,170184]
ggccccttagctcagttggttagagcaaccgactcataatcggttggtca
ctggttcgagtccagtaggggccacca
1–7tRNA–Gly(tcc)c[170269,170344]
gcgggtaaggtgtttatggatacatgcaagccttccaagtttgagtagac
cggatcgttaccggctacccgctcca
1–8tRNA–Leu(tag)c[170346,170429]
cgggatgtggtgaaattggcagacacactagatttaggttctagcgccat
aggcgtgacggttcgagtccgtccatcccgacca
1–9tRNA–Arg(tct)c[170430,170506]
gcgctgttagctcagttggatagagcaacgttcttctaaagcgtgggtca
ctggttcgaatccagtacggcgcacca
1–10tRNA–Leu(taa)c[171527,171619]
gcgtgagtggcgaagttggttgaaacgcatcggacttaaaatccgatact
gcataatggcacatcgtgggttcgattcccacctcacgcacca
>MF288919.1 Bacillus phage AaronPhadgers, complete genome
1–1tRNA–Asn(gtt)[33350,33423]
gtgcttgtagctcagtcggtagagctggtggctgttaaccactgtgtcgt
aggttcgattcctacctagcacgc
1–2tRNA–Glu(ttc)[33430,33506]
gtcgcattggtgaaattggttaacacactcggctttctaccgaggattta
ggggttcgagccccctatgcgattcca
1–3tRNA–Asp(gtc)[33511,33584]
tggggatatagtgtagaggtaaacacgcacggctgtctaccgtgaagcac
gagttcgaatctcgttatcctcgt
>MF288922.1 Bacillus phage Janet, complete genome
1–1tRNA–Asn(gtt)[32103,32177]
tgtgcttgtagctcagtcggtagagcgccagattgttaatctgagggtcg
caggttcgattcctgcttggcacgt
1–2tRNA–Trp(cca)[32939,33012]
gggagtttagtttagtggtaaaacctcggtctccaaaaccgaagtcatat
gttcgattcgtatagctcctgcca
1–3tRNA–Ser(tga)[33014,33101]
ggagggttggcagagtggattattgcagcggtcttgaaaaccgccgaggg
taaaacctccgtgagttcaaatctcacaccctcctcca

1-4tRNA-Asp(gtc)[33103,33176]
tggggatatagtgtagaggtaaacacgcacggctgtctaccgtgaagcac
gggttcgaatcccgttatcctcgt
1-5tRNA-Leu(tag)[33321,33398]
tgccgaagtaatccaatcaggtagagatagcggttttagaaaccgtccag
tgtgggttcgaccccctccttcggtatc
1-6tRNA-Tyr(gta)[33405,33488]
tggcagaatagtcaagcggcaacgacaggtgactgtaaatcacccctcat
tcgagttcgtaggttcgagtcctgcttctgctat
1-7tRNA-Ser(gct)[34401,34487]
ggaggggtactcaagcggcgaagagggggacttgctaagtctctagtacc
ttaacaggtagcgagggttcgaatcccttcccctcct
>MF288921.1 Bacillus phage OTooleKemple52, complete genome
1-1tRNA-Asn(gtt)[31940,32014]
tgtgcttgtagctcagtcggtagagctggtggctgttaaccactgtgtcg
ttggttcgattccaacctagcacgt
1-2tRNA-Trp(cca)[32179,32252]
gggagtttagtttagtggtaaaacctcggtctccaaaaccgaagtcatat
gttcgattcgtatagctcctgcca
1-3tRNA-Ser(tga)[32254,32341]
ggagggttggcagagtggattattgcagcggtcttgaaaaccgacgaggg
taaaacctccgtgagttcaaatctcacaccctcctcca
1-4tRNA-Asp(gtc)[32343,32416]
tggggatatagtgtagaggtaaacacgcacggctgtctaccgtgaagcac
gggttcgaatcccgttatcctcgt
1-5tRNA-Leu(tag)[32639,32716]
tgccgaagtaatccaatcaggtagagatagcggttttagaaaccgtccag
tgtgggttcgaccccctccttcggtatc
1-6tRNA-Tyr(gta)[32723,32806]
tggcagaatagtcaagcggcaacgacaggtgactgtaaatcacccctcat
ttgagttcgtaggttcgagtcctgcttctgctat
1-7tRNA-Ser(gct)[33719,33806]
ggagaggtactcaagcggtgaagaggttagtttgctaaactaataggtcg
agtaatcggcgcgagggttcgaatcccttcccctccac
>MF285619.1 Serratia phage 2050H1, complete genome
1-1tRNA-Met(cat)c[20552,20627]
agtcccttagctcaggggttagagcatgcgactcataatcgcttggtcgt
tggttcaaagccaacagggactacca
1-2tRNA-Cys(aca)c[22254,22345]
tggccgggtagctcaattggtagagtggcgcgcgtacagcctgaggcgac
attagcgcgatgttgtggcttcgagaaccaccccggccaccc
>MF285618.1 Serratia phage 2050HW, complete genome
1-1tRNA-Tyr(gta)[265275,265359]
ggtaggttactcaagcggcaacgagaacagactgtaaatctgttgccttc

gggcttcgatggttcgagtccatcacctaccacca

>MF498901.1 Bacillus phage Anthony, complete genome

1-1tRNA-Asn(gtt)[32463,32537]

tgtgcttgtagctcagtcggtagagctggtggctgttaaccactgtgtcg

ttggttcgattccaacctagcacgt

1-2tRNA-Trp(cca)[32704,32777]

gggagtttagtttagtggtaaaacctcggtctccaaaaccgaagtcatat

gttcgattcgtatagctcctgcca

1-3tRNA-Ser(tga)[32779,32866]

ggagggttggcagagtggattattgcagcggtcttgaaaaccgccgaggg

taaaacctccgtgagttcaaatctcacaccctcctcca

1-4tRNA-Leu(tag)[33587,33664]

tgccgaagtaatccaatcaggtagagatagcggttttagaaaccgtccag

tgtgggttcgaccccctccttcggtatc

1-5tRNA-Tyr(gta)[33671,33754]

tggcagaatagtcaagcggcaacgacaggtgactgtaaatcacccctcat

tcgagttctcaggttcgagtcctgattctgctat

1-6tRNA-Ser(gct)[34667,34753]

ggaggggtactcaagcggcgaagagggggacttgctaagtctctagtacc

ttaacaggtagcgagggttcgaatcccttcccctcct

>MF158040.1 Shigella phage Sf13, complete genome

1-1tRNA-Cys(gca)c[11656,11731]

gtgtcggtgtcagaaatggttatgtgcctgtctgcaaaacaggtttatga

gggttcgagtcccttccgatactcca

1-2tRNA-Ser(gct)c[11738,11830]

ggaagattaaccctaaaaggtaagggagcagtttgctaaactgccagtaa

ccgagaaatcggcgtaccagttcaagtctggtatcttcctcca

1-3tRNA-Phe(gaa)c[12583,12658]

agctcaattagcttatatggttaaagcgcgtgtctgaaaaacatgagaac

agggttcaaatcccgatgggctacca

1-4tRNA-His(gtg)c[12666,12742]

gtggccttatcataaatggataatgacccatgctgtgaacatggtctata

cgggttcgattcccgtaggtcacccca

1-5tRNA-Gln(ctg)c[12774,12849]

agcagtatagcataactggcaatgctccagcctctgaagctgaaagatta

aggttcaaatccttatactgctgcca

1-6tRNA-Leu(taa)c[12856,12934]

gctccattactccaattggcagagaggccagatttaaaatctgtgttatg

tatcggttcgaatccgatatggagtacca

1-7tRNA-Gln(ttg)c[12937,13012]

aggggattagtttacaaggttaaaacctcggtctttgaaatcgaagaaga

tggttcaattccatcatcccccgcca

1-8tRNA-Arg(acg)c[13021,13097]

gcagaattagttcaaatggacagagcagcaacctacgaagttgttagtta

ggggttcgaatcctctattctgcacca
1−9tRNA−Leu(caa)c[13276,13353]
gttccagtatcccaattggcagaggatgcaagctcaaaccttgtattagt
gacggttcgaatccgtcttggaacacca
1−10tRNA−Val(tac)c[13355,13429]
gctcgcttagtttatatggtaaagccccatccttacaagttggtgaagaa
ggttcaagtccttcagcgagtacca
1−11tRNA−Thr(tgt)c[13525,13601]
gctgctttcgtataattggctattgcacatcccttgtaaggatggagatg
caggttcgagtcctgtgagcagcacca
1−12tRNA−Gly(tcc)c[13608,13682]
gcatccatagtttaaatgggaaaattacagtcttccaaactgaggttgag
ggttcgattccctctggatgctcca
1−13tRNA−Ala(tgc)c[13689,13764]
ggggatgtagtttacatggttaaaacataagttttgcaaacttaagtaca
gggttcaattccctgcttctccacca
1−14tRNA−Lys(ctt)c[13771,13846]
gcaggtgtagcaaaatggttatgcggctgactcttaatcagtaagacgat
gggttcaattccctccacctgtacca
1−15tRNA−Leu(tag)c[13854,13931]
gggagattgacggtaattggtaaacctaccatccttagaagttggtgttt
gagggttcgaatcccttgtctcctacca
1−16tRNA−Ser(tga)c[14182,14271]
ggtaggtagcggctaatggaagccaaactgtcttgaaaacagttgccatt
gtagtgatacggtgagggttcgactcctttatctaccgcc
1−17tRNA−Arg(tct)c[14533,14609]
gcacccttagttcaaacggatagagcaacggtcttctaaaccgtcagtta
caggttcgaatcctgtagggtgtgcca
1−18tRNA−Ile(gat)c[14858,14933]
gctggtatagttaagaaggttataacactcccctgataagggaatatcgg
tggttcgattccacctaccagtacca
1−19tRNA−Met(cat)c[14936,15012]
ggttcagtcgcagataaggtaatgcaagggtttcataagccctatgaatg
tgggttcgattcccacctgaacctcca
1−20tRNA−Lys(ttt)c[15094,15169]
ggaagtgtagcagaatggtgatgcggcagacttttaatctgacaggcgac
gggttcgaatccctccacttctacca
1−21tRNA−Asp(gtc)c[15620,15696]
ggttatgtagtttaactggtcaaaatacctccctgtcacggaggatgatg
tgagttcgagtctcatcgtaaccgcca
1−22tRNA−Asn(gtt)c[15860,15936]
ggttaggaagcacataaggcatgtgcggtcggctgttaaccgaatggtac
agggttcgaatccctgactaaccgcca
1−23tRNA−Met(cat)c[16020,16096]

tgcgggtatagagaaagggtgtctcacatgtctcattagcatgttatcgg
taggttcgactcctacacccgcctcca
1-24tRNA-Glu(ttc)c[16184,16261]
attcccgtagacaaattaggtaaagtcaacaccctttcaaggtgtggttt
gagggttcaagtcccttcgggaatgcca
1-25tRNA-Pro(tgg)c[16268,16347]
ctccgtttagctcagtttggtttagcagcacttgctttgggagcaagggg
tcgaaggttcaaatccttcaacggagacca
>MF158041.1 Shigella phage Sf15, complete genome
1-1tRNA-Cys(gca)c[67419,67494]
gtgtcggtgtcagaaatggttatgtgcctgtctgcaaaacaggtttatga
gggttcgagtcccttccgatactcca
1-2tRNA-Ser(gct)c[67501,67593]
ggaagattaaccctaaaaggtaagggagcagtttgctaaactgccagtaa
ccgagaaatcggcgtaccagttcaagtctggtatcttcctcca
1-3tRNA-Phe(gaa)c[68346,68421]
agctcaattagcttatatggttaaagcgcgtgtctgaaaaacatgagaac
agggttcaaatcccgatgggctacca
1-4tRNA-His(gtg)c[68429,68505]
gtggccttatcataaatggataatgacccatgctgtgaacatggtctata
cgggttcgattcccgtaggtcacccca
1-5tRNA-Gln(ctg)c[68537,68612]
agcagtatagcataactggcaatgctccagcctctgaagctgaaagatta
aggttcaaatccttatactgctgcca
1-6tRNA-Leu(taa)c[68619,68697]
gctccattactccaattggcagagaggccagatttaaaatctgtgttatg
tatcggttcgaatccgatatggagtacca
1-7tRNA-Gln(ttg)c[68700,68775]
aggggattagtttacaaggttaaaacctcggtctttgaaatcgaagaaga
tggttcaattccatcatcccccgcca
1-8tRNA-Arg(acg)c[68784,68860]
gcagaattagttcaaatggacagagcagcaacctacgaagttgttagtta
ggggttcgaatcctctattctgcacca
1-9tRNA-Leu(caa)c[69039,69116]
gttccagtatcccaattggcagaggatgcaagctcaaaccttgtattagt
gacggttcgaatccgtcttggaacacca
1-10tRNA-Val(tac)c[69118,69192]
gctcgcttagtttatatggtaaagccccatccttacaagttggtgaagaa
ggttcaagtccttcagcgagtacca
1-11tRNA-Thr(tgt)c[69288,69364]
gctgctttcgtataattggctattgcacatcccttgtaaggatggagatg
caggttcgagtcctgtgagcagcacca
1-12tRNA-Gly(tcc)c[69371,69445]
gcatccatagtttaaatgggaaaattacagtcttccaaactgaggttgag

ggttcgattccctctggatgctcca
1-13tRNA-Ala(tgc)c[69452,69527]
ggggatgtagtttacatggttaaaacataagttttgcaaacttaagtaca
gggttcaattccctgcttctccacca
1-14tRNA-Lys(ctt)c[69534,69609]
gcaggtgtagcaaaatggttatgcggctgactcttaatcagtaagacgat
gggttcaattccctccacctgtacca
1-15tRNA-Leu(tag)c[69617,69694]
gggagattgacggtaattggtaaacctaccatccttagaagttggtgttt
gagggttcgaatcccttgtctcctacca
1-16tRNA-Ser(tga)c[69945,70034]
ggtaggtagcggctaatggaagccaaactgtcttgaaaacagttgccatt
gtagtgatacggtgagggttcgactcctttatctaccgcc
1-17tRNA-Arg(tct)c[70296,70372]
gcacccttagttcaaacggatagagcaacggtcttctaaaccgtcagtta
caggttcgaatcctgtagggtgtgcca
1-18tRNA-Ile(gat)c[70621,70696]
gctggtatagttaagaaggttataacactcccctgataagggaatatcgg
tggttcgattccacctaccagtacca
1-19tRNA-Met(cat)c[70699,70775]
ggttcagtcgcagataaggtaatgcaagggtttcataagccctatgaatg
tgggttcgattcccacctgaacctcca
1-20tRNA-Lys(ttt)c[70857,70932]
ggaagtgtagcagaatggtgatgcggcagacttttaatctgacaggcgac
gggttcgaatccctccacttctacca
1-21tRNA-Asp(gtc)c[71357,71433]
ggttatgtagtttaactggtcaaaatacctccctgtcacggaggatgatg
agggttcgaatcccttcgtaaccgcca
1-22tRNA-Tyr(gta)c[71440,71527]
gcgtcgttatcccgtagatggaagcggtggtgactgtaaatcacttgtca
ttatgactcgggaggttcgactccttcacggcgcacca
1-23tRNA-Asn(gtt)c[71597,71673]
ggttaggaagcacataaggcatgtgcggtcggctgttaaccgaatggtac
agggttcgaatccctgactaaccgcca
1-24tRNA-Met(cat)c[71758,71834]
tgcgggtatagagaaagggtgtctcacatgtctcattagcatgttatcgg
taggttcgactcctacacccgcctcca
1-25tRNA-Glu(ttc)c[71922,71999]
attcccgtagacaaattaggtaaagtcaacacccttcaaggtgtggttt
gagggttcaagtcccttcgggaatgcca
1-26tRNA-Pro(tgg)c[72006,72085]
ctccgtttagctcagtttggtttagcagcacttgctttgggagcaagggg
tcgaaggttcaaatccttcaacggagacca
>MF158043.1 Shigella phage Sf16, complete genome

1-1tRNA-Pro(tgg)[16042,16118]
ctccatttagctcagcttggtagagcgttccgtttggggcggtaaggccg
aaggttcaagtccttcaatggagacca
1-2tRNA-Glu(ttc)[16125,16202]
attcccgtagacaaattaggtaaagtcaacaccctttcaaggtgtggttt
gagggttcaagtcccttcgggaatgcca
1-3tRNA-Met(cat)[16290,16366]
tgcgggtatagagaaagggtgtctcacatgtctcattagcatgttatcgg
taggttcgactcctacacccgcctcca
1-4tRNA-Asn(gtt)[16451,16527]
ggttaggaagcacataaggcatgtgcggtcggctgttaaccgaatggtac
agggttcgaatccctgactaaccgcca
1-5tRNA-Asp(gtc)[16691,16767]
ggttatgtagtttaactggtcaaaatacctccctgtcacggaggatgatg
agggttcgaatcccttcgtaaccgcca
1-6tRNA-Lys(ttt)[17218,17293]
ggaagtgtagcagaatggtgatgcggcagacttttaatctgacaggcgat
gggttcgaatccctccacttctacca
1-7tRNA-Met(cat)[17375,17451]
ggttcagtcgcagataaggtaatgcaagggtttcataagccctatgaatg
tgggttcgattcccacctgaacctcca
1-8tRNA-Ile(gat)[17454,17528]
tgtgggttagcataaatggtaatgcaaacggctgataaccgttagaagag
ggttcgataccctcacctacaacca
1-9tRNA-Arg(tct)[17805,17880]
gctccgttagtctaaaggataggcaactgtcttctaaacagttcatatat
aggttcgagtcctatacggagtgcca
1-10tRNA-Ser(tga)[18500,18589]
ggtaggtagcggctaatggaagccaaatagtcttgaaaactattgccatt
gtagagatacggtgagggttcgactcctttatctaccgcc
1-11tRNA-Leu(tag)[18843,18919]
gcacctatagcccaactggaagaggcagcagacttagaatctgctcagtg
tgagttcgaatctctctaggtgtacca
1-12tRNA-Lys(ctt)[18927,19002]
gcaggtgtagcaaaatggttatgcggctgactcttaatcagtaagacgat
gggttcaattccctccacctgtacca
1-13tRNA-Ala(tgc)[19009,19084]
ggggatgtagtttacatggttaaaacataagttttgcaaacttaagtaca
gggttcaattccctgcttctccacca
1-14tRNA-Gly(tcc)[19091,19165]
gcatccatagtttaaacgggaaaattacagtcttccaaactgaggttgag
ggttcgattccctctggatgctcca
1-15tRNA-Thr(tgt)[19172,19247]
gctcttatcgtataattggctattacggttgccttgtaagcaacttatca

gggttcgaatccttgtgggagcacca
1−16tRNA−Val(tac)[19343,19417]
gctcgcttagtttatatggtaaagccccatccttacaagttggtgaagaa
ggttcaagtccttcagcgagtacca
1−17tRNA−Leu(caa)[19419,19496]
gttccagtatcccaattggcagaggatgcaagctcaaaccttgtattagt
gacggttcgaatccgtcttggaacacca
1−18tRNA−Arg(acg)[19675,19751]
gcagaattagttcaaatggacagagcagcaacctacgaagttgttagtta
ggggttcgaatcctctattctgcacca
1−19tRNA−Gln(ttg)[19760,19835]
aggggattagtttacaaggttaaaacctcggtctttgaaatcgaagaaga
tggttcaattccatcatcccccgcca
1−20tRNA−Leu(taa)[19838,19916]
gctccattactccaattggcagagaggccagatttaaaatctgtgttatg
tatcggttcgaatccgatatggagtacca
1−21tRNA−Gln(ctg)[19923,19998]
agcagtatagcataactggcaatgccccagcctctgaagctgaaagatta
aggttcaaatccttatactgctgcca
1−22tRNA−His(gtg)[20030,20106]
gtggccttatcataaatggataatgacccatgctgtgaacatggtctata
cgggttcgattcccgtaggtcacccca
1−23tRNA−Phe(gaa)[20114,20189]
agctcaattagcttatatggttaaagcgcgtgtctgaaaaacatgagaac
agggttcaaatcccgatgggctacca
1−24tRNA−Ser(gct)[20942,21034]
ggaagattaaccctaaaaggtaagggagcagtttgctaaactgccagtaa
ccgagaaatcggcgtaccagttcaagtctggtatcttcctcca
1−25tRNA−Cys(gca)[21041,21116]
gtgtcggtgtcagaaatggttatgtgcctgtctgcaaaacaggtttatga
gggttcgagtcccttccgatactcca
>MF158044.1 Shigella phage Sf18, complete genome
1−1tRNA−Pro(tgg)[66345,66424]
ctccgtttagctcagtttggtttagcagcacttgctttgggagcaagggg
tcgagggttcaaatccttcaacggagacca
1−2tRNA−Glu(ttc)[66431,66508]
attcccgtagacaaattaggtaaagtcaacaccctttcaaggtgtggttt
gagggttcaagtcccttcgggaatgcca
1−3tRNA−Met(cat)[66596,66672]
tgcgggtatagagaaagggtgtctcacatgtctcattagcatgttatcgg
taggttcgactcctacacccgcctcca
1−4tRNA−Asn(gtt)[66757,66833]
ggttaggaagcacataaggcatgtgcggtcggctgttaaccgaatggtac
agggttcgaatccctgactaaccgcca

1-5tRNA-Asp(gtc)[66997,67073]
ggttatgtagtttaactggtcaaaatacctccctgtcacggaggatgatg
tgagttcgagtctcatcgtaaccgcca
1-6tRNA-Lys(ttt)[67524,67599]
ggaagtgtagcagaatggtgatgcggcagacttttaatctgacaggcgac
gggttcgaatccctccacttctacca
1-7tRNA-Met(cat)[67681,67757]
ggttcagtcgcagataaggtaatgcaagggtttcataagccctatgaatg
tgggttcgattcccacctgaacctcca
1-8tRNA-Ile(gat)[67760,67834]
tgtgggttagcataaatggtaatgcaaacggctgataaccgttagaagag
ggttcgataccctcacctacaacca
1-9tRNA-Arg(tct)[68082,68158]
gcacccttagttcaaacggatagagcaacggtcttctaaaccgtcagtta
caggttcgaatcctgtagggtgtgcca
1-10tRNA-Ser(tga)[68420,68509]
ggtaggtagcggctaatggaagccaaatagtcttgaaaactattgccatt
gtagagatacggtgagggttcgactcctttatctaccgcc
1-11tRNA-Leu(tag)[68763,68839]
gcacctatagcccaattggaagaggcagcagacttagaatctgctcagtg
tgagttcgaatctctctaggtgtacca
1-12tRNA-Lys(ctt)[68847,68922]
gcaggtgtagcaaaatggttatgcggctgactcttaatcagtaagacgat
gggttcaattccctccacctgtacca
1-13tRNA-Ala(tgc)[68929,69004]
ggggatgtagtttacatggttaaaacataagttttgcaaacttaagtaca
gggttcaattccctgcttctccacca
1-14tRNA-Gly(tcc)[69011,69085]
gcatccatagtttaaatgggaaaattacagtcttccaaactgaggttgag
ggttcgattccctctggatgctcca
1-15tRNA-Thr(tgt)[69092,69168]
gctgctttcgtataattggctattgcacatcccttgtaaggatggagatg
caggttcgagtcctgtgagcagcacca
1-16tRNA-Val(tac)[69264,69338]
gctcgcttagtttatatggtaaagccccatccttacaagttggtgaagaa
ggttcaagtccttcagcgagtacca
1-17tRNA-Leu(caa)[69340,69417]
gttccagtatcccaattggcagaggatgcaagctcaaaccttgtattagt
gacggttcgaatccgtcttggaacacca
1-18tRNA-Arg(acg)[69596,69672]
gcagaattagttcaaatggatagagcagcaacctacgaagttgttagtta
ggggttcgaatcctctattctgcacca
1-19tRNA-Gln(ttg)[69917,69992]
aggggattagtttacaaggttaaaacctcggtctttgaaatcgaagaaga

tggttcaattccatcatcccccgcca
1-20tRNA-Leu(taa)[69995,70073]
gctccattactccaattggcagagaggccagatttaaaatctgtgttatg
tatcggttcgaatccgatatggagtacca
1-21tRNA-Gln(ctg)[70080,70155]
agcagtatagcataactggcaatgctccagcctctgaagctgaaagatta
aggttcaaatccttatactgctgcca
1-22tRNA-His(gtg)[70187,70263]
gtggccttatcataaatggataatgacccatgctgtgaacatggtctata
cgggttcgattcccgtaggtcacccca
1-23tRNA-Phe(gaa)[70271,70346]
agctcaattagcttatatggttaaagcgcgtgtctgaaaaacatgagaac
agggttcaaatcccgatgggctacca
1-24tRNA-Ser(gct)[71099,71191]
ggaagattaaccctaaaaggtaagggagcagtttgctaaactgccagtaa
ccgagaaatcggcgtaccagttcaagtctggtatcttcctcca
1-25tRNA-Cys(gca)[71198,71273]
gtgtcggtgtcagaaatggttatgtgcctgtctgcaaaacaggtttatga
gggttcgagtcccttccgatactcca
>MF158046.1 Shigella phage Sf23, complete genome
1-1tRNA-Arg(tct)c[158397,158472]
cgaggcatagctcagaaggaagagcaaggaccttctaagtcctaggtcgt
aggttcgatccctactgcctcgacca
1-2tRNA-Asn(gtt)c[158477,158551]
ggatgtgtagctcaatggcagagcgatcgcctgttaagcgattggttata
ggttcgaatcctatcacgtccgcca
1-3tRNA-Tyr(gta)c[158556,158642]
ggggagttatcccgtagaggtagcggtgtggactgtaaatccattgtcat
tgcgactcgggtggttcgactccatcactccccacca
1-4tRNA-Met(cat)c[158655,158729]
ggccctgtagctggaaggttcaagcaagcgactcataatcgccagatggt
ggttcaattccacccagggccacca
1-5tRNA-Thr(tgt)c[158731,158806]
gctgatttagctcagtaggtagagcaactcacttgtaatgagaaggtcgg
cggttcgattccgtcaatcagcacca
1-6tRNA-Ser(tga)c[158812,158901]
ggaggcgtggcagagtggtttaatgcaccggtcttgaaaaccggcagtcg
ctccggcgactcataggttcaaatcctatcgcctccgcca
1-7tRNA-Pro(tgg)c[158903,158977]
ctccgtgtagctcagtttggtagagcgcctgatttgggatcaggaggtcc
aaggttcaaatccttgtatggagac
1-8tRNA-Gly(tcc)c[158988,159061]
gcggatatcgtataatggtattacctcagacttccaatctgatgatgtga
gttcgattctcattatccgctcca

1-9tRNA-Leu(taa)c[159067,159153]
gcgagaatggtcaaattggtaaaggcacagcacttaaaatgctgcggaat
gatttccttgtgggttcgagtcccacttctcgcacca
1-10tRNA-Gln(ttg)c[159154,159227]
tgggaattagccaagttggtaaggcactggattttgattccaggatgcaa
aggttcgagtcctttattcccagc
>MF398190.1 Staphylococcus phage vB_SauM-fRuSau02, complete genome
1-1tRNA-Met(cat)c[14750,14821]
ggactcttagcttaaaggtaaagccaaccgctcataacggtttgactgta
ggttcgaatcctgcagagtcca
1-2tRNA-Trp(cca)c[37968,38039]
acacccttagtataattagtagtacaagggtctccaaaacccttagtctt
tgtgcaaatcaaagagggtgtg
1-3tRNA-Phe(gaa)c[38046,38118]
ggtttcttagctcagatggtagagcactagattgaagctctaggtgtcat
tggttcaaatccaatagaaacca
1-4tRNA-Asp(gtc)c[38124,38199]
tggctcattggtgtaactggttaacacactgccctgtcacggcagagagt
acgagttcgagtctcgtatgggtcgt
>MF479730.1 Aeromonas phage AS-gz, complete genome
1-1tRNA-Arg(tct)c[140918,140994]
ggtctcttagctcagttggatagagcagcggccttctaagccgcgggtca
ttggttcaaatccaatagggactgcca
1-2tRNA-Met(cat)c[141563,141637]
ggccctgtagctagacggttcaagcaggcggctcataaccgctcgtagta
ggttcgattcctaccagggtcacca
1-3tRNA-Met(cat)c[141840,141916]
tgcgatgtagaggagaggtcgtcctcgtcgggctcatatcccgaaaatca
gcggttcgaatccgctcatcgcttcca
1-4tRNA-Asp(gtc)c[141929,142002]
ggagccatagtttatttggttaaaatagtcccctgtcacgggacagcacc
gagttcgatcctcggtggctccgc
1-5tRNA-Ile(gat)c[142127,142202]
agtggattagctcagtaggtagagcactcgaccgataatcgagagcgcat
tggttcgatcccaatatccactacca
1-6tRNA-Trp(cca)c[142205,142280]
atgacattggtgttagcggtagcatgccggtctccaaaaccgtgcggcca
gggttcgaatccttgatgtcatgcca
1-7tRNA-Thr(tgt)c[142645,142720]
gccggtttagctcatctggtagagcacagcctttgtaacgctggggtggt
ccgttcgagtcggacaatcggcacca
1-8tRNA-Pro(tgg)c[143356,143430]
ctcagcgtaggcaagtggtatgtcgttccgtttgggacggaattttcggg
tgttcgattcaccccgctgagacca

1-9tRNA-Gly(tcc)c[143676,143751]
gcgggtaaggtgtttatggatacatgctagccttccaagcttgagtagac
cggttcgataccggctacccgctcca
1-10tRNA-His(gtg)c[143764,143838]
gtgaccttagtagtaatggtagctatcctggatgtgacccagggagtacg
ggttcgaaccccgtaggtcacccca
1-11tRNA-Lys(ttt)c[144128,144203]
gcatctctaactcaattggtagagtagcggacctttaatccgtcaagtct
gagttcgagtctcaggggatgcacca
1-12tRNA-Tyr(gta)c[144761,144843]
ggaacgttcgggtaatggtatcccaagcggctgtaacccgctcgcctctg
gcattcttggttcgagtccaaggcgttccacca
1-13tRNA-Asn(gtt)c[144857,144939]
ggtgatgtagcataacggtcatgcggccggctgttaaccggtgaggaaac
tctacgttggttcgactccaaccatcaccgcca
1-14tRNA-Ser(tga)c[145108,145201]
ggaagattggcccgagttggtttaaggcaccggtcttgaaaaccggcgat
cgtaggaatacggtccatccgttcgaatcggatatcttcctcca
1-15tRNA-Leu(taa)c[145393,145477]
gcaagtatggtggaattggtatacactggaggcttaaaacctcccgcctt
cgggattgtgggttcgagtcccactacttgcacca
>MF668280.1 Mycobacterium phage Phabba, complete genome
1-1tRNA-Ser(gct)[31867,31949]
ggagggtgagtatcaggtgatacagcgagattgctaatcccgtacggctc
atacccgtgaggttcaagtcctcctccctccgc
1-2tRNA-Leu(cag)[32069,32152]
gcctgggtggcggaatggcagacgcgccagattcagaatctggtgtcctt
cggggcgtgagggttcaagtccctctccaggtac
1-3tRNA-Leu(gag)[32157,32230]
gctcgggtagcccaaaggcagaggcagcacgttgaggacgtgtccagtgc
gggttcgactcccgctccgagtac
1-4tRNA-Leu(caa)[32273,32357]
gggtgaatggcgaaattggtagacgcgctggcctcaaaagccggtgcccg
caagggcgtgtgggttcgagtcccacttcatctac
1-5tRNA-Pyl(cta)[32921,32991]
gcggtatcgcgtacggcacgcaggtgacttctaaactcaccgtttccggg
ttcgagtcccggataccgcgc
1-6tRNA-Pro(tgg)[90881,90953]
cgggatatggtgtaagggttagcacgattggtttgggaccattctgacgg
ggttcgagtccctgtttcccgac
1-7tRNA-Trp(cca)[90992,91064]
ggggacttcgccaagtgggacggcagcggcttccaaacccgccatgaggg
agttcgattcttccagtcctcgc
1-8tRNA-Tyr(gta)[91068,91152]

gccgtgtatgcccaactggtgtttgggagctggctgtaacccagtggcct
tcgggacggcggagttcgattctctgacgcggcac
1-9tRNA-Met(cat)[91296,91370]
agcggggtagagcagttcggcagctcgctagcctcataagctagaggtcg
gaggttcaaatcctcctcccgccac
1-10tRNA-Cys(gca)[91569,91641]
gcgtctatggcgaaatggtaacgcgctggattgcaaccccagtattcctc
cgttcgattcggagtaggcgctc
1-11tRNA-Glu(ctc)[91700,91771]
ggtccaatggggtagtggtaaccctcctgattctcagtcaggcatcccga
gttcgatcctcggttggactgc
1-12tRNA-His(gtg)[91773,91845]
gtggccatagttcaatggcagaacgccgggttgtgatcccggtcgcgagg
gttcgattccctctggtcacccc
1-13tRNA-Gly(tcc)[91846,91917]
gcgtctatggtgtagcggtaacacatcagtcttccaaactgagttcgccg
gttcgatcccggctagacgctc
1-14tRNA-Ala(ggc)[92072,92145]
ggggctgtagctcaatcggtagagcgtctcgctggcagtgagaaggcagt
cggttcgatcccgaccagttccac
1-15tRNA-Phe(gaa)[92207,92279]
gcccatttagctcatgtggatgagcgtcggattgaaatcccgagggagct
ggttcgactccagcaatgggcac
1-16tRNA-Val(cac)[92353,92424]
gcccgattagttcaatgggagaacaaccggttcacatccggtagtcagag
gttcgattcctctattgggtac
1-17tRNA-Lys(ctt)[92436,92507]
ggccttgtagctgaggggattagcacctgactcttaatcaggggaccttg
gttcgattccaagtagggccac
1-18tRNA-Gly(gcc)[92582,92653]
gtgctattagtgaaatggcatcacacgactttgccaaggtcgtagcgcgg
gttcgattcccgcatagcactc
1-19tRNA-Asp(gtc)[92725,92801]
ggccctgtagcttagtttggccaaaagcgcctgcctgtcgagcaggagaa
cacgggttcaaatcccgttagggtcgc
1-20tRNA-Met(cat)[93169,93242]
gcctgtgtagctcatctggcagagcgccctgctcataacggggaggtaag
tggttcgagaccactcataggtac
1-21tRNA-Ile(gat)[93269,93343]
gcctgttagcggactggtcgtccgatccaagctgataacttggcgcaagc
agtgttcgattcactgagcaggtac
1-22tRNA-Ser(tga)[93390,93462]
tgcggggtagctcagttggtagagcgccacccttgatcggtggaggtcgt
cggttcgagtccgatccctttcc

1-23tRNA-Arg(acg)[93491,93564]
ctcccggtagctcagcggacagagtgcttgactacgaatcaagaagccgg
aggttcgattcctcctcgggagac
1-24tRNA-Thr(ggt)[93687,93758]
gctggattagcatagtggtagtgcccatccttggtatggatgttgccggg
gttcgattccccgattcagctc
1-25tRNA-Val(gac)[93764,93836]
gctcttgtagctcagtggtagagcacctgcccgacatgcaggttgtccga
ggttcgattcctcgttagagcac
1-26tRNA-Glu(ttc)[93980,94054]
ggtccgttcggctagtggcccaagccccctgcctttcaagcaggatgaca
ccggttcaaatccggtacggactac
1-27tRNA-Thr(tgt)[94171,94242]
gcctccatcgttcagtggtaggacgccgaccttgtaagtcggctgcgggc
gttcgattcgtcctggaggctc
1-28tRNA-Arg(cct)[94490,94563]
accgctgtagctcagtggacagagcagggacctcctacgtcccgtgccgg
gggttcgattcctcccagcggtgc
1-29tRNA-Gln(ttg)[94594,94667]
tgcggagcggtctgttggcaggccagtgggcctttgacccccacgtccgt
aggttcgattcctgcctccgcagc
1-31tRNA-Lys(ttt)[95471,95546]
ggggcggtagtctagcccggtcaagactctcgacttttaatcgagatgcc
gtgggttcaaatcccacccgccccac
1-32tRNA-Gln(ctg)[122516,122587]
tgcctgttggtgtaatggcaacacgcaagtttctgacacttgtattcttg
gttcgagtccagggcgggcagc
1-33tRNA-Asn(gtt)[122607,122679]
tggtctgtagttcaatggcagaacgctcgcctgttaagcgagtagttgtt
ggttcgagtccagccaggccagc
>DQ004855.1 Listeria bacteriophage P100, complete genome
1-1tRNA-Met(cat)c[123712,123785]
ttgtcccgtagctagaaggtcgagcaaggagctcataactcctcggtttg
ggttcgattcccaacggggcaatc
1-2tRNA-Pro(tgg)c[124678,124752]
cagggtgtagctcagtttggttagagtacccgctttggagacgggaagtc
gtaggttcgaatcctaccaccttga
1-3tRNA-Arg(tct)c[125870,125940]
gtccttatggtgtagcggatgcacaagggatttctactcccttagcgcgg
gttcgaatcctgctgaggact
1-4tRNA-Gly(tcc)c[126187,126257]
gcgggtatagtataagggtagtacccaaggtttccaaccatgtagtgggg
gttcgaatccccctacccgct
1-5tRNA-Asn(gtt)c[126326,126399]

gtgtccttaactcagaggtcagagtgccgtcctgttaagtcggaagtcgc
tggttcaaatccagcaggatacgc
1–6tRNA–Ser(tga)c[127020,127111]
ggaaggttggtagagcttggtaatacgctagtcttgaaaactagtttgcc
cttggaatacagggtacaagggttcaaatcccttaccttcct
1–7tRNA–Phe(gaa)c[127124,127195]
gtagtcctagctgagatggattagcgcttgcttgaaaagcaggagaggca
ggttcgatacctgcggactcca
1–8tRNA–Lys(ttt)c[127201,127272]
ggagttatggtgaaatggctatcactgcgggtttttaccccgttattcta
ggttcgaatcctagtggctcca
1–9tRNA–Trp(cca)c[127397,127470]
taggggtatagtttatctggtaaaatattggtttccaactccaatgaggt
gggttcaagtcctactatccctgt
1–10tRNA–Gln(ttg)c[127472,127544]
tggtctgtagccaagcggtaaggcaacggattttgattccgtgatacgtt
ggttcgaatccaactagaccagc
1–11tRNA–Thr(tgt)c[127563,127634]
gcttgtatagttcaattggtagaacagtggttttgtaagcctcagacgtg
ggttcaagtcctactacaagca
1–12tRNA–Tyr(gta)c[127717,127798]
gtgccattcgcatagaggcaattgcaggggactgtaactcccctcccttc
ggggttccaaggttcgagtccttgatggcgca
1–13tRNA–Leu(tag)c[128159,128243]
tgccgagatggtggaactggtatacacggtagacttagaatctgctgtcc
caaggatatgtgggttcgaatcccactctcggtat
1–14tRNA–Asp(gtc)c[128709,128781]
gtgcgtatgatataatggctattatactcgactgtctatcgagaaatagg
ggttcgattccccttacgtgcgc
1–15tRNA–Ile(gat)c[128885,128957]
accagcatagcttaggaggcaaagcaaccgaccgataatcggtagtcctt
ggttcaattccaagtgttggtac
1–16tRNA–Ser(gct)c[129134,129220]
ggagagttgtcagagaggcttaatgatacgggttgctaactcgttgtact
agtaatagtaccaagggttcgaatcccttactctcct
1–17tRNA–Cys(gca)c[129302,129372]
gcgggtataaccaactggaaaggtagtagactgcaaatctacgtatatgg
gttcaattcccattacccgct
>KX369583.1 Mycobacterium phage Littleton, complete genome
1–1tRNA–Ser(gct)[30420,30503]
ggagggtgagcatcaggtgatgcagcgagattgctaatcccgtacggtaa
ccaccccgtgaggttcgaatcctcctccctccgc
1–2tRNA–Leu(cag)[30696,30771]
gctcccgtagcccaattggcaggaggcaccagattcaggatctgggcagt

gtgagttcgaatctcaccgggagtac

1–3tRNA–Leu(gag)[30891,30965]

gtctctgtaggcaaatcggaaaagccgccatcttgagggggtggtgcgtg

cgggttcgactcccgccagagacac

1–4tRNA–Leu(caa)[30966,31039]

gccgtggtaggccatctggcgagccgccgagttcaagtttcggtgtttgc

gggttcgaatcccgcccacggtac

1–5tRNA–SeC(tca)[67826,67922]

attctggcactggtgggcgagcccaccggcggcagcttcaagctgtcgct

ggccggagaaccgaccggaaacatcccgttcaacgcgacccagggcc

1–6tRNA–Pro(tgg)[91780,91852]

cggggtgtagttcagtggaagagcgcttggtttgggaccaagatgtcgca

ggttcgaatcctgtcaccccgac

1–7tRNA–Trp(cca)[91869,91944]

tggggtgaagccgatctggaaggcagcggtctccaaagccgtctcatagc

gggttcgaatcccgtcacccctgcca

1–8tRNA–Tyr(gta)[91946,92032]

gccgcacatgcccaactggtgtttgggagcaggctgtaaccctgtggcct

tcgggacggtgaggttcgattcctcagtgcggtacca

1–9tRNA–Pyl(cta)[93141,93213]

gcaccatttgctcaatggcagagcggcggcttctaaccccgtgagtgccg

gttcgattccggcatggtgcacc

1–10tRNA–Met(cat)[93363,93437]

agcggtgtagagcagctaggtagctcgccgggctcataacccggaggacg

cgtgttcgaatcacgccaccgccac

1–11tRNA–Cys(gca)[93564,93635]

gcgcctttggcggaatggctacgtgctcggctgcaacccgagttatcccg

gttcgactccgggagggcgctc

1–12tRNA–Glu(ctc)[93703,93774]

gctcccatggggtagtggtaaccctcctggttctcagccaggcgtcccga

gttcgatcctcggtgggagtgc

1–13tRNA–His(gtg)[93776,93849]

gtggccgtagttcagccggtagaacgctgggttgtgatcccagtcgtcga

gggttcgagtccctccggtcaccc

1–14tRNA–Ala(tgc)[94011,94085]

gggcctgtagctccaattggtagagcagcatccttgcaagatgacggctg

tcggttcgaatccgacctggtccac

1–15tRNA–Phe(gaa)[94275,94347]

gccgtcatagctcagttggtagagcactggcctgaaaacccagtggccga

ggttcgagtcctcgtgtcggcac

1–16tRNA–Val(cac)[94353,94426]

gtccgtttagctcagctggaagagcgctcggtccacacccgagaggccgc

aggttcgatccctgcaatggacac

1–17tRNA–Lys(ctt)[94545,94617]

gccttcgtagctcagtggtagagctgtcgcctcttaagcgataggtcgtt
ggttcgaatccagccgggggcac
1-18tRNA-Glu(ttc)[94622,94698]
ggtcgggtcggtctgctggtatggccagtcggattttcactccggacatt
cgcgggttcaattcccgtcccgatcgc
1-19tRNA-Gly(tcc)[94778,94850]
gcgggtgtggccgaatggctcaggcaccagatttccactctggctacgca
ggttcgattcctgtcatccgctc
1-20tRNA-Thr(cgt)[94910,94984]
gctgctgtagctcacctggcagagcgtcggcgtcgtatcccgaaggcatc
cggttcgagtccggacagcagcccc
1-21tRNA-Thr(tgt)[94985,95057]
gcctctgtggtccagcggcacggacatccgccttgtaagcggaggacccc
cgttcgatccggggtagaggctc
1-22tRNA-Thr(ggt)[95465,95537]
gctgggttagctcagtggtagagcgttcctctggtatgggaaaggcccgg
ggttcaatcccccgattcagctc
1-23tRNA-Gly(gcc)[96860,96933]
gcgaaggtagctcagctggcagagcgccaccttgccaaggtggaggtcgc
gggatcgtaacccgttcttcgctc
1-24tRNA-Asp(gtc)[96937,97009]
ggccctgtagctcagaggaagagcgccggtctgtcgaatcggaggtcgcg
gtatcgtaatccgtcagggtcgc
1-25tRNA-Met(cat)[97069,97141]
gcctcactagctcattggtagagccgctcgctcataacgtgcaggtacct
ggttcgattccagggtgaggtac
1-26tRNA-Ile(gat)[97147,97221]
gcctgttagcggactggtcgtccgatccaagctgataacttggcgtaagc
ggtgttcgattcaccgagcaggtac
1-27tRNA-Arg(acg)[97315,97387]
gcctctatggtccaacggatatgacgccggtctacggaaccggagatgcg
tgttcgattcgcgctaggggcac
1-28tRNA-Val(gac)[97430,97502]
gtccgtgtagctcaggggtagagcgcctgctcgacacgcaggaggaccga
ggttcgaaacctcgcatggacac
1-29tRNA-Arg(cct)[97676,97749]
gcctctgtagctcaacggacagagcaacgcggtcctaacgcggtggctgg
aggttcgaatcctctcggaggcac
1-31tRNA-Gln(ttg)[98290,98365]
tggggtatggtggcaatctggcagtcccgccggactttgactccggaggt
gcaggttcgagtcctgctaccccatc
1-32tRNA-Arg(tct)[98369,98442]
gcccttgtagctcagtggacagagcggcgagcttctacctcgcgggccgg
gagttcgaatctctccaggggcac

1-33tRNA-Lys(ttt)[98510,98586]
gggccggtatcttagtctggtcaaagaagtggacttttaatccgcgcgcc
gtgggttcgaatcccacccggcccacc
1-34tRNA-Gln(ctg)[126425,126499]
tgctcgttggtgtaactggcaacactacggactctgactccgtcatttta
ggttcgaatcctaagcgagcagcca
1-35tRNA-Asn(gtt)[126506,126581]
tggggtgtccgttaatcaggcaaacgagcggactgttaatccgcccctgc
aggttcgaatcctgccaccccagcca
>AP011616.1 Thermus phage phiYS40 DNA, complete genome
1-1tRNA-Thr(tgt)[52068,52143]
ggcctcgtagctcaacaggaagagcactcgccttgtaagcggggggttgt
gggttcaaatcccaccggggcctcca
1-2tRNA-Met(cat)c[120023,120099]
ggggcgttagctcaagcggtgagagcggtcggctcataaccgattggttg
taggttcgagtcctacacgccccacca
1-3tRNA-Arg(tct)c[120112,120188]
gaggggttagctcaacaggacagagcaagggatttctaatccctaggttg
caggttcaagtcctgcacccctcgcca
>AB609718.1 Enterococcus phage phiEF24C-P2 DNA, complete genome
1-1tRNA-Met(cat)c[136981,137054]
ggacgtttagctcagttggtcagagcattcggctcataaccgaacggtcg
caggttcgagacctgcaatgtcca
1-2tRNA-Leu(tag)c[137677,137761]
gcagaagtgatggaactggtagacaacggtgtcttagaaacatcggtctg
taatggacgtgtgggttcgactcccgccttctgca
1-4tRNA-Arg(tct)c[138482,138557]
tgtaggtgtagctcaataggatggagcatccgccttctaagcggacggtt
gggggttcgattccctccatctacgt
1-5tRNA-Trp(cca)c[139156,139229]
tagtcggttagtgtaactggtaacacgttggtctccaaaaccaataatag
gggttcaaatcctctaccgattgt
1-6tRNA-Asp(gtc)c[139479,139554]
tggcagtatagggcagaggttgtcccaacacgttgtcagcgtggaacaca
cgggttcgagtcccgttactgtcgtc
>KM607001.1 Enterobacteria phage RB33, complete genome
1-1tRNA-Arg(tct)c[67601,67677]
cggggcatagctcaattgtatagagcaacggacttctaatccgtaggttg
aaggttagaatccttctgtctcgacca
1-2tRNA-Met(cat)c[68388,68459]
ggccctgtagctggaaggttcaagcaagcgactcataatcgccagatggt
ggttcaattccacccagggcca
1-3tRNA-Thr(tgt)c[68461,68536]
gctgatttagctcagtaggtagagcaactcacttgtaatgagaaggtcgg

cggttcgattccgtcaatcagcacca
1−4tRNA−Ser(tga)c[68538,68626]
tggaggcgtggcagagtggtttaatgcaccggtcttgaaaaccggcagtc
gctccggcgactcataggttcaaatcctatcgcctccgt
1−5tRNA−Pro(tgg)c[68627,68701]
ctccgtgtagctcagtttggtagagcgcctgatttgggatcaggaggtcc
aaggttcaaatccttgtatggagac
1−6tRNA−Gly(tcc)c[68712,68785]
gcggatatcgtataatggcattacctcagacttccaatctgatgatgtga
gttcgattctcattatccgctcca
1−7tRNA−Leu(taa)c[68791,68877]
gcgagaatggtcaaattggtaaaggcacagcacttaaaatgctgcggaat
gatttccttgtgggttcgagtcccacttctcgcacca
1−8tRNA−Gln(ttg)c[68878,68951]
tgggaattagccaagttggtaaggcatagcactttgactgctagatgcaa
aggttcgagtcctttattcccagc
>GQ303262.1 Mycobacterium phage LRRHood, complete genome
1−1tRNA−Ser(gct)[30760,30843]
ggagggtgagcatcaggtgatgcagcgagattgctaatcccgtacggtaa
ccaccccgtgaggttcgaatcctcctccctccgc
1−2tRNA−Leu(cag)[31036,31111]
gctcccgtagcccaattggcaggaggcaccagattcaggatctgggcagt
gtgagttcgaatctcaccgggagtac
1−3tRNA−Leu(gag)[31231,31305]
gtctctgtaggcaaatcggaaaagccgccatcttgagggggtggtgcgtg
cgggttcgactcccgccagagacac
1−4tRNA−Leu(caa)[31306,31379]
gccgtggtaggccatctggcgagccgccgagttcaagtttcggtgtttgc
gggttcgaatcccgcccacggtac
1−5tRNA−SeC(tca)[67722,67818]
attctggcactggtgggcgagcccaccggcggcagcttcaagctgtcgct
ggccggagaatcgaccggaaacatcccgttcaacgcgacccagggcc
1−6tRNA−Pro(tgg)[91484,91558]
cggggtgtagttcagtttggaagagcgcttggtttgggaccaagatgtcg
caggttcgaatcctgtcaccccgac
1−7tRNA−Trp(cca)[91573,91643]
gggtctgtgcacagggtgcccgacggtctccaaagccgaaggcgggggtt
cgattccctccaggcctgcca
1−8tRNA−Tyr(gta)[91645,91731]
cccgtacatgcccaactggtgtttgggagcaggctgtaaccctgtggcct
tcgggacggtgaggttcgattcctcagtgcgggacca
1−9tRNA−Pyl(cta)[92845,92917]
gcaccatttgctcaatggcagagcggcggcttctaaaaccgtgagtgccg
gttcgactccggcatggtgcacc

1–10tRNA–Met(cat)[93067,93141]
agcggtgtagagcagctaggtagctcgccgggctcataacccggaggacg
cgtgttcgaatcacgccaccgccac
1–11tRNA–Cys(gca)[93268,93339]
gcgcctttggcggaatggctacgtgctcggctgcaacccgagttatcccg
gttcgactccgggagggcgctc
1–12tRNA–Glu(ctc)[93344,93418]
ggtccgttggagtagatggatatctcgccaccctctcaaggtggagatca
cgggttcaagtcccgtacggactgc
1–13tRNA–His(gtg)[93420,93493]
gtggccgtagttcagccggtagaacgctgggttgtgatcccagtcgtcga
gggttcgagtccctccggtcaccc
1–14tRNA–Ala(tgc)[93655,93729]
gggcctgtagctccaattggtagagcagcatccttgcaagatgacggctg
tcggttcgaatccgacctggtccac
1–15tRNA–Phe(gaa)[93919,93991]
gccgtcatagctcagttggtagagcactggcctgaaaacccagtggccga
ggttcgattcctcgtgtcggcac
1–16tRNA–Val(cac)[93997,94070]
gtccgtttagctcagctggaagagcgctcggtccacacccgagaggccgc
aggttcgatccctgcaatggacac
1–17tRNA–Lys(ctt)[94190,94263]
gccttcgtagctcagttggtagagctctcgcctcttaagcgagatgtcgc
aggttcgacccctgccggaggcac
1–18tRNA–Glu(ttc)[94268,94344]
ggtcgggtcggtctgctggtatggccagtcggattttcactccggacatt
cgcgggttcaattcccgtcccgatcgc
1–19tRNA–Gly(tcc)[94424,94496]
gcgggtgtggccgaatggctcaggcaccagatttccactctggctacgca
ggttcgattcctgtcatccgctc
1–20tRNA–Thr(cgt)[94556,94630]
gctgctgtagctcacctggcagagcgtcggcgtcgtatcccgaaggcatc
cggttcgagtccggacagcagcccc
1–21tRNA–Thr(tgt)[94631,94703]
gcctctgtggtccagcggcacggacatccgccttgtaagcggaggacccc
cgttcgatccggggtagaggctc
1–22tRNA–Thr(ggt)[94763,94835]
gctgggttagctcagtggtagagcgttcctctggtatgggaaaggcccgg
ggttcaatcccccgactcagctc
1–23tRNA–Gly(gcc)[96158,96231]
gcgaaggtagctcagctggcagagcgccaccttgccaaggtggaggtcgc
gggatcgtaacccgttcttcgctc
1–24tRNA–Asp(gtc)[96235,96307]
ggccctgtagctcagaggaagagcgccggtctgtcgaatcggaggtcgcg

gtatcgtaatccgtcagggtcgc
1-25tRNA-Met(cat)[96367,96439]
gcctcactagctcattggtagagccgctcgctcataacgtgcaggtacct
ggttcgattccagggtgaggtac
1-26tRNA-Ile(gat)[96445,96519]
gcctgttagcggactggtcgtccgatccaagctgataacttggcgtaagc
ggtgttcgattcaccgagcaggtac
1-27tRNA-Arg(acg)[96613,96685]
gcctctatggtccaacggatatgacgccggtctacggaaccggagatgcg
tgttcgattcgcgctaggggcac
1-28tRNA-Val(gac)[96728,96800]
gtccgtgtagctcagtggtagagcgcctgctcgacacgcaggaggaccga
ggttcgaaacctcgcatggacac
1-29tRNA-Arg(cct)[96974,97047]
gcctctgtagctcaacggacagagcaacgcggtcctaacgcggtggctgg
aggttcgaatcctctcggaggcac
1-31tRNA-Gln(ttg)[97588,97663]
tggggtatggtggcaatctggcagtcccgccggactttgactccggaggt
gcaggttcgagtcctgctaccccatc
1-32tRNA-Arg(tct)[97667,97742]
gcccttgtagctcagtggacagagcggcgagcttctacctcgcgggccgg
gagttcgaatctctccaggggcacca
1-33tRNA-Gln(ctg)[124635,124709]
tgctcgttggtgtaactggcaacactacggactctgactccgtcatttta
ggttcgaatcctaagcgagcagcca
1-34tRNA-Asn(gtt)[124716,124791]
tggggtgtccgttaatcaggcaaacgagcggactgttaatccgcccctgc
aggttcgaatcctgccaccccagcca
>KT365400.1 Mycobacterium phage ErnieJ, complete genome
1-1tRNA-Ser(gct)[29948,30031]
ggagggtgagcatctggtgatgcaggggtcctgctaaggccctacggatt
cacacccgtgagtttcgattactcctccctccgc
1-2tRNA-Leu(cag)[30128,30204]
gccctgctgagcaaactggcaaagctgccgcattcagagtgcgggtcatt
tccgggttcgactcccgggcagggtac
1-3tRNA-Leu(gag)[30324,30398]
gtctctgtaggcaaatcggaaaagccgcccatcttgagggggtggtgcgtg
cgggttcgactcccgccagagacac
1-4tRNA-Leu(caa)[30399,30472]
gccgtggtaggccatctggcgagccgccgagttcaagtttcggtgtttgc
gggttcgaatcccgcccacggtac
1-5tRNA-Pro(tgg)[90472,90546]
cggggtgtagttcagtttggaagagcgcttggtttgggaccaagttgtcg
caggttcgaatcctgtcaccccgac

1-6tRNA-Trp(cca)[90561,90631]
gggtctgtgcacagggtgcccgacggtctccaaagccgaaggcgggggtt
cgattccctccaggcctgcca
1-7tRNA-Pyl(cta)[91768,91841]
tgcgagatcgtgcacggcgcactaggagcttctaaccctccgactcgcgg
gttcgactcccgcatctcgcaccc
1-8tRNA-Met(cat)[91989,92063]
agcggtgtagagcagctaggtagctcgccgggctcatgacccggaggacg
cgtgttcgattcacgccaccgccac
1-9tRNA-Cys(gca)[92190,92261]
gcgcctttggcggaatggctacgtgctcggctgcaacccgagttatcccg
gttcgactccgggagggcgctc
1-10tRNA-Glu(ctc)[92266,92340]
ggtccgttggagtagatggacatctcgccaccctctcaaggtggagatca
cgggttcaagtcccgtacggactgc
1-11tRNA-His(gtg)[92342,92415]
gtggccgtagttcagccggtagaacgctgggttgtgatcccagtcgtcga
gggttcgagtccctccggtcaccc
1-12tRNA-Ala(tgc)[92577,92651]
gggcctgtagctccaattggtagagcagcatccttgcaagatgacggctg
tcggttcgaatccgacctggtccac
1-13tRNA-Phe(gaa)[92841,92913]
gccgtcatagctcagttggtagagcactggcctgaaaacccagtggccga
ggttcgattcctcgtgtcggcac
1-14tRNA-Val(cac)[92919,92992]
gtccgtttagctcagctggaagagcgctcggtccacacccgagaggccgc
aggttcgatccctgcaatggacac
1-15tRNA-Lys(ctt)[93111,93183]
gccttcgtagctcagtggtagagctgtcgcctcttaagcgataggtcgtt
ggttcgaatccagccgggggcac
1-16tRNA-Glu(ttc)[93188,93264]
ggtcgggtcggtctgctggtatggccagtcggattttcactccggacatt
cgcgggttcaattcccgtcccgatcgc
1-17tRNA-Gly(tcc)[93344,93416]
gcgggtgtggccgaatggctcaggcaccagatttccactctggctacgca
ggttcgattcctgtcatccgctc
1-18tRNA-Thr(cgt)[93476,93550]
gctgctgtagctcacctggcagagcgtcggcgtcgtatcccgaaggcatc
cggttcgagtccggacagcagcccc
1-19tRNA-Thr(tgt)[93551,93623]
gcctctgtggtccagcggcacggacatccgccttgtaagcggaggacccc
cgttcgatccggggtagaggctc
1-20tRNA-Thr(ggt)[94031,94103]
gctgggttagctcagtggtagagcgttcctctggtatgggaaaggcccgg

ggttcaatcccccgactcagctc
1−21tRNA−Gly(gcc)[95426,95499]
gcgaaggtagctcagctggcagagcgccaccttgccaaggtggaggtcgc
gggatcgtaacccgttcttcgctc
1−22tRNA−Asp(gtc)[95503,95575]
ggccctgtagctcagaggaagagcgccggtctgtcgaatcggaggtcgcg
gtatcgtaatccgtcagggtcgc
1−23tRNA−Met(cat)[95635,95707]
gcctcactagctcattggtagagccgctcgctcataacgtgcaggtacct
ggttcgattccagggtgaggtac
1−24tRNA−Ile(gat)[95713,95787]
gcctgttagcggactggtcgtccgatccaagctgataacttggcgtaagc
ggtgttcgattcaccgagcaggtac
1−25tRNA−Arg(acg)[95881,95953]
gcctctatggtccaacggatatgacgccggtctacggaaccggagatgcg
tgttcgattcgcgctaggggcac
1−26tRNA−Val(gac)[95996,96068]
gtccgtgtagctcaggggtagagcgcctgctcgacacgcaggaggaccga
ggttcgaaacctcgcatggacac
1−27tRNA−Arg(cct)[96242,96315]
gcctctgtagctcaacggacagagcaacgcggtcctaacgcggtggctgg
aggttcgaatcctctcggaggcac
1−29tRNA−Gln(ttg)[96856,96931]
tggggtatggtggcaatctggcagtcccgccggactttgactccggaggt
gcaggttcgagtcctgctaccccatc
1−30tRNA−Arg(tct)[96935,97010]
gcccttgtagctcagtggacagagcggcgagcttctacctcgcgggccgg
gagttcgaatctctccaggggcacca
1−31tRNA−Gln(ctg)[123129,123203]
tgctcgttggtgtaactggcaacactacggactctgactccgtcatttta
ggttcgaatcctaagcgagcagcca
1−32tRNA−Asn(gtt)[123210,123285]
tggggtgtccgttaatcaggcaaacgagcggactgttaatccgcccctgc
aggttcgaatcctgccaccccagcca
>JN849462.1 Vibriophage phi−pp2, complete genome
1−1tRNA−Ser(gct)c[174369,174457]
ggagatatggtctaaaggtatgacagcaccctgctaaggtgtcggacgtt
aatagcgttctctgggttcgattcccagtatctccgcca
1−2tRNA−Leu(caa)c[174899,174975]
gcccaactagtccaattggcagaggcgctagtttcaaacactagatgatc
cgagttcgaatctcgggttgggcacca
1−3tRNA−Leu(taa)c[175091,175167]
gcgcacgtggtccaattggcagaggcatgaggcttaaaatctcagggatg
gcggttcgaatccgcccgtgcgtacca

1-4tRNA-Cys(gca)c[175170,175243]
gcccgaatcgtataatggaagtatgagggattgcaaatcccgcggtcaga
gttcgattctctgttcgggctcca
1-5tRNA-His(gtg)c[175347,175423]
gtggcagtgggtgaagtggaaataccccccgggttgtgattccggaagatg
cgggttcgatccccgtctgtcacccca
1-6tRNA-Val(tac)c[175741,175814]
ggtcccttagtataatggcagtacgtctcttttacacagagaaagaagtg
gctcgattccactagggactacca
1-7tRNA-Gly(tcc)c[175820,175893]
gcgggtatgatgtaatggtagcatgacgtccttccaagtcgttcgtctcg
gttcgagtccgtgtacccgctcca
1-8tRNA-Met(cat)c[176081,176156]
ggagatgtagctcaagtggtagagcaatggtctcataagccattagatcc
gatttcgagtatcggtgtctccacca
1-9tRNA-Arg(tct)c[176661,176736]
gcgctgttagctcaattggaagagcacgtcccttctaaggatggggttat
gagttcgaatctcatacggcgtgcca
1-10tRNA-Thr(tgt)c[177133,177208]
gccctgatagcacaattggcagtgcagctcacttgtaatgagcaggttcg
cggttcaaatccgtgtcagggcacca
1-11tRNA-Gln(ttg)c[177337,177410]
aggggattgatgtaaaggcagcatacgtgactttgactcacgcagtaccg
gttcgaatccgttatcccctgcca
1-12tRNA-Asp(gtc)c[177430,177505]
ggagccgaggtgtaagtggttgcatgtctccctgtcacggagaaggtagc
gggttcgaaacccgtcggttccgcca
1-13tRNA-Asn(gtt)c[177728,177803]
gccttattaactcagtcggtagagtgtctggctgttaaccggagagtcgt
tggttcgagtccaacataaggcgcca
1-14tRNA-Asn(gtt)c[177810,177884]
gggtgtgtagctcagaggcagagcagacggctgttaaccgtcaggtcgag
atttcgaaattctccacgcccgcca
1-15tRNA-Trp(cca)c[178168,178241]
agggacatgatgtaacggcagcatgacggattccaaacccgttcgttaga
gttcgaatctctatgtccctgcca
1-16tRNA-Ile(gat)c[178248,178321]
agctccatagtttaacggtaaaacacgcgaccgataatcgcacgttgaca
gttcgagtctgtctggggctacca
1-17tRNA-Ser(tga)c[178386,178471]
gaaagattctgggtagcggcaacggtcttgaaaaccgtcggtcaccggga
ggtgatgttagggttcgaatccctagtcttccgcca
1-18tRNA-Met(cat)c[178497,178568]
tgcggagtagagcagtggttagctcatcggtctcataagccgaaggtcgt

tggttcgaatccaacctcacgc
1-19tRNA-Tyr(gta)c[179134,179219]
ggagcgtaagccgcaaggtgcggcagcggactgtaaatccgtgtcccgta
atgggaagagaggttcgattcctctacgctccacca
1-20tRNA-Glu(ttc)c[179226,179299]
gctcgattcgactatcggtaggtcacttccctttcaaggaagtaggacgg
gttcgactcccgtatcgagtacca
1-21tRNA-Lys(ttt)c[179363,179439]
gcgtcggtagctcatcatggaagagcagggagcttttaactctcaggtgt
ctggttcgagtccagggcggcgtacca
1-22tRNA-Lys(ttt)c[179451,179525]
gggtcgttcgtataacggtagtacatctggcttttaaccagaacggtgag
agttcgaatctcttgcgacccacca
1-23tRNA-Phe(gaa)c[179532,179606]
gcacccttagcttatcaggaaaagcggcggattgaagtcccgcagtgctc
ggttcgattccgagggggtgcacca
1-24tRNA-Leu(tag)c[179608,179685]
gcgcaagtagcccaatctggcagaggcactggtcttagaaaccagaagtt
aagagttagaatctcttcttgcgtacca
1-25tRNA-Arg(acg)c[180128,180203]
gcccgattagctcaattggaagagcagcgccctacgaaggcgaaggttac
aagttcgaatcttgtattgggtgcca
1-26tRNA-Pro(tgg)c[180212,180288]
ccgtgactagctcaatctggtagagtactccgtttggggcggagaagtta
agcgttcgaatcgcttgtcacggacca
1-27tRNA-Pro(tgg)c[180298,180371]
cgggacgtggcgtaaaggcagcgttcatgctttgggagcatgtggtaaga
gttcgagtctcttcgtcccgacca
1-28tRNA-Met(cat)c[182211,182288]
ggccctatagctcaactaggttagagcaaccgactcataatcggtgggtt
acaggttcgagccctgttggggtcacca
>KU686203.1 Synechococcus phage S-CAM8 isolate 0810PA29, complete genome
1-1tRNA-Val(tac)[13494,13565]
gcccgaatagctcagcggtagagcagcacctttacacggtgaatgtcggg
ggttcgatcccctctttgggca
1-2tRNA-Leu(taa)[17517,17603]
tgggagtgtggcggaatcggtagacgcaccagacttaaaatctgttgacc
aataaggtcgtgggagttcaagtctccctactcctat
1-3tRNA-Thr(tgt)[17657,17729]
gcctccgtagctcagtggtagagcagggcttttgtaaagctcaggtcgca
agttcaaatcttgtcagaggctc
1-4tRNA-Asn(gtt)[17734,17805]
tcctccttagctcagcggtagagcggttgactgttaatcaatttgtccct
ggttcgatcccaggagggggag

1-5tRNA-Arg(tct)[154712,154787]
tgggtcagtagctcagatggatagagcaactgccttctaagcagtcggcc
acaggttcgagtcctgtctgacccgt
>JF461087.1 Salmonella phage FO1a, complete genome
1-1tRNA-Pro(tgg)[23621,23697]
ctctgtttagctcagcttggtagagcgttccgtttggggcggtaaggccg
gaggttcaagtcctccaacagagacca
1-2tRNA-Glu(ttc)[23705,23782]
gttccagtagacaaaatggtaaagtcaccactctttcaaagtggatatct
gagggttcaaatcccttctggaacgcca
1-3tRNA-Met(cat)[23874,23950]
tgcgggtatagagaaagggcgtctcacatgtctcattagcatggtatcgg
caggttcgactcctgcacccgcctcca
1-4tRNA-Asn(gtt)[24034,24110]
ggttaggaagcacataaggtatgtgcggtcgcctgttaagcgaatggcac
agggttcgaatccctgactaaccgcca
1-5tRNA-Tyr(gta)[24180,24267]
gtgtcgttatcccgtagatggtagcggtggggactgtaaatcccttgtca
ttgagactcggtaggttcgactcctacacggcacacca
1-6tRNA-Asp(gtc)[24273,24349]
ggctatgtagtttaactggagaaaatactcccctgtcacgggagatgatg
tgagttcaagtctcatcgtagccgcca
1-7tRNA-Lys(ttt)[24778,24853]
ggaagtgtagcagaatggtgatgcggcagacttttaatctgacaggcgat
gggttcgaatccctccacttctacca
1-8tRNA-Met(cat)[24858,24934]
ggttcagtcgcagataaggtaatgcaagggtctcataagccctatgaatg
tgggttcgattcccatctgaacctcca
1-9tRNA-Ile(gat)[25431,25506]
gctgtgaaagcacatatggatgtgcattcggctgataaccgaaaggcaga
aggttcgaatccttctcacagtacca
1-10tRNA-Ser(tga)[26551,26640]
ggtaggtagcggctaatggtagccaaactgtcttgaaaacagttgccact
gtagagatacggtgagggttcgactcctttacttaccgcc
1-11tRNA-Leu(tag)[26897,26974]
gggagattgatggtaattggtaaacctatctcgcttagaacgagatgttt
gagggttcgaatcccttgtctcctacca
1-12tRNA-Lys(ctt)[26982,27057]
gcaggtgtagcaaaatggttatgcggctgactcttaatcagtaagacgat
gggttcaattccctccacctgtacca
1-13tRNA-Ala(tgc)[27064,27139]
tgggtcatagtttatatggttaaaattcgagttttgcaaacttgggaact
gagttcaattctcagtgactccacca
1-14tRNA-Gly(tcc)[27146,27220]

gcatccatagtttaaacgggaaaattacagtcttccaaactgaggttgag
ggttcgattccctctggatgctcca
1-15tRNA-Thr(tgt)[27650,27726]
gctgctttcgtataattggctattacacatcccttgtaaggatggaaatg
caggttcaagtcctgtgagcagcacca
1-16tRNA-Val(tac)[27822,27896]
actcgcttagtttatatggtaaaacatcacccttacaagatgaagaaaaa
ggttcaagtcctttagtgagtacca
1-17tRNA-Leu(caa)[27898,27975]
gttccagtatcccaattggcagaggatgcaagctcaaaccttgtattagt
gacggttcgaatccgtcttggaacacca
1-18tRNA-Arg(acg)[28090,28165]
gcaggattagttcaaatggatagagcaacagtctacgaagctgttaatag
gggttcgaatcccttatcctgcgcca
1-19tRNA-Gln(ttg)[28750,28825]
aggggattagtttacaaggttaaaacctcggtctttgaaatcgaagaagt
tggttcaattccaacatcccccgcca
1-20tRNA-Leu(taa)[28828,28906]
gctccattactccaattggcagagaggccagacttaaaatctgtgttatg
tatcggttcgaatccgatatggagtacca
1-21tRNA-Gln(ctg)[28912,28987]
agcggtatagcataactggcaatgcagcagtctctgaagctgtcctatta
aggttcaaatccttatgccgctgcca
1-22tRNA-His(gtg)[29019,29094]
gtggccttatcataaatggtaatgacccatgctgtgaacatggtctatac
gggttcaaatcccgtaggtcacccca
1-23tRNA-Phe(gaa)[29101,29176]
agtccaagtagcttatatggttaaagcgcgtgtctgaaaaacatgagaag
agggttcaaatcccactggactacca
1-24tRNA-Ser(gct)[29930,30022]
ggaagattaaccctaaaaggtaagggagcagtttgctaaactgccagtag
ctgagaaatcggtgtaccagttcaagtctggtatcttcctcca
1-25tRNA-Cys(gca)[30027,30102]
gaatccgtgacagaaatggatatgtgcctgtctgcaaaacaggtttataa
gggttcaagtcccttcggattctcca
>KC862296.1 Pseudomonas phage P3_CHA, complete genome
1-1tRNA-Asn(gtt)[86082,86158]
tccgttcggtccctcaaggtgagggagcttgactgttaatcaagacgtgc
ctggttcgattccagggagcggagcca
1-2tRNA-Tyr(gta)[86216,86303]
ggaggggtggcagagcggtttaatgcaccggactgtaaatccggcgtccg
accgggcatcgctggttcaaatccagccccctccacca
1-3tRNA-Gln(ttg)[86505,86581]
aggcgtgtggcgaaaaggtttaacgcactggactttgactccagcatttg

tgggttcgaatcccaccacgtctgcca
>AP014715.1 Edwardsiella phage PEi26 DNA, complete sequence
1-1tRNA-Asp(gtc)c[75896,75971]
ggacctatagtttcagcggttaaaatactcccctgtcacgggagcgtcac
gagttcgaatctcgttaggtccgcca
1-2tRNA-His(gtg)c[76195,76270]
gtggccttaattcagttggtagaattcaagattgtgattcttgtagtcat
gggttcaagtcccataggtcacccca
1-3tRNA-Asn(gtt)c[76304,76379]
ggatgtgtagctcagttggtagagcggtcgcccgttaagtgattggtccg
cggttcgaatccgtgcacatccgcca
1-4tRNA-Lys(ttt)c[76925,77002]
aggacgttagctcagctggttagagcactcgacttttaatcgagatgtcg
atgggttcaaatccctcacgtcctacca
1-5tRNA-Met(cat)c[78068,78144]
ggccctatagctcaattggttagagcgcccggctcataactggtaggttt
ccggttcaagtccggatggggccacca
1-6tRNA-Leu(taa)c[78610,78698]
gcgggtgtggcggtattggcagtacgcatggcacttaaaatgccataggg
cttaagctcttacgagttcgaatctcgtcacccgtacca
>HQ634191.1 Cyanophage Syn10 genomic sequence
1-1tRNA-Asn(gtt)c[129314,129387]
ttcctctttagctcagcggtagagcgattgactgttaatcaattggtccc
tggttcgatcccaggaaggggagt
1-2tRNA-Ala(tgc)c[129462,129534]
ggggaattagctcagttggtagagcgcctgctttgcaagcaggatgtcag
cggttcgagtccgctattctcca
1-3tRNA-Thr(tgt)c[129537,129611]
gccaaactagctcagctggatagagcaacggttttgtaaaccgtaggtca
acggttcaagtccgttgtttggctc
1-4tRNA-Leu(taa)c[129799,129885]
tgggagcgtggcggaatcggtagacgcaccagacttaaaatctgttgaga
attaatctcgtgggggttcaattccccccgctcctat
1-5tRNA-Val(tac)c[133568,133639]
gggcgaatagctcagcggtagagctactcgtttacaccgagtcggtcggg
ggttcgatcccctcttcgccca
1-6tRNA-Arg(tct)c[164477,164552]
tgggtcagtagctcagctggatagagcaactgccttctaagcagtcggtc
acaggttcgaatcctgtctgacccgt
>JF974292.1 Cyanophage S-SSM2 genomic sequence
1-1tRNA-Arg(tct)c[81382,81454]
gggtcagtagctcagtggatagagcatcgcacttctaatgcgttggtcgg
gggttcaaatccctcctgacccg
>HQ634190.1 Cyanophage Syn2 genomic sequence

1-1tRNA-Pyl(cta)[98023,98097]
tgggtcagtagttcagcggatagaacaacgctcttctaaagcgtgtgtcg
ggggttcgattccctcctgacccgt
1-2tRNA-Val(tac)[127132,127204]
gggcgaatagctcagcggtagagctactcgtttacaccgagtcggtcggg
ggttcgatcccctcttcgcccac
1-3tRNA-Leu(taa)[131657,131743]
tgggagtgtggcggaatcggtagacgcaccagacttaaaatctgttgaga
attaatctcgtgggggttcaagtccccccactcctat
1-4tRNA-Thr(tgt)[131745,131816]
gcccttatagctcagtggtagagcaacgcttttgtaaagcgtaggtcgtt
ggttcaaatccgactgggggct
1-5tRNA-Ala(tgc)[131819,131892]
ggggaattagctcagttggtagagcgcctgctttgcaagcaggatgtcag
gagttcgagtctcctattctccac
1-6tRNA-Asn(gtt)[131897,131968]
tcctctttagctcagcggtagagcggttgactgttaatcaattggtccct
ggttcgatcccaggaaggggag
>HQ317391.1 Cyanophage S-SSM6a genomic sequence
1-1tRNA-Gly(tcc)[39715,39785]
gcggatgtagtttaatggtaaaatacgaggtttccaacctcttgtcctca
gttcgattctgagtatccgct
1-2tRNA-Ile(tat)[104572,104644]
gggactatcgcatattggttaatgccctctgcttataacggagtgaaccg
agttcaattctcggtagtcctac
1-3tRNA-Thr(tgt)[104647,104722]
gccactttagctcagttggatagagcaacgcatttgtaatgcgtaggtcg
tcggttcaagtccgacaagtggctcc
1-4tRNA-Arg(tct)[104725,104800]
gactcaatagctcagttggatagagcaactgccttctaagcagtcggtcg
taggttcgagtcctacttgagtcgcc
1-5tRNA-Leu(taa)[104802,104887]
tgggagtgtggtggaatcggtagacacaccagacttaaaatctgttgaca
gcaatgtcgtgggggttcaagtccccccactcccat
>HQ316603.1 Cyanophage S-SSM6b genomic sequence
1-1tRNA-Leu(taa)c[75473,75554]
gggagcatggcggaatcggtagacgcatcggacttaaaatccgctgaggt
aactcgtgggggttcaagtccccctgctccta
1-2tRNA-Val(tac)c[80679,80750]
gggagattagctcagcggtagagctattcgtttacaccgaatcggtcatt
ggttcaagtccaatatctccca
1-3tRNA-Arg(tct)c[113704,113778]
tggtccagtagctcagcggaaagagcaactgccttctaagcagttggcca
taggttcaaatcctatctggatcgt

>HQ317291.1 Synechococcus phage S-RIM2 R9_2006, complete genome

1-1tRNA-Val(tac)[13782,13853]

gcccgaatagctcagcggtagagcacctcgtttacaccgagattgtcggg

ggttcgatcccctcttcgggca

1-2tRNA-Leu(taa)[17779,17865]

tgggagtatggcggaatcggtagacgcaccagacttaaaatttgttgagg

gttaacctcgtgagagttcaagtctctctactcctat

1-3tRNA-Thr(tgt)[17922,17994]

gcccccgtagctcagtggtagagcagggcttttgtaaagctcaggtcgca

agttcaaatcttgtcaggggctc

1-4tRNA-Ala(tgc)[17997,18069]

ggggaattagctcagttggtagagcgcctgctttgcaagcaggatgtcag

cggttcgagtccgctattctcca

1-5tRNA-Asn(gtt)[18116,18189]

ttcctcagtagctcagcggcagagccatcgactgttaatcgattggtcgt

aggttcaaatcctacctggggagt

1-6tRNA-Arg(tct)[161226,161300]

tgggtcagtagctcagcggatagagcaaccgccttctaagcggttggtcg

caggttcaaatcctgcctgacccgt

>MF158045.1 Shigella phage Sf22, complete genome

1-1tRNA-Arg(tct)c[14055,14130]

cggggcatagctcagaaggaagagcaaggaccttctaagtcctaggtcgt

aggttcgatccctactgcctcgacca

1-2tRNA-His(gtg)c[14135,14210]

gtggccgtagttcagttggtagaactcgagattgtgattctcgtagtcat

gggttcgactcccatcggtcacccca

1-3tRNA-Asn(gtt)c[14325,14399]

ggatgtgtagctcaatggcagagcgatcgcctgttaagcgattggttata

ggttcaaatcctatcacgtccgcca

1-4tRNA-Tyr(gta)c[14404,14490]

ggggagttatcccgtagaggtagcggtgtggactgtaaatccattgtcat

tgcgactcgggtggttcgactccatcactccccacca

1-5tRNA-Met(cat)c[14503,14577]

ggccctgtagctggaaggttcaagcaagcgactcataatcgccagatggt

ggttcaattccacccagggccacca

1-6tRNA-Thr(tgt)c[14579,14654]

gctgatttagctcagtaggtagagcaactcacttgtaatgagaaggtcgg

cggttcgattccgtcaatcagcacca

1-7tRNA-Ser(tga)c[14660,14749]

ggaggcgtggcagagtggtttaatgcaccggtcttgaaaaccggcagtcg

ctccggcgactcataggttcaaatcctatcgcctccgcca

1-8tRNA-Pro(tgg)c[14751,14825]

ctccgtgtagctcagtttggtagagcgcctgatttgggatcaggaggtcc

aaggttcaaatccttgtatggagac

1-9tRNA-Gly(tcc)c[14835,14908]
gcggatatcgtataatggtattacctcagacttccaatctgatgatgtga
gttcgattctcattatccgctcca
1-10tRNA-Leu(taa)c[14914,15000]
gcgagaatggtcaaattggtaaaggcacagcacttaaaatgctgcggaat
gatttccttgtgggttcgagtcccacttctcgcacca
1-11tRNA-Gln(ttg)c[15001,15074]
tgggaattagccaagttggtaaggcatagcactttgactgctagatgcaa
aggttcgagtcctttattcccagc
>MF498773.1 Aeromonas phage AS-szw, complete genome
1-1tRNA-Thr(tgt)c[62300,62375]
gccgaattagctcatctggtagagcacagcctttgtaacgctggggtggt
ccgttcgagtcggacattcggcacca
1-2tRNA-Pro(tgg)c[62655,62730]
cggtgattagcgcagttggtagcgcgtctggtttgggaccagagggtcac
aggttcgagtcctgtatcaccgacca
1-3tRNA-Tyr(gta)c[62885,62975]
ggatgttcggctacgatggcggtgtagcggcggactgtaaatccgttccc
tctgggtaaacaagttggttcgaatccaacaacatccacca
1-4tRNA-Asn(gtt)c[62982,63058]
tggcgtgtaactcagtaggttagagtggcggactgttaatccgtatgtcg
tgggttcgaatcccacctcgccagcca
1-5tRNA-Met(cat)c[63060,63137]
agcggggtggagaagtcaggagttctcaccagactcattatctggaaatc
ggtggttcaaatccacccccccgcttcca
1-6tRNA-Met(cat)c[63520,63596]
ggccccttagctcagtaggttagagcagccgactcataatcggttggtca
ctggttcgagtccagtaggggccacca
1-7tRNA-Gly(tcc)c[63681,63756]
gcgggtaaggtgtttatggatacatgcaagccttccaagtttgagtagac
cggatcgttaccggctacccgctcca
1-8tRNA-Leu(tag)c[63758,63841]
cgggatgtggtgaaattggcagacacactagatttaggttctagcgccat
aggcgtgacggttcgagtccgtccatcccgacca
1-9tRNA-Arg(tct)c[63842,63918]
gcgctgttagctcagttggatagagcaacgttcttctaaagcgtgggtca
ctggttcgaatccagtacggcgcacca
1-10tRNA-Leu(taa)c[64986,65078]
gcgtgagtggcgaagttggttgaaacgcatcggacttaaaatccgatact
gcataatggcacatcgtgggttcgattcccacctcacgcacca
>MF974178.1 Pseudomonas phage YS35, complete genome
1-1tRNA-Thr(tgt)c[68775,68849]
gcccttttaagcatttatggtgatgcaccggctttgtaacccggcgaattc
tgttcaagtcaggaatggggcacca

1-2tRNA-His(gtg)c[69036,69110]
gtggagattgtgcagcccggatgctgccttccggttgtgacccggttgtt
ggggttcgagtccccgcctccaacc
1-3tRNA-Glu(ttc)c[69171,69246]
gcagttatagattaaatggataaatcgccagactttcaatctggtgttcc
gggttcgatccccggtaactgctcca
1-4tRNA-Phe(gaa)c[69253,69328]
gcgtctgaagctaactaggtagaagcaccgggttgaaattccggaggact
tggatcgttaccaagcgggcgcacca
1-5tRNA-Gly(tcc)c[69391,69467]
gcgggtttcgtatagtctggttattattcttggcttccacccaagagact
agggttcgaatccctaagcccgcacca
1-6tRNA-Pro(tgg)c[69475,69552]
ctcctcgtagctcagtctggttagagtgtcggatttggaatccgaaggtc
gaaggttcaaatccttccggggtgacca
1-7tRNA-Asn(gtt)c[69615,69691]
tccgtacggcccatcaaggcgatggggctcggctgttaaccgaaacgcgc
taggttcgattcctaggtacggagcca
1-8tRNA-Cys(gca)c[69701,69776]
ccctcgttggccgagaggatcaggcagcagattgcaaatctgccatacat
cggttcaaatccgatacgaggctcca
1-9tRNA-Met(cat)c[70015,70087]
agcgttaagactggctggagctgtcactcggctcataaccgagcacaagt
ggttcgattccacctctcgctac
1-10tRNA-Asp(gtc)c[70188,70266]
ggcccattagctcagtctggactagagcaagcccctgtctagggcaaggt
cgtcggttcgaatccgacatgggtcgcca
1-11tRNA-Ile(gat)c[70276,70351]
agccgggtagctcaattggtagagcacccgaccgataatcgggcggttga
aggttcaagtccttctctggctacca
1-12tRNA-Leu(tag)c[70562,70646]
ggccctgtggtggaattggtatacacatcagtcttagaaactgacgccga
gaggattgagggttcaagtccctccggggccacca
1-13tRNA-Arg(tct)c[71465,71540]
gctcgtatagcgtaatggaaagcgcaacggtcttctaagccgtaaggtct
aggttcgagtcctagtacgagcgcca
1-14tRNA-Gln(ttg)c[71746,71819]
tgccgcttcgttcaatggtaggacgccagactttgaatctggagatgatg
gttcgatcccatcagcggctgcca
>MF787246.1 Lactobacillus phage LpeD, complete genome
1-1tRNA-Gly(tcc)c[136332,136402]
gcggatatggtgtagtggtaacatagctggcctccaaccagttgtcgccg
gttcaaacccgactatccgct
1-2tRNA-Thr(tgt)c[136557,136629]

tgcctcgttagttcagtggtaaaatacctgttttgtacacaggggtcact
agtccgattctagtacgaggcat
1-3tRNA-Pro(tgg)c[136631,136704]
caggatataggccagtttggtaggtcgctcggcttggaaccgagaagtcg
caagttcgaatcttgctatcctga
1-4tRNA-Trp(cca)c[136724,136798]
tatcaaagtaagcttaactggaaaactgttggactccaaatccggattta
taggttcgaatcctatctttgatgt
1-5tRNA-Phe(gaa)c[137006,137078]
gtgtccgtagctcagttggtagagcactgtgttgaagccacgggggtcgt
aggtccgaatcctatcgggcaca
1-6tRNA-Leu(caa)c[137092,137165]
ggacctgtaatccaattggcagagatagcagattcaaaatctgtaaaatg
ttggttcgaatccaactaggtcca
1-7tRNA-Leu(taa)c[137376,137452]
tgccctcttagtccaatctggcagagacaatagacttaaaatctatcaag
tgtgggtccgaatcccacagagggtat
1-8tRNA-Lys(ttt)c[137514,137585]
ttatccttagctcaggtggtagagcggcgtcttttgtagcgtatgtcacc
ggttcgactccggtaggataaa
1-9tRNA-Leu(tag)c[137717,137792]
tgccctcgtagtccaattggcagagatagtggatttagaatccatccagt
gtgagttcgagtctcaccgagggtat
1-10tRNA-Ser(tga)c[137796,137885]
tggataggtataaaggtaatggtacccctctggtcttgaaaaccagaaac
gattaaaagcgtttgtaggttcgagtcctaccctatccgt
1-11tRNA-Asn(gtt)c[137915,137988]
ttgctcagtagttcagcggtagaatgtctgattgttactcagaaggtcgt
tggtccgaatccaacctgagcagt
1-12tRNA-Ser(gct)c[138674,138763]
tggaaagttgacagagtggttgattgtggctccctgctaaggagttaatc
gtgttaagcggttcgggggttcaaatccctcactttccat
1-13tRNA-Ile(tat)c[138772,138863]
agtcctgtagcataatggtagtgcacccggtttatacccggcataaggct
ccagattagagtacgatataggttcgaatcctatcaggacta
1-14tRNA-Arg(cct)c[139328,139402]
tgtactagtaatctaattggataaaatgcttccctcctaaggaagtcttc
ccagttcgagtctgggctagtgcat
1-15tRNA-Arg(tct)c[139626,139699]
gcgtccttagctcaactggatagagcaacggccttctaagccgtaggttg
tgagttcaagtctcacaggacgca
>MF805809.1 Escherichia phage vB_EcoM_PHB05, complete genome
1-1tRNA-Met(cat)[51277,51352]
ggttccatagctcactgacgagagcgcacacctcataagtgtcgagggca

gggattgtaaccctggggaaccacca
1-2tRNA-Leu(taa)[51384,51469]
gcaagtgtggtggaattggtgatacacattgtgcttaaaacgcaacgctt
aaatgattgagggttcgaatccctccacttgcacca
1-3tRNA-Ser(tga)[51797,51886]
ggaaggtagggcgcagcggtgcgcaaccaggtttgaaccctggcccattg
tagcgatacggtgatggttcgactccattatcttcctcca
1-4tRNA-Ser(gct)[52066,52151]
ggtagattggtgaaatggtagccacaacagtttgctaaactgtcgtcgga
aacggcgtgtaggttcaagtcctacatctaccgcca
1-5tRNA-Lys(ttt)[52278,52353]
acatcgttagctcagttggtagagcaaggggcttttaactccaaggtcga
aggttcgaatccttcacgatgtacca
1-6tRNA-Tyr(gta)[52359,52445]
gtaggtatcgcatagcggcaattgctggagactgtaaatctcctgccctt
cggggcttcgttggttcgagtccaactacctgcacca
1-7tRNA-Asn(gtt)[52453,52537]
gatggattggcctagtggttgggcgacggactgttaatccgtgaacgaaa
gttctagcaaggttcaaatccttgatccatcgcca
1-8tRNA-Thr(tgt)[52628,52702]
gctcctatcgtataacggctattacggttgccttgtaagcaacttatcag
ggttcgagtccttgtgggagcacca
1-9tRNA-Gly(tcc)[53105,53179]
gcgggtatagtgttaatggtagcatatgaggtttccaccctccaggtgca
ggttcaagtcctgttactcgctcca
1-10tRNA-Gln(ttg)[53279,53353]
tggagattagcctagtgggaaggcaacggcctttgaagtcgtgatgacta
ggttcgattcctagatctccagcca
1-11tRNA-Pro(tgg)[53361,53436]
cagtgcgtagcgcagttggtagcgtgggagctttggatgcttcgggtcgc
aggttcgagtcctgccgcactgacca
1-12tRNA-Phe(gaa)[53442,53516]
gcagagatagctgagacggattagtgctaccctgaaaaggtagaaaggat
ggatcgttaccatctctctgcacca
1-13tRNA-Leu(tag)[53594,53678]
ggctaagtggtggaatggtatacacgatacgcttagaacgtattgcccca
agggattgtgagttcgagtctcaccttagctacca
1-14tRNA-Met(cat)[53767,53842]
tgatagtgtgaagctggcgtttcgagtgctttcataaggcattgcgagaa
gagttcgattctcttactatcaacca
1-15tRNA-Met(cat)[53858,53936]
cgcgggatagaggagtctggtcgtcctcgccagtttcatatgctggagat
catcggttcgaatccgattcccgcctcca
1-16tRNA-Ile(gat)[54083,54158]

tgtggtatcgtacagatggttagtacactcgaccgataatcgagaaacaa
cggttcgactccgtttaccacgacca
>MF957259.1 Salmonella phage Melville, complete genome
1-1tRNA-Arg(tct)c[62213,62288]
gcttctgtagtgtaatggatagcacacgatcgttctaaggtcggtagtct
gggttcgaatcctagcagaagcgcca
1-2tRNA-Leu(taa)c[62290,62376]
gcgagaatggtcaaattggtaaaggcatagcacttaaaatgctacggaga
attctccttgtgggttcgagtcccacttctcgcacca
1-3tRNA-Met(cat)c[62383,62458]
ggtcctgtagctcagcggtagagcggtcccctcataagggattggtcggc
agttcgaatcatgcccagggccacca
1-4tRNA-Thr(tgt)c[62499,62574]
gctgatttagctcagtaggtagagcaactcatttgtaatgagaaggccgg
cggttcgattccgtcaatcagcacca
1-5tRNA-Pro(tgg)c[62579,62655]
ctccgtatagctcagtttggtagagcgcctggtttgggaccaggaggtcc
aaggttcgaatccttgtacggagacca
1-6tRNA-Gly(tcc)c[62893,62967]
gcatccatcgtataatggctattatggctggcttccacccagcagattgg
agttcaattctccatggatgctcca
1-7tRNA-Trp(cca)c[63123,63196]
aggtctctcgtatagtggtattaccctgagctccaacctcagtgacgtgg
gttcaattcctacggggcctgcca
1-8tRNA-Ser(tga)c[63201,63285]
ggagagtagcgctagtggtagcaaaccggacttgaaatccgggccaccgg
aaacggtgagggttcaactcctttactctccgcca
1-9tRNA-His(gtg)c[63291,63366]
gtgaccgtagttcagttggtagaactcgagattgtgattctcgtagtcga
cggttcaatcccgtccggtcacccca
1-10tRNA-Gln(ttg)c[63580,63655]
tgggaattagccaagttggtaaggcaccggattttgattccgggatgcac
tggttcgagcccagtattcccagcca
1-11tRNA-Met(cat)c[63662,63737]
ggccccttagctcagtggttagagcaggcgactcataatcgcttggtcgc
tggttcaagtccagcaggggccacca
>MF919493.1 Mycobacterium phage Audrick, complete genome
1-1tRNA-Ser(gct)[30441,30524]
ggagggtgagcatcaggtgatgcagcgagattgctaatcccgtacggtaa
ccaccccgtgaggttcgaatcctcctccctccgc
1-2tRNA-Leu(cag)[30717,30792]
gctcccgtagcccaattggcaggaggcaccagattcaggatctgggcagt
gtgagttcgaatctcaccgggagtac
1-3tRNA-Leu(gag)[30912,30986]

gtctctgtaggcaaatcggaaaagccgccatcttgagggggtggtgcgtg
cgggttcgactcccgccagagacac
1–4tRNA–Leu(caa)[30987,31060]
gccgtggtaggccatctggcgagccgccgagttcaagtttcggtgtttgc
gggttcgaatcccgcccacggtac
1–5tRNA–SeC(tca)[67466,67562]
attctggcactggtgggcgagcccaccggcggcagcttcaagctgtcgct
ggccggagaaccgaccggaaacatcccgttcaacgcgacccagggcc
1–6tRNA–Pro(tgg)[91423,91497]
cggggtgtagttcagtttggaagagcgcttggtttgggaccaagatgtcg
caggttcgaatcctgtcaccccgac
1–7tRNA–Trp(cca)[91512,91582]
gggtctgtgcacagggtgcccgacggtctccaaagccgaaggcgggggtt
cgattccctccaggcctgcca
1–8tRNA–Tyr(gta)[91584,91670]
cccgtacatgcccaactggtgtttgggagcaggctgtaaatctgtggcct
tcgggacggtgaggttcgattcctcagtgcgggacca
1–9tRNA–Pyl(cta)[92724,92797]
tgcgagatcgtgcacggcgcactaggagcttctaaccctccgactcgcgg
gttcgactcccgcatctcgcaccc
1–10tRNA–Met(cat)[92945,93019]
agcggtgtagagcagctaggtagctcgccgggctcataacccggaggacg
cgtgttcgattcacgccaccgccac
1–11tRNA–Cys(gca)[93146,93217]
gcgcctttggcggaatggctacgtgctcggctgcaacccgagttatcccg
gttcgactccgggagggcgctc
1–12tRNA–Glu(ctc)[93222,93296]
ggtccgttggagtagatggacatctcgccaccctctcaaggtggagatca
cgggttcaagtcccgtacggactgc
1–13tRNA–His(gtg)[93298,93371]
gtggccgtagttcagccggtagaacgctgggttgtgatcccagtcgtcga
gggttcgagtccctccggtcaccc
1–14tRNA–Ala(tgc)[93533,93607]
gggcctgtagctccaattggtagagcagcatccttgcaagatgacggctg
tcggttcgaatccgacctggtccac
1–15tRNA–Phe(gaa)[93797,93869]
gccgtcatagctcagttggtagagcactggcctgaaaacccagtggccga
ggttcgattcctcgtgtcggcac
1–16tRNA–Val(cac)[93875,93948]
gtccgtttagctcagctggaagagcgctcggtccacacccgagaggccgc
aggttcgatccctgcaatggacac
1–17tRNA–Lys(ctt)[94067,94139]
gccttcgtagctcagtggtagagctgtcgcctcttaagcgataggtcgtt
ggttcgaatccagccgggggcac

1-18tRNA-Glu(ttc)[94144,94220]
ggtcgggtcggtctgctggtatggccagtcggattttcactccggacatt
cgcgggttcaattcccgtcccgatcgc
1-19tRNA-Gly(tcc)[94300,94372]
gcgggtgtggccgaatggctcaggcaccagatttccactctggctacgca
ggttcgattcctgtcatccgctc
1-20tRNA-Thr(cgt)[94432,94506]
gctgctgtagctcacctggcagagcgtcggcgtcgtatcccgaaggcatc
cggttcgagtccggacagcagcccc
1-21tRNA-Thr(tgt)[94507,94579]
gcctctgtggtccagcggcacggacatccgccttgtaagcggaggacccc
cgttcgatccggggtagaggctc
1-22tRNA-Thr(ggt)[94987,95059]
gctgggttagctcagtggtagagcgttcctctggtatgggaaaggcccgg
ggttcaatcccccgactcagctc
1-23tRNA-Gly(gcc)[96382,96455]
gcgaaggtagctcagctggcagagcgccaccttgccaaggtggaggtcgc
gggatcgtaacccgttcttcgctc
1-24tRNA-Asp(gtc)[96459,96531]
ggccctgtagctcagaggaagagcgccggtctgtcgaatcggaggtcgcg
gtatcgtaatccgtcagggtcgc
1-25tRNA-Met(cat)[96591,96663]
gcctcactagctcattggtagagccgctcgctcataacgtgcaggtacct
ggttcgattccagggtgaggtac
1-26tRNA-Ile(gat)[96669,96743]
gcctgttagcggactggtcgtccgatccaagctgataacttggcgtaagc
ggtgttcgattcaccgagcaggtac
1-27tRNA-Arg(acg)[96837,96909]
gcctctatggtccaacggatatgacgccggtctacggaaccggagatgcg
tgttcgattcgcgctaggggcac
1-28tRNA-Val(gac)[96952,97024]
gtccgtgtagctcaggggtagagcgcctgctcgacacgcaggaggaccga
ggttcgaaacctcgcatggacac
1-29tRNA-Arg(cct)[97198,97271]
gcctctgtagctcaacggacagagcaacgcggtcctaacgcggtggctgg
aggttcgaatcctctcggaggcac
1-31tRNA-Gln(ttg)[97812,97887]
tggggtatggtggcaatctggcagtcccgccggactttgactccggaggt
gcaggttcgagtcctgctaccccatc
1-32tRNA-Arg(tct)[97891,97966]
gcccttgtagctcagtggacagagcggcgagcttctacctcgcgggccgg
gagttcgaatctctccaggggcacca
1-33tRNA-Gln(ctg)[125078,125152]
tgctcgttggtgtaactggcaacactacggactctgactccgtcattta

ggttcgaatcctaagcgagcagcca
1-34tRNA-Asn(gtt)[125159,125234]
tggggtgtccgttaatcaggcaaacgagcggactgttaatccgcccctgc
aggttcgaatcctgccaccccagcca
>MF919494.1 Mycobacterium phage BeanWater, complete genome
1-1tRNA-Ser(gct)[30053,30136]
ggagggtgagcatcaggtgatgcagcgagattgctaatcccgtacggtaa
ccaccccgtgaggttcgaatcctcctccctccgc
1-2tRNA-Leu(cag)[30329,30404]
gctcccgtagcccaattggcaggaggcaccagattcaggatctgggcagt
gtgagttcgaatctcaccgggagtac
1-3tRNA-Leu(gag)[30524,30598]
gtctctgtaggcaaatcggaaaagccgccatcttgaggggggtggtgcgtg
cgggttcgactcccgccagagacac
1-4tRNA-Leu(caa)[30599,30672]
gccgtggtaggccatctggcgagccgccgagttcaagtttcggtgtttgc
gggttcgaatcccgcccacggtac
1-5tRNA-SeC(tca)[67775,67871]
attctggcactggtgggcgagcccaccggcggcagcttcaagctgtcgct
ggccggagaaccgaccggaaacatcccgttcaacgcgacccagggcc
1-6tRNA-Pro(tgg)[91070,91144]
cggggtgtagttcagtttggaagagcgcttggtttgggaccaagatgtcg
caggttcgaatcctgtcaccccgac
1-7tRNA-Trp(cca)[91159,91229]
gggtctgtgcacagggtgcccgacggtctccaaagccgaaggcgggggtt
cgattccctccaggcctgcca
1-8tRNA-Tyr(gta)[91231,91317]
cccgtacatgcccaactggtgtttgggagcaggctgtaaccctgtggcct
tcgggacggtgaggttcgattcctcagtgcgggacca
1-9tRNA-Pyl(cta)[92431,92503]
gcaccatttgctcaatggcagagcggcggcttctaaaaccgtgagtgccg
gttcgactccggcatggtgcacc
1-10tRNA-Met(cat)[92653,92727]
agcggtgtagagcagctaggtagctcgccgggctcataacccggaggacg
cgtgttcgaatcacgccaccgccac
1-11tRNA-Cys(gca)[92854,92925]
gcgcctttggcggaatggctacgtgctcggctgcaacccgagttatcccg
gttcgactccgggagggcgctc
1-12tRNA-Glu(ctc)[92930,93004]
ggtccgttggagtagatggatatctcgccaccctctcaaggtggagatca
cgggttcaagtcccgtacggactgc
1-13tRNA-His(gtg)[93006,93079]
gtggccgtagttcagccggtagaacgctgggttgtgatcccagtcgtcga
gggttcgagtccctccggtcaccc

1-14tRNA-Ala(tgc)[93241,93315]
gggcctgtagctccaattggtagagcagcatccttgcaagatgacggctg
tcggttcgaatccgacctggtccac
1-15tRNA-Phe(gaa)[93505,93577]
gccgtcatagctcagttggtagagcactggcctgaaaacccagtggccga
ggttcgattcctcgtgtcggcac
1-16tRNA-Val(cac)[93583,93656]
gtccgtttagctcagctggaagagcgctcggtccacacccgagaggccgc
aggttcgatccctgcaatggacac
1-17tRNA-Lys(ctt)[93776,93849]
gccttcgtagctcagttggtagagctctcgcctcttaagcgagatgtcgc
aggttcgacccctgccggaggcac
1-18tRNA-Glu(ttc)[93854,93930]
ggtcgggtcggtctgctggtatggccagtcggattttcactccggacatt
cgcgggttcaattcccgtcccgatcgc
1-19tRNA-Gly(tcc)[94010,94082]
gcgggtgtggccgaatggctcaggcaccagatttccactctggctacgca
ggttcgattcctgtcatccgctc
1-20tRNA-Thr(cgt)[94142,94216]
gctgctgtagctcacctggcagagcgtcggcgtcgtatcccgaaggcatc
cggttcgagtccggacagcagcccc
1-21tRNA-Thr(tgt)[94217,94289]
gcctctgtggtccagcggcacggacatccgccttgtaagcggaggacccc
cgttcgatccggggtagaggctc
1-22tRNA-Thr(ggt)[94349,94421]
gctgggttagctcagtggtagagcgttcctctggtatgggaaaggcccgg
ggttcaatcccccgactcagctc
1-23tRNA-Gly(gcc)[95744,95817]
gcgaaggtagctcagctggcagagcgccaccttgccaaggtggaggtcgc
gggatcgtaacccgttcttcgctc
1-24tRNA-Asp(gtc)[95821,95893]
ggccctgtagctcagaggaagagcgccggtctgtcgaatcggaggtcgcg
gtatcgtaatccgtcagggtcgc
1-25tRNA-Met(cat)[95953,96025]
gcctcactagctcattggtagagccgctcgctcataacgtgcaggtacct
ggttcgattccagggtgaggtac
1-26tRNA-Ile(gat)[96031,96105]
gcctgttagcggactggtcgtccgatccaagctgataacttggcgtaagc
ggtgttcgattcaccgagcaggtac
1-27tRNA-Arg(acg)[96199,96271]
gcctctatggtccaacggatatgacgccggtctacggaaccggagatgcg
tgttcgattcgcgctaggggcac
1-28tRNA-Val(gac)[96314,96386]
gtccgtgtagctcaggggtagagcgcctgctcgacacgcaggaggaccga

ggttcgaaacctcgcatggacac
1-29tRNA-Arg(cct)[96560,96633]
gcctctgtagctcaacggacagagcaacgcggtcctaacgcggtggctgg
aggttcgaatcctctcggaggcac
1-31tRNA-Gln(ttg)[97174,97249]
tggggtatggtggcaatctggcagtcccgccggactttgactccggaggt
gcaggttcgagtcctgctaccccatc
1-32tRNA-Arg(tct)[97253,97328]
gcccttgtagctcagtggacagagcggcgagcttctacctcgcgggccgg
gagttcgaatctctccaggggcacca
1-33tRNA-Gln(ctg)[125033,125107]
tgctcgttggtgtaactggcaacactacggactctgactccgtcatttta
ggttcgaatcctaagcgagcagcca
1-34tRNA-Asn(gtt)[125114,125190]
tggggtgtagttcaatctggcagaacgctcgactgttaatcgagtagttg
aaggttcgagtccttccatcccagcca
>MF919495.1 Mycobacterium phage Bigswole, complete genome
1-1tRNA-Ser(gct)[33880,33963]
ggagggtgagcatcaggtgatgcagcgagattgctaatcccgtacggtaa
ccaccccgtgaggttcgaatcctcctccctccgc
1-2tRNA-Leu(cag)[34159,34244]
gcctcggtgacgtaatggaagccgtgctgcactcagactgcagtgtccga
aagggcgtgagggttcgactccctcccgaggtacca
1-3tRNA-Leu(gag)[34245,34318]
agcccctgtatcccaacggcagaggaagcggtctgagaggccgttcagtg
agggttcgaatccctctggggcac
1-4tRNA-Leu(caa)[34319,34393]
gccgcagtatgcaaaccggaaaagcagccgtactcaaaatgcggtgcgtg
cgggttcgatccccgcctgcggtac
1-5tRNA-SeC(tca)[69722,69818]
attctggcactggtgggcgagcccaccggcggcagcttcaagctgtcgct
ggccggagaaccgaccggaaacatcccgttcaacgcgacccagggcc
1-6tRNA-Pro(tgg)[93826,93898]
cggggtgtagttcagtggaagagcgcttggtttgggaccaagatgtcgca
ggttcgaatcctgtcaccccgac
1-7tRNA-Trp(cca)[93915,93990]
tggggtgaagccgatctggaaggcagcggtctccaaagccgtctcatagc
gggttcgaatcccgtcacccctgcca
1-8tRNA-Tyr(gta)[93992,94078]
gccgcacatgcccaactggtgtttgggagcaggctgtaaccctgtggcct
tcgggacggtgaggttcgattcctcagtgcggtacca
1-9tRNA-Pyl(cta)[95187,95259]
gcaccatttgctcaatggcagagcggcggcttctaaaaccgtgagtgccg
gttcgactccggcatggtgcacc

1-10tRNA-Met(cat)[95409,95483]
agcggtgtagagcagctaggtagctcgccgggctcataacccggaggacg
cgtgttcgaatcacgccaccgccac
1-11tRNA-Cys(gca)[95610,95681]
gcgcctttggcggaatggctacgtgctcggctgcaacccgagttatcccg
gttcgactccgggagggcgctc
1-12tRNA-Glu(ctc)[95686,95760]
ggtccgttggagtagatggatatctcgccaccctctcaaggtggagatca
cgggttcaagtcccgtacggactgc
1-13tRNA-His(gtg)[95762,95835]
gtggccgtagttcagccggtagaacgctgggttgtgatcccagtcgtcga
gggttcgagtccctccggtcaccc
1-14tRNA-Ala(tgc)[95997,96071]
gggcctgtagctccaattggtagagcagcatccttgcaagatgacggctg
tcggttcgaatccgacctggtccac
1-15tRNA-Phe(gaa)[96261,96333]
gccgtcatagctcagttggtagagcactggcctgaaaacccagtggccga
ggttcgattcctcgtgtcggcac
1-16tRNA-Val(cac)[96339,96412]
gtccgtttagctcagctggaagagcgctcggtccacacccgagaggccgc
aggttcgatccctgcaatggacac
1-17tRNA-Lys(ctt)[96531,96604]
gccttcgtagctcagttggtagagctctcgcctcttaagcgagatgtcgc
aggttcgacccctgccggaggcac
1-18tRNA-Glu(ttc)[96609,96685]
ggtcgggtcggtctgctggtatggccagtcggattttcactccggacatt
cgcgggttcaattcccgtcccgatcgc
1-19tRNA-Gly(tcc)[96765,96837]
gcgggtgtggccgaatggctcaggcaccagatttccactctggctacgca
ggttcgattcctgtcatccgctc
1-20tRNA-Gly(tcc)[96937,97009]
gcgggtgtggccgaatggctcaggcaccagatttccactctggctacgca
ggttcgattcctgtcatccgctc
1-21tRNA-Thr(cgt)[97069,97142]
gctgctgtagctcacctggcagagcgtcggcgtcgtatcccgaaggcatc
cggttcgagtccggacagcagctc
1-22tRNA-Thr(tgt)[97216,97289]
gcccttgtagctcagtggcagagcaccgatcttgtaaatcgggttgtcga
gcgttcgattcgttcctggggctc
1-23tRNA-Thr(ggt)[97333,97405]
gctgggttagctcagtggtagagcgttcccttggtatgggaaaggcccgg
ggttcaatcccccgactcagctc
1-24tRNA-Gly(gcc)[98728,98801]
gcgaaggtagctcagctggtagagcgccaccttgccaaggtggaggtcgc

gggatcgtaacccgttcttcgctc
1−25tRNA−Asp(gtc)[98805,98877]
ggccctgtagctcagaggaagagcgccggtctgtcgaatcggaggtcgcg
gtatcgtaatccgtcagggtcgc
1−26tRNA−Met(cat)[98937,99009]
gcctcactagctcattggtagagccgctcgctcataacgtgcaggtacct
ggttcgattccagggtgaggtac
1−27tRNA−Ile(gat)[99015,99089]
gcctgttagcggactggtcgtccgatccaagctgataacttggcgtaagc
ggtgttcgattcaccgagcaggtac
1−28tRNA−Arg(acg)[99183,99255]
gcctctatggtccaacggatatgacgccggtctacggaaccggagatgcg
tgttcgattcgcgctaggggcac
1−29tRNA−Val(gac)[99298,99370]
gtccgtgtagctcaggggtagagcgcctgctcgacacgcaggaggaccga
ggttcgaaacctcgcatggacac
1−30tRNA−Arg(cct)[99544,99617]
gcctctgtagctcaacggacagagcaacgcggtcctaacgcggtggctgg
aggttcgaatcctctcggaggcac
1−32tRNA−Gln(ttg)[100146,100221]
tggggtatggtggcaatccggcagtcccgccggactttgactccggaggt
gcaggttcgagtcctgctaccccatc
1−33tRNA−Arg(tct)[100225,100300]
gcccttgtagctcagtggacagagcggcgagcttctacctcgcgggccgg
gagttcgaatctctccaggggcacca
1−34tRNA−Gln(ctg)[127755,127829]
tgctcgttggtgtaactggcaacactacggactctgactccgtcatttta
ggttcgaatcctaagcgagcagcca
1−35tRNA−Asn(gtt)[127836,127912]
tggggtgtagttcaatctggcagaacgctcgactgttaatcgagtagttg
aaggttcgagtccttccatcccagcca
>MF919499.1 Mycobacterium phage Daffodil, complete genome
1−1tRNA−Ser(gct)[31551,31634]
ggagggtgagcatctggtgatgcaggggtcctgctaaggccctacggatt
cacacccgtgagtttcgattactcctccctccgc
1−2tRNA−Leu(cag)[31731,31807]
gccctgctgagcaaactggcaaagctgccgcattcagagtgcgggtcatt
tccgggttcgactcccgggcagggtac
1−3tRNA−Leu(gag)[31927,32001]
gtctctgtaggcaaatcggaaaagccgccatcttgagggggtggtgcgtg
cgggttcgactcccgccagagacac
1−4tRNA−Leu(caa)[32002,32075]
gccgtggtaggccatctggcgagccgccgagttcaagtttcggtgtttgc
gggttcgaatcccgcccacggtac

1-5tRNA-SeC(tca)[68479,68575]
attctggcactggtgggcgagcccaccggcggcagcttcaagctgtcgct
ggccggagaaccgaccggaaacatcccgttcaacgcgacccagggcc
1-6tRNA-Pro(tgg)[91784,91858]
cggggtgtagttcagtttggaagagcgcttggtttgggaccaagttgtcg
caggttcgaatcctgtcaccccgac
1-7tRNA-Trp(cca)[91873,91943]
gggtctgtgcacagggtgcccgacggtctccaaagccgaaggcgggggtt
cgattccctccaggcctgcca
1-8tRNA-Pyl(cta)[93140,93212]
gcaccatttgctcaatggcagagcggcggcttctaaaaccgtgagtgccg
gttcgactccggcatggtgcacc
1-9tRNA-Met(cat)[93362,93436]
agcggtgtagagcagctaggtagctcgccgggctcataacccggaggacg
cgtgttcgaatcacgccaccgccac
1-10tRNA-Cys(gca)[93563,93634]
gcgcctttggcggaatggctacgtgctcggctgcaacccgagttatcccg
gttcgactccgggagggcgctc
1-11tRNA-Glu(ctc)[93639,93713]
ggtccgttggagtagatggatatctcgccaccctctcaaggtggagatca
cgggttcaagtcccgtacggactgc
1-12tRNA-His(gtg)[93715,93788]
gtggccgtagttcagccggtagaacgctgggttgtgatcccagtcgtcga
gggttcgagtccctccggtcaccc
1-13tRNA-Ala(tgc)[93950,94024]
gggcctgtagctccaattggtagagcagcatccttgcaagatgacggctg
tcggttcgaatccgacctggtccac
1-14tRNA-Phe(gaa)[94214,94286]
gccgtcatagctcagttggtagagcactggcctgaaaacccagtggccga
ggttcgattcctcgtgtcggcac
1-15tRNA-Val(cac)[94292,94365]
gtccgtttagctcagctggaagagcgctcggtccacacccgagaggccgc
aggttcgatccctgcaatggacac
1-16tRNA-Lys(ctt)[94484,94556]
gccttcgtagctcagtggtagagctgtcgcctcttaagcgataggtcgtt
ggttcgaatccagccgggggcac
1-17tRNA-Glu(ttc)[94561,94637]
ggtcgggtcggtctgctggtatggccagtcggattttcactccggacatt
cgcgggttcaattcccgtcccgatcgc
1-18tRNA-Gly(tcc)[94717,94789]
gcgggtgtggccgaatggctcaggcaccagatttccactctggctacgca
ggttcgattcctgtcatccgctc
1-19tRNA-Thr(cgt)[94849,94923]
gctgctgtagctcacctggcagagcgtcggcgtcgtatcccgaaggcatc

cggttcgagtccggacagcagcccc
1-20tRNA-Thr(tgt)[94924,94996]
gcctctgtggtccagcggcacggacatccgccttgtaagcggaggacccc
cgttcgatccggggtagaggctc
1-21tRNA-Thr(ggt)[95056,95128]
gctgggttagctcagtggtagagcgttcccttggtatgggaaaggcccgg
ggttcaatcccccgactcagctc
1-22tRNA-Gly(gcc)[96451,96524]
gcgaaggtagctcagctggcagagcgccaccttgccaaggtggaggtcgc
gggatcgtaacccgttcttcgctc
1-23tRNA-Asp(gtc)[96528,96600]
ggccctgtagctcagaggaagagcgccggtctgtcgaatcggaggtcgcg
gtatcgtaatccgtcagggtcgc
1-24tRNA-Met(cat)[96660,96732]
gcctcactagctcattggtagagccgctcgctcataacgtgcaggtacct
ggttcgattccagggtgaggtac
1-25tRNA-Ile(gat)[96738,96812]
gcctgttagcggactggtcgtccgatccaagctgataacttggcgtaagc
ggtgttcgattcaccgagcaggtac
1-26tRNA-Arg(acg)[96906,96978]
gcctctatggtccaacggatatgacgccggtctacggaaccggagatgcg
tgttcgattcgcgctaggggcac
1-27tRNA-Val(gac)[97021,97093]
gtccgtgtagctcaggggtagagcgcctgctcgacacgcaggaggaccga
ggttcgaaacctcgcatggacac
1-28tRNA-Arg(cct)[97267,97340]
gcctctgtagctcaacggacagagcaacgcggtcctaacgcggtggctgg
aggttcgaatcctctcggaggcac
1-30tRNA-Gln(ttg)[97881,97956]
tggggtatggtggcaatctggcagtcccgccggactttgactccggaggt
gcaggttcgagtcctgctaccccatc
1-31tRNA-Arg(tct)[97960,98033]
gcccttgtagctcagtggacagagcggcgagcttctacctcgcgggccgg
gagttcgaatctctccaggggcac
1-32tRNA-Lys(ttt)[98101,98177]
gggccggtatcttagtctggtcaaagaagtggacttttaatccgcgcgcc
gtgggttcgaatcccacccggcccacc
1-33tRNA-Gln(ctg)[126216,126290]
tgctcgttggtgtaactggcaacactacggactctgactccgtcatttta
ggttcgaatcctaagcgagcagcca
1-34tRNA-Asn(gtt)[126297,126372]
tggggtgtccgttaatcaggcaaacgagcggactgttaatccgcccctgc
aggttcgaatcctgccaccccagcca

>MF919513.1 Mycobacterium phage Koguma, complete genome

1–1tRNA–Ser(gct)[28941,29024]
ggagggtgagcatcaggtgatgcagcgagattgctaatcccgtacggtaa
ccaccccgtgaggttcgaatcctcctccctccgc
1–2tRNA–Leu(cag)[29217,29292]
gctcccgtagcccaattggcaggaggcaccagattcaggatctgggcagt
gtgagttcgaatctcaccgggagtac
1–3tRNA–Leu(gag)[29412,29486]
gtctctgtaggcaaatcggaaaagccgccatcttgagggggtggtgcgtg
cgggttcgactcccgccagagacac
1–4tRNA–Leu(caa)[29487,29560]
gccgtggtaggccatctggcgagccgccgagttcaagtttcggtgtttgc
gggttcgaatcccgcccacggtac
1–5tRNA–SeC(tca)[65924,66020]
attctggcactggtgggcgagcccaccggcggcagcttcaagctgtcgct
ggccggagaatcgaccggaaacatcccgttcaacgcgacccagggcc
1–6tRNA–Pro(tgg)[89638,89712]
cggggtgtagttcagtttggaagagcgcttggtttgggaccaagttgtcg
caggttcgaatcctgtcaccccgac
1–7tRNA–Trp(cca)[89727,89797]
gggtctgtgcacagggtgcccgacggtctccaaagccgaaggcgggggtt
cgattccctccaggcctgcca
1–8tRNA–Pyl(cta)[90989,91061]
gcaccatttgctcaatggcagagcggcggcttctaaaaccgtgagtgccg
gttcgactccggcatggtgcacc
1–9tRNA–Met(cat)[91211,91285]
agcggtgtagagcagctaggtagctcgccgggctcataacccggaggacg
cgtgttcgaatcacgccaccgccac
1–10tRNA–Cys(gca)[91412,91483]
gcgcctttggcggaatggctacgtgctcggctgcaacccgagttatcccg
gttcgactccgggagggcgctc
1–11tRNA–Glu(ctc)[91488,91562]
ggtccgttggagtagatggatatctcgccaccctctcaaggtggagatca
cgggttcaagtcccgtacggactgc
1–12tRNA–His(gtg)[91564,91637]
gtggccgtagttcagccggtagaacgctgggttgtgatcccagtcgtcga
gggttcgagtccctccggtcaccc
1–13tRNA–Ala(tgc)[91799,91873]
gggcctgtagctccaattggtagagcagcatccttgcaagatgacggctg
tcggttcgaatccgacctggtccac
1–14tRNA–Phe(gaa)[92063,92135]
gccgtcatagctcagttggtagagcactggcctgaaaacccagtggccga
ggttcgattcctcgtgtcggcac
1–15tRNA–Val(cac)[92141,92214]
gtccgtttagctcagctggaagagcgctcggtccacacccgagaggccgc

aggttcgatccctgcaatggacac
1-16tRNA-Lys(ctt)[92334,92407]
gccttcgtagctcagttggtagagctctcgcctcttaagcgagatgtcgc
aggttcgacccctgccggaggcac
1-17tRNA-Glu(ttc)[92412,92488]
ggtcgggtcggtctgctggtatggccagtcggattttcactccggacatt
cgcgggttcaattcccgtcccgatcgc
1-18tRNA-Gly(tcc)[92568,92640]
gcgggtgtggccgaatggctcaggcaccagatttccactctggctacgca
ggttcgattcctgtcatccgctc
1-19tRNA-Thr(cgt)[92700,92774]
gctgctgtagctcacctggcagagcgtcggcgtcgtatcccgaaggcatc
cggttcgagtccggacagcagcccc
1-20tRNA-Thr(tgt)[92775,92847]
gcctctgtggtccagcggcacggacatccgccttgtaagcggaggacccc
cgttcgatccggggtagaggctc
1-21tRNA-Thr(ggt)[92907,92979]
gctgggttagctcagtggtagagcgttcctctggtatgggaaaggcccgg
ggttcaatcccccgactcagctc
1-22tRNA-Gly(gcc)[94724,94799]
gcggttatagctcagcctggccagagcatcacgttgccaacgtgaacgtc
gcgggttcaaatcccgctagccgctc
1-23tRNA-Asp(gtc)[94813,94885]
ggccctgtagctcagaggaagagcgccggtctgtcgaatcggaggtcgcg
gtatcgtaatccgtcagggtcgc
1-24tRNA-Met(cat)[94945,95017]
gcctcactagctcattggtagagccgctcgctcataacgtgcaggtacct
ggttcgattccagggtgaggtac
1-25tRNA-Ile(gat)[95023,95097]
gcctgttagcggactggtcgtccgatccaagctgataacttggcgtaagc
ggtgttcgattcaccgagcaggtac
1-26tRNA-Arg(acg)[95191,95263]
gcctctatggtccaacggatatgacgccggtctacggaaccggagatgcg
tgttcgattcgcgctaggggcac
1-27tRNA-Val(gac)[95306,95378]
gtccgtgtagctcagtggtagagcgcctgctcgacacgcaggaggaccga
ggttcgaaacctcgcatggacac
1-28tRNA-Arg(cct)[95552,95625]
gcctctgtagctcaacggacagagcaacgcggtcctaacgcggtggctgg
aggttcgaatcctctcggaggcac
1-30tRNA-Gln(ttg)[96166,96241]
tggggtatggtggcaatctggcagtcccgccggactttgactccggaggt
gcaggttcgagtcctgctaccccatc
1-31tRNA-Arg(tct)[96245,96320]

gcccttgtagctcagtggacagagcggcgagcttctacctcgcgggccgg
gagttcgaatctctccaggggcacca
1–32tRNA–Gln(ctg)[125283,125357]
tgctcgttggtgtaactggcaacactacggactctgactccgtcattctt
ggttcgaatccagggcgagcaacca
1–33tRNA–Asn(gtt)[125364,125439]
tggggtgtccgttaatcaggcaaacgagcggactgttaatccgtccctgc
aggttcgaatcctgccaccccagcca
>MF919527.1 Mycobacterium phage Phox, complete genome
1–1tRNA–Ser(gct)[31442,31525]
ggagggtgagcatctggtgatgcaggggtcctgctaaggccctacggatt
cacacccgtgagtttcgattactcctccctccgc
1–2tRNA–Leu(cag)[31622,31698]
gccctgctgagcaaactggcaaagctgccgcattcagagtgcgggtcatt
tccgggttcgactcccgggcagggtac
1–3tRNA–Leu(gag)[31818,31892]
gtctctgtaggcaaatcggaaaagccgccatcttgagggggtggtgcgtg
cgggttcgactcccgccagagacac
1–4tRNA–Leu(caa)[31893,31966]
gccgtggtaggccatctggcgagccgccgagttcaagtttcggtgtttgc
gggttcgaatcccgcccacggtac
1–5tRNA–SeC(tca)[68756,68852]
attctggcactggtgggcgagcccaccggcggcagcttcaagctgtcgct
ggccggagaaccgaccggaaacatcccgttcaacgcgacccagggcc
1–6tRNA–Pro(tgg)[92518,92592]
cggggtgtagttcagtttggaagagcgcttggtttgggaccaagatgtcg
caggttcgaatcctgtcaccccgac
1–7tRNA–Trp(cca)[92607,92677]
gggtctgtgcacagggtgcccgacggtctccaaagccgaaggcgggggtt
cgattccctccaggcctgcca
1–8tRNA–Tyr(gta)[92679,92765]
cccgtacatgcccaactggtgtttgggagcaggctgtaaccctgtggcct
tcgggacggtgaggttcgattcctcagtgcgggacca
1–9tRNA–Pyl(cta)[93879,93951]
gcaccatttgctcaatggcagagcggcggcttctaaaaccgtgagtgccg
gttcgactccggcatggtgcacc
1–10tRNA–Met(cat)[94101,94175]
agcggtgtagagcagctaggtagctcgccgggctcataacccggaggacg
cgtgttcgaatcacgccaccgccac
1–11tRNA–Cys(gca)[94302,94373]
gcgcctttggcggaatggctacgtgctcggctgcaacccgagttatcccg
gttcgactccgggagggcgctc
1–12tRNA–Glu(ctc)[94378,94452]
ggtccgttggagtagatggatatctcgccaccctctcaaggtggagatca

cgggttcaagtcccgtacggactgc
1-13tRNA-His(gtg)[94454,94527]
gtggccgtagttcagccggtagaacgctgggttgtgatcccagtcgtcga
gggttcgagtccctccggtcaccc
1-14tRNA-Ala(tgc)[94689,94763]
gggcctgtagctccaattggtagagcagcatccttgcaagatgacggctg
tcggttcgaatccgacctggtccac
1-15tRNA-Phe(gaa)[94953,95025]
gccgtcatagctcagttggtagagcactggcctgaaaacccagtggccga
ggttcgattcctcgtgtcggcac
1-16tRNA-Val(cac)[95031,95104]
gtccgtttagctcagctggaagagcgctcggtccacacccgagaggccgc
aggttcgatccctgcaatggacac
1-17tRNA-Lys(ctt)[95224,95297]
gccttcgtagctcagttggtagagctctcgcctcttaagcgagatgtcgc
aggttcgacccctgccggaggcac
1-18tRNA-Glu(ttc)[95302,95378]
ggtcgggtcggtctgctggtatggccagtcggattttcactccggacatt
cgcgggttcaattcccgtcccgatcgc
1-19tRNA-Gly(tcc)[95458,95530]
gcgggtgtggccgaatggctcaggcaccagatttccactctggctacgca
ggttcgattcctgtcatccgctc
1-20tRNA-Gly(tcc)[95630,95702]
gcgggtgtggccgaatggctcaggcaccagatttccactctggctacgca
ggttcgattcctgtcatccgctc
1-21tRNA-Thr(cgt)[95762,95836]
gctgctgtagctcacctggcagagcgtcggcgtcgtatcccgaaggcatc
cggttcgagtccggacagcagcccc
1-22tRNA-Thr(tgt)[95837,95909]
gcctctgtggtccagcggcacggacatccgccttgtaagcggaggacccc
cgttcgatccggggtagaggctc
1-23tRNA-Thr(ggt)[95969,96041]
gctgggttagctcagtggtagagcgttcctctggtatgggaaaggcccgg
ggttcaatcccccgactcagctc
1-24tRNA-Gly(gcc)[97364,97437]
gcgaaggtagctcagctggcagagcgccaccttgccaaggtggaggtcgc
gggatcgtaacccgttcttcgctc
1-25tRNA-Asp(gtc)[97441,97513]
ggccctgtagctcagaggaagagcgccggtctgtcgaatcggaggtcgcg
gtatcgtaatccgtcagggtcgc
1-26tRNA-Met(cat)[97573,97645]
gcctcactagctcattggtagagccgctcgctcataacgtgcaggtacct
ggttcgattccagggtgaggtac
1-27tRNA-Ile(gat)[97651,97725]

gcctgttagcggactggtcgtccgatccaagctgataacttggcgtaagc
ggtgttcgattcaccgagcaggtac
1-28tRNA-Arg(acg)[97819,97891]
gcctctatggtccaacggatatgacgccggtctacggaaccggagatgcg
tgttcgattcgcgctaggggcac
1-29tRNA-Val(gac)[97934,98006]
gtccgtgtagctcaggggtagagcgcctgctcgacacgcaggaggaccga
ggttcgaaacctcgcatggacac
1-30tRNA-Arg(cct)[98180,98253]
gcctctgtagctcaacggacagagcaacgcggtcctaacgcggtggctgg
aggttcgaatcctctcggaggcac
1-32tRNA-Gln(ttg)[98794,98869]
tggggtatggtggcaatctggcagtcccgccggactttgactccggaggt
gcaggttcgagtcctgctaccccatc
1-33tRNA-Arg(tct)[98873,98948]
gcccttgtagctcagtggacagagcggcgagcttctacctcgcgggccgg
gagttcgaatctctccaggggcacca
1-34tRNA-Gln(ctg)[125841,125915]
tgctcgttggtgtaactggcaacactacggactctgactccgtcattctt
ggttcgaatccagggcgagcaacca
1-35tRNA-Asn(gtt)[125922,125997]
tggggtgtccgttaatcaggcaaacgagcggactgttaatccgtccctgc
aggttcgaatcctgccaccccagcca
>KX349249.1 Synechococcus phage S-RIM2 isolate Np_20_0912, complete genome
1-1tRNA-Val(tac)[13794,13865]
gcccgaatagctcagcggtagagcacctcgtttacaccgagattgtcggc
ggttcgatcccgtcttcgggca
1-2tRNA-Leu(taa)[17791,17877]
tgggagtatggcggaatcggtagacgcaccagacttaaaatttgttgagg
gttaacctcgtgagagttcaagtctctctactcctat
1-3tRNA-Thr(tgt)[17934,18006]
gcccccgtagctcagtggtagagcagggcttttgtaaagctcaggtcgca
agttcaaatcttgtcaggggctc
1-4tRNA-Ala(tgc)[18009,18081]
ggggaattagctcagttggtagagcgcctgctttgcaagcaggatgtcag
cggttcgagtccgctattctcca
1-5tRNA-Asn(gtt)[18128,18201]
ttcctcagtagctcagcggcagagccatcgactgttaatcgattggtcgt
aggttcaaatcctacctggggagt
1-6tRNA-Arg(tct)[161241,161315]
tgggtcagtagctcagcggatagagcaaccgccttctaagcggttggtcg
caggttcaaatcctgcctgacccgt
>AF234173.1 Enterobacteria phage P1 mod1902::IS5 c1.100 rev dmt(del)MB mutant, complete genome
1-1tRNA-Asn(gtt)[69029,69104]

gatggtgtagctcagcggttagagcggttgactgttaatcaacgggtcga
tggttcaaatccatccaccatcgcca
1-2tRNA-Thr(tgt)[69107,69182]
gccggtttagctcagttggtagagcgcctgccttgtaagcaggatgtcag
cggttcgagtccgttaatcggcacca
1-3tRNA-Met(cat)[70614,70689]
ggcccttagctcagtggttagagctggcgactcataatcgcacggtcac
cggttcaagtccggtaggggccacca
>KX147096.1 Serratia phage vB_Sru_IME250, complete genome
1-1tRNA-Tyr(gta)c[65698,65781]
gtgagagtggcagagcggtcgattgcgggggactgtaaatcccttctgaa
aggcgcggtggttcgaatccatcctctcacacca
1-2tRNA-Asn(gtt)c[66666,66741]
gatggtgtagttcagtcggtagaacggcggtctgttaaatcgtatgtcgc
aggttcaagtcctgtcaccatcgcca
1-3tRNA-Trp(cca)c[66751,66826]
atctccttggtctagcggctatgatgccagcctccaaagccgtgcgaccc
aggttcgagtcctggaggggatgcca
1-4tRNA-Met(cat)c[67068,67143]
ggtcctgtagctcagtggttagagcagtcgactcataatcgattggtcat
tggttcaaaaccaatcaggatcacca
>KY073123.1 Serratia phage vB_Sru_IME250, complete genome
1-1tRNA-Tyr(gta)c[65698,65781]
gtgagagtggcagagcggtcgattgcgggggactgtaaatcccttctgaa
aggcgcggtggttcgaatccatcctctcacacca
1-2tRNA-Asn(gtt)c[66666,66741]
gatggtgtagttcagtcggtagaacggcggtctgttaaatcgtatgtcgc
aggttcaagtcctgtcaccatcgcca
1-3tRNA-Trp(cca)c[66751,66826]
atctccttggtctagcggctatgatgccagcctccaaagccgtgcgaccc
aggttcgagtcctggaggggatgcca
1-4tRNA-Met(cat)c[67068,67143]
ggtcctgtagctcagtggttagagcagtcgactcataatcgattggtcat
tggttcaaaaccaatcaggatcacca
>GU071092.1 Prochlorococcus phage P-SSM2, complete genome
1-1tRNA-Asn(gtt)[164089,164176]
ttgtgaggtgacgaaattggtaaacgtgtcagtctgtttaactgatgttc
ctggcgggacttgaaggttcgactccttccctcacagt
>KM606995.1 Enterobacteria phage RB5, complete genome
1-1tRNA-Arg(tct)c[67416,67491]
cgaggcatagctcagaaggaagagcaaggaccttctaagtcctgggtcgt
aggttcgatccctactgcctcgacca
1-2tRNA-Asn(gtt)c[67496,67570]
ggatgtgtagctcaatggcagagcgatcgcctgttaagcgattggttata

ggttcgaatcctatcacgtccgcca
1-3tRNA-Tyr(gta)c[67575,67661]
ggggagttatcccgtagaggtagcggtgtggactgtaaatccattgtcat
tgcgactcgggtggttcgactccatcactccccacca
1-4tRNA-Met(cat)c[67671,67745]
ggccctgtagctggaaggttcaagcaagcgactcataatcgccagatggt
ggttcaattccacccagggccacca
1-5tRNA-Thr(tgt)c[67747,67822]
gctgatttagctcagtaggtagagcacctcacttgtaatgaggatgtcgg
cggttcgattccgtcaatcagcgcca
1-6tRNA-Ser(tga)c[67828,67917]
ggaggcgtggcagagtggtttaatgcaccggtcttgaaaaccggcagtcg
ctccggcgactcataggttcaaatcctatcgcctccgcca
1-7tRNA-Pro(tgg)c[67919,67993]
ctccgtgtagctcagtttggtagagcgcctgatttgggatcaggaggtcc
aaggttcaaatccttgtatggagac
1-8tRNA-Gly(tcc)c[68004,68077]
gcggatatcgtataatggcattacctcagacttccaatctgatgatgtga
gttcgattctcattatccgctcca
1-9tRNA-Leu(taa)c[68083,68169]
gcgagaatggtcaaattggtaaaggcacagcacttaaaatgctgcggaat
gatttccttgtgggttcgagtcccacttctcgcacca
1-10tRNA-Gln(ttg)c[68170,68243]
tgggaattagccaagttggtaaggcactggattttgattccaggatgcaa
aggttcgagtcctttattcccagc
>KF148616.1 Campylobacter phage CP8, complete genome
1-1tRNA-Tyr(gta)c[38499,38584]
gtaagcatatgggtaattggtaacccaccagactgtaaatctggcgtctc
ttggcactgcaggttcgagtcctgctgcttacacca
1-2tRNA-Arg(tct)c[38595,38671]
ctccatgtagctcagctggatagagcaagaatcttctaagttctaggtcg
ggtgttcaaatcactccatggaggcca
1-3tRNA-Asn(gtt)c[38681,38756]
tgcggattagcacagtggtagtgcaatcgactgttaatcgatggggtcat
aggttcgaatcctatatccgtagcca
1-4tRNA-Met(cat)c[39027,39101]
agcagagtgacgcagtagtagcgtgctgggctcataacccagatgtcgta
ggtgcaaatcctacctctgtaacca
1-5tRNA-Leu(taa)c[39107,39184]
tggacatgtagcgctcaagtggtagagcagccgacttaaaatctgcagat
tgatggttcgatcccatccatgtccaac
1-6tRNA-Met(cat)c[39189,39263]
gggcgagtagctcaatggtagagcaaccggctcataaccggttggttata
ggttcgattcctatttcgcccacca

>KF156339.1 Synechococcus phage S-MbCM25, complete genome

1-1tRNA-Leu(taa)[17219,17305]
tgggagtgtggcggaatcggtagacgcaccagacttaaaatctgttgaga
attaatctcgtgggagttcaagtctccctactcccat
1-2tRNA-Thr(tgt)[19060,19132]
gcctccgtagctcagtggtagagcagggcttttgtaaagctcaggtcgca
agttcaaatcttgtcagaggctc
1-3tRNA-Asn(gtt)[19137,19209]
tcctctatagctcagttggtagagcaggtgactgttaatcaccctgtccc
tggttcgagtccaggtggaggag
1-4tRNA-Arg(tct)[158217,158291]
tgggtcagtagctcagcggatagagccacgcacttctaatgcgttggtcg
caggttcgattcctgcctgactcgt

>KJ586794.1 Listeria phage LMTA-34, complete genome

1-1tRNA-Met(cat)c[41701,41774]
ttgtcccgtagctagaaggtcgagcaaggagctcataactcctcggtttg
ggttcgattcccaacggggcaatc
1-2tRNA-Pro(tgg)c[42667,42741]
cagggtgtagctcagtttggttagagtacccgctttggagacgggaagtc
gtaggttcgaatcctaccaccttga
1-3tRNA-Arg(tct)c[43859,43929]
gtccttatggtgtagtggatgcacaagggatttctactcccttagcgcgg
gttcgaatcctgctgaggact
1-4tRNA-Gly(tcc)c[44176,44246]
gcgggtatagtataagggtagtacccaaggtttccaaccatgtagtgggg
gttcgaatccccctacccgct
1-5tRNA-Asn(gtt)c[44315,44388]
gtgtccttaactcagaggtcagagtgccgtcctgttaagtcggaagtcgc
tggttcaaatccagcaggatacgc
1-6tRNA-Ser(tga)c[44626,44717]
ggaaggttggtagagcttggtaatacgctagtcttgaaaactagcttacc
cttggaatacagggtacaagggttcgaatcccttaccttcct
1-7tRNA-Phe(gaa)c[44730,44801]
gtagtcctagctgagatggattagcgcttgcttgaaaagcaagagaggca
ggttcgatacctgcggactcca
1-8tRNA-Lys(ttt)c[44807,44878]
ggagttatggtgaaatggctatcactgcgggtttttaccccgttattcta
ggttcgaatcctagtggctcca
1-9tRNA-Tyr(gta)c[45202,45283]
gtgtcattcgcatagtggcaattgcaggggactgtaactcccctcccttc
ggggttccaaggttcgagtccttgatggcgca
1-10tRNA-Leu(tag)c[45644,45728]
tgccgagatggtggaactggtatacacggtagacttagaatctgctgtcc
caaggatatgtgggttcgaatcccactctcggtat

1-11tRNA-Asp(gtc)c[46194,46266]
gtgcgtatgatataatggctattatactcgactgtctatcgagaaatagg
ggttcgattccccttacgtgcgc
1-12tRNA-Ile(gat)c[46370,46442]
gccagcatagcttagtaggcaaagcaaccgaccgataatcggtagtcctt
ggttcaattccaagtgttggtac
1-13tRNA-Ser(gct)c[46619,46705]
ggagagttgtcagagaggcttaatgatacgggttgctaactcgttgtact
agtaatagtaccaagggttcgaatcccttactctcct
1-14tRNA-Cys(gca)c[46785,46855]
gcgggtataaccaactggaaaggtagtagactgcaaatctacgtatatgg
gttcaattcccattacccgct
>KJ591605.1 Listeria phage LMTA-57, complete genome
1-1tRNA-Cys(gca)[110865,110935]
gcgggtataaccaactggaaaggtagtagactgcaaatctacgtatatgg
gttcaattcccattacccgct
1-2tRNA-Ser(gct)[111015,111101]
ggagagttgtcagagaggcttaatgatacgggttgctaactcgttgtact
agtaatagtaccaagggttcgaatcccttactctcct
1-3tRNA-Ile(gat)[111278,111350]
gccagcatagcttagtaggcaaagcaaccgaccgataatcggtagtcctt
ggttcaattccaagtgttggtac
1-4tRNA-Asp(gtc)[111454,111526]
gtgcgtatgatataatggctattatactcgactgtctatcgagaaatagg
ggttcgattccccttacgtgcgc
1-5tRNA-Leu(tag)[111992,112076]
tgccgagatggtggaactggtatacacggtagacttagaatctgctgtcc
caaggatatgtgggttcgaatcccactctcggtat
1-6tRNA-Tyr(gta)[112437,112518]
gtgtcattcgcatagtggcaattgcaggggactgtaactcccctcccttc
ggggttccaaggttcgagtccttgatggcgca
1-7tRNA-Lys(ttt)[112842,112913]
ggagttatggtgaaatggctatcactgcgggtttttaccccgttattcta
ggttcgaatcctagtggctcca
1-8tRNA-Phe(gaa)[112919,112990]
gtagtcctagctgagatggattagcgcttgcttgaaaagcaagagaggca
ggttcgatacctgcggactcca
1-9tRNA-Ser(tga)[113003,113094]
ggaaggttggtagagcttggtaatacgctagtcttgaaaactagcttacc
cttggaatacagggtacaagggttcgaatcccttaccttcct
1-10tRNA-Asn(gtt)[113332,113405]
gtgtccttaactcagaggtcagagtgccgtcctgttaagtcggaagtcgc
tggttcaaatccagcaggatacgc
1-11tRNA-Gly(tcc)[113474,113544]

gcgggtatagtataagggtagtacccaaggtttccaaccatgtagtgggg
gttcgaatccccctacccgct
1-12tRNA-Arg(tct)[113791,113861]
gtccttatggtgtagtggatgcacaagggatttctactcccttagcgcgg
gttcgaatcctgctgaggact
1-13tRNA-Pro(tgg)[114979,115053]
cagggtgtagctcagtttggttagagtacccgctttggagacgggaagtc
gtaggttcgaatcctaccaccttga
1-14tRNA-Met(cat)[115946,116019]
ttgtcccgtagctagaaggtcgagcaaggagctcataactcctcggtttg
ggttcgattcccaacggggcaatc
>KJ586795.1 Listeria phage LMTA-94, complete genome
1-1tRNA-Cys(gca)[100460,100530]
gcgggtataaccaactggaaaggtagtagactgcaaatctacgtatatgg
gttcaattcccattacccgct
1-2tRNA-Ser(gct)[100610,100696]
ggagagttgtcagagaggcttaatgatacgggttgctaactcgttgtact
agtaatagtaccaagggttcgaatcccttactctcct
1-3tRNA-Ile(gat)[100885,100957]
accagcatagcttagtaggcaaagcaaccgaccgataatcggtagtcctt
ggttcaattccaagtgttggtac
1-4tRNA-Asp(gtc)[101062,101134]
gtgcgtatgatataatggctattatactcgactgtctatcgagaagtagg
ggttcgattccccttacgtgcgc
1-5tRNA-Leu(tag)[101600,101684]
tgccgagatggtggaactggtatacacggtagacttagaatctgctgtcc
caaggatatgtgggttcgaatcccactctcggtat
1-6tRNA-Tyr(gta)[102045,102126]
gtgtcattcgcatagtggcaattgcaggggactgtaactcccctcccttc
ggggttccaaggttcgagtccttgatggcgca
1-7tRNA-Lys(ttt)[102450,102521]
ggagttatggtgaaatggctatcactgcgggtttttaccccgttattcta
ggttcgaatcctagtggctcca
1-8tRNA-Phe(gaa)[102527,102598]
gtagtcctagctgagatggattagcgcttgcttgaaaagcaagagaggca
ggttcgatacctgcggactcca
1-9tRNA-Ser(tga)[102611,102702]
ggaaggttggtagagcttggtaatacgctagtcttgaaaactagcttacc
cttggaatacagggtacgagggttcaaatcccttacctccct
1-10tRNA-Asn(gtt)[103223,103296]
gtgtccttaactcagaggtcagagtgccgtcctgttaagtcggaagtcgc
tggttcaaacccagcaggatacgc
1-11tRNA-Gly(tcc)[103365,103435]
gcgggtatagtataagggtagtacccaaggtttccaaccatgtagtgggg

gttcgaatccctctacccgct
1-12tRNA-Arg(tct)[103693,103763]
gtccttatggtgtagtggatgcacaagggatttctactcccttagcgcgg
gttcgaatcctgctgaggact
1-13tRNA-Pro(tgg)[104882,104956]
cagggtgtagctcagtttggttagagtacccgctttggagacgggaagtc
gtaggttcgaatcctaccaccttga
1-14tRNA-Met(cat)[105850,105923]
ttgtcccgtagctagaaggtcgagcaaggagctcataactcctcggtttg
ggttcgattcccaacggggcaatc
>KT919972.1 Vibrio phage phi-Grn1, complete genome
1-1tRNA-Ser(gct)c[37085,37173]
ggagatatggtctaaaggtatgacagcaccctgctaaggtgtcggacgtt
aatagcgttctctgagttcgaatctcagtatctccgcca
1-2tRNA-Leu(caa)c[37614,37690]
gcccaactagtccaattggcagaggcactagtttcaaacactaggagttc
cgagttcgaatctcgggttgggcacca
1-3tRNA-Leu(taa)c[37807,37883]
gcgcacttggtccaattggcagaggcatgaggcttaaaatctcagggatg
gcggttcgaatccgcccgtgcgtacca
1-4tRNA-Cys(gca)c[37886,37959]
gctcgaatcgtataatggaagtatgacggattgcaaatccgcaggtcagg
gttcgattccctgttcgagctcca
1-5tRNA-His(gtg)c[37972,38048]
gtggcagtgggtgaagtggaaataccccgggttgtgattccggaagatg
cgggttcgatccccgtctgtcacccca
1-6tRNA-Val(tac)c[38440,38513]
ggtcccttagtataatggcagtacgtctcttttacacagagaaagaagtg
gctcgattccactagggactacca
1-7tRNA-Gly(tcc)c[38520,38593]
gcgggtatgatgtaatggtagcatgacgtccttccaagtcgttcgtctcg
gttcgagtccgtgtacccgctcca
1-8tRNA-Met(cat)c[38783,38858]
ggtgatgtagctcaagtggcagagcaacgctttcatacggcgtaagatcc
gatttcgagtatcggtgtcacctcca
1-9tRNA-Arg(tct)c[39383,39458]
gcgctgttagctcaattggaagagcacgtcccttctaaggatggggttat
gagttcgaatctcatacggcgtgcca
1-10tRNA-Thr(tgt)c[40180,40254]
gctcctgaagcattgatggcgatgcagcggtcttgtaaaccgcagaattc
ggttcgattccgaacgggagcacca
1-11tRNA-Gln(ttg)c[40384,40457]
tggggattggtgtaaaggcagcataacgtactttgaatgcgttggtatca
gttcgaatctgttatccccagcca

1-12tRNA-Asp(gtc)c[40477,40552]
ggagccgaggtgtaaaaggttgcatgtctccctgtcacggagaaggtagc
gggttcgaaacccgtcggttccgcca
1-13tRNA-Asn(gtt)c[40622,40697]
gcttcattaactcagttggtagagtgtctgactgttaatcagagagtcgt
tggttcgagtccaacatgaagcgcca
1-14tRNA-Asn(gtt)c[40699,40773]
gggtgtgtaactcagaggcagagtagcaagctgttaacttgcaagtcgag
atttcgaaattctccacgcccgcca
1-15tRNA-Trp(cca)c[40849,40922]
aggggcatgatgtaaaggcagcatgacggattccaaacccgttcgttaga
gttcgaatctctatgcccctgcca
1-16tRNA-Ile(gat)c[40929,41002]
agtcccttagctcaacggtagagcgtgcgaccgataatcgcttggtaaga
gttcgattctcttagggactacca
1-17tRNA-Ser(tga)c[41066,41151]
gaaagattctgggtagcggcaacggtcttgaaaaccgtcggtcaccggga
ggtgatgttagggttcgaatccctagtcttccgcca
1-18tRNA-Met(cat)c[41176,41249]
ttgcggggtagagcagtggtcagctcgtgagtttcataagctcaaggtcg
ttggttcgaatccagccccacgca
1-19tRNA-Tyr(gta)c[41351,41436]
ggagcgtacgtctcaaggtgagacaggggactgtaaatccctgacccttaa
acgggtagagaggttcgattcctctacgctccacca
1-20tRNA-Glu(ttc)c[41443,41517]
tctcccgtggactaacggtaggtcatcaccctttcaaggtgaagtgcgcg
agttcgaatctcgtcgggagaacca
1-21tRNA-Lys(ttt)c[41581,41657]
gcgtcggtagctcatcatggaagagcagggagcttttaactctcaggtgt
ctggttcgagtccagggcggcgtacca
1-22tRNA-Lys(ttt)c[41669,41742]
gggtctatgatgtaacggtagcatatgggacttttaatccctcagttaga
gttcgaatctctgtggacccacca
1-23tRNA-Phe(gaa)c[41749,41823]
gcacccttagcttatcaggaaaagcggcggattgaagtcccgcagtgctc
ggttcgattccgaggggggtgcacca
1-24tRNA-Leu(tag)c[41825,41902]
gcgcaagtagcccaatctggcagaggcactggtcttagaaaccagaagtt
aagagttcgaatctcttcttgcgtacca
1-25tRNA-Arg(acg)c[42449,42524]
gcccgattagctcaattggaagagcagcgccctacgaaggcgaaggttac
aagttcgaatcttgtattgggtgcca
1-26tRNA-Pro(tgg)c[42533,42609]
ccgtgactagctcaatctggtagagtactccgtttggggcggagaagtta

agcgttcgaatcgcttgtcacggacca
1–27tRNA–Pro(tgg)c[42619,42692]
cgggacgtggcgtaaaggtagcgttcatgctttgggagcatgtggtaaga
gttcgagtctcttcgtcccgacca
1–28tRNA–Met(cat)c[44869,44946]
ggccctatagctcaacacggttagagcaaccgactcataatcggtaggtt
acaggttcgagtcctgttggggtcacca
>KT919973.1 Vibrio phage phi–ST2, complete genome
1–1tRNA–Ser(gct)c[98264,98352]
ggaagcatggtctaaaggtatgacagcaccctgctaaggtgtcggacgtt
aatagcgttctctgggttcgattcccagtgcttccgcca
1–2tRNA–Leu(caa)c[98521,98597]
gcccaactagtccaattggcagaggcactagtttcaaacactaggagttc
cgagttcgaatctcgggttgggcacca
1–3tRNA–Leu(taa)c[98714,98790]
gcgcacgtggtccaattggcagaggcatgaggcttaaaatctcagggatg
gcggttcgaatccgcccgtgcgtacca
1–4tRNA–Cys(gca)c[98793,98866]
gctcgaatcgtataatggaagtatgacggattgcaaatccgcaggtcagg
gttcgattccctgttcgagctcca
1–5tRNA–His(gtg)c[98879,98955]
gtggcagtgggtgaagtggaaatacccccgggttgtgattccggaagatg
cgggttcgatccccgtctgtcacccca
1–6tRNA–Val(tac)c[99371,99444]
ggtcccttagtataatggcagtacgtctcttttacacagagaaagaagtg
gctcgattccactagggactacca
1–7tRNA–Gly(tcc)c[99451,99524]
gcgggtatgatgtaatggtagcatgacgtccttccaagtcgttcgtctcg
gttcgagtccgtgtacccgctcca
1–8tRNA–Met(cat)c[99714,99789]
ggtgatgtagctcaagtggcagagcaacgctttcatacggcgtaagatcc
gatttcgagtatcggtgtcacctcca
1–9tRNA–Arg(tct)c[100314,100389]
gcgctgttagctcaattggaagagcacgtcccttctaaggatggggttat
gagttcgaatctcatacggcgtgcca
1–10tRNA–Thr(tgt)c[100780,100855]
gccctgatagcacaactggcagtgcagctcacttgtaatgagcaggttcg
cggttcgaatccgtgtcagggcacca
1–11tRNA–Gln(ttg)c[100983,101056]
tggggattggtgtaaaggcagcataacgtactttgaatgcgttggtatca
gttcgaatctgttatccccagcca
1–12tRNA–Asp(gtc)c[101076,101151]
ggagccgaggtgtaagtggttgcatgtctccctgtcacggagaaggtagc
gggttcgaaacccgtcggttccgcca

1-13tRNA-Asn(gtt)c[101220,101295]
ggatcgctaactcaatggttagagtgctcgcctgttaagcgaggagttcc
gagttcgagtctcgggcggtccgcca
1-14tRNA-Trp(cca)c[101371,101444]
aggggcatgatgtaaaggcagcatgacggattccaaacccgttcgttaga
gttcgaatctctatgcccctgcca
1-15tRNA-Ile(gat)c[101451,101524]
agtcccttagctcaacggtagagcgtgcgaccgataatcgcttggtaaga
gttcgattctcttagggactacca
1-16tRNA-Ser(tga)c[101589,101674]
gagagattctgggtagcggcaacggtcttgaaaaccgtcggtcaccggga
ggtgatgttagggttcgaatccctagtcttccgcca
1-17tRNA-Met(cat)c[101987,102060]
ttgcggggtagagaagtggtcatctcgtcggtctcataagccggagaacg
ctggttcgaatccagccccacgca
1-18tRNA-Tyr(gta)c[102162,102247]
ggagcgtacgtctcaaggtgagacaggggactgtaaatccctgaccctta
acgggtagagaggttcgattcctctacgctccacca
1-19tRNA-Glu(ttc)c[102254,102328]
tctcccgtggactaacggtaggtcatcaccctttcaaggtgaagtgcgcg
agttcgaatctcgtcgggagaacca
1-20tRNA-Lys(ttt)c[102392,102468]
gcgtcggtagctcatcatggaagagcagggagcttttaactctcaggtgt
ctggttcgagtccagggcggcgtacca
1-21tRNA-Phe(gaa)c[102480,102554]
gcacccttagcttatcaggaaaagcggcggattgaagtcccgcagtgctc
ggttcgattccgaggggggtgcacca
1-22tRNA-Leu(tag)c[102556,102632]
gcgcaagtagcccaattggcagaggcactggtcttagaaaccagaagtta
agagttcgaatctcttcttgcgtacca
1-23tRNA-Arg(acg)c[102987,103062]
gcccgattagctcaattggaagagcagcgccctacgaaggcgaaggttac
aagttcgaatcttgtattgggtgcca
1-24tRNA-Pro(tgg)c[103071,103147]
ccgtgactagctcaatctggtagagtactccgtttggggcggagaagtta
agcgttcgaatcgcttgtcacggacca
1-25tRNA-Pro(tgg)c[103157,103230]
cgggacgtggcgtaaaggtagcgttcatgctttgggagcatgtggtaaga
gttcgagtctcttcgtcccgacca
1-26tRNA-Met(cat)c[105074,105151]
ggccctatagctcaacttggttagagcaaccgactcataatcggtaggtt
acaggttcgagtcctgttggggtcacca
>KU130127.1 Pseudomonas phage vB_PsyM_KIL2, complete genome
1-1tRNA-Ser(tga)[15801,15895]

ggcttggtaagccgagatggcctagcggcagcggtcttgaaaaccgaagg
ttcacggaagtggacgtgtgagttcgagtctcacccaatccgcca
1-2tRNA-Met(cat)[15902,15978]
tggcctttagcataattggttaatgcaatcaactcataattgataagata
cgagttcaagtctcgtggggccaacca
1-3tRNA-Arg(tct)[16549,16625]
atctccatagctcaactggacagagcaagggtcttctacacccaaggttg
agggttcaagtccttctggggatacca
1-4tRNA-Thr(tgt)[16960,17036]
gcccgtttagctccaattggtagagcgcctgtcttgtaatcaggatgttt
gcggttcgaatccgtaaatgggcacca
1-5tRNA-Arg(acg)[17136,17210]
agtcgcttggccgattggattaggcaaaggtccacgaagcctgctagagt
ggttcgactccactagttactacca
1-6tRNA-Asn(gtt)[17451,17536]
tggcgtgtaagcataatggtactgcactccgctgttaacggagcttccga
aaggtcttttaggttcgagtcctaccgcgtcagcca
1-7tRNA-Pro(tgg)[17804,17877]
ctcctcgaagcgttacggtagcgtacttcgtttggaacgaagtggcggtg
gttcgactccacccgaggtgacca
1-8tRNA-Leu(tag)[17938,18021]
gcccttgtgatgaaatggtagacgtgccgggtttagattccggtgtcgaa
aggcgtgtcggttcgagtccgactgagggcacca
1-9tRNA-Gly(tcc)[18508,18582]
gcgggtataactcagaggcagagtgtcagccttccaagctgttcgtcggg
atttcgaaattccctatccgctcca
>KU130128.1 Pseudomonas phage vB_PsyM_KIL3, complete genome
1-1tRNA-Ser(tga)[16011,16105]
ggcttggtaagccgagatggcctagcggcagcggtcttgaaaaccgaggg
ttcacggaagtggacgtgtgagttcgagtctcacccaatccgcca
1-2tRNA-Met(cat)[16112,16188]
tggcctttagcataattggttaatgcaatcaactcataattgataagata
cgagttcaagtctcgtggggccaacca
1-3tRNA-Arg(tct)[16759,16835]
atctccatagctcaactggacagagcaagggttttctacacccaaggttg
agggttcgaatccttctggggatacca
1-4tRNA-Thr(tgt)[17460,17535]
gccggaatagcacaattggtagtgcagtcgccttgtaagcgaaaggttga
gggttcaagtcctttttcggcacca
1-5tRNA-Arg(acg)[17725,17799]
agtcgcttggccgattggattaggcaaaggtccacgaagcctgctagagt
ggttcgactccactagttactacca
>KU130131.1 Pseudomonas phage vB_PsyM_KIL3b, complete genome
1-1tRNA-Ser(tga)[16018,16112]

ggcttggtaagccgagatggcctagcggcagcggtcttgaaaaccgaggg
ttcacggaagtggacgtgtgagttcgagtctcacccaatccgcca
1-2tRNA-Met(cat)[16119,16195]
tggcctttagcataattggttaatgcaatcaactcataattgataagata
cgagttcaagtctcgtggggccaacca
1-3tRNA-Arg(tct)[16766,16842]
atctccatagctcaactggacagagcaagggttttctacacccaaggttg
agggttcgaatccttctggggatacca
1-4tRNA-Thr(tgt)[17467,17542]
gccggaatagcacaattggtagtgcagtcgccttgtaagcgaaaggttga
gggttcaagtcctttttcggcacca
1-5tRNA-Arg(acg)[17732,17806]
agtcgcttggccgattggattaggcaaaggtccacgaagcctgctagagt
ggttcgactccactagttactacca
>KU130129.1 Pseudomonas phage vB_PsyM_KIL4, complete genome
1-1tRNA-Ser(tga)[15590,15684]
ggcttggtaagccgagatggcctagcggcagaggtcttgaaaaccgaagg
ttcacggaagtggacgtgtgagttcgagtctcacccaatccgcca
1-2tRNA-Met(cat)[15691,15767]
tggcctttagcataattggttaatgcaatcaactcataattgataagata
cgagttcaagtctcgtggggccaacca
1-3tRNA-Arg(tct)[16339,16415]
atctccatagctcaactggacagagcaagggtcttctacacccaaggttg
agggttcaagtccttctggggatacca
1-4tRNA-Thr(tgt)[16750,16826]
gcccgtttagctccaattggtagagcgcctgtcttgtaatcaggatgttt
gcggttcgaatccgtaaatgggcacca
1-5tRNA-Arg(acg)[16926,17000]
agtcgcttggccgattggattaggcaaaggtccacgaagcctgctagagt
ggttcgactccactagttactacca
1-6tRNA-Asn(gtt)[17241,17326]
tggcgtgtaagcataatggtactgcactccgctgttaacggagcttccga
aaggtcttttaggttcgagtcctaccgcgtcagcca
1-7tRNA-Pro(tgg)[17594,17667]
ctcctcgaagcgttacggtagcgtacttcgtttggaacgaagtggcggtg
gttcgactccacccgaggtgacca
1-8tRNA-Leu(tag)[17728,17811]
gcccttgtgatgaaatggtagacgtgccgggtttagattccggtgtcgaa
aggcgtgtcggttcgagtccgactgagggcacca
1-9tRNA-Gly(tcc)[18298,18372]
gcgggtataactcagaggcagagtgtcagccttccaagctgttcgtcggg
atttcgaaattccctatccgctcca
1-10tRNA-Arg(cct)[91361,91454]
gaggcgatggaagaggtaatgttcatcaaattcctagtgtggtggtcgat

cacattcatagggatagcagtggttctagtcttcattgactgac
>KU130130.1 Pseudomonas phage vB_PsyM_KIL5, complete genome
1-1tRNA-Ser(tga)[15507,15601]
ggcttggtaagccgagatggcctagcggcagcggtcttgaaaaccgaggg
ttcacggaagtggacgtgtgagttcgagtctcacccaatccgcca
1-2tRNA-Met(cat)[15608,15684]
tggcctttagcataattggttaatgcaatcaactcataattgataagata
cgagttcaagtctcgtggggccaacca
1-3tRNA-Arg(tct)[16255,16331]
atctccatagctcaactggacagagcaagggttttctacacccaaggttg
agggttcgaatccttctggggatacca
1-4tRNA-Thr(tgt)[16666,16742]
gcccgtttagctccaattggtagagcgcctgtcttgtaatcaggatgttt
gcggttcgaatccgtaaatgggcacca
1-5tRNA-Arg(acg)[16842,16916]
agtcgcttggccgattggattaggcaaaggtccacgaagcctgctagagt
ggttcgactccactagttactacca
1-6tRNA-Asn(gtt)[17157,17242]
tggcgtgtaagcataatggtactgcactccgctgttaacggagcttccga
aaggtcttttaggttcgagtcctaccgcgtcagcca
1-7tRNA-Pro(tgg)[17510,17583]
ctcctcgaagcgttacggtagcgtacttcgtttggaacgaagtggcggtg
gttcgactccacccgaggtgacca
1-8tRNA-Leu(tag)[17644,17727]
gcccttgtgatgaaatggtagacgtgccgggtttagattccggtgtcgaa
aggcgtgtcggttcgagtccgactgagggcacca
1-9tRNA-Gly(tcc)[18214,18288]
gcgggtataactcagaggcagagtgtcagccttccaagctgttcgtcggg
atttcgaaattccctatccgctcca
1-10tRNA-Arg(cct)[91930,92023]
gaggcgatggaagaggtaatgttcatcaaattcctagtgtggtggtcgat
cacattcatagggatagcagtggttctagtcttcattgactgac
>KX397370.1 Erwinia phage vB_EamM_Machina, complete genome
1-1tRNA-Trp(cca)[883,959]
gggggagtagttcaattggcgagagcgtcggattccaaatccggttgttg
caggttcgagtcctgtctcccctgcca
1-2tRNA-Cys(gca)[1281,1354]
ggggcgctggccgagtggttaggcgctggattgcaaatcctgttaccgcg
gttcgattccgcggcgtccctcca
1-3tRNA-Tyr(gta)[4114,4199]
ggcgggttactcaagcggtcaacgagagcagactgtaaatctgttgcctt
cgggcttcgtaggttcaaatccttcacacgccacca
1-4tRNA-Asp(gtc)[4501,4576]
ggtgctatcgtctagtggccgaggatactaccctgtcacggtagagaccg

gagttcgattctccgtagcaccgcca
1-5tRNA-Ile(gat)[4585,4660]
tgcgtcttagctcagttggtagagcgcacccctgataagggtgaggccac
tggttcaagtccagtagatgcaacca
1-6tRNA-Glu(ttc)[4668,4744]
attcccgtcatctagttggtccaggatacgacactttcactgtcggtaca
cgagttcaaatctcgtcgggaatacca
1-7tRNA-Ser(gct)[4750,4840]
agagaattgactgagttggtttaaggtgctcccctgctaagggagtgtat
gcgaaagtgtaccattggttcgaatccaatattctctgcca
1-8tRNA-Ile(aat)[5305,5398]
tggctccgtagctaaatcggtagaggcagactttaattagttctccaacg
attttataatcgcgtggtgtaggttcaagtcctaccggagcaag
1-9tRNA-Asn(gtt)[5482,5557]
gactctgtagttcagttggtagaacggtggactgttaatccatatgtcgc
aggttcgagtcctgccagagtcgcca
>KX397371.1 Erwinia phage vB_EamM_Parshik, complete genome
1-1tRNA-Trp(cca)[883,958]
gggggtatagttcaattggcagaatgtcggtctccaaaaccgaaggttcg
aggttcgagacctcgtgcccccgcca
1-2tRNA-Tyr(gta)[4229,4314]
ggcgggttactcaagcggtcaacgagagcagactgtaaatctgttgcctt
cgggcttcgtaggttcaaatccttcacacgccacca
1-3tRNA-Asp(gtc)[4616,4691]
ggtgctatcgtctagtggccgaggatactaccctgtcacggtagagaccg
gagttcgattctccgtagcaccgcca
1-4tRNA-Ile(gat)[4700,4775]
tgcgtcttagctcagttggtagagcgctcccctgataagggagaggtcac
tggttcaagtccagtagatgcaacca
1-5tRNA-His(gtg)[4783,4860]
gtgtatgtagttcagttggttagaattctggcttgtgacgtcagaggtcg
gtgagttcgaatctccccatacacccca
1-6tRNA-Asp(gtc)[4868,4944]
agtgctgtagtcgagcggtttaagacgcctgcctgtcacgtaggagatcg
tgggttcaaatcccatcagcactgcca
1-7tRNA-Glu(ttc)[4951,5027]
attcccgtcatctagttggtccaggatacgacactttcactgtcggtaca
cgagttcgaatctcgtcgggaatacca
1-8tRNA-Ser(gct)[5033,5123]
agagaattgactgagttggtttaaggtgctcccctgctaagggagtgtat
gcgaaagtgtaccattggttcgaatccaatattctctgcca
1-9tRNA-Ile(aat)[5588,5681]
tggctccgtagctaaatcggtagaggcagactttaattagttctccaacg
attttataatcgcgtggtgtaggttcaagtcctaccggagcaag

1−10tRNA−Asn(gtt)[5765,5840]
gactctgtagttcagttggtagaacggtggactgttaatccatatgtcgc
aggttcgagtcctgccagagtcgcca
>KM606999.1 Enterobacteria phage RB10, complete genome
1−1tRNA−Arg(tct)c[67423,67498]
cgaggcatagctcagaaggaagagcaaggaccttctaagtcctgggtcgt
aggttcgatccctactgcctcgacca
1−2tRNA−Asn(gtt)c[67503,67577]
ggatgtgtagctcaatggcagagcgatcgcctgttaagcgattggttata
ggttcgaatcctatcacgtccgcca
1−3tRNA−Tyr(gta)c[67582,67668]
ggggagttatcccgtagaggtagcggtgtggactgtaaatccattgtcat
tgcgactcgggtggttcgactccatcactccccacca
1−4tRNA−Met(cat)c[67678,67752]
ggccctgtagctggaaggttcaagcaagcgactcataatcgccagatggt
ggttcaattccacccagggccacca
1−5tRNA−Thr(tgt)c[67754,67829]
gctgatttagctcagtaggtagagcacctcacttgtaatgaggatgtcgg
cggttcgattccgtcaatcagcgcca
1−6tRNA−Ser(tga)c[67835,67924]
ggaggcgtggcagagtggtttaatgcaccggtcttgaaaaccggcagtcg
ctccggcgactcataggttcaaatcctatcgcctccgcca
1−7tRNA−Pro(tgg)c[67926,68000]
ctccgtgtagctcagtttggtagagcgcctgatttgggatcaggaggtcc
aaggttcaaatccttgtatggagac
1−8tRNA−Gly(tcc)c[68011,68084]
gcggatatcgtataatggcattacctcagacttccaatctgatgatgtga
gttcgattctcattatccgctcca
1−9tRNA−Leu(taa)c[68090,68176]
gcgagaatggtcaaattggtaaaggcacagcacttaaaatgctgcggaat
gatttccttgtgggttcgagtcccacttctcgcacca
1−10tRNA−Gln(ttg)c[68177,68250]
tgggaattagccaagttggtaaggcactggattttgattccaggatgcaa
aggttcgagtcctttattcccagc
>KM606996.1 Enterobacteria phage RB6, complete genome
1−1tRNA−Arg(tct)c[67416,67491]
cgaggcatagctcagaaggaagagcaaggaccttctaagtcctgggtcgt
aggttcgatccctactgcctcgacca
1−2tRNA−Asn(gtt)c[67496,67570]
ggatgtgtagctcaatggcagagcgatcgcctgttaagcgattggttata
ggttcgaatcctatcacgtccgcca
1−3tRNA−Tyr(gta)c[67575,67661]
ggggagttatcccgtagaggtagcggtgtggactgtaaatccattgtcat
tgcgactcgggtggttcgactccatcactccccacca

1−4tRNA−Met(cat)c[67671,67745]
ggccctgtagctggaaggttcaagcaagcgactcataatcgccagatggt
ggttcaattccacccagggccacca
1−5tRNA−Thr(tgt)c[67747,67822]
gctgatttagctcagtaggtagagcacctcacttgtaatgaggatgtcgg
cggttcgattccgtcaatcagcgcca
1−6tRNA−Ser(tga)c[67828,67917]
ggaggcgtggcagagtggtttaatgcaccggtcttgaaaaccggcagtcg
ctccggcgactcataggttcaaatcctatcgcctccgcca
1−7tRNA−Pro(tgg)c[67919,67993]
ctccgtgtagctcagtttggtagagcgcctgatttgggatcaggaggtcc
aaggttcaaatccttgtatggagac
1−8tRNA−Gly(tcc)c[68004,68077]
gcggatatcgtataatggcattacctcagacttccaatctgatgatgtga
gttcgattctcattatccgctcca
1−9tRNA−Leu(taa)c[68083,68169]
gcgagaatggtcaaattggtaaaggcacagcacttaaaatgctgcggaat
gatttccttgtgggttcgagtcccacttctcgcacca
1−10tRNA−Gln(ttg)c[68170,68243]
tgggaattagccaagttggtaaggcactggattttgattccaggatgcaa
aggttcgagtcctttattcccagc
>KM606998.1 Enterobacteria phage RB9, complete genome
1−1tRNA−Arg(tct)c[67417,67492]
cgaggcatagctcagaaggaagagcaaggaccttctaagtcctgggtcgt
aggttcgatccctactgcctcgacca
1−2tRNA−Asn(gtt)c[67497,67571]
ggatgtgtagctcaatggcagagcgatcgcctgttaagcgattggttata
ggttcgaatcctatcacgtccgcca
1−3tRNA−Tyr(gta)c[67576,67662]
ggggagttatcccgtagaggtagcggtgtggactgtaaatccattgtcat
tgcgactcgggtggttcgactccatcactccccacca
1−4tRNA−Met(cat)c[67672,67746]
ggccctgtagctggaaggttcaagcaagcgactcataatcgccagatggt
ggttcaattccacccagggccacca
1−5tRNA−Thr(tgt)c[67748,67823]
gctgatttagctcagtaggtagagcacctcacttgtaatgaggatgtcgg
cggttcgattccgtcaatcagcgcca
1−6tRNA−Ser(tga)c[67829,67918]
ggaggcgtggcagagtggtttaatgcaccggtcttgaaaaccggcagtcg
ctccggcgactcataggttcaaatcctatcgcctccgcca
1−7tRNA−Pro(tgg)c[67920,67994]
ctccgtgtagctcagtttggtagagcgcctgatttgggatcaggaggtcc
aaggttcaaatccttgtatggagac
1−8tRNA−Gly(tcc)c[68005,68078]

gcggatatcgtataatggcattacctcagacttccaatctgatgatgtga
gttcgattctcattatccgctcca
1−9tRNA−Leu(taa)c[68084,68170]
gcgagaatggtcaaattggtaaaggcacagcacttaaaatgctgcggaat
gatttccttgtgggttcgagtcccacttctcgcacca
1−10tRNA−Gln(ttg)c[68171,68244]
tgggaattagccaagttggtaaggcactggattttgattccaggatgcaa
aggttcgagtcctttattcccagc
>KM606997.1 Enterobacteria phage RB7, complete genome
1−1tRNA−Arg(tct)c[67417,67492]
cgaggcatagctcagaaggaagagcaaggaccttctaagtcctgggtcgt
aggttcgatccctactgcctcgacca
1−2tRNA−Asn(gtt)c[67497,67571]
ggatgtgtagctcaatggcagagcgatcgcctgttaagcgattggttata
ggttcgaatcctatcacgtccgcca
1−3tRNA−Tyr(gta)c[67576,67662]
ggggagttatcccgtagaggtagcggtgtggactgtaaatccattgtcat
tgcgactcgggtggttcgactccatcactccccacca
1−4tRNA−Met(cat)c[67672,67746]
ggccctgtagctggaaggttcaagcaagcgactcataatcgccagatggt
ggttcaattccacccagggccacca
1−5tRNA−Thr(tgt)c[67748,67823]
gctgatttagctcagtaggtagagcacctcacttgtaatgaggatgtcgg
cggttcgattccgtcaatcagcgcca
1−6tRNA−Ser(tga)c[67829,67918]
ggaggcgtggcagagtggtttaatgcaccggtcttgaaaaccggcagtcg
ctccggcgactcataggttcaaatcctatcgcctccgcca
1−7tRNA−Pro(tgg)c[67920,67994]
ctccgtgtagctcagtttggtagagcgcctgatttgggatcaggaggtcc
aaggttcaaatccttgtatggagac
1−8tRNA−Gly(tcc)c[68005,68078]
gcggatatcgtataatggcattacctcagacttccaatctgatgatgtga
gttcgattctcattatccgctcca
1−9tRNA−Leu(taa)c[68084,68170]
gcgagaatggtcaaattggtaaaggcacagcacttaaaatgctgcggaat
gatttccttgtgggttcgagtcccacttctcgcacca
1−10tRNA−Gln(ttg)c[68171,68244]
tgggaattagccaagttggtaaggcactggattttgattccaggatgcaa
aggttcgagtcctttattcccagc
>MF988720.1 Pseudoalteromonas phage J2−1, complete genome
1−1tRNA−Ile(gat)c[132398,132472]
cgttttgtagtatagtggttattacggtcggctgataaccgacagatggt
agttcgattctacccgaaacgacca
1−2tRNA−Met(cat)c[132477,132551]

gctcctgtaacataatggctaatgttgacgactcataatcgttaaaacct
tgttcgattcaaggcgggagcacca
1–3tRNA–Cys(gca)c[132553,132627]
gcagggttggcagagttagtaatgtgctggattgcaaatccagttataag
ggcgcgattcccttaccctgctcca
1–4tRNA–Tyr(gta)c[132919,133005]
gggtcgttgggtgaattggttaaaccaccagactgtaaatctggcgctct
ccgagcattcctagttcgagtctagggcggcccacca
1–5tRNA–Ser(gct)c[133007,133097]
ggagaattggctgagtggtttaaggcgctcccctgctaagggagtggacg
ttagtagcgtcccaatagttcgaatctattattctccgcca
1–6tRNA–Gly(tcc)c[133171,133244]
gcgactatggtgtagaggtaacacgctgtccttccaagtcggaatctcgg
gttcgatccccgatggtcgctcca
1–7tRNA–Arg(tct)c[133253,133329]
gcaagtgtagctcagttggatagagcagtagccttctaagctattggtca
gtggttcgagtccactcacttgtgcca
1–8tRNA–Trp(cca)c[133332,133406]
agggatgtggtgtaattggtaacatctcggattccaaccccgaggcagag
agttcgaaactttccgtccctgcca
1–9tRNA–Asp(gtc)c[133994,134069]
agcgacattggtgtatcggttagcatacctgcctgtcacgttggtgctcc
gagttcgatgctcggtatctccgcca
1–10tRNA–Asn(gtt)c[134353,134427]
gacggtgtagctcagaggcagagcatcggactgttaatccgcaggtcggg
atttcgaaattccccaccgtctcca
1–11tRNA–Glu(ttc)c[135052,135126]
gctcgtatcgtctaatggttaagacatctgactttctatcagaggattgg
agttcgattctccatacgagtacca
1–12tRNA–Pro(tgg)c[135129,135205]
ctccgtatagctcagtttggtagagcattccgtttgggacggaagggtcg
tatgttcaagtcgtactacggagacca
1–13tRNA–His(gtg)c[135222,135294]
ggtgacattagtttagtggtagaatttcgagttgtgacctcgaagggcag
ggttcaattcccgatgtcacccc
1–14tRNA–Lys(ttt)c[135318,135393]
gggtcgttagctcagttggtagagcagggagcttttaactctcaggtcgt
tggttcaaatccaacacggcccacca
1–15tRNA–Ala(tgc)c[135395,135470]
ggggatatagctcaattggtagagcactgcctttgcaaggcaggggttta
gggttcaaatccctatatctccacca
1–16tRNA–Thr(tgt)c[135479,135551]
gctacaatagctcagttggtagagcatctgttttgtaatcagagggtcgg
aggttcgaaaccttcttgtagca

>MF403008.1 Agrobacterium phage Atu_ph07, complete genome

6–1tRNA–Leu(caa)c[169426,169509]
gcgccgctggtgaaattggttagacacagaatcctcaaaaggttccgcga
aagcatcccggttcgagtccggggcggcgtacca
6–2tRNA–Met(cat)c[169521,169596]
aggcgggtagccaagtggttaaggccacctgctcataacaggtttatcgt
cggttcgatcccgaccccgcctacca
6–3tRNA–Ser(gct)[230506,230598]
ggaacgttggccgagtcaggtttaaggcagcacattgctaacgtgtcgtg
cgttaatagcgtaccagaagttcgaatcttctacgttccgcca
6–4tRNA–Val(aac)c[288496,288570]
agtcaaatagctcaacccggtaagagcggcctataaccaagggaatgatg
atggttcaaatccatctttgactac
6–5tRNA–Gly(gcc)c[303235,303309]
gcgggtgtagctcagtggtagagcgtcacgttgccaacgtgaatgtcgag
ggttcaaatcccttcgcccgctcca
6–6tRNA–Met(cat)c[303646,303723]
cgcggagtggagaagtcggtttaactcatcagcctcataagctgaggatc
gcaggttcaaatcctgcctccgcaacca
6–7tRNA–Asp(gtc)c[317242,317319]
gcggatgtagctcagttggtcagtagcgtcggcttgtcacgccggaggtc
gcgggttcgagtcccgtcattcgcgcca
6–8tRNA–Arg(tct)c[317484,317561]
ggttccttagttcagttggaatagaacgactgccttctaagcagtaggtc
gtaggttcgagtcctacaggaatcgcca
6–9tRNA–His(gtg)c[323350,323424]
gcttggatcgtacaatggttagtacgctggtttgtggcaccggagatcgt
ggttcaattccccgtccaagtacca
6–10tRNA–Ser(tga)c[323788,323879]
ggatgggtgtccgagtccggtttatggaactagtcttgaaaactagcgtg
cgtgagagcgtaccgtgggttcgaatcccaccccatcctcca
6–11tRNA–Leu(tag)c[332329,332418]
gcgggtgtggcggaattggtctacgcacgagttttaggtactcgcgcctc
tttagcaggcttgggggttcaagtccctccacccgtacca
6–12tRNA–Gly(tcc)c[332458,332530]
gcgggtatagcttaatggtagagcgctgtccttccaagtcagaggccgtg
ggatcggtccccactacccgctc
6–13tRNA–Gln(ttg)c[332539,332613]
tcagggttagttcagtggtagaacgcgaatttttggtattcgatgtcgga
tgttcgaatcatccaccctgatcca
6–14tRNA–Pro(tgg)c[334331,334407]
cggagtgtagcgcagtctggtagcgcatctggtttgggaccagagggtcg
ggagttcgaatctctccactccgacca
6–15tRNA–Phe(gaa)c[335908,335983]

gcacctgtagctcagttggtagagcatcgcactgaagatgcgagtgtcac
tggttcgattccagtcaggtgcacca
6-16tRNA-Gln(ctg)c[335985,336058]
ttgggattagtttaacggtagaacaacggactctgactccgttggtagtg
gttcgaatccattatcccaagcca
6-17tRNA-Pro(tgg)c[336074,336151]
cagggtataaggaagtctggtctatccggtcacacttggaatgtggcaac
gctggttcaaatccagctaccctgacca
6-18tRNA-Val(tac)c[336155,336229]
gcgcgattagctcagcgggagagcgcctcgtttacaccgaggatgtcggg
agttcaatcctctcatcgcgcacca
6-19tRNA-Glu(ttc)c[337734,337810]
acgtctgtggccaagttggttaaggcacctcactttcaatgaggaaatcg
ccggttcgagtccggtcaggcgtacca
6-20tRNA-Met(cat)c[337849,337921]
atcgggatagctcagcggtagagcggcactttcatacggtgttggtcgta
ggttcaaatcctactctcgatac
6-21tRNA-Trp(cca)c[337925,338000]
acacgtatagctcagttggtagagcggcggtctccaaaaccgcaggtcgg
gggttcgaagccctctgcgtgtgcca
6-22tRNA-Tyr(gta)c[338064,338146]
tggtggttgccagagcggtcgattgggacggactgtaaatccgtagtgtt
aagccacgttggttcgaatccaacaccaccaac
6-23tRNA-Ile(gat)c[338161,338240]
gcgcgggtagctcagtttttggtcagagcacaccactgataatggtgagg
tcgatggttcaagtccatctccgcgcacca
6-24tRNA-Pro(tgg)c[338316,338393]
cagtgcgtgggaaagtctggttaatccgctacacttgggctgtagagaac
gctgattcgaattcagccgcactgacca
6-25tRNA-Phe(gaa)c[338698,338773]
gcaatcgtaactcagttggtagagtgtcacactgaagatgtgaaggtcat
ccgttcgaaccggatcgattgcacca
6-26tRNA-Arg(acg)c[338790,338867]
gtacccgtagctcaactggactagagcgctggtctacggaaccagaggtt
gtggattcgacttctaccgggtacgcca
6-27tRNA-Ala(tgc)c[338871,338943]
ggggaatgctctgggtaagcggacaatccttgcaagattgtagcccgacc
ggttcgattccggtattctccac
6-28tRNA-Lys(ctt)[390848,390923]
gggtgcttagctcagttggtagagcaatcggctcttaaccggttggtcac
aggttcgaaccctgtagcacctacca
6-29tRNA-SeC(tca)[430713,430787]
gggagtgtggctgactggtaaagcagatgccttcaaaacattgagatgcg
ggttcgattcctgtcactcccacca

6–30tRNA–Cys(gca)c[446807,446882]
ggcgcggtgcaccgaatggttaggtagctccctgcaaaggagatttatgt
cagttcgaccctgacccgcgcctcca
>KU682439.2 Stenotrophomonas phage vB_SmaS–DLP_6, complete genome
7–1tRNA–Arg(cct)[92962,93038]
gcccttgtagctcaactggacagagcagcgatttcctaggtcgccggttg
taggctcgaatcctaccaagggcgcca
7–2tRNA–Pro(tgg)[103314,103390]
cggagtgtagcacagcttggtagtgcgcgtgctttgggagcatgaggtcg
caggttcgattcctgtcactccgacca
7–3tRNA–Asp(gtc)[103636,103709]
gggactgtaggcttctgggaagctagtggactgtctatccactcaggcgg
gttcgatccccgtcagtctcgcca
7–4tRNA–Leu(tag)[103950,104037]
gcgtcactaggggaattggcaaacccactggatttaggttccagcgtccg
aaagggcagtgagggttcgagtccctcgtgacgtacca
7–5tRNA–Met(cat)[104140,104215]
cgtgctgtacgattcgcggtgaatcgactggtctcataagccaagaaggt
gagttcgactctcaccagcacgacca
7–6tRNA–Gly(gcc)[104297,104371]
gcgggtgtagctcagtggtagagttcttccttgccaaggaagatgtcgag
agttcgaatctcttcacccgctcca
7–7tRNA–Met(cat)[104407,104483]
tgcacggtacaatcaacggaagatggacagtctcataagctgctcggttg
cgggttcgactcccgcctgtgctacca
7–8tRNA–Tyr(gta)[104488,104579]
gggtgtattacggctagcggtctaagccaccagactgtaaatctggcgcc
tcgcaagggcaatcggtggttcgaatccatctacacccacca
7–9tRNA–Leu(caa)[104656,104732]
gctcccgtgccgcaactggaagacgggcttgattcaaaaccaagatgttg
tcggttcgatcccgaccgggagtacca
7–10tRNA–Ile(tat)[104738,104832]
gcttctgtagtgtaattggaagcacgagcggatttatacaccgcataccg
ccagattagcggttagcctcggttcgattccgagcagaagcacca
7–11tRNA–Phe(gaa)[104845,104920]
ggggcagtagctgatgaggtcttagcactggcttgaaaacccagagatgt
cggatcgttaccgacctgccccacca
7–12tRNA–Arg(ccg)[106026,106102]
gcccttgtagctcatctggaaagagcctccggttccgatccggaaggtag
caggttcgactcctgtcaagggcacca
7–13tRNA–Thr(cgt)[106481,106554]
gctccattagtctaatggtatgacagctgtttcgtaatcagctggcgggg
gttcgattccctcatggagcacca
7–14tRNA–Asn(gtt)[107530,107615]

ggtacgcgtgcccagtggggaagcaataggctgttaacctatcggacgaa
agtcccggtctggttcgattccagagcgtatcgcca
7-15tRNA-Glu(ctc)[108661,108737]
gcacctatcttctaacaggttcaggaagctagactctcaatctagtaatc
ggggttcgagtccccgtaggtgtacca
7-16tRNA-Thr(tgt)[108827,108901]
gctcctaaagcattgatggcgatgcagttgccttgtaagcatcagaaccc
agttcgattccgggtgggagcacca
7-17tRNA-Ile(gat)[109099,109172]
tgcttcgtagctcaaaggtagagcaaccggctgataaccggtcgacactg
gatcgttcccagtcgaagcaacca
7-18tRNA-Asn(gtt)[109647,109721]
ggatctcgagccagctggtgtgggcagtgagctgttaactcatgaagcag
ggatcgtaaccctgggggtccgcca
7-19tRNA-Lys(ctt)[109728,109802]
gggaagttaactcaactggtagagtcctgcctcttaagcagcgagacgcg
agttcgatcctcgcacttcccacca
7-20tRNA-Glu(ttc)[109939,110012]
acaccgttgcttctggtgaggcagtcagcctttcaagctgacgcagaggg
gatcgtaacccctacggtgtgcca
7-21tRNA-Ala(tgc)[110017,110092]
gggggattagctcagctgggagagcagttgctttgcaagcatcaggtcat
cggttcgatcccgatatcctccacca
7-22tRNA-Cys(gca)[110184,110258]
gtcctgttagcagagtggctcatgcggttggctgcaacccttccgacgtc
ggttcgatcccgacacaggactcca
7-23tRNA-Gly(tcc)[110267,110341]
gcaggtatggtatattgattgttgccctagtcttccaaactagagaagcg
ggtttgattcccgctacctgctcca
7-24tRNA-Gln(ctg)[110348,110421]
agccctgtagttcaatggtagaatatcggtctctgaaaccgaagacgaag
gatcgataccttctggggctgcca
7-25tRNA-Trp(cca)[110432,110507]
gcgtcagtagctcaattggcagagttccgaactccaaatccggcggttgt
aggttcgattcctacctggcgcgcca
7-26tRNA-Val(tac)[110516,110590]
gcctgtctagttctctgggagaacaccctctttacacgtgggcttaggtg
agttcgattctcacggcaggtacca
7-27tRNA-Thr(ggt)[110832,110906]
gcttgtatagcacagtggtagtgcacctctttggtaaagaggaggtcgcg
ggttcgattccctctacaagcacca
7-28tRNA-Val(gac)[111078,111152]
agttccctagctcagtggtagagcgcctccttgacatggagtaggtcagc
ggttcgatcccgctgggaactacca

7-29tRNA-Asp(gtc)[111260,111337]
cgggcatgagactgcttggtgtggttgctcccctgtcacgggagatatag
acgggttcgatgcccgtatgcccgacca
7-30tRNA-Arg(tct)[111579,111655]
gcgccattagctcaactggatagagcaacttctttctaggctgtgggttg
agggttcgaatccttcatggcgcacca
7-31tRNA-Leu(gag)[112066,112142]
gcttccgtggcgcaattggaagacgcactaccttgaggtggtaggtgttg
agggttcgagtcccttcggaagtacca
7-32tRNA-Phe(gaa)[112149,112224]
gcttcggtagctcagttggtagagcagtggactgaaaatcctcgtgtcgg
cggttcgattccgccctgaagcacca
7-33tRNA-His(gtg)[112574,112648]
gtggtcttagtgtagtggtctgcacgggtgtctgtgaaacaccaagtcga
tgttcgatccatcgattccacccca
7-34tRNA-Lys(ctt)[112656,112731]
gggtccgtagctcaatggttagagcattcgcctcttaagcgacaggttgt
gagttcgagtctcactggacccacca
7-35tRNA-Arg(acg)[112740,112816]
gcgccgttagctcagctggatagagtacctggctacgaactaggcggtcg
ggggttcgactccctcacggcgcacca
7-36tRNA-Pro(cgg)[113092,113169]
cggagtgtagctcagtctggtagcagcgctactttcgggaagtagaggtc
gcaggttcgattcctgtcactccgacca
7-37tRNA-Leu(cag)[113330,113406]
gcccatgtgacgcaattggtagacgtactatcctcagaaggtaggtgttg
gcggttcgagtccgcctatgggcacca
7-38tRNA-Pyl(cta)[113763,113837]
gcgcgattagctctctgggagagcagccgcctctaaaacggtatgagatg
ggttcgatccccatatcgcgcgcca
>KR560069.1 Stenotrophomonas phage IME-SM1, complete genome
8-2tRNA-Arg(tct)[1537,1613]
gccccggtagctcaaccggatagagcacccgccttctaagcgggtggtta
ggggttcgattcccttccggggtgcca
8-3tRNA-Ile(gat)[1615,1689]
agcgcgttagctcagcggtagagcactcgaccgataatcgagtggtcgta
ggttcaaatcctacacgcgctacca
8-4tRNA-His(gtg)[1709,1785]
gtgtacgttgttcgtattggttcggacgcctgattgtgagtcaggagcat
gtgggttcaattcccatcgtacacccc
8-5tRNA-Trp(cca)[2143,2218]
acgtcggtagctcaattggcagagcggtggcctccaaagccactggttga
gggttcaagtccttctcggcgtgcca
8-6tRNA-Cys(gca)[2227,2301]

ggctggttggtcagagaggttatgcggatggttgcaacccatcatatggc
ggttcgagtccgtcaccagcctcca
8-7tRNA-Lys(ctt)[2716,2792]
aggttgctagctcaactggttagagcaatcgcctcttaagcgataggttc
agggttcaagtccctggcgacctacca
8-8tRNA-Asn(gtt)[3162,3238]
tctggattagctcagttggttagagcacgcgcctgttaagcgcggggtca
ctggttcaagtccagtatccagagcca
8-9tRNA-Ala(tgc)[3247,3322]
gggctgttagctcagatgggagagcgcctgccttgcacgcaggaggtcgt
cggttcgatcccgacacggtccacca
8-10tRNA-Val(tac)[3419,3494]
gggttgttagctcagttggtagagcgcctctcttacacggaggatgtcca
gggttcgagaccttgacaacccacca
8-11tRNA-Met(cat)[3503,3577]
tgcgcgatagtgaaattggcatcacgtgggtctcataagcccaaatttcg
ggttcgagccccggttgcgcaacca
8-12tRNA-Glu(ttc)[3585,3659]
gcaccgttcctctaaaggttaggagatcgggtcttcaccccgacaatagg
agttcgagtctcctacggtgtacca
8-13tRNA-Leu(caa)[3671,3754]
gcccgagtggtggaactggcagacacactgcactcaaaatgcagcgcctc
aggcgtgacggttcaaatccgtccttgggtacca
8-14tRNA-Ser(tga)[3762,3845]
gggttgtggcgtgcagggcacaaactggtcttgaaaaccaggccatccga
aaggttgagggttcgattccttcacgacccgcca
8-15tRNA-Tyr(gta)[4133,4219]
gtagtgccggtgaagttggagagtcacaccaggctgtaaacctggcgcct
tatggctgagtaggttcgattcctacgcgctgcacca
8-16tRNA-Phe(gaa)[4224,4298]
gcgtcgatagctgagatggattagcgaggggctgaaaatccctagaggtt
ggttcgataccaattcgatgcacca
8-17tRNA-Asp(gtc)[4309,4385]
cgggttgtagttcagttggttagaatgcttgcctgtcacgcaagaggtca
cgggttcgatccccgtcagcccgacca
8-18tRNA-Gln(ttg)[4430,4505]
agcgggttagctcagatggaagagcagtgggctttgatcccattggtcgg
gggttcgactccctcacccgccgcca
8-19tRNA-Ser(gct)[4517,4606]
gggtatttgcgtgagtctggtttaaacgagctccgtgctaaggagtcagg
tcgaaaggcctcgtaggttcaaatcctacaatacccgcca
8-20tRNA-Gly(tcc)[5159,5232]
gcgagagtagtttaacggtagaatgtctgccttccaagcagatggttcgg
gttcgattcccgggtctcgctcca

8−21tRNA−Pro(tgg)[149006,149082]
cggggtatagcgcagtctggtagcgcgcctgctttgggagcaggatgtcg
ggggttcgagtccctctaccccgacca
8−22tRNA−Leu(tag)[149171,149253]
gggcgtgtggtggaattggcagacacactggatttaggttccagcgcttc
ggcgtgagggttcgagtccctcctagcccacca
8−23tRNA−Thr(tgt)[149582,149657]
gccggtttagctcagctggtagagcaactgccttgtaagcagtaggtcgt
cggttcgatcccgacagccggcacca
>KC292026.1 Halovirus HGTV−1, complete genome
9−1tRNA−Ser(gga)c[42266,42352]
gccgtgacaaggggatgggcgaccccgcgatgctggaaccatcgtgtcct
gagatatggcttgcaggttcgactcctgctctcggcg
9−2tRNA−Val(gac)c[42609,42683]
gggtgtgtggtccaactcggttaagataccgcattgacattgcggtgatt
tgaggttcaaatcctctcgcaccca
9−3tRNA−Val(tac)c[42688,42762]
acgggtataattcaatctggtcagaatactccgcttacatcggagatgtt
gaaggttcaaatccttctgcccgta
9−4tRNA−Pro(tgg)c[42770,42840]
tggctggtggtccaatggtacgattcggcgtttgggacgccgagatatgg
gttcgactcccttccagccaa
9−5tRNA−Leu(taa)c[45441,45526]
aggtcggtgcgccgagtctggtcaaaggcagggcacttaagatgccccga
ttagttcttcacaggttcaaatcctgtccggcctac
9−6tRNA−Leu(caa)c[45530,45613]
gagtggatagccaagtggtcaaaggcaggagagtcaaatcctcccggtat
agtccttcgcaggttcgaatcctgctccgctcac
9−7tRNA−Leu(gag)c[45617,45700]
gcatctgtagggaactggtgaaacccataggcttgaggggccttgcccgt
aggggctttcttggttcgattccaagcgggtgca
9−8tRNA−Leu(tag)c[45786,45869]
gagtcggtacgagaaatggttaaactcacgaggcttaggacctcgcgctt
tagtgctttgcaggttcgaatcctgcccggctca
9−9tRNA−Arg(tct)c[46355,46430]
taggcgtataaggtagtggactatcccgcgagtcttctaaacttgcgaca
ggggttcgaatccccttacgcctgtc
9−10tRNA−Arg(tcg)c[46700,46775]
tgcccacgtagcctaattggataaggcaatggccttcgaagccagagatt
gcaggttcgagtcctgtcgtgggcgt
9−11tRNA−Ser(tga)c[47447,47532]
gccgagattgagtcaggcaaactcagtctgttttgaaaacagacggtcgc
atatgcggccaagaggttcaaatcctctgctcggcg
9−12tRNA−Cys(gca)c[47537,47609]

gtcgggatgccagagtggtcacggcccggacagcagattcggtgtacggg
ggttcgaatccctctcccgactc
9-13tRNA-Ile(gat)c[48011,48085]
gcgttcgtagccaaatcaggtaaggcgttccgctgataacggaaagattt
gttggttcaaatccaaccggacgca
9-14tRNA-Gln(ctg)c[48088,48162]
tagtcggtttgtgtagcggccaatcatacgaggctctgaccctcgtgaca
gaggttcgaatcctctaccgactat
9-15tRNA-Ser(gct)c[48164,48246]
gccgtggttgagtcaggcaaactcggttggattgctaatccaacggcctt
tatggtcagaaggttcgaatccttccctcggcg
9-16tRNA-Ile(gat)c[48250,48320]
acgaggatagtgtaatggaaacactccggtcagatggaccggcactacct
gttcgattcaggttcctcgta
9-17tRNA-Ser(cga)c[48383,48468]
gccggtggttgagcaatctggtcgctcacctgtctcgaaaacaggctccc
attgtggaatgcaggttcgaatcctgccctcggcgc
9-18tRNA-Trp(cca)c[48474,48544]
gaggtcttggggtaatagaatcctactcgcctccagagcgagtgatggga
gtgcaaatctctcagacctca
9-19tRNA-Gln(ttg)c[48548,48620]
agtccccgagtccgctggcgaaagacgctcggctttggaccgagagagcg
aggttcgattcctcgggggacta
9-20tRNA-Lys(ttt)c[48804,48876]
gggccggtggcgaaatggcttaacgcaggaggcttttaacctcccatcgc
gggttcgattcctgctcggccca
9-21tRNA-Thr(ggt)c[48880,48952]
atccagtatggtctaatggtagggcacggctttggtatggctgacgtgag
ggttcaactccctctgctggatt
9-22tRNA-Thr(cgt)c[48954,49029]
atccgttgtggcccaatttggacgggcactcgcctcgtaagcgagaggtt
gtgggttcgattcccaccagcggatt
9-23tRNA-Thr(tgt)c[49031,49103]
tccggtcaggccgcataggagtggcggttcccttgtaaggaacagacggt
gggttcgactcccacgaccggat
9-24tRNA-Met(cat)c[50107,50194]
tcccgacgttgagcagtctggctatgctcggcaggctcatatcctgttgt
gcttcgcacgaaatgggttcgaatcccatcgtcggaat
9-25tRNA-Lys(ctt)c[50195,50271]
gggctgatggcgaaacttggctaaacgcggaaggctcttgaccttctaca
tacgggttcaaatcctgttcagcccac
9-26tRNA-Tyr(gta)c[50274,50347]
ctctcgaagacccgctggtaagggtacacgcctgtagagcgtgttgcagt
aggttcgaatcctactcgggagac

9-27tRNA-Asn(gtt)c[50350,50424]
tgtcgcggtagttcaattagtagaacactcggctgttacccgagatgttg
gaggtgcaagtcctccctgcgacgt
9-28tRNA-Gly(gcc)c[50474,50544]
gcgtggttggtccaatggaaagacggctccctgccacggagcagactcgg
gttcgattcccgaatcgcgca
9-29tRNA-Gly(tcc)c[50549,50619]
gcgtggctgttccaatggcaagatgcgggccttccaagcctgagatacga
gttcgactctcgtgccgcgca
9-30tRNA-His(gtg)c[50624,50697]
tccggcgctggtggaaaagttaacattcggccctgtgacggtcgagatac
gagtgcgaatctcgtgcccggacc
9-31tRNA-Glu(ctc)c[51556,51632]
gctgatggagtctccgctggcgagagacgctacgttctcagcgtagaggt
cgcgggttcaattcctgtcatcagcac
9-32tRNA-Phe(gaa)c[51636,51710]
gggtcgataggataatttgggaatccacctgtttgaagaacaggcgcatc
tcggttcaaatccgggtcggcccac
9-33tRNA-Ala(agc)c[52081,52168]
gctggcttggggtagtggacgcacggcattaagccaatccttcgaccctt
tcgaggtcgagacacgagttcgaatctcgtagccagta
9-34tRNA-Asp(gtc)c[52304,52376]
tgccgaagtggtctaaaagtaagatgcgtgcctgtcacgcacgagactcg
ggagcattacccggcttcggcgt
9-35tRNA-Arg(gcg)c[53348,53420]
gggcgattggtctactggatatgacacatccctgcgaaggatgagaaggg
agttcgattctctcattgcccac
9-36tRNA-Gly(tcc)c[121104,121188]
acgccgccttccacgaggatagccgttcgtttccgtcgaacgtggctact
gtgtagggcatactcgttcggtcgagtttgacgtg
*Nudiviridae
>KM610234.1 Tipula oleracea nudivirus isolate 35, complete genome
1-1tRNA-Val(tac)[39778,39850]
gcgggtatggtctagtggttatggcgtctgttttacacgcagaaggtccc
gggttcaatccccggtgcccgta
*Phycodnaviridae
>JF411744.1 Paramecium bursaria Chlorella virus 1 (PBCV-1), complete genome
1-1tRNA-Leu(caa)[164946,165026]
gatagggtatgcaagtggtcaaagcagctggtctcaagatccagtccttc
gggttcgcgggttcgactcccgtctctatca
1-2tRNA-Ile(tat)[165052,165124]
gttcgcttagctcagtggttagagcattggtcttatgagccaagggtcac
cggttcgatcccggtagcgaaca
1-3tRNA-Asn(gtt)[165254,165327]

gacgccatagctcagttggttagagcgttcgactgttaatcgagaggtca
ccggttcgaacccggttggtgtcg
1-4tRNA-Lys(ctt)[165330,165403]
tgcccgcctagctcagtcggtagagcgccagactcttaatctggtggtcg
tgggttcgagccccacggtggcat
1-5tRNA-Asn(gtt)[165427,165500]
gacgccatagctcagttggttagagcgttcgactgttaatcgagaggtca
ccggttcgaccccggttggtgtcg
1-6tRNA-Lys(ctt)[165503,165577]
tgcccgtctagctcagtcggtagagcgccagactcttaatctggtggtcg
tgggttcgagccccacgatgggcat
1-7tRNA-Lys(ttt)[165709,165783]
tgcccgtctagctcagtcggtagagcgccagacttttaatctggtggtcg
tgggttcgagccccacgatgggcat
1-8tRNA-Arg(tct)[165805,165881]
tggtcgcgtagctcagtcggataagagcaacagacttctaatctgtaggt
cgtgggttcgagccccaccgtgatcga
1-9tRNA-Val(aac)[165914,165986]
gatcccttagctcagttggaagagtttttgcctaacacgcagaaggtcgc
aggatcgaaacctgcagggatca
>AJ890364.1 Emiliania huxleyi virus 86 isolate EhV86
1-1tRNA-Ile(tat)c[39461,39534]
gtcccattaactcagttggtagagtgtggttcctatgaagctatggtcgg
cgtttcgagcacgccatgggacac
1-2tRNA-Gln(ttg)c[43293,43364]
cctcctatagtctagcggtaaggacaatggttcttgataccatgagcatg
tgttcgaatcacattaggaggt
1-3tRNA-Asn(gtt)c[43367,43441]
tcccatgtagctcaattggtcagagcgtgcggctgttaaccgcaaggttg
tgtgttcgattcacaccgtgggagc
1-4tRNA-Arg(tct)c[203554,203628]
agccctcgtagctcagtggatagagctcttgccttctaagcaagtggtcg
cgagttcgatcctcgccgtgggttt
>DQ890022.1 Paramecium bursaria Chlorella virus FR483, complete genome
1-1tRNA-Ile(tat)[141848,141920]
gttcgcgtagctcaatggtcagagcactgatcttatgagtcaggggttgc
cggttcgagtccggtcgtgaaca
1-2tRNA-Leu(taa)[141945,142029]
ggcacgattcccaagtctggtcaacggggctggtcttaagaaccagtgca
atatgcttcgtcggttcaaatccggctcgtgctac
1-3tRNA-Arg(tct)[142053,142128]
gtctctgtggcgcaatggacagcgcgttggacttctaatccaaaggttgc
gggttcgacccccgccagagacgcca
1-4tRNA-Gly(tcc)[142129,142199]

ggatgtgtgatgcaacggtagcataacagccttccaagctgtaaacctgg
gttcgactcccagcacattca
1−5tRNA−Asn(gtt)[142222,142297]
tggcgtcctagctcagtcggttagagcgacaggctgttaacctgtaggtc
atcggttcgattccgatggatgccga
1−6tRNA−Asn(gtt)[142319,142394]
tggcgtcctagctcagtcggttagagcaacaggctgttaacctgtaggtc
atcggttcgattccgatggatgccga
1−7tRNA−Tyr(gta)[142416,142501]
ctcgccatagctcaattggaagagcgaaggactgtaattgtttatttgtc
atccttaggtacctggatcgaaaccgggtggtgaga
1−8tRNA−Lys(ctt)[142504,142576]
gcccggttagctcagtcggtagagcgctagactcttaatctagtggtcgt
gggttcgagccccacatcgggca
1−9tRNA−Thr(cgt)[142738,142810]
gccggtgtagcttagtggtaaagcgcttgcttcgtaagcaagagaccatg
agttcaatcctcatcaccggcac
>EF101928.1 Acanthocystis turfacea Chlorella virus 1, complete genome
1−1tRNA−Ser(act)[83726,83794]
gctccttagctcagaggagagcgctggtctactagaccaaagggggcggt
tcaattccgtcatggagaa
1−2tRNA−Arg(tct)[83799,83873]
gggagcgtagctcagttggatagagcgacagacttctaatctgtaggccg
tgggttcgagccccaccgttctcgc
1−3tRNA−Gly(tcc)[83898,83968]
ggactcgtgatgtagtggtaacatacttgccttccaagcaagatacctgg
gttcgattcccagcgtgttca
1−4tRNA−Asp(gtc)[83991,84062]
gtcgtggtagtatagtggttagtattcctgcctgtcacgcaggagaccgg
ggttcaattccccgccacggcg
1−5tRNA−Val(aac)[84086,84158]
gacccgatagctcagttggtagagcatctgtctaacacgcagaaggtctg
gggatcgagaccccattgggtta
1−6tRNA−Asn(gtt)[84276,84349]
ggcgctgtagctcagtcggttagagcacttggctgttaaccaaggggtca
tcggttcgattccgatcagtgccg
1−7tRNA−Lys(ctt)[84456,84528]
gcccggttagctcagtcggcagagcgcaaggctcttaaccttgtggtcgt
gggttcgagccccacatcgggca
1−8tRNA−Asn(gtt)[84551,84624]
ggcgctgtagctcagtcggttagagcacttggctgttaaccaaggggtca
tcggttcgattccgatcagtgccg
1−9tRNA−Leu(taa)[84773,84858]
ggcagcatcggttagtcaggtccagaccgggggtcttaagaacctctcct

cttatgagggcgtgggttcgaatcccactgttgcca
1-10tRNA-Thr(agt)[115793,115867]
gctcccatagctcagttggttagagcgcgactttagtaaggtcgaggtcc
gtggttcgaatccgcgtgggagcac
>DQ491003.2 Paramecium bursaria Chlorella virus AR158 genomic sequence
1-1tRNA-Leu(taa)[172099,172184]
ggtaccatggccgagttggtttaaggcgatccgcttaagacggattggac
acacgtccacggaggttcgaatccttctggtactac
1-2tRNA-Ile(tat)[172209,172281]
gttcgcttagctcagtggatagagcattggtcttatgagccaagggtcac
cggttcgatcccggtagcgaaca
1-3tRNA-Asn(gtt)[172307,172378]
gacgatgtggtgaaaaggttatcacagatggctgttaaccatccgttcag
agttcgattctctgcttcgtcg
1-4tRNA-Leu(caa)[172430,172514]
ggcagaatcggatagcctggtccattccgtcccgctcaagacgggaaccc
ttcgggggcgtgcgttcaaatcgcacttctgctac
1-5tRNA-Arg(tct)[172537,172611]
ggccacgtgatgaaatggatatcatattcgacttctaatcgaaatttccg
agttcgatcctcggcgtggctgcca
1-6tRNA-Gly(tcc)[172612,172684]
ggcacattgatgaagttggttatcatacttgccttccaagcaagattccc
ggattcgaattccggatgtgtca
1-7tRNA-Val(aac)[172708,172781]
gatcccttagctcagttggaagagtttttgcctaacacgcagaaggtcgc
aggatcgaaacctgcagggatcac
>EU304328.2 Ostreococcus tauri virus OtV5, complete genome
1-1tRNA-Gln(ttg)[145641,145713]
agcttctatggtgtagtggtaacacagtggactttgaatccaccgccaca
ggttcaatccctgttggaagctt
1-2tRNA-Asn(gtt)[145817,145890]
tcactcatcgtctagtggtttaggacaatcggctgttaaccggttaacca
gggttcgaatccctgtgagtgaac
1-3tRNA-Thr(agt)[145893,145966]
tgcatctatggccaagtggtaaggcgtctctttagtaaggagaagatcgt
gcgttcgaaccgcactagatgcat
>DQ491002.1 Paramecium bursaria Chlorella virus NY2A Chlorella virus NY2A ctg_13 genomic sequence
1-1tRNA-Leu(taa)[194698,194778]
gtgtcgttgggggagtagtcaaacccaggagacttaagttctcccgcctc
ggcttcgtcggtgcaaatccggcacgacaca
1-2tRNA-Leu(caa)[194921,195006]
gccaggatggccgagttggttaaggcgatcgcctcaagagcgattggatg
ttatgtccgcgtaggttcgagccctacttctggcac

1–3tRNA–Arg(tct)[195029,195103]
ggccacgtgatgaaatggatatcatattcgacttctaatcgaaatttccg
agttcgatcctcggcgtggctgcca
1–4tRNA–Gly(tcc)[195104,195176]
ggcacattgatgaagttggttatcatacttgccttccaagcaagattccc
ggattcgaattccggatgtgtca
1–5tRNA–Asn(gtt)[195201,195272]
gaccctatggtgaaacggttatcacaggtggctgttaaccacccgttcgc
agttcgattctgcgtggggtcg
1–6tRNA–Lys(ctt)[195382,195457]
gcccgcttagctcagcatggtcagagcgctagactcttaatctagtggtc
gtgcgttcaaatcgcacagcgggtac
1–7tRNA–Val(aac)[195482,195555]
gatcccttagctcagttggaagagtttttgcctaacacgcagaaggtcgc
aggatcgaaacctgcagggatcac
>FN600414.1 Ostreococcus tauri virus 2, complete genome
1–1tRNA–Gln(ttg)[136163,136236]
agctcttatagtgtagtggtcatcactttggactttgaatccaacaaccc
tggttcaaatccaggtaggagctt
1–2tRNA–Asn(gtt)[136348,136422]
tttccttgtaactcaatcggaagagtgtacgactgttaatcgtaaagtag
cgagatcgaaactcgccaaggaagt
1–3tRNA–Thr(agt)[136426,136499]
tgcatctatggccaagtggtaaggcgtctctttagtaaggagaagatcgt
gcgttcgaaccgcactagatgcat
>HM004431.1 Ostreococcus lucimarinus virus OlV1, complete genome
1–1tRNA–Gln(ttg)[145408,145494]
agcttttatggtgtagtggtaacactgcggactttgactttaacgaagac
gatccgccaccctaggttcgaatcctagtagaagctt
1–2tRNA–Leu(tag)[145498,145585]
ccagtgttagctcagttggaagagcagtggattgtagtagtatgatatag
atctccacgggtcgggtgttcgaatcatccacactgga
1–3tRNA–Asn(gtt)[145589,145664]
ttcccttgtaactcagttggttagagtgttcgactgttaatcgagaagcc
accggttcgaatccggtcaagggaga
1–4tRNA–Thr(agt)[145669,145741]
gcacctgtagcatagtggttaatgcgcctctttagtaaggaggagaccgc
gtgttcgaatcacgccaggtgca
>HM004432.1 Bathycoccus sp. RCC1105 virus BpV1, complete genome
1–1tRNA–Leu(aag)[131783,131864]
gatgccgtggccgagtggtctaaggcgccagattaaggctctggttcgga
agagcgcaggttcaaatcctgtcggcatcacc
1–2tRNA–Asn(gtt)[131980,132055]
ttctctcgtaactcagttggttagagtgtgcgactgttaatcgcgaagtc

accggttcaactccggtcgagagagt
>HM004429.1 Micromonas sp. RCC1109 virus MpV1, complete genome
1-1tRNA-Ile(tat)[136710,136783]
gttcttatagctcagttggttagagcgtggtgcttataacgccaaggtca
cgggttcgagccccgttgggaaca
1-2tRNA-Gln(ttg)[138249,138322]
ggctctcatagtgtagtggtcaacactgtggactttgaatccaccaccct
aggttcgactcctagtgggagctt
1-3tRNA-Tyr(gta)[138325,138398]
cctctcttagctcagttgggagagcagtggactgtagttccaagggtcag
gtgttcgattcacctagagaggac
1-4tRNA-Asn(gtt)[138403,138476]
tcttccatagctcagttggtagagcgtgcgactgttaatcgcaaggtcat
cggttcgaacccggttggaagagc
1-5tRNA-Leu(taa)[157972,158055]
tgctgagatgcccgagtggtctaagggggacgtcttaagaacgtctggcg
caagcctcacgggttcgaaccccgttctcagcat
1-6tRNA-Thr(agt)[158059,158130]
gcactcatagctcaggggtagagcgtaagtttagtaagcttaaggtcaag
ggttcaaatccctttgagtgca
>KC662249.1 Phaeocystis globosa virus strain 16T, complete genome
1-1tRNA-Leu(caa)[176759,176838]
gtattggtgcccgagtggttaagggtaaatttcaagcatatatggcgaaa
gcctcgtgggttcgaatcccgcccaataca
1-2tRNA-Ile(tat)[408944,409017]
gttctattaactcagttggttagagtgtcggtcttatgagccgaaagtcg
tgggttcgagtcccacatagaaca
1-3tRNA-Gln(ttg)[409027,409100]
agttcccatagtgtagtggttatcacggtggtttttgataccacaaactc
tggttcgaatccaggtgggaactt
1-4tRNA-Asn(gtt)[409104,409179]
tgcctccatagctcagttggtcagagcgtgcggctgttaaccgcgaggtc
accggttcgaaaccggttggaggcgt
1-5tRNA-Leu(taa)[409206,409291]
tatcacggtgtccgagtctggtttaaggagcaagatttaagaccttgtgc
gttcgcgctcgcgggttcgaatcccgcccgtgatat
1-6tRNA-Asn(gtt)[409295,409370]
tgcctccatagctcagttggtcagagcgtgcggctgttaaccgcgaggtc
accggttcgaaaccggttggaggcgt
1-7tRNA-Leu(taa)[409393,409476]
gtcacggtgtccgagtctggtttaaggagcaagatttaagaccttgtgcg
ttcgcgctcgcgggttcgaatcccgcccgtgaca
1-8tRNA-Arg(tct)[410486,410560]
tgcccgcgtgacgcaatggatagcgtgccagacttctaatctggaagttg

cgggttcgagtcccgtcgtgggtaa
>KJ645900.1 Aureococcus anophagefferens virus isolate BtV−01, complete genome
1−1tRNA−Thr(agt)c[118324,118395]
gcttttgtggcttaatggtaaagcgacatcctagtaagatgtagattccg
cgttcgattcgcggcaaaagct
1−2tRNA−Arg(tct)c[148317,148390]
gccctattggcgaaattggatatcgcgtccgccttctaagcggaagattc
tgggttcgatccccagatagggtt
1−3tRNA−Leu(taa)[322252,322333]
atctgtttggcggagtggtctaacgcgccggatttaagccccggtctcta
acgaggcgcaagttcgaaccttgcaacagata
1−4tRNA−Ile(tat)[322371,322446]
ttcgcctataattcaatctggttagaatgaccgtcttataagcggtttat
atgggttcaaatcccattaggcgaat
1−5tRNA−Ser(tga)[322625,322707]
gatactgtgatggaattggtagacatgtcagacttgaaatctgttgcccg
atgggcgtgagcgttcgagtcgctccagtatct
1−6tRNA−Leu(taa)[322727,322812]
ccgtctatagttcagttagaagaacgttcgactgtaatggttaagcatat
atcgaaaggtcgctggtgcaagtccagctagacgga
1−7tRNA−Gln(ttg)[322836,322906]
gctcatatggtgtaatggtagcactaaagattttgattctttcaatttca
gttcgattctgaatatgagct
>KP874737.1 Ostreococcus lucimarinus virus 7 isolate OlV7, complete genome
1−1tRNA−Gln(ttg)[136442,136528]
agcttttatggtgtagtggtaacactgcggactttgactttaacgaagac
gatccgccaccctaggttcgaatcctagtagaagctt
1−2tRNA−Leu(tag)[136532,136619]
ccagtgttagctcagttggaagagcagtggattgtagtagtatgatatag
atctccacgggtcgggtgttcgaatcatccacactgga
1−3tRNA−Asn(gtt)[136623,136698]
ttcccttgtaactcagttggttagagtgttcgactgttaatcgagaagcc
accggttcgaatccggtcaagggaga
1−4tRNA−Thr(agt)[136703,136775]
gcacctgtagcatagtggttaatgcgcctctttagtaaggaggagaccgc
gtgttcgaatcacgccaggtgca
>KT820662.1 Chrysochromulina ericina virus isolate CeV−01B, complete genome
1−1tRNA−Leu(caa)c[74479,74562]
tgtatggatgcccgagtggtctaagggggtagactcaagttctactgtcg
taagactcgtgggttcgaatcccactccatacaa
1−2tRNA−Ser(tga)[235796,235880]
tgacatcgtgcccgagttggttaaggggatggacttgaaatccattgggc
attgccctcgcgagttcgaatctcgtcgatgtcgt
1−3tRNA−Ala(tgc)[235890,235962]

ggggatgtagatcaattggtagatcgctcgctttgcatgcgagaggtacc
gggatcaaaacccagcatctcca
1-4tRNA-Ser(gct)[235968,236051]
tgacaccgtggccgagtggttaaggcgatggactgctaatccatttccct
atgggatcgcgagttcgaatctcgtcggtgtcga
1-5tRNA-Gly(tcc)c[383694,383764]
gcactattggtgtagtggtaacatgttcgccttccaagcgatcgcccagg
gttcgattccctgatagtgca
1-6tRNA-Ile(tat)[423375,423450]
tgctcgcgtaactcagttggttagagtgttggtcttatgagccaaaagtc
cgcggttcgagcccgcgcgtgagcat
1-7tRNA-Lys(ttt)[423493,423567]
ttctcggataactcagttggtagagtgggcgtctttttggggtctagtcc
ctagttcgagtccaggttcgagaaa
1-8tRNA-Gln(ttg)[423572,423643]
gctcctgtagtgtagaggtaatcactgaggactttgaatcctccaacgcc
agttcgaatctggccgggagct
1-9tRNA-Leu(taa)[423766,423847]
gcatggatgcccgagtggtctaagggggcagacttaagatctgctggcga
aagcctcgtgggttcaaaccccactccgtgca
1-10tRNA-Asn(gtt)[423964,424038]
gctccgttagctcagttggttagagcgtgcggctgttaaccgcaatgtca
ctggttcgatcccagtacggagcgc
1-11tRNA-Lys(ttt)[424072,424144]
gttcccttagctcagttggtagagcgcacgacttttaatcgtgtggccag
cggttcgagcccgctagggaata
1-12tRNA-Arg(tct)[424179,424252]
gcccctgtggcgtaattggatagcgcaccagacttctaatctgggggttg
cgagttcgaatctcgccaggggtt
>KP874735.1 Ostreococcus mediterraneus virus 1 isolate OmV1, complete genome
1-1tRNA-Gln(ttg)[150843,150915]
agcttctatggtgtagtggtaacacagtggactttgaatccaccgccaca
ggttcgatccctgttggaagctt
1-2tRNA-Asn(gtt)[151019,151092]
tcactcatcgtctagtggtttaggacaatcggctgttaaccggttaaccg
gggttcgaatccctgtgagtgaac
1-3tRNA-Thr(agt)[151095,151168]
tgcatctatggccaagtggtaaggcgtctctttagtaaggagaagatcgt
gcgttcgaaccgcactagatgcat
>KP874736.1 Ostreococcus lucimarinus virus 2 isolate Olv2, complete genome
1-1tRNA-Gln(ttg)[145281,145354]
agctcttatagtgtagtggtcatcactttggactttgaatccaacaaccc
tggttcaaatccaggtaggagctt
1-2tRNA-Asn(gtt)[145466,145540]

tttccttgtaactcaatcggaagagtgtacgactgttaatcgtaaagtag
cgagatcgaaactcgccaaggaagt
1−3tRNA−Thr(agt)[145544,145617]
tgcatctatggccaagtggtaaggcgtctctttagtaaggagaagatcgt
gcgttcgaaccgcactagatgcat
>LC015649.1 Yellowstone lake phycodnavirus 3 DNA, complete genome, isolate: 3
1−1tRNA−Gln(ctg)[52689,52762]
gggttccatagtataacggttagtacatcagactctgactctgtaaatgc
gtgttcgattcacgctggaacctt
1−3tRNA−His(atg)[55378,55477]
tgctccaataacacaactggttagtgtatcggtcttatgaaaattgaatt
ttcatgcaagtgagccgaaaatccgagttcgatcctcggttggagcagcc
1−4tRNA−Arg(tct)[55483,55557]
gactctgtggcgaaattggatatcgcgtgggacttctaatcccgagattg
cgggttcaaaccccgccagagccgc
1−5tRNA−Leu(taa)[55560,55646]
ttcatcagtgtccgagttggtctaaggagggagacttaagatctcctgtg
cgcaagcacgcgtgggttcgaaccccacctgatgaaa
>LC015648.1 Yellowstone lake phycodnavirus 2 DNA, complete genome, isolate: 2
1−1tRNA−Thr(agt)c[79624,79698]
tgctttcgtagctcaattggtagagcacccgtttagtaagcgggaggtaa
tgagatcaaagctcatcgaaagcag
1−2tRNA−Pro(agg)c[79979,80077]
ggctctgtagcacaattggatagtgcaccagccttctaggaggaccttcg
gtccgtcagtgtaagctggaggttgcgggttcgacccccgccagagtca
1−3tRNA−Gln(ttg)c[80084,80160]
gcatcggtgtccgagcctggttcaaggagagatctgttggtgttttcacc
acacgggttcaaatcccgtccgatgca
1−4tRNA−Ile(tat)c[80161,80234]
gctctcgtaactcagatggttagagtgttggtcttatgtgccaaaagtcg
tgagttcaatcctcaccgggagca
>LC015647.1 Yellowstone lake phycodnavirus 1 DNA, complete genome, isolate: 1
1−1tRNA−Leu(taa)[60048,60130]
agtagtgtaggagagtggtcaaatccgttgcacttaagatgcaatacctt
cgcgtttcgcgggttcgaatcccgccgctacta
1−2tRNA−Ile(tat)[60135,60209]
gttctagtaactcagttggttagagtgcgggtcttatgagcccgaagtcg
cgggttcgacccccgcctagaacac
1−3tRNA−Glu(ttc)[60211,60300]
tggccctgtggcataatcggataatgcgtcagccttctactcaagagaca
gcgagctgaagattgcgggttcgacccccgccagggtcat
1−4tRNA−Gly(tcc)[60823,60894]
gcgcatatagtatagtggttagtacagaacccttccaaggttcaaggcgg
ggttcaattccccgtgtgcgca

1−5tRNA−Thr(agt)[60898,60970]
gcctctgtagctcaattggtagagcactcgtttagtaagcgagaggtatt
gagatcaaaactcaacggaggca
1−6tRNA−Gln(ctg)[60976,61047]
ggttccatagtataacggttagtacacaggactctgaatcctgtaatggg
agttcgattctccctggaacct
1−7tRNA−Asn(gtt)[61048,61122]
tcctctataactcagctggttagagtgtcaggctgttaacctgaaagtcg
caggttcgatccctgctggaggagc
>KX857749.1 Only Syngen Nebraska virus 5, complete genome
1−1tRNA−Leu(taa)[166543,166628]
gatagtgtatgcaagtggtcaaagcacctcgacttaagatcgagtccctt
tcgggttcgcgggttcgacccccgtcgctatcacca
1−2tRNA−Ile(tat)[166629,166710]
tcgacattaagataacggtcattcgctgggtcttatacatttatgaaagc
ctggaacccgggttcaactcccggatgtcgaa
1−3tRNA−Leu(taa)[166734,166819]
gatagtgtatgcaagtggtcaaagcacctcgacttaagatcgagtccctt
tcgggttcgcgggttcgacccccgtcgctatcacca
1−4tRNA−Ile(tat)[166820,166901]
tcgacattaagataacggtcattcgctgggtcttatacatttatgaaagc
ctggaacccgggttcaactcccggatgtcgaa
1−5tRNA−Leu(taa)[166925,167010]
gatagtgtatgcaagtggtcaaagcacctcgacttaagatcgagtccctt
tcgggttcgcgggttcgacccccgtcgctatcacca
1−6tRNA−Asn(gtt)[167117,167190]
gacgccatagctcagttggttagagcgttcgactgttaatcgagaggtcg
tcggttcgaacccgactggtgtcg
1−7tRNA−Gly(tcc)[167193,167265]
tggcacattaatataacggtagtataacagccttccaagctgtaagcctg
ggttcgactcccagatgtgtcaa
1−8tRNA−Asn(gtt)[167288,167361]
gacgccatagctcagttggttagagcgttcgactgttaatcgagaggtcg
tcggttcgaacccgactggtgtcg
1−9tRNA−Lys(ctt)[167364,167438]
tgcccgtctagctcagtcggtagagcgccagactcttaatctggtggtcg
tgggttcgagccccacggtgggcaa
1−10tRNA−Arg(tct)[167464,167537]
tgtctgtatggtgtaatggatagcaccttgagcttctaactcaatgatcc
gagttcgatcctcggtacagatat
1−11tRNA−Arg(tct)[167563,167636]
tgtctgtatggtgtaatggatagcaccttgagcttctaactcaatgatcc
gagttcgatcctcggtacagatat
1−12tRNA−Asp(gtc)[168747,168819]

gacgaattagtatagtggttagtatccctgcctgtcacgcaggagaccgg
ggttcaattccccgattcgtcgc
1-13tRNA-Leu(caa)[168820,168903]
gatagtgtatgcaagtggtcaaagcgcctggtctcaagatccagtccttc
gggttcgcgggttcgactcccgtcgctatcacca
1-14tRNA-Thr(tgt)[168904,168976]
gccggtttagcatagtggtattgcaccagtcttgtaaactggaggccggg
agttcgatcctcccaatcggcac
*Podoviridae
>AF125520.1 Bacteriophage 933W, complete genome
1-1tRNA-Met(cat)[21120,21195]
ggcccctttagctcagtggtgagagcgagcgactcataatcgccaggtcgc
tggttcaaatccagcaagggccacca
1-2tRNA-Arg(tcg)[21205,21281]
ccgccattagctcatcgggacagagcgccagccttcgaagctggctgcgc
ggggttcgagtcctcgatggcggtcca
1-3tRNA-Arg(tct)[21295,21371]
gcgttgttagctcagccggacagagcaattgccttctgagcaatcggtca
ctggttcgaatccagtacaacgcgcca
>AF157835.1 Acyrthosiphon pisum bacteriophage APSE-1, complete genome
1-1tRNA-Lys(ttt)[32824,32898]
gggctgttagcttaacgatagagcagagggcttttagcccttcgcttgcg
ggtttgagtcctgcacaggccgcca
>AY078382.2 Pseudomonas aeruginosa phage PaP3, complete genome
1-1tRNA-Asn(gtt)[1628,1703]
tgggatgtagctcagttggtagagcagggagctgttaactctcaggtcgt
aggttcgagccctaccgtcccagcca
1-2tRNA-Asp(gtc)[1708,1783]
ggcccattaacttagcggttagagtgctcccctgtctagggagaagccgt
cggttcgaatccgacatgggtcgcca
1-3tRNA-Tyr(gta)[1789,1875]
ggtcgttgtagaggtagatggctactaccatccggttgtaacccggacgc
cttaggctagaggttcaattccttccttcgaccacca
1-4tRNA-Pro(tgg)[1880,1955]
ctcctcgtagctcagtctggttagagtgcctgccttggaagcaggaggtc
gtaggttcgaatcctaccggggtgac
>AY349011.3 Burkholderia cepacia phage Bcep22, complete genome
1-1tRNA-Ser(tga)[8351,8439]
ggaagcgttgagcattggcaggctccgcggatttgaaatccgtcatacgt
gtgagcgtattgggggttcgactccccccgcttccgcca
1-2tRNA-Ala(tgc)c[53700,53799]
gtcgcgttcgagcgccacggccttcgacgcggccgggttctgcgcgcccg
gctgatagatgccctcgggcaggttggcgatcgccttggcgaatgccgcg
>AY052766.1 Salmonella typhimurium bacteriophage ST64T, complete genome

1-1tRNA-Asn(gtt)[21039,21113]
gggtcagtcgtataaaggtcattacggaaggctgttaaccttcttatcgt
ggttcgagtccacgctgtcccgcca
>AY095314.2 Vibriophage VpV262, complete genome
1-1tRNA-Pro(tgg)c[45250,45327]
cggggtatgctcgcagtctggtagcgaggtggctttggaagccagcggtc
gtaggttcgaatcctactaccccgacca
>AP000363.1 Enterobacteria phage VT2-Sakai proviral DNA, complete genome
1-1tRNA-Met(cat)[20611,20686]
ggcccctttagctcagtggtgagagcgagcgactcataatcgccaggtcgc
tggttcaaatccagcaagggccacca
1-2tRNA-Arg(tcg)[20696,20772]
ccgccattagctcatcgggacagagcgccagccttcgaagctggctgcgc
ggggttcgagtcctcgatggcggtcca
1-3tRNA-Arg(tct)[20786,20862]
gcgttgttagctcagccggacagagcaattgccttctgagcaatcggtca
ctggttcgaatccagtacaacgcgcca
>AF547987.1 Enterobacteria phage Sf6, complete genome
1-1tRNA-Asn(gtt)[36065,36139]
gggtcagtcgtataaaggtcattacggaaggctgttaaccttcttatcgt
ggttcgagtccacgctgtcccgcca
1-2tRNA-Thr(tgt)[36145,36220]
gctggtttagctccaatggtagagcagtcgccttgtaagcgaatgggtag
cggttcaagtccgttaaccagcacca
>DQ126339.2 Enterobacteria phage phiV10, complete genome
1-1tRNA-Arg(tct)c[2853,2929]
gtggcattagctcagttggacagagcaaccgccttctaagcggttggtcg
caggttcgaatcctgcatgccacgcca
>EF056009.1 Enterobacteria phage N4, complete genome
1-1tRNA-Asn(gtt)[32451,32525]
gatggtgtagctcagtggtagagcagttggctgttaaccaactggtcgat
ggttcgaatccatccaccatcgcca
1-2tRNA-Ser(tga)[32531,32620]
ggaaggttgtcagagaggccgattgtacctgacttgaaatcaggaggcgc
agagatgcacccgaaggttcgaatccttcaccttccacca
1-3tRNA-Thr(tgt)[32625,32700]
gctggtttagctcagttggtagagcaattgccttgtaagcaatgggtcaa
tggttcaagtccattaaccagcacca
1-4tRNA-Pro(tgg)[33054,33130]
caggatatagctcagtttggtagagcgtctgctttgggagcagaatgtcg
taggttcgaatcctgctatcctgacca
>AB255436.1 Stx2-converting phage 86 DNA, complete genome
1-1tRNA-Met(cat)[59897,59972]
ggcccctttagctcagtggtgagagcgagcgactcataatcgccaggtcgc

tggttcaaatccagcaagggccacca
1−2tRNA−Arg(tcg)[59982,60058]
ccgccattagctcatcgggacagagcgccagccttcgaagctggctgcgc
ggggttcgagtccccgatggcggtcca
1−3tRNA−Arg(tct)[60072,60148]
gcgttgttagctcagccggacagagcaattgccttctgagcaatcggtca
ctggttcgaatccagtacaacgcgcca
>EU311208.1 Enterobacteria phage Min27, complete genome
1−1tRNA−Met(cat)[22825,22900]
ggccctttagctcagtggtgagagcgagcgactcataatcgccaggtcgc
tggttcaaatccagcaagggccacca
1−2tRNA−Arg(tcg)[22910,22986]
ccgccattagctcatcgggacagagcgccagccttcgaagctggctgcgc
ggggttcgagtcctcgatggcggtcca
1−3tRNA−Arg(tct)[23000,23076]
gcgttgttagctcagccggacagagcaattgccttctgagcaatcggtca
ctggttcgaatccagtacaacgcgcca
>DQ535032.1 Lactococcus lactis phage KSY1, complete genome
1−1tRNA−Met(cat)[70260,70332]
ggttctttagctcaataggcagagcatgcagcccataactgcaaaggcgt
aggttcaattcctacaggaacca
1−2tRNA−Arg(tct)[71312,71389]
tggatctttagctcaattggaattagagcaacattcttctaaagtgtagg
ttactggttcgagtccagtaaggtccat
1−3tRNA−Gln(ttg)[77160,77232]
tggaatatagtttagattggtagaacgcaggactttgaatcctgtagcat
tggttcgagtccagttattccag
>EU124666.1 Thalassomonas phage BA3, complete genome
1−1tRNA−Ile(gat)c[13217,13316]
tggcgactttcagaatggtcgaatttatataatggctgataactattaca
cggacgatggcgacccggttgtaagggagttcattctcccggttgttaac
>EU330206.1 Enterobacteria phage phiEco32, complete genome
1−1tRNA−Arg(tct)c[66351,66427]
gcgcccttagttcaattggacagaacatcggatttctaatccgtcggtta
caggttcgaatcctgtagggtgcgcca
>AM910650.1 Pseudomonas phage LUZ24, complete genome
1−1tRNA−Pro(tgg)c[44603,44679]
ctcctcgtagctcagttggttagagtgcctgtcttggacacaggaggtcg
taggttcgagtcctaccggggtgacca
1−2tRNA−Tyr(gta)c[44684,44770]
ggtcgttgtagaggtagatggctactaccatccggctgtaacccggacgc
cattggctagaggttcaattccttccttcgaccacca
1−3tRNA−Asn(gtt)c[44776,44851]
tgggatgtagctcagttggtagagcagggagctgttaactctcaggtcgc

aggttcgagccctgccgtcccagcca
>FJ591094.1 Roseophage EE36P1, complete genome
1−1tRNA−Pro(tgg)[72437,72513]
ccgtgtgtagcgcagtctggtagcgcactagttttgggtactaggggtct
caggttcgaatcctgatacacggacca
1−2tRNA−Ile(gat)[72520,72595]
ggtggggtagctcagttggtagagcaggcgaccgataatcgtcaggtcga
aggttcaaacccttcccccaccacca
1−3tRNA−Ser(tga)[72598,72687]
ggaagagtggccgagtctggctgaagacactggtcttgaaaaccagcaaa
ccgaaaggtttcgtgggttcgaatcccacctcttccgcca
>EU794049.1 Bacteriophage APSE−2, complete genome
1−1tRNA−Lys(ttt)[36173,36247]
gggctgttagcttaacgatagagctgagggcttttagcccatcgcttgcg
ggtttgagtcctgcacagcccgcca
>FJ591093.1 Roseophage DSS3P2, complete genome
1−1tRNA−Pro(tgg)[73610,73686]
ccgtgtatagcgcagtctggtagcgcgatagttttgggtactataggtct
caggttcgaatcctgatacacggacca
1−2tRNA−Met(cat)[73693,73767]
ggtggggtagctcagaggtagagcggaggactcataatcctcatgtcgtt
ggttcgattccttcccccaccacca
1−3tRNA−Ser(tga)[73770,73859]
ggaagagtggccgagtctggctgaaggcactggtcttgaaaaccagcaaa
ccgaaaggtttcgtgggttcgaatcccacctcttccgcca
>FJ937737.2 Burkholderia cenocepacia phage BcepIL02, complete genome
1−1tRNA−Ser(tga)[7204,7293]
ggaggcgtggcagagtggtctaatgcagcggatttgaaatccgtcatacg
tgtgagcgtatcgtgagttcgaatctcaccgcctccgcca
1−3tRNA−Ser(cga)c[23761,23856]
aggtcgtctgctgcgacggcgacgggctggcggtgttcgagctgctgcgt
tcgcgccggcacgacggcgaggcgttcctgtacgcgttcgacctgc
>GU573886.1 Salmonella phage ST160, complete genome
1−1tRNA−Asn(gtt)[20853,20927]
gggtcagtcgtataaaggtcattacggaaggctgttaaccttcttatcgt
ggttcgagtccacgctgtcccgcca
>HM997019.1 Salmonella phage 7−11, complete genome
1−1tRNA−Arg(tct)[2456,2531]
gtttccatagcttaacggatagagcaacagccttctaagctgtcggttga
aggttcgaatccttctgggaacgcca
1−2tRNA−Asn(gtt)[2537,2612]
gggtgataagcacatatggatgtgcggtggactgttaatccaatggtagt
tggttcgaatccaacattgcccgcca
1−3tRNA−Gln(ttg)[2620,2693]

agggatgtagtgtaacggtagcacttcggactttgactccgacagcctag
gttcgaaacctagcgtccctgcca
1-4tRNA-Trp(cca)[2732,2805]
gggtgattcgtatagtggtattacgctggattccaaacccagtcaccgga
gttcgattcttcgattgcccgcca
1-5tRNA-Met(cat)[2809,2886]
tgcgagttacagttctggttgaactggtcagtctcataaactgaataaag
gtaggttcgattcctgcactcgcctcca
1-6tRNA-Phe(gaa)[3053,3127]
gcaaacatagctgagatggattagcgcttgcctgaagagcttgagagctt
ggttcgattccaagtgtttgcacca
1-7tRNA-Cys(gca)c[81610,81685]
gggtagctggctgagtggcttaggcatcggattgcaaatccgaatcacac
ccgttcgaatcgggtgctaccctcca
>GU071102.1 Cyanophage NATL1A-7, complete genome
1-1tRNA-Gly(tcc)c[3479,3549]
gcgggtatagtttagaggtaaaactgcagccttccaagctgttgtcagcg
gttcgaatccgcttacccgct
>JN391180.1 Salmonella phage SPN1S, complete genome
1-1tRNA-Arg(tct)c[2813,2889]
gtggcattagctcagttggatagagcaaccgccttctaagcggttggccg
caggttcgaatcctgcatgccgcacca
>JF900176.1 Salmonella phage SPN9CC, complete genome
1-1tRNA-Asn(gtt)[20191,20265]
gggtcagtcgtataaaggtcattacggaaggctgttaaccttcttatcgt
ggttcgagtccacgctgtccagcca
1-2tRNA-Thr(tgt)[20271,20346]
gctggtttagctccaatggtagagcagtcgccttgtaagcgaatgggtag
cggttcaagtccgttaaccagcacca
>JX415536.1 Escherichia phage ECBP2, complete genome
1-1tRNA-Arg(tct)c[67610,67685]
gcacccttagctcaatggatagagcagcgaccttctaagtcgttggttac
aggttcgaatcctgtagggtgcacca
>JN662425.1 Burkholderia phage DC1, complete genome
1-1tRNA-Ser(tga)[8262,8351]
ggaggcgtggcagagtggtctaatgcagcggatttgaaatccgtcatacg
tgcgagcgtatcgtgagttcgaatctcaccgcctccgcca
>JX867715.1 Enterobacteria phage NJ01,complete sequence
1-1tRNA-Arg(tct)c[72679,72753]
gtccctatagttaaaaggatataacagatttcttctaaaaatccattcta
ggttcgagtcctagtggggacgcca
>JN882286.1 Cronobacter phage vB_CsaP_GAP52, complete genome
1-1tRNA-Asn(gtt)[2173,2248]
ggacgataagcacatatggatgtgcggtgagctgttaactcaatggtagt

tggttcgaatccaacatcgtccgcca
1−2tRNA−Trp(cca)[2254,2327]
gggggtttggtataatggcattatgacggtctccaaaaccgttgatagag
gttcgattcctctaaccccсgcca
>JX415535.1 Escherichia phage ECBP1, complete genome
1−1tRNA−Ala(cgc)[34010,34081]
ggtcgaggtagcacgtaggcatgtgcagatggtcgctacttctctcgctg
gttcgaatccagcccccgacct
1−2tRNA−Cys(aca)[34373,34451]
gtgtgtgatagctcaaataggtagagcgtgaggctacagtgctgaatggt
ttgtgggttcgattcccacccatgcctac
1−3tRNA−Ile(aat)[35154,35228]
gcccacttagcttagacggggaaagcaaccgactaataatcggaaggtca
ctggttcaaatccagtagtgggtac
>HE664024.1 Escherichia phage P13374 complete proviral genome
1−1tRNA−Met(cat)[23592,23667]
ggcccctttagctcagtggtgagagcgagcgactcataatcgccaggtcgc
tggttcaaatccagcaagggccacca
1−2tRNA−Arg(tcg)[23677,23753]
ccgccattagctcatcgggacagagcgccagccttcgaagctggctgcgc
ggggttcgagtcctcgatggcggtcca
1−3tRNA−Arg(tct)[23767,23843]
gcgttgttagctcagccggacagagcaattgccttctgagcaatcggtca
ctggttcgaatccagtacaacgcgcca
>JN939331.1 Brucella phage Tb, complete genome
1−1tRNA−Leu(cag)c[10121,10196]
gcgagcaggaggctcaggacggtcacggaactcagacttccgttcggtct
gctgatccttaacctgctcgcttgcg
>HQ641380.1 Enterobacter phage EcP1, complete genome
1−1tRNA−Asn(gtt)[55469,55543]
ggtaagttagctaataggtgaagcacctgactgttaatcagtgatagagt
ggttcgattccactacttaccgcca
1−2tRNA−Pro(tgg)[55547,55623]
cgggatatagctcagtttggtagagtgtctgctttgggagcagaatgtcg
ttggttcaagtccagctatcccgacca
1−3tRNA−Met(cat)[55628,55704]
gtgcctttagctcagacggttagagcagccgactcataatcggtaggtca
caggttcaaatcctgtagggcgcacca
>JQ011318.1 Escherichia phage TL−2011c, complete genome
1−1tRNA−Met(cat)[24095,24170]
ggcccctttagctcagtggtgagagcgagcgactcataatcgccaggtcgc
tggttcaaatccagcaagggccacca
1−2tRNA−Pro(tgg)[24180,24256]
ccgccattagctcatcgggacagagcgccagccttggaagctggctgcgc

ggggttcgagtcctcgatggcggtcca
1−3tRNA−Arg(tct)[24270,24346]
gcgttgttagctcagccggacagagcaattgccttctgagcaatcggtca
ctggttcgaatccagtacaacgcgcca
>JQ446452.1 Salinivibrio phage CW02, complete genome
1−1tRNA−Arg(tct)[44194,44270]
gccccgttagttcaattggatagagcaacggccttctaagccgtgagtta
caggttcgaatcctgtacggggtgcca
>HF543949.1 Pseudomonas phage vB_PaeP_p2−10_Or1, complete genome
1−1tRNA−Asn(gtt)[553,628]
tgggatgtagctcagttggtagagcagatagctgttaactatccggtcgt
aggttcgagccctaccatcccagcca
1−2tRNA−Asp(gtc)[633,708]
ggcccattaactcagcggtaagagtgctcccctgtctagggagaagtctg
gggttcgaatccccaatgggtcgcca
1−3tRNA−Pro(tgg)[713,787]
ctcctcgtagctcagtctggttagagtgcctgccttggaagcaggaggtc
gtaggttcgaatcctaccggggtga
>JX880034.1 Enterobacter phage IME11, complete genome
1−1tRNA−Ile(aat)c[27897,27972]
gtccacttagcttagacggggaaagcaaccgactaataatcggaaagtca
ctggttcaaatccagtagtggacacc
>JQ011317.1 Escherichia phage TL−2011b, complete genome
1−1tRNA−Arg(tct)c[14420,14496]
gtggcattagctcagttggacagagcaaccgccttctaagcggttggtcg
caggttcgaatcctgcatgccacgcca
>JX104231.1 Burkholderia phage BcepMigl, complete genome
1−1tRNA−Ser(tga)[7811,7898]
ggaggcgtggcagagtggtttaatgtactggatttgaaatccagcgacgg
taacccgtccgtgagttcgaatctcaccgcctccgcca
>KC465900.1 Pelagibacter phage HTVC011P, complete genome
1−1tRNA−Leu(tag)[5592,5676]
gggcgagtggtggaattggtagacacgccagtcttaggaactggtgtcgc
aagacgtgtaggttcaaatcctatctcgcctacca
>HQ634194.1 Vibrio phage VBP47 genomic sequence
1−1tRNA−Trp(cca)[2550,2633]
gggggagtcccatagcggcaattggagcggtttccaaaaccgttggtata
tccttcataggttcgagtcctatctcccccgcca
>KC465901.1 Pelagibacter phage HTVC019P, complete genome
1−1tRNA−Cys(gca)c[8946,9019]
ggcaacgtggcggaatggttacgcagtggattgcaaatctacctatccca
gttcaattctgggcgttgcctcca
>HQ317387.2 Sulfitobacter phage phiCB2047−B genomic sequence
1−1tRNA−Arg(tct)c[56997,57073]

ggtcccttagttcaactggatagaatgccccacttctaatgggtaggttg
taggttcgaatcctacagggactgcca
1−2tRNA−Met(cat)[68618,68691]
tgcgcgttggtgtaatggtaacacatttggttcatgcccataggctgagg
gttcgattccctcactcgcaacca
1−3tRNA−Tyr(gta)[68775,68857]
gggacggctcatctattggcgaagataggagactgtaaatctctcggtaa
actgtgctggttcgactccagcccgtcccacca
1−4tRNA−Trp(cca)[68872,68945]
gccagcgtagtatattggtattacagcggcttccaaacccgaagaacaca
gttcgattctgtgctctggtgcca
1−5tRNA−Leu(tag)[68962,69039]
gcccctctagcccaattggtaggaggcgaatgttttagacacattacagt
cccagttcgaatctggggaggggtacca
1−6tRNA−Leu(caa)[69060,69136]
gtcccgctgggcaaacggtaaagccgcagcactcaaaatgcttgattttc
ttggttcgaatccaaggcgggatacca
1−7tRNA−Ser(gct)[69150,69238]
ggaagaagaatctgttgggaacagaggctggttgctaaccagtacgaggg
gtctaactccttggggatcgtgacctccttcttccgcca
1−8tRNA−Ser(tga)[69246,69333]
ggatagtctcgctacgtgggtggcaaacggtcttgaaaaccgtgatgact
ccgaaaggatgagggttcaactccttggctatccgcca
1−9tRNA−Cys(gca)[69338,69415]
gatcgtgtggctcgaatggctttaggcatcggattgcaaccccgaattat
acaggttcgattcctgtcacgatctcca
1−10tRNA−Gln(ttg)c[69436,69511]
tgggaagtagcttaactggtaaagcccgtgactttgattcacgtgagtgc
aggttcgaaccctgccttcccaacca
1−11tRNA−Phe(gaa)c[69768,69842]
gcgtaggtagctcaggggtagagcttctggttgaagcccagagagtcggt
ggttcaattccatctctacgcacca
1−12tRNA−Lys(ttt)c[69849,69925]
gcgccgttagctcagttggttagagcacccgacttttaatcggggggtcc
agagttcaagtctctgacggcacacca
1−13tRNA−Ile(gat)c[69938,70011]
attggattagctcagatggtagagcaacgggccgataacccgtgtgtcac
tggttcaagtccagtatccaatac
1−14tRNA−Asn(gtt)c[70021,70097]
ttcctatggcccctactggtagggggatgcggctgttaaccgtacagttc
taggttcgagtcctagatagggagcca
1−15tRNA−Pro(tgg)c[70108,70183]
cagggtgtagctcagcaggtagagtgctgcatttggaatgcagaagtcgc
aggttcaagccctgccaccctgacca

1-16tRNA-Thr(tgt)c[70193,70268]
gccgctatagctcagttggtagagcgcctgccttgtaaacaggatgtcca
gagttcaattctttgtggcggcacca
>JF974296.1 Pseudoalteromonas phage pYD6-A genomic sequence
1-1tRNA-Met(cat)[13930,14007]
ggccctaaagctcattaggtatgagcacccggctcataaccgggaggtag
ataggttcgaatccttctagggtcacca
1-2tRNA-Gln(ctg)[14014,14088]
gtctcctttagcttatgtaggtaaagcagtcgcccctgtggagtccgtgc
actggttcgaatccagtagaagaca
>HQ634196.1 Vibrio phage VBP32 genomic sequence
1-2tRNA-Trp(cca)c[4581,4664]
gggggagtcccatagcggcaattggagcggtttccaaaaccgttggtata
tccttcataggttcgagtcctatctcccccgcca
>KF005317.1 Alteromonas phage vB_AmaP_AD45-P1, complete genome
1-1tRNA-Met(cat)[53466,53541]
gcccctatagctcagcgtgtagagcagacgactcataatcgttaggtcac
tggttcaaatccggttaggggcacca
>KC821633.1 Cellulophaga phage phi13:2, complete genome
1-1tRNA-Cys(gca)c[63385,63457]
gttgttgtactcaagtggttaagaggttagtctgcaaaactaatatcgtg
agttcgaatctcaccaacaactc
>KC821609.1 Cellulophaga phage phi17:2, complete genome
1-1tRNA-Pro(tgg)[87649,87725]
cgggtaatagaggagtctggtttatctcgctgcatttgggatgcagagca
cgcaggttcgaatcctgcttatccgac
1-2tRNA-Glu(ttc)[87805,87877]
gctgccatcgtctaacggctaggacctcaggttttcatcctggtaatcgg
agttcgattctccgtggcagtac
1-3tRNA-Gln(ttg)[87948,88021]
tggaatatggtgtaattggtaacacgtctggttttggtccagaagagtgg
gggttcgagaccctctattccaac
1-4tRNA-His(gtg)[88023,88097]
gtgttagaagctaaagtggtcgaagcgtctggttgtggtccagaagatag
tgagttcgagtctcatttaacaccc
1-5tRNA-Leu(tag)[88156,88231]
gcagaggtgggtaaactggtaaagccgcccgatttaggctcgggagttct
gagggttcgagtccctctctctgtac
1-6tRNA-Leu(taa)[88238,88319]
gccccaatggtgaaactggttatacacgtagcacttaaaatgctatgctt
aggcttgcaggttcgatccctgcttggagtac
1-7tRNA-Arg(acg)[88324,88397]
tttctcatagtttaactggaaaaacacaaagttacggcctttgagagtag
gggttcgagtccctttgagaaaac

1−8tRNA−Ala(tgc)[88623,88696]
ggcccagtagctaaactggcttagcacctcctttgcaaggagaagtatgt
gggttcgagccccatctggcccac
1−9tRNA−Lys(ttt)[88778,88850]
accctcgtagcttaatggcaaagctcctctcttttaaagaggcgaccgca
agatcagtacttgtcgggggtac
1−10tRNA−Ile(gat)[88953,89027]
aggaatatagctcagttggttagagtacttcgctgataccgaagatgtca
caagttcgaatcttgttattcctac
1−11tRNA−Gly(tcc)[89131,89205]
gcagcaatcgcatagcggcctattgcacctgacttccaatcaggatctcg
tgagttcgaatctcacttgctgcac
1−12tRNA−Met(cat)[89210,89284]
agcgaggtagagcagctggttagctcgctaggctcataacctagaggtcg
ggagttcgagtctccccttcgccac
1−13tRNA−Asp(gtc)[89363,89436]
agtctatcggtccagtggagtggacgctgccctgtcacggcagagatcac
gggttcgagtcccgtatagactgc
1−14tRNA−Thr(tgt)[89515,89589]
tccctacgtagctcaattggtggagcactggttttgtaaaccaaaggttg
tcggttcgagtccgacctagggatc
1−15tRNA−Val(tac)[89736,89810]
accagattagctcaggggtttagagcaactcttttacaaggagaaggtcg
atagttcgaatctatcatttggtac
1−16tRNA−Arg(tct)[89821,89894]
gggggattagtttaactggaaaaactataggcttctatcctatcgagtag
gggttcgaatcccttatccccttc
1−17tRNA−Met(cat)[89904,89978]
ggccttgtagctcagttggttagagcagctcactcataatgagaaggtct
caggttcgagtcctgactaggccac
1−18tRNA−Tyr(gta)[90192,90276]
gggacggttgctcggctggaagaggcgcaggactgtaaattctgtaatcc
actaggaatggaggttcgagtcctcctcgtcccac
1−19tRNA−Ser(gct)[90546,90633]
agaaaggtgtcagagtggtttattgagctaggttgctaacttagtggagt
gaaataccttccgtgggttcgaatcccatcctttcttc
1−20tRNA−Asn(gtt)[90675,90747]
gacagggtagctcagtggtagagcagggagctgttaactctcaggtcgtg
ggttcgaatcccactcttgtctc
1−21tRNA−Trp(cca)[90809,90882]
acttgagtagctcagttggtagagcagtggtctccaaaaccaaaggtcgt
aggttcgagtcctacctctcgtgc
1−22tRNA−Phe(gaa)[90964,91038]
ggggttgtagctgagttggtacaagcgtccgattgaagctcggaagtacg

aagggtcgaagccttccttccccac
1-23tRNA-Ile(gat)[142711,142786]
aatcgggtagttcaattggtagaacatcacactgataatgtgaaggttgt
gggttcgagtcccatcccgattacca
>KC438282.1 Vibrio phage JA-1, complete genome
1-1tRNA-Arg(tct)[13653,13729]
gtcctgttagctcaattggatagagcaagatgtttctaccgtctaggtta
taggttcgaatcctatacgggatgcca
>KC139517.1 Salmonella phage FSL SP-058, complete genome
1-1tRNA-Lys(ttt)[66706,66782]
ggattgttaactcagttggttagagtatctcacttttaatgagagggtcg
aaggttcgagtccttcacaatccacca
1-2tRNA-Pro(tgg)[66787,66863]
caggatatagctcagcttggtagagcatctgctttgggagcagactgtca
gaggttcaagtcctcttatcctgacca
1-3tRNA-Tyr(gta)[66870,66953]
gtggggttggcagagtggtcgattgcgggggactgtaaatcctcactgaa
aggcgcggtggttcgaatccatcatcccacacca
1-4tRNA-Ser(gct)[66960,67045]
ggaagattggcagagcggtaatgcagcaccctgctaaggtgtacaaccga
aaggttgcataggttcaaatcctatatcttccgcca
1-5tRNA-Met(cat)[67191,67268]
ggttccatagctcaagttggttagagcactcggctcataaccgagagtgt
ccaggttcgaaaccgggtggaaccacca
1-6tRNA-Ile(gat)[67272,67348]
tgtctcatagctcagtaggtcagagcgttcccctgataagggaaaggtca
ctagttcgaatctagttgagacaacca
1-7tRNA-Ser(tga)[67660,67745]
ggaagattccgctcagtggtgggcaatccggtttgaacccggaggtgctg
gtaacggtaggggttcgactcctcaatcttccgcca
1-8tRNA-Asn(gtt)[67751,67826]
gatgggatagctaactcggtaaagcaccagactgttaatctgcgacatta
gggttcaaatccctatcccatcgcca
1-9tRNA-Arg(tct)[68158,68234]
gcatccttagctcagcaggatagagcagtagccttctaagctattggtca
ctggttcgaatccagtagggtgtacca
1-10tRNA-Leu(taa)[68237,68322]
gggagtatggtgaaatcggtagacacaagggatttaaaatccctcggctt
atagctgtacgagttcgagtctcgttactcccacca
>KC139520.1 Salmonella phage FSL SP-076, complete genome
1-1tRNA-Lys(ttt)[61575,61651]
ggattgttaactcagttggttagagtatctcacttttaatgagagggtcg
aaggttcgagtccttcacaatccacca
1-2tRNA-Pro(tgg)[61656,61732]

caggatatagctcagcttggtagagcatctgctttgggagcagactgtca
gaggttcaagtcctcttatcctgacca
1-3tRNA-Tyr(gta)[61739,61822]
gtggggttggcagagtggtcgattgcgggggactgtaaatcctcactgaa
aggcgcggtggttcgaatccatcatcccacacca
1-4tRNA-Ser(gct)[61829,61914]
ggaagattggcagagcggtaatgcagcaccctgctaaggtgtacaaccga
aaggttgcataggttcaaatcctatatcttccgcca
1-5tRNA-Met(cat)[62060,62137]
ggttccatagctcaagttggttagagcactcggctcataaccgagagtgt
ccaggttcgaaaccgggtggaaccacca
1-6tRNA-Ile(gat)[62141,62217]
tgtctcatagctcagtaggtcagagcgttcccctgataagggaaaggtca
ctagttcgaatctagttgagacaacca
1-7tRNA-Ser(tga)[62529,62614]
ggaagattccgctcagtggtgggcaatccggtttgaacccggaggtgctg
gtaacggtaggggttcgactcctcaatcttccgcca
1-8tRNA-Asn(gtt)[62620,62695]
gatgggatagctaactcggtaaagcagcagactgttaatctgtgatatta
gggttcaaatccctatcccatcgcca
1-9tRNA-Arg(tct)[63027,63103]
gcatccttagctcagttggatagagcagtagccttctaagctattggtca
ctggttcgaatccagtagggtgtacca
1-10tRNA-Leu(taa)[63106,63191]
gggagtatggtgaaatcggtagacacaagggatttaaaatccctcggctt
atagctgtacgagttcgagtctcgttactcccacca
>KC821608.1 Cellulophaga phage phi19:3, complete genome
1-1tRNA-Cys(gca)c[67343,67415]
gttgttgtactcaagtggttaagaggttagtctgcaaaactaatatcgtg
agttcgaatctcaccaacaactc
>KC821614.1 Cellulophaga phage phi38:1, complete genome
1-2tRNA-Glu(ttc)c[28604,28676]
gctcttttcgtctagcggtaaggacattcggttttcatccgaaaaacata
ggttcgaatcctatagagagtac
1-3tRNA-Glu(ttc)c[30574,30646]
gctcttttcgtctagcggtaaggacattcggttttcatccgaaaaacata
ggttcgaatcctatagagagtac
1-4tRNA-Lys(ttt)[35522,35595]
gcccttgtagcaaacaggttgtaggcaaccgtcttttaaacggtggggct
aggttcgattcctagcaggggcac
1-5tRNA-Ile(gat)[35644,35717]
tggagtatagctcagcggttagagcactcccctgatacgggagatgtcgt
aggttcgaaaccttctactccaac
1-6tRNA-Gly(tcc)[35805,35878]

gcgtaagaggccatagtggaatggcgcctgccttccaagtaggagatagc
gagttcgaacctcgtcttacgctc
1-7tRNA-Met(cat)[35888,35957]
tgtgggtggtcgtggtgaccagagggcctcataagtccttatagatgggt
tcgactcccatacccgccac
1-8tRNA-Asp(gtc)[36032,36109]
ggtctaataagcgtgtttggtaacacgaattgcactgtcactgctctaaa
acgagggttcgaatccctcttagaccgc
1-9tRNA-Thr(tgt)[36119,36193]
gcactaatgactgaataggaaatggtacttcatttgtaacgaagataatt
cgggttcgactcctgattagtgctc
1-10tRNA-Val(tac)[36402,36476]
accagattagctcagcggtttagagcagctcttttacaaggagaaggtcg
atagttcgaatctatcatttggtac
1-11tRNA-Arg(tct)[36537,36608]
acctctatagtttaaaggaagaatacggcgcttctaacgctatggtcggg
gttcgaatccctgtagaggttc
1-12tRNA-Ser(tga)[37020,37110]
ggaagagtaagccaagtaggtctactggtcgctgtcttgaaaacagttgg
ggtttaaaagcccgtgtgggttcgagccccacttcttccgc
1-13tRNA-Ser(gct)[37115,37200]
agagaggtgtctgagtggcttaaggtactactttgctaaagtagtggggt
taacgcctccgagggttcgaatcccttcttctctac
1-14tRNA-Asn(gtt)[37209,37281]
ggctaggtagctcagaggaagagcaggcgcctgttaagcgtcaggtcgag
atatcgtaattctccctagcctc
1-15tRNA-Phe(gaa)[37440,37513]
gcgttagaagctaacttggtagaagcactggcttgaaatcccagaggagt
aggttcgattcctacctatcgcac
1-16tRNA-Pro(tgg)[38141,38213]
cgagtagtgacaaaattggcacgtgccgaccttggacgtcggagaatgca
ggttcgatccctgcctactcgac
1-17tRNA-Glu(ttc)[38222,38297]
gctatcgtcttctaaatggaataggaatccaccctttcaaggtgtgcaat
gcgagttcaagtctcgtcgatagtac
1-18tRNA-Gln(ttg)[38343,38415]
tggagtatgatgtaacggttagcatgactggttttgagccagttcgtcgg
ggttcgaatccctgtactccatc
1-19tRNA-His(gtg)[38426,38502]
cctccgttgcgccaaattggaaaggcggtcaagctgtgaacttgattaca
tgggttcgattcccatacggttgtcca
1-20tRNA-Leu(taa)[38631,38714]
gcatccatggtggaaagggctatacactacaggcttaaaacctgtcgccg
aaaggattgggggttcgaatccctctggatgcac

1–21tRNA–Arg(acg)[38721,38793]
acttctatagcttaacggaaaagcgttccgctacgaacggatagattggg
ggttcgaatccctctagaagttc
>KC821632.1 Cellulophaga phage phi4:1, complete genome
1–1tRNA–Pro(tgg)[87196,87272]
cgggtaatagaggagtctggtttatctcgctgcatttgggatgcagagca
cgcaggttcgaatcctgcttatccgac
1–2tRNA–Glu(ttc)[87352,87424]
gctgccatcgtctaacggctaggacctcaggttttcatcctggtaatcgg
agttcgattctccgtggcagtac
1–3tRNA–Gln(ttg)[87495,87568]
tggaatatggtgtaattggtaacacgtctggttttggtccagaagagtgg
gggttcgagaccctctattccaac
1–4tRNA–His(gtg)[87570,87644]
gtgttagaagctaaagtggtcgaagcgtctggttgtggtccagaagatag
tgagttcgagtctcatttaacaccc
1–5tRNA–Leu(tag)[87703,87778]
gcagaggtgggtaaactggtaaagccgcccgatttaggatcgggagttct
gagggttcgactccctctctctgtac
1–6tRNA–Leu(taa)[87785,87866]
gccccaatggtgaaactggttatacacgtagcacttaaaatgctatgctt
aggcttgcaggttcgatccctgcttggggtac
1–7tRNA–Arg(acg)[87871,87944]
tttctcatagtttaactggaaaaacacagagttacggcctttgagagtag
gggttcgagtccctttgagaaaac
1–8tRNA–Ala(tgc)[88170,88243]
ggcccagtagctaaactggcttagcacctcctttgcaaggagaagtatgt
gggttcgagccccatctggcccac
1–9tRNA–Lys(ttt)[88325,88397]
accctcgtagcttaatggcaaagctcctctcttttaaagaggcgaccgca
agatcggtacttgtcgggggtac
1–10tRNA–Ile(gat)[88687,88761]
aggaatatagctcagttggttagagtacttcgctgataccgaagatgtca
caagttcgaatcttgttattcctac
1–11tRNA–Gly(tcc)[88865,88939]
gcagcaatcgcatagcggcctattgcacctgacttccaatcaggatctcg
tgagttcgaatctcacttgctgcac
1–12tRNA–Met(cat)[88944,89018]
agcgaggtagagcagctggttagctcgctaggctcataacctagaggtcg
ggagttcgagtctccccttcgccac
1–13tRNA–Asp(gtc)[89097,89170]
agtctatcggtccagtggagtggacgctgccctgtcacggcagagatcac
gggttcgagtcccgtatagactgc
1–14tRNA–Thr(tgt)[89249,89323]

tccctacgtagctcaattggtggagcactggttttgtaaaccaaaggttg
tcggttcgagtccgacctagggatc
1-15tRNA-Val(tac)[89470,89544]
accagattagctcaggggtttagagcaactcttttacaaggagaaggtcg
atagttcgaatctatcatttggtac
1-16tRNA-Arg(tct)[89555,89628]
gggggattagtttaactggaaaaactataggcttctatcctatcgagtag
gggttcgaatcccttatccccttc
1-17tRNA-Met(cat)[89638,89712]
ggccttgtagctcagttggttagagcagctcactcataatgagaaggtct
caggttcgagtcctgactaggccac
1-19tRNA-Tyr(gta)[89926,90010]
gggacggttgctcggctggaagaggcgcaggactgtaaattctgtaatcc
actaggaatggaggttcgagtcctcctcgtcccac
1-20tRNA-Ser(gct)[90280,90367]
agaaaggtgtcagagtggcttattgagctaggttgctaacttagtggagt
gaaataccttccgtgggttcgaatcccatcctttcttc
1-21tRNA-Asn(gtt)[90397,90485]
tcttgagttgccgtagttggccgaacggtccagactgttaatctggtgag
atttatctccatcgtaggttcgaatcctacctcaagagc
1-22tRNA-Trp(cca)[90629,90702]
acttgagtagctcagttggtagagcagtggtctccaaaaccaaaggtcgt
aggttcgagtcctacctctcgtgc
1-23tRNA-Phe(gaa)[90724,90798]
ggggttgtagctgagttggtacaagcgtccgattgaagctcggaagtacg
aagggtcgaagccttccttccccac
1-24tRNA-Ile(gat)[143233,143308]
aatcgggtagttcaattggtagaacatcacactgataatgtgaaggttgt
gggttcgagtcccatcccgattacca
>KF856712.1 Pseudomonas phage phiIBB-PAA2, complete genome
1-1tRNA-Pro(tgg)c[44350,44426]
ctcctcgtagctcagttggttagagtgcctgtcttggacacaggaggtcg
taggttcgagtcctaccggggtgacca
1-2tRNA-Asp(gtc)c[44431,44506]
ggcccattaactcagcggttagagtgctcccctgtctagggagaagccgt
cggttcgaatccgacatgggtcgcca
1-3tRNA-Ile(gat)c[44511,44586]
tgggatgtagctcagttggtagagcagggagccgataactctcaggtcgt
aggttcgagccctaccgtcccaacca
>HG518155.1 Pseudomonas phage TL complete genome
1-1tRNA-Pro(tgg)c[44641,44717]
ctcctcgtagctcagttggttagagtgcctgtcttggacacaggaggtcg
taggttcgagtcctaccggggtgacca
1-2tRNA-Tyr(gta)c[44722,44808]

ggtcgttgtagaggtagatggctactaccatccggttgtaacccggacgc
cttaggctagaggttcaattccttccttcgaccacca
1−3tRNA−Asn(gtt)c[44814,44889]
tgggatgtagctcagttggtagagcagggagctgttaactctcaggtcgt
aggttcgagccctaccatcccagcca
>KF787095.1 Achromobacter phage JWAlpha, complete genome
1−1tRNA−Arg(tct)[18757,18852]
ggcgtagtggtggaattggtaaacacagagtccttctccggactccgagc
gtagccgcgaggtgctcttgcaggttcgactcctgcctacaatgcc
>KF981730.1 Escherichia phage KBNP1711, complete genome
1−1tRNA−Arg(tct)c[66381,66455]
gtccctatagttaaaaggatataacagatttcttctaaaaatccattcta
ggttcgagtcctagtggggacgcca
>KF806588.1 Erwinia phage Ea9−2, complete genome
1−1tRNA−Asn(gtt)[41009,41084]
gacgggttagttcagcaggtagaacggtggactgttaatccatatgtcat
cggttcgaatccggtactcgtcgcca
1−2tRNA−Glu(ttc)[41091,41167]
attcccgtagacaaattggtatagtcaccacactttcactgtgggatctg
tgggttcaagtcccatcgggaatgcca
1−3tRNA−Asp(gtc)[41174,41249]
agtcctatagttcagtcggtagaatacctccctgtcacggagaaggtcac
gggttcgagtcccgttaggactgcca
1−4tRNA−Ile(gat)[41253,41328]
tgcttcatagctcagttggtagagcaaccgcccgataagcggtaggtcac
tggttcaagtccagttgaagcaacca
1−5tRNA−Tyr(gta)[41504,41585]
gggggattagtcaagcggtcgaagacggcagactgtaaatctgttatccc
acacggtagttcgaatctaccatcccccacca
1−6tRNA−Ser(gct)[41689,41780]
ggaagattggctgagtggctaaaagcgtccggttgctaaccggaaggcgg
cgtaacagcttcccactggttcgaatccagtatcttcctcca
1−7tRNA−Lys(ttt)[41786,41862]
aggtcgttagctcagttggttagagcaccggacttttaatccggttgtca
ttggttcgaggccaatacgacctacca
>KJ192399.2 Vibrio phage CHOED, complete genome
1−1tRNA−Asn(gtt)[55857,55933]
ggataagaagcataagtggtatatgcgctcgcctgttaagcgaaagatag
agagttcgaacctctccttgtccgcca
1−2tRNA−Ser(gct)[55939,56028]
agagaagtgcccgagtggtttaaagggcctccctgctaaggaggtgggcg
ttaatccgctccgtgggttcaaatcccactttctctgcca
1−3tRNA−Pro(tgg)[56641,56717]
cgggatgtaatgtcaattggtagacggctcgctttggaagcgagaggttg

aaggttcgagtccttccatcccgacca

>KF192075.1 Escherichia phage vB_EcoP_PhAPEC5, complete genome

1-1tRNA-Ile(aat)[34700,34778]

tgtccgcttagcttagactgggaaagcaaccgactaataatcggaaggtc

actggttcaaatccagtagtggacaccca

>KJ135004.2 Enterobacteria phage Bp4, complete genome

1-1tRNA-Ile(aat)c[37009,37083]

gcccacttagcttagacggggaaagcaaccgactaataatcggaaggtca

ctggttcaaatccagtagtgggtac

1-2tRNA-Cys(aca)c[37785,37863]

gtgtgtgatagctcaaataggtagagcgtgaggctacagtgctgaatggt

ttgtgggttcgattcccacccatgcctac

1-3tRNA-Ala(cgc)c[38155,38226]

ggtcgaggtagcacgtaggcatgtgcagatggtcgctacttctctcgctg

gttcgaatccagcccccgacct

>KJ621082.2 Dinoroseobacter phage DFL12phi1, complete genome

1-1tRNA-Pro(tgg)c[59148,59225]

cggaatgtaggctagtctggttaagtcgctcggtttgggaccgagaaatc

gaaggttcgaatccttccattccgacca

1-2tRNA-Ile(gat)c[59229,59304]

tgtggtgtagctcagttggtagagcgttcgaccgataatcgaaatgtcgc

tggttcaagtccagccaccacaacca

>KF562340.1 Escherichia phage vB_EcoP_PhAPEC7, complete genome

1-1tRNA-Ile(aat)[35085,35163]

tgtccgcttagcttagactgggaaagcaaccgactaataatcggaaggtc

actggttcaaatccagtagtggacaccca

>KM199771.1 Mesorhizobium phagevB_MloP_Lo5R7ANS, complete genome

1-1tRNA-Leu(tag)[8569,8654]

gcgggtatggtggaattggtagacacgcttggtttaggtccaagtgcctg

aaaagcttgggggttcgagtccctctacccgcacca

>LN610578.1 Pseudomonas phage vB_PaeP_C2-10_Ab22, complete genome

1-1tRNA-Pro(tgg)c[44812,44888]

ctcctcgtagctcagttggttagagtgcctgtcttggacacaggaggtcg

taggttcgagtcctaccggggtgacca

1-2tRNA-Tyr(gta)c[44893,44979]

ggtcgttgtagaggtagatggctactaccatccggctgtaacccggacgc

cattggctagaggttcaattccttccttcgaccacca

1-3tRNA-Asn(gtt)c[44985,45060]

tgggatgtagctcagttggtagagcagggagctgttaactctcaggtcgc

aggttcgagccctgccgtcccagcca

>KM236242.1 Escherichia phage Pollock, complete genome

1-1tRNA-Pro(tgg)[64673,64749]

cggaatgtagcacagccgggtagtgcatctgctttgggagcagagggtca

taggttcaaatcctatcattccgacca

1−2tRNA−Met(cat)[64756,64832]
tcccgtttagcttaattggttaaagcacccgactcataatcggatgatta
caggttcgaatcctgtcacgggaacca
1−3tRNA−Arg(tct)[64837,64912]
gcatccttagctcaatggatagagcagtagccttctaagctattggttac
aggttcgaatcctgtaggatgcacca
1−4tRNA−Leu(taa)[64918,65002]
gggagtatggtgaaattggtaaacacaagggatttaaaatccctcgctta
attgcttacgagttcgagtctcgttactcccacca
>KM044272.1 Phage vB_EcoP_SU10, complete genome
1−1tRNA−Arg(tct)c[66656,66731]
gcgctcttagctcaatggatagagcagtagccttctaagctattggttac
aggttcgagtcctgtagggcgcacca
>HM208303.1 Stx2 converting phage vB_EcoP_24B, complete genome
1−1tRNA−Met(cat)[21679,21754]
ggccctttagctcagtggtgagagcgagcgactcataatcgccaggtcgc
tggttcaaatccagcaagggccacca
1−2tRNA−Arg(tcg)[21764,21840]
ccgccattagctcatcgggacagagcgccagccttcgaagctggctgcgc
ggggttcgagtcctcgatggcggtcca
1−3tRNA−Arg(tct)[21854,21930]
gcgttgttagctcagccggacagagcaattgccttctgagcaatcggtca
ctggttcgaatccagtacaacgcgcca
>KP682371.1 Escherichia phage PA2, complete genome
1−1tRNA−Met(cat)[27123,27198]
ggccctttagctcagtggtgagagcgagcgactcataatcgccaggtcgc
tggttcaaatccagcaagggccacca
1−2tRNA−Arg(tcg)[27208,27284]
ccgccattagctcatcgggacagagcgccagccttcgaagctggctgcgc
ggggttcgagtcctcgatggcggtcca
1−3tRNA−Arg(tct)[27298,27374]
gcgttgttagctcagccggacagagcaattgccttctgagcaatcggtca
ctggttcgaatccagtacaacgcgcca
>KR063278.1 Gordonia phage GMA7, complete genome
1−1tRNA−Asn(gtt)[13362,13435]
tgtgatatagctcaattggcagagcgctggactgttaatccggtagttga
aggttcgagtccttctatcacagc
>KT206225.1 Mycobacterium phage Phlei, complete genome
1−1tRNA−Asn(gtt)[5706,5781]
tgatctgtagctcaatcggcagagcggctccctgttaaggagttggttgg
aggttcgagtcctcccagatcagcca
1−2tRNA−Trp(cca)[5819,5892]
gcgttcctagctcaattggtagagcgacggtctccaaagccgttggttcc
aggttcgagtcctggggggcgcgc

1−3tRNA−Gln(ctg)[5945,6017]
tgctcattcgtctaaccggcaagacaccgggttctggccccggcaatcca
ggttcaaatcctgggtgagcagc
>KF971864.1 Escherichia phage phi191, complete genome
1−1tRNA−Arg(tct)c[10036,10112]
gcgttgttagctcagccggacagagcaattgccttctgagcaatcggtca
ctggttcgaatccagtacaacgcgcca
1−2tRNA−Arg(tcg)c[10126,10203]
ccgccattagctcatcgggacagagcgccagccttcgaagctggctgcgc
ggggttcgagtcctcggatggcggtcca
1−3tRNA−Met(cat)c[10213,10288]
ggcccctttagctcagtggtgagagcgagcgactcataatcgccaggtcgc
tggttcaaatccagcaagggccacca
>HQ424691.1 Enterobacteria phage VT2phi_272, complete sequence
1−1tRNA−Arg(cct)c[1320,1394]
gtcctcttagttaaatggatataacgagcccctcctaagggctaattgca
ggttcgattcctgcaggggacacca
1−2tRNA−Met(cat)[27183,27258]
ggcccctttagctcagtggtgagagcgagcgactcataatcgccaggtcgc
tggttcaaatccagcaagggccacca
1−3tRNA−Arg(tcg)[27268,27344]
ccgccattagctcatcgggacagagcgccagccttcgaagctggctgcgc
ggggttcgagtcctcgatggcggtcca
1−4tRNA−Arg(tct)[27358,27434]
gcgttgttagctcagccggacagagcaattgccttctgagcaatcggtca
ctggttcgaatccagtacaacgcgcca
>KT630648.2 Salmonella phage SEN22, complete genome
1−1tRNA−Asn(gtt)[37726,37800]
gggtcagtcgtataaaggttattacggaaggctgttaaccttcttatcgt
ggttcgagtccacgctgtcccgcca
1−2tRNA−Thr(tgt)[37806,37881]
gctggtttagctccaatggtagagcagtcgccttgtaagcgaatgggtag
cggttcaagtccgttaaccagcacca
>KR054029.1 Pseudomonas phage DL54, complete genome
1−1tRNA−Ile(gat)[888,963]
tgggatgtagctcagttggtagagcagggagccgataactctcaggtcgt
aggttcgagccctaccgtcccaacca
1−2tRNA−Asp(gtc)[968,1043]
ggcccattaactcagcggttagagtgctcccctgtctagggagaagccgt
cggttcgaatccgacatgggtcgcca
1−3tRNA−Pro(tgg)[1048,1124]
ctcctcgtagctcagttggttagagtgcctgtcttggacacaggaggtcg
taggttcgagtcctaccggggtgacca
>KP233880.1 Pseudomonas phage PhiCHU, complete genome

1−1tRNA−Pro(tgg)c[44654,44729]
ctcctcgtagctcagtctggttagagtgcctgccttggaagcaggaggtc
gtaggttcgaatcctaccggggtgac
1−2tRNA−Asp(gtc)c[44734,44809]
ggcccattaactcagcggtaagagtgctcccctgtctagggagaagtctg
gggttcgaatccccaatgggtcgcca
1−3tRNA−Asn(gtt)c[44814,44889]
tgggatgtagctcagttggtagagcaggtagctgttaactatcaggtcgt
aggttcgagccctaccgtcccagcca
>KP308307.1 Escherichia phage 172−1, complete genome
1−1tRNA−Arg(tct)c[75402,75478]
gcgccgttagctcaattgtatagagcagtttccttctaagaaattggttg
gaggttagaatcctccacggtgcgcca
>KU878967.1 Salmonella phage 118970_sal4, complete genome
1−1tRNA−Asn(gtt)[20486,20560]
gggtcagtcgtataaaggtcattacggaaggctgttaaccttcttatcgt
ggttcgagtccacgctgtccagcca
1−2tRNA−Thr(tgt)[20566,20641]
gctggtttagctccaatggtagagcagtcgccttgtaagcgaatgggtag
cggttcaagtccgttaaccagcacca
>LT594300.1 Escherichia phage LM33_P1 genome assembly, chromosome: I
1−1tRNA−Thr(cgt)[11104,11182]
gtctccatagctcaaacggtagagcggcgggtcgtgatggcctattgcga
ttagggttcgaatccccgtggagacgcca
>KU927494.1 Salmonella Phage 103203_sal5, complete genome
1−1tRNA−Asn(gtt)[40369,40442]
tgggtcagtcgtataaaggtcattacggaaggctgttaaccttcttatcg
tggttcgagtccacgctgtccaga
>KX098389.2 Erwinia phage vB_EamP_Frozen, complete genome
1−1tRNA−Asn(gtt)[40868,40943]
gacgggttagctcagttggtagagcgattgactgttaatcaattggtcat
cggttcaaatccggtactcgtcgcca
1−2tRNA−Glu(ttc)[40950,41026]
attcccgtagacaaattggtacagtcaccacactttcactgtggagtttg
tgggttcaagtcccatcgggaatgcca
1−3tRNA−Asp(gtc)[41033,41108]
agtcctatagttcagtcggtagaatacctccctgtcacggagaaggtcac
gggttcgagtcccgttaggactgcca
1−4tRNA−Ile(gat)[41112,41187]
tgcttcatagctcagtgggtagagcaaccgcccgataagcggtaggtcac
tggttcaagtccagttgaagcaacca
1−5tRNA−Tyr(gta)[41446,41527]
gggggattagtcaagcggtcgaagacggcagactgtaaatctgttatccc
acacggtagttcgaatctatcatcccccacca

1-6tRNA-Pro(tgg)[41532,41608]
cggcatttagctcagtttggcagagcatcggctttgggagtcgagggtcg
aaggttcaaatccttcagtgccgacca
1-7tRNA-Ser(gct)[41614,41705]
ggaagattggctgagtggctaaaggcgtccggttgctaaccggaaggcgg
tgtaacagcttcccactggttcgaatccagtatcttcctcca
1-8tRNA-Lys(ttt)[41711,41787]
aggtcgttagctcagttggttagagcaccggacttttaatccggatgtca
ttggttcgaggccaatacgacctacca
>AP005154.1 Stx2 converting phage II DNA, complete genome
1-1tRNA-Met(cat)[52499,52574]
ggcccctttagctcagtggtgagagcgagcgactcataatcgccaggtcgc
tggttcaaatccagcaagggccacca
1-2tRNA-Arg(tcg)[52584,52660]
ccgccattagctcatcgggacagagcgccagccttcgaagctggctgcgc
ggggttcgagtcctcgatggcggtcca
1-3tRNA-Arg(tct)[52674,52750]
gcgttgttagctcagccggacagagcaattgccttctgagcaatcggtca
ctggttcgaatccagtacaacgcgcca
>AP013406.1 Uncultured Mediterranean phage uvMED DNA, complete genome, group G15, isolate: uvMED-CGR-U-MedDCM-OCT-S46-C34
1-1tRNA-Pro(tgg)c[25878,25954]
ctgcgtgtggcggagcctggtaccgcatctgctttgggagcagaggatcg
ctggttcaaatccagccacgcagacca
>AP013543.1 Uncultured Mediterranean phage uvMED DNA, complete genome, group G8, isolate: uvMED-CGR-U-MedDCM-OCT-S30-C28
1-1tRNA-Leu(tag)c[37322,37406]
ggggcgatggtggaattggtagacacgccagtcttaggaactggtctttt
acgaggtgaaggttcgagtccttttcgccctacca
>AP013396.1 Uncultured Mediterranean phage uvMED DNA, complete genome, group G15, isolate: uvMED-CGR-C99-MedDCM-OCT-S44-C25
1-1tRNA-Pro(tgg)c[24212,24288]
ctgggtgtagcgaagcctggtatcgcactagctttgggagctagggatcg
ttggttcaaatccaaccacccagacca
>AP013400.1 Uncultured Mediterranean phage uvMED DNA, complete genome, group G15, isolate: uvMED-CGR-U-MedDCM-OCT-S30-C37
1-1tRNA-Pro(tgg)[32296,32372]
ctgggtgtagcgaagcctggtatcgcacctggtttgggaccaggggatcg
gaggttcaaatcctcccacccagacca
>AP004402.1 Stx2 converting phage I DNA, complete genome
1-1tRNA-Met(cat)[52865,52940]
ggcccctttagctcagtggtgagagcgagcgactcataatcgccaggtcgc
tggttcaaatccagcaagggccacca
1-2tRNA-Arg(tcg)[52950,53026]

ccgccattagctcatcgggacagagcgccagccttcgaagctggctgcgc
ggggttcgagtcctcgatggcggtcca
1−3tRNA−Arg(tct)[53040,53116]
gcgttgttagctcagccggacagagcaattgccttctgagcaatcggtca
ctggttcgaatccagtacaacgcgcca
>HF569091.1 Brucella phage Tb complete sequence, isolate BfR
1−1tRNA−Leu(cag)c[8179,8254]
gcgagcaggaggctcaggacggtcacggaactcagacttccgttcggtct
gctgatccttaacctgctcgcttgcg
>KC556897.1 Brucella phage Tb, complete genome
1−1tRNA−Leu(cag)c[10121,10196]
gcgagcaggaggctcaggacggtcacggaactcagacttccgttcggtct
gctgatccttaacctgctcgcttgcg
>KC438283.1 Vibrio phage VCO139, complete genome
1−1tRNA−Arg(tct)[13346,13422]
gtcctgttagctcaattggatagagcaagatgtttctaccgtctaggtta
taggttcgaatcctatacgggatgcca
>KT962247.1 Cellulophaga phage phi17:2_18, complete genome
1−1tRNA−Pro(tgg)[87649,87725]
cgggtaatagaggagtctggtttatctcgctgcatttgggatgcagagca
cgcaggttcgaatcctgcttatccgac
1−2tRNA−Glu(ttc)[87805,87877]
gctgccatcgtctaacggctaggacctcaggttttcatcctggtaatcgg
agttcgattctccgtggcagtac
1−3tRNA−Gln(ttg)[87948,88021]
tggaatatggtgtaattggtaacacgtctggttttggtccagaagagtgg
gggttcgagaccctctattccaac
1−4tRNA−His(gtg)[88023,88097]
gtgttagaagctaaagtggtcgaagcgtctggttgtggtccagaagatag
tgagttcgagtctcatttaacaccc
1−5tRNA−Leu(tag)[88156,88231]
gcagaggtgggtaaactggtaaagccgcccgatttaggctcgggagttct
gagggttcgagtccctctctctgtac
1−6tRNA−Leu(taa)[88238,88319]
gccccaatggtgaaactggttatacacgtagcacttaaaatgctatgctt
aggcttgcaggttcgatccctgcttggagtac
1−7tRNA−Arg(acg)[88324,88397]
tttctcatagtttaactggaaaaacacaaagttacggcctttgagagtag
gggttcgagtccctttgagaaaac
1−8tRNA−Ala(tgc)[88623,88696]
ggcccagtagctaaactggcttagcacctcctttgcaaggagaagtatgt
gggttcgagccccatctggcccac
1−9tRNA−Lys(ttt)[88778,88850]
accctcgtagcttaatggcaaagctcctctcttttaaagaggcgaccgca

agatcagtacttgtcgggggtac
1–10tRNA–Ile(gat)[88953,89027]
aggaatatagctcagttggttagagtacttcgctgataccgaagatgtca
caagttcgaatcttgttattcctac
1–11tRNA–Gly(tcc)[89131,89205]
gcagcaatcgcatagcggcctattgcacctgacttccaatcaggatctcg
tgagttcgaatctcacttgctgcac
1–12tRNA–Met(cat)[89210,89284]
agcgaggtagagcagctggttagctcgctaggctcataacctagaggtcg
ggagttcgagtctccccttcgccac
1–13tRNA–Asp(gtc)[89363,89436]
agtctatcggtccagtggagtggacgctgccctgtcacggcagagatcac
gggttcgagtcccgtatagactgc
1–14tRNA–Thr(tgt)[89515,89589]
tccctacgtagctcaattggtggagcactggttttgtaaaccaaaggttg
tcggttcgagtccgacctagggatc
1–15tRNA–Val(tac)[89736,89810]
accagattagctcaggggtttagagcaactcttttacaaggagaaggtcg
atagttcgaatctatcatttggtac
1–16tRNA–Arg(tct)[89821,89894]
gggggattagtttaactggaaaaactataggcttctatcctatcgagtag
gggttcgaatcccttatccccttc
1–17tRNA–Met(cat)[89904,89978]
ggccttgtagctcagttggttagagcagctcactcataatgagaaggtct
caggttcgagtcctgactaggccac
1–18tRNA–Tyr(gta)[90192,90276]
gggacggttgctcggctggaagaggcgcaggactgtaaattctgtaatcc
actaggaatggaggttcgagtcctcctcgtcccac
1–19tRNA–Ser(gct)[90546,90633]
agaaaggtgtcagagtggtttattgagctaggttgctaacttagtggagt
gaaataccttccgtgggttcgaatcccatcctttcttc
1–20tRNA–Asn(gtt)[90675,90747]
gacagggtagctcagtggtagagcagggagctgttaactctcaggtcgtg
ggttcgaatcccactcttgtctc
1–21tRNA–Trp(cca)[90809,90882]
acttgagtagctcagttggtagagcagtggtctccaaaaccaaaggtcgt
aggttcgagtcctacctctcgtgc
1–22tRNA–Phe(gaa)[90964,91038]
ggggttgtagctgagttggtacaagcgtccgattgaagctcggaagtacg
aagggtcgaagccttccttccccac
1–23tRNA–Ile(gat)[142711,142786]
aatcgggtagttcaattggtagaacatcacactgataatgtgaaggttgt
gggttcgagtcccatcccgattacca
>KC821612.1 Cellulophaga phage phi40:1, complete genome

1-2tRNA-Glu(ttc)c[28604,28676]
gctcttttcgtctagcggtaaggacattcggttttcatccgaaaaacata
ggttcgaatcctatagagagtac
1-3tRNA-Glu(ttc)c[30574,30646]
gctcttttcgtctagcggtaaggacattcggttttcatccgaaaaacata
ggttcgaatcctatagagagtac
1-4tRNA-Lys(ttt)[35520,35593]
gcccttgtagcaaacaggttgtaggcaaccgtcttttaaacggtggggct
aggttcgattcctagcaggggcac
1-5tRNA-Ile(gat)[35642,35715]
tggagtatagctcagcggttagagcactcccctgatacgggagatgtcgt
aggttcgaaaccttctactccaac
1-6tRNA-Gly(tcc)[35803,35876]
gcgtaagaggccatagtggaatggcgcctgccttccaagtaggagatagc
gagttcgaacctcgtcttacgctc
1-7tRNA-Met(cat)[35886,35955]
tgtgggtggtcgtggtgaccagagggcctcataagtccttatagatgggt
tcgactcccatacccgccac
1-8tRNA-Asp(gtc)[36030,36107]
ggtctaataagcgtgtttggtaacacgaattgcactgtcactgctctaaa
acgagggttcgaatccctcttagaccgc
1-9tRNA-Thr(tgt)[36117,36191]
gcactaatgactgaataggaaatggtacttcatttgtaacgaagataatt
cgggttcgactcctgattagtgctc
1-10tRNA-Val(tac)[36400,36474]
accagattagctcagcggtttagagcagctcttttacaaggagaaggtcg
atagttcgaatctatcatttggtac
1-11tRNA-Arg(tct)[36535,36606]
acctctatagtttaaaggaagaatacggcgcttctaacgctatggtcggg
gttcgaatccctgtagaggttc
1-12tRNA-Ser(tga)[37017,37107]
ggaagagtaagccaagtaggtctactggtcgctgtcttgaaaacagttgg
ggtttaaaagcccgtgtgggttcgagccccacttcttccgc
1-13tRNA-Ser(gct)[37112,37197]
agagaggtgtctgagtggcttaaggtactactttgctaaagtagtggggt
taacgcctccgagggttcgaatcccttcttctctac
1-14tRNA-Asn(gtt)[37206,37278]
ggctaggtagctcagaggaagagcaggcgcctgttaagcgtcaggtcgag
atatcgtaattctccctagcctc
1-15tRNA-Phe(gaa)[37437,37510]
gcgttagaagctaacttggtagaagcactggcttgaaatcccagaggagt
aggttcgattcctacctatcgcac
1-16tRNA-Pro(tgg)[38137,38209]
cgagtagtgacaaaattggcacgtgccgaccttggacgtcggagaatgca

ggttcgatccctgcctactcgac
1-17tRNA-Glu(ttc)[38218,38293]
gctatcgtcttctaaatggaataggaatccacccttcaaggtgtgcaat
gcgagttcaagtctcgtcgatagtac
1-18tRNA-Gln(ttg)[38339,38411]
tggagtatgatgtaacggttagcatgactggttttgagccagttcgtcgg
ggttcgaatccctgtactccatc
1-19tRNA-His(gtg)[38422,38498]
cctccgttgcgccaaattggaaaggcggtcaagctgtgaacttgattaca
tgggttcgattcccatacggttgtcca
1-20tRNA-Leu(taa)[38627,38710]
gcatccatggtggaaagggctatacactacaggcttaaaacctgtcgccg
aaaggattgggggttcgaatccctctggatgcac
1-21tRNA-Arg(acg)[38717,38789]
acttctatagcttaacggaaaagcgttccgctacgaacggatagattggg
ggttcgaatccctctagaagttc
>KJ803031.1 Dinoroseobacter phage vBDshPR2C, complete genome
1-1tRNA-Ile(gat)[14807,14882]
tgtggtgtagctcagttggtagagcgaacgaccgataatcgttatgtcgc
tggttcgagtccaaccaccacaacca
1-2tRNA-Pro(tgg)[14886,14962]
cggaatgtaggctagtctggtaagtcgctcggtttgggtccgagagatcg
taggttcgaatcctgccattccgacca
>KP869108.1 Escherichia coli O157 typing phage 10, complete genome
1-1tRNA-Arg(tct)[30924,31000]
gtggcattagctcagttggacagagcaaccgccttctaagcggttggtcg
caggttcgaatcctgcatgccacgcca
>KU197013.1 Xanthomonas phage XAJ24, complete genome
1-1tRNA-Trp(cca)[962,1036]
gtctcggtagtgtaatggcaacataccggcctccaaagccgtgagattag
ggttcgagtccctaccgattcgcca
>KU687351.1 Citrobacter phage SH5, complete genome
1-1tRNA-Ser(tga)[11049,11127]
gtctccatagctcaaacggtagagcggcgggtcgtgatgacctattgcga
ttagggttcgaatccccgtggagacgcca
>KY065148.1 Vibrio phage JSF3, complete genome
1-1tRNA-Arg(tct)c[54903,54979]
gtcctgttagctcaattggatagagcaagatgtttctaccgtctaggtta
taggttcgaatcctatacgggatgcca
>KY626177.1 Marinomonas phage CPG1g, complete genome
1-1tRNA-Arg(cct)[15657,15732]
ctcagtattagctcagtaggatagagcgattgcctcctaagcaataggta
actggttcgagtccagtatactgaga
>KY626176.1 Marinomonas phage CPP1m, complete genome

1−1tRNA−Arg(cct)[15658,15733]
ctcagtattagctcagtaggatagagcgattgcctcctaagcaataggta
actggttcgagtccagtatactgaga
>KY962008.1 Escherichia phage ST31, complete genome
1−1tRNA−Thr(cgt)[10055,10133]
gtctccatagctcaaacggtagagcggcgggtcgtgatggcctattgcga
ttagggttcgaatccccgtggagacgcca
>MF481197.1 Marinomonas phage CB5A, complete genome
1−1tRNA−Arg(cct)[16734,16809]
ctcagtattagctcagcaggatagagcgattgcctcctaagcaataggtc
actggttcgagtccagtatactgaga
>HF569089.1 Brucella phage Fi complete sequence
1−1tRNA−Leu(cag)c[8178,8253]
gcgagcaggaggctcaggacggtcacggaactcagacttccgttcggtct
gctgatccttaacctgctcgcttgcg
>JN254801.1 Pseudomonas phage MR299−2, complete genome
1−1tRNA−Asn(gtt)[1605,1680]
tgggatgtagctcagttggtagagcagatagctgttaactatccggtcgt
aggttcgagccctaccatcccagcca
1−2tRNA−Asp(gtc)[1685,1760]
ggcccattaactcagcggtaagagtgctcccctgtctagggagaagtctg
gggttcgaatccccaatgggtcgcca
1−3tRNA−Pro(tgg)[1765,1840]
ctcctcgtagctcagtctggttagagtgcctgccttggaagcaggaggtc
gtaggttcgaatcctaccggggtgac
>KF005320.1 Alteromonas phage vB_AmaP_AD45−P2, complete genome
1−1tRNA−Met(cat)[53590,53665]
gcccctatagctcagcgtgtagagcagacgactcataatcgttaggtcac
tggttcaaatccggttaggggcacca
>KC556894.1 Brucella phage Fz, complete genome
1−1tRNA−Leu(cag)c[10121,10196]
gcgagcaggaggctcaggacggtcacggaactcagacttccgttcggtct
gctgatccttaacctgctcgcttgcg
>KT962245.1 Cellulophaga phage phi4:1_13, complete genome
1−1tRNA−Pro(tgg)[87196,87272]
cgggtaatagaggagtctggtttatctcgctgcatttgggatgcagagca
cgcaggttcgaatcctgcttatccgac
1−2tRNA−Glu(ttc)[87352,87424]
gctgccatcgtctaacggctaggacctcaggttttcatcctggtaatcgg
agttcgattctccgtggcagtac
1−3tRNA−Gln(ttg)[87495,87568]
tggaatatggtgtaattggtaacacgtctggttttggtccagaagagtgg
gggttcgagaccctctattccaac
1−4tRNA−His(gtg)[87570,87644]

gtgttagaagctaaagtggtcgaagcgtctggttgtggtccagaagatag
tgagttcgagtctcatttaacaccc
1-5tRNA-Leu(tag)[87703,87778]
gcagaggtgggtaaactggtaaagccgcccgatttaggatcgggagttct
gagggttcgactccctctctctgtac
1-6tRNA-Leu(taa)[87785,87866]
gccccaatggtgaaactggttatacacgtagcacttaaaatgctatgctt
aggcttgcaggttcgatccctgcttggggtac
1-7tRNA-Arg(acg)[87871,87944]
tttctcatagtttaactggaaaaacacagagttacggcctttgagagtag
gggttcgagtccctttgagaaaac
1-8tRNA-Ala(tgc)[88170,88243]
ggcccagtagctaaactggcttagcacctcctttgcaaggagaagtatgt
gggttcgagccccatctggcccac
1-9tRNA-Lys(ttt)[88325,88397]
accctcgtagcttaatggcaaagctcctctcttttaaagaggcgaccgca
agatcggtacttgtcgggggtac
1-10tRNA-Ile(gat)[88687,88761]
aggaatatagctcagttggttagagtacttcgctgataccgaagatgtca
caagttcgaatcttgttattcctac
1-11tRNA-Gly(tcc)[88865,88939]
gcagcaatcgcatagcggcctattgcacctgacttccaatcaggatctcg
tgagttcgaatctcacttgctgcac
1-12tRNA-Met(cat)[88944,89018]
agcgaggtagagcagctggttagctcgctaggctcataacctagaggtcg
ggagttcgagtctccccttcgccac
1-13tRNA-Asp(gtc)[89097,89170]
agtctatcggtccagtggagtggacgctgccctgtcacggcagagatcac
gggttcgagtcccgtatagactgc
1-14tRNA-Thr(tgt)[89249,89323]
tccctacgtagctcaattggtggagcactggttttgtaaaccaaaggttg
tcggttcgagtccgacctagggatc
1-15tRNA-Val(tac)[89470,89544]
accagattagctcaggggtttagagcaactcttttacaaggagaaggtcg
atagttcgaatctatcatttggtac
1-16tRNA-Arg(tct)[89555,89628]
gggggattagtttaactggaaaaactataggcttctatcctatcgagtag
gggttcgaatcccttatccccttc
1-17tRNA-Met(cat)[89638,89712]
ggccttgtagctcagttggttagagcagctcactcataatgagaaggtct
caggttcgagtcctgactaggccac
1-19tRNA-Tyr(gta)[89926,90010]
gggacggttgctcggctggaagaggcgcaggactgtaaattctgtaatcc
actaggaatggaggttcgagtcctcctcgtcccac

1-20tRNA-Ser(gct)[90280,90367]
agaaaggtgtcagagtggcttattgagctaggttgctaacttagtggagt
gaaataccttccgtgggttcgaatcccatcctttcttc
1-21tRNA-Asn(gtt)[90397,90485]
tcttgagttgccgtagttggccgaacggtccagactgttaatctggtgag
atttatctccatcgtaggttcgaatcctacctcaagagc
1-22tRNA-Trp(cca)[90629,90702]
acttgagtagctcagttggtagagcagtggtctccaaaaccaaaggtcgt
aggttcgagtcctacctctcgtgc
1-23tRNA-Phe(gaa)[90724,90798]
ggggttgtagctgagttggtacaagcgtccgattgaagctcggaagtacg
aagggtcgaagccttccttccccac
1-24tRNA-Ile(gat)[143233,143308]
aatcgggtagttcaattggtagaacatcacactgataatgtgaaggttgt
gggttcgagtcccatcccgattacca
>KT962246.1 Cellulophaga phage phi4:1_18, complete genome
1-1tRNA-Pro(tgg)[87196,87272]
cgggtaatagaggagtctggtttatctcgctgcatttgggatgcagagca
cgcaggttcgaatcctgcttatccgac
1-2tRNA-Glu(ttc)[87352,87424]
gctgccatcgtctaacggctaggacctcaggttttcatcctggtaatcgg
agttcgattctccgtggcagtac
1-3tRNA-Gln(ttg)[87495,87568]
tggaatatggtgtaattggtaacacgtctggttttggtccagaagagtgg
gggttcgagaccctctattccaac
1-4tRNA-His(gtg)[87570,87644]
gtgttagaagctaaagtggtcgaagcgtctggttgtggtccagaagatag
tgagttcgagtctcatttaacaccc
1-5tRNA-Leu(tag)[87703,87778]
gcagaggtgggtaaactggtaaagccgcccgatttaggatcgggagttct
gagggttcgactccctctctctgtac
1-6tRNA-Leu(taa)[87785,87866]
gccccaatggtgaaactggttatacacgtagcacttaaaatgctatgctt
aggcttgcaggttcgatccctgcttggggtac
1-7tRNA-Arg(acg)[87871,87944]
tttctcatagtttaactggaaaaacacagagttacggcctttgagagtag
gggttcgagtccctttgagaaaac
1-8tRNA-Ala(tgc)[88170,88243]
ggcccagtagctaaactggcttagcacctcctttgcaaggagaagtatgt
gggttcgagccccatctggcccac
1-9tRNA-Lys(ttt)[88325,88397]
accctcgtagcttaatggcaaagctcctctcttttaaagaggcgaccgca
agatcggtacttgtcgggggtac
1-10tRNA-Ile(gat)[88687,88761]

aggaatatagctcagttggttagagtacttcgctgataccgaagatgtca
caagttcgaatcttgttattcctac
1-11tRNA-Gly(tcc)[88865,88939]
gcagcaatcgcatagcggcctattgcacctgacttccaatcaggatctcg
tgagttcgaatctcacttgctgcac
1-12tRNA-Met(cat)[88944,89018]
agcgaggtagagcagctggttagctcgctaggctcataacctagaggtcg
ggagttcgagtctccccttcgccac
1-13tRNA-Asp(gtc)[89097,89170]
agtctatcggtccagtggagtggacgctgccctgtcacggcagagatcac
gggttcgagtcccgtatagactgc
1-14tRNA-Thr(tgt)[89249,89323]
tccctacgtagctcaattggtggagcactggttttgtaaaccaaaggttg
tcggttcgagtccgacctagggatc
1-15tRNA-Val(tac)[89470,89544]
accagattagctcaggggtttagagcaactcttttacaaggagaaggtcg
atagttcgaatctatcatttggtac
1-16tRNA-Arg(tct)[89555,89628]
gggggattagtttaactggaaaaactataggcttctatcctatcgagtag
gggttcgaatcccttatccccttc
1-17tRNA-Met(cat)[89638,89712]
ggccttgtagctcagttggttagagcagctcactcataatgagaaggtct
caggttcgagtcctgactaggccac
1-19tRNA-Tyr(gta)[89926,90010]
gggacggttgctcggctggaagaggcgcaggactgtaaattctgtaatcc
actaggaatggaggttcgagtcctcctcgtcccac
1-20tRNA-Ser(gct)[90280,90367]
agaaaggtgtcagagtggcttattgagctaggttgctaacttagtggagt
gaaataccttccgtgggttcgaatcccatcctttcttc
1-21tRNA-Asn(gtt)[90397,90485]
tcttgagttgccgtagttggccgaacggtccagactgttaatctggtgag
atttatctccatcgtaggttcgaatcctacctcaagagc
1-22tRNA-Trp(cca)[90629,90702]
acttgagtagctcagttggtagagcagtggtctccaaaaccaaaggtcgt
aggttcgagtcctacctctcgtgc
1-23tRNA-Phe(gaa)[90724,90798]
ggggttgtagctgagttggtacaagcgtccgattgaagctcggaagtacg
aagggtcgaagccttccttccccac
1-24tRNA-Ile(gat)[143233,143308]
aatcgggtagttcaattggtagaacatcacactgataatgtgaaggttgt
gggttcgagtcccatcccgattacca
>KU927496.1 Salmonella phage 101962B_sal5, complete genome
1-1tRNA-Asn(gtt)[20194,20268]
gggtcagtcgtataaaggtcattacggaaggctgttaaccttcttatcgt

ggttcgagtccacgctgtccagcca
1-2tRNA-Thr(tgt)[20274,20349]
gctggtttagctccaatggtagagcagtcgccttgtaagcgaatgggtag
cggttcaagtccgttaaccagcacca
>KU927491.1 Salmonella phage 146851_sal5, complete genome
1-1tRNA-Asn(gtt)[20960,21034]
gggtcagtcgtataaaggtcattacggaaggctgttaaccttcttatcgt
ggttcgagtccacgctgtccagcca
1-2tRNA-Thr(tgt)[21040,21115]
gctggtttagctccaatggtagagcagtcgccttgtaagcgaatgggtag
cggttcaagtccgttaaccagcacca
>KU927492.1 Salmonella phage 146851_sal4, complete genome
1-1tRNA-Asn(gtt)[39490,39564]
gggtcagtcgtataaaggtcattacggaaggctgttaaccttcttatcgt
ggttcgagtccacgctgtccagcca
1-2tRNA-Thr(tgt)[39570,39645]
gctggtttagctccaatggtagagcagtcgccttgtaagcgaatgggtag
cggttcaagtccgttaaccagcacca
>KU927498.1 Salmonella phage 64795_sal4, complete genome
1-1tRNA-Asn(gtt)[39480,39554]
gggtcagtcgtataaaggtcattacggaaggctgttaaccttcttatcgt
ggttcgagtccacgctgtccagcca
1-2tRNA-Thr(tgt)[39560,39635]
gctggtttagctccaatggtagagcagtcgccttgtaagcgaatgggtag
cggttcaagtccgttaaccagcacca
>KU927495.1 Salmonella phage 103203_sal4, complete genome
1-1tRNA-Asn(gtt)[20457,20531]
gggtcagtcgtataaaggtcattacggaaggctgttaaccttcttatcgt
ggttcgagtccacgctgtccagcca
1-2tRNA-Thr(tgt)[20537,20612]
gctggtttagctccaatggtagagcagtcgccttgtaagcgaatgggtag
cggttcaagtccgttaaccagcacca
>KX098390.2 Erwinia phage vB_EamP_Rexella, complete genome
1-1tRNA-Asn(gtt)[41219,41294]
gacgggttagctcagttggtagagcgattgactgttaatcaattggtcat
cggttcaaatccggtactcgtcgcca
1-2tRNA-Glu(ttc)[41301,41377]
attcctgtagacaaattggtaaagtcaccaccctttcaaggtgttatttg
tgggttcaagtcccatcaggaatgcca
1-3tRNA-Asp(gtc)[41384,41458]
agtgctatagttcagcggtagaatacctccctgtcacggagatggtcacg
ggttcgaatcccgttagcactgcca
1-4tRNA-Ile(gat)[41462,41537]
tgcttcatagctcagtgggtagagcaaccgcccgataagcggtaggtcac

tggttcaagtccagttgaagcaacca
1-5tRNA-Tyr(gta)[41796,41877]
gggggattagtcaagcggtcgaagacggcagactgtaaatctgttatccc
acacggtagttcgaatctaccatcccccacca
1-6tRNA-Ser(gct)[41883,41974]
ggaagattggctgagtggctaaaagcgtccggttgctaaccggaaggcgg
tgtaacagcttcccactggttcgaatccagtatcttcctcca
1-7tRNA-Lys(ttt)[41980,42056]
aggtcgttagctcagttggttagagcaccggacttttaatccggatgtca
ttggttcgaggccaatacgacctacca

>KX098391.1 Erwinia phage vB_EamP_Gutmeister, complete genome

1-1tRNA-Asn(gtt)[36891,36966]
gacgggttagctcagttggtagagcgattgactgttaatcaattggtcat
cggttcaaatccggtactcgtcgcca
1-2tRNA-Glu(ttc)[36973,37049]
attcccgtagacaaattggtacagtcaccacactttcactgtggagtttg
tgggttcaagtcccatcgggaatgcca
1-3tRNA-Asp(gtc)[37056,37131]
agtcctatagttcagtcggtagaatacctccctgtcacggagaaggtcac
gggttcgagtcccgttaggactgcca
1-4tRNA-Ile(gat)[37135,37210]
tgcttcatagctcagtgggtagagcaaccgcccgataagcggtaggtcac
tggttcaagtccagttgaagcaacca
1-5tRNA-Tyr(gta)[37469,37550]
gggggattagtcaagcggtcgaagacggcagactgtaaatctgttatccc
acacggtagttcgaatctatcatcccccacca
1-6tRNA-Pro(tgg)[37555,37631]
cggcatttagctcagtttggcagagcatcggctttgggagtcgagggtcg
aaggttcaaatccttcagtgccgacca
1-7tRNA-Ser(gct)[37637,37728]
ggaagattggctgagtggctaaaggcgtccggttgctaaccggaaggcgg
tgtaacagcttcccactggttcgaatccagtatcttcctcca
1-8tRNA-Lys(ttt)[37734,37810]
aggtcgttagctcagttggttagagcaccggacttttaatccggatgtca
ttggttcgaggccaatacgacctacca

*Polydnaviridae

>AJ632313.1, AJ632317.1, AJ632321.1, AJ632305.1, AJ632310.1 Cotesia congregata virus complete genome

2-1tRNA-Thr(cgt)c[8914,8986]
gtcttagatagcttaactggaagagctcttggtgcgtaaccaaaagatcc
aggttcgattcccggccggggct
6-1tRNA-Thr(tgt)c[9967,10041]
agaccggatagctcaaatggtagagtagccgacatgtcttcggaaggttc
tgggttcgaatcccagtccgagcta

6−2tRNA−Thr(tgt)[23439,23513]
agaccggatagctcaaatggtagagtagccgacatgtcttcggaaggttc
tgggttcgtatcccagtccgagcta
10−1tRNA−Thr(cgt)[5364,5436]
atctcggatagcttaactggtagagcctttggcgcgtaaccaaatggtcc
gggttcgagtcccggtctggatt
11−1tRNA−Thr(tgt)[914,988]
agaccggatagctcaaatggtagagtagccgacatgtcttcggaaggttc
tgggttcgaatcccagtccgagcta
28−1tRNA−Thr(tgt)[9868,9942]
agaccggatagctcaaatggtagagtagccgacatgtcttcggaaggttc
tgggttcgaatcccagtccgagcta
28−2tRNA−Thr(tgt)[10188,10260]
gaccggatagctcaaatggtagagtagccgacatgtcttcggaagattct
gggttcgaatcccagtccgagca
28−3tRNA−Thr(tgt)[14305,14379]
agaccggatagctcaaatggtagagtagccgacatgtcttcggaaggttc
tgggttcgaatcccagtccgagcta
>DQ000240.1 Microplitis demolitor bracovirus segment O, complete sequence
15−1tRNA−Ser(cga)[1653,1724]
gccggatagctcaactggtagagcactcggcgcgataccgacatggtctg
ggttcgattcccagttcgggct
15−2tRNA−Ser(cga)[1875,1946]
gccggatagctcaactggtagagcactcggcgcgataccgacatggtctg
ggttcgattcccagttcgggct
15−3tRNA−Ser(cga)[8881,8952]
gccggatagctcaactggtagagcactcggcgcgataccgacatggtctg
ggttcgattcccagttcgggct
15−4tRNA−Ser(cga)[9104,9175]
gccggatagctcaactggtagagcactcggcgcgataccgacatggtctg
ggttcgattcccagttcgggct
15−5tRNA−Ser(cga)[9326,9397]
gccggatagctcaactggtagagcactcggcgcgataccgacatggtctg
ggttcgattcccagttcgggct
15−6tRNA−Ser(cga)[24254,24325]
gccggatagctcaactggtagagcactcggcgcgataccgacatggtctg
ggttcgattcccagttcgggct
15−7tRNA−Ser(cga)[24477,24548]
gccggatagctcaactggtagagcactcggcgcgataccgacatggtctg
ggttcgattcccagttcgggct
*Polyomaviridae
>KP033140.2 Adelie penguin polyomavirus isolate AdPyV_Crozier_2012, complete genome
1−1tRNA−Phe(gaa)[2769,2841]
gctgaagtagctcagttgggagagcattagactgaagatctaaaggtccc

tggttcaaccccgggcttcagca

*Poxviridae

>KM502564.1 Parapoxvirus red deer/HL953 strain HL953, complete genome

1–1tRNA–Pyl(cta)c[122496,122582]

cgctgctgtcggcgatgtacccggtgcagagctcctctagggggctctcg

aaatcgctgtcgctgttcgagtcgctgtggtggtggc

>MF503315.1 UNVERIFIED: Squirrelpox virus Berlin_2015, complete genome

1–1tRNA–Asp(gtc)[25493,25578]

attcaacatagtataatatactcgtttgtccaacgacataaagagttcgt

tgatttgtatttctcgttcgatacgataattggata

*Retroviridae

>AF033812.1 Abelson murine leukemia virus, complete genome

1–1tRNA–Gly(ccc)c[1169,1260]

cctgcgaggtagtggagtcggccactgggggctcccgtctcccacgcagg

cgagatgccattggggaggggtccggtgcctctcccgcaggg

>AF033813.1 Moloney murine sarcoma virus, complete genome

1–1tRNA–Gly(ccc)c[1105,1196]

cctgcgaggtagtggagtcggccacagggggctcccgtctcccacgcagg

cgagatgccattggggaggggtccggtgcctctcccgcaggg

>AF033811.1 Moloney murine leukemia virus, complete genome

1–1tRNA–Gly(ccc)c[905,996]

cctgcgaggtagtggagtcggccacagggggctcccgtctcccacgtagg

cgagatgccattggggaggggtccggtgcctctcccgcaggg

*Siphoviridae

>AF011378.1 Bacteriophage sk1 complete genome

1–1tRNA–Trp(cca)[27504,27576]

tgcgagcatagtataatggtaatgctacagattccaaacctgtaaacgtg

ggttcgattcctactgttcgtgt

>AF304433.1 Bacteriophage TP901–1, complete sequence

1–1tRNA–Lys(ttt)[13134,13208]

gtggctgcatggtcaaggggttaagacactgcacttttaatgcagaggcg

tgagttcgaatctcactcagtcaca

1–2tRNA–Ala(cgc)[13288,13375]

tagcaagtatatggctaaggttcgttagtaaggtcgcaccttacgactta

gtatggaattgttagggttcgactccctgacttgctat

>AF009630.1 Bacteriophage bIL170, complete genome

1–1tRNA–Pro(tgg)[31308,31379]

caggatatggtgtcaatggtagcatacgtgttttgggaacatgtggtgtt

ggttcgagtccagctatcctga

>AF323672.1 Bacteriophage bIL311, complete genome

1–1tRNA–Leu(taa)[13135,13220]

tgctcgaatggcggaattggcagacgctgcggacttaaaatccgttggtt

attaaaccgtgagggttcaagtccctctttgagcat

>AY129331.1 Mycobacteriophage CJW1, complete genome

1−1tRNA−Arg(cct)[63846,63929]
atggcccgtagctcagtcaggtagagcagccttgccccttgcgggcttgg
ttcgtccccggttcaaatccgggcgggccttgcc
1−2tRNA−Gly(tcc)[63932,64004]
gcgcccgtggtcgaattggaaagactcctggcttccacccaggttatgca
ggttcgagtcctgtcgggcgctc
>AY129333.1 Mycobacterium phage Che9c, complete sequence
1−1tRNA−Tyr(gta)[32232,32310]
aacacgttagaacgctattccacggactgtaaatccgtcggcttacgcct
acgcaggttcgaatcctgcacctgccacc
>AY129332.1 Mycobacterium virus Bxz2, complete genome
1−1tRNA−Asn(gtt)[3932,4007]
tgatctgtagctcaatcggcagagcacccggctgttaaccgggacgttgg
aggttcgagtcctcccagatcagcca
1−2tRNA−Trp(cca)[4048,4122]
aggcacgtagctcaattggtcagagcagcggtctccaaagccgccggctg
caggttcgagtcctgccgtgtctgc
1−3tRNA−Leu(cag)[4157,4231]
ggctcggtaggcaaacaggcaaagccgcctgtctcaggaacaggtgcgtg
agggttcgactccctcccgagctac
>X98106.1 Lactobacillus bacteriophage phig1e complete genomic DNA
1−1tRNA−Gly(tcc)[2594,2664]
gcgagtgtagtttagtggtaaaacgatagccttccaagctgtagtcgcgg
gtccgattcccgtcactcact
1−2tRNA−Ile(tat)c[31567,31641]
tattcccgtagctcagtggcagagcgtccaatttataattgggaggtcgc
tggttcgatgccagccgggaatatc
1−3tRNA−Tyr(gta)c[31887,31969]
ggcggtgcgtcagagatggagagctgtgtcggtctgtaaaaccgatcccg
ttgggtgagtaggtttgattcctaccgccgcca
>AF022214.1 Mycobacterium virus D29, complete genome
1−1tRNA−Asn(gtt)[4218,4292]
tgacgtgtagctcaatggcagagcatccggctgttaaccggacggttgaa
ggttcgagtccttcctcgtcagcca
1−2tRNA−Trp(cca)[4333,4408]
gtacacgtagctcaattggtagagcagcggtctccaaagccgccggttcc
aggttcgactcctggcgtgtatgcca
1−3tRNA−Gln(ctg)[4416,4488]
tccccgttcgtctaatcggtaagacgcctggctctggaccaggtaattga
ggttcgagtccttggcggggagc
1−4tRNA−Glu(ctc)[4523,4596]
ggtcccttggagtagcggataactcacctggccctcacccagaagatcgc
gggttcgaatcccgcagggactac
1−5tRNA−Tyr(gta)[4600,4680]

cgcgagatacccaagcggcaacgggatctgactgtaaatcagacgcttcg
gcttcgcaggttcgagtcctgctctcgcgac
>AY129338.1 Mycobacteriophage Omega, complete sequence
1-1tRNA-Gly(tcc)[95561,95633]
gcgcttgtggtcgaattggaaagactcctggcttccacccaggttatgca
ggttcgagtcctgtcgagcgctc
1-2tRNA-Tyr(gta)[96528,96601]
tggcccgtagctcaattggcagagcagccggcggtaatttcggcgcgtct
cggttcgagtccgggcgggctacc
>AY576796.1 Actinoplanes phage phiAsp2, complete genome
1-1tRNA-Asn(gtt)c[27417,27506]
gacctcgtagtccagttgggtgtatgacccggcgttgttgttgccgccca
tcgcccggccgatgatctggatcttgccggacggcggcgc
1-2tRNA-Ala(ggc)c[34296,34375]
gacgccaccagtttgggaggcgtgccggttggcgtccggcgcgctggctc
cggcctcggggtcgaaccccggcggcctcg
1-3tRNA-Trp(cca)c[36786,36858]
gggcgtccggggtgaggacggcctgggcgcgctccaggcgcgccaggttg
ccgatcagggcgacgaacgcccg
>AJ550940.2 Bacteriophage phi-BT1 complete genome
1-1tRNA-Thr(cgt)[31992,32066]
gctccggtagctcagcggaagagcgcccccgacgttcgagggggaagccg
ccggttcgaatccggcccggagcac
1-2tRNA-Lys(ctt)[40986,41059]
gttcccgtagctcaatgggcagagcacccggctcttaaccgggttgttgt
tggttcgagtccagccgggaacac
>AF165214.2 Bacteriophage D3, complete genome
1-1tRNA-Met(cat)[53398,53473]
cgcagggtggagaagtggtcatctcgccgggcccataacccggagatcgc
tggttcgaatccagcccttgctacca
1-2tRNA-Gly(tcc)[53980,54055]
gcggctctagctcaactggcagagcgctgtccttccaagtcagatgttgc
aggttcaagtcccgcgagccgctcca
1-3tRNA-Asn(gtt)[54068,54142]
tgacgtgtagctcagaggtatagcggtcggctgttacccgactggtcgat
ggttcgatcccatccgcgtcagcca
1-4tRNA-Thr(tgt)[54147,54222]
gccggtatggcgcaacagggagcgctgctgatttgtaatcagagggttgc
gggttcgactcctgctgccggcacca
>Z18946.1 Mycobacterium phage L5 complete genome
1-1tRNA-Asn(gtt)[4200,4272]
tgacgtgtagctcaatggcagagcgcccgactgttaatcgggtggttgaa
ggttcgagtccttccatgtcagc
1-2tRNA-Trp(cca)[4350,4423]

gtacacgtagctcaattggtagagcagcggtctccaaagccgccggttcc
aggttcgactcctggcgtgtatgc
1−3tRNA−Gln(ctg)[4479,4553]
tccccgttcgtctaatcggtaagacacccggctctggaccgggcaattga
ggttcgagtccttggcggggagcca
>AJ251789.2 Lactobacillus casei bacteriophage A2 complete genome
1−1tRNA−Leu(taa)[38396,38466]
gcaaccgtagctcagatgggagagcagtggcataagcctatcggtcgtgg
gttcgagccccaccggttgcg
>AJ006589.3 Bacteriophage phi−C31 complete genome
1−1tRNA−Gly(ccc)[31424,31511]
gctccggtagctcagcggaagagcgccgcaacccgggacgcgaaagcgtg
cacgcggaagccgccggttcgaatccggcccggagcac
1−2tRNA−Thr(cgt)[40702,40777]
gcctccctagctcagttcggttagagcgcctgtttcgtaatcagggggtc
ggcggttcgaatccgtcggggggctc
>AY543070.1 Bacteriophage T5, complete genome
1−1tRNA−Met(cat)c[28038,28113]
agttagttggcagagtggttatgcacctccttcatacggagcgactacag
tggttcaaatccactactaactacca
1−2tRNA−Ile(gat)c[28210,28286]
acttcggtagcttagcgatctaaagcactcggctgataaccgagagatcg
ggggtttaaatccctcccggagtacca
1−3tRNA−Thr(tgt)c[28667,28741]
gctcctaaagcattgttggtgatgcagttgccttgtaagcatctgaaccg
ggttcgattcctagtgggagcacca
1−4tRNA−Gln(ttg)c[29260,29337]
tggggattagcttagcttggcctaaagcttcggcctttgaagtcgagatc
attggttcaaatccaatatcccctgcca
1−5tRNA−Gln(ctg)c[29344,29419]
agaaggttagccaagtcggttaaggctgggatctctgaaaccactgatcg
tggttcgaacccactaccttctgcca
1−6tRNA−His(gtg)c[30206,30282]
gtggctatatcataattggttaatgatcctgattgtgaatcaggcctatg
tggattcgaattccactagccacccca
1−7tRNA−Ser(tga)c[30845,30934]
ggaaggtagggcgtagtggtacgcaactagtcttgaaaactagcccgctg
tagtgatacggtgatggttcgactccattaccttcctcca
1−8tRNA−Ser(gga)c[30942,31035]
ggaaaagcaaatagactggcgactaaacccgattggaaatcggttgagta
atagcaatattgccttatgggttcaactcccatcttttccgcca
1−9tRNA−Leu(tag)c[31624,31708]
ggggctatgctggaactggtagacaatacggccttagattccgtagctta
aatgcgtgggagttcgagtctccctagccccacca

1−10tRNA−Ala(tgc)c[31714,31788]
gggcgaatagtgtcagcgggagcacaccagacttgcaatctggtagggag
ggttcgagtccctctttgtccacca
1−11tRNA−Val(tac)c[32081,32154]
gctcggttagtataatgggagaaccccgcctttacacggcggttgtgata
gttcgattctatcaccgagtacca
1−12tRNA−Lys(ttt)c[32162,32239]
gggttgctagctcaactggtttagagcactggtcttttaaaccataggtt
acaggttcgagtcctgtgcaacccacca
1−13tRNA−Met(cat)c[32428,32505]
tgcgggttagatctctggtagagatcgctagtctcataagctagaaagag
gtaggttcgattcctgcacccgcttcca
1−14tRNA−Pro(tgg)c[32512,32588]
ctccgattagctcaattggctagagtacaccgtttggggcggtggggttg
aaggttcgagtccttcattggagacca
1−15tRNA−Asp(gtc)c[33578,33654]
gcgaccggggctggcttggtaatggtactcccctgtcacgggagagaatg
tgggttcaaatcccatcggtcgcgcca
1−16tRNA−Asn(gtt)c[33952,34029]
ggttccttagctctaattggttagagcggcatcttgttaagttgagggtt
gctggttcgaatccagcaggaaccgcca
1−17tRNA−Cys(gca)c[34546,34621]
cgaccgttggctgaatggcttaggcgaaggattgcaaatcctttttatgt
gagttcaaatctcatgcggtcgtcca
1−18tRNA−Phe(gaa)c[35096,35170]
gcacctttagctgagatggattagcgcttgcctgaagagcttgagaggtt
cgttcaattcgaacagggtgcacca
1−19tRNA−Glu(ttc)c[35257,35333]
gtcctgttagacaaactggtaaagtcactaccctttcaaggtagggtttg
cgggttcgatccccacacaggacgcca
1−20tRNA−Tyr(gta)c[35340,35427]
gggggagtattctgtaagtggtagcagagctgactgtaaattagttgtca
ttgcgactcgggtggttcgactccatcctcccctacca
1−21tRNA−Ser(gct)c[37170,37258]
ggaagaatagcataacggtattgcagcagattgctaatctgtcggtttga
aatatagccttgtgggttcgattcccacttcttccgcca
1−22tRNA−Arg(tct)c[40773,40847]
cggggtgtagtctaagggagaggcaggagtcttctaaattcctttatgca
ggttcgaatcctgtcacctcggcca
1−23tRNA−Ser(act)c[41431,41504]
tgccgacctagtataaacggtattatcaagccgtaactggtggaagatgt
aggttcgaatcctacggtcggtaa
>AY236756.1 Lactobacillus plantarum bacteriophage phiJL−1, complete genome
1−1tRNA−Ile(tat)[12,85]

tactcgtatagctcagtggtagagcactcaacttataattgaggggtcga
ttgttcaagccaatctacgagtat
>AM156909.1 Bacteriophage RTP, complete genome
1–1tRNA–Arg(tct)[4893,4969]
gccccgctagctcaattggatagagcaacgaccttctaagtcgtaggtta
ctggttcgaatccagtgcggagcgcca
>DQ121662.1 Bacteriophage JK06, complete genome
1–1tRNA–Arg(cct)c[22138,22213]
gttctgctggcgtaacaggataacgcagagacctcctaagtctcagttac
tggttcgagtccagtgcggaacacca
>AY131267.2 Bacteriophage Lc–Nu, complete genome
1–1tRNA–Asp(atc)[32542,32614]
gtgagagtagctaagtggaaaagcgtcccgtcatcaggcgggtaatgcgt
gggttcgattcccatctctcaca
>EF116926.2 Streptococcus phage SMP, complete genome
1–1tRNA–Ser(gct)[24448,24535]
ggagagttactcaagaggctgaagagggcaggttgctaccttgctaggcc
gaattttcggcgcatgggttcgaatcccatactctcct
>DQ398052.2 Mycobacteriophage Wildcat, complete genome
1–1tRNA–Trp(cca)[58812,58883]
gcgctgatagtgtagtggtaacacggcaagctccaaccttgctatctggg
gttcaagtcctcatcagcgtgc
1–3tRNA–Pro(tgg)[59496,59567]
cagggtgtggtgtagtggtcgcatggctgccttggaagcaggcggacgca
gttcaattctgcgcaccctgac
1–4tRNA–Phe(gaa)[59891,59965]
cggtcgttagtgaagatggagatcacgcgggcctgaaaaacccgagacgc
aggttcgattcctgcacgaccggcc
1–5tRNA–Met(cat)[59969,60045]
tgcggtgtagtggagttcggtaccatgcgaggctcataacctctgcgaca
cgggttcaaatcccgtcgccgcgacca
1–6tRNA–Tyr(gta)[60157,60239]
gcgtcagacgcacattgggtagtgcagcggtctgtaaaaccgtcgccttc
gggcattgggggttcaagtccctcctgacgcac
1–7tRNA–Ala(tgc)[60312,60383]
gggggtatcgtataatggtattacgcctgccctgcaagcaggtcattggg
gttcaattccccatacctccac
1–8tRNA–Val(tac)[60438,60509]
gcccgcttagctcaatgggagagccaccggcctacacccggtagacgaga
gttcaattctttcagcgggtac
1–9tRNA–Leu(tag)[60579,60653]
ggctcgttatgcaaaccggcaaagcagcctgccttagaagcaggtgtttc
agggttcgactccctggcgagctac
1–10tRNA–Leu(caa)[60711,60784]

ggtcgtgtaggcaaatggcaaagccgccaagctcaaaccttggtgtttgt
gggttcaactcccaccgcgactac
1-11tRNA-Gly(tcc)[60880,60953]
gagggtgaagtgttaacggtatgcacggctgtcttccaaacagcaagagc
gagttcgattctcgtcaccctcac
1-12tRNA-Gly(gcc)[61029,61100]
gtggacgtgatgtaatggtagcctccccgcttgccaagcagggtgtgcgg
gttcaattcccgtcgtccgcac
1-13tRNA-Gln(ctg)[61288,61360]
tgccctatggtgtaattgacagcacaggtggctctgaaccatctagtcga
ggtttgaatccttgtggggcagc
1-14tRNA-Asp(gtc)[61578,61650]
cccgtcatagtgtagtggttaacacgccagcttgtcacgctggtagcgag
ggttcaattccctctggcggggc
1-15tRNA-Thr(agt)[61657,61731]
ggctttgtagctcaacggttttagagcagctgtctagtaaacagccgacc
agggttcgactccctgcttagcctc
1-16tRNA-Lys(ctt)[61803,61875]
gggggcgtagtataacggcaattactgttggctcttacccttcagatggg
agttcgactctccccgccctcac
1-17tRNA-Cys(gca)[61882,61957]
gccggtgtgacagaacgatttatgtggcagtctgcaaaactgacctaagc
gggtttgactcccgccaccggttcca
1-18tRNA-Ile(gat)[61960,62032]
tggcccgtagttcaacggtaagaacggatcgctgataacgatcagacaag
agttcaactctcttcgggccaac
1-19tRNA-Arg(acg)[62139,62212]
gcccgtgtagctcagtggatagagccgtggcctacgaagccatgggccgg
gggttcgaatccctccacgggtac
1-20tRNA-Ser(gct)[62237,62319]
ggagagtgatgcaacagggtggttgcactcgcctgctaagcgatgtgtct
gcaaggatcacgttcaactcgtgctctctccgc
1-21tRNA-His(gtg)[62321,62394]
gtgtctgtagctcaatggtagagcctcccgctgtgaccgggacgacgacg
ggtcagaaccgtccagacacccca
1-22tRNA-Glu(ttc)[63026,63098]
ggttccgtggtgtaacggttagcacctgggtctttcaagcccaaggtcag
ggttcaactccctgcggaactgc
1-23tRNA-Glu(ctc)[63103,63174]
ggcccactggtgtaatgggaacacgccaccccctcaaggtggagtcaggg
gttcaattcccctgtgggctac
1-24tRNA-Asn(gtt)[63180,63251]
tccggtatcgttcaaaggcaggacaccaagctgttaacttggctatgttg
gttcgatcccagctaccggagc

>DQ054536.1 Lactococcus phage P008, complete genome
1−1tRNA−Asp(gtc)[27791,27862]
taggatatagccaaattggtatgtggtaggcagagtcgcaatctggtact
ggttcgattccagttgtcctag
1−2tRNA−Pro(tgg)[27919,27990]
caggatatagtgttaatggtagcatgcgtgttttgggaacatgtagtgtt
ggttcgagtccagctatcctga
>DQ227763.1 Lactococcus phage 712, complete genome
1−1tRNA−Trp(cca)[29387,29459]
tacgagcatagtatagtggtaatgctacagattccaaacctgtaaacgtg
ggttcgattcctactgttcgtgt
>DQ398041.1 Mycobacteriophage 244, complete genome
1−1tRNA−Arg(cct)[61979,62062]
atggcccgtagctcagtcaggtagagcagccttgccccttgcgggcttgg
ttcgtccccggttcaaatccgggcgggccttgcc
1−2tRNA−Gly(tcc)[62065,62137]
gcgcccgtggtcgaattggaaagactcctggcttccacccaggttatgca
ggttcgagtcctgtcgggcgctc
>DQ398043.1 Mycobacterium virus Che12, complete genome
1−1tRNA−Asn(gtt)[4024,4098]
tgacgtgtagctcaacggcagagcacccggctgttaaccggacggttgaa
ggttcgaatccttccacgtcagcca
1−2tRNA−Gln(ctg)[4135,4207]
tctcctttcgtctaatcggtaagacacccggctctggaccgggcaattga
ggttcgagtccttgagggagagc
1−3tRNA−Trp(cca)[4242,4315]
gggtcagtagctcaatcggtagagcagcggtctccaaagccgcgcgttgg
aggttcgagtcctccctggcccgc
>DQ227764.1 Lactococcus lactis phage jj50, complete genome
1−1tRNA−Trp(cca)[26497,26569]
tgcgagcatagtatagtggtaatgctacagattccaaacctgtaaacgtg
ggttcgattcctactgttcgtgt
>AM419438.1 Archaeal BJ1 virus complete genome
1−1tRNA−Phe(gaa)[19972,20048]
tggatcgatagctcagtcagggagagcaccggcctgaagcgctggcgtgc
ctcggttcgaatccgagtcgatccatc
>CP000917.1 Enterobacteria phage EPS7, complete genome
1−1tRNA−Met(cat)c[29711,29786]
agttagttggcagagaggttatgcacctccttcatacggagcgactacag
tggttcaaatccactactaactacca
1−2tRNA−Ile(gat)c[29884,29960]
gcttcggtagcttagcgatctaaagcactcggctgataaccgagagatcg
ggggtttaaatccctcccggagtacca
1−3tRNA−Thr(tgt)c[30533,30607]

gctcctaaagcattgctggcgatgcagttgccttgtaagcatctgaaccg
ggttcgattcctggtgggagcacca
1-4tRNA-Gly(tcc)c[31012,31086]
gcgtgattagttcagtggctagaataactggcttccaaccagtagacacg
agttcgactctcgtatcccgcacca
1-5tRNA-Gln(ttg)c[31093,31168]
tggagagtagtgtaacggttagcacaacggcctttgactccgttaatggt
aggttcgattcctccttctccagcca
1-6tRNA-Gln(ctg)c[31175,31250]
tgggatgtagatcaattggcagatcgtcggcctctgactccgaaggttcc
acgttcgatccgtggcatcccagcca
1-7tRNA-Arg(acg)c[31484,31558]
gcgtccttatttcaacggaaagaatgtaaagctacgaactttacgatcgg
ggttcgattccctgaggatgcacca
1-8tRNA-His(gtg)c[31565,31641]
gtggctatatcataattggttaatgatcctgattgtgaatcaggcctatg
tggattcgaattccactagccacccca
1-9tRNA-Ser(tga)c[32183,32272]
ggaaggtaggacatagtggtatgtaacaggtcttgaaaacctgcccgctg
tagcgatatggtgatggttcgactccattaccttcctcca
1-10tRNA-Leu(tag)c[32545,32625]
ggggatgtggcgaaattggcagacgcgctagatttagattctagtcttcg
ggtgtgggttcgagtccctccatccctacca
1-11tRNA-Ala(tgc)c[32871,32946]
ggggtcataggttatttggttaaacttactgccttgcaagcagtggaact
cagttcaattctgagtgactccacca
1-12tRNA-Ala(tgc)c[32953,33031]
ggggaatgggtctgcatggagtggacacctcgcttgcaccgaggacatca
gaacggttcgattccgttattctccacca
1-13tRNA-Val(tac)c[33949,34022]
gctcggttagtttaatgggagaaccccgtctttacacggcggttgcgata
gttcgattctatcaccgagtacca
1-14tRNA-Lys(ttt)c[34027,34104]
ggattgctagctcaactggataagagtagcggacctttaatccgtcagtt
ctgggttcgaatcccaggcaatccacca
1-15tRNA-Met(cat)c[34745,34822]
tgcgggttagatctctggtagagatcgctagtctcataagctagaaagag
gtaggttcgattcctgcacccgcttcca
1-16tRNA-Pro(tgg)c[34829,34907]
ctccgtgtagctcagtttggccagagcgtttcgtttggggcgatagggtc
ggggggttcaaatcctcccacggagacca
1-17tRNA-Gly(gcc)c[35213,35287]
gcgttcgtagttaaaaggtataatttttggttgccaaccagaagttgag
ggttcgattccctccgaccgcacca

1-18tRNA-Lys(ctt)c[35501,35576]
acatccatagctcaatggttagagctaccgcctcttaagcggagggttct
aggttcaagtcctagtgggtgtacca
1-19tRNA-Asp(gtc)c[35653,35729]
gcgatcggggctggcttggtaatggtactcccctgtcacgggagaaaatg
tgggttcgaatcccatcggtcgcgcca
1-20tRNA-Asn(gtt)c[36026,36108]
gggtcgttagccaagcggtttggcggtggactgttaatccatgtcgaaag
acaacgtaggttcgaatcctacacggcccgcca
1-21tRNA-Cys(gca)c[36116,36191]
cgaccgttggctgaatggcttaggcggaggcctgcaaaacctccttatgt
gagttcgaatctcatgcggtcgtcca
1-22tRNA-Phe(gaa)c[36931,37005]
gcgactttagctgagatggattagcgtctccctgaaaaggagaagaggta
cgatcgttacgtacaggttgcacca
1-23tRNA-Trp(cca)c[37012,37088]
attcctagagtgttactggacagcatgtcggtctccaaaaccgtacggtc
taggttcgagtcctagtaggtttgcca
1-24tRNA-Glu(ttc)c[37097,37173]
gtcctgttagacaaactggtaaagtcactaccctttcaaggtaggatttg
cgggttcgatccccgcacaggacgcca
1-25tRNA-Tyr(gta)c[37181,37271]
gttggattagtatcgtagaggtagcgaagcagactgtaaatctgccgact
cggaagggtctcgggtggttcgactccatcatccaacacca
1-26tRNA-Leu(taa)c[38186,38262]
gggggtgtaatcgaattggcataggtactggacttaaaattcaggttttg
tgggttcgaatcccaccacccctacca
1-27tRNA-Met(cat)c[38812,38889]
ggttctcaagctcatttggtatgagccgtcgcctcataagcgaaaggtag
gtaggttcgaatcctccgggagccacca
1-28tRNA-Ser(gct)c[38895,38988]
ggaagattaaccctaaaggtaagggagctgtttgctaaacagccagtagc
tgtggagacacggtgtgccagttcgaatctggcatcttcctcca
1-29tRNA-Arg(tct)c[42634,42708]
cggggtgtagtctaagggataggcaggagtcttctaaattcctttatgca
ggttcgaatcctgtcacctcggcca
>EF579802.1 Microbacterium phage Min1, complete genome
1-1tRNA-Arg(tct)c[42,117]
gcctccgtagctcaggggatagagcagcggccttctaattcgcgtgtcga
tggttcgaatccatccgggggcacca
>DQ499600.1 Corynebacterium phage P1201, complete genome
1-1tRNA-Arg(tct)c[49943,50016]
cccctagtagctcaaaggacagagcaatggatttctaccccataggttgc
aggttcgagtcctgcctaggggac

1−2tRNA−Gly(tcc)c[50276,50349]
gcctcattgttccaatggttaagatgtctgacttccaatcagacgatgcg
ggttcgatttccgcatgaggctcc
1−3tRNA−Arg(cct)c[50536,50609]
gccctggtagctcaatggatagagcagaggagtcctaatcctcaggttgg
aggttcgaatcctctctagggcgc
1−4tRNA−Tyr(gta)c[50880,50962]
cccgcaccggcgaagatggagagtcgcacctgactgtaaatcaggcactt
cggttgagtaggttcaaatcctatgtgcgggac
1−5tRNA−Trp(cca)c[51019,51092]
tcggatgtagttcaattggtagagcgctggtctccaaaaccagaagttgc
aggttcaagtcctgtcatccgggc
>EU744250.1 Mycobacterium virus Pukovnik, complete genome
1−1tRNA−Gln(ctg)[5774,5848]
tcctcgctagcacaactggtagatgcgctcggctctggaccgagaggttt
gaggttcgaatccttggcgaggagc
>EU816588.1 Mycobacterium phage Porky, complete genome
1−1tRNA−Arg(cct)[61660,61743]
atggcccgtagctcagtcaggtagagcagccttgccccttgcgggcttgg
ttcgtccccggttcaaatccgggcgggccttgcc
1−2tRNA−Gly(tcc)[61746,61818]
gcgcccgtggtcgaattggaaagactcctggcttccacccaggttatgca
ggttcgagtcctgtcgggcgctc
>EU246945.1 Lactobacillus phage Lrm1, complete sequence
1−1tRNA−Met(cat)[36665,36737]
gtgagagtagctaagtggaaaagcgtcccgtcatcaggcggacaatgcgt
gggttcgattcccatctctcaca
>EU816591.1 Mycobacterium phage Kostya, complete genome
1−1tRNA−Arg(cct)[62214,62297]
atggcccgtagctcagtcaggtagagcagccttgccccttgcgggcttgg
ttcgtccccggttcaaatccgggcgggccttgcc
1−2tRNA−Gly(tcc)[62300,62372]
gcgcccgtggtcgaattggaaagactcctggcttccacccaggttatgca
ggttcgagtcctgtcgggcgctc
>FJ188381.1 Stx2−converting phage 1717, complete genome
1−1tRNA−Met(cat)[24379,24453]
cggccctttagctcagtggtgagagcgagcgactcataatcgccaggtcg
ctggttcaaatccagcaaggccacc
1−2tRNA−Arg(tcg)[24456,24532]
ccgccattagctcatcgggatagagcgtcagccttcgaagctggttgcgc
gaggttcgagtccccggtggtggtcca
1−3tRNA−Arg(tct)[24546,24622]
gcgttgttagctcagcaggacagagcaattgccttctaagcaatcggtca
ctggttcgaatccagtacaacgcgcca

>FJ429185.1 Lactococcus phage P087, complete genome
1-1tRNA-Pro(tgg)c[59413,59486]
caggatatagctcagcttggtagagcactgcttttgggaagcaggggtca
aaggttcaaatccttttatcttga
1-2tRNA-Asn(gtt)c[59542,59614]
tgacccatagctcagttggtagagcgtcgcactgttaatgcgaatgtcgt
aggttcaagtcctactgggtcag
1-3tRNA-Asp(gtc)c[59656,59727]
ggtttattagtatagtggttattgcactcggttgtcagccgagtaacacg
agttcgattctcgtatagaccg
1-4tRNA-Thr(tgt)c[59730,59802]
gccgatttagctcagttggtagagcgtctgatttgtaatcagaaggtcac
aggttcaagtcctgtagtcggca
1-5tRNA-Cys(gca)c[59903,59975]
ctccctgtgaccgagtggtttaggtgcagctctgcaaaagctgatacgtc
ggttcgaatccgactagggagtc
>GQ478086.1 Enterococcus phage phiFL3A, complete genome
1-1tRNA-Arg(tcg)[15705,15779]
ttggcgtgtagcattgtggtaatgcaactgacttcgtgtgagataagatg
cgggttcgaatcctgtcacgccaat
1-2tRNA-Met(cat)[38661,38734]
ggaccattagctcagttggttagagccaaccgctcataacggttaggtca
taggttcgagtcctgtatggtcca
>GQ478084.1 Enterococcus phage phiFL2A, complete genome
1-1tRNA-Arg(acg)[14629,14703]
ttggcgtgtagcattgtggtaatgcaactgattacgtgtgagataagatg
cgggttcgaatcctgtcacgccaat
1-2tRNA-Met(cat)[35352,35425]
ggaccattagctcagttggttagagccaaccgctcataacggttaggtca
taggttcgagtcctgtatggtcca
>GQ478081.1 Enterococcus phage phiFL1A, complete genome
1-1tRNA-Asn(gtt)[14594,14666]
tggaatatagctcagttggtagagcgtacgactgttaatcgtagggtcat
gagttcgagtctcgttattccag
1-2tRNA-Met(cat)[37849,37922]
ggaccattagctcagttggttagagccaaccgctcataacggttaggtca
taggttcgagtcctgtatggtcca
>GQ452243.1 Enterococcus phage phiEf11, complete genome
1-1tRNA-Asn(gtt)[39646,39718]
tggaatatagctcagttggtagagcatacgactgttaatcgtagggtcat
gagttcgagtctcgttattccag
>GQ478088.1 Enterococcus phage phiFL4A, complete genome
1-1tRNA-Met(cat)[37498,37571]
ggaccattagctcagttggttagagcaaacggctcataaccgttcggtca

caggttcgagccctgtatggtcca
>HM152764.1 Mycobacterium phage Angelica, complete genome
1-1tRNA-Trp(cca)[994,1067]
gggtgtgtagctcaattggcagagcagcggtctccaaagccgccggttgc
acgttcgagtcgtgccgcgcccgc
>HM152767.1 Mycobacterium phage CrimD, complete genome
1-1tRNA-Trp(cca)[978,1051]
gggtgcgtagctcaatcggtagagcagcggtctccaaagccgccggttgc
acgttcgagtcgtgccgcgcccgc
>HM152763.1 Mycobacterium virus Bron, complete genome
1-1tRNA-Leu(caa)[60863,60937]
ggtcctgtaggcaaattggcaaagccgcgtcactcaaaatgacgtgtctg
tgggttcgagtcccaccgggactac
1-2tRNA-Thr(cgt)[60938,61012]
gctgccttagctcagatggctagagcgccgctctcgtaaagcggaggtcg
cgggttcgaggcccgcaggcagctc
1-3tRNA-Lys(ctt)[61299,61372]
gcctcgttagctcagttggtagagctgccgactcttaatcggtaggtcgc
aggttcaaatcctgcacggggtac
1-4tRNA-Tyr(gta)[61383,61466]
gcgtcggaggtacctgttggtttggtacacctgcctgtaaagcaggcgct
tcggcttcgggggttcgattccctcccggcgtac
1-5tRNA-Trp(cca)[61476,61549]
agctcggtagctcaattggtagagcagcggtctccaaagccgtgggttcc
ccgttcgagtcggggccgggttgc
1-6tRNA-Leu(tag)[61550,61624]
ggaactgtaggcaaactggaaaagccgcctgacttaggatcaggtgtttg
cgagttcgactctcgccagttctac
1-7tRNA-His(gtg)[61625,61694]
gctaagtagcttaatggtaaagcgccccgttgtggccggggtgattccgg
ttcgattccgggcttaagcc
1-8tRNA-Cys(gca)[62874,62947]
gccgctgtggccgagtggctaggcaccggcctgcaaagccggttagtccg
gttcgattccggagggcggctcca
1-9tRNA-Lys(ttt)c[63228,63302]
gcccctatagctcagttggtagagctatcgccttttaagcgacaggtcgc
aggttcgagtcctgctgggggcacc
>HM029250.1 Lactococcus phage 949, complete genome
1-1tRNA-Met(cat)[42403,42475]
ggctctttagcttaattggtaaagtcctccgcccataacggagtaagtgc
aggttcaagccctgcaagagcca
1-2tRNA-Arg(tct)[43986,44058]
gttcggttagtgtaattggataacacacataatttctaattatgtactct
ggattcgagttccagactgagca

1–3tRNA–Asp(gtc)[44843,44915]
gattcattggtgtagtggttatcacgcttgcctgtcacgcaagagaacag
gggttcgaatcccctatgaatcg
1–4tRNA–Pro(tgg)[45463,45534]
caggatatggtgttaatggtagcatgcgtgttttgggaacatgtagtgct
ggttcgagtccagctatcctga
1–5tRNA–Pyl(cta)[46865,46937]
tgcgaacatagtatagtggtaatgctacagactctaagtatgtaaacgta
ggttcgattcctactgttcgtat
1–6tRNA–Trp(cca)[46993,47064]
atccgtttagtgtagtggttaacacatcagattccaaacctgataacgtg
ggttcgattcctacaatggatg
1–7tRNA–Met(cat)[47277,47350]
tacagggtgggtggcaaggcgtcacgctagtttcataagctagaatagaa
cggttcgattccgttaccttgaat
>HM144386.1 Brochothrix phage BL3, complete genome
1–1tRNA–Asn(gtt)[40805,40876]
tgagatatggctcaatggtagagcaccgcactgttaatgcgtaggttata
ggttcgagtcctgttatctcag
>HQ406778.1 Enterobacteria phage SPC35, complete genome
1–1tRNA–Met(cat)c[27768,27843]
agttagttggcagagtggttatgcacctccttcatacggagcgactacag
tggttcaaatccactactaactacca
1–2tRNA–Ile(gat)c[27940,28016]
gcttcggtagcttagcgatctaaagcactcggctgataaccgagagatcg
ggggtttaaatccctcccggagtacca
1–3tRNA–Thr(tgt)c[28592,28666]
gctcctaaagcattgttggcgatgcagttgccttgtaagcatctgaaccg
ggttcgactcctggtgggagcacca
1–4tRNA–Gly(tcc)c[29101,29175]
gcgtgattagttcagtggctagaataactggcttccaaccagtagacacg
agttcgactctcgtatcccgcacca
1–5tRNA–Gln(ttg)c[29182,29256]
tggagagtagtgtaacggttagcactggagattttgaatctcttagttag
ggttcgaatccctactttcctgcca
1–6tRNA–Gln(ctg)c[29263,29338]
tgggatgtagatcaattggcagatcgtcggcctctgactccgaaggttcc
acgttcaatccgtggcatcccagcca
1–7tRNA–His(gtg)c[29571,29647]
gtggctatatcataattggttaatgatcctgattgtgaatcaggcctatg
tggattcgaattccactagccacccca
1–8tRNA–Ser(tga)c[30215,30304]
agaagataggacgtagtggtacgtaactggtcttgaaaaccagcccgccg
tagcgatacggtgatggttcgactccattatcttctgcca

1–9tRNA–Leu(tag)c[30514,30590]
gcgtgattgatggaatgggcatacataccgtccttagaagtcgggttttg
agggttcgaatcccttgtcacgcacca
1–10tRNA–Ala(tgc)c[30596,30674]
ggggaatgggtctgcatggagtggacacctcgcttgcaccgaggacatca
gaacggttcgattccgttattctccacca
1–11tRNA–Lys(ttt)c[31054,31132]
agatcgctagctcaataggtttagtagcatccgacttttaatcggaaggt
tctgggttcgagtcccaggcgatctacca
1–12tRNA–Met(cat)c[31322,31399]
tgcgggttagatctctggtagagatcgctagtctcataagctagaaagag
gtaggttcgattcctgcacccgcttcca
1–13tRNA–Pro(tgg)c[31406,31481]
cagtgcgtagcgcagttggtagcgtgggagccttggatgcttcgggtcgc
aggttcgagtcctgccgcactgacca
1–14tRNA–Lys(ctt)c[32516,32591]
acacccatagctcaatggttagagctaccgcctcttaagcggagggttct
aggttcaagtcctagtgggtgtacca
1–15tRNA–Asp(gtc)c[32769,32845]
gcgaccggggctggcttggtaatggtactcccctgtcacgggagggaatg
tgggttcaaatcccatcggtcgcgcca
1–16tRNA–Asn(gtt)c[33143,33225]
gggtcgttagccaagcggtttggcggtggactgttaatccatgtcgaaag
acaacgtaggttcgaatcctacacggcccgcca
1–17tRNA–Cys(gca)c[33235,33310]
cgaccgttggctgaatggcttaggcgaaggattgcaaatcctttttatgt
gagttcaaatctcatgcggtcgtcca
1–18tRNA–Phe(gaa)c[33943,34017]
gcacctttagctgagatggattagcgcttgcctgaagagcttgagaggtt
cgttcgattcgaacagggtgcacca
1–19tRNA–Glu(ttc)c[34104,34178]
gcacctatcgtctagtggttaggacaccactctttcacagtgggaacacg
agttcgatcctcgttgggtgtgcca
1–20tRNA–Tyr(gta)c[34186,34273]
gggcgtttattccgtaagtggtagcggaggggattgtaaatccctagtca
ttgcgactcgagtggttcgactccattaacgcccacca
1–21tRNA–Ser(gct)c[35304,35392]
ggaagaatagcataacggtattgcagcagattgctaatctgtcggtttga
aatatagccttgtgggttcgattcccacttcttccgcca
1–22tRNA–Arg(tct)c[38936,39010]
cggggtgtagtctaagggagaggcaggagtcttctaaattcctttatgca
ggttcgaatcctgtcacctcggcca
>JF744988.1 Mycobacterium phage Faith1, complete genome
1–1tRNA–Thr(cgt)[62646,62719]

gccaccttagctcagttggtagagcagccccttcgtaacgggcaggtcag
cggttcgattccgctaggtggctc
1−2tRNA−Pro(tgg)[62947,63021]
ctggctgtagctcaacttggtagagcgctggctttgggtgccaggggttg
caggttcaaatcctgctagccagac
1−3tRNA−Trp(cca)[63142,63215]
agctcggtagctcaattggtagagcagcggtctccaaagccgccggttcc
ccgttcgagtcggggtcgggttgc
1−4tRNA−Tyr(gta)[63326,63408]
gtggtggttgggcttgttggttggcccacctgactgtaaatcaggcgttt
cggcaccgggggttcgattccctcccaccacac
1−5tRNA−Leu(tag)[63822,63896]
ggccctctagcccaattggcagaggcacaggttttaggtacctgtcagtg
cgagttcgagtctcgcgggggccac
1−6tRNA−His(gtg)[63897,63966]
gcttagtagcctagtggtaaggcagccggttgtgatccggtgaacctgag
ttcgattctcagctaaagcc
1−7tRNA−Gln(ctg)[63971,64045]
tgaccggtagcacaactggtagttgcgccgcgctctggacgcggaggttg
ttggttcgatcccagctcggtcagc
1−8tRNA−Gly(tcc)[64161,64235]
gtgctagtaacccatgttggctgggtgcctgacttccactcaggatttcg
cgggttcgattcctgtctagcacac
1−9tRNA−Lys(ctt)[64278,64355]
tgcctcgttagctcagttggtagagctgccgactcttaatcggtaggtca
caggttcaagtcctgtacggggtaccca
1−10tRNA−Cys(gca)[65545,65618]
gccgtcatggctgagtggttaggcgtcggactgcaaatccggcttatccc
agttcgattctgggtggcggctcc
1−11tRNA−Asn(gtt)[65980,66053]
tcctccgtagctcaattggcagagcgcgcgactgttaatcgcgtggttgg
tggttcgagtccacccgttggagc
1−12tRNA−Lys(ttt)c[66247,66321]
gcccctatagctcagttggtagagcaggagacttttaatcttcgggtcct
aggttcgatccctagtgggggcacc
>HM208537.1 Escherichia phage HK639, complete genome
1−1tRNA−Ile(gat)c[33694,33763]
ggggtgtggctgggcctgcatggactgatcacccatgaaaacttcggttc
gaatccggagcactccacca
1−2tRNA−Asn(gtt)[44363,44437]
gggtcagaagcacagcggttgtgcgttcggctgttaaccgaatggtcgaa
ggttcgaatccttcctgtcccgcca
>JN035618.1 Gordonia phage GTE7, complete genome
1−1tRNA−Asn(gtt)[13421,13494]

tgtgatatagctcaattggcagagcgctggactgttaatccggtggttga
aggttcgagtccttctatcacagc
>JN116826.1 Rhodococcus phage RGL3, complete genome
1-1tRNA-Gln(ctg)[46429,46500]
tccccgttcgtctaatggcaggacgccgcgctctggacgcggtaatcgag
gttcgagtccttgacggggagc
1-3tRNA-Trp(cca)[46643,46716]
gggcacgtagctcaattggtagagcaccggtctccaaagccggtggttgg
gggttcgagtccctccgggcctgc
>HQ665011.1 Escherichia phage bV_EcoS_AKFV33, complete genome
1-1tRNA-Met(cat)c[27840,27915]
agttagttggcagagtggttatgcacctccttcatacggagcgactacag
tggttcaaatccactactaattacca
1-2tRNA-Ile(gat)c[28012,28088]
gcttcggtagcttagcgatctaaagcactcggctgataaccgagagatcg
ggggtttaaatccctcccggagtacca
1-3tRNA-Thr(tgt)c[28664,28738]
gctcctaaagcattgttggcgatgcagttgccttgtaagcatctgaaccg
ggttcgattcctggtgggagcacca
1-4tRNA-Gln(ttg)c[29227,29302]
tggagagtagtgtaacggttagcacaacggcctttgactccgttaatggt
aggttcgattcctccttctccagcca
1-5tRNA-Gln(ctg)c[29309,29384]
tggagtgtagatcaattggcagatcgtcggcctctgactccgaaggttcc
tggttcgatcccaggcactccagcca
1-6tRNA-His(gtg)c[29617,29693]
gtggctatatcataattggttaatgatcctgattgtgaatcaggcctatg
tggattcgaattccactagccacccca
1-7tRNA-Leu(tag)c[30369,30445]
gcgtgattgatggaatgggcatacataccgtccttagaagtcgggttttg
agggttcgaatcccttgtcacgcacca
1-8tRNA-Ala(tgc)c[30451,30529]
ggggaatgggtctgcatggagtggacacctcgcttgcaccgaggacatca
gaacggttcgattccgttattctccacca
1-9tRNA-Val(tac)c[31168,31241]
gctcggttagtttaatgggagaaccccgtctttacatggcggttgagata
gttcgattctatcaccgggtacca
1-10tRNA-Lys(ttt)c[31246,31324]
agatcgctagctcaataggtttagtagcatccgacttttaatcggaaggt
tctgggttcaagtcccaggcgatctacca
1-11tRNA-Met(cat)c[31514,31591]
tgcgggttagatctctggtagagatcgctagtctcataagctagaaagag
gtaggttcgattcctgcacccgcttcca
1-12tRNA-Pro(tgg)c[31598,31675]

ctccgtgtagctcagtttggccagagcgtttcgtttggggcgatagggtc
gggggttcaaatcctcccacggagacca
1-13tRNA-Lys(ctt)c[32666,32741]
acatccatagctcaatggttagagctaccgcctcttaagcggagggttct
aggttcaagtcctagtgggtgtacca
1-14tRNA-Asp(gtc)c[32919,32995]
gcgaccggggctggcttggtaatggtactcccctgtcacgggagggaatg
tgggttcaaatcccatcggtcgcgcca
1-15tRNA-Asn(gtt)c[33293,33375]
gggtcgttagccaagcggtttggcggtggactgttaatccatgtcgaaag
acaacgtaggttcgaatcctacacggcccgcca
1-16tRNA-Cys(gca)c[33382,33456]
gcatccttgtccgagtggcttaggtgacggtctgcaaaatcgtctaggtt
ggttcaattccagcagggtgctcca
1-17tRNA-Phe(gaa)c[34209,34283]
gcacctttagctgagatggattagcgcttgcctgaagagcttgagaggtt
cgttcgatacgaacagggtgcacca
1-18tRNA-Trp(cca)c[34290,34366]
attcctagagtgttactggacagcatgtcggtctccaaaaccgtacggtc
taggttcgagtcctagtaggtttgcca
1-19tRNA-Glu(ttc)c[34375,34451]
gccctgttagacaaactggtaaagtcactaccctttcaaggtaggatttg
cgggttcgatccccgcacaggacgcca
1-20tRNA-Tyr(gta)c[34459,34549]
gttggattagtatcgtagaggtagcgaagcagactgtaaatctgccgact
cggaagggtctcgggtggttcgactccatcatccaacacca
1-21tRNA-Leu(taa)c[35426,35502]
gggggtgtaatcgaattggcataggtactggacttaaaattcaggttttg
tgggttcgaatcccaccacccctacca
1-22tRNA-Ser(gct)c[36092,36180]
ggaagaatagcataacggtattgcagcagattgctaatctgtcggtttga
aatatagccttgtgggttcgattcccacttcttccgcca
1-23tRNA-Arg(tct)c[37799,37873]
cggggtgtagtctaagggagaggcaggagtcttctaaattcctttatgca
ggttcgaatcctgtcacctcggcca
>JN116823.1 Rhodococcus phage REQ2, complete genome
1-1tRNA-Leu(taa)[30212,30288]
gctccgagtcgctccggggcgtgtcagcggccgacttaaaatccgccgag
tgtgggttcgagtcccactgggggcac
1-2tRNA-Ser(cga)[48487,48575]
ggaagatgaaccgcgaatggtcgcgggaccgcctcgaaagcggatcgggc
gtgcaagcgtcaggggttcgattcctccgtcttccgcca
1-3tRNA-Ser(gct)[48577,48662]
ggagagtgacccacaccgggtgtgggatcgcctgctacgcgaatcgtgga

cacagcgccatgaggttcgactcctccactctccgc
>HQ711985.1 Pseudomonas phage vB_PaeS_PMG1, complete genome
1-1tRNA-Gly(tcc)[50917,50992]
gcggctctagctcaactggcagagcgctgtccttccaagtcagatgttgc
gggttcaagtcccgcgagccgctcca
>JN116827.1 Rhodococcus phage RER2, complete genome
1-1tRNA-Asn(gtt)[44961,45034]
tcacgtatagctcaatcggcagagcaacgcactgttaaggcgggggttga
aggttcgagtccttctacgtgagc
1-3tRNA-Trp(cca)[45160,45234]
gggcacgtagctcaattggatagagccccggtctccaaagccggtggttg
ggggttcgagtccctccgggcctgc
>HQ698895.1 Synechococcus phage S-CBS4, complete genome
1-1tRNA-Thr(tgt)c[4456,4527]
gcccttgtagctcagtggtagagcagcgcttttgtaaagcgcttgtcgca
agttcgactcttgtcgggggct
>JX182372.1 Streptomyce phage TG1, complete genome
1-1tRNA-Thr(cgt)[39556,39630]
gccttcctagctcagttggccagagcatccgtttcgtaatcggaaggtcg
acggttcgaatccgtcggaaggctc
>JF946695.1 Mycobacterium phage SWU1, complete genome
1-1tRNA-Asn(gtt)[4193,4265]
tgacgtgtagctcaatggcagagcgcccgactgttaatcgggtggttgaa
ggttcgagtccttccatgtcagc
1-2tRNA-Trp(cca)[4343,4416]
gtacacgtagctcaattggtagagcagcggtctccaaagccgccggttcc
aggttcgactcctggcgtgtatgc
1-3tRNA-Gln(ctg)[4472,4546]
tccccgttcgtctaatcggtaagacacccggctctggaccgggcaattga
ggttcgagtccttggcggggagcca
>JX182369.1 Streptomyces phage phiHau3, complete genome
1-1tRNA-Trp(cca)[1200,1270]
gcgttcgagcgcgtgggcgcggcgcggtctccaaagccgcagtcagcagg
ttcgactcctgccgggcgtgc
>AB712291.1 Enterococcus phage BC-611 DNA, complete genome
1-1tRNA-Trp(cca)[29838,29911]
cgagtggttagtgtaatggttagcacaacagtctccaaaactgttagaga
gggttcgattccttcactgcttgt
>JQ965645.1 Salmonella phage SSU5, complete genome
1-1tRNA-Lys(ttt)c[30485,30565]
aatttgctgggcaacggtagatttgcctgggccgccttttgtgccgccaa
ccacaatgatcttggtcattggtgagttccc
1-2tRNA-Asn(gtt)[42525,42599]
gatggtgtagctcagtggtagagcggttgactgttaatcaactggtcggt

ggttcgagtccacccaccatcgcca
>JX195166.1 Pectobacterium phage My1, complete genome
1−1tRNA−Gln(ctg)c[26934,27009]
tgaagtatagctcaattggtagagcgcctgactctgactcaggaggttta
aggttcgagaccttatacttcagcca
1−2tRNA−His(gtg)c[27132,27208]
gtggccgaagcatttatttggatgatgttcgatctgtgacatcgaggaag
tgggatcgttacccaccggtcacccca
1−3tRNA−Ser(gga)c[28674,28765]
ggaagaacaaatagactggcgactaaaactgcttggaaagtagctgactg
gagaaatccggcttgagagttcgaatctctcttcttccgcca
1−4tRNA−Leu(tag)c[29278,29362]
ggggttatgctggaactggtagacaatacagtcttagaatctgtagctta
aatgcgtgggagttcgagtctccctagccccacca
1−5tRNA−Val(tac)c[29933,30007]
gctcgcttagttcaacggttagaatactagccttacatgctagggatgat
agttcgattctatcagcgagtacca
1−6tRNA−Ile(gat)c[30016,30091]
gctctgatagttcagttggttagaacaggcgaccgataatcgccaaacac
tggttcgagtccagttcggagtacca
1−7tRNA−Gly(tcc)c[30706,30780]
gcgtgattagttcagtggctagaataactggcttccacccagtagacgag
ggttcgactccctcatcccgcacca
1−8tRNA−Met(cat)c[31551,31628]
agtgtatgggtagtcacggtgaatacggcactttcatacggtgcgtcgag
attggttcgatttcaatatacactacca
1−9tRNA−Leu(taa)c[31718,31794]
gcatcactggcccaattggtagaggcatgaggcttaagacttcagagttc
ccagttcgaatctggggtggtgtacca
1−10tRNA−Lys(ctt)c[32492,32568]
tgcttgctagctcaactggttagagcactcgcctcttaagcgataggtta
tgagttcaagtctcatgcaggctacca
1−11tRNA−Ala(tgc)c[32658,32736]
ggggaatgggaatgctaggagtgttcacctcgcttgcaccgaggatacca
gtggggttcgaatccccaattctccacca
1−12tRNA−Pro(tgg)c[32742,32817]
ctccgattagctcaattgatagagtgcgccgtttggggcggtgaggttga
aggtttgagtccttcattggagacca
1−13tRNA−Thr(tgt)c[33713,33787]
gcctccaaagcattagtggcgatgcagttgccttgtaagcatcagaattc
ggttcgattccggatgggggcacca
1−14tRNA−Lys(ttt)c[34480,34556]
ggattgttagccaaatggtttaaggcaccggacttttaatccggggattg
aaggttcgagtccttcacagtccacca

1-15tRNA-Gly(gcc)c[34881,34954]
gcgttcgtagttaaatggtataatttctggttgccaaccagaggttgaga
gttcgattctctccgaccgcacca
1-16tRNA-Lys(ctt)c[35161,35237]
gctttcttagctcaattggttagagctaccgactcttaatcggagggtta
taggttcaagtcctatagagagcacca
1-17tRNA-Asp(gtc)c[35406,35482]
gcgatcggggctggcttggtaatggtactcccctgtcacgggagaatatg
tgggttcaaatcccatcggtcgcgcca
1-18tRNA-Asn(gtt)c[35489,35566]
ggttccttagctctaattggttagagcggcatcttgttaagttgagggtt
gctggttcgaatccagcaggaaccgcca
1-19tRNA-Trp(cca)c[36999,37075]
atcgaccgagtgttactggacagcataccggtctccaaaaccgtgtggtt
agggttcgagtccctaggtctttgcca
1-20tRNA-Arg(acg)c[37832,37906]
gcagacatagtttaacggaaagaatctatagctacgaactatagggtggt
ggttcgattccatctgtctgtacca
1-21tRNA-Gln(ttg)c[37913,37988]
tggaacttagtgtactggttagcacaggggcctttgactcccctagagtt
aggttcaattcctacagttcctgcca
1-22tRNA-Met(cat)c[38089,38164]
tgcgagttagagttctggtgaactcatgaggctcatatcctcatccagaa
aggttcgattcctttactcgcttcca
1-23tRNA-Glu(ttc)c[38172,38246]
gcacctatcgtctaatggttaggacaactgcctttcgagcagtgaatcgg
agttcgattctccgtgggtgtacca
1-24tRNA-Tyr(gta)c[38253,38333]
gtgggattggcacagcggcgactgcgggagactgtaaatctcctatacta
cacctaggttcgagtcctagatcccacacca
1-25tRNA-Ser(gct)c[38821,38912]
ggaagggttcactaatttggtaagtgagcggactgctaatccatcgggtt
ggtaaaacagccttcagggttcaagtccctgtccttccgcca
1-26tRNA-Arg(tct)c[39776,39852]
gcgctcttagctcaattggacagagcaacgtagttctaattcgtaggtta
caggttcgaatcctgtagggcgtacca
1-27tRNA-Leu(tag)c[40909,40981]
tgccctcttggtctagttggaaatgatagtaccctaggggtacaggcgta
ggttcgagtcctacagtgggaaa
>JX182370.1 Streptomyces phage R4, complete genome
1-1tRNA-Phe(gaa)[1094,1167]
gggccggtagctcagttggaagagcggcggagtgaaatcccgcaggtcgc
aggttcgagtcctgcctggcccac
>JX100810.1 Caulobacter phage CcrColossus, complete genome

1–1tRNA–Trp(cca)[59240,59325]
acgggaattccagagcggtcaaatgggctggtctccaaaaccagcggcgc
aagccttcgggggttcgaatccctcttcccgtgcca
1–2tRNA–Gly(gcc)[59336,59412]
gcgtctatagtatattggtctgattacccttcgttgccaacgaagagagg
cgggttcgattcccgctagacgctcca
1–3tRNA–Gly(tcc)[59417,59490]
gcgtccgtagctcaatggtagagcaatgggcttccaccccgtggtcgtgg
gttcgatccccaccggacgctcca
1–4tRNA–Glu(ttc)[60055,60133]
ggggccttcgtctaaactggactaaggatagccggctttcacccggcaga
tgcgggttcgaaccccgtaggcccttcca
1–5tRNA–Asp(gtc)[60149,60223]
ggtgcgttcgtatatcggttaatacaccggactgtcgatccgggaagacg
ggttcgactcccgtacgcaccgcca
1–6tRNA–Pro(cgg)[61499,61576]
ctgcgtgtagctcagtctggttagagcgctcggtccggagccgaggggtc
gtaggttcaaatcctgctacgcagacca
1–7tRNA–Pro(tgg)[61584,61658]
cagagtctggccgagcggctaggcaccggttttgggaaccggacgaggcg
ggttcaactcctgcggctctgacca
1–8tRNA–Pro(ggg)[61660,61736]
cagtgtgtggcgcagtttggtagcgcgcttgctcgggatgcaagaggtcg
caggttcaaatcctgctacactgacca
1–9tRNA–Lys(ctt)[62019,62108]
gggtgagttctccgagcggtcgaaggggagtgactcttaatcacttggac
gaaagtcccaccgtgggttcgaatcccacctcaccctcca
1–10tRNA–Leu(cag)[62115,62202]
cccggtgtgacgaagtttggtagacgtgcgagtttcagatactcgtgtcc
gagagggcggggaggttcgaatcctctcaccgggacca
1–11tRNA–Leu(caa)[62210,62296]
gcccaactggtcggaatggcagacgcaggatactcaaaatatccggaccg
caaggtcgtcccggttcaagtccggggttgggcacca
1–12tRNA–Leu(gag)[62304,62391]
cgcagcgtggcggagtctggtagacgcgccatcttgagagggtggtgtcc
gaaagggcggggaggttcgaatcctctcgctgcgacca
1–13tRNA–His(gtg)[62400,62474]
tctggtgtagtcgagtggttaagacgccgcattgtggatgcggaggctct
ggttcaatcccaggcaccagtacca
1–14tRNA–Ala(tgc)[62479,62553]
ggggatgtagctcaggggtagagcggctgttttgcaagcagcgggtcggg
ggttcgaatccctccatctccacca
1–15tRNA–Ser(tga)[62821,62908]
ggatgtatggctgagaggctgaaagcgcccgtcttgaaaacgggtcgacc

gcaaggtcgcgggagttcgaatctctctacatccgcca
1-16tRNA-Val(gac)[68535,68609]
ggcaggttagttcagaggtagaacgccgctatgacatggcggatgtcggg
ggttcaattccctcacctgctacca
1-17tRNA-Ile(gat)[68611,68686]
cctagtgtagctcagttggtagagcagcggatcgataatccgccggtcgc
cggttcgatcccggccgctaggtcca
1-18tRNA-Ile(gat)[68695,68770]
tggggcatagctcaggggttagagcgcgcgcttgataagcgtgaggccgt
tggttcgagaccaactgtccctacca
1-19tRNA-Val(cac)[68805,68880]
cgcgaacgggccagatgggacggcaggggtgtcacatacctcgattgact
gtgttcgattcacaggttcgcgacca
1-20tRNA-Gln(ctg)[68888,68961]
tgggggatcgtctaatggtaggacggtagagtctggttctacagatatag
gttcgagtcctattcccccatcca
1-21tRNA-Gln(ttg)[68968,69041]
tctcgggtggtctaatggtaggacatctggttttggtccagagggttggg
gttcgattccttggccgagagcca
1-22tRNA-Asn(gtt)[69054,69142]
tctcatgtggcggaactggtaaacgcaccggactgttaatccggcgtctc
gcaagagacttgggggttcgaagccctccgtgagagcca
1-23tRNA-Arg(acg)[69159,69255]
gctgcggcgaaatgccctggtaacccggcgcgcactacgaatgcgcgagc
cgtagctggagttgaaaaggctggttcgaatccagcccagggcgcca
1-24tRNA-Arg(acg)[69260,69337]
agcgatgtagctcagtggatttagagcgttcccctacgaagggaagggtc
gggggttcgagtccctccatcgctgcca
1-25tRNA-Cys(gca)[132216,132302]
gctacggtggactgagtggtaaggcgtgggactgcaaatcccgctggcga
aagcctatcgtgggttcgattcccactcgtagctcca
1-26tRNA-Met(cat)[132962,133038]
tgcggggtggagcagtccggtagctcgtttggctcataaccaaaaggtcg
agggttcaaatccctcccccgcatcca
1-27tRNA-Met(cat)[133537,133623]
ggtgtggtggctcgacaggtaaggcgccggcctcataagccggagggcga
aagtcctatgtaggttcgaatcctactcacacctcca
1-28tRNA-Arg(tct)c[249029,249104]
ggtcccttggtccaactggagagggcagccctcttctaaagggcgggtac
tggttcgagtccagtagggatcgcca
>JX100809.1 Caulobacter phage CcrSwift, complete genome
1-1tRNA-Trp(cca)[47039,47114]
gtcggtctagctcatggggtagagcggcggtctccaaaaccgcgcgtggc
aggttcgagtcctgcgaccggcgcca

1-2tRNA-Gly(gcc)[47120,47194]
gcgtccatcgtatatgggttattgcttcgcgttgccaacgcgacgaaccg
ggttcgagtcccggtggacgctcca
1-3tRNA-Gly(tcc)[47201,47274]
gcggatatagctcaaagggagagctactgccttccaagcagaagatgcgg
gttcgagccccgctatccgctcca
1-4tRNA-Glu(ttc)[47431,47508]
ggcccggtcgtctagcggtttgcaggattcgtgactttcaatcacgagag
cggagttcgagtctccgtcgggcctcca
1-5tRNA-Asp(gtc)[47532,47606]
ggtgcgttcgtatatcggtcaatactccggattgtcgatccggcaagagg
ggttcgattcccctacgcaccgcca
1-6tRNA-Pro(cgg)[48467,48542]
cggggtgtagctcagtcggtagagcgctcggttcgggaccgagaggccgt
gggttcgagtcccgccacccagacca
1-7tRNA-Pro(ggg)[48550,48624]
cgggatgtagctcagcggtagagcgcgcggctgggggccgcgaggccgca
ggttcgagacctgccaccccgacca
1-8tRNA-Lys(ctt)[48813,48899]
ggatgtgtcacctcagtggcgagaggaccgggctcttaatccggcagcga
aagctcatcgtgggttcgagtcccaccacatcctcca
1-9tRNA-Lys(ttt)[48907,48996]
gcacgagtgacccgagcggcgaagggcgtcgacttttaatcgactgggcg
tccagcctcaccgagggttcgagtccctcctcgtgctcca
1-10tRNA-Leu(cag)[49003,49088]
gcgaaagtggcggaacaaggtagacgcaccggtctcaggtaccggcggct
tcggtcatgagggttcgagtccctcctttcgtacca
1-11tRNA-Leu(caa)[49096,49180]
gccggtctggcggaaatggcagacgcagtggcttcaagtcccaccgcctt
cgggcgtcccggttcgatgccggggaccggcacca
1-12tRNA-Leu(gag)[49187,49274]
tgcgaagtggcggaatttggtagacgcaccggcatgagaggccggcgccc
ttcggggcgtgagggttcgagtcccttcttcgcgacca
1-13tRNA-Ala(tgc)[49365,49435]
ggggtatgcgcagggcgcaggcatcctttgcaaggaagtcggggtgggtt
cgagtcccacatactccacca
1-14tRNA-Ser(cga)[49440,49527]
ggtaggttggctgagaggccgaaggcgcccgtttcgaaaacgggagaacc
gcaaggttccgtgggttcgaatcccacacctacctcca
1-15tRNA-Ser(gga)[49532,49619]
ggatgtatggctgagaggccgaaggcgcccgcttggaaagcgggtgaacc
gcaaggttccgtaggttcgaatcctactgcatccgcca
1-16tRNA-Phe(gaa)[50907,50982]
ggactcgtagctcagcaggtagagcgccggcttgaaaccccggaggtcgg

aggttcgattcctcccgtttccacca
1-17tRNA-Val(gac)[50988,51063]
gcccgatgagccagatgggaaggcgccgccgtgacatggcgggattgaga
aggttcgattccttcatcaggcacca
1-18tRNA-Ile(gat)[51066,51141]
gcaagtgtagctcagtcggtagagcagcggaccgataatccgcctgtccc
tggttcgagtccaggcatttgcacca
1-19tRNA-Ile(gat)[51149,51223]
tgtggcatagctcaggggtagagcgcgcgcttgataagcgtgaggtcggg
ggttcgaagccctctgccactacca
1-20tRNA-Val(cac)[51257,51332]
tggcgtttagcatagtgggagtgcaggggtgtcacatacctcgatcgaac
tggttcgattccagtaacgccaacca
1-21tRNA-Gln(ctg)[51338,51411]
tgagggttcgtctaacggtaggacggctgggtctggtccagctaatctag
gttcgagtcctagcccctcatcca
1-22tRNA-Gln(ttg)[51419,51492]
tctcgggtggtctaatggtaagacatctggttttggtccagaggattggg
gttcgattccttggccgagaacca
1-23tRNA-Ile(aat)[51508,51582]
tccagtcaatgcacaaggtgtgcagcggcactgttaatgcctgtgagccc
ggttcgattccggggtctggagcca
1-24tRNA-Arg(tct)[51785,51862]
gcgcgggtagctcaatggacccagagcagccgtcttctacacggtgggtt
gggggttcgagtccctccccgcgcgcca
1-25tRNA-Cys(gca)c[101259,101347]
gcttcggtggactgagaggttaggcgcgggattgcaaatctctgctggtg
ggaacacctatgagggttcgagtccctcccgaagctcca
1-26tRNA-Lys(ctt)c[101927,102000]
tttgcgaatagcacagtggtagtgcaacagcctcttaagctgtcggtcgc
gggttcaatccccgcttcgatgaa
1-28tRNA-Met(cat)[103075,103153]
cgcggggtagaggagtccggttgtcctcgtctggctcataaccaggagat
cgtgggttcaaatcccactcccgctccca
1-29tRNA-Met(cat)[103563,103647]
ggtacggtggctcgacaggtaaggcatcggcctcataagccgagggttcg
tcccaatcctggttcaaatccaggtcgtacctcca
>JQ806764.1 Salmonella phage vB_SosS_Oslo, complete genome
1-1tRNA-Asn(gtt)[45678,45752]
gggtcagtcgtataaaggtcattacggaaggctgttaaccttcttatcgt
ggttcgagtccacgctggcccgcca
1-2tRNA-Thr(tgt)[45758,45833]
gctggtttagctccaatggtagagcggtcgccttgtaagcgaatgggtag
cggttcaagtccgttaaccagcacca

>JN986845.1 Enterobacteria phage vB_EcoS_ACG-M12, complete genome
1-1tRNA-Arg(tct)[3487,3561]
gctctgttagcttaatggaaaaagccgcagccttctaagctgttgatggt
ggttcgactccaccacggggcgcca
>JX100813.1 Caulobacter phage phiCbK, complete genome
1-1tRNA-Trp(cca)[47786,47861]
gtcggtctagctcatggggtagagcggcggtctccaaaaccgcgcgtggc
aggttcgagtcctgcgaccggcgcca
1-2tRNA-Gly(gcc)[47867,47941]
gcgtccatcgtatatgggttattgcttcgcgttgccaacgcgacgaaccg
ggttcgagtcccggtggacgctcca
1-3tRNA-Gly(tcc)[47948,48021]
gcggatatagctcaaagggagagctactgccttccaagcagaagatgcgg
gttcgagccccgctatccgctcca
1-4tRNA-Glu(ttc)[48178,48255]
ggcccggtcgtctagcggtttgcaggattcgtgactttcaatcacgagag
cggagttcgagtctccgtcgggcctcca
1-5tRNA-Asp(gtc)[48278,48352]
ggtgcgttcgtatatcggtcaatactccggattgtcgatccggcaagagg
ggttcgattcccctacgcaccgcca
1-6tRNA-Pro(cgg)[49792,49867]
cggggtgtagctcagtcggtagagcgctcggttcgggaccgagaggccgt
gggttcgagtcccgccacccagacca
1-7tRNA-Pro(ggg)[49875,49949]
cgggatgtagctcagcggtagagcgcgcggctgggggccgcgaggccgca
ggttcgagacctgccaccccgacca
1-8tRNA-Lys(ctt)[50140,50226]
ggatgtgtcacctcagtggcgagaggaccgggctcttaatccggcagcga
aagctcatcgtgggttcgagtcccaccacatcctcca
1-9tRNA-Lys(ttt)[50233,50323]
gcacgagtgacccgagcggcgaagggcgtcgacttttaatcgactaggcg
tccgcgcctcaccgagggttcgagtccctcctcgtgctcca
1-10tRNA-Leu(cag)[50330,50413]
gcgaaagtggcggaacggcagacgcactggtctcaggtaccagcgacttc
ggtcatgagggttcgaatccctcctttcgtacca
1-11tRNA-Leu(caa)[50421,50505]
gccggtctggcggaaatggcagacgcagtggcttcaagtcccaccgcctt
cgggcgtcccggttcgaggccggggaccggcacca
1-12tRNA-Leu(gag)[50511,50599]
tgcgaagtggcggaactttggtagacgcaccggcatgagaggccggcgcc
cttcggggcgtgagggttcgagtcccttcttcgcgacca
1-13tRNA-Ala(tgc)[50683,50753]
ggggtatgcgcagggcgcaggcatcctttgcaaggaagtcggggtgggtt
cgagtcccacatactccacca

1-14tRNA-Ser(cga)[50758,50845]
ggtaggttggctgagaggccgaaggcgcccgtttcgaaaacgggaggacc
gcaaggttccgtgggttcgaatcccacacctacctcca
1-15tRNA-Ser(gga)[50850,50937]
ggatgtatggctgagaggccgaaggcgcccgcttggaaagcgggtgaacc
gcaaggttccgtaggttcgaatcctactgcatccgcca
1-16tRNA-Phe(gaa)[51631,51706]
ggactcgtagctcagcaggtagagcgccggcttgaaaccccggaggtcgg
aggttcgattcctcccgtttccacca
1-17tRNA-Val(gac)[51712,51787]
gcccgatgagccagatgggaaggcgccgccgtgacatggcgggattgaga
aggttcgattccttcatcaggcacca
1-18tRNA-Ile(gat)[51790,51865]
gcaagtgtagctcagtcggtagagcagcggaccgataatccgcctgtccc
tggttcgagtccaggcatttgcacca
1-19tRNA-Ile(gat)[51873,51947]
tgtggcatagctcaggggtagagcgcgcgcttgataagcgtgaggtcggg
ggttcgaagccctctgccactacca
1-20tRNA-Val(cac)[51981,52056]
tggcgtttagcatagtgggagtgcaggggtgtcacatacctcgatcgaac
tggttcgattccagtaacgccaacca
1-21tRNA-Gln(ctg)[52062,52135]
tgagggttcgtctaacggtaggacggctgggtctggtccagctaatctag
gttcgagtcctagcccctcatcca
1-22tRNA-Gln(ttg)[52143,52216]
tctcgggtggtctaatggtaagacatctggttttggtccagaggattggg
gttcgattccttggccgagaacca
1-23tRNA-Ile(aat)[52232,52306]
tccagtcaatgcacaaggtgtgcagcggcactgttaatgcctgtgagccc
ggttcgattccggggtctggagcca
1-24tRNA-Gly(tcc)[52509,52586]
gcgcgggtagctcaatggacccagagcagccgtcttccacacggaaggtt
gggggttcgaatccctccccgtgcgcca
1-26tRNA-Cys(gca)c[101969,102057]
gcttcggtggactgagaggttaggcgcgggattgcaaatctctgctggtg
ggaacacctatgagggttcgagtccctcccgaagctcca
1-27tRNA-Gly(tcc)c[102637,102710]
tttgcgaatagcacagtggtagtgcaacagccgtcctagctgtcggtcgc
gggttcaatccccgcttcgatgaa
1-29tRNA-Met(cat)[103783,103861]
cgcggggtagaggagtccggttgtcctcgtctggctcataaccaggagat
cgtgggttcaaatcccactcccgctccca
1-30tRNA-Met(cat)[104228,104312]
ggtacggtggctcgacaggtaaggcatcggcctcataagccgagggttcg

tcccaatcctggttcaaatccaggtcgtacctcca
>JX100814.1 Caulobacter phage CcrRogue, complete genome
1–1tRNA–Trp(cca)[48466,48541]
gtcggtctagctcatggggtagagcggcggtctccaaaaccgcgcgtggc
aggttcgagtcctgcgtccggcgcca
1–2tRNA–Gly(gcc)[48546,48620]
gcatccgtagtatataggtgattacagcgcgttgccaacgcgtagagtcg
ggttcgagtcccgacggatgctcca
1–3tRNA–Gly(tcc)[48625,48698]
gcgggtatagctcaaagggagagttgctgccttccaagcagaagatgcga
gttcgagcctcgctatccgctcca
1–4tRNA–Glu(ttc)[49141,49218]
ggcccggtcgtctagcggtttgcaggattcctgactttcaatcaggagag
cggagttcgagtctccgtcgggcttcca
1–5tRNA–Asp(gtc)[49244,49318]
ggtgccgtcgtatatgggttattatcaccggctgtctcccggtggaaacg
ggttcgagtcccgtcggcatcgcca
1–6tRNA–Pro(cgg)[49997,50072]
cggggtgtagctcagtcggtagagcgctcggttcgggaccgagaggccgt
gggttcgagtcccgccacccagacca
1–7tRNA–Pro(ggg)[50080,50156]
cgggatgtagctcagtttgggagagcgcgcggctgggggccgcgaggccg
caggttcaagtcctgccaccccgacca
1–8tRNA–Thr(ggt)[50159,50234]
aacgctgttgcgcgtggcttcgcgcccaccccttggtatggggcggtaga
aggttcaattccttccagcagcacca
1–9tRNA–Lys(ctt)[50365,50451]
ggatgtgtcacctcagtggcgagaggaacgggctcttaatccgtcagcgc
aagctcaccgtgggttcgagtcccaccacatcctcca
1–10tRNA–Lys(ttt)[50457,50546]
gcacaagtgacccgagcggcgaagggcgtcgacttttaatcgactaggcg
tccagcctcaccgagggttcgagtccctccttgtgctcca
1–11tRNA–Leu(cag)[50550,50636]
gcgaatgtggcggaactggtagacgcgccggcctcaggtgccggtgccct
tcggggcgtgtgggttcgagtcccacctttcgtacca
1–12tRNA–Leu(caa)[50644,50731]
gccttgctggcggaagtggtagacgcactgttctcaaaaagcaggccggg
cgaccgggctctgggttcgatccccaggtgaggcacca
1–13tRNA–Leu(gag)[50739,50826]
tgcgaagtggcggaaactggtagacgcaccggcatgagaggccggcgccc
ttcggggcgtgagggttcgagtcccttcttcgcgacca
1–14tRNA–His(gtg)[50907,50977]
gctgatcgccgctgggcggggagcgggttgtgtccccgctaggcacggtt
cgatcccgtgggtcagtacca

1–15tRNA–Ala(tgc)[50983,51053]
ggggtatgcgcagggcgcaggcatcctttgcaaggaagtcggggtgggtt
cgagtcccacatactccacca
1–16tRNA–Phe(gaa)[51958,52033]
ggactcgtagctcagcaggtagagcgccggcttgaaaccccggaggtcgg
aggttcgattcctcccgtttccacca
1–17tRNA–Val(gac)[52039,52114]
gcccgatgagccagatgggaaggcaccgctttgacatggcggcattgaga
aggttcgattccttcatcaggcacca
1–18tRNA–Ile(gat)[52120,52195]
gcaagtgtagctcagttggtagagcagcggaccgataatccgcccgtccc
tggttcgagtccaggcatttgcacca
1–19tRNA–Ile(gat)[52203,52278]
tgtggcatagctcaggggtcagagcgcgcgcttgataagcgtgaggtcgg
tggttcgagaccacctgtcactacca
1–20tRNA–Val(cac)[52313,52386]
aggcgattagctcaatggtagagcaggggtgccacatacctcggtcgtag
gttcgattcctacatcgcccacca
1–21tRNA–Gln(ctg)[52393,52466]
tgagggttcgtctaacggtaggacgcctgggtctggtccaggtaatctag
gttcgagtcctagcccctcatcca
1–22tRNA–Gln(ttg)[52473,52546]
tctcgggtcgtctaatggtaagacatctggttttggtccagaggattggg
gttcgattccttggccgagagcca
1–23tRNA–Ile(aat)[52564,52638]
tccagtcaatgcacaaggtgtgcagcggcactgttaatgcctgtgagccc
ggttcgattccggggtctggagcca
1–24tRNA–Arg(tct)[52840,52917]
gcgcgggtagctcaacggaaccagagcagccgtcttctacacggtgggtt
gggggttcgagtccctccccgtgcgcca
1–25tRNA–Cys(gca)c[102172,102260]
gctacggtggtccgagaggttaggcgcgggactgcaaatctctgctggtg
gcgacacctatgagggttcgagtccctcccgtagctcca
1–26tRNA–Met(cat)[103047,103125]
cgcggggtagaggagtccggttgtcctcgtctggctcataaccagaagat
caggggttcaaatccccttcccgctccca
1–27tRNA–Met(cat)[103384,103470]
ggtacggtggctcgacaggtaaggcatcggcctcataagccgagggacta
gctcccgatcctggttcaaatccaggtcgtacctcca
1–28tRNA–Ala(tgc)[149330,149403]
tccgctgctcgcgcgggtagccgcccgtcgtcttgcggatggcgccccgg
acgatcctgtcggccgtgtcggga

>JQ340389.1 Vibrio phage pVp–1, complete genome

1–1tRNA–Met(cat)[37974,38050]

tgcgagaatggagaaatggcgtcttgcgagtttcataagctcgataacgt
ggggttcgaatccccatctcgcatcca
1-2tRNA-Lys(ttt)[38057,38133]
gcgcgtgtagctcagttggttagagcatctgacttttaatcaggtggtcg
gtggttcgagtccacctacgcgcacca
1-3tRNA-Glu(ttc)[38135,38210]
acaccggaagacagatggattggtcgtcacccctttcaaggtggagtttgc
gggttcgagtcccgtccggtgtacca
1-4tRNA-Ala(tgc)[38217,38292]
ggggaattagctcagttgggagagcagggtctttgcaaggctcaggtcat
cggttcgagtccgatattctccacca
1-5tRNA-Met(cat)[38381,38457]
agaggcttggctgagttggttatagcgactccttcatgcggagtaagacg
ggggttcgagtccctcagcctctacca
1-6tRNA-Tyr(gta)[38550,38639]
ggttgaatgcgacgaattggtaagtcagcggactgtaaatccgtagccac
cgagaggggcatctaggttcaagtcctagttcaaccacca
1-7tRNA-Asn(gtt)[38646,38721]
ggggaattaactcagcggtcagagtggctgcctgttaagcagctggtcgt
aggttcaaatcctacattccccgcca
1-8tRNA-Asp(gtc)[38728,38805]
gcggctgtgtgcgagatggtttaggcaacctccctgtcacggaggagtac
gtgggttcaagtcccatcggtcgcgcca
1-9tRNA-Pro(tgg)[38812,38890]
cggggtgtaatgtcagtctggtcagacggctcgctttggatgcgagaggt
cgtaggttcgaatcctacctccccgacca
1-10tRNA-Leu(tag)[39227,39313]
gcaggagtggcgtaattggtagccgcgctggatttagattccagtggtct
ttgtaccgtgagggttcaagtccctcctcctgtacca
1-11tRNA-Gly(tcc)[39882,39968]
gcgtactgaagactgagcggtcggtcactagccttccaagctagcgccct
taatcgggatagggttcgactccctctgtacgctcca
1-12tRNA-Thr(tgt)[40148,40223]
gcttccttagctcagttgggagagcagctcacttgtaatgagcaggtcgt
ccgttcgaatcggacagggagcacca
1-13tRNA-Gln(ttg)[40431,40506]
aggggattagctcagttggtagagcatcggaatttgactccgagggtcgc
aggttcaagtcctgcatcccccgcca
1-14tRNA-Val(tac)[40772,40848]
gcaggattagctcagtagagtagagcacttcccttacaaggaaggggtcg
taggttcaagtcctacatcctgtacca
1-15tRNA-Ile(gat)[41719,41794]
acttccgtagctcagttggtagagcaaacgaccgataatcgttaggtcac
tggttcgagtccagtcgggagtacca

1-16tRNA-Met(cat)[41796,41871]
gtccctttagctcagtggttagagcgtcctgctcataacaggagggtcac
tggttcaagtccagtaaggggcacca
1-17tRNA-Phe(gaa)[42188,42263]
gcgggaatagctcagtaggtagagcacttggttgaagaccttggtgtcgc
tggttcaattccagcttcccgcacca
1-18tRNA-Arg(acg)[42272,42349]
gcgcgattagctcagatggatagaagcagcaccctacgaaggtgaaggtc
tggggttcgagtccctgatcgcgcgcca
1-19tRNA-Ser(gct)[42354,42443]
ggagagatgcccgaactggttaagggagctccctgctaaggagtcggctg
ctaacgtggtctgtgagttcaaatctcactctctccgcca
1-20tRNA-His(gtg)[44928,45004]
gtgacgttggctgatgtggaaatggctccgggttgtgattccggaagatg
cgggttcgaaccccgtacgtcacccca
1-21tRNA-Arg(tct)[45236,45312]
gcgcccttagctcagctggatagagcacgtcccttctaaggatgtggtcg
taggttcgaatcctacagggcgtgcca
1-22tRNA-Leu(taa)[45497,45583]
gctggcgtggtggaattggtaatacacgtgggacttaaaatcccatgccc
tagaggattgagagttcgagtctctccgccagtacca
>JQ182736.1 Enterobacteria phage vB_EcoS_Rogue1, complete genome
1-1tRNA-Arg(cct)[3387,3462]
gttctgctggcgtaacaggataacgcagagacctcctaagtctcagttgc
tggttcgagtccagtgcggaacacca
>JX100811.1 Caulobacter phage CcrKarma, complete genome
1-1tRNA-Trp(cca)[47887,47962]
gtcggtctagctcatggggtagagcggcggtctccaaaaccgcgcgtggc
aggttcgagtcctgcgaccggcgcca
1-2tRNA-Gly(gcc)[47968,48042]
gcgtccatcgtatatgggttattgcttcgcgttgccaacgcgacgaaccg
ggttcgagtcccggtggacgctcca
1-3tRNA-Gly(tcc)[48049,48122]
gcggatatagctcaaagggagagctactgccttccaagcagaagatgcgg
gttcgagccccgctatccgctcca
1-4tRNA-Glu(ttc)[48279,48356]
ggcccggtcgtctagcggtttgcaggattcgtgactttcaatcacgagag
cggagttcgagtctccgtcgggcctcca
1-5tRNA-Asp(gtc)[48380,48454]
ggtgcgttcgtatatcggtcaatactccggattgtcgatccggcaagagg
ggttcgattcccctacgcaccgcca
1-6tRNA-Pro(cgg)[49317,49392]
cggggtgtagctcagtcggtagagcgctcggttcgggaccgagaggccgt
gggttcgagtcccgccacccagacca

1–7tRNA–Pro(ggg)[49400,49474]
cgggatgtagctcagcggtagagcgcgcggctgggggccgcgaggccgca
ggttcgagacctgccaccccgacca
1–8tRNA–Thr(ggt)[49480,49552]
gctgctgcgcctggatcggcgccggccccttggtatggggaagtagaagg
ttcaattccttccagcagcacca
1–9tRNA–Lys(ctt)[49663,49749]
ggatgtgtcacctcagtggcgagaggaccgggctcttaatccggcagcga
aagctcatcgtgggttcgagtcccaccacatcctcca
1–10tRNA–Lys(ttt)[49755,49844]
gcacgagtgacccgagcggcgaagggcgtcgacttttaatcgactgggcg
tccagcctcaccgagggttcgagtccctcctcgtgctcca
1–11tRNA–Leu(cag)[49851,49936]
gcgaaagtggcggaacaaggtagacgcaccggtctcaggtaccggcggct
tcggtcatgagggttcgagtccctcctttcgtacca
1–12tRNA–Leu(caa)[49944,50028]
gccggtctggcggaaatggcagacgcagtggcttcaagtcccaccgcctt
cgggcgtcccggttcgatgccggggaccggcacca
1–13tRNA–Leu(gag)[50035,50122]
tgcgaagtggcggaatttggtagacgcaccggcatgagaggccggcgccc
ttcggggcgtgagggttcgagtcccttcttcgcgacca
1–14tRNA–Ala(tgc)[50213,50283]
ggggtatgcgcagggcgcaggcatcctttgcaaggaagtcggggtgggtt
cgagtcccacatactccacca
1–15tRNA–Ser(cga)[50288,50375]
ggtaggttggctgagaggccgaaggcgcccgtttcgaaaacgggagaacc
gcaaggttccgtgggttcgaatcccacacctacctcca
1–16tRNA–Ser(gga)[50380,50467]
ggatgtatggctgagaggccgaaggcgcccgcttggaaagcgggtgaacc
gcaaggttccgtaggttcgaatcctactgcatccgcca
1–17tRNA–Phe(gaa)[51757,51832]
ggactcgtagctcagcaggtagagcgccggcttgaaaccccggaggtcgg
aggttcgactcctcccgtttccacca
1–18tRNA–Val(gac)[51838,51913]
gcccgatgagccagatgggaaggcgccgccgtgacatggcgggattgaga
aggttcgattccttcatcaggcacca
1–19tRNA–Ile(gat)[51916,51991]
gcaagtgtagctcagttggtagagcagcggaccgataatccgcccgtccc
tggttcgagtccaggcatttgcacca
1–20tRNA–Ile(gat)[51999,52073]
tgtggcatagctcaggggtagagcgcgcgcttgataagcgtgaggtcggg
ggttcgaagccctctgccactacca
1–21tRNA–Val(cac)[52107,52182]
tggcgtttagcatagtgggagtgcaggggtgtcacatacctcgatcgaac

tggttcgattccagtaacgccaacca
1–22tRNA–Gln(ctg)[52188,52261]
tgagggttcgtctaacggtaggacggctgggtctggtccagctaatctag
gttcgagtcctagcccctcatcca
1–23tRNA–Gln(ttg)[52269,52342]
tctcgggtggtctaatggtaagacatctggttttggtccagaggattggg
gttcgattccttggccgagaacca
1–24tRNA–Ile(aat)[52358,52432]
tccagtcaatgcacaaggtgtgcagcggcactgttaatgcctgtgagccc
ggttcgattccggggtctggagcca
1–25tRNA–Arg(tct)[52635,52712]
gcgcgggtagctcaatggacccagagcagccgtcttctacacggtgggtt
gggggttcgaatccctccccgtgcgcca
1–26tRNA–Cys(gca)c[102537,102625]
gcttcggtggactgagaggttaggcgcgggattgcaaatctctgctggtg
ggaacacctatgagggttcgagtccctcccgaagctcca
1–27tRNA–Lys(ctt)c[103205,103278]
tttgcgaatagcacagtggtagtgcaacagcctcttaagctgtcggtcgc
gggttcaatccccgcttcgatgaa
1–29tRNA–Met(cat)[104351,104429]
cgcggggtagaggagtccggttgtcctcgtctggctcataaccaggagat
cgtgggttcaaatcccactcccgctccca
1–30tRNA–Met(cat)[104796,104880]
ggtacggtggctcgacaggtaaggcatcggcctcataagccgagggttcg
tcccaatcctggttcaaatccaggtcgtacctcca
>JQ086372.1 Enterobacteria phage HK446, complete genome
1–1tRNA–Asn(gtt)[35097,35171]
gggtcagtcgtataaaggtcattacggaaggctgttaaccttcttatcgt
ggttcgagtccacgctgtcccgcca
1–2tRNA–Thr(tgt)[35177,35252]
gctggtttagctccaatggtagagcagtcgccttgtaagcgaatgggtag
cggttcaagtccgttaaccagcacca
>JQ182727.1 Enterobacteria phage mEpX1, complete genome
1–1tRNA–Ala(cgc)c[30896,30978]
ataatgtttggcgaggattcagacgccgtatttcgccaatatgaataccc
gttattttcttcagtgcaggccgggccattttc
>HQ141411.1 Lactobacillus phage Sha1, complete genome
1–2tRNA–Lys(ttt)c[32732,32820]
tgcgagcgtagttcaacggtagaacggtactcctttgagttgctgactag
atactatgcagatgcaggttcgactcctaccgctcgcgt
1–3tRNA–Trp(cca)c[33058,33150]
tgcgagcgtagttcaacggtagaacaatattccaagtcttgaagcccatt
ctttcttggactactatgcaggttcgactcctgccgctcgcat
>JQ062992.1 Bacillus phage phIS3501, complete genome

1-1tRNA-Met(cat)c[1571,1644]
ggacctttagctcagttggtcagagcagacggctcataaccgtccggtca
taggttcaaatcctataaggtcca
>HE775250.1 Salmonella phage vB_SenS-Ent1 complete genome
1-1tRNA-Ser(tga)c[5658,5736]
aactggaataattgagggcaaaattaaggccccttgaaggggcctttcgt
tactcaggttctgagaccttagccgcttt
>JX316028.1 Erwinia phage phiEaH2, complete genome
1-1tRNA-Trp(cca)[167025,167100]
aggggcatagctcagctggcagagcaccggtctccaaaaccgggtgtcca
aggttcgaatccttgtgcccctgcca
1-2tRNA-Cys(gca)[167459,167534]
ggggcgatggctcgagcggtaaggcgagtgattgcaaatcacttttaggc
cggttcaaatccggctcgtccctcca
1-3tRNA-Gly(ccc)[169219,169294]
gcgagtatagctcagctggtagagcttctggttcccaaccagacggtcgt
gagttcgaatctcattgctcgctcca
1-4tRNA-Tyr(gta)[169404,169489]
ggcgcggtactcaagtggtcaacgagagcagactgtaaatctgttgcctt
cgggcttcgtaggttcaaatccttccctcgccacca
1-5tRNA-Glu(ttc)[169496,169570]
gttcctgtcgtctagtggtaaggacacctggttttcaaccaggcaaccgg
agttcaattctccgcaggaacgcca
1-6tRNA-Asp(gtc)[169727,169804]
aggattgtagctcagttggtctagagtgccgccctgtcacggcggaggtc
gcgggttcgagccccgtcagtcctgcca
1-7tRNA-Ile(gat)[169814,169889]
tgcgtcttagctcagttggtagagcgcacccctgataagggtgaggtcac
tggttcaagtccagtagatgcaacca
1-8tRNA-Met(cat)[169896,169971]
ggttctgtagcttagctggtagagcacgccactcataatggcgcggtcgt
tggttcgaatccagccagaaccacca
1-9tRNA-His(gtg)[169980,170056]
gtgtatgtagttcagctggttagaatactggcttgtgacgtcagtggtcg
agagttcgagtctctccatacacccca
1-10tRNA-Ser(gct)[170062,170151]
ggagaattgtccgagaggcttaaagagctcccctgctaagggagtggtgg
cgaaagctgcccgtgtgttcgaatcacacattctccgcca
1-11tRNA-Met(cat)[170349,170424]
gagcgatgctgttaagatggttaacacccgactcataatcggatattcgc
aggttcgattcctgtcatcgttcgcc
1-12tRNA-Asn(gtt)[170432,170508]
ggttccatagctcagttggttagagcggctgcctgttaagcagtaggtcc
ctggttcgagtccaggtggtaccgcca

>JX100812.1 Caulobacter phage CcrMagneto, complete genome

1-1tRNA-Trp(cca)[47077,47152]
gtcggtctagctcatggggtagagcggcggtctccaaaaccgcgcgtggc
aggttcgagtcctgcgaccggcgcca
1-2tRNA-Gly(gcc)[47158,47232]
gcgtccatcgtatatgggttattgcttcgcgttgccaacgcgacgaaccg
ggttcgagtcccggtggacgctcca
1-3tRNA-Gly(tcc)[47239,47312]
gcggatatagctcaaagggagagctactgccttccaagcagaagatgcgg
gttcgagccccgctatccgctcca
1-4tRNA-Glu(ttc)[47468,47545]
ggcccggtcgtctagcggtttgcaggattcgtgactttcaatcacgagag
cggagttcgagtctccgtcgggcctcca
1-5tRNA-Asp(gtc)[47569,47643]
ggtgcgttcgtatatcggtcaatactccggattgtcgatccggcaagagg
ggttcgattcccctacgcaccgcca
1-6tRNA-Pro(cgg)[48504,48579]
cggggtgtagctcagtcggtagagcgctcggttcgggaccgagaggccgt
gggttcgagtcccgccacccagacca
1-7tRNA-Pro(ggg)[48587,48661]
cgggatgtagctcagcggtagagcgcgcggctgggggccgcgaggccgca
ggttcgagacctgccaccccgacca
1-8tRNA-Lys(ctt)[48850,48936]
ggatgtgtcacctcagtggcgagaggaccgggctcttaatccggcagcga
aagctcatcgtgggttcgagtcccaccacatcctcca
1-9tRNA-Lys(ttt)[48942,49031]
gcacgagtgacccgagcggcgaagggcgtcgacttttaatcgactgggcg
tccagcctcaccgagggttcgagtccctcctcgtgctcca
1-10tRNA-Leu(cag)[49038,49123]
gcgaaagtggcggaacaaggtagacgcaccggtctcaggtaccggcggct
tcggtcatgagggttcgagtccctcctttcgtacca
1-11tRNA-Leu(caa)[49131,49215]
gccggtctggcggaaatggcagacgcagtggcttcaagtcccaccgcctt
cgggcgtcccggttcgatgccggggaccggcacca
1-12tRNA-Leu(gag)[49222,49309]
tgcgaagtggcggaatttggtagacgcaccggcatgagaggccggcgccc
ttcggggcgtgagggttcgagtcccttcttcgcgacca
1-13tRNA-Ala(tgc)[49400,49470]
ggggtatgcgcagggcgcaggcatcctttgcaaggaagtcggggtgggtt
cgagtcccacatactccacca
1-14tRNA-Ser(cga)[49475,49562]
ggtaggttggctgagaggcctaaggcgcccgtttcgaaaacgggagaacc
gcaaggttccgtgggttcgaatcccacacctacctcca
1-15tRNA-Ser(gga)[49567,49654]

ggatgtatggctgagaggccgaaggcgcccgcttggaaagcgggtgaacc
gcaaggttccgtaggttcgaatcctactgcatccgcca
1-16tRNA-Phe(gaa)[50349,50424]
ggactcgtagctcagcaggtagagcgccggcttgaaaccccggaggtcgg
aggttcgactcctcccgtttccacca
1-17tRNA-Val(gac)[50430,50505]
gcccgatgagccagatgggaaggcgccgccgtgacatggcgggattgaga
aggttcgattccttcatcaggcacca
1-18tRNA-Ile(gat)[50508,50583]
gcaagtgtagctcagttggtagagcagcggaccgataatccgcccgtccc
tggttcgagtccaggcatttgcacca
1-19tRNA-Ile(gat)[50591,50665]
tgtggcatagctcaggggtagagcgcgcgcttgataagcgtgaggtcggg
ggttcgaagccctctgccactacca
1-20tRNA-Val(cac)[50699,50774]
tggcgtttagcatagtgggagtgcaggggtgtcacatacctcgatcgaac
tggttcgattccagtaacgccaacca
1-21tRNA-Gln(ctg)[50780,50853]
tgagggttcgtctaatggtaggacggctgggtctggtccagctaatctag
gttcgagtcctagcccctcatcca
1-22tRNA-Gln(ttg)[50861,50934]
tctcgggtggtctaatggtaagacatctggttttggtccagaggattggg
gttcgattccttggccgagaacca
1-23tRNA-Ile(aat)[50950,51024]
tccagtcaatgcacaaggtgtgcagcggcactgttaatgcctgtgagccc
ggttcgattccggggtctggagcca
1-24tRNA-Arg(tct)[51227,51304]
gcgcgggtagctcaatggacccagagcagccgtcttctacacggtgggtt
gggggttcgagtccctccccgcgcgcca
1-25tRNA-Cys(gca)c[100693,100781]
gcttcggtggactgagaggttaggcgcgggattgcaaatctctgctggtg
ggaacacctatgagggttcgagtccctcccgaagctcca
1-26tRNA-Lys(ctt)c[101361,101434]
tttgcgaatagcacagtggtagtgcaacagcctcttaagctgtcggtcgc
gggttcaatccccgcttcgatgaa
1-28tRNA-Met(cat)[102508,102586]
cgcggggtagaggagtccggttgtcctcgtctggctcataaccaggagat
cgtgggttcaaatcccactcccgctccca
1-29tRNA-Met(cat)[102996,103080]
ggtacggtggctcgacaggtaaggcatcggcctcataagccgagggttcg
tcccaatcctggttcaaatccaggtcgtacctcca
>HE956708.2 Yersinia phage phiR201 complete genome
1-1tRNA-Met(cat)c[28563,28638]
agttagttggcagagcggttatgcacctccctcatacggagcgactacag

tggttcaaatccactactaactacca
1-2tRNA-Ile(gat)c[28645,28718]
gcccgattagttcaatggtagaacagagagccgataactctcagacctag
gatcgtaacctagattgggtacca
1-3tRNA-Thr(tgt)c[29529,29603]
gctcctaaagcattgctggcgatgcagttgccttgtaagcatctgaaccg
ggttcgattcctggtgggagcacca
1-4tRNA-Gly(tcc)c[30008,30082]
gcgtgattagttcagtggctagaataactggcttccaaccagtagacacg
agttcgactctcgtatcccgcacca
1-5tRNA-Gln(ttg)c[30089,30164]
tggagagtagtgtaacggttagcacaacggcctttgactccgttaatggt
aggttcgattcctccttctccagcca
1-6tRNA-Gln(ctg)c[30171,30246]
tgggatgtagatcaattggcagatcgtcggcctctgactccgaaggttcc
acgttcgatccgtggcatcccagcca
1-7tRNA-Arg(acg)c[30481,30555]
gcgtccttatttcaacggaaagaatgtaaagctacgaactttacgatcgg
ggttcgattccctgaggatgcacca
1-8tRNA-His(gtg)c[30562,30638]
gtggctatatcataattggttaatgatcctgattgtgaatcaggcctatg
tggattcgaattccactagccacccca
1-9tRNA-Leu(tag)c[31489,31565]
gcgtgattgatggaattggcatacataccgtccttagaagtcgggttttg
agggttcgaatcccttgtcacgcacca
1-10tRNA-Ala(tgc)c[31571,31649]
ggggaatgggtctgcatggagtggacacctcgcttgcaccgaggacatca
gaacggttcgattccgttattctccacca
1-11tRNA-Val(tac)c[32384,32457]
gctcggttagtttaatgggagaaccccgtctttacacggcggttgcgata
gttcgattctatcaccgagtacca
1-12tRNA-Lys(ttt)c[32462,32539]
ggattgctagctcaactggataagagtagcggacctttaatccgtcagtt
ctgggttcgaatcccaggcaatccacca
1-13tRNA-Met(cat)c[33179,33256]
tgcgggttagatctctggtagagatcgctagtctcataagctagaaagag
gtaggttcgattcctgcacccgcttcca
1-14tRNA-Pro(tgg)c[33263,33340]
ctccgtgtagctcagtttggccagagcgttccgtttggggcggtagggtc
tggggttcaaatcctcccacggagacca
1-15tRNA-Lys(ctt)c[33686,33762]
acatccatagctcaataggttagagctaccgcctcttaagcggagggttc
taggttcaagtcctagtggatgtacca
1-16tRNA-Asp(gtc)c[33911,33987]

gcgatcggggctggcttggtaatggtactcccctgtcacgggagagaatg
tgggttcgaatcccatcggtcgcgcca
1–17tRNA–Asn(gtt)c[34433,34508]
gagagaagcagtaagtggtataggcggtcgcctgttaagcgaatgacagt
gagttcgaatctcacctctcccgcca
1–18tRNA–Cys(gca)c[34516,34590]
gcatccttgtccgagtggtttaggtgacggtctgcaaaatcgtctaagtt
ggttcaattccaacagggtgctcca
1–19tRNA–Trp(cca)c[35423,35499]
attcctagagtgttactggacagcatgtcggtctccaaaaccgtacggtc
taggttcgagtcctagtaggtttgcca
1–20tRNA–Glu(ttc)c[35508,35584]
gtcctgttagacaaactggtaaagtcactaccctttcaaggtaggatttg
cgggttcgatccccgcacaggacgcca
1–21tRNA–Tyr(gta)c[35592,35682]
gttggattagtatcgtagaggtagcgaagcagactgtaaatctgccgact
cggaagggtctcgggtggttcgactccatcatccaacacca
1–22tRNA–Leu(taa)c[36601,36686]
ggatctgtggtggaattggcaatacacaccatgcttaaaacgtggcgctt
aaatgattgagggttcgagcccctccagatccacca
1–23tRNA–Met(cat)c[37254,37331]
ggttctcaagctcatttggtatgagccgtcgcctcataagcgaaaggtag
gtaggttcgaatcctccgggagccacca
1–24tRNA–Ser(gct)c[37337,37431]
ggaagattaaccctaattggtaagggagcatcttgctaagttgccagtag
ctgtggaaacacggtgtatcggttcaagtccgatatcttcctcca
1–25tRNA–Arg(tct)c[40505,40581]
gctccgttggccaaattggataaggcaagatcgttctaagttcttgatta
ggggttcgaatcccttacggagtacca
1–26tRNA–Ser(cga)c[41640,41713]
ctgacattagcttaatatggtagagcgtctccgggcgaaccgggggaaat
gcaggttcgagccctgaggtcaga
>JF974287.1 Vibrio phage pYD21–A genomic sequence
1–1tRNA–Gly(tcc)[10451,10526]
gcgacattagctcaggtggtagagcagaagttttccaaacttaatgtcat
cggttcgaatccgatatgtcgctcca
>HQ633071.1 Synechococcus phage S–SKS1 genomic sequence
1–1tRNA–Gly(tcc)c[52996,53066]
gcggaattagtttagaggcaaaactaaaggtttccaacctttcgtcacca
gttcgattctggtattccgct
1–2tRNA–Lys(ttt)c[53142,53215]
gggtttataactcagttggtagagtatcgggcttttaacctgcaagtcgt
cagttcgagcctgactaaacccac
1–3tRNA–Thr(tgt)c[53239,53310]

gcctttgtagctcagtggtagagcagggcttttgtaaagctcaggtcgca
agttcaaatcttgtcgaaggct
1-4tRNA-Ser(gct)c[53313,53402]
cggaggattggctgagtggtcgaaagcaacagtttgctaaattgtcgaag
gtgtaaatcttccgttggttcgaatccaacatcctccgtc
1-5tRNA-Ile(gat)c[53406,53481]
tgcccgaatagctcagatggttagagcacaccgctgataacggtgaggtc
tctggttcgagtccggattcgggcat
1-6tRNA-Ser(tga)c[53484,53569]
ggagaggtggtcgagtggtttatggctctggtcttgaaaaccagcgaggt
gaaagcctccgtgagttcgaatctcaccctctccgc
1-7tRNA-Ala(tgc)c[53740,53814]
tggggaattagctcagttggtagagcgcctgctttgcaagtaggatgtca
acggttcgagtccgttattctccat
1-8tRNA-Pro(tgg)c[53815,53888]
tcggggtgtagcgcagaggtagcgcatcgtttttgggaagcgagggtcgc
aggttcgatccctgccaccccgat
1-9tRNA-Asn(gtt)c[53927,53998]
tcccctgtagctcagcggtagagtcggtgactgttaatcactttgtcgca
agttcgaatcttgccgggggag
1-10tRNA-Arg(tct)c[54002,54077]
gggcaagtagctcagatggacagagccacgcacttctaatgcgttggtcg
ggggttcgagtccctccttgcctgcc
1-11tRNA-Leu(taa)c[178217,178293]
tgcccgtgtagtccaattggcagtagacacgaaacttaaaatttcgacag
tatcggttcgagtccgatcacgggtat
1-12tRNA-Met(cat)c[178433,178507]
tgcaggtttaccaatctggtgaatggaccgttctcataaaacggcgaagg
tgggttcaattcccacaacctgcat
1-13tRNA-Ile(tat)c[178508,178598]
tgctcgtgagacttggtagtcaggggagtcttataaactctttgcacccg
attagtgcctttgagatcgttcaaatcgatccacgagtatc

>JX899358.1 Mycobacterium phage First, complete genome
1-1tRNA-Gln(ctg)[4425,4503]
ttcccgactagcacaactggtagctgcgctcggctctggaccgagaggtt
tgaggttcgaatccttggtcgggagccca

>KC661279.1 Mycobacterium phage Severus, complete genome
1-1tRNA-Trp(cca)[1682,1755]
aggcatgtagctcaattggtagagcagcggtctccaaagccgccggttgc
aggttcgagtcctgccgtgtctgc
1-2tRNA-Gln(ctg)[1761,1833]
tcctcgttcgtctaatcggtaggacaccaggctctggacctgggggttga
ggttcgagtccttggcgaggagc
1-3tRNA-Asn(gtt)[1868,1941]

tgacgtgtagctcaaccggcagagcaggcggctgttaaccgcccggttgg
aggttcgagtcctccctcgtcagc
>KC172839.1 Mycobacterium phage BTCU-1, complete genome
1-1tRNA-Trp(cca)[3883,3956]
aggcgcgtagctcaattggtagagcggcggtctccaaagccgctggctgc
aggttcgagtcctgccgcgtctgc
>KC182552.1 Lactococcus phage phi7, complete genome
1-1tRNA-Trp(cca)[31441,31513]
tgcgagcatagtataatggtaatgccacagattccaaacctgtaaacgtg
ggttcgattcctactgttcgtgt
1-2tRNA-Asp(gtc)[31571,31642]
taggatatagccaaattggtatgtggtaggcagagtcgcaatctggtact
ggttcgattccagttgtcctag
1-3tRNA-Pro(tgg)[31699,31770]
caggatatagtgttaatggtagcatgcgtgttttgggaacatgtagtgtt
ggttcgagtccagctatcctga
>KC748969.1 Mycobacterium phage Phaux, complete genome
1-1tRNA-Arg(cct)[63838,63921]
atggcccgtagctcagtcaggtagagcagccttgccccttgcgggcttgg
ttcgtccccggttcaaatccgggcgggccttgcc
1-2tRNA-Gly(tcc)[63924,63996]
gcgcccgtggtcgaattggaaagactcctggcttccacccaggttatgca
ggttcgagtcctgtcgggcgctc
>KC661277.1 Mycobacterium phage Phrux, complete genome
1-1tRNA-Arg(cct)[61916,61999]
atggcccgtagctcagtcaggtagagcagccttgccccttgcgggcttgg
ttcgtccccggttcaaatccgggcgggccttgcc
1-2tRNA-Gly(tcc)[62002,62074]
gcgcccgtggtcgaattggaaagactcctggcttccacccaggttatgca
ggttcgagtcctgtcgggcgctc
>KC787106.1 Mycobacterium phage Chy5, complete genome
1-1tRNA-Asn(gtt)[4562,4636]
tgacgtgtagctcaatggcagagcatccggctgttaaccggacggttgaa
ggttcgagtccttcctcgtcagcca
1-2tRNA-Trp(cca)[4677,4752]
gtacacgtagctcaattggtagagcagtggtctccaaagccgccggttcc
aggttcgactcctggcgtgtatgcca
1-3tRNA-Gln(ctg)[4760,4832]
tccccgttcgtctaatcggtaagacgcctggctctggaccaggtaattga
ggttcgagtccttggcggggagc
1-4tRNA-Glu(ctc)[4867,4940]
ggtcccttggagtagcggataactcacctggccctcacccagaagatcgc
gggttcgaatcccgcagggactac
1-5tRNA-Tyr(gta)[4944,5024]

cgcgagatacccaagcggcaacgggatctgactgtaaatcagacgcttcg
gcttcgcaggttcgagtcctgctctcgcgac
>KC821607.1 Cellulophaga phage phi19:1, complete genome
1-1tRNA-Pro(tgg)[31057,31131]
ctaagtgcgatccattggtgtggttacccgacttggaatcgggattattg
taggttcgagtcctatcacttagac
1-2tRNA-Gln(ttg)[31189,31261]
tgaaatatgatgtaattggcagcatgcgagactttgactctcgtcgttta
ggttcgagtcctaatatttcaac
1-3tRNA-His(gtg)[31268,31341]
gtgtttatagtataattggttaatgcgctagactgtgaatctagatactt
gatttcgattctcaataaacaccc
1-4tRNA-Leu(tag)[31346,31420]
gcttcagtagtcgaattggcatagacaacaggcttagaccctgaaatttg
agagttcgagtctctcctgaagtac
1-5tRNA-Arg(acg)[31424,31496]
ctttttgtagtttaacggataaaacaattggctacgaactaatagataac
agttcgattctgttcaaaaagtc
>KC748971.1 Mycobacterium phage Murphy, complete genome
1-1tRNA-Arg(cct)[62896,62979]
atggcccgtagctcagtcaggtagagcagccttgccccttgcgggcttgg
ttcgtccccggttcaaatccgggcgggccttgcc
1-2tRNA-Gly(tcc)[62982,63054]
gcgcccgtggtcgaattggaaagactcctggcttccacccaggttatgca
ggttcgagtcctgtcgggcgctc
>KC701493.1 Mycobacterium phage CASbig, complete genome
1-1tRNA-Trp(cca)c[51136,51210]
tggctcttagctcaatttggtagagcagcggtctccaaaaccgccggttg
caggttcgagtcctgcagagtcagc
>KC237729.1 Clostridium phage vB_CpeS-CP51, complete genome
1-2tRNA-Ser(gct)[36204,36295]
ggaaagttactcaagttggttaagagggcagattgctaatctgttagtag
gctttatgtctagcaagagttcaaatctcttactttccgcca
>KC661281.1 Mycobacterium phage Jobu08, complete genome
1-1tRNA-Asn(gtt)[3904,3979]
tgacgtgtagctcaatcggcagagcacccggctgttaaccgggcggttga
aggttcgagtccttccatgtcagcca
1-2tRNA-Trp(cca)[4020,4094]
aggcacgtagctcaattggtcagagcagcggtctccaaagccgccggctg
caggttcgagtcctgccgtgtctgc
1-3tRNA-Leu(cag)[4129,4203]
ggctcggtaggcaaacaggcaaagccgcctgtctcaggaacaggtgcgtg
ggggttcgactccctcccgagctac
>KC182546.1 Lactococcus phage jm2, complete genome

1−1tRNA−Trp(cca)[30552,30624]
tacgaacatagtatagtggtaatgctacagattccaaacctgtaaacgtg
ggttcgattcctgctgttcgtgt
>KC691255.1 Mycobacterium phage Dumbo, complete genome
1−1tRNA−Arg(cct)[62229,62312]
atggcccgtagctcagtcaggtagagcagccttgccccttgcgggcttgg
ttcgtccccggttcaaatccgggcgggccttgcc
1−2tRNA−Gly(tcc)[62315,62387]
gcgcccgtggtcgaattggaaagactcctggcttccacccaggttatgca
ggttcgagtcctgtcgggcgctc
>KC900379.1 Mycobacterium phage PegLeg, complete genome
1−1tRNA−Arg(ccg)[32354,32449]
ctccacgtggcgaatggcaatgtggcagaggggattccggttcccgaggg
gcgggggatctcagacttccgcctgcgttcgactccgtcgtggtgg
1−2tRNA−Trp(cca)[54522,54593]
atgggcgtagctgactgggaagcaacggtctccaaagccgtgatatgcag
gttcgaatcctgccgcccgtgc
1−3tRNA−Asn(gtt)[55442,55513]
tggcctgtcggttaacggcaaaccagcgccctgttaaggcgtgactcctg
gttcgaatccaggtaggccagc
1−4tRNA−Gln(ttg)[56269,56340]
cgggacatagctcaaatggcagagtggggcaattggtgccctggattcag
gttcgactcctggtggcccgac
1−5tRNA−Tyr(gta)[56725,56807]
gcggtctctcccctattggcgaagggcccctggctgtaacccagggcttt
cgagtgtggtggttcgaatccatcaggccgcac
1−6tRNA−Gln(ctg)[56976,57048]
tgctctctcgtctaactggcaggacaccagaatctgactctggcagttga
ggttcgaatccttggggagcaac
1−7tRNA−Pro(tgg)[57052,57125]
ctgggtgaggtgtaagtggttgcatggccggtttggatccgggcggttcg
aggttcgagtcctcgcacccagac
1−8tRNA−Ser(gct)[57421,57502]
gagtggaaccatctaggtggatggccgcgattgctaatcgtggggcagcg
taacgctgtgtggatcgtgcccactcctctcc
1−9tRNA−Phe(gaa)[57612,57687]
cgggttgtagctcagattggtgagagcgctcggttgaaacccgagaggcc
cctggttcgaatccaggcatcccggc
1−10tRNA−Met(cat)[58249,58324]
tgcggtgtagagcagttcggagtgctcgcttggctcataaccaagaggcc
gtcggttcaaatccgaccaccgcaac
1−11tRNA−Arg(acg)[58604,58678]
cccgccgcagcacgacggacgtgcacctcgctacgaacgagaaggctgct
ggttcgaatccagtcggtgggtcca

1-12tRNA-His(gtg)[58681,58753]
cctcttgtagctcaatggtgagagcgccggtttgtgagaccggtgacctg
cgttcgattcgcagcagggggac
1-13tRNA-Leu(cag)[59015,59089]
ggctcgctaggcaaactggcaaagccgcctgattcagaatcaggtgcttc
cgagttcgactctcgggcgagctac
1-14tRNA-Lys(ctt)[60212,60285]
gggacgttggtgaaacggctatcaccgcacgctcttaacgtgtcagttct
gggttcgaatcccaggcgtcccac
1-15tRNA-Gly(tcc)[60444,60520]
gcgtgtgtgatgtttaacggctagcattcctgtcttccaaacaggctgtg
cgggttcgaatcccgccacgcgttcca
1-16tRNA-Val(tac)[60573,60644]
gccccgctcgtctaatgggaaggccgctgcgttacaagcagcagacggga
gttcgattctctcgcggggtac
1-17tRNA-Thr(agt)[60706,60778]
gccggtatagctcagtggctagagcacctgtctagtaaacaggagaccag
ggttcgattccctgttccggctc
1-18tRNA-Asp(gtc)[60894,60967]
cccgatgtcatctagtggaccaggatgccgccctgtcgaggcggtcacgc
gagttcgaatctcgtcgtcgggac
1-19tRNA-Glu(ctc)[61355,61429]
gttcccgtggagtaattggttatctcgcctgactctcaatcaggagattg
cgcgttcaagtcgcgtcgggaatac
>KC787105.1 Mycobacterium phage Chy4, complete genome
1-1tRNA-Asn(gtt)[4220,4294]
tgacgtgtagctcaatggcagagcatccggctgttaaccggacggttgaa
ggttcgagtccttcctcgtcagcca
1-2tRNA-Trp(cca)[4335,4410]
gtacacgtagctcaattggtagagcagtggtctccaaagccgccggttcc
aggttcgactcctggcgtgtatgcca
1-3tRNA-Gln(ctg)[4418,4490]
tccccgttcgtctaatcggtaagacgcctggctctggaccaggtaattga
ggttcgagtccttggcggggagc
1-4tRNA-Glu(ctc)[4525,4598]
ggtcccttggagtagcggataactcacctggccctcacccagaagatcgc
gggttcgaatcccgcagggactac
1-5tRNA-Tyr(gta)[4602,4682]
cgcgagatacccaagcggcaacgggatctgactgtaaatcagacgcttcg
gcttcgcaggttcgagtcctgctctcgcgac
>KC821618.1 Cellulophaga phage phi10:1, complete genome
1-1tRNA-Tyr(gta)[31599,31683]
ggggtggttgctcggctggaagaggcgcaggactgtaaattctgtaatcc
actaggaatggaggttcgagtcctcctcaccccac

1-2tRNA-Phe(gaa)[31767,31840]
ggaaatatagctcagttggtagagcgtcgcattgaagatgcgagcgtcgg
cagttcgaatctgtctatttccac
>HE962497.1 Streptococcus phage SP-QS1 complete genome
1-1tRNA-Trp(cca)[24820,24891]
gagcggttagtgtaatggttagcacaacagtctccaaaactgttagagag
ggttcgattccttcactgcttg
>KF306380.1 Mycobacterium phage DrDrey, complete genome
1-1tRNA-Arg(cct)[63869,63952]
atggcccgtagctcagtcaggtagagcagccttgccccttgcgggcttgg
ttcgtccccggttcaaatccgggcgggccttgcc
1-2tRNA-Gly(tcc)[63955,64027]
gcgcccgtggtcgaattggaaagactcctggcttccacccaggttatgca
ggttcgagtcctgtcgggcgctc
>KF416344.1 Mycobacterium phage Adzzy, complete genome
1-1tRNA-Asn(gtt)[4021,4095]
tgacgtgtagctcaacggcagagcacccggctgttaaccggacggttgaa
ggttcgaatccttccacgtcagcca
1-2tRNA-Gln(ctg)[4132,4204]
tctcctttcgtctaatcggtaagacacctggctctggaccaggcaattga
ggttcgagtccttgagggagagc
1-3tRNA-Lys(ctt)[4239,4312]
ggggcagtagctcagtcggtagagctgcggactcttaatccgtaggtcgc
aggttcgatccctgcctgccccac
>KF024731.1 Mycobacterium phage Crossroads, complete genome
1-1tRNA-Thr(cgt)[63172,63245]
gccaccttagctcagttggtagagcagcgccttcgtaacgcgcaggtcag
cggttcgattccgctaggtggctc
1-2tRNA-Pro(tgg)[63707,63780]
cggcccaaggtgtaatggcttgcatgaggcgtttggatcgccggggttcc
ctgttcgactcggggtgggccgac
1-3tRNA-Trp(cca)[63851,63924]
agcgaattggtgtaatcggtagcatagcggcctccaaaaccgcctggtag
aggttcgagtcctctattcgttgc
1-4tRNA-Leu(tag)[64297,64371]
gctccgctaggcaaactggcaaagccgcctgatttaggttcaggtgtttc
ggggttcgactccccggtggagcac
1-5tRNA-His(gtg)[64371,64443]
cgcgaggtagcttaagtagtaaagcccccgactgtgactcgggtgattcg
ggtgcaagtcccggcctgtcgcc
1-6tRNA-Tyr(gta)[64446,64528]
gtggcggttgggcttgttggttggcccgcctgactgtaaatcaggtgttt
cggcatcgggggttcgattccctctcgccacac
1-7tRNA-Gln(ctg)[64536,64611]

tgaccgctagcacaattaggcagttgcgccggaatctgactccggaggtt
ccaggttcgattcctgggtggtcagc
1−8tRNA−Gly(tcc)[64727,64801]
gtgctagtaacccatgttggctgggtgccactcttccaaagtggatttcg
cgcgttcgattcgcgtctagcacac
1−9tRNA−Lys(ctt)[64836,64911]
gcctcgttagctcagttggtagagcagccgactcttaatcggcgggtcac
aggttcaagtcctgtacggggtacca
1−10tRNA−Ser(tga)[64913,65002]
ggagagtaatgcagcgggggtggcccgcgaccggccttgaaagccgggta
gccgtaatggagcttggggttcgatccctctgctctccgc
1−11tRNA−Cys(gca)[66093,66165]
gccgttgtgtccgagtggttaggtgccaggctgcaaacttggttagtcgc
gttcgattcgcggggacggctcc
1−12tRNA−Asn(gtt)[66434,66507]
tcctccgtagctcaattggcagagcgcgcgactgttaatcgcgtggttgg
tggttcgagtccacccgttggagc
1−13tRNA−Lys(ttt)c[66701,66775]
gcccctatagctcagttggtagagcaggagacttttaatcttcgggtcct
aggttcgatccctagtgggggcacc
>KF024725.1 Mycobacterium phage Whirlwind, complete genome
1−1tRNA−Tyr(gta)[39380,39463]
gtggcgaaggcaactgttggtttgttgcacctgcctgtaaagcaggcgct
tcggcttcgggggttcaattccctctcgccacac
1−2tRNA−Thr(cgt)[63038,63110]
gccaccttagctcaggggtagagcagcgccttcgtaacgcgcaggtcaac
ggttcgattccgttaggtggctc
1−3tRNA−Pro(tgg)[63469,63543]
ctggttgtagcgcagcttggtagcgcgcctgatttggattcagggggtcg
gaggttcaaatccttccagccagac
1−4tRNA−Gly(tcc)[63884,63958]
gcgctagctacccctgttggcggggtgcctgccttccaagcaggatcacg
cgggttcgattcctgtctagtgctc
1−5tRNA−Trp(cca)[64013,64086]
agctcggtagctcaactggtagagcagtggtctccaaagccaccggttgc
aggttcgactcctgtccgggttgc
1−6tRNA−Leu(tag)[64087,64161]
ggttccgtaggcaaactggcaaagccgtctgacttagaatcaggtgtttg
ggagttcgactctccccggaactac
1−7tRNA−His(gtg)[64162,64231]
gctaagtagcttaatggtaaagcccccggttgtggtccgggcgatcacgg
ttcaactccgtgcttaagcc
1−8tRNA−Gln(ctg)[64513,64588]
tgcccgctagcacaatttggcagttgcgcccggctctgaaccgggaggtt

ccaggttcgactcctgggtgggcagc
1-9tRNA-Lys(ctt)[64717,64790]
gcgtcgttagctcagttggtagagcagccgactcttaatcggcgggtcgg
gggttcaagtccctcacggcgcac
1-10tRNA-Asn(gtt)[65920,65992]
tcccctgtcatttaaccggcaggatgcctcgctgttaacgaggtcgtgca
ggttcgatccctgctgggggagc
1-11tRNA-Lys(ttt)c[65997,66070]
gccgcgttagctcagttggtagagctgctgacttttaatcagtaggtccg
gggttcgagtccccgacgcggtac
>KF114875.1 Mycobacterium phage Redno2, complete genome
1-1tRNA-Gly(tcc)[90864,90936]
gcgcttgtggtcgagttggaaagactcctggcttccacccaggttatgca
ggttcgagtcctgtcgagcgctc
>KF279417.1 Mycobacterium phage Quink, complete genome
1-1tRNA-Arg(cct)[63291,63374]
atggcccgtagctcagtcaggtagagcagccttgccccttgcgggcttgg
ttcgtccccggttcaaatccgggcgggccttgcc
1-2tRNA-Gly(tcc)[63377,63449]
gcgcccgtggtcgaattggaaagactcctggcttccacccaggttatgca
ggttcgagtcctgtcgggcgctc
>KF562100.1 Mycobacterium phage HufflyPuff, complete genome
1-1tRNA-Arg(cct)[63504,63587]
atggcccgtagctcagtcaggtagagcagccttgccccttgcgggcttgg
ttcgtccccggttcaaatccgggcgggccttgcc
1-2tRNA-Gly(tcc)[63590,63662]
gcgcccgtggtcgaattggaaagactcctggcttccacccaggttatgca
ggttcgagtcctgtcgggcgctc
>KF562099.1 Mycobacterium phage Bruin, complete genome
1-1tRNA-Arg(cct)[61837,61920]
atggcccgtagctcagtcaggtagagcagccttgccccttgcgggcttgg
ttcgtccccggttcaaatccgggcgggccttgcc
1-2tRNA-Gly(tcc)[61923,61995]
gcgcccgtggtcgaattggaaagactcctggcttccacccaggttatgca
ggttcgagtcctgtcgggcgctc
>KC182547.1 Lactococcus phage jm3, complete genome
1-1tRNA-Trp(cca)[27491,27563]
tgcgaacatagtatagtggtaatactacagactccaaacctgtaaacgtg
ggttcgattcctgctgttcgtgt
>KF024721.1 Mycobacterium phage AnnaL29, complete genome
1-1tRNA-Trp(cca)[6178,6251]
gggcacgtagctcaatcggtagagcagcggtctccaaagccgcaggttcc
aggttcgaaccctggcgggctcgc
>KF024732.1 Mycobacterium phage Contagion, complete genome

1−1tRNA−Arg(cct)[62199,62282]
atggcccgtagctcagtcaggtagagcagccttgccccttgcgggcttgg
ttcgtccccggttcaaatccgggcgggccttgcc
1−2tRNA−Gly(tcc)[62285,62357]
gcgcccgtggtcgaattggaaagactcctggcttccacccaggttatgca
ggttcgagtcctgtcgggcgctc
>KF006818.1 Mycobacterium phage Wanda, complete genome
1−1tRNA−Gly(tcc)[91369,91441]
gcgcttgtggtcgagttggaaagactcctggcttccacccaggttatgca
ggttcgagtcctgtcgagcgctc
>KF017001.1 Mycobacterium phage LittleCherry, complete genome
1−1tRNA−Trp(cca)[4311,4384]
gggtcagtagctcaactggaagagcagcggtctccaaagccgcgagttgg
aggttcgagtcctccctggcctgc
>KF560330.1 Mycobacterium phage Conspiracy, complete genome
1−1tRNA−Trp(cca)[4132,4205]
gggtcagtagctcaattggtagagcagcggtctccaaagccgcgagttgg
aggttcgagtcctccctggcccgc
>KF562102.1 Mycobacterium phage PhatBacter, complete genome
1−1tRNA−Arg(cct)[63278,63361]
atggcccgtagctcagtcaggtagagcagccttgccccttgcgggcttgg
ttcgtccccggttcaaatccgggcgggccttgcc
1−2tRNA−Gly(tcc)[63364,63436]
gcgcccgtggtcgaattggaaagactcctggcttccacccaggttatgca
ggttcgagtcctgtcgggcgctc
>KF416343.1 Myobacteriophage Goku, complete genome
1−1tRNA−Arg(cct)[62731,62814]
atggcccgtagctcagtcaggtagagcagccttgccccttgcgggcttgg
ttcgtccccggttcaaatccgggcgggccttgcc
1−2tRNA−Gly(tcc)[62817,62889]
gcgcccgtggtcgaattggaaagactcctggcttccacccaggttatgca
ggttcgagtcctgtcgggcgctc
>KF279414.1 Mycobacterium phage Fredward, complete genome
1−1tRNA−Lys(ctt)[4055,4128]
ggggcagtagctcagtcggcagagctacggactcttaatccgcaggtcgc
aggttcgatccctgcctgccccac
>KF560334.1 Mycobacterium phage Zaka, complete genome
1−1tRNA−Asn(gtt)[5402,5474]
tgacgtgtagctcaatggcagagcacccggctgttaaccggacggttgaa
ggttcgagtccttccatgtcagc
1−2tRNA−Trp(cca)[5479,5552]
gcgctcctagctcaattggtagagcagcggtctccaaagccgcgagttcc
aggttcgaatcctggggagcgtgc
1−3tRNA−Gln(ctg)[5594,5666]

tccctgttcgtctaatcggtaagacaccgggctctggatccggtaatcga
ggttcgagtccttgatggggagc
>KF562101.1 Mycobacterium phage Nala, complete genome
1−1tRNA−Arg(cct)[63248,63331]
atggcccgtagctcagtcaggtagagcagccttgccccttgcgggcttgg
ttcgtccccggttcaaatccgggcgggccttgcc
1−2tRNA−Gly(tcc)[63334,63406]
gcgcccgtggtcgaattggaaagactcctggcttccacccaggttatgca
ggttcgagtcctgtcgggcgctc
>KF713486.1 Mycobacterium phage Validus, complete genome
1−1tRNA−Trp(cca)[996,1069]
gggtgtgtagctcaactggtagagcagcggtctccaaagccgccggttgc
acgttcgactcgtgccgcgcccgc
>KJ156985.1 Mycobacterium phage RhynO, complete genome
1−1tRNA−Trp(cca)[3811,3884]
aggcacgtagctcaattggaagagcagcggtctccaaagccgccggctgc
aggttcgagccctgccgtgtctgc
1−2tRNA−Tyr(gta)[3919,4001]
cgcgtgatacccaagcggcaacgggacctggctgtaaaccaggcgcttcg
gcttcggaggttcgagtcctcctcacgcgacca
>KJ410134.1 Mycobacterium phage CRB1, complete genome
1−1tRNA−Gln(ctg)[4415,4492]
ttcccggctagcacaacggtagatgcgctcggctctggaccgagaggttt
gaggttcgaatccttggccgggagccca
>KF669650.1 Clavibacter phage CN1A, complete genome
1−1tRNA−Met(cat)c[50108,50184]
tgcggagtagagcagttcggtagctcgccaggctcatatcctggaggccg
cgggttcaaatcccgcctccgcaacca
1−2tRNA−Trp(cca)c[50230,50303]
acacctctagctcaactggtagagcagcggcctccaaaaccgtaggttct
gagttcaaatctcggggggtgtgc
>KF861510.1 Mycobacterium phage EagleEye, complete genome
1−1tRNA−Trp(cca)[5584,5657]
gcacatgtagctcaattggtagagcagcggtctccaaagccgcgggttcc
aggttcgagtcctggcgtgtgtgc
1−2tRNA−Tyr(gta)[5661,5741]
cgcgagatacccaagcggcaaagggatctgactgtaaatcagacgcttcg
gcttcgcaggttcgagtcctgctctcgcgac
>JF770475.1 Escherichia phage phiEB49, complete genome
1−1tRNA−Val(aac)c[43830,43906]
ggcccgttaggcttacaggataagattcccgtacttaacaagtatcgatg
ctggttcgaatccagtgcgggccacca
1−2tRNA−Arg(cct)c[43908,43983]
gttctgctggcgtaacaggataacgcggagacctcctaagtctcagttgc

tggttcgagtccagcgcggaacacca
>KF493882.1 Mycobacterium phage Jovo, complete genome
1-1tRNA-Trp(cca)[4423,4496]
gggtcagtagctcaactggaagagcagcggtctccaaagccgtgcgttgg
aggttcgagtcctccctggcccgc
>KF560332.1 Mycobacterium phage CloudWang3, complete genome
1-1tRNA-Asn(gtt)[5402,5474]
tgacgtgtagctcaatggcagagcacccggctgttaaccggacggttgaa
ggttcgagtccttccatgtcagc
1-2tRNA-Trp(cca)[5479,5552]
gcgctcctagctcaattggtagagcagcggtctccaaagccgcgagttcc
aggttcgagtcctggggagcgtgc
1-3tRNA-Gln(ctg)[5594,5666]
tccctgttcgtctaatcggtaagacaccgggctctggacccggcaatcga
ggttcgaatccttgatggggagc
>KF560333.1 Mycobacterium phage Artemis2UCLA, complete genome
1-1tRNA-Asn(gtt)[5285,5357]
tgacgtgtagctcaatggcagagcacccggctgttaaccggacggttgaa
ggttcgagtccttccatgtcagc
1-2tRNA-Trp(cca)[5362,5435]
gcgctcctagctcaattggtagagcagcggtctccaaagccgcgagttcc
aggttcgagtcctggggagcgtgc
1-3tRNA-Gln(ctg)[5477,5549]
tccctgttcgtctaatcggtaagacaccgggctctggacccggcaatcga
ggttcgagtccttgatggggagc
>KC688701.1 Croceibacter phage P2559Y, complete genome
1-1tRNA-Cys(gca)[26696,26783]
ggggtggtagcttaattggttaaagcgtgggtttgcaaacattaaaacat
gataccaaaattgtaggttcgagtcctactcactccac
>KF929199.1 Staphylococcus phage vB_SepS_SEP9, complete genome
1-1tRNA-Pro(tgg)[5064,5137]
cagaatgtagttcaatttggtagaataccacatttgggatgtggtggttg
ttggttcaaatccagccattctga
>KC481682.1 Bacillus phage vB_BanS-Tsamsa, complete genome
1-1tRNA-Arg(tct)c[148615,148690]
tacctcagaagcacaattggatgtgcaggtggtttctacccacaaggttg
aaggttcgaatcctttctggggtgtc
1-2tRNA-Arg(acg)c[148694,148768]
tatctctatagtgtaatggattaacacgaaagcctacgaagcttttaatt
ctggttcgaatccagatagggatgt
1-3tRNA-Gly(tcc)c[148773,148845]
gtggtattggtctaggggttatgatttgtggcttccaaccatgagacatc
ggttcgaatccgatataccacac
1-4tRNA-Ser(gct)c[148854,148938]

ggagagttgtcagagtggcttattgtgcttggttgctaaccaagtggagg
taaaacttccaagggttcgaatcccttactctcct
1–5tRNA–Ser(tga)c[148947,149032]
ggaaggtcttgcatatcggtagtgctagctagtcttgaaaactagtggtc
agcaatggtctaggggttcgagtccgctaccttcct
1–6tRNA–Thr(tgt)c[149128,149199]
gcaggtttagttaaggggctataacaatcgccttgtaagcgataatcggg
ggttcgattccctcaacctgca
1–7tRNA–Ala(tgc)c[149201,149274]
gggggattagtttagtgggaaaacaatggctttgcaagccatagtcaagg
gttcgactcccttatcctccacca
1–8tRNA–Pro(tgg)c[149279,149352]
gtgggtatggggcagttggtagcctactggctttggaagctagatgtcgc
aggttcgagtcctgctacccgcac
1–9tRNA–Leu(tag)c[149363,149448]
tgcaggagtgatggaattggtagacttggcagatttagaatctgctgtcc
tagtggcgtgagggttcgagtccctccttctgtatc
1–10tRNA–Tyr(gta)c[149452,149537]
ggggtagcggtaacgttggagagttacggtggactgtaaatccactgcca
tcggcttagtaggttcgaatcctatctgccccacca
1–11tRNA–Ile(gat)c[149545,149618]
tggtggattcgtatagaggtaatacaatcggctgataaccgataaacgga
agttcgattcttccatccaccatc
1–12tRNA–Glu(ttc)c[149772,149845]
tggcgcattggtcaatgggttaagacggtagcctttcacgctacagtaac
gggttcgagtcccgtatgcgctat
1–13tRNA–Cys(gca)c[149854,149930]
agggctatggtgaagtggacaaacacatctggttgcaacccagacaatcg
ccagttcgaatctggttagtccttcca
1–14tRNA–Phe(gaa)c[149937,150010]
tggctctctagctgagatggattagcaaaggactgaaaatccttggaggt
tggttcgataccaacgggagccat
1–15tRNA–Met(cat)c[150014,150086]
tgcggtgaggacaaacggctaagttgcttggctcataaccaagagatagt
gggttcgattcccaccaccgtaa
1–16tRNA–His(gtg)c[150138,150210]
cagtgtgtggcgtaatggcttaacgcagtggactgtgaatccacgaatga
gggttcgattccctccatgctga
1–17tRNA–Gln(ttg)c[150269,150345]
tttccgagtagacaaactggtaaagtcactagcctttgaagctagggttt
ggaggttcgaagccttcctcggaagtc
1–18tRNA–Gly(gcc)c[150355,150427]
tgtggaattagtttagaggtaaaatttctgattgccattcagaggtcaag
ggttcgattcccttattccacat

1-19tRNA-Trp(cca)c[150428,150501]
ataccccttagtgtaatggtagcacaggagtctccaaaacttctagtctgg
gttcaaatcctagagggtatgcca
1-20tRNA-Asp(gtc)c[150503,150576]
ggctcgttagtttaaaggaaaaatactgcactgtctatgcagagttaggg
gttcgattcccctacgggtcgcca
1-21tRNA-Asn(gtt)c[150579,150653]
tggggtgtagcttaaaggtaaagcagtcggctgttaaccgaaagagtgta
ggttcaattcctatcaccccagcca
>KJ410132.1 Mycobacterium phage 20ES, complete genome
1-1tRNA-Gln(ctg)[4425,4503]
ttcccgactagcacaactggtagctgcgctcggctctggaccgagaggtt
tgaggttcgaatccttggtcgggagccca
>KF926093.1 Lactococcus phage phiL47, complete genome
1-1tRNA-Met(cat)[48524,48596]
ggttctttagcttaactggtaaagccctccgctcataacggagtaagtgc
aggttcaagtcctgcaagaacca
1-2tRNA-Arg(tct)[50343,50415]
gttcggttagtgtaattggataacacacagaatttctaattctgtactct
ggattcgagttccagactgagca
1-4tRNA-Asp(gtc)[51213,51287]
tgactcattggtgtagcggttatcacacttgcctgtcacgcaagagagca
tgggttcgaatcccatatgggtcgt
1-5tRNA-Ala(cgc)[51639,51709]
cacggatgtggtagtaagtaactacagagggtcgccacctttcatgcagg
ttcaagtcctgctgtctgtgt
1-6tRNA-Asn(att)[52830,52905]
ttgattggtaactcagttggttagagtgcttgactattaatcaagaagtc
gtgagttcgaatctcacccaatcagt
1-7tRNA-Trp(cca)[53114,53184]
atctatttagtgtagtggtaacacatcagattccaaacctgataacgtgg
gttcgattcctacaatggatg
1-8tRNA-Met(cat)[53400,53473]
tacggggtgggtggcaaggtcgtcacgcttggctcataaccaagaaagag
cggttcgattccgttaccccgaat
>JF270478.1 Psychrobacter phage Psymv2, complete genome
1-1tRNA-Leu(caa)[11082,11160]
tcgtaggtagctcagttggttagagccgtggtgatatttgccactagggt
cgcaggttcaagtcctgcccttcgagcca
>KF614509.1 Rhizobium phage vB_RleS_L338C, complete genome
1-1tRNA-Pro(ggg)c[104333,104415]
ctcgacgtagaggacggggcgctgcgcgatcacctggggcgtgatccgga
ggagcctgtgctgttcgatgccgcatgcccgga
>HG934469.1 Salmonella phage vB_SenS-Ent2 complete genome

1–1tRNA–Ser(tga)c[5636,5714]
aactggaataattgagggcaaaattaaggccccttgaaggggcctttcgt
tactcaggttctgagaccttagccgcttt
>KF977490.1 Rhizobium phage vB_RglS_P106B, complete genome
1–1tRNA–Leu(taa)c[36501,36576]
gcgcgtgtatcccaataggtagaggaaacagacttaaaatctgtaaagtg
tcggttcgaaccgtccacgcgcacca
>KF986246.1 Mycobacterium phage MichelleMyBell, complete genome
1–1tRNA–Gly(gcc)[11615,11688]
caggatagtggccgaggcagcggcccgcggctgccagacgcgccagtggt
gccgtacgactcgactattccgcc
1–2tRNA–Thr(ggt)[27759,27836]
gatcctacggacgggaaagggacgtactacggtctggtaatccgcaggtc
gctggttcgagcccagctgggggcacca
>JN680858.1 Mycobacterium phage Rumpelstiltskin, complete genome
1–1tRNA–Lys(ctt)[62637,62711]
gccaccttagctcagttggtagagctgccgactcttaatcggtaggtcac
aggttcaagtcctgtacggggtacc
>JN408461.1 Mycobacterium phage Trixie, complete genome
1–1tRNA–Trp(cca)[6009,6082]
gggcatgtagctcaatcggtagagcagcggtctccaaagccgccggttcc
aggttcgagtcctggcaggcccgc
1–2tRNA–Glu(ctc)[6117,6190]
gctcccttggagtagcggttaactcatctgactctcaatcagaagatcgc
gggttcgaaccccgcagggagtac
>JN698994.1 Mycobacterium phage DS6A, complete genome
1–1tRNA–Lys(ttt)[13981,14071]
cggacgcatgggaaggcgtcaagaccgcattttctgcggtgtgggaatgg
ttgcgcggcaagtttgaggcgttcaaatcggcgctgtccgc
>JN699005.1 Mycobacterium virus Alma, complete genome
1–1tRNA–Glu(ctc)[5913,5985]
ggtccgttggagtagcggcaactcatctgactctcactcagaagatcgcg
ggttcaaatcccgcacggactac
>KJ173786.1 Microbacterium phage vB_MoxS–ISF9, complete genome
1–1tRNA–Val(gac)[56201,56284]
gcgtcggtagctcaattggtcagagcggatctcgcgacagcagataaccg
aaagcgatgcgggttcgagtcccgtccgacgcac
>KJ194585.1 Mycobacterium phage Seabiscuit, complete genome
1–1tRNA–Trp(cca)[1893,1968]
tggctcttagctcaatttggtagagcagcggtctccaaaaccgccggttg
caggttcgagtcctgcagagtcagcc
>KJ668713.1 Escherichia phage e4/1c, complete genome
1–1tRNA–Arg(cct)[46736,46811]
gttctgctggcgtaacaggataacgcagagacctcctaagtctcagttac

tggttcgagtccagtgcggaacgcca

>JN643714.1 Mycobacterium phage BarrelRoll, complete genome

1−1tRNA−Trp(cca)[1003,1076]

gggtgtgtagctcaatcggtagagcagcggtctccaaagccgccggttgc

acgttcgagtcgtgccgcgcccgc

>JN243855.1 Mycobacterium phage Larva, complete genome

1−1tRNA−Trp(cca)[588,663]

tcgcagatagctcaattggtagagcagcggtctccaaaaccgccggttgc

aggttcgagtcctgctctgcgggcca

>GU580941.1 Rhodococcus phage ReqiPepy6, complete genome

1−1tRNA−Ala(agc)[41206,41280]

agactcgtagctcagttggaatgagcgtcgtcagcaatgacgaaggtcgc

aggttcgagtcctgccgagctttcc

>JN698997.1 Mycobacterium phage Courthouse, complete genome

1−1tRNA−Gly(tcc)[91615,91687]

gcgcttgtggtcgaattggaaagactcctggcttccacccaggttatgca

ggttcgagtcctgtcgagcgctc

1−2tRNA−Tyr(gta)[92788,92860]

tggcccgtagctcaattggcagagcagccggcggtaattcggcgcgtctc

ggttcgagtccgggcgggctacc

>JN572689.1 Mycobacterium phage Perseus, complete genome

1−1tRNA−Leu(taa)c[23731,23805]

gcccctatagcccaattggaagaggcagcgggcttaaaacccgtgtggtg

acggctcgaatccggctaggggcac

>JN408460.1 Mycobacterium phage Turbido, complete genome

1−1tRNA−Gln(ctg)[4859,4933]

tccccgttcgtctaattggcaagacgccgggttctggccccggtaattga

ggttcgagtccttgatggggaacca

>JN382248.1 Mycobacterium phage Lilac, complete genome

1−1tRNA−Arg(cct)[63338,63421]

atggcccgtagctcagtcaggtagagcagccttgccccttgcgggcttgg

ttcgtccccggttcaaatccgggcgggccttgcc

1−2tRNA−Gly(tcc)[63424,63496]

gcgcccgtggtcgaattggaaagactcctggcttccacccaggttatgca

ggttcgagtcctgtcgggcgctc

>JN153085.1 Mycobacterium phage Doom, complete genome

1−1tRNA−Trp(cca)[2409,2484]

tggctcttagctcaatttggtagagcagcggtctccaaaaccgccggttg

caggttcgagtcctgcagagtcagcc

>JN201525.1 Mycobacterium phage Thibault, complete genome

1−1tRNA−Gly(tcc)[90680,90752]

gcgcttgcggtcgagttggaaagactcctggcttccacccaggttatgca

ggttcgagtcctgtcgagcgctc

1−2tRNA−Tyr(gta)[91857,91929]

tggcccgtagctcaattggcagagcggccggcggtaattcggcgcgtctc
ggttcgagtccgggcgggctacc

>JN698999.1 Mycobacterium phage Blue7, complete genome

1-1tRNA-Asn(gtt)[5396,5468]
tggtgtgtagctcaatggcagagcacccggctgttaaccggacggttgaa
ggttcgaatccttccataccagc

1-2tRNA-Trp(cca)[5472,5545]
gcgctcctagctcaattggtagagcagcggtctccaaagccgcgagttcc
aggttcgaatcctggggagcgtgc

1-3tRNA-Gln(ctg)[5587,5659]
tccctgttcgtctaatcggtaagacaccgggctctggatccggtaatcga
ggttcgagtccttgatggggagc

>HG934470.1 Salmonella phage vB_SenS-Ent3 complete genome

1-1tRNA-Ser(tga)c[5640,5718]
aactggaataattgagggcaaaattaaggccccttgaaggggcctttcgt
tactcaggttctgagaccttagccgcttt

>JN412589.1 Mycobacterium phage Patience, complete genome

1-1tRNA-Gln(ttg)[57360,57432]
tgcctcatggtgtaattggcagcacgagtggttttggtccacttagttca
ggttcgagtcctggtgaggcagc

>JN256079.1 Mycobacterium phage Charlie, complete genome

1-1tRNA-Thr(ggt)[27442,27519]
gatcctacggacgggaaagggacgtactacggtctggtaatccgcaggtc
gctggttcgagcccagctgggggcacca

>GU580942.1 Rhodococcus phage ReqiPoco6, complete genome

1-1tRNA-Ala(agc)[42700,42775]
gagactcgtagctcagttggaatgagcgtcgtcagcaatgacgaaggtcg
caggttcgagtcctgccgagctcttc

>KJ190157.1 Escherichia phage vB_EcoS_FFH1, complete genome

1-1tRNA-Met(cat)c[27984,28059]
agttagttggcagagtggttatgcacctccttcatacggagcgactacag
tggttcaaatccactactaactacca

1-2tRNA-Ile(gat)c[28156,28232]
acttcggtagcttagcgatctaaagcactcggctgataaccgagagatcg
ggggtttaaatccctcccggagtacca

1-3tRNA-Thr(tgt)c[28613,28687]
gctcctaaagcattgctggcgatgcagttgccttgtaagcatctgaaccg
ggttcgattcctggtgggagcacca

1-4tRNA-Gln(ttg)c[29177,29254]
tggggattagcttagcctggtctaaagcttcggcctttgaagtcgagatc
attggttcaaatccaatatcccctgcca

1-5tRNA-Gln(ctg)c[29261,29336]
agaaggttagccaagtcggttaaggctggggtctctgaaaccccgatcag
tggttcgaacccactaccttctgcca

1–6tRNA–His(gtg)c[29546,29622]
gtggctatatcataattggttaatgatcctgattgtgaatcaggcctatg
tggattcgaattccactagccacccca
1–7tRNA–Leu(tag)c[30299,30375]
gcgtgattgatggaattggcatacataccgtccttagaagtcgggttttg
agggttcgaatcccttgtcacgcacca
1–8tRNA–Ala(tgc)c[30381,30459]
gggggatgggtctgctagggggtggacacctcgcttgcaccgaggacatca
gaacgattcgaattcgttatcctccacca
1–9tRNA–Val(tac)c[30972,31045]
gctcggttagtttaatgggagaaccccgtctttacacggcggttgtgata
gttcgattctatcaccgagtacca
1–10tRNA–Lys(ttt)c[31514,31592]
aggtcgctagctcaataggtttagtagcatccgacttttaatcggaaggt
tctgggttcgagtcccaggcgatctacca
1–11tRNA–Met(cat)c[31782,31859]
tgcgggttagatctctggtagagatcgctagtctcataagctagaaagag
gtaggttcgattcctgcacccgcttcca
1–12tRNA–Pro(tgg)c[31866,31941]
cagtgcgtagcgcagttggtagcgtgggagccttggatgcttcgggtcgc
aggttcgagtcctgccgcactgacca
1–13tRNA–Asp(gtc)c[32975,33051]
gcgaccggggctggcttggtaatggtactcccctgtcacgggagagaatg
tgggttcaaatcccatcggtcgcgcca
1–14tRNA–Asn(gtt)c[33349,33431]
gggtcgttagccaagcggtttggcggtggactgttaatccatgtcgaaag
acaacgtaggttcgaatcctacacggcccgcca
1–15tRNA–Cys(gca)c[33584,33659]
cgaccgttggctgaagggcttaggcgaaggattgcaaatcctttttatgt
gagttcaaatctcatgcggtcgtcca
1–16tRNA–Phe(gaa)c[34019,34093]
gcacctttagctgagatggattagcgcttgcctgaagagcttgagaggtt
cgttcgatacgaacagggtgcacca
1–17tRNA–Trp(cca)c[34100,34176]
attcctagagtgttactggacagcatgtcggtctccaaaaccgtacggtc
taggttcgagtcctagtaggtttgcca
1–18tRNA–Glu(ttc)c[34185,34261]
gtcctgttagacaaactggtaaagtcactaccctttcaaggtaggatttg
cgggttcgatccccgcacaggacgcca
1–19tRNA–Tyr(gta)c[34269,34359]
gttggattagtatcgtagaggtagcgaagcagactgtaaatctgccgact
cggaagggtctcgggtggttcgactccatcatccaacacca
1–20tRNA–Leu(taa)c[35238,35314]
gggggtgtaatcgaattggcataggtactggacttaaaattcaggttttg

tgggttcgaatcccaccacccctacca
1-21tRNA-Ser(gct)c[35614,35702]
ggaagaatagcataacggtattgcagcagattgctaatctgtcggtttga
aatatagccttgtgggttcgattcccgcttcttccgcca
1-22tRNA-Arg(tct)c[39237,39311]
cggggtgtagtctaagggagaggcaggagtcttctaaattcctttatgca
ggttcgaatcctgtcacctcggcca
>KJ510415.1 Mycobacterium phage Phantastic, complete genome
1-1tRNA-Trp(cca)[3799,3872]
aggcgcgtagctcaattggaagagcagcggtctccaaagccgccggctgc
aggttcgagccctgccgcgtctgc
>KJ127303.1 Enterococcus phage VD13, complete genome
1-1tRNA-Trp(cca)[29431,29504]
tgagttgttagtgtaatggttagcacaacagtctccaaaactgttagaga
gggttcgattccttcactgcttgt
>KJ507100.1 Pseudomonas phage phiPSA1, complete genome
1-1tRNA-His(gtg)[24232,24326]
tcgcgcatatggcgcaggtgggattggcggttctggtgaacaatcagcgt
ggtggtcatggtcatgctccggggttgtgagccccgatgcgcggg
>KJ804259.1 Staphylococcus phage 6ec, complete genome
1-1tRNA-Pro(tgg)[9981,10054]
cagaatgtagttcaatttggtagaataccacatttgggatgtggtggttg
ttggttcaaatccagccattctga
>KF771238.1 Escherichia phage bV_EcoS_AHS24, complete genome
1-1tRNA-Arg(cct)[45859,45934]
gttctgctggcgtaacaggataacgcagagacctcctaagtctcagttgc
tggttcgagtccagcgcagaacgcca
>KF771237.1 Escherichia phage bV_EcoS_AHP42, complete genome
1-1tRNA-Arg(cct)[46266,46341]
gttctgctggcgtaacaggataacgcagagacctcctaagtctcagttgc
tggttcgagtccagcgcagaacgcca
>KJ567042.1 Mycobacterium phage OkiRoe, complete genome
1-1tRNA-Trp(cca)[822,897]
tcgcaggtagctcaattggtagagcagcggtctccaaaaccgccggttgc
aggttcgagtcctgctctgcgggcca
>KF771239.1 Escherichia phage bV_EcoS_AKS96, complete genome
1-1tRNA-Arg(cct)[45370,45445]
gttctgctggcgtaacaggataacgcagagacctcctaagtctcagttac
tggttcgagtccagtgcggaacacca
>GU339467.1 Mycobacterium phage RedRock, complete genome
1-1tRNA-Trp(cca)[6249,6322]
gggcacgtagctcaatcggtagagcagcggtctccaaagccgcaggttcc
aggttcgaaccctggcgggctcgc
>KJ608188.1 Enterococcus phage EFC-1, complete genome

1−1tRNA−Met(cat)[37009,37083]
ggaccattagctcagttggttagagcaaacggctcataaccgttcggtca
caggttcgagtcctgtatggtccac
>KP027206.1 Mycobacterium phage Milly, complete genome
1−1tRNA−Arg(gcg)[665,757]
caccgcttagcgaggttgcgcgccacgtcattgcgcccgacggcatcgtg
tcgacggcctggccgtcggtgtgtgcgacgtgcgcggcgatgg
>KM101123.1 Mycobacterium phage Minerva, complete genome
1−1tRNA−Gly(tcc)[93356,93428]
gcgcttgtggtcgagttggaaagactcctggcttccacccaggttatgca
ggttcgagtcctgtcgagcgctc
>KJ567043.1 Mycobacterium phage Gaia, complete genome
1−1tRNA−Leu(caa)[39120,39203]
gcccgaatggcggaacggaagacgcgccggactcaaaatccggtgtccgc
aaggacgtgtgagttcgaatctcacttcgggcac
1−2tRNA−Lys(ttt)[78498,78572]
tggtccgtagctcaattggtcagagcagctgacttttaatcagcgggttc
cgggttcgacccccggcgggcctac
1−3tRNA−Asn(gtt)[78708,78781]
tggcccgtagctcaattggcagagcgcccgactgttaatcgggtggttcc
aggttcgagtcctggcgcgccagc
>KM514685.1 Lactobacillus phage Ldl1, complete genome
1−1tRNA−Ile(tat)c[66893,66964]
cgagcaatagctcaaaggtagagcagacaccttataagtgtaagataaga
gttcgatttctcttttgctcga
>KM101120.1 Mycobacterium phage Trike, complete genome
1−1tRNA−Trp(cca)[1673,1746]
aggcatgtagctcaattggtagagcagcggtctccaaagccgccggttgc
aggttcgagtcctgccgtgtctgc
1−2tRNA−Gln(ctg)[1753,1825]
tcctcgttcgtctaatcggtaggacaccaggctctggacctgggggttga
ggttcgagtccttggcgaggagc
1−3tRNA−Asn(gtt)[1860,1933]
tgacgtgtagctcaaccggcagagcaggcggctgttaaccgcccggttgg
aggttcgagtcctccctcgtcagc
>KM083128.1 Mycobacterium phage Sparky, complete genome
1−1tRNA−Ser(gct)[28252,28338]
ggaggtctaattcggctggggccggaaccccgttgctagcgggtgtgatc
cgattctgggttgcagttcgattctgcagacctccgc
1−2tRNA−Pro(tgg)[28557,28632]
cggggtgtagctcagcttggccagagtgccggttttgggtaccggacgtc
gcgggttcaaatcccgccaccccgac
1−3tRNA−Trp(cca)[28636,28707]
gcgcctctggtgtagtggcagcacaacggcttccaaacccgttggcggga

gttcaattctctcgaggcgcgc
1–4tRNA–Tyr(gta)[28726,28810]
gcgggtctgcaactggtgtcgcagggggcctgtaaagccctcgccgcagg
cgcggcatggagagttcgattctctcggcccgcac
>KM677185.1 Lactococcus phage WRP3, complete genome
1–1tRNA–Met(cat)[48734,48806]
ggttctttagcttaactggtaaagcccttcgctcataacggagtaagtgc
aggttcgagtcctgcaagaacca
1–2tRNA–Arg(tct)[50816,50890]
tgctcggttggtgaagctggatatcacacagaatttctaattctgtatca
agggttcaaatcccttactgagtat
1–4tRNA–Asp(gtc)[51612,51682]
ggcttattagtatatgggtactacacatccctgtcacggaggagaaccga
gttcgagtctcggataagccg
1–5tRNA–Pro(tgg)[52031,52102]
caggatatagtgttaatggtagcatgcgtgttttgggaacatgtagtgtt
ggttcgagtccagctatcctga
>JN831653.1 Mycobacterium virus Fionnbarth, complete genome
1–1tRNA–Arg(gcg)[14197,14290]
tcggcggcgctgcgcaggagatgggcagcagcgttcgcggcaccctgtcg
aacctcaaggcggcctattcgcggttcggtgccgagctgtcggg
1–2tRNA–Lys(ttt)c[30843,30918]
gccccgctagctcaatcggaagagcagccggcttttaaccggcgggtacg
gagatcgaaactccggcggggcacca
>KM979354.1 Enterobacteria phage DT57C, complete genome
1–1tRNA–Met(cat)c[24959,25034]
agttagttggcatagtggttatgcacctccttcatacggagcgactacag
tggttcaaatccaatactaactacca
1–2tRNA–Gly(tcc)c[25586,25660]
gcgtgattagttcagcggctagaataactggcttccaaccagtagacacg
agttcgactctcgtatcccgcacca
1–3tRNA–Gln(ttg)c[25672,25749]
tggggattagcttagcttggcctaaagctacggcatttgaagtcgagatc
attggttcaaatccaatatcccctgcca
1–4tRNA–Gln(ctg)c[25756,25831]
agaacctgagccaagtcggttaaggccggggtctctgaaatcctgatcag
tggttcaaatccactaccttctgcca
1–5tRNA–Arg(acg)c[26249,26323]
gcgtccttatttcaatggaaagaatgtaaagccacgaactttacgattgg
ggttcgattccctgaggatgcacca
1–6tRNA–His(gtg)c[26330,26406]
gtggctatatcataattggttaatgatcctgattgtgaatcaggcctatg
tggattcgaattccactagccaccccca
1–7tRNA–Leu(tag)c[26941,27017]

gcgtgattgatggaattggcatacataccgtccctagaagtcgggttttg
agggttcgaatcccttgtcacgcacca
1-8tRNA-Ala(tgc)c[27023,27096]
ggggatatagtttaatggtagaacatctgctttgcacgcagaagacgagg
gttcaactccctctttctccacca
1-9tRNA-Pro(cgg)c[27465,27540]
cagtgtgtagcgcagttggtagcgtgggaccctcggatgatgcgggtcgc
aggttcgagtcctgccgcactgacca
1-10tRNA-Met(cat)c[29014,29088]
gggtcggtagcttaaagttaaagcggtggcctcataagctaacgagtagg
agttagattctcctccgacccacca
1-11tRNA-Ser(gct)c[29095,29183]
ggaagaatagcataacggtattgcagcagattgctaatctgtcggtttga
aatatagccttgtgggttcgattcccgcttcttccgcca
1-12tRNA-Arg(tct)c[30917,30991]
cggggtgtagtctaagggagaggcaggagtcttctaaattcctttatgca
ggttcgaatcctgtcacctcggcca
1-13tRNA-Ser(act)c[31492,31565]
tgccgatctagtataaacggtattatcaagccgtaactggtggaagatgt
aggttcgaatcctacgatcggtaa

>CP000315.1 Clostridium phage phiSM101, complete genome

1-1tRNA-Asn(gtt)c[19562,19635]
gggaacattgttcaatggtagaacggttggctgttaaccaactaatggga
gttcaattctctctgttcccgcca

>KT945994.1 Enterococcus phage vB_EfaS_IME197, complete genome

1-1tRNA-Glu(ttc)[13671,13744]
tggcgtgtagctcaattggtgagagcggttggttttcaatcaagtacatg
caggttcgactcctgtcacgtcaa
1-2tRNA-Met(cat)[37521,37594]
ggaccattagctcagctggttagagcaaacggctcataaccgttcggtca
caggttcgagtcctgtatggtcca

>KT968831.1 Pseudomonas phage YMC11/02/R656, complete genome

1-1tRNA-Gly(tcc)c[49179,49254]
gcggctctagctcaactggcagagcgctgtccttccaagtcggatgttgc
gggttcaagtcccgcgagccgctcca

>KT372002.1 Rhodococcus phage CosmicSans, complete genome

1-1tRNA-Asn(gtt)[1503,1576]
tcacgtatagctcaatcggcagagcaacgcactgttaaggcgggggttga
aggttcgagtccttctacgtgagc
1-3tRNA-Trp(cca)[1702,1776]
gggcacgtagctcaattggatagagccccggtctccaaagccggtggttg
ggggttcgagtccctccgggcctgc

>KT588442.1 Mycobacterium phage LadyBird, complete genome

1-1tRNA-Gln(ctg)[4416,4493]

ttcccggctagcacaacggtagctgcgctcggctctggaccgagaggttt
gaggttcgaatccttggccgggagccca
>KR080199.1 Mycobacterium phage Baee, complete genome
1-1tRNA-Arg(ccg)[35179,35273]
cccggcaagttctggcactggtggggcaccaacacctccgacgagtggct
gggaccggtcgagcccggccagcggttcaacctcgcctaccgggc
>KP137435.1 Mannheimia phage vB_MhS_587AP2, complete genome
1-1tRNA-Arg(tct)c[37159,37234]
gaccgcatagttcagtggatagagcagctgccttctaagcagtgggtcga
gagttcgaatctctctgcggtcgcca
1-2tRNA-Trp(cca)[45736,45811]
aagggtgtagctcaataggtagagcaacggtctccaaaatcgttggttgt
tggtttgagtccaaccacccctttcca
>KP143763.1 Salmonella phage Shivani, complete genome
1-1tRNA-Met(cat)c[30476,30551]
agttagttggcagagtggttatgcacctccttcatacggagcgactacag
tggttcaaatccactactaactacca
1-2tRNA-Ile(gat)c[30648,30724]
gcttcggtagcttagcgatctaaagcactcggctgataaccgagagatcg
ggggtttaaatccctcccggagtacca
1-3tRNA-Asp(gtc)c[31810,31886]
gcgaccggggctggcttggtaatggtactcccctgtcacgggagggaatg
tgggttcaaatcccatcggtcgcgcca
1-4tRNA-Asn(gtt)c[32184,32266]
gggtcgttagccaagcggtttggcggcggattgttaatccgtgtcgaaag
acaacgtaggttcgaatcctacacggcccgcca
1-5tRNA-Cys(gca)c[32419,32494]
cgaccgttggctgaatggcttaggcgaaggattgcaaatcctttttatgt
gagttcaaatctcatgcggtcgtcca
1-6tRNA-Tyr(gta)c[33012,33095]
gcatcgttggcagaatgtctattgcagcggtctgtaaatccgtgcccttc
ggggttggtggttcaaatccatcacgatgcacca
1-7tRNA-Met(cat)c[33972,34046]
gggtcggtagcttaaagttaaagcggtggcctcataagctaacgagtagg
agttagattctcctccgacccacca
1-8tRNA-Ser(gct)c[34053,34141]
ggaagaatagcataacggtattgcagcagattgctaatctgtcggtttga
aatatagccttgtgggttcgattccccctcttccgcca
1-9tRNA-Arg(tct)c[36153,36227]
cggggtgtagtctaagggagaggcaggagtcttctaaattcctttatgca
ggttcgaatcctgtcacctcggcca
1-10tRNA-Ser(act)c[36728,36801]
tgccgatctagtataaacggtattatcaagccgtaactggtggaagatgt
aggttcgaatcctacggtcggtaa

>KM363596.1 Mycobacteriophage Omnicron, complete genome
1-1tRNA-Trp(cca)[789,865]
atgcgggtagctcaattcggtagagcagcggtctccaaaaccgccggttg
caggttcgagtcctgccccgtgtgcca
>KT222941.1 Mycobacterium phage NelitzaMV, complete genome
1-1tRNA-Arg(cct)[61221,61304]
atggcccgtagctcagtcaggtagagcagccttgccccttgcgggcttgg
ttcgtccccggttcaaatccgggcgggccttgcc
1-2tRNA-Gly(tcc)[61307,61379]
gcgcccgtggtcgaattggaaagactcctggcttccacccaggttatgca
ggttcgagtcctgtcgggcgctc
>KT184694.1 Mycobacterium phage Smeadley, complete genome
1-1tRNA-Trp(cca)[4055,4128]
gggtcagtagctcaattggtagagcagcggtttccaaagccgctcgttgg
gggttcgagtccctcctggcctgc
>KR011062.1 Tsukamurella phage TIN2, complete genome
1-1tRNA-Asn(gtt)[12390,12463]
tgggatatagctcaattggcagagcaggcgactgttaatcgcccggttac
aggttcgagtcctgttttcccagc
>KT989433.1 Streptomyces phage phiSAJS1, complete genome
1-1tRNA-Ala(ggc)c[54626,54723]
ccgtccagcaggtcgcggacctgaagggcgtggccgctggcaccgtccgc
agctggaagcaccgtgggaagctgcccgtgttcgacacggtggacggg
>KJ959632.1 Mycobacterium phage Equemioh13, complete genome
1-1tRNA-Gln(ctg)[4930,5004]
tccccgttcgtctaagcggcaagacaccgggttctggccccggcaatcga
ggttcgagtccttgatggggagcca
>KM101119.1 Mycobacterium phage Tiffany, complete genome
1-1tRNA-Asn(gtt)[3734,3809]
tgatctgtagctcaatcggcagagcacccggctgttaaccgggacgttgg
aggttcgagtcctcccagatcagcca
1-2tRNA-Trp(cca)[3850,3924]
aggcacgtagctcaattggtcagagcagcggtctccaaagccgccggctg
caggttcgagtcctgccgtgtctgc
1-3tRNA-Leu(cag)[3959,4033]
ggctcggtaggcaaacaggcaaagccgcctgtctcaggaacaggtgcgtg
agggttcgactccctcccgagctac
>KR080193.1 Mycobacterium phage Luchador, complete genome
1-1tRNA-Asn(gtt)[4324,4399]
tgacgtgtagctcaatcggcagagcacccgcctgttaagcgggacgttcc
aggttcgagtcctggcatgtcagcca
>FJ848883.1 Lactococcus phage CB14, complete genome
1-1tRNA-Trp(cca)[28914,28986]
tgcgagcatagtatagtggtaatgctacagattccaaacctgtaaacgtg

ggttcgattcctgctgctcgtgt
>FJ848881.1 Lactococcus phage SL4, complete genome
1-1tRNA-Trp(cca)[27455,27527]
tgcgagcatagtatagtggtaatgccacagattccaaacctgtaaacgtg
ggttcgattcctactgttcgtgt
>KM400683.1 Mycobacterium phage Ariel, complete genome
1-1tRNA-Gly(tcc)[91171,91243]
gcgcttgtggtcgaattggaaagactcctggcttccacccaggttatgca
ggttcgagtcctgtcgagcgctc
1-2tRNA-Tyr(gta)[92339,92411]
tggcccgtagctcaattggcagagcagccggcggtaattcggcgcgtctc
ggttcgagtccgggcgggctacc
>KT186228.1 Streptomyces phage Amela, complete genome
1-1tRNA-Arg(cct)c[45162,45244]
cacctgctgggacgggttcaagaccacctcctgagtggtcagcgtgagcg
gcacacaccccggttcgagtccggggcacgcac
1-2tRNA-Trp(cca)c[48673,48763]
aagtgcctacccgagtgggtaggcaagaccacccccagggtggtcggagg
gtggctggcaggcacacctggttcaagcccagggcacccac
>KR080197.1 Mycobacterium phage FlagStaff, complete genome
1-1tRNA-His(gtg)c[42689,42779]
tccggcgtggacggttccggggcggtcggctgtgggtcggccgtgggaac
gtcgctgtcgtcctcgacgggcgcgggcccggtcgccggcg
>FJ848882.1 Lactococcus phage CB13, complete genome
1-1tRNA-Trp(cca)[31651,31721]
gcgagcatagtatagtggtaatgctacagattccaaacctgtaaacgtgg
gttcgattcctgctgttcgtg
>KM402757.1 Mycobacterium phage Llama, complete genome
1-1tRNA-Ser(gga)[45752,45825]
tcatcaactggcatagcttcgctgtcgccgctggaggcggtggcggtggc
ggtggtgtttccgtcggtggtgga
>KT004677.1 Mycobacterium phage UnionJack, complete genome
1-1tRNA-Trp(cca)[4098,4171]
gggtcagtagctcaattggtagagcagcggtctccaaagccgcgagttgg
aggttcgagtcctccctggcccgc
>KR011063.1 Tsukamurella phage TIN3, complete genome
1-1tRNA-Asn(gtt)[12481,12554]
tggcatatagctcaatcggcagagcttccgactgttaatcggatggttac
aggttcaagtcctgttatgccagc
>KT932699.1 Enterococcus phage vB_EfaS_IME198, complete genome
1-1tRNA-Trp(cca)[35720,35792]
tgcgttgtaagtgtaatggctgcacaatggtctccaaaaccattagagag
ggttcgactccttcacaacgtgt
>KT438500.1 Mycobacterium phage Pari, complete genome

1−1tRNA−Trp(cca)[2393,2468]
tggctcttagctcaatttggtagagcagcggtctccaaaaccgccggttg
caggttcgagtcctgcagagtcagcc
>KT381276.1 Mycobacterium phage Serenity, complete genome
1−1tRNA−Trp(cca)[4230,4303]
gtacacgtagctcaatcggtagagcagcggtctccaaagccgccggttcc
aggttcgactcctggcgtgtatgc
1−2tRNA−Gly(tcc)[25866,25934]
tgggcgaatagtcgaatgcaacacccattcttccaaactggtgatgccgg
ttcgatcccggtcgcccgc
>KT281794.1 Mycobacterium phage Snenia, complete genome
1−1tRNA−Tyr(gta)[39565,39648]
gtggcgaaggcaactgttggtttgttgcacctgcctgtaaagcaggcgct
tcggcttcgggggttcaattccctctcgccacac
1−2tRNA−Thr(cgt)[62793,62865]
gccaccttagctcagcggtagagcagcgccttcgtaacgcgcaggtcaac
ggttcgattccgttaggtggctc
1−3tRNA−Pro(tgg)[63218,63290]
cggaaacaggtgtaatggttgcatgaggcgtttggatcgccggggttcgg
ggttcgactccctggtttccgac
1−4tRNA−Trp(cca)[63633,63706]
agctcggtagctcaactggtagagcagtggtctccaaagccaccggttgc
aggttcgactcctgtccgggttgc
1−5tRNA−Leu(tag)[63707,63781]
ggttccgtaggcaaactggcaaagccgtctgacttagaatcaggtgtttg
ggagttcgactctccccggaactac
1−6tRNA−His(gtg)[63782,63851]
gctaagtagcttaatggtaaagcccccggttgtggtccgggcgatcacgg
ttcaactccgtgcttaagcc
1−7tRNA−Gln(ctg)[64131,64205]
tgcccgctagcacaattggcagttgcgctgcgctctgaacgcagaggttc
caggttcgactcctgggtgggcagc
1−8tRNA−Lys(ctt)[64215,64288]
gcgtcgttagctcagttggtagagcagggcactcttaatgctcgggtcgg
gggttcaagtccctcacggcgcac
1−9tRNA−Pyl(cta)[65431,65523]
tgctcaaatgggtagggccgttcccctgtcatctaaaggcaggatgcctc
gctgttaacgaggtcacgcaggttcgacccctgctgggggagc
1−10tRNA−Lys(ttt)c[65528,65601]
gccgcgctagctcagttggtagagcagctgacttttaatcagcgggtccg
gggttcgatcccccggcgcggtac
>KT326768.1 Mycobacterium phage Tasp14, complete genome
1−1tRNA−Trp(cca)[2429,2504]
tggctcttagctcaatttggtagagcagcggtctccaaaaccgccggttg

caggttcgagtcctgcagagtcagcc
>KT221034.1 Streptomyces phage SF3, complete genome
1-1tRNA-Gly(ccc)c[19961,20059]
gtgtcgcccttgcgcacggtgtacgtgccgccggtgttcccgccgccggt
cccgccgggcgaccaggtcgccgggtgcttgagccgctcggcgacgcgc
>KP202969.1 Achromobacter phage JWX, complete genome
1-1tRNA-Pro(tgg)c[36081,36157]
cggagtgtagcgcagtcaggtagcgcatctgctttgggagcagagggtcc
taggttcgaatcctagtactccgacca
>KM101118.1 Mycobacterium phage Swirley, complete genome
1-1tRNA-Trp(cca)[4278,4351]
gggtcagtagctcaactggaagagcagcggtctccaaagccgcgagttgg
aggttcgagtcctccctggcctgc
>KP137433.1 Mannheimia phage vB_MhS_535AP2, complete genome
1-1tRNA-Arg(tct)c[36410,36485]
gaccgcatagttcagtggatagagcagctgccttctaagcagtgggtcga
gagttcgaatctctctgcggtcgcca
1-2tRNA-Trp(cca)[47763,47838]
aagggtgtagctcagttggtagagcagcggtctccaaaatcgttggttgt
tggtttgagtccaaccaccctttcca
>KM677210.1 Mycobacterium phage Larenn, complete genome
1-1tRNA-Gln(ctg)[4518,4592]
tccccgttcgtctaagcggcaagacaccgggttctggccccggcaatcga
ggttcgagtccttgatggggagcca
>KP719134.1 Enterobacteria phage JenK1, complete genome
1-1tRNA-Ala(tgc)[26546,26619]
gctttgattgcaacgtagttgttaacgtttgcagagttaaggcgcaggta
ctggttcaactccatttcaaagcc
>KT438501.1 Mycobacterium phage Theia, complete genome
1-1tRNA-Trp(cca)[4279,4352]
gggtcagtagctcaactggaagagcagcggtctccaaagccgcgagttgg
aggttcgagtcctccctggcccgc
>KT281791.1 Mycobacterium phage Lolly9, complete genome
1-1tRNA-Tyr(gta)[39383,39466]
gtggcgaaggcaactgttggtttgttgcacctgcctgtaaagcaggcgct
tcggcttcgggggttcaattccctctcgccacac
1-2tRNA-Thr(cgt)[62858,62930]
gccaccttagctcaggggtagagcagcgccttcgtaacgcgcaggtcaac
ggttcgattccgttaggtggctc
1-3tRNA-Pro(tgg)[63270,63344]
ctggttgtagcgcagcttggtagcgcgcctgatttggattcagggggtcg
gaggttcaaatccttccagccagac
1-4tRNA-Gly(tcc)[63683,63757]
gcgctagctacccctgttggcggggtgcctgccttccaagcaggatcacg

cgggttcgattcctgtctagtgctc
1–5tRNA–Trp(cca)[63762,63835]
agctcggtagctcaactggtagagcagtggtctccaaagccaccggttgc
aggttcgactcctgtccgggttgc
1–6tRNA–Leu(tag)[63836,63910]
ggttccgtaggcaaactggcaaagccgtctgacttagaatcaggtgtttg
ggagttcgactctccccggaactac
1–7tRNA–His(gtg)[63911,63980]
gctaagtagcttaatggtaaagcccccggttgtggtccgggcgatcacgg
ttcaactccgtgcttaagcc
1–8tRNA–Gln(ctg)[64260,64335]
tgcccgctagcacaatttggcagttgcgcccggctctgaaccgggaggtt
ccaggttcgactcctgggtgggcagc
1–9tRNA–Lys(ctt)[64344,64418]
gcctccgtagctcagatggttagagcagccgactcttaatcggcgggtcg
caggttcgactcctgccgggggtac
1–10tRNA–Asn(gtt)[65977,66048]
tcccctgtcatctaagggcaggatgccacgctgttaacgtggtcacgcag
gttcgacccctgctgggggagc
1–11tRNA–Lys(ttt)c[66053,66126]
gccgcgctagctcagttggtagagcagctgacttttaatcagcgggtccg
gggttcgatcccccggcgcggtac
>KT151955.1 Brevibacillus phage Jenst, complete genome
1–1tRNA–Asn(gtt)c[7178,7252]
agggtagtagttcaatggtagaacgacgcactgttaatgcgtatgttgca
ggttcgagtcctgccttccctgcca
1–2tRNA–Pro(tgg)c[7356,7432]
cagggtgtagttcagttcgggagaacaccgcatttgggatgcggaggtcg
caggttcaagccctgccactctgacca
1–3tRNA–Met(cat)c[7434,7507]
gggtctttagctcgaaggccgagcaggcggctcatgaccgctaggtgtgg
gttcgattcccacaagacccacca
1–4tRNA–Cys(gca)c[7546,7618]
gtgtctgtccctagtcggtctagggtacggtctgcaaaaccgtattacgt
gggttcaagtcccaccagatgct
1–5tRNA–Tyr(gta)c[8396,8478]
ggatgagtatgtaagaggttaagctgacagagtgtaaatctgtcgcctga
tggcttcgtaggttcaactcctacctcacccac
1–6tRNA–His(gtg)c[8486,8574]
gtgtacgtggtggaaatggtagacacgcaagattgtggttcttgtggggc
gcaagccccgtgcaggttcaagtcctgtcgtacacccca
>KP202970.1 Achromobacter phage 83–24, complete genome
1–1tRNA–Pro(tgg)c[36269,36345]
cggagtgtagcgcagtctggcagcgcatctgctttgggagcagagggtcc

taggttcgaatcctagtactccgacca
>KP273225.1 Mycobacterium phage Sheen, complete genome
1−1tRNA−Asn(gtt)[5552,5627]
tgacttgtagctcaatcggcagagcacccggctgttaaccgggcggttga
aggttcgagtccttccaggtcagcca
1−2tRNA−Trp(cca)[5629,5704]
aggcacctagctcaattggtagagcggcggtctccaaagccgctggttgg
gggttcgagtccctcggtgtctgcca
>JN006061.1 Mycobacterium phage Toto, complete genome
1−1tRNA−Arg(cct)[63089,63172]
atggcccgtagctcagtcaggtagagcagccttgccccttgcgggcttgg
ttcgtccccggttcaaatccgggcgggccttgcc
1−2tRNA−Gly(tcc)[63175,63247]
gcgcccgtggtcgaattggaaagactcctggcttccacccaggttatgca
ggttcgagtcctgtcgggcgctc
>KM677211.1 Mycobacterium phage Murucutumbu, complete genome
1−1tRNA−Trp(cca)[978,1051]
gggtgtgtagctcaatcggtagagcagcggtctccaaagccgccggttgc
acgttcgagtcgtgccgcgcccgc
>LN887948.1 Escherichia phage slur09 genome assembly slu09, chromosome : 1
1−1tRNA−Arg(tct)[93142,93216]
cggggtgtagtctaagggagaggcaggagtcttctaaattcctttatgca
ggttcgaatcctgtcacctcggcca
1−2tRNA−Ser(gct)[96753,96841]
ggaagaatagcataacggtattgcagcagattgctaatctgtcggtttga
aatatagccttgcgggttcgattcccgcttcttccgcca
1−3tRNA−Leu(taa)[97431,97507]
gggggtgtaatcgaattggcataggtactggacttaaaattcaggttttg
tgggttcgaatcccaccacccctacca
1−4tRNA−Tyr(gta)[98386,98473]
gggcgtttattccgtaagtggtagcggaggggattgtaaatccctggtca
ttgcgactcgagtggttcgactccattaacgcccacca
1−5tRNA−Glu(ttc)[98973,99049]
gtcctgttagacaaactggtaaagtcactaccctttcaaggtaggatttg
cgggttcgatccccgcacaggacgcca
1−6tRNA−Trp(cca)[99058,99134]
attcctagagtgttactggacagcatgtcggtctccaaaaccgtacggtc
taggttcgagtcctagtaggtttgcca
1−7tRNA−Phe(gaa)[99141,99215]
gcacctttagctgagatggattagcgcttgcctgaagagcttgagaggtt
cgttcgattcgaacagggtgcacca
1−8tRNA−Cys(gca)[99955,100030]
cgaccgttggctgaatggcttaggcgaaggattgcaaatcctttttatgt
gagttcaaatctcatgcggtcgtcca

1-9tRNA-Asn(gtt)[100183,100265]
gggtcgttagccaagcggtttggcggtggactgttaatccatgtcgaaag
acaacgtaggttcgaatcctacacggcccgcca
1-10tRNA-Asp(gtc)[100563,100639]
gcgaccggggctggcttggtaatggtactcccctgtcacgggagagaatg
tgggttcgaatcccatcggtcgcgcca
1-11tRNA-Pro(tgg)[101674,101749]
cagtgcgtagcgcagttggtagcgtgggagccttggatgcttcgggtcgc
aggttcgagtcctgccgcactgacca
1-12tRNA-Met(cat)[101756,101833]
tgcgggttagatctctggtagagatcgctagtctcataagctagaaagag
gtaggttcgattcctgcacccgcttcca
1-13tRNA-Lys(ttt)[102472,102549]
ggattgctaactcaactggataagagtaccggacctttaatccgtcagtt
ctgggttcgaatcccaggcaatccacca
1-14tRNA-Ala(tgc)[102835,102913]
ggggaatgggtctgcatggagtggacacctcgcttgcaccgaggacatca
ggacggttcgattccgttattctccacca
1-15tRNA-Leu(tag)[102919,102995]
gcgtgattgatggaattggcatacataccgtccttagaagtcgggttttg
agggttcgaatcccttgtcacgcacca
1-16tRNA-His(gtg)[103672,103748]
gtggctatatcataattggttaatgatcctgattgtgaatcaggcctatg
tggattcgaattccactagccacccca
1-17tRNA-Gln(ctg)[104210,104285]
tgggatgtagatcaattggcagatcgtcggcccctgactccgaaggttcc
tggttcgatcccaggcactccagcca
1-18tRNA-Gln(ttg)[104292,104367]
tggagagtagtgtaacggttagcacaacggcctttgactccgttaatggt
aggttcgattcctccttctccagcca
1-19tRNA-Thr(tgt)[104885,104971]
gctgggttggcagaatggttgaatgcacctgatttgtaatcaggaggagt
aatcccgttgcaggttcgaatcctgtgcccagcacca
1-20tRNA-Ile(gat)[105722,105798]
gcttcggtagcttagcgatctaaagcactcggccgataaccgagagatcg
gggtttaaatccctcccggagtacca
1-21tRNA-Met(cat)[105895,105970]
agttagttggcagagtggttatgcacctccttcatacggagcgactacag
tggttcaaatccactactaactacca
>KP719133.1 Enterobacteria phage JenP2, complete genome
1-1tRNA-Ala(tgc)[25835,25908]
gctttgattgcgacgtagttgttaacgtttgcagagttaagacgcaggta
ctggttcaattccatttcaaagcc
>KT591489.1 Mycobacterium phage Archie, complete genome

1−1tRNA−Thr(cgt)[62616,62687]
gctggattagcttagtggtcaagcgctgccttcgtaacgcagattcaccg
gttcgattccggtatccagctc
1−2tRNA−Pro(tgg)[62874,62948]
ctgcttgtagctcagcttggtagagcgcccgctttgggagcgggaggtcg
caggttcaaatcctgcttggcagac
1−3tRNA−Trp(cca)[63112,63187]
agctcggtagctcaattggtagagcagcggtctccaaagccgccggttcc
ccgttcaagtcggggccgggttgcca
1−4tRNA−Leu(tag)[63760,63834]
ggccctctagcccaattggtagaggcacaggttttaggtacctgtcagtg
cgagttcgagtctcgcgggggccac
1−5tRNA−His(gtg)[63835,63904]
gcttagtagcctagtggtaaggcagccggttgtgatccggtgaacctgag
ttcgattctcagctaaagcc
1−6tRNA−Gln(ctg)[63909,63983]
tgaccggtagcacaactggtagttgcgccgcgctctggacgcggaggttg
ttggttcgatcccagctcggtcagc
1−7tRNA−Gly(tcc)[64054,64128]
gtgttagtaacccatgttggctgggtgccactcttccaaagtggatttcg
cgggttcgattcctgtctagcacac
1−8tRNA−Lys(ctt)[64163,64238]
gcctcgttagctcagttggtagagctgccgactcttaatcggtaggtcac
aggttcaagtcctgtacggggtacca
1−9tRNA−Cys(gca)[65423,65497]
gccgtcatggctgagtggttaggcgtcggactgcaaatccggcttatccc
agttcgattctgggtggcggctcca
1−10tRNA−Asn(gtt)[65858,65931]
tcctccgtagctcaattggcagagcgcgcgactgttaatcgcgtggttgg
tggttcgagtccacccgttggagc
1−11tRNA−Lys(ttt)c[66126,66200]
gcccctatagctcagttggtagagcaggtgacttttaatcatcgggtcca
aggttcgatcccttgtgggggcacc
>KT285706.1 Mycobacterium phage Pioneer, complete genome
1−1tRNA−Glu(ctc)[5939,6011]
ggtccgttggagtagcggcaactcatctgactctcactcagaagatcgcg
ggttcgatccccgcacggactac
>KT221033.1 Streptomyces phage SF1, complete genome
1−1tRNA−Gly(ccc)c[37661,37759]
gtgtcgcccttgcgcacggtgtacgtgccgccggtgttcccgccgccggt
cccgccgggcgaccaggtcgccgggtgcttgagccgctcggcgacgcgc
>KM923971.1 Mycobacterium phage Kratio, complete genome
1−1tRNA−Trp(cca)[586,661]
tcgcagatagctcaattggtagagcagcggtctccaaaaccgccggttgc

aggttcgagtcctgctctgcgggcca
1–2tRNA–Ala(agc)c[58003,58090]
ggccggctgcggggcgttcctgttgcgcgtctagcggatgcgcgatgcgc
ggcggatgtagtgccgtgcggcgcggttggcggcctcc
>KP280063.1 Vibrio phage phi 3, complete genome
1–1tRNA–Arg(tct)c[32533,32608]
gcggtgatagtttaatcggatagaacactagccttctaagcttgtagcga
gggttcgaatccctctcactgcgcca
1–2tRNA–His(gtg)c[33160,33236]
gtggtcgaagcatttatcaggatgatgttcaggttgtgaccctgaggaaa
cgggttcaattcccgtcgatcacccca
1–3tRNA–Arg(acg)c[38491,38566]
gcgggtatgatgtaactgtatagcatgactccctacgaaggagttcgtct
aggttagaatcctagtagccgcgcca
1–4tRNA–Phe(gaa)c[38573,38648]
gcagtggtagctcagtaggtagagcacgggattgaagatcctggtgtcac
tggttcaactccagtcctctgcacca
1–5tRNA–Met(cat)c[38949,39025]
gcgcctttagcttagttggttagagcaaacccctcataagggtgaggtcg
ctggttcaagcccagcaaggtgcacca
1–6tRNA–Ile(gat)c[39032,39107]
tcccgtgtagttcagttggtagaacaggcgaccgataatcgccgcgtcac
tggttcgagtccagtcatgggaacca
1–7tRNA–Val(tac)c[39903,39980]
actcggttagcaaaatttggcgttgcacctcctttacacggaggctagaa
actggttcgattccagtaccgagtacca
1–8tRNA–Arg(cct)c[40663,40739]
gtcctagtagctcagcaggatagagcgctcccctcctaagggagaggtca
gaggttcaaatcctctctaggacgcca
>KJ944841.1 Mycobacterium phage Cheetobro, complete genome
1–1tRNA–Arg(gcg)[14207,14300]
tcggcggcgctgcgcaggagatgggcagcagcgttcgcggcaccctgtcg
aacctcaaggcggcctattcgcggttcggtgccgagctgtcggg
1–2tRNA–Lys(ttt)c[30852,30927]
gccccgctagctcaatcggaagagcagccggcttttaaccggcgggtacg
gagatcgaaactccggcggggcacca
>JQ740787.1 Lactococcus Phage ASCC191, complete genome
1–1tRNA–Trp(cca)[31597,31669]
tgcgagcatagtatagtggtaatgctacagattccaaacctgtaaacgtg
ggttcgattcctgctgttcgtgt
1–2tRNA–Pro(tgg)[31723,31794]
caggatatggtgtcaatggtagcatgcgtgttttgggaacatgtggtgtt
ggttcgagtccagctatcctga
>KR905069.1 Lactobacillus phage iLp84, complete genome

1−1tRNA−Ile(tat)[36908,36981]
gtagatgtagctcaatcggtagagcgcgcaacttataattgcgtgggtgc
aggttcaagccctgccgtctacac
>KT246486.1 Mycobacterium phage Chadwick, complete genome
1−1tRNA−Trp(cca)[4134,4207]
gggccagtagctcaactggtagagcagcggtctccaaagccgcgcgttgg
aggttcgagtcctccctggcctgc
>KM233454.1 Mycobacterium phage MarQuardt, complete genome
1−1tRNA−Asn(gtt)[3841,3916]
tgatctgtagctcaatcggcagagcacccggctgttaaccgggacgttgg
aggttcgagtcctcccagatcagcca
1−2tRNA−Trp(cca)[3957,4031]
aggcacgtagctcaattggtcagagcagcggtctccaaagccgccggctg
caggttcgagtcctgccgtgtctgc
1−3tRNA−Leu(cag)[4066,4140]
ggctcggtaggcaaacaggcaaagccgcctgtctcaggaacaggtgcgtg
agggttcgactccctcccgagctac
>KM197169.1 Mycobacterium phage Piro94, complete genome
1−1tRNA−Gln(ctg)[4937,5010]
tccccggtagtccaatcggtagcgacgctcggctctggaccgagagattg
aggttcgagtccttgctggggagc
>KF279418.1 Mycobacterium phage Anubis, complete genome
1−1tRNA−Asn(gtt)[3716,3791]
tgacgtgtagctcaattggcagagcacccggctgttaaccgggcggttga
aggttcgagtccttccatgtcagcca
1−2tRNA−Trp(cca)[3832,3906]
aggcacgtagctcaattggtcagagcagcggtctccaaagccgccggctg
caggttcgagtcctgccgtgtctgc
1−3tRNA−Leu(cag)[3941,4015]
ggctcggtaggcaaacaggcaaagccgcctgtctcaggaacaggtgcgtg
agggttcgactccctcccgagctac
>JQ067092.2 Pseudomonas phage PaMx42, complete genome
1−1tRNA−Gln(ttg)c[31254,31328]
tgcgccgtcgccaagcggtaaggcaccggattttgattccggcattcgta
ggttcgaatcctaccggcgcagcca
>KP137437.1 Mannheimia phage vB_MhS_1152AP2, complete genome
1−1tRNA−Arg(tct)c[38473,38548]
gaccgcatagttcagtggatagagcagctgccttctaagcagtgggtcga
gagttcgaatctctctgcggtcgcca
1−2tRNA−Trp(cca)[49824,49899]
aagggtgtagctcagttggtagagcagcggtctccaaaatcgttggttgt
tggtttgagtccaaccaccctttcca
>KM925136.1 Mycobacterium phage MiaZeal, complete genome
1−1tRNA−Gly(tcc)[91833,91905]

gcgcttgtggtcgaattggaaagactcctggcttccacccaggttatgca
ggttcgagtcctgtcgagcgctc
1−2tRNA−Tyr(gta)[93005,93077]
tggcccgtagctcaattggcagagcagccggcggtaattcggcgcgtctc
ggttcgagtccgggcgggctacc
>KM588359.1 Mycobacterium phage Carcharodon, complete genome
1−1tRNA−Thr(ggt)[28751,28828]
gatcctacggacgggaaagggacgtactacggtctggtaatccgcaggtc
gctggttcgagcccagctgggggcacca
>KJ743987.1 Sinorhizobium phage phiLM21, complete genome
1−1tRNA−Met(cat)[21148,21224]
gtcggagtagagcagcccggtagctcgcgtggctcataaccacgaggtcg
caggttcgaatcctgtctccgcaacca
>KR060090.1 Pseudoalteromonas phage Pq0, complete genome
1−1tRNA−Gly(tcc)c[14335,14408]
gcgtctgtagtgtaattggtagcacgctgtccttccaagtcagtagcggg
gttcgagtcccaacagacgctcca
1−2tRNA−Met(cat)c[14410,14486]
ttccctatagcttaattggcaaagcggtcgactcataatcgaatgaagga
gcggttcaaatccgcctagggaaacca
>KT222942.1 Mycobacterium phage Dusk, complete genome
1−1tRNA−Arg(cct)[62391,62474]
atggcccgtagctcagtcaggtagagcagccttgccccttgcgggcttgg
ttcgtccccggttcaaatccgggcgggccttgcc
1−2tRNA−Gly(tcc)[62477,62549]
gcgcccgtggtcgaattggaaagactcctggcttccacccaggttatgca
ggttcgagtcctgtcgggcgctc
>KM652554.1 Streptomyces phage Jay2Jay, complete genome
1−1tRNA−Thr(cgt)c[16870,16941]
gccccgctggcgtagtggaaacgcacttctttcgtaatgaagagtcaaga
gttcgattctcttgcggggctc
1−2tRNA−Gly(tcc)[72897,72969]
gcatcggtagtttaatggtagaacgtctgacttccaatcagacagcgcgg
gttcgattcccgctcggtgctcc
1−3tRNA−Gln(ctg)[73098,73170]
tccccttggtgtaaccggcaacacaacagactctgaatctgtaattcct
agttcgaatctaggttggggagc
1−4tRNA−Trp(cca)[73377,73448]
atgtccttcgtataatggtagtacaagagattccaaacctcttagcgtgg
gttcgattcctacaggacatgc
1−5tRNA−Pro(tgg)[74305,74377]
caggcacggggaagatggtaatccgttggctttggaagcctaagacactc
ggttcgattccgaggtgcctgac
1−6tRNA−Pro(tgg)[74381,74456]

cgggatatagctcagtttggtcagagcgctgcatttgggatgcagaagtc
gctggttcgaatccagttatcccgac
1-7tRNA-Ile(tat)[77444,77518]
gcgccagtagcataacggttcaatgcagtaggcttatatcctaccgagag
aaggttcaattccttcctggcgtac
1-8tRNA-Ser(gga)[78028,78114]
ggaaggtaagcgattggtttagcgggtgtcttggaaaggcactaagggtg
ttacagcccggcagggttcaattccctggccttccgc
1-9tRNA-Ser(tga)[78354,78443]
tggaggcacgcattggagtgcttatcggtcttgaaaaccgtgatgacgta
gtgatatgtcgtgggggttcgactccctctgtctccgccc
1-10tRNA-Asp(gtc)[81511,81587]
gcgtctgtagtatagtctggtctaatgcacctgattgtcaatcaggaaat
cgcgggttcaaatcccgtcaggcgcgc
1-11tRNA-Asp(gtc)[82125,82196]
cccggcgtggtgtaatggtaacatactgcgttgtcagcgcaatgtcgagg
gttcgattcccttcgtcgggac
1-12tRNA-Glu(ctc)[86672,86743]
gcccctatagtctaagggtaggatacaagattctcaatcttgtggtcgga
gttcaattctccgtaggggtac
1-13tRNA-Glu(ttc)[86992,87067]
gtcccggtagattctgctggtagaatcgctagactttcaatctagagtcc
gagggttcaattcccttccggggtac
1-14tRNA-Val(tac)[87323,87394]
gcccgcttagctcaatgggagagcgacggttttacacaccgtatacgggg
gttcgattccctcagtgggtac
1-15tRNA-Leu(tag)[87588,87663]
gcgccgttgacgggaactggcatacctagttgacttagaatcaactgttt
gggagttcgactctcccacggcgtac
1-16tRNA-Leu(tag)[87822,87896]
gcgtcggtagcccaatggcaggaggcaccagtcttaggaactggacagtg
tgagttcgagtctcactcggcgtac
1-17tRNA-Gly(gcc)[88218,88289]
tccgtagtggtgtagtggtaacatacttccttgccaaggaagcgttgcga
gttcgattctcgtctacgggac
1-18tRNA-Arg(tct)[88366,88439]
gcctcggtagcgtagtggatgacgcggatgccttctaagcatttacaggc
aggttcgattcctgtccgaggtac
1-19tRNA-Ala(tgc)[89222,89294]
gggcggttagtgttaatggtagcacgagtgctttgcacgcattaagcagg
ggttcgaatcccctactgtccac
1-20tRNA-Lys(ctt)[89455,89529]
gattcagtcgcagaattggtactgcaaaagactcttaatctttgaggatg
tgggttcgagtcccacctgaatcac

1-21tRNA-Asn(gtt)[90385,90461]
tccggtggggctactattggcagtagcgacgagctgttaactcgtttccc
tgtaggttcgattcctgcctccggagc
1-22tRNA-Asn(gtt)[91216,91289]
tgacctatagttcaattggcagagcagatggttgttacccatcaagttgc
aggttcgagtcctgctaggtcagc
1-23tRNA-Arg(cct)[91987,92059]
gtccctatagttcaagggaaagaacaagactctcctaaagtctagatgca
ggttcgaatcctgctggggatac
1-24tRNA-Ile(gat)[92308,92383]
gggagcgtagctcagtctggtcggagcaatgcactgataatgcatcggtc
gctggttcaaatccagccgttcctac
1-25tRNA-Ala(ggc)[92456,92529]
ggggtcgtagcataactggtaatgcacttgtatggcatacaagcgattgt
gggttcaaatcccatcgactccac
1-26tRNA-Lys(ctt)[92726,92799]
gctccggtagctcagttggcagagcaggggactcttaatcctcgtgtcgc
tggttcaagtccagttcggagtac
1-27tRNA-Lys(ttt)[92923,92999]
ggtccagtcgcacagtctggcagtgcaattgacttttaatctttgaggac
gtgggttcgaatcccacctggaccacc
1-28tRNA-Arg(tcg)[93002,93075]
gcccttttagctcattggatagagcgatggtcttcgaagccatgcgtagg
gagttcgattctctcaaggggcgc
1-29tRNA-Met(cat)[94369,94440]
ggcagtagggcgctcggtgtgccaacgactctcataaggtcgaggggctg
gttcgaatccagctattgctac
1-30tRNA-Met(cat)[94441,94512]
ttgcaggttagtgaaacggtatcacgtagggctcataatcctaaattcct
gttcgactcagggcctgccccc
1-31tRNA-Tyr(gta)[95146,95228]
gtgtgagcggcgaagttggagagacgcgacggtctgtaaaaccgttcctt
cgggtgagtcagttcgaatctgacctcacgcac
1-32tRNA-His(gtg)[95553,95628]
gggttggtagctcaattggattagagcgctgcgttgtggtcgcagtggtt
gagggttcgagtcccttccttcccac
1-33tRNA-Phe(gaa)[95699,95771]
gggcatgtagctcagttggtagagcttcggtgtgaagtaccgagtgcgag
ggttcgattccctacttgcccac
1-34tRNA-Cys(gca)[96362,96437]
ggggtagatgtagcgggtgctgccgacagtctgcaaaactgttcgtatat
tggagttcgactctcctctatccctc
1-35tRNA-Thr(tgt)[96453,96528]
gccaccttagctcatttggtgtagagcgacgctcttgtaaagcgtaggcg

gtcggttcgaaaccgacaggtggctc
1−36tRNA−Thr(ggt)[96621,96692]
gcccccatagtgctagtggtaacacatatccttggtacggatatatcgtc
agttcgattctgactgggggat
1−37tRNA−Arg(acg)[96823,96896]
ctctccataggctaattggataaaccagagtgctacgaacgctcaattgt
gggttcgagtcccgctggagagac
1−39tRNA−Ala(ggc)[100282,100356]
tgtcacgtaattcaataggaagaatcaatgcccttggccagggtaggtta
gaggttcgagtcctctcgtgacagc
1−40tRNA−Leu(gag)[105356,105438]
gcgcctatggtggaatggcagacacactagactgagactctaggccctga
tgggcgtccgggttcaactcccggtgggcgtac
1−41tRNA−Val(gac)[105876,105947]
acccgcttagctcaatggtagagcacttgctcgacacgcaagttacgtga
gttcaattctctcagtgggtac
1−42tRNA−Leu(caa)[106382,106458]
ggccctgtagcagaatcaggtatatgcggcggtctcaaacaccgcgtcaa
tgtgggttcgagtcccatcagggctac
1−43tRNA−Ser(gct)[106692,106775]
ggaaggtgagcgagaggtctcgcaggtagtttgctaaactgctggattgt
aaaaggttcggggttcgaatcctcctccttccgc
1−44tRNA−Gln(ttg)[116419,116492]
tcccctgtactctgctggttgggtagccagactttgaatctggtgtgcgt
aggttcgattcctgccaggggagc
>KR080204.1 Mycobacterium phage Mindy, complete genome
1−1tRNA−Arg(cct)[62055,62138]
atggcccgtagctcagtcaggtagagcagccttgccccttgcgggcttgg
ttcgtccccggttcaaatccgggcgggccttgcc
1−2tRNA−Gly(tcc)[62141,62213]
gcgcccgtggtcgaattggaaagactcctggcttccacccaggttatgca
ggttcgagtcctgtcgggcgctc
>KM401838.1 Mycobacterium phage VohminGhazi, complete genome
1−1tRNA−Asn(gtt)[5402,5474]
tgacgtgtagctcaatggcagagcacccggctgttaaccggacggttgaa
ggttcgagtccttccatgtcagc
1−2tRNA−Trp(cca)[5479,5552]
gcgctcctagctcaattggtagagcagcggtctccaaagccgcgagttcc
aggttcgagtcctggggagcgtgc
1−3tRNA−Gln(ctg)[5594,5666]
tccctgttcgtctaatcggtaagacaccgggctctggacccggcaatcga
ggttcgagtccttgatggggagc
>KP202971.1 Achromobacter phage JWF, complete genome
1−1tRNA−Lys(ctt)c[60835,60922]

acaggtgtagctcaaggagagcacccggttcttaaccggaaggcgcgcac
tgacgagtgtttaggttcgatacccgccacctgtacca
>KU998249.1 Gordonia phage Soups, complete genome
1−1tRNA−Asn(gtt)[5265,5339]
tggcttgtagctcaacggcagagcgctcggctgttaaccgagtggttgaa
ggttcgaatccttccaggccagcca
1−2tRNA−Lys(ctt)[5341,5414]
gcatccgtagctgagtggtttagctctgggctcttaacccggagacggag
gttcgattcctcccggatgcacca
1−3tRNA−Gln(ctg)[5416,5488]
tccccattcgtccaactggcaggacaccggagtctggttccggagatcga
ggttcgaatcctcggtagggagc
1−4tRNA−Arg(acg)c[49539,49618]
tcggttcgctggtcggaaggtgcgactcggtgcctacggtgatccggcga
tggtcccgttcgaggtgtgggaaccgatcc
>KU998244.1 Gordonia phage Smoothie, complete genome
1−2tRNA−Trp(cca)[6303,6376]
acggacgtagctcaactggtagagcagcggtctccaaaaccgcaggttgc
aggttcaagtcctgccggccgtgc
1−3tRNA−Tyr(gta)[6442,6524]
gtgggtgacacaccaatggtggtgtagcgggctgtaaccccgtggcgttc
tacgcgggcaggttcgatccctgccacccacac
1−4tRNA−Ser(cga)[6692,6775]
ggagggtatgcccatggtgggcacgcggtgtcgaataccgctatacggta
gccattccgggagttcgattcttctaccctccgc
1−5tRNA−Ser(gct)[6856,6939]
ggcgagttatccagtctgggactgggccccgttgctagcggacgcgcatc
cctgggtgtgaggttcgattcctcagctcgccgc
1−6tRNA−Asn(gtt)[7382,7457]
tccggtgtagctcaatcggcagagcgctcggctgttaaccgagaggttga
aggttcgaccccttccaccggagcca
1−7tRNA−Lys(ctt)[8009,8084]
gcctctgtagctcagttggtttagagcaaccgcctcttaagcggtgggtc
gcaggttcgaatccttccgggggcac
>KU998250.1 Gordonia phage Rosalind, complete genome
1−1tRNA−Asn(gtt)[5265,5339]
tggcttgtagctcaacggcagagcgctcggctgttaaccgagtggttgaa
ggttcgaatccttccaggccagcca
1−2tRNA−Lys(ctt)[5341,5414]
gcatccgtagctgagtggtttagctctgggctcttaacccggagacggag
gttcgattcctcccggatgcacca
1−3tRNA−Gln(ctg)[5416,5488]
tccccattcgtccaactggcaggacaccggagtctggttccggagatcga
ggttcgaatcctcggtagggagc

1−4tRNA−Arg(acg)c[49062,49141]
tcggttcgctggtcggaaggtgcgactcggtgcctacggtgatccggcga
tggtcccgttcgaggtgtgggaaccgatcc
>KU998251.1 Gordonia phage KatherineG, complete genome
1−1tRNA−Asn(gtt)[5265,5339]
tggcttgtagctcaacggcagagcgctcggctgttaaccgagtggttgaa
ggttcgaatccttccaggccagcca
1−2tRNA−Lys(ctt)[5341,5414]
gcatccgtagctgagtggtttagctctgggctcttaacccggagacggag
gttcgattcctcccggatgcacca
1−3tRNA−Gln(ctg)[5416,5488]
tccccattcgtccaactggcaggacaccggagtctggttccggagatcga
ggttcgaatcctcggtagggagc
>KU998245.1 Gordonia phage OneUp, complete genome
1−1tRNA−Ser(gct)[4110,4181]
tccccgaatgctcgagggcttgagcgctgctgacgagcagagtgtcgtgg
gttcgaatcccacacggggagc
1−3tRNA−Trp(cca)[4261,4333]
atgggcgtagctcaattggaagagcagcggtctccaaaaccgccggtgta
ggttcgagtcctgccgcctgtgc
1−4tRNA−Tyr(gta)[4400,4482]
gtgggtgacacaccaatggtggtgtagccggctgtaacccggtggcgttc
tacgcgggcaggttcgatccctgccacccacac
1−5tRNA−Ser(gct)[4719,4802]
ggcgagttatccagtctgggactggctaccgttgctagcggctaggcatc
ttcgggtgtgtggttcgactcctcaactcgccgc
1−6tRNA−Phe(gaa)[5272,5349]
cggcgtgtagctcagattggtgagagcgctcggttgaaacccgagaggtc
gcaggttcgagccctgccgcgccggcca
1−7tRNA−Asn(gtt)[5351,5426]
ttcgacgtagctcaattggcagagcgtccgactgttaatcggatggttga
aggttcgagtccttccgtcggagcca
1−8tRNA−Met(cat)c[48720,48795]
gccctggtagccaagttggtgaaggcctccgactcataatcggaagatgc
gtgggttcgagccccacccagggcac
>KR053199.1 Gordonia phage GMA4, complete genome
1−1tRNA−Tyr(gta)[5132,5213]
gggagtgacgacgctggtgcgtcacccggactgtaaatccggtgcgaaag
catggcaggttcgatccctgccactcccacca
>KU998237.1 Gordonia phage Vendetta, complete genome
1−1tRNA−Lys(ttt)c[24130,24203]
gcccctttagctcagttggtagagctggtgacttttaatcactaggtcgt
aggttcgagtcctacagggggcac
>KU998253.1 Gordonia phage Orchid, complete genome

1−2tRNA−Leu(taa)c[70492,70578]
gcccctgtggcgaaattcggaatacgcgggagacttaaaatcttctagcc
tttatggctttatcggttcaagtccggtcaggggtac
>KP790011.1 Gordonia phage Gsput1, complete genome
1−1tRNA−Ser(cga)[846,929]
ggagggtgtgccgatggtcggcacgcggtttcgaaagccgctacacggta
accattccgggggttcaattcctccgccctccgc
>KU998238.1 Gordonia phage Splinter, complete genome
1−1tRNA−Lys(ttt)c[24130,24203]
gcccctttagctcagttggtagagctggtgacttttaatcactaggtcgt
aggttcgagtcctacagggggcac
>KU998246.1 Gordonia phage ClubL, complete genome
1−2tRNA−Trp(cca)[6312,6385]
acggacgtagctcaactggtagagcagcggtctccaaaaccgcaggttgc
aggttcaagtcctgccggccgtgc
1−3tRNA−Tyr(gta)[6451,6533]
gtgggtgacacaccaatggtggtgtagcgggctgtaaccccgtggcgttc
tacgcgggcaggttcgatccctgccacccacac
1−4tRNA−Ser(cga)[6701,6784]
ggagggtatgcccatggtgggcacgcggtgtcgaataccgctatacggta
gccattccgggagttcgattcttctaccctccgc
1−5tRNA−Ser(gct)[6902,6985]
ggcgagttatccagtctgggactgggccccgttgctagcggacgtgcatc
ttcgggtgtgaggttcgattcctcagctcgccgc
1−6tRNA−Asn(gtt)[7067,7142]
tggcacgtagcttaattggcaaagtcgtcggttgttacccgattggatga
aggttcaaatccttccgtgtcagcca
1−7tRNA−Lys(ctt)[7940,8015]
gcctctgtagctcagttggtttagagcaaccgcctcttaagcggtgggtc
gcaggttcgaatccttccgggggcac
>KU998247.1 Gordonia phage Bachita, complete genome
1−2tRNA−Trp(cca)[6811,6884]
acggacgtagctcaactggtagagcagcggtctccaaaaccgcaggttgc
aggttcaagtcctgccggccgtgc
1−3tRNA−Tyr(gta)[6950,7032]
gtgggtgacacaccaatggtggtgtagcgggctgtaaccccgtggcgttc
tacgcgggcaggttcgatccctgccacccacac
1−4tRNA−Ser(cga)[7200,7283]
ggagggtatgcccatggtgggcgcgcggtgtcgaataccgctatacggta
tccattccgggagttcgattcttctaccctccgc
1−5tRNA−Ser(gct)[7401,7484]
ggcgagttatccagtctgggactgggccccgttgctagcggacgtgcatc
ttcgggtgtgaggttcgattcctcagctcgccgc
1−6tRNA−Asn(gtt)[7566,7641]

tggcacgtagcttaattggcaaagtcgtcggttgttacccgattggatga
aggttcaaatccttccgtgtcagcca
1-7tRNA-Lys(ctt)[8439,8514]
gcctctgtagctcagttggtttagagcaaccgcctcttaagcggtgggtc
gcaggttcgaatccttccgggggcac
>KU998240.1 Gordonia phage Woes, complete genome
1-1tRNA-Asn(gtt)[20495,20567]
tggtccgtagctcaatggcagagcgcccgactgttaatcgggtggttgaa
ggttcgagtccttccggtccagc
>KU998241.1 Gordonia phage Monty, complete genome
1-1tRNA-Asn(gtt)[19267,19341]
tgatccatagctcaattcggcagagcgcccgactgttaatcgggtggttg
taggttcaagtcctactgggtcagc
>KU199710.1 Pseudomonas phage JBD44, complete genome
1-1tRNA-Thr(tgt)[6179,6254]
gccggtatggcgcaacagggagcgctgctgatttgtaatcagagggttgc
gggttcgactcctgctgccggcacca
>KX557283.1 Gordonia phage Remus, complete genome
1-1tRNA-Asn(gtt)[5110,5185]
tcgcctgtagctcaattggcagagcacccggctgttaaccgggcggttga
aggttcgagtccttccaggcgagcca
1-2tRNA-Lys(ctt)[5187,5260]
gcatctgtagctgagtggtttagctctgggctcttaacccggagacggag
gttcgattcctcccagatgcacca
1-3tRNA-Gln(ctg)[5262,5334]
tccccattcgtccaattggtaggacaccggagtctggttccggagatcga
ggttcgaatcctcggtagggagc
>KX268652.1 Acinetobacter phage vB_AbaS_TRS1, complete genome
1-1tRNA-Arg(tct)[38342,38418]
gcgccattcgtctaattggataagacatcataattctagtgtgattgatg
tgggttcgagtcctgcatggcgtgcca
>KX130668.1 Enterobacteria phage vB_EcoS_NBD2, complete genome
1-1tRNA-Ser(gct)[9424,9511]
ggaaggatggcagagaggacgattgcgcgcggttgctaaccgtgaagccg
gaaacggctcaagggttcgaatccctttctttccacca
>KU892558.1 Lactococcus phage PLgT-1, complete genome
1-1tRNA-Met(cat)c[587,660]
ggttctttagcttagttggttaaagtcctccgctcataacggagttagcg
ctggttcgaatccagcaagaacca
1-2tRNA-Lys(ttt)c[24608,24682]
gtggctgcatggtcaaggggttaagacactgcacttttaatgcagaggcg
tgagttcgaatctcactcagtcaca
>KX129925.1 Pseudomonas phage NP1, complete genome
1-1tRNA-Leu(gag)c[11473,11566]

cagaggataccagaccggcgaaccgggaagggttttgagagaagcctctt
cggcgaaccggaggccggttacgttcgaatcgatcttgtttgcc

>KX636165.1 Mycobacterium phage Gengar, complete genome

1-1tRNA-Trp(cca)[847,922]
tcgcaggtagctcaattggtagagcagcggtctccaaaaccgccggttgc
aggttcgagtcctgctctgcgggcca

>KX557280.1 Gordonia phage JSwag, complete genome

1-1tRNA-Asn(gtt)[5142,5217]
tcgcctgtagctcaattggcagagcacccggctgttaaccgggcggttga
aggttcgagtccttccaggcgagcca

1-2tRNA-Lys(ctt)[5219,5292]
gcatctgtagctgagtggtttagctctgggctcttaacccggagacggag
gttcgattcctcccagatgcacca

1-3tRNA-Gln(ctg)[5294,5366]
tccccattcgtccaactggcaggacaccggagtctggttccggagatcga
ggttcgaatccttggtggggagc

1-4tRNA-Arg(acg)c[49167,49246]
tcggttcgctggtcggaaggtgcgactcggtgcctacggtgatccggcga
tggtcccgttcgaggtgtgggaaccgatcc

>KX557276.1 Gordonia phage Cucurbita, complete genome

1-2tRNA-Trp(cca)[6312,6385]
acggacgtagctcaactggtagagcagcggtctccaaaaccgcaggttgc
aggttcaagtcctgccggccgtgc

1-3tRNA-Tyr(gta)[7820,7902]
gtgggtgacacaccaatggtggtgtagcgggctgtaaccccgtggcgttc
tacgcgggcaggttcgatccctgccacccacac

1-4tRNA-Ser(cga)[8070,8153]
ggagggtatgcccatggtgggcgcgcggtgtcgaataccgctatacggta
tccattccgggagttcgattcttctaccctccgc

1-5tRNA-Ser(gct)[8234,8317]
ggcgagctatccggtctgggaccgggccccgttgctagcggacgtgcatc
tccgggtgtgaggttcgattcctcagctcgccgc

1-6tRNA-Asn(gtt)[8669,8744]
tccggtgtagctcaatcggcagagcgctcggctgttaaccgagaggctga
aggttcgacccccttccaccggagcca

1-7tRNA-Lys(ctt)[9298,9373]
gcctctgtagctcagttggtttagagcaaccgcctcttaagcggtgggtc
gcaggttcgaatccttccgggggcac

>KX507361.1 Mycobacterium phage Gardann, complete genome

1-1tRNA-Thr(cgt)[62844,62917]
gccaccttagctcagttggtagagcagccccttcgtaacgggcaggtcag
cggttcgactccgctaggtggctc

1-2tRNA-Pro(tgg)[63139,63213]
ctggctgtagctcaacttggtagagcgctggctttgggtgccaggggttg

caggttcaaatcctgctagccagac
1-3tRNA-Trp(cca)[63343,63416]
agctcggtagctcaattggtagagcagcggtctccaaagccgccggttcc
ccgttcgagtcggggtcgggttgc
1-4tRNA-Tyr(gta)[63527,63609]
gtggtggttgggcttgttggttggcccacctgactgtaaatcaggcgttt
tggcatcgggggttcgattccctcccaccacac
1-5tRNA-Leu(tag)[63846,63920]
ggccctctagcccaattggcagaggcacaggttttaggtacctgtcagtg
cgagttcgagtctcgcgggggccac
1-6tRNA-His(gtg)[63921,63990]
gcttagtagcctagtggtaaggcagccggttgtgatccggtgaacctgag
ttcgattctcagctaaagcc
1-7tRNA-Gln(ctg)[63995,64069]
tgaccggtagcacaactggtagttgcgccgcgctctggacgcggaggttg
ttggttcgatcccagctcggtcagc
1-8tRNA-Gly(tcc)[64185,64259]
gtgctagtaacccatgttggctgggtgcctgacttccactcaggatttcg
cgggttcgattcctgtctagcacac
1-9tRNA-Lys(ctt)[64302,64379]
tgcctcgttagctcagttggtagagctgccgactcttaatcggtaggtca
caggttcaagtcctgtacggggtaccca
1-10tRNA-Cys(gca)[65561,65633]
gccgttgtgtccgagtggttaggtgccaggctgcaaacttggttagtcgc
gttcgattcgcggggacggctcc
1-11tRNA-Asn(gtt)[65902,65975]
tcctccgtagctcaattggcagagcgcgcgactgttaatcgcgtggttgg
tggttcgagtccacccgttggagc
1-12tRNA-Lys(ttt)c[66169,66243]
gcccctatagctcagttggtagagcaggagacttttaatcttcgggtcct
aggttcgatccctagtgggggcacc
>KX557272.1 Gordonia phage Bantam, complete genome
1-2tRNA-Met(cat)c[46613,46687]
gccccggtagctcagtcggttagagccggtcactcataatgactaggtcg
caggttcgagccctgcccggggcac
>KU963248.1 Gordonia phage Yvonnetastic, complete genome
1-1tRNA-Phe(gaa)[6354,6430]
cggcgtgtagctcagttaggtaagagcgcccggttgaaacccgggaggtt
cgtaggttcgatccctaccgcgtcggc
1-2tRNA-Asn(gtt)[6541,6616]
tccggagtagcttaattggcagagctccaggttgttaccctgatgtagat
ataggttcaagtcctatctccggagc
1-3tRNA-Trp(cca)[6629,6701]
taggatctagctcaactggtagagcggtggtctccaaagccactggtagg

ggttcaagtcctctggtcctagc
1-4tRNA-Lys(ctt)[6704,6778]
gcttccgtagctcagcggtttagagcagctgattcttaatcagcgggtcg
aaggttcaaatccttccgggggcac
1-5tRNA-Asp(gtc)[6823,6896]
ccttccgtagcttagtggttaaagcactcgcctgtcgagcgaggggacgc
gcgttcgaattgcgtcggagggac
1-6tRNA-Ser(cga)[7225,7310]
ggagagtatgcccacggtgggcgcgcggtgtcgaataccgctatacggta
aacattccgggggttcaactcctctgctctccgcca
1-7tRNA-Ser(gct)[7312,7395]
ggcgagatatccagactgggtctggtccccgttgctagcgggagggcacc
tacgggtgtgggattcgatttctcatctcgccgc
>KU761559.1 Mycobacterium phage ArcherNM, complete genome
1-1tRNA-Leu(aag)[6231,6344]
GCAGCCCGTCGTTTAATCGGCAGGACGGCTGGTGAAGGCGCATAACGCGACGGCTGACGA
GTCGTCGGCGTGCCGAAGTACCAGGAGGTGGGGGTTCGACTCCCCTCGGGCGCA
>KU728633.1 Mycobacterium phage Bipper, complete genome
1-1tRNA-Met(cat)[32891,32965]
ggggcggtagctcagtcggttagagccacggactcataatccgttggtcg
tgggttcgagccccacccgccctac
>KU963245.1 Gordonia phage Hotorobo, complete genome
1-1tRNA-Asn(gtt)[20134,20208]
tgatccatagctcaattcggcagagcgcccgactgttaatcgggtggttg
taggttcaagtcctactgggtcagc
>KU761558.1 Mycobacterium phage Loser, complete genome
1-1tRNA-Trp(cca)[6166,6239]
gggcacgtagctcaatcggtagagcagcggtctccaaagccgcaggttcc
aggttcgaaccctggcgggctcgc
>KU613353.1 Mycobacterium phage Catalina, complete genome
1-1tRNA-Glu(ctc)[5907,5979]
ggtccgttggagtagcggcaactcatctgactctcactcagaagatcgcg
ggttcaaatcccgcacggactac
>KU985093.1 Mycobacterium phage EvilGenius, complete genome
1-1tRNA-Gln(ctg)[4806,4880]
tccccgttcgtctaattggcaagacgccgggttctggccccggtaattga
ggttcgaatccttgatggggaacca
>KU935727.1 Mycobacterium phage Panchino, complete genome
1-1tRNA-Thr(ggt)[29167,29244]
gatcctacggacgggaaagggacgtactacggtctggtaatccgcaggtc
gctggttcgagcccagctgggggcacca
>KU695581.1 Mycobacterium phage Mulciber, complete genome
1-1tRNA-Trp(cca)[6118,6193]
gcgttcctagctcaattggtagagcagcggtctccaaagccgcgggttcc

aggttcgagccctggggaacgtgcca
>KU935728.1 Mycobacterium phage Xeno, complete genome
1-1tRNA-Thr(ggt)[27208,27285]
gatcctacggacgggaaagggacgtactacggtctggtaatccgcaggtc
gctggttcgagcccagctgggggcacca
>KX017521.1 Salmonella phage 118970_sal2, complete genome
1-1tRNA-Met(cat)c[28730,28805]
agttagttggcagagtggttatgcacctccttcatacggagcgactacag
tggttcaaatccactactaactacca
1-2tRNA-Ile(gat)c[28812,28887]
gctctgatagttcaacaggttagaacaggcgaccgataatcgtcaaatct
tggttcgatcccaagtcggagtacca
1-3tRNA-Thr(tgt)c[29257,29331]
gctcctaaagcattgctggcgatgcagttgccttgtaagcatctgaaccg
ggttcgattcctggtgggagcacca
1-4tRNA-Gly(tcc)c[29736,29810]
gcgtgattagttcagtggctagaataactggcttccaaccagtagacacg
agttcgactctcgtatcccgcacca
1-5tRNA-Gln(ttg)c[29818,29893]
tggagagtagtgtaacggttagcacaacggcctttgactccgttaatggt
aggttcgattcctccttctccagcca
1-6tRNA-Gln(ctg)c[29902,29976]
ttgggatgtagatcaattggcagatcgtcggcctctgactccgaaggttc
cacgttcgatccgtggcatcccagt
1-7tRNA-Arg(acg)c[30209,30283]
gcgtccttatttcaacggaaagaatgtaaagctacgaactttacgatcgg
ggttcgattccctgaggatgcacca
1-8tRNA-His(gtg)c[30290,30366]
gtggctatatcataattggttaatgatcctgattgtgaatcaggcctatg
tggattcgaattccactagccaccccca
1-9tRNA-Ser(tga)c[31064,31153]
ggaaggtaggacatagtggtatgtaacaggtcttgaaaacctgcccgctg
tagcgatatggtgatggttcgactccattaccttcctcca
1-10tRNA-Ser(gga)c[31161,31252]
ggaagagcaaatcgataggtgacgaaaaccgcttggaaagtggctgactt
gttaaagcaggcttgagagttcgaatctctcttcttccgcca
1-11tRNA-Leu(tag)c[31427,31507]
ggggatgtggcgaaattggcagacgcgctagatttaggttctagtcttcg
ggtgtgggttcgagtccctccatccctacca
1-12tRNA-Ala(tgc)c[31753,31828]
ggggtcataggttatttggttaaacttactgccttgcaagcagtggaact
cagttcaattctgagtgactccacca
1-13tRNA-Ala(tgc)c[31834,31912]
ggggaatgggtctgcttggagtggacacctcgcttgcaccgaggatatca

gaagagttcgaatctcttattctccacca
1-14tRNA-Val(tac)c[32553,32626]
gctcggttagtttaatgggagaaccccgtctttacacggcggttgcgata
gttcgattctatcaccgagtacca
1-15tRNA-Lys(ttt)c[33095,33173]
agatcgctagctcaattggtttagcagcacccggcttttaaccggaaggt
tctgggttcgagtcccaggcggtctacca
1-16tRNA-Met(cat)c[33362,33439]
tgcgggttagatctctggcagagatcgctagtctcataagctagaaagag
gtaggttcgattcctgcacccgcttcca
1-17tRNA-Pro(tgg)c[33446,33522]
ctccgattagctcaattggctagagtacaccgtttggggcggtggggttg
aaggttcgagtccttcattggagacca
1-18tRNA-Gly(gcc)c[34054,34127]
gcgttcgtagttaaaaggtataatttttggttgccaaccagaagttgagg
gttcgagtccctccgaccgcacca
1-19tRNA-Lys(ctt)c[34266,34343]
gcatctgtagcttagttgatttaaagcaaccgactcttaatcggaagatc
ctgagtttgaatctcagcaggtgtacca
1-20tRNA-Asp(gtc)c[34901,34977]
gcgatcggggctggcttggtaatggtactcccctgtcacgggagagaatg
tgggttcgaatcccatcggtcgcgcca
1-21tRNA-Asn(gtt)c[35200,35282]
gggtcgttagccaagcggtttggcggtggactgttaatccatgttgaaag
acaacgtaggttcgaatcctacacggcccgcca
1-22tRNA-Cys(gca)c[35290,35365]
cgaccgttggctgaatggcttaggcggaggcctgcaaaacctccttatgt
gagttcgaatctcatgcggtcgtcca
1-23tRNA-Trp(cca)c[36186,36262]
attcctagagtgttactggacagcatgtcggtctccaaaaccgtacggtc
taggttcgagtcctagtaggtttgcca
1-24tRNA-Glu(ttc)c[36271,36347]
gtcctgttagacaaactggtaaagtcactaccctttcaaggtaggatttg
cgggttcgatccccgcacaggacgcca
1-25tRNA-Tyr(gta)c[36356,36446]
tgttggattagtatcgtagaggtagcgaagcagactgtaaatctgccgac
tcggaagggtctcgggtggttcgactccatcatccaacatc
1-26tRNA-Leu(taa)c[37360,37436]
gggggtgtaatcgaattggcataggtactggacttaaaattcaggttttg
tgggttcgaatcccaccacccctacca
1-27tRNA-Met(cat)c[38066,38143]
ggttctcaagctcatttggtatgagccgtcgcctcataagcgaaaggtag
gtaggttcgaatcctccgggagccacca
1-28tRNA-Ser(gct)c[38149,38242]

ggaagattaaccctaaaggtaagggagctgtttgctaaacagccagtagc
tgtggagacacggtgtgccagttcgaatctggcatcttcctcca
1-29tRNA-Arg(tct)c[43637,43711]
cggggtgtagtctaagggataggcaggagtcttctaaattcctttatgca
ggttcgaatcctgtcacctcggcca
>KX523699.2 Salmonella phage IME207, complete genome
1-1tRNA-Arg(tct)c[23617,23692]
acaccattagctcaacggatagagcggtagccttctaagctattggttgc
aggttcgagtcctgcatggtgtgcca
1-2tRNA-Ser(gct)c[23699,23786]
ggatagttggctgagaggccgaaagcagtcggttgctaaccgataaaccg
gaaacggttcacaggttcgaatcctgtactatccgcca
>KU927497.2 Salmonella phage 100268_sal2, complete genome
1-1tRNA-Met(cat)c[29621,29696]
agttagttggcagagtggttatgcacctccttcatacggagcgactacag
tggttcaaatccactactaactacca
1-2tRNA-Ile(gat)c[29703,29778]
gctctgatagttcaacaggttagaacaggcgaccgataatcgtcaaatct
tggttcgatcccaagtcggagtacca
1-3tRNA-Thr(tgt)c[30148,30222]
gctcctaaagcattgctggcgatgcagttgccttgtaagcatctgaaccg
ggttcgattcctggtgggagcacca
1-4tRNA-Gly(tcc)c[30627,30701]
gcgtgattagttcagtggctagaataactggcttccaaccagtagacacg
agttcgactctcgtatcccgcacca
1-5tRNA-Gln(ttg)c[30709,30784]
tggagagtagtgtaacggttagcacaacggcctttgactccgttaatggt
aggttcgattcctccttctccagcca
1-6tRNA-Gln(ctg)c[30791,30866]
tgggatgtagatcaattggcagatcgtcggcctctgactccgaaggttcc
acgttcgatccgtggcatcccagcca
1-7tRNA-Arg(acg)c[31099,31173]
gcgtccttatttcaacggaaagaatgtaaagctacgaactttacgatcgg
ggttcgattccctgaggatgcacca
1-8tRNA-His(gtg)c[31180,31256]
gtggctatatcataattggttaatgatcctgattgtgaatcaggcctatg
tggattcgaattccactagccaccccca
1-9tRNA-Ser(tga)c[31953,32042]
ggaaggtaggacatagtggtatgtaacaggtcttgaaaacctgcccgctg
tagcgatatggtgatggttcgactccattaccttcctcca
1-10tRNA-Ser(gga)c[32050,32141]
ggaagagcaaatcgataggtgacgaaaaccgcttggaaagtggctgactt
gttaaagcaggcttgagagttcgaatctctcttcttccgcca
1-11tRNA-Leu(tag)c[32316,32396]

ggggatgtggcgaaattggcagacgcgctagatttaggttctagtcttcg
ggtgtgggttcgagtccctccatccctacca
1-12tRNA-Ala(tgc)c[32642,32717]
ggggtcataggttatttggttaaacttactgccttgcaagcagtggaact
cagttcaattctgagtgactccacca
1-13tRNA-Ala(tgc)c[32723,32801]
ggggaatgggtctgcttggagtggacacctcgcttgcaccgaggatatca
gaagagttcgaatctcttattctccacca
1-14tRNA-Val(tac)c[33442,33515]
gctcggttagtttaatgggagaaccccgtctttacacggcggttgcgata
gttcgattctatcaccgagtacca
1-15tRNA-Lys(ttt)c[33984,34062]
agatcgctagctcaattggtttagcagcacccggcttttaaccggaaggt
tctgggttcgagtcccaggcggtctacca
1-16tRNA-Met(cat)c[34251,34328]
tgcgggttagatctctggcagagatcgctagtctcataagctagaaagag
gtaggttcgattcctgcacccgcttcca
1-17tRNA-Pro(tgg)c[34335,34411]
ctccgattagctcaattggctagagtacaccgtttggggcggtggggttg
aaggttcgagtccttcattggagacca
1-18tRNA-Gly(gcc)c[34943,35016]
gcgttcgtagttaaaaggtataatttttggttgccaaccagaagttgagg
gttcgagtccctccgaccgcacca
1-19tRNA-Lys(ctt)c[35155,35232]
gcatctgtagcttagttgatttaaagcaaccgactcttaatcggaagatc
ctgagtttgaatctcagcaggtgtacca
1-20tRNA-Asp(gtc)c[35790,35866]
gcgatcggggctggcttggtaatggtactcccctgtcacgggagagaatg
tgggttcgaatcccatcggtcgcgcca
1-21tRNA-Asn(gtt)c[36089,36171]
gggtcgttagccaagcggtttggcggtggactgttaatccatgttgaaag
acaacgtaggttcgaatcctacacggcccgcca
1-22tRNA-Cys(gca)c[36179,36254]
cgaccgttggctgaatggcttaggcggaggcctgcaaaacctccttatgt
gagttcgaatctcatgcggtcgtcca
1-23tRNA-Trp(cca)c[37075,37151]
attcctagagtgttactggacagcatgtcggtctccaaaaccgtacggtc
taggttcgagtcctagtaggtttgcca
1-24tRNA-Glu(ttc)c[37160,37236]
gtcctgttagacaaactggtaaagtcactaccctttcaaggtaggatttg
cgggttcgatccccgcacaggacgcca
1-25tRNA-Tyr(gta)c[37245,37335]
tgttggattagtatcgtagaggtagcgaagcagactgtaaatctgccgac
tcggaagggtctcgggtggttcgactccatcatccaacatc

1−26tRNA−Leu(taa)c[38249,38325]
gggggtgtaatcgaattggcataggtactggacttaaaattcaggttttg
tgggttcgaatcccaccacccctacca
1−27tRNA−Met(cat)c[38955,39032]
ggttctcaagctcatttggtatgagccgtcgcctcataagcgaaaggtag
gtaggttcgaatcctccgggagccacca
1−28tRNA−Ser(gct)c[39038,39131]
ggaagattaaccctaaaggtaagggagctgtttgctaaacagccagtagc
tgtggagacacggtgtgccagttcgaatctggcatcttcctcca
1−29tRNA−Arg(tct)c[44526,44600]
cggggtgtagtctaagggataggcaggagtcttctaaattcctttatgca
ggttcgaatcctgtcacctcggcca
>KT337365.1 Streptococcus phage phiARI0746, complete genome
1−1tRNA−Ser(gct)c[18374,18464]
ggagagttggcagagtcaggttgaatgcgcccgtttgctagacgggtgat
cgcctatgtgcggtccgtgggttcaaatcccacactctcct
>KM236244.1 Salmonella phage Stitch, complete genome
1−1tRNA−Met(cat)c[28496,28569]
agttagttggcagagcggttatgcacctccttcatacggagtgactacag
tggttcaaatccactactaactac
1−2tRNA−Ile(gat)c[28576,28651]
gctccgatagttcaaacggttagaacaggcgaccgataatcgtcaaatct
tggttcgatcccaagtcggagtacca
1−3tRNA−Thr(tgt)c[29021,29095]
gctcctaaagcattgctggcgatgcagttgccttgtaagcatctgaaccg
ggttcgattcctggtgggagcacca
1−4tRNA−Gly(tcc)c[29501,29575]
gcgtgattagttcagtggctagaataactggcttccaaccagtagacacg
agttcgactctcgtatcccgcacca
1−5tRNA−Gln(ttg)c[29582,29657]
tggagagtagtgtaacggttagcacaacggcctttgactccgttaatggt
aggttcgattcctccttctccagcca
1−6tRNA−Gln(ctg)c[29664,29739]
tgggatgtagatcaattggcagatcgtcggcctctgactccgaaggttcc
acgttcgatccgtggcatcccagcca
1−7tRNA−Arg(acg)c[29972,30046]
gcgtccttatttcaatggaaagaatgtaaagctacgaactttacgatcgg
ggttcgattccctgaggatgcacca
1−8tRNA−His(gtg)c[30053,30129]
gtggctatatcataattggttaatgatcctgattgtgaatcaggcctatg
tggattcgaattccactagccacccca
1−9tRNA−Ser(tga)c[30488,30577]
agaagataggacgtagtggtacgtaaccggtcttgaaaaccggcccgctg
tagtgatatggtgatggttcgactccattatcttctgcca

1-10tRNA-Ser(gga)c[30955,31044]
ggaagagcaaatcgataggtgacgaaaaccgcttggaaagtggctgactg
gtaaaacggcttgagagttcgaatctctcttcttccgcca
1-11tRNA-Leu(tag)c[31219,31299]
ggggatgtggcgaaattggcagacgcgctagatttaggttctagtcttcg
ggtgtgggttcgagtccctccatccctacca
1-12tRNA-Ala(tgc)c[31545,31620]
ggggtcataggttatttggttaaacttactgccttgcaagcagtggaact
cagttcaattctgagtgactccacca
1-13tRNA-Ala(tgc)c[31626,31704]
ggggaatgggtctgcttggagtggacacctcgcttgcaccgaggatatca
gaagagttcgaatctcttattctccacca
1-14tRNA-Val(tac)c[32367,32440]
gctcggttagtttaatgggagaaccccgtctttacacggcggttgcgata
gttcgattctatcaccgagtacca
1-15tRNA-Lys(ttt)c[32909,32987]
agatcgctagctcaattggtttagtagcacccggcttttaaccggaaggt
tctgggttcgagtcccaggcggtctacca
1-16tRNA-Met(cat)c[33176,33253]
tgcgggttagatctctggtagagatcgctagtctcataagctagaaagag
gtaggttcgattcctacacccgcttcca
1-17tRNA-Pro(tgg)c[33260,33337]
ctccgtgtagctcagtttggccagagcgttccgtttggggcggtagggtc
gggggttcaaatcctcccacggagacca
1-18tRNA-Gly(gcc)c[33757,33830]
gcgttcgtagttaaaaggtataatttttggttgccaaccagaagttgagg
gttcgattccctccgaccgcacca
1-19tRNA-Lys(ctt)c[34044,34119]
acatccatagctcaatggttagagctaccgcctcttaagcggagggttct
aggttcaagtcctagtgggtgtacca
1-20tRNA-Asp(gtc)c[34196,34272]
gcgatcggggctggcttggtaatggtactcccctgtcacgggagagaatg
tgggttcgaatcccatcggtcgcgcca
1-21tRNA-Asn(gtt)c[34571,34653]
gggtcgttagccaagtggtttggcggtggactgttaatccatgtcgaaag
acaacgtaggttcgaatcctacacggcccgcca
1-22tRNA-Cys(gca)c[34661,34736]
cgaccgttggctgaatggcttaggcggaggcctgcaaaacctccttatgt
gagttcgaatctcatgcggtcgtcca
1-23tRNA-Trp(cca)c[35557,35633]
attcctagagtgttactggacagcatgtcggtctccaaaaccgtacggtc
taggttcgagtcctagtaggtttgcca
1-24tRNA-Glu(ttc)c[35642,35718]
gtcctgttagacaaactggtaaagtcactaccctttcaaggtaggatttg

cgggttcgatccccgcacaggacgcca
1-25tRNA-Tyr(gta)c[35726,35816]
gttggattagtatcgtagaggtagcgaagcagactgtaaatctgccgact
cgaaagggtctcgggtggttcgactccatcatccaacacca
1-26tRNA-Leu(taa)c[36731,36807]
gggggtgtaatcgaattggcataggtactggacttaaaattcaggttttg
tgggttcgaatcccaccacccctacca
1-27tRNA-Met(cat)c[37359,37436]
ggttctcaagctcatttggtatgagccgtcgcctcataagcgaaaggtag
gtaggttcgaatcctccgggagccacca
1-28tRNA-Ser(gct)c[37442,37535]
ggaagattaaccctaaaggtaagggagctgtttgctaaacagccagtagc
tgtggagacacggtgtgccagttcgaatctggcatcttcctcca
1-29tRNA-Arg(tct)c[42928,43002]
cggggtgtagtctaagggataggcaggagtcttctaaattcctttatgca
ggttcgaatcctgtcacctcggcca
>JQ740791.1 Lactococcus Phage ASCC287, complete genome
1-1tRNA-Trp(cca)[31764,31836]
tgcgagcatagtatagtggtaatgctacagattccaaacctgtaaacgtg
ggttcgattcctgctgttcgtgt
1-2tRNA-Pro(tgg)[31890,31961]
caggatatggtgtcaatggtagcatgcgtgttttgggaacatgtggtgtt
ggttcgagtccagctatcctga
>JQ740793.1 Lactococcus Phage ASCC324, complete genome
1-1tRNA-Trp(cca)[31460,31532]
tgcgagcatagtatagtggtaatgctacagattccaaacctgtaaacgtg
ggttcgattcctgctgttcgtgt
1-2tRNA-Pro(tgg)[31586,31657]
caggatatggtgtcaatggtagcatgcgtgttttgggaacatgtggtgtt
ggttcgagtccagctatcctga
>JQ740794.1 Lactococcus Phage ASCC337, complete genome
1-1tRNA-Trp(cca)[31458,31530]
tgcgagcatagtatagtggtaatgctacagattccaaacctgtaaacgtg
ggttcgattcctgctgttcgtgt
1-2tRNA-Pro(tgg)[31584,31655]
caggatatggtgtcaatggtagcatgcgtgttttgggaacatgtggtgtt
ggttcgagtccagctatcctga
>JQ740798.1 Lactococcus Phage ASCC368, complete genome
1-1tRNA-Trp(cca)[31459,31531]
tgcgagcatagtatagtggtaatgctacagattccaaacctgtaaacgtg
ggttcgattcctgctgttcgtgt
1-2tRNA-Pro(tgg)[31585,31656]
caggatatggtgtcaatggtagcatgcgtgttttgggaacatgtggtgtt
ggttcgagtccagctatcctga

>JQ740799.1 Lactococcus Phage ASCC395, complete genome

1-1tRNA-Trp(cca)[31422,31494]
tgcgagcatagtatagtggtaatgctacagattccaaacctgtaaacgtg
ggttcgattcctgctgttcgtgt
1-2tRNA-Pro(tgg)[31548,31619]
caggatatggtgtcaatggtagcatgcgtgttttgggaacatgtggtgtt
ggttcgagtccagctatcctga

>JQ740800.1 Lactococcus Phage ASCC397, complete genome

1-1tRNA-Trp(cca)[31424,31496]
tgcgagcatagtatagtggtaatgctacagattccaaacctgtaaacgtg
ggttcgattcctgctgttcgtgt
1-2tRNA-Pro(tgg)[31550,31621]
caggatatggtgtcaatggtagcatgcgtgttttgggaacatgtggtgtt
ggttcgagtccagctatcctga

>JQ740801.1 Lactococcus Phage ASCC406, complete genome

1-1tRNA-Trp(cca)[31362,31434]
tgcgagcatagtatagtggtaatgctacagattccaaacctgtaaacgtg
ggttcgattcctgctgttcgtgt
1-2tRNA-Pro(tgg)[31488,31559]
caggatatggtgtcaatggtagcatgcgtgttttgggaacatgtggtgtt
ggttcgagtccagctatcctga

>JQ740802.1 Lactococcus Phage ASCC454, complete genome

1-1tRNA-Trp(cca)[31766,31838]
tgcgagcatagtatagtggtaatgctacagattccaaacctgtaaacgtg
ggttcgattcctgctgttcgtgt
1-2tRNA-Pro(tgg)[31892,31963]
caggatatggtgtcaatggtagcatgcgtgttttgggaacatgtggtgtt
ggttcgagtccagctatcctga

>JQ740803.1 Lactococcus Phage ASCC460, complete genome

1-1tRNA-Trp(cca)[31425,31497]
tgcgagcatagtatagtggtaatgctacagattccaaacctgtaaacgtg
ggttcgattcctgctgttcgtgt
1-2tRNA-Pro(tgg)[31551,31622]
caggatatggtgtcaatggtagcatgcgtgttttgggaacatgtggtgtt
ggttcgagtccagctatcctga

>JQ740806.1 Lactococcus Phage ASCC476, complete genome

1-1tRNA-Trp(cca)[31425,31497]
tgcgagcatagtatagtggtaatgctacagattccaaacctgtaaacgtg
ggttcgattcctgctgttcgtgt
1-2tRNA-Pro(tgg)[31551,31622]
caggatatggtgtcaatggtagcatgcgtgttttgggaacatgtggtgtt
ggttcgagtccagctatcctga

>JQ740809.1 Lactococcus Phage ASCC502, complete genome

1-1tRNA-Trp(cca)[31458,31530]

tgcgagcatagtatagtggtaatgctacagattccaaacctgtaaacgtg
ggttcgattcctgctgttcgtgt
1−2tRNA−Pro(tgg)[31584,31655]
caggatatggtgtcaatggtagcatgcgtgttttgggaacatgtggtgtt
ggttcgagtccagctatcctga
>JQ740810.1 Lactococcus Phage ASCC506, complete genome
1−1tRNA−Trp(cca)[31432,31504]
tgcgagcatagtatagtggtaatgctacagattccaaacctgtaaacgtg
ggttcgattcctgctgttcgtgt
1−2tRNA−Pro(tgg)[31558,31629]
caggatatggtgtcaatggtagcatgcgtgttttgggaacatgtggtgtt
ggttcgagtccagctatcctga
>JQ740811.1 Lactococcus Phage ASCC527, complete genome
1−1tRNA−Trp(cca)[31528,31600]
tgcgagcatagtatagtggtaatgctacagattccaaacctgtaaacgtg
ggttcgattcctgctgttcgtgt
1−2tRNA−Pro(tgg)[31654,31725]
caggatatggtgtcaatggtagcatgcgtgttttgggaacatgtggtgtt
ggttcgagtccagctatcctga
>JQ740814.1 Lactococcus Phage ASCC544, complete genome
1−1tRNA−Trp(cca)[31458,31530]
tgcgagcatagtatagtggtaatgctacagattccaaacctgtaaacgtg
ggttcgattcctgctgttcgtgt
1−2tRNA−Pro(tgg)[31584,31655]
caggatatggtgtcaatggtagcatgcgtgttttgggaacatgtggtgtt
ggttcgagtccagctatcctga
>KC522412.1 Lactococcus phage CaseusJM1, complete genome
1−1tRNA−Trp(cca)[30292,30364]
tgcgaacatagtatagtggtaatgctacagattccaaacctgtaaacgtg
ggttcgattcctgctgttcgtgt
>KC182544.1 Lactococcus phage 936, complete genome
1−1tRNA−Trp(cca)[26500,26570]
gcgaacatagtataatggtaatgctacagattccaaacctgtaaacgtgg
gttcgattcctactgttcgtg
1−2tRNA−Pro(tgg)[26640,26711]
caggatatagtgttaatggtagcatgcgtgttttgggaacatgtagtgtt
ggttcgagtccagctatcctga
>KC182545.1 Lactococcus phage fd13, complete genome
1−1tRNA−Trp(cca)[29914,29986]
tgcgagcatagtataatggtaatgctacagattccaaacctgtaaacgtg
ggttcgattcctactgttcgtgt
1−2tRNA−Pro(tgg)[30040,30111]
caggatatggtgttaatggtagcatgcgtgttttgggaacatgtggtgtt
ggttcgagtccagctatcctga

>KF676640.1 Lactococcus phage SK1833, complete genome
1-1tRNA-Trp(cca)[27505,27577]
tgcgagcatagtataatggtaatgctacagattccaaacctgtaaacgtg
ggttcgattcctactgttcgtgt
>KM091442.1 Lactococcus phage phi15, complete genome
1-1tRNA-Trp(cca)[31645,31717]
tgcgaacatagtatagtggtaatgctacagattccaaacctgtaaacgtg
ggttcgattcctactgttcgtgt
>KP793104.1 Lactococcus phage 936 group phage PhiB1127, complete genome
1-1tRNA-Pro(tgg)[28578,28649]
caggatatggtgtcaatggtagcatgcgtgttttggtaacatgtggtgtt
ggttcgagtccagctatcctga
>KP793105.1 Lactococcus phage 936 group phage Phi19.3, complete genome
1-1tRNA-Trp(cca)[28469,28541]
tgcgagcatagtatagtggtaatgctacagattccaaacctgtaaacgtg
ggttcgattcctactgttcgtgt
>KP793107.1 Lactococcus phage 936 group phage PhiD.18, complete genome
1-1tRNA-Trp(cca)[28243,28315]
tgcgagcatagtatagtggtaatgctacagattccaaacctgtaaacgtg
ggttcgattcctactgttcgtgt
1-2tRNA-Asp(gtc)[28321,28392]
taggatatagccaaattggtatatggtaggcagagtcgcaatctggtact
ggttcgattccagttgtcctag
>KP793109.1 Lactococcus phage 936 group phage PhiC0139, complete genome
1-1tRNA-Pro(tgg)[28825,28896]
caggatatggtgtcaatggtagcatgcgtgttttggtaacatgtggtgtt
ggttcgagtccagctatcctga
>KP793110.1 Lactococcus phage 936 group phage Phi43, complete genome
1-1tRNA-Asp(gtc)[28891,28962]
taggatatagccaaattggtatatggtaggcagagtcgcaatctggtact
ggttcgattccagttgtcctag
>KP793112.1 Lactococcus phage 936 group phage Phi129, complete genome
1-1tRNA-Trp(cca)[29072,29144]
tgcgagcatagtatagtggtaatgctacagattccaaacctgtaaacgtg
ggttcgattcctactgttcgtgt
1-2tRNA-Asp(gtc)[29150,29221]
taggatatagccaaattggtatatggtaggcagagtcgcaatctggtact
ggttcgattccagttgtcctag
>KP793113.1 Lactococcus phage 936 group phage PhiF.17, complete genome
1-1tRNA-Trp(cca)[29680,29752]
tgcgagcatagtatagtggtaatactacagattccaaacctgtaaacgtg
ggttcgattcctactgttcgtgt
1-2tRNA-Ser(tga)[29810,29896]
gggagtttgctcaagtctggtaaagaggttagtcttgaaaactaatgggc

gcttatgcgtgcaagggttcgaatcccttagcttcct
1-3tRNA-Pro(tgg)[29951,30022]
caggatatggtgttaatggtagcatgcgtgttttgggaacatgtggtgtt
ggttcaagtccagctatcctga
>KP793115.1 Lactococcus phage 936 group phage Phi114, complete genome
1-1tRNA-Trp(cca)[30189,30261]
tgcgaacatagtatagtggtaatgctacagattccaaacctgtaaacgtg
ggttcgattcctactgttcgtgt
>KP793116.1 Lactococcus phage 936 group phage Phi13.16, complete genome
1-1tRNA-Trp(cca)[30235,30307]
tgcgagcatagtatagtggtaatgctacagattccaaacctgtaaacgtg
ggttcgattcctactgttcgtgt
>KP793117.1 Lactococcus phage 936 group phage PhiG, complete genome
1-1tRNA-Trp(cca)[29954,30026]
tgcgagcatagtatagtggtaatactacagattccaaacctgtaaacgtg
ggttcgattcctactgttcgtgt
1-2tRNA-Ser(tga)[30084,30170]
gggagtttactcaagtctggtaaagaggttagtcttgaaaactaatgggc
gcttatgcgtgcaagggttcgaatcccttagcttcct
>KP793118.1 Lactococcus phage 936 group phage PhiF0139, complete genome
1-1tRNA-Trp(cca)[29688,29760]
tgcgagcatagtatagtggtaatactacagattccaaacctgtaaacgtg
ggttcgattcctactgttcgtgt
1-2tRNA-Ser(tga)[29818,29904]
ggaagtttgctcaagtctggtaaagaggttagtcttgaaaactaatgggc
gcttatgcgtgcaagggttcgaatcccttagcttcct
1-3tRNA-Pro(tgg)[29959,30030]
caggatatggtgttaatggtagcatgcgtgttttgggaacatgtggtgtt
ggttcaagtccagctatcctga
>KP793123.1 Lactococcus phage 936 group phage Phi4.2, complete genome
1-1tRNA-Trp(cca)[30519,30591]
tgcgagcatagtatagtggtaatgctacagattccaaacctgtaaacgtg
ggttcgattcctgctgttcgtgt
1-3tRNA-Pro(tgg)[30774,30845]
caggatatagtgttaatggtagcatgcgtgttttgggaacatgtagtgtt
ggttcgagtccagctatcctga
>KP793124.1 Lactococcus phage 936 group phage Phi44, complete genome
1-1tRNA-Trp(cca)[31036,31108]
tgcgaacatagtatagtggtaatgctacagattccaaacctgtaaacgtg
ggttcgattcctactgttcgtgt
>KP793129.1 Lactococcus phage 936 group phage PhiJF1, complete genome
1-1tRNA-Trp(cca)[31054,31126]
tgcgagcatagtatagtggtaatactacagattccaaacctgtaaacgtg
ggttcgattcctactgttcgtgt

1-2tRNA-Ser(tga)[31184,31270]
gggagtttactcaagtctggtaaagaggttagtcttgaaaactaatgggc
gcttatgcgtgcaagggttcgaatcccttagcttcct
1-3tRNA-Pro(tgg)[31325,31396]
caggatatggtgttaatggtagcatgcgtgttttgggaacatgtggtgtt
ggttcaagtccagctatcctga
>KP793131.1 Lactococcus phage 936 group phage PhiE1127, complete genome
1-1tRNA-Leu(gag)[31636,31707]
taggacatagccaaattggtatgtggtagtcagagtcgcaatctggtact
ggttcgattccagttgtcctag
>KP793132.1 Lactococcus phage 936 group phage PhiM1127, complete genome
1-1tRNA-Leu(gag)[32065,32136]
taggacatagccaaattggtatgtggtagtcagagtcgcaatctggtact
ggttcgattccagttgtcctag
>KP793111.1 Lactococcus phage 936 group phage Phi19.2, complete genome
1-1tRNA-Trp(cca)[29642,29714]
tgcgagcatagtatagtggtaatgctacagattccaaacctgtaaacgtg
ggttcgattcctactgttcgtgt
>KU578077.1 Mycobacterium phage Marie, complete genome
1-1tRNA-Asn(gtt)[3838,3913]
tgatctgtagctcaactggcagagcacccggctgttaaccgggtcgttgg
aggttcgagtcctcccagatcagcca
1-2tRNA-Trp(cca)[3954,4028]
aggcacgtagctcaattggtcagagcagcggtctccaaagccgccggctg
caggttcgagtcctgccgtgtctgc
1-3tRNA-Leu(cag)[4063,4137]
ggctcggtaggcaaacaggcaaagccgcctgtctcaggaacaggtgcgtg
agggttcgactccctcccgagctac
>KU984914.1 Mycobacterium phage Malinsilva, complete genome
1-1tRNA-Asn(gtt)[3832,3907]
tgatctgtagctcaatcggcagagcacccggctgttaaccgggacgttgg
aggttcgagtcctcccagatcagcca
1-2tRNA-Trp(cca)[3948,4022]
aggcacgtagctcaattggtcagagcagcggtctccaaagccgccggctg
caggttcgagtcctgccgtgtctgc
1-3tRNA-Leu(cag)[4057,4131]
ggctcggtaggcaaacaggcaaagccgcctgtctcaggaacaggtgcgtg
agggttcgactccctcccgagctac
>KU985091.1 Mycobacterium phage Hercules11, complete genome
1-1tRNA-Asn(gtt)[3847,3922]
tgatctgtagctcaactggcagagcacccggctgttaaccgggtcgttgg
aggttcgagtcctcccagatcagcca
1-2tRNA-Trp(cca)[3963,4037]
aggcacgtagctcaattggtcagagcagcggtctccaaagccgccggctg

caggttcgagtcctgccgtgtctgc
1–3tRNA–Leu(cag)[4072,4146]
ggctcggtaggcaaacaggcaaagccgcctgtctcaggaacaggtgcgtg
agggttcgactccctcccgagctac

>KX507362.1 Mycobacterium phage Aglet, complete genome
1–1tRNA–Asn(gtt)[3841,3916]
tgatctgtagctcaatcggcagagcacccggctgttaaccgggacgttgg
aggttcgagtcctcccagatcagcca
1–2tRNA–Trp(cca)[3957,4031]
aggcacgtagctcaattggtcagagcagcggtctccaaagccgccggctg
caggttcgagtcctgccgtgtctgc
1–3tRNA–Leu(cag)[4066,4140]
ggctcggtaggcaaacaggcaaagccgcctgtctcaggaacaggtgcgtg
agggttcgactccctcccgagctac

>JF704114.1 Mycobacterium phage Vix, complete genome
1–1tRNA–Asn(gtt)[3920,3995]
tgatctgtagctcaatcggcagagcacccggctgttaaccgggacgttgg
aggttcgagtcctcccagatcagcca
1–2tRNA–Trp(cca)[4036,4110]
aggcacgtagctcaattggtcagagcagcggtctccaaagccgccggctg
caggttcgagtcctgccgtgtctgc
1–3tRNA–Leu(cag)[4145,4219]
ggctcggtaggcaaacaggcaaagccgcctgtctcaggaacaggtgcgtg
ggggttcgactccctcccgagctac

>KP027202.1 Mycobacterium phage Taurus, complete genome
1–1tRNA–Asn(gtt)[3838,3913]
tgatctgtagctcaactggcagagcacccggctgttaaccgggtcgttgg
aggttcgagtcctcccagatcagcca
1–2tRNA–Trp(cca)[3954,4028]
aggcacgtagctcaattggtcagagcagcggtctccaaagccgccggctg
caggttcgagtcctgccgtgtctgc
1–3tRNA–Leu(cag)[4063,4137]
ggctcggtaggcaaacaggcaaagccgcctgtctcaggaacaggtgcgtg
agggttcgactccctcccgagctac

>KX397373.1 Erwinia phage vB_EamM_Stratton, complete genome
1–1tRNA–Trp(cca)[869,944]
aggggtatagttcaattggtagagcaccggtctccaaaaccgggtgttgg
gggttcgagtccctctgcccctgcca
1–2tRNA–Cys(gca)[1598,1673]
ggggcaatggctcgagcggttaggcgacgaattgcaaattcggatcaggt
cggttcaagtccggcttgcccctcca
1–3tRNA–Gly(ccc)[3358,3433]
gcgagtatagctcagctggtagagcttctggttcccaaccagacggtcgt
gagttcgaatctcattgctcgctcca

1-4tRNA-Tyr(gta)[3543,3628]
ggcgcggtactcaagtggtcaacgagagcagactgtaaatctgttgcctt
cgggcttcgtaggttcaaatccttccctcgccacca
1-5tRNA-Glu(ttc)[3635,3709]
gttcctgtcgtctagtggtaaggacacctggttttcaaccaggcaaccgg
agttcaattctccgcaggaacgcca
1-6tRNA-Asp(gtc)[3866,3943]
aggattgtagctcagttggtctagagtgccgccctgtcacggcggaggtc
gcgggttcgagccccgtcagtcctgcca
1-7tRNA-Ile(gat)[3953,4028]
tgcgtcttagctcagttggtagagcgcacccctgataagggtgaggtcac
tggttcaagtccagtagatgcaacca
1-8tRNA-Met(cat)[4035,4110]
ggttctgtagcttagctggtagagcacgccactcataatggcgcggtcgt
tggttcgaatccagccagaaccacca
1-9tRNA-His(gtg)[4119,4195]
gtgtatgtagttcagctggttagaatactggcttgtgacgtcagtggtcg
agagttcgagtctctccatacacccca
1-10tRNA-Ser(gct)[4201,4290]
ggagaattgtccgagaggcttaaagagctcccctgctaagggagtggtgg
cggaagctgcccgtgtgttcgaatcacacattctccgcca
1-11tRNA-Met(cat)[4488,4563]
gagcgatgctgttaagatggttaacacccgactcataatcggatattcgc
aggttcgattcctgtcatcgttcgcc
1-12tRNA-Asn(gtt)[4571,4647]
ggttccatagctcagttggttagagcggctgcctgttaagcagtaggtcc
ctggttcgagtccaggtggtaccgcca
>JF957057.1 Mycobacterium phage BPBiebs31, complete genome
1-1tRNA-Trp(cca)[2415,2490]
tggctcttagctcaatttggtagagcagcggtctccaaaaccgccggttg
caggttcgagtcctgcagagtcagcc
>JF957058.1 Mycobacterium virus Heldan, complete genome
1-1tRNA-Trp(cca)[3881,3954]
aggcgcgtagctcaattggtagagcagcggtctccaaagccgcgtgctgc
aggttcgagtcctgccgcgtctgc
>JF957059.1 Mycobacterium phage Optimus, complete genome
1-1tRNA-Gly(tcc)[94272,94344]
gcgcttgtggtcgagttggaaagactcctggcttccacccaggttatgca
ggttcgagtcctgtcgagcgctc
>JF937090.1 Mycobacterium phage Baka, complete genome
1-1tRNA-Gly(tcc)[95829,95901]
gcgcttgtggtcgagttggaaagactcctggcttccacccaggttatgca
ggttcgagtcctgtcgagcgctc
>JF937091.1 Mycobacterium phage Bask21, complete genome

1-1tRNA-Arg(cct)[61437,61520]
atggcccgtagctcagtcaggtagagcagccttgccccttgcgggcttgg
ttcgtccccggttcaaatccgggcgggccttacc
1-2tRNA-Gly(tcc)[61523,61595]
gcgcccgtggtcgaattggaaagactcctggcttccacccaggttatgca
ggttcgagtcctgtcgggcgctc
>JF937092.1 Mycobacterium phage DaVinci, complete genome
1-1tRNA-Asn(gtt)[5403,5475]
tgacgtgtagctcaatggcagagcacccggctgttaaccggacggttgaa
ggttcgagtccttccatgtcagc
1-2tRNA-Trp(cca)[5480,5553]
gcgctcctagctcaattggtagagcagcggtctccaaagccgcgagttcc
aggttcgagtcctggggagcgtgc
1-3tRNA-Gln(ctg)[5595,5667]
tccctgttcgtctaatcggtaagacaccgggctctggacccggcaatcga
ggttcgagtccttgatggggagc
>JF937094.1 Mycobacterium virus Hammer, complete genome
1-1tRNA-Asn(gtt)[5396,5468]
tggcgtgtagctcaatggcagagcccccggctgttaaccgggtggttgaa
ggttcgagtccttccatgccagc
1-2tRNA-Trp(cca)[5472,5545]
gcgttcctagctcaattggtagagcagcggtctccaaagccgcgagttcc
aggttcgaatcctggggagcgtgc
1-3tRNA-Gln(ctg)[5587,5659]
tccctgttcgtctaatcggtaagacaccgggctctggacccggtaatcga
ggttcgagtccttgatggggagc
>JF937101.1 Mycobacterium phage LittleE, complete genome
1-1tRNA-Tyr(gta)[95026,95098]
tggcccgtagctcaattggcagagcagccggcggtaattcggcgcgtctc
ggttcgagtccgggcgggctacc
>JN006062.1 Mycobacterium phage Rakim, complete genome
1-1tRNA-Arg(cct)[62326,62409]
aaggcccgtagctcagtcaggtagagcagccttgccccttgcgggcttgg
ttcgtccccggttcaaatccgggcgggccttacc
1-2tRNA-Gly(tcc)[62412,62484]
gcgcccgtggtcgaattggaaagactcctggcttccacccaggttataca
ggttcgagtcctgtcgggcgctc
>JF937096.1 Mycobacterium phage Henry, complete genome
1-1tRNA-Arg(cct)[62394,62477]
atggcccgtagctcagtcaggtagagcagccttgccccttgcgggcttgg
ttcgtccccggttcaaatccgggcgggccttgcc
1-2tRNA-Gly(tcc)[62480,62552]
gcgcccgtggtcgaattggaaagactcctggcttccacccaggttatgca
ggttcgagtcctgtcgggcgctc

>JN049605.1 Mycobacterium virus Ericb, complete genome
1-1tRNA-Asn(gtt)[5403,5475]
tgacgtgtagctcaatggcagagcacccggctgttaaccggacggttgaa
ggttcgagtccttccatgtcagc
1-2tRNA-Trp(cca)[5480,5553]
gcgctcctagctcaattggtagagcagcggtctccaaagccgcgagttcc
aggttcgagtcctggggagcgtgc
1-3tRNA-Gln(ctg)[5595,5667]
tccctgttcgtctaatcggtaagacaccgggctctggacccggcaatcga
ggttcgagtccttgatggggagc
>JN083852.1 Mycobacterium virus Benedict, complete genome
1-1tRNA-Trp(cca)[4137,4210]
gggtcagtagctcaactggtagagcagcggtctccaaagcggcgcgttgg
aggttcgagccctccctggcctgc
>JN083853.1 Mycobacterium phage Airmid, complete genome
1-1tRNA-Trp(cca)[4135,4208]
gggtcagtagctcaatcggtagagcagcggtctccaaagccgcgcgttgg
aggttcgagtcctccctggcctgc
>JN185608.1 Mycobacterium phage JAWS, complete genome
1-1tRNA-Trp(cca)[1003,1076]
gggtgtgtagctcaatcggtagagcagcggtctccaaagccgccggttgc
acgttcgagtcgtgccgcgcccgc
>JN391441.1 Mycobacterium phage Elph10, complete genome
1-1tRNA-Arg(cct)[61875,61958]
atggcccgtagctcagtcaggtagagcagccttgccccttgcgggcttgg
ttcgtccccggttcaaatccgggcgggccttgcc
1-2tRNA-Gly(tcc)[61961,62033]
gcgcccgtggtcgaattggaaagactcctggcttccacccaggttatgca
ggttcgagtcctgtcgggcgctc
>JN408459.1 Mycobacterium virus Cuco, complete genome
1-1tRNA-Trp(cca)[4309,4382]
gggtcagtagctcaattggtagagcagcggtctccaaagccgcgagttgg
aggttcgagtcctccctggcccgc
>JX262376.1 Streptomyces phage phiELB20, complete genome
1-1tRNA-Phe(gaa)[1083,1156]
gggccggtagctcagttggaagagcggcggagtgaaatcccgcaggtcgc
aggttcgagtcctgcctggcccac
>JN412590.1 Mycobacterium phage Eureka, complete genome
1-1tRNA-Arg(cct)[63002,63085]
atggcccgtagctcagtcaggtagagcagccttgccccttgcgggcttgg
ttcgtccccggttcaaatccgggcgggccttgcc
1-2tRNA-Gly(tcc)[63088,63160]
gcgcccgtggtcgaattggaaagactcctggcttccacccaggttatgca
ggttcgagtcctgtcgggcgctc

>KR997933.1 Mycobacterium phage Madruga, complete genome
1–1tRNA–Gln(ttg)[56447,56519]
tgcctcatggtgtaattggcagcacgagtggttttggtccacttagttca
ggttcgagtcctggtgaggcagc
>KU716094.1 Mycobacterium phage Eidsmoe, complete genome
1–1tRNA–Glu(ctc)[5936,6008]
ggtccgttggagtagcggcaactcatctgactctcactcagaagatcgcg
ggttcaaatcccgcacggactac
>JN699019.1 Mycobacterium virus Jeffabunny, complete genome
1–1tRNA–Asn(gtt)[5402,5474]
tgacgtgtagctcaatggcagagcacccggctgttaaccggacggttgaa
ggttcgagtccttccatgtcagc
1–2tRNA–Trp(cca)[5479,5552]
gcgctcctagctcaattggtagagcagcggtctccaaagccgcgagttcc
aggttcgagtcctggggagcgtgc
1–3tRNA–Gln(ctg)[5594,5666]
tccctgttcgtctaatcggtaagacaccgggctctggacccggcaatcga
ggttcgagtccttgatggggagc
>JN831654.1 Mycobacterium virus Saintus, complete genome
1–1tRNA–Lys(ctt)[4070,4143]
ggggcagtagctcagtcggtagagctacggactcttaatccgcaggtcgc
aggttcgatccctgcctgccccac
>JN600672.1 Mycobacterium phage Fezzik, complete genome
1–1tRNA–Leu(caa)[34724,34807]
gtggtccatgcgcggttatcgggcgtattagttcaagcctggtccgatga
ctaagcagtccggttcgattccggagtatcgcgc
1–2tRNA–Leu(caa)[61695,61769]
ggtcctgtaggcaaattggcaaagccgcgtcactcaaaatgacgtgtctg
tgggttcgagtcccaccgggactac
1–3tRNA–Thr(cgt)[61770,61844]
gctgccttagctcagatggctagagcgccgctctcgtaaagcggaggtcg
cgggttcgaggcccgcaggcagctc
1–4tRNA–Lys(ctt)[62131,62204]
gcctcgttagctcagttggtagagctgccgactcttaatcggtaggtcgc
aggttcaagtcctgcacggggtac
1–5tRNA–Tyr(gta)[62215,62298]
gcgtcggaggtacctgttggtttggtacacctgcctgtaaagcaggcgct
tcggcttcgggggttcgattccctcccggcgtac
1–6tRNA–Trp(cca)[62308,62381]
agctcggtagctcaattggtagagcagcggtctccaaagccgtgggttcc
ccgttcgagtcggggccgggttgc
1–7tRNA–Leu(tag)[62382,62456]
ggaactgtaggcaaactggaaaagccgcctgacttaggatcaggtgtttg
cgagttcgactctcgccagttctac

1-8tRNA-His(gtg)[62457,62526]
gctaagtagcttaatggtaaagcgccccgttgtggccggggtgattccgg
ttcgattccgggcttaagcc
1-9tRNA-Cys(gca)[63704,63777]
gccgctgtggccgagtggctaggcaccggcctgcaaagccggttagtccg
gttcgattccggagggcggctcca
1-10tRNA-Lys(ttt)c[64058,64132]
gcccctatagctcagttggtagagctatcgccttttaagcgacaggtcgc
aggttcgagtcctgctgggggcacc
>JQ740788.1 Lactococcus Phage ASCC273, complete genome
1-1tRNA-Trp(cca)[31765,31837]
tgcgagcatagtatagtggtaatgctacagattccaaacctgtaaacgtg
ggttcgattcctgctgttcgtgt
1-2tRNA-Pro(tgg)[31891,31962]
caggatatggtgtcaatggtagcatgcgtgttttgggaacatgtggtgtt
ggttcgagtccagctatcctga
>JX042578.1 Mycobacteriophage ElTiger69, complete genome
1-1tRNA-Trp(cca)[4135,4208]
gggtcagtagctcaatcggtagagcagcggtctccaaagccgcgcgttgg
aggttcgagtcctccctggcctgc
>JX163858.1 Caulobacter phage phiCbK, complete genome
1-1tRNA-Met(cat)c[103504,103588]
ggtacggtggctcgacaggtaaggcatcggcctcataagccgagggttcg
tcccaatcctggttcaaatccaggtcgtacctcca
1-2tRNA-Met(cat)c[103955,104033]
cgcggggtagaggagtccggttgtcctcgtctggctcataaccaggagat
cgtgggttcaaatcccactcccgctccca
1-4tRNA-Gly(tcc)[105106,105179]
tttgcgaatagcacagtggtagtgcaacagccgtcctagctgtcggtcgc
gggttcaatccccgcttcgatgaa
1-5tRNA-Cys(gca)[105759,105847]
gcttcggtggactgagaggttaggcgcgggattgcaaatctctgctggtg
ggaacacctatgagggttcgagtccctcccgaagctcca
1-7tRNA-Gly(tcc)c[155227,155304]
gcgcgggtagctcaatggacccagagcagccgtcttccacacggaaggtt
gggggttcgaatccctccccgtgcgcca
1-8tRNA-Ile(aat)c[155507,155581]
tccagtcaatgcacaaggtgtgcagcggcactgttaatgcctgtgagccc
ggttcgattccggggtctggagcca
1-9tRNA-Gln(ttg)c[155597,155670]
tctcgggtggtctaatggtaagacatctggttttggtccagaggattggg
gttcgattccttggccgagaacca
1-10tRNA-Gln(ctg)c[155678,155751]
tgagggttcgtctaacggtaggacggctgggtctggtccagctaatctag

gttcgagtcctagcccctcatcca
1-11tRNA-Val(cac)c[155757,155832]
tggcgtttagcatagtgggagtgcaggggtgtcacatacctcgatcgaac
tggttcgattccagtaacgccaacca
1-12tRNA-Ile(gat)c[155866,155940]
tgtggcatagctcaggggtagagcgcgcgcttgataagcgtgaggtcggg
ggttcgaagccctctgccactacca
1-13tRNA-Ile(gat)c[155948,156023]
gcaagtgtagctcagtcggtagagcagcggaccgataatccgcctgtccc
tggttcgagtccaggcatttgcacca
1-14tRNA-Val(gac)c[156026,156101]
gcccgatgagccagatgggaaggcgccgccgtgacatggcgggattgaga
aggttcgattccttcatcaggcacca
1-15tRNA-Phe(gaa)c[156107,156182]
ggactcgtagctcagcaggtagagcgccggcttgaaaccccggaggtcgg
aggttcgattcctcccgtttccacca
1-16tRNA-Ser(gga)c[156876,156963]
ggatgtatggctgagaggccgaaggcgcccgcttggaaagcgggtgaacc
gcaaggttccgtaggttcgaatcctactgcatccgcca
1-17tRNA-Ser(cga)c[156968,157055]
ggtaggttggctgagaggccgaaggcgcccgtttcgaaaacgggaggacc
gcaaggttccgtgggttcgaatcccacacctacctcca
1-18tRNA-Ala(tgc)c[157060,157130]
ggggtatgcgcagggcgcaggcatcctttgcaaggaagtcggggtgggtt
cgagtcccacatactccacca
1-19tRNA-Leu(gag)c[157214,157302]
tgcgaagtggcggaactttggtagacgcaccggcatgagaggccggcgcc
cttcggggcgtgagggttcgagtcccttcttcgcgacca
1-20tRNA-Leu(caa)c[157308,157392]
gccggtctggcggaaatggcagacgcagtggcttcaagtcccaccgcctt
cgggcgtcccggttcgaggccggggaccggcacca
1-21tRNA-Leu(cag)c[157400,157483]
gcgaaagtggcggaacggcagacgcactggtctcaggtaccagcgacttc
ggtcatgagggttcgaatccctcctttcgtacca
1-22tRNA-Lys(ttt)c[157490,157580]
gcacgagtgacccgagcggcgaagggcgtcgacttttaatcgactaggcg
tccgcgcctcaccgagggttcgagtccctcctcgtgctcca
1-23tRNA-Lys(ctt)c[157587,157673]
ggatgtgtcacctcagtggcgagaggaccgggctcttaatccggcagcga
aagctcatcgtgggttcgagtcccaccacatcctcca
1-24tRNA-Pro(ggg)c[157864,157938]
cgggatgtagctcagcggtagagcgcgcggctgggggccgcgaggccgca
ggttcgagacctgccaccccgacca
1-25tRNA-Pro(cgg)c[157946,158021]

cggggtgtagctcagtcggtagagcgctcggttcgggaccgagaggccgt
gggttcgagtcccgccacccagacca
1−26tRNA−Asp(gtc)c[159461,159535]
ggtgcgttcgtatatcggtcaatactccggattgtcgatccggcaagagg
ggttcgattcccctacgcaccgcca
1−27tRNA−Glu(ttc)c[159558,159635]
ggcccggtcgtctagcggtttgcaggattcgtgactttcaatcacgagag
cggagttcgagtctccgtcgggcctcca
1−28tRNA−Gly(tcc)c[159792,159865]
gcggatatagctcaaagggagagctactgccttccaagcagaagatgcgg
gttcgagccccgctatccgctcca
1−29tRNA−Gly(gcc)c[159872,159946]
gcgtccatcgtatatgggttattgcttcgcgttgccaacgcgacgaaccg
ggttcgagtcccggtggacgctcca
1−30tRNA−Trp(cca)c[159952,160027]
gtcggtctagctcatggggtagagcggcggtctccaaaaccgcgcgtggc
aggttcgagtcctgcgaccggcgcca
>JX307704.1 Mycobacterium virus Goose, complete genome
1−1tRNA−Trp(cca)[3783,3858]
aggcacgtagctcaattggaagagcagcggtctccaaagccgcccgttgc
aggttcgagtcctgccgtgtctgcca
>JX411619.1 Mycobacterium virus Rebeuca, complete genome
1−1tRNA−Trp(cca)[3968,4043]
aggcacgtagctcaattggaagagcagcggtctccaaagccgccggctgc
aggttcgagccctgccgtgtctgcca
>JX889246.1 Streptomyces phage phiCAM, complete genome
1−1tRNA−Arg(cct)c[45921,46003]
cacctgctgggacgggttcaagaccacctcctgagtggtcagcgtgagcg
gcacacactccggttcgagtccggggcacgcac
>KC333879.1 Enterobacteria phage phiJLA23, complete genome
1−1tRNA−Arg(cct)c[22052,22127]
gttctgctggcgtaacaggataacgcagagacctcctaagtctcagttac
tggttcgagtccagtgcggaacacca
>KT591491.1 Mycobacterium phage Bricole, complete genome
1−1tRNA−Arg(ccg)[32341,32436]
ctccacgtggcgaatggcaatgtggcagaggggattccggttcccgaggg
gcgggggatctcagacttccgcctgcgttcgactccgtcgtggtgg
1−2tRNA−Trp(cca)[54546,54617]
atgggcgtagctgactgggaagcaacggtctccaaagccgtgatatgcag
gttcgaatcctgccgcccgtgc
1−3tRNA−Asn(gtt)[55466,55537]
tggcctgtcggttaacggcaaaccagcgccctgttaaggcgtgactcctg
gttcgaatccaggtaggccagc
1−4tRNA−Gln(ttg)[56294,56365]

cgggacatagctcaaatggcagagtggggcaattggtgccctggattcag
gttcgactcctggtggcccgac
1-5tRNA-Tyr(gta)[56750,56832]
gcggtctctcccctattggcgaagggcccctggctgtaacccagggcttt
cgagtgtggtggttcgaatccatcaggccgcac
1-6tRNA-Gln(ctg)[57001,57073]
tgctctctcgtctaactggcaggacaccagaatctgactctggcagttga
ggttcgaatccttggggagcaac
1-7tRNA-Pro(tgg)[57077,57150]
ctgggtgaggtgtaagtggttgcatggccggtttggatccgggcggttcg
aggttcgagtcctcgcacccagac
1-8tRNA-Ser(gct)[57446,57527]
gagtggaaccatctaggtggatggccgcgattgctaatcgtggggcagcg
taacgctgtgtggatcgtgcccactcctctcc
1-9tRNA-Phe(gaa)[57637,57712]
cgggttgtagctcagattggtgagagcgctcggttgaaacccgagaggcc
cctggttcgaatccaggcatcccggc
1-10tRNA-Met(cat)[57800,57871]
tgcgcggtagtgaaacgggatcacaaaggtctcataagcctttattacgg
gttcgaatcccgtctgcgcaac
1-11tRNA-Arg(acg)[58151,58225]
cccgccgcagcacgacggacgtgcacctcgctacgaacgagaaggctgct
ggttcgaatccagtcggtgggtcca
1-12tRNA-His(gtg)[58228,58300]
ccccttgtagctcaatggtgagagcgccgttttgtgagaccggtgacctg
cgttcgattcgcagcagggggac
1-13tRNA-Cys(gca)[58306,58378]
ggcgggatggctgagttagataggcggcgggctgcaaccccgttcacgcc
ggggcagatccggctctcgtctc
1-14tRNA-Leu(cag)[58640,58714]
ggctcgctaggcaaattggaaaagccgcctgattcagaatcaggtgtttc
cgagttcgactctcgggcgagctac
1-15tRNA-Lys(ctt)[59734,59807]
gggacgttggtgaaacggctatcaccgcacgctcttaacgtgtcagttct
gggttcgaatcccaggcgtcccac
1-16tRNA-Gly(tcc)[59930,60005]
ggggcattcatctagtcaggctcaggatgcgacgcttccaacgtcgtcac
gagggttcgaatccttcatgcctcac
1-17tRNA-Val(tac)[60010,60082]
tccggattagctcagtggaagagcggcaggtttacaccctgcgcgtgggg
agttcgattctctcatccggaac
1-18tRNA-Thr(agt)[60086,60158]
gccggtatagctcagtggctagagcacctgtctagtaaacaggagaccag
ggttcgattccctgttccggctc

1−19tRNA−Asp(gtc)[60273,60348]
cccgccatcatctagcggcccaggatgccgccctgtcgaggcggtcacac
gggttcaaatcccgttggcgggacca
1−20tRNA−Glu(ctc)[60350,60421]
ggttcattggtgtaacggcagcatgtcaccctctcaaggtgaaggcaggg
gttcaactcccctatgaactgc
>KC661276.1 Mycobacterium phage Winky, complete genome
1−1tRNA−Thr(cgt)[63338,63411]
gccaccttagctcagttggtagagcagccccttcgtaacgggcaggtcag
cggttcgattccgctaggtggctc
1−2tRNA−Pro(tgg)[63639,63713]
ctggctgtagctcaacttggtagagcgctggctttgggtgccaggggttg
caggttcaaatcctgctagccagac
1−3tRNA−Trp(cca)[63834,63907]
agctcggtagctcaattggtagagcagcggtctccaaagccgccggttcc
ccgttcgagtcggggtcgggttgc
1−4tRNA−Tyr(gta)[64018,64100]
gtggtggttgggcttgttggttggcccacctgactgtaaatcaggcgttt
cggcaccgggggttcgattccctcccaccacac
1−5tRNA−Leu(tag)[64515,64589]
ggccctctagcccaattggcagaggcacaggttttaggtacctgtcagtg
cgagttcgagtctcgcgggggccac
1−6tRNA−His(gtg)[64590,64659]
gcttagtagcctagtggtaaggcagccggttgtgatccggtgaacctgag
ttcgattctcagctaaagcc
1−7tRNA−Gln(ctg)[64664,64738]
tgaccggtagcacaactggtagttgcgccgcgctctggacgcggaggttg
ttggttcgatcccagctcggtcagc
1−8tRNA−Gly(tcc)[64854,64928]
gtgctagtaacccatgttggctgggtgcctgacttccactcaggatttcg
cgggttcgattcctgtctagcacac
1−9tRNA−Lys(ctt)[64971,65048]
tgcctcgttagctcagttggtagagctgccgactcttaatcggtaggtca
caggttcaagtcctgtacggggtaccca
1−10tRNA−Cys(gca)[66238,66311]
gccgtcatggctgagtggttaggcgtcggactgcaaatccggcttatccc
agttcgattctgggtggcggctcc
1−11tRNA−Asn(gtt)[66673,66746]
tcctccgtagctcaattggcagagcgcgcgactgttaatcgcgtggttgg
tggttcgagtccacccgttggagc
1−12tRNA−Lys(ttt)c[66940,67014]
gcccctatagctcagttggtagagcaggagacttttaatcttcgggtcct
aggttcgatccctagtgggggcacc
>KC691254.1 Mycobacterium phage Breezona, complete genome

1−1tRNA−Thr(cgt)[63338,63411]
gccaccttagctcagttggtagagcagccccttcgtaacgggcaggtcag
cggttcgattccgctaggtggctc
1−2tRNA−Pro(tgg)[63639,63713]
ctggctgtagctcaacttggtagagcgctggctttgggtgccaggggttg
caggttcaaatcctgctagccagac
1−3tRNA−Trp(cca)[63834,63907]
agctcggtagctcaattggtagagcagcggtctccaaagccgccggttcc
ccgttcgagtcggggtcgggttgc
1−4tRNA−Tyr(gta)[64018,64100]
gtggtggttgggcttgttggttggcccacctgactgtaaatcaggcgttt
cggcaccgggggttcgattccctcccaccacac
1−5tRNA−Leu(tag)[64514,64588]
ggccctctagcccaattggcagaggcacaggttttaggtacctgtcagtg
cgagttcgagtctcgcgggggccac
1−6tRNA−His(gtg)[64589,64658]
gcttagtagcctagtggtaaggcagccggttgtgatccggtgaacctgag
ttcgattctcagctaaagcc
1−7tRNA−Gln(ctg)[64663,64737]
tgaccggtagcacaactggtagttgcgccgcgctctggacgcggaggttg
ttggttcgatcccagctcggtcagc
1−8tRNA−Gly(tcc)[64853,64927]
gtgctagtaacccatgttggctgggtgcctgacttccactcaggatttcg
cgggttcgattcctgtctagcacac
1−9tRNA−Lys(ctt)[64970,65047]
tgcctcgttagctcagttggtagagctgccgactcttaatcggtaggtca
caggttcaagtcctgtacggggtaccca
1−10tRNA−Cys(gca)[66237,66310]
gccgtcatggctgagtggttaggcgtcggactgcaaatccggcttatccc
agttcgattctgggtggcggctcc
1−11tRNA−Asn(gtt)[66672,66745]
tcctccgtagctcaattggcagagcgcgcgactgttaatcgcgtggttgg
tggttcgagtccacccgttggagc
1−12tRNA−Lys(ttt)c[66939,67013]
gcccctatagctcagttggtagagcaggagacttttaatcttcgggtcct
aggttcgatccctagtgggggcacc
>KT373978.1 Mycobacterium phage Ukulele, complete genome
1−1tRNA−Arg(cct)[62194,62277]
atggcccgtagctcagtcaggtagagcagccttgccccttgcgggcttgg
ttcgtccccggttcgaatccgggcgggccttgcc
1−2tRNA−Gly(tcc)[62280,62352]
gcgcccgtggtcgaattggaaagactcctggcttccacccaggttatgca
ggttcgagtcctgtcgggcgctc
>KF017927.1 Mycobacterium phage Odin, complete genome

1−1tRNA−Asn(gtt)[4130,4205]
tgacgtgtagctcaattggcagagcacccggctgttaaccggacggttga
aggttcgagtccttccacgtcagcca
1−2tRNA−Lys(ctt)[4242,4315]
ggggcagtagctcagtcggtagagctgcggactcttaatccgtaggtcgc
aggttcgatccctgcctgccccac
>KF493883.1 Mycobacterium phage Mosby, complete genome
1−1tRNA−Arg(cct)[62199,62282]
atggcccgtagctcagtcaggtagagcagccttgccccttgcgggcttgg
ttcgtccccggttcaaatccgggcgggccttgcc
1−2tRNA−Gly(tcc)[62285,62357]
gcgcccgtggtcgaattggaaagactcctggcttccacccaggttatgca
ggttcgagtcctgtcgggcgctc
>KX375815.1 Mycobacterium phage ToneTone, complete genome
1−1tRNA−Asn(gtt)[5312,5384]
tgacgtgtagctcaatggcagagcacccggctgttaaccggacggttgaa
ggttcgagtccttccatgtcagc
1−2tRNA−Trp(cca)[5389,5462]
gcgctcctagctcaattggtagagcagcggtctccaaagccgcgagttcc
aggttcgagtcctggggagcgtgc
1−3tRNA−Gln(ctg)[5504,5576]
tccctgttcgtctaatcggtaagacaccgggctctggacccggcaatcga
ggttcgagtccttgatggggagc
>KF771236.1 Escherichia phage bV_EcoS_AHP24, complete genome
1−1tRNA−Arg(cct)[46138,46213]
gttctgctggcgtaacaggataacgcagagacctcctaagtctcagttgc
tggttcgagtccagcgcagaacgcca
>KJ094032.2 Enterococcus phage VD13, complete genome
1−1tRNA−Trp(cca)[30832,30905]
tgagttgttagtgtaatggttagcacaacagtctccaaaactgttagaga
gggttcgattccttcactgcttgt
>KJ567045.1 Mycobacterium phage Emerson, complete genome
1−1tRNA−Trp(cca)[1009,1082]
gggtgtgtagctcaatcggtagagcagcggtctccaaagccgccggttgc
acgttcgagtcgtgccgcgcccgc
>KT361920.1 Mycobacterium phage Slarp, complete genome
1−1tRNA−Arg(gcg)[14207,14300]
tcggcggcgctgcgcaggagatgggcagcagcgttcgcggcaccctgtcg
aacctcaaggcggcctattcgcggttcggtgccgagctgtcggg
1−2tRNA−Lys(ttt)c[30855,30930]
gccccgctagctcaatcggaagagcagccggcttttaaccggcgggtacg
gagatcgaaactccggcggggcacca
>KM233455.1 Mycobacterium phage Farber, complete genome
1−1tRNA−Asn(gtt)[3833,3908]

tgatctgtagctcaatcggcagagcacccggctgttaaccgggacgttgg
aggttcgagtcctcccagatcagcca
1−2tRNA−Trp(cca)[3949,4023]
aggcacgtagctcaattggtcagagcagcggtctccaaagccgccggctg
caggttcgagtcctgccgtgtctgc
1−3tRNA−Leu(cag)[4058,4132]
ggctcggtaggcaaacaggcaaagccgcctgtctcaggaacaggtgcgtg
agggttcgactccctcccgagctac
>KX458237.1 Mycobacterium phage Penny1, complete genome
1−1tRNA−Asn(gtt)[3838,3913]
tgatctgtagctcaactggcagagcacccggctgttaaccgggtcgttgg
aggttcgagtcctcccagatcagcca
1−2tRNA−Trp(cca)[3954,4028]
aggcacgtagctcaattggtcagagcagcggtctccaaagccgccggctg
caggttcgagtcctgccgtgtctgc
1−3tRNA−Leu(cag)[4063,4137]
ggctcggtaggcaaataggcaaagccgcctgtctcaggaacaggtgcgtg
agggttcgactccctcccgagctac
>KM592966.1 Mycobacterium phage QuinnKiro, complete genome
1−1tRNA−Asn(gtt)[3984,4059]
tgatctgtagctcaatcggcagagcacccggctgttaaccgggacgttgg
aggttcgagtcctcccagatcagcca
1−2tRNA−Trp(cca)[4100,4173]
aggcgcgtagctcaattggtagagcagcggtctccaaagccgctggctgc
aggttcgagtcctgccgcgtctgc
>KM979355.1 Enterobacteria phage DT571/2, complete genome
1−1tRNA−Gly(tcc)c[24894,24968]
gcgtgattagttcagcggctagaataactggcttccaaccagtagacacg
agttcgactctcgtatcccgcacca
1−2tRNA−Gln(ttg)c[24980,25057]
tggggattagcttagcttggcctaaagctacggcatttgaagtcgagatc
attggttcaaatccaatatcccctgcca
1−3tRNA−Gln(ctg)c[25064,25139]
agaacctgagccaagtcggttaaggctggggtctctgaaaccctgatcag
tggttcaaatccactaccttctgcca
1−4tRNA−Arg(acg)c[25557,25631]
gcgtccttatttcaatggaaagaatgtaaagccacgaactttacgattgg
ggttcgattccctgaggatgcacca
1−5tRNA−His(gtg)c[25638,25714]
gtggctatatcataattggttaatgatcctgattgtgaatcaggcctatg
tggattcgaattccactagccacccca
1−6tRNA−Leu(tag)c[26249,26325]
gcgtgattgatggaattggcatacataccgtccctagaagtcgggttttg
agggttcgaatcccttgtcacgcacca

1−7tRNA−Ala(tgc)c[26331,26404]
ggggatatagtttaatggtagaacatctgctttgcacgcagaagacgagg
gttcaactccctctttctccacca
1−8tRNA−Met(cat)c[28310,28384]
gggtcggtagcttaaagttaaagcggtggcctcataagctaacgagtagg
agttagattctcctccgacccacca
1−9tRNA−Ser(gct)c[28391,28479]
ggaagaatagcataacggtattgcagcagattgctaatctgtcggtttga
aatatagccttgtgggttcgattcccgcttcttccgcca
1−10tRNA−Arg(tct)c[30213,30287]
cggggtgtagtctaagggagaggcaggagtcttctaaattcctttatgca
ggttcgaatcctgtcacctcggcca
1−11tRNA−Ser(act)c[30788,30861]
tgccgatctagtataaacggtattatcaagccgtaactggtggaagatgt
aggttcgaatcctacgatcggtaa
>KP017310.1 Mycobacterium phage Phoxy, complete genome
1−1tRNA−Asn(gtt)[3922,3997]
tgatctgtagctcaatcggcagagcacccggctgttaaccgggacgttgg
aggttcgagtcctcccagatcagcca
1−2tRNA−Trp(cca)[4038,4112]
aggcacgtagctcaattggtcagagcagcggtctccaaagccgccggctg
caggttcgagtcctgccgtgtctgc
1−3tRNA−Leu(cag)[4147,4221]
ggctcggtaggcaaacaggcaaagccgcctgtctcaggaacaggtgcgtg
agggttcgactccctcccgagctac
>KP017311.1 Mycobacterium phage Spike509, complete genome
1−1tRNA−Asn(gtt)[3922,3997]
tgatctgtagctcaatcggcagagcacccggctgttaaccgggacgttgg
aggttcgagtcctcccagatcagcca
1−2tRNA−Trp(cca)[4038,4112]
aggcacgtagctcaattggtcagagcagcggtctccaaagccgccggctg
caggttcgagtcctgccgtgtctgc
1−3tRNA−Leu(cag)[4147,4221]
ggctcggtaggcaaacaggcaaagccgcctgtctcaggaacaggtgcgtg
agggttcgactccctcccgagctac
>KP027195.1 Mycobacterium phage Cosmo, complete genome
1−1tRNA−Trp(cca)[58394,58465]
gcgctgatagtgtagtggtaacacggcaagctccaaccttgctatctggg
gttcaagtcctcatcagcgtgc
1−3tRNA−Pro(tgg)[59446,59517]
cagggtgtggtgtagtggtcgcatggctgccttggaagcaggcggacgca
gttcaattctgcgcaccctgac
1−4tRNA−Phe(gaa)[59841,59914]
cggtcgttagtgaagatggagatcacgcgggcctgaaaaccccgagacgc

aggttcgattcctgcacgaccggc
1–5tRNA–Met(cat)[59923,59997]
ggcagagtagagaaacggtatctcgcaaggctcataacctttgaaaaagg
cgggttcgactccccctctgctcc
1–6tRNA–Tyr(gta)[60111,60193]
gcgtcagacgcacattgggtagtgcagcggtctgtaaaaccgtcgccttc
gggcattgggggttcaagtccctcctgacgcac
1–7tRNA–Ala(tgc)[60266,60337]
gggggtatcgtataatggtattacgcctgccttgcaagcaggtcattggg
gttcaattccccatacctccac
1–8tRNA–Val(tac)[60392,60463]
gcccgcttagctcaatgggagagccaccggcctacacccggtagacgaga
gttcaattctttcagcgggtac
1–9tRNA–Leu(tag)[60534,60608]
ggctcgttatgcaaaccggcaaagcagcctgccttagaagcaggtgtttc
agggttcgactccctggcgagctac
1–10tRNA–Leu(caa)[60666,60739]
ggtcgtgtaggcaaatggcaaagccgccaagctcaaaccttggtgtttgt
gggttcaactcccaccgcgactac
1–11tRNA–Gly(tcc)[60835,60908]
gagggtgaagtgttaacggtatgcacggctgtcttccaaacagcaagagc
gagttcgattctcgtcaccccac
1–12tRNA–Gly(gcc)[60984,61055]
gtggacgtgatgtaatggtagcctccccgcttgccaagcagggtgtgcgg
gttcaattcccgtcgtccgcac
1–13tRNA–Gln(ctg)[61244,61316]
tgccctatggtgtaattgacagcacgggtggctctgaaccatctagtcga
ggtttgaatccttgtggggcagc
1–14tRNA–Asp(gtc)[61534,61606]
cccgtcatagtgtagtggttaacacgccagcttgtcacgctggtagcgag
ggttcaattccctctggcggggc
1–15tRNA–Thr(agt)[61613,61687]
ggctttgtagctcaacggttttagagcagctgtctagtaaacagccgacc
agggttcgactccctgcttagcctc
1–16tRNA–Lys(ctt)[61860,61934]
gcctcgctagtttaatggccaaaaccgcagactcttaatctgtcagactc
tgggttcgaatcccaggcggggcac
1–17tRNA–Cys(gca)[61941,62016]
gccggtgtgacagaacgatttatgtggcagtctgcaaaactgacctaagc
gggtttgactcccgccaccggttcca
1–18tRNA–Ile(gat)[62019,62091]
tggcccgtagttcaacggtaagaacggatcgctgataacgatcagacaag
agttcaactctcttcgggccaac
1–19tRNA–Arg(acg)[62198,62271]

gcccgtgtagctcagtggatagagccgtggcctacgaagccatgggccgg
gggttcgaatccctccacgggtac
1–20tRNA–Ser(gct)[62297,62379]
ggagagtgatgcaacagggtggttgcactcgcctgctaagcgatgtgtct
gcaaggatcacgttcaactcgtgctctctccgc
1–21tRNA–His(gtg)[62381,62454]
gtgtctgtagctcaatggtagagcctcccgctgtgaccgggacgacgacg
ggtcagaaccgtccagacacccca
1–22tRNA–Glu(ttc)[63073,63145]
ggttccgtggtgtaacggttagcacctgggtctttcaagcccaaggtcag
ggttcaactccctgcggaactgc
1–23tRNA–Glu(ctc)[63150,63221]
ggcccactggtgtaatgggaacacgccaccccctcaaggtggagtcaggg
gttcaattcccctgtgggctac
1–24tRNA–Asn(gtt)[63227,63298]
tccggtatcgttcaaaggcaggacaccaagctgttaacttggctatgttg
gttcgatcccagctaccggagc
>KR422353.1 Escherichia phage APCEc03, complete genome
1–1tRNA–Ser(gct)[614,702]
ggaagaatagcataacggtattgcagcagattgctaatctgtcggtttga
aatatagccttgtgggttcgattcccacttcttccgcca
1–2tRNA–Ser(tga)[1580,1669]
ggaaggtagggcgtagtggtacgcaactagtcttgaaaactagcccgctg
tagtgatacggtgatggttcgactccattaccttcctcca
1–3tRNA–His(gtg)[2260,2336]
gtggctatatcataattggttaatgatcctgattgtgaatcaggcctatg
tggattcgaattccactagccacccca
1–4tRNA–Gln(ctg)[2526,2601]
agaaggttagccaagtcggttaaggctggggtctctgaaatcctgatcag
tggttcaaatccactaccttctgcca
1–5tRNA–Gln(ttg)[2607,2684]
ttgggattagcttagcttggcctaaagcttcggcctttgaagtcgagatc
attggttcaaatccaatatcccctgcca
1–6tRNA–Thr(tgt)[3201,3287]
gctgggttggcagaatggttgaatgcacctgatttgtaatcaggaggagt
aatcccgttgcaggttcgaatcctgtgcccagcacca
1–7tRNA–Ile(gat)[4039,4115]
gcttcggtagcttagcgatctaaagcactcggctgataaccgagagatcg
ggggtttaaatccctcccggagtacca
1–8tRNA–Met(cat)[4212,4287]
agttagttggcagagtggttatgcacctccttcatacggagcgactacag
tggttcaaatccactactaactacca
1–9tRNA–Arg(tct)[102659,102733]
cggggtgtagtctaagggagaggcaggagtcttctaaattcctttatgca

ggttcgaatcctgtcacctcggcca
>JX015524.1 Mycobacterium virus Astro, complete genome
1-1tRNA-Trp(cca)[4055,4128]
gggtcagtagctcaattggtagagcagcggtctccaaagccgctcgttgg
gggttcgagtccctcctggcctgc
>KT020852.1 Mycobacterium phage NoSleep, complete genome
1-1tRNA-Arg(cct)[62056,62139]
atggcccgtagctcagtcaggtagagcagccttgccccttgcgggcttgg
ttcgtccccggttcaaatccgggcgggccttgcc
1-2tRNA-Gly(tcc)[62142,62214]
gcgcccgtggtcgaattggaaagactcctggcttccacccaggttatgca
ggttcgagtcctgtcgggcgctc
>KT186229.1 Streptomyces phage Verse, complete genome
1-1tRNA-Arg(cct)c[45153,45235]
cacctgctgggacgggttcaagaccacctcctgagtggtcagcgtgagcg
gcacacaccccggttcgagtccggggcacgcac
>KT070867.1 Bacillus phage PBC2, complete genome
1-1tRNA-Arg(tct)c[148845,148918]
tgcccttttggtgaaatggatatcatgactggtttctacccagtagttag
ggtttcgaatactctagagggcgt
1-2tRNA-Arg(acg)c[148922,148995]
taccgatatagtgaaatggatatcacgacagactacgaatctgttagtcc
aagttcgaatcttggtatcggtgt
1-3tRNA-Gly(tcc)c[149000,149074]
gtggtattagtgtagtctggtctagcacccctgccttccaagcaggaaac
atcggttcgaatccgatataccaca
1-4tRNA-Ser(gct)c[149083,149171]
ggagggttactcaagcggtgaagaggactgtttgctaaacagttagggcg
cagaaatgtgccacaggagttcgaatctcctaccctcct
1-5tRNA-Ser(tga)c[149178,149264]
tggatggttactctaattggtaagagacctgccttgaaagcagacgtagg
gtaaaaccgatgggggttcaagtccctcaccatccgt
1-6tRNA-Thr(tgt)c[149365,149435]
gcccgattagtttaatggaaaaacgaggaacttgtaattcttagttatca
gttcgattctggtattgggct
1-7tRNA-Ala(tgc)c[149440,149514]
gggggattagtttaatcggtaaaacagtggctttgcacgtcacagaaaag
agttcgaatctcttattctccacca
1-8tRNA-Pro(tgg)c[149694,149765]
gtggatatagggcaatggtagcctactccttttggaagggagatgttgca
agttcgagtcttgctatccgca
1-9tRNA-Lys(ttt)c[149771,149843]
gggttattaactcaatggtagagtaatcggcttttaaccgatatgctaga
ggttcgagtcctctatgacccac

1-10tRNA-Ile(gat)c[149857,149931]
tgactagtagctcaactggttagagcatccgattgataatcggaaggttg
ttggttcgagtccaaccttaattgt
1-11tRNA-Leu(tag)c[149979,150053]
tggcggagtaatccaacggcagagatagtggttttagaagccatccagtg
agagttcgaatctctccttcgctat
1-12tRNA-Tyr(gta)c[150063,150142]
gggcgtgcattcattggcgagataagctgactgtaaatcagtggcgaaag
ctgtgttagttcgaatctaaccatgcccac
1-13tRNA-Glu(ttc)c[150296,150370]
gctccattggacaaatcggtttaagtcgctaccctttcaaggtagagatt
gccagttcgaatctggtatggagta
1-14tRNA-Cys(gca)c[150383,150458]
ggtcgagttcctagatggtctggggtctagtctgcaaaactaggttttga
gggttcgaatcccttcttggcttcca
1-15tRNA-Leu(taa)c[150465,150549]
tgcgtgggtggtggaattggcagacacatgcggtttaagcccgcatgact
tcggtcgtgagagttcgagtctctccctacgtatc
1-16tRNA-Gln(ttg)c[150681,150759]
ttgcggtgtggacaaactggtaaagtcgcctgcctttgaagcaggagatg
ttggaggttcgaagccttccaccgcagtc
1-17tRNA-Gly(gcc)c[150864,150934]
gtgggattagtttagaggtagaatttctgattgccattcagaaggaaagg
gttcgattcccttatctcaca
1-18tRNA-Asp(gtc)c[151628,151702]
ggtttattagtatatgggttaacacgcttgcctgtcacgcaagagaaatg
agttcaagtctcgtatagaccgcca
1-19tRNA-Asn(gtt)c[151707,151781]
tgcacctatagctctaatggtagagcagagagctgttaactctttggttg
gtggttcgaatccatctaggtgcgt
>KT345706.1 Vibrio phage vB_VorS-PVo5, complete genome
1-1tRNA-Val(tac)c[244,319]
actcccttagctcagtgtgtagagcacctcccttacaaggagggggtcgt
tggttcgagtccaacagggagtacca
1-2tRNA-Gln(ttg)c[537,613]
aggggattagcttagctggccgaaagcaacggactttgaatccgtgacca
caggttcgaatcctgtatcccccgcca
1-3tRNA-Thr(tgt)c[823,898]
gcttctttagctcagttgggagagcagctcacttgtaatgagcaggtcgt
ccgttcgaatcggacaaggagcacca
1-4tRNA-Gly(tcc)c[971,1057]
gcgtactgaagactgagcggtcggtcactagccttccaagctagcgccct
taatcgggatagggttcgactccctctgtacgctcca
1-5tRNA-Ser(tga)c[1065,1158]

ggaagggcaatccgatggttggcgacggaactggtcttgaaaaccagcga
gtgctaatagcgccttgagagttcgatcctctctccttccgcca
1−6tRNA−Leu(tag)c[1165,1250]
gcaggagtggcgtaattggtagccgcgtgtgccttagaagca.atgtcct
agtgacgtgagggttcaagtccctcctcctgtacca
1−7tRNA−Pro(tgg)c[1361,1439]
cggggtgtaatgtcagtctggtcagacggctcgctttggatgcgagaggt
cgtaggttcgaatcctacctccccgacca
1−8tRNA−Asp(gtc)c[1446,1523]
gcggctgtgtgcgagatggtttaggcaacctccctgtcacggaggagtac
gtgggttcaagtcccatcggtcgcgcca
1−9tRNA−Asn(gtt)c[1529,1604]
gggggattaactcagtggtcagagtggctgcctgttaagcagctggtcat
aggttcaaatcctatatcccccgcca
1−10tRNA−Tyr(gta)c[1611,1700]
ggttgaatgcgacgaattggtaagtcagcggattgtaaatccgtagccac
cgaaaggggcatctaggttcaagtcctagttcaaccacca
1−11tRNA−Met(cat)c[1794,1870]
agaggcttggctgagctggttatagcgactccttcatgcggagtaagacg
ggggttcgaatccctcagcctctacca
1−12tRNA−Ala(tgc)c[1958,2033]
ggggaattagctcagttgggagagcagggtctttgcaaggctcaggtcat
cggttcgagtccgatattctccacca
1−13tRNA−Glu(ttc)c[2040,2115]
acaccggaagacagatggattggtcgtcaccctttcaaggtggagtttgc
gggttcgagtcccgtccggtgtacca
1−14tRNA−Lys(ttt)c[2117,2193]
gcgcgtgtagctcagttggttagagcatctgacttttaatcaggtggtcg
aaggttcgagtccttctacgcgcacca
1−15tRNA−Met(cat)c[2200,2276]
tgcgagaatggagaaatggcgtcttgcgagtttcataagctcgataacgt
ggggttcgaatccccatctcgcatcca
>KT151957.1 Brevibacillus phage SecTim467, complete genome
1−1tRNA−Asn(gtt)c[7178,7252]
agggtagtagttcaatggtagaacgacgcactgttaatgcgtatgttgca
ggttcgagtcctgccttccctgcca
1−2tRNA−Pro(tgg)c[7356,7432]
cagggtgtagttcagttcgggagaacaccgcatttgggatgcggaggtcg
caggttcaagccctgccactctgacca
1−3tRNA−Met(cat)c[7434,7507]
gggtctttagctcgaaggccgagcaggcggctcatgaccgctaggtgtgg
gttcgattcccacaagacccacca
1−4tRNA−Cys(gca)c[7546,7618]
gtgtctgtccctagtcggtctagggtacggtctgcaaaaccgtattacgt

gggttcaagtcccaccagatgct
1–5tRNA–Tyr(gta)c[8396,8478]
ggatgagtatgtaagaggttaagctgacagagtgtaaatctgtcgcctga
tggcttcgtaggttcaactcctacctcacccac
1–6tRNA–His(gtg)c[8486,8574]
gtgtacgtggtggaaatggtagacacgcaagattgtggttcttgtggggc
gcaagccccgtgcaggttcaagtcctgtcgtacacccca
>KT326767.1 Mycobacterium phage Texage, complete genome
1–1tRNA–Asn(gtt)[3984,4059]
tgatctgtagctcaatcggcagagcacccggctgttaaccgggacgttgg
aggttcgagtcctcccagatcagcca
1–2tRNA–Trp(cca)[4100,4173]
aggcgcgtagctcaattggtagagcagcggtctccaaagccgctggctgc
aggttcgagtcctgccgcgtctgc
>KT372003.1 Mycobacterium phage Lumos, complete genome
1–1tRNA–Tyr(gta)[39561,39644]
gtggcgaaggcaactgttggtttgttgcacctgcctgtaaagcaggcgct
tcggcttcgggggttcaattccctctcgccacac
1–2tRNA–Thr(cgt)[62789,62861]
gccaccttagctcagcggtagagcagcgccttcgtaacgcgcaggtcaac
ggttcgattccgttaggtggctc
1–3tRNA–Pro(tgg)[63214,63286]
cggaaacaggtgtaatggttgcatgaggcgtttggatcgccggggttcgg
ggttcgactccctggtttccgac
1–4tRNA–Trp(cca)[63629,63702]
agctcggtagctcaactggtagagcagtggtctccaaagccaccggttgc
aggttcgactcctgtccgggttgc
1–5tRNA–Leu(tag)[63703,63777]
ggttccgtaggcaaactggcaaagccgtctgacttagaatcaggtgtttg
ggagttcgactctccccggaactac
1–6tRNA–His(gtg)[63778,63847]
gctaagtagcttaatggtaaagcccccggttgtggtccgggcgatcacgg
ttcaactccgtgcttaagcc
1–7tRNA–Gln(ctg)[64127,64201]
tgcccgctagcacaattggcagttgcgctgcgctctgaacgcagaggttc
caggttcgactcctgggtgggcagc
1–8tRNA–Lys(ctt)[64211,64284]
gcgtcgttagctcagttggtagagcagggcactcttaatgctcgggtcgg
gggttcaagtccctcacggcgcac
1–9tRNA–Pyl(cta)[65427,65519]
tgctcaaatgggtagggccgttcccctgtcatctaaaggcaggatgcctc
gctgttaacgaggtcacgcaggttcgacccctgctgggggagc
1–10tRNA–Lys(ttt)c[65524,65597]
gccgcgctagctcagttggtagagcagctgacttttaatcagcgggtccg

gggttcgatcccccggcgcggtac
>KU255188.1 Mycobacterium phage JenCasNa, complete genome
1-1tRNA-Asn(gtt)[3838,3913]
tgatctgtagctcaactggcagagcacccggctgttaaccgggtcgttgg
aggttcgagtcctcccagatcagcca
1-2tRNA-Trp(cca)[3954,4028]
aggcacgtagctcaattggtcagagcagcggtctccaaagccgccggctg
caggttcgagtcctgccgtgtctgc
1-3tRNA-Leu(cag)[4063,4137]
ggctcggtaggcaaacaggcaaagccgcctgtctcaggaacaggtgcgtg
agggttcgactccctcccgagctac
>KU234099.1 Mycobacterium phage MkaliMitinis3, complete genome
1-1tRNA-Thr(cgt)[63152,63225]
gccaccttagctcagttggtagagcagcgccttcgtaacgcgcaggtcag
cggttcgattccgctaggtggctc
1-2tRNA-Pro(tgg)[63687,63760]
cggcccaaggtgtaatggcttgcatgaggcgtttggatcgccggggttcc
ctgttcgactcggggtgggccgac
1-3tRNA-Trp(cca)[63831,63904]
agcgaattggtgtaatcggtagcatagcggcctccaaaaccgcctggtag
aggttcgagtcctctattcgttgc
1-4tRNA-Leu(tag)[64277,64351]
gctccgctaggcaaactggcaaagccgcctgatttaggttcaggtgtttc
ggggttcgactccccggtggagcac
1-5tRNA-His(gtg)[64351,64423]
cgcgaggtagcttaagtagtaaagcccccgactgtgactcgggtgattcg
ggtgcaagtcccggcctgtcgcc
1-6tRNA-Tyr(gta)[64426,64508]
gtggcggttgggcttgttggttggcccgcctgactgtaaatcaggtgttt
cggcatcgggggttcgattccctctcgccacac
1-7tRNA-Gln(ctg)[64516,64591]
tgaccgctagcacaattaggcagttgcgccggaatctgactccggaggtt
ccaggttcgattcctgggtggtcagc
1-8tRNA-Gly(tcc)[64707,64781]
gtgctagtaacccatgttggctgggtgccactcttccaaagtggatttcg
cgcgttcgattcgcgtctagcacac
1-9tRNA-Lys(ctt)[64816,64891]
gcctcgttagctcagttggtagagcagccgactcttaatcggcgggtcac
aggttcaagtcctgtacggggtacca
1-10tRNA-Ser(tga)[64893,64982]
ggagagtaatgcagcgggggtggcccgcgaccggccttgaaagccgggta
gccgtaatggagcttggggttcgatccctctgctctccgc
1-11tRNA-Cys(gca)[66074,66146]
gccgttgtgtccgagtggttaggtgccaggctgcaaacttggttagtcgc

gttcgattcgcggggacggctcc
1-12tRNA-Asn(gtt)[66299,66372]
tcctccgtagctcaattggcagagcgcgcgactgttaatcgcgtggttgg
tggttcgagtccacccgttggagc
1-13tRNA-Lys(ttt)c[66566,66640]
gcccctatagctcagttggtagagcaggagacttttaatcttcgggtcct
aggttcgatccctagtgggggcacc
>KT626047.1 Mycobacterium phage Dynamix, complete genome
1-1tRNA-Trp(cca)[1897,1972]
tggctcttagctcaatttggtagagcagcggtctccaaaaccgccggttg
caggttcgagtcctgcagagtcagcc
>KU716095.1 Mycobacterium phage Myxus, complete genome
1-1tRNA-Glu(ctc)[5906,5978]
ggtccgttggagtagcggcaactcatctgactctcactcagaagatcgcg
ggttcaaatcccgcacggactac
>KU935729.1 Mycobacterium phage SkinnyPete, complete genome
1-1tRNA-Thr(ggt)[26482,26559]
gatcctacggacgggaaagggacgtactacggtctggtaatccgcaggtc
gctggttcgagcccagctgggggcacca
>KU985095.1 Mycobacterium phage ChipMunk, complete genome
1-1tRNA-Gln(ctg)[4807,4881]
tccccgttcgtctaattggcaagacgccgggttctggccccggtaattga
ggttcgaatccttgatggggaacca
>KX241618.1 Xanthomonas phage Xoo-sp2, complete genome
1-1tRNA-Met(cat)[11369,11444]
cgcggggtagagcagctggcagctcgctgggttcataacccggaggtcgt
gggttcgagtcccactcccgcaacca
>KX160205.1 Lactococcus phage 49801, complete genome
1-1tRNA-Met(cat)[96,169]
ggttctttagctcagttggtcagagctaacggctcataaccgttcggtcg
ctggttcgagtccagcaagaacca
>KX160207.1 Lactococcus phage 53801, complete genome
1-1tRNA-Met(cat)[13752,13823]
acaggatgggtggcaaggcgtcacgctagtttcataagctagaatagaac
ggttcaatcccgttatcctgaa
>KX160208.1 Lactococcus phage 53802, complete genome
1-1tRNA-Met(cat)[15054,15125]
acaggatgggtggcaaggcgtcacgctagtttcataagctagaatagaac
ggttcaatcccgttatcctgaa
>KX160214.1 Lactococcus phage 98103, complete genome
1-1tRNA-Met(cat)[115,188]
ggttctttagctcagttggtcagagctaacggctcataaccgttcggtcg
ctggttcgagtccagcaagaacca
>KX557284.1 Gordonia phage Strosahl, complete genome

1−1tRNA−Asn(gtt)[5110,5185]
tcgcctgtagctcaattggcagagcacccggctgttaaccgggcggttga
aggttcgagtccttccaggcgagcca
1−2tRNA−Lys(ctt)[5187,5260]
gcatctgtagctgagtggtttagctctgggctcttaacccggagacggag
gttcgattcctcccagatgcacca
1−3tRNA−Gln(ctg)[5262,5334]
tccccattcgtccaattggtaggacaccggagtctggttccggagatcga
ggttcgaatcctcggtagggagc
>KX758539.1 Mycobacterium phage Jaan, complete genome
1−1tRNA−Gln(ctg)[6191,6265]
tcccggctagcacaactggtagctgcgctcggctctggaccgagaggttt
gaggttcgaatccttggccgggagc
>KX758538.1 Mycobacterium phage Kerberos, complete genome
1−1tRNA−Asn(gtt)[4209,4283]
tgacgtgtagctcaatggcagagcatccggctgttaaccggacggttgaa
ggttcgagtccttcctcgtcagcca
1−2tRNA−Trp(cca)[4324,4399]
gtacacgtagctcaattggtagagcagcggtctccaaagccgccggttcc
aggttcgactcctggcgtgtatgcca
1−3tRNA−Gln(ctg)[4407,4479]
tccccgttcgtctaatcggtaagacgcctggctctggaccaggtaattga
ggttcgagtccttggcggggagc
1−4tRNA−Glu(ctc)[4514,4587]
ggtcccttggagtagcggataactcacctggccctcacccagaagatcgc
gggttcgaatcccgcagggactac
1−5tRNA−Tyr(gta)[4591,4671]
cgcgagatacccaagcggcaacgggatctgactgtaaatcagacgcttcg
gcttcgcaggttcgagtcctgctctcgcgac
>KX712237.1 Rhodococcus phage Partridge, complete genome
1−1tRNA−Asn(gtt)[1496,1569]
tcacgtatagctcaatcggcagagcaacgcactgttaaggcgggggttga
aggttcgagtccttctacgtgagc
1−3tRNA−Trp(cca)[1761,1835]
gggcacgtagctcaattggatagagccccggtctccaaagccggtggttg
ggggttcgagtccctccgggcctgc
>KX712236.1 Rhodococcus phage Yogi, complete genome
1−1tRNA−Asn(gtt)[1503,1576]
tcacgtatagctcaatcggcagagcaacgcactgttaaggcgggggttga
aggttcgagtccttctacgtgagc
1−3tRNA−Trp(cca)[1702,1776]
gggcacgtagctcaattggatagagccccggtctccaaagccggtggttg
ggggttcgagtccctccgggcctgc
>KX688047.1 Mycobacterium phage Marcoliusprime, complete genome

1−1tRNA−Arg(gcg)[665,757]
caccgcttagcgaggttgcgcgccacgtcattgcgcccgacggcatcgtg
tcgacggcctggccgtcggtgtgtgcgacgtgcgcggcgatgg
>KX685355.1 Mycobacterium phage Qobbit, complete genome
1−1tRNA−Glu(ctc)[5936,6008]
ggtccgttggagtagcggcaactcatctgactctcactcagaagatcgcg
ggttcaaatcccgcacggactac
>KX664447.1 Mycobacterium phage Phaeder, complete genome
1−1tRNA−Glu(ctc)[5906,5978]
ggtccgttggagtagcggcaactcatctgactctcactcagaagatcgcg
ggttcaaatcccgcacggactac
>KX664448.1 Mycobacterium phage Watson, complete genome
1−1tRNA−Asn(gtt)[3832,3907]
tgatctgtagctcaatcggcagagcacccggctgttaaccgggacgttgg
aggttcgagtcctcccagatcagcca
1−2tRNA−Trp(cca)[3948,4022]
aggcacgtagctcaattggtcagagcagcggtctccaaagccgccggctg
caggttcgagtcctgccgtgtctgc
1−3tRNA−Leu(cag)[4057,4131]
ggctcggtaggcaaacaggcaaagccgcctgtctcaggaacaggtgcgtg
agggttcgactccctcccgagctac
>KX670828.1 Mycobacterium phage Todacoro, complete genome
1−1tRNA−Asn(gtt)[3984,4059]
tgatctgtagctcaatcggcagagcacccggctgttaaccgggacgttgg
aggttcgagtcctcccagatcagcca
1−2tRNA−Trp(cca)[4100,4173]
aggcgcgtagctcaattggtagagcagcggtctccaaagccgctggctgc
aggttcgagtcctgccgcgtctgc
>KX640831.1 Mycobacterium phage Margo, complete genome
1−1tRNA−Asn(gtt)[4009,4084]
tgatctgtagctcaatcggcagagcacccggctgttaaccgggacgttgg
aggttcgagtcctcccagatcagcca
1−2tRNA−Trp(cca)[4125,4198]
aggcgcgtagctcaattggtagagcagcggtctccaaagccgctggctgc
aggttcgagtcctgccgcgtctgc
>KX621007.1 Mycobacterium phage Taquito, complete genome
1−1tRNA−Lys(ttt)c[31241,31316]
gccccgctagctcaatcggaagagcagccggcttttaaccggcgggtacg
gagatcgaaactccggcggggcacca
>KX619650.1 Mycobacterium phage Jerm, complete genome
1−1tRNA−Gln(ctg)[4797,4871]
tccccgttcgtctaagcggcaagacaccgggttctggccccggcaatcga
ggttcgaatccttgatggggagcca
>KX611831.1 Mycobacterium phage Pharsalus, complete genome

1-1tRNA-Arg(cct)[62609,62692]
atggcccgtagctcagtcaggtagagcagccttgccccttgcgggcttgg
ttcgtccccggttcaaatccgggcgggccttgcc
1-2tRNA-Gly(tcc)[62695,62767]
gcgcccgtggtcgaattggaaagactcctggcttccacccaggttatgca
ggttcgagtcctgtcgggcgctc
>KX580961.1 Mycobacterium phage Zakai, complete genome
1-1tRNA-Thr(cgt)[63048,63121]
gccaccttagctcagttggtagagcagccccttcgtaacgggcaggtcag
cggttcgattccgctaggtggctc
1-2tRNA-Pro(tgg)[63349,63423]
ctggctgtagctcaacttggtagagcgctggctttgggtgccaggggttg
caggttcaaatcctgctagccagac
1-3tRNA-Trp(cca)[63544,63617]
agctcggtagctcaattggtagagcagcggtctccaaagccgccggttcc
ccgttcgagtcggggtcgggttgc
1-4tRNA-Tyr(gta)[63729,63811]
gtggtggttgggcttgttggttggcccacctgactgtaaatcaggcgttt
cggcaccgggggttcgattccctccccccacac
1-5tRNA-Leu(tag)[64225,64299]
ggccctctagcccaattggcagaggcacaggttttaggtacctgtcagtg
cgagttcgagtctcgcgggggccac
1-6tRNA-His(gtg)[64300,64369]
gcttagtagcctagtggtaaggcagccggttgtgatccggtgaacctgag
ttcgattctcagctaaagcc
1-7tRNA-Gln(ctg)[64374,64448]
tgaccggtagcacaactggtagttgcgccgcgctctggacgcggaggttg
ttggttcgatcccagctcggtcagc
1-8tRNA-Gly(tcc)[64564,64638]
gtgctagtaacccatgttggctgggtgcctgacttccactcaggatttcg
cgggttcgattcctgtctagcacac
1-9tRNA-Lys(ctt)[64681,64758]
tgcctcgttagctcagttggtagagctgccgactcttaatcggtaggtca
caggttcaagtcctgtacggggtaccca
1-10tRNA-Cys(gca)[65948,66021]
gccgtcatggctgagtggttaggcgtcggactgcaaatccggcttatccc
agttcgattctgggtggcggctcc
1-11tRNA-Asn(gtt)[66383,66456]
tcctccgtagctcaattggcagagcgcgcgactgttaatcgcgtggttgg
tggttcgagtccacccgttggagc
1-12tRNA-Lys(ttt)c[66650,66724]
gcccctatagctcagttggtagagcaggagacttttaatcttcgggtcct
aggttcgatccctagtgggggcacc
>KX580962.1 Mycobacterium phage Wilder, complete genome

1-1tRNA-Thr(cgt)[62791,62862]
gctggattagcttagtggtcaagcgctgccttcgtaacgcagattcaccg
gttcgattccggtatccagctc
1-2tRNA-Pro(tgg)[63091,63165]
ctggctgtagctcaacttggtagagcgctggctttgggtgccaggggttg
cgggttcaaatcctgctagccagac
1-3tRNA-Trp(cca)[63295,63368]
agctcggtagctcaattggtagagcagcggtctccaaagccgccggttcc
ccgttcgagtcggggtcgggttgc
1-4tRNA-Tyr(gta)[63480,63562]
gtggtggttgggcttgttggttggcccacctgactgtaaatcaggcgttt
cggcaccgggggttcgattccctcccgccacac
1-5tRNA-Leu(tag)[63604,63678]
ggccctctagcccaattggcagaggcacaggttttaggtacctgtcagtg
cgagttcgagtctcgcgggggccac
1-6tRNA-His(gtg)[63679,63748]
gcttagtagcctagtggtaaggcagccggttgtgatccggtgaacctgag
ttcgattctcagctaaagcc
1-7tRNA-Gln(ctg)[63753,63827]
tgaccggtagcacaactggtagttgcgccgcgctctggacgcggaggttg
ttggttcgatcccagctcggtcagc
1-8tRNA-Gly(tcc)[63946,64020]
gtgctagtaacccatgttggctgggtacctgacttccactcaggatttcg
cgggttcgattcctgtctagcacac
1-9tRNA-Lys(ctt)[64443,64518]
gcctcgttagctcagttggtagagctgccgactcttaatcggtaggtcac
aggttcaagtcctgtacggggtacca
1-10tRNA-Cys(gca)[65700,65772]
gccgttgtgtccgagtggttaggtgccaggctgcaaacctggttagtcgc
gttcgattcgcggggacggctcc
1-11tRNA-Asn(gtt)[66041,66114]
tcctccgtagctcaattggcagagcgcgcgactgttaatcgcgtggttgg
tggttcgagtccacccgttggagc
1-12tRNA-Lys(ttt)c[66308,66382]
gcccctatagctcagttggtagagcaggagacttttaatcttcgggtcct
aggttcgatccctagtgggggcacc
>KX585253.1 Mycobacterium phage HedwigODU, complete genome
1-1tRNA-Trp(cca)[1003,1076]
gggtgtgtagctcaatcggtagagcagcggtctccaaagccgccggttgc
acgttcgagtcgtgccgcgcccgc
>KX585252.1 Mycobacterium phage HortumSL17, complete genome
1-1tRNA-Glu(ctc)[5906,5978]
ggtccgttggagtagcggcaactcatctgactctcactcagaagatcgcg
ggttcaaatcccgcacggactac

>KX585251.1 Mycobacterium phage Waterfoul, complete genome
1-1tRNA-Trp(cca)[843,918]
atgcaggtagctcaattggtagagcagcggtctccaaaaccgccggttgc
aggttcgagtcctgccctgtgtgcca
>KX670788.1 Mycobacterium phage Louie6, complete genome
1-1tRNA-Asn(gtt)[3922,3997]
tgatctgtagctcaatcggcagagcacccggctgttaaccgggacgttgg
aggttcgagtcctcccagatcagcca
1-2tRNA-Trp(cca)[4038,4112]
aggcacgtagctcaattggtcagagcagcggtctccaaagccgccggctg
caggttcgagtcctgccgtgtctgc
1-3tRNA-Leu(cag)[4147,4221]
ggctcggtaggcaaacaggcaaagccgcctgtctcaggaacaggtgcgtg
agggttcgactccctcccgagctac
>KX550082.1 Rhodococcus phage Natosaleda, complete genome
1-1tRNA-Asn(gtt)[1503,1576]
tcacgtatagctcaatcggcagagcaacgcactgttaaggcgggggttga
aggttcgagtccttctacgtgagc
1-3tRNA-Trp(cca)[1702,1776]
gggcacgtagctcaattggatagagccccggtctccaaagccggtggttg
ggggttcgagtccctccgggcctgc
>KX574455.1 Mycobacterium phage Pomar16, complete genome
1-1tRNA-Asn(gtt)[4209,4283]
tgacgtgtagctcaatggcagagcatccggctgttaaccggacggttgaa
ggttcgagtccttcctcgtcagcca
1-2tRNA-Trp(cca)[4324,4397]
gtacacgtagctcaattggtagagcagtggtctccaaagccgccggttcc
aggttcgactcctggcgtgtatgc
1-3tRNA-Gln(ctg)[4449,4521]
tccccgttcgtctaatcggtaagacgcctggctctggaccaggtaattga
ggttcgagtccttggcggggagc
1-4tRNA-Glu(ctc)[4555,4628]
ggtcccttggagtagcggagaactcatctggccctcacccagaagatcgc
gggttcgaatcccgcagggactac
1-5tRNA-Tyr(gta)[4632,4712]
cgcgagatacccaagcggcaacgggatctgactgtaaatcagacgcttcg
gcttcgcaggttcgagtcctgctctcgcgac
>KX641261.1 Mycobacterium phage Isiphiwo, complete genome
1-1tRNA-Asn(gtt)[5402,5474]
tgacgtgtagctcaatggcagagcacccggctgttaaccggacggttgaa
ggttcgagtccttccatgtcagc
1-2tRNA-Trp(cca)[5479,5552]
gcgctcctagctcaattggtagagcagcggtctccaaagccgcgagttcc
aggttcgagtcctggggagcgtgc

1−3tRNA−Gln(ctg)[5594,5666]
tccctgttcgtctaatcggtaagacaccgggctctggacccggcaatcga
ggttcgagtccttgatggggagc
>KX641263.1 Mycobacterium phage Kalpine, complete genome
1−1tRNA−Trp(cca)[6137,6210]
gggcgtgtagctcaattggtagagcagcggtctccaaagccgccggttcc
aggttcgaatcctggcgggcccgc
1−2tRNA−Glu(ctc)[6245,6318]
gctcccttggagtagcggttaactcatctgactctcaatcagaagatcgc
gggttcgaaccccgcagggagtac
>KX641264.1 Mycobacterium phage LindNT, complete genome
1−1tRNA−Trp(cca)[979,1052]
gggtgcgtagctcaatcggtagagcagcggtctccaaagccgccggttgc
acgttcgagtcgtgccgcgcccgc
>KX641260.1 Mycobacterium phage Stasia, complete genome
1−1tRNA−Trp(cca)[3994,4067]
aggcacgtagctcaatcggtagagcagcggtctccaaagccgccggctgc
aggttcgagtcctgccgtgtctgc
>KX611788.1 Rhodococcus phage Harlequin, complete genome
1−1tRNA−Asn(gtt)[1503,1576]
tcacgtatagctcaatcggcagagcaacgcactgttaaggcgggggttga
aggttcgagtccttctacgtgagc
1−3tRNA−Trp(cca)[1702,1776]
gggcacgtagctcaattggatagagccccggtctccaaagccggtggttg
ggggttcgagtccctccgggcctgc
>KX576638.1 Mycobacterium phage Gruunaga, complete genome
1−1tRNA−Asn(gtt)[5396,5468]
tggcgtgtagctcaatggcagagcccccggctgttaaccgggtggttgaa
ggttcgagtccttccacgccagc
1−2tRNA−Trp(cca)[5472,5545]
gcgttcctagctcaattggtagagcagcggtctccaaagccgcgtgttcc
aggttcgagtcctggggagcgtgc
1−3tRNA−Gln(ctg)[5587,5659]
tccctgttcgtctaatcggtaagacaccgggctctggacccggtaatcga
ggttcgagtccttgatggggagc
>KX834009.1 Mycobacterium phage Goldilocks, complete genome
1−1tRNA−Arg(cct)[62292,62375]
atggcccgtagctcagtcaggtagagcagccttgccccttgcgggcttgg
ttcgtccccggttcaaatccgggcgggccttgcc
1−2tRNA−Gly(tcc)[62378,62450]
gcgcccgtggtcgaattggaaagactcctggcttccacccaggttatgca
ggttcgagtcctgtcgggcgctc
>KX845404.2 Klebsiella phage vB_Kpn_IME260, complete genome
1−1tRNA−Trp(cca)c[30293,30369]

attccccgagtgttactggacagcacgccggtctccaaaaccgtgcagta
ggagttcgagtctcctggggtttgcca
1-2tRNA-Thr(tgt)c[31163,31238]
gcctcaatagctcagccgggagagcaaccgccttgtaagcggtaggtcgt
gggttcgattcctacttggggcacca
1-3tRNA-Pro(tgg)c[31244,31320]
ttccccttagctcagtttggtagagcgggcgctttgggagcgtcaggtca
agtgttcaaatcacttaggggagacca
1-4tRNA-Leu(taa)c[31578,31663]
acctctgtggtggaactggtaatacacaccatgcttaaaacgtggcgctt
aaatgattgagggttcgagtccctccagaggtacca
1-5tRNA-Phe(gaa)c[32653,32730]
gcaactgtagctcagcgaggtgagagcactggtttgaaagtccaggggtc
gttcgttcaaatcgaaccggttgcacca
1-6tRNA-Lys(ttt)c[32906,32981]
agatcgctagcttaatggttagagcactcgccttttaagcgataggttcc
gggttcgagtcccgggcggtctacca
1-7tRNA-Cys(gca)c[33168,33241]
gcatcattggccgagtgactaggcagaggcttgcaaaccctcgcagcatg
gttaaaatccatgatggtgctcca
1-8tRNA-Asn(gtt)c[33322,33397]
gagagaagcagtaagtggtataggcggtcgcctgttaagcgaatgacagt
gagttcgaatctcacctctcccgcca
1-9tRNA-Tyr(gta)c[33507,33593]
ggggaatattctgtaagtggtagcagagcggtctgtaaaatcgttgccat
tgcggctcgggtggttcgactccatcttcccccacca
1-10tRNA-Asp(gtc)c[33830,33906]
gcggctatggtgttcagcggtcaacataccggcctgtcacgtcggagcca
cgggttcgaatcccgttagccgcgcca
1-11tRNA-Val(tac)c[35476,35551]
acttgcttagctcaatcggtagagcatcgtctttacacggcgagggtagc
tggttcgaaaccagcagcaagtacca
1-12tRNA-Met(cat)c[35555,35631]
ggtcagttggcagagatggtttatgcactcgcttcatacgtgagactaca
gtggttcgagtccactattgaccacca
1-13tRNA-Ile(gat)c[35637,35712]
gcttcgatagctcagttggtagagcaaacgaccgataatcgttaggtcac
tggttcgagtccagttcggagtacca
1-14tRNA-Met(cat)c[36088,36164]
tgcaccgtagaggagaggccgtcctcgccagtctcataagctggagatcg
caagttcgaatcttgccggagcatcca
1-15tRNA-Gln(ctg)c[36167,36242]
tggggtatagctcagtaggtagagcggaggtctctgaagccttaggtcac
aagttcgattcttgttgcccctgcca

1-16tRNA-Arg(acg)c[36372,36446]
gcaccattagtttaatggatagaatatagagctacgaactctatggttga
ggttcgattcctcgatggtgtacca
1-17tRNA-His(gtg)c[36454,36530]
gggatattatcataactggataatgacctcgattgtggatcgagtctatc
ttggttcgaatccaagatatccctcca
1-18tRNA-Ser(gct)c[36913,36999]
ggaaaactggtgaaattggtagccacacatcactgctaatgatgtatccg
taagggtatgtaggttcaagtcctacgttttccgcca
1-19tRNA-Leu(tag)c[37211,37297]
ggggatgtggcgaaattggcagccgcgctagatttaggttctagtggtga
aatatccgtgtgggttcgacccccctccatccctacca
1-20tRNA-Ala(tgc)c[37305,37383]
gggggatgggtctgctgggagtggacaccgcacttgcaatgcgggaatca
gaacggttcaaatccgttatcctccacca
1-21tRNA-Glu(ttc)c[37657,37733]
gctcctgtcgtctaagctggttaggacactactctttcacagtgggaaca
cgggttcgaaccccgtcgggagtacca
1-22tRNA-Gln(ttg)c[37744,37819]
aagggaatagccaagtggttacggcatcggcctttgactccgagatcggt
aggttcaactcctccttcccttgcca
1-23tRNA-Met(cat)c[38847,38924]
ggttctcaagctcattgggtatgagcacccgcctcataagcggaaggtag
gcaggttcgaatcctccgggagccacca
1-24tRNA-Arg(tct)c[39400,39475]
gcttccttagctcagaggatagagcaacggtcttctaaaccgtgggtcac
aggttcgaatcctgtagggagtacca
>KX657793.1 Mycobacterium phage DarthPhader, complete genome
1-1tRNA-Gln(ctg)[6296,6368]
tccccggtagtccaactggcaagacgctcggctctggaccgagagaccga
ggttcgaatccttgccggggagc
1-2tRNA-Pro(ggg)c[21030,21120]
gttgccacgcgggtcggtcttcatctcgaccttgggggactcgaagagcg
ccaggtggagataccgggtgccagagtccggggtggtgacg
>KX657795.1 Mycobacterium phage DrHayes, complete genome
1-1tRNA-Trp(cca)[985,1058]
gggtgtgtagctcaatcggtagagcagcggtctccaaagccgccggttgc
acgttcgagtcgtgccgcgcccgc
>KX657794.1 Mycobacterium phage SamuelLPlaqson, complete genome
1-1tRNA-Trp(cca)[985,1058]
gggtgtgtagctcaatcggtagagcagcggtctccaaagccgccggttgc
acgttcgagtcgtgccgcgcccgc
>KX657796.1 Mycobacterium phage Urkel, complete genome
1-1tRNA-Trp(cca)[985,1058]

gggtgtgtagctcaatcggtagagcagcggtctccaaagccgccggttgc
acgttcgagtcgtgccgcgcccgc
>KX808129.1 Mycobacterium phage Sabinator, complete genome
1-1tRNA-Asn(gtt)[3838,3913]
tgatctgtagctcaactggcagagcacccggctgttaaccgggtcgttgg
aggttcgagtcctcccagatcagcca
1-2tRNA-Trp(cca)[3954,4028]
aggcacgtagctcaattggtcagagcagcggtctccaaagccgccggctg
caggttcgagtcctgccgtgtctgc
1-3tRNA-Leu(cag)[4063,4137]
ggctcggtaggcaaacaggcaaagccgcctgtctcaggaacaggtgcgtg
agggttcgactccctcccgagctac
>KX817173.1 Mycobacterium phage Tuco, complete genome
1-1tRNA-Arg(cct)[63996,64079]
atggcccgtagctcagtcaggtagagcagccttgccccttgcgggcttgg
ttcgtccccggttcaaatccgggcgggccttgcc
1-2tRNA-Gly(tcc)[64082,64154]
gcgcccgtggtcgaattggaaagactcctggcttccacccaggttatgca
ggttcgagtcctgtcgggcgctc
>KX897981.1 Mycobacterium phage StarStuff, complete genome
1-1tRNA-Asn(gtt)[4209,4283]
tgacgtgtagctcaatggcagagcatccggctgttaaccggacggttgaa
ggttcgagtccttcctcgtcagcca
1-2tRNA-Trp(cca)[4324,4399]
gtacacgtagctcaattggtagagcagtggtctccaaagccgccggttcc
aggttcgactcctggcgtgtatgcca
1-3tRNA-Gln(ctg)[4407,4479]
tccccgttcgtctaatcggtaagacgcctggctctggaccaggtaattga
ggttcgagtccttggcggggagc
1-4tRNA-Glu(ctc)[4514,4587]
ggtcccttggagtagcggagaactcatctggccctcacccagaagatcgc
gggttcgaatcccgcagggactac
1-5tRNA-Tyr(gta)[4591,4671]
cgcgagatacccaagcggcaacgggatctgactgtaaatcagacgcttcg
gcttcgcaggttcgagtcctgctctcgcgac
>KY087992.1 Mycobacterium phage Mitti, complete genome
1-1tRNA-Arg(gcg)[14120,14213]
tcggcggcgctgcgcaggagatgggcagcagcgttcgcggcaccctgtcg
aacctcaaggcggcctattcgcggttcggtgccgagctgtcggg
1-2tRNA-Lys(ttt)c[30768,30843]
gccccgctagctcaatcggaagagcagccggcttttaaccggcgggtacg
gagatcgaaactccggcggggcacca
>KY087993.1 Mycobacterium phage Hammy, complete genome
1-1tRNA-Trp(cca)[809,884]

gggttcctagctcaattggtagagcagcggtctccaaagccgccggttgc
aggttcgactcctgcggagcccgcca
1-2tRNA-Lys(ctt)c[32806,32885]
gcacttttggctggtgagctaccgtaacaggcagactcttaatcagcggg
tccggggttcgaaaccctgacggcgcacca
>KY130461.1 Mycobacterium phage Taptic, complete genome
1-1tRNA-Gly(tcc)[51204,51275]
gcgcgactggtgtaactggaacacaacccgcttccagcgggaggctcccc
gttcgtgacgggggtcgcgctc
>KY223999.1 Mycobacterium phage MrMagoo, complete genome
1-1tRNA-Trp(cca)[57182,57253]
atgggcgtagctgacaggaaagcaacggtctccaaagccgtgacacgcag
gttcgaatcctgccgcccgtgc
1-2tRNA-Asn(gtt)[57783,57858]
tggggtatagccacaatggcagtggcaccgggctgttaactcggaagagt
gttggttcgagtccagctaccctagc
1-3tRNA-Arg(tcg)[58707,58778]
cggatagtcgtccagcgggggcggtgggtttttcgcgagcccacgagcag
gttcgaatcctgactttccggc
1-4tRNA-Tyr(gta)[58851,58936]
ctcgtctctctcctattggtgaagtgatctcaggctgtaaccctgagccc
tgcggggtgtggtggttcgaatccatcaggcgagac
1-5tRNA-Gln(ctg)[59225,59296]
tgtcttctcgactaacggcaagtcaccaggctctgaccctggcaattgag
gttcgaatccttgggggacaac
1-6tRNA-Pro(tgg)[59302,59374]
cgggatatggtgtaagggttagcgtaccggcgttggagaccggaagacat
ggttcgagtccatgtatcccgac
1-7tRNA-Phe(gaa)[59819,59891]
cgtgttgtagctcagttggaagagcggcggcttgaaaacccgcaggccca
ggttcggctcctggcatcacggc
1-8tRNA-Met(cat)[60032,60103]
tgcgcggtggtgtaacgggaacatcctggtctcataagtcagggcttcgg
gttcgaatcccggctgcgcaac
1-9tRNA-Arg(acg)[60409,60487]
cccgatgttgagcaacggagcgctcaatgggctacgaacccatattcgca
tgcaggttcgaatcctgtcatcgggtcca
1-10tRNA-His(gtg)[60490,60562]
cctcttgtagctcaatggaagagcagcggtttgtgagaccgcagacgacg
gatcgttaccgtccagggggacc
1-11tRNA-Cys(gca)[60749,60821]
gtcgagatgactgagtggataagtagcggcctgcaaagctgacttacgcc
ggttcgaatccggctctcgattc
1-12tRNA-Leu(tag)[61075,61151]

ggatctgtagcccaaatggaagaggcaacaggcttagaccctgttcagtg
tgggttcgactccctccagattcacca
1-13tRNA-Leu(caa)[61153,61228]
gctcgactaggcaaactaggcaaagccgcaggcctcaaacgccagtgttt
gtgagttcgaatctcacgtcgagtac
1-14tRNA-Lys(ctt)[61232,61306]
gggatgttggtataaacggcaattactgcagactcttaatctgtcaaatc
tgggttcgaatcccaggcgtcccac
1-15tRNA-Ile(gat)[61307,61382]
tgtgatgtcgtccaacggtttagggcaccgatctgatacatcggaaacca
gggttcaactccctgcttcactacca
1-16tRNA-Gly(tcc)[61470,61546]
ggggtattcatctagtctggctcaggatgcgacacttccactgtcgtcac
gagggttcgaatccttcatacctcacc
1-17tRNA-Val(tac)[61593,61664]
tccccactcgtccaatgggagggcaccgcgcttacaacgcgaggacggga
gttcgattctctcgtggggaac
1-18tRNA-Thr(agt)[61725,61802]
gctggatttgctcatgttggcttgagcacctacctagtaagtaggagtac
gtcggttcgattccgacatccagcccca
1-19tRNA-Ala(tgc)[61861,61931]
gggcgcatgttccaaggtggcgaccgacccttgcaaggttggtgggcgag
ttcgattctcgtcgcttccac
1-20tRNA-Asp(gtc)[62009,62084]
cccgtcatcatctagaggcctaggatgccactctgtcgaagtggtcacac
gggttcaaatcccgttggcgggacca
1-21tRNA-Glu(ctc)[62144,62215]
ggttcattggtgtaatggcagcatcgcaggttctcaacctgttggcaggg
gatcgtaacccctatgaactac
>KY224000.1 Mycobacterium phage Rich, complete genome
1-1tRNA-Arg(ccg)[35149,35243]
cccggcaagttctggcactggtggggcaccaacacctccgacgagtggct
gggaccggtcgagcccggccagcggttcaacctcgcctaccgggc
>KY380102.1 Mycobacterium phage CREW, complete genome
1-1tRNA-Trp(cca)[1053,1126]
gggtgtgtagctcaatcggtagagcagcggtctccaaagccgccggttgc
acgttcgagtcgtgccgcgcccgc
>KY303907.1 Enterococcus phage EF-P29, complete genome
1-1tRNA-Trp(cca)c[24880,24952]
tgcgttgtaagtgtaatggctgcacaatggtctccaaaaccattagagag
ggttcgactccttcacaacgtgt
>KY319168.1 Mycobacterium phage CrystalP, complete genome
1-1tRNA-Arg(cct)[63089,63172]
atggcccgtagctcagtcaggtagagcagccttgccccttgcgggcttgg

ttcgtccccggttcaaatccgggcgggccttgcc
1-2tRNA-Gly(tcc)[63175,63247]
gcgcccgtggtcgaattggaaagactcctggcttccacccaggttatgca
ggttcgagtcctgtcgggcgctc
>KX130960.1 Escherichia phage vB_EcoS-IME253, complete genome
1-1tRNA-Arg(tct)[11634,11711]
gctctgctagctcaacaggatagcagcaacgaccttctaagtcgtaggtt
actggttcgaatccagtgcggagcgcca
>KY398841.1 Escherichia phage vB_Ecos_CEB_EC3a, complete genome
1-1tRNA-Arg(tct)[4662,4739]
gctctgctagctcaacaggacagcagcaacgaccttctaagtcgtaggtt
actggttcgaatccagtgcggagcgcca
>KY549152.1 Mycobacterium phage Maxxinista, complete genome
1-1tRNA-Arg(cct)[62523,62606]
atggcccgtagctcagtcaggtagagcagccttgccccttgcgggcttgg
ttcgtccccggttcaaatccgggcgggccttgcc
1-2tRNA-Gly(tcc)[62609,62681]
gcgcccgtggtcgaattggaaagactcctggcttccacccaggttatgca
ggttcgagtcctgtcgggcgctc
>KY549153.1 Rhodococcus phage Angryorchard, complete genome
1-1tRNA-Asn(gtt)[1496,1569]
tcacgtatagctcaatcggcagagcaacgcactgttaaggcgggggttga
aggttcgagtccttctacgtgagc
1-2tRNA-Trp(cca)[1647,1721]
gggcacgtagctcaattggatagagccccggtctccaaagccggtggttg
ggggttcgagtccctccgggcctgc
>KY549154.1 Rhodococcus phage BobbyDazzler, complete genome
1-1tRNA-Asn(gtt)[1503,1576]
tcacgtatagctcaattggcagagcagcgcactgttaaggcgttggttga
aggttcgagtccttctacgtgagc
1-3tRNA-Trp(cca)[1766,1840]
gggcacgtagctcaattggatagagccccggtctccaaagccggtggttg
ggggttcgagtccctccgggcctgc
>KY464936.1 Mycobacterium phage Idleandcovert, complete genome
1-1tRNA-Asn(gtt)[3721,3794]
tgacgtgtagctcaatcggcagagcacccggctgttaaccgggcggttga
aggttcgagtccttccatgtcagc
1-2tRNA-Trp(cca)[3829,3902]
aggcgcgtagctcaattggaagagcagcggtctccaaagccgccggctgc
aggttcgagccctgccgcgtctgc
>KY471269.1 Gordonia phage DinoDaryn, complete genome
1-1tRNA-Lys(ttt)c[24013,24086]
gccccttagctcagttggtagagctggtgacttttaatcactaggtcgt
aggttcgagtcctacagggggcac

>KY471268.1 Gordonia phage Huffy, complete genome
1−1tRNA−Lys(ttt)c[24013,24086]
gccccttagctcagttggtagagctggtgacttttaatcactaggtcgt
aggttcgagtcctacagggggcac

>KY472224.1 Enterococcus phage EF−P10, complete genome
1−1tRNA−Trp(cca)c[21555,21627]
tgcgttgtaagtgtaatggctgcacaatggtctccaaaaccattagagag
ggttcgactccttcacaacgtgt

>KY471460.1 Mycobacterium phage Jabith, complete genome
1−1tRNA−Trp(cca)[6115,6188]
gcacttctagctcaattggtagagcagcggtctccaaagccgccggttcc
aggttcaagtcctggggagtgtgc

>KY555142.1 Caulobacter phage Ccr10, complete genome
1−1tRNA−Trp(cca)[47810,47885]
gtcggtctagctcatggggtagagcggcggtctccaaaaccgcgcgtggc
aggttcgagtcctgcgaccggcgcca
1−2tRNA−Gly(gcc)[47891,47965]
gcgtccatcgtatatgggttattgcttcgcgttgccaacgcgacgaaccg
ggttcgagtcccggtggacgctcca
1−3tRNA−Gly(tcc)[47972,48045]
gcgggtatagctcaaagggagagctactgccttccaagcagaagatgcgg
gttcgagccccgctatccgctcca
1−4tRNA−Glu(ttc)[48202,48279]
ggcccggtcgtctagcggtttgcaggattcgtgactttcaatcacgagag
cggagttcgagtctccgtcgggcctcca
1−5tRNA−Asp(gtc)[48301,48375]
ggtgcgttcgtatatcggtcaatactccggattgtcgatccggcaagagg
ggttcgattcccctacgcaccgcca
1−6tRNA−Pro(cgg)[49245,49320]
cggggtgtagctcagtcggtagagcgctcggttcgggaccgagaggccgt
gggttcgagtcccgccacccagacca
1−7tRNA−Pro(ggg)[49328,49402]
cgggatgtagctcagcggtagagcgcgcggctgggggccgcgaggccgca
ggttcgagacctgccaccccgacca
1−8tRNA−Thr(ggt)[49408,49480]
gctgctgcgcctggatcggcgccggccccttggtatggggaagtagaagg
ttcaattccttccagcagcacca
1−9tRNA−Lys(ctt)[49593,49679]
ggatgtgtcacctcagtggcgagaggaccgggctcttaatccggcagcga
aagctcatcgtgggttcgagtcccaccacatcctcca
1−10tRNA−Lys(ttt)[49685,49775]
gcacgagtgacccgagtggcgaagggcgtcgacttttaatagactaggcg
tccgcgcctcaccgagggttcgagtccctcctcgtgctcca
1−11tRNA−Leu(cag)[49782,49865]

gcgaaagtggcggaacggcagacgcactggtctcaggtaccagcgacttc
ggtcatgagggttcgaatccctcctttcgtacca
1-12tRNA-Leu(caa)[49873,49957]
gccggtctggcggaaatggcagacgcagtggcttcaagtcccaccgcctt
cgggcgtcccggttcgaggccggggaccggcacca
1-13tRNA-Leu(gag)[49963,50051]
tgcgaagtggcggaactttggtagacgcaccggcatgagaggccggcgcc
cttcggggcgtgagggttcgagtcccttcttcgcgacca
1-14tRNA-Ala(tgc)[50135,50205]
ggggtatgcgcagggcgcaggcatcctttgcaaggaagtcggggtgggtt
cgagtcccacatactccacca
1-15tRNA-Ser(cga)[50210,50297]
ggtaggttggctgagaggccgaaggcgcccgtttcgaaaacgggaggacc
gcaaggttccgtgggttcgaatcccacacctacctcca
1-16tRNA-Ser(gga)[50302,50389]
ggatgtatggctgagaggccgaaggcgcccgcttggaaagcgggtgaacc
gcaaggttccgtaggttcgaatcctactgcatccgcca
1-17tRNA-Phe(gaa)[51084,51159]
ggactcgtagctcagcaggtagagcgccggcttgaaaccccggaggtcgg
aggttcgactcctcccgtttccacca
1-18tRNA-Val(gac)[51165,51240]
gcccgatgagccagatgggaaggcgccgccgcgacatggcgggattgaga
aggttcgattccttcatcaggcacca
1-19tRNA-Ile(gat)[51243,51318]
gcaagtgtagctcagttggtagagcagcggaccgataatccgcccgtccc
tggttcgagtccaggcatttgcacca
1-20tRNA-Ile(gat)[51326,51400]
tgtggcatagctcaggggtagagcgcgcgcttgataagcgtgaggacggg
ggttcgaagccctctgccactacca
1-21tRNA-Val(cac)[51434,51509]
tggcgtttagcatagtgggagtgcaggggtgtcacatacctcgatcgaac
tggttcgattccagtaacgccaacca
1-22tRNA-Gln(ctg)[51515,51588]
tgagggttcgtctaacggtaggacggctgggtctggtccagctaatctag
gttcgagtcctagcccctcatcca
1-23tRNA-Gln(ttg)[51597,51672]
tcagggttagtttagctggtaaaacactgggttttggccccggcgtcgct
aggttcgaagcctgcaccctgatcca
1-24tRNA-Ile(aat)[51684,51758]
tccagtcaatgcacaaggtgtgcagcggcactgttaatgcctgtgagccc
ggttcgattccggggtctggagcca
1-25tRNA-Arg(tct)[51961,52038]
gcgcaggtagctcaacggacccagagcgaccgtcttctacacggagggtt
gggggttcgaatccctccctgcgcgcca

1–26tRNA–Cys(gca)c[101366,101454]
gcttcggtggactgagaggttaggcgcgggattgcaaatctctgctggtg
ggaacacctatgagggttcgagtccctcccgaagctcca
1–27tRNA–Gly(tcc)c[102034,102107]
tttgcgaatagcacagtggtagtgcaacagccgtcctagctgtcggtcgc
gggttcaatccccgcttcgatgaa
1–29tRNA–Met(cat)[103180,103258]
cgcggggtagaggagtccggttgtcctcgtctggctcataaccaggagat
cgtgggttcaaatcccactcccgctccca
1–30tRNA–Met(cat)[103630,103714]
ggtacggtggctcgacaggtaaggcatcggcctcataagccgagggttcg
tcccaatcctggttcaaatccaggtcgtacctcca
>KY555143.1 Caulobacter phage Ccr2, complete genome
1–1tRNA–Trp(cca)[48285,48360]
gtcggtctagctcatggggtagagcggcggtctccaaaaccgcgcgtggc
aggttcgagtcctgcgaccggcgcca
1–2tRNA–Gly(gcc)[48366,48440]
gcgtccatcgtatatgggttattgcttcgcgttgccaacgcgacgaaccg
ggttcgagtcccggtggacgctcca
1–3tRNA–Gly(tcc)[48447,48520]
gcgggtatagctcaaagggagagctactgccttccaagcagaagatgcgg
gttcgagccccgctatccgctcca
1–4tRNA–Glu(ttc)[48677,48754]
ggcccggtcgtctagcggtttgcaggattcgtgactttcaatcacgagag
cggagttcgagtctccgtcgggcctcca
1–5tRNA–Asp(gtc)[48776,48850]
ggtgcgttcgtatatcggtcaatactccggattgtcgatccggcaagagg
ggttcgattcccctacgcaccgcca
1–6tRNA–Pro(cgg)[49720,49795]
cggggtgtagctcagtcggtagagcgctcggttcgggaccgagaggccgt
gggttcgagtcccgccacccagacca
1–7tRNA–Pro(ggg)[49803,49877]
cgggatgtagctcagcggtagagcgcgcggctgggggccgcgaggccgca
ggttcgagacctgccaccccgacca
1–8tRNA–Thr(ggt)[49883,49955]
gctgctgcgcctggatcggcgccggccccttggtatggggaagtagaagg
ttcaattccttccagcagcacca
1–9tRNA–Lys(ctt)[50068,50154]
ggatgtgtcacctcagtggcgagaggaccgggctcttaatccggcagcga
aagctcatcgtgggttcgagtcccaccacatcctcca
1–10tRNA–Lys(ttt)[50160,50250]
gcacgagtgacccgagtggcgaagggcgtcgacttttaatagactaggcg
tccgcgcctcaccgagggttcgagtccctcctcgtgctcca
1–11tRNA–Leu(cag)[50257,50340]

gcgaaagtggcggaacggcagacgcactggtctcaggtaccagcgacttc
ggtcatgagggttcgaatccctcctttcgtacca
1-12tRNA-Leu(caa)[50348,50432]
gccggtctggcggaaatggcagacgcagtggcttcaagtcccaccgcctt
cgggcgtcccggttcgaggccggggaccggcacca
1-13tRNA-Leu(gag)[50438,50526]
tgcgaagtggcggaactttggtagacgcaccggcatgagaggccggcgcc
cttcggggcgtgagggttcgagtcccttcttcgcgacca
1-14tRNA-Ala(tgc)[50610,50680]
ggggtatgcgcagggcgcaggcatcctttgcaaggaagtcggggtgggtt
cgagtcccacatactccacca
1-15tRNA-Ser(cga)[50685,50772]
ggtaggttggctgagaggccgaaggcgcccgtttcgaaaacgggaggacc
gcaaggttccgtgggttcgaatcccacacctacctcca
1-16tRNA-Ser(gga)[50777,50864]
ggatgtatggctgagaggccgaaggcgcccgcttggaaagcgggtgaacc
gcaaggttccgtaggttcgaatcctactgcatccgcca
1-17tRNA-Phe(gaa)[51559,51634]
ggactcgtagctcagcaggtagagcgccggcttgaaaccccggaggtcgg
aggttcgactcctcccgtttccacca
1-18tRNA-Val(gac)[51640,51715]
gcccgatgagccagatgggaaggcgccgccgcgacatggcgggattgaga
aggttcgattccttcatcaggcacca
1-19tRNA-Ile(gat)[51718,51793]
gcaagtgtagctcagttggtagagcagcggaccgataatccgcccgtccc
tggttcgagtccaggcatttgcacca
1-20tRNA-Ile(gat)[51801,51875]
tgtggcatagctcaggggtagagcgcgcgcttgataagcgtgaggacggg
ggttcgaagccctctgccactacca
1-21tRNA-Val(cac)[51909,51984]
tggcgtttagcatagtgggagtgcaggggtgtcacatacctcgatcgaac
tggttcgattccagtaacgccaacca
1-22tRNA-Gln(ctg)[51990,52063]
tgagggttcgtctaacggtaggacggctgggtctggtccagctaatctag
gttcgagtcctagcccctcatcca
1-23tRNA-Gln(ttg)[52072,52147]
tcagggttagtttagctggtaaaacactgggttttggccccggcgtcgct
aggttcgaagcctgcaccctgatcca
1-24tRNA-Ile(aat)[52159,52233]
tccagtcaatgcacaaggtgtgcagcggcactgttaatgcctgtgagccc
ggttcgattccggggtctggagcca
1-25tRNA-Arg(tct)[52436,52513]
gcgcaggtagctcaacggacccagagcgaccgtcttctacacggagggtt
gggggttcgaatccctccctgcgcgcca

1−26tRNA−Cys(gca)c[101841,101929]
gcttcggtggactgagaggttaggcgcgggattgcaaatctctgctggtg
ggaacacctatgagggttcgagtccctcccgaagctcca
1−27tRNA−Gly(tcc)c[102509,102582]
tttgcgaatagcacagtggtagtgcaacagccgtcctagctgtcggtcgc
gggttcaatccccgcttcgatgaa
1−29tRNA−Met(cat)[103655,103733]
cgcggggtagaggagtccggttgtcctcgtctggctcataaccaggagat
cgtgggttcaaatcccactcccgctccca
1−30tRNA−Met(cat)[104105,104189]
ggtacggtggctcgacaggtaaggcatcggcctcataagccgagggttcg
tcccaatcctggttcaaatccaggtcgtacctcca
>KY555145.1 Caulobacter phage Ccr29, complete genome
1−1tRNA−Trp(cca)[52818,52893]
gtcggtctagctcatggggtagagcggcggtctccaaaaccgcgcgtggc
aggttcgagtcctgcgaccggcgcca
1−2tRNA−Gly(gcc)[52899,52973]
gcgtccatcgtatatgggttattgcttcgcgttgccaacgcgacgaaccg
ggttcgagtcccggtggacgctcca
1−3tRNA−Gly(tcc)[52980,53053]
gcgggtatagctcaaagggagagctactgccttccaagcagaagatgcgg
gttcgagccccgctatccgctcca
1−4tRNA−Glu(ttc)[53210,53287]
ggcccggtcgtctagcggtttgcaggattcgtgactttcaatcacgagag
cggagttcgagtctccgtcgggcctcca
1−5tRNA−Asp(gtc)[53309,53383]
ggtgcgttcgtatatcggtcaatactccggattgtcgatccggcaagagg
ggttcgattcccctacgcaccgcca
1−6tRNA−Pro(cgg)[54253,54328]
cggggtgtagctcagtcggtagagcgctcggttcgggaccgagaggccgt
gggttcgagtcccgccacccagacca
1−7tRNA−Pro(ggg)[54336,54410]
cgggatgtagctcagcggtagagcgcgcggctgggggccgcgaggccgca
ggttcgagacctgccaccccgacca
1−8tRNA−Thr(ggt)[54416,54488]
gctgctgcgcctggatcggcgccggccccttggtatggggaagtagaagg
ttcaattccttccagcagcacca
1−9tRNA−Lys(ctt)[54601,54687]
ggatgtgtcacctcagtggcgagaggaccgggctcttaatccggcagcga
aagctcatcgtgggttcgagtcccaccacatcctcca
1−10tRNA−Lys(ttt)[54693,54783]
gcacgagtgacccgagtggcgaagggcgtcgacttttaatagactaggcg
tccgcgcctcaccgagggttcgagtccctcctcgtgctcca
1−11tRNA−Leu(cag)[54790,54873]

gcgaaagtggcggaacggcagacgcactggtctcaggtaccagcgacttc
ggtcatgagggttcgaatccctcctttcgtacca
1-12tRNA-Leu(caa)[54881,54965]
gccggtctggcggaaatggcagacgcagtggcttcaagtcccaccgcctt
cgggcgtcccggttcgaggccggggaccggcacca
1-13tRNA-Leu(gag)[54971,55059]
tgcgaagtggcggaactttggtagacgcaccggcatgagaggccggcgcc
cttcggggcgtgagggttcgagtcccttcttcgcgacca
1-14tRNA-Ala(tgc)[55143,55213]
ggggtatgcgcagggcgcaggcatcctttgcaaggaagtcggggtgggtt
cgagtcccacatactccacca
1-15tRNA-Ser(cga)[55218,55305]
ggtaggttggctgagaggccgaaggcgcccgtttcgaaaacgggaggacc
gcaaggttccgtgggttcgaatcccacacctacctcca
1-16tRNA-Ser(gga)[55310,55397]
ggatgtatggctgagaggccgaaggcgcccgcttggaaagcgggtgaacc
gcaaggttccgtaggttcgaatcctactgcatccgcca
1-17tRNA-Phe(gaa)[56092,56167]
ggactcgtagctcagcaggtagagcgccggcttgaaaccccggaggtcgg
aggttcgactcctcccgtttccacca
1-18tRNA-Val(gac)[56173,56248]
gcccgatgagccagatgggaaggcgccgccgcgacatggcgggattgaga
aggttcgattccttcatcaggcacca
1-19tRNA-Ile(gat)[56251,56326]
gcaagtgtagctcagttggtagagcagcggaccgataatccgcccgtccc
tggttcgagtccaggcatttgcacca
1-20tRNA-Ile(gat)[56334,56408]
tgtggcatagctcaggggtagagcgcgcgcttgataagcgtgaggacggg
ggttcgaagccctctgccactacca
1-21tRNA-Val(cac)[56442,56517]
tggcgtttagcatagtgggagtgcaggggtgtcacatacctcgatcgaac
tggttcgattccagtaacgccaacca
1-22tRNA-Gln(ctg)[56523,56596]
tgagggttcgtctaacggtaggacggctgggtctggtccagctaatctag
gttcgagtcctagcccctcatcca
1-23tRNA-Gln(ttg)[56605,56680]
tcagggttagtttagctggtaaaacactgggttttggccccggcgtcgct
aggttcgaagcctgcaccctgatcca
1-24tRNA-Ile(aat)[56692,56766]
tccagtcaatgcacaaggtgtgcagcggcactgttaatgcctgtgagccc
ggttcgattccggggtctggagcca
1-25tRNA-Arg(tct)[56969,57046]
gcgcaggtagctcaacggacccagagcgaccgtcttctacacggagggtt
gggggttcgaatccctccctgcgcgcca

1–26tRNA–Cys(gca)c[106374,106462]
gcttcggtggactgagaggttaggcgcgggattgcaaatctctgctggtg
ggaacacctatgagggttcgagtccctcccgaagctcca
1–27tRNA–Gly(tcc)c[107042,107115]
tttgcgaatagcacagtggtagtgcaacagccgtcctagctgtcggtcgc
gggttcaatccccgcttcgatgaa
1–29tRNA–Met(cat)[108188,108266]
cgcggggtagaggagtccggttgtcctcgtctggctcataaccaggagat
cgtgggttcaaatcccactcccgctccca
1–30tRNA–Met(cat)[108638,108722]
ggtacggtggctcgacaggtaaggcatcggcctcataagccgagggttcg
tcccaatcctggttcaaatccaggtcgtacctcca
>KY555146.1 Caulobacter phage Ccr32, complete genome
1–1tRNA–Trp(cca)[47830,47905]
gtcggtctagctcatggggtagagcggcggtctccaaaaccgcgcgtggc
aggttcgagtcctgcgaccggcgcca
1–2tRNA–Gly(gcc)[47911,47985]
gcgtccatcgtatatgggttattgcttcgcgttgccaacgcgacgaaccg
ggttcgagtcccggtggacgctcca
1–3tRNA–Gly(tcc)[47992,48065]
gcggatatagctcaaagggagagctactgccttccaagcagaagatgcgg
gttcgagccccgctatccgctcca
1–4tRNA–Glu(ttc)[48222,48299]
ggcccggtcgtctagcggtttgcaggattcgtgactttcaatcacgagag
cggagttcgagtctccgtcgggcctcca
1–5tRNA–Asp(gtc)[48322,48396]
ggtgcgttcgtatatcggtcaatactccggattgtcgatccggcaagagg
ggttcgattcccctacgcaccgcca
1–6tRNA–Pro(cgg)[49836,49911]
cggggtgtagctcagtcggtagagcgctcggttcgggaccgagaggccgt
gggttcgagtcccgccacccagacca
1–7tRNA–Pro(ggg)[49919,49993]
cgggatgtagctcagcggtagagcgcgcggctgggggccgcgaggccgca
ggttcgagacctgccaccccgacca
1–8tRNA–Lys(ctt)[50184,50270]
ggatgtgtcacctcagtggcgagaggaccgggctcttaatccggcagcga
aagctcatcgtgggttcgagtcccaccacatcctcca
1–9tRNA–Lys(ttt)[50277,50367]
gcacgagtgacccgagcggcgaagggcgtcgacttttaatcgactaggcg
tccgcgcctcaccgagggttcgagtccctcctcgtgctcca
1–10tRNA–Leu(cag)[50374,50457]
gcgaaagtggcggaacggcagacgcactggtctcaggtaccagcgacttc
ggtcatgagggttcgaatccctcctttcgtacca
1–11tRNA–Leu(caa)[50465,50549]

gccggtctggcggaaatggcagacgcagtggcttcaagtcccaccgcctt
cgggcgtcccggttcgaggccggggaccggcacca
1-12tRNA-Leu(gag)[50555,50643]
tgcgaagtggcggaactttggtagacgcaccggcatgagaggccggcgcc
cttcggggcgtgagggttcgagtcccttcttcgcgacca
1-13tRNA-Ala(tgc)[50727,50797]
ggggtatgcgcagggcgcaggcatcctttgcaaggaagtcggggtgggtt
cgagtcccacatactccacca
1-14tRNA-Ser(cga)[50802,50889]
ggtaggttggctgagaggccgaaggcgcccgtttcgaaaacgggaggacc
gcaaggttccgtgggttcgaatcccacacctacctcca
1-15tRNA-Ser(gga)[50894,50981]
ggatgtatggctgagaggccgaaggcgcccgcttggaaagcgggtgaacc
gcaaggttccgtaggttcgaatcctactgcatccgcca
1-16tRNA-Phe(gaa)[51675,51750]
ggactcgtagctcagcaggtagagcgccggcttgaaaccccggaggtcgg
aggttcgattcctcccgtttccacca
1-17tRNA-Val(gac)[51756,51831]
gcccgatgagccagatgggaaggcgccgccgtgacatggcgggattgaga
aggttcgattccttcatcaggcacca
1-18tRNA-Ile(gat)[51834,51909]
gcaagtgtagctcagtcggtagagcagcggaccgataatccgcctgtccc
tggttcgagtccaggcatttgcacca
1-19tRNA-Ile(gat)[51917,51991]
tgtggcatagctcaggggtagagcgcgcgcttgataagcgtgaggtcggg
ggttcgaagccctctgccactacca
1-20tRNA-Val(cac)[52025,52100]
tggcgtttagcatagtgggagtgcaggggtgtcacatacctcgatcgaac
tggttcgattccagtaacgccaacca
1-21tRNA-Gln(ctg)[52106,52179]
tgagggttcgtctaacggtaggacggctgggtctggtccagctaatctag
gttcgagtcctagcccctcatcca
1-22tRNA-Gln(ttg)[52187,52260]
tctcgggtggtctaatggtaagacatctggttttggtccagaggattggg
gttcgattccttggccgagaacca
1-23tRNA-Ile(aat)[52276,52350]
tccagtcaatgcacaaggtgtgcagcggcactgttaatgcctgtgagccc
ggttcgattccggggtctggagcca
1-24tRNA-Gly(tcc)[52553,52630]
gcgcgggtagctcaatggacccagagcagccgtcttccacacggaaggtt
gggggttcgaatccctccccgtgcgcca
1-26tRNA-Cys(gca)c[102013,102101]
gcttcggtggactgagaggttaggcgcgggattgcaaatctctgctggtg
ggaacacctatgagggttcgagtccctcccgaagctcca

1-27tRNA-Gly(tcc)c[102681,102754]
tttgcgaatagcacagtggtagtgcaacagccgtcctagctgtcggtcgc
gggttcaatccccgcttcgatgaa
1-29tRNA-Met(cat)[103827,103905]
cgcggggtagaggagtccggttgtcctcgtctggctcataaccaggagat
cgtgggttcaaatcccactcccgctccca
1-30tRNA-Met(cat)[104272,104356]
ggtacggtggctcgacaggtaaggcatcggcctcataagccgagggttcg
tcccaatcctggttcaaatccaggtcgtacctcca
>KY555147.1 Caulobacter phage Ccr34, complete genome
1-1tRNA-Trp(cca)[48270,48345]
gtcggtctagctcatggggtagagcggcggtctccaaaaccgcgcgtggc
aggttcgagtcctgcgaccggcgcca
1-2tRNA-Gly(gcc)[48351,48425]
gcgtccatcgtatatgggttattgcttcgcgttgccaacgcgacgaaccg
ggttcgagtcccggtggacgctcca
1-3tRNA-Gly(tcc)[48432,48505]
gcggatatagctcaaagggagagctactgccttccaagcagaagatgcgg
gttcgagccccgctatccgctcca
1-4tRNA-Glu(ttc)[48662,48739]
ggcccggtcgtctagcggtttgcaggattcgtgactttcaatcacgagag
cggagttcgagtctccgtcgggcctcca
1-5tRNA-Asp(gtc)[48762,48836]
ggtgcgttcgtatatcggtcaatactccggattgtcgatccggcaagagg
ggttcgattcccctacgcaccgcca
1-6tRNA-Pro(cgg)[50276,50351]
cggggtgtagctcagtcggtagagcgctcggttcgggaccgagaggccgt
gggttcgagtcccgccacccagacca
1-7tRNA-Pro(ggg)[50359,50433]
cgggatgtagctcagcggtagagcgcgcggctgggggccgcgaggccgca
ggttcgagacctgccaccccgacca
1-8tRNA-Lys(ctt)[50624,50710]
ggatgtgtcacctcagtggcgagaggaccgggctcttaatccggcagcga
aagctcatcgtgggttcgagtcccaccacatcctcca
1-9tRNA-Lys(ttt)[50717,50807]
gcacgagtgacccgagcggcgaagggcgtcgacttttaatcgactaggcg
tccgcgcctcaccgagggttcgagtccctcctcgtgctcca
1-10tRNA-Leu(cag)[50814,50897]
gcgaaagtggcggaacggcagacgcactggtctcaggtaccagcgacttc
ggtcatgagggttcgaatccctcctttcgtacca
1-11tRNA-Leu(caa)[50905,50989]
gccggtctggcggaaatggcagacgcagtggcttcaagtcccaccgcctt
cgggcgtcccggttcgaggccggggaccggcacca
1-12tRNA-Leu(gag)[50995,51083]

tgcgaagtggcggaactttggtagacgcaccggcatgagaggccggcgcc
cttcggggcgtgagggttcgagtcccttcttcgcgacca
1-13tRNA-Ala(tgc)[51167,51237]
ggggtatgcgcagggcgcaggcatcctttgcaaggaagtcggggtgggtt
cgagtcccacatactccacca
1-14tRNA-Ser(cga)[51242,51329]
ggtaggttggctgagaggccgaaggcgcccgtttcgaaaacgggaggacc
gcaaggttccgtgggttcgaatcccacacctacctcca
1-15tRNA-Ser(gga)[51334,51421]
ggatgtatggctgagaggccgaaggcgcccgcttggaaagcgggtgaacc
gcaaggttccgtaggttcgaatcctactgcatccgcca
1-16tRNA-Phe(gaa)[52115,52190]
ggactcgtagctcagcaggtagagcgccggcttgaaaccccggaggtcgg
aggttcgattcctcccgtttccacca
1-17tRNA-Val(gac)[52196,52271]
gcccgatgagccagatgggaaggcgccgccgtgacatggcgggattgaga
aggttcgattccttcatcaggcacca
1-18tRNA-Ile(gat)[52274,52349]
gcaagtgtagctcagtcggtagagcagcggaccgataatccgcctgtccc
tggttcgagtccaggcatttgcacca
1-19tRNA-Ile(gat)[52357,52431]
tgtggcatagctcaggggtagagcgcgcgcttgataagcgtgaggtcggg
ggttcgaagccctctgccactacca
1-20tRNA-Val(cac)[52465,52540]
tggcgtttagcatagtgggagtgcaggggtgtcacatacctcgatcgaac
tggttcgattccagtaacgccaacca
1-21tRNA-Gln(ctg)[52546,52619]
tgagggttcgtctaacggtaggacggctgggtctggtccagctaatctag
gttcgagtcctagcccctcatcca
1-22tRNA-Gln(ttg)[52627,52700]
tctcgggtggtctaatggtaagacatctggttttggtccagaggattggg
gttcgattccttggccgagaacca
1-23tRNA-Ile(aat)[52716,52790]
tccagtcaatgcacaaggtgtgcagcggcactgttaatgcctgtgagccc
ggttcgattccggggtctggagcca
1-24tRNA-Gly(tcc)[52993,53070]
gcgcgggtagctcaatggacccagagcagccgtcttccacacggaaggtt
gggggttcgaatccctccccgtgcgcca
1-26tRNA-Cys(gca)c[102453,102541]
gcttcggtggactgagaggttaggcgcgggattgcaaatctctgctggtg
ggaacacctatgagggttcgagtccctcccgaagctcca
1-27tRNA-Gly(tcc)c[103121,103194]
tttgcgaatagcacagtggtagtgcaacagccgtcctagctgtcggtcgc
gggttcaatccccgcttcgatgaa

1–29tRNA–Met(cat)[104267,104345]
cgcggggtagaggagtccggttgtcctcgtctggctcataaccaggagat
cgtgggttcaaatcccactcccgctccca
1–30tRNA–Met(cat)[104712,104796]
ggtacggtggctcgacaggtaaggcatcggcctcataagccgagggttcg
tcccaatcctggttcaaatccaggtcgtacctcca
>KY555144.1 Caulobacter phage Ccr5, complete genome
1–1tRNA–Trp(cca)[47682,47757]
gtcggtctagctcatggggtagagcggcggtctccaaaaccgcgcgtggc
aggttcgagtcctgcgaccggcgcca
1–2tRNA–Gly(gcc)[47763,47837]
gcgtccatcgtatatgggttattgcttcgcgttgccaacgcgacgaaccg
ggttcgagtcccggtggacgctcca
1–3tRNA–Gly(tcc)[47844,47917]
gcggatatagctcaaagggagagctactgccttccaagcagaagatgcgg
gttcgagccccgctatccgctcca
1–4tRNA–Glu(ttc)[48074,48151]
ggcccggtcgtctagcggtttgcaggattcgtgactttcaatcacgagag
cggagttcgagtctccgtcgggcctcca
1–5tRNA–Asp(gtc)[48175,48249]
ggtgcgttcgtatatcggtcaatactccggattgtcgatccggcaagagg
ggttcgattcccctacgcaccgcca
1–6tRNA–Pro(cgg)[49110,49185]
cggggtgtagctcagtcggtagagcgctcggttcgggaccgagaggccgt
gggttcgagtcccgccacccagacca
1–7tRNA–Pro(ggg)[49193,49267]
cgggatgtagctcagcggtagagcgcgcggctgggggccgcgaggccgca
ggttcgagacctgccaccccgacca
1–8tRNA–Lys(ctt)[49456,49542]
ggatgtgtcacctcagtggcgagaggaccgggctcttaatccggcagcga
aagctcatcgtgggttcgagtcccaccacatcctcca
1–9tRNA–Lys(ttt)[49548,49637]
gcacgagtgacccgagcggcgaagggcgtcgacttttaatcgactgggcg
tccagcctcaccgagggttcgagtccctcctcgtgctcca
1–10tRNA–Leu(cag)[49644,49729]
gcgaaagtggcggaacaaggtagacgcaccggtctcaggtaccggcggct
tcggtcatgagggttcgagtccctcctttcgtacca
1–11tRNA–Leu(caa)[49737,49821]
gccggtctggcggaaatggcagacgcagtggcttcaagtcccaccgcctt
cgggcgtcccggttcgatgccggggaccggcacca
1–12tRNA–Leu(gag)[49828,49915]
tgcgaagtggcggaatttggtagacgcaccggcatgagaggccggcgccc
ttcggggcgtgagggttcgagtcccttcttcgcgacca
1–13tRNA–Ala(tgc)[50006,50076]

ggggtatgcgcagggcgcaggcatcctttgcaaggaagtcggggtgggtt
cgagtcccacatactccacca
1–14tRNA–Ser(cga)[50081,50168]
ggtaggttggctgagaggccgaaggcgcccgtttcgaaaacgggagaact
gcaaggttccgtgggttcgaatcccacacctacctcca
1–15tRNA–Ser(gga)[50173,50260]
ggatgtatggctgagaggccgaaggcgcccgcttggaaagcgggtgaacc
gcaaggttccgtaggttcgaatcctactacatccgcca
1–16tRNA–Phe(gaa)[50956,51031]
ggactcgtagctcagcaggtagagcgccggcttgaaaccccggaggtcgg
aggttcgattcctcccgtttccacca
1–17tRNA–Val(gac)[51037,51112]
gcccgatgagccagatgggaaggcgccgccgtgacatggcgggattgaga
aggttcgattccttcatcaggcacca
1–18tRNA–Ile(gat)[51115,51190]
gcaagtgtagctcagtcggtagagcagcggaccgataatccgcccgtccc
tggttcgagtccaggcatttgcacca
1–19tRNA–Ile(gat)[51198,51272]
tgtggcatagctcaggggtagagcgcgcgcttgataagcgtgaggtcggg
ggttcgaagccctctgccactacca
1–20tRNA–Val(cac)[51306,51381]
tggcgtttagcatagtgggagtgcaggggtgtcacatacctcgatcgaac
tggttcgattccagtaacgccaacca
1–21tRNA–Gln(ctg)[51387,51460]
tgagggttcgtctaatggtaggacggctgggtctggtccagctaatctag
gttcgagtcctagcccctcatcca
1–22tRNA–Gln(ttg)[51468,51541]
tctcgggtggtctaatggtaagacatctggttttggtccagaggattggg
gttcgattccttggccgagaacca
1–23tRNA–Ile(aat)[51557,51631]
tccagtcaatgcacaaggtgtgcagcggcactgttaatgcctgtgagccc
ggttcgattccggggtctggagcca
1–24tRNA–Arg(tct)[51834,51911]
gcgcgggtagctcaatggacccagagcagccgtcttctacacggtgggtt
gggggttcgaatccctccccgtgcgcca
1–25tRNA–Cys(gca)c[101727,101815]
gcttcggtggactgagaggttaggcgcgggattgcaaatctctgctggtg
ggaacacctatgagggttcgagtccctcccgaagctcca
1–26tRNA–Lys(ctt)c[102395,102468]
tttgcgaatagcacagtggtagtgcaacagcctcttaagctgtcggtcgc
gggttcaatccccgcttcgatgaa
1–28tRNA–Met(cat)[103541,103619]
cgcggggtagaggagtccggttgtcctcgtctggctcataaccaggagat
cgtgggttcaaatcccactcccgctccca

1−29tRNA−Met(cat)[103986,104070]
ggtacggtggctcgacaggtaaggcatcggcctcataagccgagggttcg
tcccaatcctggttcaaatccaggtcgtacctcca
>KY619305.1 Escherichia phage vB_EcoS_ESCO41, complete genome
1−1tRNA−Arg(tct)[7289,7366]
gctccgctagctcaattggaaagaagcaacgaccttctaagtcgtaggtt
actggttcgaacccagtgcggagcgcca
>KY677846.1 Escherichia phage phiLLS, complete genome
1−1tRNA−Arg(tct)[44136,44210]
cggggtgtagtctaagggagaggcaggagtcttctaaattcctttatgca
ggttcgaatcctgtcacctcggcca
1−2tRNA−Ser(gct)[47745,47833]
ggaagaatagcataacggtattgcagcagattgctaatctgtcggtttga
aatatagccttgtgggttcgattcccgcttcttccgcca
1−3tRNA−Leu(taa)[48133,48209]
gggggtgtaatcgaattggcataggtactggacttaaaattcaggttttg
tgggttcgaatcccaccacccctacca
1−4tRNA−Tyr(gta)[49157,49244]
gggcgtttattccgtaagtggtagcggaggggattgtaaatccctggtca
ttgcgactcgagtggttcgactccattaacgccccacca
1−5tRNA−Glu(ttc)[49252,49326]
gcacctatcgtctagcggttaggacaccactctttcacagtgggaacacg
agttcgaccctcgttgggtgtgcca
1−6tRNA−Phe(gaa)[49413,49487]
gcacctttagctgagatggattagcgcttgcctgaagagcttgagaggtt
cgttcgattcgaacagggtgcacca
1−7tRNA−Cys(gca)[49847,49922]
cgaccgttggctgaatggcttaggcgaaggattgcaaatcctttttatgt
gagttcaaatctcatgcggtcgtcca
1−8tRNA−Asn(gtt)[50439,50516]
ggttccttagctctaattggttagagcggcatcttgttaagttgagggtt
gctggttcgaatccagcaggaaccgcca
1−9tRNA−Asp(gtc)[50814,50890]
gcgaccggggctggcttggtaatggtactcccctgtcacgggagggaatg
tgggttcaaatcccatcggtcgcgcca
1−10tRNA−Pro(tgg)[52062,52139]
ctccgtgtagctcagtttggccagagcgtttcgtttggggcgatagggtc
gggggttcaaatcctcccacggagacca
1−11tRNA−Met(cat)[52146,52223]
tgcgggttagatctctggtagagatcgctagtctcataagctagaaagag
gtaggttcgattcctgcacccgcttcca
1−12tRNA−Lys(ttt)[52413,52491]
agatcgctagctcaataggtttagtagcatccgacttttaatcggaaggt
tctgggttcgagtcccaggcgatctacca

1-13tRNA-Ala(tgc)[52872,52948]
ggggaatgggtctgcatggagtggacacctcgcttgcaccgaggacatca
gaacggttcgattccgttattctccac
1-14tRNA-Leu(tag)[52956,53032]
gcgtgattgatggaattggcatacataccgtccttagaagtcgggttttg
agggttcgaatcccttgtcacgcacca
1-15tRNA-His(gtg)[53708,53784]
gtggctatatcataattggttaatgatcctgattgtgaatcaggcctatg
tggattcgaattccactagccacccca
1-16tRNA-Gln(ctg)[54018,54093]
tgggatgtagatcaattggcagatcgtcggcctctgactccgaaggttcc
acgttcgatccgtggcatcccagcca
1-17tRNA-Gln(ttg)[54100,54175]
tggagagtagtgtaacggttagcacaacggcctttgactccgttaatggt
aggttcgattcctccttctccagcca
1-18tRNA-Gly(tcc)[54746,54817]
gcggttagcctcatggtggggactcagccttccaagctgatggagagggt
tcgattccctctagccgctcca
1-19tRNA-Thr(tgt)[55253,55327]
gctcctaaagcattgctggcgatgcagttgccttgtaagcatctgaaccg
ggttcgattcctggtgggagcacca
1-20tRNA-Ile(gat)[55708,55784]
gcttcggtagcttagcgatctaaagcactcggccgataaccgagagatcg
ggggtttaaatccctcccggagtacca
1-21tRNA-Met(cat)[55881,55956]
agttagttggcagagtggttatgcacctccttcatacggagcgactacag
tggttcaaatccactactaactacca

>KY554768.1 Lactococcus phage AM1, complete genome

1-1tRNA-Arg(tct)[46773,46845]
gttcggttagtgtaattggataacacacataatttctaattatgtactct
ggattcgagttccagactgagca
1-3tRNA-Asp(gtc)[47568,47638]
ggcttattagtatatgggtactacacatccctgtcacggaggagaaccga
gttcgagtctcggataagccg
1-4tRNA-Pro(tgg)[47987,48058]
caggatatagtgttaatggtagcatgcgtgttttgggaacatgtagtgtt
ggttcgagtccagctatcctga
1-5tRNA-Trp(cca)[49122,49197]
ttggttggtagctcagttggtagtagcactagtctccaaaactaggtgtc
gtgagttcgaatctcacccaatcagt
1-6tRNA-Trp(cca)[49372,49443]
atccgtttagtgtagtggttagcacatcagattccaaacctgataacgtg
ggttcgattcctacaatggata

>KY554775.1 Lactococcus phage AM11, complete genome

1-1tRNA-Arg(tct)[46546,46618]
gttcggttagtgtaattggataacacacataatttctaattatgtactct
ggattcgagttccagactgagca
1-3tRNA-Asp(gtc)[47341,47411]
ggcttattagtatatgggtactacacatccctgtcacggaggagaaccga
gttcgagtctcggataagccg
1-4tRNA-Pro(tgg)[47760,47831]
caggatatagtgttaatggtagcatgcgtgttttgggaacatgtagtgtt
ggttcgagtccagctatcctga
1-5tRNA-Trp(cca)[48895,48970]
ttggttggtagctcagttggtagtagcactagtctccaaaactaggtgtc
gtgagttcgaatctcacccaatcagt
1-6tRNA-Trp(cca)[49145,49216]
atccgtttagtgtagtggttagcacatcagattccaaacctgataacgtg
ggttcgattcctacaatggata
>KY554776.1 Lactococcus phage AM12, complete genome
1-1tRNA-Arg(tct)[46527,46599]
gttcggttagtgtaattggataacacacataatttctaattatgtactct
ggattcgagttccagactgagca
1-3tRNA-Asp(gtc)[47322,47392]
ggcttattagtatatgggtactacacatccctgtcacggaggagaaccga
gttcgagtctcggataagccg
1-4tRNA-Pro(tgg)[47741,47812]
caggatatagtgttaatggtagcatgcgtgttttgggaacatgtagtgtt
ggttcgagtccagctatcctga
1-5tRNA-Trp(cca)[48876,48951]
ttggttggtagctcagttggtagtagcactagtctccaaaactaggtgtc
gtgagttcgaatctcacccaatcagt
1-6tRNA-Trp(cca)[49126,49197]
atccgtttagtgtagtggttagcacatcagattccaaacctgataacgtg
ggttcgattcctacaatggata
>KY554769.1 Lactococcus phage AM2, complete genome
1-1tRNA-Arg(tct)[46770,46842]
gttcggttagtgtaattggataacacacataatttctaattatgtactct
ggattcgagttccagactgagca
1-3tRNA-Asp(gtc)[47565,47635]
ggcttattagtatatgggtactacacatccctgtcacggaggagaaccga
gttcgagtctcggataagccg
1-4tRNA-Pro(tgg)[47984,48055]
caggatatagtgttaatggtagcatgcgtgttttgggaacatgtagtgtt
ggttcgagtccagctatcctga
1-5tRNA-Trp(cca)[49119,49194]
ttggttggtagctcagttggtagtagcactagtctccaaaactaggtgtc
gtgagttcgaatctcacccaatcagt

1−6tRNA−Trp(cca)[49369,49440]
atccgtttagtgtagtggttagcacatcagattccaaacctgataacgtg
ggttcgattcctacaatggata
>KY554770.1 Lactococcus phage AM3, complete genome
1−1tRNA−Arg(tct)[47147,47219]
gttcggttagtgtaattggataacacacataatttctaattatgtactct
ggattcgagttccagactgagca
1−3tRNA−Asp(gtc)[47942,48012]
ggcttattagtatatgggtactacacatccctgtcacggaggagaaccga
gttcgagtctcggataagccg
1−4tRNA−Pro(tgg)[48361,48432]
caggatatagtgttaatggtagcatgcgtgttttgggaacatgtagtgtt
ggttcgagtccagctatcctga
1−5tRNA−Trp(cca)[49496,49571]
ttggttggtagctcagttggtagtagcactagtctccaaaactaggtgtc
gtgagttcgaatctcacccaatcagt
1−6tRNA−Trp(cca)[49746,49817]
atccgtttagtgtagtggttagcacatcagattccaaacctgataacgtg
ggttcgattcctacaatggata
>KY554771.1 Lactococcus phage AM4, complete genome
1−1tRNA−Met(cat)[29760,29832]
ggttctatagcttagatggtaaagtcctctgcccataacagagttaacgc
aggttcaagccctgctagaacca
1−2tRNA−Arg(tct)[31584,31657]
gttcggttagctcaattggatagagtattcgccttctaagcgaaaggtta
tgagttcaagcctcatactgaaca
1−4tRNA−Pro(tgg)[32560,32631]
caggatatagtgttaatggtagcatgcgtgttttgggaacatgtagtgct
ggttcgagtccagctatcctga
>KY554772.1 Lactococcus phage AM5, complete genome
1−1tRNA−Met(cat)[47715,47787]
ggttctatagcttagatggtaaagtcctctgcccataacagagttaacgc
aggttcaagccctgctagaacca
1−2tRNA−Arg(tct)[49539,49612]
gttcggttagctcaattggatagagtattcgccttctaagcgaaaggtta
tgagttcaagcctcatactgaaca
1−4tRNA−Pro(tgg)[50515,50586]
caggatatagtgttaatggtagcatgcgtgttttgggaacatgtagtgct
ggttcgagtccagctatcctga
>KY554766.1 Lactococcus phage AM6, complete genome
1−1tRNA−Pro(tgg)c[61438,61511]
caggatatagctcagcttggtagagcactgcttttgggaagcaggggtca
gaggttcaaatccttttatcttga
1−2tRNA−Thr(tgt)c[61576,61649]

gccgatttagctcagtttggtagagcgtctgatttgtaatcagagggtca
caggttcaagtcctgtagtcggca
1-3tRNA-Asn(gtt)c[61705,61777]
tgacccatagctcagttggtagagcgtcgcactgttaatgcgaatgtcgt
aggttcgagtcctactgggtcag
1-4tRNA-Cys(gca)c[61883,61955]
ctccctgtggccgagtggtttaggtgcagctctgcaaaagctgatacgtt
ggttcgaatccgactagggagtc
>KY554767.1 Lactococcus phage AM7, complete genome
1-1tRNA-Pro(tgg)c[61636,61709]
caggatatagctcagcttggtagagcactgcttttgggaagcaggggtca
gaggttcaaatccttttatcttga
1-2tRNA-Thr(tgt)c[61774,61847]
gccgatttagctcagtttggtagagcgtctgatttgtaatcagagggtca
caggttcaagtcctgtagtcggca
1-3tRNA-Asn(gtt)c[61903,61975]
tgacccatagctcagttggtagagcgtcgcactgttaatgcgaatgtcgt
aggttcgagtcctactgggtcag
1-4tRNA-Cys(gca)c[62081,62153]
ctccctgtggccgagtggtttaggtgcagctctgcaaaagctgatacgtt
ggttcgaatccgactagggagtc
>KY554773.1 Lactococcus phage AM8, complete genome
1-1tRNA-Arg(tct)[46543,46615]
gttcggttagtgtaattggataacacacataatttctaattatgtactct
ggattcgagttccagactgagca
1-3tRNA-Asp(gtc)[47338,47408]
ggcttattagtatatgggtactacacatccctgtcacggaggagaaccga
gttcgagtctcggataagccg
1-4tRNA-Pro(tgg)[47757,47828]
caggatatagtgttaatggtagcatgcgtgttttgggaacatgtagtgtt
ggttcgagtccagctatcctga
1-5tRNA-Trp(cca)[48892,48967]
ttggttggtagctcagttggtagtagcactagtctccaaaactaggtgtc
gtgagttcgaatctcacccaatcagt
1-6tRNA-Trp(cca)[49142,49213]
atccgtttagtgtagtggttagcacatcagattccaaacctgataacgtg
ggttcgattcctacaatggata
>KY554774.1 Lactococcus phage AM9, complete genome
1-1tRNA-Arg(tct)[46544,46616]
gttcggttagtgtaattggataacacacataatttctaattatgtactct
ggattcgagttccagactgagca
1-3tRNA-Asp(gtc)[47339,47409]
ggcttattagtatatgggtactacacatccctgtcacggaggagaaccga
gttcgagtctcggataagccg

1-4tRNA-Pro(tgg)[47758,47829]
caggatatagtgttaatggtagcatgcgtgttttgggaacatgtagtgtt
ggttcgagtccagctatcctga
1-5tRNA-Trp(cca)[48893,48968]
ttggttggtagctcagttggtagtagcactagtctccaaaactaggtgtc
gtgagttcgaatctcacccaatcagt
1-6tRNA-Trp(cca)[49143,49214]
atccgtttagtgtagtggttagcacatcagattccaaacctgataacgtg
ggttcgattcctacaatggata
>KY554762.1 Lactococcus phage LW31, complete genome
1-1tRNA-Thr(tgt)c[60012,60085]
gccgatttagctcagtttggtagagcgtctgatttgtaatcagagggtca
caggttcaagtcctgtagtcggca
1-2tRNA-Asn(gtt)c[60142,60214]
tgacccatagctcagttggtagagcgtcgcactgttaatgcgaatgtcgt
aggttcgagtcctactgggccag
1-3tRNA-Cys(gca)c[60381,60452]
ggtcttatagtgaagtggctaacacaggagactgcaaatctctaatcgtc
ggttcaaatccgactaggacct
>KY554763.1 Lactococcus phage LW32, complete genome
1-1tRNA-Thr(tgt)c[59622,59695]
gccgatttagctcagtttggtagagcgtctgatttgtaatcagagggtca
caggttcaagtcctgtagtcggca
1-2tRNA-Asn(gtt)c[59752,59824]
tgacccatagctcagttggtagagcgtcgcactgttaatgcgaatgtcgt
aggttcgagtcctactgggccag
1-3tRNA-Cys(gca)c[59991,60062]
ggtcttatagtgaagtggctaacacaggagactgcaaatctctaatcgtc
ggttcaaatccgactaggacct
>KY554764.1 Lactococcus phage LW33, complete genome
1-1tRNA-Thr(tgt)c[59359,59432]
gccgatttagctcagtttggtagagcgtctgatttgtaatcagagggtca
caggttcaagtcctgtagtcggca
1-2tRNA-Asn(gtt)c[59489,59561]
tgacccatagctcagttggtagagcgtcgcactgttaatgcgaatgtcgt
aggttcgagtcctactgggccag
1-3tRNA-Cys(gca)c[59728,59799]
ggtcttatagtgaagtggctaacacaggagactgcaaatctctaatcgtc
ggttcaaatccgactaggacct
>KY554765.1 Lactococcus phage LW4, complete genome
1-1tRNA-Thr(tgt)c[59678,59751]
gccgatttagctcagtttggtagagcgtctgatttgtaatcagagggtca
caggttcaagtcctgtagtcggca
1-2tRNA-Asn(gtt)c[59808,59880]

tgacccatagctcagttggtagagcgtcgcactgttaatgcgaatgtcgt
aggttcgagtcctactgggccag
1-3tRNA-Cys(gca)c[60047,60118]
ggtcttatagtgaagtggctaacacaggagactgcaaatctctaatcgtc
ggttcaaatccgactaggacct
>KY554777.1 Lactococcus phage LW81, complete genome
1-1tRNA-Met(cat)[49462,49534]
ggttctatagcttagatggtaaagtcctctgcccataacagagttaacgc
aggttcaagccctgctagaacca
1-2tRNA-Arg(tct)[52085,52158]
gttcggttagctcaattggatagagtattcgccttctaagcgaaaggtta
tgagttcaagcctcatactgaaca
1-4tRNA-Asp(gtc)[53062,53134]
ggtttgttggtgtagtggttatcacgcttgcctgtcacgcaagagaacac
gggttcgaatcccgtacaaatcg
1-5tRNA-Trp(cca)[54119,54194]
ttggttggtagctcagttggtagtagcactagtctccaaaactaggtgtc
gtgagttcgaatctcacccaatcagt
1-6tRNA-Trp(cca)[54369,54440]
atccgtttagtgtagtggttagcacatcagattccaaacctgataacgtg
ggttcgattcctacaatggata
1-7tRNA-Met(cat)[54660,54733]
tacggggtgggtggcaaggagtcacattagtttcataggctaaataaggg
cggttcgattccgtcaccccgaat
>KY554760.1 Lactococcus phage R3.4, complete genome
1-1tRNA-Trp(cca)[26909,26981]
tgcgagcatagtatagtggtaatgctacagattccaaacctgtaaacgtg
ggttcgattcctactgttcgtgt
1-2tRNA-Pro(tgg)[27035,27106]
caggatatggtgtcaatggtagcatacgtgttttgggaacatgtggtgtt
ggttcgagtccagctatcctga
>KY554761.1 Lactococcus phage R31, complete genome
1-1tRNA-Trp(cca)[26702,26774]
tgcgagcatagtatagtggtaatgctacagattccaaacctgtaaacgtg
ggttcgattcctactgttcgtgt
1-2tRNA-Pro(tgg)[26828,26899]
caggatatggtgtcaatggtagcatacgtgttttgggaacatgtggtgtt
ggttcgagtccagctatcctga
>KY783914.1 Mycobacterium phage GardenSalsa, complete genome
1-1tRNA-Trp(cca)[57182,57253]
atgggcgtagctgacaggaaagcaacggtctccaaagccgtgacacgcag
gttcgaatcctgccgcccgtgc
1-2tRNA-Asn(gtt)[57783,57858]
tggggtatagccacaatggcagtggcaccgggctgttaactcggaagagt

gttggttcgagtccagctaccctagc
1-3tRNA-Arg(tcg)[58707,58778]
cggatagtcgtccagcgggggcggtgggtttttcgcgagcccacgagcag
gttcgaatcctgactttccggc
1-4tRNA-Tyr(gta)[58851,58936]
ctcgtctctctcctattggtgaagtgatctcaggctgtaaccctgagccc
tgcggggtgtggtggttcgaatccatcaggcgagac
1-5tRNA-Gln(ctg)[59225,59296]
tgtcttctcgactaacggcaagtcaccaggctctgaccctggcaattgag
gttcgaatccttgggggacaac
1-6tRNA-Pro(tgg)[59302,59373]
cgggatatggtgtaagggtagcgtaccggcgttggagaccggaagacatg
gttcgagtccatgtatcccgac
1-7tRNA-Phe(gaa)[59818,59890]
cgtgttgtagctcagttggaagagcggcggcttgaaaacccgcaggccca
ggttcggctcctggcatcacggc
1-8tRNA-Met(cat)[60031,60102]
tgcgcggtggtgtaacgggaacatcctggtctcataagtcagggcttcgg
gttcgaatcccggctgcgcaac
1-9tRNA-Arg(acg)[60408,60486]
cccgatgttgagcaacggagcgctcaatgggctacgaacccatattcgca
tgcaggttcgaatcctgtcatcgggtcca
1-10tRNA-His(gtg)[60489,60561]
cctcttgtagctcaatggaagagcagcggtttgtgagaccgcagacgacg
gatcgttaccgtccagggggacc
1-11tRNA-Cys(gca)[60748,60820]
gtcgagatgactgagtggataagtagcggcctgcaaagctgacttacgcc
ggttcgaatccggctctcgattc
1-12tRNA-Leu(tag)[61074,61150]
ggatctgtagcccaaatggaagaggcaacaggcttagaccctgttcagtg
tgggttcgactccctccagattcacca
1-13tRNA-Leu(caa)[61152,61227]
gctcgactaggcaaactaggcaaagccgcaggcctcaaacgccagtgttt
gtgagttcgaatctcacgtcgagtac
1-14tRNA-Lys(ctt)[61231,61305]
gggatgttggtataaacggcaattactgcagactcttaatctgtcaaatc
tgggttcgaatcccaggcgtcccac
1-15tRNA-Ile(gat)[61306,61381]
tgtgatgtcgtccaacggtttagggcaccgatctgatacatcggaaacca
gggttcaactccctgcttcactacca
1-16tRNA-Gly(tcc)[61469,61545]
ggggtattcatctagtctggctcaggatgcgacacttccactgtcgtcac
gagggttcgaatccttcatacctcacc
1-17tRNA-Val(tac)[61592,61663]

tccccactcgtccaatgggagggcaccgcgcttacaacgcgaggacggga
gttcgattctctcgtggggaac
1−18tRNA−Thr(agt)[61724,61801]
gctggatttgctcatgttggcttgagcacctacctagtaagtaggagtac
gtcggttcgattccgacatccagcccca
1−19tRNA−Ala(tgc)[61860,61930]
gggcgcatgttccaaggtggcgaccgacccttgcaaggttggtgggcgag
ttcgattctcgtcgcttccac
1−20tRNA−Asp(gtc)[62008,62083]
cccgtcatcatctagaggcctaggatgccactctgtcgaagtggtcacac
gggttcaaatcccgttggcgggacca
1−21tRNA−Glu(ctc)[62143,62214]
ggttcattggtgtaatggcagcatcgcaggttctcaacctgttggcaggg
gatcgtaacccctatgaactac
>KY705261.1 Streptococcus phage P5652, complete genome
1−1tRNA−Val(aac)[32001,32092]
ggtgaactaggccgtgacaaagaaagtgttaaacgcacactatataaagc
gatttatatgggcttgtgagggttcgactccctcgctcgctg
>KF192053.1 Enterococcus phage IME−EF1, complete genome
1−1tRNA−Trp(cca)c[39478,39550]
tgcgttgtaagtgtaatggctgcacaatggtctccaaaaccattagagag
ggttcgactccttcacaacgtgt
>JF731128.1 Enterococcus phage SAP6, complete genome
1−1tRNA−Trp(cca)c[41169,41240]
gagcggttagtgtaatggttagcacaacagtctccaaaactgttagagag
ggttcgattccttcactgcttg
>KR011064.1 Tsukamurella phage TIN4, complete genome
1−1tRNA−Asn(gtt)[12481,12554]
tggcatatagctcaatcggcagagcttccgactgttaatcggatggttac
aggttcaagtcctgttatgccagc
>KY965063.1 Mycobacterium phage PurpleHaze, complete genome
1−1tRNA−Asn(gtt)[3872,3947]
tgacgtgtagctcaatcggcagagcacccggctgttaaccgggcggttga
aggttcgagtccttccatgtcagcca
1−2tRNA−Trp(cca)[3988,4062]
aggcacgtagctcaattggtcagagcagcggtctccaaagccgccggctg
caggttcgagtcctgccgtgtctgc
1−3tRNA−Leu(cag)[4097,4171]
ggctcggtaggcaaacaggcaaagccgcctgtctcaggaacaggtgcgtg
agggttcgactccctcccgagctac
>JN699628.1 Mycobacterium phage Bongo, complete genome
1−1tRNA−Arg(ccg)[32355,32450]
ctccacgtggcgaatggcaatgtggcagaggggattccggttcccgaggg
gcgggggatctcagacttccgcctgcgttcgactccgtcgtggtgg

1-2tRNA-Trp(cca)[54782,54853]
atgggcgtagctgactgggaagcaacggtctccaaagccgtgatatgcag
gttcgaatcctgccgcccgtgc
1-3tRNA-Asn(gtt)[55903,55976]
tggcctgtcggttaacggcaaaccagcgccctgttaaggcgtgactcctg
gttcgaatccaggtaggccagcca
1-4tRNA-Arg(acg)[56531,56604]
cggaatgtgcgtctaacggcaagacgagctggccacgggcaagctgatac
aggttcgaatcctggctttccggc
1-5tRNA-Tyr(gta)[56609,56686]
cccgtcgcggattggcgaccaaccctgactgtaaatcaggcgcttcggct
ctgcaggttcaactcctgacggcgggac
1-6tRNA-Gln(ctg)[56854,56926]
tgctctctcgtctaactggcaggacaccagaatctgactctggcagttga
ggttcgaatccttggggagcaac
1-7tRNA-Pro(tgg)[56930,57003]
ctgggtgaggtgtaagtggttgcatggccggtttggatccgggcggttcg
aggttcgagtcctcgcacccagac
1-8tRNA-Ser(gct)[57299,57380]
gagtggaaccatctaggtggatggccgcgattgctaatcgtggggcagcg
taacgctgtgtggatcgtgcccactcctctcc
1-9tRNA-Phe(gaa)[57490,57565]
cgggttgtagctcagattggtgagagcgctcggttgaaacccgagaggcc
cctggttcgaatccaggcatcccggc
1-10tRNA-Met(cat)[57653,57724]
tgcgcggtagtgaaacgggatcacaaaggtctcataagcctttattacgg
gttcgaatcccgtctgcgcaac
1-11tRNA-Arg(acg)[58004,58078]
cccgccgcagcacgacggacgtgcacctcgctacgaacgagaaggctgct
ggttcgaatccagtcggtgggtcca
1-12tRNA-His(gtg)[58081,58152]
cctcttgtagctcaatggtagagcgccgttttgtgagaccggtgacctgc
gttcgattcgcagcagggggac
1-13tRNA-Cys(gca)[58158,58230]
ggcgggatggctgagttagataggcggcgggctgcaaccccgttcacgcc
ggggcagatccggctctcgtctc
1-14tRNA-Leu(cag)[58492,58566]
ggctcgctaggcaaattggaaaagccgcctgattcagaatcaggtgtttc
cgagttcgactctcgggcgagctac
1-15tRNA-Lys(ctt)[59586,59659]
gggacgttggtgaaacggctatcaccgcacgctcttaacgtgtcagttct
gggttcgaatcccaggcgtcccac
1-16tRNA-Gly(tcc)[59782,59857]
ggggcattcatctagtcaggctcaggatgcgacgcttccaacgtcgtcac

gagggttcgaatccttcatgcctcac
1-17tRNA-Val(tac)[59862,59934]
tccggattagctcagtggaagagcggcaggtttacaccctgcgcgtgggg
agttcgattctctcatccggaac
1-18tRNA-Thr(agt)[59938,60010]
gccggtatagctcagtggctagagcacctgtctagtaaacaggagaccag
ggttcgattccctgttccggctc
1-19tRNA-Asp(gtc)[60125,60200]
cccgccatcatctagcggcccaggatgccgccctgtcgaggcggtcacac
gggttcaaatcccgttggcgggacca
1-20tRNA-Glu(ctc)[60202,60273]
ggttcattggtgtaacggcagcatgtcaccctctcaaggtgaaggcaggg
gttcaactcccctatgaactgc
>JF937105.1 Mycobacterium phage Rey, complete genome
1-1tRNA-Trp(cca)[57088,57160]
atgggcgtggcttgataggaaagcagcggtctccaaagccgccacacgca
ggttcgaatcctgccgcccgtgc
1-3tRNA-Asn(gtt)[57913,57986]
tggcctgtagcttaattggtagagcagcgagctgttaactcgccaggtga
aggttcgaatccttcctagccagc
1-4tRNA-Ser(gga)[58475,58546]
aaacatttagctcagcagtagagcatctctttggaatagagacggcggcg
gtgcgattccgccaatgtaacc
1-5tRNA-Ala(cgc)[58843,58915]
cggaatgtgcagcctaatggtaaggcggggagttcgcgactcccgatcca
ggttcgagtcctggcgctccggc
1-6tRNA-Tyr(gta)[59005,59082]
ctcgtcgcgaattggcgatcacagctggctgtaaactagctccttcgggt
gtgcaggttcgactcctgacggcgagac
1-7tRNA-Gln(ctg)[59197,59268]
tccaggttggtgtaatggcagcatggcagaatctgactctgccgattgag
gttcgattccttgccctggatc
1-8tRNA-Pro(tgg)[59359,59434]
cagtctgaggtgtaagtggttgcatgaggcgtttggatcgccggggttgg
gagttcgagtctctccagactgacca
1-9tRNA-Phe(gaa)[59833,59908]
cggttcgtagctcagtttggcaagagcgctggcttgaaaccccagaggcc
ccaggttcgactcctggcgttccggc
1-10tRNA-Met(cat)[60048,60120]
tgcgcggtagtgaaatcgggatcacaggagtttcatacgcttccattatg
ggttcgagtcccatctgcgcaac
1-11tRNA-Arg(acg)[60409,60487]
cccgatgttgagcagtggagcgctcaatgggctacgaacccatattcgca
tgcaggttcgaatcctgtcatcgggtcca

1-12tRNA-His(gtg)[60490,60564]
cctcttgtagctcaatcggttagagcagcggtttgtgaagccgcagacca
cggatcgtaaccgtgcagggggacc
1-13tRNA-Cys(gca)[60672,60744]
gtcgagatgacagagtggcgatgtgacggtctgcaaagccgatctacgcc
ggttcgaatccggctctcgattc
1-14tRNA-Leu(tag)[60998,61072]
ggatctgtagcccaaatggaagaggcaacaggcttagaccctgttcagtg
tgggttcgactccctccagattcac
1-15tRNA-Leu(caa)[61076,61151]
gctcgactaggcaaactaggcaaagccgcaggcctcaaaagccagtgttt
gcgagttcgaatctcgcgtcgagtac
1-16tRNA-Lys(ctt)[61189,61260]
gggctcgtagctgagtggtttagctccgtcctcttaagtcggagacgtag
gttcgattcctaccgggctcac
1-17tRNA-Ile(gat)[61261,61336]
tgtgatgtcgtccaacggtttagggcaccgatctgatacatcggaaacca
gggttcaactccctgcttcactacca
1-18tRNA-Gly(tcc)[61425,61500]
ggggtattcatctagtctggctcaggatgcgacacttccactgtcgtcac
gagggttcgaatccttcatacctcac
1-19tRNA-Val(tac)[61563,61635]
tcgggattagctcagtggaagagcggctggtttacacccagcgcgtgggg
agttcgattctctcattccgaac
1-20tRNA-Thr(agt)[61696,61773]
gctggatttgctcatgttggcttgagcacctacctagtaagtaggagtac
gtcggttcgattccgacatccagcccca
1-21tRNA-Asp(gtc)[62051,62124]
cccgatgtcatctagtggcctaggatgccgccctgtcgaggcggtcacgc
gagttcaaatctcgtcgtcgggac
1-22tRNA-Glu(ctc)[62184,62255]
ggttcattggtgtaatggcagcatcccaggttctcaacctgtaggcaggg
gatcgtaacccctatgaactac
>JQ512844.1 Mycobacterium phage Twister, complete genome
1-1tRNA-Trp(cca)[3802,3877]
aggcacgtagctcaattggaagagcagcggtctccaaagccgccggctgc
aggttcgagccctgccgtgtctgcca
>JF957060.1 Mycobacterium phage Timshel, complete genome
1-1tRNA-Trp(cca)[5374,5449]
aggcacctagctcaactggtagagcagcggtctccaaagccgctggttgg
gggttcgagtccctcggtgtctgcca
>JQ684677.1 Mycobacterium phage Tiger, complete genome
1-1tRNA-Trp(cca)[4131,4204]
gggtcagtagctcaattggtagagcagcggtctccaaagccgcgagttgg

aggttcgagtcctccctggcccgc
>JF937106.1 Mycobacterium phage SirDuracell, complete genome
1-1tRNA-Arg(cct)[61928,62011]
atggcccgtagctcagtcaggtagagcagccttgccccttgcgggcttgg
ttcgtccccggttcaaatccgggcgggccttacc
1-2tRNA-Gly(tcc)[62014,62086]
gcgcccgtggtcgaattggaaagactcctggcttccacccaggttatgca
ggttcgagtcctgtcgggcgctc
>MF072690.1 Mycobacterium phage Porcelain, complete genome
1-1tRNA-Gly(tcc)[91633,91705]
gcgcttgtggtcgaattggaaagactcctggcttccacccaggttatgca
ggttcgagtcctgtcgagcgctc
1-2tRNA-Tyr(gta)[92676,92748]
tggcccgtagctcaattggcagagcagccggcggtaattcggcgcgtctc
ggttcgagtccgggcgggctacc
>MF133445.1 Mycobacterium phage Lucky2013, complete genome
1-1tRNA-Gly(tcc)[90687,90759]
gcgcttgtggtcgaattggaaagactcctggcttccacccaggttatgca
ggttcgagtcctgtcgagcgctc
1-2tRNA-Tyr(gta)[91730,91802]
tggcccgtagctcaattggcagagcagccggcggtaattcggcgcgtctc
ggttcgagtccgggcgggctacc
>MF358541.1 Streptomyces phage Warpy, complete genome
1-1tRNA-Thr(cgt)c[16890,16961]
gccccgctggcgtagtggaaacgcacttctttcgtaatgaagagtcaaga
gttcaattctcttgcggggctc
1-2tRNA-Gly(tcc)[72410,72482]
gcatcggtagtttaatggtagaacgtctgacttccaatcagacagcgcgg
gttcgattcccgctcggtgctcc
1-3tRNA-Gln(ctg)[72611,72683]
tccccttggtgtaaccggcaacacaacagactctgaatctgtaattcct
agttcgaatctaggttggggagc
1-4tRNA-Trp(cca)[72890,72961]
atgtccttcgtataatggtagtacaagagattccaaacctcttagcgtgg
gttcgattcctacaggacatgc
1-5tRNA-Pro(tgg)[73818,73890]
caggcacggggaagatggtaatccgttggctttggaagcctaagacactc
ggttcgattccgaggtgcctgac
1-6tRNA-Pro(tgg)[73894,73969]
cgggatatagctcagtttggtcagagcgctgcatttgggatgcagaagtc
gctggttcgaatccagttatcccgac
1-7tRNA-Ile(tat)[76953,77027]
gcgccagtagcataacggtccaatgcagtaggcttatatcctaccgagag
aaggttcaattccttcctggcgtac

1–8tRNA–Ser(gga)[77536,77621]
ggaaggtaagcgattggttagcgggtgtcttggaaaggcactaagggtgt
tacagcccggcagggttcaattccctggccttccgc
1–9tRNA–Ser(tga)[77861,77950]
tggaggcacgcattggagtgcttatcggtcttgaaaaccgtgatgacgta
gtgatatgtcgtgggggttcgactccctctgtctccgccc
1–10tRNA–Asp(gtc)[81078,81154]
gcgtctgtagtatagtctggtctaatgcacctgattgtcaatcaggaaat
cgcgggttcaaatcccgtcaggcgcgc
1–11tRNA–Asp(gtc)[81778,81849]
cccggcgtggtgtaatggtaacatactgcgttgtcagcgcaatgtcgagg
gttcgattcccttcgtcgggac
1–12tRNA–Glu(ctc)[86336,86407]
gcccctatagtctaagggtaggatacaagattctcaatcttgtggtcgga
gttcaattctccgtaggggtac
1–13tRNA–Glu(ttc)[86656,86731]
gtcccggtagattctgctggtagaatcgctagactttcaatctagagtcc
gagggttcaattcccttccggggtac
1–14tRNA–Val(tac)[86989,87060]
gcccgcttagctcaatgggagagcgacggttttacacaccgtatacgggg
gttcgattccctcagtgggtac
1–15tRNA–Leu(tag)[87254,87329]
gcgccgttgacgggaactggcatacctagttgacttagaatcaactgctt
gggagttcgactctcccacggcgtac
1–16tRNA–Leu(tag)[87488,87562]
gcgtcggtagcccaatggcaggaggcaccagtcttaggaactggacagtg
tgagttcgaatctcactcggcgtac
1–17tRNA–Gly(gcc)[87884,87955]
tccgtagtggtgaaatggtatcacatggccttgccaaggctaaagcgcga
gttcgattctcgtctacgggac
1–18tRNA–Arg(tct)[88032,88105]
gcctcggtagcgtagtggatgacgcggatgccttctaagcatttatagac
aggttcgattcctgtccgaggtac
1–19tRNA–Ala(tgc)[88879,88951]
gggcggttagtgttaatggtagcacgagtgctttgcacgcattaagcagg
ggttcgaatcccctactgtccac
1–20tRNA–Lys(ctt)[89112,89187]
ggttcggtcgcacaggctggtagtgcaaaggactcttaatccttgagaac
gtgggttcaaatcccacccgaatcac
1–21tRNA–Asn(gtt)[90044,90120]
tccggtggggctactattggcagtagcgacgagctgttaactcgtttccc
tgtaggttcgattcctgcctccggagc
1–22tRNA–Asn(gtt)[90875,90948]
tgacctatagttcaattggcagagcagatggttgttacccatcaagttgc

aggttcgagtcctgctaggtcagc
1-23tRNA-Arg(cct)[91646,91718]
gtccctatagttcaagggaaagaacaagactctcctaaagtctagatgca
ggttcgaatcctgctggggatac
1-24tRNA-Ile(gat)[91967,92042]
gggagcgtagctcagtctggtcggagcaatgcactgataatgcatcggtc
gctggttcaaatccagccgttcctac
1-25tRNA-Ala(ggc)[92115,92188]
ggggtcgtagcataactggtaatgcacttgtatggcatacaagcgattgt
gggttcaaatcccatcgactccac
1-26tRNA-Lys(ctt)[92385,92458]
gctccggtagctcagttggcagagcaggggactcttaatcctcgtgtcgc
tggttcaagtccagttcggagtac
1-27tRNA-Lys(ttt)[92582,92658]
ggtccagtcgcacagtctggcagtgcaattgacttttaatctttgaggac
gtgggttcgaatcccacctggaccacc
1-28tRNA-Arg(tcg)[92661,92734]
gcccttttagctcattggatagagcgatggtcttcgaagccatgcgtagg
gagttcgattctctcaaggggcgc
1-29tRNA-Met(cat)[94028,94099]
ggcagtagggcgctcggtgtgccaacgactctcataaggtcgaggggctg
gttcgaatccagctattgctac
1-30tRNA-Met(cat)[94100,94171]
ttgcaggttagtgaaacggtatcacgtagggctcataatcctaaattcct
gttcgactcagggcctgccccc
1-31tRNA-Tyr(gta)[94805,94887]
gtgtgagcggcgaagttggagagacgcgacggtctgtaaaaccgttcctt
cgggtgagtcagttcgaatctgacctcacgcac
1-32tRNA-Phe(gaa)[95178,95250]
gggcatgtagctcagttggtagagcttcggtgtgaagtaccgagtgcgag
ggttcgattccctacttgcccac
1-33tRNA-Cys(gca)[95841,95916]
ggggtagatgtagcgggtgctgccgacagtctgcaaaactgttcgtatat
tggagttcgactctcctctatccctc
1-34tRNA-Thr(tgt)[95932,96007]
gccaccttagctcatttggtgtagagcgacgctcttgtaaagcgtaggcg
gtcggttcgaaaccgacaggtggctc
1-35tRNA-Thr(ggt)[96100,96171]
gcccccatagtgctagtggtaacacatatccttggtacggatatatcgtc
agttcgattctgactgggggat
1-36tRNA-Arg(acg)[96302,96375]
ctctccataggctaattggataaaccagagtgctacgaacgctcaattgt
gggttcgagtcccgctggagagac
1-38tRNA-Ala(ggc)[99723,99797]

tgtcacgtaattcaatgggaagaatcaatgcccttggccagggtaggtta
gaggttcgagtcctctcgtgacagc
1-39tRNA-Leu(gag)[104794,104876]
gcgcctatggtggaatggcagacacactagactgagactctaggccctga
tgggcgtccgggttcaactcccggtgggcgtac
1-40tRNA-Val(gac)[105314,105385]
acccgcttagctcaatggtagagcacttgctcgacacgcaagttacgtga
gttcaattctctcagtgggtac
1-41tRNA-Leu(caa)[105820,105896]
ggccctgtagcagaatcaggtatatgcggcggtctcaaacaccgcgtcaa
tgtgggttcgagtcccatcagggctac
1-42tRNA-Ser(gct)[106130,106213]
ggaaggtgagcgagaggtctcgcaggtagtttgctaaactgctggattgt
aaaaggttcggggttcgaatcctcctccttccgc
1-43tRNA-Gln(ttg)[115857,115937]
tccccagttgctctgtttggtaggggccgcctgactttgaatcaggacta
gcgacgtaggttcgaatcctgcctggggagc
>MF358542.1 Streptomyces phage Sushi23, complete genome
1-1tRNA-Thr(cgt)c[16909,16981]
gccccattagtgttaacggaaacatacttccttcgtaacgaagtgtcctc
ggttcgactccgagatggggctc
1-2tRNA-Gly(tcc)[72227,72298]
gcatcggtagtttaatggtagaacgtctgacttccaatcagacagcgcgg
gttcgattcccgctcggtgctc
1-3tRNA-Gln(ctg)[72416,72488]
tcccctttggtgtaaccggcaacacagcagactctgaatctgtagttcct
agttcgaatctaggttggggagc
1-4tRNA-Trp(cca)[72696,72767]
atgtccttcgtataatggtagtacaagagattccaaacctcttagcgtgg
gttcgattcctacaggacatgc
1-5tRNA-Pro(tgg)[73642,73714]
caggtgcggggaagatggtaatccgttggttttggaaacctaagacaccc
ggttcgattccggggcacctgac
1-6tRNA-Pro(tgg)[73718,73793]
cgggatatagctcagcttggtcagagcgctgcatttgggatgcagaagtc
gctggttcgaatccagttatcccgac
1-7tRNA-Ile(tat)[76791,76865]
gcgccagtagcataacggttcaatgcagtaggcttatatcctaccgagag
aaggttcaattccttcctggcgtac
1-8tRNA-Ser(gga)[77383,77468]
ggaaagtaagcgattggctagcaggtatcttggaacggtactaagggtgt
tacagcccggctgggttcgattcccaggctttccgc
1-9tRNA-Ser(tga)[77709,77795]
ggaggcacgcattggagtgcttataggtcttgaaaaccttgaaggtgtag

tgatatgccgtgggggttcgactccctctgtctccgc
1-10tRNA-Asp(gtc)[80900,80976]
gcgtctgtagtatagtctggtctaatgcacctgattgtcaatcaggaaat
cgcgggttcgaatcccgtcaggcgcgc
1-11tRNA-Asp(gtc)[81340,81411]
cccggcgtggtgtaatggaaacacacttcgttgtcagcgaaatgtcgagg
gttcgattcccttcgtcgggac
1-12tRNA-Glu(ctc)[86244,86315]
gcccctatagtctaagggtaggatacaagattctcaatcttgtggtcgga
gttcaattctccgtaggggtac
1-13tRNA-Glu(ttc)[86572,86646]
gccccgttggtctagcggttaggataccggactttcaatccgtgtgaacg
tcggttcgaatccgacacggggtac
1-14tRNA-Val(tac)[86658,86729]
gcccggttagctcaatgggagagcggcgcgtttacaccgcgtatacgggg
gttcgattccctcactgggtac
1-15tRNA-Leu(tag)[86883,86958]
gcgccgttgacgggaactggcatacctagttgacttagaatcaactgctt
gggagttcgactctcccacggcgtac
1-16tRNA-Leu(tag)[87097,87171]
gcgtcggtagcccaatggcaggaggcaccagtcttaggaactggacagtg
tgagttcgagtctcacccggcgtac
1-17tRNA-Gly(gcc)[87494,87565]
tccgtagtggtgaaatggtatcacatggccttgccaaggctaaagcgcga
gttcgattctcgtctacgggac
1-18tRNA-Arg(tct)[87672,87745]
gccctggtagcgtagtggataacgcggctgtcttctaaacagttacaggc
aggttcgattcctgtccagggtac
1-19tRNA-Ala(tgc)[88365,88437]
gggcgattagtgttaatggtagcacgtctgttttgcaagcagatagaggg
ggttcgagtcccccattgtccac
1-20tRNA-Lys(ctt)[88603,88677]
gattcagtcgcagaattggtactgcaaaagactcttaatctttgaggatg
tgggttcgagtcccacctgaatcac
1-21tRNA-Asn(gtt)[89535,89611]
tccggtggggctactattggcagtagcgacgagctgttaactcgtttccc
tgtaggttcgattcctgcctccggagc
1-22tRNA-Asn(gtt)[90363,90435]
tggcctatagcttaacggcagagcgattgactgttaatcaattaggtgct
ggttcgaatccagctaggtcagc
1-23tRNA-Arg(cct)[92094,92166]
gtccctatagttcaagggaaagaacaagactctcctaaagtctagatgca
ggttcgagtcctgctggggatac
1-24tRNA-Ile(gat)[92411,92486]

gggagcgtagctcagtctggtcggagcaatgcactgataatgcatcggtc
gatggttcaaatccagccgttcctac
1-25tRNA-Ala(ggc)[92490,92562]
ggggttgtagcataacggtaatgcacttgcatggcatgcaagcgattgtg
ggttcaaatcccatcgactccac
1-26tRNA-Lys(ctt)[92781,92854]
gctccggtagctcagttggtagagcaggtgactcttaatcatcaggtcca
cagttcaagtctgtgtcggagtac
1-27tRNA-Lys(ttt)[92973,93048]
ggtccagtcgcacagtctggtagtgcaattgacttttaatctttgtggac
gtgggttcgaatcccacctggaccac
1-28tRNA-Arg(tcg)[93052,93125]
gcccttttagctcattggatagagcgttggtcttcgaagccaagcgtagg
gagttcgattctctcaaggggcgc
1-29tRNA-Met(cat)[94383,94455]
ggcagtgtggtgaaacggctatcacgttcccctcataagggaaaatcgaa
ggttcgattccttccattgctac
1-30tRNA-Met(cat)[94456,94527]
ttgcagattagtgaaacggtatcacgctgggctcataatccagaattcct
gttcgaatcagggtctgccccc
1-31tRNA-Tyr(gta)[95156,95238]
gcatgagcggcgaagttggagaggcgcggtggtctgtaaaaccattcctt
cgggtgagtcagttcgaatctgacctcatgcac
1-32tRNA-His(gtg)[95368,95440]
gtgacgttgttgtaatggtggcatagaagattgtgactcttccagaggcg
ggttcgattcccccacgtcacac
1-33tRNA-Phe(gaa)[95508,95580]
gggcatgtagctcagttggtagagcgtcggtgtgaagtaccgagtgcgag
ggttcgattccctacttgcccac
1-34tRNA-Cys(gca)[96164,96238]
gaggtagaagcagcgggtgctgccgacagtctgcaaaactgtttgtattt
ggagttcaactctcctctatctctc
1-35tRNA-Thr(tgt)[96472,96543]
gcctcattcgcttaatggtaaagccctactcttgtaaagtagtgccgtca
gttcgattctgacatgaggctc
1-36tRNA-Thr(ggt)[97147,97220]
gcctcgttagtgctaatggttaacacatatccttggtacggatataaaga
gagttcgattctctcacgaggctc
1-37tRNA-Arg(acg)[97389,97462]
ctctccataggctaattggataaaccagagtgctacgaacgctcaattgt
gggttcgagtcccgctggagagac
1-39tRNA-Ala(ggc)[100659,100734]
tgtcacgtaattcaatcaggcagaatcaatgcccttggccagggtaggtt
agaggttcaaatcctctcgtgacagc

1-40tRNA-Leu(gag)[105401,105483]
gcgcctatggtggaatggtagacacactagactgagactctaggctcgaa
tgagcgtccgggttcgactcccggtgggcgtac
1-41tRNA-Val(gac)[105920,105991]
acccgcttagctcaatggtagagcgcctgctcgacacgcaggagacatga
gttcaattctcttagtgggtac
1-42tRNA-Leu(caa)[106416,106492]
ggctctgtagcagaatcaggtatatgcgccggtctcaaacaccgggtcaa
tgtgggttcgagtcccatcagggctac
1-43tRNA-Ser(gct)[106726,106809]
ggaaggtgagcgagaggtctcgcaggtagtttgctaaactgctgggttgt
aaaaggctcggggttcgaatcctcctccttccgc
1-44tRNA-Gln(ttg)[117198,117278]
tccccagttgctctgtttggtaggggccgcctgactttgaatcaggacta
gcgacgtaggttcgaatcctgcctggggagc
>MF373840.1 Mycobacterium phage Fred313, complete genome
1-1tRNA-Asn(gtt)[3421,3494]
tgatctgtagctcaaccggcagagcacccggctgttaaccgggccgttgg
aggttcgagtcctcccagatcagc
1-2tRNA-Trp(cca)[3530,3603]
aggcgcgtagctcaattggtagagcagcggtctccaaagccgcgtgctgc
aggttcgagtcctgccgcgtctgc
>MF373842.1 Rhodococcus phage Jester, complete genome
1-1tRNA-Asn(gtt)[1503,1576]
tcacgtatagctcaatcggcagagcaacgcactgttaaggcgggggttga
aggttcgagtccttctacgtgagc
1-3tRNA-Trp(cca)[1702,1776]
gggcacgtagctcaattggatagagccccggtctccaaagccggtggttg
ggggttcgagtccctccgggcctgc
>MF324915.1 Mycobacterium phage Amgine, complete genome
1-1tRNA-Gln(ctg)[34683,34774]
tcggcgctagttggcgtgcgagcagccctcgccgctctgggcgtcgtcga
ggggccttacgcgctgccggtgcgattccagcgtgtcggccc
>MF324913.1 Mycobacterium phage Cain, complete genome
1-1tRNA-Trp(cca)[889,963]
gggtgtgtagctcaacccggtagagcagcggtctccaaagccgccggttg
caggttcgattcctgccgcgcccgc
1-2tRNA-Lys(ttt)c[31432,31505]
gcccctgtagctcagttggtagagcaggagacttttaatcttcgggtcca
aggttcgatcccttgcgggggcac
>MF324910.1 Mycobacterium phage GuuelaD, complete genome
1-1tRNA-Thr(cgt)[62780,62851]
gctggattagcttagtggtcaagcgctgccttcgtaacgcagattcaccg
gttcgattccggtatccagctc

1−2tRNA−Pro(tgg)[63080,63154]
ctggctgtagctcaatttggtagagcgctggctttgggtgccaggggttg
caggttcaaatcctgctagccagac
1−3tRNA−Trp(cca)[63275,63350]
agctcggtagctcaattggtagagcagcggtctccaaagccgccggttcc
ccgttcgagtcggggccgggttgcca
1−4tRNA−Leu(tag)[64035,64109]
ggccctctagcccaattggcagaggcacaggttttaggtacctgtcagtg
cgagttcgagtctcgcgggggccac
1−5tRNA−His(gtg)[64110,64179]
gcttagtagcctagtggtaaggcagccggttgtgatccggtgaacctgag
ttcgattctcagctaaagcc
1−6tRNA−Gln(ctg)[64184,64258]
tgaccggtagcacaactggtagttgcgccgcgctctggacgcggaggttg
ttggttcgatcccagctcggtcagc
1−7tRNA−Gly(tcc)[64307,64381]
gtgctagtaacccatgttggctgggtacctgacttccactcaggatttcg
cgggttcgattcctgtctagcacac
1−8tRNA−Lys(ctt)[64424,64501]
tgcctcgttagctcagttggtagagctgccgactcttaatcggtaggtca
caggttcaagtcctgtacggggtaccca
1−9tRNA−Cys(gca)[65683,65754]
gccgttgtgtccgagtggttaggtgccaggctgcaaacttggttagtcgc
gttcgattcgcggggacggctc
1−10tRNA−Asn(gtt)[66162,66235]
tcctccgtagctcaattggcagagcgcgcgactgttaatcgcgtggttgg
tggttcgagtccacccgttggagc
1−11tRNA−Lys(ttt)c[66429,66503]
gcccctatagctcagttggtagagcaggagacttttaatcttcgggtcct
aggttcgatccctagtgggggcacc
>MF324914.1 Mycobacterium phage Krueger, complete genome
1−1tRNA−Lys(ttt)c[32445,32518]
gcccctgtagctcagtcggtagagcaggagacttttaatcttcgggtcca
aggttcgatcccttgcgggggcac
>MF324899.1 Mycobacterium phage Lokk, complete genome
1−1tRNA−Gln(ctg)[6114,6195]
gccctgttcgtttaatcggcaggactcccgctctggacgcagagacaacg
ggcaatggaggttcgactcctctacggggcac
>MF324909.1 Mycobacterium phage PhelpsODU, complete genome
1−1tRNA−Trp(cca)[893,967]
gggtgtgtagctcaacccggtagagcagcggtctccaaagccgccggtta
caggttcgattcctgtcgcgcccgc
1−2tRNA−Lys(ttt)c[31464,31537]
gcctccgtagctcaattggcagagcagctgacttttaatcagcgggtcgc

aggttcgatccctgccgggggcac
>MF324912.1 Mycobacterium phage Phrank, complete genome
1-1tRNA-Trp(cca)[895,969]
gggtgtgtagctcaacccggtagagcagcggtctccaaagccgccggttg
caggttcgattcctgccgcgcccgc
1-2tRNA-Lys(ttt)c[31421,31494]
gcccctgtagctcagttggtagagcaggagacttttaatcttcgggtcca
aggttcgatcccttgcgggggcac
>MF324911.1 Mycobacterium phage SirPhilip, complete genome
1-1tRNA-Trp(cca)[784,857]
gggtgtctagctcaattggtagagcagcggtctccaaagccgccggttgc
aggttcgagtcctgcggcgctcgc
>MF324908.1 Mycobacterium phage Unicorn, complete genome
1-1tRNA-Trp(cca)[893,967]
gggtgtgtagctcaacccggtagagcagcggtctccaaagccgccggtta
caggttcgattcctgtcgcgcccgc
1-2tRNA-Lys(ttt)c[31464,31537]
gcctccgtagctcaattggcagagcagctgacttttaatcagcgggtcgc
aggttcgatccctgccgggggcac
>MF324905.1 Rhodococcus phage Alatin, complete genome
1-1tRNA-Asn(gtt)[1503,1576]
tcacgtatagctcaatcggcagagcaacgcactgttaaggcgggggttga
aggttcgagtccttctacgtgagc
1-3tRNA-Trp(cca)[1702,1776]
gggcacgtagctcaattggatagagccccggtctccaaagccggtggttg
ggggttcgagtccctccgggcctgc
>MF324903.1 Rhodococcus phage AppleCloud, complete genome
1-2tRNA-Trp(cca)[1409,1483]
gggcacgtagctcaattggatagagccccggtctccaaagccggtggttg
ggggttcgagtccctccgggcctgc
>MF324898.1 Rhodococcus phage Hiro, complete genome
1-1tRNA-Asn(gtt)[1503,1576]
tcacgtatagctcaatcggcagagcaacgcactgttaaggcgggggttga
aggttcgagtccttctacgtgagc
1-3tRNA-Trp(cca)[1702,1776]
gggcacgtagctcaattggatagagccccggtctccaaagccggtggttg
ggggttcgagtccctccgggcctgc
>MF324902.1 Rhodococcus phage Krishelle, complete genome
1-1tRNA-Asn(gtt)[1503,1576]
tcacgtatagctcaatcggcagagcaacgcactgttaaggcgggggttga
aggttcgagtccttctacgtgagc
1-3tRNA-Trp(cca)[1702,1776]
gggcacgtagctcaattggatagagccccggtctccaaagccggtggttg
ggggttcgagtccctccgggcctgc

>MF324901.1 Rhodococcus phage Naiad, complete genome
1-1tRNA-Asn(gtt)[1503,1576]
tcacgtatagctcaatcggcagagcaacgcactgttaaggcgggggttga
aggttcgagtccttctacgtgagc
1-3tRNA-Trp(cca)[1702,1776]
gggcacgtagctcaattggatagagccccggtctccaaagccggtggttg
ggggttcgagtccctccgggcctgc
>MF324904.1 Rhodococcus phage RexFury, complete genome
1-1tRNA-Asn(gtt)[1534,1607]
tcacgtatagctcaatcggcagagcaacgcactgttaaggcgggggttga
aggttcgagtccttctacgtgagc
1-3tRNA-Trp(cca)[1733,1807]
gggcacgtagctcaattggatagagccccggtctccaaagccggtggttg
ggggttcgagtccctccgggcctgc
>MF324900.1 Rhodococcus phage StCroix, complete genome
1-1tRNA-Asn(gtt)[1503,1576]
tcacgtatagctcaatcggcagagcaacgcactgttaaggcgggggttga
aggttcgagtccttctacgtgagc
1-3tRNA-Trp(cca)[1702,1776]
gggcacgtagctcaattggatagagccccggtctccaaagccggtggttg
ggggttcgagtccctccgggcctgc
>MF185717.1 Mycobacterium phage AlleyCat, complete genome
1-1tRNA-Trp(cca)[847,920]
atgcgggtagctcaattggtagagcagcggtctccaaaaccgccggttgc
aggttcgagtcctgccccgtgtgc
>MF185726.1 Mycobacterium phage Appletree2, complete genome
1-1tRNA-Leu(caa)[60774,60848]
ggtcctgtaggcaaattggcaaagccgcgtcactcaaaatgacgtgtctg
tgggttcgagtcccaccgggactac
1-2tRNA-Thr(cgt)[60849,60923]
gctgccttagctcagatggctagagcgccgctctcgtaaagcggaggtcg
cgggttcgaggcccgcaggcagctc
1-3tRNA-Lys(ctt)[61210,61283]
gcctcgttagctcagttggtagagctgccgactcttaatcggtaggtcgc
aggttcaagtcctgcacggggtac
1-4tRNA-Tyr(gta)[61294,61377]
gcgtcggaggtacctgttggtttggtacacctgcctgtaaagcaggcgct
tcggcttcgggggttcgattccctcccggcgtac
1-5tRNA-Trp(cca)[61387,61462]
agctcggtagctcaattggtagagcaacggtccccaaagtcgtgggttcc
ccgttcgagtcggggccgggttgcca
1-6tRNA-Leu(tag)[62011,62085]
ggcccgctaggcgaattggcatagccgccagatttaggttctggtgtttc
cgagttcgattctcgggcgggccac

1−7tRNA−Cys(gca)[63311,63384]
gccgctgtggccgagtggctaggcaccggcctgcaaagccggttagtccg
gttcgattccggagggcggctcca
1−8tRNA−Lys(ttt)c[63665,63739]
gcccctatagctcagttggtagagctatcgccttttaagcgacaggtcgc
aggttcgagtcctgctgggggcacc
>MF185727.1 Mycobacterium phage BobSwaget, complete genome
1−1tRNA−Gln(ctg)[6109,6190]
gccctgttcgtttaatcggcaggactcccgctctggacgcagagacaacg
ggcaatggaggttcgactcctctacggggcac
>MF185719.1 Mycobacterium phage Edugator, complete genome
1−1tRNA−Trp(cca)[847,920]
atgcgggtagctcaattggtagagcagcggtctccaaaaccgccggttgc
aggttcgagtcctgccccgtgtgc
>MF185720.1 Mycobacterium phage Guillsminger, complete genome
1−1tRNA−Trp(cca)[822,897]
tcgcaggtagctcaattggtagagcagcggtctccaaaaccgccggttgc
aggttcgagtcctgctctgcgggcca
>MF185729.1 Mycobacterium phage KADY, complete genome
1−1tRNA−Asn(gtt)[3922,3997]
tgatctgtagctcaatcggcagagcacccggctgttaaccgggacgttgg
aggttcgagtcctcccagatcagcca
1−2tRNA−Trp(cca)[4038,4112]
aggcacgtagctcaattggtcagagcagcggtctccaaagccgccggctg
caggttcgagtcctgccgtgtctgc
1−3tRNA−Leu(cag)[4147,4221]
ggctcggtaggcaaacaggcaaagccgcctgtctcaggaacaggtgcgtg
agggttcgactccctcccgagctac
>MF185730.1 Mycobacterium phage Miley16, complete genome
1−1tRNA−Thr(cgt)[63338,63411]
gccaccttagctcagttggtagagcagccccttcgtaacgggcaggtcag
cggttcgattccgctaggtggctc
1−2tRNA−Pro(tgg)[63639,63713]
ctggctgtagctcaacttggtagagcgctggctttgggtgccaggggttg
caggttcaaatcctgctagccagac
1−3tRNA−Trp(cca)[63834,63907]
agctcggtagctcaattggtagagcagcggtctccaaagccgccggttcc
ccgttcgagtcggggtcgggttgc
1−4tRNA−Tyr(gta)[64018,64100]
gtggtggttgggcttgttggttggcccacctgactgtaaatcaggcgttt
cggcaccgggggttcgattccctcccaccacac
1−5tRNA−Leu(tag)[64514,64588]
ggccctctagcccaattggcagaggcacaggttttaggtacctgtcagtg
cgagttcgagtctcgcgggggccac

1–6tRNA–His(gtg)[64589,64658]
gcttagtagcctagtggtaaggcagccggttgtgatccggtgaacctgag
ttcgattctcagctaaagcc
1–7tRNA–Gln(ctg)[64663,64737]
tgaccggtagcacaactggtagttgcgccgcgctctggacgcggaggttg
ttggttcgatcccagctcggtcagc
1–8tRNA–Gly(tcc)[64853,64927]
gtgctagtaacccatgttggctgggtgcctgacttccactcaggatttcg
cgggttcgattcctgtctagcacac
1–9tRNA–Lys(ctt)[64970,65047]
tgcctcgttagctcagttggtagagctgccgactcttaatcggtaggtca
caggttcaagtcctgtacggggtaccca
1–10tRNA–Cys(gca)[66237,66310]
gccgtcatggctgagtggttaggcgtcggactgcaaatccggcttatccc
agttcgattctgggtggcggctcc
1–11tRNA–Asn(gtt)[66672,66745]
tcctccgtagctcaattggcagagcgcgcgactgttaatcgcgtggttgg
tggttcgagtccacccgttggagc
1–12tRNA–Lys(ttt)c[66939,67013]
gcccctatagctcagttggtagagcaggagacttttaatcttcgggtcct
aggttcgatccctagtgggggcacc
>MF185722.1 Mycobacterium phage Peanam, complete genome
1–1tRNA–Trp(cca)[1038,1111]
gggtgtgtagctcaattggcagagcagcggtctccaaagccgccggttgc
acgttcgatccgtgccgcgcccgc
>MF185732.1 Mycobacterium phage Stagni, complete genome
1–1tRNA–Asn(gtt)[3842,3917]
tgatctgtagctcaatcggcagagcacccggctgttaaccgggacgttgg
aggttcgagtcctcccagatcagcca
1–2tRNA–Trp(cca)[3958,4032]
aggcacgtagctcaattggtcagagcagcggtctccaaagccgccggctg
caggttcgagtcctgccgtgtctgc
1–3tRNA–Leu(cag)[4067,4141]
ggctcggtaggcaaacaggcaaagccgcctgtctcaggaacaggtgcgtg
agggttcgactccctcccgagctac
>MF185733.1 Mycobacterium phage StepMih, complete genome
1–1tRNA–Asn(gtt)[3833,3908]
tgatctgtagctcaatcggcagagcacccggctgttaaccgggacgttgg
aggttcgagtcctcccagatcagcca
1–2tRNA–Trp(cca)[3949,4023]
aggcacgtagctcaattggtcagagcagcggtctccaaagccgccggctg
caggttcgagtcctgccgtgtctgc
1–3tRNA–Leu(cag)[4058,4132]
ggctcggtaggcaaacaggcaaagccgcctgtctcaggaacaggtgcgtg

agggttcgactccctcccgagctac
>MF140398.1 Mycobacterium phage Amohnition, complete genome
1-1tRNA-Trp(cca)[812,887]
gggttcctagctcaattggtagagcagcggtctccaaagccgccggttgc
aggttcgactcctgcggagcccgcca
>MF140402.1 Mycobacterium phage Chancellor, complete genome
1-1tRNA-Arg(gcg)[14207,14300]
tcggcggcgctgcgcaggagatgggcagcagcgttcgcggcaccctgtcg
aacctcaaggcggcctattcgcggttcggtgccgagctgtcggg
1-2tRNA-Lys(ttt)c[30855,30930]
gccccgctagctcaatcggaagagcagccggcttttaaccggcgggtacg
gagatcgaaactccggcggggcacca
>MF140403.1 Mycobacterium phage Changeling, complete genome
1-1tRNA-Trp(cca)[6187,6260]
gggcacgtagctcaatcggtagagcagcggtctccaaagccgcaggttcc
aggttcgaaccctggcgggctcgc
>MF140405.1 Mycobacterium phage Clautastrophe, complete genome
1-1tRNA-Tyr(gta)[39564,39647]
gtggcgaaggcaactgttggtttgttgcacctgcctgtaaagcaggcgct
tcggcttcgggggttcaattccctctcgccacac
1-2tRNA-Thr(cgt)[62792,62864]
gccaccttagctcagcggtagagcagcgccttcgtaacgcgcaggtcaac
ggttcgattccgttaggtggctc
1-3tRNA-Pro(tgg)[63217,63289]
cggaaacaggtgtaatggttgcatgaggcgtttggatcgccggggttcgg
ggttcgactccctggtttccgac
1-4tRNA-Trp(cca)[63633,63706]
agctcggtagctcaactggtagagcagtggtctccaaagccaccggttgc
aggttcgactcctgtccgggttgc
1-5tRNA-Leu(tag)[63707,63781]
ggttccgtaggcaaactggcaaagccgtctgacttagaatcaggtgtttg
ggagttcgactctccccggaactac
1-6tRNA-His(gtg)[63782,63851]
gctaagtagcttaatggtaaagcccccggttgtggtccgggcgatcacgg
ttcaactccgtgcttaagcc
1-7tRNA-Gln(ctg)[64131,64205]
tgcccgctagcacaattggcagttgcgctgcgctctgaacgcagaggttc
caggttcgactcctgggtgggcagc
1-8tRNA-Lys(ctt)[64215,64288]
gcgtcgttagctcagttggtagagcagggcactcttaatgctcgggtcgg
gggttcaagtccctcacggcgcac
1-9tRNA-Pyl(cta)[65432,65524]
tgctcaaatgggtagggccgttcccctgtcatctaaaggcaggatgcctc
gctgttaacgaggtcacgcaggttcgacccctgctgggggagc

1-10tRNA-Lys(ttt)c[65529,65602]
gccgcgctagctcagttggtagagcagctgacttttaatcagcgggtccg
gggttcgatcccccggcgcggtac
>MF140406.1 Mycobacterium phage DarthP, complete genome
1-1tRNA-Trp(cca)[812,887]
gggttcctagctcaattggtagagcagcggtctccaaagccgccggttgc
aggttcgactcctgcggagcccgcca
>MF140408.1 Mycobacterium phage DismalFunk, complete genome
1-1tRNA-Arg(gcg)[665,757]
caccgcttagcgaggttgcgcgccacgtcattgcgcccgacggcatcgtg
tcgacggcctggccgtcggtgtgtgcgacgtgcgcggcgatgg
>MF140410.1 Mycobacterium phage Et2Brutus, complete genome
1-1tRNA-Trp(cca)[6118,6193]
gcgttcctagctcaattggtagagcagcggtctccaaagccgcgggttcc
aggttcgagccctggggaacgtgcca
>MF140412.1 Mycobacterium phage Jeckyll, complete genome
1-1tRNA-Trp(cca)[1053,1126]
gggtgtgtagctcaatcggtagagcagcggtctccaaagccgccggttgc
acgttcgagtcgtgccgcgcccgc
>MF140413.1 Mycobacterium phage Kingsolomon, complete genome
1-1tRNA-Tyr(gta)[39564,39647]
gtggcgaaggcaactgttggtttgttgcacctgcctgtaaagcaggcgct
tcggcttcgggggttcaattccctctcgccacac
1-2tRNA-Thr(cgt)[62901,62973]
gccaccttagctcagcggtagagcagcgccttcgtaacgcgcaggtcaac
ggttcgattccgttaggtggctc
1-3tRNA-Pro(tgg)[63324,63396]
cggaaacaggtgtaatggttgcatgaggcgtttggatcgccggggttcgg
ggttcgactccctggtttccgac
1-4tRNA-Trp(cca)[63739,63812]
agctcggtagctcaactggtagagcagtggtctccaaagccaccggttgc
aggttcaagtcctgtccgggttgc
1-5tRNA-Leu(tag)[63813,63887]
ggttccgtaggcaaactggcaaagccgtctgacttagaatcaggtgtttg
ggagttcgactctccccggaactac
1-6tRNA-His(gtg)[63888,63957]
gctaagtagcttaatggtaaagcccccggttgtggtccgggcgatcacgg
ttcaactccgtgcttaagcc
1-7tRNA-Gln(ctg)[64237,64311]
tgcccgctagcacaattggcagttgcgctgcgctctgaacgcagaggttc
caggttcgactcctgggtgggcagc
1-8tRNA-Lys(ctt)[64321,64394]
gcgtcgttagctcagttggtagagcagggcactcttaatgctcgggtcgg
gggttcaagtccctcacggcgcac

1-9tRNA-Pyl(cta)[65537,65629]
tgctcaaatgggtagggccgttcccctgtcatctaaaggcaggatgcctc
gctgttaacgaggtcacgcaggttcgacccctgctgggggagc
1-10tRNA-Lys(ttt)c[65634,65707]
gccgcgctagctcagttggtagagcagctgacttttaatcagcgggtccg
gggttcgatcccccggcgcggtac
>MF140414.1 Mycobacterium phage Krypton555, complete genome
1-1tRNA-Tyr(gta)[39801,39884]
gtggcgaaggtaactgctggtttgttacacctgcctgtaaagcaggcgct
tcggcttcgggggttcaattccctctcgccacac
1-2tRNA-Thr(cgt)[63180,63252]
gccaccttagctcaggggtagagcagcgccttcgtaacgcgcaggtcaac
ggttcgattccgttaggtggctc
1-3tRNA-Pro(tgg)[63599,63673]
ctggttgtagcgcagcttggtagcgcgcctgatttggattcagggggtcg
gaggttcaaatccttccagccagac
1-4tRNA-Gly(tcc)[64014,64088]
gcgctagctacccctgttggcggggtgcctgccttccaagcaggatcacg
cgggttcgattcctgtctagtgctc
1-5tRNA-Trp(cca)[64144,64217]
agctcggtagctcaactggtagagcagtggtctccaaagccaccggttgc
aggttcgactcctgtccgggttgc
1-6tRNA-Leu(tag)[64218,64292]
ggttccgtaggcaaactggcaaagccgtctgacttagaatcaggtgtttg
ggagttcgactctccccggaactac
1-7tRNA-His(gtg)[64293,64362]
gctaagtagcttaatggtaaagcccccggttgtggtccgggcgatcacgg
ttcaactccgtgcttaagcc
1-8tRNA-Gln(ctg)[64644,64719]
tgcccgctagcacaatttggcagttgcgcccggctctgaaccgggaggtt
ccaggttcgactcctgggtgggcagc
1-9tRNA-Lys(ctt)[64731,64804]
gcgtcgttagctcagttggtagagcagccgactcttaatcggcgggtcgg
gggttcaagtccctcacggcgcac
1-10tRNA-Asn(gtt)[65933,66005]
tcccctgtcatttaaccggcaggatgcctcgctgttaacgaggtcgtgca
ggttcgatccctgctgggggagc
1-11tRNA-Lys(ttt)c[66010,66083]
gccgcgctagctcagttggtagagcagctgacttttaatcagcgggtccg
gggttcgatcccccggcgcggtac
>MF140416.1 Mycobacterium phage LastHope, complete genome
1-1tRNA-Trp(cca)[1015,1088]
gggtgtgtagctcaatcggtagagcagcggtctccaaagccgccggttgc
acgttcgagtcgtgccgcgcccgc

>MF140421.1 Mycobacterium phage Nicholas, complete genome
1-1tRNA-Tyr(gta)[39564,39647]
gtggcgaaggcaactgttggtttgttgcacctgcctgtaaagcaggcgct
tcggcttcgggggttcaattccctctcgccacac
1-2tRNA-Thr(cgt)[62901,62973]
gccaccttagctcagcggtagagcagcgccttcgtaacgcgcaggtcaac
ggttcgattccgttaggtggctc
1-3tRNA-Pro(tgg)[63324,63396]
cggaaacaggtgtaatggttgcatgaggcgtttggatcgccggggttcgg
ggttcgactccctggtttccgac
1-4tRNA-Trp(cca)[63739,63812]
agctcggtagctcaactggtagagcagtggtctccaaagccaccggttgc
aggttcaagtcctgtccgggttgc
1-5tRNA-Leu(tag)[63813,63887]
ggttccgtaggcaaactggcaaagccgtctgacttagaatcaggtgtttg
ggagttcgactctccccggaactac
1-6tRNA-His(gtg)[63888,63957]
gctaagtagcttaatggtaaagcccccggttgtggtccgggcgatcacgg
ttcaactccgtgcttaagcc
1-7tRNA-Gln(ctg)[64237,64311]
tgcccgctagcacaattggcagttgcgctgcgctctgaacgcagaggttc
caggttcgactcctgggtgggcagc
1-8tRNA-Lys(ctt)[64321,64394]
gcgtcgttagctcagttggtagagcagggcactcttaatgctcgggtcgg
gggttcaagtccctcacggcgcac
1-9tRNA-Pyl(cta)[65537,65629]
tgctcaaatgggtagggccgttcccctgtcatctaaaggcaggatgcctc
gctgttaacgaggtcacgcaggttcgacccctgctgggggagc
1-10tRNA-Lys(ttt)c[65634,65707]
gccgcgctagctcagttggtagagcagctgacttttaatcagcgggtccg
gggttcgatcccccggcgcggtac
>MF140422.1 Mycobacterium phage Nicholasp3, complete genome
1-1tRNA-Thr(cgt)[62844,62917]
gccaccttagctcagttggtagagcagccccttcgtaacgggcaggtcag
cggttcgactccgctaggtggctc
1-2tRNA-Pro(tgg)[63139,63213]
ctggctgtagctcaacttggtagagcgctggctttgggtgccaggggttg
caggttcaaatcctgctagccagac
1-3tRNA-Trp(cca)[63343,63416]
agctcggtagctcaattggtagagcagcggtctccaaagccgccggttcc
ccgttcgagtcggggtcgggttgc
1-4tRNA-Tyr(gta)[63527,63609]
gtggtggttgggcttgttggttggcccacctgactgtaaatcaggcgttt
cggcatcgggggttcgattccctcccaccacac

1–5tRNA–Leu(tag)[63846,63920]
ggccctctagcccaattggcagaggcacaggttttaggtacctgtcagtg
cgagttcgagtctcgcgggggccac
1–6tRNA–His(gtg)[63921,63990]
gcttagtagcctagtggtaaggcagccggttgtgatccggtgaacctgag
ttcgattctcagctaaagcc
1–7tRNA–Gln(ctg)[63995,64069]
tgaccggtagcacaactggtagttgcgccgcgctctggacgcggaggttg
ttggttcgatcccagctcggtcagc
1–8tRNA–Gly(tcc)[64185,64259]
gtgctagtaacccatgttggctgggtgcctgacttccactcaggatttcg
cgggttcgattcctgtctagcacac
1–9tRNA–Lys(ctt)[64302,64379]
tgcctcgttagctcagttggtagagctgccgactcttaatcggtaggtca
caggttcaagtcctgtacggggtaccca
1–10tRNA–Cys(gca)[65561,65633]
gccgttgtgtccgagtggttaggtgccaggctgcaaacttggttagtcgc
gttcgattcgcggggacggctcc
1–11tRNA–Asn(gtt)[65902,65975]
tcctccgtagctcaattggcagagcgcgcgactgttaatcgcgtggttgg
tggttcgagtccacccgttggagc
1–12tRNA–Lys(ttt)c[66169,66243]
gcccctatagctcagttggtagagcaggagacttttaatcttcgggtcct
aggttcgatccctagtgggggcacc
>MF140428.1 Mycobacterium phage Slimphazie, complete genome
1–1tRNA–Trp(cca)[1003,1076]
gggtgtgtagctcaatcggtagagcagcggtctccaaagccgccggttgc
acgttcgagtcgtgccgcgcccgc
>MF140430.1 Mycobacterium phage Tachez, complete genome
1–1tRNA–Trp(cca)[972,1045]
gggtgtgtagctcaatcggtagagcagcggtctccaaagccgccggttgc
acgttcgagtcgtgtcgcgcccgc
>MF140435.1 Mycobacterium phage Wintermute, complete genome
1–1tRNA–Arg(gcg)[14196,14289]
tcggcggcgctgcgcaggagatgggcagcagcgttcgcggcaccctgtcg
aacctcaaggcggcctattcgcggttcggtgccgagctgtcggg
1–2tRNA–Lys(ttt)c[30842,30917]
gccccgctagctcaatcggaagagcagccggcttttaaccggcgggtacg
gagatcgaaactccggcggggcacca
>MF402939.1 Escherichia phage OSYSP, complete genome
1–1tRNA–Met(cat)c[899,974]
agttagttggcagagcggttatgcacctccttcatacggagcgactacag
tggttcaaatccactactaactacca
1–2tRNA–Ile(gat)c[981,1056]

gctctgatagttcaacaggttagaacaggcgaccgataatcgtcaaatct
tggttcgatcccaagtcggagtacca
1-3tRNA-Thr(tgt)c[1426,1500]
gctcctaaagcattgctggcgatgcagttgccttgtaagcatctgaaccg
ggttcgattcctggtgggagcacca
1-4tRNA-Gly(tcc)c[1905,1979]
gcgtgattagttcagtggctagaataactggcttccaaccagtagacacg
agttcgactctcgtatcccgcacca
1-5tRNA-Gln(ttg)c[1986,2061]
tggagagtagtgtaatggttagcacaacggcctttgactccgttaatggt
aggttcgattcctccttctccagcca
1-6tRNA-Gln(ctg)c[2068,2143]
tgggatgtagatcaattggcagatcgtcggcctctgactccgaaggtccc
acgttcgatccgtggcatcccagcca
1-7tRNA-His(gtg)c[2377,2453]
gtggctatatcataattggttaatgatcctgattgtgaatcaggcctatg
tggattcgaattccactagccacccca
1-8tRNA-Ser(tga)c[3020,3109]
agaagataggacgtagtggtacgtaactggtcttgaaaaccagcccgctg
tagtgatacggtgatggttcgactccattatcttctgcca
1-9tRNA-Leu(tag)c[3693,3769]
gcgtgattgatggaattggcatacataccgtccttagaagtcgggttttg
agggttcgaatcccttgtcacgcacca
1-10tRNA-Ala(tgc)c[3775,3853]
gggggatgggtctgctaggggtggacacctcgcttgcaccgaggacatca
gaacgattcgaattcgttatcctccacca
1-11tRNA-Val(tac)c[4493,4566]
gctcggttagtttaatgggagaaccccgtctttacacggcggttgcgata
gttcgattctatcaccgagtacca
1-12tRNA-Lys(ttt)c[5035,5113]
agatcgctagctcaataggtttagtagcatccgacttttaatcggaaggt
tctgggttcgagtcccaggcgatctacca
1-13tRNA-Met(cat)c[5303,5380]
tgcgggttagatctctggtagagatcgctagtctcataagctagaaagag
gtaggttcgattcctgcacccgcttcca
1-14tRNA-Pro(tgg)c[5387,5464]
ctccgtgtagctcagtttggccagagcgtttcgtttggggcgatagggtc
gggggttcaaatcctcccacggagacca
1-15tRNA-Asp(gtc)c[6806,6882]
gcgaccggggctggcttggtaatggtactcccctgtcacgggagagaatg
tgggttcgaatcccatcggtcgcgcca
1-16tRNA-Asn(gtt)c[7180,7262]
gggtcgttagccaagcggtttggcggtggactgttaatccatgtcgaaag
acaacgtaggttcgaatcctacacggcccgcca

1–17tRNA–Cys(gca)c[7270,7345]
cgaccgttggctgaatggcttaggcgaaggattgcaaatcctttttatgt
gagttcaaatctcatgcggtcgtcca
1–18tRNA–Phe(gaa)c[8083,8157]
gcacctttagctgagatggattagcgcttgcctgaagagcttgagaggtt
cgttcaattcgaacagggtgcacca
1–19tRNA–Glu(ttc)c[8244,8318]
gcacctatcgtctaatggctaggacatcaggatttcaatctgagaatcgg
agttcaattctccgtgggtgtgcca
1–20tRNA–Tyr(gta)c[8327,8414]
gggcgtttattccgtaagtggtagcggaggggattgtaaatccctggtca
ttgcgactcgagtggttcgactccattaacgcccacca
1–21tRNA–Leu(taa)c[9291,9367]
gggggtgtaatcgaattggcataggtactggacttaaaattcaggttttg
tgggttcgaatcccaccacccctacca
1–22tRNA–Ser(gct)c[9958,10046]
ggaagaatagcataacggtattgcagcagattgctaatctgtcggtttga
aatatagccttgtgggttcgattcccacttcttccgcca
1–23tRNA–Arg(tct)c[13581,13652]
cggggtgtagtctaagggagaggcaggagtcttctaaattcctttatgca
ggttcgaatcctgtcacctcgg
1–24tRNA–Met(cat)c[109359,109434]
agttagttggcagagtggttatgcacctccttcatacggagcgactacag
tggttcaaatccactactaactacca
1–25tRNA–Ile(gat)c[109531,109607]
gcttcggtagcttagcgatctaaagcactcggctgataaccgagagatcg
gggggtttaaatccctcccggagtacca
1–26tRNA–Thr(tgt)c[110183,110257]
gctcctaaagcattgctggcgatgcagttgccttgtaagcatctgaaccg
ggttcgattcctggtgggagcacca
1–27tRNA–Gln(ttg)c[110776,110851]
tggagagtagtgtaatggttagcacaacggcctttgactccgttaatggt
aggttcgattcctccttctccagcca
>MF155946.1 Streptomyces phage Mildred21, complete genome
1–1tRNA–Thr(cgt)c[15486,15558]
gccccgctagtgatattggtagcacacttctctcgtaaagaagaagactc
ggttcgattccgaggcggggctc
1–2tRNA–Gly(tcc)[71525,71597]
gcatcggtggtgtagtggtaacacgctgtccttccaagtcagaattgcgg
gttcgattcccgctcggtgctcc
1–3tRNA–Gln(ctg)[71730,71802]
tccccttttggtgtaaccggcaacacaacagactctgaatctgtagttcct
agttcgaatctaggttggggagc
1–4tRNA–Trp(cca)[72009,72080]

atgtcctaagtgttacggaagcacggaaggctccaacccttcaggcgtgg
gttcgactcctacaggacatgc
1-5tRNA-Pro(tgg)[72723,72796]
caggcacggggaagatggtaatccgttggctttggaagcctaagaaactc
ggttcgattccgaggtgcctgacc
1-6tRNA-Pro(tgg)[72930,73005]
cgggatatagctcagcttggtcagagcgctgcatttgggatgcagaagtc
gctggttcgaatccagttatcccgac
1-7tRNA-Ile(tat)[75913,75986]
gcgcctgtagcataacggtcgatgcatctgtcttataagcagacgatagg
gggttcaattccctccaggcgtac
1-8tRNA-Ser(gga)[76553,76638]
ggaaagtaagcgattggctagcaggcatcttggaacggtgctaagggtgt
tacagcccggctgggttcgattcccaggctttccgc
1-9tRNA-Ser(tga)[76879,76965]
ggaaatgttaacgttggcagcgtggaaggctcttgaaaagcctacgccgg
tgaaacggcgcgtcggttcgaatccgaccatttccgc
1-10tRNA-Asp(gtc)[78881,78957]
gcgtctgtagtatagtctggtccaatgcacctgattgtcaatcaggaaat
cgcgggttcaaatcccgtcaggcgcgc
1-11tRNA-Asp(gtc)[79822,79893]
cccggcatagtgtagtggaaacacgatacgttgtcagcgtattatcgagg
gttcaattccctctgtcgggac
1-12tRNA-Glu(ctc)[84530,84606]
gctccggtggtctagtttggtctaggatacttgactctcaatcaagagga
cacgggttcaaatcccgttcggagtac
1-13tRNA-Glu(ttc)[84653,84728]
gccccgttagtctagtggcctaggattccagactttcaatctgcgagaac
gtcggttcgaatccgacacggggtac
1-14tRNA-Val(tac)[84807,84880]
gcgggtgtcgtctagcggctaaggccccatccttacaagatggtcatcgg
aggttcgagtcctctcacccgtac
1-15tRNA-Leu(tag)[85156,85231]
gcgccgttgacgggaactggcatacctagttgacttagaatcaactgctt
gggagttcgactctcccacggcgtac
1-16tRNA-Leu(tag)[85837,85919]
gcgcctgtggtggaatttaggtagacacgctggtcttaggaaccagttct
tcggagtgcaggttcaagtcctgtcaggcgtac
1-17tRNA-Gly(gcc)[86303,86374]
tccgtagtggtgtagtggcaacacgtcaccttgccaaggtgaagttgcga
gttcgattctcgtctacgggac
1-18tRNA-Ala(tgc)[87077,87149]
gggcgattagtgttaatggtagcacgtctgttttgcaagcagatagaggg
ggttcgagtcccccattgtccac

1-19tRNA-Lys(ctt)[87307,87382]
gattcggtcgcacagtctggcagtgcaaaggactcttaacccttgagaac
gtgggttcaaatcccacccgaatcac
1-20tRNA-Asn(gtt)[88535,88612]
tcccacgatacacctgtgtggctcggtgtagcagactgttaatctgccgg
tcgctggttcgactccagccgtgggagc
1-21tRNA-Asn(gtt)[89144,89217]
tggcctatagcttaatcggcagagcggttggttgttacccaatctggtgt
aggttcgaatcctgctaggtcagc
1-22tRNA-Arg(cct)[89973,90045]
gtccctatagtttagaggatagaacgtcactctcctaaagtgaagacgca
ggttcgactcctgctggggatac
1-23tRNA-Ile(gat)[90283,90358]
gggagcgtagctcagtctggtcggagcaatgcactgataatgcatcggtc
gctggttcaaatccagccgttcctac
1-24tRNA-Ala(ggc)[90362,90434]
ggggtcgtagcataacggtaatgcacttgtatggcatacaagcgattgtg
ggttcaaatcccatcgactccac
1-25tRNA-Lys(ctt)[90603,90676]
gctccggtagctcagttggcagagcaggggactcttaatcctcgtgtcgc
tggttcaagtccagttcggagtac
1-26tRNA-Lys(ttt)[90803,90878]
ggtccagtcgcacagtctggtagtgcaattgacttttaatctttgtggac
gtgggttcgaatcccacctggaccac
1-27tRNA-Met(cat)[92544,92616]
ggcagtgtggtgaaacggctatcacgttcccctcataagggaaaatcgaa
ggttcgattccttccattgctac
1-28tRNA-Met(cat)[92617,92688]
ttgcaggttagtgaaacggtatcacgtagggctcataatcctaaattcct
gttcgaatcagggcctgccccc
1-29tRNA-Tyr(gta)[93079,93161]
gcgtaagcggcgaagttggagagacgcggtggtctgtaaaaccattcctt
cgggtgagtcagttcgaatctgaccttacgcac
1-30tRNA-His(gtg)[93487,93562]
gggttggtagctcaattggattagagcgctgcgttgtggtcgcagtggtt
gagggttcgagtcccttccttcccac
1-31tRNA-Phe(gaa)[93744,93816]
gggcatgtagctcagttggtagagcgtcggtgtgaagtaccgagtgcgag
ggttcgattccctacttgcccac
1-32tRNA-Cys(gca)[94407,94481]
gaggtagaagcaacgggtgttgccgacagtctgcaaaactgttcgtattt
ggagttcgactctcctctatctctc
1-33tRNA-Thr(tgt)[94497,94572]
gcctcgttagctcatttggtgtagagcgacgctcttgtaaagcgtaggtg

gtcggttcgaaaccgacacgaggctc
1-34tRNA-Thr(ggt)[94665,94738]
gcctagttagtgacagtggtagaacatttccttggtacggaaaaggtcgt
gggttcgattcccacactaggctc
1-37tRNA-Leu(gag)[102844,102926]
gcgctcatggtggaatggcagacacactagactgagactctaggcccta
agggcgtccgggttcgactcccggtgggcgtac
1-38tRNA-Val(gac)[103328,103399]
acccgcttagctcaatggtagagcgcttgttcgacacacaagagacgtga
gttcgattctctcagtgggtac
1-39tRNA-Leu(caa)[103870,103944]
ggccctgtagcagaatggcatatgcggttccctcaaaaggaatgtcaatg
tgggttcgagtcccatcagggctac
1-40tRNA-Ser(gct)[104174,104257]
ggaaggtaagcgaggggtctcgcaggttggttgctaaccaactggactgt
aaaaggtccggggttcgaatcctctgccttccgc
1-41tRNA-Gln(ttg)[114821,114901]
tccccagttgctctgtttggtaggggccgcctgactttgaatcaggatta
gcgacgttggttcgaatccagcctggggagc
>MF347636.1 Streptomyces phage NootNoot, complete genome
1-1tRNA-Thr(cgt)c[14356,14427]
gccccgctggcgtaatggtaacgcacttctttcgtaatgaagagttaaga
gttcgattctcttgcggggctc
1-2tRNA-Gly(tcc)[71446,71517]
gcatcggtagtttaatggtagaacgtctgacttccaatcagacagcgcgg
gttcgattcccgctcggtgctc
1-3tRNA-Gln(ctg)[71639,71713]
tcccccgtggtccgtgtggctggccactgagactctgaatctcaggtacg
taggttcgactcctgccgggggagc
1-4tRNA-Trp(cca)[71801,71874]
gcatccttagtgtaatggtttagcacacgagcttccaaacctcttagagt
gggttcgattcctacaggatgtgc
1-5tRNA-Pro(tgg)[72744,72816]
caggcacggggaagatggtaatccgttggctttggaagcctaagaaactc
ggttcgattccgaggtgcctgac
1-6tRNA-Pro(tgg)[72820,72895]
cgggatatagctcagcttggtcagagcgctgcatttgggatgcagaagtc
gctggttcgaatccagttatcccgac
1-7tRNA-Ile(tat)[75889,75963]
gcgccagtagcataacggttcaatgcagtaggcttatatcctaccgagag
aaggttcaattccttcctggcgtac
1-8tRNA-Ser(gga)[76483,76568]
ggaaagtaagcgattggctagcaggtatcttggaacggtaccaagggtgt
tacagcccggctgggttcgattcccaggctttccgc

1-9tRNA-Ser(tga)[76809,76895]
ggaggcacgcattggtgggcttatcggtcttgaaaaccgtgatggcgtag
tgatatgccgtgggggttcgattccctctgtctccgc
1-10tRNA-Asp(gtc)[78270,78346]
gcgtctgtagtatagtctggtccaatgcacctgattgtcaatcaggaaat
cgcgggttcaaatcccgtcaggcgcgc
1-11tRNA-Asp(gtc)[78786,78857]
cccggcgtggtgtaatggtaacatactgcgttgtcagcgcaatgtcgagg
gttcgattcccttcgtcgggac
1-12tRNA-Glu(ctc)[83499,83570]
gctcctatagtctaagggtaggatacaagattctcaatcttgtggtcgga
gttcaattctccgtaggagtac
1-13tRNA-Glu(ttc)[83824,83898]
gccccgttggtctagcggttaggataccggactttcaatccgtgtgaacg
tcggttcgaatccgacacggggtac
1-14tRNA-Val(tac)[84157,84228]
gcccggttagctcaatgggagagcggcggttttacacaccgtatacgggg
gttcgattccctcactgggtac
1-15tRNA-Leu(tag)[84490,84565]
gcgccgttgacgggaactggcatacctagttgacttagaatcaactgctt
gggagttcgactctcccacggcgtac
1-16tRNA-Leu(tag)[84704,84778]
gcgtcggtagcccaatggcaggaggcaccagtcttaggaactggacagtg
tgagttcgagtctcacccggcgtac
1-17tRNA-Gly(gcc)[85102,85173]
tccgtagtggtgtagtggtaacatacctccttgccaaggaggcgttgcga
gttcgattctcgtctacgggac
1-18tRNA-Arg(tct)[85314,85387]
gccctggtagcgtagtggataacgcggctgtcttctaaacagttacaggc
aggttcgattcctgtccagggtac
1-19tRNA-Ala(tgc)[86009,86081]
gggcgattagtgttaatggtagcacgtcagttttgcacactgatagaggg
ggttcgagtcccccattgtccac
1-20tRNA-Lys(ctt)[86247,86321]
gattcagtcgcagaattggtactgcaaaagactcttaatctttgaggatg
tgggttcgagtcccacctgaatcac
1-21tRNA-Asn(gtt)[87180,87257]
tccggtggggctactattggcagtagcgacgagctgttaactcgtttccc
tgtaggttcgattcctgcctccggagcc
1-22tRNA-Asn(gtt)[87902,87974]
tggcctatagcttaacggcagagcgattgactgttaatcaattaggtgct
ggttcgaatccagctaggtcagc
1-23tRNA-Arg(cct)[89645,89717]
gtctccatagttcaagggacagaacaagactctcctaaagtctagatgca

ggttcgaatcctgctggggatac
1-24tRNA-Ile(gat)[89763,89838]
gggagcgtagctcagtctggtcggagcaatgcactgataatgcatcggtc
gctggttcaaatccagccgttcctac
1-25tRNA-Ala(ggc)[89911,89984]
ggggtcgtagcataactggtaatgcacttgtatggcatacaagcgattgt
gggttcaaatcccatcgactccac
1-26tRNA-Lys(ctt)[90197,90270]
gctccggtagctcagttggcagagcaggggactcttaatcctcgtgtcgc
tggttcaagtccagttcggagtac
1-27tRNA-Lys(ttt)[90397,90472]
ggtccagtcgcacagtctggtagtgcaattgacttttaatctttgtggac
gtgggttcgaatcccacctggaccac
1-28tRNA-Arg(tcg)[90807,90880]
gcccttttagctcactggatagagcgatggtcttcgaagccatgcgtagg
gagttcgattctctcaaggggcac
1-29tRNA-Met(cat)[91981,92053]
ggcagtgtggtgaaacggctatcacgttcccctcataagggaaaatcgaa
ggttcgattccttccattgctac
1-30tRNA-Met(cat)[92054,92125]
ttgcaggttagtgaaacggtatcacgtagggctcataatcctaaattcct
gttcgaatcagggcctgccccc
1-31tRNA-Tyr(gta)[92755,92837]
gcatgagcggcgaagttggagagtcgcggcggtctgtaaaaccgttctct
agggtgagtcagttcgaatctgacctcatgcac
1-32tRNA-His(gtg)[93163,93238]
gggttggtagctcaattggattagagcgctgcgttgtggtcgcagtggtt
gagggttcgagtcccttccttcccac
1-33tRNA-Phe(gaa)[93420,93492]
gggcatgtagctcagttggtagagcttcggtgtgaagtaccgagtgcgag
ggttcgattccctacttgcccac
1-34tRNA-Cys(gca)[94086,94161]
ggggtagatgtagcgggtgctgccgacagtctgcaaaactgttcgtatat
tggagttcgactctcctctatccctc
1-35tRNA-Thr(tgt)[94177,94252]
gccaccttagctcatttggtgtagagcgacgctcttgtaaagcgtaggcg
gtcggttcgaaaccgacaggtggctc
1-36tRNA-Thr(ggt)[94345,94416]
gcccccatagtgctagtggtaacacatatccttggtacggatatatcgtc
agttcgattctgactgggggat
1-37tRNA-Arg(acg)[94547,94620]
ctctccataggctaattggacaaaccagagtgctacgaacgctcaattgt
gggttcgagtcccgctggagagac
1-39tRNA-Ala(ggc)[97594,97668]

tgtcacgtaattcaattggcagaatcaatgcccttggccagggtaggtta
gaggttcgagtcctctcgtgacagc
1-40tRNA-Leu(gag)[103012,103094]
gcgcccatggtggaatggtagacacactggactgagactccaggctcgaa
tgagcgtccgggttcgactcccggtgggcgtac
1-41tRNA-Val(gac)[103532,103603]
acccgcttagctcaatggtagagcgcttgctcgacacgcaagagacgtga
gttcaattctctcagtgggtac
1-42tRNA-Leu(caa)[104033,104109]
ggccctgtagcagaatttggtatatgcgctggtctcaaaaaccgggtcaa
tgcgggttcgagtcccgtcagggctac
1-43tRNA-Ser(gct)[104343,104426]
ggaaggtgagcgagaggtctcgcaggtagtttgctaaactgctgggctgt
aaaaggttcggggttcgaatcctcctccttccgc
1-44tRNA-Gln(ttg)[114474,114554]
tccccagttgctctgtttggtaggggccgcctgactttgaatcaggacta
gcgacgtaggttcgaatcctgcctggggagc
>MF347637.1 Streptomyces phage Paradiddles, complete genome
1-1tRNA-Thr(cgt)c[14347,14418]
gccccgctggcgtaatggtaacgcacttctttcgtaatgaagagttaaga
gttcgattctcttgcggggctc
1-2tRNA-Gly(tcc)[71710,71781]
gcatcggtagtttaatggtagaacgtctgacttccaatcagacagcgcgg
gttcgattcccgctcggtgctc
1-3tRNA-Gln(ctg)[71788,71862]
tcccccgtggtccgtgtggctggccactgagactctgaatctcaggtacg
taggttcgactcctgccgggggagc
1-4tRNA-Trp(cca)[71950,72023]
gcatccttagtgtaatggtttagcacacgagcttccaaacctcttagagt
gggttcgattcctacaggatgtgc
1-5tRNA-Pro(tgg)[72893,72965]
caggcacggggaagatggtaatccgttggctttggaagcctaagaaactc
ggttcgattccgaggtgcctgac
1-6tRNA-Pro(tgg)[72969,73044]
cgggatatagctcagcttggtcagagcgctgcatttgggatgcagaagtc
gctggttcgaatccagttatcccgac
1-7tRNA-Ile(tat)[76038,76112]
gcgccagtagcataacggttcaatgcagtaggcttatatcctaccgagag
aaggttcaattccttcctggcgtac
1-8tRNA-Ser(gga)[76632,76717]
ggaaagtaagcgattggctagcaggtatcttggaacggtaccaagggtgt
tacagcccggctgggttcgattcccaggctttccgc
1-9tRNA-Ser(tga)[76958,77044]
ggaggcacgcattggtgggcttatcggtcttgaaaaccgtgatggcgtag

tgatatgccgtgggagttcgaatctctctgtctccgc
1-10tRNA-Asp(gtc)[80172,80248]
gcgtctgtagtatagtctggtccaatgcacctgattgtcaatcaggaaat
cgcgggttcaaatcccgtcaggcgcgc
1-11tRNA-Asp(gtc)[80688,80759]
cccggcgtggtgtaatggtaacatactgcgttgtcagcgcaatgtcgagg
gttcgattcccttcgtcgggac
1-12tRNA-Glu(ctc)[85797,85868]
gctcctatagtctaagggtaggatacaagattctcaatcttgtggtcgga
gttcaattctccgtaggagtac
1-13tRNA-Glu(ttc)[86122,86196]
gccccgttggtctagcggttaggataccggactttcaatccgtgtgaacg
tcggttcgaatccgacacggggtac
1-14tRNA-Val(tac)[86455,86526]
gcccggttagctcaatgggagagcggcggttttacacaccgtatacgggg
gttcgattccctcactgggtac
1-15tRNA-Leu(tag)[86788,86863]
gcgccgttgacgggaactggcatacctagttgacttagaatcaactgctt
gggagttcgactctcccacggcgtac
1-16tRNA-Leu(tag)[87002,87076]
gcgtcggtagcccaatggcaggaggcaccagtcttaggaactggacagtg
tgagttcgagtctcacccggcgtac
1-17tRNA-Gly(gcc)[87400,87471]
tccgtagtggtgtagtggtaacatacctccttgccaaggaggcgttgcga
gttcgattctcgtctacgggac
1-18tRNA-Arg(tct)[87612,87685]
gccctggtagcgtagtggataacgcggctgtcttctaaacagttacaggc
aggttcgattcctgtccagggtac
1-19tRNA-Ala(tgc)[88307,88379]
gggcgattagtgttaatggtagcacgtcagttttgcacactgatagaggg
ggttcgagtcccccattgtccac
1-20tRNA-Lys(ctt)[88545,88619]
gattcagtcgcagaattggtactgcaaaagactcttaatctttgaggatg
tgggttcgagtcccacctgaatcac
1-21tRNA-Asn(gtt)[89478,89555]
tccggtggggctactattggcagtagcgacgagctgttaactcgtttccc
tgtaggttcgattcctgcctccggagcc
1-22tRNA-Asn(gtt)[90200,90272]
tggcctatagcttaacggcagagcgattgactgttaatcaattaggtgct
ggttcgaatccagctaggtcagc
1-23tRNA-Arg(cct)[91938,92010]
gtctccatagttcaagggacagaacaagactctcctaaagtctagatgca
ggttcgaatcctgctggggatac
1-24tRNA-Ile(gat)[92056,92131]

gggagcgtagctcagtctggtcggagcaatgcactgataatgcatcggtc
gctggttcaaatccagccgttcctac
1-25tRNA-Ala(ggc)[92204,92276]
ggggtcgtagcataacggtaatgcacttgtatggcatacaagcgattgtg
ggttcaaatcccatcgactccac
1-26tRNA-Lys(ctt)[92500,92573]
gctccggtagctcagttggcagagcaggggactcttaatcctcgtgtcgc
tggttcaagtccagttcggagtac
1-27tRNA-Lys(ttt)[92700,92775]
ggtccagtcgcacagtctggtagtgcaattgacttttaatctttgtggac
gtgggttcgaatcccacctggaccac
1-28tRNA-Arg(tcg)[93110,93183]
gcccttttagctcactggatagagcgatggtcttcgaagccatgcgtagg
gagttcgattctctcaaggggcac
1-29tRNA-Met(cat)[94284,94356]
ggcagtgtggtgaaacggctatcacgttcccctcataagggaaaatcgaa
ggttcgattccttccattgctac
1-30tRNA-Met(cat)[94357,94428]
ttgcaggttagtgaaacggtatcacgtagggctcataatcctaaattcct
gttcgaatcagggcctgccccc
1-31tRNA-Tyr(gta)[95058,95140]
gcatgagcggcgaagttggagagtcgcggcggtctgtaaaaccgttctct
agggtgagtcagttcgaatctgacctcatgcac
1-32tRNA-His(gtg)[95466,95541]
gggttggtagctcaattggattagagcgctgcgttgtggtcgcagtggtt
gagggttcgagtcccttccttcccac
1-33tRNA-Phe(gaa)[95723,95795]
gggcatgtagctcagttggtagagcttcggtgtgaagtaccgagtgcgag
ggttcgattccctacttgcccac
1-34tRNA-Cys(gca)[96389,96464]
ggggtagatgtagcgggtgctgccgacagtctgcaaaactgttcgtatat
tggagttcgactctcctctatccctc
1-35tRNA-Thr(tgt)[96480,96555]
gccaccttagctcatttggtgtagagcgacgctcttgtaaagcgtaggcg
aagagttcgaaactcttaggtggctc
1-36tRNA-Thr(ggt)[97158,97229]
gcccccatagtgctagtggtaacacatatccttggtacggatatatcgtc
agttcgattctgactgggggat
1-37tRNA-Arg(acg)[97360,97433]
ctctccataggctaattggacaaaccagagtgctacgaacgctcaattgt
gggttcgagtcccgctggagagac
1-39tRNA-Ala(ggc)[100407,100481]
tgtcacgtaattcaattggcagaatcaatgcccttggccagggtaggtta
gaggttcgagtcctctcgtgacagc

1-40tRNA-Leu(gag)[105387,105469]
gcgcccatggtggaatggtagacacactggactgagactccaggctcgaa
tgagcgtccgggttcgactcccggtgggcgtac
1-41tRNA-Val(gac)[105907,105978]
acccgcttagctcaatggtagagcgcttgctcgacacgcaagagacgtga
gttcaattctctcagtgggtac
1-42tRNA-Leu(caa)[106408,106484]
ggctctgtagcagaatttggtatatgcgctggtctcaaaaaccgggtcaa
tgcgggttcgagtcccgtcagggctac
1-43tRNA-Ser(gct)[106718,106801]
ggaaggtgagcgagaggtctcgcaggtagtttgctaaactgctgggctgt
aaaaggttcggggttcgaatcctcctccttccgc
1-44tRNA-Gln(ttg)[116849,116929]
tccccagttgctctgtttggtaggggccgcctgactttgaatcaggacta
gcgacgtaggttcgaatcctgcctggggagc
>MF347638.1 Streptomyces phage Peebs, complete genome
1-1tRNA-Thr(cgt)c[16513,16585]
gcctcattagtgttaacggaaacatacttccttcgtaacgaagtgtcctc
ggttcgactccgagatggggctc
1-2tRNA-Gly(tcc)[71869,71940]
gcatcggtagtttaatggtagaacgtctgacttccaatcagacagcgcgg
gttcgattcccgctcggtgctc
1-3tRNA-Gln(ctg)[72058,72130]
tcccctttggtgtaaccggcaacacagcagactctgaatctgtagttcct
agttcgaatctaggttggggagc
1-4tRNA-Trp(cca)[72338,72409]
atgtccttcgtataatggtagtacaagagattccaaacctcttagcgtgg
gttcgattcctacaggacatgc
1-5tRNA-Pro(tgg)[73284,73356]
caggtgcggggaagatggtaatccgttggttttggaaacctaagacaccc
ggttcgattccggggcacctgac
1-6tRNA-Pro(tgg)[73360,73435]
cgggatatagctcagcttggtcagagcgctgcatttgggatgcagaagtc
gctggttcgaatccagttatcccgac
1-7tRNA-Ile(tat)[76433,76507]
gcgccagtagcataacggttcaatgcagtaggcttatatcctaccgagag
aaggttcaattccttcctggcgtac
1-8tRNA-Ser(gga)[77027,77112]
ggaaggtaagcgattggttagcaggagtcttggaaaggctctaagggtgt
tacagcccggcagggttcaattccctggccttccgc
1-9tRNA-Ser(tga)[77353,77439]
ggaggcacgcattggagtgcttataggtcttgaaaaccttgaaggtgtag
tgatatgccgtgggggttcgactccctctgtctccgc
1-10tRNA-Asp(gtc)[80544,80620]

gcgtctgtagtatagtctggtctaatgcacctgattgtcaatcaggaaat
cgcgggttcaaatcccgtcaggcgcgc
1-11tRNA-Asp(gtc)[80984,81055]
cccggcgtggtgtaatggaaacacacttcgttgtcagcgaaatgtcgagg
gttcgattcccttcgtcgggac
1-12tRNA-Glu(ctc)[85888,85959]
gcccctatagtctaagggtaggatacaagattctcaatcttgtggtcgga
gttcaattctccgtaggggtac
1-13tRNA-Glu(ttc)[86216,86290]
gccccgttggtctagcggttaggataccggactttcaatccgtgtgaacg
tcggttcgaatccgacacggggtac
1-14tRNA-Val(tac)[86302,86373]
gcccggttagctcaatgggagagcggcgcgtttacaccgcgtatacgggg
gttcgattccctcactgggtac
1-15tRNA-Leu(tag)[86527,86602]
gcgccgttgacgggaactggcatacctagttgacttagaatcaactgctt
gggagttcgactctcccacggcgtac
1-16tRNA-Leu(tag)[86741,86815]
gcgtcggtagcccaatggcaggaggcaccagtcttaggaactggacagtg
tgagttcgaatctcacccggcgtac
1-17tRNA-Gly(gcc)[87136,87207]
tccgtagtggtgaaatggtatcacatggccttgccaaggctaaagcgcga
gttcgattctcgtctacgggac
1-18tRNA-Arg(tct)[87314,87387]
gccctggtagcgtagtggataacgcggctgtcttctaaacagttacaggc
aggttcgattcctgtccagggtac
1-19tRNA-Ala(tgc)[88007,88079]
gggcgattagtgttaatggtagcacgtctgttttgcaagcagatagaggg
ggttcgagtcccccattgtccac
1-20tRNA-Lys(ctt)[88246,88320]
gattcagtcgcagaattggtactgcaaaagactcttaatctttgaggatg
tgggttcgagtcccacctgaatcac
1-21tRNA-Asn(gtt)[89178,89254]
tccggtggggctactattggcagtagcgacgagctgttaactcgtttccc
tgtaggttcgattcctgcctccggagc
1-22tRNA-Asn(gtt)[90006,90078]
tggcctatagcttaacggcagagcgattgactgttaatcaattaggtgct
ggttcgaatccagctaggtcagc
1-23tRNA-Arg(cct)[91737,91809]
gtccctatagttcaagggaaagaacaagactctcctaaagtctagatgca
ggttcgaatcctgctggggatac
1-24tRNA-Ile(gat)[92054,92129]
gggagcgtagctcagtctggtcggagcaatgcactgataatgcatcggtc
gctggttcaaatccagccgttcctac

1–25tRNA–Ala(ggc)[92133,92205]
ggggttgtagcataacggtaatgcacttgcatggcatgcaagcgattgtg
ggttcaaatcccatcgactccac
1–26tRNA–Lys(ctt)[92424,92497]
gctccggtagctcagttggtagagcaggtgactcttaatcatcaggtcca
cagttcaagtctgtgtcggagtac
1–27tRNA–Lys(ttt)[92616,92691]
ggtccagtcgcacagtctggtagtgcaattgacttttaatctttgtggac
gtgggttcgaatcccacctggaccac
1–28tRNA–Arg(tcg)[92695,92768]
gcccttttagctcattggatagagcgttggtcttcgaagccaagcgtagg
gagttcgattctctcaaggggcgc
1–29tRNA–Met(cat)[94026,94098]
ggcagtgtggtgaaacggctatcacgttcccctcataagggaaaatcgaa
ggttcgattccttccattgctac
1–30tRNA–Met(cat)[94099,94170]
ttgcagattagtgaaacggtatcacgctgggctcataatccagaattcct
gttcgaatcagggtctgccccc
1–31tRNA–Tyr(gta)[94798,94880]
gcatgagcggcgaagttggagaggcgcggtggtctgtaaaaccattcctt
cgggtgagtcagttcgaatctgacctcatgcac
1–32tRNA–His(gtg)[95010,95082]
gtgacgttgttgtaatggtggcatagaagattgtgactcttccagaggcg
ggttcgattcccccacgtcacac
1–33tRNA–Phe(gaa)[95150,95222]
gggcatgtagctcagttggtagagcgtcggtgtgaagtaccgagtgcgag
ggttcgattccctacttgcccac
1–34tRNA–Cys(gca)[95806,95880]
gaggtagaagcagcgggtgctgccgacagtctgcaaaactgtttgtattt
ggagttcaactctcctctatctctc
1–35tRNA–Thr(tgt)[95930,96005]
gccaccttagctcatttggtgtagagcgacgctcttgtaaagcgtaggcg
gtcggttcgaaaccgacaggtggctc
1–36tRNA–Thr(ggt)[96097,96170]
gcctcgttagtgctaatggttaacacatatccttggtacggatataaaga
gagttcgattctctcacgaggctc
1–37tRNA–Arg(acg)[96339,96412]
ctctccataggctaattggataaaccagagtgctacgaacgctcaattgt
gggttcgagtcccgctggagagac
1–39tRNA–Ala(ggc)[99595,99670]
tgtcacgtaattcaatcaggcagaatcaatgcccttggccagggtaggtt
agaggttcaaatcctctcgtgacagc
1–40tRNA–Leu(gag)[104809,104891]
gcgcccatggtggaatggtagacacactagactgagactctaggctcgaa

tgagcgtccgggttcgactcccggtgggcgtac

1–41tRNA–Val(gac)[105328,105399]

acccgcttagctcaatggtagagcgcctgctcgacacgcaggagacatga

gttcaattctcttagtgggtac

1–42tRNA–Leu(caa)[105825,105901]

ggctctgtagcagaatcaggtatatgcgccggtctcaaacaccgggtcaa

tgtgggttcgagtcccatcagggctac

1–43tRNA–Ser(gct)[106135,106218]

ggaaggtgagcgagaggtctcgcaggtagtttgctaaactgctgggttgt

aaaaggctcggggttcgaatcctcctccttccgc

1–44tRNA–Gln(ttg)[116332,116412]

tccccagttgctctgtttggtaggggccgcctgactttgaatcaggacta

gcgacgtaggttcgaatcctgcctggggagc

>MF347639.1 Streptomyces phage Samisti12, complete genome

1–1tRNA–Thr(cgt)c[17283,17355]

gccccattagtgttaacggaaacatacttccttcgtaacgaagtgtcctc

ggttcgactccgagatggggctc

1–2tRNA–Gly(tcc)[73311,73383]

gcatcggtagtttaatggtagaacgagtgacttccaatcattcagcgcgg

gttcgattcccgctcggtgctcc

1–3tRNA–Gln(ctg)[73514,73588]

tcccccgtggtccgtgtggctggccactgagactctgaatctcaggtacg

taggttcgactcctgccgggggagc

1–4tRNA–Trp(cca)[73716,73787]

atgccctaagtgttacggtagcacgttggtctccaaaaccaaaagcgtag

gttcgactcctacagggcgtgc

1–5tRNA–Pro(tgg)[74663,74735]

caggtgcggggaagatggtaatccgttggttttggaaacctaagacaccc

ggttcgattccggggcacctgac

1–6tRNA–Pro(tgg)[74739,74814]

cgggatatagctcagcttggtcagagcgctgcatttgggatgcagaagtc

gctggttcgaatccagttatcccgac

1–7tRNA–Ile(tat)[77812,77886]

gcgccagtagcataacggttcaatgcagtaggcttatatcctaccgagag

aaggttcaattccttcctggcgtac

1–8tRNA–Ser(gga)[78406,78491]

ggaaggtaagcgattggttagcaggagtcttggaaaggctctaagggtgt

tacagcccggcagggttcaattccctggccttccgc

1–9tRNA–Ser(tga)[78732,78818]

ggaggcacgcattggagtgcttataggtcttgaaaaccttgaaggtgtag

tgatatgccgtgggggttcgactccctctgtctccgc

1–10tRNA–Asp(gtc)[81923,81999]

gcgtctgtagtatagtctggtctaatgcacctgattgtcaatcaggaaat

cgcgggttcgaatcccgtcaggcgcgc

1-11tRNA-Asp(gtc)[82363,82434]
cccggcgtggtgtaatggaaacacacttcgttgtcagcgaaatgtcgagg
gttcgattcccttcgtcgggac
1-12tRNA-Glu(ctc)[87270,87341]
gcccctatagtctaagggtaggatacaagattctcaatcttgtggtcgga
gttcaattctccgtaggggtac
1-13tRNA-Glu(ttc)[87598,87672]
gccccgttggtctagcggttaggataccggactttcaatccgtgtgaacg
tcggttcgaatccgacacggggtac
1-14tRNA-Val(tac)[87684,87755]
gcccggttagctcaatgggagagcggcgcgtttacaccgcgtatacgggg
gttcgattccctcactgggtac
1-15tRNA-Leu(tag)[87909,87984]
gcgccgttgacgggaactggcatacctagttgacttagaatcaactgctt
gggagttcgactctcccacggcgtac
1-16tRNA-Leu(tag)[88123,88197]
gcgtcggtagcccaatggcaggaggcaccagtcttaggaactggacagtg
tgagttcgagtctcacccggcgtac
1-17tRNA-Gly(gcc)[88520,88591]
tccgtagtggtgaaatggtatcacatggccttgccaaggctaaagcgcga
gttcgattctcgtctacgggac
1-18tRNA-Arg(tct)[88698,88771]
gccctggtagcgtagtggataacgcggctgtcttctaaacagttacaggc
aggttcgattcctgtccagggtac
1-19tRNA-Ala(tgc)[89391,89463]
gggcgattagtgttaatggtagcacgtctgttttgcaagcagatagaggg
ggttcgagtcccccattgtccac
1-20tRNA-Lys(ctt)[89630,89704]
gattcagtcgcagaattggtactgcaaaagactcttaatctttgaggatg
tgggttcgagtcccacctgaatcac
1-21tRNA-Asn(gtt)[90562,90638]
tccggtggggctactattggcagtagcgacgagctgttaactcgtttccc
tgtaggttcgattcctgcctccggagc
1-22tRNA-Asn(gtt)[91390,91462]
tggcctatagcttaacggcagagcgattgactgttaatcaattaggtgct
ggttcgaatccagctaggtcagc
1-23tRNA-Arg(cct)[93121,93193]
gtccctatagttcaagggaaagaacaagactctcctaaagtctagatgca
ggttcgaatcctgctggggatac
1-24tRNA-Ile(gat)[93438,93513]
gggagcgtagctcagtctggtcggagcaatgcactgataatgcatcggtc
gctggttcaaatccagccgttcctac
1-25tRNA-Ala(ggc)[93517,93589]
ggggttgtagcataacggtaatgcacttgcatggcatgcaagcgattgtg

ggttcaaatcccatcgactccac
1–26tRNA–Lys(ctt)[93808,93881]
gctccggtagctcagttggtagagcaggtgactcttaatcatcaggtcca
cagttcaagtctgtgtcggagtac
1–27tRNA–Lys(ttt)[94000,94075]
ggtccagtcgcacagtctggtagtgcaattgacttttaatctttgtggac
gtgggttcgaatcccacctggaccac
1–28tRNA–Arg(tcg)[94079,94152]
gcccttttagctcattggatagagcgttggtcttcgaagccaagcgtagg
gagttcgattctctcaaggggcgc
1–29tRNA–Met(cat)[95410,95482]
ggcagtgtggtgaaacggctatcacgttcccctcataagggaaaatcgaa
ggttcgattccttccattgctac
1–30tRNA–Met(cat)[95483,95554]
ttgcagattagtgaaacggtatcacgctgggctcataatccagaattcct
gttcgaatcagggtctgccccc
1–31tRNA–Tyr(gta)[96182,96264]
gcatgagcggcgaagttggagagacgcggtggtctgtaaaaccattcctt
cgggtgagtcagttcgaatctgacctcatgcac
1–32tRNA–His(gtg)[96394,96466]
gtgacgttgttgtaatggtggcatagaagattgtgactcttccagaggcg
ggttcgattcccccacgtcacac
1–33tRNA–Phe(gaa)[96534,96606]
gggcatgtagctcagttggtagagcgtcggtgtgaagtaccgagtgcgag
ggttcgattccctacttgcccac
1–34tRNA–Cys(gca)[97190,97264]
gaggtagaagcagcgggtgctgccgacagtctgcaaaactgtttgtattt
ggagttcaactctcctctatctctc
1–35tRNA–Thr(tgt)[97314,97389]
gccaccttagctcatttggtgtagagcgacgctcttgtaaagcgtaggcg
gtcggttcgaaaccgacaggtggctc
1–36tRNA–Thr(ggt)[97481,97554]
gcctcgttagtgctaatggttaacacatatccttggtacggatataaaga
gagttcgattctctcacgaggctc
1–37tRNA–Arg(acg)[97723,97796]
ctctccataggctaactggataaaccagagtgctacgaacgctcaattgt
gggttcgaatcccgctggagagac
1–39tRNA–Pro(tgg)[101009,101084]
tgtcacgtaattcaatcaggcagaatcaatgctcttggccagggtaggtt
agaggttcaaatcctctcgtgacagc
1–40tRNA–Leu(gag)[106391,106473]
gcgcccatggtggaatggtagacacactagactgagactctaggctcgaa
tgagcgtccgggttcgactcccggtgggcgtac
1–41tRNA–Val(gac)[106910,106981]

acccgcttagctcaatggtagagcgcctgctcgacacgcaggagacatga
gttcaattctcttagtgggtac
1-42tRNA-Leu(caa)[107406,107482]
ggctctgtagcagaatcaggtatatgcgccggtctcaaacaccgggtcaa
tgtgggttcgagtcccatcagggctac
1-43tRNA-Ser(gct)[107716,107799]
ggaaggtgagcgagaggtctcgcaggtagtttgctaaactgctgggttgt
aaaaggctcggggttcgaatcctcctccttccgc
1-44tRNA-Gln(ttg)[117530,117610]
tccccagttgctctgtttggtaggggccgcctgactttgaatcaggacta
gcgacgtaggttcgaatcctgcctggggagc
>MF377440.1 Mycobacterium phage Bella96, complete genome
1-1tRNA-Trp(cca)[964,1037]
gggtgtgtagctcaatcggtagagcagcggtctccaaagccgccggttgc
acgttcgagtcgtgccgcgcccgc
>MF319184.1 Mycobacterium phage GenevaB15, complete genome
1-1tRNA-Trp(cca)[56831,56903]
atgggcgtggcttgatagggaagcaacggtctccaaagccgtgaaatgca
ggttcgaatcctgccgcccgtgc
1-3tRNA-Asn(gtt)[57540,57615]
tggggtatagccacaatggcagtggcaccgggctgttaactcggaagagt
gttggttcgagtccagctaccctagc
1-4tRNA-Ala(cgc)[58532,58604]
cggaatgtgcagcctaatggtaaggcggggagttcgcgactcccgatcca
ggttcgaatcctggcgctccggc
1-5tRNA-Tyr(gta)[58681,58758]
ctcgtcgcgaataggcgatcacagctggctgtaaaccagctccttcgggt
gtgcaggttcgactcctgacggcgagac
1-6tRNA-Gln(ctg)[59048,59119]
tgtcttctcgactaacggcaagtcactgggctctgaccccagcaattgag
gttcgaatccttgggggacaac
1-7tRNA-Pro(tgg)[59125,59197]
cgggatatggtgtaagggttagcgtaccggcgttggagaccggaagacat
ggttcgagtccatgtatcccgac
1-8tRNA-Phe(gaa)[59736,59811]
cggttcgtagctcagtttggcaagagcgctggcttgaaaccccagaggcc
ccaggttcgactcctggcgttccggc
1-9tRNA-Met(cat)[59951,60022]
tgcgcggtggtgtaacgggaacatcctggtctcataagtcagggcttcgg
gttcgaatcccggctgcgcaac
1-10tRNA-Arg(acg)[60292,60370]
cccggtgttgagcaacggagcgctcaatgggctacgaacccatattcgca
tgcaggttcgaatcctgtcatcgggtcca
1-11tRNA-His(gtg)[60373,60446]

cctcttgtagctcaatcggttagagcagcggtttgtgaagccgcagacca
cggatcgtaaccgtgcagggggac
1-12tRNA-Cys(gca)[60555,60627]
gtcgagatgacagagtggttatgtgacggtctgcaaagccgatctacgcc
ggttcaaatccggctctcgattc
1-13tRNA-Leu(tag)[60881,60955]
ggatctgtagcccaaatggaagaggcggcaggcttagaccctgttcagtg
tgggttcgactccctccagattcac
1-14tRNA-Leu(caa)[60959,61034]
gctcgactaggcaaactaggcaaagccgcaggcctcaaaagccagtgtgt
gtgagttcgattctcacgtcgagtac
1-15tRNA-Lys(ctt)[61038,61112]
gggatgttggtataaacggcaattactgcagactcttaatctgtcaaatc
tgggttcgaatcccaggcgtcccac
1-16tRNA-Ile(gat)[61113,61188]
tgtgatgtcgtccaacggtttagggcaccgatctgatacatcggaaacca
gggttcaactccctgcttcactacca
1-17tRNA-Gly(tcc)[61274,61350]
ggggtattcatctagtctggctcaggatgcgacacttccactgtcgtcac
gagggttcgaatccttcatacctcacc
1-18tRNA-Val(tac)[61397,61468]
tccccactcgtccaatgggagggcaccgcgcttacaacgcgaggacggga
gttcgattctctcgtggggaac
1-19tRNA-Thr(agt)[61529,61606]
gctggatttgctcatgttggcttgagcacctacctagtaagtaggagtac
gtcggttcgattccgacatccagcccca
1-20tRNA-Ala(tgc)[61665,61735]
gggcgcatgttccaaggtggcgaccgacccttgcaaggttggtgggcgag
ttcgattctcgtcgcttccac
1-21tRNA-Asp(gtc)[61813,61886]
cccgtcatcatctagaggcctaggatgccactctgtcgaagtggtcacac
gggttcaaatcccgttggcgggac
1-22tRNA-Glu(ctc)[61963,62034]
ggttcattggtgtaatggcagcattccagattctcaatctggcggcaggg
gttcaactcccctatgaactac
>MF537628.1 Rhodococcus phage Bonanza, complete genome
1-1tRNA-Asn(gtt)[1503,1576]
tcacgtatagctcaatcggcagagcaacgcactgttaaggcgggggttga
aggttcgagtccttctacgtgagc
1-3tRNA-Trp(cca)[1702,1776]
gggcacgtagctcaattggatagagccccggtctccaaagccggtggttg
ggggttcgagtccctccgggcctgc
>MF189179.1 Rhodococcus phage Yoncess, complete genome
1-1tRNA-Asn(gtt)[1503,1576]

tcacgtatagctcaatcggcagagcaacgcactgttaaggcgggggttga
aggttcgagtccttctacgtgagc
1−3tRNA−Trp(cca)[1702,1776]
gggcacgtagctcaattggatagagccccggtctccaaagccggtggttg
ggggttcgagtccctccgggcctgc
>MF467948.1 Streptomyces phage DrGrey, complete genome
1−1tRNA−Ala(cgc)c[44692,44764]
gaggtggtagaagggcagctcgccctcctcgcgcgggggcttgaccttga
cgttccacccggcggccagcatg
>MF472896.1 Mycobacterium phage JoshKayV, complete genome
1−1tRNA−Gln(ctg)[4810,4886]
ttccccgttcgtctaagcggcaagacaccgggttctggccccggcaatcg
aggttcgaatccttgatggggaaccca
>MF472895.1 Mycobacterium phage Kimona, complete genome
1−1tRNA−Trp(cca)[1483,1558]
aggcacctagctcaattggtagagcagcggtctccaaagccgctggttgg
gggttcgagtccctcggtgtctgcca
1−2tRNA−Tyr(gta)[1560,1644]
cgcgagatacccaagcggcaacgggagctgcctgtaaagcagtcgccatc
tggcttcgcaggttcgagtcctgctctcgcgacca
>MF472893.1 Mycobacterium phage MissWhite, complete genome
1−1tRNA−Trp(cca)[4198,4273]
gtacacgtagctcaattggtagagcagcggtctccaaagccgccggttcc
aggttcgactcctggcgtgtatgcca
1−2tRNA−Tyr(gta)[4278,4362]
cgcgagatacccaagcggcaacgggatctgactgtaaatcagacgccttc
gggcttcgcaggttcgagtcctgctctcgcgacca
>MF472892.1 Mycobacterium phage TreyKay, complete genome
1−1tRNA−Trp(cca)[979,1052]
gggtgcgtagctcaatcggtagagcagcggtctccaaagccgccggttgc
acgttcgagtcgtgccgcgcccgc
>MF158042.1 Shigella phage Sd1, complete genome
1−1tRNA−Arg(cct)c[39476,39551]
gttctgctggtgtaacaggataacgcagagacctcctaagtctcagttgc
tggttcgagtccagcgcggaacgcca
1−2tRNA−Trp(cca)c[39557,39630]
atggggttagttcattggtagaatagtggcctccaaaaccaaagaggttg
gttcgattccaacatcccgtgcca
>MF158038.1 Shigella phage Sf11, complete genome
1−1tRNA−Ser(gct)[20558,20645]
ggatagttggctgagaggccgaaagcggtcggttgctaaccgataaaccg
gaaacggttcacaggttcgaatcctgtactatccgcca
1−2tRNA−Arg(tct)[20652,20727]
acaccattagctcaatggatagagcagtagccttctaagctattggttgc

aggttcgaatcctgcatggtgtgcca
>MF158039.1 Shigella phage Sf12, complete genome
1-1tRNA-Arg(cct)c[43468,43543]
gttctgctggcgtaacaggataacgcagagacctcctaagtctcagttgc
tggttcgagtccagcgcggaacacca
>MF668282.1 Gordonia phage ShayRa, complete genome
1-1tRNA-Asn(gtt)[5265,5339]
tggcttgtagctcaacggcagagcgctcggctgttaaccgagtggttgaa
ggttcgaatccttccaggccagcca
1-2tRNA-Lys(ctt)[5341,5414]
gcatccgtagctgagtggtttagctctgggctcttaacccggagacggag
gttcgattcctcccggatgcacca
1-3tRNA-Gln(ctg)[5416,5488]
tccccattcgtccaactggcaggacaccggagtctggttccggagatcga
ggttcgaatcctcggtaggggagc
1-4tRNA-Arg(acg)c[48016,48095]
tcggttcgctggtcggaaggtgcgactcggtgcctacggtgatccggcga
tggtcccgttcgaggtgtgggaaccgatcc
>MF668267.1 Mycobacterium phage Apocalypse, complete genome
1-1tRNA-Trp(cca)[987,1060]
gggtgtgtagctcaatcggtagagcagcggtctccaaagccgccggttgc
acgttcgagtcgtgccgcgcccgc
>MF668269.1 Mycobacterium phage Drake55, complete genome
1-1tRNA-Gln(ctg)[4936,5009]
tccccggtagtccaatcggtagtgacgctcggctctggaccgagagattg
aggttcgagtccttgctggggagc
>MF668277.1 Mycobacterium phage MadamMonkfish, complete genome
1-1tRNA-Arg(cct)[62046,62129]
atggcccgtagctcagtcaggtagagcagccttgccccttgcgggcttgg
ttcgtccccggttcaaatccgggcgggccttgcc
1-2tRNA-Gly(tcc)[62132,62204]
gcgcccgtggtcgaattggaaagactcctggcttccacccaggttatgca
ggttcgagtcctgtcgggcgctc
>MF668284.1 Mycobacterium phage Squint, complete genome
1-1tRNA-Tyr(gta)[92617,92689]
tggcccgtagctcaattggcagagcagccggcggtaattcggcgcgtctc
ggttcgagtccgggcgggctacc
>MF668288.1 Mycobacterium phage Wiks, complete genome
1-1tRNA-Asn(gtt)[5402,5474]
tgacgtgtagctcaatggcagagcacccggctgttaaccggacggttgaa
ggttcgagtccttccatgtcagc
1-2tRNA-Trp(cca)[5479,5552]
gcgctcctagctcaattggtagagcagcggtctccaaagccgcgagttcc
aggttcgagtcctggggagcgtgc

1–3tRNA–Gln(ctg)[5594,5666]
tccctgttcgtctaatcggtaagacaccgggctctggacccggcaatcga
ggttcgaatccttgatggggagc
>MF773750.1 Mycobacterium phage OKCentral2016, complete genome
1–1tRNA–Trp(cca)[3483,3558]
aggcacgtagctcaattggaagagcagcggtctccaaagccgcccgttgc
aggttcgagtcctgccgtgtctgcca
>GQ979703.1 Lactococcus lactis phage p2, complete genome
1–1tRNA–Trp(cca)[26661,26733]
tgcgagcatagtatagtggtaatgctacagattccaaacctgtaaacgtg
ggttcgattcctactgttcgtgt
>GQ303265.1 Mycobacterium phage Pumpkin, complete genome
1–1tRNA–Arg(cct)[62256,62339]
atggcccgtagctcagtcaggtagagcagccttgccccttgcgggcttgg
ttcgtccccggttcaaatccgggcgggccttgcc
1–2tRNA–Gly(tcc)[62342,62414]
gcgcccgtggtcgaattggaaagactcctggcttccacccaggttatgca
ggttcgagtcctgtcgggcgctc
>FJ848884.1 Lactococcus phage CB19, complete genome
1–1tRNA–Trp(cca)[28086,28158]
tgcgagcatagtataatggtaatgccacagattccaaacctgtaaacgtg
ggttcgattcctactgttcgtgt
>FJ848885.1 Lactococcus phage CB20, complete genome
1–1tRNA–Trp(cca)[28068,28140]
tgcgagcatagtataatggtaatgccacagattccaaacctgtaaacgtg
ggttcgattcctactgttcgtgt
>GQ478085.1 Enterococcus phage phiFL2B, complete genome
1–1tRNA–Arg(acg)[15189,15263]
ttggcgtgtagcattgtggtaatgcaactgattacgtgtgagataagatg
cgggttcgaatcctgtcacgccaat
1–2tRNA–Met(cat)[35911,35984]
ggaccattagctcagttggttagagccaaccgctcataacggttaggtca
taggttcgagtcctgtatggtcca
>GQ478087.1 Enterococcus phage phiFL3B, complete genome
1–1tRNA–Arg(tcg)[16011,16085]
ttggcgtgtagcattgtggtaatgcaactgacttcgtgtgagataagatg
cgggttcgaatcctgtcacgccaat
1–2tRNA–Met(cat)[39360,39433]
ggaccattagctcagttggttagagccaaccgctcataacggttaggtca
taggttcgagtcctgtatggtcca
>JF704105.1 Mycobacterium phage Adephagia, complete sequence
1–1tRNA–Trp(cca)[1003,1076]
gggtgtgtagctcaatcggtagagcagcggtctccaaagccgccggttgc
acgttcgagtcgtgccgcgcccgc

>JF704107.1 Mycobacterium virus George, complete genome

1-1tRNA-Trp(cca)[4309,4382]

gggtcagtagctcaattggtagagcagcggtctccaaagccgcgagttgg

aggttcgagtcctccctggcccgc

>JF704097.1 Mycobacterium virus Gladiator, complete genome

1-1tRNA-Asn(gtt)[5396,5468]

tggcgtgtagctcaatggcagagcccccggctgttaaccgggtggttgaa

ggttcgagtccttccacgccagc

1-2tRNA-Trp(cca)[5472,5545]

gcgttcctagctcaattggtagagcagcggtctccaaagccgcgtgttcc

aggttcgagtcctggggagcgtgc

1-3tRNA-Gln(ctg)[5587,5659]

tccctgttcgtctaatcggtaagacaccgggctctggacccggtaatcga

ggttcgagtccttgatggggagc

>JF704108.1 Mycobacterium phage JoeDirt, complete sequence

1-1tRNA-Leu(caa)[61808,61882]

ggtcctgtaggcaaattggcaaagccgcgtcactcaaaatgacgtgtctg

tgggttcgagtcccaccgggactac

1-2tRNA-Thr(cgt)[61883,61957]

gctgccttagctcagatggctagagcgccgctctcgtaaagcggaggtcg

cgggttcgaggcccgcaggcagctc

1-3tRNA-Lys(ctt)[62244,62317]

gcctcgttagctcagttggtagagctgccgactcttaatcggtaggtcgc

aggttcaagtcctgcacggggtac

1-4tRNA-Tyr(gta)[62328,62411]

gcgtcggaggtacctgttggtttggtacacctgcctgtaaagcaggcgct

tcggcttcgggggttcgattccctcccggcgtac

1-5tRNA-Trp(cca)[62421,62496]

agctcggtagctcaattggtagagcaacggtctccaaagccgtgggttcc

ccgttcgagtcggggtcgggttgcca

1-6tRNA-Leu(tag)[62914,62988]

ggcccgctaggcgaattggcatagccgccagatttaggttctggtgtttc

cgagttcgactctcgggcgggccac

1-7tRNA-Cys(gca)[64210,64283]

gccgctgtggccgagtggctaggcaccggcctgcaaagccggttagtccg

gttcgattccggagggcggctcca

1-8tRNA-Asn(gtt)[64626,64698]

tcccctgtcgtttaattggcaggacgccgggttgttgccccggttgttta

ggttcgagtcctaacgggggagc

1-9tRNA-Lys(ttt)c[64823,64896]

gcccctatagctcagttggtagagctgctgacttttaatcagtaggtcgc

acgttcgagtcgtgctgggggcac

>JF704110.1 Mycobacterium virus Packman, complete genome

1-1tRNA-Glu(ctc)[5906,5978]

ggtccgttggagtagcggcaactcatctgactctcactcagaagatcgcg
ggttcaaatcccgcacggactac
>JF704113.1 Mycobacterium phage UPIE, complete sequence
1−1tRNA−Leu(caa)[61206,61280]
ggtcctgtaggcaaattggcaaagccgcgtcactcaaaatgacgtgtctg
tgggttcgagtcccaccgggactac
1−2tRNA−Thr(cgt)[61281,61355]
gctgccttagctcagatggctagagcgccgctctcgtaaagcggaggtcg
cgggttcgaggcccgcaggcagctc
1−3tRNA−Lys(ctt)[61642,61715]
gcctcgttagctcagttggtagagctgccgactcttaatcggtaggtcgc
aggttcaagtcctgcacggggtac
1−4tRNA−Tyr(gta)[61726,61809]
gcgtcggaggtacctgttggtttggtacacctgcctgtaaagcaggcgct
tcggcttcgggggttcgattccctcccggcgtac
1−5tRNA−Trp(cca)[61819,61892]
agctcggtagctcaattggtagagcagcggtctccaaagccgtgggttcc
ccgttcgagtcggggccgggttgc
1−6tRNA−Leu(tag)[61893,61967]
ggaactgtaggcaaactggaaaagccgcctgacttaggatcaggtgtttg
cgagttcgactctcgccagttctac
1−7tRNA−His(gtg)[61968,62037]
gctaagtagcttaatggtaaagcgccccgttgtggccggggtgattccgg
ttcgattccgggcttaagcc
1−8tRNA−Cys(gca)[63215,63288]
gccgctgtggccgagtggctaggcaccggcctgcaaagccggttagtccg
gttcgattccggagggcggctcca
1−9tRNA−Lys(ttt)c[63569,63643]
gcccctatagctcagttggtagagctatcgccttttaagcgacaggtcgc
aggttcgagtcctgctgggggcacc
>JF704106.1 Mycobacterium phage Anaya, complete sequence
1−1tRNA−Trp(cca)[1035,1108]
gggtgtgtagctcaattggcagagcagcggtctccaaagccgccggttgc
acgttcgagtcgtgccgcgcccgc
>JF704111.1 Mycobacterium phage Rockstar, complete genome
1−1tRNA−Trp(cca)[3699,3772]
aggcgcgtagctcaattggtagagcggcggtctccaaagccgctggctgc
aggttcgagtcctgccgcgtctgc
>KU997639.1 Mycobacterium phage Loadrie, complete genome
1−1tRNA−Thr(cgt)[63222,63295]
gccaccttagctcagttggtagagcagccccttcgtaacgggcaggtcag
cggttcgactccgctaggtggctc
1−2tRNA−Pro(tgg)[63693,63767]
ctggctgtagctcaacttggtagagcgctggctttgggtgccaggggttg

caggttcaaatcctgctagccagac
1−3tRNA−Trp(cca)[64005,64080]
agctcggtagctcaattggtagagcagcggtctccaaagccgccggttcc
ccgttcgagtcggggtcgggttgcca
1−4tRNA−Leu(tag)[64749,64823]
ggccctctagcccaattggcagaggcacaggttttaggtacctgtcagtg
cgagttcgagtctcgcgggggccac
1−5tRNA−His(gtg)[64824,64893]
gcttagtagcctagtggtaaggcagccggttgtgatccggtgaacctgag
ttcgattctcagctaaagcc
1−6tRNA−Gln(ctg)[64898,64972]
tgaccggtagcacaactggtagttgcgccgcgctctggacgcggaggttg
ttggttcgatcccagctcggtcagc
1−7tRNA−Gly(tcc)[65021,65095]
gtgccagtaacccatgttggctgggtacctgacttccactcaggatttcg
cgggttcgattcctgtctggcacac
1−8tRNA−Lys(ctt)[65131,65206]
gcctcgttagctcagttggtagagctgccgactcttaatcggtaggtcac
aggttcaagtcctgtacggggtacca
1−9tRNA−Cys(gca)[66388,66460]
gccgttgtgtccgagtggttaggtgccaggctgcaaacttggttagtcgc
gttcgattcgcggggacggctcc
1−10tRNA−Asn(gtt)[66995,67068]
tcctccgtagctcaattggcagagcgcgcgactgttaatcgcgtggttgg
tggttcgagtccacccgttggagc
1−11tRNA−Lys(ttt)c[67262,67336]
gcccctatagctcagttggtagagcaggagacttttaatcttcgggtcct
aggttcgatccctagtgggggcacc
>JF704098.1 Mycobacterium virus JHC117, complete genome
1−1tRNA−Asn(gtt)[3838,3913]
tgatctgtagctcaactggcagagcacccggctgttaaccgggtcgttgg
aggttcgagtcctcccagatcagcca
1−2tRNA−Trp(cca)[3954,4028]
aggcacgtagctcaattggtcagagcagcggtctccaaagccgccggctg
caggttcgagtcctgccgtgtctgc
1−3tRNA−Leu(cag)[4063,4137]
ggctcggtaggcaaacaggcaaagccgcctgtctcaggaacaggtgcgtg
agggttcgactccctcccgagctac
>KY114934.1 Salmonella phage SP01, complete genome
1−1tRNA−Arg(tct)[80907,80981]
cggggtgtagtctaagggagaggcaggagtcttctaaattcctttatgca
ggttcgaatcctgtcacctcggcca
1−2tRNA−Ser(gct)[82599,82687]
ggaagaatagcataatggtattgcagcagattgctaatctgtcggtttga

aatatagccttgtgggttcgattcccacttcttccgcca
1-3tRNA-Leu(taa)[82986,83062]
gggggtgtaatcgaattggcataggtactggacttaaaattcaggttttg
tgggttcgaatcccaccacccctacca
1-4tRNA-Tyr(gta)[83945,84031]
gggaaatattctgtaagtggtagcagagctgactgtaaattagttgtcat
tgcgactcgggtggttcgactccatctttccccacca
1-5tRNA-Glu(ttc)[84039,84113]
gcacctatcgtctagcggttaggacaccactctttcacagtgggaacacg
agttcgatcctcgttgggtgtgcca
1-6tRNA-Phe(gaa)[84200,84274]
gcacctttagctgagatggattagcgcttgcctgaagagcttgagaggtt
cgttcgatacgaacagggtgcacca
1-7tRNA-Cys(gca)[84907,84982]
cgaccgttggctgaatggcttaggcgaaggattgcaaatcctttttatgt
gagttcaaatctcatgcggtcgtcca
1-8tRNA-Asn(gtt)[85135,85217]
gggtcgttagccaagcggtttggcggtggactgttaatccatgtcgaaag
acaacgtaggttcgaatcctacacggcccgcca
1-9tRNA-Asp(gtc)[85515,85591]
gcgaccggggctggcttggtaatggtactcccctgtcacgggagagaatg
tgggttcgaatcccatcggtcgcgcca
1-10tRNA-Lys(ctt)[85769,85844]
acatccatagctcaatggttagagctaccgcctcttaagcggagggttct
aggttcaagtcctagtgggtgtacca
1-11tRNA-Pro(tgg)[86459,86536]
ctccgtgtagctcagtttggccagagcgtttcgtttggggcgatagggtc
gggggttcaaatcctcccacggagacca
1-12tRNA-Met(cat)[86543,86620]
tgcgggttagatctctggcagagatcgctagtctcataagctagaaagag
gtaggttcgattcctgcacccgcttcca
1-13tRNA-Lys(ttt)[86810,86888]
agatcgctagctcaataggtttagtagcatccgacttttaatcggaaggt
tctgggttcgagtcccaggcgatctacca
1-14tRNA-Ala(tgc)[87268,87342]
gggcgaatagtgtcagcgggagcacaccagacttgcaatctggtagggag
ggttcgagtccctctttgtccacca
1-15tRNA-Leu(tag)[87348,87424]
gcgtgattgatggaatgggcatacataccgtccttagaagtcgggttttg
agggttcgaatcccttgtcacgcacca
1-16tRNA-Ser(tga)[87634,87723]
agaagataggacgtagtggtacgtaactggtcttgaaaaccagcccgctg
tagcgatacggtgatggttcgactccattatcttctgcca
1-17tRNA-His(gtg)[88288,88364]

gtggctatatcataattggttaatgatcctgattgtgaatcaggcctatg
tggattcgaattccactagccacccca
1–18tRNA–Gln(ctg)[88826,88899]
tggagtgtagatcaattggcagatcgtcggcctctgactccgaaggttcc
tggttcgaccccaggcactccagc
1–19tRNA–Gln(ttg)[88908,88983]
tggagagtagtgtaacggttagcacaacggcctttgactccgttaatggt
aggttcgattcctccttctccagcca
1–20tRNA–Thr(tgt)[89444,89530]
gctgggttggcagaatggctgaatgcacctgatttgtaatcaggaggagt
aatcccgttgcaggttcgaatcctgcgcccagcacca
1–21tRNA–Ile(gat)[90086,90162]
acttcggtagcttagcgatctaaagcactcggctgataactgagagatcg
ggggtttaaatccctcccggagtacca
1–22tRNA–Met(cat)[90259,90334]
agttagttggcagagtggttatgcacctccttcatacggagcgactacag
tggttcaaatccactactaactacca
>KC579452.1 Enterobacteriophage phiKP26, complete genome
1–1tRNA–Arg(cct)[2921,2996]
gttctgctggcgtaacaggataacgcagagacctcctaagtctcagttac
tggttcgagtccagtgcggaacacca
>MF185728.1 Mycobacterium phage Finemlucis, complete genome
1–1tRNA–Thr(cgt)[63805,63878]
gccaccttagctcagttggtagagcagccccttcgtaacgggcaggtcag
cggttcgactccgctaggtggctc
1–2tRNA–Pro(tgg)[64100,64175]
cggcccaaggtgtaatggcttgcatgaggcgtttggatcgccggggttcc
ctgttcgactcggggtgggccgacca
1–3tRNA–Trp(cca)[64786,64859]
agcgagggagcacgattggttgtgcagcggattccaaacccgcaggttgc
cggttcgaccccggtcctcgttgc
1–4tRNA–Leu(tag)[64881,64955]
ggtcccgtagcccaattggcagaggcgcaggttttaggtacctgtcagtg
tgcgttcgagtcgcaccgggaccac
1–5tRNA–His(gtg)[64955,65027]
cgcgaggtagcttaagtagtaaagcccccgactgtgactcgggtgattcg
ggtgcaagtcccggcctgtcgcc
1–6tRNA–Gln(ctg)[65030,65105]
tggccgctagcacaattaggcagttgcgccggaatctgactccggaggtt
ccaggttcgattcctgggtggccagc
1–7tRNA–Gly(tcc)[65245,65319]
gtgctagtaacccatgttggctgggtgccactcttccaaagtggatttcg
cgtgttcgattcacgtctagcacac
1–8tRNA–Lys(ctt)[65355,65430]

gccgcgttagctcagttggtagagctgctgactcttaatcagtaggtcgg
gggttcgactccctcacgcggtacca
1-9tRNA-Ser(tga)[65432,65521]
ggagagtaatgcagcgggggtggcccgcgaccggccttgaaagccgggta
gccgttatggagcttggggttcgatccctctgctctccgc
1-10tRNA-Cys(gca)[66615,66686]
gccgttgtgtccgagtggttaggtgccaggctgcaaacctggttagtcgc
gttcgattcgcggggacggctc
1-11tRNA-Asn(gtt)[67008,67081]
tcctccgtagctcaattggcagagcgcgcgactgttaatcgcgtggttgg
tggttcgagtccacccgttggagc
1-12tRNA-Lys(ttt)c[67275,67349]
gcccctatagctcagttggtagagcaggagacttttaatcttcgggtcct
aggttcgatccctagtgggggcacc
>MF919520.1 Gordonia phage Lozinak, complete genome
1-2tRNA-Trp(cca)[6303,6376]
acggacgtagctcaactggtagagcagcggtctccaaaaccgcaggttgc
aggttcaagtcctgccggccgtgc
1-3tRNA-Tyr(gta)[6442,6524]
gtgggtgacacaccaatggtggtgtagcgggctgtaaccccgtggcgttc
tacgcgggcaggttcgatccctgccacccacac
1-4tRNA-Ser(cga)[6692,6775]
ggagggtatgcccatggtgggcacgcggtgtcgaataccgctatacggta
gccattccgggagttcgattcttctaccctccgc
1-5tRNA-Ser(gct)[6856,6939]
ggcgagttatccagtctgggactgggccccgttgctagcggacgcgcatc
cctgggtgtgaggttcgattcctcagctcgccgc
1-6tRNA-Asn(gtt)[7382,7457]
tccggtgtagctcaatcggcagagcgctcggctgttaaccgagaggttga
aggttcgacccccttccaccggagcca
1-7tRNA-Lys(ctt)[8009,8084]
gcctctgtagctcagttggtttagagcaaccgcctcttaagcggtgggtc
gcaggttcgaatccttccgggggcac
>MF919504.1 Mycobacterium phage DmpstrDiver, complete genome
1-1tRNA-Gly(tcc)[93735,93807]
gcgcttgtggtcgaattggaaagactcctggcttccacccaggttatgca
ggttcgagtcctgtcgagcgctc
>MF919506.1 Mycobacterium phage FireRed, complete genome
1-1tRNA-Arg(cct)[63278,63361]
atggcccgtagctcagtcaggtagagcagccttgccccttgcgggcttgg
ttcgtccccggttcaaatccgggcgggccttgcc
1-2tRNA-Gly(tcc)[63364,63436]
gcgcccgtggtcgaattggaaagactcctggcttccacccaggttatgca
ggttcgagtcctgtcgggcgctc

>MF919512.1 Mycobacterium phage Klein, complete genome
1-1tRNA-Gly(tcc)[93720,93792]
gcgcttgtggtcgagttggaaagactcctggcttccacccaggttatgca
ggttcgagtcctgtcgagcgctc
>MF919525.1 Mycobacterium phage Murica, complete genome
1-1tRNA-Arg(cct)[64538,64621]
atggcccgtagctcagtcaggtagagcagccttgccccttgcgggcttgg
ttcgtccccggttcaaatccgggcgggccttgcc
1-2tRNA-Gly(tcc)[64624,64696]
gcgcccgtggtcgaattggaaagactcctggcttccacccaggttatgca
ggttcgagtcctgtcgggcgctc
>MF919529.1 Mycobacterium phage Sassay, complete genome
1-1tRNA-Arg(cct)[61161,61244]
atggcccgtagctcagtcaggtagagcagccttgccccttgcgggcttgg
ttcgtccccggttcaaatccgggcgggccttgcc
1-2tRNA-Gly(tcc)[61247,61319]
gcgcccgtggtcgaattggaaagactcctggcttccacccaggttatgca
ggttcgagtcctgtcgggcgctc
>MF919535.1 Mycobacterium phage Terminus, complete genome
1-1tRNA-Arg(cct)[63835,63918]
atggcccgtagctcagtcaggtagagcagccttgccccttgcgggcttgg
ttcgtccccggttcaaatccgggcgggccttgcc
1-2tRNA-Gly(tcc)[63921,63993]
gcgcccgtggtcgaattggaaagactcctggcttccacccaggttatgca
ggttcgagtcctgtcgggcgctc
>MF919536.1 Mycobacterium phage TipsytheTRex, complete genome
1-1tRNA-Trp(cca)[4183,4256]
gtacacgtagctcaatcggtagagcagcggtctccaaagccgccggttcc
aggttcgactcctggcgtgtatgc
>MF919538.1 Mycobacterium phage Updawg, complete genome
1-1tRNA-Gln(ctg)[4930,5004]
tccccgttcgtctaagcggcaagacaccgggttctggccccggcaatcga
ggttcgagtccttgatggggagcca
>MF919540.1 Mycobacterium phage Willez, complete genome
1-1tRNA-Arg(cct)[61161,61244]
atggcccgtagctcagtcaggtagagcagccttgccccttgcgggcttgg
ttcgtccccggttcaaatccgggcgggccttgcc
1-2tRNA-Gly(tcc)[61247,61319]
gcgcccgtggtcgaattggaaagactcctggcttccacccaggttatgca
ggttcgagtcctgtcgggcgctc
>MF919541.1 Mycobacterium phage YassJohnny, complete genome
1-1tRNA-Arg(cct)[61182,61265]
atggcccgtagctcagtcaggtagagcagccttgccccttgcgggcttgg
ttcgtccccggttcaaatccgggcgggccttgcc

1–2tRNA–Gly(tcc)[61268,61340]
gcgcccgtggtcgaattggaaagactcctggcttccacccaggttatgca
ggttcgagtcctgtcgggcgctc
>KT825490.1 Escherichia phage C119, complete genome
1–1tRNA–Arg(cct)[42465,42540]
gttctgctggcgtaacaggataacgcagagacctcctaagtctcagttac
tggttcgagtccagtgcggaacacca
>KC661272.1 Mycobacterium phage Methuselah, complete genome
1–1tRNA–Asn(gtt)[3921,3996]
tgatctgtagctcaatcggcagagcacccggctgttaaccgggacgttgg
aggttcgagtcctcccagatcagcca
1–2tRNA–Trp(cca)[4037,4111]
aggcacgtagctcaattggtcagagcagcggtctccaaagccgccggctg
caggttcgagtcctgccgtgtctgc
1–3tRNA–Leu(cag)[4146,4220]
ggctcggtaggcaaacaggcaaagccgcctgtctcaggaacaggtgcgtg
agggttcgactccctcccgagctac
>KF188414.1 Mycobacterium phage ABCat, complete genome
1–1tRNA–Arg(cct)[63183,63266]
atggcccgtagctcagtcaggtagagcagccttgccccttgcgggcttgg
ttcgtccccggttcaaatccgggcgggccttgcc
1–2tRNA–Gly(tcc)[63269,63341]
gcgcccgtggtcgaattggaaagactcctggcttccacccaggttatgca
ggttcgagtcctgtcgggcgctc
>KT359365.1 Mycobacterium phage DaHudson, complete genome
1–1tRNA–Asn(gtt)[3832,3907]
tgatctgtagctcaatcggcagagcacccggctgttaaccgggacgttgg
aggttcgagtcctcccagatcagcca
1–2tRNA–Trp(cca)[3948,4022]
aggcacgtagctcaattggtcagagcagcggtctccaaagccgccggctg
caggttcgagtcctgccgtgtctgc
1–3tRNA–Leu(cag)[4057,4131]
ggctcggtaggcaaacaggcaaagccgcctgtctcaggaacaggtgcgtg
agggttcgactccctcccgagctac
>KT375356.1 Rhodococcus phage Rhodalysa, complete genome
1–1tRNA–Asn(gtt)[1503,1576]
tcacgtatagctcaatcggcagagcaacgcactgttaaggcgggggttga
aggttcgagtccttctacgtgagc
1–3tRNA–Trp(cca)[1702,1776]
gggcacgtagctcaattggatagagccccggtctccaaagccggtggttg
ggggttcgagtccctccgggcctgc
>KT381277.1 Mycobacterium phage Wooldri, complete genome
1–1tRNA–Asn(gtt)[3751,3826]
tgatctgtagctcaatcggcagagcacccggctgttaaccgggacgttgg

aggttcgagtcctcccagatcagcca
1−2tRNA−Trp(cca)[3867,3941]
aggcacgtagctcaattggtcagagcagcggtctccaaagccgccggctg
caggttcgagtcctgccgtgtctgc
1−3tRNA−Leu(cag)[3976,4050]
ggctcggtaggcaaacaggcaaagccgcctgtctcaggaacaggtgcgtg
agggttcgactccctcccgagctac
>KT959213.1 Rhodococcus phage TWAMP, complete genome
1−1tRNA−Asn(gtt)[1503,1576]
tcacgtatagctcaatcggcagagcaacgcactgttaaggcgggggttga
aggttcgagtccttctacgtgagc
1−3tRNA−Trp(cca)[1702,1776]
gggcacgtagctcaattggatagagccccggtctccaaagccggtggttg
ggggttcgagtccctccgggcctgc
>KT990218.1 Rhodococcus phage Lillie, complete genome
1−1tRNA−Asn(gtt)[1503,1576]
tcacgtatagctcaatcggcagagcaacgcactgttaaggcgggggttga
aggttcgagtccttctacgtgagc
1−3tRNA−Trp(cca)[1702,1776]
gggcacgtagctcaattggatagagccccggtctccaaagccggtggttg
ggggttcgagtccctccgggcctgc
>KU695582.1 Mycobacterium phage McFly, complete genome
1−1tRNA−Asn(gtt)[5402,5474]
tgacgtgtagctcaatggcagagcacccggctgttaaccggacggttgaa
ggttcgagtccttccatgtcagc
1−2tRNA−Trp(cca)[5479,5552]
gcgctcctagctcaattggtagagcagcggtctccaaagccgcgagttcc
aggttcgagtcctggggagcgtgc
1−3tRNA−Gln(ctg)[5594,5666]
tccctgttcgtctaatcggtaagacaccgggctctggacccggcaatcga
ggttcgagtccttgatggggagc
>KU865303.1 Mycobacterium phage TeardropMSU, complete genome
1−1tRNA−Arg(cct)[61937,62020]
atggcccgtagctcagtcaggtagagcagccttgccccttgcgggcttgg
ttcgtccccggttcaaatccgggcgggccttgcc
1−2tRNA−Gly(tcc)[62023,62095]
gcgcccgtggtcgaattggaaagactcctggcttccacccaggttatgca
ggttcgagtcctgtcgggcgctc
>KU935726.1 Mycobacterium phage Xerxes, complete genome
1−1tRNA−Thr(ggt)[28768,28845]
gatcctacggacgggaaagggacgtactacggtctggtaatccgcaggtc
gctggttcgagcccagctgggggcacca
>KU935730.1 Mycobacterium phage Pipsqueaks, complete genome
1−1tRNA−Thr(ggt)[28748,28825]

gatcctacggacgggaaagggacgtactacggtctggtaatccgcaggtc
gctggttcgagcccagctgggggcacca
>KU985096.1 Mycobacterium phage Kazan, complete genome
1-1tRNA-Asn(gtt)[5408,5480]
tgacgtgtagctcaatggcagagcacccggctgttaaccggacggttgaa
ggttcgagtccttccatgtcagc
1-2tRNA-Trp(cca)[5485,5558]
gcgctcctagctcaattggtagagcagcggtctccaaagccgcgagttcc
aggttcgagtcctggggagcgtgc
1-3tRNA-Gln(ctg)[5600,5672]
tccctgttcgtctaatcggtaagacaccgggctctggacccggcaatcga
ggttcgagtccttgatggggagc
>KU998254.1 Gordonia phage Kampe, complete genome
1-2tRNA-Leu(taa)c[70491,70577]
gcccctgtggcgaaattcggaatacgcgggagacttaaaatcttctagcc
tttatggctttatcggttcaagtccggtcaggggtac
>KU998252.1 Gordonia phage PatrickStar, complete genome
1-2tRNA-Leu(taa)c[70491,70577]
gcccctgtggcgaaattcggaatacgcgggagacttaaaatcttctagcc
tttatggctttatcggttcaagtccggtcaggggtac
>KX523125.1 Mycobacterium phage BuzzBuzz, complete genome
1-1tRNA-Asn(gtt)[3922,3997]
tgatctgtagctcaatcggcagagcacccggctgttaaccgggacgttgg
aggttcgagtcctcccagatcagcca
1-2tRNA-Trp(cca)[4038,4112]
aggcacgtagctcaattggtcagagcagcggtctccaaagccgccggctg
caggttcgagtcctgccgtgtctgc
1-3tRNA-Leu(cag)[4147,4221]
ggctcggtaggcaaacaggcaaagccgcctgtctcaggaacaggtgcgtg
agggttcgactccctcccgagctac
>GQ478082.1 Enterococcus phage phiFL1B, complete genome
1-1tRNA-Asn(gtt)[14841,14913]
tggaatatagctcagttggtagagcgtacgactgttaatcgtagggtcat
gagttcgagtctcgttattccag
1-2tRNA-Met(cat)[38073,38146]
ggaccattagctcagttggttagagccaaccgctcataacggttaggtca
taggttcgagtcctgtatggtcca
>GQ478083.1 Enterococcus phage phiFL1C, complete genome
1-1tRNA-Asn(gtt)[14575,14647]
tggaatatagctcagttggtagagcgtacgactgttaatcgtagggtcat
gagttcgagtctcgttattccag
1-2tRNA-Met(cat)[37804,37877]
ggaccattagctcagttggttagagccaaccgctcataacggttaggtca
taggttcgagtcctgtatggtcca

>KR063281.1 Gordonia phage GMA2, complete genome

2-1tRNA-Arg(tct)c[57692,57765]
gccctcatggtctaaaggataaggcacatgtcttctaaacatgacgatgt
cggttcgagtccggctgagggcac
2-2tRNA-Thr(tgt)c[57883,57955]
gccccattagcatattggcagatgcaccgctcttgtaaagcggatacagg
agttcgattctcctatggggctc
2-3tRNA-Glu(ttc)c[58076,58153]
gccggagttgctctgctggtaggggcaactggattttcactccaggtaag
acgcgggttcgattcccgtctccggtac
2-4tRNA-Ser(gct)c[58157,58240]
ggtgagttatcttgactgggtcaaggctccgttgctagcggaagcgcgtc
ttagggcgtggcgttcgattcgtcaactcaccgc
2-5tRNA-Ser(tga)c[58319,58403]
ggagagtatgctactggtgtagcaagcggtcttgaaaaccgctatacggt
atccattccgggggttcgactcctctactctccgc
2-6tRNA-Lys(ctt)c[58406,58478]
gctcctatagctcagcggtagagctgcggactcttaatccgtaggtccta
ggttcaaatcctagtgggagcac
2-7tRNA-Lys(ttt)c[58481,58552]
gggtctctcgtataacggcattaccgctggcttttacccagcagaacagg
gttcgactccctggggacccac
2-8tRNA-Glu(ctc)c[58733,58806]
gccgcattggagtaacggttatctcatctgattctcaatcagaagactac
gggttcgattcccgtatgcggtac
2-9tRNA-Leu(tag)c[58894,58978]
gccccattggcgaaattggcagacgcgctagatttaggatctagtgtcct
ttagggcgtgagggttcgagtcccttgtggggtac
2-10tRNA-Asp(gtc)c[58993,59068]
gccgatatagaggagtctggagtcctcgccaccctgtcacggtggagatc
acgggttcaaatcccgttattggcgc
2-11tRNA-Tyr(gta)c[59077,59160]
ggggagtaggcaactgttggttggttgcggctggctgtaaaccagttctg
agaggctcgggggttcgattccctcacttcccac
2-12tRNA-Pro(tgg)c[59301,59375]
cgggatgtagttcaacttggtagagcacttgctttgggtgcaagatgttg
caggttcaaatcctgtcatcccgac
2-13tRNA-Ile(gat)c[59483,59557]
cggtctttagctcagttggttagagcatcgccctgataaggcgaaggtcc
gaggttcaaatcctcgaagatcgac
2-14tRNA-Met(cat)c[59632,59706]
agcgcggtagagcagtttggtagctcaggagtctcataaacttcaggtca
cgggttcaaatcccgttcgcgccac
2-15tRNA-Asn(gtt)c[59744,59816]

tacccggtagctcaatggcagagcgctagactgttaatctggtggttgaa
ggttcgagtccttctcgggtagc
2–16tRNA–Gln(ttg)c[59821,59895]
tgcccgctagcacaattggtagttgcgccgcgctttggtcgcggatgttc
caggttcgagtcctgggtgggcagc
2–17tRNA–Trp(cca)c[60083,60154]
atggacgtagttcaatggtagaacatcggtctccaaaaccgagtacgtgg
gttcgattcctaccgtccgtgc
>KY629563.1 Sphingobium phage Lacusarx, complete genome
3–1tRNA–Pro(cgg)[66918,66995]
ctgggtgtagcgcagtccggttagcgtgtgcggttcggagccgtgaggtc
gtaggttcaaatcctaccatccagacca
3–2tRNA–Val(tac)[67270,67344]
tggggattagctcagcggtagagcagccgttttacatgcggccggtcggc
ggttcgatcccgtcatccctaacca
3–3tRNA–Val(gac)[67347,67421]
gcctcgttagctcagcggtagagcgctgctatgacatggcagatgacggt
ggttcaaacccatcacgaggcacca
3–4tRNA–Ala(tgc)[67694,67768]
ggggatgtagctcagaggtagagcacccgttttgcaagcggaagcgcgtc
ggttcgactccgaccatctccacca
3–5tRNA–Asn(gtt)[67903,67984]
ttggtagtgacagagtggtaatgtgcccggctgttaaccggtgacccgaa
aggcggtggttcgatcccaccctaccgaacca
3–6tRNA–Ile(gat)[67986,68062]
agcggtgtagctcagcccggtagagcaaccgaccgataatcggtgtgtcg
caggttcaaatcctgccatcgctacca
3–7tRNA–Lys(ctt)[68807,68882]
ggatcggtagctcagtaggtagagcaggggattcttactcccaaggtgga
cggttcaagcccgtctcgatcctcca
3–8tRNA–Lys(ttt)[68955,69030]
gggccggtagctcagtaggcagagcagggggcttttaacctcaaggtggt
cggttcaagcccgacccggctcacca
3–9tRNA–Gly(gcc)[69039,69113]
gcaggcgtcgttcagacggtaggacacctccttgccatggaggagacgcg
ggttcgagacccgccgcccgctcca
3–10tRNA–Asp(gtc)[69126,69202]
ggttctgtagcttagctggtgaaagcgccgggttgtcaactcggagatcg
cgggttcaagtcccgtcagaaccgcca
3–11tRNA–Glu(ctc)[69204,69278]
ggggcgttcgtctagcggacaggacgctggcttctcaagccggaagcagg
ggttcgattcccctacgcctcacca
3–12tRNA–Glu(ttc)[69287,69362]
agcgccatcgtctagcgagcaggacacgttggctttcacccttcggacgc

gggtttgaatcccgttggcgctgcca
3–13tRNA–Gly(tcc)[69389,69463]
gcgacattagctcagcggtagagcggcggttttccagtccgcgcgtcagg
agttcgatcctcctatgtcgctcca
3–14tRNA–Arg(acg)[69473,69548]
ggcccggaagcacaactggacgtgcgctccgctacgaacggaaaggttgt
gggttcgagtcccacgcgggcctcca
3–15tRNA–Phe(gaa)[69554,69628]
ggtgaggtagctcagaggtagagcgccggtttgaaaagccgggcgtcggt
ggttcgatcccgcctctcaccacca
3–16tRNA–Ser(cga)[70140,70228]
ggagggtggctgcaagaggtgcgtaaccggtctcgaaaaccgggctgggg
ttaggagccctacgggttcgatccctgcaccctccgcca
3–17tRNA–Leu(cag)[70382,70466]
ggcccgctggtgaaatgtagacgcactcgcctcagaagcgagcgaccgag
aggtcatcccggttcaaatccggggcgggccacca
3–18tRNA–Leu(gag)[70472,70556]
ggggatgtggtgaaataggtaaacgcagcgggtcgagagcccgccgcctg
atggcttgagggttcaagtccctccatccccacca
3–19tRNA–Thr(cgt)[70588,70661]
gcccggttagctcaatggtagagctacgccgtcgtaacgcgaggacgagg
gttcgattccctcaccgggctcca
3–20tRNA–His(gtg)[70676,70751]
gtgaccgaggcagagttggatcatgtgccgggtcgtgatcccggatacgg
cgggttcgagccccgccggtcacccc
3–21tRNA–Gln(ctg)[70770,70843]
ttcccgttcgttcaatggtaggacgcccggctctgaaccgggagatcgag
gttcgagtccttgacggggaacca
3–22tRNA–Ser(gct)[70859,70945]
ggagagttgccagagcggtaatgggctggcttgctaagccaagacaacct
cacggttgcgagggttcgatcccctcactctcctcca
3–23tRNA–Cys(gca)[70954,71027]
gccgccttggccgagtggcaaggctccggattgcaaccccggcatctccc
gttcgatccgggaaggcggctcca
3–24tRNA–Trp(cca)[71032,71107]
atctccaaagcattaaggggcgatgcaccggtctccaaaaccggcaagct
gggttcgagtcctagtggaggtgcca
>KT870145.1 Roseobacter phage DSS3P8, complete genome
4–1tRNA–Glu(ttc)[6433,6509]
gccgcatgggacttcttggcgaaggtcgctgccctttcaaggcggagaag
cgggttcgaaacccgcatgcggcacca
4–2tRNA–Glu(ctc)[6542,6618]
ggagcattggacttcttggtgaaggtcgccgcgttctcaacgcggagaag
cgggttcgaaacccgcatgctctacca

4-3tRNA-Met(cat)[7058,7132]
ggcgacgtagctcagaggtagagcgcttggctcataaccgagtggtcggt
ggttcgatcccacccgtcgccacca
4-4tRNA-Pro(tgg)[7206,7281]
cagggtgtagctcagtaggtagagtgccggacttggaatccggaggccgt
cggttcgagaccgaccaccctgacca
4-5tRNA-Met(cat)[7712,7787]
tgcgggatagagcagaggtcagctcgccatcctcataaggtggaggtcgt
cggttcgaatccggctcccgcaacca
4-6tRNA-Tyr(gta)[11149,11235]
gggagggttcagccacaggtcgtggtaggtgggttgtaaccccactccgg
gtttccgggcgcagttcgattctgcactctcccacca
4-7tRNA-Ala(cgc)[11429,11515]
tggcgagtggcgaaatggtaaacgtacgggttcgctacccggagcaggtt
gtccctgttggtggttcaagtccaccctcgccgacca
4-8tRNA-Val(tac)[13913,13988]
gcccggttagctcagcggtagagcttcgcctttacacggcgagtaagcgg
cggttcgatcccgccactgggtacca
4-9tRNA-Val(gac)[14102,14176]
ggtcccttagctcagcggtagagcgtctggtcgacatccagaaggtcact
ggttcaatcccagtagggtccacca
4-10tRNA-Ala(tgc)[14314,14389]
gggctgttagctcatctgggagagcgcctgccttgcaagcaggaggtggc
gagttcgagactcgcacggtccacca
4-11tRNA-Ala(tgc)[14399,14472]
ggggcacgtacatgggtgtcgggcgatccttgcaagattgctgtctcggg
gttcgattccccggtgctccacca
4-12tRNA-Lys(ctt)[15211,15298]
gcgccggtgctccgagtggacgatggggtcggactcttaatccgaatgcg
aaagcacaccgtgggttcgaatcccacccggcacacca
4-13tRNA-Asn(gtt)[15304,15381]
tggcatgtagtcttccgggtgaaggcgctcggctgttaaccgagattgag
gctggttcgagtccagccgtgccagcca
4-14tRNA-Cys(gca)[15385,15458]
gacgatagagccgggtggttaggccggggattgcaaatcctcttaggtga
gttcgattctcactgtcgtctcca
4-15tRNA-His(gtg)[15464,15539]
gctgactaagtgttgatggatacacgtctggttgtggaccagaaatagcg
aggttcgatacctgcagtcagttcca
4-16tRNA-Asp(gtc)[15544,15617]
ggcctatttgccgtcagggaaggctacccgctgtctacgggtaaagacgg
gttcgagtcccgtataggtcgcca
4-17tRNA-Gly(tcc)[16689,16762]
gcggatgtagccaagtggtaaggcagcaggcttccaccctgccatcgtgg

gttcgatccccatcatccgctcca
4−18tRNA−Gln(ctg)[16805,16878]
tgccccatcgtctagcggtaggacgcctgtctctgaaacaggaaagcgcg
gttcgaatcctcgtggggcatcca
4−19tRNA−Gly(gcc)[16969,17043]
gcaggtatagctcagcggaagagcatctggttgccaatcagaaggccgtg
ggttcgacccccactacctgctcca
4−20tRNA−Ile(gat)[17144,17218]
ggtcctgtggcgtaaatggaaacgtacctgcctgattagcgggggttaga
ggttcgatccctctcgggactacca
4−21tRNA−Leu(cag)[17226,17310]
gcggatgtggcgaaatggtagacgcgctgtcttcagaaggcagtgtccta
gtggcgtgagggttcaaatccctccatccgcacca
4−22tRNA−Phe(gaa)[17381,17456]
gtgtctgtagtgaggctggtgctcacgctggtttgaaaagccagagacgg
aggctcgatccctcccggacacacca
4−23tRNA−Leu(gag)[17462,17545]
gcccatgtggtgaaaaggtagacacgcagtcttgagagggctgtgccgca
aggtgtgctggttcaaatccagtcatgggcacca
4−24tRNA−Thr(tgt)[17554,17629]
gccgtcatagctcagttggaagagcgctcgccttgtaagcgagatgtcgc
gggttcgattcctgctggcggcacca
4−25tRNA−Leu(caa)[17638,17713]
gcgggaatgggcaaatggtaaagtcgccccccttcaaacgggggagcctga
gggttcgagtccctcttcccgtacca
4−26tRNA−Lys(ctt)[17991,18065]
gcaccggtagctcagaggcagagcggcaggcccttaacctgcgcgacggg
atttcgaaattcccccggtacacca
4−27tRNA−Thr(ggt)[18147,18221]
gctgccatagctcagtaggtagagcatgcccttggtacgggtaagacaca
ggttcgagacctgttggcagcacca
4−28tRNA−Arg(tct)[20440,20515]
ctgctcgtagctcagaggacagagcgtggggcttctacccccagcggcga
gggttcgaatccttccgggcaggcca
4−29tRNA−Thr(cgt)[20632,20707]
gctgatgtagctcagtaggcagagcgctcgcctcgtaagcgagaggtcgt
cggttcgaaaccgaccgtcagcacca
4−30tRNA−Pro(ggg)[20712,20786]
caggatgtagctcaggggtagagtgcttggtcgggaaccaagaggccgcg
ggttcaatccccgccatcctgacca
4−31tRNA−Phe(gaa)[55152,55227]
gcccggatagctcagttggtagagcacacgactgaaaatcgtggtgtcgg
cggttcgaccccgtctccgggcacca
>KX925554.1 Streptomyces phage BRock, complete genome

5-1tRNA-Tyr(gta)c[95476,95558]
gcgtgtgtaacccgtaattggtagcggtgcggcctgtaaagccgttgctt
cggctctgggggttcaagtccctccgcacgcac
5-2tRNA-His(gtg)c[95711,95784]
gggtcggtagctcaattggtagagcgccgcgttgtggtcgcggtggtttc
gggttcgaatcccggccttccctc
5-3tRNA-Ile(gat)c[96139,96222]
cggcgcgtatccaagtctggttaaggagccggtctgataaaccggtgcgg
aaacgtacgtaggttcaaatcctaccgcgtcgac
5-4tRNA-Ser(gga)c[96433,96518]
ggaaggtaagcaatttggctagcagcttgattggaaatcaagtacgactc
tcaaggtctaacgggttcgattcccgtaccttccgc
5-5tRNA-Ser(gct)c[96606,96695]
ggagatgtggctgagtccggtttaaggcgctcgactgctaatcgagtggg
tgtaaaagccccgcaggttcaaatcctgtcatctccgcca
5-6tRNA-Arg(tcg)c[96956,97037]
gcggtactagccgagatccatgaggcatttggagttcgcctgcaaagcga
atggcatacggttcgattccgtagtatcgctc
5-7tRNA-Val(tac)c[97297,97369]
gccggtatagctcagtgggagagcgtcactcttacaaagtgagggtcggg
ggttcgaatccctctgccggtac
5-8tRNA-Arg(tct)c[97773,97846]
gcctccgtagctcagtggaaagagcagcagccttctaagctgacggtcgc
aggttcgaatcctgccgggggtac
5-9tRNA-Arg(acg)c[98530,98602]
gcgctcgtggtgtaatggacagcacaacagtctacggaactgttagaggg
ggttcgaatccctccgggcgtac
5-10tRNA-Lys(ttt)c[101007,101080]
gattccttagctcagttggcagagcagtgggcttttaacccaacggtcgt
cggttcgagcccgacaggaatcac
5-11tRNA-Lys(ctt)c[101248,101321]
gctccgttagctcagttggtagagcggcggcctcttaagccgatggtcgg
gggttcaagtccctcacggagcac
5-12tRNA-Ala(tgc)c[101394,101467]
gggcccatagctccaacggtagagcaacacccttgcaaggtgaaggttgt
cggttcgaatccgactgggtccac
5-13tRNA-Asn(gtt)c[103903,103975]
tctcctgtagctcaatggcagagcagacggctgttaaccgtcaggttgtt
ggttcgagtccagccgggagagc
5-14tRNA-Trp(cca)c[104182,104253]
aggttgttgatgtagtggtaacatgatggtctccaaaaccattctcaggg
gttcgaatcctctacagcctgc
5-15tRNA-Leu(tag)c[104257,104342]
gccggtgtggcggaatttaggcagacgcgcggggtttaggttcccgtgtc

ctagtggcgtgcaggttcaagtcctgtcaccggtac
5-16tRNA-Leu(caa)c[104356,104438]
gcccacttggcgtaatggcagccgcgccggattcaagtcccggtgtccta
gtgacgtgtcagttcgagtctgacagtgggtac
5-17tRNA-Thr(ggt)c[104606,104681]
gccgacttaactcagcaggtagagtactgtcttggtaagacagaagtcac
gggttcgagtcccgtagtcggctcca
5-18tRNA-Thr(cgt)c[104781,104852]
gccctactagttcaatggtagaatgactccttcgtacggagtaggttggg
gttcgattcctcagtggggctc
5-19tRNA-Thr(tgt)c[104889,104962]
gcccctatagctcagtggtagagcaatcgccttgtaagcgataggtcgtg
ggttcaaatcctactgggggctcc
5-20tRNA-Glu(ttc)c[105390,105463]
gtccccgtagtgtagcggccaagcacgctggactttcaattcggtagcgc
gggttcgaatcccgtcgggggtac
5-21tRNA-Glu(ctc)c[105631,105704]
gtccccatagtgtagtttggtagcacgacagcctctcaagctgttagcac
gggttcaaatcccgttgggggtac
5-22tRNA-Asp(gtc)c[105866,105939]
ggacctgtagtgtatgggagtagcacggtaccctgtcaaggtgcaagggc
gggttcgaatcccgtcaggtccgc
5-23tRNA-Gln(ttg)c[105988,106061]
tgggatatggggtaattggcagcccgcctgactttggatcaggacagtct
aggttcgagtcctagtgtcccagc
5-24tRNA-Gln(ctg)c[106315,106386]
tccccggtggtgtaatggcagcacaacggtctctggaaccgttggtcttg
gttcgagtccaggctggggagc
5-25tRNA-Gly(tcc)c[106484,106557]
gcggtattagtgttcaatggcagcacgtcagtcttccaaactgaaagtag
gggttcgaatcccctatgccgttc
5-26tRNA-Met(cat)c[106776,106859]
tgcagggtagagaagttcggtcatctcgctagcctcataagctagaaatc
gccttaagtggttcaaatcccgcccctgctccca
5-27tRNA-Met(cat)c[106861,106935]
ggctgtgtagctcaattaggcagagcacccgactcataatcgggcggtcg
atggttcaaatccatccacagccac
5-28tRNA-Phe(gaa)c[107014,107086]
gcccctctagctgagatggattagcgctagcctgaaaagcttgagaggtc
ggttcgataccggcgggggcac
5-29tRNA-Pro(tgg)c[107164,107238]
ccggatatcgtctagtttggtaaggcgcctgctttgggagcaggatatcg
ggagttcaaatctctctatccggac

*Virgaviridae

>L07937.1 Soil–borne wheat mosaic virus (SBWMV) complete RNA 2–1tRNA–Val(cac)[3505,3575]
gatgaggtgcgactggtggtcgcggctgctatcacacagcagctaggagg
ggttcaattccccccccatcc
