# Supplementary figures and images for: Global In-Silico Scenario of tRNA Genes and Their Organization in Virus Genomes
Source: Viruses. 2019 Feb 21;11(2):180. doi: 10.3390/v11020180 (PMC6409571; doi:10.3390/v11020180)

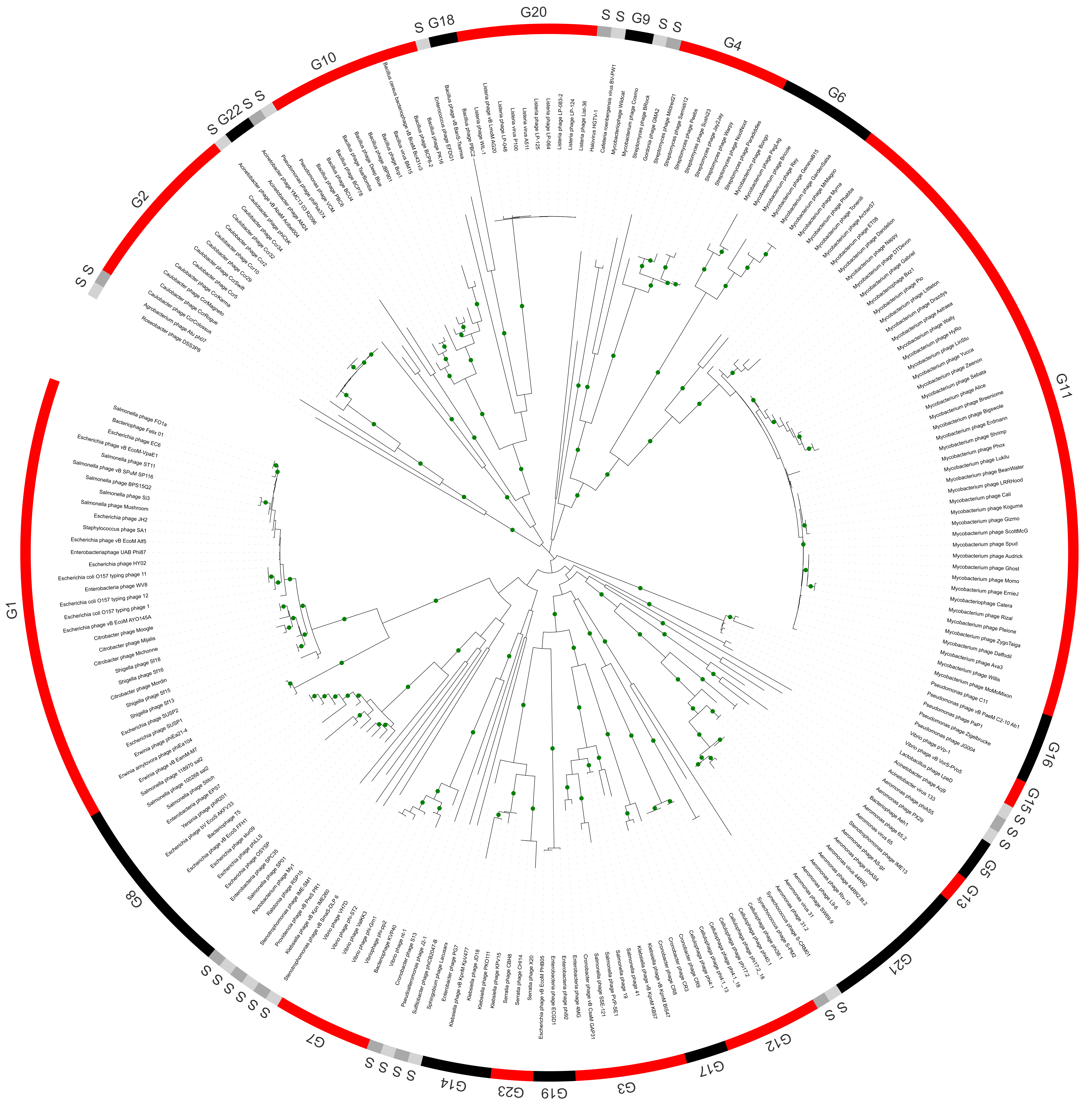

Supplement: Supplementary file 1 [file viruses-11-00180-s001.zip › viruses-406888-supplementary/FigS2.tiff]

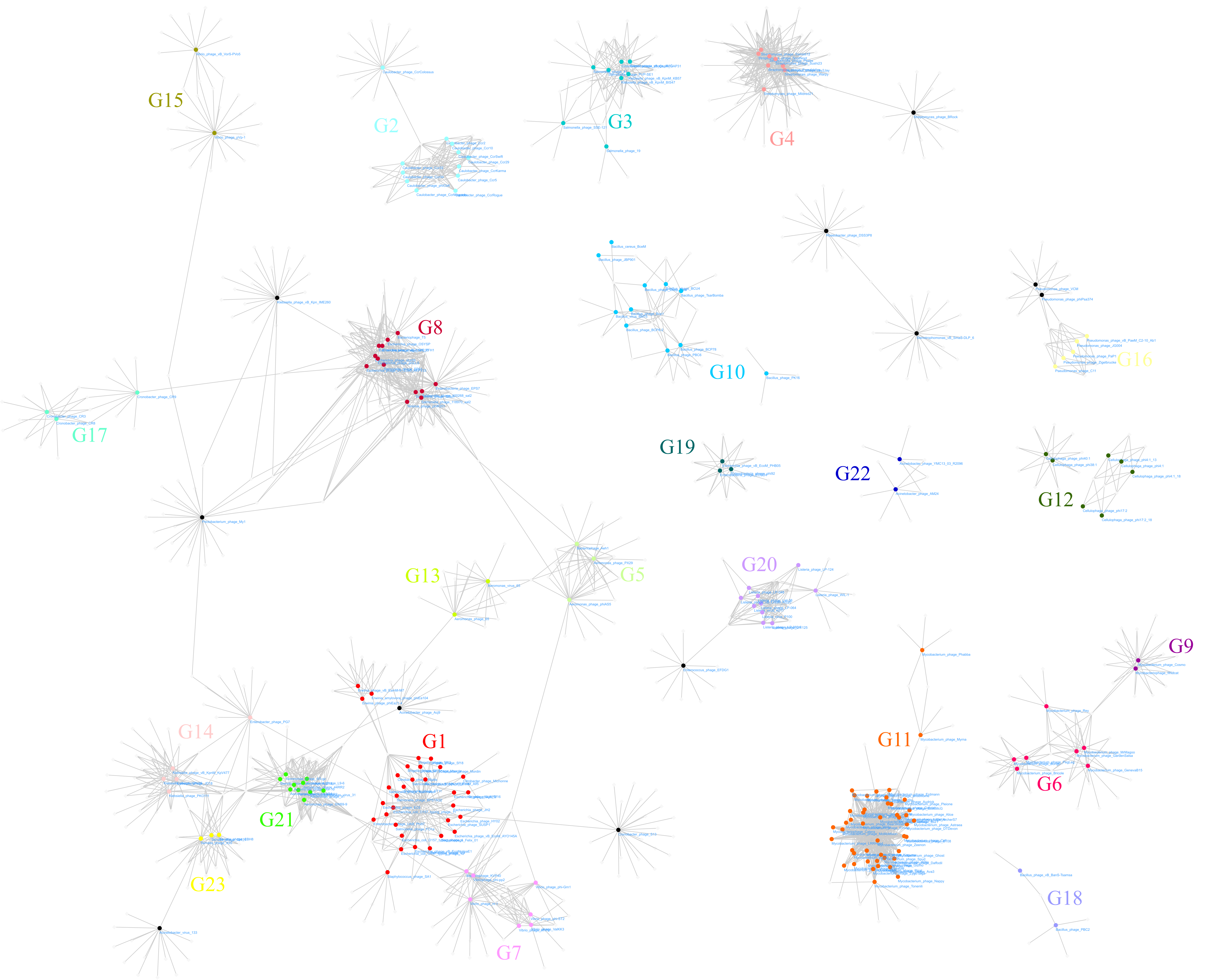

Supplement: Supplementary file 1 [file viruses-11-00180-s001.zip › viruses-406888-supplementary/FigS5.tiff]

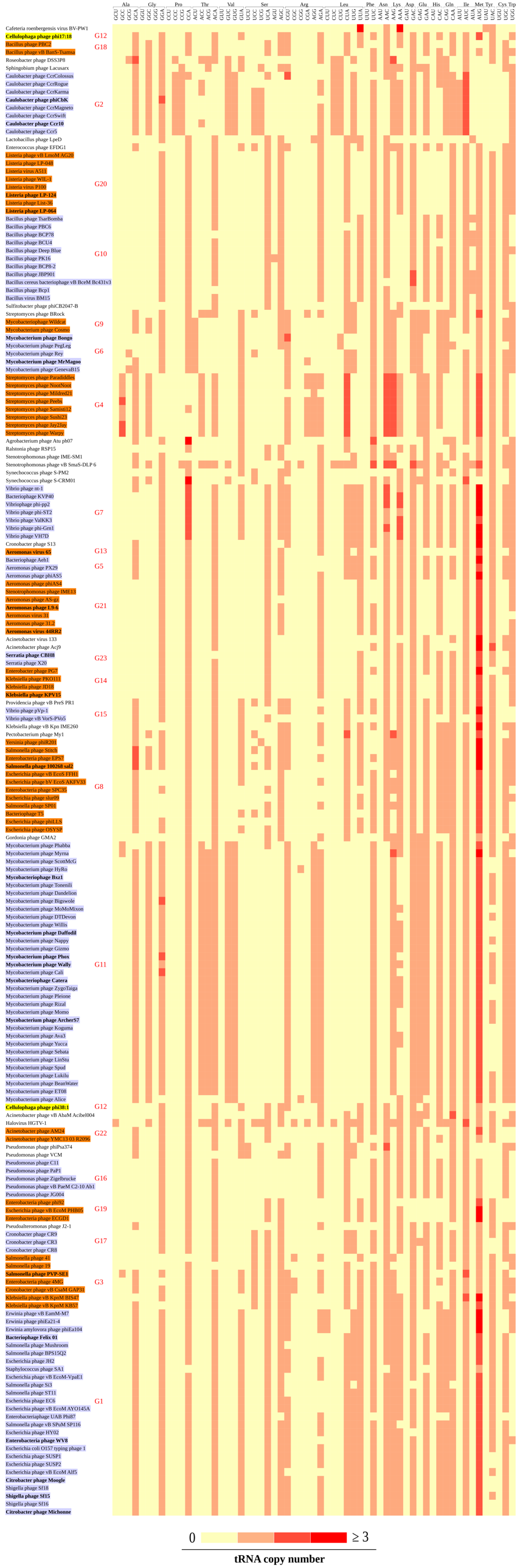

Supplement: Supplementary file 1 [file viruses-11-00180-s001.zip › viruses-406888-supplementary/FigS4.tiff]
